# Supplementary material for: Therapeutic modulation of ROCK overcomes metabolic adaptation of cancer cells to OXPHOS inhibition and drives synergistic anti-tumor activity
Source: bioRxiv. 2024 Sep 20:2024.09.16.613317. Preprint. [Version 1] doi: 10.1101/2024.09.16.613317 (PMC11429714; doi:10.1101/2024.09.16.613317)
Supplement: Supplement 1 [file media-1.pdf]

Supplementary Figure Legends

Supplementary Figure 1. Analysis of CRISPR-Cas9 FDA-ome library screens in the presence of IACS-10759 in *SMARCA4*-mutant lung cancer cells. **(A)** Box plots of $\log_2$-transformed sgRNA normalized read counts from A549, H1299, and H2023 cell lines prior to drug treatment (baseline), at early timepoints, and at late timepoints with DMSO or 4nM IACS-10759 treatment. (**B**) Pearson correlation of all genes across all treatment groups in A549, H1299, and H2023 cell lines. Data indicate (n=2) independent experiements. (**C**) Precision-recall curves of BAGEL results for A549, H1299, and H2023 cell lines using the FDAome library. (**D**) Mean fold change distributions of core essential genes or non-essential genes included in FDA-ome library in A549, H1299, and H2023 cell lines at early and late time points, where fold change is calculated as $\log_2$ (normalized read counts from early or late time points) $-\log_2$ (normalized T0 read counts).

Supplementary Figure 2. Chemo-genomic analysis of FDA-ome CRISPR hits (**A**-**B**) Scatterplot of NormZ scores in A549 (**A**) and H2023 (**B**) cell lines similar to as shown in Figure.1B. (**C-E**) Rug plots showing NormZ score distributions of individual sgRNAs for all FDA-ome library genes in A549 (**C**) , H1299 (**D**) , and H2023 (**E**) cell lines at early and late time points following 4nM IACS-10759 treatment. In each plot, red lines show individual sgRNAs targeting the indicated genes and grey lines show all other genes in FDA-ome library.

Supplementary Figure 3. Combinatorial drug screening of FDA-ome library targets with IACS-10759 using FDA approved and clinical grade therapeutics. (**A**-**E**) Treatment of three *SMARCA4*-mutant cell lines with a 3×6 matrix titration using different concentrations of IACS-10759 and either 6-AN (**A**), KD025 (**B**), Palbociclib (**C**), Amlexanox (**D**) or CB-839 (**E**). Heatmaps of each cell line depict percent growth inhibition across all concentrations for indicated

therapeutic. Synergy scores were determined using the Bliss independence model. A synergy score above 10 is considered a synergistic drug interaction. A score between -10 to 10 is considered additive. A score below -10 is considered antagonistic.

Supplementary Figure 4. KD025 is highly synergistic with biguanide class of OXPHOS inhibitors. (**A**-**B**) Dose-response curves of A549 (**A**) and H2023 (**B**) cell lines treated with increasing concentrations of KD025 in the presence or absence of IACS-10759. Cell growth (measured as % Confluency) was assayed 5 days after drug exposure. Data are presented as mean +/- SEM of three independent experiments. (**C-D**) Clonogenic growth assays of A549 (**C**) and H2023 (**D**) cell lines cultured in the presence of KD025 and IACS-10759 alone or in combination for 12 days. Surviving cells after the treatment were fixed and visualized by crystal violet staining. Representative images of three independent experiments are shown. (**E-F**) Dose-response curves of H1299 cells treated with increasing concentrations of KD025 in the presence or absence of Metformin (**E**) or presence or absence of Phenformin (**F**). Cell growth (measured as % Confluency) was assayed 5 days after drug exposure. Data are presented as mean +/- SEM of three independent experiments. (**G-H**) Clonogenic growth assays of H1299 cells cultured in the presence of KD025 and Metformin (**G**) or KD025 and Phenformin (**H**) alone or in combination for 12 days. Surviving cells after treatment were fixed and visualized by crystal violet staining. Representative images of three independent experiments are shown. (**I**-**K**) Treatment of H1299 cells with a 3×6 matrix titration using different concentrations of KD025 with either Metformin (**I**), Phenformin (**J**), or IM156 (**K**). Heatmaps of each combination depict percent growth inhibition across all concentrations for indicated therapeutic. Synergy scores were determined using the Bliss independence model. A synergy score above 10 is considered a synergistic drug

interaction. A score between -10 to 10 is considered additive. A score below -10 is considered antagonistic.

Supplementary Figure 5: Genetic validation of KD025 and IACS-10759 combination in H1299 cells. (A) Immunoblot showing shRNA knockodown of both ROCK1 and ROCK2 in H1299 cells after dox addition (1μg/ml). **(B)** Clonogenic growth assay of H1299 cells stably transduced with ROCK1/2 shRNA and cultured in the presence or absence of doxycycline (1μg/ml). Cells were treated with IACS-10759 for 12 days. Surviving cells after treatment were fixed and visualized by crystal violet staining. Representative images of three independent experiments are shown.

Supplementary Figure 6. Metabolomic analysis of KD025 and IACS-10759 combination in H1299 lung cancer cells. (A) Heatmap and unsupervised clustering showing $\log_2$ fold change in abundance changes in all quantitated metabolites following DMSO, 2μM KD025, 4nM IACS-10759 or the combined treatment for 24- and 48 hours. Yellow indicates up-regulated metabolites and blue indicates down-regulated metabolites. White indicates no change. **(B)** Principal component analysis (PCA) of all quantitated metabolites in H1299 cells following treatment with DMSO, IACS-10759, KD025, or the combination for 24 hours (top panel) and 48 hours (bottom panel) treatment. **(C)** Heatmap showing enriched metabolic pathways (FDR < 0.05) from significantly different up- and down-regulated metabolite abundances ($\log_2$ FC >1.5, P value < 0.05) by MSEA. The color is scaled according to significance (FDR), with blue representing non-significant. (**D**) Volcano plots of $\log_2$ fold change metabolite abundance of KD025, IACS-10759, or the combination following 24- or 48-hours treatment. Metabolite abundance ($\log_2$ FC (treatment/DMSO) > 1.5, P value < 0.05) were determined as significantly different as indicated by dotted lines. Red dots indicate glycolysis, TCA cycle, and PPP metabolites that were

significantly up-regulated. Blue dots indicate glycolysis, TCA cycle, and PPP metabolites that were significantly down-regulated.

Supplementary Figure 7. Depletion of ATP, NADH, and NADPH cellular pools in the IACS-10759 and KD025 combination. (A) Isotope tracing diagram for $^{13}C_6$-glucose through glycolysis and into TCA cycle metabolic pathways. Solid red circle, ^{13}C; open circle, ^{12}C. (**B**) Isotope tracing diagram for $^{13}C_5$-glutamine through TCA cycle metabolic pathway. Solid red circle, ^{13}C; open circle, ^{12}C. (**C**) Relative ratios of NADPH/NADP+ (left) and NADH/NAD+ (right) following treatment with IACS-10759, KD025, or the combination for 48 hours. (**D**) Relative abundance of AMP (left) and ATP (right) following treatment with IACS-10759, KD025, or the combination for 48 hours. (E) Relative ratio of ATP/AMP following treatment with IACS-10759, KD025, or the combination for 48 hours.

Supplementary Figure 8. Proteomic and phosphoproteomic analysis of KD025 and IACS-10759 combination in H1299 lung cancer cells. (A-B) Quantitation of proteome (**A**) and phosphoproteome (**B**) across all treatment groups by TMT mass spectrometry. "Changed by" refers to $\log_2$ fold change (FC) of proteins or phosphoproteins ($\log_2$ FC ≥ 1.5, P value 0.05) compared to DMSO. (n =4) biological replicates (**C**) PCA plots of all quantitated proteins (left) or phosphoproteins (right) in H1299 cells following treatment with DMSO, 4nM IACS-10759, 2μM KD025, or the combination for 6 hours. Data indicate independent experiments(n=4) (**D**) Pearson correlation of all quantified proteins (left) or phosphoproteins (right) across all treatment groups. Data indicate four independent experiments (n=4).

Supplementary Figure 9. Proteomic analysis of top enriched pathways and protein-protein interaction networks from combined treatment of IACS-10759 and KD025. (**A**) Top Reactome, KEGG, GOBP pathway gene sets significantly enriched ($P < 0.5$) in down-regulated

proteins. (**B**) Top Reactome, KEGG, GOBP pathway gene sets significantly enriched (P< 0.05) in up-regulated proteins. (**C**) PPI network of significantly down-regulated proteins from combined KD025 and IACS-10759 treatment. (**D**) PPI network of significantly up-regulated proteins from combined KD025 and IACS-10759 treatment. PPI, Protein-protein interaction; Top three enriched terms for each PPI cluster are indicated using GOBP, KEGG, and Reactome pathway gene sets. PPI, Protein-protein interaction.

Supplementary Figure 10. Phosphoproteomic analysis of single agent IACS-10759 and KD025 treatment. **(A)** Top Reactome, KEGG, GOBP pathway gene sets significantly enriched in up-regulated proteins of IACS-10759-treated cells. (**B**) Top Reactome, KEGG, GOBP pathway gene sets significantly enriched in down-regulated proteins of KD025-treated cells. (**C**) Top Reactome, KEGG, GOBP pathway gene sets significantly enriched in up-regulated proteins of KD025-treated cells. (**D**) PPI network with differentially expressed phosphoproteins either up-regulated by IACS-10769 (left), down-regulated by KD025 (middle), or up-regulated by KD025 (right). Top three enriched terms for each PPI cluster are indicated using GOBP, KEGG, and Reactome pathway gene sets. PPI, Protein-protein interaction.

Supplementary Figure 11. Top protein-protein interaction networks from combined treatment of IACS-10759 and KD025. (**A-B**) PPI network with differentially expressed phosphoproteins either up-regulated (**A**) or down-regulated (**B**) from combined treatment of KD025 and IACS-10759. Color scale represent the different MCODE clusters identified. PPI, Protein-protein interaction; MCODE, Molecular Complex Detection. (**C**) Top 3 down-regulated PPI clusters identified using MCODE with top three enriched terms for each cluster indicated from GOBP, KEGG, and Reactome gene sets. (**D**) Top 3 up-regulated PPI clusters identified using

MCODE with top three enriched terms for each cluster indicated from GOBP, KEGG, and Reactome gene sets.

Supplementary Figure 12. Relative kinase activities and clustering of 303 Ser/Thr kinases on the basis of their motif class following IACS-10759, KD025, or combination treatment for 6 hours. (**A**) Basophilic kinases subcategorized into 11 motif classes. (**B**) Proline-directed kinases subcategorized into 5 motif classes. (**C**) Acidophilic kinases subcategorized into 8 motif classes. (**D**) Unique kinases subcategorized into 13 motif classes.

Supplementary Figure 13. Characterization of kinase regulation in top Reactome gene sets of co-regulated phosphoproteins (A) Immunoblot analysis of p-AMPK T172 and AMPK in H1299 cells following a 6-48 hr timecourse with 4nM IACS-10759, 2μM KD025, or the combination. β-actin serves as a loading control. (**B**) Volcano plots showing kinase-motif enrichment of three phosphoprotein subsets in the combination, down-regulated phosphoproteins, co-regulated phosphoproteins, and up-regulated phosphoproteins. The enrichments in these plots were determined using one-sided exact Fisher's tests and corrected for multiple hypotheses using the Benjamini–Hochberg method. (**C**) Motif logo analysis of 37 up-regulated phosphosites with AMPK motif. (**D**) Top kinase interaction scores for pathways in Reactome gene sets co-regulated phosphoproteins. Kinase-pathway interactions were constructed using kinase-motif specificities [54], kinase-substrate specificities from PhosphoSitePlus [52], and kinase-substrate interactions from BioGRID[65] for all phosphoproteins annotated for each pathway. (**E**) Top significantly enriched Reactome gene sets in non-essential co-regulated phosphoproteins. (**F**) Top significantly enriched Reactome gene sets in essential co-regulated phosphoproteins.

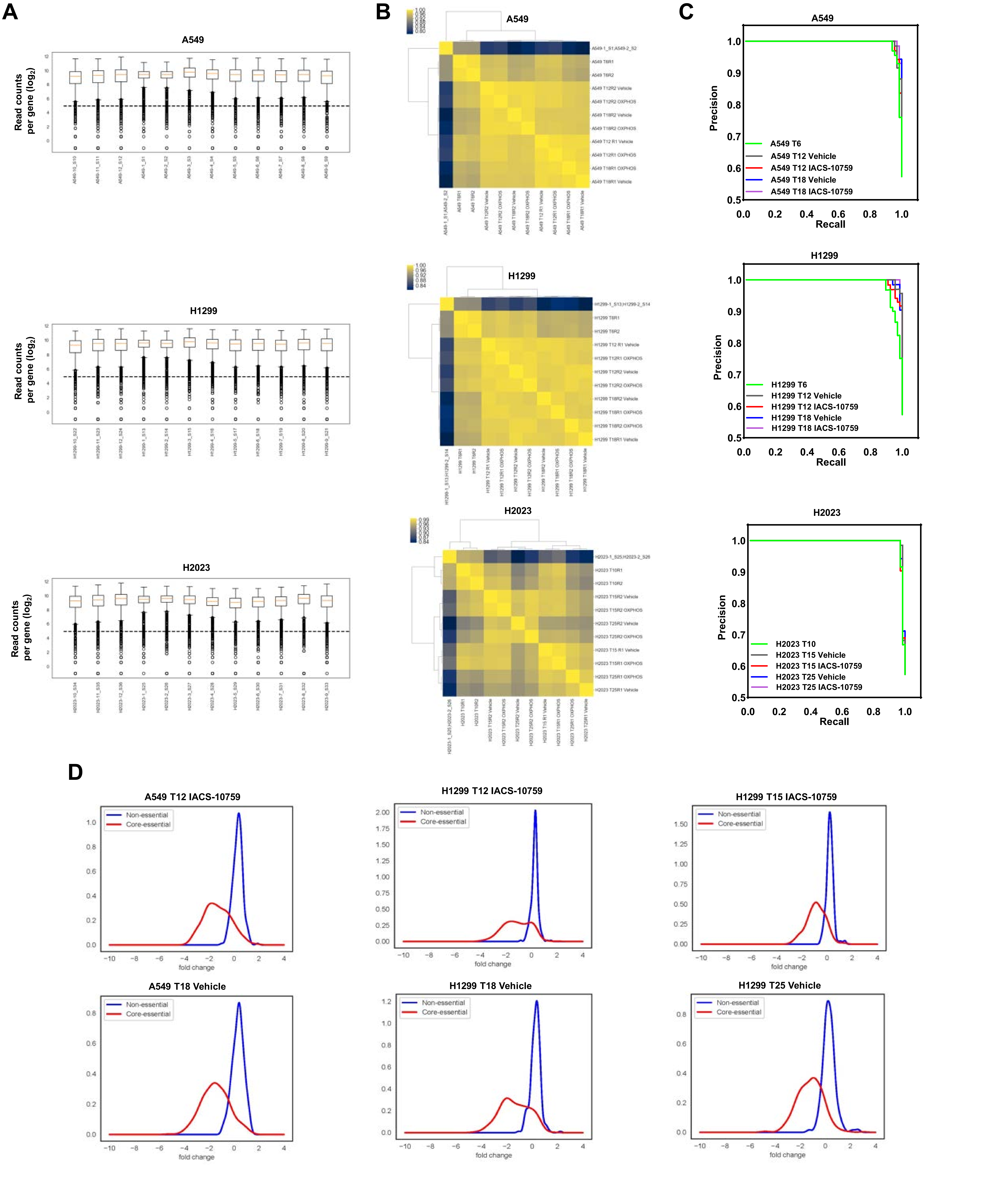

Supplementary Figure 1

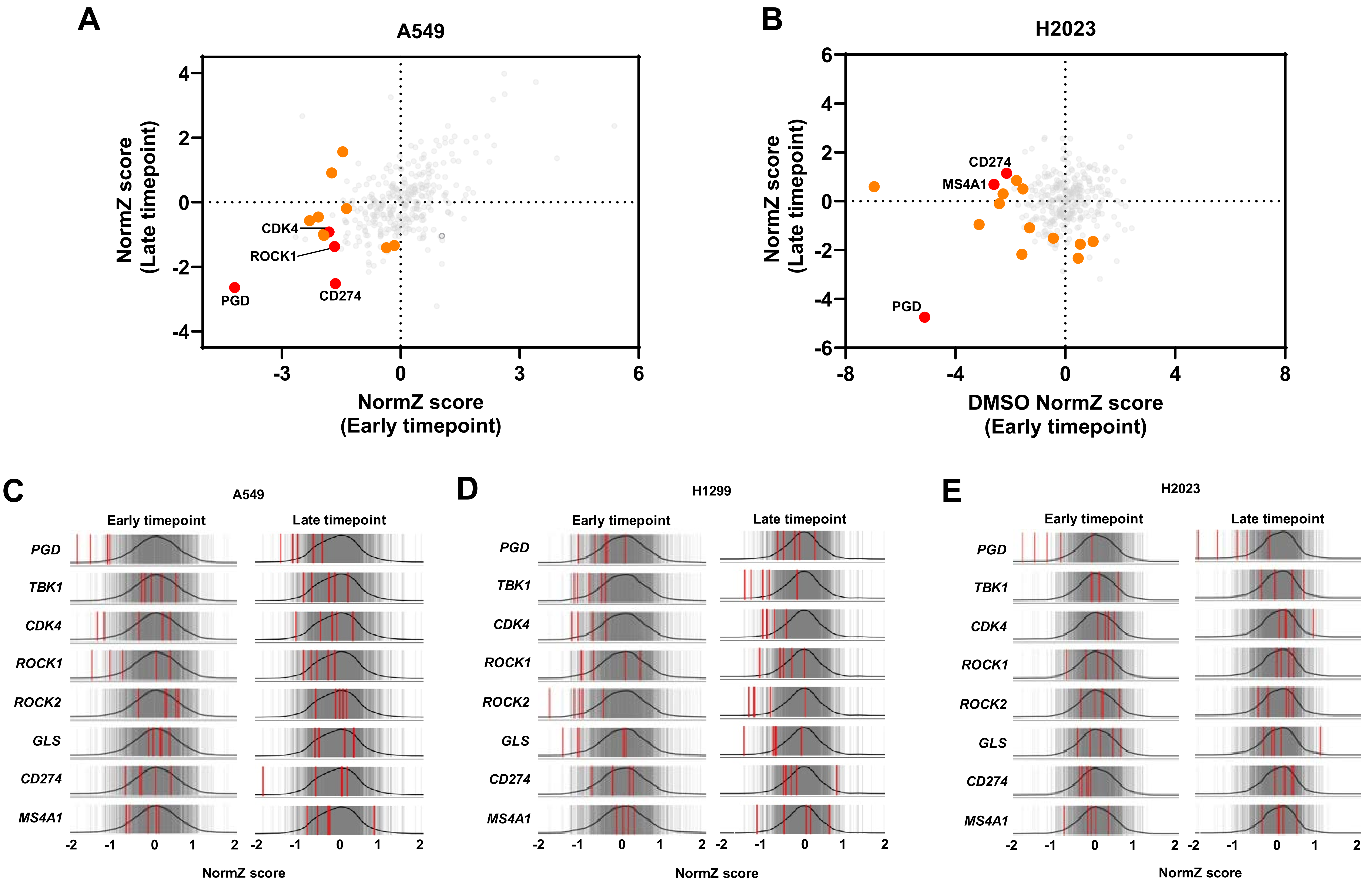

Supplementary Figure 2

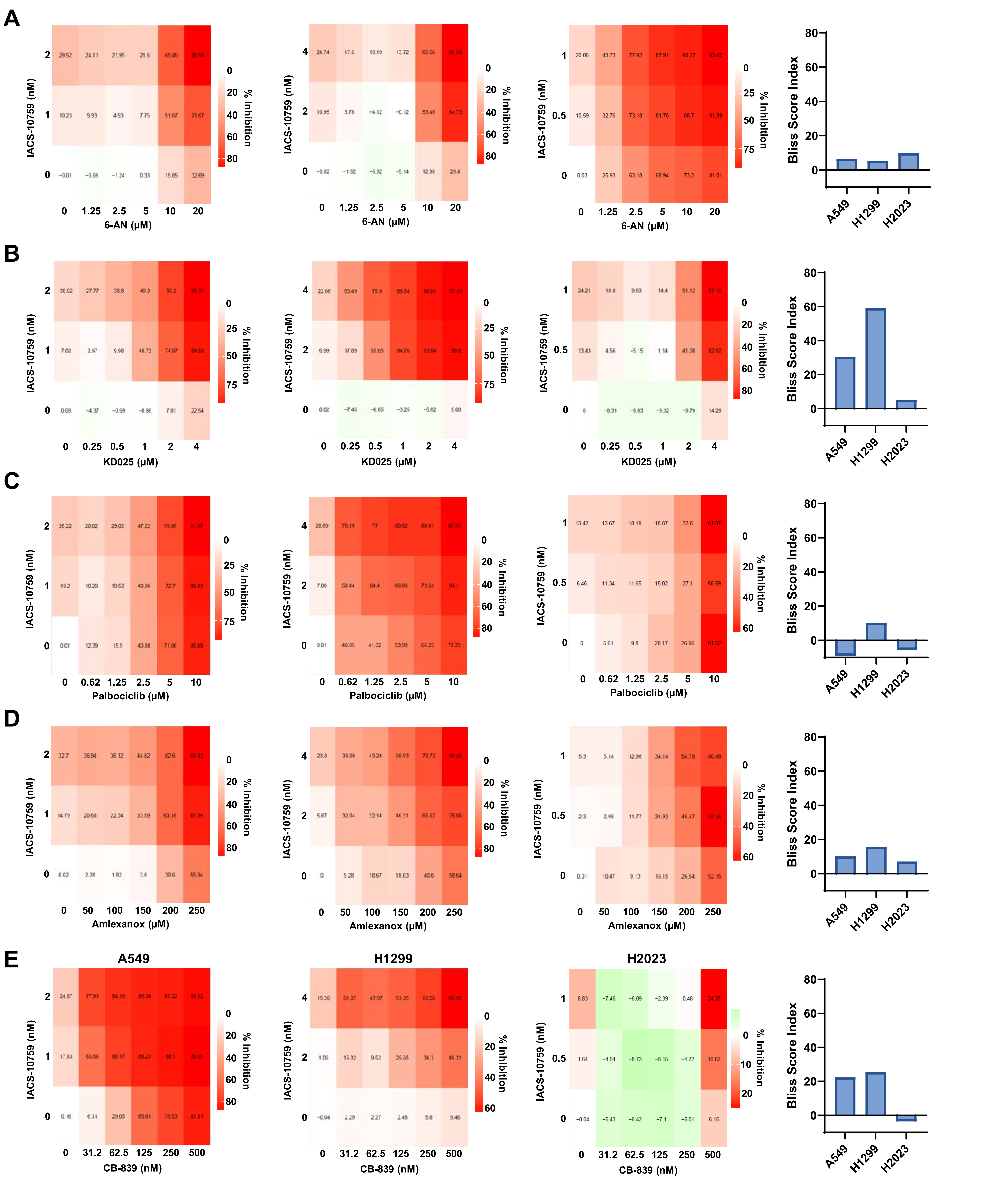

Supplementary Figure 3

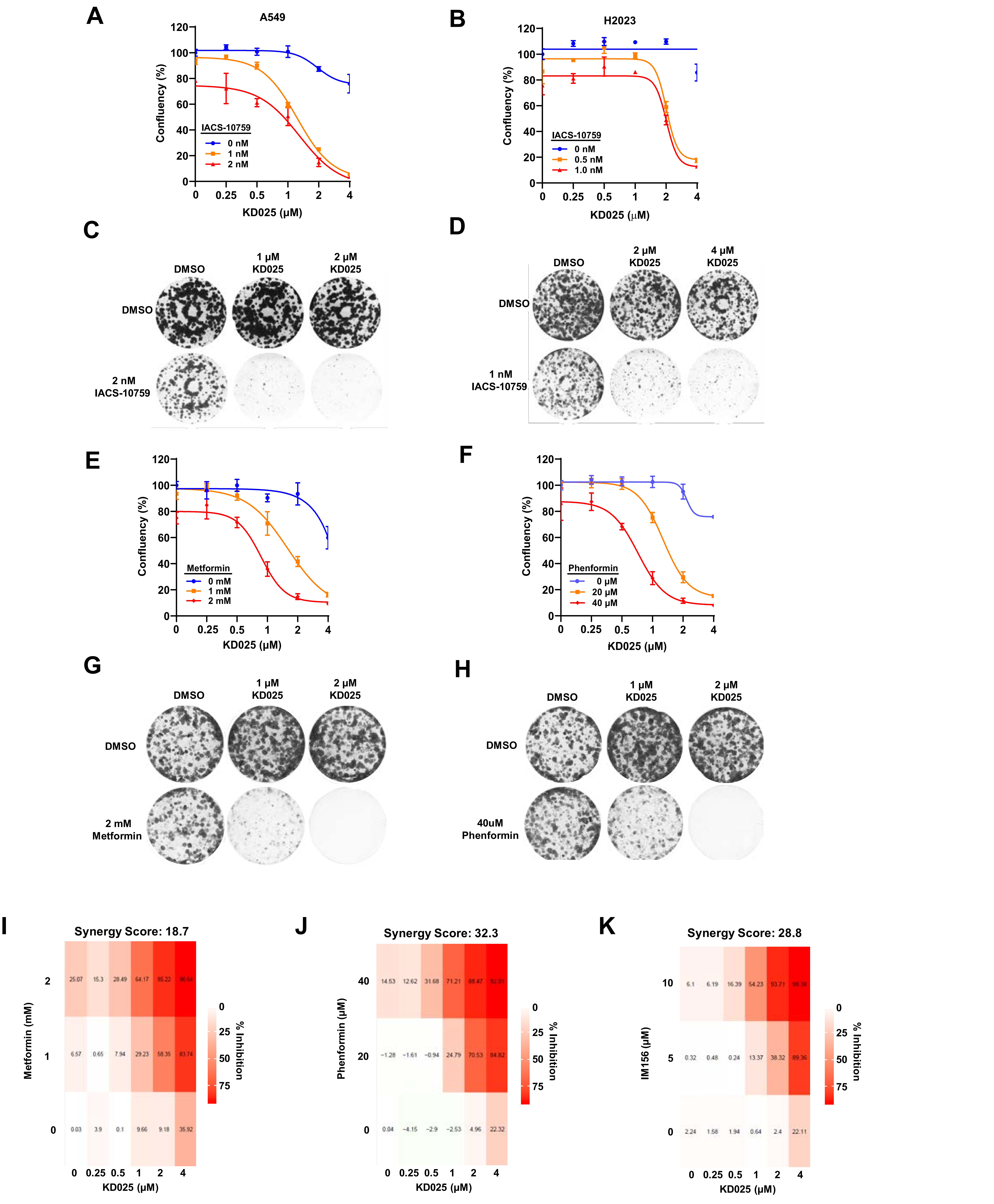

Supplementary Figure 4

A

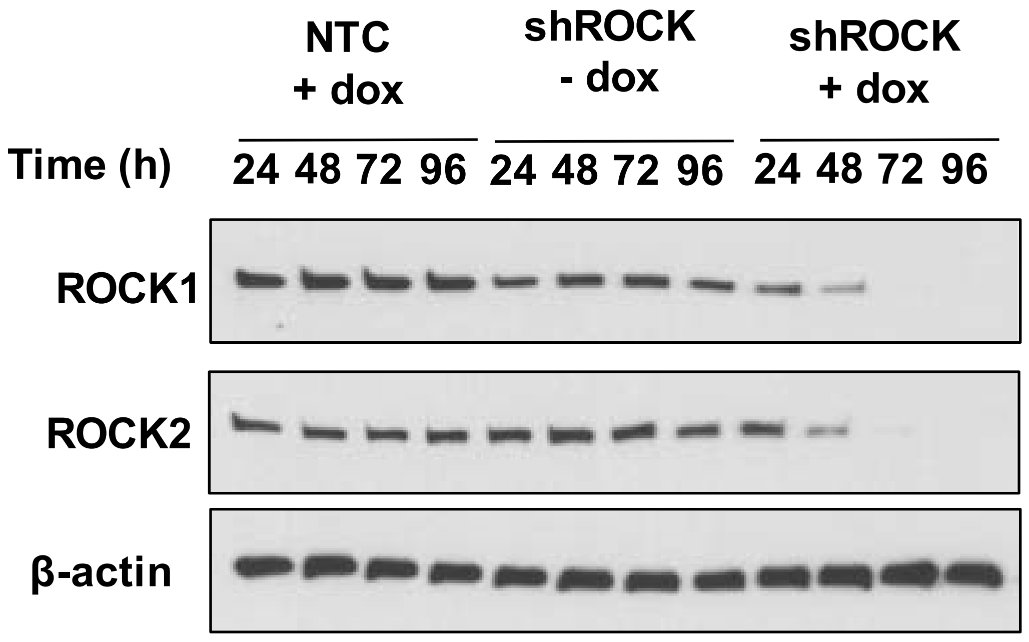

B

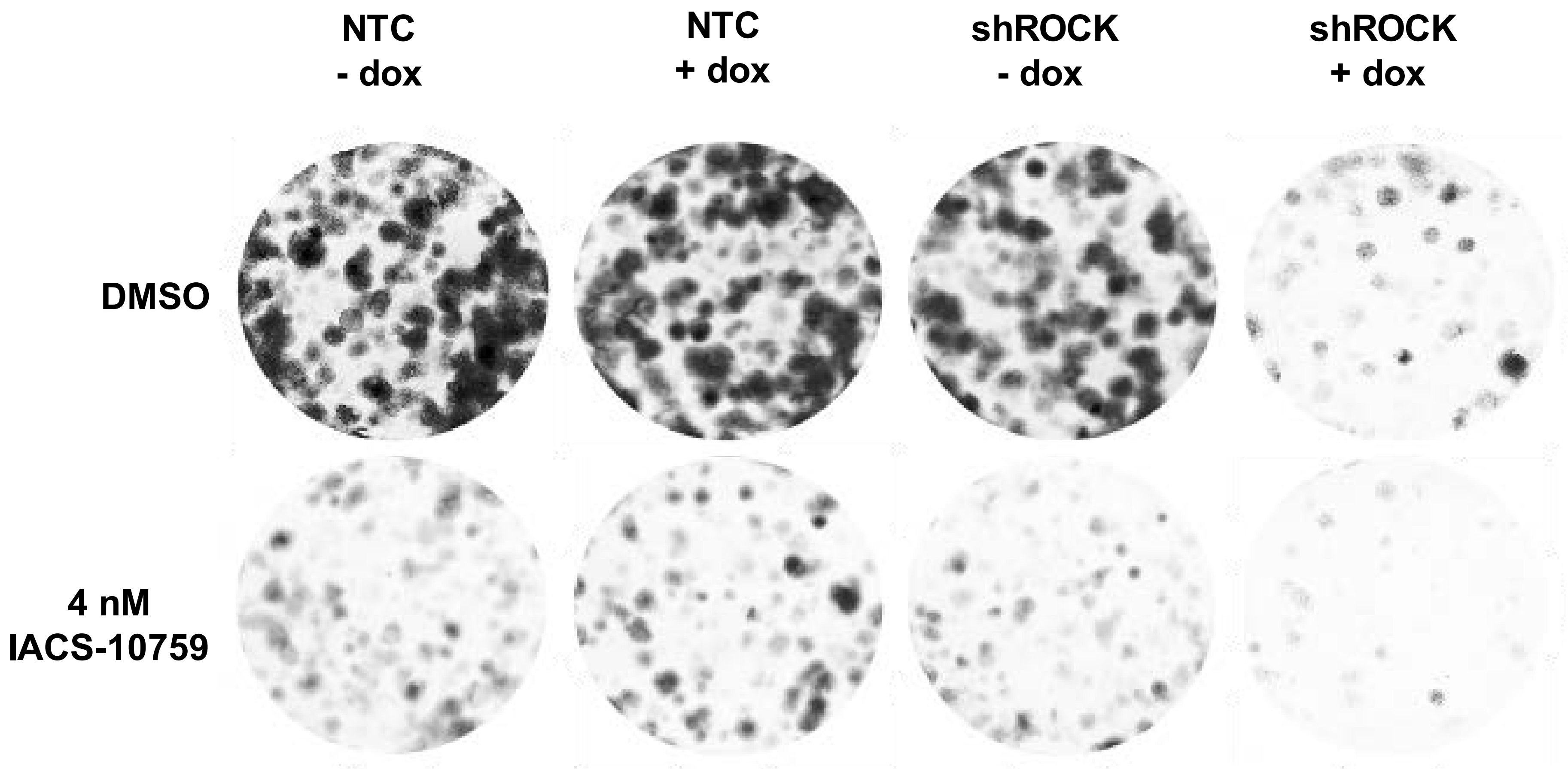

Supplementary Figure 5

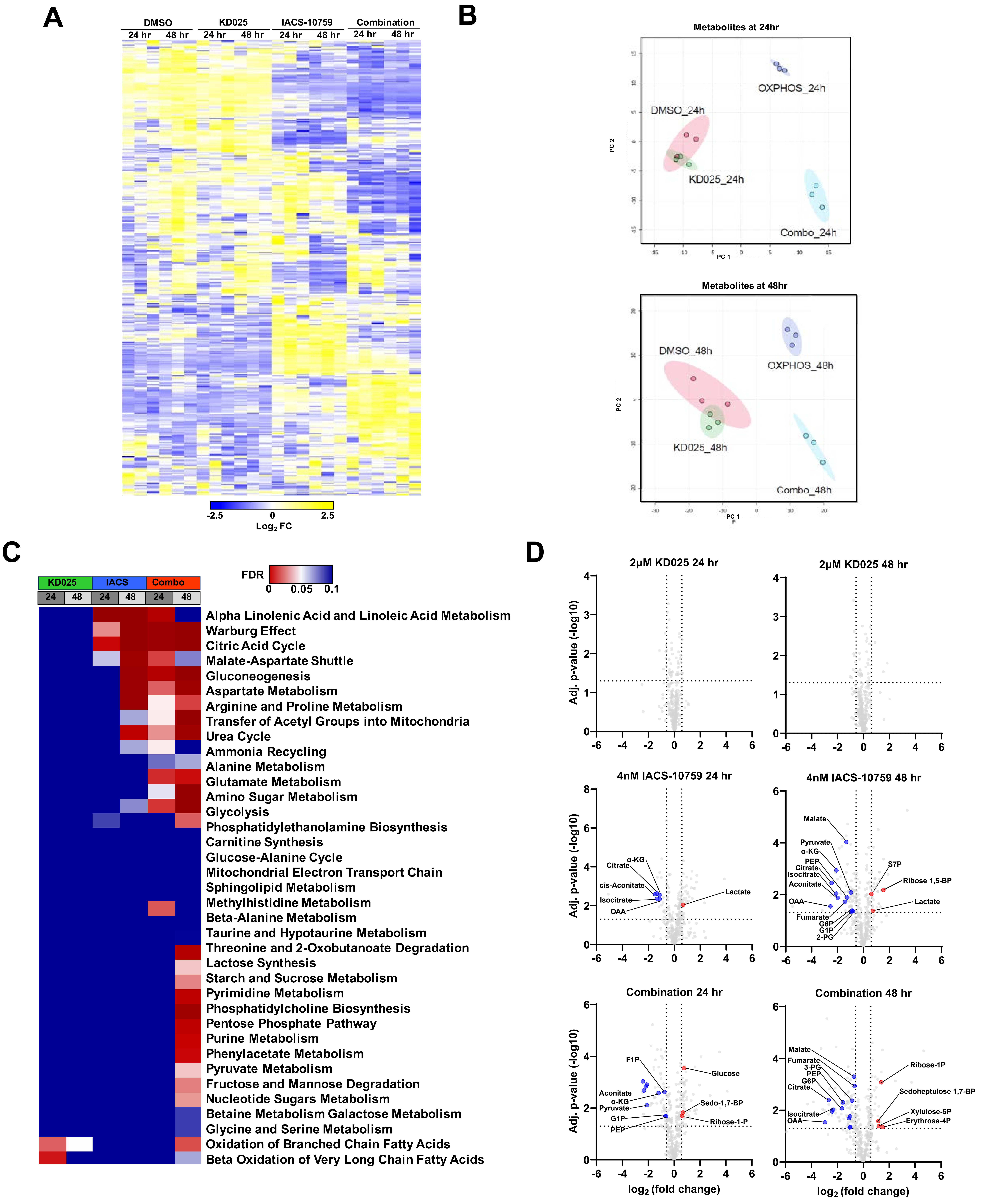

Supplementary Figure 6

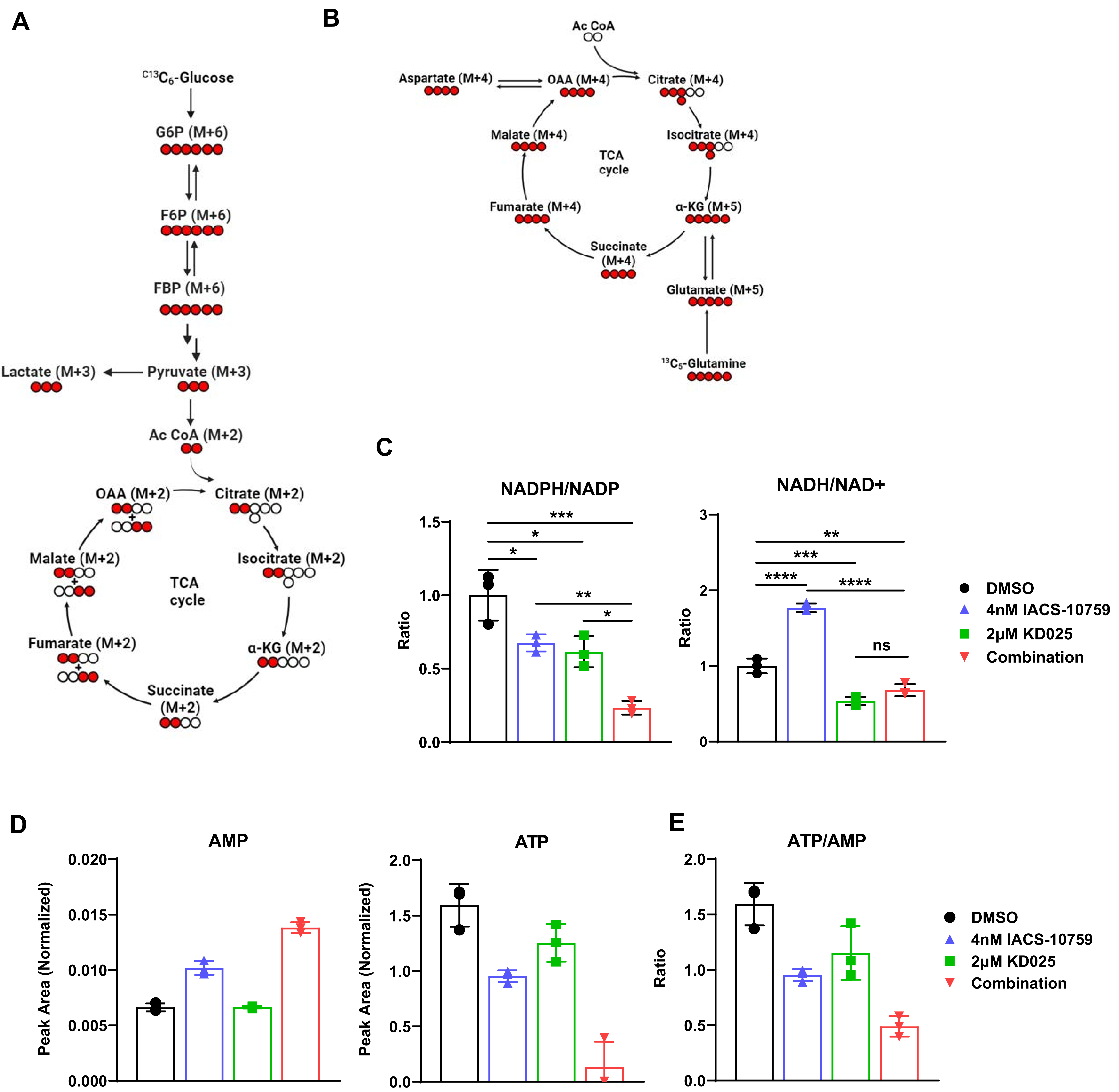

Supplementary Figure 7

A

Proteome

9,050	Total
1	Changed by IACS-10759
0	Changed by KD025
229	Changed by Combination

B

Phosphoproteome

Quantified	Changed by IACS-10759	Changed by KD025	Changed by Combination	
55,376	87	296	9,927	Phosphopeptides
17,539	27	94	3,733	Phosphosites
4,474	22	87	1,822	Phosphoproteins

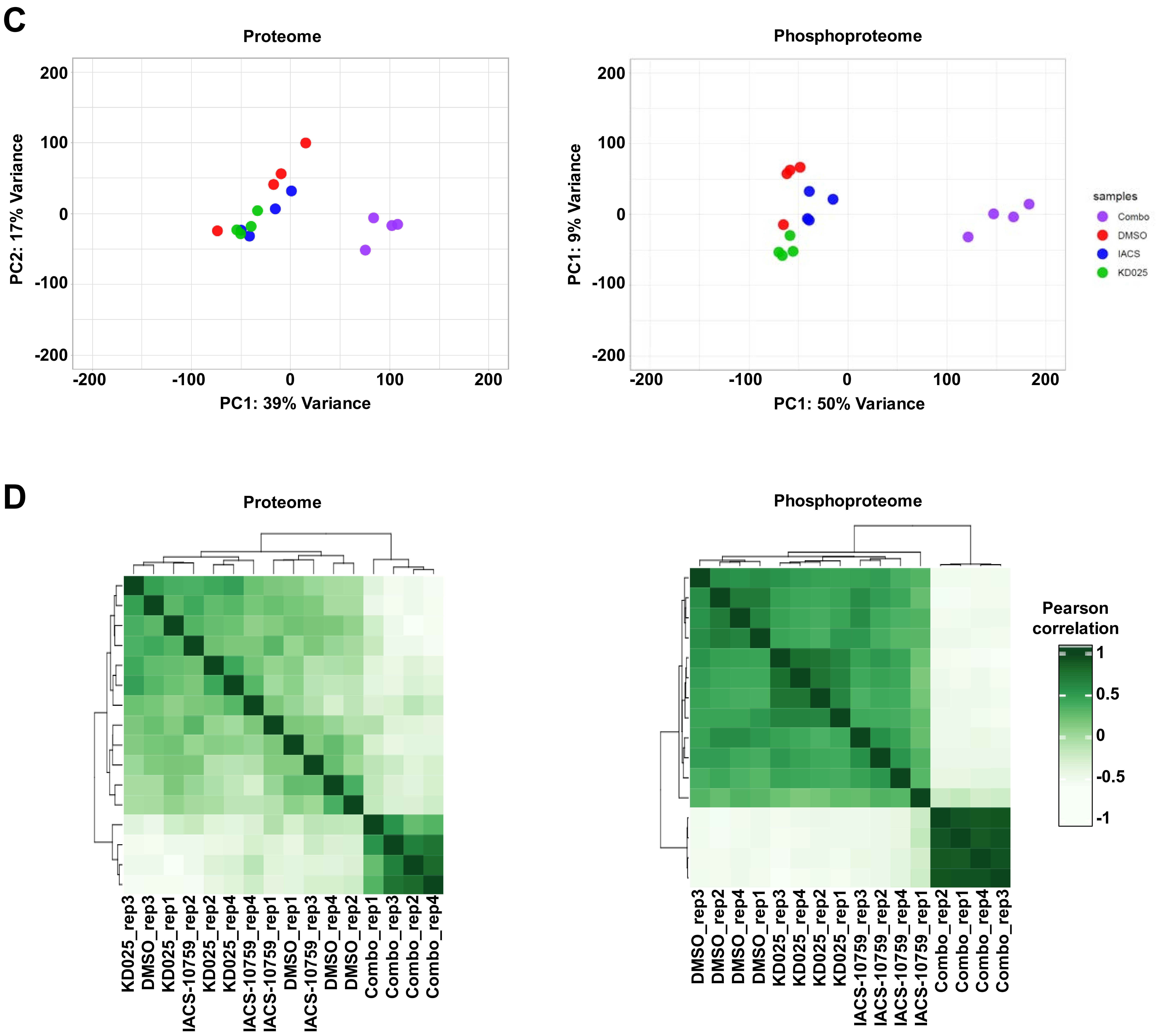

Supplementary Figure 8

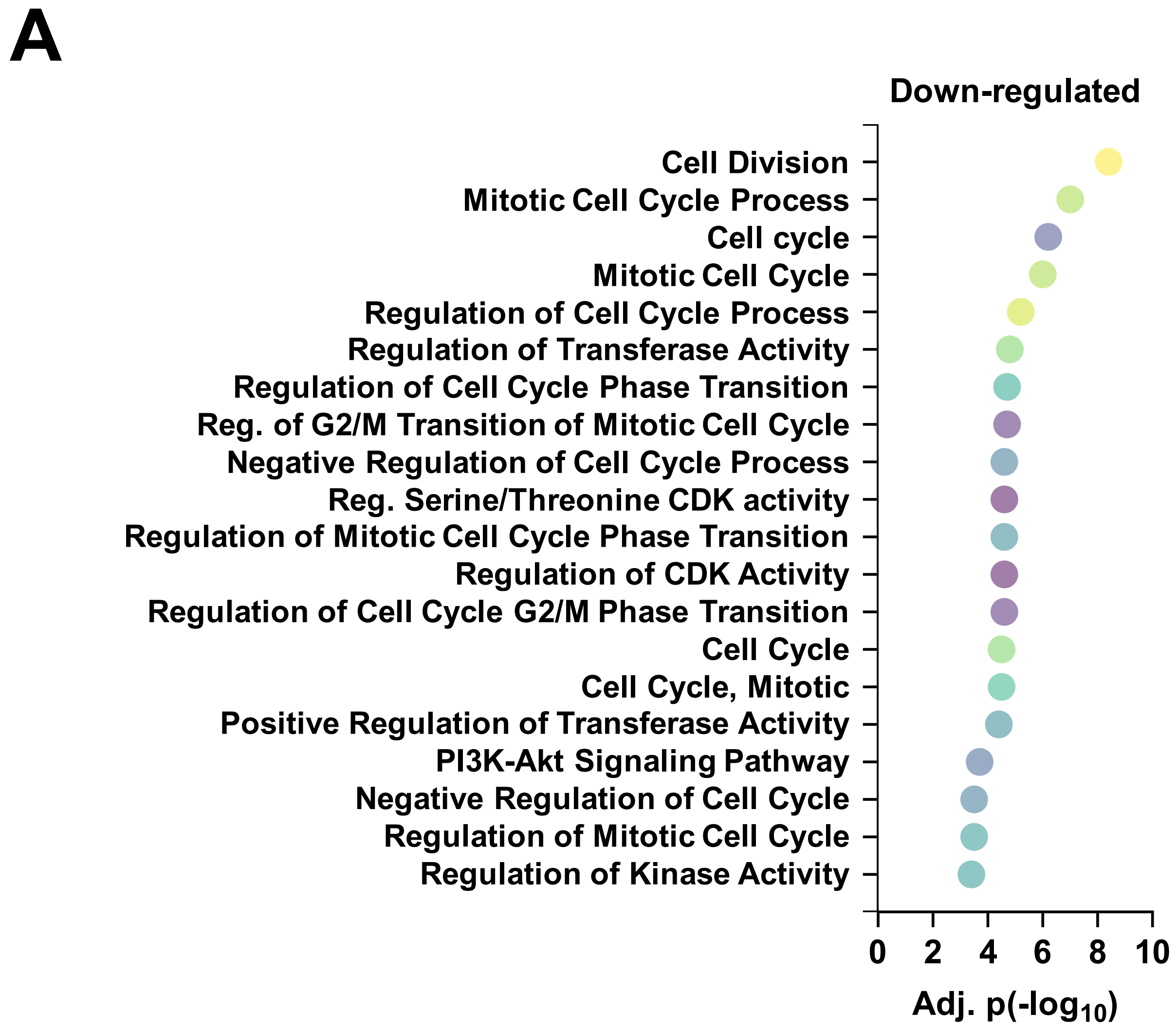

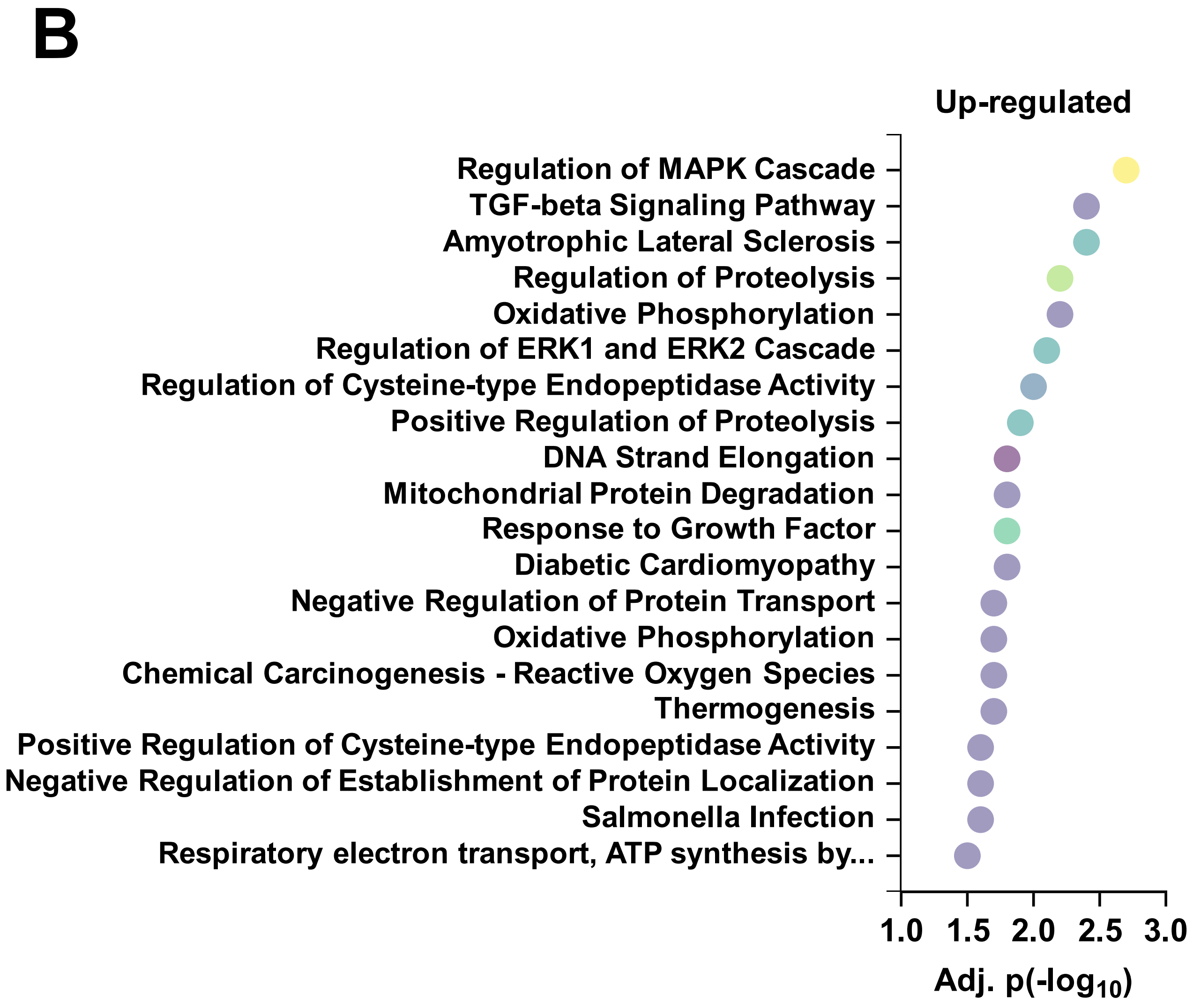

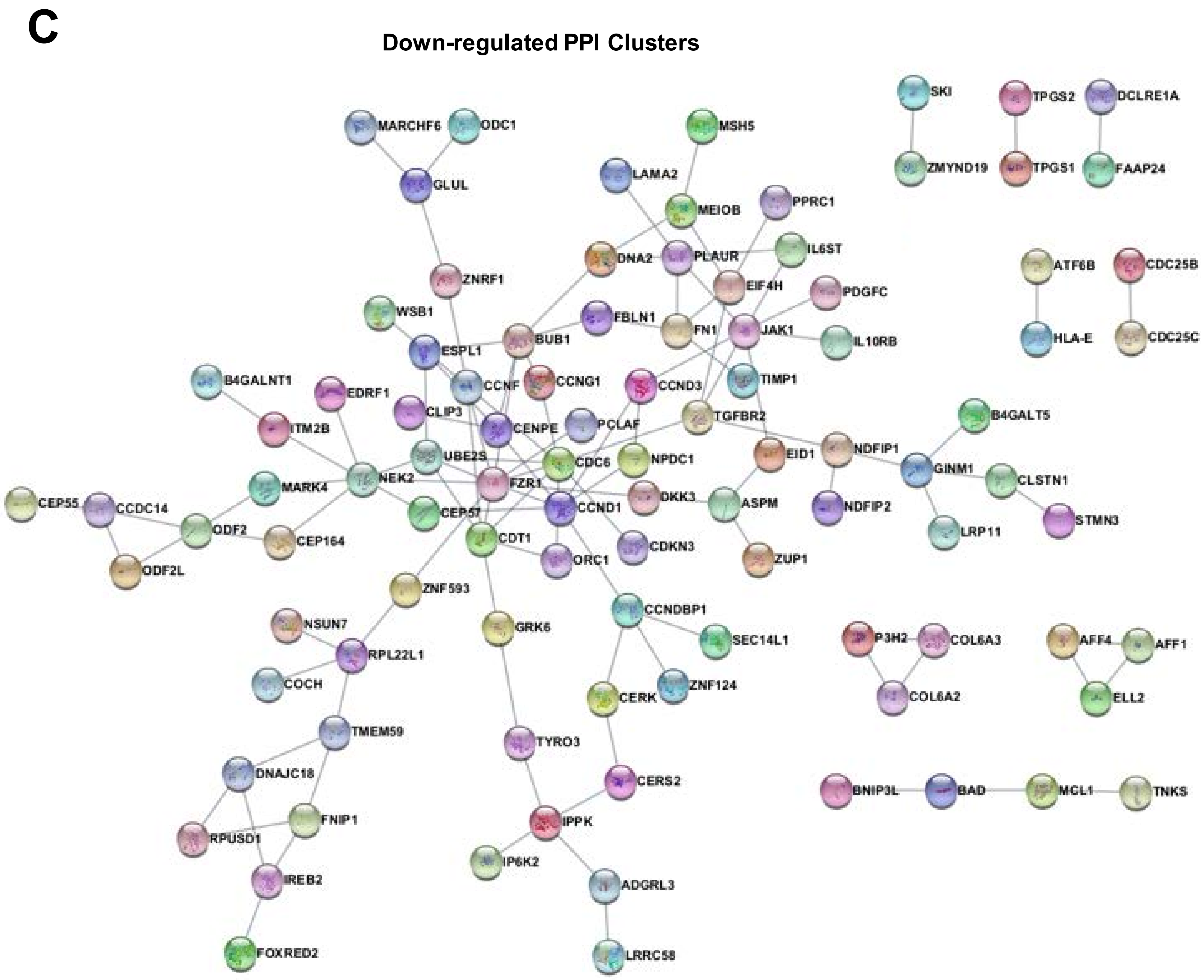

Reactome
1. Cell Cycle, Mitotic; 2. Cell Cycle; 3. S Phase

KEGG
1. Cell Cycle; 2. PI3K-Akt Signaling Pathway; 3. Focal Adhesion

GOBP
1. Cell Division; 2. Mitotic Cell Cycle Process; 3. Mitotic Cell Cycle

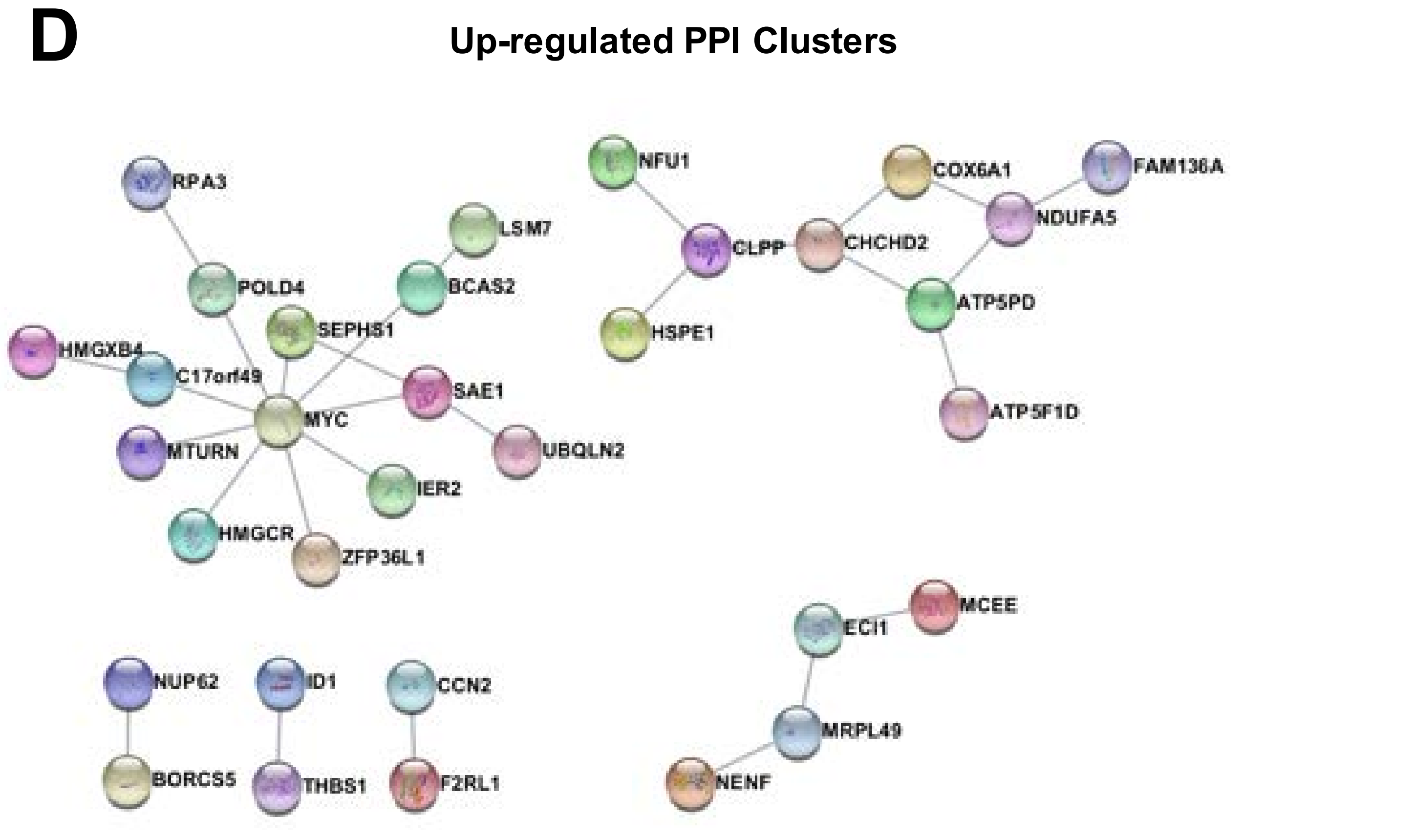

Reactome
1. Mitochondrial Protein Degradation; 2. Respiratory Electron Transport, ATP synthesis by Chemiosmotic Coupling, and Heat Production by Uncoupling Proteins; 3. Aerobic Respiration and Respiratory Electron Transport

KEGG
1. Amyotrophic lateral sclerosis; 2. Oxidative phosphorylation; 3. Diabetic cardiomyopathy

GOBP
1. Regulation of MAPK cascade; 2. Oxidative phosphorylation; 3. Response to growth factor

Supplementary Figure 9

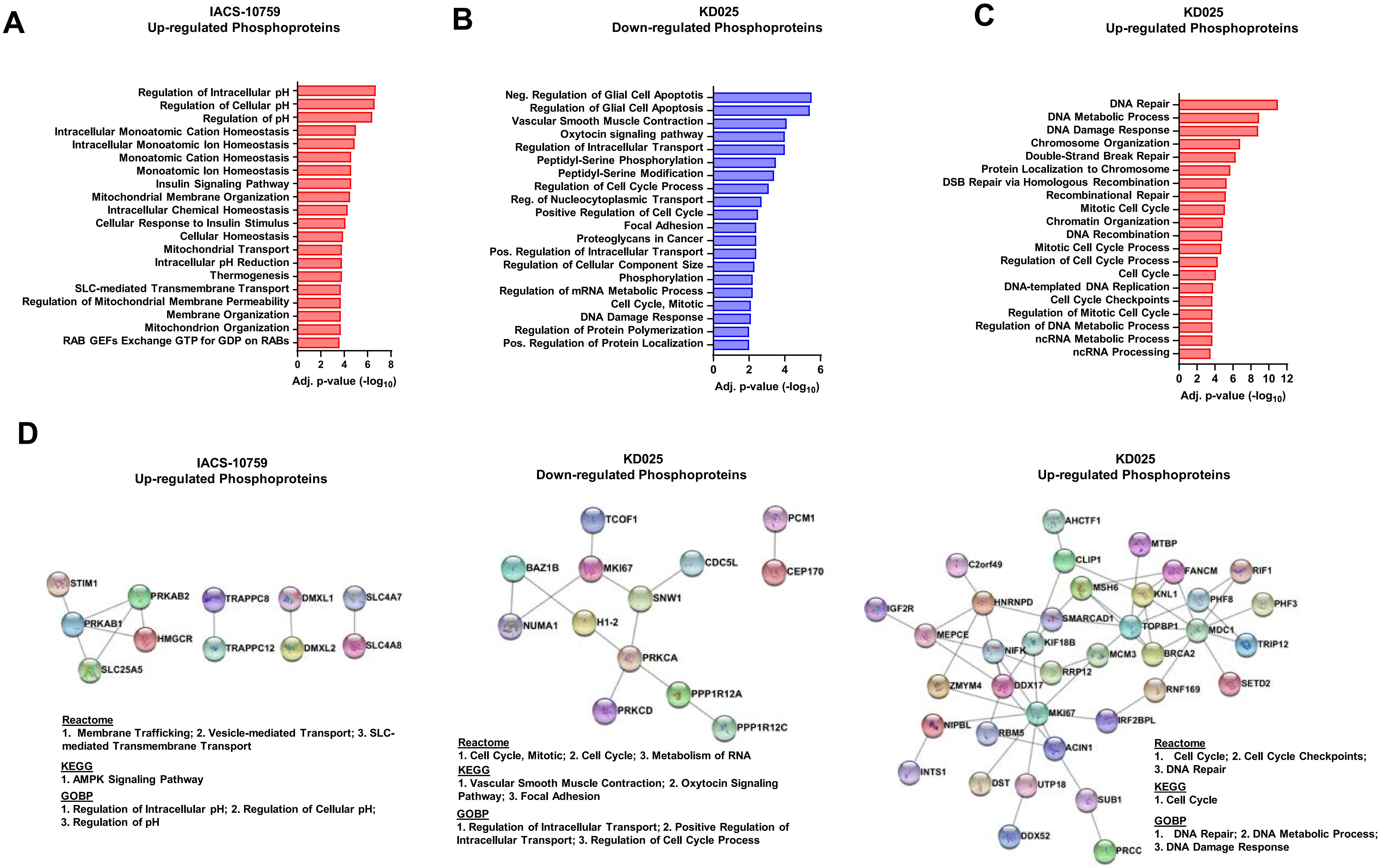

Supplementary Figure 10

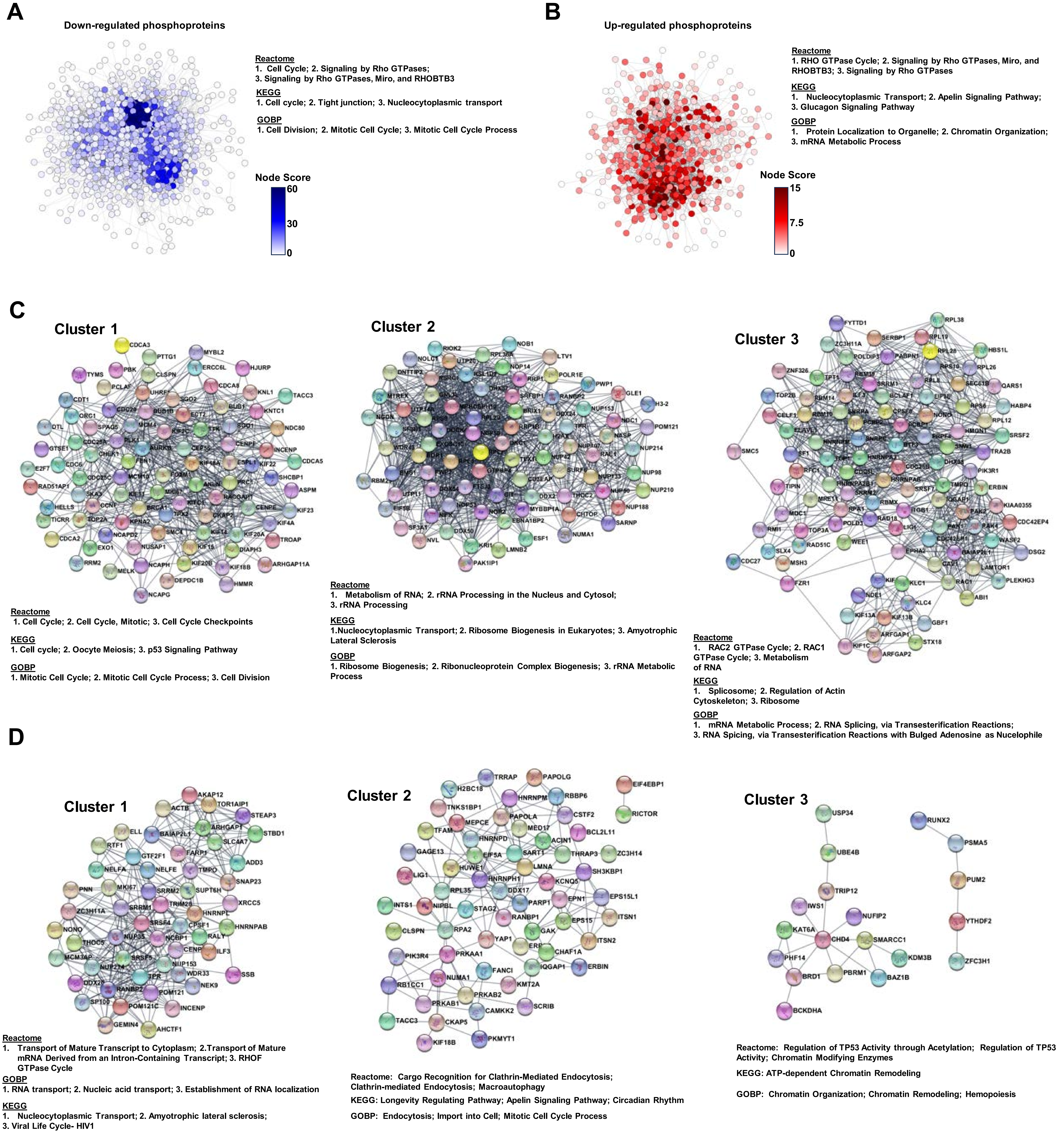

Supplementary Figure 11

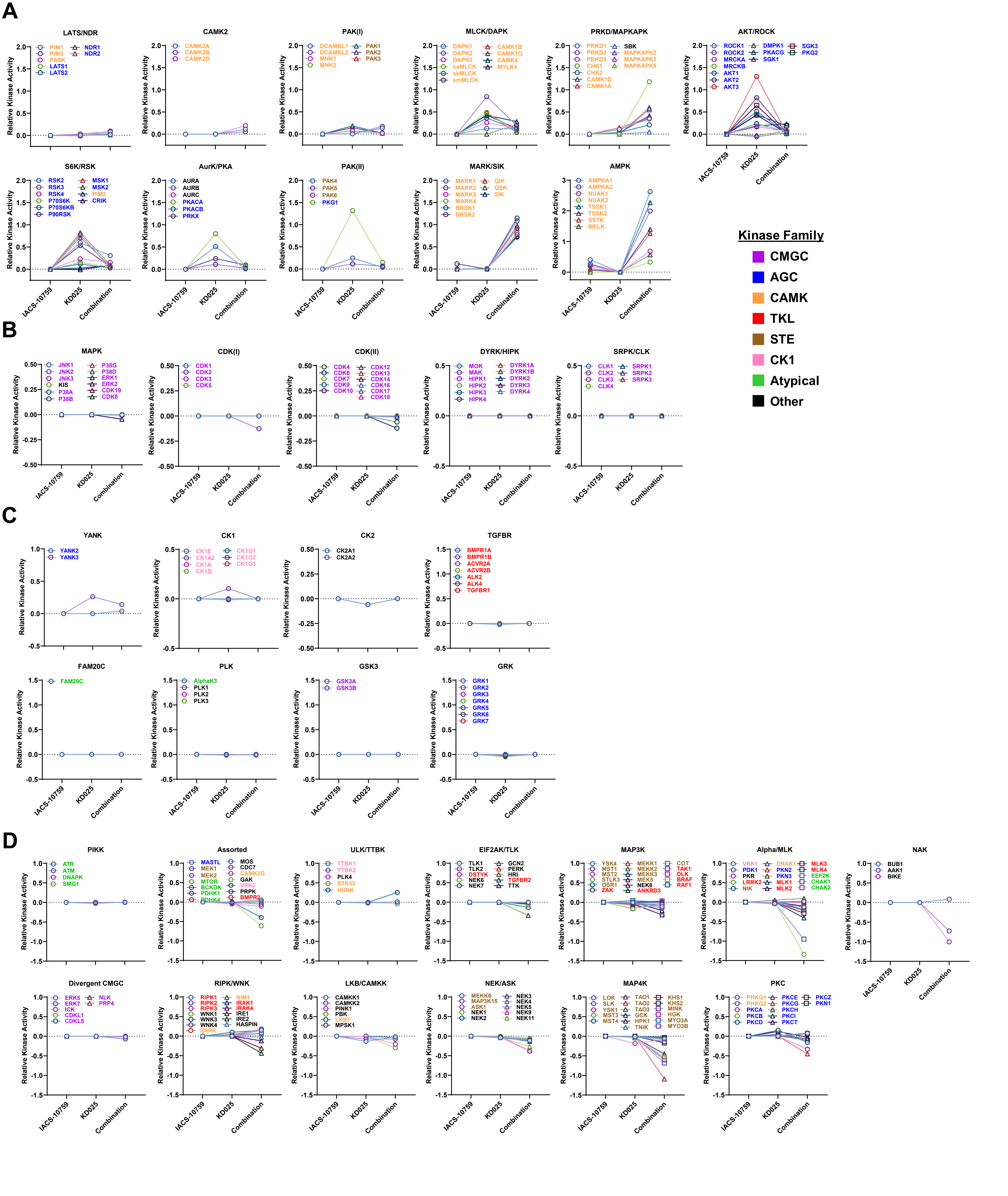

Supplementary Figure 12

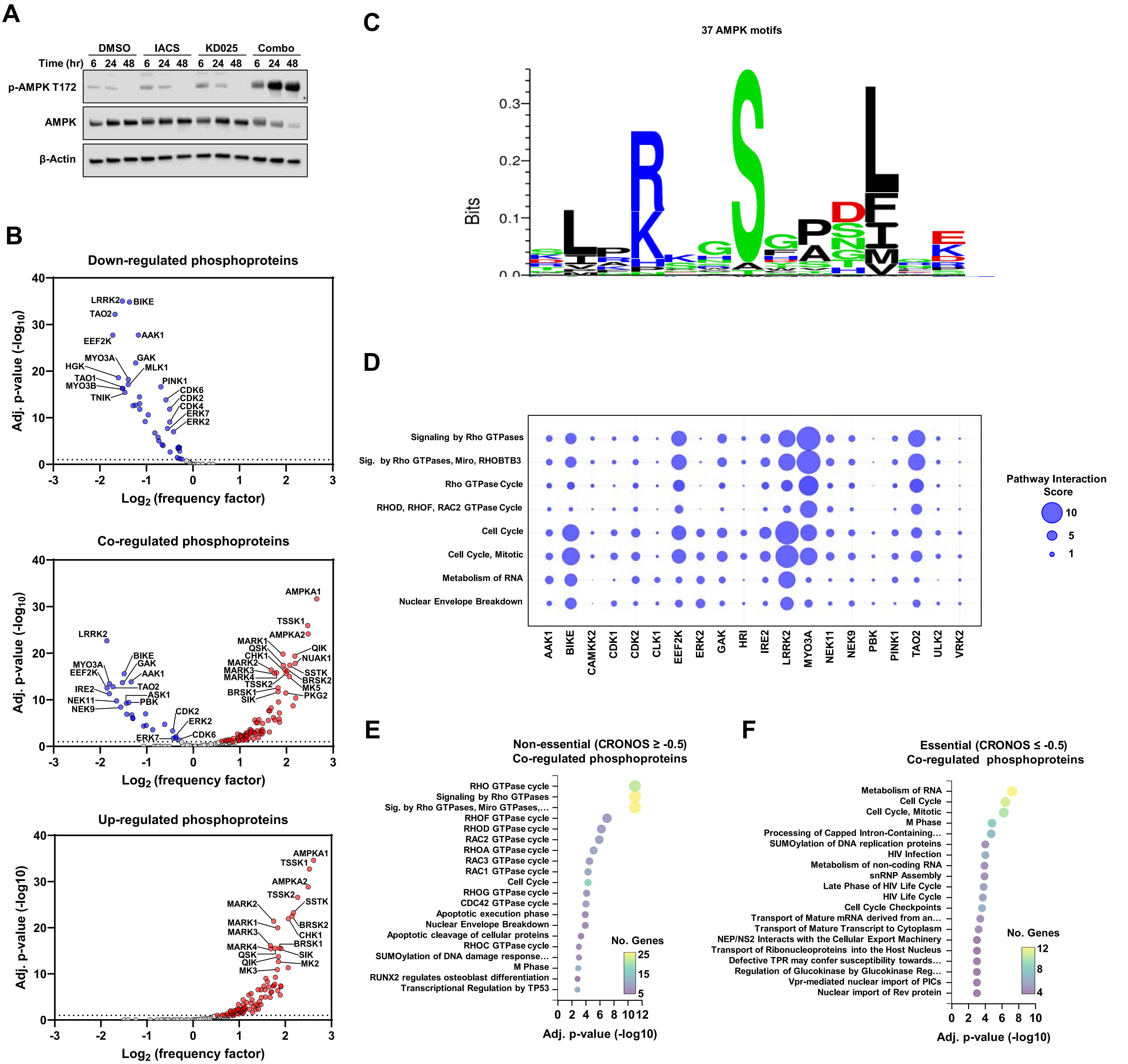

Supplementary Figure 13

Gene Symbol	guide RNA	gRNA strand position	Target domain
AARS	AGAGCCCAGCATCTCGAAGA	chr16:70310893-70310912_AARS_+	exon_03
AARS	GTAGGAATTGGCATGTAAGA	chr16:70310519-70310538_AARS_-	exon_04
AARS	GAAGGATAACTTCTGGGAGA	chr16:70305823-70305842_AARS_-	exon_05
AARS	GGCCTGACAACACAGGGCGT	chr16:70303523-70303542_AARS_-	exon_07
AARS	GGGCGTATCGGACAGCTCGG	chr16:70302239-70302258_AARS_+	exon_08
ABL1	TGGGCTGCAAATCCAAGAAG	chr9:133710859-133710878_ABL1_+	exon_01
ABL1	CATTGCGGGACACAGGCCCA	chr9:133730321-133730340_ABL1_-	exon_03
ABL1	GAAGAAATACAGCCTGACGG	chr9:133738383-133738402_ABL1_+	exon_04
ABL1	CAAGAACTCTTCCACCTCCA	chr9:133747526-133747545_ABL1_-	exon_05
ABL1	TCAGTGATGATATAGAACGG	chr9:133748267-133748286_ABL1_-	exon_06
ABL2	GAAGCTTTGCATCGTCCCTA	chr1:179100598-179100617_ABL2_-	exon_03
ABL2	GCGAGATGGACAGCTGCCCA	chr1:179095568-179095587_ABL2_+	exon_04
ABL2	TGTACACCATCACTCCACAG	chr1:179090939-179090958_ABL2_-	exon_05
ABL2	TTTCTAGGAAGATACCATGG	chr1:179089397-179089416_ABL2_-	exon_06
ABL2	TAATCCAGCAAATTCCCGTA	chr1:179087830-179087849_ABL2_+	exon_07
ADH7	GAAGCAACCCTTCTCCATTG	chr4:100350736-100350755_ADH7_-	exon_02
ADH7	ATCTGTGCGACAGATTCCTG	chr4:100349758-100349777_ADH7_+	exon_03
ADH7	AACAGCAGCGCCATATCCAG	chr4:100348942-100348961_ADH7_+	exon_05
ADH7	GAAATGACAGGCAACAACGT	chr4:100341730-100341749_ADH7_-	exon_06
ADH7	TAGGTGAGCATCTTGGCTGA	chr4:100340196-100340215_ADH7_+	exon_07
ADORA2A	CTCCTCGGTGTACATCACGG	hr22:24829387-24829406_ADORA2A_	exon_02
ADORA2A	AGCACACCAGCACATTGCCC	hr22:24829438-24829457_ADORA2A_	exon_02
ADORA2A	AGGCAATGAAGAGGCAGCCG	hr22:24829597-24829616_ADORA2A_	exon_02
ADORA2A	GAGGGCAAGAACCACTCCCA	hr22:24836669-24836688_ADORA2A_	exon_03
ADORA2A	GGAGAGCCAGCCTCTGCCGG	hr22:24836851-24836870_ADORA2A_	exon_03
AKT1	GACGTGGCTATTGTGAAGGA	chr14:105258955-105258974_AKT1_-	exon_01
AKT1	GCAGGATGTGGACCAACGTG	chr14:105246455-105246474_AKT1_-	exon_02
AKT1	GAAGGTGCGTTCGATGACAG	chr14:105243019-105243038_AKT1_+	exon_03
AKT1	GGAGGAGATGGACTTCCGGT	chr14:105242060-105242079_AKT1_-	exon_04
AKT1	GGGCAAGGGCACTTTCGGCA	chr14:105241493-105241512_AKT1_-	exon_05
AKT2	TGGGTACAAGGAGAGGCCCG	chr19:40762881-40762900_AKT2_-	exon_03
AKT2	CATCGAGAGGACCTTCCACG	chr19:40761084-40761103_AKT2_-	exon_04
AKT2	GTGGATGCGGGCCATCCAGA	chr19:40748569-40748588_AKT2_-	exon_05
AKT2	TATCTCAAACTCCTTGGCAA	chr19:40747939-40747958_AKT2_-	exon_06
AKT2	CCGGGAGCGTGTCTTCACAG	chr19:40743962-40743981_AKT2_-	exon_09
AKT3	AACCCAACCTTCTTTCACAA	chr1:244006437-244006456_AKT3_+	exon_02
AKT3	GAGAGAGCGGGTGTTCTCTG	chr1:243736314-243736333_AKT3_-	exon_09
AKT3	ATGGAACCGAAGCCTACCTC	chr1:243727005-243727024_AKT3_+	exon_10
AKT3	CATCATCTGGTCCTCCACCA	chr1:243708877-243708896_AKT3_+	exon_12
AKT3	ACTGCATGGACAATGAGAGG	chr1:243668599-243668618_AKT3_-	exon_14
ALK	GATGCCCGAGAAGAAGGCGT	chr2:30142940-30142959_ALK_-	exon_01
ALK	CATCTGGGAGGCCTCCTCGG	chr2:29917765-29917784_ALK_+	exon_03
ALK	TGGGCAATGTACCTTCCAGA	chr2:29754844-29754863_ALK_+	exon_04
ALK	AAACCGCAGCTTGTCTGCAG	chr2:29606631-29606650_ALK_-	exon_05
ALK	AATGGGACAGTCCTCCAGCT	chr2:29551278-29551297_ALK_-	exon_06
APOBEC1	ATAGAAGACGTCAAACTCCC	chr12:7805395-7805414_APOBEC1_+	exon_03
APOBEC1	TAGAAGACGTCAAACTCCCA	chr12:7805396-7805415_APOBEC1_+	exon_03
APOBEC1	CAGGAGAAGAATCGAACCCT	chr12:7805415-7805434_APOBEC1_-	exon_03
APOBEC1	TCAGGAGAAGAATCGAACCC	chr12:7805416-7805435_APOBEC1_-	exon_03
APOBEC1	TACAACATCATCCACAGAGG	chr12:7803647-7803666_APOBEC1_+	exon_04

ARCN1	GGTGTTCAGAGCCGTCAGAG	hr11:118454052-118454071_ARCN1_	exon_03
ARCN1	AGTACGCTTGCCCATACTAG	:hr11:118455296-118455315_ARCN1_	exon_05
ARCN1	CAGCCATTTCCACTCTCCGA	:hr11:118464319-118464338_ARCN1_	exon_08
ARCN1	ACAGCCATTTCCACTCTCCG	:hr11:118464320-118464339_ARCN1_	exon_08
ARCN1	GTCGACGAAATACCCTGGAG	hr11:118468478-118468497_ARCN1_	exon_09
AR	CAGCAGGAAGCAGTATCCGA	chrX:66765580-66765599_AR_+	exon_01
AR	TCTCTGCTAGACGACAGCGC	chrX:66765865-66765884_AR_+	exon_01
AR	AGAGAGACAGCTTGTACACG	chrX:66931485-66931504_AR_+	exon_04
AR	GGTGAAGGATCGCCAGCCCA	chrX:66937395-66937414_AR_-	exon_05
AR	TCATGCACAGGAATTCCTGG	chrX:66941762-66941781_AR_-	exon_06
ATM	GATGGCAGATATCTGTCACC	chr11:108117833-108117852_ATM_+	exon_09
ATM	GACACAATGCAACTTCCGTA	chr11:108121569-108121588_ATM_-	exon_11
ATM	AAAGTCAAACAGCATACTGC	chr11:108122568-108122587_ATM_-	exon_12
ATM	TTGCTTGAGCAACTGTACCA	chr11:108128312-108128331_ATM_+	exon_16
ATM	GGAGAGAGCCAAAGTACCAT	chr11:108138048-108138067_ATM_+	exon_18
ATP6V1A	TCTCTCCAACCAATTCGCTG	hr3:113499955-113499974_ATP6V1A_	exon_03
ATP6V1A	GGTTACACAGACATACCCGT	hr3:113503282-113503301_ATP6V1A_	exon_04
ATP6V1A	GAGGTATCATAATTCCCAGG	hr3:113503660-113503679_ATP6V1A_	exon_05
ATP6V1A	TGTAAAGGAGAAGTTCACCA	ır3:113505108-113505127_ATP6V1A_	exon_06
ATP6V1A	AGACCTCTGGGAAGTCCCGG	hr3:113507707-113507726_ATP6V1A_	exon_07
ATR	GAAATCAAGCAACATCACGG	chr3:142285042-142285061_ATR_+	exon_03
ATR	CTTGTGTAACAAATGACAGG	chr3:142281881-142281900_ATR_+	exon_04
ATR	GTGATGGAATATCACCCAAA	chr3:142280144-142280163_ATR_-	exon_05
ATR	TGTTCAGAAACTGGACCTGG	chr3:142278147-142278166_ATR_-	exon_07
ATR	GCTGACTCTTACCTGACAGA	chr3:142272057-142272076_ATR_+	exon_13
AURKA	GCTAGTTTACCAGGTGCCGA	chr20:54961303-54961322_AURKA_+	exon_03
AURKA	TTACCAGGTGCCGATGGCAG	chr20:54961309-54961328_AURKA_+	exon_03
AURKA	TCTGAGCTGATGCTCCACTC	chr20:54958070-54958089_AURKA_+	exon_05
AURKA	CTTCGAATGACAGTAAGACA	chr20:54948568-54948587_AURKA_+	exon_07
AURKA	TTCAGGAGGACCACTCTCTG	chr20:54945701-54945720_AURKA_-	exon_08
AURKB	GGGTGACAGGCTCTTTCCGG	chr17:8111101-8111120_AURKB_+	exon_03
AURKB	TGACGAGCAGCGAACAGCCA	chr17:8110070-8110089_AURKB_-	exon_06
AURKB	AGGCCACCATACCTCAGGGA	chr17:8109797-8109816_AURKB_+	exon_07
AURKB	TGTGATGCACTCTCAAAGGG	chr17:8108562-8108581_AURKB_+	exon_08
AURKB	GGTCCGGGCCAACTCTCGGA	chr17:8108227-8108246_AURKB_-	exon_09
AURKC	AGATGAACAGCGCACAGCCA	chr19:57744027-57744046_AURKC_+	exon_04
AURKC	TGGGAGGCGCTCTCAAAGGG	chr19:57746379-57746398_AURKC_-	exon_06
AURKC	ACTGTGGGAGGCGCTCTCAA	chr19:57746383-57746402_AURKC_-	exon_06
AURKC	TCGGGAGTGGGCCTGAACCC	chr19:57746730-57746749_AURKC_-	exon_07
AURKC	GAAGCCATCTGAGCACAGGG	chr19:57746762-57746781_AURKC_-	exon_07
AXL	AGCGCCAAGCACCAGGCCAG	chr19:41725334-41725353_AXL_-	exon_01
AXL	GAACCTGGAGCTGACACCGA	chr19:41726617-41726636_AXL_-	exon_02
AXL	CGTGTCGGAAAGCTGCAGGG	chr19:41727059-41727078_AXL_-	exon_03
AXL	TGACCTGGAGCCGTGGCCAG	chr19:41727913-41727932_AXL_-	exon_04
AXL	CTGCGAAGCCCATAACGCCA	chr19:41736897-41736916_AXL_+	exon_05
BCL2	ACCTGGATCCAGGATAACGG	chr18:60985322-60985341_BCL2_-	exon_01
BCL2	GAAGAGCTCCTCCACCACCG	chr18:60985486-60985505_BCL2_+	exon_01
BCL2	ACATCTCGGCGAAGTCGCGG	chr18:60985554-60985573_BCL2_+	exon_01
BCL2	GTGGCCCAGATAGGCACCCA	chr18:60795864-60795883_BCL2_+	exon_02
BCL2	GGTGATGCAAGCTCCCACCA	chr18:60795885-60795904_BCL2_+	exon_02
BCR	AGAGGAGAAGTCCTCCTCGC	chr22:23524169-23524188_BCR_-	exon_01

BCR	GCAGCGCAGCCGTATCCAGG	chr22:23596006-23596025_BCR_-	exon_02
BCR	GTGGCTCCACTGCTGCACGC	chr22:23603675-23603694_BCR_-	exon_04
BCR	TCAGTTTGCAGAAATCTCCG	chr22:23610681-23610700_BCR_+	exon_05
BCR	GGTCGGTGAACAGGAAGACG	chr22:23626229-23626248_BCR_-	exon_09
BIRC5	GATGCGGTGGTCCTTGAGAA	chr17:76210435-76210454_BIRC5_-	exon_01
BIRC5	CAAGAACTGGCCCTTCTTGG	chr17:76210463-76210482_BIRC5_+	exon_01
BIRC5	GCAGGCGCAGCCCTCCAAGA	chr17:76210477-76210496_BIRC5_-	exon_01
BIRC5	GGGCAGTCTCACCCGCTCCG	chr17:76210501-76210520_BIRC5_-	exon_01
BIRC5	GCAGTGGATGAAGCCAGCCT	chr17:76210768-76210787_BIRC5_-	exon_02
BIRC8	TCAGCCCTGAAGAGCCGCTA	chr19:53793088-53793107_BIRC8_-	exon_01
BIRC8	GAAGCCCAAGGAAGATCCTT	chr19:53793462-53793481_BIRC8_-	exon_01
BIRC8	GGAAGCCCAAGGAAGATCCT	chr19:53793463-53793482_BIRC8_-	exon_01
BIRC8	GCTAGCCAACTGGAAGCCCA	chr19:53793474-53793493_BIRC8_-	exon_01
BIRC8	GTGGAGGAGGGCTAGCCAAC	chr19:53793484-53793503_BIRC8_-	exon_01
BLK	GAAGGTCAGCGCCCAAGACA	chr8:11400811-11400830_BLK_+	exon_02
BLK	TTTAGACAAGCATTTCGTGG	chr8:11405536-11405555_BLK_+	exon_04
BLK	GAAGGATGTCACCACCCAGG	chr8:11412271-11412290_BLK_+	exon_07
BLK	ACCAGCCTGAGAGACTGCCG	chr8:11412933-11412952_BLK_-	exon_08
BLK	GCTGGTCCGACTCTACGCAG	chr8:11414279-11414298_BLK_+	exon_09
BMP15	GCTTCCTTGGCTGTTCGCCA	chrX:50653942-50653961_BMP15_-	exon_01
BMP15	CATGCGAGTCAGCTGAACGC	chrX:50654008-50654027_BMP15_-	exon_01
BMP15	GAAGGCTAAATTTCTTCCCA	chrX:50659160-50659179_BMP15_+	exon_02
BMP15	TGTCTCCGAGTACTACGCGA	chrX:50659398-50659417_BMP15_+	exon_02
BMP15	TACGGGACACAGGAGGGCCG	chrX:50659488-50659507_BMP15_-	exon_02
BMX	ATGAAGAGAGCCGAAGTCAG	chrX:15529600-15529619_BMX_+	exon_04
BMX	ACACATTACATGCTTCCCAG	chrX:15534344-15534363_BMX_-	exon_05
BMX	GCTAGAGTGGTACTTGAAGA	chrX:15540514-15540533_BMX_-	exon_07
BMX	GCTAGACTGGTACTTGAAGA	chrX:15540580-15540599_BMX_-	exon_07
BMX	TGTATCCATACCATTTCCCA	chrX:15554542-15554561_BMX_-	exon_13
BPIFB6	GCACAGGTGGTGCCATGCTG	chr20:31619440-31619459_BPIFB6_+	exon_01
BPIFB6	CCAGGGATGAAACCTATCAA	chr20:31620858-31620877_BPIFB6_+	exon_02
BPIFB6	ACAGCCCTCACTCTTGAACA	chr20:31622658-31622677_BPIFB6_-	exon_04
BPIFB6	TCTGGGTCACTCACCAGCCC	chr20:31622945-31622964_BPIFB6_-	exon_05
BPIFB6	TAGGGCTACACTCACCACTG	chr20:31623474-31623493_BPIFB6_-	exon_06
BRAF	AAGATGGCGGCGCTGAGCGG	chr7:140624487-140624506_BRAF_-	exon_01
BRAF	GAGAGAAGAAACCAATTGGT	chr7:140507844-140507863_BRAF_-	exon_05
BRAF	GAACTTCTGTACTACAACGC	chr7:140501243-140501262_BRAF_+	exon_06
BRAF	ATACCCAATAGAGTCCGAGG	chr7:140500158-140500177_BRAF_+	exon_07
BRAF	GATGACTTCCTTTCTCGCTG	chr7:140482849-140482868_BRAF_+	exon_10
BRD4	TTAGGGTTGGAGGTCTCTGG	chr19:15383750-15383769_BRD4_+	exon_02
BRD4	ACAGGAGGAGGATTCGGCTG	chr19:15376361-15376380_BRD4_+	exon_05
BRD4	GGAGCCCAAGACCACCAAGC	chr19:15375475-15375494_BRD4_-	exon_06
BRD4	TTCGACTGATGACTCTGAGG	chr19:15367813-15367832_BRD4_-	exon_08
BRD4	CACGCGGCCCAGCTTCTCGC	chr19:15366256-15366275_BRD4_+	exon_10
BTK	GATGCTCTCCAGAATCACTG	chrX:100630246-100630265_BTK_+	exon_02
BTK	ACCGGAATCTGTCTTTCTGG	chrX:100629522-100629541_BTK_+	exon_03
BTK	CTGAAGAACTAAGGAAGCGG	chrX:100625008-100625027_BTK_-	exon_05
BTK	ATGCAAATGATCTACAGCTG	chrX:100615627-100615646_BTK_-	exon_08
BTK	GAATAGTAGCACTCACCCTG	chrX:100613589-100613608_BTK_+	exon_11
BTLA	AAAGGGATCTCCTGCTAAGA	chr3:112198543-112198562_BTLA_+	exon_02
BTLA	TGCCTGAGAAGGCACCAAGG	chr3:112190058-112190077_BTLA_-	exon_03

BTLA	AAACAGGTAGTGATGAGTAG	chr3:112190091-112190110_BTLA_+	exon_03
BTLA	AACTCTCTGACACAGCAGGA	chr3:112188620-112188639_BTLA_-	exon_04
BTLA	GCATCCTGAAACAAAGGTCA	chr3:112185116-112185135_BTLA_+	exon_05
BUD31	AATCAACTCCCAGCCATCTG	chr7:99008750-99008769_BUD31_-	exon_03
BUD31	GGAGTTGATTGAGCCAACAC	chr7:99008760-99008779_BUD31_+	exon_03
BUD31	GCTGAAACAGAACCGCATGA	chr7:99013760-99013779_BUD31_+	exon_04
BUD31	AAAGAGGTCGAAGATGTAGC	chr7:99013839-99013858_BUD31_-	exon_04
BUD31	AAGCGGAAAGCCATCAGCAG	chr7:99013862-99013881_BUD31_+	exon_04
C3orf30	TCAGCCAGAGCCTCTTGCGG	chr3:118865049-118865068_C3orf30_	exon_01
C3orf30	GAAGAACCAGGCCGAAAGGA	chr3:118865141-118865160_C3orf30_	exon_01
C3orf30	TCAGGTGTCTGGCCTGACGG	chr3:118865432-118865451_C3orf30_	exon_01
C3orf30	GGACCGCAGAATGTCTGGCG	chr3:118865618-118865637_C3orf30_	exon_01
C3orf30	TATGAGCTTGGTCAATAGCC	chr3:118865999-118866018_C3orf30_	exon_01
C8B	ATGGGCTTGGAGGGCGCCGG	chr1:57431585-57431604_C8B_-	exon_01
C8B	GGTGAAAGGCCACATTCCTT	chr1:57425829-57425848_C8B_-	exon_02
C8B	CATGAAATGGACCAATACTG	chr1:57420383-57420402_C8B_-	exon_04
C8B	ACCTGGCTGTCTCTTACCTG	chr1:57417704-57417723_C8B_+	exon_05
C8B	TGGGACCCACTACATCACAG	chr1:57411560-57411579_C8B_-	exon_07
CA9	GGATCAACAGAGGGAGCCAG	chr9:35673980-35673999_CA9_-	exon_01
CA9	AGGCGCAGTTCTGGGAGCGG	chr9:35675889-35675908_CA9_-	exon_03
CA9	AGAGCCCGGTACTCCCGCCC	chr9:35676105-35676124_CA9_-	exon_04
CA9	TCGCTTGGAAGAAATCGCTG	chr9:35677828-35677847_CA9_+	exon_06
CA9	GACTACACCGCCCTGTGCCC	chr9:35679267-35679286_CA9_+	exon_07
CADM1	GAGCGGATCCCAGTGTGCGG	hr11:115375073-115375092_CADM1_	exon_01
CADM1	AAAGACGTGACAGTGATCGA	hr11:115111098-115111117_CADM1_	exon_02
CADM1	ATGGTGGTGTAACTTTCCTG	hr11:115109231-115109250_CADM1_	exon_03
CADM1	TATCCAGAAAGACACTGCGG	hr11:115102166-115102185_CADM1_	exon_04
CADM1	AAGGTGCACAAGGAGGACGA	hr11:115099913-115099932_CADM1_	exon_05
CASP3	AGTTTCTGAATGTTTCCCTG	chr4:185553488-185553507_CASP3_+	exon_05
CASP3	GTTTGTGTGCTTCTGAGCCA	chr4:185553040-185553059_CASP3_-	exon_06
CASP3	CGTGGTACAGAACTGGACTG	chr4:185552286-185552305_CASP3_-	exon_07
CASP3	TCTAACACCCAGGCCTGCCG	chr4:185552304-185552323_CASP3_-	exon_07
CASP3	TCTTGGCGAAATTCAAAGGA	chr4:185550628-185550647_CASP3_-	exon_08
CCR2	GATAAACCGAGAACGAGATG	chr3:46399029-46399048_CCR2_-	exon_01
CCR2	TGGAAATTATTCCATCCTCG	chr3:46399604-46399623_CCR2_-	exon_01
CCR2	TCAACTGGACCAAGCCACGC	chr3:46399861-46399880_CCR2_+	exon_01
CCR2	ACTCCTGGACCTCCACACAC	chr3:46401217-46401236_CCR2_-	exon_02
CCR2	ACTACACAAGGACTCCTCGA	chr3:46401262-46401281_CCR2_+	exon_02
CCT2	AGACTTGGTAAAGAGCACCT	chr12:69980562-69980581_CCT2_+	exon_03
CCT2	GGGTTGGAGAGAAGCCACGA	chr12:69981980-69981999_CCT2_+	exon_06
CCT2	GAAAGGCTCTGGCAACCTGG	chr12:69983388-69983407_CCT2_+	exon_07
CCT2	GAACGCCTAGCTCTTGTCAC	chr12:69987372-69987391_CCT2_+	exon_10
CCT2	GACTCTAGAACAGTTTATGG	chr12:69991522-69991541_CCT2_+	exon_12
CCT7	GAGGCTGTAAGAACTACCCT	chr2:73466864-73466883_CCT7_+	exon_02
CCT7	GTACTGCCATACCAGCTGGG	chr2:73470250-73470269_CCT7_-	exon_04
CCT7	GCCACAGCAGGCTTACCTCG	chr2:73471840-73471859_CCT7_-	exon_06
CCT7	AAGAAGACTTTCTCTTACGC	chr2:73474920-73474939_CCT7_+	exon_07
CCT7	CCTGTCAGCAAAGTACTGGG	chr2:73476234-73476253_CCT7_-	exon_08
CD274	GTTGAAGGACCAGCTCTCCC	chr9:5457287-5457306_CD274_+	exon_03
CD274	TTGAAGGACCAGCTCTCCCT	chr9:5457288-5457307_CD274_+	exon_03
CD274	TCTGAAGTGCAGCATTTCCC	chr9:5457308-5457327_CD274_-	exon_03

CD274	GAACTGACATGTCAGGCTGA	chr9:5462893-5462912_CD274_+	exon_04
CD274	TGAATGTCAGTGCTACACCA	chr9:5465569-5465588_CD274_-	exon_05
CD276	GCTGACAGATACCAAACAGC	chr15:73994720-73994739_CD276_+	exon_03
CD276	GGTGGCATCGGTGCCCACTA	chr15:73996042-73996061_CD276_-	exon_05
CD276	CACAAGGCAATGCATCCCTG	chr15:73996222-73996241_CD276_+	exon_05
CD276	TGTGCACAGCGTCCTGCGGG	chr15:73996707-73996726_CD276_+	exon_06
CD276	TCGCACCCAGCACCACCCGC	chr15:73996722-73996741_CD276_-	exon_06
CD27	TGGCACGGCCACATCCCTGG	chr12:6554263-6554282_CD27_+	exon_01
CD27	AGAGAGGCACTACTGGGCTC	chr12:6554345-6554364_CD27_+	exon_01
CD27	AGTGCCGACAGCTCTCACAG	chr12:6554693-6554712_CD27_-	exon_02
CD27	GACGACCGAGCGGTCAGCGA	chr12:6559449-6559468_CD27_-	exon_03
CD27	TGGGTAGAGAGAGTCCGGGC	chr12:6559769-6559788_CD27_-	exon_04
CD28	ATTGTCGTACGCTACAAGCA	chr2:204591386-204591405_CD28_-	exon_02
CD28	TGTAGGAATACTTGCAGCTA	chr2:204591417-204591436_CD28_-	exon_02
CD28	TTCCGGGCATCCCTTCACAA	chr2:204591454-204591473_CD28_+	exon_02
CD28	GGGCTGGTAATGCTTGCGGG	chr2:204599577-204599596_CD28_-	exon_04
CD28	TAGGCTGCGAAGTCGCGTGG	chr2:204599606-204599625_CD28_-	exon_04
CD38	CCTCGTCGTGGTGCTCGCGG	chr4:15780139-15780158_CD38_+	exon_01
CD38	CCACCGCGAGCACCACGACG	chr4:15780142-15780161_CD38_-	exon_01
CD38	CCAGCGGGACATGTTCACCC	chr4:15826554-15826573_CD38_+	exon_03
CD38	ACTACTTGGTACTTACCCTG	chr4:15835922-15835941_CD38_-	exon_04
CD38	GATGTGCAAGATGAATCCTC	chr4:15850196-15850215_CD38_-	exon_08
CD40LG	CATGCTGATGGGCAGTCCAG	hrX:135730451-135730470_CD40LG_	exon_01
CD40LG	GATTCTCTCGAATCTACCGG	hrX:135741381-135741400_CD40LG_	exon_05
CD40LG	GATCCAAGCCAAGTGAGCCA	hrX:135741515-135741534_CD40LG_	exon_05
CD40LG	AGCCAAGTGAGCCATGGCAC	hrX:135741521-135741540_CD40LG_	exon_05
CD40LG	AGGACGTGAAGCCAGTGCCA	hrX:135741535-135741554_CD40LG_	exon_05
CD40	TCTGCTGGACCCGAAGCCCT	chr20:44751253-44751272_CD40_-	exon_04
CD40	GAGTGCTTATACCTTGTCCA	chr20:44751851-44751870_CD40_-	exon_05
CD40	TAGCTGTGAGACCAAAGACC	chr20:44755276-44755295_CD40_+	exon_06
CD40	AGGGCTCTCAGCCGATCCTG	chr20:44756782-44756801_CD40_-	exon_07
CD40	AGAGAGTCGCATCTCAGTGC	chr20:44757646-44757665_CD40_+	exon_09
CD52	GAAGAGGTGGATTATGGCAT	chr1:26646762-26646781_CD52_-	exon_02
CD52	GAAGCAGAAGAGGTGGATTA	chr1:26646768-26646787_CD52_-	exon_02
CD52	CTCAACTGAAGCAGAAGAGG	chr1:26646775-26646794_CD52_-	exon_02
CD70	GCTGAGCCTGTGCGAAGCGC	chr19:6590878-6590897_CD70_+	exon_01
CD70	GTGCATCCAGCGCTTCGCAC	chr19:6590887-6590906_CD70_-	exon_01
CD70	ATGGGACCAAAGCAGCCCGC	chr19:6590938-6590957_CD70_+	exon_01
CD70	GGCGGGAGAGCAGATTCCCA	chr19:6586205-6586224_CD70_+	exon_03
CD70	AGAGCAGATTCCCACGGCCA	chr19:6586211-6586230_CD70_+	exon_03
CD80	AAGCCATGGGCCACACACGG	chr3:119276561-119276580_CD80_-	exon_02
CD80	GCGCAGAGCCAGGATCACAA	chr3:119263494-119263513_CD80_+	exon_03
CD80	GAGCCAGGATCACAATGGAG	chr3:119263499-119263518_CD80_+	exon_03
CD80	TGAGATTAAGGTAATGGCCC	chr3:119248726-119248745_CD80_+	exon_05
CD80	ATTAAGGTAATGGCCCAGGA	chr3:119248730-119248749_CD80_+	exon_05
CD86	GTGGATGCGAATCATTCCTG	chr3:121822647-121822666_CD86_-	exon_03
CD86	ACAGTTCAGAATTCATCTGG	chr3:121822666-121822685_CD86_-	exon_03
CD86	GAATGAAACAGACAAGCTGA	chr3:121825246-121825265_CD86_-	exon_04
CD86	GGAAGTACAGCTGTAATCCA	chr3:121828150-121828169_CD86_-	exon_05
CD86	TCTTTCAGGAACCAACACAA	chr3:121836887-121836906_CD86_+	exon_06
CDC16	AAAGTAGCTTCACTCTCTCG	hr13:115002153-115002172_CDC16_	exon_02

CDC16	TTTGTAGCTGTAGGTAGCCA	:hr13:115007657-115007676_CDC16_	exon_06
CDC16	TCAGGGATGACCGTTTCACT	:hr13:115009343-115009362_CDC16_	exon_08
CDC16	GTAGAGCTGAATAAAGCCAA	hr13:115010423-115010442_CDC16_	exon_09
CDC16	AGTGGGATGTTACTATCTCA	hr13:115012420-115012439_CDC16_	exon_11
CDH1	GCTGCGGCTCCAAGGGCCCA	chr16:68771320-68771339_CDH1_-	exon_01
CDH1	AGAGGCCGCGTCCTGGGCAG	chr16:68772293-68772312_CDH1_+	exon_02
CDH1	GCAATGCGTTCTCTATCCAG	chr16:68842722-68842741_CDH1_-	exon_05
CDH1	CAAGCCCGAATTCACCCAGG	chr16:68844186-68844205_CDH1_+	exon_06
CDH1	GCTGAGGATGGTGTAAGCGA	chr16:68845653-68845672_CDH1_-	exon_07
CDK1	ACCCTTATACACAACTCCAT	chr10:62544469-62544488_CDK1_-	exon_03
CDK1	AATCCATGTACTGACCAGGA	chr10:62545512-62545531_CDK1_-	exon_04
CDK1	GAATCCATGTACTGACCAGG	chr10:62545513-62545532_CDK1_-	exon_04
CDK1	GATCTCCAGAAGTATTGCTG	chr10:62551667-62551686_CDK1_+	exon_06
CDK1	GCACTCCCAATAATGAAGTG	chr10:62551914-62551933_CDK1_+	exon_07
CDK2	AAGCAGAGAGATCTCTCGGA	chr12:56361670-56361689_CDK2_-	exon_02
CDK2	AATGGCAGAAAGCTAGGCCC	chr12:56362585-56362604_CDK2_-	exon_04
CDK2	TCTGAGGTTTAAGGTCTCGG	chr12:56362621-56362640_CDK2_-	exon_04
CDK2	TCAGGCATAGAAGTAACTCC	chr12:56364924-56364943_CDK2_-	exon_06
CDK2	TAACAAGCGGATTTCGGCCA	chr12:56365325-56365344_CDK2_+	exon_07
CDK4	TTCGTGAGGTGGCTTTACTG	chr12:58145321-58145340_CDK4_-	exon_02
CDK4	GTTTCGGCTGGCAAGCCTGG	chr12:58144997-58145016_CDK4_+	exon_03
CDK4	CCAGATGGCACTTACACCCG	chr12:58144708-58144727_CDK4_-	exon_04
CDK4	CAGAATCTACAGCTACCAGA	chr12:58144723-58144742_CDK4_-	exon_04
CDK4	GTACCACCGACTGCACTGGG	chr12:58143000-58143019_CDK4_+	exon_07
CDK6	GGCCTTGAACACCTTCCCAT	chr7:92462548-92462567_CDK6_+	exon_02
CDK6	ATGATGTTTCAGCTTCTCCG	chr7:92355085-92355104_CDK6_-	exon_04
CDK6	AACACTCCAGAGATCCACGG	chr7:92300772-92300791_CDK6_+	exon_05
CDK6	TAGGCCAGTCTTCTTCTCCT	chr7:92247487-92247506_CDK6_+	exon_07
CDK6	TCAGGCTGTATTCAGCTCCG	chr7:92244454-92244473_CDK6_+	exon_08
CDK7	ATCTCTGGCCTTGTAAACGG	chr5:68531228-68531247_CDK7_-	exon_02
CDK7	AGCTCCAAATAGTAACTCGG	chr5:68558046-68558065_CDK7_-	exon_08
CDK7	TAGCTCCAAATAGTAACTCG	chr5:68558047-68558066_CDK7_-	exon_08
CDK7	TGTGATGCAAAGGTATTCCA	chr5:68568763-68568782_CDK7_-	exon_10
CDK7	TGACATCCAGGTGTTGGCCC	chr5:68572403-68572422_CDK7_-	exon_11
CDK9	GGCAAAGCAGTACGACTCGG	chr9:130548430-130548449_CDK9_+	exon_01
CDK9	GCACCGCAAGACCGGCCAGA	chr9:130548998-130549017_CDK9_+	exon_02
CDK9	AAGGATCTTGATCTCCCGCA	chr9:130549813-130549832_CDK9_-	exon_03
CDK9	GGTATATACTACCCTTGCAG	chr9:130550242-130550261_CDK9_-	exon_04
CDK9	TCACCGAGCAACAGCTCCGG	chr9:130550649-130550668_CDK9_-	exon_05
CDX4	AAAGAAGCAGGCATGTACCC	chrX:72667114-72667133_CDX4_+	exon_01
CDX4	ACACGCTCCAGTCTTCTCGC	chrX:72667326-72667345_CDX4_-	exon_01
CDX4	GTCGGTCGAGCAGAAAGCGG	chrX:72667406-72667425_CDX4_-	exon_01
CDX4	TGGCGGGTCGCTTGTCCCGA	chrX:72667485-72667504_CDX4_+	exon_01
CDX4	TCAGAGCTGGCAGTTAACCT	chrX:72673460-72673479_CDX4_+	exon_02
CHEK1	TTGGTGCAAACCCTGGGAGA	hr11:125496694-125496713_CHEK1_	exon_02
CHEK1	CTTCCATCAACTCATGGCAG	hr11:125499167-125499186_CHEK1_	exon_04
CHEK1	GGAATGGTAATTCTTGCTGA	:hr11:125507376-125507395_CHEK1_	exon_08
CHEK1	GGTGTGTCAGAGTCTCCCAG	hr11:125513716-125513735_CHEK1_	exon_09
CHEK1	CATAAGGAAAGACCTGTGCG	:hr11:125514026-125514045_CHEK1_	exon_10
CHEK2	GATTGGCAAATCCATCCTGA	chr22:29130394-29130413_CHEK2_+	exon_02
CHEK2	CTTAGACTGCAAACTGGCCG	chr22:29126407-29126426_CHEK2_+	exon_03

CHEK2	GCATACATAGAAGATCACAG	chr22:29121066-29121085_CHEK2_-	exon_05
CHEK2	TGCTGGGTATAACCGTGCTG	chr22:29091735-29091754_CHEK2_-	exon_12
CHEK2	AAGAAGCCTTAAGACACCCG	chr22:29090029-29090048_CHEK2_-	exon_14
CKAP5	TGTCACTGATTCCAATGCAG	chr11:46839925-46839944_CKAP5_-	exon_03
CKAP5	TAAAGCTAAAGCCAAGGAGC	chr11:46837885-46837904_CKAP5_-	exon_04
CKAP5	TGAAGCCAAACTAATTGCTG	chr11:46832622-46832641_CKAP5_-	exon_05
CKAP5	TGGGAACGAAGAAATCGAGT	chr11:46831351-46831370_CKAP5_+	exon_06
CKAP5	AGTGGTACAAGCCCTGCAGG	chr11:46819659-46819678_CKAP5_-	exon_10
CLDN17	GAGGCATTAGACATAACTGG	chr21:31538255-31538274_CLDN17_+	exon_01
CLDN17	AGTGCACAGGCTCTAACGAG	chr21:31538600-31538619_CLDN17_-	exon_01
CLDN17	GAATTGCATCCGACAAGCCA	chr21:31538761-31538780_CLDN17_-	exon_01
CLDN17	AAAGCTGATACTCTCCACTG	chr21:31538832-31538851_CLDN17_+	exon_01
CLDN17	ACCAGCCCAGCAATTTGCAA	chr21:31538901-31538920_CLDN17_+	exon_01
CLRN1	ACTGCCTCACACCCTCTCCG	chr3:150690276-150690295_CLRN1_+	exon_01
CLRN1	ACAGTGGCTTTGATCCACAA	chr3:150690383-150690402_CLRN1_+	exon_01
CLRN1	CCAAGGCTGTCACAACTCCG	chr3:150690411-150690430_CLRN1_+	exon_01
CLRN1	CAAGGCTGTCACAACTCCGA	chr3:150690412-150690431_CLRN1_+	exon_01
CLRN1	GGACCATGCAGAGTTTCAAA	chr3:150659401-150659420_CLRN1_+	exon_02
CPSF3	AATAGGAGATCAATCTCAGC	chr2:9570121-9570140_CPSF3_-	exon_03
CPSF3	GATCTCAATCATGAACATGG	chr2:9572786-9572805_CPSF3_-	exon_05
CPSF3	GTCCACGATATTGTAAACAG	chr2:9576406-9576425_CPSF3_+	exon_07
CPSF3	TGTAGAAGGGACACTTGCCA	chr2:9582084-9582103_CPSF3_+	exon_09
CPSF3	CATAGACTAACCACATGAGG	chr2:9583782-9583801_CPSF3_-	exon_10
CREBBP	GCTCAGAGGGCTCTTGCCCA	chr16:3900724-3900743_CREBBP_+	exon_02
CREBBP	ACGAGAGCAAGCAAACGGAG	chr16:3843477-3843496_CREBBP_-	exon_04
CREBBP	TTTGTCGTGAAGATGCACAA	chr16:3842069-3842088_CREBBP_+	exon_05
CREBBP	GAGTTCCTTCACCTACCCAG	chr16:3832669-3832688_CREBBP_+	exon_06
CREBBP	AATCCAATGAACATTCCAGC	chr16:3831283-3831302_CREBBP_-	exon_07
CST9	GTTCAAGGCAAACTCCACTG	chr20:23586334-23586353_CST9_+	exon_01
CST9	GAGAAGCAGTGACAGTGCCC	chr20:23586448-23586467_CST9_+	exon_01
CST9	AGAGGAGGAAGGCTATGCCC	chr20:23586469-23586488_CST9_-	exon_01
CST9	AGCTGACAAAGCCATTCCGA	chr20:23584164-23584183_CST9_-	exon_02
CST9	CAGCTGACAAAGCCATTCCG	chr20:23584165-23584184_CST9_-	exon_02
CTLA4	TATCCAAGGACTGAGGGCCA	chr2:204735544-204735563_CTLA4_+	exon_02
CTLA4	ATGCCCAGGTAGTATGGCGG	chr2:204735608-204735627_CTLA4_-	exon_02
CTLA4	AGAGGAGGAAGTCAGAATCT	chr2:204736117-204736136_CTLA4_-	exon_03
CTLA4	TAACTGCTGCAAGGATCCAG	chr2:204736135-204736154_CTLA4_-	exon_03
CTLA4	AAGAAGCCCTCTTACAACAG	chr2:204737439-204737458_CTLA4_+	exon_04
CTNNB1	GATGGAGTTGGACATGGCCA	chr3:41266024-41266043_CTNNB1_+	exon_03
CTNNB1	AAGGTTATGCAAGGTCCCAG	chr3:41266973-41266992_CTNNB1_-	exon_05
CTNNB1	ATGGCAGTGCGTTTAGCTGG	chr3:41267227-41267246_CTNNB1_+	exon_06
CTNNB1	GAAGCTGAACAAGAGTCCCA	chr3:41275040-41275059_CTNNB1_-	exon_09
CTNNB1	TTGCTGCTGTGTCCCACCCA	chr3:41275763-41275782_CTNNB1_-	exon_10
CXCL1	AGAGAGCAGCGCGGGCCATG	chr4:74735187-74735206_CXCL1_-	exon_01
CXCL1	GAGAGAGCAGCGCGGGCCAT	chr4:74735188-74735207_CXCL1_-	exon_01
CXCL1	GGAGAGAGCAGCGCGGGCCA	chr4:74735189-74735208_CXCL1_-	exon_01
CXCL3	GGGACCTTACATTCACACTT	chr4:74904038-74904057_CXCL3_+	exon_02
CXorf66	CCTGGAATCTAACTTACGAA	chrX:139038193-139038212_CXorf66_	exon_03
CXorf66	AAGGTCAGTGAGTCTAGGCA	chrX:139038384-139038403_CXorf66_	exon_03
CXorf66	AAAGGTCAGTGAGTCTAGGC	chrX:139038385-139038404_CXorf66_	exon_03
CXorf66	TAAAGTGGCTTGAATAGCTG	chrX:139038491-139038510_CXorf66_	exon_03

CXorf66	AGTGGCTTGAATAGCTGAGG	hrX:139038494-139038513_CXorf66_	exon_03
DDOST	TGTCCAGCAGCACTAAGGTG	chr1:20987738-20987757_DDOST_+	exon_01
DDOST	TGAATGTGAGCTCAAAGCCC	chr1:20987461-20987480_DDOST_+	exon_02
DDOST	TAGAGATGATTTCCCAACGA	chr1:20981959-20981978_DDOST_+	exon_05
DDOST	TTTGGTGCTGGACATCCTGA	chr1:20981157-20981176_DDOST_-	exon_06
DDOST	GGAGCCGCTGAAGATGACGC	chr1:20980781-20980800_DDOST_+	exon_07
DDX20	TGACGGCGCGGCTACCATGG	hr1:112298531-112298550_DDX20_+	exon_01
DDX20	TAAAGAGGCGTATACTGCCT	chr1:112303136-112303155_DDX20_-	exon_04
DDX20	ATTGGGAGACATACATGCAT	hr1:112305360-112305379_DDX20_+	exon_09
DDX20	ACAGTGACCTACTGTTGCCG	hr1:112305542-112305561_DDX20_+	exon_10
DDX20	GAAGACAAGACTTAAAGAGG	hr1:112309203-112309222_DDX20_+	exon_11
DGAT2L6	CAGTCGGGAGAAGAAAGCCA	hrX:69397434-69397453_DGAT2L6_-	exon_01
DGAT2L6	GATTGGAACACCCACAGTCA	hrX:69419210-69419229_DGAT2L6_-	exon_02
DGAT2L6	GGGTACGAAACTGGACCCTA	hrX:69419680-69419699_DGAT2L6_-	exon_03
DGAT2L6	TCCACCCACCACAATAACCA	hrX:69421806-69421825_DGAT2L6_-	exon_05
DGAT2L6	CAATCAGGAGACCTTCCCTG	hrX:69424200-69424219_DGAT2L6_-	exon_06
DHFRL1	TCATTCCTGAGCGGCGGCCT	chr3:93780264-93780283_DHFRL1_+	exon_02
DHFR	CCCGGCAGATACCTGAGCGG	chr5:79950211-79950230_DHFR_+	exon_01
DHFR	AGATACCTGAGCGGTGGCCA	chr5:79950217-79950236_DHFR_+	exon_01
DHFR	AACCTTAGGGAACCTCCACA	chr5:79933817-79933836_DHFR_-	exon_04
DHX15	AGAGTGCGAGGATGTCCAAG	chr4:24586008-24586027_DHX15_-	exon_01
DHX15	GAATGGGAAGCTTTCAAAGG	chr4:24578128-24578147_DHX15_+	exon_02
DHX15	ATGTACTCCACACACCACTG	chr4:24572445-24572464_DHX15_+	exon_03
DHX15	TACACCATAACGCTCCAGGA	chr4:24557967-24557986_DHX15_+	exon_04
DHX15	TCAACAGGATGTGTACGCCC	chr4:24556469-24556488_DHX15_+	exon_05
DHX8	GTTTGGCAAGTTCTTCCGCG	chr17:41561459-41561478_DHX8_-	exon_01
DHX8	ACCGGGACAAATATGGAGAG	chr17:41570255-41570274_DHX8_+	exon_06
DHX8	TGGCCACACGACCCTCCCGC	chr17:41570852-41570871_DHX8_-	exon_07
DHX8	TAGACGGCGAAATCTTGTCG	chr17:41571089-41571108_DHX8_+	exon_08
DHX8	GAAGAGCCTCCATTCCTGAG	chr17:41576259-41576278_DHX8_+	exon_10
DOT1L	GCTGAGACTGAAGTCGCCCG	chr19:2164201-2164220_DOT1L_+	exon_01
DOT1L	CAGGCCGTGCACTCACCGGA	chr19:2180752-2180771_DOT1L_-	exon_02
DOT1L	TCAGCTTCGAGAGCATGCAG	chr19:2189727-2189746_DOT1L_+	exon_04
DOT1L	GATATGGCGCAGGAGTCCAG	chr19:2191057-2191076_DOT1L_-	exon_05
DOT1L	CATCCCGGCCAAGTATGCGG	chr19:2193761-2193780_DOT1L_+	exon_06
DPAGT1	AGAGGCGCGCAGCAATGAAG	r11:118972242-118972261_DPAGT1_	exon_03
DPAGT1	AGCACCGCTGATCACTCCCT	r11:118971818-118971837_DPAGT1_	exon_04
DPAGT1	GATGCAGGCCAAGTATCGGG	r11:118971349-118971368_DPAGT1_	exon_05
DPAGT1	TGCTGGCATGACCTTTGCCG	r11:118968692-118968711_DPAGT1_	exon_08
DPAGT1	GGTGGCAGAGAGCCTCCAGC	r11:118967989-118968008_DPAGT1_	exon_10
DRD2	CCTGATCGTCAGCCTCGCAG	chr11:113295142-113295161_DRD2_-	exon_02
DRD2	TAGGTGAGTGGAAATTCAGC	chr11:113288835-113288854_DRD2_-	exon_03
DRD2	GAAGGACAGGACCCAGACGA	hr11:113287625-113287644_DRD2_+	exon_04
DRD2	GATGGAGGAGTAGACCACGA	hr11:113286281-113286300_DRD2_+	exon_05
DRD2	GAGATCCAGACCATGCCCAA	chr11:113283373-113283392_DRD2_-	exon_07
DSPP	GGTTCCTCAAAGCAAACCAC	chr4:88533256-88533275_DSPP_+	exon_02
DSPP	AATACCCAAGATGGTCACAA	chr4:88533549-88533568_DSPP_+	exon_03
DSPP	CATGATCATCTTCTTTCCCA	chr4:88534193-88534212_DSPP_-	exon_03
DSPP	GACAATAACAGCAGTAGCCG	chr4:88535258-88535277_DSPP_+	exon_04
DSPP	GACAATGACAAATCAGACAG	chr4:88535420-88535439_DSPP_+	exon_04
ECD	GCTGTGAATAGGCGCATCAG	chr10:74916036-74916055_ECD_-	exon_05

ECD	GCAGCCTGTATCCACTCCGC	chr10:74912101-74912120_ECD_+	exon_07
ECD	AAAGGTTTGTGCCAGACCGG	chr10:74912120-74912139_ECD_-	exon_07
ECD	AGAGTGGTGAGGCAGTCACA	chr10:74908078-74908097_ECD_+	exon_08
ECD	CTGGTGAGAGATCTAACCAC	chr10:74899227-74899246_ECD_+	exon_11
EGFR	AGCGATGCGACCCTCCGGGA	chr7:55086967-55086986_EGFR_+	exon_01
EGFR	GATGTTCAATAACTGTGAGG	chr7:55210049-55210068_EGFR_+	exon_02
EGFR	ATAACCAGCCACCTCCTGGA	chr7:55211002-55211021_EGFR_-	exon_03
EGFR	TGTGATCCAAGCTGTCCCAA	chr7:55218995-55219014_EGFR_+	exon_05
EGFR	CCAGGCAGTCGCTCTCCCGG	chr7:55220339-55220358_EGFR_-	exon_06
EHMT2	GCTGCACTCACCTCTCTCGG	chr6:31864689-31864708_EHMT2_+	exon_01
EHMT2	TAGTGAAGAAACCCTGCCCA	chr6:31864554-31864573_EHMT2_-	exon_02
EHMT2	TCCAGCCACCACGAGCCCAG	chr6:31864099-31864118_EHMT2_-	exon_03
EHMT2	TCACTGGGAAAGGTGACCTC	chr6:31860605-31860624_EHMT2_-	exon_04
EHMT2	GGAGAAGTGACCCTGACGAA	chr6:31860290-31860309_EHMT2_-	exon_06
EIF2S1	TGAGGTGGAAGATGTAGTGA	chr14:67831526-67831545_EIF2S1_+	exon_02
EIF2S1	TGAGGTGTTAGAATACACCA	chr14:67843182-67843201_EIF2S1_+	exon_04
EIF2S1	AGCTGGAAAGCCTATTCCAG	chr14:67843211-67843230_EIF2S1_+	exon_04
EIF2S1	GTAGTCATTACATACCGAGG	chr14:67849182-67849201_EIF2S1_-	exon_07
EIF2S1	TAACAGCCATAGCTTGACTG	chr14:67849238-67849257_EIF2S1_-	exon_07
EIF4E	AGAAAGCTTACCTGTTCTGT	chr4:99823016-99823035_EIF4E_+	exon_03
EIF4E	GAAAGCTTACCTGTTCTGTA	chr4:99823017-99823036_EIF4E_+	exon_03
EIF4E	TTCTCCTCTTCTGTAGTCGG	chr4:99823090-99823109_EIF4E_+	exon_03
EIF4E	CTAGGAGGGTATACAAGGAA	chr4:99802278-99802297_EIF4E_-	exon_08
EIF6	GGCGATAGACGCGTGCACCA	chr20:33872010-33872029_EIF6_+	exon_03
EIF6	GGAGGAGCGGCTCTCAGCCT	chr20:33868516-33868535_EIF6_-	exon_04
EIF6	GTGTTGCAGCTCCTGGTCGG	chr20:33868577-33868596_EIF6_+	exon_04
EIF6	TCTGGCAGATGTGCTCAAGG	chr20:33867888-33867907_EIF6_-	exon_05
EIF6	AATGAGGGAATCCCGCATGC	chr20:33867374-33867393_EIF6_+	exon_06
ENTPD1	AAAGGAGAATGACACAGGCG	chr10:97599513-97599532_ENTPD1_-	exon_03
ENTPD1	CAAGAGACACCCGTTTACCT	chr10:97602202-97602221_ENTPD1_-	exon_04
ENTPD1	ACTCTGGCAGAAACTGGCCA	chr10:97605323-97605342_ENTPD1_-	exon_06
ENTPD1	TAGTCACTTACCTCCTCCCA	chr10:97620331-97620350_ENTPD1_-	exon_08
ENTPD1	AATGAAATGGATGTGCTCCC	chr10:97624593-97624612_ENTPD1_-	exon_09
EPHA2	CAGCAGGGCGAAGCAGGCGC	chr1:16482389-16482408_EPHA2_+	exon_01
EPHA2	GAACGTGGAGGAGCGCTCCG	chr1:16475188-16475207_EPHA2_-	exon_03
EPHA2	GCTCAGGGCACTCCAAGCAG	chr1:16464863-16464882_EPHA2_+	exon_04
EPHA2	CATGAACTACACCTTCACCG	chr1:16464426-16464445_EPHA2_-	exon_05
EPHA2	ACTCGGCTCTGCTGCGGCGG	chr1:16462181-16462200_EPHA2_+	exon_06
ERBB2	TTGGGTGCTCGCGGCTCCGG	chr17:37856544-37856563_ERBB2_-	exon_01
ERBB2	GGTGGCGGAGCATGTCCAGG	chr17:37863295-37863314_ERBB2_-	exon_02
ERBB2	ACAATCCGCAGCCTCTGCAG	chr17:37864634-37864653_ERBB2_-	exon_03
ERBB2	ACGCGCACTGTCTGTGCCGG	chr17:37866341-37866360_ERBB2_+	exon_06
ERBB2	TGAGTCCATGCCCAATCCCG	chr17:37866670-37866689_ERBB2_+	exon_07
ERCC2	GCGGGAGCTCAAACGCACGC	chr19:45873402-45873421_ERCC2_-	exon_02
ERCC2	TTATCGGCAGGCATATCCGC	chr19:45872241-45872260_ERCC2_-	exon_04
ERCC2	AAGGAACAGGTGCTCACCTC	chr19:45871871-45871890_ERCC2_+	exon_05
ERCC2	ATCCAGGTTGTAGATGCCAG	chr19:45868156-45868175_ERCC2_+	exon_07
ERCC2	GTAGCTATAAACCACCACAT	chr19:45867773-45867792_ERCC2_+	exon_08
ESR1	TACCTGGAGAACGAGCCCAG	chr6:152129438-152129457_ESR1_+	exon_01
ESR1	AATTCAGATAATCGACGCCA	chr6:152163736-152163755_ESR1_+	exon_02
ESR1	GAAGTGGGAATGATGAAAGG	chr6:152201885-152201904_ESR1_+	exon_03

ESR1	TCATGAGCGGGCTTGGCCAA	chr6:152265420-152265439_ESR1_-	exon_04
ESR1	GAAGGTGGACCTGATCATGG	chr6:152332810-152332829_ESR1_-	exon_05
ESR2	ACAGATGTGATAACTGGCGA	chr14:64749409-64749428_ESR2_+	exon_02
ESR2	TACGCATCGGGATATCACTA	chr14:64746752-64746771_ESR2_-	exon_03
ESR2	AGGCCTGGCAGCTCTTGCGC	chr14:64735555-64735574_ESR2_+	exon_04
ESR2	GGTCAGGGACATCATCATGG	chr14:64727222-64727241_ESR2_+	exon_05
ESR2	GAGCAAAGATGAGCTTGCCG	chr14:64723963-64723982_ESR2_+	exon_06
EZH2	ACTGGGAAGAAATCTGAGAA	chr7:148544362-148544381_EZH2_-	exon_02
EZH2	ACCAAGAATGGAAACAGCGA	chr7:148543619-148543638_EZH2_-	exon_03
EZH2	GATCTGGAGGATCACCGAGA	chr7:148525834-148525853_EZH2_-	exon_06
EZH2	TCAGAAGGAAATTTCCGAGG	chr7:148524322-148524341_EZH2_+	exon_07
EZH2	ATTCAGGAGGAAGTGCGCCT	chr7:148523675-148523694_EZH2_+	exon_08
F13B	TCAGGAGAACTCTATGCAGA	chr1:197036292-197036311_F13B_-	exon_01
F13B	AAGGCTGGTCTCCAGAGCCA	chr1:197031997-197032016_F13B_-	exon_02
F13B	TAGAATGTCTCACATACGGA	chr1:197030054-197030073_F13B_-	exon_04
F13B	CCAGAATCTCCTGTATGCGA	chr1:197029498-197029517_F13B_-	exon_05
F13B	AACGTATTTCTGCTGACCCA	chr1:197026455-197026474_F13B_+	exon_06
FGF4	GCGCCACCAGGCTCTCCCAG	chr11:69589696-69589715_FGF4_+	exon_01
FGF4	GTTGGGTGCAGTGGGTGCGG	chr11:69589745-69589764_FGF4_+	exon_01
FGF4	CGTGGTGAGCATCTTCGGCG	chr11:69588845-69588864_FGF4_-	exon_02
FGF4	CCTGCTGGAGCTCTCGCCCG	chr11:69588875-69588894_FGF4_-	exon_02
FGF4	TCAGGGCGATGAACATGCCG	chr11:69588154-69588173_FGF4_+	exon_03
FGFR1	TAGGTGGTGTCACTGCCCGA	chr8:38287220-38287239_FGFR1_+	exon_03
FGFR1	ATCATCATCATCCTCCGAGG	chr8:38285922-38285941_FGFR1_+	exon_04
FGFR1	AAGTTCAAATGCCCTTCCAG	chr8:38285518-38285537_FGFR1_-	exon_05
FGFR1	GGTGTAGTTGCCCTTGTCAG	chr8:38283698-38283717_FGFR1_+	exon_06
FGFR1	GAACTCCACGTTGCTACCCA	chr8:38282138-38282157_FGFR1_+	exon_07
FGFR2	GATACCACATTAGAGCCAGA	chr10:123353225-123353244_FGFR2_	exon_02
FGFR2	CTTAGTCCAACTGATCACGG	hr10:123325106-123325125_FGFR2_	exon_03
FGFR2	AAACGGGAAGGAGTTTAAGC	chr10:123310830-123310849_FGFR2_	exon_05
FGFR2	ACTCACCCACAACATCCAGG	hr10:123298100-123298119_FGFR2_	exon_06
FGFR2	GCCGGCAAATGCCTCCACAG	chr10:123279627-123279646_FGFR2_	exon_07
FGFR3	CACGGCCACGCAGAGCGCGA	chr4:1795684-1795703_FGFR3_-	exon_01
FGFR3	GAGCAGTTGGTCTTCGGCAG	chr4:1801010-1801029_FGFR3_+	exon_02
FGFR3	CATCCGGCAGACGTACACGC	chr4:1803439-1803458_FGFR3_+	exon_05
FGFR3	GCTGCCGGCCAACCAGACGG	chr4:1803596-1803615_FGFR3_+	exon_06
FGFR3	GGGCGCTAACACCACCGACA	chr4:1805424-1805443_FGFR3_+	exon_07
FGFR4	ACCAAGCTCCACTTCCTCAG	chr5:176516677-176516696_FGFR4_-	exon_02
FGFR4	GATTGCCAGCTTCCTACCTG	chr5:176517560-176517579_FGFR4_+	exon_03
FGFR4	AGACCTACTGACCTTGCTGG	chr5:176517819-176517838_FGFR4_-	exon_04
FGFR4	GGATGGACAGGCCTTTCATG	chr5:176518060-176518079_FGFR4_+	exon_05
FGFR4	CCCGGCCAACACCACAGCCG	chr5:176519359-176519378_FGFR4_+	exon_07
FGR	CTGCAAGAAATTGGAGCCGG	chr1:27950394-27950413_FGR_-	exon_03
FGR	GATGTGGAACTTCTCGCCCT	chr1:27949564-27949583_FGR_+	exon_04
FGR	CGGGACTGGGATCAGACCAG	chr1:27943478-27943497_FGR_-	exon_07
FGR	GAACGGCAGCACTAAGGTGG	chr1:27942098-27942117_FGR_-	exon_09
FGR	ATTGGTGGACATGGCAGCCC	chr1:27941363-27941382_FGR_-	exon_10
FLT1	GGTGTCCCAGTAGCTGACCA	chr13:29068960-29068979_FLT1_+	exon_01
FLT1	AGCATAACTAAATCTGCCTG	chr13:29041186-29041205_FLT1_-	exon_03
FLT1	TCAGGGATCAAAGTGTCAAG	chr13:29008332-29008351_FLT1_+	exon_05
FLT1	TACCATACAACTTCCGGCGA	chr13:29004184-29004203_FLT1_+	exon_08

FLT1	AGACCCGGCTCTCTACCCAC	chr13:29001396-29001415_FLT1_-	exon_10
FLT3	GAAGTCATCATCATATCCCA	chr13:28644630-28644649_FLT3_-	exon_02
FLT3	AGTGTACGAAGCTGCCGCTG	chr13:28636131-28636150_FLT3_-	exon_03
FLT3	AAAGTAGGTATTCTCCAGCT	chr13:28631538-28631557_FLT3_+	exon_04
FLT3	GGTGCTTTGCGATTCACAGG	chr13:28626689-28626708_FLT3_-	exon_05
FLT3	AAAGCTGTTCATGTGAACCA	chr13:28623818-28623837_FLT3_-	exon_07
FLT4	GCAGCGGCCGGAGATGCAGC	chr5:180076539-180076558_FLT4_-	exon_01
FLT4	CCCGACCTTGAACATCACGG	chr5:180058731-180058750_FLT4_-	exon_02
FLT4	CAAGGACAGCGAGGACACGG	chr5:180057720-180057739_FLT4_-	exon_03
FLT4	AGGTGGTGTGGGATGACCGG	chr5:180057055-180057074_FLT4_-	exon_05
FLT4	GCGATTTCGGGAGAGCACCG	chr5:180056274-180056293_FLT4_-	exon_07
FMR1NB	AGTGAGCCACACGCATGGCA	hrX:147062973-147062992_FMR1NB_	exon_01
FMR1NB	TGGCTGAGGCCTGTCAGCCA	hrX:147063068-147063087_FMR1NB_	exon_01
FMR1NB	GAGAGCATGAGCATCCCAAA	hrX:147063127-147063146_FMR1NB_	exon_01
FMR1NB	TCTGGAAGAAGATTCCGCAT	rX:147084788-147084807_FMR1NB_	exon_02
FMR1NB	AACCGGCCGATGATTTACAA	rX:147106387-147106406_FMR1NB_	exon_05
FRK	GAGGACTTGAGCTTCCGAGC	chr6:116381281-116381300_FRK_-	exon_01
FRK	GGATCCAAATGACTTCCTGA	chr6:116277747-116277766_FRK_-	exon_05
FRK	GCTGCCAGAAATGTCCTCGT	chr6:116265462-116265481_FRK_-	exon_06
FRK	CTCCAGATAGGCCATTCCAG	chr6:116265509-116265528_FRK_+	exon_06
FRK	TAGACACGAAATAAAGCTGC	chr6:116264303-116264322_FRK_-	exon_07
FYN	AACAACTTCCACGCAGCCGG	chr6:112041085-112041104_FYN_-	exon_04
FYN	TGGATACTACATTACCACCC	chr6:112024133-112024152_FYN_-	exon_08
FYN	TGTGACAGGGAACTACTAGG	chr6:112021427-112021446_FYN_+	exon_09
FYN	GCTGGTCCAGCTCTATGCAG	chr6:112017528-112017547_FYN_-	exon_10
FYN	GATCAGCAAACATTCTAGTG	chr6:112015657-112015676_FYN_-	exon_12
GABRA1	AAGGACCTATGACAGTCCTC	hr5:161300181-161300200_GABRA1_	exon_06
GABRA1	CCATTAGGTTATTTAACCGG	hr5:161300200-161300219_GABRA1_	exon_06
GABRA1	GCATTTGGAGGACTTCCCTA	hr5:161302593-161302612_GABRA1_	exon_07
GABRA1	TGCATAAGCCACCTTAGGGA	hr5:161322726-161322745_GABRA1_	exon_10
GABRA1	GATTCCAAATAGCAGCGGGA	hr5:161324337-161324356_GABRA1_	exon_11
GFRAL	AGTGGCAGAGGCATGTGTAG	chr6:55216079-55216098_GFRAL_+	exon_05
GFRAL	GCACTTGAAGGACCGTCCCA	chr6:55223833-55223852_GFRAL_-	exon_06
GFRAL	CATGCCTTTGACATTAGACA	chr6:55263990-55264009_GFRAL_-	exon_07
GFRAL	AACCAACAGAAGGATTCCAC	chr6:55264206-55264225_GFRAL_-	exon_08
GFRAL	GCTTGACCATAACCAACAGA	chr6:55264216-55264235_GFRAL_-	exon_08
GLRA2	TCTTGGACAAGTTAATGGGA	chrX:14550432-14550451_GLRA2_+	exon_02
GLRA2	TTGCAAGTAACGTTTACTGG	chrX:14592460-14592479_GLRA2_-	exon_03
GLRA2	ACAGTGGAATGATTCACGGC	chrX:14599334-14599353_GLRA2_+	exon_04
GLRA2	GGACTTGAAGAACTTTCCGA	chrX:14622464-14622483_GLRA2_+	exon_05
GLRA2	GCCCAGTGCGACCCTGGCAG	chrX:14627248-14627267_GLRA2_-	exon_07
GLS	GCGTGAGCGCGACTCTGCGG	chr2:191745869-191745888_GLS_+	exon_01
GLS	GATTGCGAACGTCTGATCCC	chr2:191760346-191760365_GLS_+	exon_03
GLS	ATTGCTCCAGCATTTACCAT	chr2:191775006-191775025_GLS_-	exon_07
GLS	GCTGCGACACTGGCTAATGG	chr2:191792074-191792093_GLS_+	exon_12
GLS	TAGGTGGAATAACACTCCCA	chr2:191827553-191827572_GLS_+	exon_18
GPR50	TTACAGCCAATACAGCCATA	chrX:150345224-150345243_GPR50_	exon_01
GPR50	ATAAAGATGATTAGAGCCGG	chrX:150345266-150345285_GPR50_	exon_01
GPR50	AGACCTAATCGGCAACTCCA	chrX:150345316-150345335_GPR50_-	exon_01
GPR50	GGTGCGAGGATCGTACTCGA	chrX:150348561-150348580_GPR50_	exon_02
GPR50	GTCAGCCTTGAAATGGACAG	chrX:150349383-150349402_GPR50_	exon_02

GPX6	GAAGGTCTTTACTTTCCTGA	chr6:28473482-28473501_GPX6_-	exon_04
GPX6	CAGTCGCTAGCCCTTCCTAG	chr6:28472053-28472072_GPX6_+	exon_05
GPX6	AAAGCAGTTCAATACCCACT	chr6:28472071-28472090_GPX6_-	exon_05
GPX6	GGTGGAACCAATGCATGACA	chr6:28472134-28472153_GPX6_+	exon_05
GPX6	TGAAGGTCCATGATATCCGC	chr6:28472195-28472214_GPX6_-	exon_05
GSK3A	GGAAGCTAGTGCCTGCGCCG	chr19:42746359-42746378_GSK3A_+	exon_01
GSK3A	GTCGTAGCCACTCTAGGCCA	chr19:42744252-42744271_GSK3A_-	exon_02
GSK3A	TCAGCCTCACAATATTGCAG	chr19:42741039-42741058_GSK3A_+	exon_03
GSK3A	GGTGGCCCGCCACTTCACCA	chr19:42740793-42740812_GSK3A_-	exon_04
GSK3A	TTGGCCTACATCCACTCCCA	chr19:42738784-42738803_GSK3A_-	exon_05
GSK3B	AGAGAGTGATCATGTCAGGG	chr3:119812273-119812292_GSK3B_-	exon_01
GSK3B	CGGCAGCAAGGTGACAACAG	chr3:119721057-119721076_GSK3B_-	exon_02
GSK3B	TCTGCGGTTTAATATCCCGA	chr3:119634943-119634962_GSK3B_+	exon_05
GSK3B	AGGACAACCAATATTTCCAG	chr3:119624640-119624659_GSK3B_-	exon_07
GSK3B	TAAGGCACATCCTTGGACTA	chr3:119595262-119595281_GSK3B_-	exon_08
GSX2	GGATGAAGAAATCCGGCCCG	chr4:54966592-54966611_GSX2_-	exon_01
GSX2	GATGCGCATGGTTCACCCGC	chr4:54966871-54966890_GSX2_-	exon_01
GSX2	TGAGGCAGTGGAATCTCCGC	chr4:54967060-54967079_GSX2_-	exon_01
GSX2	ACGCAGAGGAACAGTCACGC	chr4:54967964-54967983_GSX2_+	exon_02
GSX2	GGTGCACTACGCGCGCTCCG	chr4:54968008-54968027_GSX2_+	exon_02
GTF2B	GGCGGGACATACTAACCGGC	chr1:89357150-89357169_GTF2B_+	exon_01
GTF2B	TTAGTGGAGGACTACAGAGC	chr1:89352982-89353001_GTF2B_-	exon_02
GTF2B	AAATCTCCATCACTCAGAAG	chr1:89329683-89329702_GTF2B_+	exon_03
GTF2B	ATATGAACAGAAGAGCCTGA	chr1:89325646-89325665_GTF2B_-	exon_05
GTF2B	GAAAGCGCTAGAAACCAGTG	chr1:89323090-89323109_GTF2B_-	exon_06
HAVCR2	AGAAGTGGAATACAGAGCGG	hr5:156533947-156533966_HAVCR2_	exon_02
HAVCR2	TCCAAGGATGCTTACCACCA	hr5:156531692-156531711_HAVCR2_	exon_03
HAVCR2	ATAGCAGAGACACAGACACT	hr5:156525897-156525916_HAVCR2_	exon_04
HAVCR2	GATGTAGATGCCTATTCTGA	hr5:156522378-156522397_HAVCR2_	exon_05
HAVCR2	ATTGCAAAGCGACAACCCAA	hr5:156514120-156514139_HAVCR2_	exon_07
HDAC1	GAGCAAGATGGCGCAGACGC	chr1:32757764-32757783_HDAC1_+	exon_01
HDAC1	CCCTCACAAAGCCAATGCTG	chr1:32782268-32782287_HDAC1_+	exon_03
HDAC1	GCACCATGCAAAGAAGTCCG	chr1:32792601-32792620_HDAC1_+	exon_05
HDAC1	TAAAGGGCGTTCTCACCCGT	chr1:32793275-32793294_HDAC1_-	exon_06
HDAC1	GATGTTCCAGCCTAGTGCGG	chr1:32796199-32796218_HDAC1_+	exon_08
HDAC2	ATGGCGTACAGTCAAGGAGG	chr6:114292053-114292072_HDAC2_-	exon_01
HDAC2	AAACCGACAACAGACTGATA	chr6:114277280-114277299_HDAC2_-	exon_05
HDAC2	GATGTATCAACCTAGTGCTG	chr6:114270212-114270231_HDAC2_-	exon_08
HDAC2	GATAAATCCAAGGACAACAG	chr6:114262896-114262915_HDAC2_-	exon_13
HDAC2	GGTGAGACTGTCAAATTCAG	chr6:114262206-114262225_HDAC2_-	exon_14
HDAC3	GACGTGGGCAACTTCCACTA	chr5:141016305-141016324_HDAC3_-	exon_01
HDAC3	TATGGGTCAATGCCAGGCGA	chr5:141016153-141016172_HDAC3_-	exon_02
HDAC3	CATGTGCCGCTTCCACTCCG	chr5:141014469-141014488_HDAC3_-	exon_03
HDAC3	GAGCAGAACTCAAAGAGCCC	chr5:141009660-141009679_HDAC3_-	exon_04
HDAC3	GTTGACATAGCAGAAGCCAG	chr5:141009283-141009302_HDAC3_-	exon_06
HDAC6	GAAGTGACACTGGAGTCCTG	chrX:48661172-48661191_HDAC6_-	exon_02
HDAC6	AATGGAAGAAGACCTAATCG	chrX:48661370-48661389_HDAC6_+	exon_03
HDAC6	AGCCTCACCTGTCATCCCAG	chrX:48661612-48661631_HDAC6_-	exon_04
HDAC6	TGAGGTGGTCTGGTCCGGAG	chrX:48681649-48681668_HDAC6_-	exon_25
HDAC6	TCTGGACCAGACCACCTCAG	chrX:48681692-48681711_HDAC6_+	exon_25
HDAC8	GCGGAAGATGGAGGAGCCGG	chrX:71792599-71792618_HDAC8_-	exon_01

HDAC8	GAAGGTGGCCATCTCCTCCA	chrX:71788689-71788708_HDAC8_+	exon_03
HDAC8	ACTATGCAGCAGCTATAGGA	chrX:71787828-71787847_HDAC8_-	exon_04
HDAC8	GTGGATTTGGATCTGCACCA	chrX:71715014-71715033_HDAC8_-	exon_05
HDAC8	GACGTGTCTGATGTTGGCCT	chrX:71708864-71708883_HDAC8_-	exon_07
HLA-G	ACAGCGACTCGGCGTGTCCG	chr6:29795932-29795951_HLA-G_+	exon_02
HLA-G	AGAGGAGACACGGAACACCA	chr6:29796005-29796024_HLA-G_+	exon_02
HLA-G	GCAGCCAATCATCCACTGGA	chr6:29796332-29796351_HLA-G_-	exon_03
HLA-G	ACAAAGGAGAGCCTACCTGG	chr6:29796510-29796529_HLA-G_+	exon_03
HLA-G	CATAGTCAAAGACAGGGTGG	chr6:29797220-29797239_HLA-G_-	exon_04
HSP90AA1	GGTGTCGTTCCAGATGCCTG	14:102552709-102552728_HSP90AA	exon_02
HSP90AA1	GATCTGTCAAGCTTTCATAC	14:102552425-102552444_HSP90AA	exon_03
HSP90AA1	TTTCCGAAGACGTTCCACAA	14:102549587-102549606_HSP90AA	exon_09
HSP90AA1	TAAGAACGACAAGTCTGTGA	14:102548559-102548578_HSP90AA	exon_10
HSP90AA1	AGATGACGACACATCACGCA	14:102548066-102548085_HSP90AA	exon_11
HTR5A	TTGGAGACCAACCACAGCCT	hr7:154862661-154862680_HTR5A_	exon_01
HTR5A	ACTCCGAAGACCGAGAGCAG	chr7:154862712-154862731_HTR5A_-	exon_01
HTR5A	GGTGGAAGGTGCGTACACGG	chr7:154862807-154862826_HTR5A_-	exon_01
HTR5A	AATGAGGATGCCCACCATGA	chr7:154875977-154875996_HTR5A_-	exon_02
HTR5A	AGTTGGAGTAGCCAAGCCAC	chr7:154876095-154876114_HTR5A_-	exon_02
ICOSLG	GGTGACGTTGAACAAGCGCA	hr21:45656844-45656863_ICOSLG_	exon_03
ICOSLG	TGGGATGTGGTAGGTCACCA	hr21:45656952-45656971_ICOSLG_	exon_03
ICOSLG	GCAGCAGCCAATGTTCACGC	hr21:45655204-45655223_ICOSLG_	exon_04
ICOSLG	GACAGCCAGGATGCTCCACG	hr21:45651239-45651258_ICOSLG_	exon_05
ICOSLG	TGTCTCCGGACTCACAGCCC	hr21:45649947-45649966_ICOSLG_	exon_06
ICOS	CAAACATGAAGTCAGGCCTC	chr2:204801533-204801552_ICOS_+	exon_01
ICOS	AAATGCAGTTGCTGAAAGGG	chr2:204820455-204820474_ICOS_+	exon_02
ICOS	TGGGTAACCAGAACTTCAGC	chr2:204821401-204821420_ICOS_-	exon_03
ICOS	ACAAAGGCTGCACATCCTAT	chr2:204821420-204821439_ICOS_-	exon_03
ICOS	ACCGTTAGGGTCGTGCACAC	chr2:204822535-204822554_ICOS_-	exon_04
IDH1	ATGTAGATCCAATTCCACGT	chr2:209116156-209116175_IDH1_+	exon_03
IDH1	ACCCATCCACTCACAAGCCG	chr2:209113133-209113152_IDH1_+	exon_04
IDH1	TAGGCTCATCGACGACATGG	chr2:209106805-209106824_IDH1_-	exon_07
IDH1	ATCTGGACAAACCAGCACGC	chr2:209104681-209104700_IDH1_+	exon_08
IDH1	TGAGGCTGGCTTCATGACCA	chr2:209103829-209103848_IDH1_-	exon_09
IDH2	AGAGCGAGCGCACGACCCGC	chr15:90645589-90645608_IDH2_+	exon_01
IDH2	GATCAAGGTGGCGAAGCCCG	chr15:90634844-90634863_IDH2_-	exon_02
IDH2	ACACCCTCGCACCTTCCACA	chr15:90633699-90633718_IDH2_+	exon_03
IDH2	AACTATCCGGAACATCCTGG	chr15:90631923-90631942_IDH2_-	exon_04
IDH2	GGGCATGTACAACACCGACG	chr15:90631593-90631612_IDH2_-	exon_05
IGF1R	GATCAGGGACCAGTCCACAG	chr15:99251157-99251176_IGF1R_-	exon_02
IGF1R	GCCGTTGCGGATGAAGCCCG	chr15:99434839-99434858_IGF1R_-	exon_03
IGF1R	TCGGGCAAGGACCTTCACAA	chr15:99440001-99440020_IGF1R_-	exon_04
IGF1R	ACTAAAGGGCGCCAAAGCAA	chr15:99452068-99452087_IGF1R_+	exon_06
IGF1R	GATGATGCGATTCTTCGACG	chr15:99454580-99454599_IGF1R_-	exon_07
IGSF11	GGAGCAGCAAAGGCGCCAGA	hr3:118753390-118753409_IGSF11_-	exon_01
IGSF11	GGGAGTATCCAGGTGGCCCG	hr3:118649068-118649087_IGSF11_	exon_02
IGSF11	GGGCCACCTGGATACTCCCA	hr3:118649069-118649088_IGSF11_-	exon_02
IGSF11	TTTGTGACAGACCAGGTCCA	hr3:118624556-118624575_IGSF11_	exon_05
IGSF11	TTGGCAGAAGAACACTTGGG	hr3:118621773-118621792_IGSF11_-	exon_07
IKBKE	GTTGCGGGCCTTGTACACAC	chr1:206646635-206646654_IKBKE_-	exon_03
IKBKE	TCAACACTACCAGCTACCTG	chr1:206647705-206647724_IKBKE_+	exon_04

IKBKE	ATAGGGCGGAAGCCGGCAGA	chr1:206648204-206648223_IKBKE_+	exon_05
IKBKE	TGCATCGCGACATCAAGCCG	chr1:206649560-206649579_IKBKE_+	exon_06
IKBKE	GATGAAGGGCAGGCTGCCAG	chr1:206650127-206650146_IKBKE_-	exon_07
IL1B	GAGCTCAGGTACTTCTGCCA	chr2:113593786-113593805_IL1B_+	exon_02
IL1B	GCTCAGGTCATTCTCCTGGA	chr2:113590979-113590998_IL1B_+	exon_04
IL1B	AGCTGGATGCCGCCATCCAG	chr2:113591103-113591122_IL1B_+	exon_04
IL1B	GCTGGATGCCGCCATCCAGA	chr2:113591104-113591123_IL1B_+	exon_04
IL1B	AGAGGTGCTGATGTACCAGT	chr2:113588025-113588044_IL1B_+	exon_07
IL6R	GGAGGAAGCATGCTGGCCGT	chr1:154378097-154378116_IL6R_+	exon_01
IL6R	TGGGACTGTGCACTTGCTGG	chr1:154401895-154401914_IL6R_+	exon_02
IL6R	ACTCACAAACAACATTGCTG	chr1:154403005-154403024_IL6R_-	exon_03
IL6R	CTGCCAGTTAGCAGTCCCGG	chr1:154407061-154407080_IL6R_+	exon_04
IL6R	CAACATCACAGTCACTGCCG	chr1:154407485-154407504_IL6R_+	exon_05
IL6	GGAGAAGGCAACTGGACCGA	chr7:22767069-22767088_IL6_-	exon_03
IL6	TCAGGGCTGAGATGCCGTCG	chr7:22767226-22767245_IL6_-	exon_03
IL6	CATGTGTGAAAGCAGCAAAG	chr7:22768329-22768348_IL6_+	exon_04
IL6	AGCTGCAGGCACAGAACCAG	chr7:22771086-22771105_IL6_+	exon_06
IL6	TTGCCGAAGAGCCCTCAGGC	chr7:22771167-22771186_IL6_-	exon_06
IMP3	GAGCAAGGGCACGTACGCGT	chr15:75932097-75932116_IMP3_-	exon_02
IMP3	GGAGCAAGGGCACGTACGCG	chr15:75932098-75932117_IMP3_-	exon_02
IMP3	GCGCAGCTTGAGGAGCACGG	chr15:75932153-75932172_IMP3_+	exon_02
IMP3	GGAGCTCTGCGACTTCGTCA	chr15:75932206-75932225_IMP3_-	exon_02
IMP3	ATACAGCTTGTCCAGCAGCG	chr15:75932258-75932277_IMP3_+	exon_02
INSR	AACCTGATGAACATCACCCG	chr19:7267586-7267605_INSR_-	exon_02
INSR	ACAAATGCAAGAACTCGCGG	chr19:7184410-7184429_INSR_-	exon_03
INSR	GAGGTGGCACACCTTGGGAC	chr19:7174694-7174713_INSR_+	exon_04
INSR	GGAAAGTGACACCAGAGCGT	chr19:7172348-7172367_INSR_+	exon_05
INSR	GAAGAAGTTTCAGGAACCAA	chr19:7170604-7170623_INSR_-	exon_06
IRAK4	TATGTGCGCTGCCTCAATGT	chr12:44161942-44161961_IRAK4_+	exon_02
IRAK4	TCTGGACTTGAGGAGTCAGG	chr12:44166117-44166136_IRAK4_-	exon_04
IRAK4	GATGAACGACCCATTTCTGT	chr12:44166765-44166784_IRAK4_+	exon_05
IRAK4	CATGAGATGCAAGATTGCTC	chr12:44172022-44172041_IRAK4_+	exon_08
IRAK4	ATGGCACCAGAAGCTTTGCG	chr12:44176231-44176250_IRAK4_+	exon_09
ITK	TGATGAGCTGTTCTTCCAGG	chr5:156608006-156608025_ITK_-	exon_01
ITK	ACAGAAGAAGCGCACGCTGA	chr5:156635896-156635915_ITK_+	exon_02
ITK	TGGGTGCTGGCCCTTAAAGA	chr5:156638358-156638377_ITK_+	exon_03
ITK	CAACGAAGAGTACTGCCTGC	chr5:156649959-156649978_ITK_+	exon_06
ITK	TGAAGGCTCCTTCTTTGCCC	chr5:156665118-156665137_ITK_-	exon_09
JAK1	CAGTGGAGAGTACACAGCAG	chr1:65348993-65349012_JAK1_-	exon_03
JAK1	ATGACGAGAACACCAAGCTC	chr1:65344776-65344795_JAK1_-	exon_04
JAK1	AAACAGATACTCCAGTGAGC	chr1:65339059-65339078_JAK1_+	exon_05
JAK1	GCAGTTGCCAGAACTGCCCA	chr1:65335004-65335023_JAK1_-	exon_06
JAK1	GAGAAGGTTCCTCTGTCTGA	chr1:65332834-65332853_JAK1_+	exon_07
JAK2	ATCTGCCTCAGATTTCCCAA	chr9:5022127-5022146_JAK2_-	exon_03
JAK2	TATCGGCATGGAATATCTCG	chr9:5044446-5044465_JAK2_+	exon_05
JAK2	ATAGAGTTATAGATGGCCAG	chr9:5050809-5050828_JAK2_-	exon_06
JAK2	GAGGTGCTACTTCTTTACAG	chr9:5064975-5064994_JAK2_-	exon_09
JAK2	AATGAAGAGTACAACCTCAG	chr9:5069070-5069089_JAK2_+	exon_11
JAK3	ACTAGGGATGCACTCACCGC	chr19:17955026-17955045_JAK3_+	exon_01
JAK3	CCCGAGCCACATCTTCTCCG	chr19:17954626-17954645_JAK3_-	exon_02
JAK3	CCTTGCGTAGCCCGAAGCGG	chr19:17954242-17954261_JAK3_+	exon_03

JAK3	TCAGCAGCTCTCCCGGCCGC	chr19:17953846-17953865_JAK3_+	exon_04
JAK3	TGACGCGGAGGCGTATTCGG	chr19:17953344-17953363_JAK3_-	exon_05
KCNK10	AGCTGGACTTCACTTACCCA	chr14:88792746-88792765_KCNK10_+	exon_01
KCNK10	GATGGAAGGCACCTCCCAAG	chr14:88729752-88729771_KCNK10_-	exon_02
KCNK10	AGGCACTGCCGAGGTCCCAG	chr14:88707080-88707099_KCNK10_+	exon_03
KCNK10	GCCGGCCAAGATGAACAGGA	chr14:88658692-88658711_KCNK10_+	exon_05
KCNK10	GGAGAACGGAATGATACCCA	chr14:88651935-88651954_KCNK10_-	exon_07
KDM1A	GAATAGCAGAGACTCCGGAG	chr1:23346388-23346407_KDM1A_+	exon_01
KDM1A	GGATACTACAACTCACCCGA	chr1:23357124-23357143_KDM1A_-	exon_02
KDM1A	TTCAAGACGACAGTTCTGGA	chr1:23370935-23370954_KDM1A_+	exon_03
KDM1A	TGGACCACAACAGACCCAGA	chr1:23376965-23376984_KDM1A_+	exon_04
KDM1A	CGAGTTGCCACATTTCGCAA	chr1:23383992-23384011_KDM1A_+	exon_08
KDR	GGGCTCCTTACCCACAGAGG	chr4:55991383-55991402_KDR_+	exon_01
KDR	GAGGTGACTGAGTGCAGCGA	chr4:55984887-55984906_KDR_-	exon_03
KDR	AATTTCCTGGGACAGCAAGA	chr4:55981152-55981171_KDR_-	exon_05
KDR	CTGGGAATACCCTTCTTCGA	chr4:55980295-55980314_KDR_-	exon_06
KDR	GAAGAACAGCACATTTGTCA	chr4:55979480-55979499_KDR_-	exon_07
KIF11	GAAGTTAGTGTACGAACTGG	chr10:94366089-94366108_KIF11_+	exon_02
KIF11	TGAAGAGTATACCTGGGAAG	chr10:94366973-94366992_KIF11_+	exon_04
KIF11	CGTGGAATTATACCAGCCAA	chr10:94368783-94368802_KIF11_-	exon_05
KIF11	AATCCCTGTTGACTTTGGGA	chr10:94373213-94373232_KIF11_+	exon_08
KIF11	TCTTGTGTAGGAGTATACGG	chr10:94381132-94381151_KIF11_+	exon_10
KIT	TCAGACTTAATAGTCCGCGT	chr4:55561740-55561759_KIT_+	exon_02
KIT	GAAAGAAGACAACGACACGC	chr4:55564490-55564509_KIT_+	exon_03
KIT	GCAAGCTATCTTCTTAGGGA	chr4:55565831-55565850_KIT_+	exon_04
KIT	CCAAACCTGAACACCAGCAG	chr4:55573360-55573379_KIT_+	exon_06
KIT	ACCACTAGCTTTCCAAACGG	chr4:55592078-55592097_KIT_-	exon_09
KLK12	AGAACTCACCAAGAACACAC	chr19:51537832-51537851_KLK12_+	exon_02
KLK12	GAGGGCACCAGCCTGCGCTG	chr19:51537293-51537312_KLK12_-	exon_03
KLK12	GACGTGGCACTCGGTGCCAG	chr19:51535163-51535182_KLK12_+	exon_04
KLK12	ACCGAGCAGATCCGGCACAG	chr19:51535323-51535342_KLK12_-	exon_04
KLK12	TGTGTGCAGGCGGCGTCCCG	chr19:51534062-51534081_KLK12_-	exon_05
LAG3	GCAGAAGGCTGAGATCCTGG	chr12:6882449-6882468_LAG3_-	exon_02
LAG3	GCGGTCCCTGAGGTGCACCG	chr12:6883105-6883124_LAG3_-	exon_03
LAG3	GTTCCGGAACCAATGCACAG	chr12:6883845-6883864_LAG3_-	exon_04
LAG3	GCCAAGTGGACTCCTCCTGG	chr12:6884543-6884562_LAG3_+	exon_05
LAG3	GCAATGCCAGCTGTACCAGG	chr12:6886602-6886621_LAG3_+	exon_06
LAP3	CTCCACCGCAGACATGACGA	chr4:17579169-17579188_LAP3_+	exon_01
LAP3	TCTGGACCACCTCTGAAGGC	chr4:17583384-17583403_LAP3_+	exon_03
LAP3	GAGGCCAACTAGCACCACGC	chr4:17583921-17583940_LAP3_-	exon_04
LAP3	TCTGTGGAGGTGGATCCCTG	chr4:17585141-17585160_LAP3_+	exon_05
LAP3	AATGGGATCATTCCTCAGTG	chr4:17590469-17590488_LAP3_+	exon_07
LAS1L	GCCAGGCGACCACGATGCCG	chrX:64754460-64754479_LAS1L_+	exon_01
LAS1L	GCAGTAGAAGCCACTGCCAG	chrX:64753577-64753596_LAS1L_+	exon_02
LAS1L	GAGGAAGACAAAGTTTGCCA	chrX:64752470-64752489_LAS1L_-	exon_03
LAS1L	TGTGGGTCAACTCATGGCGA	chrX:64751270-64751289_LAS1L_+	exon_04
LAS1L	CCAGGACACATTCTACACGT	chrX:64748171-64748190_LAS1L_+	exon_07
LCK	TATCCCATAGTCCCACTGGA	chr1:32740003-32740022_LCK_+	exon_02
LCK	GAGCCTTCGTAGGTAACCAG	chr1:32740374-32740393_LCK_-	exon_03
LCK	GAAGGCGCAGTCCCTGACCA	chr1:32740937-32740956_LCK_+	exon_05
LCK	CCGGGAGAGCGAGAGCACCG	chr1:32741251-32741270_LCK_+	exon_06

LCK	AAGATCCGTAATCTGGACAA	chr1:32741577-32741596_LCK_+	exon_07
LDHA	TACGCTGGACCAAATTAAGA	chr11:18422457-18422476_LDHA_-	exon_04
LDHA	TACAGAGAGTCCAATAGCCC	chr11:18427034-18427053_LDHA_-	exon_07
LDHA	TAGACCTACCTTAATCATGG	chr11:18427109-18427128_LDHA_-	exon_07
LGALS3	GAAACCCAAACCCTCAAGGA	chr14:55604788-55604807_LGALS3_+	exon_03
LGALS3	CATGCGCCAGGCCATCCTTG	chr14:55604802-55604821_LGALS3_-	exon_03
LGALS3	GTCTACCCAGGGCCACCCAG	chr14:55604976-55604995_LGALS3_+	exon_03
LGALS3	TCTCACCAGTGGCCCAGCAG	chr14:55605073-55605092_LGALS3_-	exon_03
LGALS3	GCTGATAACAATTCTGGGCA	chr14:55607040-55607059_LGALS3_+	exon_04
LGALS9B	GCGGTGGAGATGGCCTTCAG	chr17:20370773-20370792_LGALS9B_	exon_01
LGALS9	GACACACATGCCTTTCCAGA	chr17:25967730-25967749_LGALS9_+	exon_03
LGALS9	ACACACATGCCTTTCCAGAA	chr17:25967731-25967750_LGALS9_+	exon_03
LUC7L3	AGAAGCGCAGCAACGTGCGG	chr17:48797158-48797177_LUC7L3_+	exon_01
LUC7L3	GTATTTGTGAACAATTCCGC	chr17:48814354-48814373_LUC7L3_-	exon_02
LUC7L3	GTAGAACGTAGGATCAGACG	chr17:48818542-48818561_LUC7L3_+	exon_04
LUC7L3	ACTGCTAAGGTCCACAACGT	chr17:48821150-48821169_LUC7L3_+	exon_06
LUC7L3	AGACACAAACACTGAATCGA	chr17:48827926-48827945_LUC7L3_+	exon_10
LYN	CAACGTCCAATAAACAGCAA	chr8:56854525-56854544_LYN_+	exon_02
LYN	TGAAAGACAAGTCGTCCGGG	chr8:56860232-56860251_LYN_-	exon_04
LYN	GTTTGGCCACATAGTTGCTG	chr8:56863075-56863094_LYN_-	exon_05
LYN	TTGGCACCAGGAAATAGCGC	chr8:56863286-56863305_LYN_+	exon_06
LYN	ACCAACTTGATGGACTCCCG	chr8:56866483-56866502_LYN_-	exon_08
MAP2K1	AGTTAACGGGACCAGCTCTG	chr15:66679742-66679761_MAP2K1_-	exon_01
MAP2K1	TTGAGAAGATCAGTGAGCTG	chr15:66727487-66727506_MAP2K1_-	exon_02
MAP2K1	TATGGTGCGTTCTACAGCGA	chr15:66729180-66729199_MAP2K1_-	exon_03
MAP2K1	AACATCCTAGTCAACTCCCG	chr15:66774107-66774126_MAP2K1_-	exon_06
MAP2K1	AATGGACAGCCGACCTCCCA	chr15:66779573-66779592_MAP2K1_-	exon_08
MAP2K2	CATCAACCCTACCATCGCCG	chr19:4123815-4123834_MAP2K2_-	exon_01
MAP2K2	CAAAGTCCAGCACAGACCCT	chr19:4117440-4117459_MAP2K2_-	exon_02
MAP2K2	TGATCTGGTGCTTCTCTCGG	chr19:4101236-4101255_MAP2K2_+	exon_05
MAP2K2	GTAGGAGCGCGTGCCCACGA	chr19:4101023-4101042_MAP2K2_+	exon_06
MAP2K2	ACAGCATCTCGCCTCGGCCG	chr19:4099224-4099243_MAP2K2_-	exon_07
MAP2	TAAGGATCAAGGCGGAGCAG	chr2:210517987-210518006_MAP2_+	exon_04
MAP2	AGTTCAAGTAGTCACTGCTG	chr2:210543315-210543334_MAP2_+	exon_05
MAP2	AGAACAGACTGTCACAGTGG	chr2:210545526-210545545_MAP2_+	exon_06
MAP2	AGTGTGCCCAAACAAAGCTA	chr2:210557562-210557581_MAP2_-	exon_07
MAP2	TCTCCTGACACACCTCGCCG	chr2:210570370-210570389_MAP2_-	exon_11
MAP3K8	TGCCAAGTATACCTTTCCAA	chr10:30736820-30736839_MAP3K8_-	exon_04
MAP3K8	GTGACAAAGCATGTTCTCAA	chr10:30739379-30739398_MAP3K8_-	exon_05
MAP3K8	TAATTACCTCTGTTCCTCGG	chr10:30740661-30740680_MAP3K8_-	exon_06
MAP3K8	AGGAGGGATAGGCTGAGCGA	chr10:30747135-30747154_MAP3K8_-	exon_07
MAP3K8	TTCGTGCACAGGAAGCACCG	chr10:30749639-30749658_MAP3K8_-	exon_09
MAP4	TCAGAAACTAGCCAGATTGA	chr3:48040130-48040149_MAP4_-	exon_02
MAP4	AAACCAACACTCCTAGCCAA	chr3:48019390-48019409_MAP4_-	exon_04
MAP4	AATCTGCCAGGTCATCATCA	chr3:47963330-47963349_MAP4_+	exon_06
MAP4	AAACAAGATAGTCCCAGCCA	chr3:47958095-47958114_MAP4_-	exon_08
MAP4	AAAGTGCAGCTTGCCGGCCG	chr3:47956388-47956407_MAP4_-	exon_09
MAPK11	GCCGGTAGAAGCCGGCGCGA	chr22:50708691-50708710_MAPK11_-	exon_01
MAPK11	GCAGAACGTACCGGGAGCTG	chr22:50706279-50706298_MAPK11_-	exon_02
MAPK11	GCTTCTGGACGTCTTCACGC	chr22:50706029-50706048_MAPK11_-	exon_03
MAPK11	CAGGTCGGCGCCCATCAGGG	chr22:50705878-50705897_MAPK11_-	exon_04

MAPK11	CTGCGGTCGCACCTACCCGG	chr22:50705670-50705689_MAPK11_-	exon_05
MAPK12	GGAGGTGACCAAGACGGCCT	chr22:50699878-50699897_MAPK12_-	exon_01
MAPK12	CTTGATGGCCACCTTAGCGC	chr22:50699683-50699702_MAPK12_-	exon_02
MAPK12	ACAGATCCTGGACTTCGGCC	chr22:50694613-50694632_MAPK12_-	exon_07
MAPK12	ACAGGCAAGACGCTGTTCAA	chr22:50694250-50694269_MAPK12_-	exon_08
MAPK12	CGCTGCACAAACTCAGCCGG	chr22:50694060-50694079_MAPK12_-	exon_09
MAPK13	CAAGTCATAGAAGTTGCGCA	chr6:36100438-36100457_MAPK13_-	exon_03
MAPK13	ATAGTACATCCACTCTGCTG	chr6:36103792-36103811_MAPK13_+	exon_05
MAPK13	GCAGAAGCTGAACGACAAAG	chr6:36106200-36106219_MAPK13_+	exon_09
MAPK13	GCTGAGTGAAATCCTTCCTG	chr6:36106514-36106533_MAPK13_-	exon_10
MAPK13	AGAACACGAGAAACTCACAG	chr6:36106798-36106817_MAPK13_+	exon_11
MAPK14	GCTGAACAAGACAATCTGGG	chr6:35995970-35995989_MAPK14_+	exon_01
MAPK14	AAAGAACCTACAGAGAACTG	chr6:36020556-36020575_MAPK14_+	exon_02
MAPK14	CCTGTTAATGAGATAAGCGG	chr6:36068010-36068029_MAPK14_-	exon_09
MAPK14	TATATGGTCAACTTACCCAG	chr6:36070423-36070442_MAPK14_-	exon_10
MAPK14	AGCAAAGTAGGCATGTGCAA	chr6:36075298-36075317_MAPK14_-	exon_11
MAPK1	CCCGGCAGCCAACATGGCGG	chr22:22221724-22221743_MAPK1_-	exon_01
MAPK1	CCTACTGCCAGAGAACCCTG	chr22:22162048-22162067_MAPK1_-	exon_02
MAPK1	GATCCAGACCATGATCACAC	chr22:22153368-22153387_MAPK1_-	exon_04
MAPK1	TTTGGGAACAGCCTGTTCCA	chr22:22142560-22142579_MAPK1_+	exon_06
MAPK1	TGACATTCAACCCACACAAG	chr22:22127228-22127247_MAPK1_-	exon_07
MAPK3	GCAGTTGCAGTACATCGGCG	chr16:30134383-30134402_MAPK3_-	exon_01
MAPK3	AGTAGGTCTGATGTTCGAAG	chr16:30133254-30133273_MAPK3_+	exon_02
MAPK3	TGGAGGGCTTTAGATCTCGG	chr16:30129702-30129721_MAPK3_+	exon_03
MAPK3	TGACCACACCGGCTTCCTGA	chr16:30129424-30129443_MAPK3_-	exon_04
MAPK3	GATCCAGGTAGTGCTTGCCA	chr16:30129009-30129028_MAPK3_+	exon_05
MAPK7	GAAGGAGCGGGCTTTAAGCA	chr17:19282329-19282348_MAPK7_-	exon_02
MAPK7	GAATTCGCCATAGGGCACGG	chr17:19283233-19283252_MAPK7_-	exon_03
MAPK7	AAAGGCAAAGTCAAAGGGCG	chr17:19284604-19284623_MAPK7_-	exon_04
MAPK7	GCGGGAGCGAAAGGAACGGG	chr17:19285227-19285246_MAPK7_+	exon_05
MAPK7	TGTGTTCTCAGGCACACCAA	chr17:19286146-19286165_MAPK7_+	exon_06
MAPK8	TCTTTGCCAAGTGATTCAGA	chr10:49618103-49618122_MAPK8_+	exon_05
MAPK8	TCGCTACTACAGAGCACCCG	chr10:49628311-49628330_MAPK8_+	exon_06
MAPK8	TCAACGTAAGTCCTTACTGT	chr10:49634005-49634024_MAPK8_-	exon_08
MAPK8	GGGAACACACAATAGAAGAG	chr10:49635160-49635179_MAPK8_+	exon_10
MAPK8	CCAAGAATGGAGTTATACGG	chr10:49639275-49639294_MAPK8_+	exon_11
MAPK9	GAAGGATACGGTCAGTGCCT	chr5:179674430-179674449_MAPK9_-	exon_07
MAPK9	TCACGGCTTCTGCTTCGGCG	chr5:179668027-179668046_MAPK9_-	exon_09
MAPK9	TAGTGATTGATCCTGACAAG	chr5:179668103-179668122_MAPK9_-	exon_09
MAPK9	TGGGCATCATAAATTTGAGG	chr5:179666962-179666981_MAPK9_-	exon_10
MAPK9	TGAGGCCAGCGTCTGCTCAG	chr5:179663438-179663457_MAPK9_-	exon_12
MAPT	GAGATCCCAGAAGGAACCAC	chr17:44051816-44051835_MAPT_+	exon_03
MAPT	TGGAGAGGAAATCCACAGGG	chr17:44060971-44060990_MAPT_-	exon_05
MAPT	CACCCGGGAGCCCAAGAAGG	chr17:44073865-44073884_MAPT_+	exon_10
MAPT	AATATCAAACACGTCCCGGG	chr17:44087739-44087758_MAPT_+	exon_11
MAPT	AATATCACCCACGTCCCTGG	chr17:44096061-44096080_MAPT_+	exon_13
MCL1	AGGCGCTGGAGACCTTACGA	chr1:150551365-150551384_MCL1_-	exon_01
MCL1	TCTGGCGTGAGGGTGGACGG	chr1:150551792-150551811_MCL1_+	exon_01
MCL1	GCTTCCGCCAATCACCGCGC	chr1:150551827-150551846_MCL1_+	exon_01
MCL1	ATCGGACTCAACCTCTACTG	chr1:150551960-150551979_MCL1_-	exon_01
MCL1	CATGTAGAGGACCTAGAAGG	chr1:150549927-150549946_MCL1_-	exon_03

MDM2	TTGGTAGTAGTCAATCAGCA	chr12:69214133-69214152_MDM2_+	exon_05
MDM2	AACAGGTGTCACCTTGAAGG	chr12:69218175-69218194_MDM2_+	exon_06
MDM2	GAGAACATTACCGGATTCGA	chr12:69222703-69222722_MDM2_-	exon_08
MDM2	AGGAAGCCAATTCTCACGAA	chr12:69233127-69233146_MDM2_-	exon_11
MDM2	CAGGAAGCCAATTCTCACGA	chr12:69233128-69233147_MDM2_-	exon_11
MEP1A	ATAGGGCTTGAAATCCACAC	chr6:46777214-46777233_MEP1A_-	exon_06
MEP1A	GTGGGACAGAACATTTCCAT	chr6:46787294-46787313_MEP1A_+	exon_07
MEP1A	TTTGAGAAGGCAAACATCTG	chr6:46794123-46794142_MEP1A_+	exon_09
MEP1A	AAGAATCCGAGACTCCAGTA	chr6:46797150-46797169_MEP1A_-	exon_10
MEP1A	CAGCCGACAGAAGCGGTCGG	chr6:46803146-46803165_MEP1A_+	exon_13
MET	TCTGTGAGAATACACTCCAG	chr7:116340024-116340043_MET_-	exon_02
MET	GCACCAGCCACACTGAACAA	chr7:116380982-116381001_MET_-	exon_05
MET	GCTGGGACTTTGGATTTCGG	chr7:116395461-116395480_MET_+	exon_06
MET	AGCAGGAAGGAACTTTACAG	chr7:116399523-116399542_MET_+	exon_10
MET	AAAGCCAGTGATGATCTCAA	chr7:116403274-116403293_MET_+	exon_11
MKNK1	GGTGCCGTGAGCCTACAGAA	chr1:47046224-47046243_MKNK1_-	exon_04
MKNK1	TCGGAGTAGGGTGTTTCGAG	chr1:47042281-47042300_MKNK1_-	exon_05
MKNK1	TGAGCGAGAAGCCAGCCGAG	chr1:47037780-47037799_MKNK1_-	exon_07
MKNK1	ACACCCTGCAGACCTCGCCC	chr1:47028326-47028345_MKNK1_+	exon_11
MKNK1	GTGGTCAGAGCATTTACCTC	chr1:47025889-47025908_MKNK1_+	exon_13
MS4A1	CAGGAGGATGTCTTCACTGG	chr11:60229940-60229959_MS4A1_+	exon_02
MS4A1	TCATGAAGAAGCTTTGCGTG	chr11:60229967-60229986_MS4A1_-	exon_02
MS4A1	CTCATGAAGAAGCTTTGCGT	chr11:60229968-60229987_MS4A1_-	exon_02
MS4A1	TGGGCTCTTCCACATTGCCC	chr11:60230492-60230511_MS4A1_+	exon_03
MS4A1	GTACCACACAGTCACACAGA	chr11:60230554-60230573_MS4A1_-	exon_03
MTOR	GAGCCGGAATGAGGAAACCA	chr1:11319358-11319377_MTOR_-	exon_02
MTOR	AGAGACTCTGTCCTTACCTA	chr1:11318525-11318544_MTOR_+	exon_03
MTOR	TGGCCGTCTTGCCATGGCAG	chr1:11317085-11317104_MTOR_-	exon_04
MTOR	TGAGAATCAGACAGGCACGA	chr1:11316096-11316115_MTOR_+	exon_05
MTOR	TGAGACCTTGGCCAAAGAGA	chr1:11313979-11313998_MTOR_-	exon_06
MYC	GCTGCACCGAGTCGTAGTCG	chr8:128750550-128750569_MYC_-	exon_02
MYC	AGGGCGAGCAGAGCCCGGAG	chr8:128750706-128750725_MYC_-	exon_02
MYC	GAAGGGTGTGACCGCAACGT	chr8:128750729-128750748_MYC_-	exon_02
MYC	GAAGGCGCTGGAGTCTTGCG	chr8:128751137-128751156_MYC_-	exon_02
MYC	CGAGGACGGAGAGAAGGCGC	chr8:128751149-128751168_MYC_-	exon_02
NAMPT	CCGGCCCGAGATGAATCCTG	chr7:105925321-105925340_NAMPT_-	exon_01
NAMPT	AGGAAATGTTCTCTTCACGG	chr7:105913023-105913042_NAMPT_-	exon_04
NAMPT	GTCCTGGTATCCAATCACAG	chr7:105909725-105909744_NAMPT_-	exon_05
NAMPT	CAAACCTCCACCAGAACCGA	chr7:105894882-105894901_NAMPT_-	exon_09
NAMPT	AGGGCCGATTATCTTTACAT	chr7:105893529-105893548_NAMPT_-	exon_10
NDST4	TGAGAAAGGCCCTCTTCCTG	chr4:115997544-115997563_NDST4_-	exon_02
NDST4	TCTCAACAAGGAATTTGCAC	chr4:115891588-115891607_NDST4_-	exon_04
NDST4	AATCAACATGGGCTATGCTG	chr4:115858626-115858645_NDST4_-	exon_05
NDST4	AACAACCCACAAGTCTGTCG	chr4:115856462-115856481_NDST4_+	exon_06
NDST4	ATGAAGATCCAGCTGCTCTG	chr4:115760672-115760691_NDST4_-	exon_11
NELFB	GACTGGTGGAACTCCAGCCG	chr9:140150428-140150447_NELFB_-	exon_02
NELFB	GATGCCGTCCCTGCAGCCCG	chr9:140150829-140150848_NELFB_+	exon_03
NELFB	GCTGTATCGAGCCTGCGCCG	chr9:140151317-140151336_NELFB_+	exon_04
NELFB	CGTGGGTGAAATCTGCACCG	chr9:140157638-140157657_NELFB_+	exon_05
NELFB	GCGGTTCGTGGACAGCAAGA	chr9:140158729-140158748_NELFB_+	exon_06
NFKB1	GAACAAGAAGTCTTACCCTC	chr4:103459086-103459105_NFKB1_+	exon_05

NFKB1	ACTGGAAGCACGAATGACAG	:hr4:103498084-103498103_NFKB1_+	exon_07
NFKB1	CAAGGAGATGGACCTCAGCG	:hr4:103500081-103500100_NFKB1_+	exon_08
NFKB1	ACAGCTGGATGTGTGACTGG	:hr4:103501736-103501755_NFKB1_+	exon_09
NFKB1	CCGAAGCTGGACAAACACAG	chr4:103505897-103505916_NFKB1_-	exon_11
NOL6	GCAACAGTGAGGGTTTCAGG	chr9:33469509-33469528_NOL6_+	exon_05
NOL6	GGCCTGGTGGAAGTCAGCCA	chr9:33468394-33468413_NOL6_+	exon_10
NOL6	AGAGGCAGACCAGCCTGAGG	chr9:33467391-33467410_NOL6_-	exon_13
NOL6	ACACTCACAGTGCCAAGAGG	chr9:33467104-33467123_NOL6_+	exon_14
NOL6	ACAGGATGGACCCTCAGCCC	chr9:33464037-33464056_NOL6_-	exon_22
NOS2	GAGGTAGAAGCTCATCTGGA	chr17:26114747-26114766_NOS2_+	exon_05
NOS2	ATTGCGCCAGGCCTGCTTGG	chr17:26110012-26110031_NOS2_+	exon_06
NOS2	TACCTGAGTGAATTCCACGT	chr17:26108059-26108078_NOS2_+	exon_08
NOS2	GAAGCAGAATGTGACCATCA	chr17:26101459-26101478_NOS2_-	exon_12
NOS2	TATGCTGATGCGCAAGACAA	chr17:26099441-26099460_NOS2_-	exon_14
NOTCH1	TGAAGCGGCCAATGGCACGG	hr9:139438486-139438505_NOTCH1_	exon_02
NOTCH1	GAACGCCGGGACATGCCACG	hr9:139418346-139418365_NOTCH1_	exon_03
NOTCH1	GCACTGGCCACCGTTGGCGC	hr9:139417579-139417598_NOTCH1_	exon_04
NOTCH1	TCCTGCCAGAACACCCACGG	hr9:139405179-139405198_NOTCH1_	exon_17
NOTCH1	TCCTCGCCCTGCAAGAACGG	hr9:139402797-139402816_NOTCH1_	exon_20
NR2C2	AATCCAGATAGTCACCGCAG	chr3:15055125-15055144_NR2C2_+	exon_03
NR2C2	AAACAGGTCTTCTTGATCCA	chr3:15065624-15065643_NR2C2_+	exon_07
NR2C2	TAATAGGCTGAAACAAGCCA	chr3:15070087-15070106_NR2C2_+	exon_08
NR2C2	GCAGATGGGATAGACACCAG	chr3:15071843-15071862_NR2C2_+	exon_09
NR2C2	GGTGGAGAGACTCATGACCT	chr3:15076251-15076270_NR2C2_-	exon_11
NT5E	GAGCCACGCCACCCATGCAG	chr6:86160025-86160044_NT5E_-	exon_01
NT5E	GACGCCGGCGACCAGTACCA	chr6:86160101-86160120_NT5E_+	exon_01
NT5E	GATCGAGCCACTCCTCAAAG	chr6:86176819-86176838_NT5E_+	exon_02
NT5E	CAAAGGGCAATACAGCAGCC	chr6:86200248-86200267_NT5E_-	exon_07
NT5E	AGGTGACCAAGATATCAACG	chr6:86203557-86203576_NT5E_+	exon_09
NTRK1	CCGGGTGCATCGCAGTCCCG	chr1:156830863-156830882_NTRK1_-	exon_01
NTRK1	ACGGAGACCACTCTTCACGA	chr1:156834525-156834544_NTRK1_-	exon_03
NTRK1	AGGGCACAAGAACAGTGCAG	:hr1:156837915-156837934_NTRK1_-	exon_05
NTRK1	GGGCACCTGGACCTTCAGCG	chr1:156838306-156838325_NTRK1_-	exon_06
NTRK1	TGTGCAGCTGCACACGGCGG	:hr1:156843435-156843454_NTRK1_+	exon_08
NUDT1	ATGCCCAGGAGAACTCGCTG	chr7:2284255-2284274_NUDT1_-	exon_03
NUDT1	GCAAGAAGGAGAGACCATCG	chr7:2284326-2284345_NUDT1_+	exon_03
NUDT1	GGAGGAGAGCGGTCTGACAG	chr7:2289501-2289520_NUDT1_+	exon_04
NUDT1	AGTGGACGCCCTGCACAAGG	chr7:2289519-2289538_NUDT1_+	exon_04
NUDT1	CCTGGACTACACACTCCGCG	chr7:2290600-2290619_NUDT1_+	exon_05
NUP133	GACCGGCGAGAAGAGCACTG	hr1:229643841-229643860_NUP133_	exon_01
NUP133	GAAGCCCTAACATTGGCTGA	:hr1:229641789-229641808_NUP133_	exon_02
NUP133	AGTAGAATGTGCTTCACCTG	hr1:229636506-229636525_NUP133_	exon_04
NUP133	TGGTGAAGATACCTACACAG	:hr1:229635487-229635506_NUP133_	exon_05
NUP133	AATGATAGGAACACCACCAC	hr1:229622229-229622248_NUP133_	exon_11
OPN5	CAGGCGCTCGTCCTGAGGCA	chr6:47749825-47749844_OPN5_-	exon_01
OPN5	ATCCAGCCCATCCATACCAG	chr6:47759608-47759627_OPN5_-	exon_03
OPN5	TTTGGAAGAGGACTTAACCT	chr6:47763217-47763236_OPN5_-	exon_04
OPN5	TTTGCCTGTTGCCAAACTGG	chr6:47776070-47776089_OPN5_+	exon_05
OPN5	AGCAGAAGACTTCCTGACTG	chr6:47779427-47779446_OPN5_-	exon_06
ORAOV1	CCTCTCATCCGCCATCACGA	:hr11:69489957-69489976_ORAOV1_-	exon_01
ORAOV1	CATATTCGATGCCATCGTGA	:hr11:69489971-69489990_ORAOV1_	exon_01

ORAOV1	TATCGGGAAGGCTATGAAGA	:hr11:69488054-69488073_ORAOV1_	exon_02
ORAOV1	GCACAGTTGCACCACTGAGA	:hr11:69486524-69486543_ORAOV1_	exon_03
ORAOV1	GAAGACTTAGACAAGATCAG	:hr11:69482679-69482698_ORAOV1_	exon_04
P4HB	GACGTGGTCCTCCTCCTCGG	chr17:79818270-79818289_P4HB_+	exon_01
P4HB	GTTGGCCAAGGTGGACGCCA	chr17:79817156-79817175_P4HB_-	exon_02
P4HB	GCTGCCACCACCCTGCCTGA	chr17:79813390-79813409_P4HB_-	exon_03
P4HB	AGGCCGGAACAACTTTGAAG	chr17:79805196-79805215_P4HB_-	exon_05
P4HB	CAAGTACAAGCCCGAATCGG	chr17:79804367-79804386_P4HB_-	exon_07
PAK1	GATGCTGGAACCCTAAACCA	chr11:77103459-77103478_PAK1_-	exon_02
PAK1	ACACATCCAGAACAGCCTGC	chr11:77090343-77090362_PAK1_+	exon_04
PAK1	GGGCGTGGAGCAATCACTGG	chr11:77069959-77069978_PAK1_+	exon_06
PAK1	GGTTTGAGAAGATTGGACAA	chr11:77064583-77064602_PAK1_-	exon_08
PAK1	ACAGCAATGGATGTGGCCAC	chr11:77060294-77060313_PAK1_-	exon_09
PARP1	AAGAAGACAGCGGAAGCTGG	chr1:226589926-226589945_PARP1_-	exon_02
PARP1	AGCTAGGCATGATTGACCGC	chr1:226578260-226578279_PARP1_-	exon_04
PARP1	CAAGCAGCAAGTGCCTTCTG	chr1:226574038-226574057_PARP1_-	exon_06
PARP1	GACACAGACACCCAACCGGA	chr1:226573225-226573244_PARP1_-	exon_07
PARP1	AGAAACCAGCGCCTCCGTGG	chr1:226570800-226570819_PARP1_-	exon_08
PARP2	TCAGCGTTCGAATTCCATGG	chr14:20811785-20811804_PARP2_+	exon_01
PARP2	AGGACAGAAGACAAGCAAGA	chr14:20813225-20813244_PARP2_+	exon_02
PARP2	GGACCCAGAGTGTACAGCCA	chr14:20813593-20813612_PARP2_+	exon_03
PARP2	TATTAGAAGATGATGCCCAG	chr14:20818728-20818747_PARP2_+	exon_05
PARP2	AGGCAATCTCAACAAGGCCA	chr14:20819243-20819262_PARP2_+	exon_06
PARP3	AGGGCCCTCAGTCTGTACCC	chr3:51977394-51977413_PARP3_-	exon_02
PARP3	ACTTATCGAAGTACAGGCAG	chr3:51978546-51978565_PARP3_+	exon_04
PARP3	GGAGATGTTCAAGAACACCA	chr3:51978889-51978908_PARP3_+	exon_05
PARP3	GAGCAAGCAACAGATTGCAC	chr3:51979042-51979061_PARP3_+	exon_06
PARP3	TGAGCAGGAGAAGACGGTGG	chr3:51979555-51979574_PARP3_+	exon_07
PDCD1LG2	CCAGGCTCAACATTAGCAGG	chr9:5522558-5522577_PDCD1LG2_-	exon_02
PDCD1LG2	GTGGAACGAGGCCTTCCCTA	chr9:5534934-5534953_PDCD1LG2_-	exon_03
PDCD1LG2	TGTGGAACGAGGCCTTCCCT	chr9:5534935-5534954_PDCD1LG2_-	exon_03
PDCD1LG2	GGCCAGGATACTTCTGCCAG	chr9:5549421-5549440_PDCD1LG2_-	exon_04
PDCD1LG2	TGTTCTGGAATACTCACGTG	chr9:5549551-5549570_PDCD1LG2_+	exon_04
PDCD1	TCCAGGCATGCAGATCCCAC	chr2:242800978-242800997_PDCD1_-	exon_01
PDCD1	TCACCCTGAGCTCTGCCCGC	:hr2:242794776-242794795_PDCD1_-	exon_02
PDCD1	CGTGTCACACAACTGCCCAA	:hr2:242794904-242794923_PDCD1_-	exon_02
PDCD1	AGCCGGCCAGTTCCAAACCC	:hr2:242794437-242794456_PDCD1_-	exon_03
PDCD1	TCAGTGGCTGGGCACTCCGA	:hr2:242793245-242793264_PDCD1_-	exon_05
PDGFRA	AGGGTAATGAAAGCTGGCAG	chr4:55127278-55127297_PDGFRA_-	exon_03
PDGFRA	GGATTATTTAGTCATCGTGG	chr4:55129868-55129887_PDGFRA_+	exon_04
PDGFRA	ATCTCCACTGTCTTTCACCG	chr4:55133538-55133557_PDGFRA_-	exon_06
PDGFRA	CGTGCTAAGGAAGAAGACAG	chr4:55136825-55136844_PDGFRA_+	exon_08
PDGFRA	GACTTGGTCGATGATCACCA	chr4:55138578-55138597_PDGFRA_+	exon_09
PDGFRB	TGCACCCACAATGACTCCCG	hr5:149515165-149515184_PDGFRB_	exon_03
PDGFRB	ATTAGGGAGGAAGCCCACGG	hr5:149514554-149514573_PDGFRB_	exon_04
PDGFRB	CCCACATTACTTCTTTGCGG	hr5:149513434-149513453_PDGFRB_	exon_05
PDGFRB	AAAGGCCATCAACATCACCG	hr5:149513155-149513174_PDGFRB_	exon_06
PDGFRB	GTTCAAAGACAACCGCACCC	hr5:149512371-149512390_PDGFRB_	exon_07
PDILT	CAAGCCTGTGCACATCCTGG	chr16:20410502-20410521_PDILT_-	exon_02
PDILT	AGTGGACATTACCATAGAGA	chr16:20396057-20396076_PDILT_-	exon_03
PDILT	GAAGAAGCCAACGATGACCA	chr16:20387393-20387412_PDILT_+	exon_04

PDILT	ATGACTCCAAACGTTAGCTC	chr16:20386208-20386227_PDILT_+	exon_05
PDILT	TGTGATCGAATACAACACTG	chr16:20384336-20384355_PDILT_-	exon_06
PDK1	TTGCCGCAGAAACATAAATG	chr2:173423463-173423482_PDK1_-	exon_02
PDK1	AAAGTACTGAACATTCTGGC	chr2:173429341-173429360_PDK1_-	exon_04
PDK1	ACCAGGACAGCCAATACAAG	chr2:173433476-173433495_PDK1_+	exon_07
PDK1	ATGGAACACCATGCCAACAG	chr2:173435472-173435491_PDK1_+	exon_08
PDK1	TCAAAGGAACGCCACCTCCT	chr2:173451017-173451036_PDK1_-	exon_09
PGD	GCGAGTCACTCACTGGGCCA	chr1:10459176-10459195_PGD_-	exon_01
PGD	CGCCGCTTACCACAAAGCCG	chr1:10459752-10459771_PGD_-	exon_02
PGD	ACCAGGAGGATGATCCGCCG	chr1:10460570-10460589_PGD_-	exon_03
PGD	GGTGACATCATCATTGACGG	chr1:10463146-10463165_PGD_+	exon_04
PGD	GTGCCGAGACCTCAAGGCCA	chr1:10464223-10464242_PGD_+	exon_05
PHB2	TTCGCGCACACCGTAGGCCA	chr12:7079593-7079612_PHB2_+	exon_01
PHB2	GCAGGACACTATCCTGGCCG	chr12:7079375-7079394_PHB2_-	exon_02
PHB2	CAAGTGCTCAGACCTACCTT	chr12:7078644-7078663_PHB2_+	exon_03
PHB2	GGGTCGAGACAACACTCGCA	chr12:7077715-7077734_PHB2_+	exon_04
PHB2	GAAGCACTGAGCAAGAACCC	chr12:7076386-7076405_PHB2_-	exon_07
PIGF	GGTCCTAACAAACATAAGCA	chr2:46839406-46839425_PIGF_+	exon_04
PIGF	CATGGTCTTTCCCAATCCAG	chr2:46819618-46819637_PIGF_+	exon_05
PIGF	CACTGGATTGGGAAAGACCA	chr2:46819620-46819639_PIGF_-	exon_05
PIGF	TGGAGCACTTCCTATTCCAC	chr2:46819637-46819656_PIGF_-	exon_05
PIGF	CTGGAGACTATTCTCCCATA	chr2:46819692-46819711_PIGF_+	exon_05
PIK3CA	TTATTAATGTAGCCTCACGG	chr3:178916724-178916743_PIK3CA_	exon_02
PIK3CA	GTTCGAACAGGTATCTACCA	chr3:178922297-178922316_PIK3CA_	exon_06
PIK3CA	AGGAACACTGTCCATTGGCA	chr3:178927972-178927991_PIK3CA_	exon_08
PIK3CA	GAATAGGCAAGTCGAGGCAA	chr3:178938864-178938883_PIK3CA_	exon_14
PIK3CA	ACAGCCACACACTACATCAG	chr3:178947182-178947201_PIK3CA_	exon_18
PIK3CB	TAAGAAATGGTAGCTTCCCG	chr3:138478025-138478044_PIK3CB_	exon_02
PIK3CB	TGACAAGAAGTTGTGACCCA	chr3:138474639-138474658_PIK3CB_	exon_03
PIK3CB	TGTAGCGTGGGTAAATACGA	chr3:138431119-138431138_PIK3CB_	exon_09
PIK3CB	GAAGCTCTAGGGCCTCCCGG	chr3:138413692-138413711_PIK3CB_	exon_13
PIK3CB	AAAGAGCACTTGGTAATCGG	chr3:138409874-138409893_PIK3CB_	exon_14
PIK3CD	AGAGCGGCTCATACTGGGCG	chr1:9775613-9775632_PIK3CD_-	exon_04
PIK3CD	GATGTGCCAATTCTGCGAGG	chr1:9775962-9775981_PIK3CD_+	exon_05
PIK3CD	GTCCACCAAGGACGTGCCGC	chr1:9776518-9776537_PIK3CD_+	exon_06
PIK3CD	ATCTGCAGCTGCCTGCACAG	chr1:9777020-9777039_PIK3CD_+	exon_07
PIK3CD	GATGCGGAACGGCTGCTCCA	chr1:9777620-9777639_PIK3CD_-	exon_08
PIK3CG	GAAGGCTTGGGACTCCTCGG	chr7:106508437-106508456_PIK3CG_	exon_02
PIK3CG	GCTTCAGCAGAAATCTGGCA	chr7:106513017-106513036_PIK3CG_	exon_03
PIK3CG	AGTCCAGACACTATCAGCAG	chr7:106513210-106513229_PIK3CG_	exon_04
PIK3CG	TGGACTGAAAGCAGGAGCGC	chr7:106515224-106515243_PIK3CG_	exon_05
PIK3CG	AAATGCAACCATATGGCAGG	chr7:106522615-106522634_PIK3CG_	exon_07
PIM1	GTGGGCCCGCTACTGGGCAG	chr6:37138584-37138603_PIM1_+	exon_02
PIM1	GAAGGACCGGATTTCCGACT	chr6:37138777-37138796_PIM1_+	exon_03
PIM1	AAGTCGAAGAGATCTTGCAC	chr6:37139036-37139055_PIM1_-	exon_04
PIM1	GAGTGTATAGCCCTCCAGAG	chr6:37140778-37140797_PIM1_+	exon_05
PIM1	TGGATCTCAGCAGTTTCCTG	chr6:37141814-37141833_PIM1_-	exon_06
PIM2	TGTTGACCAAGCCTCTACAG	chrX:48776091-48776110_PIM2_-	exon_01
PIM2	GCTCCGAAGGTACTCACCAA	chrX:48775041-48775060_PIM2_+	exon_03
PIM2	ATCCTGATAGACCTACGCCG	chrX:48772368-48772387_PIM2_-	exon_04
PIM2	GATGCCCAGTGACCAGACAG	chrX:48771766-48771785_PIM2_+	exon_05

PIM2	CTGGATGCAAACACCAGCCG	chrX:48771473-48771492_PIM2_-	exon_06
PIM3	GCTGCAGGATCTTCACCGGG	chr22:50354658-50354677_PIM3_-	exon_01
PIM3	GCGGGTAGCCGCATCGCCGA	chr22:50354853-50354872_PIM3_+	exon_02
PIM3	GAAGGAGCGGGTGACCGAGT	chr22:50354986-50355005_PIM3_+	exon_03
PIM3	GCTGTGGCAGTGGCGCACGG	chr22:50355313-50355332_PIM3_-	exon_04
PIM3	GAGGCCGCCTGCTCTTCCGG	chr22:50356478-50356497_PIM3_+	exon_05
PLA2G2F	AGAACCCGCAACATGGCAGA	chr1:20465909-20465928_PLA2G2F_+	exon_01
PLA2G2F	TATGAAGAAGTTCTTCACCG	chr1:20466671-20466690_PLA2G2F_+	exon_02
PLA2G2F	GTAGCAACCGTAGCCCACGA	chr1:20470020-20470039_PLA2G2F_-	exon_03
PLA2G2F	TACCAGGAACTCTTTGACCA	chr1:20471098-20471117_PLA2G2F_+	exon_04
PLA2G2F	CATGAACCAGACGTACCGAG	chr1:20474750-20474769_PLA2G2F_+	exon_05
PLK1	ACCGGCGAAAGAGATCCCGG	chr16:23690358-23690377_PLK1_+	exon_01
PLK1	AGCCAAGCACAATTTGCCGT	chr16:23691467-23691486_PLK1_-	exon_02
PLK1	GAAAGGGCACAGTTTCGAGG	chr16:23692332-23692351_PLK1_+	exon_03
PLK1	CCTGCCTGACCATTCCACCA	chr16:23695324-23695343_PLK1_+	exon_05
PLK1	GCTCTGTGATAACAGCGTGG	chr16:23700566-23700585_PLK1_+	exon_08
PNPLA5	TACCTGGGCGCCCACCACGT	chr22:44287687-44287706_PNPLA5_-	exon_01
PNPLA5	TCAGCGAAATGCCCAGCCGC	chr22:44287007-44287026_PNPLA5_+	exon_02
PNPLA5	AAAGTATAAGGTGCAGACCA	chr22:44285721-44285740_PNPLA5_+	exon_03
PNPLA5	ACAGGGCCAGACCTACCTCG	chr22:44285193-44285212_PNPLA5_+	exon_04
PNPLA5	AAGGCTACCTGGATGCCCTG	chr22:44283490-44283509_PNPLA5_-	exon_05
POLR1C	GATGGCGGCTTCTCAGGCGG	chr6:43484847-43484866_POLR1C_+	exon_01
POLR1C	GGACCAGGACCGCTTCGAGA	chr6:43485094-43485113_POLR1C_+	exon_02
POLR1C	AAACTCACTGGAGTTTGACA	chr6:43487103-43487122_POLR1C_+	exon_03
POLR1C	CCAGGCTGATCTCTTTCCAG	chr6:43488050-43488069_POLR1C_+	exon_06
POLR1C	ATTGAGGTGCAGGAAGTCCA	chr6:43488491-43488510_POLR1C_+	exon_07
POLR2B	GTTTGGCAATATGTACGACG	chr4:57845142-57845161_POLR2B_+	exon_02
POLR2B	GAGCACCATCTCTTTCCCAA	chr4:57860620-57860639_POLR2B_-	exon_05
POLR2B	AAATAGCCACCAGGATCCAA	chr4:57860991-57861010_POLR2B_-	exon_06
POLR2B	AATCACTGACACCAACATGA	chr4:57871558-57871577_POLR2B_-	exon_09
POLR2B	TAGGAGAATTTAAACGACGC	chr4:57876568-57876587_POLR2B_-	exon_12
POLR2E	AATCCGCAAGACCATCATGC	chr19:1095260-1095279_POLR2E_-	exon_01
POLR2E	TTGGGCTTTGAACTCCTCCA	chr19:1094006-1094025_POLR2E_+	exon_02
POLR2E	GCAGGAGGAGAACATCACAC	chr19:1091838-1091857_POLR2E_-	exon_03
POLR2E	GGAGCTGCTCATCAACATCA	chr19:1090918-1090937_POLR2E_-	exon_04
POLR2E	TGAGCACGTCGTCATGACCA	chr19:1090117-1090136_POLR2E_-	exon_05
POLR3C	AGAGTAATTGCCCATGACAC	chr1:145609209-145609228_POLR3C_	exon_02
POLR3C	TTGGTAGTATAGATGTACCG	chr1:145608520-145608539_POLR3C_	exon_03
POLR3C	TTATACAGATGGCAAGACCA	chr1:145608282-145608301_POLR3C_	exon_04
POLR3C	GAGCGCAGTTGCTAACAGGA	chr1:145601756-145601775_POLR3C_	exon_06
POLR3C	GGAAAGTCTGGCGACAGTGG	chr1:145598264-145598283_POLR3C_	exon_09
PORCN	GATGGGAGGAATGTCGGCAG	chrX:48369810-48369829_PORCN_-	exon_03
PORCN	TATCCAGGGCCCGAAGACGA	chrX:48370834-48370853_PORCN_-	exon_05
PORCN	GATCCCTACAGAGCTGCCGG	chrX:48370966-48370985_PORCN_+	exon_06
PORCN	TCTGTAGACTCACCTTACCA	chrX:48372523-48372542_PORCN_-	exon_07
PORCN	GAAGGAGACAGCACTCTCGT	chrX:48372642-48372661_PORCN_-	exon_08
PPM1D	GGAGGCGGCACAGTTTGCCC	chr17:58678105-58678124_PPM1D_+	exon_01
PPM1D	AAGGAAAGAGAACGAATCGA	chr17:58701076-58701095_PPM1D_+	exon_02
PPM1D	ATTGAGATGGCATCTTGTGG	chr17:58725390-58725409_PPM1D_-	exon_04
PPM1D	GGAGCATACGCTGCCTCCAG	chr17:58734013-58734032_PPM1D_-	exon_05
PPM1D	TGGAGGAGGATCCATGGCCA	chr17:58740360-58740379_PPM1D_+	exon_06

PPP3R2	GATGGTGGGCAACAACCTGA	chr9:104356831-104356850_PPP3R2_	exon_01
PPP3R2	GGAGCTGCGCCACAACCCGT	chr9:104357044-104357063_PPP3R2_	exon_01
PPP3R2	GTTGTGGCGCAGCTCCGGCA	hr9:104357048-104357067_PPP3R2_	exon_01
PPP3R2	AAGTGGGAGCACATCTCCGC	hr9:104357160-104357179_PPP3R2_	exon_01
PPP3R2	AAACGAGGCCAGTTACCCGG	chr9:104357179-104357198_PPP3R2_	exon_01
PRKAA1	GTGATGGAATATGTCTCAGG	chr5:40775549-40775568_PRKAA1_-	exon_03
PRKAA1	TTTGAAACATATGCTGCAGG	chr5:40767605-40767624_PRKAA1_-	exon_06
PRKAA1	GAGGTAGATATATGGAGCAG	chr5:40767757-40767776_PRKAA1_-	exon_06
PRKAA1	AGAATGGTACTCTTTCAGGA	chr5:40765030-40765049_PRKAA1_+	exon_07
PRKAA1	GCAACTATCGATCTTGCCAA	chr5:40763048-40763067_PRKAA1_-	exon_09
PRKCA	GAGGCAGAAGAACGTGCACG	chr17:64299047-64299066_PRKCA_+	exon_01
PRKCA	AAACAAGGCTTCCAGTGCCA	chr17:64302224-64302243_PRKCA_+	exon_02
PRKCA	GCTCCACACTAAATCCGCAG	chr17:64683346-64683365_PRKCA_+	exon_06
PRKCA	GCTCCGAAACTCCAAAGGAA	chr17:64684510-64684529_PRKCA_-	exon_07
PRKCA	TACAACGTACCCATTCCGGA	chr17:64685103-64685122_PRKCA_+	exon_08
PRKCB	ATGCCCATTACTTACATCGG	chr16:23999907-23999926_PRKCB_-	exon_03
PRKCB	GCTGTATGGACTCATCCACC	chr16:24043531-24043550_PRKCB_+	exon_04
PRKCB	ACGGACCACACGGAGCGCCG	chr16:24046796-24046815_PRKCB_+	exon_05
PRKCB	GTAGGGATCTGACAGGCCAT	chr16:24104148-24104167_PRKCB_-	exon_06
PRKCB	CTGAAGTTCAGAAATCCCAA	chr16:24105579-24105598_PRKCB_-	exon_07
PRKCD	GATGCGCAGGAACGGCGCCA	chr3:53212440-53212459_PRKCD_-	exon_02
PRKCD	GGCCAGCACCGACACACCCA	chr3:53213734-53213753_PRKCD_-	exon_03
PRKCD	CTGAACAGACATCAACACCT	chr3:53215245-53215264_PRKCD_-	exon_04
PRKCD	TTCCCAACGATGAACCGCCG	chr3:53215498-53215517_PRKCD_+	exon_05
PRKCD	GCTGCCGCAGTGGTCACAGA	chr3:53217538-53217557_PRKCD_-	exon_08
PRKCE	AAGGCCATTGAACACTACCA	chr2:45879241-45879260_PRKCE_-	exon_01
PRKCE	ACCAGATGAAGTCTCTGCAA	chr2:46203710-46203729_PRKCE_-	exon_03
PRKCE	AATGCCTCTTACCTTGACAC	chr2:46206142-46206161_PRKCE_-	exon_04
PRKCE	ACCAACACTTACCTGGTCGG	chr2:46207513-46207532_PRKCE_-	exon_05
PRKCE	AACTGTGGAGTGGATGCCAG	chr2:46228590-46228609_PRKCE_+	exon_07
PRKCG	CCTCTGAATCGCCTACGCCG	chr19:54385766-54385785_PRKCG_-	exon_01
PRKCG	GGTGCACAAGCCCGTAGAGG	chr19:54392966-54392985_PRKCG_-	exon_04
PRKCG	GTGGACCACACCGAGCGCCG	chr19:54393196-54393215_PRKCG_+	exon_05
PRKCG	GACGAAACAGAAGACCCGAA	chr19:54395019-54395038_PRKCG_+	exon_06
PRKCG	GGAGCTGCTCAAGGCGCCCG	chr19:54395868-54395887_PRKCG_+	exon_07
PRKCH	GGAAGACGGCCAACTCGAGG	chr14:61789062-61789081_PRKCH_-	exon_01
PRKCH	GTAGGTGGATCTCGAGCCAG	chr14:61857939-61857958_PRKCH_+	exon_02
PRKCH	AGCGCCAAAGGGCTATGCGA	chr14:61909871-61909890_PRKCH_+	exon_03
PRKCH	AAACAGGGTTATCAGTGCCA	chr14:61912423-61912442_PRKCH_+	exon_04
PRKCH	GCAGGGATGGGTCTCCAACC	chr14:61920030-61920049_PRKCH_+	exon_07
PRKCI	GTGAGGAGATGCCGACCCAG	chr3:169940450-169940469_PRKCI_+	exon_01
PRKCI	TGTCTCGAACCTCATTGCAA	chr3:169953066-169953085_PRKCI_-	exon_02
PRKCI	ACTGAGATGATACTGTACAC	chr3:169977764-169977783_PRKCI_-	exon_03
PRKCI	ACCGTAGAGGTGCACGCCGC	chr3:169985712-169985731_PRKCI_+	exon_05
PRKCI	TCAACAGGCAATGAACACCA	chr3:169998008-169998027_PRKCI_+	exon_09
PRKCQ	AATCCGAAGAAATGGCGACA	chr10:6557077-6557096_PRKCQ_+	exon_02
PRKCQ	CAGCGAGTAGAGCTCCACGG	chr10:6552999-6553018_PRKCQ_+	exon_03
PRKCQ	TTTGCTTTGCATCAGCGCCG	chr10:6540463-6540482_PRKCQ_-	exon_05
PRKCQ	AACACTGTGGGACCCTGCTG	chr10:6533679-6533698_PRKCQ_-	exon_08
PRKCQ	GAAGCTAATGGCTGAAGCGC	chr10:6528023-6528042_PRKCQ_-	exon_09
PRKCSH	GCTACCCATGTGCTGGGCCG	chr19:11546962-11546981_PRKCSH_	exon_02

PRKCSH	GGTGGCCGAACCGTCCAGGC	:hr19:11547246-11547265_PRKCSH_	exon_03
PRKCSH	TCTGCCACAGACTGCTGCGA	:hr19:11548878-11548897_PRKCSH_	exon_05
PRKCSH	GAAGTCTCTGGAAGACCAGG	:hr19:11553230-11553249_PRKCSH_	exon_07
PRKCSH	GACGCGGTCGTAGAAAGAGG	:hr19:11557923-11557942_PRKCSH_	exon_10
PRKCZ	CAGCATTAAAGACGACTCGG	chr1:2080342-2080361_PRKCZ_+	exon_05
PRKCZ	TAAGCCAGTTATCGATGGGA	chr1:2082234-2082253_PRKCZ_+	exon_06
PRKCZ	GGAGTGTAATCCGACCAGGA	chr1:2087492-2087511_PRKCZ_-	exon_07
PRKCZ	CATTGAGTACGTCAACGGCG	chr1:2100969-2100988_PRKCZ_+	exon_08
PRKCZ	TGTGCCCGTCCGCATCCAGG	chr1:2103581-2103600_PRKCZ_-	exon_09
PRPF19	ACTGGAGCAGATTAGGGACA	:hr11:60673834-60673853_PRPF19_	exon_01
PRPF19	CAACAACCAGCCTCTCTCCG	chr11:60671208-60671227_PRPF19_-	exon_02
PRPF19	GAAGTCACTGCTGCCCGAGA	chr11:60670214-60670233_PRPF19_-	exon_04
PRPF19	GGCACAATGAGGCCAGCCTG	:hr11:60670089-60670108_PRPF19_	exon_05
PRPF19	GCGCTCCGTGGTTAGCACAG	:hr11:60668977-60668996_PRPF19_	exon_07
PRSS37	TGATGAGGACGCCCACACAG	hr7:141539178-141539197_PRSS37_	exon_02
PRSS37	ATGAGCATGAGGTCATCCTG	hr7:141537782-141537801_PRSS37_	exon_03
PRSS37	TGAGCATGAGGTCATCCTGT	hr7:141537783-141537802_PRSS37_	exon_03
PRSS37	TTCTCGATCAGACATCACGG	hr7:141536993-141537012_PRSS37_	exon_04
PRSS37	TTTGCAGATGACAGTAGCAA	hr7:141536306-141536325_PRSS37_	exon_05
PSMA7	CGTCAAGAAGGGCTCGACCG	chr20:60718266-60718285_PSMA7_-	exon_01
PSMA7	AGTGCGGAAGATCTGTGCTT	chr20:60715904-60715923_PSMA7_-	exon_02
PSMA7	TGTGGAGGACCCGGTCACTG	chr20:60714875-60714894_PSMA7_-	exon_03
PSMA7	CAGGCATGGTATGTGCCCGA	chr20:60714135-60714154_PSMA7_+	exon_04
PSMA7	GAGGCGAGATCAATCCCTCA	chr20:60712422-60712441_PSMA7_-	exon_06
PSMB1	TCGCAGCTGCAAAGGGCCCG	:hr6:170862244-170862263_PSMB1_-	exon_01
PSMB1	GATGGAACCGCACAGAGCCG	:hr6:170862264-170862283_PSMB1_	exon_01
PSMB1	TAATAAGGCCATGACTACGG	:hr6:170852782-170852801_PSMB1_	exon_04
PSMB1	TGAGAGAGATGTGTACACTG	:hr6:170844382-170844401_PSMB1_	exon_06
PSMB1	GCTGAGAGAGATGTGTACAC	:hr6:170844384-170844403_PSMB1_	exon_06
PSMD1	GGGCGCAGCCATGATCACCT	:hr2:231921730-231921749_PSMD1_-	exon_01
PSMD1	AAAGGAAGACAACCTCCTGA	:hr2:231936959-231936978_PSMD1_-	exon_07
PSMD1	AATGGCAACATACCTTCCCA	:hr2:231944881-231944900_PSMD1_-	exon_12
PSMD1	ACATAACCAAACCTAGGGCC	:hr2:231948297-231948316_PSMD1_	exon_14
PSMD1	ACCAGAGCCACAATAAGCCA	:hr2:231949773-231949792_PSMD1_	exon_15
PSMG3	GTTTGGGAAGATGGGCACCC	chr7:1608843-1608862_PSMG3_-	exon_01
PSMG3	GAAGGCCGTACACACCACCT	chr7:1608892-1608911_PSMG3_+	exon_01
PSMG3	ATCGAAGCAGAAGACGGAGG	chr7:1608933-1608952_PSMG3_-	exon_01
PSMG3	GATATCGAAGCAGAAGACGG	chr7:1608936-1608955_PSMG3_-	exon_01
PSMG3	CAGAGCAGTCCTCCTCGCCG	chr7:1607408-1607427_PSMG3_-	exon_02
PTCH1	GGAAATCTGCTCCAGAGCGA	chr9:98270446-98270465_PTCH1_+	exon_01
PTCH1	GAACCTCGAGACCAACGTGG	chr9:98268707-98268726_PTCH1_-	exon_02
PTCH1	CATGATCACACACTTACAGG	chr9:98244214-98244233_PTCH1_+	exon_05
PTCH1	GAGGTAAACCTCCTTTGCGG	chr9:98242852-98242871_PTCH1_-	exon_06
PTCH1	AAAGTATATGCACTGGCAGG	chr9:98242303-98242322_PTCH1_-	exon_07
PTGS2	TCAAGACAGATCATAAGCGA	chr1:186646814-186646833_PTGS2_-	exon_05
PTGS2	TTCAAGACAGATCATAAGCG	chr1:186646815-186646834_PTGS2_-	exon_05
PTGS2	TGAGCATCTACGGTTTGCTG	chr1:186645752-186645771_PTGS2_-	exon_07
PTGS2	GATGCTCAGGGACTTGAGGA	:hr1:186645764-186645783_PTGS2_	exon_07
PTGS2	CCAAAGATGGCATCTGGCCG	:hr1:186643786-186643805_PTGS2_	exon_10
PTK2	GCAATAGTGAGCCAACCACC	chr8:141900684-141900703_PTK2_-	exon_03
PTK2	GATTCCGCCTCAGTCACCTG	chr8:141889662-141889681_PTK2_-	exon_04

PTK2	AATAGATGGGTACTGCCGGC	chr8:141810603-141810622_PTK2_-	exon_12
PTK2	TCAGCTTCACAATATGAGGA	chr8:141756918-141756937_PTK2_+	exon_18
PTK2	AGGGTAGGAGGACAATTTGG	chr8:141745428-141745447_PTK2_+	exon_22
PTPN11	GGAGGAACATGACATCGCGG	ır12:112856908-112856927_PTPN11_	exon_01
PTPN11	GTAGGATCTGCACAGTTCAG	hr12:112888288-112888307_PTPN11_	exon_03
PTPN11	GTGCGCACTGGTGATGACAA	ır12:112891117-112891136_PTPN11_	exon_04
PTPN11	CTGACAGCGAATCATAACAT	hr12:112891172-112891191_PTPN11_	exon_04
PTPN11	ACGGCGTGCCCAGCGACCCT	ır12:112924331-112924350_PTPN11_	exon_11
PTPN6	GATGCAGAGACCCTGCTCAA	chr12:7060800-7060819_PTPN6_+	exon_02
PTPN6	GGTGTCCTGCAGGACCGCGA	chr12:7061264-7061283_PTPN6_+	exon_03
PTPN6	AAAGCACGAAGTCTCCAGGC	chr12:7064064-7064083_PTPN6_-	exon_04
PTPN6	CCAGGGTGGACGCTACACAG	chr12:7064310-7064329_PTPN6_+	exon_05
PTPN6	GGAGTCCGAGGATACAGCCA	chr12:7064603-7064622_PTPN6_+	exon_06
PVR	AACTGGCATGGCCCGAGCCA	chr19:45147390-45147409_PVR_+	exon_01
PVR	CAACCCGAACATCCTCAGCG	chr19:45150735-45150754_PVR_-	exon_02
PVR	TTGAGGGCACCAATATCCAG	chr19:45153268-45153287_PVR_-	exon_03
PVR	GGGTGGCCTCATTCTGGCCA	chr19:45157215-45157234_PVR_-	exon_04
PVR	ACAGTGCCAAAGGACCTCAC	chr19:45162133-45162152_PVR_-	exon_06
RABGGTB	TCCTGTATGAACTTTGACGG	hr1:76257194-76257213_RABGGTB_	exon_06
RABGGTB	TGTGAACGACAATTACCCTC	hr1:76257944-76257963_RABGGTB_	exon_07
RABGGTB	GAACGACAATTACCCTCAGG	hr1:76257947-76257966_RABGGTB_	exon_07
RABGGTB	ATGTCAAGATGAAGAAACGG	hr1:76259870-76259889_RABGGTB_	exon_08
RABGGTB	TGTCAAGATGAAGAAACGGG	hr1:76259871-76259890_RABGGTB_	exon_08
RAC1	TCACATCTAGTGGTATCCTG	chr7:6441489-6441508_RAC1_+	exon_05
RAC1	TCTAGTGGTATCCTGAGGTG	chr7:6441494-6441513_RAC1_+	exon_05
RAC1	CTATCCGCAGGGTCTAGCCA	chr7:6441624-6441643_RAC1_+	exon_05
RAC1	GCAGGGTCTAGCCATGGCTA	chr7:6441630-6441649_RAC1_+	exon_05
RAC1	TTAGCCATGGCTAGACCCTG	chr7:6441631-6441650_RAC1_-	exon_05
RAF1	GCCGAACAAGCAAAGAACAG	chr3:12660016-12660035_RAF1_-	exon_02
RAF1	TATGAAAGCACTCAAGGTGA	chr3:12653504-12653523_RAF1_-	exon_03
RAF1	TGTCAGAAATTCCTGCTCAA	chr3:12650364-12650383_RAF1_-	exon_05
RAF1	ATACGACGCATAGTCAAAGA	chr3:12647730-12647749_RAF1_+	exon_06
RAF1	GGAGAGGGAACCTTCAGATG	chr3:12645713-12645732_RAF1_+	exon_07
RARA	AGAGTGGTCAGAGCGCCTGG	chr17:38487582-38487601_RARA_-	exon_02
RARA	GCAGTCGGCAGTACTGGCAG	chr17:38506125-38506144_RARA_-	exon_04
RARA	TGAGAGCTACACGCTGACGC	chr17:38508217-38508236_RARA_+	exon_05
RARA	GAGGGTGATCTGGTCGGCGA	chr17:38508713-38508732_RARA_-	exon_06
RARA	GGGCAGCAGCTGGTTGGCGA	chr17:38510681-38510700_RARA_-	exon_07
RARB	TAGAAATCCAGGATTTGCCC	chr3:25470259-25470278_RARB_-	exon_01
RARB	GCAGGGTTTGTACACTCGAG	chr3:25502750-25502769_RARB_-	exon_02
RARB	TCTGCCAGGACAAATCATCA	chr3:25502774-25502793_RARB_+	exon_02
RARB	CAGGCAGGCGGCCTTCAGCA	chr3:25622185-25622204_RARB_-	exon_05
RARB	TAGCATCTCACCCAGCTCAG	chr3:25638047-25638066_RARB_+	exon_08
RARG	AAAGAGTCGCTCCTTATTGG	chr12:53621303-53621322_RARG_+	exon_03
RARG	AAGCATGGCTTGTAGACCCG	chr12:53609491-53609510_RARG_+	exon_04
RARG	GCTACAGAAGTGCTTCGAAG	chr12:53609095-53609114_RARG_-	exon_05
RARG	GTTAGAAGAGCTCATCACCA	chr12:53608292-53608311_RARG_-	exon_06
RARG	CCAGATCCAGCTGCACGCGG	chr12:53607983-53608002_RARG_+	exon_07
RAX	GGCAAGCGAGAAGCTCCCGT	chr18:56940385-56940404_RAX_+	exon_01
RAX	TGCGCGCCAGCCATGGCCGA	chr18:56940404-56940423_RAX_-	exon_01
RAX	CGAAGCGAAACTGTCAGAGG	chr18:56939751-56939770_RAX_-	exon_02

RAX	TGACAGTTTCGCTTCGCCGG	chr18:56939755-56939774_RAX_+	exon_02
RAX	TTCAGGAAGGGCGGAGGCGG	chr18:56936423-56936442_RAX_+	exon_03
RCC1	TTGGTGCTGACACTAGGCCA	chr1:28858347-28858366_RCC1_+	exon_06
RCC1	CTTGCAGCTCCACTTTCCCA	chr1:28858757-28858776_RCC1_-	exon_07
RCC1	CCTGAGGCCACCTTTACCAC	chr1:28861640-28861659_RCC1_-	exon_08
RCC1	TGATGGTGACCTCTACACCT	chr1:28861798-28861817_RCC1_+	exon_09
RCC1	AGAGGCCGAAGCCGTACACG	chr1:28862501-28862520_RCC1_-	exon_10
RD3	ATAGCAGGCCCACAATAGGA	chr1:211654468-211654487_RD3_+	exon_02
RD3	GCTGGCTGGCCAGCACACCC	chr1:211654557-211654576_RD3_-	exon_02
RD3	GCGGTCAGAAAGGTCTGCAC	chr1:211654591-211654610_RD3_-	exon_02
RD3	GAAGGGCGAGATGCGCGCGC	chr1:211652480-211652499_RD3_+	exon_03
RD3	GAAGGTGGCCAGGCTGCCGC	chr1:211652507-211652526_RD3_+	exon_03
RET	GAAGGCGACGTCCGGTGCCG	chr10:43572712-43572731_RET_+	exon_01
RET	ATTGGGCCTCTACTTCTCGA	chr10:43595911-43595930_RET_+	exon_02
RET	GAAGAAGGAGAAGTATACGC	chr10:43597883-43597902_RET_-	exon_03
RET	CTTCCGCTTGAACTCCACCA	chr10:43600619-43600638_RET_-	exon_04
RET	GGCCCAGCAGACCTTCCGGG	chr10:43601928-43601947_RET_+	exon_05
RNF17	AGTGCACCAGGTGTGGAAGG	chr13:25338433-25338452_RNF17_+	exon_01
RNF17	GTCCACATTGAAGTTCACAA	chr13:25341417-25341436_RNF17_-	exon_02
RNF17	TAGACAACGCTACTACCCAA	chr13:25348971-25348990_RNF17_+	exon_03
RNF17	TCAGGGCTAGATCCCATCTG	chr13:25367427-25367446_RNF17_-	exon_10
RNF17	CTTGATCTTGAACTGGACAG	chr13:25399840-25399859_RNF17_-	exon_16
ROCK1	CAGCAGCAACATGTCGACTG	chr18:18690862-18690881_ROCK1_-	exon_01
ROCK1	AAGGCATAAATCCACCAGGA	chr18:18629169-18629188_ROCK1_-	exon_04
ROCK1	TTGGATGCAATCCATTCCAT	chr18:18625268-18625287_ROCK1_-	exon_05
ROCK1	GAAGCCTGATAACATGCTGC	chr18:18624122-18624141_ROCK1_-	exon_06
ROCK1	GGAGCGTTTCCCAAGCCCAC	chr18:18619439-18619458_ROCK1_+	exon_09
ROCK2	GAGCCGCCAGAGGAAGCTGG	chr2:11484172-11484191_ROCK2_-	exon_01
ROCK2	TGCTTACCTGAACCACCCAG	chr2:11389780-11389799_ROCK2_+	exon_04
ROCK2	TCTGGATGCAATACACTCCA	chr2:11375929-11375948_ROCK2_-	exon_05
ROCK2	GAGGTTCTGAAATCACAAGG	chr2:11367451-11367470_ROCK2_-	exon_06
ROCK2	GAGTTGATTCACTTTCTCAG	chr2:11355456-11355475_ROCK2_+	exon_15
RPA1	GAACATCCGTCCCATTACTA	chr17:1747213-1747232_RPA1_+	exon_03
RPA1	ACAGTTGAACCCTCTCGTGG	chr17:1747888-1747907_RPA1_+	exon_04
RPA1	GTAAGGAGTGAGGCTGGCAA	chr17:1779060-1779079_RPA1_-	exon_07
RPA1	GTACCTGGAGCAACTCCCGA	chr17:1780547-1780566_RPA1_+	exon_08
RPA1	GGACGACCATCATTTACCTA	chr17:1782619-1782638_RPA1_+	exon_10
RPL8	TGAGCGGCACGGCTACATCA	chr8:146017391-146017410_RPL8_-	exon_02
RPL8	GTGCCGCTCAGCGAAATCCA	chr8:146017401-146017420_RPL8_+	exon_02
RPL8	CCGGAAGACCACCTTGGCGA	chr8:146017246-146017265_RPL8_+	exon_03
RPL8	GATAACCTTCTTGGAGCCGG	chr8:146016687-146016706_RPL8_+	exon_04
RPL8	CGCCCGGCCAGCCTTCAAGA	chr8:146015800-146015819_RPL8_+	exon_05
RPS5	CCTGTCCCAGGATGACCGAG	chr19:58899494-58899513_RPS5_+	exon_01
RPS5	GCCCTACCTCGCCTGTGAGC	chr19:58904540-58904559_RPS5_-	exon_02
RPS5	ACCTGGTTCACACGGCGCAG	chr19:58904837-58904856_RPS5_-	exon_03
RPS5	TCACCTGGTTCACACGGCGC	chr19:58904839-58904858_RPS5_-	exon_03
RPS5	CATTAAGAAGAAGGACGAGC	chr19:58906069-58906088_RPS5_+	exon_05
RPS6KB1	CATAGACCTGGACCAGCCAG	hr17:57970632-57970651_RPS6KB1_	exon_01
RPS6KB1	AATGAAAGCATGGACCATGG	hr17:57987932-57987951_RPS6KB1_	exon_02
RPS6KB1	TCAGAAACTAGTGTGAACAG	hr17:57990076-57990095_RPS6KB1_	exon_03
RPS6KB1	AGTAAAGCATCCCTTCATCG	hr17:58003861-58003880_RPS6KB1_	exon_05

RPS6KB1	TTACTTGGCAGAAATCTCCA	hr17:58008986-58009005_RPS6KB1_	exon_07
RRM1	GTAATCCAAGGCTTGTACAG	chr11:4127294-4127313_RRM1_+	exon_03
RRM1	TAATGGCAAACACTCTCCCA	chr11:4128702-4128721_RRM1_+	exon_04
RRM1	TACTGGCAGCTACATTGCTG	chr11:4139670-4139689_RRM1_+	exon_08
RRM1	AAGAGCAACCAGCAGAACCT	chr11:4144534-4144553_RRM1_+	exon_12
RRM1	AAACTACTATCCTGTACCAG	chr11:4148042-4148061_RRM1_+	exon_13
RS1	ATTGGGATTATCGTCTACCG	chrX:18675762-18675781_RS1_-	exon_02
RS1	GCATGCAAGTGCGATTGCCA	chrX:18674829-18674848_RS1_-	exon_03
RS1	TTGGCTGCAGGATGAAGGCG	chrX:18674869-18674888_RS1_-	exon_03
RS1	ATTTCAGGGATCCTCACCCA	chrX:18662656-18662675_RS1_-	exon_05
RS1	ATGCGGACGTGCCAGCCCAG	chrX:18660170-18660189_RS1_+	exon_06
RXFP3	GGTGCGGATTCTCATCAGCG	chr5:33937082-33937101_RXFP3_+	exon_01
RXFP3	CTTACACATGGCCTTGCCGA	chr5:33937294-33937313_RXFP3_-	exon_01
RXFP3	GATCCACACACACAGCGCCT	chr5:33937489-33937508_RXFP3_-	exon_01
RXFP3	GAAGAAGGACAGGACAACGA	chr5:33937834-33937853_RXFP3_-	exon_01
RXFP3	GGCGTCGTGGTCTACAGCGG	chr5:33938196-33938215_RXFP3_+	exon_01
RXRA	GGTGAGGGAGGAGTTCACCT	chr9:137293490-137293509_RXRA_-	exon_02
RXRA	GAGGACGCCATTGAGGCCCA	chr9:137300047-137300066_RXRA_-	exon_03
RXRA	TATGGAGTGTACAGCTGCGA	chr9:137300794-137300813_RXRA_+	exon_04
RXRA	CAAGACCGAGACCTACGTGG	chr9:137309125-137309144_RXRA_+	exon_05
RXRA	GATCCCGTCCTTCACGGCGA	chr9:137320996-137321015_RXRA_-	exon_07
RXRB	AGGCGGAGAACAACAAACCC	chr6:33168073-33168092_RXRB_-	exon_01
RXRB	ACTGGAAAGGGAGAGCCCAG	chr6:33167025-33167044_RXRB_+	exon_02
RXRB	GTGGCTTCACATCTTCAGGG	chr6:33166181-33166200_RXRB_+	exon_03
RXRB	GGACAACAAAGACTGCACAG	chr6:33165614-33165633_RXRB_-	exon_04
RXRB	GTCCACAGGCATCTCCTCGG	chr6:33164298-33164317_RXRB_+	exon_05
SAMD4B	GAAACGGGTCACCCGTACCC	:hr19:39847623-39847642_SAMD4B_-	exon_05
SAMD4B	TAGCCAACCGCTCTTCCAGG	:hr19:39860545-39860564_SAMD4B_	exon_06
SAMD4B	CAGCCGGAACACCTTCCAGG	:hr19:39866489-39866508_SAMD4B_-	exon_07
SAMD4B	TTATCACAGAACGTCACCAA	:hr19:39867336-39867355_SAMD4B_-	exon_09
SAMD4B	GGAGGCTCAGCACCTGGCAG	:hr19:39868278-39868297_SAMD4B_	exon_10
SERPINA7	TCATTCAAGAACTTTGCCAG	rX:105280604-105280623_SERPINA7	exon_01
SERPINA7	AGCTGCAGAAATGCTCACAG	rX:105280822-105280841_SERPINA7	exon_01
SERPINA7	AGTGCCCATGATGCACCAGA	rX:105279278-105279297_SERPINA7	exon_02
SERPINA7	TCTGGACTCACAGAGGACAA	rX:105278245-105278264_SERPINA7	exon_03
SERPINA7	AGGGAAAGTTGTGAACCCAA	rX:105277502-105277521_SERPINA7	exon_04
SERPINB12	ACCCAAGCGTACCATACCAA	r18:61223508-61223527_SERPINB12	exon_01
SERPINB12	TGGGTTGAATGTCAATCCCA	r18:61228414-61228433_SERPINB12	exon_04
SERPINB12	AATCAAGGAACTCTTCAGCA	r18:61231215-61231234_SERPINB12	exon_05
SERPINB12	CCTGGAAATGAGGTACACCA	r18:61232758-61232777_SERPINB12	exon_06
SERPINB12	GAGATCATAGCTGTCTTCCA	r18:61233937-61233956_SERPINB12	exon_07
SF3B3	ACTGGCCAGGAACGATGCGA	chr16:70563044-70563063_SF3B3_-	exon_03
SF3B3	GGACCACATGATTTAAACCA	chr16:70566456-70566475_SF3B3_-	exon_05
SF3B3	GGAGGGTCAGATGGTCCAAG	chr16:70569216-70569235_SF3B3_+	exon_06
SF3B3	AATGTGTCTCCTTCTTCCAG	chr16:70575639-70575658_SF3B3_-	exon_09
SF3B3	TCTCTGAGAGTCCTAAGACA	chr16:70578407-70578426_SF3B3_+	exon_10
SGK3	AATGCAAAGAGATCACACCA	chr8:67705971-67705990_SGK3_+	exon_02
SGK3	GGATGAAAGAAGTTCTCAGA	chr8:67734673-67734692_SGK3_+	exon_06
SGK3	TACCTCACAGAACATCAACC	chr8:67740897-67740916_SGK3_+	exon_07
SGK3	ACATACTGAGGATTTCCAGA	chr8:67740925-67740944_SGK3_-	exon_07
SGK3	CAGGCTGTAAGACTCACTCC	chr8:67759500-67759519_SGK3_-	exon_15

SLC13A1	GTGGCTCAGCAACACCTCGA	hr7:122809328-122809347_SLC13A1_	exon_04
SLC13A1	ACCAACCAGGAACAAATCCG	r7:122768915-122768934_SLC13A1_	exon_10
SLC13A1	ACAAGAATGGCTATATCCCA	r7:122763214-122763233_SLC13A1_	exon_12
SLC13A1	CATGCTGGTAATGAACCCAG	r7:122759235-122759254_SLC13A1_	exon_13
SLC13A1	CTCATAGCAGGAGCCCACGA	r7:122755591-122755610_SLC13A1_	exon_15
SLC36A3	TCAGAGAATGTCCATCCTGC	hr5:150682806-150682825_SLC36A3_	exon_01
SLC36A3	GATGCCTCTTACCAACAAGC	r5:150678142-150678161_SLC36A3_	exon_02
SLC36A3	AGAAGCAGGCCTCTGACCTC	r5:150675724-150675743_SLC36A3_	exon_03
SLC36A3	CCTGCCCGAACACCTGGCTG	hr5:150672948-150672967_SLC36A3_	exon_04
SLC36A3	GCAGATGTTGGAGGTCACGT	r5:150666990-150667009_SLC36A3_	exon_06
SMO	TGTAGCGCAGCGGCTCGCAG	chr7:128829199-128829218_SMO_-	exon_01
SMO	ATGTATACGGCACACAGCAG	chr7:128843266-128843285_SMO_-	exon_02
SMO	CCAGGGTGAAGAGCGTGCAG	chr7:128845235-128845254_SMO_-	exon_03
SMO	CACGGCAGACGATCTCTCGG	chr7:128845573-128845592_SMO_-	exon_04
SMO	GATGACAAAGATGATGACGC	chr7:128846011-128846030_SMO_-	exon_05
SPC24	GGTGCCGAGAAGCAGCTGCG	chr19:11266295-11266314_SPC24_-	exon_01
SPC24	ACATAGAGGAGGTGAGCCAG	chr19:11266417-11266436_SPC24_-	exon_01
SPC24	GGAGGAGGACACCCGTCTGA	chr19:11259783-11259802_SPC24_-	exon_02
SPC24	CCAGAGCCTTCTCAATGCGA	chr19:11259867-11259886_SPC24_-	exon_02
SPC24	TGGCAGAACCTACACGGCCG	chr19:11258676-11258695_SPC24_+	exon_03
SPEM1	CGGCCGCTCAACCATGGCCA	chr17:7323705-7323724_SPEM1_-	exon_01
SPEM1	GGGCCTCGGCCGCTCAACCA	chr17:7323711-7323730_SPEM1_-	exon_01
SPEM1	TAGCTCTGGAGCCGATTCCG	chr17:7323943-7323962_SPEM1_+	exon_02
SPEM1	GCTCCTAGCCACAAGAACGG	chr17:7324640-7324659_SPEM1_+	exon_03
SPEM1	GCTTCGGGAACTGACCCGGG	chr17:7324804-7324823_SPEM1_+	exon_03
SRC	TAGCAACAAGAGCAAGCCCA	chr20:36012562-36012581_SRC_+	exon_04
SRC	GTCATAGAGGGCCACAAAGG	chr20:36014490-36014509_SRC_-	exon_05
SRC	TCAATGCAGAGAACCCGAGA	chr20:36022624-36022643_SRC_+	exon_07
SRC	GTCTGACTTCGACAACGCCA	chr20:36024584-36024603_SRC_+	exon_08
SRC	TCCAGCCGCAGCGACTCCCG	chr20:36026197-36026216_SRC_-	exon_09
SRSF2	CCAAGAGGGAATCCAAATCC	chr17:74732303-74732322_SRSF2_-	exon_02
SRSF2	GGATTCCCTCTTGGACACTG	chr17:74732309-74732328_SRSF2_+	exon_02
SRSF2	GATTCCCTCTTGGACACTGG	chr17:74732310-74732329_SRSF2_+	exon_02
SRSF2	AGGACTCCTGGACCGAGACC	chr17:74732333-74732352_SRSF2_+	exon_02
SRSF2	ACGAGATCTGGAGACCGACG	chr17:74732369-74732388_SRSF2_+	exon_02
STAT3	CTACAGTGACAGCTTCCCAA	chr17:40500453-40500472_STAT3_-	exon_02
STAT3	GCAGGAAGCGGCTATACTGC	chr17:40498643-40498662_STAT3_+	exon_03
STAT3	GAAGGCGTGATTCTTCCCAC	chr17:40497603-40497622_STAT3_+	exon_04
STAT3	GGAACAGATGCTCACTGCGC	chr17:40489798-40489817_STAT3_-	exon_07
STAT3	ACAGCACCGGCCGATGCTGG	chr17:40485949-40485968_STAT3_-	exon_09
SYK	TTTCGGCAACATCACCCGGG	chr9:93606228-93606247_SYK_+	exon_02
SYK	GTGCAGCAGGCACAGGGCGT	chr9:93624517-93624536_SYK_-	exon_04
SYK	TGAGCCAGAACTTGCACCCT	chr9:93636539-93636558_SYK_+	exon_08
SYK	GGACCGAAAGCTGCTGACGC	chr9:93637045-93637064_SYK_+	exon_09
SYK	ATGGAAAGTGGCCTGTCAAG	chr9:93650041-93650060_SYK_+	exon_12
TBK1	TTTGAACATCCACTGGACGA	chr12:64854019-64854038_TBK1_-	exon_03
TBK1	ACAGTGTATAAACTCCCACA	chr12:64858156-64858175_TBK1_-	exon_04
TBK1	AATGGACCAATTGACTGGAG	chr12:64873850-64873869_TBK1_+	exon_07
TBK1	AACGTGGATGTACTTTAGGG	chr12:64879242-64879261_TBK1_-	exon_10
TBK1	GATGAAGATCAACCTGGAAG	chr12:64883833-64883852_TBK1_+	exon_13
TEC	AAACACTTACTTCACTGCGG	chr4:48158687-48158706_TEC_+	exon_09

TEC	GGAAGGTGCAATGTGCGAGG	chr4:48147456-48147475_TEC_-	exon_13
TEC	AATGACCTTGTCTCTGTCGG	chr4:48147185-48147204_TEC_+	exon_14
TEC	TCAGGTGGACACCACTTCAC	chr4:48140971-48140990_TEC_+	exon_16
TEC	ATGTGGGAAGTATTCACGGA	chr4:48140812-48140831_TEC_-	exon_17
TEK	AAAGCCAAACCTTACCAGAA	chr9:27109636-27109655_TEK_-	exon_01
TEK	GAACCAGCACCAGGATCCGC	chr9:27157971-27157990_TEK_+	exon_02
TEK	AGGAGGAAACCTCTTCACCT	chr9:27169581-27169600_TEK_+	exon_04
TEK	TATGGGAAGGACGTGTGAGA	chr9:27172723-27172742_TEK_+	exon_05
TEK	TGTGGCACAGGAACACCCAT	chr9:27173313-27173332_TEK_-	exon_06
TKTL2	GTGGAGGATCACTACAGGGA	chr4:164393181-164393200_TKTL2_-	exon_01
TKTL2	AGGCGGCCGAGTTATCACAG	chr4:164393200-164393219_TKTL2_-	exon_01
TKTL2	TATCTAGCCGCCAATACCAA	chr4:164393481-164393500_TKTL2_-	exon_01
TKTL2	AGAAGATGGAGTCTCCCAGA	chr4:164393590-164393609_TKTL2_-	exon_01
TKTL2	GGAGACTCCATCTTCTCCAG	chr4:164393594-164393613_TKTL2_+	exon_01
TNFRSF14	CAAGGAGGACGAGTACCCAG	chr1:2489221-2489240_TNFRSF14_+	exon_02
TNFRSF14	ACTTGCTTAGGCCATTGAGG	chr1:2489861-2489880_TNFRSF14_-	exon_03
TNFRSF14	GGTGGCGTAAGCGCGGCACG	chr1:2491367-2491386_TNFRSF14_-	exon_04
TNFRSF14	CTGGTGCTGACATTCCTCCA	chr1:2492129-2492148_TNFRSF14_-	exon_05
TNFRSF14	CTGGGACCAGCAGCTCCCAC	chr1:2493141-2493160_TNFRSF14_+	exon_06
TNFRSF4	GCCGGGCTTCTACAACGACG	chr1:1149088-1149107_TNFRSF4_-	exon_02
TNFRSF4	GCAGGCCTGGTTGTCGCCTG	chr1:1148032-1148051_TNFRSF4_+	exon_04
TNFRSF4	ACAGTGATGGGCCTGGCCGG	chr1:1147390-1147409_TNFRSF4_+	exon_05
TNFRSF4	GGCCATGAGGCACTCACCAG	chr1:1147067-1147086_TNFRSF4_+	exon_06
TNFRSF4	CCCAGGTCAGATCTTGGCCA	chr1:1146929-1146948_TNFRSF4_+	exon_07
TNFRSF8	GGCGCTACGAGCCTTCCCAC	chr1:12123697-12123716_TNFRSF8_-	exon_01
TNFRSF8	ACTATGACAAGGCTGTCAGG	chr1:12144564-12144583_TNFRSF8_-	exon_02
TNFRSF8	ACCTCGAGAACAAGTCACGC	chr1:12157257-12157276_TNFRSF8_	exon_03
TNFRSF8	GCCCGACTACTACCTGGACG	chr1:12172013-12172032_TNFRSF8_-	exon_07
TNFRSF8	ACCTCGAGAACAGCTCACGC	chr1:12172054-12172073_TNFRSF8_	exon_07
TNFRSF9	GCGCTGGAGAAACTATTTGG	chr1:7998822-7998841_TNFRSF9_+	exon_03
TNFRSF9	TCCAGGGTTTCACTGCCTGG	chr1:7998313-7998332_TNFRSF9_-	exon_04
TNFRSF9	AACGTGGCATCTGTCGACCC	chr1:7997758-7997777_TNFRSF9_-	exon_05
TNFRSF9	AAGGAGAGGGACGTGGTCTG	chr1:7995144-7995163_TNFRSF9_-	exon_06
TNFRSF9	TGGGACGAAGGAGAGGGACG	chr1:7995151-7995170_TNFRSF9_-	exon_06
TNFSF11	GTTCTTCTATTTCAGAGCGC	hr13:43148637-43148656_TNFSF11_	exon_01
TNFSF11	TGCAGATAGAGCTTACCAGA	chr13:43175114-43175133_TNFSF11_	exon_04
TNFSF11	GAAGCACCAAGTATTGGTCA	hr13:43180876-43180895_TNFSF11_	exon_05
TNFSF11	TGGAGAGGAAATCAGCATCG	hr13:43180955-43180974_TNFSF11_	exon_05
TNFSF11	GATCCGGATCCAGTAAGGAG	chr13:43180988-43181007_TNFSF11_	exon_05
TNFSF13B	GCTGTCTTGCTGCCTCACGG	13:108922411-108922430_TNFSF13B	exon_01
TNFSF13B	AGAGCTGCAGGGCCACCACG	13:108922486-108922505_TNFSF13B	exon_01
TNFSF13B	GAAGCTCCAGCTGTCACCGC	13:108922553-108922572_TNFSF13B	exon_01
TNFSF13B	TACTGATAAGACCTACGCCA	13:108955810-108955829_TNFSF13B	exon_05
TNFSF13B	AATTAGATGTCCCATGGCGT	13:108955824-108955843_TNFSF13B	exon_05
TNFSF4	GAGGAACAAGCTATTGCTGG	chr1:173176237-173176256_TNFSF4_	exon_01
TNFSF4	GATGACTTCCATGTGAATGG	chr1:173155707-173155726_TNFSF4_	exon_03
TNFSF4	CACATGGAAGTCATCCAGGG	hr1:173155712-173155731_TNFSF4_	exon_03
TNFSF4	AGTCAGAGAGGCCACCATCA	hr1:173155775-173155794_TNFSF4_	exon_03
TNFSF4	CCTGAAGGGCTACTTCTCCC	chr1:173155876-173155895_TNFSF4_	exon_03
TNFSF9	GCCAGCCCGAGACTCCGCGA	chr19:6531243-6531262_TNFSF9_+	exon_01
TNFSF9	GCAGGCGTGTCCCTGACGGG	chr19:6534658-6534677_TNFSF9_+	exon_03

TNFSF9	GAGCTACAAAGAGGACACGA	chr19:6534684-6534703_TNFSF9_+	exon_03
TNFSF9	GAAGATGGACGCCCAGGCGC	chr19:6534942-6534961_TNFSF9_-	exon_03
TNFSF9	CAGCCGGACTCCCTTCACCG	chr19:6535046-6535065_TNFSF9_+	exon_03
TNF	AGGCACTCACCTCTTCCCTC	chr6:31543695-31543714_TNF_-	exon_01
TNF	GAGACACTTACTGACTGCCT	chr6:31544348-31544367_TNF_-	exon_02
TNF	GCTGAGAGATAACCAGCTGG	chr6:31544966-31544985_TNF_+	exon_04
TNF	AGATAGATGGGCTCATACCA	chr6:31545180-31545199_TNF_-	exon_04
TNF	TATGAGCCCATCTATCTGGG	chr6:31545183-31545202_TNF_+	exon_04
TOMM34	GCGGAAACTCTCATTGCCGG	hr20:43588912-43588931_TOMM34_	exon_01
TOMM34	GCAGCATGTCACTTGAAGGA	hr20:43585063-43585082_TOMM34_	exon_02
TOMM34	GCAGATGCTCGCCGCAGCAG	hr20:43583808-43583827_TOMM34_	exon_03
TOMM34	TGAGCTGAAACAGGCACCAA	hr20:43580560-43580579_TOMM34_	exon_04
TOMM34	GCTGGATGGAAAGAACGTGA	hr20:43572135-43572154_TOMM34_	exon_06
TONSL	AAAGCCAAGGCGCAGAGGGC	chr8:145669625-145669644_TONSL_	exon_02
TONSL	GGCGCTCTCCGATCTTGCGG	hr8:145669300-145669319_TONSL_	exon_03
TONSL	AATAGCCAAGCTCTTCTCAA	hr8:145668540-145668559_TONSL_	exon_04
TONSL	CTTCCAGGGACACTGGCCCA	chr8:145668177-145668196_TONSL_	exon_05
TONSL	GCTCCCAGAAGCCTGTGCAG	chr8:145667451-145667470_TONSL_	exon_07
TOP1	GTACCTGGGAATCGTTGTGG	chr20:39657725-39657744_TOP1_-	exon_01
TOP1	AACACAAAGATCGAGAACAC	chr20:39690055-39690074_TOP1_+	exon_03
TOP1	GAGAAGACCAAACACAAAGA	chr20:39704848-39704867_TOP1_+	exon_04
TOP1	GAAGAAGAGCGCTATCCTGA	chr20:39721116-39721135_TOP1_+	exon_09
TOP1	AGAAATACGAGACTGCTCGG	chr20:39741438-39741457_TOP1_+	exon_14
TOP2A	ATGCTGCGGACAACAAACAA	chr17:38572298-38572317_TOP2A_-	exon_04
TOP2A	TATGGGAAGAGCTGGTGAGA	chr17:38569190-38569209_TOP2A_-	exon_07
TOP2A	TTAAACACACTTCCCACCTG	chr17:38567956-38567975_TOP2A_+	exon_08
TOP2A	TGTACGCTTATCCTGACTGA	chr17:38564337-38564356_TOP2A_-	exon_12
TOP2A	TAATCAGCAAGCCTTTGATG	chr17:38563158-38563177_TOP2A_+	exon_14
TOP2B	GCAGCCACCCGACTTGGCCA	chr3:25705768-25705787_TOP2B_+	exon_01
TOP2B	GTCAGTGGAGCCATTGACGC	chr3:25686793-25686812_TOP2B_-	exon_02
TOP2B	TGTAGGAATGAATTGCAGGG	chr3:25685233-25685252_TOP2B_-	exon_03
TOP2B	ATGCTGCTGACAATAAACAG	chr3:25683854-25683873_TOP2B_-	exon_04
TOP2B	AAAGTGATGGCCAATTGTGA	chr3:25670570-25670589_TOP2B_+	exon_14
TPH2	AATGACGACAAAGGCAACAA	chr12:72335388-72335407_TPH2_+	exon_02
TPH2	TCCAGAGAACATTTGGACAG	chr12:72338229-72338248_TPH2_+	exon_03
TPH2	CTAACTCAGAGATCTTCCGA	chr12:72338391-72338410_TPH2_-	exon_04
TPH2	TAAATACTGTGGCTACAGAG	chr12:72366431-72366450_TPH2_+	exon_06
TPH2	TGGCTTCACGGTGAGGCCGG	chr12:72372739-72372758_TPH2_+	exon_07
TRIM24	CAGGACAATGGAGGTGGCGG	hr7:138145287-138145306_TRIM24_	exon_01
TRIM24	GAGGACAACGCAGAAGCCAA	hr7:138199972-138199991_TRIM24_	exon_03
TRIM24	TCTAAATGGGCAGTTTCCAG	hr7:138223492-138223511_TRIM24_	exon_07
TRIM24	ATGCATCACACCTTGCACGA	hr7:138235834-138235853_TRIM24_	exon_08
TRIM24	AGAGGCAACAGGTGCAACGG	hr7:138239620-138239639_TRIM24_	exon_09
TRPC7	AGCGCCGGCACACAACGCTG	chr5:135693025-135693044_TRPC7_	exon_02
TRPC7	TCCGGCTCAGACTTGGACGG	hr5:135651317-135651336_TRPC7_	exon_03
TRPC7	GAAGTGGCATACTTCACCTA	chr5:135587339-135587358_TRPC7_	exon_06
TRPC7	GCGAGAGAAGCTCAGCACGA	hr5:135583341-135583360_TRPC7_	exon_07
TRPC7	GTTGAGCAACACTACCACCA	hr5:135567084-135567103_TRPC7_	exon_08
TRPV1	CTCGGAGGAGGCTTTCCCGG	chr17:3495464-3495483_TRPV1_-	exon_02
TRPV1	CTGGCAGTTATTCTGAGCAA	chr17:3494548-3494567_TRPV1_+	exon_03
TRPV1	GCGGCAAACGGACAGCCTGA	chr17:3494300-3494319_TRPV1_-	exon_04

TRPV1	GACGGCCGACATCAGCGCCA	chr17:3493304-3493323_TRPV1_-	exon_06
TRPV1	GCAGGACAGGTCGTACAGCG	chr17:3491545-3491564_TRPV1_+	exon_07
TSSK1B	TGTGGCCATCAACAAGGAGG	:hr5:112769675-112769694_TSSK1B_	exon_01
TSSK1B	GTGGCAGTACTTGATGGCCA	hr5:112770153-112770172_TSSK1B_	exon_01
TSSK1B	GCCAGAATCTCAATTTCCCG	hr5:112770343-112770362_TSSK1B_	exon_01
TSSK1B	TTTCTCCAAGAAGTCTGCGG	hr5:112770372-112770391_TSSK1B_	exon_01
TSSK1B	GAGGACAGCAGCGTCATCCA	hr5:112770516-112770535_TSSK1B_	exon_01
TUBB	ACCCAAAGTGTCTGACACCG	chr6:30691355-30691374_TUBB_+	exon_04
TUBB	TGAGCTGGCCAGGGAAACGG	chr6:30691559-30691578_TUBB_-	exon_04
TUBB	TGAGGTATCGGCCGTGGCGG	chr6:30691754-30691773_TUBB_-	exon_04
TUBB	ACCGTGGCTGCTGTCTTCCG	chr6:30691773-30691792_TUBB_+	exon_04
TUBB	GGAGGAGGATTTCGGTGAGG	chr6:30692135-30692154_TUBB_+	exon_04
TUBGCP2	AGCCTGCTGCGTGTCCACGG	r10:135116384-135116403_TUBGCP2	exon_01
TUBGCP2	TTTGACAACAGGTACACCAG	r10:135113508-135113527_TUBGCP2	exon_02
TUBGCP2	CAGCATCAACGTCCCTGCCG	r10:135113014-135113033_TUBGCP2	exon_03
TUBGCP2	GGTGCACAGGATCCTCCCAG	r10:135107094-135107113_TUBGCP2	exon_05
TUBGCP2	AATCAGGTGCTCCTTCACCA	r10:135106652-135106671_TUBGCP2	exon_06
TXN	GGTGAAGCAGATCGAGAGCA	chr9:113018694-113018713_TXN_-	exon_01
TYMS	ATCCAACACATCCTCCGCTG	chr18:657851-657870_TYMS_+	exon_01
TYMS	GTCAGGAAGGACGACCGCAC	chr18:657875-657894_TYMS_+	exon_01
TYMS	ACACGTTTGGTTGTCAGCAG	chr18:659652-659671_TYMS_-	exon_02
TYMS	TCACCACATAGAACTGGCAG	chr18:670729-670748_TYMS_-	exon_05
TYMS	TTTCGAAGAATCCTGAGCTT	chr18:672887-672906_TYMS_-	exon_07
TYRO3	AGACACTGTCAGCTTCACCG	chr15:41853346-41853365_TYRO3_-	exon_02
TYRO3	TCAGTGGAGCGCTCTGACGC	chr15:41853744-41853763_TYRO3_+	exon_03
TYRO3	AGAGGAACTACGAAGATCGG	chr15:41854859-41854878_TYRO3_+	exon_04
TYRO3	GCTGCTGGAAAGCTTTGTCA	chr15:41857254-41857273_TYRO3_-	exon_06
TYRO3	GGTGCGCTGTGCCAATGCCT	chr15:41859665-41859684_TYRO3_+	exon_07
T	ACAGAGCGCGAACTGCGCGT	chr6:166580949-166580968_T_-	exon_02
T	TCACCAACAAGCTCAACGGA	chr6:166580089-166580108_T_-	exon_03
T	TGGCTGGTGATCATGCGCTG	chr6:166579246-166579265_T_+	exon_04
T	TGATCACAAAGAGATGATGG	chr6:166578132-166578151_T_-	exon_06
T	GAGAGGGCACCTCCAAACTG	chr6:166576033-166576052_T_+	exon_07
UBQLN3	TCAGGCCCTCATTGCTACGG	chr11:5528865-5528884_UBQLN3_-	exon_02
UBQLN3	GGAGACCTAAGCTAAAGGCA	chr11:5530380-5530399_UBQLN3_+	exon_02
UBQLN3	GAGACCTAAGCTAAAGGCAG	chr11:5530381-5530400_UBQLN3_+	exon_02
UBQLN3	CAAGAGGCAGCACCGTGCCA	chr11:5530497-5530516_UBQLN3_-	exon_02
UBQLN3	AGGTGGAGAAGCCCTGCCAC	chr11:5530761-5530780_UBQLN3_-	exon_02
UTS2R	GACCGAGCCCAGCTCCCTGG	chr17:80332317-80332336_UTS2R_+	exon_01
UTS2R	ACATGGAGGCCACCGCACGC	chr17:80332440-80332459_UTS2R_-	exon_01
UTS2R	GACCACGTAGACGTACATGG	chr17:80332454-80332473_UTS2R_-	exon_01
UTS2R	GAGCAGCGAGCGCTACGCTG	chr17:80332632-80332651_UTS2R_+	exon_01
UTS2R	ACAGCGCTGGAAGCGGGCGC	chr17:80333243-80333262_UTS2R_-	exon_01
VCP	CAACAATTAACCGATTGGGA	chr9:35068292-35068311_VCP_+	exon_02
VCP	AATAACCTTCGTGTACGCCT	chr9:35067903-35067922_VCP_-	exon_03
VCP	TTAGCTCTCACCTTTCCGGA	chr9:35066661-35066680_VCP_+	exon_04
VCP	GACACAGTGATCCACTGCGA	chr9:35065270-35065289_VCP_-	exon_05
VCP	AAAGAGGGCAGGATGTCTCA	chr9:35064169-35064188_VCP_+	exon_06
VEGFA	GCTCTACCTCCACCATGCCA	chr6:43739028-43739047_VEGFA_+	exon_01
VEGFA	GGAGGGCAGAATCATCACGA	chr6:43742108-43742127_VEGFA_+	exon_02
VEGFA	GGTGATGTTGGACTCCTCAG	chr6:43745377-43745396_VEGFA_-	exon_03

VEGFA	ATGCGGATCAAACCTCACCA	chr6:43746200-43746219_VEGFA_+	exon_04
VEGFA	TCAGATGTGACAAGCCGAGG	chr6:43752274-43752293_VEGFA_+	exon_07
VTCN1	GAGCAGGATGAAATGTTCAG	chr1:117699348-117699367_VTCN1_-	exon_03
VTCN1	GTGATACAATGGCTGAAGGA	chr1:117699420-117699439_VTCN1_-	exon_03
VTCN1	TGGGCATCCCAAGTTGACCA	chr1:117695877-117695896_VTCN1_-	exon_04
VTCN1	TTGGGATGCCCAGACCACTG	chr1:117695885-117695904_VTCN1_+	exon_04
VTCN1	ACACCGCAAGGTCTCTGAGC	chr1:117695933-117695952_VTCN1_+	exon_04
WDR3	GATGGGCTACACTTAGCTGT	chr1:118477150-118477169_WDR3_+	exon_03
WDR3	AAGGTCCCACCATTTCACCA	chr1:118481117-118481136_WDR3_-	exon_05
WDR3	AGAGGATGGTGCCTTTGAGA	chr1:118483524-118483543_WDR3_+	exon_07
WDR3	TCTGCAAGATGAAATCCAGC	chr1:118485114-118485133_WDR3_+	exon_10
WDR3	TCAGTGAATACAATTCCACC	chr1:118486085-118486104_WDR3_-	exon_11
WDR43	AGAGCAGCAATGGCGGCGGG	chr2:29117556-29117575_WDR43_+	exon_01
WDR43	TTCGCTTTGGCCTCTACCGA	chr2:29117649-29117668_WDR43_+	exon_01
WDR43	ACAGAGTCAACTGCATACAG	chr2:29129342-29129361_WDR43_+	exon_03
WDR43	ATACAGTGGCATCAAGACAG	chr2:29129356-29129375_WDR43_+	exon_03
WDR43	TTTCCAGTTCTTCTTACCCA	chr2:29152523-29152542_WDR43_+	exon_11
WEE1	GTCGCCGGTCAAGTCGCCGG	chr11:9595846-9595865_WEE1_+	exon_01
WEE1	TCTACGACGACACTGTCCTG	chr11:9597601-9597620_WEE1_-	exon_02
WEE1	TCATCAACAGAGCCCGCCAA	chr11:9598187-9598206_WEE1_-	exon_04
WEE1	AGTTCGATATTTCTCTGCGT	chr11:9598746-9598765_WEE1_+	exon_05
WEE1	TCAGACAGGGTAGATTACCT	chr11:9608102-9608121_WEE1_+	exon_09
XPO1	GTGGTGAATTGCTTATACCA	chr2:61760923-61760942_XPO1_-	exon_02
XPO1	CTTCCAAGGAACCAGTGCGA	chr2:61749748-61749767_XPO1_-	exon_04
XPO1	AACAAGAATGGCCCAAACAT	chr2:61727003-61727022_XPO1_-	exon_07
XPO1	TCTCAGGGAAACTCTTATGG	chr2:61722592-61722611_XPO1_-	exon_11
XPO1	ACTTCCAGAAAGCAACGGAG	chr2:61721083-61721102_XPO1_+	exon_12

GENE	A549 T12 (sumZ, numOb, normZ, val, svr rnk, svr dr, svnl val, sui rnk, sui dr)	A549 T18 (sumZ, numOb, normZ, val, svr rnk, svr dr, svnl val, sui rnk, sui dr)	H1299 T12 (sumZ, numOb, normZ, val, svr rnk, svr dr, svnl val, sui rnk, sui dr)	H1299 T18 (sumZ, numOb, normZ, val, svr rnk, svr dr, svnl val, sui rnk, sui dr)	H2023 T15 (sumZ, numOb, normZ, val, svr rnk, svr dr, svnl val, sui rnk, sui dr)	H2023 T25 (sumZ, numOb, normZ, val, svr rnk, svr dr, svnl val, sui rnk, sui dr)
AARS	-3.64 10 -0.9 0.184 47 0.916 0.816 281 0.943	-1.64 10 -0.36 0.36 127 0.903 0.64 201 0.945	-2.35 10 -0.51 0.305 87 0.912 0.695 241 0.933	-1.8 10 -0.46 0.321 102 0.927 0.679 226 0.944	2.81 10 0.59 0.721 255 0.925 0.279 73 0.913	1.25 10 0.17 0.569 179 0.958 0.431 149 0.943
ABL1	[illegible]	[illegible]	[illegible]	[illegible]	[illegible]	[illegible]
ABL2	[illegible]	[illegible]	[illegible]	[illegible]	[illegible]	[illegible]
ADH7	[illegible]	[illegible]	[illegible]	[illegible]	[illegible]	[illegible]
ADORA2A	[illegible]	[illegible]	[illegible]	[illegible]	[illegible]	[illegible]
AKT1	[illegible]	[illegible]	[illegible]	[illegible]	[illegible]	[illegible]
AKT2	[illegible]	[illegible]	[illegible]	[illegible]	[illegible]	[illegible]
AKT3	[illegible]	[illegible]	[illegible]	[illegible]	[illegible]	[illegible]
ALK	[illegible]	[illegible]	[illegible]	[illegible]	[illegible]	[illegible]
APOBEC1	[illegible]	[illegible]	[illegible]	[illegible]	[illegible]	[illegible]
AR	[illegible]	[illegible]	[illegible]	[illegible]	[illegible]	[illegible]
ARCN1	[illegible]	[illegible]	[illegible]	[illegible]	[illegible]	[illegible]
ATM	[illegible]	[illegible]	[illegible]	[illegible]	[illegible]	[illegible]
ATP6V1A	[illegible]	[illegible]	[illegible]	[illegible]	[illegible]	[illegible]
ATR	[illegible]	[illegible]	[illegible]	[illegible]	[illegible]	[illegible]
AURKA	[illegible]	[illegible]	[illegible]	[illegible]	[illegible]	[illegible]
AURKB	[illegible]	[illegible]	[illegible]	[illegible]	[illegible]	[illegible]
AURKC	[illegible]	[illegible]	[illegible]	[illegible]	[illegible]	[illegible]
AXL	[illegible]	[illegible]	[illegible]	[illegible]	[illegible]	[illegible]
BCL2	[illegible]	[illegible]	[illegible]	[illegible]	[illegible]	[illegible]
BCR	[illegible]	[illegible]	[illegible]	[illegible]	[illegible]	[illegible]
BIRC5	[illegible]	[illegible]	[illegible]	[illegible]	[illegible]	[illegible]
BIRC8	[illegible]	[illegible]	[illegible]	[illegible]	[illegible]	[illegible]
BLK	[illegible]	[illegible]	[illegible]	[illegible]	[illegible]	[illegible]
BMP15	[illegible]	[illegible]	[illegible]	[illegible]	[illegible]	[illegible]
BMX	[illegible]	[illegible]	[illegible]	[illegible]	[illegible]	[illegible]
BPIFB6	[illegible]	[illegible]	[illegible]	[illegible]	[illegible]	[illegible]
BRAF	[illegible]	[illegible]	[illegible]	[illegible]	[illegible]	[illegible]
BRD4	[illegible]	[illegible]	[illegible]	[illegible]	[illegible]	[illegible]
BTK	[illegible]	[illegible]	[illegible]	[illegible]	[illegible]	[illegible]
BTLA	[illegible]	[illegible]	[illegible]	[illegible]	[illegible]	[illegible]
BUD31	[illegible]	[illegible]	[illegible]	[illegible]	[illegible]	[illegible]
C3orf30	[illegible]	[illegible]	[illegible]	[illegible]	[illegible]	[illegible]
C8B	[illegible]	[illegible]	[illegible]	[illegible]	[illegible]	[illegible]
CA9	[illegible]	[illegible]	[illegible]	[illegible]	[illegible]	[illegible]
CADM1	[illegible]	[illegible]	[illegible]	[illegible]	[illegible]	[illegible]
CASP3	[illegible]	[illegible]	[illegible]	[illegible]	[illegible]	[illegible]
CCR2	[illegible]	[illegible]	[illegible]	[illegible]	[illegible]	[illegible]
CCT2	[illegible]	[illegible]	[illegible]	[illegible]	[illegible]	[illegible]
CCT7	[illegible]	[illegible]	[illegible]	[illegible]	[illegible]	[illegible]
CD27	[illegible]	[illegible]	[illegible]	[illegible]	[illegible]	[illegible]
CD274	[illegible]	[illegible]	[illegible]	[illegible]	[illegible]	[illegible]
CD276	[illegible]	[illegible]	[illegible]	[illegible]	[illegible]	[illegible]
CD28	[illegible]	[illegible]	[illegible]	[illegible]	[illegible]	[illegible]
CD38	[illegible]	[illegible]	[illegible]	[illegible]	[illegible]	[illegible]
CD40	[illegible]	[illegible]	[illegible]	[illegible]	[illegible]	[illegible]
CD40LG	[illegible]	[illegible]	[illegible]	[illegible]	[illegible]	[illegible]
CD52	[illegible]	[illegible]	[illegible]	[illegible]	[illegible]	[illegible]
CD70	[illegible]	[illegible]	[illegible]	[illegible]	[illegible]	[illegible]
CD80	[illegible]	[illegible]	[illegible]	[illegible]	[illegible]	[illegible]
CD86	[illegible]	[illegible]	[illegible]	[illegible]	[illegible]	[illegible]
CDC16	[illegible]	[illegible]	[illegible]	[illegible]	[illegible]	[illegible]
CDH1	[illegible]	[illegible]	[illegible]	[illegible]	[illegible]	[illegible]
CDK1	[illegible]	[illegible]	[illegible]	[illegible]	[illegible]	[illegible]
CDK2	[illegible]	[illegible]	[illegible]	[illegible]	[illegible]	[illegible]
CDK4	[illegible]	[illegible]	[illegible]	[illegible]	[illegible]	[illegible]
CDK6	[illegible]	[illegible]	[illegible]	[illegible]	[illegible]	[illegible]
CDK7	[illegible]	[illegible]	[illegible]	[illegible]	[illegible]	[illegible]
CDK9	[illegible]	[illegible]	[illegible]	[illegible]	[illegible]	[illegible]
CDX4	[illegible]	[illegible]	[illegible]	[illegible]	[illegible]	[illegible]
CHEK1	[illegible]	[illegible]	[illegible]	[illegible]	[illegible]	[illegible]
CHEK2	[illegible]	[illegible]	[illegible]	[illegible]	[illegible]	[illegible]
CKAP5	[illegible]	[illegible]	[illegible]	[illegible]	[illegible]	[illegible]
CLDN17	[illegible]	[illegible]	[illegible]	[illegible]	[illegible]	[illegible]
CLRN1	[illegible]	[illegible]	[illegible]	[illegible]	[illegible]	[illegible]
CPSF3	[illegible]	[illegible]	[illegible]	[illegible]	[illegible]	[illegible]
CREBBP	[illegible]	[illegible]	[illegible]	[illegible]	[illegible]	[illegible]
CSTB	[illegible]	[illegible]	[illegible]	[illegible]	[illegible]	[illegible]
CTLA4	[illegible]	[illegible]	[illegible]	[illegible]	[illegible]	[illegible]
CTNNB1	[illegible]	[illegible]	[illegible]	[illegible]	[illegible]	[illegible]
CXCL1	[illegible]	[illegible]	[illegible]	[illegible]	[illegible]	[illegible]
CXCL3	[illegible]	[illegible]	[illegible]	[illegible]	[illegible]	[illegible]
CXorf66	[illegible]	[illegible]	[illegible]	[illegible]	[illegible]	[illegible]
DDOST	[illegible]	[illegible]	[illegible]	[illegible]	[illegible]	[illegible]
DDX20	[illegible]	[illegible]	[illegible]	[illegible]	[illegible]	[illegible]
DGAT2L6	[illegible]	[illegible]	[illegible]	[illegible]	[illegible]	[illegible]
DHFR	[illegible]	[illegible]	[illegible]	[illegible]	[illegible]	[illegible]
DHFRL1	[illegible]	[illegible]	[illegible]	[illegible]	[illegible]	[illegible]
DHX15	[illegible]	[illegible]	[illegible]	[illegible]	[illegible]	[illegible]
DHX8	[illegible]	[illegible]	[illegible]	[illegible]	[illegible]	[illegible]
DOT1L	[illegible]	[illegible]	[illegible]	[illegible]	[illegible]	[illegible]
DPAGT1	[illegible]	[illegible]	[illegible]	[illegible]	[illegible]	[illegible]
DRD2	[illegible]	[illegible]	[illegible]	[illegible]	[illegible]	[illegible]
DSPP	[illegible]	[illegible]	[illegible]	[illegible]	[illegible]	[illegible]
ECD	[illegible]	[illegible]	[illegible]	[illegible]	[illegible]	[illegible]
EGFR	[illegible]	[illegible]	[illegible]	[illegible]	[illegible]	[illegible]
EHMT2	[illegible]	[illegible]	[illegible]	[illegible]	[illegible]	[illegible]
EIF2S1	[illegible]	[illegible]	[illegible]	[illegible]	[illegible]	[illegible]
EIF4E	[illegible]	[illegible]	[illegible]	[illegible]	[illegible]	[illegible]
EIF6	[illegible]	[illegible]	[illegible]	[illegible]	[illegible]	[illegible]
ENTPD1	[illegible]	[illegible]	[illegible]	[illegible]	[illegible]	[illegible]
EPHA2	[illegible]	[illegible]	[illegible]	[illegible]	[illegible]	[illegible]
ERBB2	[illegible]	[illegible]	[illegible]	[illegible]	[illegible]	[illegible]
ERCC2	[illegible]	[illegible]	[illegible]	[illegible]	[illegible]	[illegible]
ESR1	[illegible]	[illegible]	[illegible]	[illegible]	[illegible]	[illegible]
ESR2	[illegible]	[illegible]	[illegible]	[illegible]	[illegible]	[illegible]
EZH2	[illegible]	[illegible]	[illegible]	[illegible]	[illegible]	[illegible]
F13B	[illegible]	[illegible]	[illegible]	[illegible]	[illegible]	[illegible]
FGF4	[illegible]	[illegible]	[illegible]	[illegible]	[illegible]	[illegible]
FGFR1	[illegible]	[illegible]	[illegible]	[illegible]	[illegible]	[illegible]
FGFR2	[illegible]	[illegible]	[illegible]	[illegible]	[illegible]	[illegible]
FGFR3	[illegible]	[illegible]	[illegible]	[illegible]	[illegible]	[illegible]
FGFR4	[illegible]	[illegible]	[illegible]	[illegible]	[illegible]	[illegible]
FGR	[illegible]	[illegible]	[illegible]	[illegible]	[illegible]	[illegible]
FLT1	[illegible]	[illegible]	[illegible]	[illegible]	[illegible]	[illegible]
FLT3	[illegible]	[illegible]	[illegible]	[illegible]	[illegible]	[illegible]
FLT4	[illegible]	[illegible]	[illegible]	[illegible]	[illegible]	[illegible]
FMR1NB	[illegible]	[illegible]	[illegible]	[illegible]	[illegible]	[illegible]
FRK	[illegible]	[illegible]	[illegible]	[illegible]	[illegible]	[illegible]
FYN	[illegible]	[illegible]	[illegible]	[illegible]	[illegible]	[illegible]
GABRA1	[illegible]	[illegible]	[illegible]	[illegible]	[illegible]	[illegible]
GFRAL	[illegible]	[illegible]	[illegible]	[illegible]	[illegible]	[illegible]
GLRA2	[illegible]	[illegible]	[illegible]	[illegible]	[illegible]	[illegible]
GLS	[illegible]	[illegible]	[illegible]	[illegible]	[illegible]	[illegible]
GPR50	[illegible]	[illegible]	[illegible]	[illegible]	[illegible]	[illegible]
GPX6	[illegible]	[illegible]	[illegible]	[illegible]	[illegible]	[illegible]
GSK3A	[illegible]	[illegible]	[illegible]	[illegible]	[illegible]	[illegible]
GSK3B	[illegible]	[illegible]	[illegible]	[illegible]	[illegible]	[illegible]
GSX2	[illegible]	[illegible]	[illegible]	[illegible]	[illegible]	[illegible]
GTF2B	[illegible]	[illegible]	[illegible]	[illegible]	[illegible]	[illegible]
HAVCR2	[illegible]	[illegible]	[illegible]	[illegible]	[illegible]	[illegible]
HDAC1	[illegible]	[illegible]	[illegible]	[illegible]	[illegible]	[illegible]
HDAC2	[illegible]	[illegible]	[illegible]	[illegible]	[illegible]	[illegible]
HDAC3	[illegible]	[illegible]	[illegible]	[illegible]	[illegible]	[illegible]
HDAC6	[illegible]	[illegible]	[illegible]	[illegible]	[illegible]	[illegible]
HDAC8	[illegible]	[illegible]	[illegible]	[illegible]	[illegible]	[illegible]
HLA-G	[illegible]	[illegible]	[illegible]	[illegible]	[illegible]	[illegible]
HSP90AA1	[illegible]	[illegible]	[illegible]	[illegible]	[illegible]	[illegible]
HTR5A	[illegible]	[illegible]	[illegible]	[illegible]	[illegible]	[illegible]
ICOS	[illegible]	[illegible]	[illegible]	[illegible]	[illegible]	[illegible]
ICOSLG	[illegible]	[illegible]	[illegible]	[illegible]	[illegible]	[illegible]
IDH1	[illegible]	[illegible]	[illegible]	[illegible]	[illegible]	[illegible]
IDH2	[illegible]	[illegible]	[illegible]	[illegible]	[illegible]	[illegible]
IGF1R	[illegible]	[illegible]	[illegible]	[illegible]	[illegible]	[illegible]
IGSF11	[illegible]	[illegible]	[illegible]	[illegible]	[illegible]	[illegible]
IKBKE	[illegible]	[illegible]	[illegible]	[illegible]	[illegible]	[illegible]
IL1B	[illegible]	[illegible]	[illegible]	[illegible]	[illegible]	[illegible]
IL6	[illegible]	[illegible]	[illegible]	[illegible]	[illegible]	[illegible]
IL6R	[illegible]	[illegible]	[illegible]	[illegible]	[illegible]	[illegible]
IMP3	[illegible]	[illegible]	[illegible]	[illegible]	[illegible]	[illegible]
INSR	[illegible]	[illegible]	[illegible]	[illegible]	[illegible]	[illegible]
IRAK4	[illegible]	[illegible]	[illegible]	[illegible]	[illegible]	[illegible]
ITK	[illegible]	[illegible]	[illegible]	[illegible]	[illegible]	[illegible]
JAK1	[illegible]	[illegible]	[illegible]	[illegible]	[illegible]	[illegible]
JAK2	[illegible]	[illegible]	[illegible]	[illegible]	[illegible]	[illegible]
JAK3	[illegible]	[illegible]	[illegible]	[illegible]	[illegible]	[illegible]
KCNK10	[illegible]	[illegible]	[illegible]	[illegible]	[illegible]	[illegible]
KDM1A	[illegible]	[illegible]	[illegible]	[illegible]	[illegible]	[illegible]
KDR	[illegible]	[illegible]	[illegible]	[illegible]	[illegible]	[illegible]
KIF11	[illegible]	[illegible]	[illegible]	[illegible]	[illegible]	[illegible]
KIT	[illegible]	[illegible]	[illegible]	[illegible]	[illegible]	[illegible]
KLK12	[illegible]	[illegible]	[illegible]	[illegible]	[illegible]	[illegible]
LAG3	[illegible]	[illegible]	[illegible]	[illegible]	[illegible]	[illegible]
LAP3	[illegible]	[illegible]	[illegible]	[illegible]	[illegible]	[illegible]
LAS1L	[illegible]	[illegible]	[illegible]	[illegible]	[illegible]	[illegible]
LCK	[illegible]	[illegible]	[illegible]	[illegible]	[illegible]	[illegible]
LDHA	[illegible]	[illegible]	[illegible]	[illegible]	[illegible]	[illegible]
LGALS3	[illegible]	[illegible]	[illegible]	[illegible]	[illegible]	[illegible]
LGALS9	[illegible]	[illegible]	[illegible]	[illegible]	[illegible]	[illegible]
LGALS9B	[illegible]	[illegible]	[illegible]	[illegible]	[illegible]	[illegible]
LUC7L3	[illegible]	[illegible]	[illegible]	[illegible]	[illegible]	[illegible]
LYN	[illegible]	[illegible]	[illegible]	[illegible]	[illegible]	[illegible]
MAP2	[illegible]	[illegible]	[illegible]	[illegible]	[illegible]	[illegible]
MAP2K1	[illegible]	[illegible]	[illegible]	[illegible]	[illegible]	[illegible]
MAP2K2	[illegible]	[illegible]	[illegible]	[illegible]	[illegible]	[illegible]
MAP3K8	[illegible]	[illegible]	[illegible]	[illegible]	[illegible]	[illegible]
MAPK1	[illegible]	[illegible]	[illegible]	[illegible]	[illegible]	[illegible]
MAPK11	[illegible]	[illegible]	[illegible]	[illegible]	[illegible]	[illegible]
MAPK12	[illegible]	[illegible]	[illegible]	[illegible]	[illegible]	[illegible]
MAPK13	[illegible]	[illegible]	[illegible]	[illegible]	[illegible]	[illegible]
MAPK14	[illegible]	[illegible]	[illegible]	[illegible]	[illegible]	[illegible]
MAPK3	[illegible]	[illegible]	[illegible]	[illegible]	[illegible]	[illegible]
[illegible]	[illegible]	[illegible]	[illegible]	[illegible]	[illegible]	[illegible]
MAPK7	[illegible]	[illegible]	[illegible]	[illegible]	[illegible]	[illegible]
MAPK8	[illegible]	[illegible]	[illegible]	[illegible]	[illegible]	[illegible]
MAPK9	[illegible]	[illegible]	[illegible]	[illegible]	[illegible]	[illegible]
MAPT	[illegible]	[illegible]	[illegible]	[illegible]	[illegible]	[illegible]
MCL1	[illegible]	[illegible]	[illegible]	[illegible]	[illegible]	[illegible]
MDM2	[illegible]	[illegible]	[illegible]	[illegible]	[illegible]	[illegible]
MEP1A	[illegible]	[illegible]	[illegible]	[illegible]	[illegible]	[illegible]
MET	[illegible]	[illegible]	[illegible]	[illegible]	[illegible]	[illegible]
MKNK1	[illegible]	[illegible]	[illegible]	[illegible]	[illegible]	[illegible]
MS4A1	[illegible]	[illegible]	[illegible]	[illegible]	[illegible]	[illegible]
MTOR	[illegible]	[illegible]	[illegible]	[illegible]	[illegible]	[illegible]
MYC	[illegible]	[illegible]	[illegible]	[illegible]	[illegible]	[illegible]
NAMPT	[illegible]	[illegible]	[illegible]	[illegible]	[illegible]	[illegible]
NDST4	[illegible]	[illegible]	[illegible]	[illegible]	[illegible]	[illegible]
NELFB	[illegible]	[illegible]	[illegible]	[illegible]	[illegible]	[illegible]
NFKB1	[illegible]	[illegible]	[illegible]	[illegible]	[illegible]	[illegible]
NOL6	[illegible]	[illegible]	[illegible]	[illegible]	[illegible]	[illegible]
NOS2	[illegible]	[illegible]	[illegible]	[illegible]	[illegible]	[illegible]
NOTCH1	[illegible]	[illegible]	[illegible]	[illegible]	[illegible]	[illegible]
NR2C2	[illegible]	[illegible]	[illegible]	[illegible]	[illegible]	[illegible]
NRP1	[illegible]	[illegible]	[illegible]	[illegible]	[illegible]	[illegible]
NT5E	[illegible]	[illegible]	[illegible]	[illegible]	[illegible]	[illegible]
NTRK1	[illegible]	[illegible]	[illegible]	[illegible]	[illegible]	[illegible]
NUDT1	[illegible]	[illegible]	[illegible]	[illegible]	[illegible]	[illegible]
NUP133	[illegible]	[illegible]	[illegible]	[illegible]	[illegible]	[illegible]
OPN5	[illegible]	[illegible]	[illegible]	[illegible]	[illegible]	[illegible]
ORAOV1	[illegible]	[illegible]	[illegible]	[illegible]	[illegible]	[illegible]
P4HB	[illegible]	[illegible]	[illegible]	[illegible]	[illegible]	[illegible]
PAK1	[illegible]	[illegible]	[illegible]	[illegible]	[illegible]	[illegible]
PARP1	[illegible]	[illegible]	[illegible]	[illegible]	[illegible]	[illegible]
PARP2	[illegible]	[illegible]	[illegible]	[illegible]	[illegible]	[illegible]
PARP3	[illegible]	[illegible]	[illegible]	[illegible]	[illegible]	[illegible]
PDCD1	[illegible]	[illegible]	[illegible]	[illegible]	[illegible]	[illegible]
PDCD1LG2	[illegible]	[illegible]	[illegible]	[illegible]	[illegible]	[illegible]
PDGFRA	[illegible]	[illegible]	[illegible]	[illegible]	[illegible]	[illegible]
PDGFRB	[illegible]	[illegible]	[illegible]	[illegible]	[illegible]	[illegible]
PDILT	[illegible]	[illegible]	[illegible]	[illegible]	[illegible]	[illegible]
PDK1	[illegible]	[illegible]	[illegible]	[illegible]	[illegible]	[illegible]
PGD	[illegible]	[illegible]	[illegible]	[illegible]	[illegible]	[illegible]
PHB2	[illegible]	[illegible]	[illegible]	[illegible]	[illegible]	[illegible]
PIGF	[illegible]	[illegible]	[illegible]	[illegible]	[illegible]	[illegible]
PIK3CA	[illegible]	[illegible]	[illegible]	[illegible]	[illegible]	[illegible]
PIK3CB	[illegible]	[illegible]	[illegible]	[illegible]	[illegible]	[illegible]
PIK3CD	[illegible]	[illegible]	[illegible]	[illegible]	[illegible]	[illegible]
PIK3CG	[illegible]	[illegible]	[illegible]	[illegible]	[illegible]	[illegible]
PIM1	[illegible]	[illegible]	[illegible]	[illegible]	[illegible]	[illegible]
PIM2	[illegible]	[illegible]	[illegible]	[illegible]	[illegible]	[illegible]
PIM3	[illegible]	[illegible]	[illegible]	[illegible]	[illegible]	[illegible]
PLA2G2F	[illegible]	[illegible]	[illegible]	[illegible]	[illegible]	[illegible]
PLK1	[illegible]	[illegible]	[illegible]	[illegible]	[illegible]	[illegible]
PNPLA5	[illegible]	[illegible]	[illegible]	[illegible]	[illegible]	[illegible]
POLR1C	[illegible]	[illegible]	[illegible]	[illegible]	[illegible]	[illegible]
POLR2B	[illegible]	[illegible]	[illegible]	[illegible]	[illegible]	[illegible]

**POLR2E**	-1.29	10	-0.4	0.346	107	0.916	0.654	221	0.943	-6.07	10	-1.32	0.0926	21	0.903	0.907	307	0.959	3.52	10	0.65	0.742	262	0.922	0.258	66	0.933	1.28	10	0.26	0.602	207	0.927	0.398	121	0.944	8.46	10	1.93	0.973	320	0.992	0.027	8	0.913	-3.68	10	-0.96	0.17	49	0.958	0.83	279	0.969
**POLR3C**	3.18	10	0.57	0.715	251	0.916	0.285	77	0.943	-4.53	10	-0.99	0.162	45	0.903	0.838	283	0.945	1.7	10	0.29	0.614	219	0.912	0.386	109	0.933	-1.03	10	-0.28	0.388	127	0.927	0.612	201	0.944	5.72	10	1.28	0.899	301	0.976	0.101	27	0.913	4.53	10	0.92	0.822	276	0.969	0.178	52	0.935
**PORCN**	2.38	10	0.39	0.653	223	0.916	0.347	105	0.943	3.67	10	0.8	0.788	272	0.948	0.212	56	0.945	0.19	10	-0.01	0.497	170	0.912	0.503	158	0.933	-2.38	10	-0.6	0.274	83	0.927	0.726	245	0.944	-1.28	10	-0.38	0.351	97	0.923	0.649	231	0.913	-3.66	10	-0.95	0.171	50	0.958	0.829	278	0.969
**PPM1D**	6.53	10	1.28	0.901	305	0.963	0.099	23	0.943	8.61	10	1.88	0.97	313	1	0.03	15	0.619	-1.74	10	-0.39	0.348	107	0.912	0.652	221	0.933	-2.53	10	-0.64	0.262	75	0.927	0.738	253	0.944	-2.92	10	-0.77	0.22	52	0.923	0.78	276	0.924	-0.3	10	-0.18	0.428	133	0.958	0.572	195	0.948
**PPP3R2**	-9.14	10	-2.08	0.019	6	0.916	0.981	322	0.997	-2.11	10	-0.46	0.323	107	0.903	0.677	221	0.945	-6.47	10	-1.32	0.0927	22	0.912	0.907	306	0.965	-6.32	10	-1.53	0.0632	20	0.927	0.937	308	0.98	-1.51	10	-0.44	0.331	94	0.923	0.669	234	0.913	-6.15	10	-1.52	0.0641	22	0.94	0.936	306	0.993
**PRKAA1**	2.22	10	0.36	0.64	218	0.916	0.36	110	0.943	1.88	10	0.41	0.66	236	0.903	0.34	92	0.945	3.74	10	0.69	0.756	265	0.932	0.244	63	0.933	7.68	10	1.76	0.961	314	1	0.0389	14	0.894	-3.88	10	-1	0.159	37	0.923	0.841	291	0.945	-0.29	10	-0.18	0.429	134	0.958	0.571	194	0.948
**PRKCA**	2.68	10	0.46	0.677	235	0.916	0.323	93	0.943	-3.85	10	-0.84	0.2	62	0.903	0.8	266	0.945	3.54	10	0.66	0.744	263	0.922	0.256	65	0.933	5.35	10	1.22	0.888	291	0.986	0.112	37	0.944	2.7	10	0.56	0.713	251	0.923	0.287	77	0.913	-7.72	10	-1.88	0.03	10	0.85	0.97	318	0.997
**PRKCB**	-0.18	10	-0.16	0.438	143	0.916	0.562	185	0.943	-0.62	10	-0.13	0.446	157	0.903	0.554	171	0.945	0.63	10	0.08	0.532	184	0.912	0.468	144	0.933	1.5	10	0.31	0.622	218	0.927	0.378	110	0.944	-4.13	10	-1.06	0.145	34	0.923	0.855	294	0.951	-2.31	10	-0.64	0.26	81	0.958	0.74	247	0.948
**PRKCD**	-3.24	10	-0.81	0.208	54	0.916	0.792	274	0.943	-8.52	10	-1.86	0.0315	8	0.903	0.968	320	0.988	1.49	10	0.25	0.598	211	0.912	0.402	117	0.933	4.37	10	0.99	0.838	283	0.965	0.162	45	0.944	5.83	10	1.3	0.904	302	0.976	0.0964	26	0.913	2.18	10	0.39	0.65	208	0.958	0.35	120	0.935
**PRKCE**	0.36	10	-0.04	0.484	160	0.916	0.516	168	0.943	0.61	10	0.13	0.553	195	0.903	0.447	133	0.945	0.97	10	0.15	0.559	197	0.912	0.441	131	0.933	-2.68	10	-0.67	0.25	70	0.927	0.75	258	0.944	-0.48	10	-0.19	0.423	128	0.923	0.577	200	0.913	5.37	10	1.12	0.868	290	0.973	0.132	38	0.935
**PRKCG**	3	10	0.53	0.701	242	0.916	0.299	86	0.943	2.08	10	0.45	0.675	240	0.903	0.325	88	0.945	2.02	10	0.35	0.638	228	0.914	0.362	100	0.933	-1.76	10	-0.46	0.324	105	0.927	0.676	223	0.944	-1.02	10	-0.32	0.374	107	0.923	0.626	221	0.913	2.9	10	0.55	0.709	237	0.958	0.291	91	0.935
**PRKCH**	4.17	10	0.78	0.782	276	0.926	0.218	52	0.943	1.34	10	0.29	0.616	221	0.903	0.384	107	0.945	-4.31	10	-0.9	0.185	46	0.912	0.815	282	0.945	0.12	10	-0.01	0.494	166	0.927	0.506	162	0.944	-1.15	10	-0.35	0.363	101	0.923	0.637	227	0.913	-0.75	10	-0.28	0.388	121	0.958	0.612	207	0.948
**PRKCI**	0.94	10	0.08	0.534	175	0.916	0.466	153	0.943	2.78	10	0.61	0.728	262	0.908	0.272	66	0.945	-0.97	10	-0.24	0.407	132	0.912	0.593	196	0.933	-2.43	10	-0.61	0.27	81	0.927	0.73	247	0.944	0.93	10	0.14	0.556	184	0.923	0.444	144	0.913	-6.27	10	-1.55	0.0608	20	0.94	0.939	308	0.993
**PRKCQ**	-0.43	10	-0.21	0.417	134	0.916	0.583	194	0.943	2.36	10	0.52	0.697	250	0.903	0.303	78	0.945	-2.9	10	-0.62	0.268	73	0.912	0.732	255	0.933	0.68	10	0.12	0.547	182	0.927	0.453	146	0.944	-5.31	10	-1.34	0.0905	20	0.923	0.91	308	0.966	-0.14	10	-0.15	0.442	140	0.958	0.558	188	0.948
**PRKCSH**	7.35	10	1.46	0.928	310	0.979	0.072	18	0.943	5.8	10	1.27	0.897	295	0.992	0.103	33	0.945	-2.34	10	-0.51	0.306	88	0.912	0.694	240	0.933	3.97	10	0.89	0.813	278	0.955	0.187	50	0.944	1.79	10	0.35	0.635	217	0.923	0.365	111	0.913	4.47	10	0.91	0.819	275	0.969	0.181	53	0.935
**PRKCZ**	3.12	10	0.55	0.71	250	0.916	0.29	78	0.943	2.01	10	0.44	0.67	239	0.903	0.33	89	0.945	-5.34	10	-1.1	0.136	33	0.912	0.864	295	0.956	-2.11	10	-0.54	0.295	94	0.927	0.705	234	0.944	-1.61	10	-0.46	0.323	89	0.923	0.677	239	0.913	0.63	10	0.03	0.512	163	0.958	0.488	165	0.948
**PRPF19**	3.55	10	0.65	0.741	262	0.916	0.259	66	0.943	2.29	10	0.5	0.692	247	0.903	0.308	81	0.945	-2.45	10	-0.53	0.298	83	0.912	0.702	245	0.933	-2.19	10	-0.56	0.289	91	0.927	0.711	237	0.944	0.89	10	0.13	0.552	182	0.923	0.448	146	0.913	-1.91	10	-0.55	0.291	89	0.958	0.709	239	0.948
**PRSS37**	1.13	10	0.13	0.55	184	0.916	0.45	144	0.943	-3.33	10	-0.73	0.233	73	0.903	0.767	255	0.945	-3.88	10	-0.81	0.208	54	0.912	0.792	274	0.945	-5.76	10	-1.4	0.0813	23	0.927	0.919	305	0.98	8.28	10	1.88	0.97	319	0.992	0.0298	9	0.913	0.59	10	0.02	0.509	162	0.958	0.491	166	0.948
**PSMA7**	2	10	0.31	0.622	209	0.916	0.378	119	0.943	-0.98	10	-0.21	0.415	141	0.903	0.585	187	0.945	4.05	10	0.75	0.775	270	0.933	0.225	58	0.933	7.39	10	1.69	0.955	313	0.998	0.0451	15	0.894	5.95	10	1.33	0.909	303	0.976	0.0915	25	0.913	-1.77	10	-0.52	0.302	95	0.958	0.698	233	0.948
**PSMB1**	-0.59	10	-0.24	0.404	128	0.916	0.596	200	0.943	2.26	10	0.49	0.689	246	0.903	0.311	82	0.945	-0.76	10	-0.19	0.423	140	0.912	0.577	188	0.933	-3.66	10	-0.9	0.183	47	0.927	0.817	281	0.944	9.21	10	2.11	0.982	323	0.992	0.0176	5	0.913	4.04	10	0.81	0.792	268	0.966	0.208	60	0.935
**PSMD1**	2.52	10	0.42	0.664	230	0.916	0.336	98	0.943	-0.18	10	-0.04	0.484	170	0.903	0.516	158	0.945	-0.95	10	-0.23	0.408	133	0.912	0.592	195	0.933	-0.25	10	-0.1	0.46	154	0.927	0.54	174	0.944	1.47	10	0.27	0.607	210	0.923	0.393	118	0.913	0.43	10	-0.01	0.494	155	0.958	0.506	173	0.948
**PSMG3**	6.41	10	1.26	0.896	304	0.963	0.104	24	0.943	-1.64	10	-0.36	0.36	126	0.903	0.64	202	0.945	2.23	10	0.4	0.654	231	0.914	0.346	97	0.933	1.32	10	0.27	0.606	209	0.927	0.394	119	0.944	6.52	10	1.47	0.929	309	0.978	0.0713	19	0.913	6.5	10	1.38	0.916	306	0.976	0.0843	22	0.935
**PTCH1**	1.46	10	0.2	0.578	198	0.916	0.422	130	0.943	1.75	10	0.38	0.649	229	0.903	0.351	99	0.945	-2.38	10	-0.52	0.303	86	0.912	0.697	242	0.933	-1.31	10	-0.35	0.363	119	0.927	0.637	209	0.944	0.35	10	0	0.502	163	0.923	0.498	165	0.913	2.48	10	0.45	0.675	222	0.958	0.325	106	0.935
**PTGS2**	10.99	10	2.24	0.988	320	1	0.012	8	0.507	5.42	9	1.25	0.894	294	0.992	0.106	34	0.945	1.33	10	0.22	0.586	206	0.912	0.414	122	0.933	5.62	9	1.35	0.911	298	0.986	0.0887	30	0.944	4.84	10	1.07	0.857	287	0.976	0.143	41	0.913	7	10	1.49	0.932	311	0.978	0.0681	17	0.935
**PTK2**	3.29	10	0.59	0.722	257	0.916	0.278	71	0.943	6.78	10	1.48	0.93	302	1	0.0695	26	0.874	5.93	10	1.13	0.87	293	0.971	0.13	35	0.933	2.45	10	0.53	0.703	245	0.935	0.297	83	0.944	-4.81	10	-1.22	0.111	25	0.923	0.889	303	0.958	9.89	10	2.15	0.984	321	0.996	0.0157	7	0.663
**PTPN11**	10.73	10	2.19	0.986	319	1	0.014	9	0.522	9.41	10	2.05	0.98	319	1	0.02	9	0.619	-0.99	10	-0.24	0.405	128	0.912	0.595	200	0.933	-2.03	10	-0.52	0.301	95	0.927	0.699	233	0.944	0.19	10	-0.03	0.487	156	0.923	0.513	172	0.913	2.86	10	0.54	0.706	232	0.958	0.294	96	0.935
**PTPN6**	4.85	10	0.93	0.823	285	0.944	0.177	43	0.943	3.66	10	0.8	0.788	271	0.948	0.212	57	0.945	-0.82	10	-0.21	0.418	137	0.912	0.582	191	0.933	0.15	10	-0.01	0.497	168	0.927	0.503	160	0.944	4.95	10	1.09	0.863	288	0.976	0.137	40	0.913	1.33	10	0.19	0.576	183	0.958	0.424	145	0.943
**PVR**	6.9	10	1.36	0.914	308	0.97	0.086	20	0.943	9.33	10	2.04	0.979	318	1	0.0208	10	0.619	2.83	10	0.51	0.696	249	0.914	0.304	79	0.933	4.44	10	1	0.841	285	0.965	0.159	43	0.944	-0.29	10	-0.15	0.442	135	0.923	0.558	193	0.913	-1.16	10	-0.38	0.352	112	0.958	0.648	216	0.948
**RABGGTB**	4.82	8	1.04	0.851	289	0.955	0.149	39	0.943	-7.83	8	-1.91	0.0281	7	0.903	0.972	321	0.988	9.9	10	1.91	0.972	315	1	0.0279	13	0.703	2.86	10	0.63	0.736	254	0.943	0.264	74	0.944	7.45	9	1.78	0.963	316	0.992	0.0373	12	0.913	-2.3	10	-0.64	0.261	82	0.958	0.739	246	0.948
**RAC1**	-10.98	10	-2.48	0.007	3	0.659	0.993	325	0.999	12.23	10	2.67	0.996	322	1	0.0038	6	0.207	12.84	10	2.49	0.994	322	1	0.0063	6	0.345	9.35	10	2.15	0.984	320	1	0.0156	8	0.632	6.86	10	1.55	0.939	314	0.978	0.0608	14	0.913	1.79	10	0.3	0.617	196	0.958	0.383	132	0.935
**RAF1**	8.48	10	1.7	0.956	315	0.992	0.044	13	0.943	1.91	10	0.42	0.662	237	0.903	0.338	91	0.945	1.27	10	0.21	0.581	205	0.912	0.419	123	0.933	4.95	10	1.12	0.869	288	0.986	0.131	40	0.944	8.88	10	2.03	0.979	321	0.992	0.0214	7	0.913	0.12	10	-0.09	0.466	146	0.958	0.534	182	0.948
**RARA**	2.59	10	0.44	0.67	232	0.916	0.33	96	0.943	5.14	10	1.12	0.869	287	0.99	0.131	41	0.945	0.14	10	-0.02	0.493	168	0.912	0.507	160	0.933	-6.43	10	-1.55	0.06	18	0.927	0.94	310	0.98	-3.49	10	-0.91	0.182	41	0.923	0.818	287	0.932	3.55	10	0.7	0.758	258	0.958	0.242	70	0.935
**RARB**	2.9	10	0.51	0.693	239	0.916	0.307	89	0.943	8.6	10	1.88	0.97	312	1	0.0303	16	0.619	-2.43	10	-0.53	0.3	85	0.912	0.7	243	0.933	-1.14	10	-0.31	0.378	122	0.927	0.622	206	0.944	-2.31	10	-0.63	0.266	68	0.923	0.734	260	0.92	1.56	10	0.24	0.597	187	0.958	0.403	141	0.935
**RARG**	-2.04	10	-0.56	0.289	85	0.916	0.711	243	0.943	2.53	10	0.55	0.71	257	0.903	0.29	71	0.945	-6.52	10	-1.33	0.0912	21	0.912	0.909	307	0.965	-9.05	10	-2.17	0.015	7	0.702	0.985	321	0.999	-4.35	10	-1.11	0.134	32	0.923	0.866	296	0.952	3.57	10	0.7	0.759	259	0.958	0.241	69	0.935
**RAX**	1.42	10	0.19	0.575	195	0.916	0.425	133	0.943	1.13	10	0.25	0.598	213	0.903	0.402	115	0.945	-5.61	10	-1.15	0.124	30	0.912	0.876	298	0.959	1.07	10	0.21	0.583	200	0.927	0.417	128	0.944	-3.27	10	-0.85	0.196	45	0.923	0.804	283	0.926	-2.51	10	-0.69	0.246	72	0.958	0.754	256	0.948
**RCC1**	2.62	10	0.45	0.672	233	0.916	0.328	95	0.943	1.71	10	0.37	0.646	228	0.903	0.354	100	0.945	0.13	10	-0.02	0.492	167	0.912	0.508	161	0.933	0.87	10	0.16	0.565	194	0.927	0.435	134	0.944	2.55	10	0.53	0.7	244	0.923	0.3	84	0.913	3.84	10	0.77	0.778	264	0.964	0.222	64	0.935
**RD3**	0.23	10	-0.07	0.473	155	0.916	0.527	173	0.943	1.76	10	0.38	0.649	231	0.903	0.351	97	0.945	-3.94	10	-0.82	0.205	53	0.912	0.795	275	0.945	-4.59	10	-1.12	0.131	35	0.927	0.869	293	0.97	2.37	10	0.48	0.685	235	0.923	0.315	93	0.913	3.27	10	0.64	0.738	249	0.958	0.262	79	0.935
**RET**	-5.99	10	-1.4	0.08	20	0.916	0.92	308	0.974	-5.59	10	-1.22	0.111	24	0.903	0.889	304	0.956	-0.21	10	-0.09	0.466	157	0.912	0.534	171	0.933	-0.26	10	-0.1	0.459	153	0.927	0.541	175	0.944	-4.53	10	-1.15	0.125	29	0.923	0.875	299	0.957	-0.78	10	-0.29	0.386	120	0.958	0.614	208	0.948
**RNF17**	5.85	10	1.14	0.873	299	0.955	0.127	29	0.943	0.27	10	0.06	0.523	184	0.903	0.477	144	0.945	-1.41	10	-0.32	0.373	114	0.912	0.627	214	0.933	-1.45	10	-0.38	0.351	115	0.927	0.649	213	0.944	-1	10	-0.32	0.376	109	0.923	0.624	219	0.913	-7.29	10	-1.78	0.0374	11	0.85	0.963	317	0.993
**ROCK1**	-7.23	10	-1.67	0.047	14	0.916	0.953	314	0.992	-6.27	10	-1.37	0.0856	17	0.903	0.914	311	0.959	-5.82	10	-1.2	0.116	27	0.912	0.884	301	0.961	-6.64	10	-1.6	0.0545	16	0.927	0.945	312	0.98	-0.43	10	-0.18	0.428	130	0.923	0.572	198	0.913	3.77	10	0.75	0.774	262	0.964	0.226	66	0.935
**ROCK2**	3.79	10	0.7	0.757	267	0.916	0.243	61	0.943	-0.72	10	-0.16	0.438	152	0.903	0.562	176	0.945	-14.61	10	-2.93	0.0017	3	0.182	0.998	325	1	-11.99	10	-2.86	0.0021	3	0.231	0.998	325	0.999	1.83	10	0.36	0.639	219	0.923	0.361	109	0.913	1.06	10	0.13	0.552	174	0.958	0.448	154	0.948
**RPA1**	3.24	10	0.58	0.719	253	0.916	0.281	75	0.943	-2.5	10	-0.54	0.293	94	0.903	0.707	234	0.945	2.76	10	0.5	0.692	245	0.914	0.308	83	0.933	4.09	10	0.92	0.821	281	0.955	0.179	47	0.944	3.91	10	0.85	0.802	274	0.954	0.198	54	0.913	4.09	10	0.82	0.795	269	0.966	0.205	59	0.935
**RPL8**	-2.46	10	-0.65	0.259	79	0.916	0.741	249	0.943	-2.36	10	-0.52	0.303	98	0.903	0.697	230	0.945	3.55	10	0.66	0.744	264	0.922	0.256	64	0.933	1.92	10	0.41	0.659	227	0.932	0.341	101	0.944	5.2	10	1.15	0.876	291	0.976	0.124	37	0.913	-1.52	10	-0.46	0.323	104	0.958	0.677	224	0.948
**RPS5**	7.98	10	1.6	0.945	311	0.991	0.055	17	0.943	-2.33	10	-0.51	0.306	99	0.903	0.694	229	0.945	-1.91	10	-0.42	0.336	103	0.912	0.664	225	0.933	2.73	10	0.6	0.726	249	0.943	0.274	79	0.944	0.43	10	0.02	0.509	167	0.923	0.491	161	0.913	3.16	10	0.61	0.729	248	0.958	0.271	80	0.935
**RPS6KB1**	-8.37	10	-1.92	0.028	9	0.916	0.972	319	0.993	-4.56	10	-0.99	0.16	44	0.903	0.84	284	0.945	-3.02	10	-0.64	0.26	70	0.912	0.74	258	0.933	5.5	10	1.25	0.894	293	0.986	0.106	35	0.944	-0.51	10	-0.2	0.42	127	0.923	0.58	201	0.913	-1.04	10	-0.35	0.363	117	0.958	0.637	211	0.948
**RRM1**	3.5	10	0.63	0.737	260	0.916	0.263	68	0.943	1.81	10	0.4	0.654	233	0.903	0.346	95	0.945	1.45	10	0.24	0.595	208	0.912	0.405	120	0.933	2.1	10	0.45	0.675	234	0.932	0.325	94	0.944	5.42	10	1.21	0.886	295	0.976	0.114	33	0.913	-0.27	10	-0.17	0.431	136	0.958	0.569	192	0.948
**RS1**	0.05	10	-0.11	0.458	149	0.916	0.542	179	0.943	1.86	10	0.41	0.657	235	0.903	0.343	93	0.945	-9.16	10	-1.86	0.0317	11	0.912	0.968	317	0.998	0.22	10	0.01	0.504	169	0.927	0.496	159	0.944	-2.53	10	-0.68	0.249	64	0.923	0.751	264	0.924	4.91	10	1.01	0.844	285	0.969	0.156	43	0.935
**RXFP3**	1.05	10	0.11	0.543	181	0.916	0.457	147	0.943	-0.33	10	-0.07	0.471	166	0.903	0.529	162	0.945	2.5	10	0.45	0.674	238	0.914	0.326	90	0.933	-1.07	10	-0.29	0.384	124	0.927	0.616	204	0.944	2.76	10	0.57	0.717	254	0.923	0.283	74	0.913	3.06	10	0.59	0.722	245	0.958	0.278	83	0.935
**RXRA**	5.74	10	1.12	0.868	295	0.955	0.132	33	0.943	8.43	10	1.84	0.967	311	1	0.0329	17	0.633	1.48	10	0.25	0.597	210	0.912	0.403	118	0.933	-3.23	10	-0.8	0.211	60	0.927	0.789	268	0.944	0.35	10	0	0.501	161	0.923	0.499	167	0.913	1.46	10	0.22	0.588	185	0.958	0.412	143	0.943
**RXRB**	-5.12	10	-1.22	0.112	30	0.916	0.888	298	0.974	0.24	10	0.05	0.521	183	0.903	0.479	145	0.945	5.58	10	1.06	0.855	284	0.971	0.145	44	0.933	6	10	1.37	0.914	300	0.986	0.0856	28	0.944	-3.84	10	-0.99	0.161	38	0.923	0.839	290	0.945	4.84	10	0.99	0.84	282	0.969	0.16	46	0.935
**SAMD4B**	2.41	10	0.4	0.656	225	0.916	0.344	103	0.943	-0.83	10	-0.18	0.428	150	0.903	0.572	178	0.945	5.77	10	1.1	0.863	289	0.971	0.137	39	0.933	8.18	10	1.88	0.97	316	1	0.03	12	0.817	4.1	10	0.89	0.814	279	0.954	0.186	49	0.913	6.57	10	1.39	0.918	307	0.976	0.082	21	0.935
**SERPINA7**	2.22	10	0.36	0.641	219	0.916	0.359	109	0.943	-1.93	10	-0.42	0.337	113	0.903	0.663	215	0.945	4.16	10	0.78	0.781	274	0.933	0.219	54	0.933	-2.51	10	-0.63	0.264	77	0.927	0.736	251	0.944	5.35	10	1.19	0.883	293	0.976	0.117	35	0.913	-1.28	10	-0.41	0.342	109	0.958	0.658	219	0.948
**SERPINB1**	-1.17	10	-0.37	0.356	108	0.916	0.644	220	0.943	-4.42	10	-0.96	0.168	48	0.903	0.832	280	0.945	4.11	10	0.77	0.779	273	0.933	0.221	55	0.933	0.57	10	0.09	0.537	179	0.927	0.463	149	0.944	-2.21	10	-0.6	0.273	69	0.923	0.727	259	0.917	-5.55	10	-1.38	0.0833	28	0.94	0.917	300	0.993
**SF3B3**	8.19	10	1.64	0.95	313	0.991	0.05	15	0.943	1.03	10	0.23	0.589	208	0.903	0.411	120	0.945	4.38	10	0.82	0.794	275	0.943	0.206	53	0.933	-3.53	10	-0.87	0.192	50	0.927	0.808	278	0.944	0.59	10	0.06	0.524	171	0.923	0.476	157	0.913	-6.93	10	-1.7	0.0447	17	0.85	0.955	311	0.993
**SGK3**	3.08	10	0.54	0.707	248	0.916	0.293	80	0.943	0.83	10	0.18	0.572	202	0.903	0.428	126	0.945	2.75	10	0.5	0.691	244	0.914	0.309	84	0.933	-0.21	10	-0.09	0.463	156	0.927	0.537	172	0.944	6.01	10	1.34	0.911	305	0.976	0.0893	23	0.913	0.66	10	0.04	0.515	165	0.958	0.485	163	0.948
**SLC13A1**	1.01	10	0.1	0.54	180	0.916	0.46	148	0.943	-0.94	10	-0.2	0.419	144	0.903	0.581	184	0.945	6.07	10	1.15	0.876	294	0.971	0.124	34	0.933	2.83	10	0.62	0.734	253	0.943	0.266	75	0.944	-1.01	10	-0.32	0.375	108	0.923	0.625	220	0.913	2.87	10	0.55	0.707	235	0.958	0.293	93	0.935
**SLC36A3**	2.1	10	0.33	0.631	214	0.916	0.369	114	0.943	1.48	10	0.32	0.627	225	0.903	0.373	103	0.945	-5.36	10	-1.1	0.135	32	0.912	0.865	296	0.956	-1.74	10	-0.45	0.326	106	0.927	0.674	222	0.944	-2.77	10	-0.74	0.231	59	0.923	0.769	269	0.924	6.63	10	1.41	0.92	308	0.976	0.08	20	0.935
**SMO**	-4.4	10	-1.06	0.144	38	0.916	0.856	290	0.962	2.36	10	0.52	0.697	251	0.903	0.303	77	0.945	-0.43	10	-0.13	0.448	151	0.912	0.552	177	0.933	1.72	10	0.36	0.642	223	0.932	0.358	105	0.944	-3.09	10	-0.81	0.208	48	0.923	0.792	280	0.925	-1.26	10	-0.4	0.344	111	0.958	0.656	217	0.948
**SPC24**	0.97	10	0.09	0.537	176	0.916	0.463	152	0.943	3.26	10	0.71	0.762	265	0.94	0.238	63	0.945	1.72	10	0.29	0.616	220	0.912	0.384	108	0.933	1.52	10	0.31	0.623	219	0.927	0.377	109	0.944	-3.79	10	-0.98	0.164	40	0.923	0.836	288	0.945	3.82	10	0.76	0.777	263	0.964	0.223	65	0.935
**SPEM1**	5.07	10	0.97	0.834	287	0.951	0.166	41	0.943	8.04	10	1.75	0.96	309	1	0.0397	19	0.65	-0.69	10	-0.18	0.428	142	0.912	0.572	186	0.933	-0.15	10	-0.08	0.469	157	0.927	0.531	171	0.944	-4.08	10	-1.05	0.147	35	0.923	0.853	293	0.951	-2.73	10	-0.74	0.23	65	0.958	0.77	263	0.948
**SRC**	3.62	10	0.66	0.745	263	0.916	0.255	65	0.943	2.89	10	0.63	0.736	263	0.915	0.264	65	0.945	0.89	10	0.13	0.552	194	0.912	0.448	134	0.933	1.37	10	0.28	0.61	211	0.927	0.39	117	0.944	-1.37	10	-0.4	0.343	96	0.923	0.657	232	0.913	-2.77	10	-0.75	0.228	63	0.958	0.772	265	0.948
**SRSF2**	-0.6	10	-0.25	0.403	127	0.916	0.597	201	0.943	14.89	10	3.25	0.999	324	1	0.0006	4	0.0471	15.58	10	3.04	0.999	325	1	0.0012	3	0.131	-1.84	10	-0.48	0.317	101	0.927	0.683	227	0.944	-0.28	10	-0.15	0.442	136	0.923	0.558	192	0.913	0.57	10	0.02	0.507	160	0.958	0.493	168	0.948
**STAT3**	1.14	10	0.13	0.551	185	0.916	0.449	143	0.943	-0.49	10	-0.11	0.457	160	0.903	0.543	168	0.945	-0.5	10	-0.14	0.443	148	0.912	0.557	180	0.933	-1.22	10	-0.33	0.371	121	0.927	0.629	207	0.944	-5.64	10	-1.42	0.0784	18	0.923	0.922	310	0.972	-2.09	10	-0.59	0.277	87	0.958	0.723	241	0.948
**SYK**	-6.21	10	-1.45	0.074	18	0.916	0.927	310	0.976	0.29	10	0.06	0.526	185	0.903	0.474	143	0.945	-4.18	10	-0.87	0.192	49	0.912	0.808	279	0.945	1.68	10	0.35	0.638	222	0.932	0.362	106	0.944	2.1	10	0.42	0.662	225	0.923	0.338	103	0.913	0.35	10	-0.03	0.487	153	0.958	0.513	175	0.948
**T**	3.3	10	0.59	0.723	258	0.916	0.277	70	0.943	-0.68	10	-0.15	0.442	153	0.903	0.558	175	0.945	-3.45	10	-0.73	0.233	61	0.912	0.767	267	0.933	-0.14	10	-0.07	0.47	158	0.927	0.53	170	0.944	0.39	10	0.01	0.505	165	0.923	0.495	163	0.913	0.88	10	0.09	0.536	170	0.958	0.464	158	0.948
**TBK1**	-0.08	10	-0.14	0.446	145	0.916	0.554	183	0.943	-4.76	10	-1.04	0.149	39	0.903	0.851	289	0.945	-11.04	10	-2.23	0.013	6	0.642	0.987	322	0.999	-13.63	10	-3.25	0.0006	1	0.191	0.999	327	0.999	2.18	10	0.44	0.669	229	0.923	0.331	99	0.913	1.89	10	0.32	0.625	198	0.958	0.375	130	0.935
**TEC**	-10.15	10	-2.3	0.011	5	0.706	0.989	323	0.999	-2.6	10	-0.57	0.285	88	0.903	0.715	240	0.945	-7.08	10	-1.45	0.0742	16	0.912	0.926	312	0.968	-1.99	10	-0.51	0.305	98	0.927	0.695	230	0.944	-1.97	10	-0.55	0.292	77	0.923	0.708	251	0.914	-3.98	10	-1.03	0.153	43	0.958	0.847	285	0.972
**TEK**	5.39	10	1.04	0.851	290	0.955	0.149	38	0.943	-4.77	10	-1.04	0.149	38	0.903	0.851	290	0.945	2.68	10	0.48	0.686	242	0.914	0.314	86	0.933	-2.76	10	-0.69	0.245	68	0.927	0.755	260	0.944	1.29	10	0.23	0.589	201	0.923	0.411	127	0.913	1.56	10	0.25	0.597	188	0.958	0.403	140	0.935
**TKTL2**	4.42	10	0.83	0.797	280	0.931	0.203	48	0.943	-3.89	10	-0.85	0.198	61	0.903	0.802	267	0.945	-1.19	10	-0.28	0.39	118	0.912	0.61	210	0.933	4.42	10	1	0.841	284	0.965	0.159	44	0.944	3.85	10	0.83	0.798	272	0.954	0.202	56	0.913	1.1	10	0.14	0.555	176	0.958	0.445	152	0.948
**TNF**	-6.42	10	-1.5	0.067	16	0.916	0.933	312	0.977	-6.62	10	-1.44	0.0744	13	0.903	0.926	315	0.961	0.96	10	0.14	0.557	196	0.912	0.443	132	0.933	-3.05	10	-0.76	0.224	64	0.927	0.776	264	0.944	-1.61	10	-0.46	0.322	88	0.923	0.678	240	0.913	2.46	10	0.45	0.674	217	0.958	0.326	111	0.935
**TNFRSF14**	-2.51	10	-0.66	0.256	77	0.916	0.744	251	0.943	0.21	10	0.05	0.518	182	0.903	0.482	146	0.945	-3.16	10	-0.67	0.251	67	0.912	0.749	261	0.933	0.83	10	0.15	0.561	190	0.927	0.439	138	0.944	1.7	10	0.32	0.627	216	0.923	0.373	112	0.913	4.65	10	0.95	0.83	280	0.969	0.17	48	0.935
**TNFRSF4**	-2.69	10	-0.69	0.244	68	0.916	0.756	260	0.943	-2.65	10	-0.58	0.282	87	0.903	0.718	241	0.945	0.47	10	0.05	0.519	177	0.912	0.481	151	0.933	3.69	10	0.82	0.795	275	0.943	0.205	53	0.944	-3.47	10	-0.9	0.183	42	0.923	0.817	286	0.932	0.36	10	-0.03	0.487	154	0.958	0.513	174	0.948
**TNFRSF8**	-11.8	10	-2.65	0.004	2	0.655	0.996	326	0.999	-4.94	10	-1.08	0.14	32	0.903	0.86	296	0.945	3.03	10	0.55	0.71	254	0.914	0.29	74	0.933	1.32	10	0.27	0.605	208	0.927	0.395	120	0.944	0.59	10	0.06	0.525	172	0.923	0.475	156	0.913	-2.44	10	-0.67	0.251	75	0.958	0.749	253	0.948
**TNFRSF9**	0.2	10	-0.07	0.47	153	0.916	0.53	175	0.943	1.17	10	0.26	0.601	214	0.903	0.399	114	0.945	-1.23	10	-0.29	0.386	117	0.912	0.614	211	0.933	1.17	10	0.23	0.592	204	0.927	0.408	124	0.944	-5.1	10	-1.29	0.0989	22	0.923	0.901	306	0.962	-3.31	10	-0.87	0.192	52	0.958	0.808	276	0.957
**TNFSF11**	-0.44	10	-0.21	0.416	132	0.916	0.584	196	0.943	-1.25	10	-0.27	0.393	133	0.903	0.607	195	0.945	-1.95	10	-0.43	0.333	101	0.912	0.667	227	0.933	-2.88	10	-0.72	0.236	67	0.927	0.764	261	0.944	1.92	10	0.38	0.646	221	0.923	0.354	107	0.913	-0.48	10	-0.22	0.412	127	0.958	0.588	201	0.948
**TNFSF13B**	-1.14	10	-0.36	0.358	110	0.916	0.642	218	0.943	4.79	10	1.05	0.852	283	0.983	0.148	45	0.945	-0.97	10	-0.24	0.406	130	0.912	0.594	198	0.933	-3.4	10	-0.84	0.2	54	0.927	0.8	274	0.944	0.07	10	-0.06	0.475	151	0.923	0.525	177	0.913	3.91	10	0.78	0.783	265	0.966	0.217	63	0.935
**TNFSF4**	3.79	10	0.7	0.757	268	0.916	0.243	60	0.943	4.62	10	1.01	0.843	281	0.981	0.157	47	0.945	-5.08	10	-1.05	0.147	37	0.912	0.853	291	0.956	1.25	10	0.25	0.599	206	0.927	0.401	122	0.944	1.41	10	0.26	0.601	208	0.923	0.399	120	0.913	1.99	10	0.34	0.635	201	0.958	0.365	127	0.935
**TNFSF9**	-3.66	10	-0.9	0.183	45	0.916	0.817	283	0.943	-4	10	-0.87	0.191	56	0.903	0.809	272	0.945	3.18	10	0.58	0.72	255	0.918	0.28	73	0.933	4.51	10	1.02	0.846	286	0.967	0.154	42	0.944	-2.14	10	-0.59	0.279	70	0.923	0.721	258	0.914	-0.41	10	-0.21	0.418	130	0.958	0.582	198	0.948
**TOMM34**	2.2	10	0.36	0.639	217	0.916	0.361	111	0.943	0.06	10	0.01	0.505	179	0.903	0.495	149	0.945	-2.94	10	-0.63	0.266	71	0.912	0.734	257	0.933	2.21	10	0.48	0.683	237	0.932	0.317	91	0.944	-0.95	10	-0.31	0.38	112	0.923	0.62	216	0.913	0.99	10	0.11	0.545	173	0.958	0.455	155	0.948
**TONSL**	2.66	10	0.46	0.675	234	0.916	0.325	94	0.943	-1.02	10	-0.22	0.412	137	0.903	0.588	191	0.945	6.66	10	1.27	0.898	301	0.974	0.102	27	0.933	-1.53	10	-0.4	0.343	113	0.927	0.657	215	0.944	-0.27	10	-0.14	0.443	137	0.923	0.557	191	0.913	-1.71	10	-0.51	0.307	98	0.958	0.693	230	0.948
**TOP1**	5.77	10	1.12	0.869	296	0.955	0.131	32	0.943	9.12	10	1.99	0.977	316	1	0.0232	12	0.619	-0.86	10	-0.21	0.415	135	0.912	0.585	193	0.933	3.54	10	0.79	0.785	270	0.943	0.215	58	0.944	6.49	10	1.46	0.928	308	0.978	0.0723	20	0.913	-6.22	10	-1.54	0.0622	21	0.94	0.938	307	0.993
**TOP2A**	3.2	10	0.57	0.716	252	0.916	0.284	76	0.943	-1.77	10	-0.39	0.35	121	0.903	0.65	207	0.945	4	10	0.74	0.772	269	0.933	0.228	59	0.933	2.33	10	0.51	0.693	242	0.935	0.307	86	0.944	2.62	10	0.54	0.706	248	0.923	0.294	80	0.913	-2.39	10	-0.66	0.254	77	0.958	0.746	251	0.948
**TOP2B**	0.64	10	0.02	0.509	168	0.916	0.491	160	0.943	1.35	10	0.3	0.616	222	0.903	0.384	106	0.945	1.45	10	0.24	0.596	209	0.912	0.404	119	0.933	-2.76	10	-0.69	0.245	69	0.927	0.755	259	0.944	-2.84	10	-0.75	0.226	56	0.923	0.774	272	0.924	5.53	10	1.15	0.876	294	0.973	0.124	34	0.935
**TPH2**	2.29	10	0.37	0.646	222	0.916	0.354	106	0.943	1.4	10	0.31	0.62	224	0.903	0.38	104	0.945	-0.51	10	-0.15	0.442	147	0.912	0.558	181	0.933	-6.67	10	-1.61	0.0536	15	0.927	0.946	313	0.98	2.32	10	0.47	0.682	234	0.923	0.318	94	0.913	-9.71	10	-2.34	0.0098	5	0.579	0.99	323	1
**TRIM24**	-5.9	10	-1.38	0.083	22	0.916	0.917	306	0.974	-4.86	10	-1.06	0.145	35	0.903	0.855	293	0.945	1.59	10	0.27	0.606	215	0.912	0.394	113	0.933	-1.71	10	-0.44	0.328	109	0.927	0.672	219	0.944	0.69	10	0.08	0.533	175	0.923	0.467	153	0.913	4.01	10	0.81	0.79	266	0.966	0.21	62	0.935
**TRPC7**	1.4	10	0.18	0.573	192	0.916	0.427	136	0.943	-3.69	10	-0.81	0.21	68	0.903	0.79	260	0.945	-1.78	10	-0.4	0.345	106	0.912	0.655	222	0.933	-4.1	10	-1.01	0.157	41	0.927	0.843	287	0.954	3.27	10	0.7	0.757	263	0.937	0.243	65	0.913	-0.06	10	-0.13	0.449	142	0.958	0.551	186	0.948
**TRPV1**	2.23	10	0.36	0.641	220	0.916	0.359	108	0.943	2.36	10	0.52	0.697	249	0.903	0.303	79	0.945	-2.06	10	-0.45	0.325	99	0.912	0.675	229	0.933	0.29	10	0.03	0.51	173	0.927	0.49	155	0.944	-0.73	10	-0.25	0.4	116	0.923	0.6	212	0.913	0.84	10	0.08	0.531	168	0.958	0.469	160	0.948
**TSSK1B**	-2.67	10	-0.69	0.245	69	0.916	0.755	259	0.943	-0.01	10	0	0.499	176	0.903	0.501	152	0.945	-5.95	10	-1.22	0.111	26	0.912	0.889	302	0.963	-2.27	10	-0.58	0.282	86	0.927	0.718	242	0.944	2.65	10	0.55	0.709	249	0.923	0.291	79	0.913	2.53	10	0.47	0.68	224	0.958	0.32	104	0.935
**TUBB**	3.83	10	0.71	0.76	270	0.916	0.24	58	0.943	3.58	10	0.78	0.783	268	0.948	0.217	60	0.945	6.69	10	1.28	0.899	302	0.974	0.101	26	0.933	5.01	10	1.14	0.872	289	0.986	0.128	39	0.944	1.26	10	0.22	0.586	198	0.923	0.414	130	0.913	6.19	10	1.31	0.904	303	0.973	0.0959	25	0.935
**TUBGCP2**	-1.43	10	-0.43	0.335	104	0.916	0.665	224	0.943	0.3	10	0.07	0.526	186	0.903	0.474	142	0.945	-4.54	10	-0.94	0.173	42	0.912	0.827	286	0.946	3.32	10	0.74	0.77	261	0.943	0.23	67	0.944	0.98	10	0.15	0.561	188	0.923	0.439	140	0.913	-2.36	10	-0.65	0.257	79	0.958	0.743	249	0.948
**TXN**	0.6	2	0.17	0.568	191	0.916	0.432	137	0.943	0.62	2	0.3	0.62	223	0.903	0.38	105	0.945	1.06	2	0.42	0.663	235	0.914	0.337	93	0.933	2.69	2	1.37	0.915	301	0.986	0.0851	27	0.944	-0.03	2	-0.09	0.463	144	0.923	0.537	184	0.913	-5.39	2	-2.87	0.002	3	0.22	0.998	325	1
**TYMS**	6.75	10	1.33	0.909	307	0.968	0.091	21	0.943	5.9	10	1.29	0.901	297	0.992	0.0989	31	0.945	14.03	10	2.73	0.997	323	1	0.0032	5	0.209	12.26	10	2.84	0.998	324	1	0.0023	4	0.185	-2.66	10	-0.71	0.239	62	0.923	0.761	266	0.924	-5.5	10	-1.37	0.085	29	0.94	0.915	299	0.993
**TYRO3**	-1.53	10	-0.44	0.328	101	0.916	0.672	227	0.943	-0.45	10	-0.1	0.461	162	0.903	0.539	166	0.945	-6.97	10	-1.42	0.0775	17	0.912	0.923	311	0.968	-5.48	10	-1.33	0.0916	25	0.927	0.908	303	0.979	2.2	10	0.44	0.671	230	0.923	0.329	98	0.913	-0.41	10	-0.21	0.418	131	0.958	0.582	197	0.948
**UBQLN3**	4.93	9	1	0.841	288	0.955	0.159	40	0.943	-4.91	10	-1.07	0.142	34	0.903	0.858	294	0.945	-7.97	9	-1.71	0.0441	13	0.912	0.956	315	0.992	-6.34	9	-1.61	0.0534	14	0.927	0.947	314	0.98	-5.15	10	-1.3	0.0968	21	0.923	0.903	307	0.962	-4.04	9	-1.09	0.138	40	0.958	0.862	288	0.976
**UTS2R**	-0.34	10	-0.19	0.424	137	0.916	0.576	191	0.943	-0.99	10	-0.22	0.414	139	0.903	0.586	189	0.945	-4.93	10	-1.02	0.154	40	0.912	0.846	288	0.956	-1.07	10	-0.29	0.384	123	0.927	0.616	205	0.944	-0.57	10	-0.21	0.415	121	0.923	0.585	207	0.913	1.07	10	0.13	0.552	175	0.958	0.448	153	0.948
**VCP**	8.03	10	1.61	0.946	312	0.991	0.054	16	0.943	0.33	10	0.07	0.529	187	0.903	0.471	141	0.945	-2.33	10	-0.51	0.306	89	0.912	0.694	239	0.933	-7.07	10	-1.7	0.0442	9	0.927	0.956	319	0.98	2.6	10	0.54	0.705	247	0.923	0.295	81	0.913	-7.2	10	-1.76	0.0391	14	0.85	0.961	314	0.993
**VEGFA**	-5.82	10	-1.37	0.086	23	0.916	0.914	305	0.974	-2.84	10	-0.62	0.268	85	0.903	0.732	243	0.945	5.06	10	0.95	0.83	280	0.967	0.17	48	0.933	3.7	10	0.83	0.796	276	0.943	0.204	52	0.944	-0.16	10	-0.12	0.454	140	0.923	0.546	188	0.913	-2.05	10	-0.58	0.28	88	0.958	0.72	240	0.948
**VTCN1**	-5.8	10	-1.36	0.086	25	0.916	0.914	303	0.974	-0.24	10	-0.05	0.48	168	0.903	0.52	160	0.945	0.57	10	0.07	0.527	182	0.912	0.473	146	0.933	-1.49	10	-0.39	0.347	114	0.927	0.653	214	0.944	-0.3	10	-0.15	0.44	134	0.923	0.56	194	0.913	0.9	10	0.09	0.537	171	0.958	0.463	157	0.948
**WDR3**	-1.43	10	-0.43	0.335	105	0.916	0.665	223	0.943	-1.74	10	-0.38	0.352	122	0.903	0.648	206	0.945	6.61	10	1.26	0.896	299	0.974	0.104	29	0.933	3.51	10	0.78	0.783	267	0.943	0.217	61	0.944	2.94	10	0.62	0.732	257	0.929	0.268	71	0.913	2.33	10	0.42	0.663	211	0.958	0.337	117	0.935
**WDR43**	-0.67	10	-0.26	0.397	123	0.916	0.603	205	0.943	-2.86	10	-0.62	0.267	83	0.903	0.733	245	0.945	11.25	10	2.18	0.985	318	1	0.0147	10	0.48	1.04	10	0.2	0.58	198	0.927	0.42	130	0.944	5.65	10	1.26	0.896	299	0.976	0.104	29	0.913	-0.39	10	-0.2	0.42	132	0.958	0.58	196	0.948
**WEE1**	1.84	10	0.28	0.61	206	0.916	0.39	122	0.943	1.34	10	0.29	0.616	219	0.903	0.384	109	0.945	-6.24	10	-1.28	0.101	25	0.912	0.899	303	0.965	3.64	10	0.81	0.792	274	0.943	0.208	54	0.944	0.02	10	-0.07	0.471	149	0.923	0.529	179	0.913	9.83	10	2.14	0.984	320	0.996	0.0162	8	0.663
**XPO1**	18.9	10	3.94	1	326	1	#####	2	0.007	6.22	10	1.36	0.913	299	0.997	0.0872	29	0.945	2.77	10	0.5	0.692	246	0.914	0.308	82	0.933	6.08	10	1.39	0.917	303	0.986	0.0828	25	0.944	3.83	10	0.83	0.797	270	0.954	0.203	58	0.913	6.44	10	1.36	0.913	305	0.976	0.0865	23	0.935

High confidence hits (P-value ≤ 0.05 in either early or late time points for one cell line or P-value ≤ 0.01 at either early or late timepoint for 2 cell lines)

A549		H1299		H2023	
**Early (Day 12)**	**Late (Day 18)**	**Early (Day 12)**	**Late (Day 18)**	**Early (Day 15)**	**Late (Day 25)**
AR	BTLA	AR	BRAF	BCL2	FYN
CA9	CD274	BCL2	CDK4	BTLA	PGD
CD274	LCK	BRAF	GLS	CA9	PPP3R2
CDK4	PGD	CA9	LCK	CD274	VCP
EPHA2	ROCK1	CDK4	MS4A1	CXCL1	
FYN		CXCL1	PPP3R2	EPHA2	
NDST4		EPHA2	ROCK1	FGF4	
PGD		FGF4	ROCK2	FYN	
PPP3R2		FYN	TBK1	MS4A1	
ROCK1		GLS	TPH2	NDST4	
TEC		MS4A1	VCP	PGD	
		NDST4		TPH2	
		PGD		UBQLN3	
		PPP3R2			
		ROCK2			
		TBK1			
		TEC			
		UBQLN3			

		Metabolite abundance																								
**Metabolite**	**Method**	**DMSO 0h 1**	**DMSO 0h 2**	**DMSO 0h 3**	**DMSO 24h 1**	**DMSO 24h 2**	**DMSO 24h 3**	**DMSO 48h 1**	**DMSO 48h 2**	**DMSO 48h 3**	**KD025 24h 1**	**KD025 24h 2**	**KD025 24h 3**	**KD025 48h 1**	**KD025 48h 2**	**KD025 48h 3**	**OXPHOS 24h**	**OXPHOS 24h**	**OXPHOS 24h**	**OXPHOS 48h**	**OXPHOS 48h**	**OXPHOS 48h**	**Combo**	**Combo**	**Combo**	**Combo**	**Combo**	**Combo 48h 3**
(2R)-2-Hydroxy-3-(phosphonatooxy)propanoate	HILIC	4.388901e-02	4.795234e-02	5.217186e-02	4.512890e-02	4.437392e-02	3.800034e-02	5.485923e-02	5.747767e-02	3.781530e-02	3.812795e-02	4.780915e-02	4.314875e-02	4.482995e-02	4.483548e-02	4.654346e-02	3.795125e-02	4.853251e-02	3.018891e-02	2.129954e-02	2.848852e-02	2.963688e-02	3.70302	2.76080	2.62345	2.46018	2.29144	2.617495e-02
1-Pyrroline-2-carboxylic acid	HILIC	9.072932e-05	8.875973e-05	9.422309e-05	7.138778e-05	7.116045e-05	6.721553e-05	6.397417e-05	6.532372e-05	5.352241e-05	7.787024e-05	8.523694e-05	7.102449e-05	6.175914e-05	6.059821e-05	6.329795e-05	5.379754e-05	4.576922e-05	4.843422e-05	3.071068e-05	3.201672e-05	3.795393e-05	5.51186	4.87814	5.41804	3.62271	3.27329	3.255019e-05
2'-Deoxyguanosine 5'-monophosphate	HILIC	5.189971e-03	3.512378e-03	3.760765e-03	3.778769e-03	3.816289e-03	4.729207e-03	5.779016e-03	5.125279e-03	3.547276e-03	4.275076e-03	5.139398e-03	4.871686e-03	5.064854e-03	4.533909e-03	4.091831e-03	6.352102e-03	5.717030e-03	8.576276e-03	5.631893e-03	5.164410e-03	6.372419e-03	7.28733	8.13221	8.14699	7.49626	7.59631	5.822638e-03
2,3-Diphosphoglyceric acid	HILIC	7.988024e-04	3.633074e-04	4.749338e-04	2.197258e-04	1.833555e-04	1.875686e-04	2.038711e-04	2.425939e-04	8.392951e-05	1.860328e-04	2.373046e-04	1.609644e-04	1.483740e-04	9.842493e-05	9.181434e-05	1.382121e-04	1.764010e-04	1.578359e-04	3.026533e-04	2.319919e-05	2.740614e-05	4.34656	6.85222	3.86148	2.69461	2.13735	3.228619e-05
2-Aminomuconic acid	HILIC	4.867162e-04	5.707651e-04	5.933627e-04	4.723447e-04	4.611408e-04	4.339868e-04	4.247152e-04	4.203446e-04	3.233439e-04	4.891734e-04	5.320772e-04	4.657322e-04	4.119783e-04	4.175765e-04	4.414444e-04	3.216666e-04	3.028318e-04	3.372083e-04	1.720087e-04	1.940532e-04	2.324144e-04	3.75989	3.29449	3.56789	2.27342	2.00296	2.211721e-04
2-Aminomuconic acid semialdehyde	HILIC	2.765234e-04	1.878943e-04	1.961622e-04	1.428525e-04	1.516501e-04	1.365150e-04	1.891404e-04	1.942816e-04	5.723063e-04	1.554593e-04	1.658610e-04	1.696782e-04	1.853331e-04	2.138409e-04	1.696809e-04	1.594242e-04	1.623756e-04	1.517245e-04	3.645573e-04	2.151803e-04	1.789071e-04	2.21869	1.63485	1.82604	1.60859	1.57892	5.051083e-04
2-Hydroxybutyric acid	HILIC	1.885970e-04	1.009154e-04	9.153247e-05	8.280627e-05	7.848572e-05	8.140122e-05	8.472192e-05	7.765674e-05	2.574216e-04	9.825159e-05	7.025775e-05	7.927161e-05	7.815742e-05	9.392829e-05	7.488846e-05	9.699595e-05	9.870536e-05	8.866851e-05	1.792238e-04	1.093873e-04	1.004835e-04	9.14278	9.24188	8.83857	8.37424	9.10771	8.415856e-05
2-Hydroxyglutarate	HILIC	4.505759e-04	4.819086e-04	4.368142e-04	4.351602e-04	4.318169e-04	4.418375e-04	3.965823e-04	3.850087e-04	3.616421e-04	4.131625e-04	4.321609e-04	4.017477e-04	3.022289e-04	2.855466e-04	3.272581e-04	8.678203e-04	5.480974e-04	5.213963e-04	1.752375e-04	2.866795e-04	1.704269e-04	4.41022	3.68949	3.92799	3.98207	3.48555	3.688676e-04
2-Methylglutaric acid	HILIC	3.196605e-04	1.411083e-04	1.156979e-04	9.170391e-05	7.442574e-05	9.602925e-05	5.251738e-05	4.994881e-05	1.181125e-04	1.183759e-04	5.133396e-05	8.231920e-05	1.236437e-04	2.495600e-04	1.601621e-04	3.401966e-04	7.089633e-05	8.792176e-05	2.007873e-04	3.549729e-04	1.895525e-04	9.27315	1.31405	9.34286	2.07126	1.54983	1.867691e-04
2-Phospho-D-glyceric acid	HILIC	4.291382e-02	4.699754e-02	5.100719e-02	4.413947e-02	4.340759e-02	3.716562e-02	5.368010e-02	5.627597e-02	3.699498e-02	3.730186e-02	4.676034e-02	4.219894e-02	4.364833e-02	4.372501e-02	4.533988e-02	3.711661e-02	4.745469e-02	2.939500e-02	2.072225e-02	2.772805e-02	2.882423e-02	3.62334	2.70308	2.57302	2.38874	2.23033	2.547671e-02
2-Propylglutaric acid	HILIC	3.600791e-04	9.565309e-05	1.030781e-04	9.528766e-05	5.690587e-05	1.259730e-04	4.480437e-05	5.625760e-05	6.951606e-05	9.436217e-05	5.255073e-05	5.920315e-05	7.874416e-05	1.070301e-04	5.983383e-05	1.713700e-04	5.718130e-05	1.340003e-04	1.592429e-04	2.087879e-04	8.550006e-05	8.90509	1.05016	8.48615	1.04060	7.46994	1.688061e-04
3-Dehydro-L-gulonate	HILIC	4.063601e-05	1.784237e-05	1.707558e-05	1.580822e-05	1.633376e-05	1.376571e-05	1.360885e-05	1.108772e-05	8.911554e-06	2.418332e-05	1.069345e-05	1.097084e-05	7.201987e-06	8.882873e-06	6.079198e-06	2.811667e-05	2.155730e-05	2.705918e-05	8.237167e-06	1.713293e-05	1.936854e-05	1.41789	1.56424	1.41892	1.29525	1.11143	9.059639e-06
3-Hydroxybutyric acid	HILIC	2.580965e-04	5.957537e-05	5.491526e-05	4.510945e-05	4.303356e-05	4.772327e-05	3.690506e-05	3.462919e-05	1.422679e-04	5.238618e-05	3.693961e-05	4.388465e-05	4.861566e-05	5.974369e-05	4.515139e-05	4.386530e-05	3.710063e-05	4.252331e-05	1.065419e-04	5.285360e-05	4.634289e-05	4.38499	5.15322	4.75711	4.86119	4.89984	4.907649e-05
3-Hydroxymethylglutaric acid	HILIC	2.314626e-04	1.408431e-04	1.259502e-04	1.173491e-04	1.010843e-04	1.327822e-04	1.132142e-04	1.043602e-04	1.270606e-04	1.322859e-04	1.137740e-04	1.154325e-04	1.166420e-04	1.203680e-04	1.155402e-04	3.427968e-04	1.039888e-04	1.178239e-04	1.265349e-04	1.749676e-04	1.161975e-04	1.09622	1.04063	9.99916	1.40636	9.74193	1.109167e-04
3-Sulfinoalanine	HILIC	8.085827e-05	6.786547e-05	2.389328e-05	2.713962e-05	1.672097e-05	5.164981e-05	1.460104e-05	1.389511e-05	1.812345e-05	1.421214e-05	1.387997e-05	1.397191e-05	1.508643e-05	1.095698e-04	1.313564e-05	4.701443e-05	1.375126e-05	1.505194e-05	1.328301e-05	1.177185e-05	1.505969e-05	3.27719	1.90766	2.16686	1.86223	1.45708	1.893568e-05
5'-Phosphoribosyl-N-formylglycinamide	HILIC	2.338743e-04	2.995581e-04	3.677421e-04	2.003045e-04	2.171475e-04	2.552104e-04	3.112693e-04	3.125458e-04	1.654785e-04	2.315223e-04	3.428101e-04	2.498088e-04	4.362883e-04	3.922903e-04	4.010761e-04	2.816298e-04	2.804925e-04	3.667840e-04	2.456045e-05	3.475744e-05	2.749404e-05	2.01075	1.87280	2.40049	1.50064	1.27305	1.159541e-04
5-L-Glutamyl-L-alanine	HILIC	5.504365e-04	2.138572e-04	2.872328e-04	2.786345e-04	2.964878e-04	2.579526e-04	4.760534e-04	4.105190e-04	3.871919e-04	5.473599e-04	2.607292e-04	2.606319e-04	3.399399e-04	3.601654e-04	3.600063e-04	6.553541e-04	7.388059e-04	6.713982e-04	3.974193e-04	4.950994e-04	5.416019e-04	8.44470	8.07747	8.86872	9.77941	8.51538	9.146182e-04
5-L-Glutamyl-taurine	HILIC	8.287553e-05	1.046780e-04	1.070568e-04	1.090780e-04	1.031366e-04	9.728466e-05	1.403874e-04	1.318436e-04	6.359764e-05	1.004805e-04	1.111491e-04	1.052452e-04	9.800239e-05	8.572810e-05	1.027079e-04	1.481573e-04	1.454786e-04	1.543304e-04	1.576518e-04	1.963325e-04	2.394013e-04	8.51829	7.80878	6.85277	7.32087	6.55087	6.366367e-05
5-Thymidylic acid	HILIC	4.415260e-05	2.779573e-05	4.096958e-05	1.607285e-05	1.353098e-05	2.092817e-05	1.769021e-05	1.507953e-05	1.401834e-05	2.405319e-05	2.488790e-05	2.375021e-05	3.202265e-05	2.711222e-05	2.689748e-05	8.046695e-05	2.060419e-05	1.472415e-04	2.465752e-05	2.590019e-05	2.125872e-05	2.00099	1.71796	1.91834	2.11713	1.93326	8.600340e-06
6-Phosphogluconic acid	HILIC	4.028600e-04	3.907573e-04	3.138706e-04	4.142344e-04	2.859926e-04	4.454839e-04	4.515015e-04	3.606219e-04	2.757341e-04	3.390889e-04	2.735149e-04	3.502729e-04	3.290122e-04	2.177706e-04	3.227378e-04	4.214455e-04	4.952643e-04	2.356349e-04	2.663269e-04	3.665628e-04	3.571512e-04	3.63329	4.09281	3.62935	3.47448	3.40642	1.760241e-04
ADP	HILIC	5.208569e-03	5.553968e-03	5.541395e-03	6.133494e-03	5.102160e-03	6.775318e-03	4.204409e-03	4.083614e-03	2.386372e-03	4.584610e-03	4.800204e-03	4.035706e-03	2.785132e-03	2.798157e-03	2.379560e-03	7.045640e-03	7.602841e-03	4.182674e-03	4.187345e-03	3.724459e-03	4.634204e-03	6.01604	7.32006	8.30494	5.47838	5.42072	3.940587e-03
ADP-glucose	HILIC	1.326130e-04	1.568892e-04	1.561270e-04	2.188166e-04	1.898865e-04	1.963663e-04	2.430099e-04	2.111575e-04	1.945459e-04	2.112328e-04	2.334341e-04	2.270816e-04	2.194095e-04	1.544196e-04	1.950741e-04	3.080719e-04	3.683541e-04	2.830468e-04	6.548074e-04	6.441828e-04	5.793762e-04	2.70099	2.82362	2.41570	2.52436	2.36007	2.119755e-04
ADP-ribose 2'-phosphate	HILIC	1.110708e-04	1.385158e-04	1.402246e-04	1.902082e-04	1.993521e-04	2.280114e-04	3.581013e-04	3.226251e-04	2.994362e-04	1.891240e-04	3.081718e-04	3.079043e-04	2.974601e-04	2.748353e-04	3.106690e-04	2.493365e-04	2.967796e-04	1.991501e-04	2.307019e-04	2.366255e-04	2.481821e-04	2.81377	3.07935	2.86799	2.89796	2.81829	2.495330e-04
AICAR	HILIC	3.604903e-05	3.558784e-05	3.890561e-05	8.130613e-05	4.977911e-05	6.258389e-05	1.066557e-03	4.855802e-04	5.349956e-04	5.404542e-05	5.251031e-05	4.738302e-05	2.997341e-03	1.881251e-03	2.468277e-03	5.404083e-05	5.929427e-05	4.978030e-05	9.569694e-07	3.504436e-06	3.593943e-06	2.36390	2.93491	3.09400	1.40373	1.46267	5.791407e-06
Acetylcysteine	HILIC	3.200989e-05	1.699295e-05	9.438754e-06	8.934218e-06	9.268201e-06	6.855980e-06	2.070371e-05	1.583032e-05	2.058814e-05	9.607447e-06	1.459942e-05	1.170205e-05	2.044694e-05	2.057717e-05	2.239780e-05	4.466183e-05	1.571217e-05	1.297466e-05	7.759426e-06	5.527107e-06	7.888444e-06	3.03173	2.31705	2.29829	2.08901	2.40956	1.569065e-05
Adenosine diphosphate ribose	HILIC	5.470972e-04	6.909173e-04	7.583005e-04	5.890633e-04	5.606499e-04	6.991276e-04	4.772255e-04	4.793631e-04	2.539805e-04	4.499708e-04	4.843939e-04	4.547391e-04	3.457757e-04	4.759209e-04	3.415028e-04	8.914127e-04	8.743647e-04	7.185751e-04	4.413552e-04	4.209225e-04	5.941103e-04	9.23249	1.04784	1.18894	8.76885	9.02453	1.310448e-03
Adenosine monophosphate	HILIC	5.000545e-03	3.434911e-03	3.665589e-03	3.677694e-03	3.692068e-03	4.579250e-03	5.642674e-03	4.964223e-03	3.397854e-03	4.188948e-03	5.068354e-03	4.773910e-03	4.876690e-03	4.241672e-03	3.926172e-03	6.173002e-03	5.535844e-03	8.343752e-03	5.320671e-03	4.967266e-03	6.134056e-03	7.12430	7.92065	7.92264	7.22339	7.31515	5.568947e-03
Adenosine triphosphate	HILIC	9.984208e-04	1.313801e-03	1.424643e-03	1.570730e-03	1.547194e-03	1.494855e-03	2.005983e-03	1.864004e-03	1.596228e-03	1.255393e-03	1.828590e-03	1.739526e-03	1.583939e-03	1.104830e-03	1.494862e-03	1.578934e-03	1.940520e-03	1.114985e-03	1.666299e-03	1.912585e-03	1.822849e-03	1.78375	1.74044	1.64915	1.52540	1.55615	1.223525e-03
Alpha-D-Glucose 1,6-bisphosphate	HILIC	3.246075e-03	3.354972e-03	3.463883e-03	4.255043e-03	4.018964e-03	3.967506e-03	4.445071e-03	4.005158e-03	2.607425e-03	4.070407e-03	4.134089e-03	4.349424e-03	3.250519e-03	2.461197e-03	3.343223e-03	4.027345e-03	4.524930e-03	3.008818e-03	2.033401e-03	2.830094e-03	3.236048e-03	4.01256	3.59664	3.60932	2.57528	2.48260	2.375896e-03
Arabinonic acid	HILIC	7.396273e-05	1.263726e-04	9.005385e-05	7.387108e-05	4.972675e-05	6.266740e-05	7.635511e-05	5.766853e-05	3.499759e-05	5.810469e-05	6.570734e-05	6.752718e-05	4.683670e-05	3.830745e-05	4.546036e-05	5.691823e-05	7.334108e-05	5.700640e-05	2.955215e-05	3.544648e-05	4.550885e-05	8.90551	6.00312	5.20629	3.95683	5.44628	3.414659e-05
Beta-Citryl-L-glutamic acid	HILIC	3.215418e-03	3.561429e-03	3.637430e-03	3.990623e-03	3.935193e-03	3.791767e-03	4.836013e-03	4.823901e-03	3.650903e-03	3.664443e-03	4.503827e-03	4.125252e-03	4.573439e-03	4.145815e-03	4.775717e-03	1.920967e-03	2.013385e-03	2.055574e-03	8.417285e-04	1.028981e-03	1.062166e-03	1.34229	1.14474	1.20573	5.98218	5.16407	5.514550e-04
Beta-D-Glucose 6-phosphate	HILIC	1.606916e-02	1.827098e-02	1.725322e-02	1.809241e-02	1.718094e-02	1.765045e-02	2.097642e-02	1.956809e-02	1.395383e-02	1.556320e-02	1.455936e-02	1.636727e-02	1.823486e-02	1.468471e-02	1.853283e-02	1.285416e-02	1.317984e-02	1.373283e-02	6.356418e-03	1.133603e-02	1.187116e-02	1.46866	1.14744	1.05410	1.04459	1.04354	3.166544e-03
Butyric acid	HILIC	2.664049e-05	2.560544e-05	2.309132e-05	2.074685e-05	2.244845e-05	2.018552e-05	2.444825e-05	2.360772e-05	5.280392e-05	2.333963e-05	2.234879e-05	2.227226e-05	3.570276e-05	4.150337e-05	3.184443e-05	1.928819e-05	1.853098e-05	2.394555e-05	3.506929e-05	2.965550e-05	2.662106e-05	1.55536	1.74223	1.49172	2.66421	2.51461	2.563922e-05
CDP	HILIC	1.075674e-03	1.488018e-03	2.304816e-03	6.217634e-04	7.215472e-04	4.986605e-04	5.718005e-04	8.472600e-04	3.297599e-04	5.331843e-04	1.166195e-03	7.710030e-04	4.360049e-04	8.217020e-04	4.071236e-04	3.845735e-04	2.883834e-04	4.072406e-04	2.719923e-03	3.090999e-04	2.906489e-04	8.93770	4.43306	6.61368	5.66290	4.51307	4.504389e-04
CDP-Ethanolamine	HILIC	6.503938e-05	9.653039e-05	8.957783e-05	6.781932e-05	5.938873e-05	6.526635e-05	4.333590e-05	4.567125e-05	1.671639e-05	4.962382e-05	6.949722e-05	6.360066e-05	4.062085e-05	3.168782e-05	4.539519e-05	1.314383e-04	1.421549e-04	1.234460e-04	1.162904e-04	1.345975e-04	1.390669e-04	1.10382	1.00616	1.17797	8.96774	8.92577	8.891625e-04
Citric acid	HILIC	3.505966e-02	2.034618e-02	2.207017e-02	2.888927e-02	2.606072e-02	2.980703e-02	3.217426e-02	2.986579e-02	2.653366e-02	2.414833e-02	2.639339e-02	2.611554e-02	2.754150e-02	2.535906e-02	2.778581e-02	1.124916e-02	1.167714e-02	1.062910e-02	4.708068e-03	5.398280e-03	5.845191e-03	7.16435	6.35207	5.99780	4.45575	5.02017	4.446548e-03
CoA	HILIC	6.726610e-04	4.000962e-04	3.684033e-04	3.892156e-04	3.490585e-04	3.161892e-04	2.549808e-04	2.725291e-04	2.309020e-04	1.838625e-04	2.726506e-04	2.677506e-04	2.007645e-04	1.870832e-04	1.963934e-04	2.893520e-04	2.434259e-04	1.358358e-04	1.758278e-04	1.801607e-04	1.668521e-04	2.25460	1.96775	1.86733	1.87635	1.58745	1.435228e-04
Cyclic AMP	HILIC	2.259671e-04	4.274586e-04	4.639713e-04	1.263376e-04	1.693848e-04	1.470001e-04	1.421772e-04	1.967160e-04	1.276746e-04	1.983035e-04	4.000922e-04	1.816308e-04	1.875112e-04	2.879722e-04	1.978687e-04	1.640991e-04	1.350876e-04	1.691203e-04	5.202896e-05	6.115327e-05	6.335980e-05	2.99817	2.26556	3.00013	2.33428	1.57106	1.835985e-04
Cysteic acid	HILIC	4.224219e-04	2.344859e-05	3.984647e-05	2.502176e-05	1.619167e-05	3.039157e-05	2.952172e-05	3.033589e-05	2.042339e-05	3.360710e-05	3.105759e-05	2.322886e-05	3.098582e-05	3.885041e-05	3.121194e-05	9.611031e-05	1.920211e-05	4.058714e-05	1.596275e-05	2.830628e-05	2.447971e-05	5.75627	3.20780	4.51921	3.51469	2.97497	3.304509e-05
Cytidine monophosphate	HILIC	1.267801e-03	1.396084e-03	2.184375e-03	1.001711e-03	1.168704e-03	1.138843e-03	1.139655e-03	1.279881e-03	7.729771e-04	8.572838e-04	1.319115e-03	1.088165e-03	1.179580e-03	1.644763e-03	1.130319e-03	1.094383e-03	1.017030e-03	1.399775e-03	8.733012e-04	9.133285e-04	1.125358e-03	1.59851	1.34652	1.61506	1.47163	1.50193	1.511635e-03
Cytidine monophosphate N-acetylneuraminic acid	HILIC	1.270431e-05	1.378724e-05	1.103318e-05	1.542531e-05	1.463084e-05	1.449275e-05	1.594968e-05	1.192775e-05	1.441946e-05	1.476353e-05	1.508648e-05	1.242858e-05	1.859431e-05	1.541404e-05	1.613311e-05	1.092358e-05	1.254507e-05	1.420857e-05	1.999516e-05	1.449766e-05	1.519667e-05	8.65993	7.51981	7.93711	9.29841	8.94734	9.449706e-06
Cytidine triphosphate	HILIC	1.719185e-04	3.678253e-04	5.981796e-04	1.645750e-04	1.877728e-04	1.207101e-04	3.695999e-04	5.199337e-04	2.967490e-04	1.398227e-04	4.573602e-04	4.206886e-04	2.688527e-04	3.365445e-04	2.920199e-04	1.023715e-04	1.070270e-04	1.347935e-04	1.373059e-04	2.346271e-04	1.573786e-04	3.69283	1.51841	1.48952	2.31707	2.27189	2.344620e-04
D-Erythrose 4-phosphate	HILIC	2.262227e-04	1.340772e-04	1.356573e-04	1.107186e-04	1.013387e-04	1.501295e-04	7.378232e-05	7.439603e-05	2.686336e-05	6.833521e-05	8.055238e-05	5.853981e-05	3.891301e-05	9.131119e-05	4.129264e-05	1.977130e-04	2.174657e-04	1.393377e-04	8.640442e-05	5.746411e-05	1.164149e-04	1.72924	2.28227	2.75227	1.86919	1.98662	1.079635e-04
D-Glucaric acid	HILIC	1.250568e-04	8.996170e-05	6.378104e-05	5.523466e-05	4.375440e-05	5.388131e-05	5.606307e-05	4.615710e-05	2.798773e-05	5.614621e-05	5.317578e-05	5.227375e-05	3.819330e-05	3.755571e-05	3.986162e-05	2.032425e-04	5.683015e-05	1.045372e-04	2.505173e-05	3.072430e-05	4.004029e-05	5.99374	5.20709	4.39997	3.80048	3.94697	3.331213e-05
D-Glucose	HILIC	1.660461e-02	1.123014e-02	1.212615e-02	1.156633e-02	1.278560e-02	1.174624e-02	1.556509e-02	1.755180e-02	2.160562e-02	1.833628e-02	1.674097e-02	1.573656e-02	2.274238e-02	2.628359e-02	2.352853e-02	1.207308e-02	1.287275e-02	1.285982e-02	1.469270e-02	1.540346e-02	1.472813e-02	1.98022	1.99575	2.14200	2.35306	2.15915	2.344194e-02
D-Glucuronic acid 1-phosphate	HILIC	3.459133e-04	3.402467e-04	3.092267e-04	4.024064e-04	3.802250e-04	4.053307e-04	4.475280e-04	3.965187e-04	3.087973e-04	3.000500e-04	2.785953e-04	2.779437e-04	3.579765e-04	2.919181e-04	4.194369e-04	3.235977e-04	3.842633e-04	1.565868e-04	1.527869e-04	2.237071e-04	2.154663e-04	1.42752	1.74054	1.66593	1.44574	1.29193	1.991266e-04
D-Mannose 1-phosphate	HILIC	1.989155e-04	3.153027e-04	3.073329e-04	2.858557e-04	2.759716e-04	2.801999e-04	2.581924e-04	2.705920e-04	1.663889e-04	2.905692e-04	2.662824e-04	2.826127e-04	2.494847e-04	2.345936e-04	2.597404e-04	2.662649e-04	2.934528e-04	2.518924e-04	1.346879e-04	1.562764e-04	1.443126e-04	2.99096	2.65790	2.65804	2.17812	2.02006	1.853811e-04
D-Ribose 5-phosphate	HILIC	5.028349e-03	7.577913e-03	7.533833e-03	8.296878e-03	7.960221e-03	8.264688e-03	8.414087e-03	8.068067e-03	5.842978e-03	7.832756e-03	8.428893e-03	8.237451e-03	7.413943e-03	5.730649e-03	7.339993e-03	8.643305e-03	9.657227e-03	7.638924e-03	6.953651e-03	6.998475e-03	6.522344e-03	9.91087	9.93226	9.68999	8.82414	8.47706	7.420786e-03
D-Sedoheptulose 7-phosphate	HILIC	5.206108e-04	6.571670e-04	5.781753e-04	9.382580e-04	8.509971e-04	8.305876e-04	1.178286e-03	9.343111e-04	6.796015e-04	8.178136e-04	8.784013e-04	8.506068e-04	1.016534e-03	6.996117e-04	1.126299e-03	1.055698e-03	1.459556e-03	1.002766e-03	7.455919e-04	1.232701e-03	1.238292e-03	9.25750	8.33004	8.30121	8.28923	7.07288	8.228112e-04
Deoxyadenosine monophosphate	HILIC	2.981903e-05	5.082716e-05	5.269299e-05	2.599555e-05	2.913102e-05	2.443169e-05	2.032395e-05	1.241099e-05	5.031544e-07	3.546954e-05	4.719818e-05	3.852044e-05	1.425890e-05	1.010530e-05	1.477866e-05	2.440964e-05	1.706395e-05	2.931619e-05	9.286417e-06	5.265532e-06	1.452505e-05	3.15483	1.82667	2.25972	9.27115	7.86608	2.564845e-07
Deoxyadenosine triphosphate	HILIC	7.381145e-05	9.059393e-05	9.680382e-05	5.812657e-05	5.292631e-05	6.605569e-05	2.933828e-05	3.486122e-05	1.882892e-05	4.136142e-05	6.106446e-05	5.203321e-05	2.303764e-05	2.360366e-05	2.338219e-05	7.417946e-05	6.416068e-05	4.139495e-05	5.912806e-05	5.249742e-05	6.490681e-05	6.10202	5.26408	6.38998	4.94834	5.14600	3.814867e-05
Deoxyribose 5-phosphate	HILIC	6.784036e-05	5.547347e-05	5.612456e-05	5.329450e-05	5.555887e-05	5.858502e-05	5.410106e-05	7.216626e-05	4.173114e-05	5.758313e-05	6.430668e-05	5.766118e-05	5.081765e-05	4.823127e-05	5.921218e-05	6.440035e-05	6.916669e-05	5.673554e-05	3.780219e-05	6.211961e-05	5.280249e-05	4.80320	5.20927	5.99497	5.83937	4.91137	5.528658e-05
Diadenosine triphosphate	HILIC	6.020099e-04	7.430093e-04	8.003237e-04	7.649338e-04	7.992753e-04	7.351365e-04	8.264996e-04	8.556403e-04	7.949877e-04	8.218293e-04	1.101069e-03	9.585155e-04	7.735404e-04	7.362572e-04	7.485058e-04	9.231608e-04	9.646342e-04	8.946448e-04	3.924516e-04	4.162041e-04	4.320445e-04	1.85640	1.40985	1.71636	1.24893	1.22830	1.025676e-03
Flavin Mononucleotide	HILIC	8.728961e-04	2.482859e-04	1.856229e-04	1.810362e-04	1.641417e-04	1.563774e-04	1.147721e-04	9.815714e-05	9.075734e-05	1.049898e-04	1.159146e-04	1.114716e-04	1.220253e-04	8.023734e-05	1.153409e-04	1.703607e-04	1.790373e-04	1.217813e-04	1.639711e-04	1.569843e-04	1.582241e-04	1.17391	1.31793	9.19395	1.17693	1.18231	1.079880e-04
Fructose 1,6-bisphosphate	HILIC	1.654143e-04	1.614634e-04	2.525280e-04	1.585467e-04	1.509041e-04	1.199921e-04	1.484174e-04	1.580270e-04	8.314579e-05	1.057133e-04	1.610026e-04	1.241584e-04	1.421910e-04	1.215267e-04	1.236207e-04	1.248471e-04	1.321620e-04	8.201461e-05	6.853466e-05	8.261603e-05	1.038513e-04	9.02509	8.53447	9.26118	7.82394	7.45634	7.422463e-05
Fructose 1-phosphate	HILIC	9.919212e-04	1.325031e-03	1.372238e-03	1.343990e-03	1.303646e-03	1.319653e-03	1.358581e-03	1.401825e-03	7.536003e-04	1.090563e-03	1.214406e-03	1.164463e-03	9.970630e-04	9.384317e-04	1.067407e-03	1.344796e-03	1.408344e-03	1.261922e-03	4.439491e-04	5.105973e-04	5.241378e-04	8.51611	7.67645	7.26106	6.25797	6.07415	5.181359e-04
Fructose 6-phosphate	HILIC	5.101087e-03	5.596786e-03	5.381040e-03	5.953780e-03	5.673653e-03	5.975211e-03	7.012365e-03	6.734144e-03	4.719327e-03	5.403482e-03	4.851303e-03	5.663266e-03	6.156935e-03	4.694786e-03	6.573013e-03	4.053093e-03	4.177894e-03	4.573878e-03	2.961065e-03	4.745383e-03	4.067182e-03	4.88307	3.53521	3.24129	3.83344	3.90993	1.524038e-03
Fumaric acid	HILIC	7.967602e-04	9.221163e-04	8.882689e-04	1.026798e-03	9.298718e-04	1.003400e-03	1.186112e-03	1.151030e-03	1.090244e-03	9.279580e-04	1.088488e-03	9.588908e-04	1.056701e-03	7.885702e-04	1.121981e-03	1.088811e-03	1.135575e-03	1.034611e-03	3.272071e-04	4.613795e-04	5.705991e-04	8.20013	7.47300	7.60898	7.43533	6.96463	7.169431e-04
Galactaric acid	HILIC	1.166156e-04	2.454537e-05	2.013137e-05	2.438898e-05	2.353007e-05	2.133237e-05	3.035855e-05	2.898181e-05	1.444500e-05	2.463172e-05	2.840173e-05	2.429472e-05	2.212329e-05	1.924460e-05	2.323961e-05	3.636317e-05	2.496738e-05	2.588124e-05	7.823807e-06	1.253435e-05	1.916609e-05	2.19208	2.39841	2.02303	1.59502	1.35888	1.600199e-05
Galactose 1-phosphate	HILIC	1.323338e-03	1.452127e-03	1.326467e-03	1.593251e-03	1.497553e-03	1.520792e-03	1.701530e-03	1.445536e-03	9.890976e-04	1.482722e-03	1.438506e-03	1.398889e-03	1.417502e-03	1.236245e-03	1.458368e-03	1.504314e-03	1.566313e-03	1.409585e-03	6.060748e-04	1.104960e-03	1.201456e-03	1.15380	1.15947	1.12463	9.79116	9.23706	9.153568e-04
Galacturonic acid	HILIC	5.444761e-05	4.561992e-05	4.163952e-05	3.427248e-05	3.394442e-05	2.912587e-05	4.261148e-05	4.460389e-05	3.541824e-05	4.151966e-05	3.136446e-05	3.027672e-05	3.541629e-05	5.217599e-05	3.531356e-05	6.794027e-05	4.908401e-05	1.146246e-04	4.112740e-05	3.039496e-05	2.900802e-05	2.97125	1.79893	2.19263	2.10465	2.91232	1.482151e-05
Gamma Glutamylglutamic acid	HILIC	7.183511e-04	4.392358e-04	4.004631e-04	3.548917e-04	3.474878e-04	3.632320e-04	3.818772e-04	3.094992e-04	1.704642e-04	3.931594e-04	3.898597e-04	3.918931e-04	2.756237e-04	3.398733e-04	2.848327e-04	4.358000e-04	4.314285e-04	3.306336e-04	2.593103e-04	2.936150e-04	3.092045e-04	4.99798	5.20790	4.96663	3.71540	3.74718	3.459564e-04
Glucaric acid	HILIC	1.212590e-04	8.768360e-05	6.223198e-05	5.351964e-05	4.255930e-05	5.266467e-05	5.493059e-05	4.515615e-05	2.734836e-05	5.449579e-05	5.211877e-05	5.116423e-05	3.749921e-05	3.705183e-05	3.946134e-05	1.947784e-04	5.523960e-05	1.018450e-04	2.575679e-05	2.997522e-05	3.933623e-05	5.86590	5.07830	4.30189	3.71292	3.86915	3.292575e-05
Gluconic acid	HILIC	2.139655e-03	1.302165e-03	1.365581e-03	1.273571e-03	1.188091e-03	1.210345e-03	1.242563e-03	1.177545e-03	1.141513e-03	1.491807e-03	1.183092e-03	1.181697e-03	9.564216e-04	9.596588e-04	9.811272e-04	2.116110e-03	1.604747e-03	2.572020e-03	1.281672e-03	1.265751e-03	1.378028e-03	1.35233	1.18466	1.30754	1.25955	1.17274	1.109922e-03
Glucose 1-phosphate	HILIC	4.198894e-03	4.479158e-03	4.259421e-03	3.958870e-03	3.756917e-03	3.901003e-03	4.539115e-03	4.254604e-03	3.173815e-03	3.886372e-03	3.424083e-03	3.801392e-03	3.830469e-03	3.419897e-03	3.961984e-03	2.916193e-03	2.809466e-03	3.919774e-03	2.020310e-03	2.260451e-03	2.385955e-03	2.95958	2.38432	2.21590	2.07382	2.11457	1.438411e-03
Glutaric acid	HILIC	9.218795e-05	5.135966e-05	4.252567e-05	4.128048e-05	4.360477e-05	4.356495e-05	3.145674e-05	3.721461e-05	9.150712e-05	4.400445e-05	3.108696e-05	3.594029e-05	5.282917e-05	7.408506e-05	4.909412e-05	5.075172e-05	4.133020e-05	5.222639e-05	7.156933e-05	6.878234e-05	6.685535e-05	4.13824	4.61479	4.51642	6.76641	7.21935	7.039411e-05
Glutathione	HILIC	8.115596e-02	9.441343e-02	9.759835e-02	1.052636e-01	1.113198e-01	1.037368e-01	8.056905e-02	9.145462e-02	5.940113e-02	9.727708e-02	1.163413e-01	1.068641e-01	7.070546e-02	6.648275e-02	7.099153e-02	9.459046e-02	9.943454e-02	1.012811e-01	7.724354e-02	8.189450e-02	8.724046e-02	8.48173	7.99588	8.86264	7.75078	6.95522	7.212867e-02
Glyceric acid	HILIC	1.974861e-04	2.611709e-04	2.553088e-04	2.369726e-04	2.421632e-04	2.084198e-04	2.753121e-04	3.160962e-04	4.638103e-04	2.213616e-04	2.410332e-04	2.203496e-04	3.340009e-04	3.085091e-04	3.036432e-04	1.996046e-04	2.558715e-04	1.644801e-04	3.101426e-04	2.635609e-04	2.803446e-04	1.89267	1.38763	1.32984	1.47872	1.24726	1.441430e-04
Glycerol 3-phosphate	HILIC	4.544120e-03	5.175343e-03	5.695233e-03	3.635290e-03	4.122200e-03	3.407181e-03	2.796643e-03	3.606929e-03	2.270066e-03	4.877420e-03	3.770009e-03	3.458358e-03	2.886285e-03	4.111661e-03	2.992193e-03	3.334508e-03	3.230961e-03	3.438458e-03	3.582625e-03	2.928767e-03	3.416552e-03	3.88424	3.49696	3.34271	3.78162	3.63630	3.942811e-03
Glycerophosphocholine	HILIC	1.107797e-04	6.490139e-05	6.482037e-05	8.886986e-05	8.954057e-05	9.011153e-05	7.576001e-05	7.046033e-05	6.530353e-05	1.422970e-04	9.689558e-05	1.051869e-04	8.268823e-05	8.706040e-05	9.276313e-05	7.076158e-05	8.001077e-05	7.774655e-05	1.482964e-05	4.940243e-05	5.661576e-05	3.61889	4.33083	3.77808	3.64925	3.84338	3.686678e-05
Glycerylphosphorylethanolamine	HILIC	1.018601e-02	9.001607e-03	1.296086e-02	1.388037e-02	1.601933e-02	1.635175e-02	1.471756e-02	1.537802e-02	1.270896e-02	1.609336e-02	1.581012e-02	1.897959e-02	1.994176e-02	2.010810e-02	1.515925e-02	1.704397e-02	2.080056e-02	2.569370e-02	1.579231e-02	1.866868e-02	2.494607e-02	1.72847	1.90588	1.83467	1.88219	2.44489	2.693172e-02
Guanosine diphosphate	HILIC	8.210765e-03	9.908565e-03	1.126808e-02	9.996181e-03	9.430393e-03	9.333944e-03	6.120976e-03	6.245725e-03	4.908417e-03	7.293203e-03	7.598091e-03	6.908102e-03	5.013659e-03	4.686241e-03	4.634629e-03	8.297274e-03	8.859002e-03	4.165139e-03	5.934945e-03	5.331149e-03	6.060220e-03	6.99784	6.87186	7.09899	6.96058	4.90496	4.705307e-03
Guanosine diphosphate mannose	HILIC	1.060801e-04	1.560071e-04	1.436423e-04	1.018167e-04	1.091652e-04	7.207894e-05	1.106538e-04	1.220987e-04	1.170257e-04	9.561666e-05	1.120287e-04	1.098281e-04	1.022151e-04	1.107255e-04	1.050283e-04	1.216610e-04	9.731061e-05	1.314313e-04	2.845689e-04	2.494746e-04	2.690391e-04	7.43905	4.64735	4.94534	8.45961	9.30228	5.934656e-05
Guanosine monophosphate	HILIC	2.405790e-03	7.727477e-04	1.308620e-03	1.626765e-03	2.115603e-03	2.400021e-03	3.238923e-03	3.157829e-03	2.567033e-03	1.180007e-03	1.276630e-03	2.008632e-03	3.694692e-03	4.783885e-03	3.606635e-03	3.123856e-03	2.754946e-03	6.960922e-03	3.845285e-03	3.479719e-03	3.804964e-03	2.82652	3.55025	3.57126	4.25161	4.48384	4.408612e-03
Guanosine triphosphate	HILIC	7.525757e-04	1.298214e-03	1.421511e-03	1.129507e-03	1.276840e-03	9.130858e-04	1.855858e-03	1.952562e-03	6.331568e-04	1.164593e-03	1.861893e-03	1.924905e-03	1.171253e-03	8.700305e-04	1.437154e-03	1.093319e-03	1.293001e-03	7.634491e-04	3.378500e-04	1.237333e-03	6.834812e-04	1.66947	1.41567	9.50059	1.11707	1.11396	1.119201e-03
Homocysteinesulfinic acid	HILIC	2.045891e-04	2.139182e-04	1.447841e-04	1.313715e-04	1.041422e-04	1.173417e-04	1.281551e-04	1.208161e-04	1.153965e-04	1.140416e-04	1.080994e-04	1.057713e-04	9.779300e-05	1.005262e-04	1.007460e-04	7.758558e-04	9.628959e-05	9.073076e-05	6.671300e-05	8.224643e-05	9.077052e-05	8.26850	6.62322	6.17339	5.71096	5.83837	4.689209e-05
Hydroxypropanedioic acid	HILIC	1.008811e-03	8.189073e-05	9.029514e-05	6.570934e-05	7.277802e-05	7.712719e-05	5.447993e-05	4.543123e-05	5.772579e-05	8.744753e-05	4.497229e-05	6.254551e-05	4.949228e-05	9.771294e-05	6.244279e-05	1.002050e-04	7.018594e-05	5.830534e-05	6.091984e-05	6.735451e-05	6.334810e-05	8.04722	5.83328	5.94029	8.76666	5.82276	6.949728e-05
Inosine	HILIC	1.654626e-02	1.847247e-02	1.938071e-02	2.044185e-02	2.069671e-02	1.974601e-02	2.384121e-02	2.446476e-02	2.739384e-02	1.972535e-02	2.223599e-02	2.023834e-02	2.240515e-02	2.268221e-02	2.277008e-02	2.135864e-02	2.282541e-02	2.203117e-02	1.773467e-02	1.732729e-02	1.707835e-02	1.98380	1.93803	1.95969	1.92976	1.76797	1.644767e-02
Inosinic acid	HILIC	1.062823e-04	4.746083e-05	1.656737e-04	6.451903e-05	3.078993e-05	4.812714e-05	3.338507e-05	4.019017e-05	2.380350e-05	2.115041e-05	2.891433e-05	2.638903e-05	4.113903e-05	4.405462e-05	2.818471e-05	7.044805e-05	3.709651e-05	4.179352e-05	4.640567e-05	2.909629e-05	3.667351e-05	3.21691	3.65285	6.55558	3.61268	3.61215	3.539341e-05
Isocitric acid	HILIC	3.231140e-03	2.036119e-03	2.280817e-03	3.013634e-03	2.765296e-03	3.224623e-03	3.443604e-03	3.299188e-03	2.630841e-03	2.637783e-03	2.812890e-03	2.727461e-03	3.172158e-03	2.977004e-03	3.167476e-03	1.209580e-03	1.220869e-03	1.158419e-03	6.413881e-04	7.509200e-04	7.874977e-04	7.29727	6.51863	5.90007	5.96458	6.66245	6.184735e-04
Itaconic acid	HILIC	3.229335e-04	1.665690e-04	1.477387e-04	1.282279e-04	1.282658e-04	1.270550e-04	9.345662e-05	1.047682e-04	2.109311e-04	1.188301e-04	9.557787e-05	1.069696e-04	1.552835e-04	2.310818e-04	1.476286e-04	1.679122e-04	1.350825e-04	1.712431e-04	1.989475e-04	2.117952e-04	1.982092e-04	1.31358	1.43673	1.36331	2.04860	2.03860	2.094069e-04
Ketoleucine	HILIC	3.979027e-04	3.319892e-04	3.458332e-04	4.641371e-04	3.353860e-04	4.526920e-04	4.544638e-04	3.949503e-04	6.603202e-04	4.471319e-04	2.661939e-04	3.565450e-04	4.742210e-04	4.176491e-04	4.750006e-04	4.569972e-04	4.639618e-04	4.432511e-04	2.777366e-04	2.727520e-04	2.918610e-04	4.70322	3.57143	3.61809	3.95080	4.29843	3.330857e-04
L-4-Hydroxyglutamate semialdehyde	HILIC	2.695742e-03	1.860033e-03	2.035390e-03	2.208272e-03	2.384005e-03	2.147435e-03	2.506295e-03	2.459791e-03	2.814378e-03	2.775176e-03	2.367595e-03	2.371621e-03	2.200348e-03	2.740857e-03	2.380574e-03	2.225619e-03	2.318156e-03	2.215025e-03	2.707740e-03	2.005082e-03	2.071224e-03	2.19072	2.18677	2.28316	2.21551	1.92485	2.071980e-03
L-Acetylcarnitine	HILIC	2.604746e-05	1.688820e-05	1.668950e-05	3.873843e-05	3.638504e-05	3.209345e-05	1.218777e-04	1.005716e-04	1.146870e-04	7.415457e-05	4.179450e-05	3.905492e-05	9.666463e-05	9.614830e-05	1.089539e-04	1.147922e-04	1.258178e-04	1.120687e-04	1.468254e-04	1.317280e-04	1.466487e-04	1.55183	1.43382	1.52740	2.23281	1.99415	2.169256e-04
L-Aspartic acid	HILIC	3.014056e-02	2.527752e-02	2.421345e-02	2.087106e-02	1.997771e-02	1.971521e-02	2.328750e-02	2.141651e-02	1.926077e-02	2.328146e-02	2.202885e-02	2.156257e-02	2.234682e-02	2.455326e-02	2.297170e-02	1.509554e-02	1.550147e-02	1.390513e-02	1.175189e-02	1.364055e-02	1.429530e-02	1.69475	1.68041	1.66572	1.49404	1.51924	1.595908e-02
L-Glutamic acid	HILIC	9.528343e-02	1.061402e-01	1.119106e-01	1.021505e-01	1.037739e-01	9.578336e-02	1.059584e-01	1.075759e-01	9.456093e-02	1.068259e-01	1.141625e-01	1.046814e-01	9.132151e-02	1.009061e-01	9.422540e-02	7.763971e-02	7.475526e-02	7.252733e-02	4.992222e-02	4.911822e-02	5.162298e-02	1.16972	1.09868	1.21879	9.47822	9.00996	9.200126e-02
L-Lactic acid	HILIC	3.813499e-02	3.225425e-02	3.499832e-02	6.073907e-02	5.827634e-02	6.000242e-02	6.860067e-02	6.230425e-02	1.045952e-01	6.126291e-02	5.319836e-02	5.683366e-02	7.020737e-02	6.621082e-02	6.969819e-02	8.975207e-02	9.554010e-02	1.029231e-01	1.478111e-01	1.194442e-01	1.201063e-01	7.67053	6.97434	6.54269	8.36286	8.61851	7.773433e-02
L-Malic acid	HILIC	1.395475e-02	1.638655e-02	1.612155e-02	1.775344e-02	1.661255e-02	1.636220e-02	1.981153e-02	1.828041e-02	1.998062e-02	1.770355e-02	1.861025e-02	1.766029e-02	1.846999e-02	1.607895e-02	1.866730e-02	1.699296e-02	1.944555e-02	1.862485e-02	6.653608e-03	8.010020e-03	8.274951e-03	1.51631	1.28914	1.34057	1.24532	1.13393	1.129253e-02
L-beta-aspartyl-L-glutamic acid	HILIC	3.970102e-04	4.080677e-04	3.568307e-04	4.096624e-04	4.323030e-04	3.961872e-04	4.744794e-04	4.298843e-04	2.773705e-04	4.567862e-04	4.313773e-04	4.190505e-04	4.045337e-04	4.588751e-04	4.464645e-04	4.980788e-04	5.340325e-04	5.310239e-04	2.025530e-04	2.635064e-04	2.727619e-04	4.69820	4.57110	4.64313	3.88731	3.74473	3.928666e-04
Malonic acid	HILIC	4.864447e-04	2.288613e-04	1.902824e-04	1.810258e-04	2.014623e-04	2.453987e-04	1.656627e-04	2.178411e-04	7.461225e-04	1.495249e-04	1.299530e-04	1.639312e-04	4.131640e-04	5.521082e-04	3.604945e-04	3.016969e-04	2.329184e-04	3.920356e-04	7.839707e-04	6.920544e-04	5.634989e-04	2.14393	2.46095	2.57065	4.91556	4.90215	5.104834e-04
Mannose 6-phosphate	HILIC	7.444026e-03	8.435551e-03	8.042897e-03	8.399123e-03	7.889156e-03	8.350573e-03	9.920792e-03	9.457497e-03	6.534055e-03	7.472188e-03	6.580501e-03	7.820262e-03	8.844038e-03	6.739629e-03	9.093391e-03	6.007951e-03	6.043423e-03	6.639897e-03	3.742344e-03	5.984649e-03	5.358606e-03	6.88171	5.35304	4.88485	5.24729	5.35660	1.873057e-03
Mesaconic acid	HILIC	5.984465e-05	3.455998e-05	3.024781e-05	2.704496e-05	3.293826e-05	3.326849e-05	2.054904e-05	2.608996e-05	5.486899e-05	2.820760e-05	2.239992e-05	2.633338e-05	4.175178e-05	6.039773e-05	3.710203e-05	3.376742e-05	3.118987e-05	3.980686e-05	5.441948e-05	5.876954e-05	5.099900e-05	2.84157	3.35496	3.18157	5.40362	5.73324	5.994128e-05
Methylmalonic acid	HILIC	7.920135e-04	4.926905e-04	4.212998e-04	4.080715e-04	4.330501e-04	4.415763e-04	3.186304e-04	3.977539e-04	9.495251e-04	4.477845e-04	3.414172e-04	3.905384e-04	5.539024e-04	7.111881e-04	5.345825e-04	2.982926e-03	3.721652e-04	5.918930e-04	8.729202e-04	6.568575e-04	6.237602e-04	3.73070	3.90511	3.78208	5.84618	6.19702	6.251664e-04
N-Acetyl-D-Glucosamine 6-Phosphate	HILIC	6.057155e-04	5.526466e-04	4.751412e-04	9.373283e-04	7.833805e-04	1.018343e-03	1.530531e-03	1.028834e-03	1.028417e-03	8.657943e-04	6.799891e-04	7.831188e-04	1.317738e-03	7.250060e-04	1.538063e-03	5.986977e-04	1.038526e-03	5.621913e-04	1.105271e-03	1.312151e-03	1.122492e-03	2.50484	3.30634	2.70120	2.49929	2.38120	4.823091e-04
N-Acetyl-L-aspartic acid	HILIC	1.572158e-02	1.762157e-02	1.813381e-02	1.494030e-02	1.486175e-02	1.366213e-02	1.344252e-02	1.336944e-02	1.089776e-02	1.573567e-02	1.707459e-02	1.519093e-02	1.337741e-02	1.307968e-02	1.387613e-02	9.096102e-03	9.658768e-03	1.029169e-02	5.842582e-03	6.610632e-03	7.684215e-03	1.19749	1.05937	1.16411	7.76427	6.87396	7.342045e-03
N-Acetyl-L-methionine	HILIC	3.228365e-04	2.592947e-04	3.017858e-04	4.883236e-04	4.858990e-04	4.598742e-04	6.195256e-04	5.917154e-04	6.645767e-04	5.252392e-04	5.462620e-04	4.917155e-04	5.875675e-04	4.919784e-04	5.682516e-04	6.389991e-04	7.646079e-04	7.735460e-04	3.651838e-04	4.350463e-04	3.917445e-04	3.84705	3.82609	4.20647	3.40217	2.97151	2.829259e-04
N-Acetyl-glucosamine 1-phosphate	HILIC	2.206586e-03	1.665279e-03	1.112780e-03	1.964726e-03	1.958608e-03	2.579393e-03	2.644142e-03	1.914761e-03	2.096953e-03	2.815732e-03	1.679124e-03	2.386412e-03	2.143809e-03	1.437331e-03	2.659087e-03	2.836940e-03	3.701898e-03	2.022160e-03	2.185738e-03	2.778836e-03	2.092132e-03	1.24799	2.11821	1.51595	1.49956	1.55248	2.231235e-03
N-Acetylglutamic acid	HILIC	4.145820e-04	4.779756e-04	4.657218e-04	4.322902e-04	4.438370e-04	4.155133e-04	5.374449e-04	5.344783e-04	4.450777e-04	4.255619e-04	4.939942e-04	4.351191e-04	4.686977e-04	4.914999e-04	4.888390e-04	1.049947e-03	1.111340e-03	1.215309e-03	4.683833e-04	5.798773e-04	6.033682e-04	1.26169	1.10574	1.23591	1.42851	1.37382	1.434075e-03
N-Acetylleucine	HILIC	7.740338e-05	4.546733e-05	3.483205e-05	3.282975e-05	4.680838e-05	4.333861e-05	2.609464e-05	2.404187e-05	3.302703e-05	4.962071e-05	2.981926e-05	3.366899e-05	2.630540e-05	4.175203e-05	2.991135e-05	8.665801e-05	4.250489e-05	2.757327e-05	2.123921e-05	3.471217e-05	3.394182e-05	3.86122	6.54266	4.50271	3.85457	4.38153	7.917472e-05
N-Acetylneuraminic acid	HILIC	3.384134e-03	2.373954e-03	2.079074e-03	2.471449e-03	2.174838e-03	2.415844e-03	2.177733e-03	1.885437e-03	1.897640e-03	2.613314e-03	1.700880e-03	1.876231e-03	1.907088e-03	1.633872e-03	1.951390e-03	3.848690e-03	4.078544e-03	3.359379e-03	3.803159e-03	5.461025e-03	5.290044e-03	2.79818	3.76282	3.04937	2.95313	2.81874	2.681043e-03
N-Formyl-L-methionine	HILIC	1.572231e-05	1.445160e-05	1.630070e-05	2.009257e-05	2.038458e-05	1.727507e-05	1.977683e-05	1.708696e-05	2.039810e-05	2.759458e-05	3.066572e-05	2.505676e-05	2.414209e-05	1.817407e-05	2.404907e-05	2.207185e-05	2.383684e-05	2.240078e-05	1.649398e-05	1.720477e-05	1.826815e-05	2.67094	2.43506	2.56027	2.30844	2.02055	1.870833e-05
N-acetyl-alpha-D-galactosamine 1-phosphate	HILIC	3.532209e-04	4.272643e-04	3.108456e-04	5.843876e-04	4.667421e-04	5.160094e-04	8.191624e-04	3.939327e-04	4.222508e-04	1.958527e-04	3.503772e-04	3.409781e-04	6.423193e-04	3.134796e-04	7.533598e-04	4.194432e-04	6.100354e-04	2.972156e-04	6.744513e-04	6.007791e-04	5.170669e-04	1.74956	2.54087	1.96492	1.83262	1.41325	3.519749e-04
NADH	HILIC	4.358151e-05	5.115704e-05	8.326068e-05	2.543742e-05	2.889117e-05	1.215778e-05	6.444603e-07	5.229124e-06	2.322347e-06	6.061400e-06	1.723610e-06	4.045588e-06	5.378485e-07	1.556875e-06	3.270284e-06	1.245078e-05	4.805330e-06	4.299655e-07	1.969901e-06	4.735468e-06	4.789352e-07	6.16378	1.24679	6.40100	9.50011	3.31898	3.568286e-05
O-Phosphoethanolamine	HILIC	1.039659e-04	6.078988e-05	6.824739e-05	6.452900e-05	6.186331e-05	7.711289e-05	5.664215e-05	5.930717e-05	4.222278e-05	6.098720e-05	6.351129e-05	7.216757e-05	6.832227e-05	5.351713e-05	5.090168e-05	1.259721e-04	1.560391e-04	1.532841e-04	8.798291e-05	1.263592e-04	1.431336e-04	6.94955	7.48161	9.40176	6.65166	6.84624	7.441868e-04
Orotidine	HILIC	1.962369e-05	2.566668e-05	2.867631e-05	2.575939e-05	2.696785e-05	2.200607e-05	2.929383e-05	2.790139e-05	1.944725e-05	2.417400e-05	3.185166e-05	2.621236e-05	2.800018e-05	2.136812e-05	2.779741e-05	2.898868e-05	2.754305e-05	3.072069e-05	2.194658e-05	2.097905e-05	2.513710e-05	1.62697	1.58961	1.99495	2.23498	1.79838	1.737897e-05
Oxalacetic acid	HILIC	3.466871e-05	1.969413e-05	2.104348e-05	2.612161e-05	2.517804e-05	3.036371e-05	2.812209e-05	2.658962e-05	4.215242e-05	2.230158e-05	2.287236e-05	2.542505e-05	3.286361e-05	3.168059e-05	3.513594e-05	1.100072e-05	1.330365e-05	1.303389e-05	3.529413e-06	5.873390e-06	7.036682e-06	6.56651	5.22023	3.41546	3.44485	4.34485	4.809381e-06
Oxalic acid	HILIC	4.399356e-03	7.870282e-04	1.014847e-03	9.456164e-04	3.245237e-04	7.310075e-04	5.240779e-04	7.244939e-04	4.807491e-04	8.837160e-04	4.347005e-04	4.696552e-04	6.383241e-04	3.439271e-04	4.059221e-04	9.840481e-04	5.536735e-04	7.758796e-04	1.271373e-03	6.757605e-04	8.365697e-04	6.53250	7.37375	6.25867	7.56345	3.32856	1.104759e-03
Oxoglutaric acid	HILIC	1.740322e-03	2.104198e-04	2.149319e-04	2.782956e-04	2.674081e-04	2.289607e-04	2.277202e-04	2.103411e-04	2.351977e-04	2.715375e-04	2.525812e-04	2.247873e-04	2.430259e-04	1.608592e-04	2.828631e-04	1.450436e-04	9.254968e-05	1.192836e-04	4.890168e-05	5.295104e-05	5.483495e-05	1.44469	1.00506	9.21671	1.12535	1.45844	2.243308e-04
Pantothenic acid	HILIC	3.103349e-03	2.449613e-03	2.510122e-03	2.538903e-03	2.577464e-03	2.560720e-03	1.878837e-03	1.886575e-03	2.228455e-03	3.776989e-03	3.298191e-03	3.263451e-03	2.506103e-03	2.436332e-03	2.475141e-03	2.137852e-03	2.348746e-03	2.201965e-03	1.775259e-03	1.818935e-03	1.741358e-03	2.79626	2.84775	3.10261	2.45686	2.22893	2.400696e-03
Phenylpyruvic acid	HILIC	2.442507e-04	1.163741e-04	1.015210e-04	9.404368e-05	9.848082e-05	9.165215e-05	7.327201e-05	7.540318e-05	9.233027e-05	1.214652e-04	8.253311e-05	8.830007e-05	6.369102e-05	9.922244e-05	7.085284e-05	9.946879e-05	9.363330e-05	9.598259e-05	1.038937e-04	8.861777e-05	8.239844e-05	1.08269	1.17643	1.12901	9.97410	1.10848	1.326496e-04
Phosphoadenosine phosphosulfate	HILIC	9.941406e-05	1.365758e-04	1.604695e-04	1.199046e-04	1.181917e-04	1.029914e-04	1.242836e-04	1.510794e-04	2.750911e-05	1.086686e-04	1.498767e-04	1.241935e-04	8.148069e-05	6.902819e-05	9.306886e-05	7.519508e-05	7.301748e-05	2.955495e-05	2.566600e-05	3.567011e-05	4.761221e-05	1.11840	7.96242	9.06764	7.55406	6.68926	6.586377e-05
Phosphocreatine	HILIC	4.230638e-04	4.887775e-04	5.033570e-04	3.383172e-04	3.503801e-04	2.698074e-04	3.235598e-04	2.886296e-04	1.770455e-04	4.057428e-04	2.840880e-04	2.787587e-04	1.744263e-04	1.045213e-04	1.561591e-04	1.740469e-04	1.899504e-04	1.146512e-04	1.017134e-04	9.160163e-05	1.713455e-04	1.59713	9.07273	9.70093	9.11944	1.10680	1.855249e-04
Phosphoenolpyruvic acid	HILIC	5.033772e-03	6.331752e-03	7.183436e-03	5.821602e-03	5.215353e-03	4.547931e-03	6.818309e-03	6.801702e-03	3.781833e-03	4.370054e-03	5.865270e-03	4.829567e-03	4.656401e-03	4.043212e-03	5.075699e-03	4.285990e-03	5.881378e-03	2.459534e-03	1.989659e-03	2.721680e-03	2.814446e-03	3.97673	2.93933	2.83087	2.32655	2.13005	2.865962e-03
Phosphoribosyl pyrophosphate	HILIC	2.468032e-04	4.394324e-04	5.217672e-04	8.761787e-05	1.355678e-04	3.396586e-05	1.006117e-04	1.642425e-04	1.974797e-05	3.293384e-05	1.712034e-04	1.197043e-04	3.889429e-05	6.780108e-05	4.339694e-05	6.261528e-05	6.795628e-05	3.263806e-05	2.783333e-05	3.657112e-05	5.436686e-05	1.41865	2.84806	5.66665	2.55142	1.99522	7.236028e-05
Phosphoric acid	HILIC	2.533751e-01	2.519124e-01	2.252220e-01	2.129892e-01	2.226852e-01	2.379194e-01	1.845961e-01	1.869342e-01	2.842060e-01	2.331632e-01	1.938425e-01	2.213311e-01	2.538301e-01	2.723100e-01	2.485007e-01	2.439858e-01	2.333478e-01	2.880541e-01	3.879452e-01	3.660607e-01	3.286907e-01	2.69297	3.11546	2.97240	3.45076	3.61117	3.783608e-01
Pyridoxal 5'-phosphate	HILIC	7.313037e-05	9.074484e-05	8.980450e-05	8.671386e-05	8.682112e-05	7.374415e-05	1.279670e-04	9.134556e-05	1.152189e-04	6.996242e-05	1.034139e-04	1.101379e-04	9.337890e-05	7.091644e-05	1.068566e-04	1.152617e-04	1.197683e-04	8.463890e-05	1.414340e-04	1.783672e-04	1.440341e-04	1.81287	1.48228	1.25536	1.33803	1.36435	1.488633e-04
Pyroglutamic acid	HILIC	1.261847e-02	8.399950e-03	8.621287e-03	1.142152e-02	9.722959e-03	1.057636e-02	1.742234e-02	1.560204e-02	1.721507e-02	1.285955e-02	1.031140e-02	1.139394e-02	1.448794e-02	1.290389e-02	1.447351e-02	1.311645e-02	1.560877e-02	1.390452e-02	1.213583e-02	1.366736e-02	1.544980e-02	1.60909	1.30098	1.34708	1.51917	1.60502	1.367492e-02
Pyruvic acid	HILIC	3.516078e-04	2.230194e-04	2.456737e-04	1.884614e-04	2.136353e-04	1.910843e-04	2.584291e-04	2.389239e-04	2.810519e-04	2.157485e-04	1.914999e-04	2.377583e-04	1.878565e-04	2.956843e-04	1.824762e-04	3.165564e-04	2.256924e-04	8.039438e-04	4.134674e-04	3.148325e-04	2.664333e-04	2.84934	2.23774	2.35175	2.33048	2.58738	4.390603e-04
Riboflavin cyclic-4',5'-phosphate	HILIC	3.841485e-04	2.682204e-04	2.300854e-04	3.114210e-04	2.452619e-04	2.895175e-04	3.209672e-04	2.711842e-04	2.479642e-04	2.514549e-04	2.919253e-04	2.906687e-04	3.074559e-04	2.209478e-04	2.729809e-04	3.535010e-04	4.018585e-04	3.259999e-04	4.347145e-04	4.583963e-04	4.315716e-04	2.75523	3.25513	2.64831	2.84962	2.76768	2.402087e-04
Ribonic acid	HILIC	6.548399e-04	5.089809e-04	2.627705e-04	2.415380e-04	2.196642e-04	2.351097e-04	2.604463e-04	2.333019e-04	2.488515e-04	2.914481e-04	2.317716e-04	2.304320e-04	2.263384e-04	2.478385e-04	2.372519e-04	1.428231e-03	2.604968e-04	3.258899e-04	2.838112e-04	2.361199e-04	2.563670e-04	2.12775	1.82497	1.95716	2.26956	2.08561	2.117648e-04
Ribose 1,5-bisphosphate	HILIC	5.801439e-05	6.219377e-05	6.058939e-05	6.963754e-05	6.870045e-05	6.741771e-05	8.272405e-05	8.061029e-05	5.189151e-05	5.722267e-05	6.972048e-05	6.536372e-05	7.349704e-05	6.759217e-05	7.401986e-05	1.552002e-04	1.900898e-04	1.092524e-04	1.733313e-04	2.098376e-04	2.347716e-04	8.63721	7.60671	8.09919	9.24259	7.73768	8.403105e-05
Ribose 1-phosphate	HILIC	3.887723e-04	3.678916e-04	3.253124e-04	3.388795e-04	3.457097e-04	3.815672e-04	2.221632e-04	2.205558e-04	2.162080e-04	3.696463e-04	2.971361e-04	3.187898e-04	2.567602e-04	2.267142e-04	2.740390e-04	4.695580e-04	4.239008e-04	2.827047e-04	2.333430e-04	3.057587e-04	3.353674e-04	4.77525	5.84982	5.75031	5.72686	5.57456	5.970272e-04
Sedoheptulose 1,7-bisphosphate	HILIC	2.457115e-05	2.973072e-05	3.297630e-05	3.018022e-05	2.959466e-05	3.001236e-05	2.551290e-05	2.774031e-05	1.283901e-05	2.111134e-05	2.904319e-05	2.508203e-05	1.705340e-05	1.334111e-05	1.706187e-05	1.091074e-04	1.272109e-04	5.769646e-05	1.943818e-05	3.274548e-05	3.258110e-05	5.14726	4.38280	4.91242	4.78306	5.02032	4.904379e-05
Succinic acid	HILIC	2.348035e-03	1.932763e-03	1.569959e-03	1.504327e-03	1.599812e-03	1.609233e-03	1.411425e-03	1.507459e-03	2.078784e-03	1.693254e-03	1.723221e-03	1.680748e-03	1.734781e-03	1.955965e-03	1.641626e-03	2.246153e-02	9.189923e-04	2.187941e-03	1.185600e-03	1.018221e-03	1.003039e-03	1.32666	1.03128	1.11025	1.17775	1.08945	1.491966e-03
Succinic acid semialdehyde	HILIC	1.563309e-03	2.419648e-04	2.783819e-04	2.874233e-04	2.688216e-04	2.541945e-04	2.646676e-04	2.573594e-04	2.719902e-04	3.191000e-04	2.814532e-04	2.762360e-04	3.004573e-04	2.480467e-04	3.151168e-04	1.672184e-04	1.206791e-04	1.610627e-04	1.283085e-04	1.100247e-04	9.768518e-05	1.94967	1.76615	1.57991	1.68485	2.03260	2.401096e-04
Sulfate	HILIC	2.709938e-02	2.820550e-02	2.500536e-02	2.402368e-02	1.835585e-02	2.284604e-02	2.422823e-02	2.076140e-02	1.611987e-02	2.104711e-02	1.959941e-02	2.315652e-02	1.928680e-02	1.694475e-02	2.052352e-02	2.702740e-02	2.724305e-02	2.471193e-02	1.921589e-02	2.120084e-02	2.534436e-02	2.57959	2.20582	2.12000	2.08290	2.38876	1.938351e-02
Taurine	HILIC	1.667149e-02	1.564868e-02	1.654876e-02	1.673460e-02	1.811922e-02	1.527793e-02	1.886423e-02	2.040584e-02	1.786842e-02	1.735360e-02	1.742300e-02	1.646085e-02	1.667539e-02	1.797684e-02	1.736029e-02	1.897779e-02	1.447622e-02	1.517874e-02	1.611150e-02	1.820330e-02	2.073569e-02	1.06359	1.02086	1.10679	1.05820	8.52263	9.783043e-03
Thiamine pyrophosphate	HILIC	1.704958e-06	8.813165e-06	8.569780e-06	1.165472e-05	9.573218e-06	1.058700e-05	1.439483e-05	1.201646e-05	8.999816e-06	1.034798e-05	1.368846e-05	1.146965e-05	1.183532e-05	1.001229e-05	1.432796e-05	1.846921e-05	1.915072e-05	9.257975e-06	1.826199e-05	1.845667e-05	2.056588e-05	1.73124	1.78631	1.69363	2.08260	1.85396	1.586973e-05
Threonic acid	HILIC	1.184369e-03	9.742033e-04	8.855865e-04	8.419878e-04	8.394356e-04	7.482920e-04	1.080875e-03	8.332898e-04	7.988753e-04	1.158752e-03	9.380546e-04	8.908288e-04	9.768339e-04	1.040420e-03	1.020648e-03	9.799366e-04	6.588597e-04	6.643964e-04	6.800733e-04	4.930717e-04	5.533984e-04	5.08973	4.14514	4.56028	3.32597	3.15341	3.130089e-04
Thymidine 5'-triphosphate	HILIC	3.078833e-05	5.247162e-05	6.343920e-05	1.749108e-05	1.899429e-05	1.236588e-05	2.723373e-05	3.380558e-05	6.288241e-06	1.563059e-05	4.177658e-05	4.575508e-05	2.516331e-05	1.714838e-05	2.936887e-05	1.381111e-05	1.310920e-05	1.350805e-05	4.952238e-06	1.372595e-05	1.591567e-05	3.42228	2.21863	1.84279	2.23228	2.11251	2.087111e-05
Ureidosuccinic acid	HILIC	7.633525e-05	4.316064e-05	5.525391e-05	5.279948e-05	5.677237e-05	5.553239e-05	3.679429e-05	4.086471e-05	2.238993e-05	6.381495e-05	6.656577e-05	5.587399e-05	2.574249e-05	2.459121e-05	2.470428e-05	4.770782e-05	3.867273e-05	4.420253e-05	8.493372e-07	5.123088e-07	8.744305e-07	2.19838	1.55450	1.81590	1.21549	1.10324	1.073754e-05
Uridine	HILIC	1.609596e-03	1.864668e-03	1.951328e-03	2.058914e-03	1.864794e-03	1.872431e-03	2.399785e-03	2.359574e-03	2.504620e-03	1.718454e-03	1.964920e-03	2.066542e-03	2.388517e-03	2.756738e-03	2.547965e-03	1.559890e-03	1.711578e-03	1.772925e-03	7.840028e-04	1.119690e-03	9.769984e-04	1.06938	8.61945	8.83166	8.83568	8.85175	5.202625e-04
Uridine 5'-diphosphate	HILIC	4.369719e-03	5.519581e-03	6.857446e-03	4.013290e-03	3.888188e-03	3.583861e-03	1.914359e-03	2.300818e-03	1.426512e-03	2.757779e-03	2.653909e-03	2.403691e-03	1.693872e-03	1.812324e-03	1.712320e-03	2.280430e-03	1.594191e-03	1.210711e-03	1.120877e-03	1.111741e-03	1.315948e-03	1.62648	1.53232	1.90136	1.25126	1.12865	1.103674e-03
Uridine 5'-monophosphate	HILIC	2.147601e-02	2.452345e-02	2.515747e-02	2.607678e-02	2.520813e-02	2.607825e-02	2.619724e-02	2.578127e-02	1.895545e-02	2.460978e-02	2.683754e-02	2.516193e-02	2.330600e-02	2.220155e-02	2.428108e-02	2.057930e-02	2.099429e-02	2.016224e-02	1.020581e-02	1.168712e-02	1.163531e-02	1.63317	1.66553	1.61290	1.36127	1.32749	1.227624e-02
Uridine diphosphate glucose	HILIC	1.573405e-03	1.732019e-03	1.761885e-03	1.716573e-03	1.622158e-03	1.715433e-03	1.711998e-03	1.555166e-03	1.046371e-03	1.594193e-03	1.799273e-03	1.640314e-03	1.583707e-03	1.440829e-03	1.455507e-03	1.223360e-03	1.327180e-03	1.073105e-03	8.890554e-04	8.649345e-04	8.800977e-04	1.04996	1.06568	8.85127	6.13112	6.67675	6.492684e-04
Uridine diphosphate-N-acetylgalactosamine	HILIC	1.416102e-03	1.590647e-03	1.452309e-03	1.578093e-03	1.493685e-03	1.488240e-03	1.617008e-03	1.520843e-03	1.348567e-03	1.383906e-03	1.532024e-03	1.414616e-03	1.443249e-03	1.314190e-03	1.433231e-03	1.012239e-03	1.087120e-03	9.645161e-04	1.037585e-03	1.333193e-03	1.389637e-03	7.08314	8.10061	7.53131	6.31106	5.75452	5.021971e-04
Uridine diphosphate-N-acetylglucosamine	HILIC	3.110293e-03	3.323414e-03	3.504149e-03	3.531759e-03	3.578517e-03	3.547878e-03	3.564445e-03	3.605538e-03	2.950595e-03	3.609895e-03	3.935454e-03	3.661777e-03	3.304389e-03	3.317672e-03	2.986568e-03	2.308006e-03	2.345505e-03	2.322978e-03	3.212259e-03	2.591755e-03	3.002773e-03	1.72902	1.80278	1.86203	1.43624	1.38601	1.041418e-03
Uridine triphosphate	HILIC	1.039058e-03	1.682525e-03	2.350546e-03	1.202912e-03	1.312693e-03	8.571626e-04	1.394435e-03	1.687314e-03	1.245587e-03	9.529604e-04	1.514668e-03	1.640175e-03	1.205188e-03	1.147269e-03	1.428223e-03	7.502014e-04	7.633725e-04	6.868960e-04	6.984388e-04	1.124347e-03	9.265431e-04	9.94598	6.88188	6.74591	8.23319	8.26367	8.259448e-04
Xanthylic acid	HILIC	1.247898e-05	1.235379e-05	1.751758e-05	8.358699e-06	8.970074e-06	6.280169e-06	6.114926e-06	8.242135e-06	0.000000e+00	8.828173e-06	1.285510e-05	1.084393e-05	2.167472e-06	4.225899e-06	3.185370e-06	6.454964e-06	4.865568e-06	4.595816e-06	0.000000e+00	1.625022e-06	2.698050e-06	1.07313	5.61440	8.07867	4.08904	2.55096	0.000000e+00
cis-Aconitic acid	HILIC	2.044487e-04	1.932982e-04	2.125919e-04	2.899174e-04	2.632768e-04	3.080353e-04	3.326227e-04	2.989585e-04	4.019648e-04	2.433087e-04	2.652896e-04	2.521393e-04	3.208156e-04	2.901688e-04	3.004145e-04	1.108530e-04	1.003128e-04	9.757647e-05	8.916158e-05	8.778113e-05	8.336342e-05	6.27091	5.44604	5.28289	6.42996	7.02984	6.303149e-05
dADP	HILIC	7.381145e-05	9.059393e-05	9.680382e-05	5.812657e-05	5.292631e-05	6.605569e-05	2.933828e-05	3.486122e-05	1.882892e-05	4.136142e-05	6.106446e-05	5.203321e-05	2.303764e-05	2.360366e-05	2.338219e-05	7.417946e-05	6.416068e-05	4.139495e-05	5.912806e-05	5.249742e-05	6.490681e-05	6.10202	5.26408	6.38998	4.94834	5.14600	3.814867e-05
dCDP	HILIC	1.625143e-04	1.934695e-04	1.837301e-04	1.548743e-04	1.644976e-04	1.575379e-04	1.103185e-04	1.291399e-04	7.418704e-05	1.465158e-04	1.642050e-04	1.276743e-04	1.177695e-04	1.329600e-04	1.000124e-04	1.337445e-04	1.271787e-04	5.234698e-05	5.912595e-05	4.578048e-05	6.632047e-05	1.08128	9.67071	1.28849	8.54154	7.42937	6.956901e-05
dCMP	HILIC	3.727842e-05	4.768269e-05	4.100736e-05	5.022197e-05	4.861335e-05	5.074944e-05	5.841173e-05	4.317826e-05	1.373077e-05	5.573646e-05	6.292612e-05	5.678904e-05	4.347229e-05	3.059896e-05	4.166798e-05	5.242323e-05	5.277312e-05	8.476397e-05	1.413036e-05	1.803083e-05	3.034354e-05	4.53111	4.18904	4.62004	3.21067	3.01896	2.556999e-05
dCTP	HILIC	2.212222e-05	4.011572e-05	4.278253e-05	2.571052e-05	2.724158e-05	1.920255e-05	5.821990e-05	6.278850e-05	4.621246e-05	2.592822e-05	4.911746e-05	5.963967e-05	4.232500e-05	3.483396e-05	5.998651e-05	2.095725e-05	2.422523e-05	1.877127e-05	1.820487e-05	3.904118e-05	2.980865e-05	4.37412	2.31400	1.91714	3.13600	3.11761	2.655584e-05
dGTP	HILIC	9.420167e-04	1.274216e-03	1.390855e-03	1.539132e-03	1.523465e-03	1.450894e-03	1.993635e-03	1.855512e-03	1.584567e-03	1.242857e-03	1.814991e-03	1.735418e-03	1.563789e-03	1.075781e-03	1.486167e-03	1.537654e-03	1.911684e-03	1.084748e-03	1.675891e-03	1.894701e-03	1.794016e-03	1.75313	1.69941	1.59946	1.48377	1.51902	1.185720e-03
dTDP	HILIC	7.950082e-04	1.024307e-03	1.059709e-03	3.625873e-04	3.998714e-04	3.632154e-04	1.861099e-04	1.918680e-04	1.224519e-04	4.014855e-04	4.226580e-04	3.380445e-04	2.261334e-04	2.008757e-04	2.139244e-04	2.969386e-04	2.139479e-04	1.260589e-04	1.316540e-04	1.103885e-04	1.384406e-04	3.11516	2.77284	3.91864	2.24360	1.84661	1.765211e-04
dUMP	HILIC	1.019961e-05	1.504455e-05	1.722774e-05	7.800793e-06	1.091682e-05	8.021245e-06	1.082801e-05	1.493106e-05	5.145971e-06	1.021344e-05	1.704056e-05	1.150464e-05	3.959972e-05	6.531809e-05	2.040057e-05	1.131617e-05	9.158578e-06	1.441878e-05	4.188082e-06	4.826003e-06	8.740688e-06	1.93984	1.09784	1.65046	1.34070	1.14846	4.439621e-06
(1E)-N-Hydroxy-4-(methylsulfanyl)-1-butanimine	IC-MS	3.224726e-04	3.090933e-04	2.953335e-04	2.797839e-04	2.936632e-04	3.143986e-04	2.419662e-04	2.527208e-04	2.660184e-04	3.113346e-04	2.612168e-04	2.795150e-04	2.763490e-04	2.461758e-04	2.759591e-04	3.000166e-04	2.700955e-04	2.876285e-04	3.037263e-04	3.124310e-04	3.061070e-04	2.88546	3.10662	3.02490	3.08161	3.34171	3.737349e-04
(2R)-2,3-Dihydroxypropanoic acid	IC-MS	2.623748e-04	2.146741e-04	1.848340e-04	1.739681e-04	1.426574e-04	1.468854e-04	1.565156e-04	1.320033e-04	1.218289e-04	1.797909e-04	1.683256e-04	1.502771e-04	1.434748e-04	1.183645e-04	1.405496e-04	1.710393e-04	1.562772e-04	1.211701e-04	1.291530e-04	1.285946e-04	1.292428e-04	1.32090	1.22621	9.85815	1.23728	1.10883	1.209184e-04
(2S)-3-Hydroxy-2-(octanoyloxy)propyl decanoate	IC-MS	1.348651e-02	1.138346e-02	1.318082e-02	1.103561e-02	1.308260e-02	1.431251e-02	1.033862e-02	1.182213e-02	1.349268e-02	1.366439e-02	1.272424e-02	1.170739e-02	1.135342e-02	1.037938e-02	1.286118e-02	1.213419e-02	1.264246e-02	1.346461e-02	1.206263e-02	1.339848e-02	1.558720e-02	1.12638	1.54715	1.43382	1.51618	1.36976	1.752122e-02
(3S,5R,10R,12S,14S,15R,16R)-3,5,10,14,15-Pentahydr	IC-MS	6.000912e-04	6.160055e-04	5.613764e-04	5.995863e-04	6.573258e-04	6.495886e-04	4.642758e-04	5.234027e-04	5.632052e-04	5.538619e-04	5.072199e-04	6.952754e-04	3.991670e-04	4.170740e-04	4.942082e-04	5.658805e-04	6.214254e-04	6.569757e-04	7.677758e-04	5.626203e-04	6.652138e-04	6.46987	5.90457	6.09057	5.50380	6.37148	6.582708e-04
(E)-p-coumaric acid	IC-MS	3.064549e-04	3.522424e-04	3.596714e-04	3.778403e-04	3.375406e-04	3.355799e-04	5.111747e-04	4.674725e-04	4.077747e-04	2.984716e-04	3.521808e-04	3.368032e-04	4.901522e-04	5.052122e-04	4.489100e-04	3.911303e-04	4.417596e-04	4.437406e-04	4.226169e-04	4.197376e-04	4.144169e-04	5.22837	4.59061	4.69819	4.65007	4.30205	3.287617e-04
(R)-3-hydroxybutyrylcarnitine	IC-MS	1.464329e-05	1.777883e-05	1.683669e-05	1.493637e-05	1.494640e-05	1.407529e-05	2.968771e-05	2.952804e-05	2.097813e-05	7.986806e-06	8.813550e-06	8.898101e-06	1.168057e-05	1.409230e-05	1.327829e-05	2.066093e-04	2.108045e-04	2.212507e-04	2.745357e-04	2.718631e-04	2.772985e-04	1.62019	1.63490	1.66500	1.60799	1.42578	1.401359e-04
.alpha.-Aminoadipic acid	IC-MS	2.033517e-04	2.233877e-04	2.397268e-04	2.655040e-04	2.802075e-04	2.244715e-04	3.235095e-04	3.564416e-04	2.638549e-04	2.712027e-04	3.591852e-04	2.897434e-04	3.229278e-04	3.654804e-04	3.186388e-04	2.621283e-04	2.496376e-04	2.740092e-04	2.680205e-04	2.679438e-04	2.697776e-04	1.86694	1.67328	1.90154	1.98745	1.60072	1.665555e-04
1,2-dihydroxyheptadec-16-yn-4-yl acetate	IC-MS	4.034481e-04	3.150208e-04	3.788514e-04	2.910937e-04	3.854826e-04	4.227360e-04	2.827434e-04	3.341500e-04	3.960552e-04	3.963745e-04	3.568306e-04	3.230090e-04	2.883378e-04	2.668045e-04	3.490945e-04	3.165404e-04	3.628440e-04	3.637261e-04	3.336059e-04	3.721742e-04	4.238022e-04	3.38862	4.16862	4.13521	4.14877	3.84661	4.603328e-04
1-(beta-D-Ribofuranosyl)-1,3,4,7-tetrahydro-2H-1,3-diaze	IC-MS	4.884868e-05	5.901568e-05	7.156139e-05	9.021216e-05	9.286471e-05	7.229663e-05	1.090458e-04	9.379349e-05	8.613994e-05	7.752756e-05	9.346767e-05	8.347936e-05	8.848601e-05	7.646332e-05	7.825152e-05	9.020110e-05	9.304315e-05	8.884394e-05	4.099393e-05	4.754566e-05	3.812506e-05	9.11261	7.86900	1.04190	6.28933	6.74100	6.108240e-05
1-(Propyldisulfanyl)-1-(propylsulfinyl)propane	IC-MS	1.552793e-03	2.103846e-03	8.501419e-04	1.876538e-03	1.267322e-03	1.002694e-03	1.109033e-03	1.100180e-03	9.055495e-04	2.029206e-03	1.496711e-03	1.259875e-03	9.993471e-04	6.394052e-04	8.626207e-04	1.169524e-03	1.119451e-03	1.065311e-03	1.307037e-03	9.684819e-04	9.351566e-04	1.49776	1.30919	1.28250	1.13651	1.15058	1.542007e-03
1-ACETYL-3-MONOSTEARIN	IC-MS	2.876917e-04	2.529710e-04	2.746011e-04	2.491477e-04	2.946870e-04	3.133694e-04	2.274213e-04	2.582305e-04	3.267052e-04	3.084054e-04	3.043878e-04	2.572049e-04	2.634860e-04	2.430313e-04	3.012942e-04	3.005701e-04	2.839930e-04	2.991668e-04	2.707610e-04	3.090559e-04	3.548565e-04	2.31612	3.57510	3.36628	3.32742	2.83622	4.117004e-04
1-Hexadecyllysophosphatidylcholine	IC-MS	9.904642e-05	9.155048e-05	9.168985e-05	1.032199e-04	7.095957e-05	8.632504e-05	1.817841e-04	1.563645e-04	1.387319e-04	8.104909e-05	1.054597e-04	8.824805e-05	1.375878e-04	1.403447e-04	1.047380e-04	6.124983e-05	6.879333e-05	4.791545e-05	5.149306e-05	5.261359e-05	3.925225e-05	1.65797	1.68529	1.71362	1.17869	7.24494	4.938092e-06
1-Methyladenosine	IC-MS	3.035840e-04	3.089953e-04	2.839567e-04	4.813378e-04	4.067909e-04	4.855330e-04	6.441737e-04	5.561473e-04	5.142180e-04	6.030147e-04	5.637176e-04	5.935160e-04	6.413587e-04	5.767940e-04	5.889931e-04	6.114304e-04	6.614486e-04	6.037242e-04	7.236041e-04	8.070373e-04	6.653935e-04	4.23169	5.52026	4.81871	4.38231	3.72566	3.984980e-04
1-Oleoyl-2-hydroxy-sn-glycero-3-PE	IC-MS	8.604927e-05	9.290293e-05	7.776513e-05	1.489984e-04	1.194274e-04	1.319654e-04	2.189252e-04	1.888493e-04	1.317215e-04	9.108042e-05	1.178892e-04	9.850756e-05	1.013570e-04	1.054636e-04	8.157156e-05	2.160520e-04	2.278337e-04	2.215509e-04	1.733372e-04	2.005048e-04	1.484178e-04	8.71297	8.47091	7.62705	6.67914	4.36341	3.901736e-05
1-oleoyl-sn-glycero-3-phospho-D-myo-inositol	IC-MS	7.787668e-05	9.016362e-05	8.750776e-05	1.023266e-04	8.264735e-05	9.148432e-05	2.006415e-04	1.578594e-04	1.538604e-04	7.655338e-05	1.004306e-04	7.952684e-05	1.458443e-04	1.393429e-04	1.100005e-04	9.105255e-05	1.184036e-04	9.772673e-05	8.381634e-05	9.357570e-05	6.835249e-05	4.97152	5.73957	5.45805	3.94009	3.54465	2.826403e-05
1-Palmitoyl-2-hydroxy-sn-glycero-3-PE	IC-MS	8.424263e-05	9.068156e-05	8.005653e-05	1.433790e-04	9.662149e-05	1.259941e-04	1.841523e-04	1.482820e-04	1.153452e-04	9.515617e-05	1.077551e-04	1.036606e-04	1.004944e-04	9.439837e-05	7.669155e-05	1.872730e-04	2.121459e-04	1.532856e-04	1.566368e-04	1.630350e-04	1.225492e-04	6.25660	5.98374	6.20180	5.69887	3.32825	2.789629e-05
1-Pentofuranosyl-2,4(1H,3H)-pyrimidinedione	IC-MS	2.535092e-03	2.948096e-03	2.850405e-03	2.707365e-03	2.494243e-03	2.336983e-03	3.446361e-03	3.297166e-03	2.584868e-03	2.842821e-03	2.961892e-03	3.154808e-03	2.870954e-03	3.143480e-03	2.792391e-03	1.757318e-03	2.022909e-03	1.846460e-03	1.132026e-03	1.184767e-03	1.152650e-03	1.22424	9.79028	7.37088	6.36776	6.38319	3.427913e-04
1-Stearoyl-2-hydroxy-sn-glycero-3-PE	IC-MS	3.088951e-04	2.938816e-04	2.608615e-04	4.883860e-04	4.118138e-04	4.760324e-04	6.741153e-04	5.416518e-04	4.158164e-04	3.393844e-04	3.777674e-04	3.451009e-04	3.584370e-04	3.272416e-04	2.778742e-04	6.313313e-04	6.930482e-04	6.407456e-04	5.717878e-04	6.601707e-04	5.036470e-04	2.30732	2.37662	2.13803	2.03855	1.52310	1.171996e-04
1-Stearoylglycerol	IC-MS	8.254664e-04	7.530242e-04	7.918340e-04	7.588191e-04	8.526290e-04	9.251188e-04	7.683580e-04	8.569843e-04	1.034752e-03	9.419208e-04	8.759693e-04	9.291915e-04	9.038872e-04	7.823152e-04	9.716157e-04	8.741755e-04	8.458218e-04	9.606017e-04	9.655066e-04	1.065363e-03	1.266357e-03	7.55770	1.07018	1.07893	1.08339	9.64597	1.401344e-03
11(Z),14(Z)-Eicosadienoic acid	IC-MS	7.388748e-04	5.790322e-04	5.632299e-04	1.013973e-03	8.134642e-04	1.027480e-03	1.163314e-03	9.329615e-04	9.551528e-04	9.353912e-04	9.761550e-04	8.631243e-04	9.289048e-04	8.890813e-04	7.747822e-04	1.164479e-03	1.330464e-03	1.179365e-03	1.119330e-03	1.274099e-03	9.993949e-04	8.11617	1.02361	7.72499	7.56613	6.10825	4.314901e-04
11(Z)-Eicosenoic acid	IC-MS	3.080851e-03	2.613182e-03	2.648354e-03	3.903464e-03	2.933188e-03	3.428251e-03	4.666947e-03	3.559303e-03	3.770347e-03	3.576851e-03	3.767687e-03	3.015696e-03	3.840587e-03	3.814193e-03	3.253454e-03	3.503446e-03	4.224311e-03	3.800963e-03	2.901395e-03	3.295392e-03	2.627002e-03	2.25999	2.90886	2.28965	1.65792	1.35567	1.011947e-03
12,13-Epoxytrichothec-9-ene-3,4,8,15-tetrol	IC-MS	4.302383e-04	7.727498e-04	3.447989e-04	6.138423e-04	4.437042e-04	4.043704e-04	4.519552e-04	5.328789e-04	5.185145e-04	4.584627e-04	2.711748e-04	1.177101e-03	2.955795e-04	5.548836e-04	6.379664e-04	4.854283e-04	6.074625e-04	5.866124e-04	1.250171e-03	6.512675e-04	6.578123e-04	1.14170	3.84106	5.55871	5.72450	8.88328	8.250749e-04
12-HAS_RT1	IC-MS	1.048202e-03	8.558098e-04	1.002944e-03	8.303411e-04	9.181987e-04	1.078010e-03	9.937343e-04	8.415827e-04	9.645763e-04	8.427294e-04	8.187407e-04	8.631614e-04	7.894169e-04	7.207381e-04	8.648809e-04	9.205775e-04	9.623785e-04	1.006362e-03	9.115656e-04	9.252484e-04	1.044386e-03	8.08628	9.17852	9.05751	9.33871	9.76400	1.148506e-03
12-HAS_RT2	IC-MS	5.415091e-04	1.033184e-03	7.359794e-04	1.045775e-03	9.388959e-04	6.792259e-04	8.219675e-04	8.045911e-04	8.889710e-04	8.339905e-04	1.088253e-03	8.056162e-04	5.863378e-04	4.700229e-04	8.700090e-04	8.631310e-04	7.806862e-04	7.826376e-04	6.141878e-04	7.990328e-04	8.353419e-04	8.75589	1.25079	5.78677	3.95511	3.78818	4.336151e-04
16-Hydroxyhexadecanoic acid_RT1	IC-MS	2.444136e-04	2.163156e-04	2.615415e-04	2.534055e-04	2.791344e-04	2.446491e-04	3.297667e-04	2.918470e-04	2.780759e-04	2.499784e-04	3.189576e-04	3.512422e-04	2.688508e-04	2.950857e-04	2.717511e-04	2.787432e-04	2.699191e-04	4.497210e-04	1.876318e-04	2.519554e-04	2.285719e-04	1.88350	2.37569	2.31956	1.78414	3.34389	1.881698e-04
16-Hydroxyhexadecanoic acid_RT2	IC-MS	9.897619e-05	9.546196e-05	9.216309e-05	1.347173e-04	1.191701e-04	1.111644e-04	1.490741e-04	1.348561e-04	1.341522e-04	1.102695e-04	1.261328e-04	1.015007e-04	1.203386e-04	1.233872e-04	1.121846e-04	1.942031e-04	2.007696e-04	1.963386e-04	1.486867e-04	1.537733e-04	1.312722e-04	1.00133	1.11031	9.62083	6.52867	5.68670	5.737376e-05
2,3,-DIHYDROXYBENZOYLSERINE	IC-MS	9.111769e-05	1.130843e-04	1.179262e-04	1.223466e-04	1.235927e-04	1.143453e-04	1.389504e-04	1.342131e-04	1.284218e-04	1.139387e-04	1.454242e-04	1.274979e-04	1.429768e-04	1.391289e-04	1.329580e-04	5.403760e-05	4.887635e-05	6.141253e-05	3.385941e-05	3.869597e-05	3.767300e-05	3.52706	3.02346	3.84860	2.69978	2.10832	1.444895e-05
2,4,6-Octatriyn-1-ol	IC-MS	1.115996e-04	1.346556e-04	1.373402e-04	1.429681e-04	1.278544e-04	1.381298e-04	2.123021e-04	1.915265e-04	1.620156e-04	1.233919e-04	1.554201e-04	1.336670e-04	1.910508e-04	2.132415e-04	1.898816e-04	1.594463e-04	1.813160e-04	1.730908e-04	1.705483e-04	1.709409e-04	1.751379e-04	2.08542	1.88305	1.91477	1.93148	1.84544	1.376049e-04
2,6-Diamino-4-methyleneheptanedioic acid	IC-MS	4.430942e-05	5.799065e-05	5.363259e-05	8.231398e-05	6.649613e-05	8.178558e-05	1.668274e-04	1.229269e-04	8.862914e-05	7.915291e-05	9.731557e-05	9.834002e-05	1.395172e-04	1.164043e-04	1.036179e-04	1.044511e-04	1.233613e-04	1.168087e-04	1.440492e-04	1.317913e-04	1.031249e-04	1.06529	1.05749	9.87091	1.03502	1.19488	5.889213e-05
2,6-dibromohydroquinone	IC-MS	3.603084e-05	4.208736e-05	4.763853e-05	4.783839e-05	5.089835e-05	4.537178e-05	4.941854e-05	5.128112e-05	4.883809e-05	4.901961e-05	5.095859e-05	5.087258e-05	4.828411e-05	5.080611e-05	5.202615e-05	5.033237e-05	4.815080e-05	4.791265e-05	5.121147e-05	5.138494e-05	5.234061e-05	6.02493	5.27091	5.67461	5.69050	5.49858	5.734620e-05
2-(3-phenylpropyl)oxolane	IC-MS	1.159735e-03	9.080519e-04	1.101709e-03	8.011721e-04	1.353075e-03	1.217509e-03	5.925668e-04	1.211927e-03	7.087188e-04	7.799794e-04	7.090093e-04	1.119314e-03	8.943931e-04	9.077136e-04	8.486844e-04	7.268647e-04	1.166659e-03	1.148266e-03	7.419468e-04	7.808174e-04	9.259137e-04	7.69904	8.80795	1.11532	1.08723	3.13252	1.311110e-03
2-Acetamido-2-deoxy-D-gluconolactone	IC-MS	4.587077e-05	4.997880e-05	4.700176e-05	4.091237e-05	4.414599e-05	3.559270e-05	6.780761e-05	7.093791e-05	6.239772e-05	3.723300e-05	5.116311e-05	3.908298e-05	5.888812e-05	6.989390e-05	5.696476e-05	7.486660e-05	7.933377e-05	8.187524e-05	5.410708e-05	5.691077e-05	5.771513e-05	6.27677	5.23103	5.64030	7.72096	6.13421	6.576868e-05
2-Amino-4-[(3-aminopropyl)amino]butanoic acid	IC-MS	3.558291e-04	3.837401e-04	3.756853e-04	3.025587e-04	2.923525e-04	2.640991e-04	2.785151e-04	3.275970e-04	2.515321e-04	2.552240e-04	2.988706e-04	2.763543e-04	3.184630e-04	3.594015e-04	2.746420e-04	2.579695e-04	2.638790e-04	2.732645e-04	2.048577e-04	2.118989e-04	2.117782e-04	2.99414	2.42575	2.77426	2.45781	2.81840	2.325424e-04
2-Aminoethyl (2R)-3-[(1Z)-1-hexadecen-1-yloxy]-2-hydro	IC-MS	1.395214e-04	1.405570e-04	1.383183e-04	2.514598e-04	2.125604e-04	2.191496e-04	3.451396e-04	2.879312e-04	2.333474e-04	1.675937e-04	1.926953e-04	1.737157e-04	1.961033e-04	2.158747e-04	1.659499e-04	2.360296e-04	2.427858e-04	2.720317e-04	1.880010e-04	2.146704e-04	1.805648e-04	9.06144	9.03761	8.62612	6.11914	5.97711	4.154075e-05
2-Isobutyl-3-methylpyrazine	IC-MS	4.720669e-04	4.315473e-04	4.547660e-04	3.741185e-04	3.713134e-04	4.214703e-04	3.463940e-04	3.490906e-04	3.414243e-04	4.032854e-04	3.861029e-04	4.434595e-04	3.278849e-04	3.399312e-04	3.300593e-04	3.823728e-04	3.702640e-04	3.672446e-04	4.080601e-04	3.786181e-04	4.174399e-04	4.35434	4.54304	4.49138	4.28477	4.77220	4.744715e-04
2-Methyl-4-quinolinol	IC-MS	2.947768e-04	3.361148e-04	3.424878e-04	2.814288e-04	2.104509e-04	2.401084e-04	3.404669e-04	2.742992e-04	2.506887e-04	2.544380e-04	2.672233e-04	2.728218e-04	2.494404e-04	2.475448e-04	2.427846e-04	2.389653e-04	2.708861e-04	2.453442e-04	2.437754e-04	2.377104e-04	2.494155e-04	2.69042	2.06914	2.02097	1.73567	2.00422	1.371211e-04
2-methylbutyrylcarnitine_RT1	IC-MS	6.403664e-04	7.202612e-04	8.041217e-04	8.372602e-04	9.499821e-04	8.086914e-04	1.379015e-03	1.574289e-03	1.268348e-03	5.244371e-04	6.113757e-04	5.277044e-04	7.658822e-04	9.152358e-04	7.772114e-04	2.788509e-05	2.256493e-05	2.522915e-05	1.941942e-05	1.886056e-05	1.776971e-05	1.35794	9.02503	1.17890	1.01459	1.05284	6.412491e-06
2-methylbutyrylcarnitine_RT2	IC-MS	6.778835e-04	8.643188e-04	9.521812e-04	9.173856e-04	1.052333e-03	8.582979e-04	1.274013e-03	1.407401e-03	9.733919e-04	5.238232e-04	7.105777e-04	5.678445e-04	6.147319e-04	7.113417e-04	5.840652e-04	1.139902e-05	1.362757e-05	1.524139e-05	7.783380e-06	7.288425e-06	8.405552e-06	1.13546	8.66791	7.21924	8.19893	8.50025	5.823934e-06
2-Naphthalenesulfonic acid	IC-MS	4.180920e-04	4.357900e-04	4.971616e-04	3.859015e-04	3.619022e-04	4.421738e-04	3.612843e-04	3.524216e-04	4.998015e-04	1.540590e-04	1.304313e-04	3.555658e-04	3.513986e-04	2.554838e-04	3.267175e-04	3.049026e-04	2.744102e-04	6.104939e-04	5.199633e-04	1.935568e-04	3.328434e-04	9.49683	4.00461	3.20034	5.05399	4.11915	5.906392e-04
2-oleoyl-sn-glycero-3-phosphocholine	IC-MS	1.552386e-04	1.663684e-04	1.684329e-04	3.573964e-04	2.790823e-04	2.413883e-04	5.210167e-04	4.406183e-04	3.658436e-04	2.159171e-04	2.817517e-04	2.588701e-04	3.182001e-04	2.917752e-04	2.542177e-04	3.079235e-04	3.902560e-04	3.673667e-04	2.973284e-04	3.886359e-04	2.868884e-04	1.59380	1.59822	1.39766	1.10692	1.08375	8.092627e-05
2-palmitoyl-sn-glycero-3-phosphocholine	IC-MS	7.875343e-04	7.375953e-04	8.403601e-04	1.210400e-03	1.045316e-03	9.569905e-04	1.369978e-03	1.223390e-03	9.889497e-04	8.211906e-04	1.022285e-03	8.170622e-04	8.489150e-04	8.801715e-04	6.300843e-04	1.282863e-03	1.486894e-03	1.318580e-03	1.077662e-03	1.104577e-03	9.539551e-04	5.81887	5.74751	4.24328	5.15631	3.70696	3.332394e-04
3,4-dihydroxybutyric acid	IC-MS	6.036719e-04	6.468534e-04	5.458794e-04	3.845274e-04	2.911416e-04	4.048501e-04	1.895461e-04	1.587261e-04	2.036961e-04	3.814736e-04	2.483248e-04	3.114981e-04	1.823463e-04	1.559632e-04	1.942719e-04	2.936075e-04	1.868362e-04	1.505852e-04	1.531105e-04	1.582649e-04	1.508672e-04	3.01493	2.31579	2.50811	2.57631	2.58765	1.612698e-04
3-Hydroxy-1-phenyl-1-icosanone	IC-MS	4.665146e-05	3.791694e-05	4.357910e-05	1.288080e-04	1.078152e-04	1.549375e-04	2.810139e-04	2.072886e-04	1.923242e-04	1.667370e-04	1.463244e-04	1.610608e-04	2.640328e-04	2.858112e-04	2.194587e-04	2.191558e-04	2.655065e-04	2.463174e-04	4.442864e-04	5.643314e-04	3.804295e-04	1.65313	1.91879	1.64376	2.76498	2.14628	1.671672e-04
3-Methylhistidine	IC-MS	1.150599e-04	1.480056e-04	1.380506e-04	1.534252e-04	1.325843e-04	1.341696e-04	3.390018e-04	3.027981e-04	2.940959e-04	1.293421e-04	1.556328e-04	1.427323e-04	3.563148e-04	4.322143e-04	3.821989e-04	1.351268e-04	1.440620e-04	1.363548e-04	1.655998e-04	1.680187e-04	1.777908e-04	2.89707	2.42302	2.76997	3.04070	3.07234	2.711268e-04
3-Methylsulfolene	IC-MS	1.694314e-04	2.054654e-04	2.041769e-04	1.925470e-04	1.584600e-04	1.724096e-04	2.456852e-04	2.208071e-04	1.891407e-04	1.642817e-04	1.743480e-04	1.754999e-04	2.384842e-04	2.370620e-04	2.038988e-04	2.263761e-04	2.614678e-04	2.409625e-04	2.570857e-04	2.697779e-04	2.562721e-04	3.28340	2.79549	3.15858	3.09677	2.78170	2.386813e-04
3-Phenylpropyl hydrogen sulfate	IC-MS	3.767800e-03	3.526502e-03	3.553939e-03	3.168198e-03	3.464773e-03	3.443970e-03	2.578612e-03	2.775178e-03	2.984965e-03	3.267378e-03	2.831846e-03	2.993790e-03	3.965370e-03	3.727348e-03	4.066322e-03	2.947269e-03	3.067816e-03	3.133801e-03	3.536320e-03	3.743565e-03	4.550672e-03	3.34272	3.53251	3.51316	5.32997	5.32560	5.968210e-03
4-Dodecylbenzenesulfonic acid	IC-MS	3.813806e-03	3.861288e-03	5.773046e-03	2.124418e-03	7.216074e-03	4.426728e-03	3.111247e-03	3.294559e-03	4.567581e-03	1.685757e-03	9.812740e-03	4.549362e-03	3.661896e-03	2.921233e-03	2.929959e-03	2.553048e-03	3.725783e-03	5.951503e-03	4.737773e-03	2.435419e-03	3.390600e-03	5.69373	2.25554	1.87186	3.43431	4.04252	4.876241e-03
4-guanidinobutanal	IC-MS	1.323774e-03	1.544932e-03	1.461312e-03	1.315118e-03	1.078124e-03	1.175751e-03	1.505646e-03	1.416222e-03	1.280733e-03	1.167132e-03	1.163790e-03	1.207312e-03	1.395649e-03	1.440280e-03	1.358040e-03	1.256191e-03	1.337380e-03	1.265854e-03	1.350667e-03	1.456397e-03	1.350155e-03	1.52119	1.36490	1.38438	1.28820	1.41333	1.138612e-03
4-Hydroxy-2-oxoheptanedioic acid	IC-MS	1.041864e-04	1.077671e-04	1.126875e-04	8.900973e-05	9.692518e-05	8.920220e-05	7.735410e-05	7.697297e-05	7.352075e-05	9.361029e-05	7.735940e-05	8.477660e-05	7.224500e-05	7.258807e-05	7.726396e-05	7.744036e-05	8.199448e-05	9.015359e-05	8.523007e-05	8.330724e-05	8.160061e-05	9.55753	8.22195	8.07424	8.76395	7.10994	9.476834e-05
4-Oxoproline_RT1	IC-MS	1.057150e-03	1.210046e-03	1.323023e-03	1.262507e-03	1.259919e-03	1.088792e-03	1.266893e-03	1.333432e-03	1.129722e-03	1.199169e-03	1.338087e-03	1.196887e-03	1.255650e-03	1.303737e-03	1.159997e-03	9.495389e-04	8.243358e-04	8.799462e-04	6.130974e-04	6.572275e-04	6.565035e-04	1.28348	1.22286	1.40333	1.14104	1.10943	1.126740e-03
4-Oxoproline_RT2	IC-MS	1.978344e-04	2.016729e-04	2.079176e-04	1.759529e-04	1.840094e-04	1.467524e-04	2.003649e-04	2.251470e-04	1.798302e-04	1.551460e-04	2.234308e-04	1.712656e-04	1.980562e-04	2.261075e-04	1.914950e-04	4.266193e-04	4.276003e-04	4.472632e-04	2.047578e-04	2.326867e-04	2.177841e-04	5.21176	4.10929	5.52851	5.53676	4.89278	5.335329e-04
4-Oxoproline_RT3	IC-MS	2.187650e-03	2.016964e-03	1.876341e-03	1.961590e-03	2.053037e-03	1.946873e-03	1.733900e-03	1.853100e-03	2.296876e-03	1.815560e-03	1.741458e-03	1.816058e-03	2.097125e-03	2.000090e-03	2.172868e-03	2.016432e-03	1.636911e-03	1.873514e-03	2.441176e-03	2.596498e-03	2.307637e-03	2.33144	2.97699	2.98214	3.64563	3.75640	4.090167e-03
4-tert-Octylphenol	IC-MS	1.323595e-03	1.319089e-03	1.597579e-03	1.183698e-03	1.133026e-03	1.376219e-03	8.052109e-04	1.132131e-03	1.525914e-03	1.008197e-03	1.174690e-03	1.476520e-03	1.047394e-03	1.023293e-03	1.411935e-03	1.169866e-03	1.311334e-03	1.241577e-03	1.424054e-03	1.169032e-03	1.407333e-03	1.38953	1.38035	1.63153	1.69059	1.88001	2.209392e-03
4-Undecylbenzenesulfonic acid	IC-MS	3.377567e-03	3.522620e-03	5.506088e-03	2.084551e-03	6.079370e-03	4.112748e-03	3.522380e-03	2.998007e-03	4.023494e-03	1.413091e-03	8.696013e-03	3.957063e-03	3.189263e-03	2.592726e-03	2.662672e-03	2.246835e-03	3.279393e-03	5.103018e-03	4.217922e-03	2.148055e-03	3.264861e-03	4.80109	2.05943	1.73785	3.20224	3.94035	4.141147e-03
5-Aminovaleric acid	IC-MS	8.203624e-04	9.082505e-04	8.857650e-04	7.623194e-04	6.826464e-04	7.038800e-04	8.618515e-04	7.965855e-04	7.059496e-04	6.751709e-04	7.733993e-04	7.221654e-04	8.780400e-04	8.112167e-04	7.701703e-04	9.208036e-04	1.045273e-03	1.018889e-03	1.083290e-03	1.251934e-03	1.125437e-03	1.27571	1.16465	1.28166	1.31061	1.22732	1.140805e-03
5-Butyl-2-ethyloxazole	IC-MS	1.180450e-04	5.946357e-05	1.278742e-04	1.300348e-04	1.537658e-04	8.746306e-04	5.363438e-04	3.670629e-04	7.795779e-04	1.015384e-04	2.614152e-04	1.633138e-04	9.073619e-04	4.670837e-04	9.265595e-04	2.379715e-04	1.413316e-04	1.796710e-04	9.157900e-04	9.709967e-04	8.798485e-04	3.08591	2.27745	2.47211	1.35836	6.13682	4.950670e-04
5-Methyl-2-octyl-3(2H)-furanone	IC-MS	3.671183e-04	2.635889e-04	3.980408e-04	2.195912e-04	3.813106e-04	3.551829e-04	1.437306e-04	3.096972e-04	1.964392e-04	1.883615e-04	1.848413e-04	3.498057e-04	2.308278e-04	2.295534e-04	2.157040e-04	1.973695e-04	3.320093e-04	3.161205e-04	2.003113e-04	1.997358e-04	2.413182e-04	1.69417	2.25203	3.02099	2.64748	8.80798	3.281165e-04
5beta-cholanoic acid	IC-MS	1.599927e-04	1.159695e-04	1.406374e-04	2.758760e-04	2.286358e-04	3.114653e-04	4.667811e-04	3.504009e-04	3.254552e-04	3.094826e-04	2.981936e-04	3.102225e-04	4.220918e-04	4.265468e-04	3.447400e-04	4.960293e-04	6.013779e-04	5.439915e-04	8.207065e-04	1.031674e-03	7.114547e-04	4.10134	4.91169	4.18795	6.27004	5.25625	4.318809e-04
6-Hydroxy-1a,5,7a-trimethyl-2,2a,6,6a,7a,8,9,9a-octahyd	IC-MS	2.391559e-03	3.978895e-03	2.359573e-03	3.418849e-03	2.881828e-03	2.454461e-03	2.580194e-03	2.861149e-03	2.569593e-03	2.760184e-03	1.960948e-03	4.893830e-03	2.153012e-03	3.163287e-03	2.757841e-03	3.432191e-03	3.360037e-03	3.455096e-03	5.077768e-03	2.931635e-03	3.102100e-03	4.56497	2.70941	3.12610	2.77289	4.02103	3.862884e-03
6-Oxohexanoic acid	IC-MS	2.799576e-03	3.003657e-03	3.005894e-03	4.254641e-03	3.122220e-03	3.731028e-03	4.195885e-03	3.619636e-03	4.756093e-03	3.485178e-03	2.755133e-03	3.367838e-03	4.729723e-03	4.757242e-03	4.699610e-03	3.922756e-03	3.709108e-03	3.973130e-03	1.990542e-03	1.919456e-03	2.141767e-03	5.03203	3.35060	3.38378	2.32387	3.87807	2.846392e-03
8Z,11Z,14Z-Eicosatrienoic acid	IC-MS	9.256457e-04	7.688229e-04	6.767929e-04	2.085179e-03	1.662277e-03	1.957010e-03	2.218492e-03	1.846598e-03	1.739231e-03	1.775688e-03	1.773319e-03	1.609698e-03	1.661889e-03	1.530918e-03	1.405384e-03	2.761253e-03	3.240021e-03	2.783477e-03	2.983996e-03	3.385463e-03	2.736141e-03	2.07913	2.79551	2.34266	2.42020	1.87485	1.572665e-03
Linolenic Acid	IC-MS	1.875091e-04	1.927019e-04	1.938912e-04	3.167150e-04	2.081772e-04	2.639560e-04	3.563207e-04	3.083840e-04	3.433351e-04	2.950568e-04	2.783763e-04	2.550923e-04	3.039633e-04	3.012217e-04	2.858189e-04	4.612183e-04	5.657982e-04	4.851968e-04	6.754812e-04	7.701562e-04	6.575925e-04	4.94418	5.69126	4.86303	6.17811	5.69118	4.048566e-04
ACETONE CYANOHYDRIN	IC-MS	5.296806e-04	6.170541e-04	5.783931e-04	5.494648e-04	5.458280e-04	4.858049e-04	1.519948e-03	1.462673e-03	1.391270e-03	6.622332e-04	7.113773e-04	7.077796e-04	1.739091e-03	2.140661e-03	1.772360e-03	4.844156e-04	4.750011e-04	5.095884e-04	4.155285e-04	4.822109e-04	4.726950e-04	1.01014	8.22678	9.46035	9.56657	7.31043	7.602872e-04
Acetyl-L-carnitine	IC-MS	1.996853e-03	2.144118e-03	2.264551e-03	4.327223e-03	4.186559e-03	3.724749e-03	6.636457e-03	6.966214e-03	5.369664e-03	2.449807e-03	2.641341e-03	2.640392e-03	3.481570e-03	3.938132e-03	3.473576e-03	5.355750e-03	5.325792e-03	5.849065e-03	1.130496e-02	1.054092e-02	1.109267e-02	3.48645	3.27197	3.59040	3.38531	2.92195	3.567821e-03
Acetylcholine	IC-MS	1.468179e-03	1.676984e-03	1.827099e-03	1.501622e-03	1.598874e-03	1.309405e-03	2.578594e-03	2.720669e-03	2.207638e-03	1.110581e-03	1.445696e-03	1.172031e-03	1.729910e-03	2.015998e-03	1.713077e-03	1.567021e-03	1.542506e-03	1.647685e-03	1.844654e-03	1.856840e-03	1.920742e-03	1.13755	9.84560	1.12877	1.34085	1.18542	1.190139e-03
acetylserine	IC-MS	2.648419e-03	2.938778e-03	3.367863e-03	3.630303e-03	3.237949e-03	3.272048e-03	5.543706e-03	5.391008e-03	4.521383e-03	3.354211e-03	3.700030e-03	3.681204e-03	5.166852e-03	4.993016e-03	5.075051e-03	4.311334e-03	4.350289e-03	4.870302e-03	4.872231e-03	4.679320e-03	4.969643e-03	5.16567	4.08547	4.33026	5.34297	5.46655	4.874564e-03
Adenosine	IC-MS	1.065507e-03	9.609845e-04	9.367062e-04	1.036286e-03	9.158457e-04	1.029817e-03	8.248384e-04	7.800178e-04	9.425016e-04	1.151525e-03	1.009811e-03	9.720824e-04	6.346764e-04	9.245749e-04	5.834477e-04	1.147913e-03	1.017060e-03	1.033149e-03	7.186237e-04	6.075079e-04	4.772913e-04	1.04523	9.75118	8.57400	5.45313	5.47248	5.907466e-04
Allantoin	IC-MS	1.431094e-04	1.439352e-04	1.350346e-04	1.159966e-04	8.600533e-05	9.495856e-05	1.327483e-04	1.153800e-04	9.054231e-05	9.276476e-05	1.124654e-04	1.104238e-04	9.892408e-05	9.731271e-05	9.247269e-05	1.050182e-04	1.154899e-04	9.927847e-05	1.110123e-04	1.021833e-04	1.104648e-04	1.20382	8.20691	8.49884	7.39276	8.05541	5.643733e-05
alpha-methylstyrene_RT1	IC-MS	6.402828e-03	1.084292e-02	6.711135e-03	9.865198e-03	8.471025e-03	7.465051e-03	8.639766e-03	9.776564e-03	8.577438e-03	8.997619e-03	6.540820e-03	1.610322e-02	8.574996e-03	1.199013e-02	1.085383e-02	1.036570e-02	1.055473e-02	1.082435e-02	1.715654e-02	9.924997e-03	1.058839e-02	1.48454	9.04118	1.05321	1.10188	1.60694	1.489305e-02
alpha-methylstyrene_RT2	IC-MS	1.367828e-04	3.377560e-04	1.242853e-04	2.668734e-04	2.251920e-04	1.902458e-04	2.426898e-04	2.434926e-04	1.939671e-04	2.208830e-04	1.179324e-04	5.510686e-04	9.496522e-05	2.386517e-04	2.394697e-04	2.706209e-04	2.895370e-04	3.100200e-04	5.650180e-04	2.517331e-04	2.587396e-04	5.27505	1.56833	2.96498	2.72654	4.30137	3.198821e-04
Arabinosylhypoxanthine	IC-MS	3.198766e-03	3.606438e-03	3.475072e-03	2.669240e-03	3.007428e-03	2.560945e-03	2.620423e-03	2.943042e-03	2.620057e-03	2.814890e-03	3.246471e-03	2.821729e-03	2.798089e-03	3.142949e-03	2.472103e-03	2.225579e-03	2.090054e-03	2.099956e-03	1.544544e-03	1.754032e-03	1.511525e-03	2.50770	2.52971	2.73809	2.12895	1.99500	2.069549e-03
Arachidic Acid_RT1	IC-MS	2.811738e-03	2.478167e-03	2.618409e-03	2.151671e-03	2.323910e-03	2.569240e-03	1.616072e-03	2.019247e-03	2.304249e-03	2.348956e-03	2.231657e-03	2.274600e-03	2.068444e-03	1.870984e-03	2.198852e-03	2.367076e-03	2.059011e-03	2.101254e-03	2.440840e-03	2.391306e-03	2.664001e-03	2.29439	2.60240	2.73243	2.70340	2.85659	3.214738e-03
Arachidic Acid_RT2	IC-MS	7.824421e-04	7.641353e-04	8.426511e-04	8.446605e-04	7.912842e-04	8.720757e-04	7.919163e-04	7.275043e-04	7.404074e-04	8.212443e-04	7.491175e-04	8.164219e-04	7.494421e-04	6.711652e-04	7.407185e-04	8.520301e-04	9.480139e-04	8.911786e-04	8.332732e-04	7.627531e-04	8.128292e-04	8.11537	7.68574	7.82217	7.42612	8.02839	7.477424e-04
Arachidonic acid	IC-MS	2.586495e-03	2.134995e-03	1.944110e-03	5.901584e-03	5.068578e-03	6.290863e-03	7.136267e-03	5.828473e-03	5.537274e-03	6.229191e-03	6.153626e-03	5.043192e-03	5.367222e-03	5.181586e-03	4.471602e-03	9.953734e-03	1.149564e-02	1.016713e-02	1.122653e-02	1.224226e-02	1.030470e-02	8.40545	1.10988	9.24582	9.16646	7.39248	5.864113e-03
Argininosuccinic acid	IC-MS	1.423821e-04	7.442516e-05	6.869203e-05	7.040596e-05	6.240579e-05	5.184294e-05	1.189307e-04	1.233649e-04	9.121740e-05	5.655410e-05	7.965259e-05	6.934611e-05	1.000337e-04	1.169061e-04	9.209297e-05	8.246474e-05	7.793074e-05	7.391504e-05	2.103600e-05	1.739341e-05	1.953525e-05	9.88885	7.67887	1.08645	6.08894	5.47392	5.070123e-05
asp-ser	IC-MS	3.829088e-05	4.567132e-05	4.408660e-05	7.855682e-05	6.996512e-05	7.611359e-05	1.016155e-04	8.453999e-05	8.601916e-05	6.222640e-05	7.057950e-05	6.995704e-05	9.714130e-05	8.486952e-05	7.545798e-05	8.974466e-05	9.253300e-05	9.276836e-05	9.333657e-05	1.139038e-04	8.648719e-05	6.16685	7.22283	7.25208	7.28181	5.89054	4.103611e-05
Asparagine	IC-MS	5.437622e-04	5.573686e-04	5.680124e-04	5.273209e-04	5.134402e-04	4.511195e-04	9.811364e-04	8.166565e-04	8.481233e-04	5.014887e-04	5.217007e-04	5.125868e-04	1.015053e-03	1.057818e-03	1.014080e-03	4.852766e-04	4.619292e-04	4.834066e-04	4.680983e-04	4.887122e-04	5.221450e-04	8.38369	7.44392	8.96536	8.11393	7.54924	7.592688e-04

Benzylideneacetone	IC-MS	1.255878e-03	1.109341e-03	1.203605e-03	1.141730e-03	1.080521e-03	8.671825e-04	1.390356e-04	1.284530e-04	1.305966e-04	5.563832e-04	4.508359e-04	3.939490e-04	1.207992e-02	1.076194e-02	1.098736e-02	6.975497e-04	5.600682e-04	5.422888e-04	1.314743e-04	1.169334e-04	1.003410e-03	2.93737	3.29964	2.42874	1.46120	1.39806	1.483438e-02
beta-Aspartylaspartic acid	IC-MS	6.741826e-05	6.631691e-05	7.243469e-05	7.397103e-05	7.951154e-05	7.336079e-05	8.151383e-05	7.763203e-05	8.966978e-05	8.127839e-05	7.909592e-05	8.079954e-05	9.450758e-05	8.993969e-05	9.513496e-05	8.573681e-05	7.632441e-05	9.833363e-05	5.966614e-05	6.510169e-05	6.627434e-05	1.11115	1.07030	1.34617	1.24381	9.92441	1.001479e-04
beta-D-Mannopyranose	IC-MS	9.166377e-04	1.084141e-03	1.125671e-03	9.386593e-04	9.687846e-04	8.515258e-04	1.281723e-03	1.370379e-03	1.163269e-03	1.236695e-03	1.552261e-03	1.289416e-03	1.707070e-03	1.878264e-03	1.680424e-03	7.713143e-04	8.137392e-04	7.905343e-04	7.490215e-04	7.757203e-04	7.679706e-04	1.45368	1.25860	1.41021	1.36059	1.23143	1.297009e-03
Betaine	IC-MS	1.268832e-02	1.444896e-02	1.550367e-02	1.274747e-02	1.362206e-02	1.259986e-02	1.659182e-02	1.941265e-02	1.548925e-02	1.490983e-02	1.667758e-02	1.568814e-02	1.899098e-02	2.268620e-02	1.660191e-02	1.285967e-02	1.343846e-02	1.400085e-02	1.231582e-02	1.322023e-02	1.343165e-02	1.68811	1.41476	1.57421	1.49332	1.71892	1.435897e-02
Caprolactam	IC-MS	1.396204e-03	1.329954e-03	1.293340e-03	1.265413e-03	1.467512e-03	2.255142e-03	1.170554e-03	1.095547e-03	1.430673e-03	1.183566e-03	1.064626e-03	1.050232e-03	1.230830e-03	1.172121e-03	1.187464e-03	1.290503e-03	1.074464e-03	1.245616e-03	1.495278e-03	1.428516e-03	1.282497e-03	1.39205	1.37403	1.41766	1.85958	1.54821	1.952639e-03
Caprylic acid	IC-MS	2.202585e-04	2.255445e-04	2.326828e-04	1.764494e-04	1.841038e-04	1.250250e-04	1.674694e-04	1.626419e-04	1.604139e-04	1.553853e-04	1.712277e-04	1.445276e-04	1.607071e-04	1.488080e-04	1.646962e-04	1.850836e-04	1.742206e-04	1.498450e-04	1.658959e-04	1.509131e-04	1.629641e-04	1.88394	1.52452	1.61281	1.61991	1.51907	1.756791e-04
capuride	IC-MS	2.125101e-04	2.594018e-04	2.178963e-04	2.269080e-04	4.996080e-04	5.303701e-04	4.102417e-04	3.637935e-04	3.899595e-04	2.235070e-04	2.197107e-04	3.038919e-04	3.331078e-04	3.211400e-04	3.929975e-04	2.472296e-04	1.884085e-04	1.697128e-04	3.767355e-04	3.527369e-04	4.088604e-04	2.62126	2.36891	5.09099	5.43306	5.11394	5.799678e-04
carglumic acid	IC-MS	9.651610e-05	1.143651e-04	1.261901e-04	1.183093e-04	1.194085e-04	1.015119e-04	1.582473e-04	1.525573e-04	1.337785e-04	9.888674e-05	1.316929e-04	1.060997e-04	1.254334e-04	1.442917e-04	1.357205e-04	1.247841e-04	1.362771e-04	1.358553e-04	7.821196e-05	8.120041e-05	8.374234e-05	1.41372	1.11786	1.38492	1.11734	1.00917	1.026907e-04
CHLORIC ACID	IC-MS	1.798429e-04	2.171405e-04	3.346302e-04	1.535431e-04	9.439490e-04	1.915084e-04	1.345837e-04	1.213193e-04	2.636869e-04	7.326792e-05	6.263680e-05	1.111652e-04	1.471396e-04	7.774471e-05	2.801185e-04	1.299613e-04	8.284728e-05	2.157586e-04	2.120475e-04	6.490091e-05	1.653361e-04	5.04494	1.59824	1.32660	1.44373	1.25766	2.393794e-04
Cholesterol sulfate	IC-MS	1.141491e-03	1.080212e-03	8.799962e-04	1.618760e-03	1.080723e-03	1.334946e-03	1.423759e-03	1.249144e-03	1.233795e-03	1.405274e-03	1.590256e-03	1.242580e-03	1.089540e-03	1.254240e-03	1.174999e-03	1.835255e-03	1.800035e-03	1.650476e-03	1.526524e-03	1.923931e-03	1.343903e-03	1.41169	1.63060	1.55876	1.26606	1.00991	1.136934e-03
cis-4-Hydroxy-D-proline	IC-MS	7.301190e-04	4.834979e-04	8.356645e-04	7.324528e-04	6.806546e-04	5.792378e-04	2.180644e-03	2.032332e-03	2.011760e-03	9.426537e-04	1.035630e-03	1.056284e-03	3.124477e-03	2.533531e-03	2.722481e-03	6.228157e-04	8.689798e-04	6.523637e-04	1.214120e-03	6.442823e-04	6.159459e-04	1.62479	1.25853	1.82624	1.39445	1.86803	1.706599e-03
Creatine	IC-MS	2.312346e-02	2.951691e-02	3.351121e-02	3.127423e-02	3.173771e-02	2.815574e-02	4.770800e-02	4.977079e-02	4.230173e-02	3.735302e-02	4.136341e-02	3.624700e-02	5.641169e-02	6.367095e-02	5.733294e-02	2.842028e-02	2.943361e-02	3.247171e-02	4.015756e-02	4.542674e-02	4.690996e-02	3.18396	2.79199	3.36553	4.51058	3.56551	3.948956e-02
Creatinine	IC-MS	4.343628e-04	5.053233e-04	5.121673e-04	4.196533e-04	3.491611e-04	3.631920e-04	4.923710e-04	4.468278e-04	3.776583e-04	4.114917e-04	4.328060e-04	4.498404e-04	4.025181e-04	3.920977e-04	3.913790e-04	3.686231e-04	4.112910e-04	3.693974e-04	3.833511e-04	3.453623e-04	3.603239e-04	4.74973	3.37926	3.46691	3.18845	3.69803	2.720413e-04
Crotonic acid	IC-MS	2.653335e-04	2.942924e-04	2.209728e-04	2.526091e-04	6.504126e-05	2.266602e-04	2.016361e-04	1.457687e-04	1.371478e-04	2.072694e-04	1.772063e-04	1.943886e-04	1.610706e-04	2.452682e-04	1.623866e-04	1.981787e-04	1.682131e-04	2.182604e-04	1.618874e-04	1.625344e-04	1.987292e-04	2.31877	2.33541	2.35903	2.07271	2.38237	2.098761e-04
Cytarabine	IC-MS	5.120688e-04	6.153591e-04	6.266795e-04	5.889277e-04	5.576179e-04	5.100050e-04	5.989517e-04	6.096222e-04	5.805079e-04	5.825772e-04	6.255498e-04	5.017686e-04	5.761999e-04	6.236789e-04	5.625859e-04	5.137190e-04	4.691582e-04	5.021655e-04	3.857567e-04	4.071559e-04	3.237114e-04	4.76531	4.33399	4.62821	3.52860	3.20844	3.461746e-04
Cytosine_RT1	IC-MS	1.612034e-03	1.878602e-03	1.918206e-03	1.878878e-03	1.880218e-03	1.618401e-03	2.086482e-03	2.119883e-03	1.916469e-03	1.908197e-03	2.099771e-03	1.723033e-03	2.003231e-03	2.239349e-03	1.903021e-03	1.684609e-03	1.683667e-03	1.729429e-03	1.317878e-03	1.373315e-03	1.270189e-03	1.59403	1.47490	1.52386	1.32225	1.10163	1.415916e-03
Cytosine_RT2	IC-MS	1.292602e-04	1.491206e-04	1.202056e-04	1.816981e-04	1.650666e-04	1.706226e-04	2.456241e-04	1.942323e-04	2.002991e-04	2.035081e-04	2.247653e-04	1.862562e-04	2.461549e-04	2.087359e-04	2.270737e-04	2.365296e-04	2.875773e-04	2.243485e-04	1.373310e-04	1.549665e-04	1.460066e-04	1.43663	2.00069	2.08391	1.50800	1.62062	2.239938e-04
D-(+)-Malic acid	IC-MS	6.776436e-03	7.830497e-03	7.558126e-03	8.865327e-03	7.752742e-03	6.959343e-03	1.091849e-02	1.064197e-02	8.587909e-03	7.081262e-03	1.012438e-02	8.197159e-03	9.877764e-03	9.351691e-03	8.923345e-03	7.978087e-03	7.894615e-03	7.960528e-03	2.749284e-03	3.084063e-03	3.043644e-03	6.41982	4.41396	5.04064	4.77388	3.78201	3.673245e-03
D-(+)-Octopine	IC-MS	1.472324e-05	2.168573e-05	5.324370e-05	5.157825e-05	5.119738e-05	5.649176e-05	4.376043e-04	4.627449e-04	4.250720e-04	7.757125e-05	9.692803e-05	7.830537e-05	4.698089e-04	6.055699e-04	4.872003e-04	2.362170e-04	2.664265e-04	3.013929e-04	7.501190e-04	7.673493e-04	7.495648e-04	3.78545	3.30232	3.90044	8.56167	7.68238	7.928885e-04
D-(+)-Proline	IC-MS	1.994590e-02	2.120586e-02	2.203780e-02	1.942923e-02	1.869232e-02	1.697762e-02	2.588031e-02	2.599802e-02	2.423799e-02	1.824093e-02	1.786107e-02	1.827057e-02	2.478453e-02	2.746678e-02	2.428974e-02	1.678785e-02	1.660476e-02	1.699228e-02	1.906739e-02	1.889506e-02	1.817292e-02	2.12043	1.94651	2.15647	2.02998	1.80150	2.008966e-02
D-(+)-Tryptophan	IC-MS	1.358514e-04	1.599161e-04	1.623272e-04	1.208200e-04	1.082791e-04	1.138324e-04	1.495966e-04	1.406409e-04	1.117469e-04	1.169109e-04	1.232707e-04	1.181648e-04	1.484454e-04	1.430325e-04	1.249018e-04	1.375328e-04	1.517460e-04	1.378689e-04	1.469336e-04	1.645563e-04	1.477616e-04	2.08235	1.88000	2.08688	2.08807	1.89061	1.676485e-04
D-(-)-3-Phosphoglyceric acid	IC-MS	1.815613e-04	2.705233e-04	2.577315e-04	1.972482e-04	2.242095e-04	1.511891e-04	2.671187e-04	2.678936e-04	2.232223e-04	1.770257e-04	2.435009e-04	1.772027e-04	2.392516e-04	2.735476e-04	2.173549e-04	1.773721e-04	1.908531e-04	1.926708e-04	1.776838e-04	1.974469e-04	1.558808e-04	2.31668	1.34620	1.60007	1.50401	1.13665	1.465810e-04
D-benzylpenicilloic acid	IC-MS	3.375304e-05	3.831671e-05	3.847140e-05	1.616282e-04	9.971062e-05	1.223239e-04	1.622249e-04	1.278650e-04	1.043159e-04	8.530881e-05	8.026335e-05	1.275985e-04	7.723347e-05	9.758496e-05	9.410493e-05	8.523355e-05	1.188000e-04	8.360183e-05	1.036088e-04	8.055934e-05	1.131985e-04	1.39794	7.81541	5.19813	5.74089	7.76698	4.173169e-05
D-Glucose 6-phosphate_RT1	IC-MS	4.267378e-04	5.456249e-04	5.205890e-04	5.570811e-04	5.310559e-04	4.725649e-04	6.148076e-04	6.619964e-04	5.875933e-04	4.750513e-04	5.572204e-04	4.950235e-04	6.057391e-04	5.535045e-04	5.909934e-04	4.213577e-04	4.730930e-04	5.823556e-04	5.423589e-04	6.333184e-04	5.187366e-04	4.66620	3.28603	3.53960	3.61210	3.21023	8.824227e-05
D-Glucose 6-phosphate_RT2	IC-MS	9.547487e-05	1.338908e-04	1.179058e-04	1.362145e-04	1.458255e-04	1.140410e-04	1.928227e-04	2.000910e-04	1.678403e-04	1.335773e-04	1.347670e-04	1.331612e-04	1.870799e-04	1.521398e-04	1.597350e-04	1.138513e-04	1.258434e-04	1.486054e-04	1.508225e-04	1.713392e-04	1.453618e-04	1.26079	8.25337	7.95400	8.30417	7.21757	2.317523e-05
D-PANTOTHENIC ACID	IC-MS	3.064282e-03	3.641677e-03	3.736275e-03	3.878694e-03	3.958315e-03	3.422374e-03	3.191893e-03	3.421772e-03	2.547097e-03	4.389626e-03	5.690940e-03	4.549563e-03	4.171808e-03	4.119844e-03	3.569650e-03	3.387779e-03	3.323634e-03	3.426777e-03	2.484206e-03	2.623674e-03	2.554658e-03	3.61833	3.15632	3.71253	3.10649	2.57030	2.634523e-03
D-Sedoheptulose 7-phosphate	IC-MS	3.481869e-04	4.058686e-04	3.781399e-04	4.828895e-04	4.831872e-04	4.491360e-04	4.879850e-04	4.007940e-04	4.808365e-04	5.201037e-04	4.798895e-04	4.945251e-04	4.476347e-04	3.786095e-04	4.476509e-04	5.808575e-04	7.398811e-04	5.941379e-04	6.921814e-04	6.989355e-04	6.705991e-04	5.40872	4.65094	5.01410	3.70168	3.61170	3.516828e-04
D-Xylulose 5-phosphate_RT1	IC-MS	1.586836e-04	1.990013e-04	1.044331e-04	1.533067e-04	1.648198e-04	9.420332e-05	6.560231e-05	1.898558e-05	3.875120e-05	9.921399e-05	8.957399e-05	1.384038e-04	1.221286e-04	1.182516e-04	1.133236e-04	1.844500e-04	1.914192e-04	1.988700e-04	1.255909e-04	6.794773e-05	5.789097e-05	1.22575	1.25752	1.51432	8.26855	1.15517	8.270479e-05
D-Xylulose 5-phosphate_RT2	IC-MS	5.738032e-04	5.849646e-04	5.821855e-04	5.611964e-04	5.602807e-04	5.804024e-04	4.403498e-04	4.969836e-04	5.301814e-04	5.069977e-04	5.471576e-04	5.912144e-04	4.930354e-04	5.210379e-04	4.961789e-04	6.182791e-04	5.978956e-04	6.047545e-04	4.049380e-04	4.406914e-04	4.594562e-04	7.06891	6.73156	7.64268	6.67251	6.22068	6.868593e-04
Decanoic acid	IC-MS	5.419735e-04	8.391385e-04	6.503718e-04	7.241634e-04	6.237571e-04	4.121855e-04	3.860735e-04	5.488834e-04	4.103628e-04	6.586096e-04	5.718170e-04	6.628600e-04	7.295532e-04	6.462507e-04	5.114164e-04	7.760102e-04	5.988662e-04	5.737022e-04	3.106999e-04	5.208377e-04	3.843393e-04	7.22994	3.51495	3.09207	5.02330	5.94006	3.195661e-04
Dimethyl sulfoxide	IC-MS	8.352481e-03	9.795356e-03	9.008301e-03	8.170191e-03	6.320004e-03	6.930838e-03	8.548656e-03	7.237846e-03	6.317000e-03	7.488085e-03	7.533107e-03	7.917184e-03	7.195044e-03	6.471635e-03	6.355490e-03	7.273530e-03	8.096734e-03	7.093156e-03	6.687425e-03	6.353396e-03	6.720735e-03	8.26627	5.89417	5.87587	4.94919	5.68892	4.147725e-03
DL-Alanine	IC-MS	4.177048e-04	4.634357e-04	5.201480e-04	5.714293e-04	5.319499e-04	4.770207e-04	1.679262e-03	1.528636e-03	1.526728e-03	4.903425e-04	6.677459e-04	5.545943e-04	1.714323e-03	2.081118e-03	1.766847e-03	4.769351e-04	5.164888e-04	5.308971e-04	6.067396e-04	5.985584e-04	6.123414e-04	5.78947	4.87168	6.18803	7.03496	7.79545	6.272268e-04
DL-Glutamine	IC-MS	1.023917e-02	1.173174e-02	1.246318e-02	8.540133e-03	7.690610e-03	6.672254e-03	8.263214e-03	7.933797e-03	6.568669e-03	7.026252e-03	7.536145e-03	7.098991e-03	7.489672e-03	7.904853e-03	7.005649e-03	7.576188e-03	7.757666e-03	7.826017e-03	6.203994e-03	6.089824e-03	6.287814e-03	1.35864	1.14278	1.34275	1.10355	1.08773	1.051724e-02
DL-Homoserine	IC-MS	2.514874e-04	2.736213e-04	2.716512e-04	2.269185e-04	2.004237e-04	2.023013e-04	3.009503e-04	2.794998e-04	2.655752e-04	1.857757e-04	2.205388e-04	2.010600e-04	3.429155e-04	3.517399e-04	3.056422e-04	2.619470e-04	3.031447e-04	2.938243e-04	2.661079e-04	3.626640e-04	2.725893e-04	3.32348	3.54810	3.85765	4.28832	3.71302	3.591228e-04
DL-Beta-Leucine_RT1	IC-MS	2.870436e-03	3.289794e-03	3.192581e-03	3.103018e-03	2.777274e-03	2.789835e-03	4.620695e-03	4.281699e-03	4.017784e-03	2.699750e-03	3.217443e-03	2.929050e-03	4.741938e-03	5.133357e-03	4.555252e-03	3.401862e-03	3.469667e-03	3.308706e-03	3.746477e-03	4.038439e-03	3.921594e-03	5.00967	4.35990	4.90114	5.12762	4.91617	4.505082e-03
DL-Beta-Leucine_RT2	IC-MS	1.977874e-03	2.318208e-03	2.179483e-03	2.080552e-03	1.861237e-03	1.879796e-03	2.977199e-03	2.731950e-03	2.436727e-03	1.698370e-03	2.108796e-03	1.994091e-03	2.931754e-03	2.954626e-03	2.739194e-03	2.385929e-03	2.606162e-03	2.445940e-03	2.715018e-03	2.998061e-03	2.733368e-03	3.22260	2.84441	3.25492	3.43809	2.97620	2.797096e-03
Docosahexaenoic Acid	IC-MS	1.195086e-03	9.861849e-04	1.047724e-03	2.122318e-03	1.746195e-03	2.144172e-03	2.494513e-03	1.884114e-03	1.859806e-03	2.186772e-03	2.123630e-03	2.062606e-03	2.000825e-03	2.053438e-03	1.691147e-03	3.393261e-03	4.199639e-03	3.654344e-03	3.361363e-03	4.062733e-03	3.159782e-03	3.13013	3.81256	3.29361	3.33685	2.59763	2.492305e-03
Docosanoic acid_RT1	IC-MS	1.330350e-03	1.110926e-03	1.316129e-03	9.702074e-04	1.064208e-03	1.184635e-03	6.830312e-04	9.098148e-04	1.034467e-03	1.145430e-03	1.017735e-03	1.084697e-03	9.397668e-04	8.674779e-04	9.927960e-04	1.141127e-03	9.106188e-04	9.526585e-04	1.115377e-03	1.033166e-03	1.234594e-03	1.04564	1.19460	1.34045	1.27634	1.42385	1.531605e-03
Docosanoic acid_RT2	IC-MS	1.349002e-04	1.225961e-04	1.671724e-04	1.220707e-04	1.198353e-04	1.526558e-04	1.457380e-04	1.195045e-04	1.246693e-04	1.402143e-04	1.233158e-04	1.312835e-04	1.400546e-04	1.211484e-04	1.244376e-04	1.461474e-04	1.738312e-04	1.618797e-04	1.170056e-04	1.374860e-04	1.541275e-04	1.30971	1.38345	1.20926	1.26688	1.18917	1.189886e-04
Docosatetraenoic acid	IC-MS	4.252882e-04	3.453299e-04	3.392410e-04	8.538830e-04	7.072626e-04	9.139189e-04	1.149481e-03	8.734715e-04	7.930957e-04	9.607376e-04	8.808592e-04	7.971070e-04	9.796833e-04	9.112071e-04	7.620727e-04	1.634210e-03	2.009185e-03	1.825466e-03	2.365533e-03	2.802185e-03	2.112754e-03	1.68358	2.22096	1.82103	2.45273	1.93142	1.532702e-03
Drostanolone propionate	IC-MS	7.122495e-05	7.309229e-05	5.856243e-05	1.438271e-04	1.062233e-04	1.268495e-04	2.006441e-04	1.816099e-04	1.540311e-04	1.178924e-04	1.565452e-04	1.468814e-04	1.587060e-04	1.446932e-04	1.255895e-04	1.732588e-04	2.483227e-04	2.131427e-04	2.095382e-04	2.528527e-04	2.000348e-04	1.46763	1.59923	1.31846	1.17765	1.05649	7.844843e-05
e,z-2,6-DODECADIEN-1-AL	IC-MS	3.460672e-04	2.671003e-04	3.318404e-04	2.483532e-04	4.136703e-04	3.763924e-04	1.717812e-04	3.748896e-04	2.293070e-04	2.326216e-04	2.278453e-04	3.298526e-04	2.623157e-04	2.610630e-04	2.553482e-04	2.376788e-04	3.451462e-04	3.447489e-04	2.268521e-04	2.430739e-04	2.853822e-04	2.21775	2.71569	3.36287	3.16433	9.41875	3.448215e-04
Erucic acid	IC-MS	3.431492e-04	2.680813e-04	3.229654e-04	3.687741e-04	3.058355e-04	3.735309e-04	5.117506e-04	3.654623e-04	3.916806e-04	3.854454e-04	3.981226e-04	3.526407e-04	4.389614e-04	4.528997e-04	3.779085e-04	3.932396e-04	4.763860e-04	4.389816e-04	3.100885e-04	3.788885e-04	2.866054e-04	2.77074	3.32517	2.64582	2.19310	1.89055	1.408687e-04
Fumaric acid	IC-MS	2.563598e-04	2.641944e-04	2.705287e-04	3.173875e-04	2.885980e-04	2.682088e-04	3.936096e-04	3.612739e-04	2.884724e-04	2.708374e-04	3.582124e-04	3.185040e-04	3.561720e-04	3.498601e-04	3.233883e-04	3.044432e-04	2.969239e-04	3.227039e-04	1.218504e-04	1.315363e-04	1.308400e-04	2.44805	1.86019	2.14768	1.80203	1.70538	1.638446e-04
g-Guanidinobutyrate	IC-MS	7.730754e-05	8.596504e-05	1.297094e-04	8.856974e-05	9.708631e-05	1.028688e-04	1.616053e-04	1.774045e-04	1.411259e-04	9.000549e-05	1.024340e-04	9.859309e-05	1.688208e-04	2.298413e-04	2.048454e-04	8.493383e-05	8.769291e-05	1.564169e-04	1.542837e-04	1.445371e-04	1.522581e-04	1.07655	7.40374	8.55914	1.35177	1.47937	1.387866e-04
gamma-Aminobutyric acid	IC-MS	1.229312e-03	1.497583e-03	1.440123e-03	1.086556e-03	1.210146e-03	9.875458e-04	1.072461e-03	1.207602e-03	9.394131e-04	9.665383e-04	1.170495e-03	1.021373e-03	1.037223e-03	1.199847e-03	1.033033e-03	1.341334e-03	1.299923e-03	1.326176e-03	1.478314e-03	1.383161e-03	1.515520e-03	1.07265	8.76745	1.09438	1.19490	1.07311	1.149286e-03
glu-asp	IC-MS	9.598090e-05	1.127992e-04	1.237252e-04	1.562704e-04	1.496614e-04	1.312194e-04	1.770003e-04	1.664838e-04	1.575241e-04	1.279232e-04	1.468353e-04	1.341390e-04	1.458947e-04	1.511709e-04	1.433509e-04	1.692617e-04	1.771002e-04	1.575025e-04	1.378758e-04	1.438227e-04	1.260304e-04	1.56949	1.44585	1.72514	1.57507	1.33983	1.336421e-04
Gluconic acid	IC-MS	2.563394e-04	3.291830e-04	3.335454e-04	3.234805e-04	2.921782e-04	2.423002e-04	3.172225e-04	3.121658e-04	2.427502e-04	2.601411e-04	3.473230e-04	2.749865e-04	2.658372e-04	2.531987e-04	2.398885e-04	4.198457e-04	4.619827e-04	4.222957e-04	3.338398e-04	3.354127e-04	3.258402e-04	3.72947	2.55806	3.22947	2.97213	2.46119	2.362790e-04
glyceraldehyde 3-phosphate	IC-MS	9.175883e-05	9.644887e-05	1.389393e-04	1.283116e-04	1.430941e-04	1.371205e-04	2.032792e-04	2.701697e-04	1.952979e-04	1.085534e-04	2.772396e-04	1.793542e-04	1.910912e-04	2.235772e-04	7.610580e-05	3.511279e-04	3.188096e-04	3.336486e-04	1.292558e-04	1.852206e-04	1.336586e-04	4.92960	3.57542	4.40848	4.55261	3.52906	4.033679e-04
glycerin	IC-MS	3.786265e-04	2.787351e-04	2.820679e-04	2.318373e-04	2.613045e-04	2.705447e-04	1.633235e-04	1.900691e-04	2.157473e-04	2.302010e-04	2.150017e-04	1.938786e-04	1.813372e-04	1.674894e-04	2.149520e-04	2.225936e-04	2.113009e-04	2.445421e-04	1.905692e-04	2.301314e-04	2.517529e-04	1.89559	2.43774	2.30715	2.17533	2.33381	2.822972e-04
Glycerophosphoglycerol	IC-MS	8.245351e-05	1.065940e-04	1.049786e-04	1.136209e-04	1.132680e-04	1.011068e-04	1.228121e-04	1.328965e-04	1.147539e-04	9.668769e-05	1.221850e-04	1.058406e-04	1.190052e-04	1.163479e-04	1.049725e-04	1.618766e-04	1.643015e-04	1.870161e-04	2.833089e-04	2.695525e-04	2.675260e-04	1.13083	9.76854	1.08765	1.21636	9.50547	9.277195e-05
glycidyl oleate_RT1	IC-MS	1.466120e-03	1.354528e-03	1.330360e-03	1.435423e-03	1.310867e-03	1.302623e-03	2.342431e-03	1.905964e-03	1.894933e-03	1.327807e-03	1.428789e-03	1.341617e-03	1.978458e-03	1.791660e-03	1.790443e-03	9.754158e-04	1.189113e-03	1.034347e-03	8.511203e-04	9.979732e-04	7.903322e-04	5.89546	7.48135	6.10915	3.68662	3.01944	2.940485e-04
glycidyl oleate_RT2	IC-MS	2.762528e-04	2.815155e-04	2.475548e-04	3.073126e-04	2.505591e-04	2.562032e-04	6.067026e-04	4.736460e-04	4.256189e-04	2.577796e-04	3.021983e-04	2.712246e-04	4.650178e-04	4.466588e-04	4.038998e-04	2.260232e-04	2.894632e-04	2.528752e-04	2.302511e-04	2.644294e-04	2.047249e-04	1.66604	1.71178	1.54157	9.32642	7.80577	6.576428e-05
glycidyl oleate_RT3	IC-MS	1.247524e-04	1.209030e-04	9.610186e-05	1.227859e-04	9.386733e-05	1.081649e-04	2.412675e-04	1.973246e-04	1.833764e-04	9.959045e-05	1.336050e-04	1.181855e-04	1.895721e-04	1.657981e-04	1.529810e-04	9.944787e-05	1.483136e-04	1.209392e-04	1.061915e-04	1.302365e-04	1.030002e-04	6.78991	7.64158	6.24883	5.00534	5.08516	3.975900e-05
Glycidyl Stearate	IC-MS	3.447533e-04	3.679728e-04	3.381756e-04	3.391292e-04	3.742076e-04	4.199432e-04	2.997627e-04	3.256144e-04	3.573596e-04	3.407577e-04	3.213707e-04	3.996604e-04	2.495600e-04	2.605924e-04	3.357606e-04	3.301491e-04	3.698228e-04	4.143426e-04	4.041712e-04	3.499690e-04	4.077200e-04	3.55721	3.60996	3.88825	3.75373	3.83372	4.278992e-04
Guanine_RT1	IC-MS	8.564577e-04	9.721282e-04	9.728747e-04	1.097960e-03	1.087216e-03	9.605249e-04	1.291570e-03	1.265025e-03	1.131707e-03	1.049863e-03	1.195576e-03	9.914055e-04	1.173655e-03	1.238444e-03	1.117302e-03	1.109521e-03	1.156202e-03	1.183919e-03	9.542671e-04	1.070573e-03	9.292825e-04	8.72637	8.09257	8.19230	8.07091	6.93616	7.964478e-04
Guanine_RT2	IC-MS	4.635655e-04	5.313078e-04	5.853890e-04	5.702872e-04	5.474154e-04	4.973519e-04	7.087153e-04	6.898113e-04	6.673223e-04	6.199171e-04	6.828543e-04	5.007744e-04	6.737870e-04	8.112506e-04	6.357748e-04	6.830579e-04	6.521452e-04	7.210655e-04	4.517170e-04	5.157480e-04	4.822468e-04	4.98248	5.26951	4.68503	4.46945	4.24812	5.203335e-04
Guanosine	IC-MS	3.759194e-04	4.115407e-04	3.878042e-04	3.422780e-04	3.432568e-04	3.157319e-04	4.275243e-04	4.160073e-04	4.114462e-04	3.786077e-04	4.270179e-04	3.288271e-04	4.206170e-04	4.629734e-04	3.625806e-04	4.134368e-04	3.765534e-04	4.358444e-04	3.078410e-04	3.041064e-04	2.631386e-04	3.53810	3.22086	3.20889	2.98106	2.34042	2.938508e-04
Hypoxanthine_RT1	IC-MS	2.270777e-02	2.661888e-02	2.673990e-02	2.544840e-02	2.673864e-02	2.227813e-02	2.776213e-02	2.999960e-02	2.679903e-02	2.539941e-02	3.029077e-02	2.589721e-02	2.800361e-02	2.540966e-02	2.630907e-02	2.617153e-02	2.945571e-02	2.884931e-02	2.773724e-02	2.980693e-02	2.499332e-02	2.73387	2.32397	2.50209	2.25149	2.18045	2.033449e-02
Hypoxanthine_RT2	IC-MS	5.130811e-03	6.161974e-03	6.550592e-03	6.318511e-03	6.594609e-03	5.732264e-03	7.782474e-03	8.015810e-03	7.024970e-03	6.511622e-03	7.725984e-03	6.677219e-03	7.438580e-03	8.397027e-03	6.952129e-03	6.590523e-03	6.441185e-03	6.775574e-03	5.167031e-03	5.318287e-03	5.084579e-03	6.57130	6.05571	6.59561	5.84025	5.25097	5.366900e-03
Imagabalin	IC-MS	4.639983e-04	4.543841e-04	4.399070e-04	3.957804e-04	4.027671e-04	3.646384e-04	2.411027e-04	3.555011e-04	4.286689e-04	4.622285e-04	3.159967e-04	4.387173e-04	2.606159e-04	2.930165e-04	3.772316e-04	3.679237e-04	3.558486e-04	4.250529e-04	5.415199e-04	3.646110e-04	4.991867e-04	4.14868	6.54428	4.17771	4.09964	5.98521	5.858816e-04
L(-)-Carnitine	IC-MS	6.792386e-03	8.091740e-03	9.403846e-03	4.729139e-03	4.891382e-03	4.340760e-03	8.790234e-03	9.089426e-03	6.980311e-03	3.251526e-03	3.925997e-03	3.404371e-03	5.134374e-03	5.865321e-03	4.715993e-03	7.230488e-03	7.444914e-03	7.737382e-03	8.059876e-03	9.087875e-03	8.188557e-03	5.21704	4.24779	4.82966	5.97112	5.15838	4.646075e-03
L-(+)-Arginine	IC-MS	3.707236e-02	4.162281e-02	3.856241e-02	3.396892e-02	2.889411e-02	3.038612e-02	3.171438e-02	2.791568e-02	2.547471e-02	2.757059e-02	2.676082e-02	2.801104e-02	2.628086e-02	2.554577e-02	2.512324e-02	3.149603e-02	3.302759e-02	2.963756e-02	2.717170e-02	2.834878e-02	2.726112e-02	3.33092	2.96783	2.97629	2.40153	2.62021	2.140983e-02
L-(+)-Tartaric acid	IC-MS	2.958422e-04	3.860573e-04	2.310486e-04	3.635817e-04	2.759205e-04	2.697580e-04	2.571588e-04	1.972102e-04	1.949827e-04	4.164348e-04	3.344123e-04	3.211642e-04	2.314100e-04	1.664927e-04	2.063680e-04	2.083573e-04	2.155590e-04	2.023560e-04	3.141902e-04	2.561021e-04	2.472782e-04	3.48962	2.87774	2.86432	2.47499	2.06747	3.006323e-04
L-(-)-methionine	IC-MS	2.019881e-04	2.151374e-04	2.129419e-04	2.051230e-04	1.713349e-04	1.838981e-04	2.619355e-04	2.312855e-04	2.043518e-04	1.632703e-04	2.049312e-04	1.823429e-04	2.645335e-04	2.487944e-04	2.231239e-04	2.323489e-04	2.617867e-04	2.448857e-04	2.518732e-04	2.995667e-04	2.532335e-04	3.17021	2.72760	3.09575	3.14594	2.74792	2.529815e-04
L-alpha-Glycerylphosphorylcholine	IC-MS	2.553385e-02	2.930170e-02	3.184991e-02	4.064262e-02	3.959850e-02	3.520994e-02	3.091774e-02	3.246722e-02	2.701044e-02	4.512032e-02	5.022805e-02	4.656498e-02	3.725348e-02	4.226318e-02	3.612193e-02	3.004219e-02	3.131316e-02	2.910799e-02	2.317192e-02	2.451432e-02	2.350678e-02	1.32680	1.30429	1.29061	9.83521	9.17385	9.201184e-03
L-Aspartic acid_RT1	IC-MS	1.598874e-03	1.875951e-03	2.008835e-03	1.573233e-03	1.433308e-03	1.290289e-03	2.090094e-03	2.049328e-03	1.680462e-03	1.501085e-03	1.921210e-03	1.609608e-03	2.140656e-03	2.311116e-03	1.994337e-03	7.152946e-04	8.355497e-04	7.957905e-04	8.312152e-04	8.110698e-04	8.083329e-04	9.34799	6.77091	7.38134	5.93144	5.20118	6.625285e-04
L-Aspartic acid_RT2	IC-MS	5.846423e-04	6.260941e-04	6.592730e-04	4.785041e-04	3.888272e-04	3.702724e-04	4.477682e-04	4.658316e-04	4.338352e-04	4.939440e-04	4.391677e-04	4.144380e-04	5.114328e-04	5.217009e-04	4.843177e-04	2.569159e-04	2.752519e-04	2.701433e-04	3.105735e-04	3.001518e-04	3.051109e-04	3.32013	2.72242	3.11506	2.31251	2.01000	2.730441e-04
L-Glutamic acid	IC-MS	1.281686e-02	1.522540e-02	1.879316e-02	1.644226e-02	1.679104e-02	1.284076e-02	2.007744e-02	2.148914e-02	1.609813e-02	1.577188e-02	2.029633e-02	1.746751e-02	1.767348e-02	2.040248e-02	1.711059e-02	1.099788e-02	1.041321e-02	1.127793e-02	7.207012e-03	7.854353e-03	8.031961e-03	1.67056	1.48356	1.93560	1.50553	1.28881	1.250771e-02
L-Glutathione (reduced)	IC-MS	6.072074e-03	8.170353e-03	1.055094e-02	1.063135e-02	1.108164e-02	8.420509e-03	9.351406e-03	1.144636e-02	7.213348e-03	9.531033e-03	1.361527e-02	1.086396e-02	7.822327e-03	8.379964e-03	6.728142e-03	8.083825e-03	7.860282e-03	8.652168e-03	6.437359e-03	7.143191e-03	6.395796e-03	7.84580	6.36163	8.38452	6.25115	4.95702	4.906917e-03
L-Hydroxyproline	IC-MS	4.026357e-03	5.282883e-03	6.488209e-03	5.444150e-03	4.763899e-03	4.227605e-03	1.437832e-02	1.273234e-02	1.293341e-02	5.411757e-03	4.921735e-03	6.009345e-03	1.599825e-02	1.838803e-02	1.740253e-02	4.364989e-03	4.215831e-03	4.090002e-03	3.798850e-03	3.774393e-03	4.177665e-03	8.12391	7.32279	7.86236	8.54178	6.89031	6.214438e-03
L-Iditol	IC-MS	4.461955e-04	5.228148e-04	4.865271e-04	4.464016e-04	3.426357e-04	3.861613e-04	5.135552e-04	4.522062e-04	3.944829e-04	3.566551e-04	3.573145e-04	3.980385e-04	4.004788e-04	3.903681e-04	3.482625e-04	4.767049e-04	4.979598e-04	4.760878e-04	5.256080e-04	5.088264e-04	5.576188e-04	4.57369	3.43069	3.25311	3.12166	3.87349	2.578346e-04
L-Phenylalanine	IC-MS	8.358456e-04	9.530000e-04	9.287413e-04	8.416699e-04	7.224531e-04	7.789274e-04	1.017767e-03	8.950997e-04	7.852979e-04	7.089999e-04	8.293022e-04	7.629404e-04	9.953557e-04	9.363116e-04	8.538705e-04	9.849328e-04	1.126842e-03	1.036162e-03	1.041793e-03	1.262480e-03	1.093974e-03	1.26572	1.17120	1.23935	1.29391	1.16193	1.008635e-03
L-Serine	IC-MS	3.011714e-04	3.069842e-04	2.778355e-04	1.708816e-04	1.624054e-04	1.704725e-04	1.691826e-04	1.383485e-04	1.254279e-04	1.346233e-04	1.613464e-04	1.444963e-04	1.389988e-04	1.110611e-04	1.097357e-04	2.196897e-04	2.553174e-04	2.826931e-04	2.502464e-04	2.863104e-04	2.571760e-04	2.45608	2.20151	2.14867	1.68545	1.89384	1.334071e-04
L-Threonic acid_RT1	IC-MS	1.024138e-03	1.241626e-03	1.563923e-03	1.361433e-03	1.310288e-03	1.120479e-03	1.714390e-03	1.861622e-03	1.485975e-03	1.301375e-03	1.584063e-03	1.418516e-03	1.724118e-03	2.479666e-03	1.533386e-03	1.183041e-03	1.145597e-03	1.233784e-03	9.906202e-04	9.086572e-04	9.958948e-04	9.31639	6.67544	8.14559	7.06274	6.10148	6.057305e-04
L-Threonic acid_RT2	IC-MS	2.374458e-03	1.339086e-03	1.031657e-03	9.282717e-04	7.682615e-04	7.303776e-04	8.390519e-04	6.389738e-04	5.108374e-04	1.290852e-03	8.137172e-04	7.667095e-04	8.326384e-04	7.340642e-04	9.190472e-04	9.642269e-04	7.078160e-04	5.924753e-04	8.127555e-04	6.912666e-04	8.046605e-04	7.44936	7.94218	5.56005	7.26655	6.86891	7.512650e-04
L-Tyrosine	IC-MS	5.443707e-04	6.181288e-04	5.807391e-04	5.617739e-04	5.160247e-04	5.255722e-04	7.695103e-04	7.090713e-04	6.477269e-04	5.091971e-04	5.729910e-04	5.535149e-04	7.952944e-04	7.596882e-04	6.941714e-04	6.407575e-04	6.904967e-04	6.875248e-04	7.419491e-04	8.030649e-04	7.346257e-04	8.77033	8.00417	8.68416	8.66292	7.98520	7.126404e-04
Lauric acid	IC-MS	3.041321e-04	5.106024e-04	1.021901e-03	3.490256e-04	3.238401e-04	2.961068e-04	3.530278e-04	3.102823e-04	2.868494e-04	3.321031e-04	3.079586e-04	3.325340e-04	4.057064e-04	4.020275e-04	3.151365e-04	4.705305e-04	5.461982e-04	4.383577e-04	4.460106e-04	4.888476e-04	3.551131e-04	3.79708	3.56177	3.22311	4.03838	1.38244	3.258574e-04
lentiginosine	IC-MS	1.842214e-04	2.120098e-04	2.169384e-04	1.907180e-04	1.712808e-04	1.618392e-04	2.961636e-04	2.825462e-04	2.461439e-04	1.704328e-04	1.971800e-04	1.728086e-04	2.510359e-04	2.716390e-04	2.283800e-04	1.821026e-04	1.952223e-04	1.839146e-04	1.964953e-04	2.017141e-04	2.193103e-04	2.08826	1.53535	1.92323	1.87286	1.85574	1.678093e-04
Lignoceric acid	IC-MS	2.165618e-04	1.831191e-04	2.402578e-04	2.257049e-04	2.115173e-04	2.761785e-04	3.153360e-04	2.184651e-04	2.284393e-04	2.708608e-04	2.205365e-04	2.274873e-04	3.007389e-04	2.674081e-04	2.289823e-04	2.972449e-04	3.577404e-04	3.252702e-04	2.424361e-04	3.221875e-04	2.778394e-04	1.81078	2.26678	1.68751	1.67276	1.55921	1.329035e-04
Linoleic acid	IC-MS	2.911545e-03	3.172740e-03	3.130127e-03	4.999761e-03	4.187300e-03	4.640029e-03	5.897351e-03	5.168790e-03	5.341604e-03	4.681073e-03	4.860746e-03	4.617593e-03	5.004034e-03	4.770507e-03	4.628615e-03	6.770173e-03	7.764026e-03	7.128713e-03	8.324699e-03	9.516529e-03	7.881700e-03	6.54739	7.71557	6.44171	7.29111	7.11019	4.744513e-03
Lithocholic acid	IC-MS	9.146350e-05	2.127805e-04	1.578409e-04	2.165191e-04	2.003141e-04	1.463475e-04	1.357608e-04	1.499024e-04	1.672903e-04	1.418999e-04	2.311362e-04	1.613465e-04	8.589187e-05	6.173194e-05	1.606397e-04	1.751754e-04	1.446664e-04	1.394237e-04	7.125138e-05	1.131300e-04	1.460983e-04	1.89665	3.19760	1.31588	6.17114	6.18317	7.314452e-05
Maleamic acid	IC-MS	6.099988e-03	6.915659e-03	7.068433e-03	5.618053e-03	5.438471e-03	4.359838e-03	4.973572e-03	5.232499e-03	4.244795e-03	5.097856e-03	6.955250e-03	5.427761e-03	5.632483e-03	5.540475e-03	4.996803e-03	3.023513e-03	2.858851e-03	3.375252e-03	2.491985e-03	2.307188e-03	2.307022e-03	4.09372	3.17925	3.80761	2.25760	1.84879	1.959854e-03
Margaric acid_RT1	IC-MS	9.652727e-04	1.044233e-03	9.672513e-04	1.190943e-03	1.083808e-03	1.230594e-03	1.129714e-03	9.709368e-04	1.024340e-03	1.163531e-03	1.132669e-03	1.190741e-03	9.923654e-04	1.024882e-03	1.015554e-03	1.277124e-03	1.403895e-03	1.396818e-03	1.314082e-03	1.294562e-03	1.276066e-03	1.19981	1.23511	1.10736	1.18935	1.31406	9.872064e-04
Margaric acid_RT2	IC-MS	7.060382e-04	6.318403e-04	6.568778e-04	6.357078e-04	6.774959e-04	6.947452e-04	5.284228e-04	5.712550e-04	6.593196e-04	6.589544e-04	6.297705e-04	6.078227e-04	5.691353e-04	4.890335e-04	5.808923e-04	6.466265e-04	6.461048e-04	6.439345e-04	6.596409e-04	6.826324e-04	6.374742e-04	6.77339	6.94259	6.98154	7.42458	6.94577	7.150903e-04
Megastigma-5,8(Z)-dien-4-one	IC-MS	4.844046e-04	3.665037e-04	5.588863e-04	3.189161e-04	5.524319e-04	5.114690e-04	2.285422e-04	4.869484e-04	2.830451e-04	3.175741e-04	2.944446e-04	5.444854e-04	3.378906e-04	3.580647e-04	3.170175e-04	2.991498e-04	4.799674e-04	4.492392e-04	2.886366e-04	3.118159e-04	3.868444e-04	2.71814	3.52372	4.54015	4.12959	1.30689	4.783677e-04
Mephedrone	IC-MS	1.798013e-03	1.831194e-03	1.711508e-03	1.597923e-03	1.665175e-03	1.535972e-03	1.558539e-03	1.553505e-03	1.529001e-03	1.847275e-03	1.574159e-03	1.815902e-03	1.503355e-03	1.504831e-03	1.589317e-03	1.705059e-03	1.576028e-03	1.629881e-03	1.800614e-03	1.755934e-03	1.797499e-03	1.87304	1.84744	1.96336	1.87016	2.02444	2.009778e-03
Methyl (9E)-9-octadecenoate	IC-MS	5.328803e-04	4.304567e-04	4.446686e-04	8.518594e-04	6.275856e-04	8.303540e-04	9.790476e-04	7.985502e-04	7.997909e-04	8.296156e-04	8.082118e-04	6.938023e-04	8.397189e-04	7.979638e-04	7.070754e-04	8.525136e-04	9.904243e-04	9.087510e-04	8.030448e-04	9.324470e-04	7.389975e-04	5.47040	7.47754	5.32070	4.89252	4.10084	2.825125e-04
Methyl acetoacetate	IC-MS	2.778013e-04	2.850254e-04	2.893680e-04	3.807506e-04	2.600766e-04	2.771301e-04	2.517621e-04	2.423989e-04	2.528744e-04	3.105764e-04	2.510114e-04	2.759704e-04	2.538093e-04	2.368686e-04	2.457729e-04	3.541990e-04	3.267384e-04	3.215762e-04	1.763967e-04	1.833812e-04	1.778773e-04	3.57234	2.85928	2.97799	2.76739	2.90854	2.184220e-04
Methyl stearate	IC-MS	9.447357e-05	9.922671e-05	1.113673e-04	1.309911e-04	1.106552e-04	1.333604e-04	1.669207e-04	1.102253e-04	1.196347e-04	1.366895e-04	1.296464e-04	1.132025e-04	1.414058e-04	1.206044e-04	1.165051e-04	1.566777e-04	1.730223e-04	1.672701e-04	1.579063e-04	1.654466e-04	1.620660e-04	1.34826	1.41041	1.31303	1.48045	1.46475	1.144922e-04
Methylmalonic acid	IC-MS	3.736227e-04	3.962285e-04	3.712596e-04	3.777479e-04	4.050081e-04	3.516485e-04	3.253169e-04	3.694205e-04	3.244937e-04	3.434928e-04	4.166144e-04	3.830550e-04	3.467645e-04	3.727063e-04	3.201451e-04	2.724371e-04	2.332220e-04	2.653668e-04	2.153170e-04	2.090966e-04	2.067670e-04	3.02783	2.33016	2.95992	2.55269	2.44803	3.610849e-04
Myristic acid	IC-MS	2.596089e-03	3.391915e-03	3.848736e-03	3.236328e-03	2.864907e-03	3.041035e-03	3.760604e-03	2.913449e-03	2.992658e-03	3.186574e-03	3.246827e-03	3.013743e-03	3.238841e-03	3.409409e-03	2.693086e-03	4.253892e-03	4.905019e-03	4.405241e-03	3.477759e-03	4.390119e-03	3.221051e-03	3.05101	3.09089	2.56640	2.65316	3.88221	2.189970e-03
Myristoleic acid	IC-MS	8.207504e-05	1.298860e-04	9.735297e-05	9.559174e-05	8.275731e-05	8.258185e-05	1.960417e-04	1.486620e-04	1.652702e-04	9.279167e-05	9.747983e-05	1.026322e-04	1.666089e-04	1.803975e-04	1.458988e-04	2.669148e-04	2.707891e-04	2.374090e-04	3.719903e-04	4.613777e-04	3.247584e-04	2.31857	2.27975	1.92093	2.70544	2.52163	1.828225e-04
Myristyl sulfate	IC-MS	3.122624e-04	3.869914e-04	7.350124e-04	1.144623e-04	9.236161e-04	4.111525e-04	7.409446e-04	4.180057e-04	5.864532e-04	3.825243e-04	7.189798e-05	4.283201e-04	7.240324e-04	4.677482e-04	2.605604e-04	1.462643e-04	3.244054e-04	9.667991e-04	1.012158e-03	3.916968e-04	1.025508e-03	2.70133	6.49660	2.16290	7.81102	5.04363	3.484308e-04
N'-nitrosoanatabine	IC-MS	2.316384e-04	2.158834e-04	2.131184e-04	2.079740e-04	1.974826e-04	1.939415e-04	2.081693e-04	1.919747e-04	1.766215e-04	2.041135e-04	2.070942e-04	2.228268e-04	1.519308e-04	1.685581e-04	1.772752e-04	2.074140e-04	2.116152e-04	1.977264e-04	1.938196e-04	1.682476e-04	1.982695e-04	2.31931	2.20083	2.15905	1.75771	2.05136	1.657882e-04
N-(3-carboxypropanoyl)-N-hydroxycadaverine	IC-MS	6.122425e-05	7.837853e-05	9.447602e-05	1.527959e-04	1.307016e-04	1.123927e-04	1.500286e-04	1.474836e-04	1.351900e-04	9.705441e-05	9.127973e-05	9.906219e-05	1.491817e-04	1.683174e-04	1.571088e-04	1.971964e-04	2.026069e-04	1.169749e-04	2.006937e-04	2.584371e-04	2.116145e-04	8.41152	1.51129	1.66271	2.73926	2.48615	1.070379e-04
N-(tert-Butoxycarbonyl)-L-leucine	IC-MS	2.134628e-03	2.696897e-03	3.050565e-03	2.813441e-03	3.108150e-03	2.417547e-03	4.606284e-03	4.289263e-03	4.021888e-03	1.663243e-03	2.057174e-03	1.709380e-03	2.529337e-03	2.947050e-03	2.338009e-03	1.495919e-03	1.507856e-03	1.666496e-03	1.034266e-03	1.085314e-03	1.048877e-03	9.05617	7.02641	8.34893	1.05474	9.26197	8.915335e-04
N-[3-Carboxy-2-(carboxymethyl)-2-hydroxypropanoyl]glut	IC-MS	1.698686e-03	1.922547e-03	1.950067e-03	1.954098e-03	2.022428e-03	1.814617e-03	2.145997e-03	2.076974e-03	2.044541e-03	1.810102e-03	2.319987e-03	1.939945e-03	2.209903e-03	2.202753e-03	2.137272e-03	9.826023e-04	8.809615e-04	1.051972e-03	6.298688e-04	6.782132e-04	6.534570e-04	6.72398	5.91300	7.00881	3.61748	2.44092	2.903626e-04
N-Acetyl-1-aspartylglutamic acid	IC-MS	5.071543e-05	8.186004e-05	1.251382e-04	1.341008e-04	1.338338e-04	1.136961e-04	1.403867e-04	1.287454e-04	1.016537e-04	1.559277e-04	1.611457e-04	1.508921e-04	1.249442e-04	1.277629e-04	1.097375e-04	3.573421e-04	3.333541e-04	3.860509e-04	1.957936e-04	2.206982e-04	2.107014e-04	4.74359	3.55308	4.28504	3.18232	2.58475	2.320870e-04
N-acetyl-L-2-aminoadipic acid 6-phosphate	IC-MS	3.710731e-04	4.243419e-04	4.206613e-04	3.841755e-04	3.520566e-04	3.305838e-04	4.668530e-04	4.781562e-04	3.810481e-04	4.130869e-04	4.220705e-04	4.580501e-04	4.158782e-04	4.668601e-04	4.156210e-04	2.449584e-04	2.971530e-04	2.676751e-04	1.646982e-04	1.705399e-04	1.697617e-04	1.76953	1.00193	9.52421	3.50297	2.18376	3.487474e-05
N-Acetyl-L-methionine	IC-MS	1.841799e-04	2.260816e-04	2.671033e-04	4.138183e-04	4.196827e-04	3.408148e-04	5.677624e-04	5.464380e-04	4.375334e-04	3.569083e-04	5.017753e-04	4.084335e-04	5.118381e-04	5.283203e-04	4.450258e-04	5.223177e-04	5.846864e-04	6.111158e-04	3.597245e-04	3.970546e-04	3.438032e-04	3.02489	2.84827	3.22408	2.59706	2.04169	2.018073e-04
N-Acetylalanine	IC-MS	6.859127e-04	6.938832e-04	7.080368e-04	6.955730e-04	7.932917e-04	7.003730e-04	7.298529e-04	7.321926e-04	7.337753e-04	8.012270e-04	9.188014e-04	7.834969e-04	7.999244e-04	8.073262e-04	8.165267e-04	7.886159e-04	7.030314e-04	7.462946e-04	6.680039e-04	7.199290e-04	6.402728e-04	6.82670	7.75913	7.34615	7.48865	6.65966	9.491049e-04
N-Acetylneuraminic acid	IC-MS	1.839598e-04	2.058093e-04	1.481732e-04	2.154611e-04	1.864174e-04	1.914173e-04	2.511924e-04	2.118896e-04	2.224875e-04	2.086970e-04	1.802783e-04	1.673951e-04	2.227731e-04	1.867060e-04	2.017472e-04	3.504877e-04	3.366426e-04	3.458271e-04	5.568598e-04	5.571675e-04	4.881363e-04	2.46108	2.99414	2.62691	2.53394	2.20173	1.880378e-04
N-Acetylornithine	IC-MS	7.361266e-05	9.589747e-05	1.125016e-04	1.163753e-04	9.763666e-05	9.001847e-05	2.815554e-04	2.696146e-04	2.788615e-04	1.097289e-04	1.328508e-04	1.225154e-04	3.444218e-04	4.063387e-04	3.334460e-04	1.022445e-04	1.032693e-04	1.064341e-04	1.107531e-04	1.184727e-04	1.269453e-04	2.27596	1.70933	1.94610	2.13538	1.80886	1.497881e-04
N-Methylisopelletierine	IC-MS	2.663529e-04	3.028078e-04	2.581935e-04	2.958242e-04	4.579648e-04	4.574438e-04	2.990440e-04	2.677082e-04	2.924068e-04	2.800226e-04	3.071863e-04	3.529859e-04	2.705232e-04	2.741640e-04	3.339945e-04	3.177107e-04	2.262037e-04	2.127831e-04	3.174711e-04	3.167652e-04	3.201587e-04	3.70405	3.03721	5.75015	4.22579	3.99409	4.730702e-04
N-Undecanoylglycine	IC-MS	1.116487e-04	1.086279e-04	1.036011e-04	9.226525e-05	9.587916e-05	9.256039e-05	6.896188e-05	7.204318e-05	7.496352e-05	8.523459e-05	7.955220e-05	8.053694e-05	7.630285e-05	6.998603e-05	7.015087e-05	8.480876e-05	8.399226e-05	9.780102e-05	8.174033e-05	9.107733e-05	7.856136e-05	9.18352	9.08994	9.58355	8.96289	1.04079	9.772534e-05
N3,N4-Dimethyl-L-arginine	IC-MS	4.595198e-04	5.653350e-04	4.827895e-04	1.058828e-03	8.520012e-04	9.517684e-04	1.875022e-03	1.533674e-03	1.038455e-03	6.978697e-04	8.801695e-04	8.417004e-04	1.201781e-03	1.092990e-03	1.077605e-03	1.187975e-03	1.595003e-03	1.389255e-03	1.189756e-03	1.424937e-03	1.154165e-03	5.15565	5.81493	5.96304	5.19835	5.08913	3.922898e-04
N3-Metyladenine	IC-MS	8.883119e-03	8.551705e-03	8.350191e-03	7.804544e-03	8.485412e-03	7.931774e-03	6.291187e-03	6.320592e-03	6.619647e-03	8.290304e-03	6.843183e-03	7.509006e-03	6.506570e-03	6.349583e-03	6.834586e-03	7.814282e-03	6.821340e-03	7.526706e-03	7.704779e-03	7.721823e-03	7.673477e-03	7.64496	8.16738	7.82267	7.88816	8.57914	9.111254e-03
Nervonic acid	IC-MS	5.405332e-04	4.204991e-04	4.758792e-04	6.181139e-04	5.196937e-04	6.252548e-04	9.518888e-04	6.673716e-04	7.572949e-04	6.461848e-04	6.148933e-04	6.091006e-04	8.336959e-04	8.664630e-04	7.198104e-04	7.416806e-04	8.617523e-04	8.323875e-04	6.718955e-04	8.117288e-04	6.104094e-04	4.86988	6.02748	4.83515	4.09046	3.30833	2.630042e-04
Nonanoic acid	IC-MS	1.950656e-03	1.965260e-03	2.010716e-03	1.623762e-03	1.771704e-03	1.352526e-03	1.441823e-03	1.476313e-03	1.709649e-03	1.560231e-03	1.533442e-03	1.486620e-03	1.388597e-03	1.547119e-03	1.602943e-03	1.662001e-03	1.499072e-03	1.531948e-03	1.728510e-03	1.621858e-03	1.738087e-03	1.78316	1.55239	1.60468	1.98571	1.71961	1.802848e-03
NP-011548	IC-MS	3.428359e-03	2.857435e-03	3.022380e-03	2.762414e-03	3.027612e-03	3.047891e-03	2.517176e-03	2.710488e-03	2.906078e-03	2.954880e-03	2.923442e-03	2.812747e-03	2.511887e-03	2.295073e-03	2.827911e-03	2.757976e-03	2.945211e-03	3.217320e-03	2.874825e-03	3.023509e-03	3.343248e-03	2.73600	3.11121	2.93349	3.02749	3.06080	3.347960e-03
Octodrine	IC-MS	2.962081e-03	2.743241e-03	2.591006e-03	2.243112e-03	2.264864e-03	2.183426e-03	1.823330e-03	1.835338e-03	1.775376e-03	2.277579e-03	2.026891e-03	2.216423e-03	1.867769e-03	1.836153e-03	1.892446e-03	2.280016e-03	2.016784e-03	2.118999e-03	2.192853e-03	2.129172e-03	2.103587e-03	2.43259	2.56428	2.55646	2.53567	2.63223	2.758796e-03
Oleic acid	IC-MS	6.115786e-02	5.510684e-02	5.186704e-02	8.079911e-02	6.708376e-02	7.508378e-02	9.677622e-02	8.397621e-02	8.643170e-02	7.372369e-02	7.754946e-02	6.986928e-02	8.350004e-02	8.235099e-02	7.524823e-02	8.085349e-02	9.516668e-02	8.570930e-02	7.650769e-02	8.563248e-02	7.168211e-02	6.23521	7.33279	5.88169	4.92361	4.24311	3.234014e-02
Ornithine	IC-MS	4.190196e-04	4.699733e-04	4.411927e-04	3.930760e-04	3.115926e-04	3.420817e-04	5.030502e-04	4.351995e-04	4.122090e-04	3.348860e-04	3.677358e-04	3.664580e-04	4.619975e-04	4.357608e-04	4.201715e-04	3.855400e-04	4.287375e-04	3.878902e-04	4.261879e-04	4.527560e-04	4.254303e-04	4.64107	4.00567	4.09004	3.68987	3.70098	2.998981e-04
Osbond acid	IC-MS	6.559208e-04	5.175655e-04	5.373159e-04	1.407894e-03	1.076118e-03	1.316645e-03	1.526355e-03	1.122242e-03	1.096106e-03	1.240607e-03	1.294314e-03	1.111537e-03	1.134404e-03	1.057033e-03	9.492958e-04	2.306345e-03	2.621347e-03	2.426479e-03	2.327771e-03	2.737858e-03	2.216712e-03	2.09282	2.80806	2.21168	2.42854	1.95149	1.516490e-03
oxolamine	IC-MS	2.689609e-04	2.640646e-04	2.656057e-04	2.212068e-04	2.090974e-04	2.153976e-04	2.354047e-04	2.226281e-04	1.865250e-04	2.425834e-04	2.244303e-04	2.608117e-04	1.660232e-04	1.858119e-04	1.835826e-04	2.247695e-04	2.339984e-04	2.078608e-04	2.238375e-04	1.860685e-04	2.160352e-04	2.60440	2.28878	2.14986	1.71214	2.20520	1.752732e-04
p-Cresylsulfate	IC-MS	2.969697e-04	3.227871e-04	6.773146e-04	2.459431e-04	1.562132e-04	1.840220e-04	2.344269e-04	1.868029e-04	1.634271e-04	2.198341e-04	2.287441e-04	2.120092e-04	1.731988e-04	1.681488e-04	1.804933e-04	2.016932e-04	2.147283e-04	1.690993e-04	1.848329e-04	1.823021e-04	1.874089e-04	2.62996	1.56623	1.56315	1.38309	1.37395	1.104882e-04
P-DMEA_RT1	IC-MS	5.603179e-04	7.194605e-04	7.676842e-04	8.178914e-04	8.677349e-04	7.403043e-04	6.992418e-04	7.761742e-04	5.712978e-04	6.551596e-04	8.613802e-04	6.829738e-04	5.280398e-04	5.420784e-04	4.909230e-04	7.467772e-04	7.256932e-04	7.687309e-04	3.533626e-04	3.831465e-04	3.548479e-04	4.65844	3.95271	4.72189	3.54028	2.99535	3.377829e-04
P-DMEA_RT2	IC-MS	1.670377e-04	2.235767e-04	2.140451e-04	3.521365e-04	3.920683e-04	3.485882e-04	2.237488e-04	2.415615e-04	1.937666e-04	2.700290e-04	3.640067e-04	2.881233e-04	1.535253e-04	1.554723e-04	1.166719e-04	3.963165e-04	3.389917e-04	4.018370e-04	2.519826e-04	2.594912e-04	2.471495e-04	2.73312	2.34158	2.92130	1.62198	1.52219	1.256598e-04
Palmitic acid_RT1	IC-MS	6.344278e-02	5.920340e-02	5.803612e-02	6.802525e-02	6.142987e-02	6.752225e-02	6.652012e-02	5.790637e-02	6.419489e-02	6.587216e-02	6.294233e-02	6.655217e-02	5.721498e-02	5.693675e-02	5.914423e-02	6.149113e-02	6.466659e-02	6.354405e-02	5.833055e-02	5.597375e-02	5.866463e-02	5.52041	5.90606	5.57987	5.36851	5.73618	5.308840e-02
Palmitic acid_RT2	IC-MS	3.551299e-02	3.192029e-02	3.285129e-02	3.051168e-02	3.278851e-02	3.245213e-02	2.775539e-02	2.711863e-02	3.009753e-02	3.258661e-02	3.025309e-02	3.185921e-02	2.642872e-02	2.646371e-02	2.832279e-02	3.212229e-02	3.072475e-02	3.108402e-02	3.234618e-02	3.089430e-02	3.125201e-02	3.22037	3.32815	3.15734	3.33414	3.33560	3.571423e-02
Pentadecanoic acid	IC-MS	4.937272e-04	8.975520e-04	6.219017e-04	6.467580e-04	5.978030e-04	7.997181e-04	6.970257e-04	5.256712e-04	5.642674e-04	6.410054e-04	7.386932e-04	7.330385e-04	5.878519e-04	5.996939e-04	4.927686e-04	9.338664e-04	9.367573e-04	7.899376e-04	6.640419e-04	9.139109e-04	7.758572e-04	7.44198	8.20690	6.19349	7.72252	1.06575	6.303629e-04
Pentonic acid	IC-MS	1.637112e-03	2.060214e-03	1.248000e-03	1.879523e-03	1.470703e-03	1.471201e-03	1.324192e-03	1.033568e-03	1.017030e-03	2.081800e-03	1.765623e-03	1.557643e-03	1.159471e-03	9.114112e-04	1.065086e-03	1.122971e-03	1.161059e-03	1.111740e-03	1.625932e-03	1.371466e-03	1.260884e-03	1.84304	1.53616	1.48712	1.33215	1.15586	1.591485e-03
phendimetrazine	IC-MS	2.654624e-04	2.766051e-04	2.762736e-04	2.477666e-04	2.533945e-04	2.510092e-04	2.472181e-04	2.420509e-04	2.310271e-04	2.882460e-04	2.429505e-04	2.823834e-04	2.285938e-04	2.274948e-04	2.347427e-04	2.509069e-04	2.506936e-04	2.465710e-04	2.737229e-04	2.664767e-04	2.680486e-04	2.78752	2.89633	2.98494	2.82334	3.05537	3.076288e-04
Phosphoenolpyruvic acid	IC-MS	8.431383e-04	1.145425e-03	1.340274e-03	9.325399e-04	9.031928e-04	6.453037e-04	1.755005e-03	1.759710e-03	1.390271e-03	7.425684e-04	1.144076e-03	8.640117e-04	1.585944e-03	1.566785e-03	1.459771e-03	7.366981e-04	8.332788e-04	8.120064e-04	6.690445e-04	7.342483e-04	6.403424e-04	9.37372	5.51891	6.08273	6.09554	4.36125	6.116547e-04
Phosphoric acid_RT1	IC-MS	3.778771e-02	4.051670e-02	3.726048e-02	3.472069e-02	3.624826e-02	3.487770e-02	2.477639e-02	2.392056e-02	2.786989e-02	3.297604e-02	2.845919e-02	2.997941e-02	2.529546e-02	2.358977e-02	2.464422e-02	3.756073e-02	3.178563e-02	3.367209e-02	3.145513e-02	3.320312e-02	3.144833e-02	3.61586	4.12690	4.13500	3.95792	3.80612	4.367938e-02
Phosphoric acid_RT2	IC-MS	2.977162e-04	2.864001e-04	1.342818e-04	4.418785e-04	4.578947e-04	3.746996e-04	4.178631e-04	2.987580e-04	3.840962e-04	2.662055e-04	4.972735e-04	3.576688e-04	4.353058e-04	4.625048e-04	1.279674e-04	5.209219e-04	5.018666e-04	5.308806e-04	2.621625e-04	2.884852e-04	2.683167e-04	5.55943	5.18401	5.51571	5.07899	4.58109	4.285339e-04
Platelet-activating factor	IC-MS	3.779009e-04	3.620688e-04	3.856657e-04	5.310115e-04	4.735558e-04	4.901733e-04	4.699913e-04	4.239140e-04	3.014988e-04	4.115409e-04	4.318160e-04	3.931204e-04	2.585573e-04	2.730321e-04	2.254288e-04	6.048680e-04	5.930801e-04	6.598132e-04	4.777732e-04	5.298381e-04	4.378272e-04	2.32027	2.66550	2.51056	2.00131	1.90710	1.393232e-04
Propionylcarnitine	IC-MS	2.652843e-04	3.520309e-04	3.650985e-04	2.790974e-04	2.436099e-04	2.299050e-04	4.920751e-04	4.878329e-04	4.144106e-04	1.875170e-04	2.267180e-04	1.672125e-04	2.855252e-04	2.976368e-04	2.626645e-04	3.139900e-04	3.124663e-04	3.131257e-04	4.119989e-04	4.987203e-04	4.930326e-04	2.33182	1.93925	1.94660	2.10124	2.29987	2.206690e-04
Pyrazolidine	IC-MS	2.971578e-04	2.765304e-04	2.734032e-04	2.418078e-04	2.593344e-04	2.451017e-04	2.016761e-04	2.098067e-04	2.102877e-04	2.817258e-04	2.411655e-04	2.679319e-04	1.938764e-04	2.064449e-04	2.004124e-04	2.758720e-04	2.451418e-04	2.419013e-04	2.439006e-04	2.364680e-04	2.357070e-04	2.80018	2.84607	3.04824	2.45717	2.87026	2.799471e-04
Pyridoxine	IC-MS	8.492463e-04	9.597499e-04	8.461804e-04	7.743177e-04	6.367572e-04	6.753007e-04	7.305079e-04	6.403274e-04	5.242968e-04	6.724769e-04	6.813808e-04	7.040454e-04	5.542289e-04	5.272783e-04	5.167325e-04	6.964323e-04	7.901119e-04	6.920734e-04	5.645266e-04	5.610301e-04	5.698492e-04	6.98524	5.71828	5.64416	4.40039	4.62494	3.385869e-04
Pyrogallol	IC-MS	6.355518e-05	8.260855e-05	8.325031e-05	8.711989e-05	9.656999e-05	8.276913e-05	1.292781e-04	1.318975e-04	1.078612e-04	8.346390e-05	9.979650e-05	8.430680e-05	1.155730e-04	1.052670e-04	9.119445e-05	6.669604e-05	8.462388e-05	8.877659e-05	8.929972e-05	1.092671e-04	9.068525e-05	8.12441	5.27709	5.43939	5.27194	4.69154	1.082588e-05
Pyrophosphoric Acid	IC-MS	4.352471e-03	4.671182e-03	3.996504e-03	3.998594e-03	4.298602e-03	4.307333e-03	2.670810e-03	2.622929e-03	3.258231e-03	3.813995e-03	3.276917e-03	3.300577e-03	3.011910e-03	2.689445e-03	3.015551e-03	4.711167e-03	3.768023e-03	4.171075e-03	3.993875e-03	4.200707e-03	4.087081e-03	4.63326	5.69469	5.51855	5.37971	5.23972	6.241808e-03
Pyruvic acid	IC-MS	3.897336e-03	3.836726e-03	1.387699e-03	2.668076e-03	1.775413e-03	2.187542e-03	9.845728e-04	8.797084e-04	1.027713e-03	1.707111e-03	2.346373e-03	1.464608e-03	6.895163e-04	5.832727e-04	1.408413e-03	3.014556e-03	1.925088e-03	1.044383e-03	3.760480e-04	4.679796e-04	6.219643e-04	8.48125	4.40147	2.48366	6.33522	3.23670	4.772607e-04
S-Adenosylhomocysteine	IC-MS	1.737445e-04	1.305859e-04	1.201118e-04	2.225415e-04	1.940248e-04	1.842627e-04	3.208099e-04	2.848063e-04	2.912391e-04	1.973983e-04	2.260024e-04	1.991756e-04	3.026431e-04	3.364280e-04	2.808568e-04	1.810016e-04	2.035358e-04	1.900364e-04	1.752440e-04	1.755862e-04	1.791835e-04	1.85203	1.50997	1.49673	1.37614	1.30774	1.245825e-04
Seryl-4-hydroxyproline	IC-MS	1.634384e-05	1.767791e-05	2.056492e-05	2.420662e-05	2.515175e-05	1.631912e-05	5.068092e-05	4.783359e-05	4.046548e-05	1.889726e-05	2.835057e-05	2.142603e-05	4.476018e-05	5.163472e-05	4.599247e-05	6.689398e-05	7.488378e-05	7.714462e-05	6.266323e-05	6.457924e-05	6.426026e-05	9.02741	7.50828	9.00159	1.19536	9.48220	1.051695e-04
Stearic acid_RT1	IC-MS	3.442226e-01	3.048391e-01	3.056248e-01	2.711067e-01	2.979653e-01	3.177298e-01	2.240402e-01	2.502531e-01	2.825892e-01	2.986736e-01	2.777208e-01	2.799045e-01	2.485920e-01	2.355242e-01	2.665351e-01	2.994958e-01	2.673926e-01	2.752415e-01	3.040928e-01	2.986609e-01	3.186092e-01	3.00259	3.26123	3.24077	3.28506	3.45567	3.691987e-01
Stearic acid_RT2	IC-MS	5.137285e-02	5.541552e-02	5.088774e-02	6.159647e-02	5.676947e-02	5.644573e-02	5.312863e-02	5.057257e-02	5.209220e-02	5.597652e-02	5.147583e-02	6.280747e-02	5.078713e-02	4.368910e-02	5.474541e-02	5.289730e-02	5.955050e-02	5.692773e-02	5.760940e-02	4.810616e-02	5.386479e-02	5.73750	4.99176	5.05140	5.16045	5.11593	4.694810e-02
Styrene	IC-MS	4.803443e-04	6.176405e-04	5.191312e-04	5.608508e-04	4.976878e-04	5.221010e-04	5.374183e-04	4.862239e-04	5.440682e-04	5.980087e-04	5.052740e-04	7.755378e-04	4.806151e-04	5.331439e-04	5.449851e-04	5.134304e-04	5.806096e-04	6.146072e-04	8.182277e-04	5.565294e-04	6.569279e-04	7.02477	6.71176	6.06285	6.12819	6.95761	7.596725e-04
Sulfuric acid_RT1	IC-MS	9.541475e-03	9.383615e-03	8.864531e-03	7.814949e-03	7.702711e-03	7.613502e-03	6.359297e-03	6.238873e-03	6.093239e-03	8.127067e-03	6.847979e-03	7.456377e-03	6.198649e-03	5.820226e-03	5.970047e-03	7.794006e-03	7.128165e-03	7.426336e-03	7.422619e-03	7.425610e-03	7.288364e-03	8.82019	8.63718	8.52779	7.91296	8.55020	8.890917e-03
Sulfuric acid_RT2	IC-MS	2.974037e-03	3.137683e-03	2.869101e-03	2.778674e-03	2.788304e-03	2.443327e-03	1.890868e-03	2.028308e-03	2.272409e-03	2.831340e-03	2.592077e-03	2.532515e-03	3.739463e-03	2.546961e-03	2.601355e-03	2.857646e-03	2.541147e-03	2.696318e-03	2.740772e-03	2.719277e-03	3.606358e-03	3.64964	4.90734	4.17915	5.15410	3.73744	5.733553e-03
Taurine	IC-MS	1.417300e-03	1.669703e-03	1.793514e-03	1.869425e-03	2.017199e-03	1.505310e-03	2.374683e-03	2.555320e-03	2.017662e-03	1.806244e-03	2.247070e-03	1.895902e-03	2.213846e-03	2.347496e-03	2.025729e-03	1.702966e-03	1.578129e-03	1.896451e-03	2.564857e-03	2.349992e-03	2.510326e-03	1.17364	9.86278	1.15030	1.16489	8.55326	9.669510e-04
Thiosulfic acid	IC-MS	2.829008e-04	2.389569e-04	2.778971e-04	2.304966e-04	2.184736e-04	1.860903e-04	1.713565e-04	1.859248e-04	1.742108e-04	2.416710e-04	2.232244e-04	2.138143e-04	1.923732e-04	1.860156e-04	1.748193e-04	1.553378e-04	1.254549e-04	1.448426e-04	1.430670e-04	1.346992e-04	1.395058e-04	1.91446	1.79754	1.74388	1.65822	1.52440	1.680112e-04
trans-10-Heptadecenoic acid	IC-MS	8.620837e-04	9.026336e-04	7.937821e-04	1.386790e-03	1.179449e-03	1.263665e-03	1.638909e-03	1.475290e-03	1.517225e-03	1.324783e-03	1.344369e-03	1.246669e-03	1.383228e-03	1.425916e-03	1.315529e-03	1.881582e-03	2.086967e-03	1.983755e-03	2.089092e-03	2.356109e-03	1.995444e-03	1.57565	1.80618	1.57330	1.63707	1.52787	1.127823e-03
trans-3-Indoleacrylic acid	IC-MS	1.444009e-04	1.742365e-04	1.773106e-04	1.483735e-04	1.333505e-04	1.240558e-04	1.839128e-04	1.643584e-04	1.447541e-04	1.382459e-04	1.592991e-04	1.497752e-04	1.830300e-04	1.787023e-04	1.536476e-04	1.771649e-04	1.950421e-04	1.985959e-04	1.935391e-04	2.201134e-04	2.006555e-04	2.69304	2.29654	2.58832	2.54785	2.42584	2.184617e-04
trans-Aconitic acid	IC-MS	9.471110e-04	9.228273e-04	1.034425e-03	1.311786e-03	1.176196e-03	1.389097e-03	1.200803e-03	1.190389e-03	1.073537e-03	1.011206e-03	1.133030e-03	1.125895e-03	1.048809e-03	1.097140e-03	1.024173e-03	4.352915e-04	4.046804e-04	3.494426e-04	2.764853e-04	2.436327e-04	2.600691e-04	2.51497	2.37745	2.09729	1.78397	1.99639	1.844861e-04
UDP-N-acetylglucosamine	IC-MS	1.614997e-04	1.891412e-04	1.889428e-04	1.748458e-04	1.652429e-04	1.605905e-04	7.768074e-05	1.222256e-04	9.662355e-05	1.530969e-04	1.769370e-04	1.488063e-04	1.142078e-04	1.154046e-04	6.573772e-05	7.377846e-05	5.512295e-05	7.268137e-05	1.116035e-04	7.895347e-05	9.305804e-05	6.53818	5.71089	6.03104	4.45854	5.35331	3.465063e-05
Uracil	IC-MS	6.970307e-03	7.413081e-03	7.326298e-03	7.492408e-03	8.146730e-03	7.112612e-03	7.591212e-03	8.296187e-03	7.276575e-03	6.560704e-03	8.470694e-03	6.756884e-03	7.814427e-03	7.885974e-03	7.701019e-03	1.092144e-02	1.129135e-02	1.117358e-02	9.668973e-03	1.035783e-02	9.111331e-03	1.23723	1.20936	1.34579	1.23110	1.05416	1.281261e-02
Uric acid	IC-MS	1.821198e-04	2.171527e-04	1.770918e-04	1.814894e-04	1.332032e-04	1.595938e-04	2.120211e-04	1.761713e-04	1.649420e-04	1.709252e-04	1.693816e-04	1.870366e-04	1.769765e-04	1.412643e-04	1.662316e-04	1.539650e-04	1.869522e-04	1.579452e-04	1.982426e-04	1.905946e-04	2.135037e-04	1.71719	1.26097	1.17956	1.21770	1.42275	8.389681e-05
vinylglycine	IC-MS	7.573395e-04	1.038902e-03	1.159034e-03	1.045375e-03	1.107611e-03	9.042403e-04	1.417789e-03	1.465241e-03	1.121108e-03	1.147282e-03	1.367888e-03	1.253733e-03	1.302610e-03	1.478374e-03	1.244065e-03	7.900188e-04	7.511693e-04	8.148141e-04	5.517956e-04	6.332890e-04	6.231161e-04	1.24719	1.09456	1.39166	1.14832	9.51337	9.459269e-04
Xanthine	IC-MS	5.316736e-04	5.566758e-04	4.907813e-04	3.749666e-04	3.574035e-04	3.340240e-04	3.680405e-04	3.589287e-04	3.323608e-04	3.580173e-04	3.833449e-04	3.810410e-04	3.526687e-04	3.042359e-04	3.126292e-04	3.372362e-04	3.261637e-04	3.284110e-04	3.145567e-04	3.185251e-04	2.988476e-04	3.87681	3.47272	3.35486	2.97090	4.98189	2.830026e-04
Ximenic acid	IC-MS	5.549265e-04	4.267501e-04	4.423331e-04	6.905655e-04	6.352407e-04	7.229417e-04	1.199778e-03	8.683255e-04	9.062294e-04	7.024381e-04	6.968879e-04	7.034648e-04	1.008718e-03	1.054124e-03	8.964076e-04	8.214115e-04	9.370245e-04	9.149197e-04	8.988882e-04	1.062964e-03	8.188250e-04	4.22002	5.45764	4.25243	3.77364	2.92512	2.453103e-04
Y-Aminobutyric acid (GABA)	IC-MS	6.709867e-04	8.351333e-04	8.869042e-04	8.156270e-04	8.384506e-04	7.335815e-04	8.833894e-04	9.047360e-04	7.633795e-04	8.050662e-04	9.222797e-04	8.017051e-04	8.347098e-04	8.698847e-04	7.965001e-04	5.829858e-04	5.140948e-04	5.625469e-04	3.798537e-04	3.967691e-04	4.042263e-04	8.82559	7.98211	9.41291	7.73307	7.28282	7.442527e-04
Beta-Alanine	IC-MS	6.991005e-04	9.219556e-04	1.057348e-03	1.076856e-03	1.149178e-03	1.049197e-03	2.175224e-03	2.128201e-03	1.889325e-03	1.446321e-03	1.626817e-03	1.419739e-03	2.513006e-03	3.024469e-03	2.577009e-03	1.075361e-03	1.185217e-03	1.271340e-03	1.549561e-03	1.819054e-03	1.880367e-03	1.57193	1.18069	1.68077	1.84645	1.47717	1.587084e-03

Protein Identification		TMT Quantitation															
Gene	Uniprot	DMSO_1	DMSO_2	DMSO_3	DMSO_4	IACS_1	IACS_2	IACS_3	IACS_4	KD025_1	KD025_2	KD025_3	KD025_4	Combo_1	Combo_2	Combo_3	Combo_4
ACTN4	O43707	6.20954	6.12299	5.7296	5.84045	6.29036	6.07775	6.12107	5.99345	6.43828	5.90566	6.00305	6.03694	6.80369	6.76372	6.80971	6.85375
PLEC	Q15149	6.2188	6.25297	5.90772	6.08305	6.13341	6.14663	6.12378	5.99908	6.10885	6.11178	6.14589	6.07038	6.63471	6.71241	6.70668	6.64386
MACF1	Q9UPN3	6.17538	6.28099	6.28471	6.08344	6.12238	6.27919	6.18053	6.3001	6.07643	6.18493	6.30074	6.14924	6.32211	6.4765	6.49728	6.28604
HSP90AA1	P07900-2	6.11965	6.20256	6.50696	5.89883	6.24661	6.58719	6.37366	6.56783	6.34564	6.09355	6.27979	6.32379	5.94805	6.30857	6.07036	6.12697
FLNA	P21333-2	6.17369	6.24872	6.49961	6.06141	6.30643	6.40518	6.01369	6.4621	6.53137	6.41853	6.46469	6.48008	6.03823	6.20556	5.88237	5.80834
VIM	P08670	6.33314	6.41839	5.75826	6.59792	6.1633	5.83302	6.69287	6.03125	6.37424	5.86739	5.91376	6.11599	6.42262	6.41094	6.36226	6.70465
HSPA8	P11142	5.9562	6.0328	6.37764	6.17614	6.167	6.3156	6.4116	6.23498	6.33773	5.9529	6.17352	6.28644	6.20974	6.50954	6.2594	6.59878
LMNA	P02545	6.21947	6.18716	5.60743	6.23531	6.21461	6.0534	6.3744	5.98673	6.01254	5.89633	5.77349	5.71739	7.11893	6.87725	6.90563	6.81992
HSPD1	P10809	6.32505	6.25851	5.85364	6.53207	6.49876	6.08476	6.4324	6.22563	6.30549	5.95614	6.06367	6.04336	6.78167	6.0953	6.22076	6.32279
DST	Q03001	6.22037	6.30533	6.44412	6.31057	6.12753	6.31235	6.24074	6.21168	6.29036	6.35965	6.35961	6.30604	6.07198	6.12975	6.22436	6.08556
HNRNPM	P52272	6.24061	6.21939	5.84563	6.51415	6.24734	5.92159	6.40968	5.81254	6.18692	5.93917	5.91006	5.91179	6.84647	6.52614	6.80541	6.66309
VCP	P55072	6.04258	6.1083	6.1312	6.12729	6.28201	6.25469	6.17282	6.19865	6.24551	6.12918	6.40372	6.39951	6.53967	6.39778	6.29597	6.27112
TUBA1A	Q71U36	6.61156	6.50179	6.99077	6.00877	6.40165	6.91044	6.2018	6.84102	6.41004	6.40131	6.58853	6.61562	5.47846	5.54754	5.10871	5.382
DYNC1H1	Q14204	6.31724	6.14461	6.80585	6.10313	6.30618	6.55402	6.30372	6.41366	6.38904	6.33232	6.57838	6.56938	5.86157	6.00472	5.65903	5.65716
CLTC	Q00610	6.18353	6.29504	6.38594	5.94547	6.27031	6.40247	6.27267	6.37706	6.30245	6.31379	6.30102	6.22676	6.16199	6.12048	6.3851	6.05593
MYOF	Q9NZM1	6.32826	6.58598	6.21638	6.53752	6.07103	6.3279	6.4273	6.16124	6.09378	6.35291	6.20781	6.24582	5.90537	6.22031	6.13812	6.18026
MYH9	P35579	6.26727	6.26921	6.68484	6.02289	6.2643	6.48239	6.32898	6.4809	6.53296	6.28726	6.45947	6.42288	5.84309	6.12569	5.67829	5.84959
LRPPRC	P42704	6.4307	6.51437	5.80903	6.61693	6.41209	6.019	6.53584	6.17031	6.12338	5.92415	5.93299	6.00084	6.44823	6.20768	6.52048	6.33399
HSPA9	P38646	6.41308	6.21456	5.87665	6.53073	6.48346	6.07186	6.26969	6.18383	6.30681	5.91941	6.13891	6.16615	6.94007	6.02086	6.23462	6.22932
SYNE2	Q8WXH0-2	6.44737	6.37679	5.7586	6.60682	6.19282	6.06826	6.33142	6.07515	6.0828	6.21246	5.93829	5.87029	6.75198	6.29385	6.64188	6.35124
SMCHD1	A6NHR9	6.38569	6.45516	5.84585	6.57573	6.25939	6.03438	6.41941	6.06467	6.11609	5.98847	5.7351	5.89042	6.60486	6.47192	6.54982	6.60303
FLNB	O75369-8	6.14571	6.10591	6.19369	5.94173	6.26412	6.27956	6.17217	6.16987	6.34132	6.22541	6.41564	6.33956	6.37272	6.50979	6.28592	6.23687
HSP90B1	P14625	6.27412	6.3257	5.90873	6.24906	6.362	6.12817	6.40036	6.00048	6.07544	5.93089	5.91722	5.84546	6.76797	6.4695	6.76165	6.58325
NUMA1	Q14980	6.38732	6.50171	5.85302	6.60798	6.17749	6.0382	6.29515	5.97889	5.99805	6.0898	5.91592	5.90511	6.51787	6.55989	6.66378	6.50982
UBR4	Q5T4S7	6.25603	6.18603	6.83758	6.10445	6.32624	6.65503	6.30558	6.52373	6.36844	6.41252	6.50863	6.45662	5.71376	5.90966	5.71737	5.71833
EEF1A2	Q05639	6.43611	6.46934	7.00241	6.15156	6.39203	6.68663	6.32049	6.43371	6.55342	6.458	6.51393	6.51168	5.54358	5.60592	5.4795	5.44168
NCL	P19338	5.89424	6.22999	5.71512	6.15033	6.17008	5.98707	6.29328	6.02137	6.10985	6.01151	5.92522	6.01738	6.76313	6.77981	6.92038	7.01124
ACTN1	P12814	6.20282	6.19519	5.91952	5.96778	6.26727	6.15313	6.22572	6.05891	6.62723	5.92474	6.03738	6.11816	6.44405	6.4259	6.62699	6.7052
EEF1A1	P68104	6.42774	6.08791	6.9708	6.18912	6.16802	6.45361	6.70888	6.17328	6.84351	6.07862	6.34429	6.4975	5.47085	5.96641	5.50527	6.11419
PRPF8	Q6P2Q9	6.26485	6.38642	5.93255	6.71294	6.18781	6.01972	6.34416	5.99366	6.15385	6.0917	5.93442	5.9163	6.48448	6.38985	6.52819	6.65911
IQGAP1	P46940	5.97683	6.1344	6.34675	5.69599	6.38617	6.38203	6.04477	6.12511	6.44031	6.36161	6.34075	6.29334	6.29189	6.6444	6.31394	6.22172
PKM	P14618-2	6.1787	6.17552	6.78832	5.92029	6.32487	6.90998	6.11691	6.72921	6.38117	6.64952	6.44447	6.4672	5.71352	5.86955	5.63843	5.69234
ILF3	Q12906-7	6.24068	6.35258	5.9189	6.53628	6.28853	6.08123	6.33345	6.02061	6.31011	5.94472	5.95981	5.8996	6.61928	6.33699	6.64264	6.5146
UTRN	P46939	6.29109	6.53936	6.08639	6.52732	6.21652	6.08531	6.19026	6.15245	6.18207	6.21486	6.15905	6.28755	6.43819	6.21006	6.19194	6.22759
SNRNP200	O75643	6.30938	6.2697	5.91061	6.57974	6.31337	6.07699	6.40565	5.90131	6.24088	5.95535	6.0613	5.91487	6.53336	6.28557	6.5489	6.69303
HNRNPU	Q00839	6.09597	6.11917	5.61314	6.66032	5.97862	5.73861	6.31231	5.8813	6.25633	5.88863	5.87706	6.17253	6.58617	6.92924	6.92719	6.9634
MYH10	P35580-3	6.13104	6.13187	6.58198	5.83477	6.36709	6.41115	6.18153	6.32933	6.41716	6.33539	6.44276	6.1804	6.3074	6.29546	6.05578	5.99688
XPO1	O14980	5.9383	6.10879	5.9557	6.13238	6.02715	6.17751	6.19966	6.09393	6.18868	5.93304	6.01835	6.06864	6.56527	6.83752	6.76049	6.9946
RANBP2	P49792	6.30857	6.36417	5.87915	6.5392	6.28212	6.05435	6.30676	6.00679	6.15536	6.1581	5.97114	5.98196	6.56172	6.3405	6.45169	6.63843
GOLGB1	Q14789-2	6.4944	6.45761	5.99058	6.47167	6.24999	6.10722	6.38721	6.04533	6.12091	6.05245	6.13295	6.13247	6.56471	6.16543	6.34127	6.28579
MAP4	P27816	6.07998	6.0679	6.36621	5.77684	6.07057	6.37074	5.9525	6.57468	6.33351	6.46033	6.49288	6.51449	5.92422	6.76351	5.97285	6.27879
HSPA4	P34932	5.86059	5.95537	6.55848	5.81209	6.11212	6.42418	6.08531	6.13344	6.19472	6.13938	6.33286	6.2019	6.64017	6.53641	6.61375	6.39922
ATP5F1A	P25705	6.38255	6.43005	5.91465	6.51749	6.3133	5.96446	6.49709	6.04154	6.23611	5.91013	5.98192	5.8888	6.7055	6.21903	6.62695	6.37044
COPA	P53621-2	6.28005	6.42129	6.29135	6.062	6.31183	6.48627	6.29088	6.45846	6.26209	6.47275	6.38001	6.33689	5.93185	6.16847	5.92772	5.91808
PRKDC	P78527	5.96878	6.0925	5.89515	6.27506	6.08437	6.01378	6.30435	5.95742	6.14481	6.22452	6.13841	6.0741	6.69595	6.59932	6.72965	6.80184
SPTBN1	Q01082	6.20388	6.31515	6.00783	6.01992	6.18402	6.27914	6.03671	6.17948	6.04903	6.04535	6.02818	5.9157	6.78552	6.54828	6.79394	6.60788
ENO1	P06733	6.12532	6.0871	6.58122	5.74589	6.21962	6.8741	6.39568	6.88699	6.42443	6.27271	6.332	6.39505	5.72409	6.21199	5.57596	5.84481
NPM1	P06748	6.36791	6.23968	5.77974	6.68855	6.43249	5.85273	6.46219	6.11125	6.81139	5.6477	6.06599	6.35392	6.33616	6.14428	6.14563	6.56041
MDN1	Q9NU22	6.47864	6.47899	6.16102	6.94684	6.10012	6.02277	6.34306	5.98467	6.18654	6.03465	6.24183	6.26041	6.11314	6.03849	6.26634	6.34249
MDC1	Q14676	6.25436	6.21915	5.75436	6.74747	6.05376	5.80151	6.31644	6.20061	5.91387	5.89731	5.85268	6.05359	6.32214	7.00472	6.70748	7.099
GAPDH	P04406	6.14789	6.08262	6.71838	5.73778	6.4885	6.62712	6.4455	6.68402	6.47329	6.45351	6.62906	6.45728	5.61648	6.06204	5.74896	5.6276
EIF4G1	Q04637	6.34231	6.4363	6.63316	6.16174	6.2659	6.55596	6.33729	6.41661	6.64574	6.32514	6.5822	6.65883	5.57082	5.79876	5.46938	5.79984
ATP5F1B	P06576	6.21185	6.18226	5.73367	6.50544	6.40837	5.9517	6.56065	5.98023	6.47181	5.8654	5.95769	6.04166	6.56022	6.26324	6.56041	6.7454
FLNC	Q14315	6.25186	6.2243	6.57415	5.96981	6.21597	6.3338	6.05348	6.42181	6.55139	6.44522	6.45011	6.6446	5.98485	6.05282	5.87459	5.95123
SYNE1	Q8NF91-4	6.3779	6.29548	5.98126	6.50555	6.15667	6.02321	6.32264	5.9335	6.21362	6.14578	6.00819	6.05591	6.62979	6.24046	6.57006	6.54
ATP2A2	P16615	6.26378	6.40876	5.94914	6.76343	6.14294	5.94979	6.41444	5.83282	6.14424	5.94465	5.66742	5.81156	6.45129	6.44999	6.90276	6.90299
LARS1	Q9P2J5	6.26975	6.2471	6.53178	5.92925	6.26065	6.56669	6.28883	6.35991	6.28323	6.39964	6.48155	6.37084	6.10519	6.18748	5.93731	5.78079
AHNAK2	Q8IVF2	6.24534	6.37414	6.24756	6.0163	6.17411	6.34112	6.24064	6.30607	6.22358	6.2495	6.48908	6.265	6.21329	6.27461	6.23623	6.10345
IGF2R	P11717	6.40579	6.54887	6.25271	6.40886	6.23505	6.28588	6.22955	6.0073	6.25682	6.49897	6.3641	6.28547	6.02068	6.03278	6.14487	6.0223
HNRNPR	O43390-2	6.32559	6.43554	6.05505	6.52134	6.21367	6.11184	6.34803	6.08651	6.06288	6.08106	5.99616	6.09615	6.43875	6.27058	6.50594	6.45091
EZR	P15311	6.14211	6.16203	6.65612	5.88709	6.29856	6.4704	6.38655	6.42132	6.52967	6.33947	6.37756	6.36625	5.96646	6.11576	6.00794	5.87271
KTN1	Q86UP2	6.31836	6.52302	6.06406	6.60885	6.03416	5.91581	6.27198	5.79197	6.10041	6.04509	5.95842	5.95001	6.48395	6.64559	6.77339	6.51493
HNRNPA1	P09651	6.49725	6.8803	5.68399	7.01016	6.42965	6.33435	6.19466	7.12705	6.14904	5.85536	5.78905	5.84831	5.99629	6.06078	6.19763	5.94613
TPR	P12270	6.57829	6.47745	5.7038	6.53053	6.41666	6.18717	6.10433	6.27765	6.11368	6.04656	5.98474	6.15457	6.82227	6.0781	6.22499	6.2992
UBA1	P22314	5.87732	5.86134	6.44562	5.35324	6.10779	6.60261	5.86019	6.41014	6.05465	6.19647	6.25208	6.18469	6.60731	7.02194	6.60268	6.56193
AKAP9	Q99996-6	6.49591	6.42804	6.50619	6.2511	6.1206	6.5235	6.27973	6.29462	6.27843	6.6034	6.3686	6.41077	5.92951	5.9089	5.8831	5.71763
DNMT1	P26358-2	5.77748	6.32042	6.28895	6.27612	5.96809	6.26549	6.17415	6.07149	6.20703	6.31648	6.48959	6.44179	6.05662	6.51278	6.47028	6.36325
LMNB1	P20700	6.34229	6.422	5.70619	6.62731	6.32631	5.93855	6.39773	5.90162	6.09249	5.95332	5.91362	5.91746	6.74945	6.4633	6.64474	6.60359
CAND1	Q86VP6	5.70527	5.7551	6.1672	5.31771	6.06176	6.42893	5.94997	6.28149	6.0562	6.12535	6.22399	5.9902	6.98245	7.18266	7.02273	6.74899
EPRS1	P07814	6.10044	6.27696	6.2524	5.94041	6.16586	6.57428	6.00035	6.44002	6.1808	6.475	6.12689	6.32103	6.3351	6.62242	6.08599	6.10208
PARP1	P09874	5.56965	5.98002	5.85788	5.74407	5.88741	6.06305	5.97042	5.82535	6.0722	6.20351	6.26907	6.14028	6.93443	7.12782	7.3174	7.03744
HNRNPK	P61978-2	6.20124	6.15518	5.86553	6.13801	6.31465	6.08839	6.09209	6.02653	6.26615	6.04568	6.16889	6.1396	7.04149	6.32417	6.55354	6.56184
SPTAN1	Q13813-3	6.27679	6.32807	6.11756	6.01776	6.29159	6.19734	5.91124	6.27278	6.13592	6.11921	6.16802	6.11943	6.9063	6.28867	6.54618	6.30315
AHNAK	Q09666	6.34386	6.664	6.05637	6.43245	6.18487	6.05941	6.40316	6.21578	6.29126	5.9625	6.06551	6.14175	6.1757	6.39138	6.25038	6.36163
AARS1	P49588	6.39336	6.21437	6.75765	5.81434	6.46801	6.9176	6.22673	6.7709	6.45173	6.47417	6.48064	6.45681	5.83469	5.79912	5.45602	5.48388
CNOT1	A5YKK6	6.21146	6.27497	6.48617	6.0916	6.22307	6.44242	6.25878	6.43123	6.31192	6.42032	6.47472	6.43306	5.99206	6.05325	5.99661	5.89834
SON	P18583-5	6.09288	6.06403	5.76597	6.57731	5.8587	5.75295	6.22874	5.74758	5.91245	5.84398	5.80491	5.81512	6.6231	7.2038	7.19794	7.51055
KPNB1	Q14974	6.16909	6.00157	6.61534	6.34223	6.32129	6.45529	6.35665	6.11734	6.477	6.01685	6.37704	6.53282	6.105	5.94195	5.91566	6.2549
SF3B3	Q15393	6.17741	6.33268	5.93173	6.45835	6.25073	6.09002	6.35262	5.96807	6.20313	6.07968	5.97207	5.99406	6.60369	6.28473	6.72551	6.57552
DDB1	Q16531	5.97011	6.19121	6.15003	6.02735	6.20772	6.1465	6.04448	6.11405	6.04134	6.08874	5.98492	6.02165	6.84047	6.63351	6.90969	6.62823
ADAR	P55265-4	6.36069	6.40517	5.76652	6.57591	6.25538	6.03522	6.26769	6.04786	6.09284	6.00207	5.93181	5.90669	6.69865	6.51181	6.58871	6.55299
DDX39B	Q13838-2	6.41546	6.38623	5.89485	6.53151	6.37642	6.11134	6.40237	5.92814	6.1982	6.06158	6.01415	5.93978	6.6039	6.22184	6.57441	6.33982
H2BC18	Q5QNW6-2	6.43911	6.55731	5.46733	6.61519	6.15506	5.8351	6.58715	6.04204	5.97032	5.58519	5.3698	5.60217	6.37236	7.22069	7.0635	7.11768
VCL	P18206	6.25818	6.20523	6.83491	5.90297	6.34842	6.65648	6.49908	6.59785	6.52008	6.26738	6.37669	6.46034	5.84613	5.96148	5.57063	5.69416
AFDN	P55196-5	6.42337	6.48978	6.16188	6.38491	6.30986	6.15259	6.34686	6.11527	6.33474	6.20561	5.90141	6.01664	6.36001	6.3644	6.28339	6.14928
CAD	P27708	6.35709	6.28797	6.79284	5.99036	6.4698	6.89309	6.23743	6.6566	6.33022	6.32921	6.59636	6.69978	5.55766	5.87441	5.47628	5.4609
CANX	P27824-2	6.20825	6.16771	5.96464	6.33579	6.1071	5.85895	6.33517	5.70211	6.08137	6.14081	6.03209	5.91335	6.98693	6.50842	6.70923	6.94807
GANAB	Q14697-2	6.28532	6.27201	5.81458	6.47345	6.45624	6.28835	6.21781	6.13436	5.91779	5.74017	6.00706	5.8717	6.78984	6.49327	6.67357	6.56447
ACTC1	P68032	6.4941	6.16774	6.15382	5.76807	6.2146	6.84585	6.32566	6.06909	6.32491	5.73083	6.11286	6.32388	6.59424	6.52048	6.60349	6.75038
EPB41L2	O43491	6.12972	6.4802	5.79524	6.28287	6.38625	6.33486	6.27034	6.24218	6.10328	6.03374	5.87994	5.91696	6.49741	6.67612	6.47317	6.4977
HNRNPA2B1	P22626	6.35831	6.26763	5.77974	6.36392	6.35342	5.99173	6.15607	5.91596	6.17939	6.12656	6.18353	6.21934	6.93016	6.18098	6.48342	6.50984
HDLBP	A0A024R4E5	6.6783	6.57619	6.53366	6.51079	6.44263	6.43718	6.33873	6.29481	6.64224	6.53348	6.49882	6.31503	5.71913	5.53699	5.55761	5.38444
TP53BP1	Q12888-2	6.28274	6.45853	5.86541	6.34863	6.28838	6.09891	6.26004	6.01418	5.85896	5.93737	5.8177	5.84563	6.92805	6.70134	6.66542	6.62872
PRRC2C	Q9Y520-7	6.33489	6.48262	6.6486	6.43608	6.3731	6.47823	6.24164	6.63486	6.33315	6.33738	6.6057	6.48628	5.61277	5.81705	5.55084	5.62681
CHD4	A0A2R8Y521	6.30175	6.52786	6.02634	6.60416	6.12307	5.971	6.5348	5.94471	6.0136	6.08408	6.21542	6.06533	6.11325	6.59026	6.51608	6.36828
HUWE1	Q7Z6Z7	6.06616	6.28835	6.35273	6.01687	6.22154	6.4224	6.18573	6.28251	6.14302	6.18001	6.15396	6.18964	6.36017	6.36728	6.37328	6.39633
EFTUD2	Q15029	6.29193	6.22586	5.8321	6.53035	6.32265	6.06492	6.68105	6.17296	6.24928	5.7411	5.92957	6.01129	6.41352	6.31158	6.54278	6.67907
EEF2	P13639	6.25149	6.11903	6.9851	6.14204	6.4208	6.60889	6.33067	6.5212	6.78383	6.22147	6.62474	6.73064	5.49769	5.75791	5.38641	5.61808
DDX5	P17844	6.38852	6.3446	6.09898	6.67175	6.50345	6.2203	6.88423	6.10137	6.36489	5.95863	5.91103	5.9981	6.12757	5.95254	6.22318	6.25086
RAD50	Q92878-2	6.16131	6.12394	5.92387	6.44546	6.31381	6.0044	6.24729	5.84197	6.22273	6.02511	5.99222	5.85482	6.871	6.48661	6.79928	6.68619
VDAC1	P21796	5.6892	6.10586	5.49944	6.31466	6.39652	6.05662	6.54289	6.47139	6.41257	6.33378	6.24424	6.34623	6.1398	6.19505	6.71372	6.53803
TCOF1	Q13428-4	6.11104	6.2281	5.74037	6.54392	5.92987	5.77981	6.33604	5.7917	5.96982	5.84275	5.73827	5.79282	6.47592	7.27139	7.10053	7.34764
SF3B2	Q13435	6.07521	6.36198	5.81198	6.52073	6.13431	5.90476	6.40056	6.02738	6.07965	6.0619	5.89556	6.01027	6.64902	6.62842	6.68046	6.75784

HSP90AB1	P08238	6.18338	6.15397	6.66879	6.07753	6.27416	6.72271	6.32446	6.63182	6.36247	6.18142	6.65663	6.51974	5.85541	5.85951	5.70668	5.82133
GARS1	P41250	6.52497	6.28252	6.59278	6.0488	6.51765	6.67456	6.34181	6.60897	6.28719	6.0882	6.3759	6.37744	5.88732	5.96973	5.72395	5.6982
CCT8	P50990	6.26826	6.1219	6.53374	5.95618	6.47338	6.53484	6.32298	6.5014	6.39484	6.24045	6.41946	6.3235	6.05206	5.96631	6.02896	5.86174
ANLN	Q9NQW6	5.66745	5.99098	6.11585	5.76672	6.08495	6.38464	6.37675	6.17831	6.19768	5.93798	6.3139	6.19654	6.55851	6.61509	6.79474	6.81991
TOP2B	Q02880	6.38477	6.31623	5.85097	6.64695	6.12447	5.90496	6.38697	5.95638	6.06787	5.85887	5.68293	5.62473	6.46228	6.81342	7.04296	6.87523
MATR3	A8MXP9	6.42266	6.43349	6.02314	6.82375	6.26907	6.07509	6.58862	6.07656	6.17386	5.8646	6.01371	5.93368	6.42614	6.27603	6.29109	6.30851
GPI	P06744	6.40971	6.15614	6.91734	5.79989	6.64567	6.90911	6.19566	6.27695	6.19655	6.37058	6.37179	6.38519	5.7187	6.34877	5.70255	5.59541
YWHAE	P62258	6.08127	5.79234	6.61232	5.60878	6.29977	6.48443	6.26999	6.31781	6.45506	6.2341	6.38096	6.30792	6.29069	6.2455	6.39759	6.22147
HNRNPL	P14866	6.28059	6.2333	5.92353	6.65777	6.37034	6.01414	6.17908	6.06432	6.13063	5.88315	6.09187	6.09422	6.91195	6.17456	6.48284	6.50771
VARS1	P26640	6.19788	6.24595	6.67233	5.9312	6.35452	6.33939	6.17237	6.363	6.59643	6.34359	6.37717	6.36379	6.01328	6.24311	5.89746	5.88852
HNRNPC	P07910	6.12945	6.40646	5.96233	6.79846	6.32424	6.31823	6.62228	5.90832	6.15825	6.00324	5.87248	5.78221	6.52375	6.34668	6.50321	6.34042
DDX17	Q92841	6.25625	6.30714	5.84301	6.49278	6.29787	6.09177	6.44873	6.01374	6.07472	6.07718	6.08631	5.93661	6.83832	6.3055	6.5106	6.41946
RRBP1	Q9P2E9	6.27832	6.56018	5.78596	6.39435	6.10008	5.92003	6.13417	6.00505	6.02249	6.14502	5.85752	5.79835	6.21528	7.09073	6.75188	6.94058
P4HB	P07237	6.58323	6.29759	5.91687	6.36192	6.5305	6.15299	6.10215	6.32794	6.20023	6.02394	6.09541	6.09624	6.8215	6.14402	6.17371	6.17175
TPI1	P60174-3	6.29374	6.04284	6.76179	5.65067	6.55627	6.83281	5.94707	6.6256	6.24728	6.4471	6.31651	6.27788	6.07497	6.26559	5.84478	5.81513
KHSRP	Q92945	6.36252	5.70835	6.03451	5.47928	6.74836	5.70926	5.8348	6.49638	6.08242	6.11456	6.35973	6.6045	7.2255	6.31122	6.36388	6.56474
HSPA5	P11021	6.21656	6.2095	5.83182	6.15796	6.5017	6.0645	6.36636	6.1501	6.26606	5.96959	5.78915	5.82202	6.97448	6.51639	6.57186	6.59197
RIF1	Q5UIP0	6.51858	6.50507	6.05682	7.05956	6.14483	5.87639	6.25151	6.00221	6.17439	5.97172	5.78502	6.13553	6.30736	6.18752	6.43549	6.58801
HADHA	P40939	6.33117	6.28678	5.78547	6.55548	6.26862	6.05315	6.41616	5.96861	6.23002	6.02674	5.95833	5.8698	6.64221	6.37173	6.73707	6.49865
CCT7	Q99832	6.19241	5.96446	6.68049	5.94705	6.4695	6.53425	6.54401	6.3393	6.58796	6.11867	6.37915	6.26967	5.98852	6.02459	5.95686	6.00312
DHX9	Q08211	6.31144	6.45659	5.94575	6.67174	6.16383	6.08628	6.30623	5.81378	6.14643	6.0479	6.07046	6.01893	6.57042	6.23753	6.63367	6.51903
FASN	P49327	6.33486	6.16153	6.90536	6.01021	6.48296	6.7806	6.42596	6.69713	6.69296	6.38643	6.72634	6.58657	5.45612	5.56107	5.39969	5.39221
GOLGA4	Q13439-5	6.66455	6.66324	6.24684	6.47926	6.46996	6.17397	6.12549	6.0542	6.63532	6.36726	6.3752	6.41384	6.35643	5.66335	5.6157	5.69539
SF3B1	O75533	6.23436	6.3462	5.8083	6.47234	6.21987	5.9503	6.52149	5.93855	6.20791	5.83023	5.78736	5.80421	6.84338	6.53731	6.73199	6.76618
NONO	Q15233	6.32943	6.36831	5.91968	6.56984	6.30407	6.04381	6.51203	6.03565	6.13564	5.98495	5.82235	5.98404	6.46527	6.35619	6.49532	6.67342
PHB2	Q99623	6.40964	6.44492	5.81288	6.54975	6.2785	5.98794	6.4682	5.94904	6.08035	6.05589	5.83636	5.90158	6.58276	6.41806	6.67955	6.54458
MKI67	P46013	6.47028	6.46798	5.95445	7.06607	6.03194	5.88346	6.35712	6.20258	6.10162	6.07876	6.11886	6.32835	5.76838	6.40057	6.27136	6.49823
DDX3X	O00571	6.37132	6.51441	6.58926	6.5289	6.41754	6.55255	6.40727	6.4178	6.44717	6.53304	6.40843	6.42307	5.68604	5.55882	5.4976	5.6468
XRCC5	P13010	5.69337	6.00282	5.98646	5.90958	6.09845	6.02993	6.12732	5.82554	5.98305	5.85667	6.2452	6.02776	6.96252	7.02638	7.23422	6.99073
YLPM1	P49750	6.42562	6.29612	6.0078	6.65997	6.16515	5.96606	6.43531	6.01275	6.21541	6.01557	6.13036	6.07746	6.59341	6.27171	6.36229	6.36499
HSPA1B	P0DMV9	5.8831	6.08182	6.25048	5.82786	6.08132	6.43115	6.13304	6.21	6.3104	6.03809	6.32234	6.22595	6.60789	6.58822	6.58187	6.42647
TUBB3	Q13509	6.42613	6.27977	6.88428	6.15749	6.48399	6.97718	6.29216	7.13267	6.42524	6.43752	6.66213	6.74705	5.33559	5.56152	4.92408	5.2732
TOP1	P11387	6.11403	5.98126	5.69491	6.31159	5.91692	5.67656	6.36608	5.79505	5.92505	5.81071	5.86033	5.69371	6.52919	7.42155	7.29224	7.61081
RPS2	P15880	6.42167	6.21279	6.50332	6.32979	6.32444	6.46136	6.19763	6.23081	6.41424	6.49044	6.58093	6.4291	5.82233	5.92067	5.76769	5.89278
ACO2	Q99798	6.42695	6.42846	5.93596	6.57423	6.36824	6.13697	6.26941	6.19895	6.09708	6.03166	6.17368	5.90182	6.71691	6.17984	6.3944	6.16544
GLUD1	P00367	6.27506	6.26459	5.64356	6.39993	6.47648	5.98376	6.43902	6.09188	6.35771	5.94222	5.91962	5.84472	6.84793	6.20832	6.83283	6.47236
MYBBP1A	Q9BQG0-2	6.04968	5.94836	5.59511	6.65163	6.065	5.78725	6.45941	6.04465	6.25494	5.87816	6.01013	6.09192	6.59611	6.72657	6.74095	7.10014
PABPC4	Q13310-3	6.10153	6.52678	6.5851	6.21388	6.31958	6.62369	6.33877	6.59072	6.40451	6.3575	6.57617	6.55895	5.69173	5.92721	5.56268	5.62121
RPS3	P23396	6.58309	6.58382	6.56906	6.0829	6.57359	6.59313	6.33884	6.41494	6.56337	6.54325	6.3823	6.21331	5.80721	5.55202	5.75047	5.44868
TJP1	Q07157	6.43153	6.53185	5.99203	6.45708	6.30093	6.33383	6.01814	6.39122	6.23491	6.09261	6.09528	6.17824	6.27944	6.24991	6.27421	6.1388
MCM4	P33991	5.59995	6.26954	5.82562	5.82057	6.06159	6.41488	5.85426	6.27103	5.83965	6.1122	6.05481	5.99537	6.91496	7.14472	6.90036	6.9205
EIF4A2	Q14240-2	6.46133	6.59352	6.87609	6.39295	6.51091	6.81027	6.60711	6.55558	6.69324	6.57196	6.61611	6.64364	5.11737	5.37465	5.03422	5.14105
TCP1	P17987	6.16519	6.28188	6.44607	5.92529	6.528	6.81538	6.30023	6.65519	6.48313	6.12519	6.31642	6.19979	5.87934	6.08546	5.93001	5.86343
SACS	Q9NZJ4	6.34984	6.21039	6.80778	6.31998	6.16912	6.47787	6.05942	6.2835	6.60944	6.52701	6.61434	6.69255	5.56957	5.92899	5.69759	5.68263
USP7	Q93009	5.98169	6.10331	5.85158	6.09941	6.1772	6.13393	6.21195	5.98222	6.07129	6.06058	5.95886	5.88268	6.91373	6.68432	7.04072	6.84654
ACTB	A0A2R8Y793	6.42265	6.23267	6.2054	6.28686	6.74806	6.37883	6.11828	6.38174	6.36313	5.82006	5.85692	6.1758	6.13085	6.26776	6.34491	6.26608
NT5E	P21589	6.12554	6.27622	5.75635	6.20065	6.24067	6.26216	6.17911	6.27011	6.02217	6.18498	6.05712	5.94161	6.64603	6.81541	6.61353	6.40834
IMMT	Q16891-4	6.49096	6.29383	5.91393	6.51274	6.4352	6.01092	6.30322	6.15221	6.13674	5.96063	6.02135	5.93015	6.79123	6.2771	6.40354	6.36626
NUP214	P35658	6.3865	6.11066	6.08315	6.23887	6.24834	6.05504	6.10716	6.09827	6.35133	6.12119	6.0907	6.18746	6.63779	6.24548	6.39333	6.64472
PDS5B	Q9NTI5	5.98357	6.06425	5.78059	6.27319	6.00564	5.91018	6.19122	5.84578	5.92779	5.95892	5.92459	5.76499	6.8806	7.29086	7.12395	7.07386
CCT3	P49368	6.22893	6.10318	6.66952	5.95083	6.46903	6.70685	6.32369	6.5747	6.27376	6.28646	6.37605	6.36302	6.0025	6.06177	5.81077	5.79896
RNF213	Q63HN8-4	6.1029	6.34143	6.54199	6.05884	6.14587	6.44089	6.16221	6.21036	6.30516	6.48183	6.37434	6.28343	6.02361	6.42673	6.0128	6.08762
HNRNPUL1	Q9BUJ2	6.08446	6.24201	5.56248	6.4303	6.17226	5.87977	6.68272	5.67836	6.16202	5.72976	5.59894	5.66435	6.64467	7.04323	6.99685	7.42782
HSPH1	Q92598	6.05358	6.15365	6.64731	5.86357	6.28517	6.56307	6.0881	6.27395	6.14707	6.29937	6.47733	6.34149	6.39419	6.1514	6.2205	6.04024
ECPAS	Q5VYK3	6.2467	6.06277	6.47153	5.88672	6.28959	6.56627	6.27869	6.3942	6.23199	6.40584	6.52586	6.38871	6.22663	6.13147	5.98662	5.90641
VDAC2	P45880	5.9647	5.95198	5.67035	6.47999	6.39661	6.12061	6.40769	6.10455	6.356	6.18359	6.36332	6.19924	6.30186	6.09495	6.71128	6.69327
ALDOA	P04075-2	6.34354	6.11585	6.68789	5.68631	6.65503	6.94558	6.00311	6.69792	6.50943	6.36666	6.52044	6.43577	5.98919	5.98196	5.48623	5.57509
TRIM28	Q13263	5.97744	6.20925	5.83318	6.33182	6.26384	6.11507	6.15563	6.08327	5.99462	6.11748	6.04347	6.02932	6.7964	6.65673	6.683	6.70948
SMC1A	Q14683	6.256	6.51107	5.86368	6.38287	6.19711	6.16827	6.3989	6.17136	6.02887	5.9503	5.82701	5.77874	6.60701	6.54913	6.65546	6.65422
DARS1	P14868	6.19156	6.24329	6.53663	5.81297	6.47326	6.49024	6.18041	6.49207	6.22708	6.48071	6.3719	6.25147	6.13526	6.28364	6.06417	5.76534
GCN1	Q92616	6.42609	6.42833	6.66937	6.10737	6.33612	6.68799	6.23035	6.8245	6.47259	6.41136	6.62791	6.55109	5.54411	5.84341	5.43431	5.40511
EIF4G2	P78344	6.33816	6.49819	6.53734	6.30048	6.50356	6.66745	6.21403	6.53568	6.33965	6.36897	6.46501	6.38296	5.7272	5.84444	5.64295	5.63392
TMPO	P42166	6.01026	6.41574	5.92929	6.69837	6.04496	6.17424	6.08615	6.07295	5.98049	5.96167	6.24829	6.1096	6.55795	6.46319	6.79428	6.45256
CALR	P27797	6.30393	6.20611	6.07774	6.46048	6.35774	5.90356	6.08646	5.79917	5.9473	6.09372	6.05468	5.95647	7.0143	6.28631	6.57349	6.87854
STIP1	P31948-2	6.24698	6.26122	6.45844	5.71368	6.50571	6.66443	5.97828	6.67163	6.25335	6.35792	6.54778	6.39436	6.4731	5.99588	5.73819	5.73905
CCAR2	Q8N163	6.36523	6.16882	6.07026	6.47431	6.37022	5.96259	6.21407	6.04384	6.31032	5.90944	5.99118	6.00002	6.98339	6.25805	6.52127	6.35698
SFPQ	P23246	6.30772	6.1187	5.97722	6.69852	6.17809	5.66411	6.67935	5.91311	6.48489	5.64992	5.95667	6.03405	6.50802	6.33594	6.78877	6.70493
NASP	P49321	5.53318	5.79266	6.01147	5.25444	6.0239	6.32671	5.76409	6.1417	5.84248	6.03325	6.28165	6.19084	7.72086	7.05929	7.15816	6.86533
TRAP1	Q12931	6.32707	6.335	5.99241	6.69187	6.32444	5.99017	6.4564	5.96098	6.07573	5.89285	6.0212	5.95822	6.54171	6.30322	6.62882	6.49992
SYNCRIP	O60506	6.18744	6.22612	6.10264	6.38459	6.27092	6.21933	6.31518	6.22201	6.38668	6.11084	6.19113	6.10533	6.16533	6.35816	6.39457	6.35971
NUP98	P52948	6.43466	6.29582	5.81803	6.54252	6.42175	6.14172	6.23357	5.9047	6.1316	6.1827	6.05985	5.98828	6.77535	6.30817	6.35397	6.40731
XRCC6	P12956	5.8498	6.11975	5.88678	5.84701	5.95124	6.08007	6.07199	5.86791	5.90432	6.07441	6.00937	5.92605	7.03846	7.07273	7.39379	6.90633
PSMD2	Q13200	6.17691	6.26667	6.37902	6.05094	6.30141	6.58775	6.27993	6.46165	6.23458	6.33825	6.34933	6.20888	6.14326	6.08423	6.04865	6.08853
TLN1	Q9Y490	6.37951	6.18675	6.71094	5.74296	6.4181	6.65622	6.25143	6.46185	6.54978	6.36361	6.50036	6.44995	6.09824	5.92451	5.7026	5.60319
NSF	P46459	6.24962	6.15183	5.91148	6.08895	6.16672	6.27233	6.22714	6.27105	6.22264	6.07904	6.14746	5.93173	6.58256	6.34957	6.53982	6.80807
PDIA3	P30101	6.45354	6.28599	5.88489	6.18987	6.48053	6.04859	6.29881	6.02458	6.07897	5.95104	6.08059	5.82318	7.10002	6.45143	6.5697	6.27827
NUP93	Q8N1F7	6.27759	6.22486	6.03115	6.49482	6.13055	6.08868	6.32433	6.16202	6.29248	6.13758	6.17294	6.05737	6.54762	6.09913	6.59973	6.35913
MCM7	P33993	5.63031	6.06056	6.10866	5.95755	6.02244	6.20761	6.12846	6.14175	6.09105	6.13372	6.22922	6.0501	6.61469	6.77665	6.90215	6.94509
ACADVL	P49748-3	6.28327	6.38507	5.9201	6.52999	6.31945	6.08096	6.51846	6.1078	6.05834	5.99667	5.93892	5.95319	6.62867	6.35181	6.59612	6.33119
RPN1	P04843	6.34961	6.38615	5.85391	6.48916	6.27333	6.14335	6.29939	6.0361	6.16265	6.05671	5.89746	5.82006	6.60592	6.42365	6.70118	6.50139
GLG1	Q92896-2	6.58541	6.33875	5.92198	6.53602	6.54519	6.19056	6.46841	5.94067	6.37272	5.96392	5.88738	5.72652	6.49088	6.29041	6.37242	6.36876
LDHB	P07195	6.38274	5.99268	7.00318	5.75525	6.49252	6.6228	6.41784	6.51088	6.48716	6.16229	6.4874	6.34193	5.81532	5.92316	5.80609	5.79877
ZNF638	Q14966	6.14793	6.33774	5.98226	6.91987	6.06798	5.99944	6.38773	6.0811	6.01572	6.00865	5.85526	5.82767	6.39692	6.51531	6.87798	6.57843
LAP3	P28838	6.45888	6.49596	6.17352	6.51408	6.66808	6.28613	6.36708	6.39412	6.29918	6.10415	6.23009	6.06263	6.2654	5.78673	6.04552	5.84847
PA2G4	Q9UQ80	5.99924	6.04847	6.55362	5.97814	6.28739	6.5904	6.38118	6.33357	6.62968	6.3381	6.67357	6.66119	5.67783	6.09321	5.85588	5.8985
AP2B1	P63010-2	6.28456	6.30494	6.37335	6.26222	6.34536	6.40276	6.36698	6.30388	6.30571	6.13346	6.28157	6.20007	6.117	6.18253	6.08621	6.04941
AKAP12	Q02952	6.63714	6.51003	6.49312	6.3354	6.32547	6.09123	5.97865	6.2165	6.2925	6.02322	6.38028	6.49041	6.55703	5.82606	5.92771	5.91525
POLR2A	P24928	6.68142	6.7811	6.28837	7.02665	6.32471	6.23885	6.57284	6.3168	6.52804	6.3259	6.28436	6.14581	5.62689	5.58553	5.60721	5.66552
IARS1	P41252	6.30621	6.08379	6.42407	5.78105	6.5711	6.39154	6.17077	6.1494	6.39243	6.49228	6.47494	6.28028	6.30705	6.26372	5.95374	5.95764
SMARCA1	P28370	6.54216	6.59888	5.99223	6.77866	6.1863	6.08232	6.46072	6.00593	6.12648	6.03203	5.89734	5.99941	6.44518	6.1487	6.42901	6.27465
MSN	P26038	6.05128	6.01075	6.42559	5.83094	6.33072	6.48526	6.21392	6.22585	6.59981	6.49942	6.45341	6.50872	6.00529	6.15865	6.13456	6.06584
DDX46	Q7L014	5.916	6.14878	5.92201	6.01247	6.15945	5.99105	6.19676	5.82661	6.18408	6.08101	5.92525	5.83243	6.76361	6.87229	7.15669	7.01151
SRRM2	Q9UQ35	6.266	6.22791	5.97974	6.5739	6.18304	5.99114	6.47694	5.80086	6.34433	5.82364	6.06063	5.99105	6.53698	6.5468	6.40522	6.79182
MAP1B	P46821	5.93507	5.99692	6.37863	5.70503	6.07857	6.428	5.96109	6.29628	6.07904	6.49288	6.422	6.4251	5.95993	7.003	6.28212	6.55635
DIDO1	Q9BTC0	6.23828	6.32513	5.91241	6.74275	6.00324	6.0242	6.32103	5.79348	6.06627	6.1849	5.90478	6.02351	6.62463	6.53922	6.58571	6.71045
PSMD1	Q99460	6.17675	6.27608	6.32726	6.22276	6.32528	6.37493	6.12151	6.3451	6.11178	6.20342	6.26308	6.20491	6.37171	6.18898	6.23295	6.2535
TRRAP	Q9Y4A5	6.52814	6.69869	6.10521	7.06899	6.19771	6.04261	6.48689	6.09378	6.55167	6.48965	6.30528	6.24976	5.88304	5.54571	5.89353	5.85933
RACK1	P63244	6.36441	6.5008	6.56349	6.18189	6.51787	6.44733	6.17539	6.54652	6.52813	6.60972	6.61928	6.57431	5.62852	5.59183	5.60678	5.54373
NPEPPS	P55786	6.3442	6.08706	6.75799	5.72576	6.34531	6.60676	6.2385	6.56759	6.30629	6.52205	6.41278	6.44209	5.89173	6.1453	5.78935	5.81724
SMC4	Q9NTJ3	6.50376	6.41433	6.93341	6.43748	6.37401	6.52162	6.34445	6.22926	6.48418	6.56067	6.53309	6.47035	5.62987	5.54274	5.58362	5.43717
H4C1	P62805	6.21044	6.24154	5.36345	6.48863	6.16784	6.02917	6.52394	6.35497	5.96914	5.9009	5.57114	5.91772	6.78353	6.88812	6.48105	7.10844
HERC2	O95714	6.21732	6.24482	6.57823	6.34865	6.09751	6.3414	6.40886	6.32704	6.45563	6.60857	6.64086	6.67542	5.69983	5.88261	5.66524	5.808
OPA1	O60313-2	6.46724	6.36222	5.87346	6.62756	6.39179	5.99986	6.49354	6.06649	6.20581	5.95819	6.02417	5.87314	6.62866	6.145	6.44092	6.44195
CCT2	P78371	6.15133	6.1335	6.61085	6.05346	6.5033	6.81305	6.40209	6.64417	6.50352	6.0188	6.45551	6.43786	5.79554	5.96431	5.72312	5.7896
ACIN1	Q9UKV3-5	6.26468	6.19668	5.85673	6.62225	6.14976	5.87947	6.26853	6.10791	6.12003	6.01174	5.95704	6.06088	6.81947	6.485	6.47024	6.72958

HEATR1	Q9H583	6.29583	6.15205	5.76517	6.62592	6.34888	5.96482	6.52113	5.86647	6.25454	5.84871	5.94135	5.84334	6.62968	6.42046	6.63022	6.89143
CKAP5	Q14008	6.19109	6.32374	6.57248	6.20712	6.26589	6.50851	6.27521	6.3924	6.43335	6.65506	6.53755	6.50856	5.80695	5.88548	5.62767	5.80894
OGDH	Q02218	6.3243	5.97638	5.89163	6.51623	6.37503	5.7708	6.88903	5.73875	6.46023	5.67106	5.71216	5.70261	6.53665	6.55299	6.77957	7.10258
MDH2	P40926	6.40195	6.37021	5.77348	6.39007	6.41853	6.08388	6.33843	6.20701	6.215	6.11292	6.0961	6.03776	6.77745	6.194	6.32877	6.25445
XPO5	Q9HAV4	5.93525	5.88917	6.07919	5.59856	5.99784	6.16697	5.96861	5.99437	6.41475	6.26983	6.27941	6.14847	6.75089	6.93091	6.82475	6.75105
CCT6A	P40227	6.17705	6.26864	6.63006	6.02898	6.40091	6.61194	6.28146	6.48115	6.30538	6.25538	6.6154	6.48535	5.96533	5.87113	5.72804	5.89381
TRIP11	Q15643	6.58426	6.36395	6.22329	6.63573	6.29804	6.16822	6.27294	6.14511	6.42772	6.07312	6.49114	6.38498	6.65144	5.72816	5.85808	5.69381
GTF2I	P78347	6.10058	6.27674	5.7745	6.602	6.05638	5.76023	6.70439	6.12928	6.07608	5.7655	5.77854	6.27846	6.33806	6.75935	6.51802	7.08189
NSUN2	Q08J23	5.31617	5.76386	6.03762	5.27412	5.7483	6.17683	5.75253	5.92532	5.91443	5.91687	6.12909	6.11236	7.2116	7.54411	7.69921	7.47758
RPL4	P36578	6.18396	6.17832	5.68209	5.83963	6.02772	6.21627	6.29121	6.63464	6.52649	6.13692	6.69112	6.8106	5.97156	6.92957	5.76441	6.11548
CSE1L	P55060	5.64407	5.94423	6.15879	5.7728	6.04494	6.21648	5.93381	6.22504	6.34126	6.05147	6.18322	6.19749	6.36082	7.0504	6.77256	7.10262
UBR5	O95071	6.29847	6.35528	6.15399	6.38528	6.42083	6.23765	6.39543	6.14636	6.17628	6.03874	5.91891	6.04973	6.50788	6.23023	6.29942	6.38552
BIRC6	Q9NR09	6.34111	6.31856	6.97018	6.08195	6.31284	6.74678	6.17408	6.56299	6.389	6.65548	6.35589	6.43088	5.68098	5.78993	5.64753	5.74183
PCNT	O95613	6.28481	6.39325	6.40441	6.3765	6.10409	6.29477	6.12477	6.15977	6.27619	6.38337	6.38012	6.27211	6.2007	6.18033	6.10591	6.0589
HYOU1	Q9Y4L1	6.22265	6.46196	5.97077	6.44991	6.38088	6.26926	6.38413	6.16411	6.01868	5.93309	6.03117	5.92428	6.61264	6.36702	6.48423	6.32521
NOMO3	P69849	6.45285	6.45659	5.96985	6.69099	6.26354	6.16619	6.47607	6.05847	6.15936	5.98701	5.93852	5.9202	6.33705	6.11515	6.58888	6.41928
ACACA	Q13085-4	6.47266	6.4891	7.01843	6.26138	6.23193	6.4689	6.23689	6.58497	6.58694	6.57571	6.56394	6.60114	5.43834	5.58432	5.39424	5.49111
PUF60	Q9UHX1-2	6.27342	6.18319	6.06079	6.34306	6.38108	6.01174	6.39749	6.05553	6.35505	5.70133	5.82043	6.05014	6.45604	6.54567	6.59801	6.76704
DDX1	Q92499	5.74196	6.08052	6.11815	5.72102	5.93805	6.23097	6.08237	6.20477	6.08597	6.21988	6.41212	6.08844	6.80471	6.78119	6.7795	6.71037
BPTF	Q12830	6.39615	6.47196	6.04353	6.89843	6.00839	5.99185	6.39152	5.79365	6.09974	6.20973	6.11708	6.1354	6.26473	6.53376	6.31685	6.32723
EIF3I	Q13347	6.00858	5.96888	6.5438	6.01519	6.56177	6.74928	6.25046	6.89348	6.62533	6.86069	7.00849	7.07544	5.21037	5.4033	5.25041	5.57453
CPSF1	Q10570	6.4673	6.58878	6.23543	6.85744	6.14308	6.06335	6.26824	5.89434	5.99951	6.17936	6.08176	6.126	6.36297	6.07541	6.37147	6.28557
LMNB2	Q03252	6.40969	6.3653	5.92646	6.72154	6.18193	6.07322	6.33695	5.96907	6.5428	5.76837	5.76975	5.89288	6.6179	6.3394	6.53731	6.54744
CHD3	Q12873-3	6.3454	6.48486	5.98742	6.6992	6.31239	5.94033	6.39096	6.10734	6.12248	6.06579	5.91971	5.85505	6.24238	6.72634	6.4383	6.36205
PHF3	Q92576	6.29052	6.52419	5.96294	6.93713	6.04338	5.99059	6.40314	6.00742	6.15987	6.34408	5.98649	6.16685	6.0818	6.37685	6.36501	6.35973
MTHFD1	P11586	6.22979	6.0157	6.75182	5.70782	6.4807	6.74025	6.14891	6.75473	6.43535	6.4457	6.70577	6.70141	5.92433	5.84859	5.58067	5.52847
PGK1	P00558	6.0583	5.74905	6.49343	5.68305	6.47617	6.65952	6.46426	6.4963	6.57035	6.10501	6.31496	6.46855	5.97817	6.52303	5.7965	6.16333
EIF3A	Q14152	6.35806	6.5886	6.73116	6.20223	6.31449	6.51702	6.3406	6.28166	6.42794	6.56995	6.54182	6.57325	5.55789	5.80696	5.54038	6.54798
ACLY	P53396	5.93594	6.19691	6.69301	5.69569	6.1201	6.68168	6.00249	6.42034	6.29555	6.27525	6.48344	6.35145	6.0929	6.43648	6.19405	6.12472
ASCC3	Q8N3C0	6.32585	6.22349	6.50544	6.0623	6.39506	6.58709	6.16377	6.4203	6.23657	6.40524	6.31993	6.32315	6.01139	6.08381	5.89527	6.04134
CCT5	P48643	6.25453	6.09748	6.3921	6.0799	6.57424	6.68549	6.49829	6.47716	6.5754	6.92385	6.20261	6.26554	5.81803	6.13734	5.94221	6.07584
CUL4B	Q13620	5.84939	6.24973	6.07509	6.04703	6.1149	6.45586	6.12767	6.06255	6.08799	6.30032	6.22198	6.11526	6.50317	6.59259	6.68809	6.50838
MCM5	P33992	5.6355	6.08704	5.82565	5.85587	5.90709	6.1221	6.10462	6.02396	5.96514	6.02744	5.96353	5.76655	7.04044	7.13086	7.51401	7.0302
KMT2A	Q03164-3	6.40868	6.47257	5.98259	6.91383	6.18287	6.04987	6.49917	6.08018	6.27393	6.21183	6.07173	6.04167	5.90962	6.37921	6.3102	6.21205
GPD2	P43304	6.33903	6.17483	5.79105	6.44013	6.30015	5.98309	6.39994	6.20578	6.16311	5.97405	5.88072	5.69032	6.73409	6.40099	6.70213	6.82058
PPFIBP1	Q86W92	6.02049	6.01254	6.15893	5.89385	6.26924	6.14166	6.10198	6.00125	6.55945	6.18734	6.1484	6.01787	7.03354	6.69973	6.52402	6.22971
KARS1	Q15046-2	6.36462	6.6104	6.49892	6.28773	6.52055	6.60676	6.27885	6.64107	6.26539	6.20081	5.99783	6.2347	5.75987	6.12394	5.70226	5.90632
PPIA	P62937	5.50948	5.70902	6.1675	5.56254	6.63245	6.40513	6.38985	7.03285	6.78502	6.39424	6.54463	6.87911	5.54157	6.19782	5.78553	6.46326
POR	P16435	6.3529	6.51097	5.98463	6.65719	6.00463	6.06102	6.25162	5.87642	6.08214	5.99435	6.11304	5.97984	6.56505	6.36877	6.63527	6.56217
MSH6	P52701	5.3231	5.68566	5.84529	5.25587	5.73863	6.28866	5.91443	5.82585	5.97317	6.03205	6.07278	5.86171	7.32392	7.5739	7.79871	7.48628
SND1	Q7KZF4	6.37115	6.4208	6.15501	6.37744	6.34768	6.24652	6.27889	6.4553	6.27928	6.21885	6.31898	6.2102	6.04009	6.21403	5.99674	6.06904
MARS1	P56192	6.08612	6.07275	6.55376	5.84937	6.33322	6.41078	6.28734	6.25151	6.47235	6.42238	6.40532	6.27861	6.23813	6.21346	6.19038	5.93452
NCAPD2	Q15021	6.49385	6.66188	6.96051	6.65732	6.30282	6.54515	6.38691	6.73252	6.65227	6.70405	6.86598	6.91715	4.99754	5.21649	4.70089	5.20466
SSB	P05455	5.20363	5.70096	5.79282	5.1978	5.52916	6.0567	5.81033	5.9036	5.73251	6.04689	5.92933	5.7546	7.46084	7.93432	8.24927	7.70416
CLIP1	P30622	6.38804	6.40517	7.00471	6.28658	6.62685	6.69015	6.11138	6.86168	6.53313	6.38597	6.51503	6.8229	5.65437	5.31708	5.16887	5.2281
GBF1	Q92538	6.0883	6.03086	6.27114	5.92644	6.24379	6.35282	6.07156	6.36519	6.26359	6.29878	6.25388	6.28888	6.24639	6.4361	6.36442	6.49786
NBAS	A2RRP1	6.36877	6.41161	6.02598	6.4852	6.23933	6.06673	6.4644	5.88643	6.02767	6.13524	6.08991	5.90857	6.47517	6.2788	6.62873	6.50748
GART	P22102	6.30733	6.30215	6.94813	6.19894	6.464	6.85516	6.27307	6.74442	6.34545	6.27007	6.42426	6.57507	5.56233	5.80096	5.3661	5.56254
PDS5A	Q29RF7	6.061	6.00845	5.9662	6.34115	6.2721	6.15149	6.28241	5.91638	5.92514	6.12208	6.14796	6.04792	6.6531	6.73269	6.63393	6.738
HCFC1	P51610-4	6.26834	6.26009	5.79104	6.36659	6.48395	6.32807	6.09595	6.13034	5.99061	6.10818	5.87528	5.96686	6.95417	6.46139	6.4822	6.43692
TUFM	P49411	6.28057	6.25924	5.72379	6.52521	6.38039	6.25014	6.29604	6.30098	6.11046	5.97608	5.87942	6.03615	6.57769	6.5279	6.42652	6.44941
SEPTIN9	Q9UHD8	6.13313	6.131	6.37881	5.93843	6.41429	6.53621	6.18829	6.38673	6.29521	6.47887	6.27889	6.21837	6.40118	6.25094	6.02098	5.94869
TDRD12	A0A2R8Y872	6.56314	6.47623	7.42659	6.59851	6.44184	6.81409	6.37931	6.61561	6.7155	6.8306	6.73415	6.99958	4.84884	4.96503	4.73237	4.85862
NAT10	Q9H0A0	6.3625	6.34055	5.90377	6.76924	6.07234	5.79424	6.48232	5.94867	6.13305	5.76219	5.97316	6.00209	6.44893	6.38756	6.91889	6.70051
FRYL	O94915	6.14706	6.07549	6.63087	5.86585	6.23907	6.58901	6.21686	6.57893	6.40027	6.48277	6.44672	6.35181	5.99418	6.10395	5.90669	5.97046
NAA15	Q9BXJ9	6.10923	6.29873	6.83883	6.05207	6.30797	6.6263	6.20618	6.40054	6.52275	6.40812	6.68712	6.52594	5.7425	5.88569	5.62738	5.76064
MTREX	P42285	6.30862	6.35351	5.93646	6.47125	6.42746	6.31428	6.37997	6.03043	6.10872	6.02047	6.00601	5.8109	6.59829	6.26583	6.48773	6.48007
GCC2	Q8IWJ2	6.48683	6.41453	6.2432	6.51479	6.53572	6.29218	6.14856	6.2262	6.52065	6.29358	6.36255	6.53974	6.31617	5.72985	5.71596	5.65948
EIF3B	P55884-2	6.32682	6.32938	6.92977	6.25535	6.17786	6.38225	6.57646	6.41312	6.59159	6.20763	6.624	6.49388	5.52224	5.76089	5.63897	5.7698
USP9X	Q93008-3	6.27436	6.23323	6.6738	5.94585	6.19085	6.72249	6.21372	6.40145	6.14977	6.41555	6.4306	6.49336	6.02571	6.09658	5.83157	5.90111
PRDX1	Q06830	5.83836	5.27326	6.45161	4.91062	6.42971	6.42119	6.04787	6.86438	6.04928	6.63291	6.46003	6.90147	6.4201	6.72258	6.19809	6.37854
EIF4A3	P38919	6.24693	6.35554	6.12422	6.4876	6.42066	6.16173	6.42457	6.12773	6.28346	6.22743	5.99665	6.00533	6.26738	6.36815	6.31344	6.18919
ANXA6	P08133	6.43124	6.1295	6.83259	5.82533	6.4444	6.79661	6.35049	6.83582	6.29819	6.45753	6.5242	6.41076	5.6899	5.85934	5.56288	5.55121
RPS27A	P62979	6.57987	6.74269	6.31559	6.75575	6.29625	6.23094	6.29939	6.46421	6.04326	6.18238	6.13383	6.33264	5.39019	6.31038	5.705	6.21764
H3C1	P68431	6.31942	6.24515	5.55567	6.38899	6.09061	5.86646	6.55582	6.30163	5.86696	5.90398	5.71077	5.93886	6.62966	6.84603	6.80691	6.97308
SVIL	O95425	6.13459	6.40126	5.79627	6.10464	6.35377	6.22628	6.29845	6.03643	5.9652	6.12581	5.6258	5.76204	6.85846	6.78787	6.87677	6.65535
QARS1	P47897	6.34218	6.16816	6.49092	5.92121	6.56879	6.41975	5.9875	6.36742	6.50891	6.43026	6.46359	6.42025	6.16623	6.1367	5.95227	5.65587
TPP2	P29144	6.38478	6.47219	6.25466	6.58327	6.42681	6.39905	6.37713	6.23151	6.18044	6.02817	5.97024	6.13392	6.21995	6.12592	6.15686	6.05511
RPS3A	P61247	6.20127	6.55457	6.67109	6.33171	6.26404	6.31067	6.37724	6.1475	6.40242	6.58963	6.54592	6.37302	5.68132	5.96166	5.78129	5.80664
TARS1	P26639	6.30425	6.10036	6.9388	5.95119	6.3611	6.66544	6.45316	6.54422	6.47819	6.36565	6.49058	6.50119	5.76791	5.74626	5.68562	5.64607
THOC2	Q8NI27	6.13507	6.14878	5.83839	6.52205	6.06549	5.89633	6.33616	5.72048	6.09964	5.98034	5.87601	5.77445	6.58262	7.04615	6.87578	7.10225
CCT4	P50991	6.29756	6.13464	6.48253	5.97499	6.42996	6.69656	6.14039	6.60308	6.38295	6.4012	6.2578	6.40727	6.09902	6.025	5.71351	5.95355
GTF3C1	Q12789	6.41815	6.56731	6.12408	6.91681	6.25398	6.07421	6.40826	6.09856	6.20501	6.13719	6.18764	6.02271	6.06848	6.11386	6.26394	6.13982
AHCTF1	Q8WYP5-2	6.49735	6.45669	5.72701	6.71253	6.26633	5.79198	6.46307	6.06084	6.02297	5.91266	5.75349	5.82683	6.46583	6.66156	6.87239	6.53827
DICER1	Q9UPY3	6.41502	6.36906	6.72105	6.03262	6.31089	6.61308	6.08371	6.40783	6.61136	6.51579	6.61358	6.42131	5.76425	5.82716	5.57636	5.71693
BOD1L1	Q8NFC6	6.24868	6.35286	6.07283	6.58302	6.03309	5.96542	6.11663	5.8636	6.11014	6.14888	6.06642	6.01794	6.12704	6.94757	6.54656	6.79933
CS	O75390	6.41267	6.08978	6.01189	6.4031	6.34361	6.01407	6.4522	6.18472	6.11357	6.03507	6.12247	5.96903	6.66833	6.24307	6.44627	6.49015
ATM	Q13315	5.79535	6.14537	6.0859	5.78124	6.05432	6.25379	6.02511	5.86607	6.10554	6.43011	6.24469	6.03351	6.87768	6.74296	6.9356	6.62275
DDX21	Q9NR30	6.39056	6.37914	6.02718	6.98749	6.22163	5.94163	6.41697	6.04537	6.19785	5.78852	5.9604	5.89777	6.0664	6.35738	6.70634	6.61538
SLC25A5	P05141	6.57924	6.58296	5.86051	6.69746	6.29232	5.89222	6.34334	6.00275	6.19503	6.01426	5.93031	5.96552	6.49608	6.25744	6.51892	6.37166
PI4KA	P42356	6.38398	6.64378	6.46109	6.48636	6.17571	6.32038	6.32047	6.00223	6.16149	6.35016	6.25871	6.32857	6.03084	5.93002	6.1546	5.9916
IPO7	O95373	6.27396	5.98132	6.21513	6.41409	6.17413	6.44535	6.16359	5.92772	6.54874	6.29018	6.38406	6.45403	5.99196	6.14622	6.03787	6.55165
UTP20	O75691	6.30867	6.31625	5.81142	6.60185	6.05843	5.82695	6.46753	6.1134	6.17131	5.96143	5.98884	6.04054	6.4041	6.42204	6.54417	6.96306
MTHFD1L	Q6UB35	6.42668	6.37202	6.05167	6.6462	6.31369	5.91296	6.42951	6.05339	6.23205	6.01877	6.06824	6.14534	6.57573	6.06399	6.30721	6.38256
DCTN1	Q14203-4	6.30663	6.23591	6.74792	6.02851	6.46367	6.64376	6.10459	6.67904	6.46228	6.5619	6.48504	6.5933	5.80061	5.72825	5.55765	5.60094
CENPF	P49454	6.24943	6.06309	5.86915	6.19672	6.27736	6.07603	6.43832	6.05501	6.17342	5.93831	6.12882	6.0272	6.93157	6.28643	6.62132	6.66781
BAZ1B	Q9UIG0	6.4892	6.44625	5.85912	6.99454	6.10866	5.80716	6.35757	6.13113	6.09394	5.97501	6.04554	5.96167	6.44193	6.25991	6.59771	6.43063
RPL3	P39023	6.43029	6.33315	6.34625	6.24105	6.18092	6.29369	6.26668	6.50915	6.63143	6.34654	6.48091	6.24966	5.79376	6.18612	5.78503	5.92537
TOP2A	P11388-4	6.48421	6.65377	6.01187	7.00697	6.11432	5.99923	6.45386	6.06981	6.34594	6.34244	6.24537	6.37087	5.7373	5.98095	6.00987	6.17321
SMC2	O95347	6.22324	6.36516	6.52372	6.13433	6.51061	6.57599	6.19471	6.51799	6.38458	6.36383	6.31219	6.19147	5.90778	6.01216	5.81941	5.96282
MVP	Q14764	6.11208	6.13685	6.49125	5.647	6.33624	6.69113	6.16888	6.75177	6.36906	6.57314	6.58942	6.4926	5.98476	6.32846	5.73537	5.59199
NUP205	Q92621	6.31877	6.16896	6.0307	6.64022	6.18292	5.99146	6.48089	6.06391	6.13582	5.87567	6.00503	5.90052	6.56325	6.29114	6.67944	6.6713
SF3A1	Q15459	6.38422	6.3204	5.77982	6.53278	6.4366	6.0915	6.43188	6.14924	6.1796	5.78751	5.87409	5.99627	6.8556	6.29859	6.38951	6.49239
ERBIN	Q96RT1-9	6.4965	6.41386	6.30439	6.64582	6.31367	6.00695	6.39632	6.06805	6.16714	6.20136	6.32301	6.23329	6.27121	5.84055	5.99038	6.3275
PRMT1	Q99873	5.66557	6.0918	5.94152	5.88985	6.12583	6.28652	6.1076	6.05198	5.79947	6.24592	5.99982	5.81097	7.02069	6.81034	7.36751	6.7846
RAN	P62826	6.02364	6.08309	6.37834	6.17145	6.04547	6.42492	6.00498	6.085	6.24135	6.27666	6.37103	6.30678	6.41201	6.34154	6.5264	6.30733
AP2A1	O95782	6.11566	6.27721	6.11913	6.44447	6.08385	6.17069	6.24772	6.06562	6.24851	6.32033	6.25678	6.29957	6.34488	6.30019	6.35817	6.34722
VPS13C	Q709C8	6.5306	6.24059	6.79142	6.29964	6.232	6.39292	6.31394	6.40433	6.57127	6.52079	6.33704	6.37144	5.69159	5.845	5.55762	5.89981
H1-5	P16401	6.18661	6.16349	5.34936	6.26325	6.03959	5.8409	6.27933	6.05932	5.78217	5.87606	5.64058	5.74239	6.67983	7.60328	7.07974	7.41408
NIPBL	Q6KC79	6.16662	6.42327	5.85788	6.82312	5.9746	6.04486	6.20834	5.92231	5.99866	5.98429	5.90842	5.92572	6.4088	6.65253	6.86495	6.83562
DDX23	Q9BUQ8	6.19688	6.3283	5.81496	6.45951	6.06557	5.95703	6.21953	5.93088	5.91451	6.14832	5.8722	5.95869	6.59012	6.7435	7.00722	6.79277
SMC3	Q9UQE7	6.16235	6.40479	5.67452	6.47133	6.20178	6.0653	6.45109	6.1196	6.25746	5.87748	5.57053	5.88086	6.50834	6.631	6.74541	6.97816
AP3D1	O14617-5	6.12685	6.23001	6.6977	6.12914	5.92571	6.34289	6.02265	6.36366	6.19509	6.44053	6.13128	6.35434	5.70226	6.67895	6.28456	6.37439
IPO5	O00410-3	6.35006	5.95582	6.85196	6.04596	6.2162	6.50611	6.27645	6.41829	6.55841	6.20668	6.5584	6.5827	5.73376	5.96276	5.77319	6.00325
MCM3	P25205-2	5.58071	6.26489	5.99259	6.10577	6.18249	6.47234	6.18302	5.97859	5.7778	5.82294	5.99414	5.76531	7.05494	7.21492	6.92867	6.68088

KNTC1	P50748	6.48831	6.21996	6.87211	6.06035	6.31469	6.64778	6.41135	6.59749	6.48336	6.53046	6.43034	6.42343	5.5641	5.77861	5.54944	5.62822
SUPT6H	Q7KZ85	6.2369	6.28475	6.15786	6.85163	6.34884	6.11083	6.43385	6.10635	6.27462	6.18698	6.25013	6.11075	6.25639	5.87169	6.21579	6.30263
MIA3	Q5JRA6	6.46185	6.35383	6.11902	6.43484	6.18822	5.91864	6.46604	6.0521	6.13156	5.86331	6.21269	6.11149	6.77824	6.15927	6.31499	6.43392
MGA	Q8IWI9-3	6.28729	6.71531	6.15171	6.86764	6.2164	6.18512	6.43966	6.15474	6.14173	6.08587	5.9999	6.19389	5.89656	6.13037	6.08471	6.4491
ILF2	Q12905	6.35335	6.23778	5.92941	6.42919	6.27342	5.98082	6.20117	5.97736	6.2524	6.08997	6.00996	6.06546	6.73425	6.2827	6.60683	6.57593
LARP1	Q6PKG0	6.4524	6.5282	6.79496	6.21687	6.49264	6.57304	6.26108	6.33327	6.43225	6.34636	6.357	6.14867	5.73536	6.02453	5.65358	6.54979
ITGB1	P05556	6.37721	6.53253	5.93379	6.24551	6.41916	6.16012	6.42331	5.96968	6.18689	5.97502	5.79659	5.88637	6.66643	6.22408	6.54443	6.65889
SMARCC2	Q8TAQ2	6.09249	6.23102	6.01613	6.3357	6.37567	6.16244	6.32453	5.92898	6.16325	6.0422	6.07779	5.84523	6.77107	6.562	6.58661	6.4849
USP34	Q70CQ2	5.87166	6.16703	6.25875	6.32265	6.14444	6.16951	6.20911	5.91109	6.00917	6.19468	6.14804	6.12511	6.55954	6.57173	6.73051	6.60697
RBM14	Q96PK6	6.27098	6.42631	5.98521	6.81017	6.30599	5.84349	6.31698	5.95102	6.13292	5.90068	5.9875	5.99869	6.72841	6.16398	6.65231	6.52537
SYMPK	Q92797	6.31632	6.51046	5.80586	6.61631	6.2702	6.12976	6.20493	6.21738	5.93459	6.09813	5.89678	5.94643	6.71776	6.36906	6.44198	6.52405
ANXA5	P08758	6.26274	6.04475	6.93857	5.54167	6.55254	6.93298	6.23401	6.53192	6.1625	6.37969	6.63278	6.44761	5.95669	6.28595	5.5614	5.53418
HSD17B4	P51659	6.30562	6.53794	5.89451	6.4982	6.29448	6.14051	6.34886	6.00989	6.04284	6.05855	5.89588	5.83782	6.67607	6.29701	6.71153	6.4503
ASNS	P08243	6.28803	6.24639	6.76905	5.82634	6.50163	6.63754	6.41857	6.86764	6.66159	6.23166	6.41551	6.47768	5.70056	5.57245	5.74885	5.63651
CIT	O14578-4	6.57232	6.74815	6.62011	6.77236	6.35005	6.3192	6.41324	6.43416	6.56837	6.50568	6.33912	6.44153	5.52932	5.32156	5.50519	5.55964
YARS1	P54577	6.24966	5.96444	6.88119	5.70065	6.62199	6.88563	6.09658	6.4893	6.35243	6.33334	6.48984	6.30366	6.39327	5.96695	5.67175	5.59932
CUL3	Q13618	5.72841	6.03037	6.28526	5.68758	5.96398	6.25067	6.12352	6.2167	6.13967	6.29346	6.28874	6.18055	6.58496	6.74864	6.85977	6.61772
PFAS	O15067	6.09102	6.01461	6.93763	5.96955	6.2491	6.74507	6.26743	6.59192	6.44409	6.22091	6.36923	6.49086	5.72461	6.17136	5.83017	5.88245
PSIP1	O75475	6.24762	6.20139	5.57371	6.58159	6.07671	5.7448	6.50773	5.87128	6.11091	5.77535	5.63084	5.97336	6.52879	7.02667	6.96614	7.1791
POTEF	A5A3E0	6.55703	6.30772	6.21218	5.9634	6.41265	6.30293	6.09892	6.03243	5.93255	6.17581	6.18376	6.42375	6.62963	6.26447	6.26471	6.23807
TNPO1	Q92973	6.05909	5.9427	6.37702	5.88737	6.23933	6.15999	6.10285	6.17966	6.30272	6.15815	6.40837	6.26055	6.317	6.32227	6.59888	6.68407
NSD2	O96028	6.35413	6.53428	6.03344	6.97393	6.23994	6.03944	6.42782	6.07099	6.30053	6.14028	6.23075	6.13939	5.90279	6.21575	6.29393	6.10262
PRKCSH	P14314-2	6.63502	6.31305	5.92211	6.25926	6.86998	6.12235	6.22741	6.30602	6.1935	6.01613	6.01112	5.74846	7.14181	6.06371	6.16417	6.0059
COPG1	Q9Y678	6.30226	6.32875	6.43173	5.99135	6.3928	6.53405	6.3242	6.37867	6.43658	6.47828	6.50354	6.43692	6.0021	5.90245	5.78976	5.76656
LRBA	P50851	6.26588	6.6329	6.87628	6.58114	6.44207	6.77119	6.1848	6.42019	6.14023	6.34017	6.38439	6.12082	5.76849	5.92117	5.70113	5.44916
NUP155	O75694	6.35444	6.3073	6.04331	6.61503	6.31482	6.05042	6.37929	5.95339	6.18862	5.98286	6.17823	6.058	6.63149	6.12094	6.44406	6.3778
MYCBP2	O75592	6.51433	6.198	6.64224	6.5144	6.33199	6.42306	6.34424	6.41895	6.27702	6.58636	6.36948	6.25843	5.70025	5.81151	5.59221	5.61573
PDIA4	P13667	6.24765	6.52215	5.84236	6.39339	6.49826	6.16192	6.23928	6.06795	5.97466	6.00641	5.88699	5.82252	6.80288	6.42743	6.68006	6.4261
GTPBP4	Q9BZE4	6.29107	6.17557	6.01085	6.64838	6.30456	5.86104	6.54322	5.93674	6.33156	5.89858	6.01381	5.84667	6.55454	6.28808	6.80529	6.49003
RARS1	P54136	6.43216	6.17789	6.50583	5.83949	6.46823	6.52845	6.2662	6.48806	6.34456	6.19445	6.3085	6.40029	6.07105	6.27524	5.84623	5.85337
PABPC1	P11940	6.47046	6.66473	6.701	6.23648	6.42734	6.59602	6.32111	6.56671	6.3015	6.49924	6.49576	6.49417	5.54949	5.66264	5.56562	5.44773
PSMC3	P17980	6.42435	6.29224	6.38961	6.04575	6.66864	6.53819	6.05508	6.65056	6.14048	6.25257	6.49889	6.31664	6.43977	5.84801	5.72089	5.71834
KNL1	Q8NG31	6.34892	6.44801	6.2634	6.66176	6.2698	6.22673	6.36705	6.19021	6.39422	6.25615	6.29634	6.44163	6.06498	5.7153	5.93408	6.1214
FLII	Q13045	6.39389	6.25627	6.95908	6.17181	6.36804	6.49637	6.32263	6.46166	6.60865	6.58954	6.61252	6.64522	5.56161	5.62788	5.36243	5.5624
SRRT	Q9BXP5	6.01257	6.37355	5.69298	6.50606	6.15034	6.09327	6.13643	5.99405	5.95691	6.03434	5.94491	6.08865	6.84943	6.65524	6.62461	6.88664
NUP153	P49790	6.38843	6.22921	6.02984	6.70264	6.18103	5.86985	6.38986	5.89491	6.02857	6.1221	5.97764	5.85336	6.85376	6.43135	6.50635	6.5411
NME2	P22392-2	6.84443	6.4338	7.06815	6.06672	6.9567	6.96288	6.4934	7.04685	6.76626	6.39797	6.14588	5.98387	5.2005	5.19618	5.3145	5.12191
PLIN3	O60664	6.33478	5.62982	6.46585	5.29912	6.38289	6.54707	6.23406	6.8301	6.3457	6.24546	6.4393	6.73468	6.45566	6.191	5.89779	5.96582
NNT	Q13423	6.4746	6.44683	5.90038	6.44096	6.22449	5.9879	6.2872	5.97232	5.94308	6.21221	5.72091	5.72363	6.68128	6.65189	6.75783	6.57449
PHB	P35232	6.3351	5.26928	5.44521	5.28855	6.36871	5.49736	6.07948	6.38956	5.44512	6.01867	5.65158	6.38763	6.7358	7.65575	7.85277	7.57943
COPB2	P35606	6.20073	6.34489	6.64142	6.21503	6.24443	6.51905	6.37634	6.41972	6.36934	6.33597	6.41857	6.4617	5.8627	6.08656	5.92994	5.84361
PTPRF	P10586	6.25904	8.02474	6.12484	6.75363	6.07656	5.93045	6.05776	6.7356	6.44445	6.56438	6.29731	6.53135	5.3715	5.55778	5.75768	5.51293
RPSA	P08865	6.48622	5.376	6.23394	4.87826	6.22217	5.91738	6.07901	6.62508	5.75665	6.70409	6.27051	7.07072	5.78106	7.04776	6.66922	6.88193
SLC3A2	P08195-4	6.40482	6.49935	5.95217	6.34443	6.58379	6.18269	6.30852	6.10045	6.14821	5.78905	5.97914	6.20947	6.72433	6.13197	6.28868	6.35295
GOT2	P00505	6.28967	6.34956	5.87936	6.37833	6.58242	5.93982	6.66954	5.85431	5.7803	6.70499	6.08543	5.84005	6.48687	6.37357	6.58756	6.19823
PCBP2	Q15366-3	6.35916	6.15184	6.47168	6.05986	6.31144	6.54826	5.93886	6.30898	6.53136	6.63774	6.53802	6.36252	6.12489	5.98963	5.8078	5.85794
KIF20B	Q96Q89-3	6.66703	6.66405	6.47478	7.11528	6.54445	5.9657	6.68481	6.11016	6.49462	6.23302	6.45238	6.41391	5.48675	5.27961	5.62234	5.79109
DYNC2H1	Q8NCM8-2	6.6328	6.71509	7.27779	6.51568	6.34637	6.37663	6.42848	6.5133	6.79782	6.86097	7.12257	7.05224	4.79245	5.0325	4.69494	4.84037
TPX2	Q9ULW0	6.34344	6.15258	5.76606	6.40103	6.07096	5.94175	6.42705	5.83552	6.08356	5.8469	5.9555	5.91062	6.96711	6.53128	6.85455	6.92209
OGA	O60502	6.17498	6.26031	6.90905	5.95798	6.42741	6.68567	6.39314	6.47847	6.20742	6.28608	6.43023	6.23798	5.96403	6.08031	5.74301	5.76391
SUPT5H	O00267	5.55166	6.02641	5.99971	5.74048	5.95551	6.11289	6.19904	5.79785	6.22928	5.90629	6.24291	5.98316	6.93039	6.93177	7.33161	7.06105
HELLS	Q9NRZ9	6.15605	6.44043	6.43326	6.61097	6.26988	6.08506	6.22106	6.15565	6.43701	6.36534	6.3694	6.43013	5.9669	5.91177	6.0162	6.1309
RPL5	P46777	6.06707	6.11998	6.30374	6.27702	6.11434	6.14689	6.49291	6.15587	6.70545	5.95132	6.22773	6.298	5.73307	6.5805	6.23415	6.59195
GDI2	P50395	6.25453	5.97259	7.02512	5.88173	6.30394	6.73469	6.34042	6.62098	6.57966	6.53873	6.46535	6.67744	5.72925	5.68901	5.52265	5.6639
PITRM1	Q5JRX3-2	6.25378	6.44388	5.65216	6.67311	6.26488	6.23307	6.34812	6.22622	6.13825	6.1415	5.96993	5.92916	6.54184	6.25699	6.52194	6.40519
RPN2	P04844	6.32483	6.5067	5.88196	6.52676	6.23341	6.08491	6.44937	6.00705	5.91433	6.1086	5.90914	5.86159	6.61587	6.35013	6.76233	6.46301
EXOSC10	Q01780	6.33802	6.37143	5.95034	6.68353	6.28103	6.0156	6.45008	5.97904	6.11186	5.97949	5.98488	5.8977	6.52123	6.44396	6.58687	6.40494
SMARCC1	Q92922	5.99508	5.95582	5.9331	6.64546	6.18673	5.68467	6.84485	5.73426	6.60641	5.4716	5.8271	6.00198	6.3172	6.7601	6.73	7.30565
PLXNA1	Q9UIW2	6.4374	6.44948	5.92727	6.38154	6.14758	6.32268	6.18275	5.98429	6.04828	6.12706	6.17081	5.95392	6.41772	6.49343	6.47976	6.47605
RFC1	P35251	5.95441	6.22986	5.95233	6.26905	5.92177	5.98279	6.10757	6.03078	5.87581	6.06984	6.00651	6.00308	6.18039	7.29887	6.87083	7.24614
CLASP1	Q7Z460	6.30605	6.23393	6.4528	6.17346	6.25142	6.43008	6.17861	6.26396	6.33082	6.28537	6.31182	6.24499	6.28887	6.13092	6.12927	5.98763
PRRC2A	P48634	6.75805	6.87373	6.7819	6.71342	6.45098	6.24719	6.25452	6.28425	6.37117	6.50303	6.48804	6.60285	5.38676	5.54663	5.32344	5.41405
SHMT2	P34897	6.26922	6.40272	5.62898	6.38737	6.47483	6.12061	6.35955	6.48501	6.09759	5.94637	6.13517	5.93742	6.81802	6.45026	6.24285	6.24403
ABCF1	Q8NE71	6.48936	6.41608	6.61244	6.37577	6.36199	6.3789	6.38745	6.56094	6.45479	6.26867	6.33221	6.24623	5.3218	6.24777	5.62762	5.91795
CUX1	P39880-3	6.45391	6.10252	6.13259	6.4774	6.25823	5.96295	6.16416	5.9684	6.24887	6.19689	6.30264	6.22933	6.90172	6.07573	6.31237	6.2123
ANXA1	P04083	5.99104	6.23201	6.70551	5.82813	6.34738	7.05537	6.10475	7.01396	6.11244	6.42292	6.07883	6.60321	5.94479	6.24133	5.45848	5.85985
TASOR	Q9UK61	6.4964	6.64153	5.92963	6.77144	6.10463	6.06975	6.29842	6.11087	6.36483	6.14147	5.96065	5.96697	6.46072	6.13558	6.1759	6.3712
MICAL3	Q7RTP6	6.10397	6.3574	6.46133	6.14676	6.11239	6.33152	6.21585	6.19198	6.36033	6.53311	6.44862	6.45683	6.00807	6.15904	6.07126	6.04154
ELAC2	Q9BQ52	6.04716	6.04358	6.17243	6.11906	6.00801	6.32011	6.098	6.18292	6.11203	6.13048	6.07573	6.04775	6.39583	6.56411	6.95235	6.73046
KIF23	Q02241	6.38917	6.5866	6.38129	7.01373	6.09885	6.34299	6.68283	6.17217	6.06723	6.22527	6.19732	6.28216	6.57825	5.58752	6.10498	6.18963
DNM1L	O00429-6	6.42323	6.13484	6.85704	5.99678	6.42666	6.81601	6.13683	6.77345	6.30915	6.45443	6.49168	6.34431	5.85971	5.95369	5.59875	5.42344
CLUH	A0A494C0R8	6.38976	6.26583	7.192	6.41774	6.3947	6.75717	6.30377	6.74431	6.68736	6.49716	6.91912	6.77304	5.28675	5.18754	5.00852	5.17523
NOL11	Q9H8H0	6.4114	6.27202	6.10208	6.78242	6.38533	6.0346	6.27098	5.97371	6.46525	5.95012	6.04507	5.90551	6.64821	5.8899	6.62789	6.23551
RTN4	Q9NQC3	6.21455	6.35517	5.82616	6.23298	5.84702	6.06793	6.03383	6.28307	5.95184	5.93493	5.9428	6.11354	6.42594	6.68123	6.87242	7.23128
ATIC	P31939	6.23153	6.05356	6.78828	5.8048	6.42496	6.61914	6.34959	6.83671	6.50902	6.35449	6.56778	6.45909	5.81523	5.84999	5.77851	5.55732
GSN	P06396-2	6.39799	6.02515	6.70542	5.69764	6.3	6.85972	6.15764	6.62574	6.27804	6.49062	6.51518	6.4528	6.06931	6.24869	5.65573	5.52034
ERAP1	Q9NZ08-2	6.39924	6.50358	5.95331	6.3531	6.30452	6.10509	6.22192	6.00787	6.04312	6.15377	6.02306	5.90274	6.65773	6.19148	6.81699	6.36249
NUP160	Q12769	6.64979	6.47992	6.27778	6.91999	6.35298	6.18302	6.56604	6.03253	6.43758	6.08129	5.79937	5.7238	6.29763	5.80029	6.2415	6.1565
FKBP4	Q02790	6.33821	6.08424	6.56899	5.92929	6.48634	6.63919	6.22179	6.32233	6.26176	6.43941	6.53802	6.36725	6.18073	5.9205	5.90622	5.79575
EPHA2	P29317	6.14214	6.44656	5.95458	6.56863	6.04719	6.07016	6.30673	6.12051	5.98724	5.9553	5.99947	5.84446	6.47191	6.49681	6.8315	6.75681
USP5	P45974	5.85585	5.93956	6.60297	5.69856	5.93724	6.47451	5.94592	6.46979	6.04431	6.30923	6.59002	6.40018	6.37446	6.61603	6.49736	6.244
EIF3D	O15371	6.2489	6.31414	6.42114	6.28478	6.26972	6.32596	6.71693	6.02859	6.65136	6.17531	6.21206	6.37712	5.61632	6.15796	5.87319	6.32652
PFN1	P07737	6.01286	5.7093	6.75563	5.41421	6.66006	6.50492	6.13602	6.91289	6.43274	6.67791	6.79537	6.87271	5.62583	5.95128	5.67046	5.86782
HNRNPDL	O14979	6.3488	6.35066	5.90738	6.49205	6.51457	6.16351	6.51124	6.0558	6.30422	5.94406	6.07865	6.09092	6.53421	6.1339	6.31676	6.25327
NOP56	O00567	6.18683	5.94967	5.47108	6.68311	6.07522	5.51801	6.81593	5.68746	6.2099	5.34787	5.59932	5.83817	6.30359	7.32893	7.29227	7.69264
EP400	Q96L91	6.78585	6.72427	6.32637	7.16683	6.2509	5.9222	6.52115	5.92255	6.50538	6.47526	6.27388	6.31143	5.73678	5.55224	5.72114	5.80375
TFRC	P02786	6.0002	6.32781	6.05883	6.43116	6.31746	6.11354	6.72041	6.39423	6.11449	5.89262	6.00812	6.05765	6.21177	6.16173	6.67117	6.5188
EEF1G	P26641-2	6.27862	6.25025	6.71319	5.96326	6.27087	6.37549	5.99786	6.43081	6.55027	6.3746	6.4505	6.56884	6.01502	6.04954	5.91802	5.79287
NAP1L1	P55209	6.44466	6.18618	6.95249	6.19924	6.60631	6.83419	6.11942	6.75418	6.67293	6.52188	6.84958	6.93839	5.63675	5.2742	4.89194	5.11767
NUP188	Q5SRE5	6.40089	6.46956	6.09112	6.52698	6.37192	6.25183	6.24504	6.2704	6.25697	6.1785	6.11268	6.13232	6.00187	6.17163	6.10124	6.41705
MTOR	P42345	6.43886	6.42558	6.55204	6.42804	6.13541	6.61253	6.09211	6.20111	6.44845	6.58262	6.63279	6.68886	5.67419	5.91698	5.51154	5.65889
TUBB2B	Q9BVA1	6.57158	6.18654	7.02544	6.03738	6.59454	6.86406	6.0131	6.83351	6.43333	6.38833	6.69108	6.714	5.75775	5.4327	5.14461	5.31204
RECQL	P46063	5.15161	5.44385	5.72676	5.05883	5.57042	6.15742	5.73907	5.77523	5.75173	6.01565	6.07169	5.80473	7.61508	8.24409	8.24568	7.62816
FTSJ3	Q8IY81	5.88908	6.24208	5.53778	6.72759	5.82655	5.97748	6.34904	6.10895	6.30609	5.98878	6.02141	6.08059	6.34563	6.8504	6.73276	7.01579
CAST	P20810-6	6.25315	6.28945	6.61343	5.72395	6.17231	6.51386	5.98215	6.54195	6.36735	6.40296	6.30526	6.43936	6.06448	6.39758	5.86218	6.0706
TNKS1BP1	Q9C0C2	6.14233	6.28884	6.33487	5.84478	6.13723	6.27982	5.97238	6.30578	6.28626	6.5743	6.4303	6.39818	6.51893	6.25637	6.145	6.08462
SLC25A13	Q9UJS0-2	6.42596	6.09284	5.89493	6.51075	6.36145	6.00324	6.57789	6.07111	6.10582	6.03928	6.02109	5.89213	6.74519	6.3767	6.53673	6.34489
CHD7	Q9P2D1	6.43975	6.41461	6.29986	7.0583	5.97927	6.04095	6.47501	6.07334	6.164	6.05647	5.96332	6.0899	5.85374	6.302	6.26398	6.52549
ITPR2	Q14571	6.41964	6.41315	5.84607	6.5978	6.21398	5.84511	6.3244	5.92291	5.96179	6.11103	5.8228	5.76691	6.66876	6.59211	6.86914	6.6244
ARFGEF1	Q9Y6D6	6.26007	6.17459	6.85579	6.27074	6.1671	6.54668	6.20991	6.36645	6.30601	6.5148	6.50603	6.44105	5.90743	5.88705	5.64811	5.93816
EMC1	Q8N766	6.39208	6.55028	6.13757	6.85427	6.20719	5.9092	6.41995	6.10324	6.21418	6.0865	5.81234	5.89851	6.35624	6.04552	6.49103	6.5219
ABCE1	P61221	6.30686	6.26815	6.9307	6.02967	6.36642	6.81927	6.37891	6.5907	6.64812	6.38546	6.31121	6.22986	5.64676	5.71259	5.77522	5.6001
UGGT1	Q9NYU2	6.27751	6.44142	5.75755	6.51082	6.26937	6.16395	6.28664	6.08733	6.06687	5.96048	5.75628	5.60624	6.70118	6.64774	6.76931	6.69734
ITGA6	P23229	6.2613	6.35722	6.11737	6.57463	6.27318	6.26785	6.48815	6.2457	6.14305	5.98497	6.36477	6.34176	6.22794	5.93138	6.08002	6.34072
ATAD2	Q6PL18	6.45398	6.43341	5.99812	6.81021	6.23053	5.82295	6.26784	5.85097	6.51797	6.25828	6.26162	6.18769	6.39413	5.91415	6.31641	6.28175

RPLP0	P05388	6.3874	6.41087	6.45532	6.19441	6.29864	6.52682	6.07994	6.39788	6.4883	6.68371	6.45582	6.68168	5.7642	5.87012	5.66038	5.64453
MCM2	P49736	5.64013	6.18711	5.9454	5.7906	6.03589	6.12982	5.99965	6.16955	6.01357	6.01822	6.00565	6.09177	6.89597	6.9822	7.07023	7.02425
RPL7	P18124	5.99524	6.44866	5.75314	5.80967	6.13953	6.33854	6.52053	6.6362	7.03493	6.50832	6.43614	6.41606	5.96933	6.24142	5.62749	6.12479
ESYT1	Q9BSJ8	6.17909	6.5275	6.06057	6.44053	6.33292	6.14313	6.33359	6.15083	6.07621	6.18267	6.16603	5.96514	6.45562	6.32488	6.46406	6.19721
PPP1CC	P36873-2	6.20955	6.13981	6.28134	6.51567	6.15622	6.24107	6.1733	6.04589	6.27725	6.29417	6.3597	6.46885	6.25544	5.95189	6.39498	6.23486
CARS1	P49589-3	6.11092	6.31411	6.5906	6.03609	6.23387	6.71921	6.14038	6.6483	6.04659	6.4672	6.36807	6.36847	5.84323	6.31996	5.97865	5.81434
NDUFS1	P28331-2	6.38954	6.27953	5.75626	6.55187	6.32582	5.72783	6.63805	5.88274	6.46493	5.60971	5.77515	5.8522	6.52683	6.63684	6.57386	7.00883
PNPT1	Q8TCS8	6.37219	6.30743	5.99398	6.83238	6.37585	5.95785	6.43699	5.94718	6.35243	6.14361	6.06263	6.01692	6.59128	5.96164	6.38661	6.26103
SLC25A3	Q00325	6.36049	6.39121	5.79058	6.68638	5.96107	5.76069	6.40334	5.97761	6.10452	5.91486	5.84866	5.87803	6.50299	6.82134	6.72547	6.87274
UFL1	O94874	6.32155	6.55137	5.89361	6.43256	6.37999	6.29204	6.44078	6.01922	5.96432	6.06991	5.90061	5.92025	6.60267	6.26569	6.57166	6.37376
FSCN1	Q16658	6.32618	6.44243	6.80735	5.87003	6.35021	6.82875	6.29847	6.57562	6.39818	6.65474	6.31935	6.3749	5.7606	5.74223	5.76237	5.48859
TOMM40	O96008	6.03747	5.99341	5.65477	6.89413	6.47722	5.69578	7.23785	5.79634	6.68109	5.22806	5.58053	5.97751	6.26511	6.44862	6.82884	7.20326
MYO6	A0A0A0MRM8	6.22791	6.41964	6.63995	6.01135	6.30577	6.65911	6.21083	6.46654	6.37542	6.5788	6.52656	6.21063	5.9356	6.02284	5.7045	5.70455
WDR1	O75083	6.16326	6.04385	6.56161	5.67001	6.68881	6.61554	6.35413	6.60088	6.26189	6.2687	6.18827	6.07489	6.12239	6.22402	6.17231	5.98943
LDHA	P00338-3	6.17809	6.0851	6.82332	5.74032	6.45486	6.8578	6.27322	6.56361	6.29653	6.33334	6.37375	6.4259	5.77132	6.33036	5.73416	5.75832
NOP58	Q9Y2X3	6.12213	6.22407	5.58507	6.67806	6.11357	5.87838	6.28429	5.79098	6.03152	5.74049	5.69135	5.68557	6.55246	7.21163	7.2155	7.19493
KIF5B	P33176	6.27021	6.1999	6.92895	5.9668	6.3729	6.52638	6.40924	6.65259	6.54918	6.45087	6.47361	6.42815	5.78267	5.72271	5.73843	5.52739
AQR	O60306	6.28626	6.3159	6.25961	6.55879	6.18666	6.11036	6.41669	5.86203	6.2284	6.1413	6.15666	6.10481	6.45195	6.24912	6.33701	6.33446
ITGA3	P26006	6.47307	6.50067	5.88382	6.24456	6.34075	6.07078	6.33927	5.96534	6.2115	6.06631	5.83525	5.97433	6.62676	6.66433	6.37857	6.42468
RUVBL2	Q9Y230	6.32461	6.52773	6.44556	6.3872	6.443	6.24087	6.38793	6.40904	6.42842	6.09356	6.22357	6.29296	5.9656	5.88625	5.98512	5.95857
MYO1C	O00159	6.06696	6.13964	5.87165	5.92423	6.22505	6.05124	6.38026	5.89997	6.52489	6.2753	6.13319	6.09176	6.72084	6.54524	6.66554	6.48423
XRN2	Q9H0D6	6.24147	5.98619	6.00766	6.38736	6.43833	5.96387	6.35535	5.87339	6.37651	6.12715	6.22798	6.00117	6.65268	6.29274	6.47698	6.59117
WDR11	Q9BZH6	6.27259	6.27007	6.48168	6.09926	6.30939	6.3423	6.33465	6.17969	6.38873	6.59083	6.42511	6.35446	5.97265	5.99048	6.0462	5.94194
DDX27	Q96GQ7	6.15368	6.22653	5.71018	6.65537	6.27999	5.93761	6.2965	6.07752	6.06644	6.0558	5.88698	5.8578	6.43084	6.57984	6.99625	6.78868
DDOST	P39656	6.27427	6.2284	6.04129	6.53371	6.47783	5.88002	6.44275	5.83909	6.14905	6.41677	6.06288	5.80666	6.57231	6.01778	6.79772	6.45946
POLA1	P09884	5.63745	6.04499	6.13919	5.8966	5.80521	6.25673	6.08382	5.98911	6.09541	6.06502	6.15691	6.11911	6.80406	6.954	7.03537	6.91704
PDCD6IP	Q8WUM4	6.67706	6.40398	6.86323	5.99993	6.51792	6.78302	6.19238	6.84389	6.32871	6.16693	6.32751	6.63645	5.7116	5.68205	5.41891	5.44644
ATRX	P46100	6.35975	6.35003	5.97655	6.54073	6.15134	5.92234	6.29448	5.71391	6.1477	6.24808	6.09235	5.93956	6.64231	6.58548	6.60674	6.42865
LRP1	Q07954	6.27954	6.81394	5.94044	6.18163	6.3204	6.30501	6.07492	6.09606	5.86177	6.3567	6.07854	5.97353	6.38754	6.49355	6.53898	6.29746
PSMA1	P25786-2	6.08299	6.15873	6.32808	5.97805	6.39448	6.44654	6.0799	6.3866	6.13828	6.24468	6.36839	6.28588	6.64977	6.19741	6.21666	6.04358
SIPA1L3	O60292	6.05481	6.35315	5.91587	6.42788	6.03818	5.97171	6.3103	5.89303	5.9154	5.99537	5.99479	6.02429	6.86289	6.5631	6.80455	6.87468
PSMC5	P62195	6.21878	6.03154	6.25147	6.07156	6.49789	6.24204	6.63309	6.29058	6.42995	5.78365	6.33076	6.27538	6.0914	6.14561	6.29148	6.41483
ARID2	Q68CP9	6.48199	6.42942	6.10667	6.84305	6.39238	6.25708	6.3717	6.18351	6.05798	6.34831	6.23037	6.12693	6.10751	5.90265	6.19956	5.9609
DLST	P36957	6.42564	6.42197	5.99411	6.70345	6.75443	6.293	6.48201	6.20849	6.08827	6.0743	5.87548	5.76077	6.77813	5.97219	6.23921	5.92855
ACSL4	O60488	6.30589	6.35203	6.231	6.80381	6.50167	6.35951	6.67473	6.00418	6.27928	6.04944	6.40312	6.3394	5.88065	5.75352	6.04112	6.02062
ACSL3	O95573	6.31869	6.39902	5.99718	6.62666	6.19379	6.08626	6.40968	5.9066	6.02606	5.85609	5.90304	5.91429	6.6261	6.39838	6.6683	6.66986
CCAR1	Q8IX12	6.3156	6.2718	5.874	6.42117	6.21494	6.04555	6.36822	6.06992	6.0685	6.04922	5.89699	5.67288	6.80811	6.49015	6.76565	6.66731
ITGAV	P06756	6.40403	6.37394	6.0158	6.39461	6.21186	6.24879	6.23619	6.13422	6.26921	6.24955	6.22023	6.16735	6.3989	6.23981	6.22012	6.21539
DHX15	O43143	6.00319	6.25286	6.10334	6.41241	6.02356	6.01894	6.24999	5.96577	6.18762	6.09253	6.05708	6.10295	6.44149	6.39982	6.90284	6.78563
PSAT1	Q9Y617	6.37641	6.15709	6.68653	5.68939	6.64789	6.92666	6.20023	6.69056	6.36938	6.36646	6.39488	6.45723	5.96083	5.92573	5.69962	5.4511
PGAM1	P18669	6.17989	6.14592	6.85078	5.91271	6.50803	6.81015	6.09967	6.98877	6.24753	6.53732	6.55921	6.61447	5.69689	5.90753	5.5001	5.44103
DNMBP	Q6XZF7	6.48371	6.13274	6.72468	6.0196	6.4416	6.74861	6.44488	6.6464	6.45316	6.2501	6.36258	6.30575	5.92246	5.80332	5.69653	5.56388
MED12	Q93074-2	6.44042	6.61197	6.20985	6.54793	6.3496	6.11488	6.50441	5.93983	6.36442	6.41008	6.06262	6.11015	6.13277	5.81212	6.18429	6.20464
SERPINH1	P50454	6.31824	6.49567	6.19157	6.59132	6.35079	6.27786	6.31323	5.98265	6.03106	6.04369	6.02258	5.88768	6.57777	6.32969	6.46417	6.22202
ROCK2	O75116	6.41628	6.364	6.97007	6.22715	6.35475	6.5848	6.39464	6.45796	6.34027	6.21064	6.42026	6.20029	5.82723	5.97651	5.55371	5.70143
HNRNPAB	Q99729-3	6.23206	6.42587	5.85163	6.80462	6.12591	6.00601	6.35928	6.12938	6.14448	6.13629	5.99121	6.17858	6.53843	6.22707	6.27955	6.56964
CAPRIN1	Q14444	6.49873	6.49591	6.7901	6.27895	6.56074	6.67119	6.11428	6.65381	6.60574	6.55725	6.65096	6.69867	5.6161	5.32099	5.21208	5.27449
EIF3E	P60228	6.52314	6.40652	6.56535	6.20351	6.44248	6.63129	6.40965	6.5934	6.45264	6.51325	6.35135	6.42765	5.59103	5.79246	5.45891	5.63739
POLR1A	O95602	6.51243	6.39246	6.11966	6.97412	6.25092	6.02845	6.46529	6.09867	6.2338	5.9503	6.14092	6.06818	6.30054	5.86338	6.28096	6.3199
COPB1	P53618	6.2942	6.15693	6.30076	5.98213	6.46059	6.47204	6.14335	6.40939	6.51534	6.4707	6.64901	6.68369	5.92128	5.9063	5.79134	5.84296
PCM1	Q15154-5	6.52034	6.56356	6.577	6.46697	6.38746	6.65917	6.29851	6.5154	6.32233	6.46453	6.54365	6.66544	5.48442	5.6704	5.35506	5.50576
EHBP1L1	Q8N3D4	6.47557	6.50455	6.36357	6.42002	6.24232	6.11982	6.38115	6.33976	6.53302	6.42812	6.50775	6.4124	5.9602	5.83576	5.69145	5.78453
RRP12	Q5JTH9	5.97366	5.94545	5.41439	6.5605	5.96669	6.03378	6.42037	6.05046	6.71312	5.77704	5.73239	5.88177	6.57165	6.93769	6.82396	7.1971
H1-3	P16402	6.02101	5.98787	5.48934	6.36264	6.01373	5.7504	6.35923	5.94241	5.89802	5.69651	5.55062	5.65572	6.72899	7.65766	7.31914	7.5667
RNF40	O75150	5.85808	6.33032	6.28237	6.39341	6.29726	6.11478	6.58671	6.01724	6.31819	6.18589	6.21115	6.26472	6.19329	6.23431	6.29184	6.42043
PSMD11	O00231	6.31264	6.39141	6.16301	6.10514	6.67022	6.53908	6.22389	6.69393	6.17266	6.20508	6.04752	5.80655	6.20891	6.20189	6.1823	6.07577
TUBB6	Q9BUF5	6.3524	6.07545	6.92653	6.0841	6.47461	6.55072	6.44727	7.1492	6.56046	6.25271	6.72169	6.84329	5.54775	5.49577	5.0593	5.45874
PYGL	P06737	6.42262	6.43331	6.94731	6.13328	6.17123	6.88991	6.05223	6.42909	6.51368	6.53069	6.72375	6.53975	5.4982	5.83341	5.40599	5.47556
PDCD11	Q14690	6.22528	6.576	5.79912	6.92809	6.14861	6.0283	6.39438	6.20291	6.0906	6.04156	5.653	6.0162	6.45056	6.32917	6.56072	6.55549
SMG1	Q96Q15	6.23171	6.35958	6.73984	6.24262	6.22708	6.4871	6.30743	6.27215	6.56793	6.52335	6.57617	6.71895	5.68319	5.52897	5.67871	5.85521
DDX24	Q9GZR7	6.10921	6.0142	5.94289	6.71155	6.18681	5.9483	6.54119	5.96405	6.09106	5.92282	5.9647	5.91524	6.52878	6.72121	6.57838	6.85962
MCM6	Q14566	5.7313	6.01683	6.10641	5.85374	5.83481	6.01199	6.00783	5.85248	6.03398	5.96209	6.45631	6.09768	7.08365	6.80355	7.26071	6.88664
FAM120A	Q9NZB2-6	6.52179	6.42379	6.60582	6.41389	6.38718	6.50321	6.19332	6.49204	6.55577	6.42759	6.4017	6.39761	5.80258	5.77956	5.45288	5.64128
SYNM	O15061	6.25232	6.24194	5.94463	6.47271	6.16917	6.11529	6.16801	6.14011	6.11404	6.02017	6.00128	5.94847	7.00995	6.4496	6.44682	6.5055
ZNF318	Q5VUA4	6.31629	6.50029	6.3692	6.96951	6.09666	6.08054	6.37488	5.96162	6.18109	6.4436	6.34576	6.15913	6.2782	5.8715	5.98512	6.06663
VCPIP1	Q96JH7	6.1144	6.35127	6.3752	6.15476	6.28075	6.51682	6.10552	6.4301	6.20387	6.26094	6.39983	6.42055	6.20071	6.13405	6.09114	5.96006
KDM3B	Q7LBC6	5.76785	5.99535	5.92323	5.93162	5.91645	6.06589	6.07378	6.00642	5.96455	6.3336	6.2493	6.10018	6.70172	6.97758	7.06129	6.93119
PSMA6	P60900	6.01957	6.00191	6.13958	6.06093	6.49691	6.33036	6.40419	6.14915	6.38447	5.94218	6.16907	5.91082	6.33515	6.38487	6.62948	6.64138
RPL6	Q02878	6.1128	6.5517	6.07819	6.27804	6.40624	6.79672	6.2772	6.64633	6.40956	6.17932	6.4529	6.22258	5.55301	6.6648	5.78821	5.58242
PSMD3	O43242	6.26758	6.33158	6.12352	6.25024	6.43886	6.50866	6.14002	6.25803	6.06784	6.22053	6.13889	6.08137	6.31871	6.37559	6.17261	6.30598
ZC3H14	Q6PJT7-2	6.30096	6.44373	6.06379	6.68476	6.4459	6.35036	6.12133	5.98367	6.15195	6.10927	6.13481	6.19661	6.75896	5.997	6.12829	6.12859
LETM1	O95202	6.43226	6.42049	5.72281	6.51884	6.23215	6.04552	6.35431	6.14868	6.14391	5.88138	5.88706	5.76445	6.72328	6.42862	6.65422	6.64201
SART3	A0A499FI31	5.86354	5.89278	5.86887	6.22498	6.1529	5.99075	6.38654	5.92997	6.0953	5.87425	5.82907	5.67893	7.03996	7.0025	7.05644	7.11325
HNRNPF	P52597	5.99204	6.14204	5.59989	6.312	6.37122	6.05736	6.76297	6.15557	6.61545	5.62954	5.87766	6.05522	6.36436	6.4693	6.53064	7.06475
RUVBL1	Q9Y265	6.21416	6.15384	6.45474	6.30187	6.43962	6.43705	6.26677	6.31035	6.31897	6.3507	6.3526	6.23178	6.21417	5.99891	5.92022	6.03424
AP3B1	O00203	6.4107	6.31192	6.69467	6.15814	6.19108	6.46047	6.38027	6.66792	6.23051	6.51482	6.42062	6.20165	5.86923	6.02044	5.86389	5.60367
PNPLA6	Q8IY17	6.07836	6.40493	5.88143	6.38652	5.84578	5.78231	6.12882	5.89495	6.30664	6.36423	6.29547	5.94608	6.60907	6.70397	6.70609	6.66595
RPA1	P27694	5.54805	5.63567	5.92346	5.31173	5.7852	6.27399	5.72385	5.72953	6.09268	6.20598	6.06224	5.86365	7.30736	7.40779	7.81068	7.31814
CSDE1	O75534-4	6.59877	6.64852	7.02174	6.483	6.41931	6.60554	6.39535	6.67702	6.84197	6.75701	6.86986	6.77792	5.0298	5.17709	4.81526	4.88183
TKT	P29401-2	5.75858	5.73718	6.21775	5.38304	6.0799	6.54091	5.91355	6.43185	6.12223	6.86156	6.04862	5.84224	6.39045	7.61198	7.18319	6.87696
PMPCA	Q10713	6.21154	6.21564	5.7881	6.62087	6.40415	5.98231	6.5672	6.01357	6.21858	5.78243	5.87672	5.8901	6.47468	6.39263	6.73315	6.82833
UCHL1	P09936	6.14792	4.94795	6.13231	4.34652	6.26906	5.86099	5.73231	7.16003	5.69528	6.72983	5.91411	6.95688	6.23283	7.54947	7.16972	7.1548
FUBP1	Q96AE4-2	6.43045	5.83036	6.13743	5.6789	6.74483	5.88464	6.00449	6.23629	6.26411	6.1539	6.39912	6.32068	7.19671	6.086	6.40697	6.22512
SDHA	P31040	6.28556	6.33503	5.56987	6.43172	6.34551	6.11157	6.17788	6.08205	6.104	6.12413	5.89755	5.96841	6.77203	6.4246	6.6423	6.7278
SPEN	Q96T58	6.4537	6.66689	6.02921	7.05264	6.0528	5.83558	6.21872	6.00192	6.18124	5.91933	6.1275	6.12854	5.84196	6.30775	6.6679	6.51432
MYO1B	O43795	6.16083	6.3899	6.03044	6.25944	6.1802	6.10631	6.12215	5.83805	6.1971	6.16682	6.1556	6.14931	6.5622	6.45088	6.78018	6.45057
AP1G1	O43747-2	6.23989	6.43411	6.7884	6.63755	6.0547	6.64291	6.24162	6.28598	6.07871	6.3938	6.3041	6.25429	5.92458	5.97487	5.79701	5.94747
DDX54	Q8TDD1-2	6.42008	6.52356	5.87824	6.72495	5.95657	5.88987	6.4629	6.13048	6.24925	6.15877	6.11792	6.38029	5.92132	6.37666	6.27154	6.5376
CEP170	Q5SW79	6.18051	5.92533	6.55174	6.02298	6.1617	6.17905	6.16749	6.04967	6.65187	6.53846	6.67838	6.49023	6.04787	6.09692	6.01067	6.24713
POLD1	P28340	5.66651	6.02933	6.08894	5.61229	5.86901	6.2864	6.01089	5.87136	6.02412	6.13403	6.32287	6.05537	7.03222	7.04033	7.15303	6.8033
POLE	Q07864	5.97468	6.38134	6.33241	6.41885	5.98948	6.24248	6.08696	6.12622	6.29223	6.31259	6.35507	6.54667	6.04198	6.22395	6.20017	6.47491
ACAT1	P24752	6.27682	6.02236	5.81825	6.27798	6.47557	6.01108	6.50771	6.30719	6.19542	5.98673	6.25473	6.2531	6.54759	6.22973	6.46759	6.36815
UBE4B	O95155-4	6.0563	6.3572	6.64797	6.14131	6.1787	6.5755	6.24919	6.47512	6.25955	6.26187	6.22515	6.15726	6.05351	6.11672	6.10371	6.14094
NCOR2	C9J0Q5	6.43168	6.19281	6.37723	6.53378	6.37593	6.08557	6.16924	5.91814	6.28686	6.41513	6.4438	6.38184	6.38465	6.15128	5.96163	5.89044
UBA2	Q9UBT2	5.70204	5.73911	6.26488	5.16661	5.62935	6.26318	5.67297	6.1651	5.96743	5.99564	6.34916	5.92732	6.85443	7.64241	7.71102	6.94936
EIF5B	O60841	6.52721	6.35266	6.52797	5.90023	6.30244	6.38765	6.10724	6.3358	6.34426	6.35211	6.28481	6.26996	5.53422	6.59928	5.8715	6.30265
PSMC1	P62191	5.82982	6.29293	6.26595	6.20537	6.06537	6.65068	6.71573	6.40089	6.30349	6.14235	6.5233	6.2669	5.86187	6.37818	6.0453	6.05187
HSPA4L	O95757	6.14499	6.16654	6.59734	5.99279	6.3041	6.46512	6.19117	6.26501	6.24838	6.43564	6.47329	6.21512	6.21756	6.11437	6.12706	6.04151
NRDC	O43847-2	6.21562	6.32789	7.11386	6.10948	6.37403	6.7161	6.25301	6.60231	6.31821	6.45994	6.73246	6.55741	5.54112	5.73692	5.47573	5.46591
ANKLE2	Q86XL3	6.37957	6.49301	6.32395	6.56811	6.10746	6.07972	6.26155	5.9815	6.21552	6.05965	6.07848	6.12496	6.51461	6.17579	6.38159	6.25452
LONP1	P36776	6.31909	6.35311	5.90007	6.47621	6.39225	6.11313	6.25379	6.11512	6.04321	5.99055	5.93495	5.70203	6.88994	6.36254	6.78271	6.37132
RAB2B	Q8WUD1	6.21091	6.25852	5.99538	6.43165	6.24268	6.15638	6.50966	5.94095	6.17136	6.25806	6.0337	5.89669	6.44756	6.12381	6.86962	6.45306
HLTF	Q14527	5.31542	5.82714	6.13464	5.55769	5.66554	6.1904	5.95347	5.92004	6.3086	6.34442	6.36407	6.30362	7.0341	7.16499	6.8684	7.04747
DNAJC13	O75165	6.00159	6.49243	6.06216	6.35623	5.88322	6.48355	6.26067	6.10773	5.93227	6.36174	6.58971	6.64642	5.93596	6.20212	6.32999	6.35421
GMPS	P49915	5.91495	5.90034	6.3899	5.61354	5.92414	6.40161	5.76655	6.34263	6.04591	6.17862	6.29948	6.2895	6.33793	7.11499	6.95356	6.52635

PLS3	P13797	6.2182	6.00679	6.47953	5.79273	6.35216	6.53408	5.97793	6.36217	6.2097	6.22634	6.15957	6.28542	6.81457	6.34267	6.25675	5.98138
ME2	P23368	6.3804	6.22011	6.09879	6.66621	6.1655	5.946	6.45815	5.93429	6.21631	6.13301	6.04447	6.00638	6.47379	6.20515	6.4872	6.56424
NCBP1	Q09161	6.03331	6.24756	5.82039	6.21621	6.15419	6.2781	6.04078	5.98552	6.07304	6.05817	6.05692	5.92973	6.95158	6.49382	7.00907	6.6516
CD2AP	Q9Y5K6	6.36445	6.25265	7.05638	6.15045	6.46166	6.71255	6.22393	6.28744	6.41706	6.20203	6.47499	6.42364	5.90294	5.8961	5.48679	5.68694
DIS3	Q9Y2L1	5.55409	5.77467	5.96321	5.45477	6.05732	5.85844	5.94295	5.92314	6.17194	5.97662	6.1514	5.91964	7.2272	7.25225	7.85009	6.92229
NUP54	Q7Z3B4	6.29186	6.23439	6.04959	6.26204	6.50866	6.04197	6.14854	6.19182	6.2401	6.03195	6.2959	6.29533	6.82829	5.84967	6.5434	6.18648
USP10	Q14694-2	6.28309	6.24613	6.53804	6.09136	6.36101	6.48586	6.19925	6.37996	6.36598	6.31103	6.40577	6.47255	5.83709	6.03536	5.97137	6.01614
THOC1	Q96FV9	6.23722	6.41537	6.08296	6.58266	6.33253	6.04587	6.16154	5.79804	6.33395	6.09163	6.15284	6.04383	6.45915	6.1764	6.63652	6.44951
DOCK7	Q96N67	6.39016	6.42327	6.4955	6.17552	6.21244	6.5399	6.10551	6.3455	6.20021	6.33702	6.1788	6.15054	6.06562	6.12917	6.1561	6.09474
CTNNA1	P35221	6.62108	6.45777	6.2628	6.40677	6.10716	6.28008	6.64671	6.12434	6.52269	6.14305	6.21939	6.13047	5.89258	5.97748	6.00285	6.20479
LIG3	P49916	5.84011	6.05559	5.83228	6.01071	5.96194	6.18767	6.15423	5.93581	5.85373	6.04419	6.28159	6.14386	6.86074	7.01374	7.04749	6.77632
SUN1	O94901-9	6.3744	6.29867	5.85285	6.55944	6.11142	6.01554	6.34043	5.8523	6.10625	6.96209	5.81357	5.73127	7.00923	6.50208	6.945	6.52544
KIAA1671	Q9BY89	5.88406	5.99361	5.81123	6.05864	6.08265	6.18012	6.19779	5.81749	5.92695	5.92256	5.73026	5.97653	7.36792	6.85229	7.12644	7.07146
LUZP1	Q86V48	6.04498	5.989	5.93743	6.28419	6.04188	5.94278	6.09201	5.93333	6.53389	6.22741	6.38112	6.20672	6.71674	6.54639	6.63859	6.48354
PFKP	Q01813	5.94921	6.22206	6.8926	6.08303	6.40286	6.97752	6.52597	6.4375	6.60515	6.26019	6.47764	6.42738	5.63758	5.78182	5.5767	5.74279
EHMT2	Q96KQ7-2	6.31813	6.5777	6.04564	6.74301	6.35398	6.1051	6.25796	6.08778	6.29996	6.16001	6.13698	6.03517	6.3762	6.06205	6.30457	6.13577
PWP2	Q15269	6.3699	6.27339	5.934	6.78251	6.40928	5.80965	6.48255	5.90961	6.19186	5.93528	6.01154	5.98307	6.64391	6.14904	6.62773	6.48668
UQCRC1	P31930	5.90214	5.59198	5.40414	5.8054	6.42781	5.97983	6.32141	6.76093	6.09772	6.3128	6.12948	6.47867	6.49421	6.55812	6.77281	6.96255
PKN2	Q16513-2	6.47193	6.33059	6.54432	6.37012	6.30052	6.47735	6.3607	6.23898	6.31814	6.59004	6.3867	6.48324	5.94712	5.80628	5.76332	5.61063
INF2	Q27J81	6.22213	6.40258	6.34149	6.22267	5.98698	6.45486	6.10678	6.33587	6.50425	6.42267	6.58543	6.34166	5.86401	6.07625	6.20714	5.92525
AXL	P30530	6.63049	6.51848	6.61017	7.15739	6.31268	6.50542	6.58841	6.31315	6.72001	6.68201	6.66381	7.04441	4.91143	4.97123	4.96907	5.40185
LAS1L	Q9Y4W2	6.46584	6.04005	6.24333	6.56736	6.19089	5.67044	6.58086	5.85887	6.4889	5.95043	6.42258	6.05563	6.58461	5.86524	6.56375	6.45122
SPAG9	O60271-4	6.40889	6.35721	7.01904	6.19515	6.52437	6.67024	6.41161	6.7507	6.46037	6.36026	6.77836	6.55751	5.56977	5.47369	5.25588	5.20695
AHCY	P23526	6.17573	6.09119	6.85285	5.93904	6.4776	6.77236	6.38338	6.65641	6.49122	6.4104	6.27973	6.22766	5.71386	5.82757	5.90799	5.79302
THADA	Q6YHU6	6.41507	6.32587	6.93841	6.20593	6.27006	6.8067	6.3911	6.8087	6.61791	6.63025	6.54381	6.64404	5.42759	5.47101	5.16914	5.33444
DHX16	O60231	5.90466	6.42041	6.09447	6.11034	6.1151	6.18748	6.15567	5.9256	6.18159	6.11939	6.0392	6.11786	6.56627	6.55887	6.96007	6.54302
ZFR	Q96KR1	6.49943	6.55507	6.23112	6.71788	6.50675	6.12708	6.62043	6.00989	6.10684	5.97645	5.88073	5.78635	6.28676	6.15052	6.31199	6.2327
TXNDC5	Q8NBS9	6.46889	6.29381	5.89118	6.61903	6.38838	6.1512	6.14951	6.28954	6.28356	6.0175	5.98684	6.47178	6.67903	5.87483	6.10582	6.32911
PDIA6	Q15084-2	6.44717	6.49307	6.02447	6.10849	6.42372	6.20822	6.18175	6.13312	6.04013	6.04134	5.91576	5.95216	7.05115	6.27893	6.28238	6.41817
MSH2	P43246	5.37133	5.9684	5.82501	5.48154	5.84094	6.18124	5.83525	5.83352	5.87386	6.15375	6.3443	5.94151	7.33109	7.48829	7.42215	7.10782
EIF5	P55010	6.07221	6.0087	6.82958	6.039	6.45888	6.6369	6.45195	6.48507	6.51204	6.18357	6.51014	6.42689	5.91327	5.90918	5.72517	5.83745
RBM25	P49756	6.24636	6.23566	5.92469	6.55964	6.13464	5.92641	6.36902	5.85583	6.19451	5.9368	6.01794	6.03225	6.78698	6.34977	6.7576	6.67189
THRAP3	Q9Y2W1	6.14139	6.42303	5.96274	6.54629	5.91874	5.87965	6.32498	5.8649	6.16796	6.16632	6.26667	6.28504	6.6294	6.30582	6.45979	6.65728
AP2M1	Q96CW1	6.07413	6.33089	6.45529	6.57281	6.10118	6.29969	6.32605	5.91192	6.30286	6.41801	6.17366	6.406	6.1844	6.012	6.44954	5.98155
ERO1A	Q96HE7	6.34129	6.49367	5.89929	6.53526	6.38804	6.17224	6.26842	6.17986	5.95447	6.12446	5.91071	5.93796	6.41628	6.49253	6.51176	6.37377
DHX38	Q92620	5.75321	6.19827	5.90134	5.9839	6.12496	6.15503	6.11633	5.85288	5.95948	6.13972	5.97076	6.00384	6.7613	6.91531	7.24832	6.91538
PHIP	Q8WWQ0	6.17434	6.31841	5.70704	6.97831	5.77335	5.98899	6.30204	6.08793	5.98213	6.11929	5.84535	6.1286	6.32903	6.78938	6.67328	6.80253
CPSF2	Q9P2I0	6.38027	6.18957	6.14211	6.54073	6.27675	6.09629	6.28089	5.98372	6.25144	6.04624	6.087	6.16263	6.6531	5.86596	6.48716	6.55613
G3BP1	Q13283	6.43043	6.50842	6.68859	6.33924	6.46027	6.4708	6.24274	6.51501	6.57523	6.54307	6.74658	6.77922	5.72265	5.40362	5.18437	5.38976
PPIB	P23284	6.17905	6.44451	5.65986	6.369	6.32208	6.07872	6.23814	6.18372	6.19998	6.02492	6.04358	6.09923	6.67956	6.32461	6.75583	6.39721
PLAA	Q9Y263	6.20067	6.17499	6.48204	6.35844	6.38846	6.28359	6.24677	6.22518	6.30001	6.42397	6.62382	6.33625	6.06904	5.93875	6.11212	5.83592
LMAN2	Q12907	5.95548	6.39452	5.89136	6.35572	6.07273	6.05944	6.10516	6.10501	5.99301	5.96773	6.11864	6.24185	6.75088	6.71097	6.71956	6.55793
STAT1	P42224	6.08022	6.45786	6.64291	6.05042	6.23633	6.92598	6.26357	6.84752	6.10316	6.57143	6.2728	6.34019	5.60785	6.02522	5.6713	5.90322
PRPH	P41219-2	6.53641	6.32349	6.3733	6.59401	6.66638	5.7909	6.41997	6.11695	6.45316	5.75891	5.92551	5.98416	6.39454	6.07205	6.30849	6.28177
EDC4	Q6P2E9	5.97789	5.96711	6.67584	5.91313	6.17159	6.46344	6.11839	6.0792	6.56948	6.36801	6.58984	6.7143	6.21092	6.14179	6.08237	5.95671
DBN1	Q16643	6.44183	6.34007	6.03477	6.17319	6.41494	6.11041	6.13167	6.21499	6.21365	6.17193	6.32564	6.10551	7.01643	6.04401	6.12527	6.13568
YWHAZ	P63104	6.18917	5.86502	6.62909	5.65494	6.36051	6.51138	6.05386	6.22701	6.48919	6.51307	6.55089	6.2918	6.4185	6.172	6.14345	5.93012
CRYBG1	Q9Y4K1	6.00187	6.27164	6.10409	6.07261	5.96329	6.25192	6.01915	6.18787	6.24684	6.17042	6.32585	6.23347	6.37236	6.56422	6.66827	6.54613
ARID1A	O14497	6.39346	6.32537	6.10755	6.40196	6.23527	6.09961	6.37022	6.10689	6.08534	6.16	6.23928	6.06533	6.75723	6.05815	6.37545	6.21889
HADHB	P55084	6.12576	6.2694	5.75332	6.43527	6.42759	6.10263	6.59601	6.07767	6.14944	5.99395	5.80618	5.67773	6.75873	6.65184	6.60088	6.57359
EEA1	Q15075	6.87522	6.61024	6.20894	6.32375	6.43737	6.06273	6.16162	6.08051	6.35115	6.23371	6.5722	6.41054	6.56472	5.64899	5.824	5.6343
HK1	P19367-3	6.36799	6.38454	6.09545	6.48717	6.31186	6.11328	6.17027	6.01135	6.23414	6.13853	5.82029	5.87349	6.55316	6.27481	6.67615	6.4875
DLD	P09622	6.30637	6.56115	5.74857	6.50484	6.32629	6.05639	6.13077	6.12866	6.00404	6.06575	5.97278	5.98339	6.86512	6.40781	6.50734	6.43072
SLC25A12	O75746	6.41058	6.37058	5.98197	6.64599	6.20632	5.93342	6.41424	5.92572	6.11309	6.12302	6.08157	5.94052	6.6402	6.24365	6.48518	6.48395
ETFA	P13804	6.45692	6.41076	5.74065	6.41341	6.70093	6.36365	6.1943	6.44016	6.39811	6.22624	6.17851	5.88621	6.55741	5.90056	6.09064	6.04154
PPL	O60437	6.33649	6.40277	5.75915	5.97973	6.26219	5.99395	5.98091	6.15881	5.99446	6.29426	5.99006	5.84966	7.35402	6.84098	6.5245	6.27807
EHMT1	Q9H9B1	6.2672	6.51875	6.02999	7.03722	6.05106	6.11214	6.57747	6.14669	6.22538	6.13674	6.21069	6.13913	6.1549	6.09577	6.18727	6.1096
IARS2	Q9NSE4	6.396	6.46871	5.79316	6.66102	6.27972	5.98992	6.29781	6.15415	6.09702	6.07823	5.89101	5.83675	6.70176	6.36986	6.52452	6.46038
FERMT2	Q96AC1-3	6.12795	5.98652	6.36665	5.91385	6.19463	6.35942	6.30564	6.10179	6.47358	6.64866	6.28811	6.22581	6.49439	6.19639	6.50168	5.81495
UPF1	Q92900	6.42364	6.26891	6.94666	6.38379	6.17056	6.43315	6.28275	6.14123	6.47828	6.67117	6.60886	6.623	5.72501	5.77525	5.52643	5.54131
WDR62	O43379-4	6.21707	6.08184	7.02664	6.04881	6.18699	6.40705	6.12804	6.34339	6.54781	6.69826	7.01581	7.01028	5.47443	5.76638	5.50052	5.54666
UTP14A	Q9BVJ6	6.22934	6.31995	6.05182	6.88392	6.15917	5.9403	6.50033	6.08219	6.16459	5.96971	6.02854	6.20997	6.35965	6.16238	6.49448	6.44367
IGF2BP2	Q9Y6M1	6.23867	6.35531	6.43814	6.06198	6.4612	6.61412	6.15108	6.47692	6.40685	6.4751	6.40193	6.45415	5.92542	5.9761	5.84811	5.71492
UBTF	P17480	6.2994	6.40631	5.92178	6.62432	6.07408	6.07125	6.16731	6.06745	6.02177	6.25832	5.88955	5.97503	6.53926	6.559	6.5217	6.60347
SRPRA	P08240	6.31321	6.10157	5.64129	6.49162	6.07494	5.78892	6.82902	5.88741	6.22992	5.4018	5.58392	5.84036	6.52832	6.95514	7.00508	7.32748
POLR2B	P30876	6.60194	6.67453	6.33799	6.73119	6.34082	6.19398	6.56967	6.48019	6.30284	6.19163	6.34136	6.19328	5.79221	5.71818	5.68698	5.84321
CORO1C	Q9ULV4-3	6.19428	6.5332	6.06144	6.38662	6.43402	6.51493	6.15782	6.2274	5.99534	5.94618	6.01596	5.91166	6.49776	6.48823	6.44922	6.18594
RPS7	P62081	6.31373	6.50724	6.87634	6.26605	6.33018	6.31727	6.27122	6.39522	6.43109	6.61323	6.68397	6.55688	5.62319	5.7131	5.62548	5.47582
HECTD1	A0A087X2H1	6.44376	6.32421	7.3641	6.32022	6.44858	6.81	6.2794	6.46007	6.69255	6.5947	6.76014	7.0256	5.25319	4.97946	4.97578	5.26825
CEBPZ	Q03701	6.29437	6.26742	5.85327	6.66901	6.19337	5.76756	6.39176	5.89251	6.00559	5.81625	5.95372	5.8629	6.55721	6.59066	6.95059	6.93382
HEXB	P07686	6.57861	5.9921	5.89727	5.79861	6.63043	5.84688	6.0811	6.47396	5.85511	6.47256	6.12338	6.42436	6.65516	6.41778	6.37077	6.38194
AK2	P54819	5.85244	5.11164	5.62219	5.2992	6.75678	5.16571	5.9897	6.69423	5.7174	6.46249	6.11129	6.63998	6.98287	6.84913	7.41082	7.3341
SEC31A	D6REX3	6.25737	6.01066	6.88883	5.85517	6.07482	6.42803	6.08978	6.38264	6.14923	6.40732	6.50839	6.06394	6.50026	6.26232	6.21515	5.9061
ZC3HAV1	Q7Z2W4	6.32962	6.4296	6.36894	6.44385	6.0818	6.1941	6.2408	6.00211	6.23679	6.48136	6.46613	6.30079	6.16722	6.11509	6.12645	6.01534
USO1	O60763-2	6.28708	6.15947	6.3945	6.0351	6.09331	6.01864	6.07179	6.3957	6.34771	6.36204	6.19866	6.29871	6.416	6.27847	6.43295	6.20987
EIF2A	Q9BY44	6.14637	6.38403	6.58127	6.36764	6.22411	6.48664	6.33484	6.33351	6.56026	6.32173	6.4931	6.36062	5.52207	5.88656	5.80646	6.19078
KIF11	P52732	6.55368	6.37806	6.83827	6.20325	6.38621	6.60211	6.28288	6.6281	6.90983	6.4756	6.76309	6.69708	5.42745	5.37523	5.17046	5.3087
MOGS	Q13724	6.07617	6.08542	5.88218	6.73334	6.1666	5.97072	6.13189	5.88917	6.08519	6.15943	6.09288	6.20696	6.69689	6.61966	6.54808	6.65542
UMPS	P11172	6.24959	6.1104	6.68379	5.87421	6.293	6.61522	6.18779	6.71082	6.49676	6.40268	6.61139	6.50185	5.78911	5.80899	5.86063	5.80375
U2SURP	O15042	6.22525	6.32989	5.85747	6.75096	6.08413	5.95848	6.29084	5.97684	6.04436	6.14994	6.05335	6.01412	6.30569	6.55641	6.62915	6.77311
ARF3	P61204	6.33887	6.48491	6.79464	6.54182	6.28953	6.44079	6.60993	6.24058	6.33523	6.30008	6.31741	6.21124	5.73022	5.95619	5.80302	5.60557
SARS1	P49591	6.33848	6.07914	6.87846	5.9936	6.55071	6.73631	6.44449	6.59486	6.45095	6.31621	6.62943	6.37399	5.84881	5.84152	5.59092	5.33212
EIF2S3	P41091	6.37788	6.25284	6.62913	6.16461	6.35068	6.39091	6.14697	6.29324	6.3642	6.43443	6.41819	6.3154	6.0847	6.07788	5.97507	5.72388
SNRNP70	P08621	6.13463	6.34023	5.63627	6.5058	6.2215	5.85799	6.40413	6.08063	6.01117	5.83195	5.86807	5.68838	6.61015	6.81734	7.09766	6.89409
RCC1	P18754-2	6.23544	6.4231	5.78591	6.51576	6.0947	5.99656	6.39204	6.19858	6.00687	6.11363	5.84178	6.0228	6.50095	6.42724	6.82078	6.62386
HTT	P42858	6.11581	6.20528	6.56754	5.8297	6.23476	6.67441	6.17066	6.50059	6.53372	6.4788	6.3548	6.41252	5.82791	6.10604	6.01948	5.96796
SETX	Q7Z333-4	6.29363	6.49023	6.19375	6.6535	6.15874	6.2604	6.32592	6.23564	6.19453	6.37573	6.27799	6.2655	5.92429	6.11493	6.07687	6.15833
GOLGA2	Q08379	6.63129	6.54424	5.99658	6.83024	6.54603	6.30206	6.33776	6.22927	6.24568	6.27316	6.26149	6.12177	6.34189	5.67676	5.88503	5.77675
RSL1D1	O76021	6.16042	6.23907	5.67934	6.52177	6.07032	5.66286	6.35732	5.73042	5.97812	5.78477	5.57133	5.68847	6.58874	7.34411	7.17312	7.44982
AARS2	Q5JTZ9	6.3422	6.26508	5.92388	6.60371	6.26952	5.98819	6.20967	6.04341	6.15927	6.16576	5.95484	5.96224	6.74524	6.40881	6.47114	6.48702
MYO9B	Q13459	6.26369	6.21359	6.82582	6.0094	6.26751	6.76466	6.26756	6.57953	6.27728	6.59497	6.50705	6.4285	5.69651	6.00006	5.74624	5.55763
PARP14	Q460N5	6.18124	6.26069	6.85346	6.28405	5.96999	6.40777	6.21007	6.29018	6.71853	6.91871	6.87814	6.78753	5.53716	5.67676	5.50672	5.51901
PRRC2B	Q5JSZ5	6.68719	6.46006	6.69749	6.46607	6.04602	6.29303	6.38173	6.08666	6.48618	6.67579	6.7326	6.61769	5.67783	5.76877	5.47023	5.45265
IPO4	Q8TEX9-2	6.30204	6.08209	6.84436	6.17895	6.29962	6.90143	6.36884	6.75367	6.54913	6.1662	6.64194	6.79916	5.56586	5.7197	5.19691	5.63011
ASPM	Q8IZT6	6.78842	6.83616	7.0749	7.06801	6.23409	6.54921	6.6601	6.84948	6.92558	6.80226	7.0461	7.20637	4.21245	4.78559	4.14361	4.81769
MYO5A	Q9Y4I1-3	6.15269	6.36256	6.38579	5.94005	6.18903	6.23781	6.19332	6.16417	6.44954	6.62505	6.55515	6.49341	5.8166	6.15168	6.06677	6.21637
NOLC1	Q14978-2	6.17613	6.45189	5.76181	6.88334	5.99593	5.94418	6.26033	6.09178	6.13716	5.95897	5.80685	6.1226	6.42251	6.34372	6.63568	7.00712
PYGB	P11216	6.63749	6.42381	7.0227	6.31539	6.24105	6.69288	6.0616	6.34678	6.46236	6.68694	6.69106	6.61907	5.52194	5.62656	5.41467	5.2357
WASHC2A	Q641Q2	5.9671	6.0323	6.41301	5.78539	6.01653	6.3675	6.02796	6.23222	6.35894	6.53932	6.41971	6.46644	6.31845	6.47148	6.26899	6.31466
LTN1	O94822-3	6.04755	5.87847	6.41325	5.83089	6.14829	6.41594	6.02926	6.3609	6.55312	6.54694	6.44862	6.65646	6.09289	6.36308	6.02551	6.18882
PSMC6	P62333	6.15722	6.26555	6.35119	6.04686	6.34034	6.3115	6.18018	6.21776	6.20727	6.24128	6.49182	6.51275	6.64194	5.98328	6.05069	6.00037
WAPL	Q7Z5K2-3	5.94028	6.47598	6.03202	6.44362	6.07105	6.18194	6.20621	6.02451	6.06847	6.08104	5.98636	6.11099	6.56026	6.62684	6.61312	6.5773
PSME4	Q14997	6.02116	6.08627	6.25771	6.35278	6.09353	6.20756	6.26963	6.31999	6.28475	6.18187	6.37309	6.38327	6.23328	6.14658	6.2182	6.57031
PSMC4	P43686	6.26503	6.20045	6.54874	6.12723	6.70443	6.76225	6.19863	6.40128	6.16125	6.28793	6.42149	6.35815	6.28064	5.71784	5.70117	5.8635
SEPTIN7	Q16181-2	6.15707	6.17787	6.57936	6.11263	6.23652	6.4518	6.20076	6.28594	6.2666	6.20384	6.32627	6.08786	6.19919	6.37897	6.24144	6.09388

IDE	P14735	6.04658	6.35454	6.62693	5.88624	6.442	6.6553	6.1641	6.67595	6.25237	6.54915	6.32244	6.40388	5.84085	6.11738	5.82786	5.83441
SLK	Q9H2G2-2	6.27782	6.51212	6.68842	6.12734	6.27331	6.72809	6.28687	6.72822	6.29648	6.70819	6.44804	6.44087	6.60382	6.07269	5.48905	5.41866
SRCAP	Q6ZRS2	6.30278	6.15241	6.04374	6.81719	6.009	5.9226	6.31625	5.89449	6.15491	6.25702	6.03927	5.95573	6.44344	6.49711	6.60232	6.59175
UACA	Q9BZF9	6.42953	6.57099	6.35957	6.35681	6.63498	6.51097	6.35661	6.58796	6.50042	6.44185	6.56585	6.40389	5.83633	5.5719	5.40627	5.46607
PREP	P48147	6.33863	6.04524	6.73017	5.67339	6.32775	6.84898	6.21894	6.86931	6.32979	6.36927	6.19557	6.12868	5.91939	6.20099	5.87458	5.92932
SCRIB	Q14160-3	6.53532	6.35091	6.0809	6.38077	6.32506	5.90907	6.53108	6.03794	6.62037	6.13297	6.35562	6.16415	6.48892	5.87469	6.1165	6.09574
DPP3	Q9NY33	6.17637	6.20899	6.72582	5.71632	6.44463	6.74608	6.28867	6.66231	6.41801	6.27751	6.62415	6.47023	5.72878	6.05043	5.72833	5.73338
H2AX	P16104	6.35704	6.22234	5.63626	6.40349	6.06512	5.6206	6.62765	5.85111	6.06256	5.94614	5.85506	5.83998	6.96794	6.62098	6.83025	7.09347
MON2	Q7Z3U7-5	6.38711	6.13349	6.87285	5.96942	6.17694	6.49383	6.22058	6.32531	6.51711	6.47955	6.44428	6.66586	6.05831	5.92955	5.80805	5.51775
PGD	P52209	6.23439	6.255	6.86887	5.86433	6.37587	6.74101	6.19336	6.69848	6.26973	6.35163	6.44333	6.43328	5.66223	6.14942	5.74641	5.71267
KMT2B	Q9UMN6	6.27751	6.72642	5.72352	6.96628	6.05045	5.87063	6.54092	6.25903	6.44509	6.38131	6.38746	6.2493	5.94577	6.2011	5.87574	6.09947
FXR1	P51114	6.48239	6.8325	6.61208	6.48063	6.46356	6.66513	6.09504	6.56166	6.29985	6.59388	6.46577	6.34599	5.55885	5.56287	5.59159	5.38819
TRIO	O75962	6.37475	6.36551	6.87925	6.14341	6.32459	6.50939	6.3271	6.4899	6.58416	6.33491	6.60739	6.51335	5.6717	5.82138	5.58056	5.47263
OLA1	Q9NTK5	6.19831	6.14993	6.95541	6.08832	6.4776	6.74766	6.39957	6.5934	6.50244	6.50676	6.49812	6.50807	5.53953	5.76547	5.38055	5.68887
PSMA7	O14818	6.23304	6.33021	6.23063	5.88212	6.49502	6.45636	6.27076	6.40503	6.11241	6.14972	6.20459	6.07905	6.5225	6.31842	6.26214	6.04801
SERBP1	Q8NC51	6.22436	6.51874	6.49147	5.92818	6.33773	6.62123	6.18209	6.65896	6.64757	6.29462	6.52318	6.31889	5.71729	5.87285	5.76223	5.90064
VIRMA	Q69YN4	6.41305	6.41622	6.01311	6.7204	6.15633	6.13852	6.36382	6.11732	6.0868	6.05661	6.04866	6.01657	6.32241	6.20742	6.32578	6.59698
NCAPG	Q9BPX3	6.39232	6.4122	6.92228	6.32571	6.22174	6.60402	6.46136	6.46214	6.59597	6.59789	6.47375	6.67486	5.45462	5.60908	5.28352	5.50851
IMPDH2	P12268	6.27432	6.18689	6.82335	5.97018	6.38708	6.53192	6.05799	6.73127	7.00362	6.31479	6.69026	6.58472	5.73733	5.80189	5.46467	5.43973
FAT1	Q14517	6.51341	6.40958	6.31989	6.81567	6.24131	6.32417	6.59384	6.22456	6.4163	5.9511	6.19569	6.05009	6.30876	5.78555	5.92739	5.92269
UGP2	Q16851	6.33947	6.2452	6.56839	5.91708	6.44167	6.76784	6.20773	6.61407	6.26823	6.38195	6.35598	6.25934	5.99562	6.07384	5.77874	5.78485
OSBPL8	Q9BZF1	6.21637	6.37383	5.80446	6.30941	5.97268	5.92402	6.27723	5.83703	5.95816	6.07108	6.06435	6.2451	6.5959	5.65645	6.8751	6.91883
NDUFS2	O75306	6.20498	6.11271	5.81642	6.34235	6.35858	6.0634	6.2653	6.20711	6.06289	6.16881	5.91875	5.87361	6.74039	6.37097	6.70665	6.7871
ATP2B1	P20020-1	6.18383	6.45774	5.9549	6.39578	6.06119	6.07904	6.25154	5.92174	6.02309	6.08936	5.98746	5.80432	6.65502	6.52572	6.79657	6.8127
UQCRC2	P22695	6.30098	6.33065	5.99275	6.73186	6.2822	6.09721	6.56959	6.04347	6.26562	5.95049	6.00316	6.03946	6.48985	6.10688	6.5123	6.28353
MPDZ	O75970	6.25193	6.29203	6.29195	6.29648	6.12046	6.41862	5.97558	6.51593	6.17477	6.22437	6.35401	6.35163	6.25528	6.00172	6.21874	6.25651
CPD	O75976	6.79084	6.55566	6.31107	6.84062	6.24116	6.09105	6.22495	6.13645	6.03584	6.1411	6.33165	6.23746	6.19417	6.06477	5.97823	5.82497
GSPT1	P15170-3	6.42864	6.28339	6.63363	6.10277	6.49602	6.83774	6.27642	6.58403	6.4838	6.36583	6.52574	6.50821	5.74768	5.71575	5.44509	5.56526
ZFYVE16	Q7Z3T8	6.35303	6.58539	6.8849	6.39884	6.29557	6.47105	6.05527	6.18523	6.53841	6.65185	6.89815	7.00671	5.51255	5.38473	5.33905	5.43927
HNRNPD	Q14103-2	6.17109	6.35975	5.68876	6.42314	6.18154	6.17294	6.17559	6.18868	6.11726	6.0211	5.78379	6.06584	6.66704	6.51415	6.75331	6.71602
PNN	Q9H307	6.2633	6.2968	5.94252	6.6899	6.03855	5.92201	6.2698	5.96001	6.18101	6.00256	6.11336	6.00059	6.6205	6.32657	6.58453	6.78798
GDI1	P31150	6.29654	6.12195	6.88203	5.97434	6.15598	6.75152	6.23264	6.5587	6.41831	6.47483	6.69425	6.74502	5.79659	5.71193	5.53932	5.64602
FKBP10	Q96AY3	6.66913	6.51231	6.26413	6.66589	6.61958	6.0182	6.1126	6.00414	6.13744	6.02539	6.23664	6.1342	6.66212	5.96889	5.90083	6.06851
ATP5F1C	P36542	6.36593	6.44902	5.71219	6.62881	6.38714	6.00065	6.64892	5.93164	6.21569	5.91058	6.00542	6.00606	6.49308	6.08812	6.65908	6.49767
CLPX	O76031	6.51988	6.36017	5.95207	6.75523	6.42933	6.09812	6.29626	6.18523	6.29339	6.07368	6.07846	5.78857	6.43325	6.03503	6.37044	6.33088
INTS1	Q8N201	6.43415	6.48576	5.79376	6.82759	6.25592	6.22775	6.37697	6.34278	6.14752	6.15802	6.14703	6.21314	6.11567	6.19585	6.03104	6.24706
HNRNPUL2	Q1KMD3	6.18255	6.55426	5.8151	6.63669	6.09999	5.98812	6.38136	5.92593	5.93339	6.21227	5.82508	5.84792	6.70174	6.58543	6.62947	6.68069
PRPF6	O94906	6.26394	6.18555	6.0388	6.5916	6.16674	5.85415	6.51191	5.85906	6.31096	5.92498	6.19694	5.98091	6.55732	6.29964	6.47788	6.78572
WDR3	Q9UNX4	6.32923	6.30525	5.94327	6.7925	6.0226	5.91412	6.44906	5.88155	6.10612	5.85022	6.05295	5.94283	6.64546	6.40356	6.74647	6.61481
TBL3	Q12788	6.48753	6.39998	6.07218	6.91837	6.26163	6.14808	6.5989	6.23904	6.36958	5.83412	5.80653	5.83651	6.44016	6.01699	6.34566	6.22475
DIAPH1	O60610	6.22721	6.23269	6.65555	6.02394	6.28251	6.64478	6.25005	6.63014	6.45482	6.49666	6.51947	6.40458	5.8144	6.00258	5.5498	5.81082
MACROH2A1	O75367	6.24328	6.28387	5.67306	6.69383	6.15979	5.79609	6.36024	5.8276	6.2252	5.91676	5.83306	5.74058	6.64191	6.70348	7.05427	6.847
CHD1	O14646	6.15463	6.42774	6.05932	6.64297	6.34512	6.16962	6.33787	5.91668	6.12325	6.04251	6.10788	5.90242	6.37567	6.29063	6.53002	6.57365
ITPR1	Q14643	6.2693	6.30373	5.98709	6.7392	6.08342	5.95219	6.2308	5.87769	6.29714	6.08906	5.99418	5.94921	6.57312	6.48545	6.61155	6.60607
NOL6	Q9H6R4	6.23668	6.18208	6.03664	6.85301	6.12627	6.00606	6.19556	6.10058	6.23491	5.96652	6.0291	6.13223	6.60141	6.12218	6.59935	6.57743
SAMHD1	Q9Y3Z3	5.16298	5.44898	5.77879	5.09301	5.60909	6.14534	5.81969	5.75776	5.97633	6.07806	6.1293	5.81755	7.47536	7.82149	8.19862	7.68765
DYNC1LI1	Q9Y6G9	6.33571	6.06678	6.87997	5.99119	6.50599	6.70994	5.96508	6.68864	6.22062	6.33664	6.71009	6.73706	5.94634	5.80302	5.50829	5.59464
CDK5RAP2	Q96SN8	6.63753	6.76641	6.85747	6.75946	6.44445	6.36441	6.29817	6.4769	6.57971	6.7051	6.92636	6.64838	5.26684	4.98922	5.1307	5.1489
ALDH18A1	P54886	6.20781	6.36617	5.96275	6.60478	6.15258	5.94302	6.20727	6.14474	6.18702	5.99735	6.21684	6.27147	6.53698	6.24889	6.57219	6.37533
CAP1	Q01518	5.9937	5.95863	6.62472	5.70857	6.36403	6.74984	5.94543	6.27459	6.06489	6.24888	6.56523	6.40065	6.46889	6.38995	6.21888	6.02312
RANGAP1	P46060	6.39551	6.23362	6.32564	6.4358	6.33016	6.32261	6.41918	6.23447	6.56711	6.11849	6.33644	6.48397	6.02996	5.80465	5.89157	6.07083
ANAPC1	Q9H1A4	6.49617	6.10706	6.45467	6.3382	6.31839	6.20762	6.28024	6.18357	6.16966	6.22108	6.34786	6.31789	6.20429	6.11165	5.93964	6.30201
UBR1	Q8IWV7	5.85123	6.16549	6.54495	5.72156	6.0097	6.51509	6.08722	6.14612	6.1312	6.16181	6.24168	6.20056	6.37596	6.64387	6.67687	6.52671
RDX	P35241	6.13249	5.8726	6.79292	5.86308	6.39038	6.45048	6.33411	6.21206	6.75332	6.23702	6.8671	6.36106	6.21283	6.14106	5.88618	5.49331
PES1	O00541	6.2197	6.21381	5.78645	7.12166	6.0067	5.98069	6.39411	5.94714	6.32746	6.02908	5.88002	6.10945	6.34599	6.23998	6.4319	6.96585
MTA2	O94776	6.22785	6.06933	6.01756	6.3728	6.09756	5.9434	6.50547	5.77235	6.27901	6.09939	6.11493	5.98123	6.89871	6.47199	6.37162	6.77679
TXNRD1	Q16881	6.07632	6.06046	6.5845	5.71573	6.17766	6.8936	6.31455	6.49909	6.10385	6.24266	6.36311	6.23534	6.23118	6.33151	6.23525	5.93519
MOV10	Q9HCE1	6.36509	6.13493	6.4789	6.03803	6.15835	6.38716	6.37218	6.32783	6.40704	6.6126	6.57178	6.35069	5.85606	6.13271	5.99889	5.80774
NUP133	Q8WUM0	6.38476	6.18356	5.8101	6.47086	6.32177	5.76828	6.50045	5.93312	6.17436	5.87521	5.98999	6.02136	6.87625	6.38699	6.7249	6.57805
DARS2	Q6PI48	6.19903	6.17691	5.67583	6.43575	6.26913	6.02949	6.22123	6.03198	6.17043	5.97324	5.88392	5.73299	6.93459	6.55784	6.90983	6.7978
RSF1	Q96T23	6.50398	6.35464	5.99295	6.61934	6.3263	6.0303	6.31296	6.13853	6.23519	6.06017	6.20364	5.96876	6.45086	6.07844	6.38952	6.33444
DHX30	Q7L2E3-2	6.28335	6.34444	6.00904	6.68095	6.36843	6.22203	6.31422	6.23188	6.29443	6.24835	6.33552	6.27425	6.09255	5.9703	6.09782	6.23244
TSR1	Q2NL82	5.58406	5.73532	5.8257	5.83027	6.01021	6.20908	5.95251	6.08888	6.03658	5.94283	6.20959	6.18494	6.82644	7.18701	7.1728	7.20379
RPS19	P39019	6.53443	6.49539	6.49821	6.1495	6.32913	6.38974	6.30707	6.47272	6.51043	6.48279	6.68892	6.68603	5.58679	5.82872	5.46438	5.47574
KIF4A	O95239	5.59067	6.27447	6.05611	6.1116	5.85839	6.22985	6.15915	6.2246	6.02688	6.26498	6.10143	6.20734	6.56588	6.85692	6.74994	6.7218
SRP72	O76094	6.00101	6.22136	5.89706	5.95933	6.05516	6.13448	6.04555	5.97576	6.03862	6.10182	5.93307	5.8771	6.47499	7.33297	7.00703	6.9447
TRIM25	Q14258	6.29897	6.44438	6.43842	6.0807	6.45611	6.83332	6.11967	6.87781	6.34194	6.3934	6.45049	6.04303	5.6627	6.17969	5.8935	5.48589
SRP68	Q9UHB9	6.13158	6.22075	6.40928	6.06477	6.18997	6.39947	6.2687	5.94819	6.45364	6.0967	6.10667	6.14067	6.51735	6.3862	6.36748	6.29859
SLC4A7	Q9Y6M7-13	6.1364	6.44582	6.04376	6.55299	6.02832	6.11984	6.33721	6.13845	6.18937	6.12128	5.93573	6.19774	6.39928	6.36547	6.42526	6.56307
HNRNPH2	P55795	6.25635	6.21539	5.78063	6.48917	6.2894	6.1641	6.14318	6.13833	6.27876	6.06976	6.28635	6.40131	6.62688	6.16264	6.40147	6.29627
SPG11	Q96JI7	6.85976	6.81864	7.05841	6.64898	6.39637	6.38339	6.10211	6.34468	6.69362	6.85985	6.98581	6.80323	4.87276	5.09381	4.97718	5.10139
KIF2C	Q99661	5.87501	6.28488	6.6453	6.22966	5.93928	6.46225	6.37946	6.08383	6.43588	6.62116	6.67399	6.61017	5.85592	5.81431	5.91469	6.17421
EPS15L1	Q9UBC2-2	6.17979	5.78962	6.20314	5.26326	6.61098	6.13109	6.10035	6.50289	6.33783	6.42378	6.62533	6.49563	6.76494	6.22482	6.29202	6.05454
TOPBP1	Q92547	6.20413	6.7935	6.32083	6.92284	6.15228	6.15866	6.2456	5.99065	6.19998	6.3694	6.19695	6.29965	5.97574	5.72794	6.25851	6.17577
RBBP6	Q7Z6E9	7.43746	6.04179	5.95277	6.08518	5.39212	5.33874	8.32682	6.95082	6.54313	5.63657	5.36906	5.39974	5.6776	7.03195	6.23296	6.58329
RPS9	P46781	6.3978	6.49125	6.0978	6.0472	6.30099	6.24296	6.24514	6.66663	6.84084	6.33923	5.92853	6.11058	6.17169	6.31712	5.68712	6.11514
CSTF3	Q12996	6.27516	6.26956	5.86011	6.55805	6.28782	6.07206	6.73525	5.93472	6.10069	6.0522	5.98467	5.9647	6.69911	6.17096	6.70272	6.33221
BRD4	O60885	6.32251	6.49595	6.16932	6.79971	6.20481	6.1059	6.18659	5.93288	6.14818	6.26853	6.18781	6.27982	6.17352	6.06038	6.25622	6.40487
ITPRID2	P28290	6.44573	6.50903	6.00997	6.48327	6.11617	6.27673	6.17975	6.16367	6.2188	6.43438	6.39304	6.29799	6.20371	6.06659	6.03134	6.16985
DIP2B	Q9P265	5.97858	6.11373	6.38761	6.03021	6.00671	6.37264	5.95086	6.37965	6.27692	6.38558	6.60801	6.57282	6.28395	6.32627	6.13539	6.19107
TPM3	P06753	6.33783	6.07204	6.52819	6.38734	6.71053	6.36182	6.63036	6.5314	6.63277	6.50253	6.1958	6.29662	6.10465	6.21162	5.37742	6.11908
TPM1	P09493-3	6.71613	5.97099	7.04739	5.98807	6.52884	6.1023	5.95653	6.81581	6.46693	6.04433	6.71911	6.54578	6.49965	5.54035	5.42437	5.63343
PRPF3	O43395	6.24465	6.55061	6.03169	6.61742	6.14063	6.13396	6.36995	6.13575	6.02875	6.00123	5.87129	5.90252	6.41344	6.50033	6.4327	6.62508
DPYSL2	A0A1C7CYX9	5.9696	5.99596	6.51797	5.80489	6.31083	6.70084	6.42944	6.73637	6.37566	6.43823	6.45811	6.43564	5.78061	6.29928	5.68945	6.05713
DPYSL3	Q14195-2	6.30149	6.1951	6.70012	5.94801	6.38892	6.7369	6.14698	6.67764	6.42697	6.54534	6.69321	6.37123	5.76928	5.83556	5.58776	5.67548
LIMA1	Q9UHB6-4	6.39994	6.57467	5.96559	6.41909	6.4374	6.22903	6.10188	6.25258	6.20348	5.89309	6.00504	5.98016	6.60935	6.17576	6.43354	6.3194
RBM28	Q9NW13	6.40917	6.21225	6.00568	6.55064	6.15581	5.66716	6.39982	5.99218	6.26156	6.02465	5.9898	5.96083	6.55248	6.38486	6.64791	6.78519
WDHD1	O75717	5.91695	6.1146	6.14366	5.99341	5.8217	6.05922	6.10264	6.00424	6.09045	6.10554	6.35592	6.29172	6.66073	6.65511	6.72616	6.95795
CDC5L	Q99459	6.51389	6.57973	5.91322	6.67152	6.12166	6.15074	6.51691	5.85932	6.04443	6.05167	5.7969	5.97505	6.31194	6.48481	6.25427	6.75395
ERC1	X6RLX0	6.27037	6.39485	6.59018	6.05664	6.29606	6.59994	5.89448	6.53829	6.35975	6.36853	6.5001	6.36387	6.27594	5.96769	5.78357	5.73976
HARS1	P12081	6.43119	6.16561	6.82991	6.00169	6.23236	6.85959	6.26151	6.67421	6.31754	6.61919	6.70217	6.54641	5.71114	5.70649	5.36076	5.58021
SUPT16H	Q9Y5B9	5.97523	6.14444	5.9016	6.10716	6.16178	6.09361	6.19045	5.84289	6.20837	6.30158	6.01341	5.85694	6.78308	6.59396	7.00795	6.81754
TALDO1	P37837	5.98657	6.0018	6.25421	5.22291	6.05095	6.49431	5.77632	6.53148	5.81079	6.14303	5.97604	6.04458	6.33902	7.60303	7.09441	6.67055
NCLN	Q969V3-2	6.48772	6.72558	5.95934	7.00241	6.24281	6.01911	6.40674	6.15498	6.08449	5.88675	6.084	5.90135	6.24207	6.1433	6.40742	6.25193
LAMB1	P07942	6.44935	6.20096	6.39567	6.6965	6.23163	6.2402	6.37089	5.96396	6.26063	6.05732	6.40619	6.32695	6.22309	5.84451	6.04758	6.28458
USP36	Q9P275	6.21823	6.2182	5.67362	6.97737	6.0704	5.80704	6.35001	5.79099	5.93924	5.9361	5.94813	5.86581	6.22625	6.77441	7.08283	7.12136
STRAP	Q9Y3F4-2	6.24579	6.21396	6.74284	6.02043	6.65439	6.65922	6.11143	6.82017	6.40266	6.45216	6.69857	6.57464	5.54092	5.71575	5.44688	5.70017
FYCO1	Q9BQS8-4	6.64472	6.60275	6.23837	6.53153	6.11021	6.03148	5.98881	6.09373	6.6211	6.54228	6.57463	6.5353	6.09936	5.95936	5.69756	5.72881
NUP85	Q9BW27	6.37852	6.18179	6.06861	6.56074	6.15035	6.08746	6.55936	6.18425	6.20928	6.01862	6.13796	6.18427	6.33554	6.27809	6.25937	6.40577
NCOR1	O75376	6.27796	6.32426	6.30653	6.60382	6.12922	6.16586	6.27301	6.21157	6.30576	6.19787	6.05356	6.19257	6.49892	6.09168	6.15437	6.21305
EIF2S1	P05198	6.37167	6.42402	6.52755	6.10664	6.31504	6.47361	6.39882	6.59712	6.29592	6.37515	6.3462	6.30957	5.70066	6.0902	5.81795	5.84985
ANKHD1	Q8IWZ3-6	6.27927	6.25691	6.6741	6.04579	6.2036	6.38663	6.25138	6.40185	6.69217	6.55689	6.65306	6.65521	5.7499	5.81283	5.69728	5.68124
SSRP1	Q08945	5.78688	6.13629	5.78824	6.41024	5.96128	6.0725	6.24537	5.74983	6.09776	6.11458	6.08443	6.13695	6.69437	6.70885	7.01378	6.99866
BCLAF1	Q9NYF8	6.34065	6.38456	5.74376	6.80095	6.27779	5.98148	6.28254	5.90892	6.15249	6.07802	6.1149	6.13352	6.60391	6.33041	6.47028	6.39579
ATP13A1	Q9HD20	6.23454	6.4929	6.03154	6.6601	6.14109	5.86883	6.32891	5.91847	6.04056	6.07585	6.04641	5.96773	6.60121	6.52284	6.55941	6.50962

GSTP1	P09211	5.97854	5.9226	6.92443	5.41878	6.38407	6.75302	5.97813	6.75638	6.20447	6.54783	6.64399	6.70824	6.07136	6.02737	5.93069	5.75011
PLD3	Q8IV08	6.74645	6.88356	6.20312	6.53952	6.29481	6.34977	6.36837	6.20082	6.25923	6.42335	6.25976	6.46048	5.77451	5.84589	5.71555	5.67481
KIF21A	Q7Z4S6-4	5.84038	5.98013	6.62355	5.75804	5.88662	6.36336	5.95695	6.20457	6.25317	6.29733	6.67001	6.86672	6.30274	6.58694	6.11047	6.29903
DKC1	O60832	6.01871	6.079	5.57839	6.3567	6.05042	5.64463	6.33486	5.88496	6.08515	5.97258	5.73861	5.73431	6.6716	7.16648	7.31454	7.36907
RAD23B	P54727	6.54188	6.10528	6.31479	5.25756	6.44871	6.26328	6.00568	6.67934	6.15881	6.17794	6.24973	6.55788	6.54963	6.36205	6.0427	6.28475
MFN2	O95140	6.88053	6.93975	6.54027	7.22883	6.45385	6.09268	6.63805	6.0317	6.68358	6.41926	6.43969	6.50771	5.44034	5.19408	5.20505	5.30464
ALMS1	Q8TCU4	6.69665	6.44155	6.91418	6.46797	6.3197	6.48761	6.23535	6.29763	6.58773	6.30062	6.78376	6.77313	5.45711	5.49709	5.32485	5.41507
GAK	O14976	6.13771	6.13735	6.64959	5.96521	6.01616	6.36816	6.0618	6.16244	6.32357	6.48124	6.5218	6.61227	6.07014	6.31734	6.01411	6.16112
NUP107	P57740	6.50209	6.34901	6.08132	6.63025	6.30318	6.07456	6.43507	6.16105	6.27151	6.00089	5.88887	6.10777	6.4831	5.9934	6.50629	6.21165
RPL10	P27635	6.39082	6.06348	6.56954	6.38768	6.26932	6.30656	6.54847	6.01167	6.90601	6.28359	6.59982	6.19889	5.81596	5.93403	5.77496	5.93918
WASHC5	Q12768	6.05663	6.2411	6.25299	6.03676	6.23982	6.28368	6.12113	6.45345	6.21739	6.42083	6.32765	6.3284	6.16082	6.52932	6.16896	6.16107
SUCLG2	Q96I99	6.32382	6.37405	5.68701	6.11232	6.61257	6.14515	6.02066	6.30745	6.07728	6.03507	5.96872	6.16173	7.01428	6.43359	6.32331	6.40298
TCERG1	O14776	6.12102	6.39279	5.85027	6.32532	6.33679	6.14307	6.37015	6.20518	6.09339	6.02711	5.85535	5.76639	6.8956	6.58937	6.51817	6.51003
PSMD13	Q9UNM6	6.15932	5.50409	6.18603	5.54261	6.34216	6.39214	6.41872	6.19078	5.91333	6.04542	6.01611	6.42078	6.31534	6.79255	6.71507	7.04555
SCFD1	Q8WVM8	6.4638	6.32299	6.0443	6.32885	6.21812	6.21428	6.52371	5.80903	6.05373	5.92225	6.04755	5.91754	6.59047	6.30042	6.7256	6.51735
SEC16A	O15027-5	6.18815	6.05393	6.94444	6.1147	6.04187	6.43607	6.02316	6.22769	6.26347	6.55705	6.70455	6.63796	6.17708	6.14514	5.74963	5.7351
SH3KBP1	Q96B97	6.14922	6.09177	6.68785	5.76079	6.22449	6.53804	6.07122	6.74268	6.56863	6.46462	6.49007	6.45664	6.02214	6.11178	5.78385	5.83621
SEC63	Q9UGP8	6.21096	6.39222	5.85207	6.35384	6.40748	6.03547	6.47849	6.2704	5.9695	5.98317	5.70061	5.56963	6.76495	6.63048	6.87664	6.5041
PTBP1	P26599-3	6.4833	6.77532	6.2631	6.87712	6.84179	6.43329	6.41415	6.46302	6.3426	5.74025	5.59589	5.65148	6.24098	5.9793	5.96086	5.93755
CDK11B	P21127	5.9912	6.39259	6.06571	6.49317	6.09831	6.00505	6.09042	6.0211	5.99779	6.01757	6.25049	6.1167	6.4596	6.51353	6.64538	6.84138
RPL8	P62917	6.0174	6.4589	6.35579	5.96213	5.9796	7.09588	6.23276	6.53594	7.50751	6.19463	6.10703	5.89693	6.00175	6.19306	5.63632	5.82438
API5	Q9BZZ5	6.00674	6.01884	5.83962	5.8864	5.99741	5.92084	6.08248	5.87493	6.11699	6.01648	6.01569	5.92317	7.13426	7.03872	7.22688	6.90052
NF1	P21359	6.26064	6.27183	6.31936	6.33486	6.02923	6.40269	6.27244	6.2492	6.32911	6.28856	6.13291	6.26167	6.27624	6.19643	6.29059	6.08426
ACAD9	Q9H845	6.35103	6.41515	6.10492	6.78484	6.4474	5.95986	6.39413	5.93626	6.18089	6.0315	5.92705	5.91397	6.48311	6.13584	6.54583	6.38821
CFL2	Q9Y281	5.9822	6.00355	6.44819	5.95783	6.30353	6.71426	6.09458	6.51899	6.18302	6.46517	6.62589	6.58393	6.1745	6.21163	5.82148	5.91123
PLOD3	O60568	6.32918	6.29783	6.11305	6.56796	6.20487	6.03067	6.31608	6.00774	6.0744	6.03484	6.24029	6.14984	6.38319	6.18241	6.72941	6.33826
ZMYM3	Q14202-2	6.23173	6.5109	6.35598	6.80822	6.17985	6.14111	6.5281	5.8015	6.32339	6.4275	6.44691	6.06067	6.08166	5.98491	6.14285	5.97473
RABGAP1	Q9Y3P9	6.09582	6.19522	6.81959	6.1929	6.29577	6.55199	6.21367	6.21973	6.26246	6.50962	6.61228	6.35998	6.14463	6.05446	5.72437	5.7475
USP24	Q9UPU5	6.23837	6.23254	6.79787	6.11918	6.11509	6.53885	6.16428	6.46432	6.26359	6.39109	6.54268	6.5715	5.96912	5.80608	5.91949	5.86595
INTS3	Q68E01	6.3766	6.3685	6.23047	6.42531	6.26473	6.06096	6.30102	6.04197	6.1327	6.02643	6.06302	5.91872	6.44227	6.37399	6.52378	6.44953
SRSF1	Q07955-2	6.5718	6.11189	5.98868	6.47007	6.64345	5.82889	6.17992	5.94564	6.40523	5.81948	6.21752	5.83612	7.23794	6.05569	6.31533	6.37236
PPP1R12A	O14974	6.41011	6.37874	6.57108	6.12509	6.36173	6.67495	6.16438	6.55492	6.44646	6.29858	6.45965	6.33351	5.93574	5.98947	5.6713	5.6243
NOP2	P46087-4	6.05643	6.14555	5.86059	6.63741	5.85636	5.77223	6.42811	5.83615	6.10209	5.75916	5.84952	5.9198	6.39079	6.9646	7.17023	7.25098
NUDC	Q9Y266	6.52882	6.1645	6.93844	5.84493	6.48342	6.70877	6.27494	6.53738	6.69463	6.3425	6.47838	6.45158	5.76242	5.55882	5.63737	5.59311
TTF2	Q9UNY4	6.49962	6.27711	6.53237	6.54722	6.14154	6.27584	6.50368	6.48474	6.66772	6.3535	6.60703	6.66577	5.87045	5.43618	5.64528	5.49195
HSD17B10	Q99714	6.34001	6.10718	5.71175	6.51574	6.41629	5.91171	6.40845	5.96752	6.36824	6.15412	6.24991	6.15684	6.69798	6.03459	6.67366	6.28602
NCOA2	Q15596	5.75985	5.98422	6.09386	5.68342	6.20173	6.28354	6.2933	6.13206	5.99239	6.0325	6.14629	5.72798	7.03766	6.96218	6.99492	6.6741
FH	P07954	6.38989	6.19663	5.91512	6.10634	6.46808	6.0782	6.36581	6.06769	6.43539	5.93822	6.16671	5.9272	7.33099	6.04681	6.48536	6.08156
TOMM70	O94826	6.39132	6.49596	5.72746	6.6262	6.39504	6.20754	6.47489	6.17793	6.05272	6.03702	5.58655	5.56541	6.67266	6.54442	6.46514	6.57973
CTNND1	O60716-3	6.28833	6.32423	6.05176	6.39182	6.16627	6.2269	6.36434	5.97328	6.31798	6.2698	6.29928	6.2393	6.45223	6.35926	6.17178	6.10342
BTAF1	O14981	5.38486	5.69609	5.90191	5.26467	5.79333	6.11268	5.9305	5.80747	5.88847	6.31602	6.15886	5.96427	7.4897	7.45324	7.56268	7.27525
SIN3A	Q96ST3	6.44601	6.79182	5.91787	6.85462	6.3453	6.21649	6.24623	6.22352	5.94708	6.00489	5.93689	5.97572	6.1323	6.28225	6.37158	6.30744
NCKAP1	Q9Y2A7-2	6.20051	6.09973	6.5491	5.90401	6.07996	6.50216	6.33749	6.31594	6.53976	6.47415	6.48964	6.51841	5.96216	6.14446	6.04119	5.84134
SMC6	Q96SB8	6.38997	6.29974	6.12562	6.42293	6.15253	5.86907	6.20029	5.92079	6.18349	5.9267	6.07643	6.14095	6.71512	6.20956	6.85788	6.50894
FARSB	Q9NSD9	6.21763	6.19361	6.51294	5.85369	6.53125	6.58235	6.03794	6.5552	6.47199	6.47975	6.47859	6.52725	6.0186	5.99124	5.68627	5.86172
CDK1	P06493	6.32859	6.17057	6.55445	5.97942	6.39692	6.882	6.37682	6.97668	6.26844	6.55315	6.59545	6.60969	5.68983	5.65603	5.27646	5.68551
ATXN2	A0A2R8Y5A6	6.29767	6.13678	6.67749	5.77416	6.56376	6.37313	6.19459	6.36519	6.28253	6.43245	6.37256	6.4588	6.41006	5.9758	5.93614	5.74889
NIBAN2	Q96TA1	6.65361	6.05613	6.9176	5.64811	6.49068	6.60972	6.25327	6.50828	6.5301	6.14648	6.43858	6.25187	6.14334	6.07987	5.77906	5.4933
RARS2	Q5T160	6.48507	6.44344	5.71822	6.61389	6.39295	6.06171	6.50926	6.06477	6.2413	5.95135	5.91013	5.99016	6.63124	6.15483	6.45456	6.37711
CHAMP1	Q96JM3	6.35053	6.2999	5.91933	6.40304	6.19974	5.97452	6.14602	6.01261	6.11439	6.25652	6.0375	6.08092	6.95796	6.25505	6.51026	6.48172
CHD8	Q9HCK8	6.0466	6.35004	6.06054	6.53806	5.98984	6.20899	6.30322	6.08025	6.06107	6.24703	6.54955	6.23639	6.10754	6.46144	6.47086	6.28858
ATP1A3	P13637-3	6.47519	6.60243	6.42049	6.76622	6.07079	6.10948	6.32716	5.92921	6.49701	6.34921	6.52505	6.31312	5.90627	5.84602	5.83256	6.02977
ATP1A1	P05023-4	6.66194	6.6966	6.172	6.74989	6.39872	6.25419	6.37651	6.06177	6.24647	6.28524	5.98929	5.96436	5.96845	6.13223	6.03113	6.0112
UBA6	A0AVT1	6.1181	6.02313	6.84314	5.91114	6.30497	6.58555	6.1529	6.47315	6.20477	6.43098	6.64368	6.62723	6.01578	6.09465	5.85751	5.71331
DNAAF5	Q86Y56	6.01381	6.10716	6.66325	5.80277	6.36536	6.67366	6.30107	6.72169	6.41348	6.3473	6.68397	6.50321	5.87882	5.85639	5.77402	5.89404
SCP2	P22307	5.93063	5.85319	5.68882	6.09741	6.65287	5.88594	6.42597	6.30209	6.11299	5.88047	5.98479	6.07736	6.72308	6.4934	6.74445	7.14655
ARHGEF1	Q92888-3	6.41266	6.4483	6.60431	6.3144	6.27333	6.7397	6.27164	6.70083	6.37724	6.41659	6.47211	6.51792	5.73728	5.79549	5.42303	5.49517
EHD1	Q9H4M9	5.78976	5.96385	6.03469	5.79129	6.30738	6.18222	6.52908	6.29883	6.72903	5.79388	6.1968	6.3265	6.22487	6.68766	6.42471	6.71945
EML4	Q9HC35	6.26034	6.09007	6.7625	5.76424	6.11634	6.32665	6.14502	6.25498	6.15796	6.51098	6.44269	6.33162	6.1819	6.39885	6.17864	6.07721
NUP50	Q9UKX7	6.39157	6.08578	6.22767	6.86375	6.35496	6.07495	6.12478	6.12319	6.20746	6.16206	6.25597	6.29833	6.82014	5.93315	5.87114	6.20512
ATAD3B	Q5T9A4	6.40878	6.26952	6.11256	6.7432	6.29162	6.0894	6.5947	5.84797	6.01844	5.9899	6.05128	5.99193	6.39075	6.27647	6.42763	6.49586
ZCCHC8	Q6NZY4	6.43177	6.16295	5.86844	6.55135	6.60078	6.37543	6.08045	6.14899	6.3941	5.96564	6.35263	6.24352	6.81477	5.87056	6.02251	6.1161
ESF1	Q9H501	6.05268	6.02503	6.05159	6.57564	6.03251	5.7613	6.45842	5.91303	6.26177	5.78086	5.95436	5.84794	6.84069	6.57011	6.92394	6.95162
FHOD1	Q9Y613	6.16775	6.16236	6.47099	6.03003	6.19469	6.76157	6.12308	6.33836	6.29535	6.57465	6.44975	6.28254	5.98251	6.32773	5.94455	5.89408
SQOR	Q9Y6N5	6.1291	6.39416	5.72927	6.69555	5.85666	6.17886	6.15997	6.01002	5.82595	6.33301	5.7692	6.10856	6.68908	6.70215	6.54878	6.86967
SMARCA5	O60264	6.52552	6.49522	6.07189	6.83719	6.26751	6.03608	6.38827	5.93974	6.09547	6.05291	5.89775	5.9937	6.51795	6.20988	6.49037	6.18056
BZW1	Q7L1Q6-3	6.41443	6.44307	6.51775	6.00585	6.4739	6.64482	6.54393	6.46877	6.47266	6.27847	6.2127	6.2242	5.60199	5.98349	5.75694	5.95703
EIF4A1	P60842	6.76038	6.73231	6.93408	6.42775	6.50927	6.71433	6.72766	6.72323	6.89976	6.12968	6.38996	6.36694	5.04739	5.34968	5.00096	5.28663
LMO7	Q8WWI1	6.41539	6.39926	6.07612	6.61575	6.13489	5.89853	6.45528	6.00081	6.14588	6.17092	6.09568	6.07747	6.48881	6.29554	6.49147	6.23819
DNM2	P50570	6.28415	6.15897	6.69785	5.89451	6.23696	6.44434	6.15988	6.40903	6.3806	6.37991	6.33102	6.32167	6.21922	6.16485	6.08426	5.83278
NFKB2	Q00653	6.66934	6.36056	6.88824	6.25876	6.58459	6.85298	6.42365	6.73574	6.2622	6.07877	6.01877	6.10225	5.80608	5.75542	5.54108	6.66157
PTCD3	Q96EY7	6.40246	6.25669	5.88119	6.81525	6.33826	6.12335	6.41606	6.17305	6.00943	6.01744	5.99733	6.01428	6.42388	6.21798	6.43431	6.47904
MTA1	Q13330	6.34943	6.35726	5.96582	6.49893	6.31671	5.81779	6.5182	5.90374	6.37739	6.07741	6.18791	5.74536	6.55846	6.28393	6.43592	6.60574
VWA8	A3KMH1	6.45663	6.47811	5.87284	6.65747	6.28948	6.14466	6.27498	5.97689	6.18601	5.93551	5.86434	5.91423	6.49907	6.38603	6.49875	6.55599
MED14	O60244	6.3322	6.33716	6.03637	6.80133	6.43871	6.23842	6.28927	6.11778	6.12633	6.03604	5.92666	5.951	6.44025	6.20504	6.43182	6.29162
PAFAH1B1	P43034	6.32486	6.2345	6.84394	5.9767	6.3737	6.74079	6.3676	6.39774	6.33163	6.46756	6.58326	6.3491	5.88966	5.61745	5.95636	5.54514
RPAP3	Q9H6T3	6.11808	6.34446	6.76733	6.09782	6.23971	6.63554	6.1117	6.5061	6.18344	6.49683	6.44162	6.48639	5.85866	5.99927	5.81211	5.90094
PUS7	Q96PZ0-2	5.39699	5.79761	6.21931	5.31009	5.81608	6.38376	5.53182	6.14047	5.82244	6.14938	6.30288	6.29245	6.77746	7.52945	7.29115	7.23867
WDR33	Q9C0J8	6.35591	6.40814	6.14176	6.89001	6.13612	5.98693	6.47508	6.0756	6.01634	6.04801	6.02345	6.18394	6.31112	6.18005	6.41338	6.35418
UAP1	Q16222	6.29281	6.033	7.1035	6.20464	6.39611	6.61953	6.25407	6.82268	6.48447	6.06647	6.68351	6.99395	5.39571	5.31003	5.74462	5.59493
SUGP2	Q8IX01	6.29593	6.51188	5.86247	6.91346	6.00033	5.9913	6.44403	5.94433	6.09495	6.14368	5.87744	5.98142	6.34378	6.51412	6.54197	6.53892
HSDL2	Q6YN16	6.27864	6.3771	5.95118	6.67376	5.91867	5.7314	6.1073	6.11487	6.17136	6.22972	6.04653	6.1532	6.63925	6.42425	6.58085	6.60191
DYNC1I2	Q13409-2	6.19199	6.00637	6.73145	6.30009	6.2324	6.47416	6.24981	6.41853	6.26851	6.42187	6.48939	6.71465	5.72598	6.22788	5.5681	5.97881
ELP1	O95163	6.30954	6.09903	6.7144	6.13979	6.38405	6.64583	6.30678	6.49234	6.50921	6.56515	6.68197	6.55362	5.68608	5.65985	5.67474	5.57761
ZMYND8	Q9ULU4-7	6.34518	6.4612	6.04774	6.80367	6.17325	6.1828	6.14333	5.99884	5.96793	6.13039	6.14871	6.20684	6.45766	6.2693	6.19484	6.46834
UBR2	Q8IWV8	6.14424	6.40752	6.80111	6.28504	6.21865	6.71798	6.06044	6.48398	6.30929	6.44878	6.44384	6.4481	5.76412	6.05709	5.71141	5.69842
EIF3H	O15372	6.22849	6.50172	6.88594	6.19323	6.37251	6.54636	6.36659	6.55128	6.55153	6.48077	6.71643	6.69815	5.35177	5.60365	5.52223	5.42934
HNRNPH1	G8JLB6	6.26018	6.33396	5.66237	6.81082	6.26469	6.50086	5.98109	6.5261	6.16646	6.1142	6.16304	6.2791	6.32114	6.1878	5.99277	6.43542
RBM12B	Q8IXT5	6.29451	6.52311	5.83263	6.71593	6.24598	6.08183	6.26772	6.06792	6.14321	6.0849	6.01152	6.02213	6.55286	6.23884	6.52875	6.38817
BAIAP2L1	Q9UHR4	6.16346	6.28967	6.15882	5.86115	6.18361	6.41468	6.15568	6.295	6.74035	6.42493	6.32003	6.68222	6.12795	6.21387	5.93439	6.03422
ATP5PO	P48047	6.28732	6.27474	5.88204	6.66581	6.30675	5.9225	6.62629	6.14053	6.35092	5.73767	5.79856	5.99332	6.68922	6.17656	6.64096	6.50681
DHX57	Q6P158	6.19439	6.33587	6.69976	6.12063	6.08911	6.589	6.05982	6.25199	6.60681	6.50428	6.66099	6.43696	6.54478	6.01789	5.95494	5.83277
NXF1	Q9UBU9	6.17471	6.25452	6.00086	6.61514	6.01663	5.76689	6.53366	5.81421	6.24977	6.11294	5.89976	6.0382	6.64783	6.29822	6.92085	6.6558
PTK2	Q05397	6.11802	6.06769	6.75243	5.99268	6.19737	6.40899	6.35056	6.34193	6.44662	6.44517	6.76023	6.39048	5.96345	6.08429	5.97009	5.70999
TRIP12	Q14669-3	6.29685	6.46032	6.09261	6.87255	6.11872	6.00969	6.30478	6.16475	6.29497	6.05033	5.9693	6.0265	5.87919	6.55995	6.3108	6.58869
SCAF11	Q99590	6.23527	6.38777	5.92921	6.81607	6.034	5.80751	6.23431	5.95751	6.24643	6.29373	6.1186	6.20701	6.36072	6.39122	6.51843	6.46219
RPL23A	P62750	5.86706	6.43827	6.3222	6.27925	6.19878	6.87304	6.06	6.46944	6.60212	6.17324	6.05207	6.24923	5.67761	6.59286	6.02969	6.11516
UGDH	O60701	5.45793	5.75675	6.144	5.2298	5.88739	6.56484	5.83031	6.07689	5.79633	6.23088	6.21565	6.15623	7.15121	7.10481	7.33076	7.0662
PPP2R1A	P30153	6.183	6.02307	6.35399	5.60757	6.33222	6.4372	6.01684	6.6176	6.16793	6.2442	6.48766	6.54475	6.30058	6.34925	6.03303	6.3011
BOP1	Q14137	6.34179	6.52073	6.05002	6.9339	6.22963	6.0245	6.33089	5.98738	6.04833	6.19781	6.08373	6.25202	6.21237	6.10322	6.31014	6.37353
EIF3C	Q99613	6.40314	6.61181	6.62287	6.33896	6.38098	6.65167	6.33664	6.39106	6.40336	6.47201	6.53341	6.42978	5.42654	5.73193	5.6353	5.63054
ZNF462	Q96JM2-3	6.40577	6.743	6.18357	6.87669	6.12453	5.9799	6.32451	6.04897	6.19423	6.18293	6.33824	6.1035	6.16759	5.87247	6.16989	6.28419
CREBBP	Q92793	6.08134	6.3258	6.18105	6.29845	6.21981	6.28588	6.06905	6.14055	6.16712	6.31013	6.32449	6.03911	6.48874	6.34423	6.4014	6.32286
SF3A3	Q12874	6.46276	6.36621	5.80771	6.63286	6.47939	6.36393	5.98987	6.2101	6.13803	6.04881	6.18654	6.16957	6.79537	6.09363	6.11553	6.13968
GTF3C4	Q9UKN8	6.10649	6.34154	6.15206	6.25762	6.23279	6.29262	6.01604	5.95972	6.08602	6.29852	6.10711	6.1317	6.66119	6.1455	6.87998	6.3311

GEMIN5	Q8TEQ6	6.3316	6.70144	6.74477	6.18246	6.33736	6.49698	6.25781	6.66006	6.39824	6.35422	6.46982	6.41467	5.66412	5.66612	5.79221	5.52813
IDH1	O75874	6.28751	6.18752	6.68533	5.54142	6.3782	6.75086	6.11417	6.52823	6.2946	6.46913	6.72435	6.26295	6.16266	6.03733	5.91848	5.65725
PSMD7	P51665	6.37366	6.32812	6.43144	6.25337	6.34807	6.5343	6.03844	6.47164	6.15659	6.18569	6.45554	6.37016	6.11742	6.01558	5.89298	6.02699
WDR43	Q15061	6.348	6.29845	5.78049	6.82905	6.40199	6.16338	6.37499	5.97247	6.14156	5.96732	5.8785	6.04711	6.8085	6.12405	6.30673	6.5574
EIF3L	Q9Y262	6.32213	6.46892	6.64089	6.23315	6.39851	6.55502	6.30021	6.48066	6.46246	6.38122	6.44525	6.4112	5.6429	5.96165	5.67529	5.62055
NDUFA9	Q16795	5.97639	6.10501	5.23876	6.292	6.39657	6.30616	6.79261	6.5772	6.25595	5.82467	5.75929	5.74552	6.98121	6.57188	6.5007	6.67609
RTCB	Q9Y3I0	5.65335	5.84603	5.92317	5.64758	6.26639	6.22132	6.45329	6.10934	6.26012	5.98253	6.28266	5.97296	6.53647	6.97075	6.79713	7.07691
USP14	P54578	6.02562	6.1078	6.49049	6.03781	6.20903	6.47127	6.36623	6.52743	6.36895	6.07761	6.52923	6.56843	5.80033	6.29474	5.80273	6.3223
PDHA1	P08559-4	6.35976	6.35826	5.87596	6.6828	6.16371	5.89204	6.55541	5.88389	6.25296	5.8408	5.78794	5.91723	6.85974	6.22683	6.70868	6.634
XAB2	Q9HCS7	6.31985	6.33301	6.08258	6.84417	6.41828	6.15474	6.37868	5.99326	6.19544	6.19491	6.23864	6.20743	6.23036	5.89706	6.31061	6.20097
RAI1	Q7Z5J4	6.55075	6.2732	6.03991	6.82984	6.06229	6.14319	6.34423	5.78196	6.12724	6.10725	6.27098	6.00577	6.1879	6.37823	6.47726	6.42
DDX42	Q86XP3	6.18665	6.33933	6.073	6.31145	6.27834	5.97875	6.36013	5.89722	6.22019	6.07185	6.06407	5.73442	6.82445	6.3218	6.75928	6.57907
RNF20	Q5VTR2	5.99748	6.52017	6.20524	6.11921	6.47893	6.38091	6.44931	6.1701	6.29174	6.56793	6.1893	5.88847	6.33775	6.17001	6.14727	6.08616
RAB11B	Q15907	6.46083	6.33136	6.3833	6.24558	6.26549	6.30582	6.1752	5.99244	6.34327	6.29058	6.30687	6.38292	5.96166	5.87449	6.36956	6.31064
SMU1	Q2TAY7	6.2504	6.28487	5.79933	6.40635	6.39884	5.96758	6.43553	5.95043	6.02737	6.01459	6.00696	5.7814	6.66789	6.44171	6.88851	6.67825
ATXN2L	Q8WWM7-3	6.34089	6.39644	6.56466	6.25395	6.47861	6.63973	5.86425	6.44327	6.46632	6.60445	6.49023	6.70823	6.0706	5.77568	5.37141	5.66229
ACAA2	P42765	6.2869	6.26246	5.68579	6.41299	6.49308	6.27296	6.13576	6.29524	6.16285	5.93504	5.93631	6.10297	6.89411	6.19798	6.51201	6.41354
AIFM1	O95831	6.27945	6.29505	5.71893	6.51642	6.20966	6.03161	6.53771	6.05052	6.17379	5.97578	5.95022	5.9151	6.66392	6.6149	6.53167	6.53527
DNTTIP2	Q5QJE6	6.33401	6.37995	5.91477	6.81776	6.22367	5.91124	6.45668	6.17439	6.03265	6.03743	5.78927	5.97735	6.59421	6.1828	6.53778	6.63604
BLM	P54132	6.29264	6.13728	5.85429	6.5075	6.07225	6.02906	6.37012	5.96471	6.13147	6.01334	5.80923	5.84256	6.52968	6.63105	6.97775	6.8371
PPA2	Q9H2U2	6.43653	6.10206	5.85767	6.06814	6.54012	5.93747	6.13291	6.32437	6.02147	6.11193	5.97426	6.01562	7.04142	6.52586	6.6173	6.29286
PSPC1	Q8WXF1	6.18977	5.92587	5.80342	6.17261	6.40688	5.87952	6.16567	6.28369	6.19545	5.9208	6.22305	6.02545	7.23614	6.39538	6.52255	6.65377
AGPS	O00116	6.25827	6.38722	6.19197	6.73618	6.19539	6.09028	6.41005	6.08846	6.14719	6.01574	6.06133	5.97855	6.244	6.2113	6.47836	6.50572
ITSN2	Q9NZM3	6.19089	6.21746	6.02515	5.92123	6.29165	6.23612	6.29649	6.14151	6.46619	6.26103	6.22648	6.24521	6.51504	6.32986	6.45465	6.18103
ACSF2	Q96CM8-2	6.29748	6.19192	5.90652	6.28464	6.20345	6.01951	6.23104	5.94994	6.07939	5.97388	6.04326	5.82997	7.06812	6.60545	6.82577	6.48965
EIF4B	P23588	6.53469	6.58913	6.7433	6.25603	6.23917	6.53733	6.306	6.26452	6.65195	6.60406	6.77953	6.77028	5.52137	5.56843	5.21996	5.41421
GOLGA3	Q08378	6.33994	6.35109	6.43604	6.47565	6.31395	6.2767	6.14036	6.69707	6.55173	6.39184	6.51568	6.32678	6.11504	5.76322	5.64492	5.66628
FBL	P22087	6.40702	6.20158	5.90569	6.4562	5.92141	5.80731	6.4315	5.79687	6.27769	6.10466	6.22614	6.18847	6.68175	6.26997	6.56571	6.75803
DDX41	Q9UJV9	5.96586	6.19592	5.94137	6.2294	6.07435	5.95877	6.23048	5.87298	6.20535	6.24005	6.17998	5.99404	6.90077	6.61924	6.72748	6.66398
ECT2	Q9H8V3	5.9254	6.18278	6.20357	6.07824	5.99467	6.11558	6.35481	6.33949	6.30735	5.98599	6.17951	6.18747	6.63045	6.32879	6.62553	6.56039
AHSA1	O95433	6.38785	6.15507	6.9243	5.90761	6.4782	6.69364	6.53548	6.48471	6.43906	6.18609	6.65754	6.19536	5.96276	5.68832	5.68817	5.61583
PRDX4	Q13162	6.34897	6.02915	5.97063	5.83053	6.46918	6.1228	5.90168	6.47802	6.00478	6.15	6.00555	6.31111	6.83237	6.47728	6.31926	6.74869
EHD4	Q9H223	6.06113	5.98619	6.35195	5.86814	6.06679	6.31637	6.01374	6.40153	6.67059	6.4212	6.60882	6.62529	6.47473	6.0287	6.03934	6.06551
ZC3H18	E7ERS3	6.24761	6.46745	5.93128	6.7794	6.17793	5.98249	6.32368	6.01356	6.21552	6.09501	5.95233	5.78643	6.5823	6.42374	6.48795	6.5333
RPS6KA1	Q15418-2	6.04419	6.19905	6.75947	5.8326	6.44872	6.74052	6.15915	6.45847	6.38563	6.42843	6.59392	6.46717	5.93471	5.79354	6.01997	5.73446
SMPD4	Q9NXE4-4	6.42907	6.45016	6.11973	6.77563	6.33715	6.05901	6.38609	5.95465	6.30679	6.20946	6.30778	6.24849	6.26654	5.91502	6.11774	6.11669
PSMC2	P35998	6.21835	5.88662	6.80087	6.07114	6.36972	6.44945	6.40308	6.16263	6.04811	6.07916	6.11147	6.24625	6.30368	6.17123	6.16286	6.51538
PAPSS1	O43252	5.56371	5.73719	6.13801	5.42374	5.89555	6.21524	5.88138	6.29134	6.00868	6.0958	6.30099	6.24554	6.85886	7.15144	7.4467	6.74582
XRN1	Q8IZH2	6.09231	6.23684	6.50506	5.92785	6.34141	6.84362	6.24889	6.44602	6.32869	6.45509	6.86383	6.42763	5.92607	6.25112	5.63435	5.47122
PCNA	P12004	5.98282	6.24958	6.14527	5.64891	5.7235	5.97412	5.93431	6.03219	6.3444	6.11335	5.7953	6.08485	6.76018	6.90392	7.11731	7.18999
PBRM1	Q86U86	6.40871	6.40604	6.11271	6.66742	5.90892	5.90223	6.40256	5.92768	6.22923	6.22765	5.87965	5.91	6.51797	6.36834	6.58863	6.54618
CACYBP	Q9HB71	6.36859	6.03644	6.51961	5.86716	6.82755	6.73256	6.41841	6.44166	6.35425	6.4632	6.32364	6.27926	6.09261	5.92298	5.70636	5.64574
ACSL1	P33121	6.33132	6.3945	5.90171	6.70215	6.15722	5.92833	6.3706	5.76685	6.08353	6.10717	5.95334	5.99676	6.57634	6.43476	6.58417	6.71125
ZNF292	O60281	6.25213	6.34029	5.96342	6.8768	5.97675	6.09223	6.48913	5.78812	6.09778	6.03926	5.9701	6.16977	6.34374	6.48274	6.64148	6.38625
ARHGEF2	Q92974	6.44156	6.49991	6.317	6.19783	6.44814	6.59306	6.21811	6.324	6.15577	6.41948	6.46702	6.54394	5.96785	5.9282	5.6762	5.80192
USP39	Q53GS9	6.06811	6.39005	5.9567	6.58838	6.00525	5.97224	6.03739	5.90368	5.90664	5.98181	5.90212	5.91314	6.67882	6.80564	6.84601	7.04404
MME	P08473	6.38312	6.5323	5.95675	6.06087	6.32687	6.13535	6.36686	6.09883	6.21077	6.22448	5.88629	5.78068	6.60516	6.48035	6.58809	6.36324
STAG2	Q8N3U4-2	6.33554	6.54641	5.93036	6.54975	6.15378	6.17179	6.20731	6.18116	6.02252	6.24756	5.80997	5.78812	6.6991	6.30106	6.52042	6.53515
NOTCH2	Q04721	6.32494	6.23063	6.05711	6.4255	6.22908	6.05117	6.32852	6.08143	6.15363	5.90395	6.10717	6.20829	6.71399	6.29256	6.43019	6.46183
PCK2	Q16822	6.34376	6.04996	5.8864	6.29084	6.3932	6.00613	6.29738	5.83244	6.12184	5.9832	5.97041	5.82527	7.25216	6.52303	6.74996	6.47403
UBAP2L	Q14157-5	6.38878	6.32992	6.74836	6.12099	6.35882	6.45704	6.08414	6.43197	6.50736	6.64468	6.87646	6.60786	5.67971	5.89861	5.44455	5.42075
CACNA2D1	P54289	6.21881	6.14943	6.07073	6.25362	6.2536	5.96267	6.17014	5.94294	6.209	6.3003	6.13183	5.86707	6.87531	6.41162	6.78341	6.39951
UHRF1	Q96T88-2	6.26518	6.23217	6.20858	6.66237	6.0535	5.8946	6.26523	5.79429	6.32814	6.21719	6.54509	6.40269	6.27806	6.09257	6.34221	6.41812
ATL3	Q6DD88	6.37946	6.22934	5.90596	6.36054	6.26007	6.19107	6.32817	6.02903	6.08081	6.12596	5.81025	5.98259	6.61352	6.48831	6.55365	6.66127
TIMM44	O43615	6.34433	6.69461	5.90878	6.9006	6.21685	6.2833	6.21751	6.20828	5.929	5.91237	6.095	5.97135	6.29851	6.46246	6.39181	6.16525
PLCB4	Q15147-4	6.18352	6.77806	6.28391	6.67819	6.25706	6.20793	6.46741	6.44722	6.59227	6.25952	6.52378	6.85495	5.59458	5.47874	5.51754	5.87533
GRIPAP1	Q4V328	6.48121	6.08884	6.79909	5.90715	6.57616	6.59579	5.98515	6.6873	6.39911	6.45475	6.49961	6.51061	6.37801	5.72252	5.57265	5.34205
HBS1L	Q9Y450	6.40698	6.27998	6.65568	6.17195	6.41475	6.82474	6.18573	6.40155	6.31677	6.42575	6.32172	6.38658	5.82068	5.82081	5.93165	5.63468
DDX18	Q9NVP1	6.28674	6.24936	5.64184	6.85993	6.0799	5.8755	6.36754	5.96216	6.07428	5.78077	5.91109	5.85161	6.35403	6.71988	6.86289	7.12247
RAB7A	P51149	6.147	6.19326	6.01894	6.23875	6.25054	6.04635	6.4582	6.35999	6.15309	6.11856	6.18496	5.92185	6.61853	6.43831	6.79402	6.55765
RPRD2	Q5VT52	6.47145	6.37844	6.16139	6.48209	6.32843	6.1906	6.29552	5.85045	6.32374	6.15306	6.21168	5.96764	6.54828	6.14938	6.22013	6.26774
KPNA2	P52292	6.4489	6.37947	6.78295	6.64278	6.14777	6.3997	6.65709	6.47909	6.51892	6.45295	6.66161	6.86392	5.29936	5.31288	5.19813	5.7545
NBN	O60934	6.02899	6.4434	6.00609	6.46358	5.97977	6.15645	6.14995	5.936	6.05856	6.16177	5.88142	5.96122	6.7479	6.36888	6.69583	6.96017
AKAP13	Q12802-2	5.86919	5.9223	6.55301	6.00059	6.00625	6.37443	6.07152	6.30674	6.50986	6.69932	6.64566	6.78021	5.93144	6.22508	6.07248	6.03193
RPL28	P46779-3	6.30261	6.24949	6.24781	6.27224	6.17598	6.46916	6.1921	6.24121	6.7865	6.46427	6.51256	6.40495	5.86376	6.20408	5.80536	5.80792
ZYX	Q15942	6.29996	5.32488	6.70285	5.10656	6.70938	6.37355	5.78551	6.95464	6.50052	6.43785	6.87351	6.88033	6.75926	5.89701	5.71638	5.73732
POLR1B	Q9H9Y6-3	6.47796	6.21014	6.22973	6.82892	6.0969	5.8483	6.48276	5.78685	6.5009	5.97229	6.30618	6.2509	6.38623	5.79485	6.36335	6.46375
ARCN1	P48444	6.40062	6.39237	6.7	6.27668	6.41993	6.44472	6.29214	6.41043	6.40397	6.51218	6.76257	6.55191	5.77775	5.68739	5.53313	5.43421
NOC2L	Q9Y3T9	6.39119	6.28658	6.01447	6.68503	6.282	5.80558	6.59515	5.99273	6.42351	5.93351	6.02507	5.84037	6.61538	6.0701	6.45348	6.58584
RBM15	Q96T37	6.32403	6.4361	5.957	6.76983	6.0275	5.84047	6.39322	5.76351	6.14375	6.11511	5.99608	6.20361	6.4398	6.20894	6.76816	6.61288
INA	Q16352	6.44553	6.36612	5.81748	6.42756	6.43918	6.19569	6.23639	5.95001	6.23264	5.98978	6.06226	6.08107	7.05976	6.00215	6.41832	6.27607
ETFB	P38117	6.3529	5.96304	5.80033	6.05237	6.40142	6.11256	6.40358	6.35439	6.29334	5.91103	5.66779	5.96458	7.10127	6.45232	6.5851	6.58397
DEK	H0Y993	6.04394	6.44261	6.09203	6.50735	6.17093	6.18479	6.25245	6.10539	5.96749	6.2763	5.98449	6.02284	6.52671	6.56123	6.4233	6.43815
FUBP3	Q96I24	6.58596	6.41303	6.39231	6.51545	6.68507	6.42402	6.06333	6.13067	6.2674	6.06573	6.62186	6.35252	6.56083	5.64819	5.68747	5.58617
RBM39	Q14498	6.08271	6.40694	5.7667	6.54164	6.18296	5.99583	6.27455	5.89813	5.91286	5.88084	5.82098	5.76643	6.80426	6.71721	6.9801	6.96787
UBE3A	Q05086	5.84438	5.98542	6.20459	5.90711	6.0411	6.33167	6.16964	6.13901	6.14496	6.16182	6.23293	6.27035	6.67664	6.70166	6.67334	6.51538
YBX3	P16989	6.48571	6.36202	6.45245	6.51746	6.30176	6.16689	6.59338	6.36876	6.76262	6.03733	6.35557	6.31232	5.28145	6.21979	5.67418	6.1083
PRC1	O43663	6.06476	6.05325	5.88593	6.20296	5.99745	6.38976	6.2772	6.1929	6.12725	6.12877	6.09906	6.17014	6.71293	6.40588	6.57811	6.71365
XPO7	Q9UIA9	5.55269	5.66348	6.168	5.43954	5.92348	6.23418	5.79063	6.06913	6.07139	6.11708	6.28535	6.05474	7.15741	7.33963	7.19249	6.94077
PDLIM7	Q9NR12	5.98331	6.05436	5.76242	5.65644	6.28423	6.31339	6.13298	6.12382	6.38178	6.38848	6.13237	6.18033	6.90843	6.63896	6.61674	6.44196
TUBGCP2	Q9BSJ2-4	6.28843	6.24522	6.89635	6.23894	6.22278	6.69026	6.21357	6.56453	6.3895	6.4203	6.86493	6.53607	5.56617	5.8	5.53011	5.53282
ILVBL	A1L0T0	6.27781	6.28886	5.85852	6.67547	6.2451	5.97762	6.42171	5.93229	6.14979	6.00106	5.9374	5.89828	6.47529	6.52894	6.69651	6.63536
CHD2	O14647	6.28319	6.07423	6.0505	6.52992	6.31148	5.75116	6.37088	5.70179	6.08679	6.20542	5.86477	5.64086	6.6674	6.69154	7.10565	6.66441
WNK1	Q9H4A3-7	6.35414	6.3189	6.7108	5.93258	6.29623	6.57325	6.12018	6.55188	6.4962	6.65005	6.58284	6.42417	5.78414	5.89026	5.60192	5.71246
SIPA1L1	O43166-2	6.42908	6.49497	6.03879	6.57961	6.19955	5.94199	6.46817	5.97984	6.17217	5.97446	5.9728	5.83172	6.524	6.29349	6.56473	6.53462
SEPTIN2	Q15019-2	5.89843	5.83956	6.61098	5.71322	6.42205	6.6788	6.25896	6.4487	6.39007	6.46728	6.28161	6.22432	6.41109	6.11891	6.17954	6.05647
KIF22	Q14807	6.27189	6.17068	5.67697	6.24026	6.25152	6.03284	6.40841	6.20178	6.14102	5.92557	5.73164	5.84277	6.70926	6.666	6.89114	6.83824
IGF2BP1	Q9NZI8	6.24768	6.34013	6.42318	6.05625	6.60763	6.87326	6.32416	6.52556	6.49919	6.32376	6.73873	6.16419	5.92266	5.92363	5.57524	5.45476
HTATSF1	O43719	6.16373	6.3443	6.11688	6.59134	6.03276	6.05247	6.16938	5.87138	6.05977	6.33915	6.31847	6.26202	6.55666	6.33551	6.39405	6.39212
VPS35	Q96QK1	6.03472	6.14558	5.88906	5.83876	6.15866	6.49433	5.91066	6.45728	6.2128	6.29292	6.02223	6.21565	6.30571	6.92023	6.53662	6.5648
PRPF40A	O75400	6.22845	6.41479	5.8483	6.6742	6.27387	5.93941	6.48266	5.88775	6.06211	5.8704	5.92263	5.93959	6.83583	6.55329	6.46832	6.59842
RRP1B	Q14684	6.18423	6.17321	5.83124	6.76974	6.16393	5.841	6.50155	5.74491	5.97318	5.85866	6.11088	5.97612	6.65943	6.55014	6.91423	6.74757
TRAPPC8	Q9Y2L5	6.14154	6.1889	6.46733	6.00831	6.22667	6.69885	6.03881	6.4429	6.37312	6.47851	6.42909	6.50337	5.98938	6.1675	5.88902	5.95671
CRYBG3	Q68DQ2	6.18724	6.1773	6.69176	6.06663	6.10605	6.36169	6.08095	6.37544	6.50957	6.5903	6.89863	6.74872	5.96503	5.76858	5.78007	5.69204
THOC5	Q13769	6.35322	6.49308	5.93361	6.68894	6.16029	6.0208	6.43835	6.05681	6.11241	6.01687	6.02999	5.91226	6.6396	6.26232	6.5176	6.36386
CLCC1	Q96S66	6.29637	6.34186	6.09645	6.60459	6.19848	6.03575	6.11548	6.09974	6.03637	6.03239	6.0105	5.89614	6.59552	6.20581	6.62693	6.80763
HMOX2	P30519	6.36964	6.07826	5.93673	5.93132	6.62334	5.86392	6.29736	6.37879	6.021	6.01581	6.12025	6.10721	6.82222	6.47014	6.52528	6.43874
RPL10A	P62906	6.27509	6.52747	6.29521	6.27503	6.17594	6.59954	6.37872	6.32071	6.45422	6.40123	6.3251	6.42473	5.85231	5.84447	5.83052	6.01972
MAPRE1	Q15691	6.08293	5.74132	6.8034	5.53488	6.39366	6.56575	6.02409	6.65593	6.17648	6.40513	6.72661	6.886	6.13029	5.98554	5.84196	6.04602
CNN3	Q15417	6.1417	6.09153	7.09516	5.98603	6.07903	6.74051	5.9393	6.72817	6.64474	6.75019	6.98799	7.01711	5.88603	5.63718	5.09392	5.18139
PELP1	Q8IZL8	6.48202	6.31606	5.97262	6.82822	6.19863	6.03542	6.27478	5.94099	6.02875	5.91941	6.20919	6.18015	6.71262	6.37892	6.32906	6.19316
DECR1	Q16698	6.33262	6.20117	5.80459	6.39706	6.18537	5.78714	6.35623	6.14641	6.21101	5.92215	5.78733	5.86432	6.80055	6.61773	6.82908	6.75722
RPL7A	P62424	6.28422	6.14391	6.15461	5.98219	6.33273	6.24494	6.38669	6.40287	6.63082	6.51035	6.3042	6.24711	5.87293	6.28753	6.08566	6.12923
PUM3	Q15397	6.20413	6.23899	5.85061	6.9513	6.13732	5.63918	6.41974	5.87798	6.01585	5.86491	5.88089	6.20555	6.3787	6.44179	6.87907	7.01399
RPL18A	Q02543	6.32525	6.273	6.34314	6.39368	6.20951	6.34678	6.39298	6.32887	6.61408	6.59503	6.4411	6.51333	5.74676	5.83415	5.69062	5.95174

DHX8	Q14562	6.47167	6.78319	5.94208	6.85533	6.18794	6.07828	6.39497	6.18766	6.3112	6.15295	6.23179	6.10036	5.89568	6.14284	6.17873	6.08533
CDC42BPB	Q9Y5S2	6.4401	6.3793	7.03167	6.0422	6.34271	6.66294	6.27671	6.29924	6.49267	6.56107	6.65054	6.54451	5.83879	5.56183	5.6003	5.27544
ATR	Q13535	6.02243	6.35039	6.22997	6.33351	6.319	6.28914	6.24878	6.10832	6.25894	6.3752	6.2702	6.20311	6.16907	6.25399	6.20366	6.3643
GFM1	Q96RP9	6.38738	6.21426	5.83925	6.49656	6.46322	6.0457	6.3284	6.15595	6.1367	5.83433	5.8497	5.81196	6.77373	6.4376	6.80566	6.41959
RFC3	P40938	6.04669	6.41837	6.31189	6.21914	6.22718	6.31757	6.1713	6.14148	5.97331	6.20992	6.2699	5.98892	6.54844	6.27774	6.5536	6.32454
KLC1	Q07866-6	6.37976	6.40603	6.80315	5.84015	6.47507	6.7808	6.27737	6.757	6.58503	6.45097	6.59529	6.54963	5.85281	5.5627	5.30185	5.38237
CSNK2A1	P68400	6.16139	6.43859	6.41063	6.498	6.38829	6.57838	6.20513	6.44051	6.06064	6.22002	6.33253	6.09239	6.06862	6.02959	6.19809	5.87719
KIF1B	O60333-2	6.31249	6.35823	7.01666	6.16867	6.23461	6.72574	6.3632	6.34763	6.4124	6.48757	6.48101	6.55175	5.6411	5.87578	5.4078	5.61534
MED23	Q9ULK4	6.03548	6.25532	5.97817	6.34078	6.01061	6.07408	6.06007	5.93619	6.26518	6.2503	6.23495	6.2275	6.58915	6.45226	6.69253	6.59745
EMSY	Q7Z589-7	6.56299	6.44399	6.16434	6.74803	6.18837	6.13974	6.12359	5.95938	6.17866	6.36473	6.09533	6.11072	6.4502	5.96898	6.29353	6.20741
NARS1	O43776	6.21478	6.14811	6.5972	5.83883	6.05827	6.52691	6.23517	6.48315	6.46003	6.38672	6.62992	6.51508	5.89581	6.21718	5.77809	6.01476
PRPF19	Q9UMS4	5.8888	6.34528	5.33933	6.37259	6.45974	6.41319	6.08769	6.64665	6.14622	6.38204	5.88362	6.05005	6.22427	6.34228	6.62844	6.78982
NAA25	Q14CX7	6.14612	6.38814	6.80056	5.97771	6.30585	6.71293	6.17895	6.78696	6.31472	6.61419	6.6354	6.40849	5.66814	5.80556	5.62842	5.62786
CD44	P16070	6.43233	6.47085	5.9028	6.54887	6.18629	6.10726	6.17604	6.49173	6.21375	5.91393	5.87981	5.93222	6.46515	6.54075	6.17282	5.6554
TBCD	Q9BTW9	6.49534	6.13617	7.00457	6.24417	6.39176	6.89812	6.28652	6.63322	6.44796	6.33285	6.65276	6.68663	5.50721	5.56687	5.29029	5.42558
INTS4	Q96HW7	6.22917	6.32017	5.98897	6.53765	6.24619	6.06099	6.21364	5.95856	6.13813	6.24219	6.17711	6.0045	6.51987	6.2548	6.5765	6.53156
CTTN	Q14247	6.19886	6.29882	6.41169	6.29873	6.32826	6.63797	6.03967	6.75085	6.01509	5.93438	6.44291	6.14453	6.25666	6.11647	6.18897	5.93616
BAZ1A	Q9NRL2	6.45799	6.6076	6.15845	7.06223	6.12091	5.88945	6.65406	6.18397	6.23967	6.08767	6.16679	6.19253	5.99018	5.88645	6.07607	6.22596
NCAPH	Q15003	6.41304	6.32106	6.73135	6.27407	6.34148	6.63018	6.04387	6.77183	6.40277	6.50306	6.67938	6.85531	5.65257	5.6371	5.38895	5.35398
TRIP10	Q15642-2	6.17935	6.39225	6.92996	6.01851	6.42989	6.88639	6.26104	6.46042	6.4511	6.61991	6.59162	6.7724	5.6945	5.67866	5.26752	5.36648
SACM1L	Q9NTJ5	6.32931	6.28851	6.12371	6.52018	6.2153	6.08819	6.39315	6.00402	6.12151	6.00907	6.04814	5.85681	6.6188	6.33316	6.64224	6.4079
ARPC2	O15144	6.28448	6.40505	6.48422	6.10289	6.27009	6.3893	6.40416	6.55638	6.15788	6.38643	6.21098	6.11266	5.92284	6.02297	6.33294	5.95674
GNL2	Q13823	6.36236	6.24568	6.10183	6.77265	6.18572	5.8971	6.37033	5.91728	6.22535	6.03152	6.2022	6.15919	5.98577	6.35283	6.50866	6.68153
PLRG1	O43660	6.31591	6.41516	5.81329	6.7768	6.04362	5.84837	6.56142	6.0488	6.2828	6.42004	5.88716	5.98411	6.08361	6.47686	6.55814	6.48391
CKAP4	Q07065	6.38481	6.51838	6.1805	6.43844	6.37448	5.93155	6.2758	6.09381	6.1909	5.9541	5.96987	5.77601	6.71789	6.25797	6.57304	6.36244
GFPT1	Q06210	6.36397	6.36234	6.77434	6.00069	6.69079	7.02573	6.3369	6.71157	6.49119	6.08233	6.32038	6.36655	5.47925	6.03216	5.48327	5.47854
MSH3	P20585	5.65405	6.05214	5.87361	5.8399	6.0146	6.23968	6.18314	5.56711	5.99652	6.10872	6.26847	5.96585	6.91143	6.99452	7.1405	6.89119
TWF1	Q12792	6.28597	6.50134	6.83451	6.45683	6.35898	6.87616	6.35362	6.74597	6.46177	6.29606	6.24595	6.37035	5.45216	5.63236	5.63809	5.48989
NOL10	Q9BSC4	6.17467	6.1351	5.86186	6.72188	6.14199	5.89628	6.50571	5.78492	6.19709	6.05547	5.92931	6.07469	6.62515	6.34388	6.82854	6.72344
ZNF644	Q9H582	6.49098	6.6607	6.08145	6.87738	6.11497	6.14571	6.29051	5.99775	6.252	6.29661	6.06097	6.29132	6.09311	6.14843	5.98846	6.20965
WDR44	Q5JSH3	6.21781	5.94672	6.74138	5.70722	6.36771	6.51815	6.45631	6.53201	6.3907	6.34354	6.45257	6.28097	6.08374	6.15761	5.97215	5.8314
RPL12	P30050	6.37786	6.40444	6.36815	6.14172	6.44868	6.59764	6.22901	6.26481	6.50051	6.40099	6.53112	6.51108	5.86127	5.88067	5.73445	5.74758
VAPA	Q9P0L0	6.42763	6.1219	5.90858	6.19713	6.32731	6.12082	6.24046	6.44377	6.06379	6.2642	5.86101	6.00727	6.97523	6.4243	6.33455	6.28206
RPS4X	P62701	6.74143	6.3447	6.63161	6.71623	6.7562	6.46905	6.48255	6.23953	6.51326	6.0028	6.01418	6.13984	5.71082	5.87305	5.54807	5.81667
EIF2S2	P20042	6.02796	6.30071	6.53697	6.16382	6.11732	6.44383	6.16718	6.39771	6.24	6.59586	6.24395	6.52171	5.80666	6.21415	6.06163	6.16056
PRDX6	P30041	6.14061	5.27879	6.74754	4.99758	6.54945	6.25921	5.83016	7.22997	5.8051	6.94941	6.33829	7.15478	5.94278	6.62718	6.05172	6.09743
ASH2L	Q9UBL3	5.99945	6.38179	5.67903	6.43811	6.18092	6.18396	6.25984	6.17537	6.19617	6.10303	5.92653	6.08248	6.66488	6.45834	6.65219	6.61791
SNW1	Q13573	5.87035	6.04295	5.73888	6.43703	6.10459	5.76926	6.86704	5.83042	6.50944	5.57447	5.79579	5.8319	6.30651	6.86315	6.95355	7.50106
MDH1	P40925-3	6.32641	6.2663	6.98747	5.86665	6.36167	6.77664	6.25546	6.33292	6.31738	6.56645	6.65146	6.31822	5.83502	5.95008	5.70996	5.47791
CKB	P12277	6.22577	6.22216	6.95624	6.03393	6.59558	6.8348	6.13785	6.46488	6.27887	6.40234	6.31841	6.46215	5.85337	5.99226	5.59322	5.62817
SRP54	P61011	6.29619	6.30435	6.26963	6.20369	6.28756	6.23501	6.26335	6.12292	6.29844	6.25794	6.19548	6.09973	6.2753	6.23629	6.41214	6.23838
FARSA	Q9Y285	6.07273	6.22441	6.73426	6.0786	6.25277	6.63103	6.08625	6.45496	6.64489	6.50004	6.79528	6.72567	5.81664	5.70137	5.65483	5.62627
CLPTM1	O96005	6.23748	6.56754	6.03589	6.82873	6.09979	6.15151	6.29942	5.99534	6.30088	6.24906	6.15391	6.30485	6.07518	6.17157	6.10397	6.42486
VDAC3	Q9Y277	6.21267	6.31087	5.82742	6.63041	6.47422	5.98113	6.44573	6.05327	6.24252	6.1	5.75092	5.89367	6.68663	6.14245	6.94398	6.33111
BRD2	P25440-2	6.32536	6.57302	6.09412	6.64974	6.09533	6.15	6.35873	5.9093	6.38172	6.20222	6.17683	6.23292	6.06309	6.05479	6.20623	6.5266
TPM3	P06753-2	6.60656	6.14509	6.3796	6.18463	6.85581	6.18508	6.0093	6.74078	6.37341	5.96173	6.66906	6.54473	6.49212	5.84808	5.30319	5.70082
DGLUCY	Q7Z3D6-2	6.33921	6.21634	5.67441	6.01649	6.33256	6.1521	6.26013	5.88853	6.02463	6.28621	6.08175	6.13582	6.69827	6.57847	6.85642	6.45867
RCN1	Q15293	6.5309	6.25956	6.04772	6.23247	6.54529	6.01427	6.10441	6.11703	6.1731	6.01503	6.4019	6.15254	7.1063	5.95909	6.1844	6.156
TTC37	Q6PGP7	6.46573	6.35773	6.61491	6.03863	6.30653	6.65829	6.15169	6.67768	6.42629	6.55487	6.27226	6.2896	5.85252	6.08755	5.64635	5.59936
NEMF	O60524	6.03154	5.92346	6.30602	5.9504	5.95108	6.32345	6.07574	6.22158	6.03126	6.44971	6.53737	6.43697	6.17783	6.75929	6.65856	6.16574
SUPV3L1	Q8IYB8	6.14285	6.28219	6.05435	6.60423	6.1453	6.03561	6.39957	5.8021	5.98093	6.04093	5.96897	6.01232	6.77692	6.32041	6.66423	6.76908
EIF4G3	A0A0U1RQK7	6.15439	6.30805	6.63431	6.06849	6.02739	6.45602	6.11112	6.31001	6.36742	6.587	6.38197	6.52154	6.01172	6.28933	5.83537	5.93586
ECH1	Q13011	6.15947	6.10831	5.94267	6.32647	6.26403	6.03219	6.60232	6.02115	6.42385	5.9583	6.08423	5.95875	6.67461	6.29427	6.53785	6.61154
RAI14	Q9P0K7	6.15123	6.23962	6.15587	6.02474	6.43355	6.28155	5.85044	6.20347	6.22282	6.43081	6.34137	6.20319	6.7212	6.27018	6.246	6.22396
PSMA2	P25787	6.08175	6.21794	6.20664	6.0744	6.40225	6.69871	6.24118	6.6111	6.3267	6.07686	6.2912	6.04854	6.43389	6.14236	6.15659	5.98988
ITSN1	Q15811-2	6.04387	6.13737	6.33972	5.95572	6.23947	6.38209	6.04594	6.23645	6.36782	6.41762	6.55944	6.26447	6.61352	6.14996	6.24028	6.00626
SEC23A	Q15436	6.07415	6.27901	6.31523	5.98746	6.16629	6.21242	6.26981	6.14701	6.0222	6.22621	6.14067	6.09403	6.33767	6.63168	6.68123	6.41492
RALY	Q9UKM9	6.52447	6.48413	5.86161	6.7116	6.44891	5.92857	6.46923	6.00812	6.12596	5.95722	5.74377	5.76623	6.65747	6.15973	6.6587	6.49429
PARP4	Q9UKK3	6.4589	6.34802	6.72164	6.09146	6.2352	6.63813	6.23087	6.5332	6.56991	6.49152	6.49229	6.41857	6.59842	5.8996	5.58621	5.58605
FKBP15	Q5T1M5	5.97743	6.05892	6.19055	5.78393	6.17326	6.20387	5.83471	6.02443	6.38434	6.62369	6.69952	6.56032	6.53029	6.28025	6.34103	6.33346
DDX10	Q13206	6.23877	5.95764	5.69783	6.69347	6.09239	5.86746	6.44292	6.07437	6.06423	5.52828	5.67976	5.66432	6.94832	6.68998	7.10969	7.25057
PLOD2	O00469-2	6.52405	6.27943	5.82368	6.42103	6.65688	6.06134	6.40033	6.00394	6.02897	6.03572	6.05794	5.84519	6.51303	6.66243	6.55487	6.13115
MMS19	Q96T76-8	6.1241	6.15707	6.56852	5.91647	6.20356	6.61409	6.08098	6.46011	6.31976	6.41433	6.54956	6.36062	5.88949	6.28176	5.85644	6.20314
CTR9	Q6PD62	6.17717	6.31935	5.93344	6.9416	6.2575	6.2499	6.38443	5.95913	6.1258	5.94916	5.98384	5.97906	6.21885	6.3419	6.68333	6.49553
ISYNA1	Q9NPH2	5.75786	5.62034	6.60057	5.37381	6.51667	6.91084	6.56863	6.6898	6.06018	6.47973	6.5434	6.24732	6.47052	6.21043	6.00056	5.94935
SART1	O43290	6.43622	6.4064	5.93904	6.54357	5.97332	5.84331	6.40057	5.82586	6.13515	6.09696	6.29563	5.81737	6.26825	6.8403	6.58	6.59806
PITPNB	P48739-3	6.16208	6.12234	6.86974	6.1186	6.36935	6.81821	6.27958	6.6582	6.3454	6.38311	6.53753	6.61238	5.81658	5.80003	5.57817	5.5287
EEF1D	P29692-2	6.59633	6.27463	6.68119	5.76119	6.2859	6.42264	5.90211	6.64324	6.52326	6.45768	6.44087	6.50044	6.11407	5.9181	5.53849	5.93986
DCAF1	Q9Y4B6	5.89388	6.18903	6.04099	6.25165	6.20207	5.93211	6.12598	5.95157	5.9425	6.00041	5.92371	5.99061	6.97944	6.53594	7.16827	6.87184
LUC7L2	Q9Y383	5.63983	6.08198	5.95816	5.40853	5.97797	6.22523	5.80319	5.89523	6.07363	6.20095	6.09358	5.91911	6.89391	7.44863	7.30176	7.07833
PKM	P14618	6.32369	6.19415	6.98249	5.89277	6.59377	7.10077	6.46396	6.14082	6.5407	6.70503	6.38423	6.30415	5.89733	5.41469	5.78401	5.27742
ASPH	Q12797	6.31191	6.35176	5.90119	6.34446	6.17529	5.83564	6.27689	6.01258	6.10329	6.16332	5.93153	5.71099	6.76165	6.60189	6.90722	6.61037
SNRPB	P14678-3	6.21388	6.4144	5.95264	6.36159	6.46649	6.11893	6.35989	6.12802	6.23328	6.29563	6.06738	5.96941	6.77059	5.8855	6.4422	6.32018
NCAPD3	P42695	5.89819	6.55189	6.28221	6.69928	6.09248	6.44705	6.3245	6.2683	6.02115	6.48976	6.16146	6.48651	5.96659	6.12863	5.95044	6.23157
CCDC88C	Q9P219	6.23533	6.62851	6.60927	6.52088	6.30863	6.32961	6.21961	6.60995	6.37674	6.50816	6.65202	6.59168	5.585	5.69734	5.45334	5.67392
MCCC2	Q9HCC0	6.35099	6.46914	5.97948	6.73805	6.51049	5.89042	6.22433	6.04486	6.08062	6.11269	6.14204	6.11126	6.62942	6.12066	6.43088	6.16468
SDHB	P21912	6.2211	5.99442	5.64673	6.61056	6.45036	5.80312	6.84442	5.6937	6.68653	5.71849	5.59867	6.00138	6.67537	6.29749	6.50113	6.86654
HDGFL2	Q7Z4V5-2	5.97574	6.19203	5.97745	5.75823	6.08341	5.99908	6.07808	5.77385	5.92275	6.10803	6.33252	6.16493	6.91331	7.0706	6.86409	6.7859
OXCT1	P55809	5.99484	6.14449	5.64293	6.20081	6.33148	5.9683	6.50846	6.40295	6.58121	6.27772	6.37382	5.96553	6.647	6.14836	6.60487	6.20724
SETDB1	Q15047	6.10956	6.55367	6.12271	6.70144	6.09135	6.19912	6.37851	6.2097	6.01833	6.49215	6.38029	6.35698	6.18189	5.86638	6.18449	6.15345
RAD21	O60216	6.35376	6.53551	6.02898	6.50113	6.23752	6.24381	6.34651	6.28469	6.10704	5.97435	6.02339	5.75999	6.42199	6.34579	6.39892	6.43661
ELAVL1	Q15717-2	6.36999	6.45819	5.78361	6.51776	6.36448	6.20297	6.25851	6.3272	5.8164	6.11801	5.94891	6.07427	6.75052	6.45334	6.32169	6.23416
POLRMT	O00411	6.30686	6.33802	6.21747	7.08014	6.25951	6.29461	6.43319	6.07606	6.37156	6.17304	6.18041	6.26462	6.30075	5.48238	6.11759	6.10379
GEMIN4	P57678	6.30239	6.59922	6.66045	6.26682	6.23437	6.44675	6.17554	6.38383	6.39885	6.33483	6.49458	6.3902	5.71233	5.9142	5.97574	5.7099
KIF4B	Q2VIQ3	5.66208	6.12947	6.22301	5.95739	6.15292	6.36205	6.2014	5.80782	5.97898	6.60685	6.49643	6.21703	6.55369	6.51276	6.6537	6.4844
XPOT	O43592	5.69478	5.91614	6.31171	5.61057	5.7501	6.27412	5.97026	6.39474	6.28315	6.55593	6.28542	6.2694	6.20447	6.60752	7.13132	6.74037
METAP2	P50579	6.0235	6.02966	6.79366	6.08021	6.01239	6.17647	6.28932	6.1366	6.41228	6.31672	6.87514	6.71385	5.84858	6.31944	5.79798	6.17419
GLYR1	Q49A26	6.11081	6.2016	5.86123	6.5469	6.05361	5.99302	6.57228	6.06823	6.0052	6.06676	5.87663	5.794	6.64649	6.58094	6.77111	6.8512
RBMXL1	Q96E39	6.39751	6.50636	5.79647	6.84099	5.98526	5.87296	6.32439	6.01928	6.04952	5.98419	5.92154	5.9505	6.59623	6.47941	6.55762	6.71778
FKBP5	Q13451	5.47387	5.98316	6.10168	5.52001	5.94374	5.98024	5.91473	5.82055	5.82902	6.04078	6.06349	5.83681	7.59233	7.04808	7.6058	7.24572
TMF1	P82094-2	6.3193	6.08255	5.94374	6.21879	6.40086	6.21747	6.25776	6.1914	6.2996	6.06607	6.56459	6.34143	6.69889	5.96436	6.19423	6.23896
RFC5	P40937	6.01733	6.16152	6.1998	6.03017	6.34205	6.38373	6.1596	5.9529	6.2198	6.20625	6.10243	6.20454	6.68644	6.43666	6.54311	6.35366
KDM2A	Q9Y2K7	6.63385	6.65413	6.14942	6.9472	6.11653	5.99114	6.36518	6.05934	6.32501	6.24744	6.16117	6.17029	5.93345	5.85148	6.15154	6.24282
RANBP3	Q9H6Z4	5.39915	5.51342	5.9622	5.38187	5.52358	5.94607	5.89619	5.74174	5.98159	6.13698	6.24477	6.19172	7.43547	7.42892	7.72703	7.48931
PAK2	Q13177	6.1775	6.02382	6.71713	6.00276	6.6248	6.67356	6.33926	6.69834	6.54095	6.41122	6.49162	6.60597	5.82922	5.7192	5.5168	5.62786
DPP9	Q86TI2-2	6.03142	6.21728	6.86469	5.77422	6.38601	6.83141	6.19183	6.35348	6.36982	6.55922	6.70655	6.41554	5.80183	5.96096	5.95728	5.57844
RPL15	P61313	6.25178	6.24067	6.20636	6.11142	6.29848	6.33312	6.4308	6.22046	6.67456	6.60338	6.56261	6.44733	5.89535	6.06709	5.78869	5.86789
SAE1	Q9UBE0	5.42164	5.47478	5.96069	4.97033	5.87385	6.28032	5.56351	6.21376	5.88238	5.90767	6.00541	5.89477	7.20503	7.96005	7.95182	7.43397
GRSF1	Q12849	6.5822	6.40415	6.165	6.47644	6.75662	6.24474	6.51564	6.43756	6.21175	6.01573	6.33618	6.34796	6.30434	5.46621	5.81981	5.91566
LRRC59	Q96AG4	6.5131	6.41514	6.00406	6.67389	6.14962	5.97976	6.52087	5.97148	6.11857	6.0475	6.09767	5.94693	6.11511	6.2889	6.72376	6.43365
ADNP	Q9H2P0	6.26275	6.42523	5.96726	6.94922	6.08755	6.02835	6.17134	5.76067	6.14684	6.16158	6.10953	6.2399	6.43153	6.4459	6.41884	6.39349
SNRPA	P09012	6.44419	6.18869	5.99962	6.37027	6.32212	5.61033	6.43377	6.16192	6.30895	5.95288	5.81345	6.0268	6.69805	6.47238	6.54068	6.65588
CUL5	Q93034	5.73799	5.93831	6.11099	5.66823	5.93109	6.18717	5.98686	5.99925	6.14743	6.15416	6.14497	6.09075	6.90247	7.16725	7.03017	6.80292
SF1	Q15637-6	5.97177	6.36571	5.8843	6.02968	6.28475	6.22825	6.005	6.09135	6.1983	6.36365	6.31712	6.13091	6.97589	6.3142	6.50143	6.3377
WASHC4	Q2M389	5.99199	6.48311	6.19554	5.93913	6.0726	6.38266	5.94961	6.20514	6.18704	6.43192	6.40423	6.44604	6.28048	6.55468	6.37355	6.10229

INTS11	Q5TA45	6.24925	6.45479	6.17455	6.72522	6.28576	6.33135	6.21149	6.10629	6.0107	6.23689	6.45784	6.44591	6.11868	5.90647	6.27461	6.0102
PTPN1	P18031	6.36544	6.68203	5.85363	6.84086	6.06732	6.11469	6.27393	6.08481	6.00426	6.0279	5.70865	5.94803	6.4914	6.33748	6.59018	6.6094
NFXL1	Q6ZNB6	6.20511	6.03551	6.02381	6.73094	6.18471	5.77689	6.20064	5.70898	6.11806	6.18679	6.08465	5.87206	6.67647	6.67171	6.80228	6.72139
USP15	Q9Y4E8	6.18794	6.13577	6.59159	6.10825	6.22868	6.4048	6.18228	6.36005	6.20519	6.28732	6.41675	6.39367	6.22457	5.95216	6.04059	6.28038
G6PD	P11413-2	6.38355	6.03843	6.81781	5.93962	6.50678	6.69244	6.26469	6.56289	6.45402	6.39646	6.50038	6.3835	5.91761	5.88236	5.66287	5.59659
TARDBP	Q13148	6.05302	6.01079	5.89127	6.22144	6.27884	5.8709	6.2541	5.86853	6.33882	6.11607	6.21474	6.15681	7.0244	6.208	6.88672	6.60556
APMAP	Q9HDC9	6.26896	6.41125	5.93539	6.38117	6.2332	6.02323	6.25097	5.95103	6.25542	6.03267	5.80011	5.77588	6.85869	6.45494	6.85445	6.51264
NUDCD1	Q96RS6	5.49541	6.30539	6.48383	5.61975	5.97617	6.157	5.92587	6.40611	5.99647	6.04843	6.30064	6.02631	6.5597	6.87083	7.14118	6.6869
ALDH3A2	P51648-2	6.49496	6.62297	5.80096	6.57513	6.52972	6.40169	6.29921	6.39363	5.74603	5.93816	6.06919	5.77799	6.37357	6.43132	6.5299	6.01556
GLDC	P23378	6.15649	6.18001	5.80507	6.55856	6.37612	6.1313	6.46083	5.98702	5.89927	5.84647	6.03576	5.77727	6.79609	6.48334	6.76354	6.74285
ALDH7A1	P49419-2	6.30904	6.34031	5.81497	6.17974	6.33617	6.3807	6.42732	6.14211	6.12517	5.92197	6.13404	5.86609	6.60026	6.36654	6.58286	6.47269
DDX6	P26196	6.35517	6.38443	6.54925	6.03612	6.31054	6.45338	6.20743	6.2714	6.39073	6.50922	6.49617	6.44587	5.77634	6.1158	5.80589	5.89226
ABCF2	Q9UG63-2	6.86632	6.58901	6.95301	6.75738	6.57047	6.68213	6.52557	6.56088	6.79097	6.53405	6.55737	6.53915	5.02091	5.1274	5.04532	4.88006
KIF20A	O95235	6.73901	7.03242	6.59673	7.79443	6.59311	6.13663	6.59561	6.11691	6.4153	6.45877	6.46404	6.6242	5.0837	5.09183	5.20993	5.64738
SOGA1	O94964-2	6.42515	6.57925	6.69473	6.56783	6.2827	6.70536	6.37321	6.68151	6.53436	6.75282	6.69214	6.70927	5.16092	5.35072	5.11089	5.37913
ERCC6L	Q2NKX8	6.41374	6.2267	6.85455	6.32727	6.1615	6.7548	6.21073	6.63371	6.46682	6.45594	6.75287	6.809	5.47575	5.61965	5.31982	5.51715
GALNT2	Q10471	6.4772	6.38278	6.05826	6.73341	6.04021	5.72461	6.54847	5.99963	6.27452	5.87565	6.27051	6.02342	6.40972	6.17885	6.5501	6.45266
CPT1A	P50416	6.35664	6.31586	5.92039	6.78935	6.25562	5.93054	6.60947	5.63291	6.33071	5.86778	5.8703	5.89002	6.33672	6.40203	6.81241	6.67926
CUL7	Q14999	6.57484	6.65809	6.96118	6.38569	6.36795	6.8193	6.31991	6.59908	6.66332	6.89321	6.93383	6.79881	4.99404	5.28355	4.79461	4.9526
IQGAP3	Q86VI3	6.29663	6.43308	6.78904	6.4175	6.10913	6.42864	6.4138	6.69897	6.58707	6.46572	6.63023	6.69026	5.49244	5.63591	5.3625	5.54907
SMARCD2	Q92925	6.10764	6.41512	6.0805	6.22099	6.1078	6.10888	6.25185	6.16267	6.05756	6.34571	5.97917	5.99573	6.52925	6.35363	6.71593	6.56756
ARID1B	Q8NFD5-3	6.1771	6.49495	6.26309	6.25636	6.09374	6.20926	6.27034	5.90847	6.25884	6.19088	6.10864	5.97549	6.75213	6.28265	6.40274	6.35531
RPL11	P62913	6.31588	6.35034	6.67317	6.35355	6.46131	6.74897	6.36062	5.95669	6.27939	6.43047	6.59129	6.47	5.88541	5.82513	5.63632	5.56146
ANPEP	P15144	6.15114	6.42009	5.87433	6.22119	6.27376	6.07578	5.84754	5.65825	6.23692	6.1769	6.09257	6.0368	6.91406	6.82894	6.64749	6.54423
ZNF598	Q86UK7	6.09789	6.3964	6.71459	6.2348	6.0573	6.47805	6.23071	6.31696	6.64157	6.52322	6.65109	6.70669	5.60772	5.9777	5.67853	5.68677
ADH5	P11766	6.24734	5.89896	6.74596	5.8197	6.33036	6.69163	6.16419	6.38033	6.27249	6.45293	6.54144	6.45873	6.07781	6.16822	5.92854	5.82137
BAZ2A	Q9UIF9	6.84866	6.90095	6.30528	7.21048	6.34728	6.11777	6.50274	5.86283	6.43359	6.13524	6.18576	6.14566	5.3256	5.89127	5.6986	6.0883
UBE2O	Q9C0C9	6.27739	6.18284	7.10603	6.08355	6.26503	6.68145	6.28294	6.58045	6.53834	6.54264	6.57128	6.64083	5.7923	5.473	5.41818	5.56374
IWS1	Q96ST2	6.05841	6.32737	5.97073	6.61735	6.11545	6.35358	6.2014	5.96202	6.04969	6.10987	6.05119	6.23681	6.62988	6.54366	6.24841	6.52418
CTNNB1	P35222	6.23206	6.41103	6.15239	6.48504	6.05055	6.26221	6.12605	5.99263	6.31875	5.99136	6.12104	6.44047	6.29255	6.27301	6.38915	6.46171
GATAD2A	Q86YP4-3	6.36097	6.57931	6.00562	6.66086	6.34039	6.07141	6.219	6.16568	6.08976	6.16424	6.15835	6.42438	6.30453	5.96709	6.17529	6.31313
CMTR1	Q8N1G2	5.78462	6.02234	5.95626	5.33464	5.86663	6.03787	5.49328	5.84372	6.28771	6.34665	6.45262	6.16508	6.84153	7.11721	7.38131	7.06851
HEATR5B	Q9P2D3	6.47437	6.25345	6.98723	6.4107	6.18515	6.64633	6.32761	6.52724	6.46607	6.49397	6.48542	6.35992	5.55424	5.83064	5.38399	5.61367
PPP6R3	Q5H9R7-5	6.35598	6.04898	6.59599	6.17577	6.28038	6.70061	6.22699	6.38487	6.36717	6.35968	6.33953	6.46646	6.10984	5.8772	5.82663	5.88394
ANXA4	P09525	6.28648	6.14087	6.64942	5.75904	6.39407	6.80092	6.09716	6.62983	6.33546	6.48792	6.48732	6.3762	5.92247	6.41958	5.67712	5.53615
OGT	O15294	5.42216	5.22432	5.41132	5.15554	6.79957	6.78026	6.56355	6.73308	6.38079	6.07081	6.46237	6.17568	7.08513	6.58041	6.69553	6.45947
SARS2	Q9NP81-2	6.52915	6.55955	5.74307	6.49499	6.45948	6.14192	6.14015	6.09008	6.17367	6.09643	6.11243	6.15697	6.43942	6.26105	6.4742	6.12745
PFKM	P08237-3	6.40528	6.21668	6.8429	5.82222	6.39175	6.91556	6.39752	6.57396	6.27135	6.61686	6.49779	6.44886	5.55799	5.94735	5.48858	5.60534
RPS10	P46783	6.39795	6.56236	6.61746	6.3417	6.37652	6.60611	6.2003	6.64139	6.55338	6.75473	6.54227	6.5836	5.5334	5.63445	5.26313	5.39124
GNL3	Q9BVP2	6.27354	6.3862	5.73281	6.87326	5.91733	5.94321	6.47307	5.82616	6.28755	5.6104	5.59068	5.84727	6.261	6.75318	6.93795	7.28639
EGFR	P00533	6.43501	6.78518	5.98522	6.63489	6.35062	6.2986	6.3587	6.37164	6.19487	6.05179	6.10836	6.04757	6.18684	6.86914	6.03566	6.28591
PAICS	P22234-2	6.27627	6.1963	7.11184	6.03678	6.38742	6.87072	6.26758	6.86805	6.40664	6.29302	6.4713	6.55418	5.71319	5.56595	5.60255	5.3782
CIC	I3L2J0	6.46712	6.6413	6.2604	6.47338	6.23414	6.1665	6.10361	5.94993	6.36957	6.45717	6.62151	6.28153	6.37956	5.8694	5.86062	5.86426
CTPS1	P17812	6.49998	6.32462	6.81032	6.17425	6.61836	6.95065	6.15484	6.91158	6.52704	6.2254	6.48159	6.43408	5.70671	5.3819	5.30021	5.49848
NCAPG2	Q86XI2	6.25593	6.27609	6.27734	6.52332	6.25576	6.21517	6.27901	6.17696	6.37901	6.48924	6.33964	6.2709	5.94749	6.07415	6.08315	6.15684
APLP2	Q06481	6.77207	6.75624	6.20743	6.64032	6.31581	6.09099	6.33495	6.13857	6.23651	6.47879	6.50701	6.18063	5.75992	5.86776	5.78515	5.92784
ANKFY1	Q9P2R3-4	6.58093	6.69184	6.51043	6.47726	6.42885	6.38733	6.41013	6.19627	6.32606	6.3929	6.55741	6.13075	5.90287	5.57023	5.78915	5.6476
NUP210	Q8TEM1	6.50182	6.39046	5.76272	6.6561	6.25549	5.90915	6.24811	6.06541	6.073	5.92938	5.92876	5.97181	6.70448	6.49362	6.49652	6.61317
DNAJC7	Q99615	6.40455	6.16179	6.57416	6.14363	6.22285	6.53429	6.30544	6.24154	6.42325	6.56642	6.52877	6.40724	5.93794	5.91915	5.92406	5.70492
ANXA2	P07355-2	6.03514	6.39405	6.39005	6.14178	6.26987	6.42261	6.2639	6.0805	6.21087	6.34339	6.36875	6.36755	6.21564	6.21283	6.14056	6.14251
PRPF4B	Q13523	5.92962	6.311	5.52589	6.53135	5.93098	5.75379	6.17034	5.89854	5.81661	5.71691	5.44256	5.71246	6.48776	7.67166	7.21054	7.88999
GPATCH1	Q9BRR8	5.88768	6.24443	6.06245	6.2543	5.95209	6.12216	6.09203	5.95213	6.00796	6.06365	6.12024	6.07479	6.40684	6.98305	6.84185	6.93436
NOC4L	Q9BVI4	6.14403	6.25598	5.81974	6.79812	6.21567	5.98408	6.29331	6.24437	6.24589	5.98611	5.89474	6.19416	6.39241	6.23106	6.6315	6.66883
BCAP31	P51572-2	6.14155	6.14882	5.75676	6.56078	6.04743	5.67777	6.55867	5.93613	6.21316	5.69726	5.81096	5.974	6.56109	6.88713	6.97212	7.05639
ACTR3	P61158	6.06898	5.98835	6.77756	6.24268	6.25521	6.59759	6.36164	6.23828	6.24769	6.4083	6.4731	6.36616	6.00395	5.944	5.97155	6.05497
ZFC3H1	O60293	6.20113	6.19122	6.04975	6.84205	6.0143	6.00449	6.52762	6.04488	6.18024	6.206	6.09113	5.97111	6.34715	6.4426	6.31957	6.56676
RAP1B	P61224	6.02532	6.33421	5.84847	6.48838	6.37878	6.39414	6.39181	6.11468	6.20278	5.98376	5.92244	5.98059	6.45546	6.50862	6.50953	6.46102
UTP18	Q9Y5J1	6.13267	6.23352	5.95797	7.00891	6.4672	5.88043	6.66617	6.24041	6.19972	5.99261	6.03049	6.02053	6.28208	6.1442	6.38217	6.36093
CROCC	Q5TZA2	6.19798	5.99734	5.96774	5.77477	6.26214	6.21001	6.03773	6.40042	6.09726	6.11339	6.21064	6.09098	7.07114	6.49743	6.64191	6.42912
HDGF	P51858	5.87176	5.85571	6.20138	5.13435	5.94014	6.16358	5.54898	6.23882	6.19611	6.35087	6.43522	5.91913	7.06542	7.37483	7.12587	6.57783
BAG6	P46379-3	6.39436	6.11882	6.83686	5.69818	6.53462	6.41253	6.12903	6.55025	6.14557	6.75543	6.67546	6.83793	5.84138	5.58599	5.57404	5.90954
BYSL	Q13895	5.99345	5.99408	6.29253	6.19183	6.10034	5.91522	6.23565	6.11338	6.30554	6.00503	6.22448	5.87063	6.88209	6.19095	7.00904	6.67575
NRDE2	Q9H7Z3	5.90431	6.3659	6.032	6.38741	5.82658	5.79924	6.06636	5.57636	6.09881	6.00761	6.02373	6.13896	6.85743	6.96072	6.98423	6.97037
PLXNB2	O15031	6.45909	6.5452	6.02375	6.76389	5.99504	6.08634	6.13566	6.03358	6.23784	6.34624	6.30683	6.20272	6.31364	6.23834	6.23974	6.07211
ATP6V1A	P38606	6.64862	6.4819	6.75018	6.36216	6.28075	6.84725	5.94877	6.35992	6.61867	6.70891	6.78623	6.64871	5.39329	5.45083	5.43218	5.28162
CAPN2	P17655	6.08006	6.39847	6.70338	6.00452	6.6065	6.98524	6.37173	6.67823	6.36104	6.47219	6.65969	6.575	5.81837	5.91776	5.23926	5.12856
UTP6	Q9NYH9	6.24046	6.27409	5.9119	6.77363	6.45098	5.87329	6.40087	5.89827	6.2051	6.0487	5.8216	5.73571	6.82308	6.15645	6.81096	6.57489
NOL9	Q5SY16	6.21731	6.25746	5.86034	6.69342	6.09783	5.91811	6.44713	6.02253	6.27694	5.81175	6.10975	5.89692	6.68631	6.36027	6.68881	6.65511
RPS16	P62249	6.28955	6.60288	6.6223	6.34376	6.35758	6.60672	6.63145	6.33258	6.69221	6.51429	6.56842	6.409	5.59744	5.48655	5.62968	5.3156
ZW10	O43264	6.51615	6.56582	6.23487	6.55943	6.46724	6.44683	6.20716	6.50139	5.94032	6.28973	6.06462	6.23094	6.01765	6.07756	5.81545	6.06485
NIBAN1	Q9BZQ8	6.09549	6.31869	6.25917	5.87547	6.27406	6.26687	5.98458	6.48597	6.26666	6.21217	6.28873	6.33961	6.47172	6.30401	6.35609	6.20071
NUCB1	Q02818	7.79992	6.05475	6.13837	5.61574	6.99824	4.70372	5.63137	7.26687	5.43847	7.335	6.12563	7.63512	5.56413	6.05833	5.55124	6.0831
LTA4H	P09960	6.15519	5.99185	6.76969	5.59701	6.46155	6.93346	6.15936	6.67466	6.21591	6.43849	6.51707	6.48351	5.90112	6.24988	5.84353	5.60773
PLCG1	P19174-2	6.50538	6.06728	7.24192	6.06437	6.26366	6.84892	6.46664	6.36496	6.43123	6.70729	6.91495	6.44219	5.43181	5.45825	5.41511	5.37605
SH3GL1	Q99961	6.54887	6.25555	6.89025	6.13868	6.51375	6.76724	6.2024	6.47333	6.50612	6.25122	6.68515	6.6015	5.97825	5.65471	5.34519	5.18779
OSBP	P22059	6.15396	6.54959	6.70445	6.55474	6.15658	6.67417	6.41624	6.36531	6.65751	7.03822	7.06226	6.80413	5.13053	5.45985	4.95663	5.31583
CCDC88A	Q3V6T2	6.2361	6.28128	6.64181	5.98273	6.25642	6.52971	6.27819	6.55839	6.56432	6.45417	6.48218	6.59829	6.06079	5.83111	5.65444	5.59008
RASA1	P20936	6.14967	6.30981	6.67221	5.88029	6.27525	6.84238	6.19586	6.74943	6.30468	6.26812	6.29295	6.31677	5.9166	6.20931	5.84332	5.77336
UTP4	Q969X6	6.46978	6.41174	5.95957	6.78637	6.26415	5.89783	6.486	5.99451	6.35794	5.98291	5.99988	5.93851	6.42127	6.01117	6.52949	6.48889
ATF7IP	Q6VMQ6-4	6.76766	6.56821	6.25199	6.68143	6.47074	6.14233	6.03452	6.27969	6.15022	6.34436	6.22884	6.3479	6.21732	5.58547	5.79267	6.13664
CFL1	P23528	6.19113	6.08265	6.54395	5.65258	6.19986	6.53624	6.04778	6.67209	6.16933	6.40311	6.19146	6.40475	6.25559	6.22402	6.01742	6.40803
POGZ	Q7Z3K3	6.47041	6.73682	6.00513	6.80945	6.27472	6.04969	6.33405	5.80483	5.93509	6.24264	6.17905	6.18033	6.34538	6.13327	6.26898	6.23015
DAP3	P51398	6.57244	6.27908	5.95056	6.82612	6.6219	6.29362	6.65029	6.07446	6.25644	5.9335	5.99907	5.8811	6.42504	5.83066	6.25532	6.1504
AGL	P35573	6.04799	6.0302	6.57781	5.65687	6.30465	6.34263	6.11721	6.26374	6.17638	6.27669	6.55437	6.42604	6.38451	6.34415	6.27665	6.22012
CIP2A	Q8TCG1	6.10544	6.21639	6.89722	5.87068	6.15803	6.62691	6.3607	6.7624	6.53809	6.47095	6.73007	6.86347	5.5509	5.67168	5.39228	5.7848
DNAJA1	P31689	6.38124	6.26243	6.71155	6.60367	6.04927	6.56743	6.48462	6.58157	6.60603	6.32608	6.32283	6.57325	5.59854	5.8631	5.34359	5.7248
SBDS	Q9Y3A5	6.17838	6.2545	6.8349	5.91093	6.03764	6.56349	6.22806	6.31349	6.41642	6.39486	6.55831	6.39323	5.91946	6.04889	6.04693	5.9005
PDHB	P11177	6.08112	6.18185	5.52541	6.36926	6.63876	6.05618	6.54159	6.16158	6.2734	6.28324	6.39028	6.14497	6.61038	6.2294	6.354	6.29404
LYAR	Q9NX58	6.04642	6.21395	5.9894	6.51537	5.94204	5.59035	6.22631	5.69278	5.97859	5.80183	5.7959	5.88018	6.6259	7.242	7.12224	7.33675
TAGLN2	P37802-2	5.74767	4.70039	6.35903	4.64351	6.62184	5.62336	5.95423	7.33661	5.9142	6.74523	6.30915	7.53995	5.5126	7.33888	6.37195	7.28141
TRMT10C	Q7L0Y3	6.48393	6.38488	5.9242	6.50208	6.26229	6.04756	6.40257	6.00252	6.03429	5.95275	5.82296	5.72015	6.82317	6.40625	6.62747	6.60292
RPL9	P32969	6.45178	6.17252	6.59409	6.42894	6.21103	5.96604	6.40942	6.07618	6.96836	6.49436	6.31224	6.39681	5.71005	5.93723	5.69344	6.17749
LNPEP	Q9UIQ6	6.29131	6.54563	6.30342	6.60782	6.43506	6.14785	6.21362	6.09045	6.15452	6.42908	6.07656	5.91896	6.12857	6.09514	6.3805	6.18151
CALU	O43852-4	6.21213	6.15602	5.74719	6.42354	6.76227	6.25849	6.20541	6.36465	6.31815	5.85697	5.90177	6.02673	7.12292	6.22685	6.21619	6.20071
TAF2	Q6P1X5	6.21496	6.4014	6.19639	6.38213	6.09542	6.11278	6.37819	6.02721	6.08733	6.00861	6.11604	5.99902	6.01277	6.57182	6.69066	6.70526
RNH1	P13489	6.2216	5.86061	6.85058	5.66835	6.30525	6.42777	6.15266	6.54265	6.57612	6.50596	6.76496	6.70346	5.71739	5.83335	5.79923	6.07008
AFG3L2	Q9Y4W6	6.38371	6.58712	5.72728	6.84294	6.2755	5.85466	6.75159	6.05361	6.13369	5.75114	5.59435	5.86364	6.58482	6.36709	6.52273	6.70614
SGO2	Q562F6	6.38523	6.593	5.93809	6.66398	6.01298	5.91465	6.39476	6.05639	6.22074	6.07925	6.03104	5.90405	5.9262	6.70727	6.50948	6.6629
PSMD12	O00232	6.14883	6.18185	6.23127	6.23486	6.34946	6.53	6.36939	6.51851	5.98653	6.03609	6.31845	6.22943	6.17467	6.33978	6.26096	6.0899
CMAS	Q8NFW8	5.86016	6.11199	5.78537	6.45353	6.12776	5.91205	6.12312	5.89453	5.86023	5.99199	5.90165	5.93263	7.04479	6.85203	7.10719	7.04098
SWAP70	Q9UH65	6.23129	6.4087	6.82882	5.99278	6.05124	6.74965	6.07705	6.44283	6.43694	6.61936	6.28782	6.53884	5.83698	5.84645	5.82729	5.82397
HMGA1	P17096	5.92	5.88486	5.56409	5.78524	5.85226	5.72215	6.01509	5.78884	5.99924	5.89213	5.44696	5.37866	7.08513	7.95136	7.87042	7.84356
EIF5A	P63241-2	6.2882	5.94457	6.54023	5.46058	6.66819	6.70312	5.93892	7.27485	6.3904	6.4537	6.47623	6.66082	5.98153	5.91857	5.57045	5.72966
RPS24	P62847-4	6.00779	6.30511	6.03738	6.20151	5.97639	6.03457	6.85343	5.99033	7.10132	5.82626	6.29319	6.44377	5.49879	6.81594	6.11617	6.49804
ATAD5	Q96QE3	6.75751	6.84758	6.1278	7.18252	6.1531	6.01092	6.51387	5.93421	6.28531	6.14402	6.10194	6.35203	5.68451	5.86729	5.8439	6.19348

TIMELESS	Q9UNS1	5.81661	6.21285	6.30013	5.6625	5.93852	6.15221	6.13028	6.2144	6.33829	6.21582	6.55966	6.11774	6.5695	6.47639	6.59079	6.70432
COPG2	Q9UBF2	6.4206	6.5913	6.54599	6.12523	6.42663	6.53236	6.1826	6.3259	6.18838	6.48081	6.63905	6.20131	5.98207	6.02852	5.72025	5.60902
POP1	Q99575	6.10295	6.42534	6.04766	6.56047	6.16571	6.18873	6.34081	6.01641	5.90527	6.02478	6.65483	5.93627	6.40169	6.46896	6.74812	6.61201
WIZ	O95785	6.31541	6.03838	5.97489	6.75391	6.25427	6.02049	6.31252	6.09149	6.27496	6.00706	6.13432	6.16416	6.58447	6.37494	6.37268	6.32604
CLASP2	O75122-3	6.29971	6.0594	6.44111	6.14451	6.24555	6.3418	6.05771	6.5065	6.4685	6.34317	6.42561	6.36953	6.20212	6.19992	5.89281	6.00205
SMARCD1	Q96GM5	6.45692	6.6773	6.24821	6.58837	6.22034	5.93439	6.20937	6.11999	6.06704	6.03686	6.28851	6.10546	6.44614	6.3792	6.24507	5.97682
RBM17	Q96I25	6.36185	6.23967	6.01509	6.74471	6.23997	6.08933	6.23819	6.06674	6.22808	6.08119	6.19449	6.14827	6.66632	5.9606	6.47916	6.24636
VPS35L	Q7Z3J2	6.01057	6.10943	6.3154	5.77748	6.15718	6.30148	6.03156	6.01193	6.08208	6.54431	6.20985	6.2264	6.51657	6.66893	6.66252	6.37429
TUBB1	Q9H4B7	6.54252	6.23606	7.10035	6.0706	6.41148	6.87995	6.27678	6.54865	6.47536	6.41032	6.82922	6.98374	5.69765	5.34965	4.94024	5.24743
UPF2	Q9HAU5	6.04558	6.57456	6.72561	6.29523	5.87093	6.51391	6.13838	6.26594	6.39938	6.57889	6.28121	6.66014	5.92902	6.02668	5.94547	5.74907
BAG3	O95817	6.62702	5.74913	6.86484	5.38635	6.4582	6.61939	5.82609	6.94566	6.18952	6.5452	6.98542	6.69167	6.33626	5.93825	5.39831	5.43869
GPRIN1	Q7Z2K8	6.50656	6.43775	6.12672	6.34572	6.39919	5.9759	6.23092	6.2369	6.53255	6.09488	6.1569	6.23184	6.32566	6.02279	6.07753	6.29821
RPL32	P62910	5.86115	6.38811	5.70591	5.983	6.12907	6.26749	6.63301	6.43066	7.12531	6.10129	6.23179	6.14199	6.03321	6.46701	5.92318	5.57782
RB1CC1	Q8TDY2	6.18179	6.07422	6.35095	6.00875	6.3681	6.53693	6.168	6.05648	6.23112	6.19657	6.42473	6.1974	6.43592	6.27782	6.37603	6.11519
DBT	P11182	6.09604	6.16158	5.86526	6.32978	6.3207	6.08039	6.43553	5.86561	6.26248	6.09405	6.08637	6.06345	6.80754	6.38588	6.64468	6.50066
PPWD1	Q96BP3	5.85382	6.085	6.21744	5.84238	5.84293	5.95894	5.94153	5.83219	6.38711	6.1208	6.32216	5.94228	6.86973	6.66198	7.26332	6.85839
STXBP3	O00186	6.38992	6.54496	5.9548	6.39298	6.2383	6.229	6.42193	6.07536	6.20676	6.26429	6.10847	6.00765	6.41592	6.2348	6.30974	6.20509
ITPR3	Q14573	6.46435	6.40188	5.84513	6.65753	6.12531	5.92761	6.35988	5.82869	6.13445	6.21687	5.90512	6.01237	6.63415	6.35695	6.69453	6.43517
KIFC1	Q9BW19	5.95106	6.22095	5.94594	6.10487	6.13882	6.19088	6.23151	5.92166	6.1011	6.20643	6.14229	5.8972	6.69053	6.65655	6.79993	6.80029
RPS8	P62241	6.10922	6.22036	6.57057	5.87328	6.49034	6.41537	6.02414	6.40079	6.88147	6.45055	6.39384	6.32809	5.64064	6.28999	6.12997	5.78139
PMPCB	O75439	6.38624	6.3483	5.75317	6.33285	6.59349	6.14024	6.30069	6.17664	6.14561	6.08857	6.03292	5.9296	6.74743	6.23944	6.45661	6.32819
APP	P05067	6.64862	6.80168	6.25446	6.83871	6.54538	6.46924	6.45732	6.13754	6.26792	6.40328	6.33529	6.31416	5.64939	5.5516	5.62028	5.70511
EHD2	Q9NZN4	5.99478	6.1747	6.34136	6.29025	6.18318	6.6645	6.18458	6.54069	6.4061	6.43919	6.84668	6.79499	6.16736	6.02529	5.4686	5.47776
TASOR2	Q5VWN6	6.57806	6.38535	6.14668	6.97072	6.32051	6.19473	6.41327	6.14959	6.12069	5.82395	5.87963	6.03259	6.38767	5.85107	6.31234	6.43315
SPATA5	Q8NB90	6.40703	6.24799	6.6148	6.08185	6.44222	6.51508	6.06384	6.32593	6.48796	6.29241	6.5083	6.31747	6.10877	5.8756	5.99583	5.71492
TARS2	Q9BW92	6.30081	6.9104	5.68335	6.77087	6.34429	6.26936	6.24538	6.58973	5.9415	5.97172	6.06738	6.16716	6.12732	6.1466	6.26002	6.2041
MED24	O75448	6.25317	6.52897	5.8965	6.45008	6.28879	6.10337	6.21533	6.33539	6.06958	6.19658	5.90254	5.90371	6.54481	6.44791	6.42782	6.43546
SEC24B	O95487-3	6.14649	6.18738	6.47386	6.35276	6.28115	6.50105	6.14801	6.40982	6.26016	6.25587	6.58429	6.22282	5.96907	6.12045	5.99455	6.09226
HIP1R	O75146	5.95167	6.42455	6.42861	6.42702	6.0573	6.25381	6.35102	6.41347	6.1207	6.61294	6.45794	6.49294	5.82178	6.15924	6.07079	5.95621
LEMD2	Q8NC56	6.35188	6.27197	5.83704	6.3707	6.16545	6.21671	6.06914	6.2211	6.24689	6.31541	6.01628	6.36493	6.95625	6.33319	6.15815	6.10494
NPLOC4	Q8TAT6-2	6.22233	6.49424	6.12061	6.04464	6.48324	6.34191	6.03863	6.24378	6.17505	6.23212	6.16963	5.94988	6.63374	6.23882	6.32604	6.28535
TBL2	Q9Y4P3	6.31667	6.52485	6.07789	6.57647	6.07982	6.02921	6.47622	5.78685	6.02217	5.98192	5.97266	6.06771	6.40248	6.35839	6.70771	6.61899
GNS	P15586	6.45058	6.54285	5.86331	6.46074	6.36722	6.4681	6.24804	6.29867	6.13769	6.19121	6.31618	6.09163	6.49178	6.10044	6.14879	5.82277
CPT2	P23786	6.37904	6.35839	5.84336	6.70024	6.28838	5.96411	6.12073	6.00375	6.04757	6.14549	5.95024	6.24424	6.75309	6.43229	6.45273	6.31635
PAF1	Q8N7H5	6.10494	6.39834	5.9319	6.48685	6.11925	6.29478	6.2939	6.07342	6.37103	6.30611	6.11824	5.99858	6.47215	6.2014	6.31962	6.50949
KANK2	Q63ZY3	6.24548	5.90536	5.96682	5.45768	6.31824	5.99196	6.04451	6.04547	6.7169	6.32804	6.27327	6.16706	7.20012	6.54885	6.57879	6.21144
SLTM	Q9NWH9	6.17706	6.27867	6.03141	6.63817	6.0074	5.73069	6.33762	5.87985	5.97171	6.03085	5.92153	5.77772	6.45711	6.77696	7.09654	6.8867
SKIV2L	Q15477	6.22765	6.37339	6.70611	6.09082	6.38212	6.84861	6.25599	6.82592	6.34949	6.36862	6.57607	6.46043	5.69384	5.83521	5.57499	5.43074
AP1M1	Q9BXS5-2	6.21754	6.56154	6.85625	6.5358	6.08025	6.52726	6.33624	6.6222	6.10993	6.45791	6.23868	6.36049	5.64519	5.99929	5.64108	5.81035
ZZEF1	O43149	6.12054	6.32792	6.54105	6.08755	6.05694	6.62063	6.19383	6.4584	6.36653	6.4697	6.66267	6.45774	5.9893	5.91483	5.72863	6.00374
GSS	P48637	6.15462	6.0009	6.90483	5.53773	6.36263	6.84148	6.20914	6.86019	6.20807	6.53569	6.57979	6.33334	6.01277	6.00573	5.99327	5.45981
MLH1	P40692	5.60681	5.87908	6.13841	5.55502	5.98119	6.27504	6.20543	5.92747	6.01992	6.38886	6.37998	6.23387	6.8032	6.84791	7.05063	6.70718
KHDRBS1	Q07666	6.33383	6.44066	5.9205	6.60497	6.39627	6.14997	6.24055	6.16597	6.25742	6.02878	6.3146	6.11209	6.60795	6.04177	6.38609	5.99858
UBE4A	Q14139-2	6.21365	6.1311	6.16027	6.51026	6.18955	6.19465	6.03118	6.00493	6.01533	6.30281	5.98071	6.15045	6.63068	6.2744	6.58676	6.36327
MCM3AP	O60318	6.33145	5.9164	5.85846	6.77761	6.17261	6.05531	6.49403	5.70782	6.10129	6.17668	6.18364	6.23419	6.61978	6.27652	6.41021	6.68399
TSFM	P43897	6.31842	6.27186	6.03325	6.58055	6.38659	6.03853	6.28635	5.88606	6.38074	6.00936	5.71597	5.66203	6.75136	6.11096	6.89503	6.67293
HP1BP3	Q5SSJ5	6.25443	6.45697	5.37286	6.11938	6.15632	5.74391	6.32057	5.97362	5.71018	5.93616	5.6455	5.53888	6.52494	7.61275	7.24325	7.39029
FANCI	Q9NVI1	5.69425	6.12038	5.8688	5.86282	6.16851	6.22147	6.30606	6.38134	6.20239	6.21409	6.15872	6.24908	6.50792	6.50653	6.71277	6.82486
HNRNPH3	P31942	6.10369	6.14255	5.89586	6.28617	6.34051	6.09692	6.17215	6.2643	6.30915	6.43246	6.43031	6.38666	6.48486	6.03686	6.14768	6.46987
RBM22	Q9NW64	6.40226	6.2839	6.08733	6.56278	6.10565	5.91177	6.17843	5.92194	6.2932	6.24147	6.17615	6.00559	6.34962	6.35904	6.62642	6.49447
MRPL13	Q9BYD1	6.34777	6.51824	5.92234	6.69379	6.65343	6.16908	6.44161	6.21914	6.13313	6.12793	6.03555	5.95321	6.61508	5.95176	6.22144	5.9965
DLAT	P10515	6.37172	6.32315	5.88561	6.45784	6.43604	6.11014	6.33558	6.08498	6.15251	5.92108	5.79969	6.05186	6.64937	6.41218	6.63728	6.37095
NOL8	Q76FK4	6.11696	6.4251	6.00732	6.81348	5.95944	5.91859	6.39851	5.85054	6.08269	6.04931	5.78348	6.09744	6.48976	6.41009	6.75267	6.84461
ACOX3	O15254	6.25868	6.28342	6.13613	6.66237	6.36903	5.91802	6.45281	5.88463	6.04141	6.25265	6.07912	5.91791	6.65484	6.03823	6.67276	6.37801
PAPOLA	P51003	5.3237	5.78153	6.16204	5.31855	5.44857	5.95691	5.83533	5.87274	5.96916	6.13782	6.29794	6.18095	7.08823	7.38499	7.90553	7.33599
PSMA3	P25788	5.96407	6.3696	6.36523	6.03511	6.44259	6.14104	6.41426	6.25221	6.17674	5.97334	6.03733	5.92401	6.6578	6.35577	6.46979	6.42114
CPNE8	Q86YQ8	6.32218	6.4784	6.06009	6.54082	6.52041	6.40863	6.46519	6.38843	6.36061	5.98641	6.24059	6.19445	5.91173	6.1741	5.69038	6.25759
EPG5	Q9HCE0	6.33239	6.22206	6.96779	6.39459	6.23762	6.50121	6.30786	6.38328	6.53736	6.33045	6.4835	6.51694	5.58849	5.78404	5.73128	5.68117
NAMPT	P43490	6.2842	6.19002	6.88659	6.10598	6.29571	6.67151	6.06271	6.85452	6.20483	6.18286	6.16914	6.38121	5.57562	6.27097	6.02297	5.74118
ADAM9	Q13443	6.18506	6.20969	6.16898	6.30781	6.16989	5.98118	6.54054	6.09579	6.01538	6.04667	6.0569	6.11061	6.46324	6.53178	6.53723	6.55464
HNRNPA3	P51991	6.55091	6.44036	5.84654	6.93954	6.33003	6.16233	6.18354	6.09577	6.0987	5.83488	6.00492	6.02999	6.53274	6.11801	6.35604	6.47571
PPP2CB	P62714	5.86771	5.89856	6.74966	5.69194	6.20703	6.55969	6.5029	6.40774	6.39593	6.46369	6.74514	6.67773	5.84684	5.90731	6.12841	5.94973
SORL1	Q92673	6.49018	6.4026	6.62431	6.6576	6.43029	6.36612	6.63405	6.16646	6.62381	6.78445	6.70299	6.27616	5.39365	5.57146	5.3466	5.52928
RPS11	P62280	6.43001	6.42094	6.47222	6.25314	6.56343	6.44011	6.33979	6.28973	6.55344	6.54221	6.50428	6.22019	5.80658	5.90874	5.68243	5.57276
CBX1	P83916	6.17789	6.46571	5.82034	6.66569	6.11113	6.43514	5.82118	6.1915	6.00351	6.04163	5.9413	6.33633	6.54861	6.55287	6.15534	6.73183
SRSF4	Q08170	6.26605	6.36687	5.93692	6.49455	6.21125	5.98131	6.29111	5.92891	6.23419	6.25517	5.90795	5.9168	6.69054	6.26026	6.52022	6.73791
EIF3G	O75821	6.29427	6.24177	6.77773	6.26845	6.38074	6.65421	6.33699	6.61447	6.58199	6.40007	6.70679	6.40924	5.5699	5.70078	5.44736	5.61525
SYNJ2	O15056	6.1996	6.10643	6.63311	6.07297	6.23246	6.58782	6.10289	6.61505	6.56413	6.72036	6.56796	6.57436	5.66603	6.30593	5.58073	5.47018
AKAP8L	Q9ULX6	6.02137	6.15988	6.07604	6.5518	6.23398	6.24369	6.12271	5.83756	6.21862	6.20296	6.09439	5.98831	6.78374	6.37644	6.57723	6.5113
TBC1D5	Q92609-2	6.3505	6.33463	6.659	6.20852	6.41234	6.52435	6.27663	6.40579	6.34381	6.45102	6.71	6.59229	5.83428	5.77248	5.45275	5.67162
STAT3	P40763-3	6.18181	6.11703	6.6561	5.84696	5.99567	6.52752	6.12078	6.42513	6.35109	6.57374	6.54807	6.54842	5.92469	6.16089	6.04645	5.97563
SLC25A24	Q6NUK1	6.47644	6.57518	5.58477	6.53225	6.59265	6.16356	6.42779	6.25535	5.86183	5.93075	5.54671	5.77719	6.50287	6.59365	6.42372	6.75529
ETF1	P62495	6.31117	6.08517	6.94124	6.09044	6.57427	6.49234	6.19765	6.48769	6.70714	6.36001	6.49695	6.35936	5.77835	5.58308	5.79203	5.74312
UNC45A	Q9H3U1	6.16295	6.25645	6.92913	6.22363	6.25613	6.53478	6.34275	6.53644	6.73766	6.52868	6.51139	6.63665	5.55725	5.76356	5.36638	5.65616
MAGED2	Q9UNF1	5.99379	6.04706	6.36793	5.60264	6.08793	6.49443	6.39684	6.37562	6.26079	6.32628	6.41933	6.03303	6.4395	6.42265	6.46866	6.26352
RUFY1	Q96T51	6.25186	6.20842	6.82933	5.8839	6.49856	6.41202	6.26542	6.34115	6.70104	6.08841	6.60026	6.43029	6.16839	5.87547	5.74008	5.70541
RAB3GAP1	Q15042	6.05192	6.14103	6.60555	6.01225	5.98463	6.30115	6.20746	6.33847	6.22043	6.46405	6.57258	6.57458	5.99591	6.26288	6.15411	6.113
AP2A2	O94973-2	6.06312	6.23925	6.0185	6.27207	5.95868	6.3151	6.07012	5.98402	6.33995	6.49637	6.19021	6.12686	6.33398	6.3039	6.80821	6.47966
BMS1	Q14692	6.21048	6.36717	5.65787	6.70324	6.26477	5.92873	6.46889	5.88429	6.23045	5.92381	5.70704	5.76232	6.62683	6.6575	6.62559	6.98102
HGS	O14964	6.52253	6.31885	6.47877	6.17719	6.55548	6.77243	6.25715	6.57057	6.26506	6.54515	6.62458	6.46013	6.01886	5.65714	5.35559	5.42052
LAMB3	Q13751	6.66694	6.44378	6.16648	6.61348	6.81442	6.20646	6.57881	6.34999	6.19204	5.91015	6.00234	6.27798	6.3533	5.6857	5.80535	5.93279
SPATS2L	Q9NUQ6-3	5.8306	6.28055	6.05587	6.05329	5.89571	6.45719	6.10659	6.60885	6.31217	6.71407	6.48133	6.80607	6.02321	6.22887	5.99536	6.15027
IK	Q13123	6.26282	6.41745	5.91897	6.62133	6.06113	5.96939	6.33782	5.83399	6.05145	6.07247	6.10773	6.00935	6.62639	6.36122	6.5762	6.77228
RBM4	Q9BWF3	6.40119	6.32181	6.18751	6.51871	6.45538	5.87423	6.34242	6.31505	6.23245	5.97359	6.48802	6.31952	6.47264	5.74017	6.20831	6.14899
FARP1	Q9Y4F1-2	6.53321	6.51337	6.07381	6.78791	6.04142	5.99019	6.29269	5.97345	6.18826	6.17326	6.32099	6.11637	6.22412	6.31077	6.2933	6.16688
SMARCAL1	Q9NZC9	5.27581	5.88452	6.09337	5.39512	5.94058	6.35231	5.91544	5.79754	5.81981	6.43356	6.26459	6.055	7.13024	7.43401	7.1319	7.07619
RHPN2	Q8IUC4	6.74399	6.55314	7.05941	6.36206	6.66083	6.67505	6.15853	6.7862	6.36931	6.57563	6.39973	6.51702	5.40618	5.3325	5.10032	5.30011
PPA1	Q15181	6.20455	6.1845	6.78422	5.91101	6.7716	6.91145	5.9031	7.37065	6.6662	6.5778	6.72696	6.72794	5.39367	5.39521	5.33282	5.13833
ACOT7	O00154	5.81656	5.76034	6.0734	6.04702	6.32054	6.4205	6.74648	6.25409	6.40883	5.8099	6.82955	6.47701	6.14818	6.51344	6.1243	6.24986
DSG2	Q14126	6.42964	6.52245	6.16093	6.66473	6.3762	6.2678	6.26769	6.38106	6.38535	6.18604	6.13717	6.17361	6.13353	5.77596	6.09586	6.04199
H0YHG0	H0YHG0	6.42384	5.86981	5.98637	6.3257	6.46276	6.17293	6.33164	5.69368	6.1781	6.24512	5.74966	5.93799	6.98336	6.44744	6.6001	6.5915
PFKL	P17858-2	6.10209	6.32624	6.59005	6.02052	6.34803	6.79176	6.61546	6.73239	6.58545	6.21242	5.94694	6.134	5.49893	6.20539	5.78082	6.10953
COTL1	Q14019	6.30427	5.87856	6.8186	5.79008	6.38652	6.565	6.5074	6.48123	6.8727	6.17689	6.46451	6.51268	5.65095	5.9976	5.71117	5.88186
SEC23IP	Q9Y6Y8	6.11978	6.32456	6.78202	5.81559	6.1857	6.76219	6.19604	6.59516	6.57607	6.43226	6.52063	6.50886	5.88358	5.86162	5.79054	5.64539
SERPINB6	P35237	6.17265	6.26461	6.69596	5.72066	6.36254	7.07968	6.15947	6.91209	6.04607	6.70854	6.13621	6.31344	5.76563	6.32604	5.74648	5.58992
RPS18	P62269	6.52197	6.36348	6.68561	6.44331	6.46124	6.34456	6.51752	6.39582	6.54317	6.24899	6.38872	6.29908	5.67486	5.87378	5.51346	5.72444
KIF15	Q9NS87	6.50483	6.46715	7.04651	6.48271	6.46593	6.67348	6.25478	6.75129	6.59753	6.73276	6.79571	6.84998	5.05697	5.31492	4.97329	5.03216
SEC24C	P53992	6.07953	6.10464	6.42647	6.1176	6.24652	6.78025	6.19628	6.03153	6.04818	6.19854	6.21641	6.19493	6.4459	6.30197	6.37992	6.23134
CAT	P04040	6.39823	6.12912	5.72182	6.13805	6.44537	6.39823	6.1428	6.24009	6.13521	6.08514	6.10258	6.09468	6.83102	6.22628	6.55024	6.36114
CAMSAP1	Q5T5Y3-3	6.5841	6.47584	6.98008	6.14095	6.08388	6.37173	6.28007	6.45671	6.30303	6.27653	6.31546	6.38644	5.76413	6.00235	5.83506	5.74364
KDM5C	P41229	5.81743	6.33143	6.11505	6.23859	6.37091	6.38076	6.24153	6.42172	6.24025	6.27631	6.50072	6.46489	6.09816	6.07462	6.27194	6.15569
RACGAP1	Q9H0H5	6.66407	6.32035	6.44152	6.89512	5.96627	6.15372	6.332	6.01677	6.45433	6.40068	6.57242	6.3003	6.88015	5.74136	5.79034	6.0706
ZFHX4	Q86UP3-5	6.14337	6.51614	6.21144	6.80177	6.33054	6.12951	6.46117	6.10067	6.29831	6.21501	6.32938	6.3972	6.22552	5.75354	5.90546	6.18098
IGF2BP3	O00425	6.37197	6.25003	6.26136	6.16948	6.51122	6.35911	6.32835	6.20278	6.49484	6.52902	6.17164	6.14976	6.03171	6.09623	6.05368	6.01884
BUB1B	O60566-3	6.61849	6.39845	7.39045	6.49885	6.41278	6.52931	6.59634	6.48673	7.09538	6.46638	6.79676	6.98352	5.03688	4.97121	4.71507	5.00341

HIP1	O00291	6.40581	6.34245	6.04011	6.08196	6.51673	6.19956	6.33447	6.17321	6.3012	6.37375	6.08763	6.05884	6.39838	6.28151	6.29808	6.10633
PHF14	A0A0U1RRH6	6.59788	6.69737	6.31946	6.87332	5.72417	5.99532	6.27936	6.00714	6.24015	6.44511	6.31359	6.39433	5.99276	6.05548	6.06258	6.00199
COG1	Q8WTW3	6.3722	6.32972	6.43986	6.53012	6.2312	6.42169	6.46492	6.34971	6.38094	6.36462	6.3054	6.17715	5.97493	6.19233	5.82412	5.64107
SCPEP1	Q9HB40	6.31903	5.84386	5.86003	5.76192	6.56083	5.81576	6.34017	6.34152	5.91936	6.27346	5.79931	6.17157	6.45747	6.90666	6.80941	6.81965
GPATCH8	Q9UKJ3	6.27387	6.39962	6.24822	6.2445	5.82145	5.99692	6.10565	5.73157	5.84114	5.98509	5.92518	5.6869	6.37535	7.12599	6.99541	7.24313
SPECC1L	Q69YQ0	6.76723	6.45322	6.51325	6.26079	6.20157	6.22549	6.32858	5.85921	6.61879	6.22371	6.30596	6.5038	6.35203	6.10378	5.51538	5.76722
DHX37	Q8IY37	6.05204	6.43753	6.27465	6.90359	6.13617	5.86617	6.39108	5.94007	6.31179	6.31444	6.06748	6.27592	6.02743	6.04025	6.71969	6.24169
ECHS1	P30084	6.43755	6.29893	5.71776	6.28464	6.54543	6.00111	6.41978	6.11343	6.11186	6.01459	5.98176	5.96384	6.78444	6.5201	6.48726	6.31753
CPVL	Q9H3G5	6.3662	6.32979	5.79364	6.09022	6.3831	6.27248	6.39176	6.35831	6.0644	6.16758	6.06385	5.93654	6.63116	6.43384	6.50931	6.20783
PXDN	Q92626	7.33546	7.11652	5.93587	7.53517	6.37047	6.17254	6.62329	6.07128	6.5859	6.41904	6.33852	6.52216	5.15296	5.08728	5.15392	5.57963
RAB1A	P62820	6.03031	6.03844	6.15103	6.17849	6.11079	6.09979	6.45471	6.21289	6.32255	6.03154	6.20549	6.01173	6.27647	6.66882	6.71777	6.48916
YTHDC2	Q9H6S0	6.32325	6.32841	6.55044	6.28844	6.15786	6.30273	6.20224	6.29068	6.45543	6.60265	6.56331	6.56631	5.89826	5.87544	5.82103	5.77351
HADH	Q16836-2	5.87036	6.20252	5.75127	6.5048	6.20097	5.98393	6.28469	6.03152	6.17618	6.14126	6.12521	6.05038	6.59642	6.54402	6.71927	6.81717
HSPG2	P98160	6.84616	7.0479	6.38118	6.9241	6.34475	6.62967	6.65887	6.23121	6.71161	6.93574	6.88098	6.65337	4.95925	5.06397	4.81036	4.92088
JMJD1C	Q15652	6.10171	6.61249	6.13766	6.7295	6.10539	6.30356	6.22925	6.22428	6.11825	6.17893	5.99634	5.9333	6.2537	6.34038	6.44749	6.28775
WRNIP1	Q96S55	5.99717	6.21405	6.09936	5.91606	6.1525	6.05097	6.00471	5.96422	6.07052	6.11875	6.09761	5.94903	7.01466	6.57721	7.139	6.63418
URB1	O60287	6.38876	6.40796	5.7861	7.01501	6.21675	5.8581	6.341	6.16237	6.38972	5.90359	6.09448	6.24176	6.24566	6.06105	6.39783	6.48987
CDCA2	Q69YH5	6.94232	7.01963	6.54968	7.49303	6.20732	5.98506	6.61356	6.11191	6.46767	6.54058	6.45578	6.58552	4.90356	5.30326	5.40188	5.41925
PHGDH	O43175	6.33156	6.0032	6.69152	5.67624	6.50578	6.82396	5.94732	6.66268	6.42201	6.5102	6.55859	6.42109	6.248	6.01994	5.64904	5.52885
FXR2	P51116	6.27545	6.51916	6.49871	6.24983	6.26137	6.28226	6.20754	6.36797	6.25028	6.4473	6.71658	6.37033	5.80405	6.13384	5.83549	5.77983
TNPO2	O14787	5.82653	5.6797	6.2927	5.7621	5.9279	6.23505	5.86379	6.31997	6.42558	6.23771	6.3684	6.49879	6.52486	6.3646	6.80421	6.86811
MRPL39	Q9NYK5	6.40795	6.3489	5.80676	6.54373	6.56928	6.36498	6.40605	6.38917	6.05354	6.08109	5.90766	5.86331	6.47893	6.19252	6.29668	6.28945
CNP	P09543	6.26598	6.46057	6.00735	6.26408	6.20059	5.94317	6.31311	5.94367	6.29361	6.42184	6.10686	6.1989	6.51256	6.35264	6.4713	6.24375
VPS39	Q96JC1	6.28396	6.67684	6.37027	6.03076	6.06215	6.53089	6.05454	6.26932	6.27721	6.53208	6.37364	6.22098	5.93917	6.39298	6.10744	5.87776
ADAM10	O14672	6.36089	6.50123	6.00492	6.5819	6.16695	6.25246	6.48989	5.9535	5.98787	6.1542	6.11639	5.95395	6.48315	6.09794	6.38684	6.50793
FANCD2	Q9BXW9-1	6.02198	6.15438	6.13258	6.05876	5.96844	6.03268	6.18435	5.9073	6.54093	6.38527	6.22087	6.44831	6.49753	6.03848	6.77287	6.63526
LARP7	Q4G0J3-3	5.77022	6.54567	5.87524	6.1469	6.14126	5.98485	6.05426	5.80638	5.78568	6.08448	6.03158	5.80169	6.58511	7.20983	7.25988	6.91697
ARHGAP1	Q07960	6.57525	6.16877	6.71846	5.68327	6.5544	6.4148	6.1819	6.44196	6.56004	6.51569	6.55031	6.28469	6.21691	5.71931	5.803	5.61123
CPSF6	Q16630-2	6.23975	6.47523	5.947	6.3192	6.30295	5.99087	5.99389	5.88998	6.22922	6.28725	6.02589	5.98926	6.76707	6.54012	6.53608	6.46625
PTPN13	Q12923-4	6.36585	6.11187	6.19073	6.60731	6.25797	6.18533	6.33121	5.85141	6.39584	6.36241	6.20674	6.25425	6.44927	6.09782	6.25786	6.07413
COX4I1	P13073	6.38878	6.33544	5.67703	6.59683	6.58824	6.07045	6.60127	6.10987	6.19515	5.97795	5.81728	5.95651	6.63253	6.13064	6.44363	6.4784
TBRG4	Q969Z0	6.30187	6.35561	5.92464	6.86172	6.25453	6.30972	6.25482	6.21301	6.0133	5.96518	6.12618	6.15053	6.25392	6.14458	6.42459	6.44582
RPL24	P83731	6.52434	6.32601	6.39675	5.94413	6.274	6.39128	6.3389	6.2732	6.39533	6.4854	6.37742	6.35905	5.87557	6.16873	5.91821	5.95166
DDX39A	O00148	6.39281	6.37799	6.09147	6.95207	6.44049	5.96003	6.25596	5.87597	6.22071	6.12293	6.17012	6.04819	6.39908	6.06953	6.44769	6.17496
ECE1	P42892	6.4253	6.32404	6.11835	6.27047	6.12758	6.24336	6.10817	6.22528	5.99669	6.59202	6.42913	6.37952	6.27365	6.43341	6.18204	5.87099
RPTOR	Q8N122	6.15993	6.51571	6.65378	6.35025	6.07899	6.23945	6.14199	6.39105	6.50355	6.47253	6.4333	6.69792	5.79519	5.65557	6.01906	5.89176
NCOA5	Q9HCD5	6.32183	6.25243	5.74655	6.75827	6.00062	5.984	6.66445	5.97089	6.33175	5.76776	5.72997	6.11654	6.30658	6.5178	6.5255	7.00508
RBMX	P38159-2	6.31868	6.31941	5.88067	6.82217	5.99299	5.97926	6.37764	6.02694	6.24679	6.21699	6.09465	6.22173	6.55698	6.22615	6.3553	6.36765
PRPF4	O43172	5.65734	5.97997	6.02311	6.4985	6.42441	6.25151	6.33721	5.79637	6.20905	5.91671	6.49473	6.10879	6.56686	6.40047	6.70491	6.63006
WDR36	Q8NI36	6.17824	6.41774	5.79203	6.82595	6.2977	6.00489	6.34702	6.26214	5.99772	5.84452	5.87712	5.99214	6.49099	6.21513	6.72223	6.73444
SUCLA2	Q9P2R7	6.58547	6.32342	6.07243	6.43857	6.48994	5.8725	6.28224	5.98694	6.33553	5.96512	6.00837	5.83042	6.79376	6.08053	6.61346	6.3279
MYO18A	Q92614	6.18058	6.18547	6.44612	6.14889	6.24063	6.19145	6.17323	5.92543	6.58034	6.58946	6.08588	5.90422	6.46221	6.30563	6.22727	6.35318
CEP192	Q8TEP8	6.55435	6.4745	6.90581	6.65	6.21577	6.55133	6.21681	6.55057	6.66857	6.62808	7.03308	7.02068	5.14013	5.28357	4.92421	5.18254
AATF	Q9NY61	6.31178	6.27163	6.00986	6.89783	6.28026	5.8356	6.25942	6.02508	6.11747	5.99308	6.13696	6.20341	6.37148	5.94645	6.56493	6.77475
TACC3	Q9Y6A5	6.92546	6.18705	7.12564	5.69537	6.49132	6.6544	5.80362	6.7327	6.86402	6.52158	7.05068	7.15087	5.67145	5.23455	4.83364	5.05765
HMMR	O75330-3	6.26527	6.24574	6.7614	5.93192	6.22553	6.37593	6.47102	6.37248	6.97748	6.69774	6.65619	6.67183	5.60733	5.66791	5.52077	5.55144
BCL2L2	Q92843-2	6.69869	6.82613	5.93984	6.58398	6.68456	6.27423	6.31981	6.21464	6.22504	6.1909	5.77919	5.62759	6.39526	5.90261	6.22579	6.11173
RTF1	Q92541	5.25632	5.86479	5.83128	5.15897	5.67757	6.02253	5.5837	6.00647	5.91209	6.22745	6.18164	5.86828	7.47836	7.87929	7.7097	7.34158
ABCC1	P33527	6.41139	6.60651	6.01699	6.72144	6.11996	5.9837	6.23238	5.94406	6.32022	6.17407	5.98636	6.08384	6.32733	6.1601	6.44332	6.46834
GNAS	P63092-3	6.33051	6.58029	6.17383	6.49084	6.45468	6.30747	6.36659	6.29892	6.46354	6.24708	5.91328	6.15167	5.95	6.16589	6.00125	6.10417
RICTOR	Q6R327-3	6.42388	6.18263	6.58682	6.05624	6.35671	6.82075	6.30133	6.6045	6.34834	6.63735	6.41545	6.33933	5.86227	5.82853	5.56599	5.66987
VPS11	A0A087WXL6	6.12429	6.34018	6.49579	5.9009	6.10092	6.40731	6.26633	6.30933	6.34237	6.40852	6.49052	6.10986	5.93366	6.12733	6.615	6.0277
SLC4A1AP	Q9BWU0	5.478	5.91081	5.99801	5.74494	6.01106	6.15567	5.93503	5.89021	6.2771	6.12092	6.28483	6.39325	7.08406	6.6512	6.79484	7.27006
ARHGEF10	O15013	6.39826	6.26841	6.86095	6.57766	6.19359	6.70676	6.36242	6.42441	6.28277	6.32614	6.40713	6.6907	5.54101	5.81614	5.36067	5.78299
IDH3A	P50213	6.18398	5.8046	5.43777	6.05449	6.44709	5.71011	6.68889	6.40484	6.19243	5.83981	5.62184	5.58978	6.47019	6.79859	7.00187	7.35909
CAPN1	P07384	6.39066	6.50364	6.68694	5.83905	6.36594	6.74218	6.2366	6.66643	6.41014	6.62426	6.30262	6.35527	5.68959	5.84134	5.67918	5.66616
TBCB	Q99426	6.39157	6.05423	6.72373	5.97465	6.31822	6.41339	6.21695	6.78743	6.44482	6.28091	6.24247	6.73469	5.56239	6.10995	5.69077	6.05385
DHX36	Q9H2U1	6.33114	6.29822	6.65998	6.22603	6.24568	6.26671	6.32081	6.34473	6.48078	6.27999	6.55935	6.41584	6.02938	6.01813	5.85683	5.66642
DHX29	Q7Z478	6.40617	6.45704	6.73371	6.2637	6.37427	6.55408	6.31124	6.20067	6.53388	6.45513	6.50764	6.20049	5.72579	6.00441	5.72637	5.54541
LIG1	P18858	5.20412	5.41313	5.9281	5.15185	5.29954	5.76274	5.93086	5.8231	6.3761	5.95524	6.40487	6.04373	7.77415	7.3672	8.06057	7.5047
MPRIP	Q6WCQ1-2	5.88034	6.26649	5.9083	5.81199	6.27411	5.92661	5.87267	5.99808	6.33082	6.32794	6.53852	6.21162	6.84132	6.6407	6.57587	6.59463
PKP4	Q99569	6.48745	6.97982	6.1853	6.98382	6.21788	6.22135	6.30129	6.52313	6.08148	5.84561	6.09473	5.98927	6.17022	6.10439	6.02612	5.78814
KIF2A	O00139-4	5.79235	6.25325	6.15647	5.89902	6.23321	6.47107	6.17908	6.60082	6.40184	6.5646	6.23397	6.5271	6.31046	6.42218	6.2873	6.26229
PRPF31	Q8WWY3	6.17084	6.14744	5.87369	6.29392	6.08007	5.97347	6.36524	5.91425	6.12919	5.9164	5.776	5.99003	6.80398	6.81884	6.87328	6.87334
SBNO1	A3KN83	5.4635	6.12209	6.47424	5.56572	5.58871	6.23027	5.75713	6.04293	6.13337	6.39362	6.67097	6.30871	6.31558	6.75286	7.14367	7.03662
RNMT	O43148-2	5.97708	6.22782	5.84158	5.89552	6.26409	5.9749	6.3404	6.05891	6.18909	6.00008	5.67425	5.68978	7.01376	6.90869	6.94842	6.99565
HDAC2	Q92769	6.45422	6.61352	6.0446	6.72875	6.39315	6.33999	6.2992	6.30261	6.30493	6.05866	5.9247	5.99492	6.42445	5.88794	6.24581	5.98255
RBM12	Q9NTZ6	5.57987	5.76186	5.96657	5.31398	5.97646	6.342	5.57622	6.10945	6.05586	6.24985	6.30432	6.12017	7.44796	7.17883	7.1095	6.90711
FBN1	P35555	6.18916	6.36218	5.63465	6.54836	6.42609	6.49966	6.76698	6.44954	5.9568	6.38505	6.31997	5.93897	6.38765	6.03927	5.93662	6.1618
PDPR	Q8NCN5	6.39489	6.25909	5.8315	6.39512	6.38849	6.02564	6.50082	6.13343	6.10193	6.1691	6.09974	5.77044	6.6117	6.24478	6.64568	6.42767
LPP	Q93052	6.4437	5.97137	6.92296	5.61949	6.48682	6.59691	5.83902	6.74523	6.55436	6.29929	6.66155	6.50745	6.56361	5.74209	5.52004	5.52612
ATG7	O95352	6.3096	6.03695	6.99382	6.02066	6.21766	6.64031	6.24193	6.51317	6.6045	6.43701	6.60027	6.53022	5.78675	5.75953	5.79114	5.51651
UBR7	Q8N806	5.59557	5.67601	6.43029	5.60125	5.85932	6.44756	5.73586	6.29188	6.1195	6.27404	6.71384	6.33282	6.55999	7.16065	6.66142	6.53999
BRIX1	Q8TDN6	6.21477	6.02862	5.87231	6.61521	6.21779	5.65908	6.23371	5.90139	6.17005	5.5255	5.76118	5.88419	6.77115	6.82644	7.09055	7.22807
LEMD3	Q9Y2U8	6.33693	6.36145	6.07402	6.75337	6.34944	6.0693	6.30283	5.7745	6.13274	6.13357	5.99052	6.03722	6.67968	6.19599	6.40614	6.40231
CDK5RAP3	Q96JB5-4	6.43135	6.37328	6.12457	6.28666	6.29644	6.12907	6.09746	6.21032	6.24516	6.17256	5.93541	5.86185	6.78363	6.11962	6.4859	6.44674
TMX3	Q96JJ7	6.39533	6.27435	5.92374	6.4659	6.25539	5.87242	6.40557	5.91821	6.23942	5.94299	5.87178	5.84016	6.81877	6.13198	6.95527	6.68872
PLEKHA5	Q9HAU0-6	6.3299	6.44416	6.23778	6.02755	6.13252	6.14126	6.29831	6.39714	6.31119	6.45024	6.14542	6.16971	6.4579	6.24092	6.18559	6.03041
ERP29	P30040	6.09975	5.78086	5.78402	5.51991	6.77879	5.8664	5.99078	6.59983	5.97277	6.47743	6.30096	6.3981	6.98057	6.4206	6.65457	6.37465
CYB5R3	P00387-3	6.27636	6.44956	5.97844	6.62667	6.48496	6.25334	6.20648	6.19498	6.02129	5.90593	6.1474	6.03209	6.41104	6.49853	6.44432	6.11363
CAPZA2	P47755	6.21899	6.17273	6.6489	6.29116	6.40324	6.60777	6.09548	6.17396	6.55127	6.29499	6.46587	6.09308	6.17818	5.86791	6.08613	5.85035
TMED10	P49755	6.47939	6.79989	5.64747	6.43585	6.78617	6.50736	6.11565	6.32685	6.03689	5.83024	5.79704	5.75182	6.62489	6.4489	6.38247	6.0291
PACS1	Q6VY07	6.50633	6.30595	6.47143	6.39065	6.22569	6.66613	6.02298	6.34441	6.20651	6.4728	6.57776	6.484	5.65318	6.32145	5.84012	5.51062
RBM19	Q9Y4C8	6.18287	6.42721	5.99378	6.94437	6.16633	6.0255	6.44212	5.87751	6.22065	5.97423	5.8014	5.952	6.35924	6.26663	6.7074	6.65877
TRMT1	Q9NXH9	5.24525	5.55798	6.17624	5.68025	5.81664	6.16664	6.12178	5.95465	5.96847	6.1091	6.24346	5.93744	7.04293	7.06608	7.83189	7.0812
SNRPD2	P62316	6.11567	6.21872	5.92624	6.71637	6.09707	5.94737	6.93789	5.59841	6.27324	5.61107	5.68006	5.80221	6.42326	6.65474	6.79382	7.20386
POFUT1	Q9H488	6.40364	6.16093	5.95054	6.57037	6.46383	5.92624	6.34126	6.19888	6.13786	5.97684	5.89864	5.90618	6.79057	6.28341	6.53046	6.46033
METAP1	P53582	6.54777	6.32296	6.55828	6.20491	6.62028	6.54941	6.3708	6.76043	6.51496	6.18079	6.44253	6.41483	5.892	5.39029	5.55581	5.67394
PSME1	Q06323	6.12452	5.23124	6.46754	4.68463	6.48454	6.06446	5.48929	7.06715	5.6711	6.67701	6.72159	6.98417	6.80896	6.71916	6.36454	6.44011
RPS13	P62277	6.26868	6.47172	6.35421	6.24145	6.1708	6.38739	6.06433	6.48875	6.41924	6.46975	6.47975	6.54016	5.77735	6.16588	5.76184	5.9387
WDR82	Q6UXN9	6.31751	6.59482	6.24396	6.68691	6.25841	6.44748	6.24439	6.11948	6.40604	6.2886	6.19488	6.11875	6.2685	5.74554	6.07754	5.98719
POLR3A	O14802	6.17142	6.42772	6.47402	6.24521	6.02243	6.15648	6.13243	6.15487	6.3491	6.27787	6.36778	6.59144	6.1869	6.16998	6.05048	6.22187
RPL17	P18621-3	6.27294	6.47345	6.60711	6.11328	6.23775	6.24638	6.42033	6.10094	6.60873	6.15616	6.50178	6.56335	5.84337	6.19768	5.73696	5.91978
WARS1	P23381	6.27833	6.09663	6.75083	5.75511	6.66122	6.64549	6.3453	6.40455	6.62631	6.32406	6.63665	6.414	6.02546	5.63607	5.71458	5.68542
SPTLC1	O15269	6.313	6.35595	5.98719	6.63373	6.33963	6.08377	6.37769	6.0632	5.87691	5.93778	5.92719	6.01699	6.47323	6.46922	6.59	6.55451
NSD1	Q96L73	6.58246	6.84483	6.28956	7.4756	6.04666	5.79947	6.28325	6.238	6.38418	6.03908	6.58536	6.42726	5.32539	5.82605	5.94389	5.90896
QRSL1	Q9H0R6	6.30188	6.37832	5.9934	6.5545	6.52595	6.29716	6.42555	6.1498	6.41306	6.04889	6.09907	6.04407	6.61989	5.6888	6.3243	6.13536
LTBP4	Q8N2S1	7.09947	6.36106	4.8848	7.07842	5.47824	5.92525	7.29811	6.22697	7.0706	5.9278	5.69811	6.0908	6.74257	5.7371	6.2172	6.16349
MAN2A1	Q16706	6.35814	6.29275	6.22725	6.8006	6.16428	6.0536	6.51972	5.82108	6.10011	6.14863	6.30624	6.18544	6.47077	6.0566	6.32127	6.17351
POLR1E	Q9GZS1	6.33187	6.45027	5.85268	7.01257	5.97101	5.88576	6.70791	5.80558	6.28442	5.63547	6.01423	6.04638	6.42349	6.07019	6.56526	6.94292
SCAF8	Q9UPN6-2	6.53781	6.51982	5.90803	6.40692	6.3404	6.09802	6.36224	5.92589	6.09345	6.00469	5.84093	5.97607	6.78267	6.28502	6.56352	6.35451
RRP1	P56182	6.48745	6.55288	5.37755	6.66375	6.23552	5.65194	6.4721	6.12903	6.45852	5.60377	5.83683	5.99834	6.66466	6.79656	6.43594	7.53516
TACC1	O75410-2	6.59031	6.38876	6.18823	6.6491	6.38158	6.15911	6.206	6.17998	6.13817	5.94021	6.34258	6.13221	6.63425	5.87345	6.07602	6.12004
SYNE3	Q6ZMZ3	6.44969	6.37988	5.69655	6.38163	6.07453	5.91476	6.28319	5.93651	5.94225	6.11597	5.75865	5.55641	6.97665	6.85379	6.93425	6.74529
NAPA	P54920	6.3849	6.03375	6.1434	5.87076	6.20051	6.1272	6.23655	6.37254	5.92815	6.19376	6.26832	6.28494	6.32572	6.1321	6.85916	6.63823

DLGAP5	Q15398	6.18709	6.11781	6.94245	6.20651	6.34902	6.42685	6.61748	6.92965	6.4847	6.3499	6.30291	6.78477	5.65493	5.59154	5.37343	5.68093
MAP7D3	Q8IWC1	6.48286	6.43956	6.42992	6.63157	6.13481	6.35612	6.16716	6.45808	6.26876	6.19532	6.56009	6.62456	5.69693	5.82694	5.70704	6.02027
TRA2B	P62995	6.4419	6.22759	5.78744	6.4476	6.51103	5.91326	6.45954	6.09738	6.40292	5.96246	5.9477	5.70015	6.99282	6.17934	6.49405	6.43483
CEP350	Q5VT06	6.27481	6.16464	6.38663	6.22873	6.10465	6.38918	6.24658	6.27918	6.42231	6.70445	6.35563	6.44431	5.88346	6.04561	6.07747	5.99235
SGPL1	O95470	6.28554	6.50542	5.99621	6.62144	6.11985	6.00025	6.29726	5.82389	6.05157	6.15361	5.79751	5.64794	6.72399	6.44999	6.9154	6.55012
ARPC1B	O15143	6.01609	6.09826	6.5118	6.05194	6.4149	6.32538	6.31321	6.37475	6.29934	6.4126	6.26098	6.35096	6.16498	6.14677	6.21199	6.04605
SEC23B	Q15437	6.14942	6.17078	6.6398	6.15388	6.20534	6.57617	6.15451	6.36114	6.39091	6.20156	6.40445	6.37935	5.95273	6.21842	6.34434	5.69718
EWSR1	Q01844-5	6.22805	6.34421	5.97045	6.36017	6.37241	6.17078	6.03123	6.30614	6.16728	6.07072	6.00699	5.81692	6.69894	6.55785	6.35651	6.54132
ENO2	P09104	6.12966	6.00237	6.91628	5.70645	6.63029	6.98425	6.06892	6.88899	6.29435	6.71291	6.69479	6.52182	5.6037	5.72605	5.64081	5.47833
WBP11	Q9Y2W2	6.28099	6.56552	6.24465	6.09041	6.08114	6.23486	6.38832	6.11384	6.11712	6.15864	5.82894	5.75139	6.67755	6.46266	6.76003	6.24393
SHPRH	Q149N8	6.22774	6.57842	6.01182	6.80522	6.32926	6.22633	6.43679	5.9871	6.40113	6.08106	6.09886	6.15705	6.16178	6.03312	6.19232	6.27201
SAFB2	Q14151	6.40175	6.25649	5.90001	6.65959	6.24594	5.85938	6.4622	5.95507	6.28183	5.98874	6.05632	5.86235	6.63468	6.26082	6.75846	6.41635
FKBP8	Q14318	6.44447	6.42898	6.12601	6.73933	6.39778	6.05352	6.45606	6.00161	6.1265	6.13736	6.14621	6.01654	6.26766	5.9792	6.53107	6.14769
EEF1B2	P24534	6.80112	6.18922	6.7644	5.47876	6.70659	6.21148	5.83476	6.76318	6.3308	6.48528	6.73391	6.60984	6.43561	5.76113	5.40504	5.4889
KDM5A	P29375	6.43676	6.69515	6.32842	7.10382	6.32156	6.08632	6.73008	5.77358	6.10489	6.18043	5.98687	6.22112	5.98508	5.86058	6.14229	6.04305
MTIF2	P46199	6.38513	6.40993	6.08924	6.6947	6.25862	6.04445	6.3821	6.04627	6.07499	6.00179	6.10593	5.82196	6.66238	6.16441	6.55866	6.29945
MRPS9	P82933	6.5443	6.37613	5.92441	6.5903	6.24989	6.01891	6.33869	6.07069	6.19845	6.04078	6.1335	6.07411	6.43162	6.12709	6.49806	6.38308
FIP1L1	Q6UN15	6.18485	6.26839	5.81057	6.76021	6.23094	5.96898	6.30762	5.86304	6.19298	5.79378	5.59649	5.84789	6.60249	6.63259	6.7592	7.17998
ABCF3	Q9NUQ8	6.50243	6.48823	6.90301	6.3403	6.18673	6.71438	6.35109	6.71679	6.63982	6.58348	6.89402	6.86986	5.15335	5.39976	4.8902	5.36655
STRBP	Q96SI9	6.49723	6.49454	6.04124	7.08926	6.29062	6.00579	6.5238	5.78605	6.2656	6.18473	6.19959	5.96595	6.19395	6.05459	6.13503	6.27204
PPP4R3B	Q5MIZ7	5.84066	6.11122	6.19041	5.9942	6.31232	6.14692	5.99463	6.01314	6.27549	6.2243	6.10279	6.03441	6.7008	6.56985	6.68485	6.804
PPP4R3A	Q6IN85	5.8491	6.08033	5.95473	5.83552	6.41864	6.28531	6.21524	5.91756	5.96105	6.02854	5.84298	5.58782	7.07134	6.7172	7.38621	6.84842
PML	P29590	6.35185	6.37227	5.98841	6.16571	6.24589	5.82517	6.02069	6.0025	6.24764	6.06302	6.43521	6.20815	6.95587	6.33322	6.58269	6.20172
MYEF2	Q9P2K5	6.61316	6.55776	5.93201	7.00372	6.47005	5.93868	6.25063	6.01397	6.21367	5.95186	6.27492	5.80068	6.66282	5.88664	6.29511	6.13431
CCDC86	Q9H6F5	5.8085	5.98312	5.66431	6.58738	5.82835	5.60713	6.24248	5.8335	5.86096	5.69012	5.81488	5.94279	7.08081	7.02386	7.56602	7.46578
QSOX2	Q6ZRP7	6.39173	6.28796	5.95745	6.75606	6.3674	6.06352	6.38128	6.25298	6.36099	6.02666	6.00328	6.12561	6.38169	6.01237	6.36743	6.26358
NSDHL	Q15738	6.31227	6.39332	5.80743	6.4294	6.43992	6.0661	6.43575	6.15308	5.95595	5.88074	5.97389	5.83208	6.66972	6.52714	6.56487	6.55835
BAIAP2	I3L4C2	6.4331	6.50137	6.53006	6.14599	6.65396	6.25035	6.57716	6.71093	6.62662	6.57253	6.35954	6.17056	5.99518	5.55174	5.58131	5.33963
PIKFYVE	Q9Y2I7	6.25073	6.29743	6.6412	6.46579	6.15468	6.37362	6.05235	6.29122	6.34686	6.27343	6.50903	6.16772	6.06965	6.05386	6.11216	5.94027
BCAM	P50895	6.5022	6.44498	6.27132	6.62197	6.10107	6.18703	6.34796	6.04893	6.24379	5.96318	6.0237	6.0873	6.54492	6.2382	6.21273	6.1607
DHX40	Q8IX18	5.81894	6.30158	6.13819	6.29901	6.03229	6.22932	6.27494	5.91496	6.24511	6.40935	6.2475	6.29294	6.38011	6.25389	6.61564	6.54625
NEK9	Q8TD19	6.39067	6.21893	6.72885	5.94942	6.19762	6.80119	6.13344	6.61163	6.28079	6.44245	6.38546	6.72331	5.78408	5.89246	5.73544	5.72426
VAC14	Q08AM6	6.15209	5.93022	6.44435	6.20036	6.10837	6.34025	6.11569	6.29992	5.98117	6.55549	6.09813	6.07948	6.5061	6.46438	6.58368	6.14033
MRC2	Q9UBG0	6.10203	6.46456	6.15545	6.28704	6.21215	6.47506	6.00623	6.0212	5.87002	6.35443	6.39407	6.20672	6.15669	6.49392	6.39341	6.40702
CLIC1	O00299	6.28788	5.93564	7.08552	5.57544	6.50533	6.75133	6.0932	6.53855	6.31503	6.57075	6.64107	6.6673	6.04313	5.76735	5.57326	5.64922
ACTL6A	O96019	6.06072	6.1552	6.07801	6.56135	6.30007	6.20402	6.39592	6.29574	6.38746	6.13054	6.22177	6.33755	6.08321	6.13625	6.17737	6.47482
EBNA1BP2	Q99848	6.11531	6.31758	5.66597	6.58416	6.03159	5.82046	6.36757	5.92241	5.80353	5.83608	5.78283	5.91652	6.66938	7.02272	7.09028	7.05362
DNAJC10	Q8IXB1	6.14917	6.31165	5.92329	6.32667	6.08188	6.06686	6.11702	6.01157	5.98355	6.00591	5.99501	6.12183	6.87977	6.66811	6.82866	6.52904
ERP44	Q9BS26	6.30501	6.04269	6.01139	6.17596	6.21452	6.00982	6.18818	6.19758	6.25677	5.91796	6.06785	6.127	6.7036	6.2872	6.57962	6.54484
AGO2	Q9UKV8	6.41597	6.24534	6.64273	6.38361	6.47407	6.46497	6.00788	6.15927	6.41845	6.35704	6.53446	6.33251	6.03081	5.94201	5.95546	5.63542
NMT1	P30419	6.23263	6.51949	6.59198	5.91629	6.28978	6.6489	6.20338	6.29829	6.39243	6.56472	6.47362	6.52908	5.61398	6.29661	5.72514	5.70369
CORO1B	Q9BR76	6.27735	6.50202	6.25698	5.9045	6.64225	6.46146	6.10211	6.29287	6.44845	6.229	6.21124	5.99362	6.41591	6.24512	6.04817	5.96894
DOCK5	Q9H7D0	6.09267	6.15459	6.77397	6.03067	6.32651	6.6118	6.15152	6.47206	6.42752	6.50331	6.78343	6.54478	5.73927	5.92152	5.66714	5.79924
PLBD1	Q6P4A8	6.39313	6.45344	6.07378	6.42913	6.11008	5.97735	6.46752	6.01753	6.29196	6.35566	6.19801	6.27556	6.11209	6.28495	6.3075	6.25353
TCF25	Q9BQ70	6.28062	6.32222	6.52218	6.43905	6.18824	6.43502	6.24312	6.2389	6.34472	6.11609	6.39205	6.22019	5.89746	6.06282	6.20015	6.09717
DOCK1	Q14185	6.23504	6.23302	6.90766	6.09667	6.32589	6.64575	6.15125	6.5007	6.39539	6.49645	6.40655	6.25552	6.00342	6.01594	5.79423	5.53653
PPIE	Q9UNP9	6.46583	6.31963	5.96561	6.33807	6.47161	6.34968	6.27463	6.55522	6.34954	6.06273	6.4966	6.16285	6.60638	5.83175	5.8272	5.92267
DDX47	Q9H0S4	6.22551	6.24693	5.85473	6.65615	6.42671	5.99906	6.58734	6.04928	6.18977	6.10355	5.8577	5.84646	6.61886	6.15269	6.64357	6.5417
PSMB5	P28074	6.22319	6.76446	6.29669	6.13398	6.38062	6.5306	6.14915	6.41488	6.10669	6.27227	6.07961	5.83682	6.2747	5.94852	6.66236	5.92545
SMC5	Q8IY18	6.36596	6.54806	5.92502	6.53435	6.11216	5.94901	6.39032	6.02586	6.20053	5.91018	5.93396	6.13957	6.51199	6.39256	6.49728	6.56319
PICALM	Q13492	5.96145	6.12698	6.57056	6.36227	5.85378	6.0853	6.4188	6.17059	6.1127	6.18269	6.42395	6.57193	6.11393	6.23691	6.35781	6.45035
CPSF3	Q9UKF6	6.34923	6.26292	5.8232	6.67257	6.23184	6.12743	6.63408	6.0862	6.34502	5.90425	5.78068	6.07589	6.26777	6.31382	6.44194	6.68318
FNDC3B	Q53EP0	6.0843	6.28831	6.06988	6.46768	6.34992	6.11004	6.6059	6.12748	6.04615	5.8365	6.0871	5.93343	6.72358	6.20033	6.57307	6.49631
SIPA1	Q96FS4	6.21862	6.59866	5.90326	6.33617	6.10802	6.32267	6.1261	6.26808	6.21958	6.28919	5.96001	6.35639	6.05673	6.44843	6.388	6.40011
HACL1	Q9UJ83	6.26163	5.95641	5.91432	6.36586	6.69336	6.2407	6.37423	6.25123	6.07349	6.09982	6.01447	6.03437	6.77075	6.22455	6.31956	6.42522
SRI	P30626	5.73289	4.95812	5.73704	4.77178	5.33697	5.09759	5.56321	6.51642	4.93469	5.43565	5.02082	5.59835	4.93186	11.2322	8.43947	10.693
CUL2	Q13617-2	5.95697	6.07916	6.46634	5.91685	6.13773	6.5695	6.10742	5.71994	6.21966	6.62523	6.34986	6.01973	6.31309	6.39247	6.54591	6.58017
RBM27	Q9P2N5	6.00769	6.29155	5.82749	6.22452	6.31327	6.13824	6.0704	6.15333	6.06041	6.11513	5.84859	5.91288	6.84265	6.62096	6.78865	6.78424
DIAPH3	Q9NSV4	6.53678	6.24577	7.14062	6.29788	6.00254	6.41629	6.52951	6.23702	6.68952	6.30734	6.86164	7.1486	5.48237	5.37773	5.19852	5.52787
U2AF2	P26368	5.93099	6.16856	5.75838	6.48896	6.30887	6.07098	6.56513	6.0995	6.08632	5.91445	5.82746	5.73016	6.63595	6.554	6.93362	6.92657
PSMD6	Q15008	6.12075	6.08758	6.37382	6.09331	6.39223	6.36672	6.33044	5.91659	6.10827	5.87618	6.16578	6.10936	6.52659	6.58657	6.41315	6.53265
NOP14	P78316	6.07294	6.26931	5.53445	6.68694	6.31218	5.54759	6.52816	6.35994	6.51604	5.73129	5.84405	5.77033	6.71779	6.54416	6.56818	6.99664
RBM6	P78332	6.52205	6.5495	6.34041	6.93659	6.03398	5.85229	6.03492	5.89456	6.47455	5.96763	6.06924	6.32236	5.99202	6.1811	6.2711	6.5577
LRWD1	Q9UFC0	5.92678	6.04325	6.34806	6.40542	6.16114	5.83141	6.05967	6.13528	6.27533	6.19768	6.28137	6.46059	6.24687	6.16741	6.76712	6.69262
DRG2	P55039	6.30909	6.22463	6.89885	6.28235	6.35279	6.87871	6.43025	6.52095	6.21296	6.6699	6.75069	6.51056	5.62048	5.5351	5.42637	5.37633
TBC1D4	O60343	6.44855	6.37026	7.039	6.13091	6.23914	6.58132	6.10797	6.56477	6.38142	6.19578	6.62256	6.52733	5.91936	5.62702	5.51828	5.72635
AIMP1	Q12904-2	6.15947	6.07226	6.63958	5.91588	6.35117	6.48744	6.08089	6.37684	6.27135	6.40058	6.75879	6.71014	6.44102	5.94204	5.86948	5.52311
NDC80	O14777	6.67137	6.92095	6.90341	6.68042	6.48744	6.96714	6.53551	6.85268	6.62829	6.80062	6.68966	6.86104	4.76387	4.96054	4.45009	4.82698
RBM10	P98175-5	6.37127	6.34204	5.96846	6.44428	6.40821	6.1146	6.47909	5.95476	6.12007	6.12423	5.85993	5.77228	6.78162	6.18354	6.69561	6.34401
DCTN2	Q13561	6.63479	5.08733	6.2374	4.60018	6.35739	5.40702	5.91587	7.415	5.57392	6.53284	5.88089	7.45591	6.08086	7.15949	6.6028	7.05832
ECI2	O75521	6.307	6.25052	5.75389	6.48897	6.30351	6.30662	6.2482	5.9669	6.14946	6.2081	6.05108	5.95296	7.04894	6.13164	6.44806	6.38414
MRPL3	P09001	6.16982	6.50514	5.98865	7.22844	6.55083	6.29003	6.31675	6.19889	5.94351	6.1699	5.80566	6.02134	5.83149	6.13771	6.17046	6.67138
YME1L1	Q96TA2	6.56934	6.65534	6.05798	7.00404	6.08478	5.85998	6.2536	5.76311	6.23061	6.18799	6.33255	6.21554	6.10815	5.89086	6.46994	6.31619
MCMBP	Q9BTE3-2	5.74147	5.83628	6.22285	5.39753	6.25479	6.41032	5.81348	6.31833	6.05329	6.02353	6.26366	6.28512	6.92771	6.8637	6.7219	6.86602
EIF2AK4	Q9P2K8	6.41624	6.42733	6.81706	5.97332	6.21119	6.55557	6.23767	6.32711	6.43386	6.55439	6.67948	6.50569	5.83002	5.81018	5.6748	5.54609
TUT7	Q5VYS8	5.99995	6.12611	6.68204	6.04507	6.14771	6.49127	6.45421	6.21604	6.32298	6.60007	6.56825	6.56432	5.86852	6.24464	5.82553	5.84329
PCF11	O94913	6.16326	6.46685	5.90682	6.67279	5.98297	6.10217	6.26479	6.21475	6.08594	6.13339	6.15935	5.95191	6.14208	6.4881	6.68883	6.57602
EIF4H	Q15056	6.62805	6.78221	7.21761	6.16129	6.52865	7.08832	5.9607	6.6435	6.63839	6.49516	7.01073	6.96863	5.32209	5.14712	4.6304	4.77715
KDM1A	O60341	6.07607	6.36658	6.2456	6.11158	6.28315	5.96237	6.57239	5.9772	6.34673	5.99231	6.25409	5.88647	6.45943	6.20869	6.75858	6.49875
CCN1	O00622	6.14664	4.42339	3.23571	5.87571	4.16359	5.02784	8.58902	4.93768	5.74614	4.648	4.05643	4.87041	10.9011	6.83742	10.2553	10.2856
COBLL1	Q53SF7-2	5.76918	5.79215	5.97662	5.79969	6.08945	6.24707	5.97084	5.52856	6.11709	6.32278	6.35734	6.49227	7.26714	6.71514	6.75855	6.79612
RPL13A	P40429	6.06736	6.38651	5.98644	5.95242	5.97272	6.28454	6.47832	6.45823	6.83263	6.50651	6.40105	6.33138	5.8678	6.25712	5.92467	6.2923
CUL1	Q13616	5.6828	5.84104	6.03948	5.67259	6.11483	6.30673	6.17564	5.94957	6.11394	6.32349	6.15769	5.96658	6.88005	6.74771	7.24418	6.78367
ACTR2	P61160	6.17013	5.9871	6.66779	6.16391	6.23933	6.45757	6.44246	6.5035	6.35832	6.05449	6.31853	6.51749	5.8658	6.14612	5.93027	6.17719
USP4	Q13107	6.05368	6.05247	6.77724	6.02948	6.03158	6.59141	6.13848	6.53538	6.26265	6.51707	6.5731	6.67825	5.84556	6.15151	5.81703	5.94512
ARMC8	Q8IUR7	6.3288	6.52899	6.3554	6.14425	6.30961	6.14849	6.03276	6.15435	6.12606	6.21284	6.17662	5.97992	6.61106	6.353	6.5985	6.53935
VPS13A	Q96RL7	6.26069	6.49261	6.17133	6.26522	6.01236	6.06726	6.1773	5.80183	6.39906	6.46112	6.11662	6.16644	6.36296	6.43313	6.462	6.35006
CBX5	P45973	5.92196	6.14414	5.75686	6.45774	6.27716	5.92495	6.42538	5.8578	6.20092	5.92559	5.99029	5.91191	6.82431	6.67982	6.71309	6.98808
PCYOX1	Q9UHG3	6.25491	6.26291	5.70922	6.39809	6.29345	5.71812	6.5311	5.98923	6.12266	5.96847	5.81552	5.82542	6.77852	6.45799	7.2247	6.64969
NFS1	Q9Y697	6.30253	6.37054	6.01348	6.64442	6.40797	5.92387	6.48041	5.95063	6.19028	6.20719	6.15367	6.03785	6.55525	6.08549	6.39224	6.28418
UBE3C	Q15386	6.14009	6.36103	6.36206	6.32939	6.30762	6.44809	6.25898	6.36882	6.13421	6.43204	6.05482	6.16662	6.104	6.09796	6.2034	6.23088
CHERP	Q8IWX8	6.47141	6.46291	6.03553	6.72439	6.325	6.16032	6.16096	5.92849	6.18277	5.97724	6.12712	6.12938	6.62408	5.94797	6.17417	6.56826
ZBTB10	Q96DT7	6.21642	6.65794	6.44039	6.78031	6.22266	6.18664	6.36022	5.99703	6.10997	6.31263	6.13436	6.11838	6.22032	6.00879	6.081	6.15294
NOC3L	Q8WTT2	6.18434	6.25261	5.44946	6.70617	6.03283	5.79362	6.54558	6.14177	6.25255	5.94736	5.68289	5.75192	6.76199	6.56991	6.67293	7.25407
COL12A1	Q99715	6.28238	6.19027	6.02232	6.82	6.41129	6.17931	6.41973	6.21748	6.36295	6.07583	6.18468	6.35901	6.14163	5.78019	6.27846	6.27447
VPS33A	Q96AX1	6.59796	6.59647	6.56377	6.42083	6.25726	6.30199	6.41629	6.35097	6.49742	6.655	6.55402	6.31011	5.68382	5.75012	5.48351	5.56047
PSAP	P07602-3	6.82371	6.00332	5.84643	5.86922	6.12975	5.51345	5.70845	6.08368	5.59221	5.81303	5.68567	5.81652	6.22966	8.10155	6.69148	8.09188
EPHX1	P07099	6.38584	6.42451	5.92666	6.36987	6.11369	5.72465	6.35313	5.78704	6.08428	6.01976	6.18816	5.83878	6.67599	6.59042	6.87957	6.63766
COMT	P21964	6.04029	6.22114	5.92214	6.26675	6.28441	6.24062	6.15098	6.13015	5.86729	6.20183	5.88577	6.15753	6.75822	6.70795	6.59512	6.5698
FAM3C	Q92520	6.55032	6.33753	5.71372	6.26436	6.46692	6.27047	6.44406	6.22088	6.07007	5.93783	5.82234	5.82581	6.94689	6.23602	6.36586	6.52691
COL6A1	P12109	6.66074	6.71948	5.92028	6.57457	6.64002	6.1976	6.42414	6.18263	6.04838	6.36486	6.17503	6.16549	5.98518	5.87793	5.88306	6.18061
CNDP2	Q96KP4	6.11019	5.93389	6.93968	5.82472	6.49596	6.84779	6.06541	6.70337	6.29726	6.46031	6.74172	6.63544	5.91969	5.92674	5.63301	5.46483
GGH	Q92820	6.54741	6.3933	6.18978	6.51301	6.48252	6.11303	6.37797	6.29188	6.16789	6.01889	6.06686	5.87252	6.41784	6.07474	6.27324	6.19911
LARS2	Q15031	6.33733	6.42262	5.94488	6.68884	6.35943	6.06525	6.4312	6.14716	5.9201	6.18462	5.91603	5.81235	6.6611	6.21211	6.5558	6.34119
ABCB1	P08183	6.11105	6.53635	6.04737	6.37647	5.79332	5.71209	6.11128	5.79136	6.59694	6.63114	6.38953	6.38258	6.30613	6.23487	6.53948	6.44004

IMPA1	P29218-3	6.21611	6.23767	6.8676	5.65616	6.49167	6.96007	6.30926	6.93423	6.37664	6.41893	6.39312	6.2524	5.85977	5.87564	5.67302	5.47771
NELFE	P18615	5.60154	5.93035	6.07756	5.73982	6.0058	6.1419	6.3926	6.11961	5.96484	6.17845	6.20432	5.99423	6.89875	7.00584	6.91296	6.83143
INCENP	Q9NQS7	6.1475	6.49582	5.73292	6.87903	6.32432	5.88629	6.41859	5.92537	6.09662	6.11299	6.07391	5.93373	6.28311	6.31146	6.42465	6.95369
RBM42	Q9BTD8	6.03402	6.26339	5.97765	6.45728	6.05244	5.92033	6.2574	6.18772	6.32336	6.25398	6.23397	6.12761	6.49629	6.3994	6.48623	6.52894
PSMD4	P55036	6.26657	6.29616	6.45023	6.15943	6.36924	6.44551	6.00661	6.29749	6.37317	6.40241	6.45412	6.18136	6.33924	5.92382	5.94067	6.09397
MRPL11	Q9Y3B7	6.57671	6.54892	5.93697	6.51598	6.39612	5.77606	6.4917	6.11834	6.21798	6.01004	5.85356	5.94064	6.57266	6.2066	6.41601	6.4217
IRF2BPL	Q9H1B7	6.26917	6.00361	6.1149	5.74707	6.50845	6.25195	5.92205	6.1514	6.0655	5.97526	5.84623	5.89913	7.52263	6.48498	6.6094	6.62826
ADK	P55263	6.12783	6.07525	6.83134	5.75944	6.39443	6.84462	6.1554	6.5294	6.26659	6.53588	6.53635	6.4099	5.83245	6.06881	6.05313	5.5792
WDR70	Q9NW82	5.63551	5.9635	6.19538	5.77226	5.91387	6.36948	5.64547	5.93638	6.22664	6.26054	6.08408	6.12568	6.49447	7.20704	7.17345	6.99624
GNPDA1	P46926	6.1831	6.12362	6.93013	6.01019	6.21094	6.7534	6.24512	6.29249	6.4155	6.50439	6.57635	6.40003	6.11621	5.79104	5.94745	5.50004
ASPSCR1	Q9BZE9-2	5.77213	5.8006	6.76078	5.56626	5.93003	6.2767	5.76708	6.04801	6.20292	6.19227	6.4119	6.14778	7.11407	6.45958	6.8308	6.71909
TAF15	Q92804	6.37074	6.35454	5.69462	6.38877	6.3968	5.94136	6.34446	6.0779	6.24703	5.98811	5.82322	5.96362	6.88045	6.51951	6.35788	6.65101
CEP250	Q9BV73	6.08641	6.30644	6.59113	6.0107	6.02697	6.47053	6.08149	6.24474	6.44348	6.44097	6.56573	6.65297	5.9817	6.10441	5.99433	5.99802
PTPN23	Q9H3S7	6.22915	6.64484	6.49576	6.07913	6.27518	6.59597	5.87967	6.67558	6.44405	6.34624	6.49558	6.26042	5.82096	5.96007	6.00158	5.79584
PPP2R5D	Q14738	6.27994	6.39366	6.65625	6.09635	6.41486	6.70101	6.18837	6.56664	6.17649	6.28346	6.48093	6.33357	5.93814	5.92747	5.70557	5.85731
TUBB	P07437	6.33135	6.19952	6.69036	5.78066	6.37014	6.79715	6.15613	7.26198	6.58739	6.41964	6.59905	6.98252	5.40995	5.77765	5.14305	5.49346
WFS1	O76024	6.47896	6.42619	5.94568	6.63581	6.28027	5.88633	6.51701	6.05676	6.0443	6.10545	5.90416	5.86752	6.49336	6.5213	6.49783	6.33908
NDUFS3	O75489	6.22016	6.31996	5.91	5.90556	6.47162	6.09474	6.31943	5.97211	5.68367	6.13119	6.29449	6.21059	6.73343	6.39146	6.68711	6.6545
STAU1	O95793	6.38077	6.61908	6.60928	6.21697	6.2175	6.59486	6.29028	6.34826	6.51747	6.61341	6.7642	6.70218	5.47944	5.73328	5.38511	5.52791
STAU2	Q9NUL3	6.19118	6.2878	6.6199	5.92923	6.13446	6.44092	6.40339	6.44543	6.17869	6.57515	6.38722	6.3651	6.58955	6.35749	5.91077	5.78374
AKAP2	Q9Y2D5-6	6.58779	6.27863	6.58728	6.21158	6.36541	6.10731	5.7112	5.64813	6.14965	5.7761	6.08992	6.18366	7.35093	6.32321	6.42001	6.20919
MACROH2A2	Q9P0M6	6.16831	6.37303	5.56458	6.48875	6.1193	5.93584	6.41935	6.14043	6.08502	6.02788	5.81002	5.61963	6.47421	6.79914	7.26894	6.70561
CHD6	Q8TD26	6.47791	6.47398	6.24592	6.51196	6.11576	6.04873	6.2507	5.97853	6.23788	6.46839	6.0834	6.44696	6.54715	6.47636	6.07198	6.46438
FLOT1	O75955	6.49298	7.06131	6.20931	6.89008	6.3373	6.16395	6.3858	6.12238	6.21937	6.6779	6.37235	6.33779	5.71124	5.58127	5.86109	5.57588
ALDH5A1	P51649-2	6.46819	6.34557	5.75143	6.46315	6.30645	5.98166	6.4723	6.12486	6.05745	6.00659	5.95359	5.84561	6.78118	6.35565	6.69665	6.38966
LSS	P48449	6.53458	6.49491	6.1032	6.61042	6.26145	6.14008	6.38629	6.09568	6.05291	6.37393	6.03701	5.94174	6.14383	6.38529	6.34079	6.0979
OAS3	Q9Y6K5	5.94325	6.56453	6.26555	6.00438	6.70064	6.65578	5.9583	6.53183	6.36211	6.46414	6.54791	6.12174	6.18255	6.15563	5.84324	5.69842
GNL3L	Q9NVN8	6.70916	6.64165	6.55524	6.57488	6.51849	6.50868	6.72029	6.21936	6.43678	6.40182	6.51442	6.3586	5.42083	5.4956	5.40531	5.51887
URB2	Q14146	6.28032	6.36519	5.85687	6.59609	6.1234	5.90301	6.56736	5.91039	6.2325	5.88166	5.9597	5.91591	6.65196	6.25377	6.64732	6.85454
ACTR1B	P42025	6.31626	6.21754	6.63189	6.15186	6.28348	6.71378	6.09683	6.61804	6.4387	6.32579	6.4612	6.58988	5.77031	6.13887	5.62653	5.61906
MAD1L1	Q9Y6D9	6.26514	6.18773	6.24667	6.40479	6.43498	6.09755	6.24204	6.33743	6.24756	5.99479	6.23149	6.16911	6.74272	5.84482	6.30249	6.2507
HSPE1	P61604	5.7199	4.8911	5.22544	4.87633	6.09257	5.2344	5.85368	6.71335	5.72977	6.21663	5.67113	6.27932	6.74003	8.31309	7.99346	8.4498
XPO4	Q9C0E2	6.23657	6.18409	6.35286	5.96504	6.11486	6.16204	6.16006	6.08485	6.30681	6.21374	6.06071	5.95762	6.55706	6.37185	6.70112	6.57073
PGM1	P36871	6.21939	6.06359	6.64766	5.70111	6.42586	6.94	6.07714	6.799	6.36208	6.68602	6.45298	6.35253	5.77888	6.28945	5.70969	5.49462
ALDH1L2	Q3SY69	6.58605	6.35508	5.72168	6.3319	6.32492	5.98595	6.2926	6.02732	6.03256	5.99635	5.74382	5.85191	7.06206	6.49404	6.57192	6.62183
SNTB2	Q13425	5.92318	6.88213	5.78901	5.60687	5.74081	6.26972	6.37388	5.9861	6.29038	6.18965	6.13527	6.10257	6.02399	6.27771	7.68278	6.72595
SRGAP2	O75044	6.05058	6.12555	6.65203	6.05185	6.17287	6.77262	6.28624	6.27589	6.30252	6.22653	6.3827	6.3327	6.03439	6.25944	5.93295	6.14114
ZNF148	Q9UQR1	6.63246	6.46659	6.04158	7.08761	5.95269	5.97871	6.08467	6.0553	6.12587	6.47497	5.98015	6.17053	6.18872	6.13916	6.16596	6.45502
MORC3	Q14149	5.799	6.19846	5.99218	6.19039	5.90908	6.12403	5.94775	5.82611	6.18825	6.22598	6.19463	5.99118	7.08135	6.70086	6.79723	6.83353
DYNC1LI2	O43237	6.28987	6.07298	6.91276	5.83315	6.32119	6.67423	6.06425	6.66825	6.45101	6.40229	6.52123	6.39755	6.02808	5.97773	5.61131	5.77414
LCOR	Q96JN0-3	6.40814	6.3784	6.17006	6.83914	6.24074	5.88307	6.04056	5.98274	5.99835	5.9753	5.8119	5.82241	6.01272	6.71201	6.69558	7.02887
CSNK1A1	P48729	6.65799	6.66444	6.64312	6.59906	6.49548	6.37028	6.79203	6.46753	6.78725	6.33755	6.25291	6.30642	5.1936	5.40005	5.35444	5.67785
EPS15	P42566	6.2642	5.99389	6.53082	5.69751	6.56083	6.36512	6.05005	6.537	6.64327	6.29121	6.60458	6.51056	6.52332	5.93672	5.67529	5.81563
MAGEA4	P43358	6.24562	6.02717	6.74729	5.94574	6.41633	6.68976	6.09844	6.46243	6.35354	6.40108	6.51279	6.43156	6.04508	6.07113	5.76003	5.79202
LRRFIP1	Q32MZ4	6.27079	6.11466	6.68901	5.77928	6.5736	6.65727	5.94005	6.62888	6.53595	6.28903	6.79416	6.50797	6.21672	5.85457	5.66136	5.48671
GPKOW	Q92917	5.69592	6.16552	5.84462	5.86516	6.3979	6.49774	6.06279	6.18518	6.15266	6.0335	6.3969	6.21944	7.04434	6.76139	6.42206	6.25487
FNDC3A	Q9Y2H6	6.23251	6.25556	6.07752	6.54396	6.20179	5.82544	6.41166	5.8417	6.29763	6.00328	6.02117	6.11065	6.724	6.41907	6.50837	6.52568
APPL1	Q9UKG1	6.48298	6.51666	6.88546	6.00776	6.5767	6.74712	6.33586	6.70691	6.51635	6.64216	6.50723	6.53964	6.51417	5.2678	5.37748	5.27572
CUL4A	Q13619	6.20905	6.16612	6.60285	6.10421	6.44389	6.17334	6.40133	5.98329	6.23081	6.29008	5.9956	5.91972	6.50794	6.11691	6.51093	6.34393
IVD	P26440	6.17493	5.92977	6.17205	6.58215	6.28958	6.09539	6.36461	6.05114	6.14381	6.09728	6.10384	6.1683	6.80957	6.09576	6.40597	6.51584
NSFL1C	Q9UNZ2-5	5.91811	6.104	6.40371	5.88595	6.15021	6.59327	5.76105	6.75273	6.35867	6.28097	6.50614	6.84053	6.59626	6.06677	5.69793	6.08369
XPNPEP1	Q9NQW7-3	6.43888	6.26489	6.98529	6.03723	6.3617	6.81896	5.99824	6.46125	6.18463	6.52148	6.69144	6.57211	5.88036	5.82347	5.44104	5.51901
SLC12A2	P55011	6.49123	6.42588	6.33429	6.74295	6.0961	6.1321	6.25864	5.80661	6.29925	6.29992	6.33901	6.44712	6.12609	5.84138	6.18537	6.17409
TXNL1	O43396	6.13611	6.30852	7.18925	6.39187	6.15163	6.9127	6.01255	6.34197	5.88798	6.59322	6.27345	6.70893	5.82574	5.78762	5.5077	5.97074
AASS	Q9UDR5	6.46034	6.40118	5.67603	6.49395	6.44263	6.24369	6.23653	5.9972	5.90019	6.00202	6.01692	5.99284	6.59261	6.44977	6.49206	6.60206
TCF20	Q9UGU0	6.34631	6.37631	6.2028	6.865	6.15177	6.15519	6.33369	5.99567	5.89703	6.29895	6.03713	5.97406	6.33814	6.33642	6.5195	6.17204
SFXN1	Q9H9B4	6.36269	5.88023	5.81065	6.1691	6.23841	5.78729	6.49897	5.95617	6.1527	6.09783	5.83437	6.00307	6.73051	6.51721	6.86494	7.09587
STUB1	Q9UNE7	6.42382	6.35829	6.58686	6.1561	6.20844	6.49139	5.94846	6.5306	6.42184	6.76998	6.80156	6.53453	6.10763	5.58575	5.48396	5.5908
SETD2	Q9BYW2	6.5909	6.72357	6.45115	7.21571	6.18149	5.94038	6.42766	6.04792	6.47104	6.06678	6.22333	6.22747	5.70318	5.73485	6.15683	5.83774
LARP4B	Q92615	6.342	6.42281	6.47452	5.93402	6.54022	6.25615	6.20335	6.39347	6.30578	6.26628	6.43833	6.5308	5.97858	6.04631	5.94135	5.92603
IPO9	Q96P70	6.15056	6.04261	6.78721	5.97845	6.34353	6.81342	6.2928	6.42731	6.43863	6.6088	6.47636	6.2428	6.01145	5.76947	5.91493	5.70168
TMED2	Q15363	6.35045	6.30179	5.92035	6.44169	6.05865	5.88874	6.01907	6.08788	6.07353	5.90208	6.12796	6.10267	6.65402	6.57475	6.91061	6.58577
DDX56	Q9NY93	6.48099	6.25614	5.93172	6.85901	6.00325	5.79563	6.12213	5.96334	6.23685	5.95086	6.06691	6.1049	6.38312	6.44569	6.5894	6.81008
AKT2	P31751	6.17453	6.39107	6.72676	5.9363	6.29894	6.9042	6.23972	6.58194	6.26109	6.52637	6.49938	6.35546	5.60287	6.15274	5.73018	5.61844
VPS41	P49754	6.28667	6.22939	6.3455	5.96834	6.41363	6.40695	6.1702	6.16671	6.32963	6.55368	6.23733	6.23385	6.32907	6.30346	6.21243	5.81317
EHHADH	Q08426	6.3077	6.16814	6.02683	6.38314	6.16779	5.86283	6.37887	6.00344	6.34532	6.16433	6.2213	6.03637	6.49988	6.30724	6.70102	6.42581
SLC25A1	P53007	6.44054	6.41229	5.91003	6.69937	6.14044	5.96245	6.42723	5.82406	6.07053	6.00737	6.0635	5.89922	6.64971	6.28719	6.72663	6.47943
THOP1	P52888	6.23604	6.0848	6.90963	5.76918	6.43292	6.76696	6.27849	6.49143	6.54658	6.63721	6.60908	6.50495	5.6067	5.95807	5.68202	5.48596
WDR46	O15213	6.11494	6.15956	5.58654	6.44694	6.1009	5.91583	6.20502	6.16285	5.93841	5.86173	5.60606	5.7462	6.78074	7.06005	7.28583	7.0284
EIF2B3	Q9NR50	6.03029	5.69685	6.79342	6.02233	6.47034	6.57981	6.08845	6.45097	6.60696	6.71781	6.68935	6.82945	6.05907	5.68352	5.66014	5.62123
TMEM214	Q6NUQ4	6.4169	6.41564	5.74531	6.76039	6.30646	6.16386	6.44565	6.05138	6.04491	6.21852	5.9475	5.97282	6.38946	6.37869	6.38824	6.35426
SUGT1	Q9Y2Z0	6.1116	6.07906	6.52585	6.07554	6.31222	6.68748	6.08144	6.39771	6.5134	6.43727	6.38831	6.4483	6.28171	6.12804	5.83818	5.69389
NUDT21	O43809	4.85552	5.03939	5.76124	5.51071	6.44209	5.53933	6.02158	6.58912	6.11636	6.79942	6.33359	6.93329	7.13593	6.53687	7.36176	7.02383
KIFBP	Q96EK5	6.26473	6.3895	6.61221	6.32417	6.38636	6.43643	6.49097	6.53948	6.52926	6.2501	6.34475	6.23189	5.8098	5.8714	5.82048	5.69847
TELO2	Q9Y4R8	6.56567	6.6271	6.74074	6.34095	6.45461	6.61469	5.97846	6.64845	6.81727	6.70566	6.59634	6.8552	5.29393	5.50815	5.16413	5.08866
TXLNA	P40222	6.58957	6.29568	6.86633	6.18164	6.47837	6.56731	6.23562	6.46504	6.99938	6.70647	6.63858	6.75688	5.32045	5.39514	5.08996	5.41359
RFC2	P35250	6.00235	6.16814	6.13112	6.20456	6.47842	6.30722	6.09027	6.36474	6.13395	6.17772	6.01705	6.32046	6.52714	6.19894	6.35448	6.54144
CYFIP2	Q96F07	6.47136	6.08749	6.64007	5.95663	6.32045	6.42613	6.215	6.34663	6.42199	6.47308	6.62076	6.47468	5.95163	5.91667	5.92605	5.75138
NOP9	Q86U38	5.989	6.23729	5.62867	6.39667	6.34841	6.09742	6.31954	6.20951	6.11092	6.18228	6.02841	5.89233	6.74767	6.44822	6.5025	6.86115
PRDX5	P30044	5.91078	5.47735	5.98415	5.31233	6.41141	6.11295	6.02541	6.69253	6.31649	6.62039	6.37562	6.5654	6.36685	6.49896	6.76677	6.56261
RIOX1	Q9H6W3	5.89275	6.30144	5.84811	6.89258	5.99763	5.65969	6.40886	5.94012	6.02179	6.02258	6.06345	6.10833	6.78905	6.39534	6.71561	6.94267
COLGALT1	Q8NBJ5	6.26249	6.3052	5.83615	6.91883	5.97408	5.87035	6.48479	5.93228	6.19752	5.58495	5.84903	5.97194	6.54239	6.47923	6.81422	6.97657
DDX31	Q9H8H2	6.16954	5.90159	5.81278	6.61251	6.04446	5.7249	6.39569	5.87535	6.30142	6.10469	6.14925	6.0241	6.7663	6.66031	6.64604	6.81109
MORC2	Q9Y6X9	5.80545	5.98125	6.14882	6.3684	5.99065	6.02025	6.30052	5.80462	6.05068	6.12784	6.10205	5.94032	6.95613	6.48496	7.04906	6.86899
AKAP8	O43823	6.56176	6.35602	5.9455	6.69277	6.0583	6.06037	6.19733	6.0544	6.31754	6.13531	6.04896	6.25324	6.69368	6.09881	6.17107	6.35494
SPTLC2	O15270	6.4214	6.68242	6.34769	6.82829	6.26986	6.00816	6.17955	6.05877	6.15957	6.16695	6.23519	6.08617	6.30089	5.84603	6.44801	5.96105
KIDINS220	Q9ULH0	6.65797	6.53925	6.31527	6.84466	6.11779	6.05917	6.22277	5.92288	6.26883	6.1518	6.0434	6.33353	6.14705	6.09188	6.14162	6.14211
FBXL18	Q96ME1-4	6.05386	5.88094	6.70787	5.77476	6.00258	6.54758	6.01622	6.25347	6.2868	6.61735	6.53277	6.4135	6.15986	6.43058	6.35857	5.96327
TIMM50	Q3ZCQ8-2	6.51228	6.34163	5.94053	6.70461	6.56091	6.0818	6.21805	6.24822	6.17791	5.98565	6.27599	6.42376	6.54385	5.98198	6.01909	5.98373
WDR5	P61964	6.0919	6.45802	5.99895	6.30402	6.50899	6.31957	6.31704	6.16963	6.00578	6.04929	5.93442	5.74548	6.82967	6.45157	6.61892	6.19676
CC2D1A	Q6P1N0	6.169	6.26698	6.85336	6.26071	6.18614	6.59604	6.31596	6.27526	6.51043	6.77147	6.67934	6.71103	5.61855	5.85504	5.18416	5.74653
ANAPC4	Q9UJX5-3	6.2275	6.17278	6.40615	6.54349	6.252	6.12111	6.54001	6.15576	6.41794	6.06033	6.10706	6.38334	5.93226	6.07907	6.18114	6.42006
TNPO3	Q9Y5L0-1	5.91373	6.01341	6.15583	5.66788	6.11215	6.47895	6.07827	6.37146	6.37118	6.32951	6.19175	6.26126	6.45692	6.60489	6.39887	6.59394
NDC1	Q9BTX1	6.27418	6.2245	5.69313	6.80237	6.08005	5.87301	6.50695	6.05489	6.21641	6.01001	5.87651	5.90784	6.59363	6.31379	6.68918	6.88354
ETHE1	O95571	6.38317	6.16864	6.10864	6.36026	6.41538	5.91738	6.06601	6.27136	6.19737	6.06218	6.17259	6.22733	6.72953	5.98617	6.49128	6.44273
MED1	Q15648	6.28298	6.52174	5.95067	6.74028	6.10466	5.98314	6.26337	5.83945	5.84287	6.04209	6.09299	5.97553	6.16016	6.73206	6.67053	6.79748
POLR1G	O15446-2	5.82766	5.81173	5.77107	6.70272	5.80598	5.84859	6.43756	5.6719	6.00764	5.71593	5.66775	5.92488	6.33249	7.4753	7.21999	7.77881
P3H1	Q32P28	6.48758	6.59674	5.93721	6.54478	6.24195	6.29053	6.02245	6.09697	5.81522	6.15995	6.16718	6.15926	6.59417	6.27859	6.31284	6.29458
NVL	O15381	6.14589	6.56103	6.27159	6.82628	6.14295	5.92216	6.20339	5.86917	6.41704	6.08654	6.15033	6.27114	6.23826	6.06373	6.3864	6.4441
U2AF1	Q01081-2	5.96157	6.1841	5.93224	6.32593	5.992	5.76626	6.43301	5.79639	6.24251	6.23761	6.13828	5.89746	6.56738	6.72144	6.85112	6.9527
CDC42	P60953	6.33954	5.97095	6.16077	5.86088	6.37641	6.16421	6.17368	6.53072	6.28237	6.43335	6.27538	6.39328	6.25909	6.39907	6.18287	6.19741
TFIP11	Q9UBB9	6.09714	6.25302	6.09584	6.24289	6.14061	6.06789	6.4414	5.9859	6.06151	6.23881	6.05807	6.13126	6.61988	6.44955	6.731	6.38523
CYRIB	Q9NUQ9	6.3464	6.32603	6.49711	6.15516	6.17449	6.54522	6.09664	6.61911	6.15668	6.45325	6.46052	6.22926	5.93431	6.27207	5.72079	6.01295
ERCC3	P19447	5.93798	6.15582	6.1313	6.42657	6.08779	6.24745	6.47799	5.95511	6.19431	6.32915	6.21182	6.24619	6.38948	6.18363	6.59856	6.42685
CORO7	P57737-3	6.33013	6.04681	6.26994	5.64202	6.67415	6.59114	6.48724	6.64347	6.28058	6.10357	6.04304	6.10495	6.42895	6.17041	6.1301	6.0535

ZC3H13	Q5T200	6.19417	6.37459	6.03172	6.70128	5.86515	5.98054	5.83496	5.39872	6.07351	5.87051	6.19792	6.234	6.34535	6.95057	6.84979	7.09721
EXOC4	Q96A65	6.34688	6.4268	6.57814	5.97918	6.43056	6.55693	6.29817	6.45959	6.50554	6.56588	6.4132	6.30643	6.82558	5.89237	5.6337	5.78106
AKAP1	Q92667	6.41609	6.22777	6.18132	6.59944	6.16552	5.74882	6.29777	5.88306	6.20369	5.8136	6.08795	5.92706	6.73434	6.2949	6.71017	6.70851
NCEH1	Q6PIU2	6.22468	5.92602	6.08313	6.64278	6.38755	5.84492	6.42757	6.143	5.81535	5.60241	5.92492	5.77038	6.76286	6.58725	6.78243	7.07475
DOCK10	Q96BY6	5.80036	5.89636	6.06973	5.85696	5.78984	6.27005	6.2232	6.20051	6.47268	6.29974	6.40208	6.39749	6.60815	6.80481	6.4363	6.47175
POGLUT3	Q7Z4H8	6.45661	6.35893	6.10501	6.64615	6.36303	6.2536	6.44168	6.06441	5.99559	5.9421	6.39961	6.28102	6.25145	5.9929	6.25572	6.1922
EIF3M	Q7L2H7	6.39955	6.38593	6.74605	6.17051	6.51872	6.68067	6.20948	6.49151	6.38102	6.44763	6.5735	6.37611	5.52984	5.71826	5.69285	5.67837
ATP6V1C1	P21283	6.31485	6.26246	6.56445	6.10545	6.36959	6.45982	6.058	6.80829	6.28843	6.65748	6.55545	6.66494	5.78907	5.89612	5.52074	5.68486
CALM1	P0DP23	7.16554	5.8561	6.0953	5.07229	5.62569	5.4494	5.61694	6.81146	5.8568	7.10726	6.5433	7.20436	5.77507	6.99588	6.35294	6.47167
LGALS1	P09382	5.29127	4.68042	4.29317	4.28719	4.98886	4.10075	5.42894	6.83702	4.27862	5.39643	4.30094	6.12296	5.43127	12.2436	9.51697	12.8016
CDKAL1	Q5VV42	6.36887	6.31685	5.96467	6.73422	5.99857	6.16585	6.47362	6.14867	6.07596	6.2157	6.20312	6.23439	6.21663	6.30369	6.11586	6.46332
PTPN12	Q05209	6.23615	6.16666	6.7203	6.14545	6.35032	6.67461	6.0443	6.75683	6.44376	6.35299	6.32877	6.69853	6.70876	5.96362	5.62187	5.78706
MRPS22	P82650	6.39381	6.34974	5.56586	6.52327	6.53975	6.24148	6.31396	6.40707	6.32732	6.18142	6.18606	6.30805	6.31844	5.94127	6.16041	6.24207
ZMPSTE24	O75844	6.15923	6.07008	5.72438	6.47038	6.35413	6.21301	6.35685	6.11242	6.1775	6.01633	5.82505	5.83316	6.96736	6.61249	6.59656	6.51105
CHAF1A	Q13111	6.50996	6.8825	6.19888	7.33648	6.17241	6.14613	6.28636	6.02446	6.04994	6.44123	6.23229	6.42699	5.81762	5.80312	5.57985	6.09179
RAP1GDS1	P52306-5	6.42355	5.72432	6.95508	5.37355	6.4068	6.49053	6.3513	6.57292	6.28823	6.60009	6.35351	6.94218	5.70629	6.09638	5.72955	5.98572
SF3A2	Q15428	6.35863	6.43547	5.79803	6.58804	6.50774	6.21729	6.07581	6.00716	6.06968	6.38575	5.92528	5.91386	6.52104	6.14784	6.60916	6.43922
MAT2A	P31153	5.88487	6.14506	6.71559	5.57881	6.09257	6.30313	6.14651	6.42076	6.10678	6.13669	6.4233	6.29741	6.23654	6.59973	6.64238	6.26987
CRKL	P46109	6.10115	5.92191	6.52555	5.62417	6.46643	6.32757	5.80083	6.83326	6.46404	6.57634	6.72702	6.92544	5.79521	5.9775	5.9198	6.01379
CDC37	Q16543	6.01884	5.84269	6.67352	5.77566	6.13102	6.4724	6.06508	6.43349	6.24285	6.11188	6.43117	6.36777	6.51964	6.38152	6.31364	6.21882
MTDH	Q86UE4	6.10968	6.58296	5.91983	6.49285	5.94633	5.92486	6.33993	6.01117	6.03212	6.1451	5.83717	5.74312	6.19873	7.13325	6.8286	6.7543
NIFK	Q9BYG3	6.24992	6.44435	5.83457	6.94145	6.18705	5.833	6.34234	6.04819	5.83442	5.67519	5.80633	5.93105	6.40586	6.4919	7.00857	6.96583
TBCE	Q15813	6.2258	6.16761	6.86276	5.91349	6.56858	6.84124	6.39503	6.87187	6.37792	6.28353	6.58476	6.47092	5.87433	5.61458	5.40927	5.53833
PRKAR1A	P10644	5.68414	5.93387	6.22638	6.09443	5.98129	6.21648	5.96897	5.94645	6.50984	6.78862	6.85461	6.7105	6.3428	6.12487	6.43511	6.18164
GLS	O94925-3	6.41416	6.06417	6.02609	6.86184	6.37059	5.87434	6.46899	5.8959	6.23347	5.91309	5.83321	6.03344	6.76881	6.07956	6.53072	6.63161
SNRPA1	P09661	6.0592	6.3973	5.87458	6.52775	6.2079	6.13765	6.36613	6.1793	5.86947	5.92741	6.07691	6.31396	6.79045	6.31837	6.47393	6.47967
INPPL1	O15357	6.27899	6.18285	6.48919	5.75761	6.39697	6.57154	6.16379	6.45953	6.19975	6.52137	6.3849	6.43908	6.00976	6.38038	5.83184	5.93245
BCAR1	P56945-6	6.44085	6.34081	7.37456	5.89898	6.33273	6.73395	6.38918	6.92818	6.38104	6.26237	6.91558	6.78866	5.44084	5.44866	5.22914	5.09446
RAB3GAP2	Q9H2M9	5.71381	6.07721	6.41089	5.74252	6.0175	6.2202	5.80594	6.26206	6.2335	6.2432	6.14816	6.4035	6.52794	6.82309	6.71385	6.65665
FMNL3	Q8IVF7	6.16513	6.50029	6.24588	6.12502	6.43608	6.26905	6.34205	5.90461	6.61694	6.3795	6.19765	6.24271	5.98328	6.0212	6.33811	6.23248
AGK	Q53H12	6.23597	6.52191	5.88894	6.63217	6.29703	5.99272	6.20213	5.8354	6.22675	5.91309	6.00127	5.80068	6.89957	6.30417	6.7056	6.54257
ATP2C1	P98194-7	6.60853	6.46188	6.20741	6.77593	6.27705	6.11164	6.11385	6.00449	6.44091	6.15336	6.00714	6.07963	6.10358	6.09942	6.4658	6.08938
ZNF512B	Q96KM6	6.54723	6.49198	5.81296	6.89793	5.78577	5.65522	6.26358	6.16284	5.94255	6.35982	5.59566	5.89429	6.30182	6.71775	6.78555	6.78507
YWHAG	P61981	6.23329	6.21152	6.24131	5.61738	6.59925	6.58558	6.58125	6.52026	6.61955	6.52932	6.59096	6.11765	6.27215	6.30542	6.19675	6.07836
LMAN1	P49257	6.15958	6.28116	5.80108	6.28923	5.9961	5.84332	6.20851	5.94938	6.20479	6.0985	5.93189	6.06186	6.67066	6.61819	7.05437	6.83137
SAMD4B	Q5PRF9	6.3057	6.36046	6.94652	5.96575	6.24278	6.70786	6.22383	6.53842	6.44505	6.49417	6.82258	6.69336	5.78809	5.78587	5.37312	5.30643
COPS2	P61201-2	6.10489	6.24547	6.34123	6.04272	6.42195	6.52339	6.16272	6.33624	6.14974	6.16547	6.26065	6.09473	6.5281	6.26749	6.32857	6.02664
MAP7D1	Q3KQU3	6.14904	6.31601	6.614	5.92632	6.08193	6.41455	6.0488	6.51436	6.30826	6.58617	6.40787	6.48441	5.93612	6.25728	6.02125	5.93364
ESYT2	H7BXI1	6.38321	6.43409	5.78861	6.47066	6.30449	6.1842	6.25918	5.8002	6.12826	6.18506	5.78806	5.68684	6.84611	6.41601	6.77738	6.54767
HK2	P52789	5.86327	5.92871	6.06131	5.82775	6.98049	7.07316	7.23695	6.59686	5.5513	5.48114	5.68063	5.59225	6.77246	6.27389	6.63732	6.44251
VPS53	Q5VIR6	6.34027	6.33935	6.4987	5.852	6.45795	6.66093	6.09612	6.45675	6.31094	6.51153	6.31399	6.41735	6.05814	6.09382	5.80608	5.78607
TOR1AIP1	Q5JTV8	6.34357	6.43466	5.85984	6.88131	6.32667	5.91967	6.2323	5.99808	6.20196	5.79773	6.05712	5.91216	6.72608	6.34656	6.49238	6.46992
GNL1	P36915	6.08723	6.08395	7.00161	6.20255	5.91579	6.40594	6.4053	6.64624	6.27981	6.4593	6.62139	6.37135	5.5905	6.22696	5.85226	5.84984
NXF2	Q9GZY0	6.4062	6.5309	6.23989	6.83924	6.32795	6.23841	6.4338	5.87607	6.23861	6.5144	6.30699	6.42738	6.07162	5.52251	6.07591	5.95013
ERLIN1	O75477	6.43763	6.5047	6.12119	6.48394	6.06612	6.25799	6.25355	6.01798	6.23162	5.86836	6.03011	6.21449	6.41196	6.12478	6.474	6.50158
CALCOCO2	Q13137-4	6.8335	6.87803	7.50191	6.37989	6.60613	6.94273	6.31375	6.79423	6.54423	6.51679	6.77829	6.82841	4.75153	4.87177	4.51245	4.94637
OPTN	Q96CV9	6.44938	6.24862	6.92981	6.21656	6.54298	6.90095	6.07151	6.6055	6.26314	6.45189	6.74718	6.90265	5.95558	5.49332	5.08603	5.13491
SEPTIN10	Q9P0V9-2	6.14914	6.25325	6.45753	6.04651	6.37047	6.56278	6.17999	6.57619	6.38123	6.46103	6.27304	6.34141	5.9864	6.14928	6.09039	5.72136
DDX19B	Q9UMR2	6.35279	6.02459	6.7177	6.03805	6.54759	6.65151	6.31018	6.40245	6.40641	6.43648	6.59554	6.65215	5.88391	5.80773	5.65883	5.71409
SETD3	Q86TU7	6.52869	6.34703	6.98533	6.36647	6.09244	6.33095	6.27105	6.37756	6.64447	6.46819	6.80608	6.86183	5.49217	5.66964	5.41165	5.34646
RNPEP	Q9H4A4	5.98998	6.17983	6.89345	5.79497	6.18747	6.81316	6.26789	6.69214	6.3125	6.37638	6.45449	6.35346	5.82533	6.23996	5.88725	5.73174
NDUFV3	P56181-2	6.40525	6.43999	5.96525	6.79906	6.01582	5.88954	6.151	5.8528	6.21541	6.14159	6.06155	6.09202	6.80384	6.25204	6.53124	6.38362
ARMT1	Q9H993	5.94161	5.91473	6.8824	5.41376	6.35013	7.07418	6.16402	6.67794	6.15899	6.66335	6.27069	6.39382	5.90099	6.51824	5.74503	5.93013
ALDH6A1	Q02252	6.19614	6.45279	5.81368	6.28158	6.38293	6.14706	5.99525	6.07201	6.14695	6.11267	5.79962	5.96762	7.16053	6.50601	6.52523	6.43994
LSG1	Q9H089	6.6598	6.47537	6.27485	6.37845	5.99774	6.05343	6.31338	6.08144	5.91802	5.94605	5.72707	5.82693	6.62945	6.50405	6.57288	6.64107
MARK3	P27448	6.18696	6.18462	6.39651	6.13973	5.95865	6.19977	6.15593	6.05571	6.22881	6.34076	6.58286	6.60182	6.32157	6.06906	6.14266	6.43458
CHORDC1	Q9UHD1	5.95606	6.14063	6.69562	5.95679	6.36597	6.85622	6.25732	6.4142	6.33629	6.45332	6.75942	6.49217	5.92536	5.84621	5.68632	5.8581
CKAP2L	Q8IYA6	6.25209	5.70578	6.16209	5.87452	5.94665	6.08114	6.15727	5.89017	6.46096	6.36023	6.51167	6.54221	6.20242	6.65761	6.37569	6.8195
PCCA	P05165	6.40473	6.29724	6.0365	6.57789	6.03367	5.95573	6.3076	5.87754	6.27232	6.22975	5.9951	5.84212	6.94019	6.23107	6.85624	6.14231
PGM2	Q96G03	6.08816	6.19976	6.70807	5.71837	6.12766	6.58295	6.08175	6.46586	6.25671	6.39417	6.5409	6.48393	6.03837	6.10849	6.44898	5.75588
CBR1	P16152	6.23589	6.00424	6.8161	5.83828	6.58773	6.88733	6.04518	6.58507	6.24698	6.56638	6.73776	6.56116	6.04712	5.93541	5.58872	5.31664
TYK2	P29597	6.42143	6.64857	6.4645	6.58042	6.34077	6.40453	6.1338	6.03008	6.55032	6.57888	6.62931	6.67025	5.70129	5.62354	5.47917	5.74314
MPP6	Q9NZW5	6.26334	6.22153	5.93189	6.57769	6.14638	6.07795	6.19688	6.11371	6.28436	5.98591	6.01598	6.17296	6.63509	6.13486	6.60319	6.63828
PRPSAP1	Q14558-2	6.18055	6.21848	6.91728	6.04627	6.27175	6.76608	6.0955	6.71323	6.17446	6.43252	6.41149	6.44058	5.82677	6.09437	5.66256	5.74814
CNN2	Q99439	6.40216	6.09515	6.78027	5.97597	6.6226	6.58717	6.27218	6.92822	6.38756	6.12956	6.71033	6.64348	6.0269	5.73365	5.22489	5.47991
ANAPC7	Q9UJX3	6.39083	6.71939	6.25095	6.68506	6.51684	6.50532	6.48127	6.45012	6.40849	6.1302	6.45139	6.05558	5.59167	5.83553	5.79155	5.73581
ZFYVE26	Q68DK2	6.77514	6.79797	6.76269	6.76845	6.19809	6.41559	6.19597	6.28031	6.49606	6.65594	6.89213	6.68946	5.17448	5.45326	5.24545	5.19001
CLIC4	Q9Y696	6.14273	5.92662	6.70406	5.38559	6.54305	6.33151	5.74011	6.50202	6.26637	6.30312	6.10792	6.441	6.55347	6.63066	6.27101	6.15077
KMT2D	O14686-3	6.40601	6.65641	6.08666	6.67603	5.95515	6.08258	6.24406	6.08894	6.29776	6.41195	6.25823	6.47829	6.13915	6.03885	6.10487	6.07501
NSUN5	Q96P11-2	5.9404	6.1232	5.94929	6.05411	6.2473	6.04436	6.31183	6.02915	6.29512	6.40835	6.10905	6.21776	6.62023	6.39983	6.6952	6.55483
MFN1	Q8IWA4	6.64589	6.29072	6.21883	7.04951	6.59727	6.53866	6.38392	6.14407	6.25829	5.98939	6.0183	5.91543	6.16609	5.93606	5.69881	6.14877
LDLR	P01130	5.67915	5.89289	5.99894	6.40203	5.41774	5.48121	5.79907	5.60332	6.05944	6.30318	6.45732	6.12376	7.27288	6.85173	7.46883	7.1885
TAP2	X5CMH5	6.20122	6.33618	5.84245	6.59214	6.12399	6.07964	6.23584	6.01881	5.90846	6.0491	6.16369	6.01872	6.30429	6.58606	6.88202	6.6574
LAMC1	P11047	6.54388	6.59363	6.17282	6.83674	6.48189	6.46567	6.23828	6.31673	6.20751	6.17045	6.16033	6.36862	6.16984	5.72017	5.65323	5.90019
FECH	P22830-2	6.49537	6.61659	5.83865	6.39434	6.30387	5.81293	6.3688	5.98716	6.15363	5.99709	6.00291	5.96329	6.92255	6.49923	6.41904	6.22456
CRNKL1	Q9BZJ0	6.33478	6.4545	6.04396	6.77609	6.32587	5.93319	6.37153	5.86615	6.219	6.09645	5.94188	5.90692	6.29481	6.39534	6.54748	6.49205
SMARCA2	P51531	6.32134	6.58806	5.69172	6.51743	6.26107	5.85284	6.20891	6.04542	5.92128	6.20335	5.71612	5.93438	6.5896	6.66938	6.82229	6.65682
CKAP2	Q8WWK9	6.13102	5.95717	6.22693	6.09731	5.80364	5.92748	6.13657	5.99994	6.74936	6.63979	6.89714	6.86476	6.00192	6.19245	6.01568	6.35885
TTK	P33981	6.36713	6.66458	6.92825	6.29984	6.08332	6.75306	6.4733	6.71561	6.74769	6.67948	6.64565	6.65961	5.06429	5.33632	5.0659	5.51598
GALNT7	Q86SF2	6.33453	6.42372	5.87649	6.49905	6.23113	5.92862	6.57969	5.90946	6.17264	6.03773	6.02016	5.80501	6.56554	6.35837	6.81835	6.44443
RHOT2	Q8IXI1	6.60334	6.5494	5.98548	6.42887	6.54588	6.30486	6.66387	6.20553	6.28299	5.99615	5.80688	5.83557	6.27925	5.96842	6.36835	6.17516
DCAF8	Q5TAQ9	6.0343	6.15314	6.47576	6.11725	6.2704	6.43635	6.18164	6.09008	6.2637	6.16154	6.52092	6.31229	6.58403	5.92911	6.30935	6.16014
PBXIP1	Q96AQ6	6.69321	6.97465	6.5039	6.62251	6.12626	6.18858	6.29019	6.20793	6.09818	6.47241	6.14703	6.20636	5.87045	5.96004	5.91733	5.72097
DLG1	Q12959-7	6.65276	6.52215	6.23251	6.76854	5.92036	6.35261	6.27081	6.08954	6.19604	6.08323	6.13752	5.75064	6.17686	6.14586	6.17125	6.52932
NT5DC2	Q9H857-2	6.64931	6.72204	5.89228	7.054	6.15491	6.0703	6.15733	6.19297	6.47053	6.4956	6.16412	6.3727	6.03117	5.64886	6.05006	5.87383
HERC1	Q15751	6.24195	6.4885	6.9766	6.174	5.95747	6.39954	5.95981	6.42655	6.65487	6.39263	6.43614	6.8768	5.66362	5.9593	5.66892	5.7233
GLOD4	Q9HC38-2	6.44233	5.9297	6.90522	5.85962	6.5287	6.6375	6.01502	6.5744	6.41408	6.37171	6.63175	6.61559	6.0946	5.84261	5.49723	5.63995
NACA	E9PAV3	6.75553	6.5873	7.11467	6.10877	6.3614	6.71516	6.25775	6.4902	6.85192	6.67507	7.06753	7.11073	5.16297	5.23578	4.79384	4.9114
CYP51A1	Q16850	6.86967	6.74389	6.43162	6.73886	6.54831	6.11811	6.46017	5.83852	6.39337	6.14922	6.5018	6.31418	5.70449	5.56694	5.91086	5.70999
CYFIP1	Q7L576	6.08364	6.19908	6.57743	5.85693	6.06543	6.57985	6.25932	6.37676	6.56056	6.55071	6.5427	6.36413	6.00452	6.12949	6.06675	5.78268
COG2	Q14746	6.49384	6.31713	6.44718	6.6892	6.25798	6.28891	6.31132	6.29069	6.58781	6.43886	6.13074	6.14783	6.14214	5.82437	5.95363	5.67837
RIPOR1	Q6ZS17-4	6.16695	6.03544	6.82168	6.15888	6.34673	6.72503	6.25057	6.68537	6.45236	6.3834	6.48706	6.48486	5.96213	5.69629	5.6583	5.68496
PARK7	Q99497	6.47925	5.77257	6.81831	5.00985	7.07842	6.37983	5.96953	7.03505	6.2866	6.53381	6.33466	6.64243	6.188	6.19837	5.69136	5.58197
SUGP1	Q8IWZ8	6.29316	6.46604	6.23057	6.31361	6.11296	5.94925	6.31155	6.04332	6.35369	6.51433	6.23815	6.40478	6.48878	6.01166	6.20307	6.06507
CDCP1	Q9H5V8	6.02358	6.27559	6.00888	6.58225	6.09882	5.97559	6.37725	5.93293	6.11081	6.14435	5.9913	5.93167	6.63856	6.32511	6.79506	6.78824
PIK3R4	Q99570	6.29485	6.28146	6.8034	6.21165	6.44989	6.50064	6.36907	6.56109	6.54713	6.42143	6.76265	6.47834	5.75078	5.66247	5.57295	5.33221
CDC16	Q13042	6.27888	6.38952	6.14767	6.20633	6.19785	6.16347	6.34542	6.2974	6.21102	5.91561	6.08376	6.11621	6.35242	6.26914	6.40997	6.61533
DDX28	Q9NUL7	6.43895	6.14548	5.96712	7.09649	6.11262	5.84855	6.51833	5.83644	6.07205	6.24325	6.17667	5.96391	6.53148	6.31336	6.35647	6.37883
POM121	Q96HA1	6.34592	6.20421	6.0353	6.78418	6.09558	5.73844	6.46427	5.97317	6.17508	6.11906	6.17788	6.13193	6.48954	6.28066	6.48428	6.50052
CGN	Q9P2M7	6.45597	6.54496	6.40792	6.79544	6.28064	6.30509	6.00982	6.32675	6.34663	6.31438	6.06642	6.41071	6.28191	5.80162	5.85212	5.7996
MELTF	P08582	6.43912	6.54278	5.91775	6.81448	6.45869	6.53234	6.05935	6.26742	6.2938	6.07725	6.2949	5.69176	6.29335	6.06091	6.24051	6.01558
KPNA4	O00629	6.02321	6.07116	6.52588	6.18655	6.24989	6.4924	5.88759	5.90919	6.32655	6.07936	6.23626	6.25238	6.48144	6.14659	6.48797	6.64358
GUF1	Q8N442	6.39578	6.48718	5.79423	6.79056	6.36521	6.21275	6.22103	6.12138	6.22317	6.02677	5.89051	5.93707	6.53965	6.38382	6.41137	6.19952
THUMPD1	Q9NXG2	5.23013	5.52699	6.07753	5.3599	5.49946	6.19813	5.45077	5.65755	5.73094	6.24688	6.22715	6.08258	7.2898	7.71065	7.99256	7.71897
ZC3H11A	O75152	6.20293	6.02211	6.39328	6.91638	6.19327	6.01444	6.30589	6.00376	5.97676	6.00057	5.88295	6.03622	6.30185	6.3965	6.5506	6.80248

PSMA4	P25789	5.97376	6.21139	6.58041	6.09247	6.36904	6.22562	6.26176	6.21871	6.3824	5.94312	6.3967	6.09495	6.08393	6.14444	6.52631	6.49499
ACO1	P21399	6.32701	6.09287	6.78761	5.87284	6.2868	6.85035	6.22674	6.44945	6.33926	6.60146	6.5544	6.21038	5.74639	6.08272	5.8149	5.65683
FBN2	P35556	6.57455	6.77112	6.04708	6.02672	6.56673	6.55039	6.6481	6.35782	6.01456	5.85391	6.59808	6.42434	6.48691	5.88329	5.74441	6.06199
CPNE3	O75131	6.25216	6.17865	6.90905	5.92309	6.43295	6.64641	6.34122	6.26489	6.38952	6.48119	6.5668	6.37962	5.86341	5.9546	5.81778	5.59865
PPP2R1B	P30154-2	6.28424	6.06072	6.79521	5.70102	6.45745	6.5706	6.1345	6.45823	6.11109	6.35369	6.39645	6.47586	6.02174	6.00694	6.12174	6.05051
UFD1	Q92890-1	5.73013	6.38825	5.79042	6.1428	6.33765	6.41891	6.16941	6.69016	6.37822	6.44774	6.02501	6.22977	6.03779	6.46633	6.1747	6.57271
USP19	O94966-5	6.4158	6.36698	6.58136	6.25079	6.35478	6.37676	6.54934	6.24808	6.41139	6.55262	6.37189	6.23622	5.72923	5.84816	5.94743	5.75917
ZMYM2	Q9UBW7	6.41621	6.7375	6.23051	6.55008	6.23727	6.33136	6.31858	5.92479	6.23066	6.36478	6.18537	6.25365	6.17558	6.10238	5.91776	6.02351
ERCC2	P18074	6.35031	6.30391	6.64647	6.42868	6.18404	6.31932	6.40576	6.43104	6.68977	6.62343	6.68418	6.43682	5.47503	5.6153	5.6127	5.79324
AKR1A1	P14550	6.04961	5.88538	6.46112	5.14856	6.58757	6.60155	5.99431	7.16989	6.26976	6.66554	6.56446	6.69882	5.97717	6.16385	5.97315	5.78927
BRCA1	P38398-7	6.37392	6.46851	6.45353	6.88219	5.8242	5.80332	6.43745	5.74134	6.30952	6.47647	6.31294	6.385	6.25846	6.10646	6.13683	6.02985
FLOT2	Q14254	6.37923	6.51521	6.01154	6.42517	6.3482	6.14954	6.30553	6.1345	6.36442	6.00958	6.03664	6.06096	6.29988	6.42093	6.33847	6.2002
TES	Q9UGI8	6.47066	6.19998	6.70883	5.52198	6.53326	6.70235	6.11454	6.69343	6.41174	6.47061	6.42441	6.35408	6.3031	5.93615	5.61094	5.54394
SLMAP	Q14BN4	6.25098	6.20881	6.02847	6.29368	6.31793	5.96935	6.32849	6.14906	6.13244	5.99858	6.03839	6.00601	6.80603	6.20377	6.7061	6.5619
RELCH	Q9P260-2	6.17966	6.3068	7.00835	6.01236	6.35389	6.55084	5.98698	6.28337	6.10825	6.37252	6.5081	6.2781	6.15294	6.38876	5.7108	5.7983
MAGOHB	Q96A72	6.4161	6.0602	5.97038	5.92178	6.3529	5.92262	6.01774	6.06098	6.31397	6.19899	6.12646	6.31635	6.85172	6.05398	7.02526	6.39056
INTS6	Q9UL03	6.27408	6.39906	5.8418	6.72325	6.2317	5.84735	6.4568	6.07333	6.18618	6.26818	5.8961	5.90586	6.65728	6.27259	6.46438	6.50208
PDE12	Q6L8Q7	6.48224	6.22916	6.21866	6.55125	6.36448	6.25258	6.35555	6.41432	6.50205	6.0218	6.11498	6.0758	6.01457	6.06219	6.19142	6.14893
ADGRE5	P48960	6.68626	6.42974	6.06106	6.51126	6.41969	6.34996	6.27229	6.32314	6.43984	6.11445	6.30789	6.31181	6.30215	5.84268	5.68636	5.94141
CTCF	P49711	6.20879	6.40992	5.90848	6.95847	5.99047	6.06165	6.24103	6.15196	5.84432	6.13822	5.95315	6.03075	6.2941	6.51604	6.79951	6.49314
PRKAA1	Q13131-2	5.48444	5.63747	5.94364	5.49226	5.84211	6.19937	5.89657	6.15835	6.0224	6.22277	6.33105	6.33766	7.02594	7.26668	6.99919	7.14009
ITGB5	P18084	6.44503	6.64221	6.42527	6.37214	6.21011	6.1555	6.20129	6.02474	6.36788	6.47791	6.30338	6.10004	6.24552	5.83067	6.19928	5.99903
ZC3H7A	Q8IWR0	5.45666	6.755	6.95498	6.26755	6.39585	6.40254	6.15788	6.15802	6.39657	6.7649	6.53167	6.5698	5.44979	5.71635	5.49667	5.52576
CASK	O14936	6.38099	6.73289	6.11609	6.66795	6.22347	6.09696	6.11197	6.16267	6.24728	6.1822	5.99012	6.21155	6.23596	6.11368	6.23918	6.28705
RBBP7	Q16576	5.89579	6.00614	6.0356	5.88837	6.56775	6.09612	6.15167	6.23932	6.1227	5.91368	6.33341	6.14336	6.95561	6.63831	6.63062	6.38156
G3BP2	Q9UN86	6.28499	6.42527	6.62589	6.12205	6.55654	6.37942	6.36886	6.38443	6.7114	6.57329	6.56618	6.5707	5.69783	5.46282	5.56766	5.70267
REPS1	Q96D71	6.44483	6.25799	6.12776	5.882	6.40791	6.22211	6.11731	6.20807	6.81855	6.56068	6.28991	6.70694	6.07994	5.84166	6.05913	5.97521
CDC40	O60508	6.50071	6.53012	5.89164	6.70755	6.16623	6.12837	6.39083	6.10339	6.14253	6.192	6.11561	5.98537	6.40402	5.99705	6.48296	6.26161
MRE11	P49959-3	6.272	6.16867	6.03813	6.34323	6.3336	6.1734	6.14154	6.08201	6.03782	6.2272	6.16039	5.95348	6.73326	6.44853	6.4367	6.45004
ARHGEF12	Q9NZN5	6.51185	6.46653	6.82641	6.00706	6.36101	6.64567	6.03679	6.32032	6.17606	6.22432	6.32082	6.26666	6.06034	6.03085	5.79528	5.95005
EXOSC2	Q13868	6.27604	6.33655	5.96419	6.67691	6.27818	6.19585	6.07647	6.10229	6.13122	6.15043	6.19838	6.21004	6.28461	6.07956	6.73807	6.30121
RPS6	P62753	6.1123	6.36357	6.0523	5.96632	6.05794	6.30594	6.39749	6.69724	6.74438	6.56869	6.31443	6.47173	5.7835	6.18323	5.87057	6.11035
VPS26A	O75436	6.14577	6.5051	6.03748	6.15677	6.157	6.6236	6.26401	6.58968	6.21673	5.94779	6.28662	6.11053	5.72154	6.46168	6.58801	6.1877
WDR18	Q9BV38	6.32077	6.51723	5.87059	6.58098	6.40625	6.0232	6.5698	6.00081	6.16578	5.88206	5.86863	6.12808	6.52023	6.06262	6.64026	6.44273
TRIP4	Q15650	6.40011	6.39684	6.64699	6.03821	6.36808	6.51065	6.18836	6.37871	6.42557	6.48069	6.35841	6.2804	5.92292	6.08535	5.7121	5.80661
RB1	P06400	5.31977	5.71381	6.20876	5.48437	5.31399	6.10826	5.62244	5.93167	5.8942	6.23972	6.27271	6.06664	6.77409	7.64679	8.00476	7.39803
TNRC6B	Q9UPQ9	6.17919	6.30658	6.8799	6.1577	6.22329	6.36485	5.8981	6.25702	6.41459	6.61601	6.86143	6.72463	6.20134	5.67731	5.7049	5.53315
CSPP1	Q1MSJ5	6.31695	6.22299	6.56334	6.28871	5.96372	6.73515	6.51184	6.564	6.49899	6.50144	6.4871	6.53843	5.67948	5.82791	5.5457	5.75424
PPFIA1	Q13136	6.09748	5.97059	5.89731	5.99992	6.25544	6.10842	6.09674	5.8394	6.48152	6.20956	6.12048	6.10839	6.8637	6.62822	6.76376	6.5591
CDH2	P19022	6.64695	6.77202	6.08026	6.70811	6.1676	5.97818	6.00051	6.11997	6.37	6.32967	6.05924	6.32636	6.31289	6.04912	5.91146	6.16767
PRPS2	P11908-2	6.49255	6.07278	6.99659	6.10512	6.69371	6.71676	6.42201	6.5649	6.23642	6.16214	6.52883	6.2768	5.90329	5.73118	5.59909	5.49785
CALD1	Q05682-4	5.82712	5.93046	6.34592	6.08063	5.88299	5.92866	6.04615	6.12717	6.11129	5.73641	6.16664	6.17064	7.02348	6.81809	6.99936	6.80499
FEN1	P39748	5.46195	5.88677	6.04133	5.65489	5.74814	6.46996	5.5168	6.13759	5.82899	6.23571	6.43728	6.23927	6.37442	7.70998	7.48678	6.77011
ACBD5	Q5T8D3-3	6.36813	6.11673	6.01809	6.58906	6.39806	5.9395	6.40456	5.98914	6.37597	6.10375	6.28284	6.14926	6.71153	5.90139	6.38699	6.26501
GSTO1	P78417	6.38145	6.06705	6.88366	5.87713	6.41548	6.71079	5.96217	6.74828	6.18658	6.52052	6.32966	6.62819	5.86729	5.96128	5.59092	5.86956
PSMA5	P28066	6.01746	5.11649	6.06523	5.00666	6.1184	6.06835	6.22708	6.18408	5.9763	6.29015	6.34388	6.6889	6.56511	7.16652	7.17885	6.98652
NEFH	P12036	6.7052	6.21176	5.75621	6.11729	6.39434	5.77833	6.54937	6.04758	6.34657	5.85742	6.22462	6.15676	6.99709	6.26155	6.42803	6.1679
HERC4	Q5GLZ8	6.65094	6.59676	7.00962	6.29936	6.45567	6.71586	6.26467	6.96161	6.79665	6.74496	6.902	7.01198	4.71905	4.98772	4.80199	5.08115
MRPS35	P82673	6.24236	6.50407	5.80176	6.78043	6.25633	6.05412	6.54208	6.22382	5.8891	6.07146	5.93626	6.08512	6.51842	6.19412	6.53323	6.3673
NUSAP1	Q9BXS6	5.97862	6.24735	5.54266	6.09102	6.22968	6.39591	6.58783	6.01709	6.10261	5.88618	5.99233	5.94133	6.51257	6.84646	6.5694	7.05895
PTPN11	Q06124-1	6.26409	6.41781	6.64528	6.06107	6.10649	6.45243	6.18323	6.45589	6.11053	6.52867	6.86886	6.64205	5.75447	5.9642	5.83708	5.70784
CLGN	O14967	6.30999	6.28633	5.93765	6.62338	6.06322	5.99365	6.21486	6.1129	6.11308	6.22127	6.13674	5.89781	6.59519	6.479	6.57563	6.43933
QKI	Q96PU8	5.87937	5.45736	6.07601	5.78806	6.48521	6.09669	5.66548	6.26697	6.2879	6.46214	6.4976	6.7249	6.99418	6.33105	6.49332	6.49376
RPL30	P62888	6.3834	6.40006	6.60545	6.46187	6.30687	6.46611	6.46682	6.18938	6.513	6.41545	6.74103	6.29022	5.73899	5.72838	5.87984	5.41315
TLN2	Q9Y4G6	6.17871	6.24467	6.45935	5.97804	6.00442	6.57409	6.15653	6.34153	6.21327	6.48461	6.30172	6.40602	6.27725	6.25606	5.99889	6.12486
RRM2	P31350-2	6.75065	6.24782	7.11219	5.91755	6.71901	6.69659	6.1189	6.64065	6.31761	6.20616	6.54068	7.08545	5.59582	5.56971	5.28659	5.19462
FMNL2	Q96PY5-3	6.34161	6.48097	6.3251	6.24644	6.02788	6.18391	6.36161	5.97763	6.50891	6.24736	6.33988	6.22821	6.23469	6.12086	6.42025	5.95468
MTHFD2	P13995	6.51654	6.2684	5.84387	6.5261	6.84967	6.17179	6.58473	6.58394	6.50837	5.77878	6.18242	5.96458	6.31051	5.71145	6.02098	6.17785
CDC73	Q6P1J9	6.24298	6.25687	6.10751	6.71528	6.09768	5.64659	6.39241	5.77854	6.41246	5.92754	6.35206	6.01502	6.68642	6.16115	6.70764	6.49984
BCAT2	O15382	6.51641	6.08763	5.89673	6.08625	6.71413	6.08229	6.3815	6.09493	5.95837	6.12533	5.92057	5.75805	6.66978	6.50798	6.60898	6.59107
EPS8	Q12929	6.19764	6.28267	6.41526	6.21599	6.07049	6.19037	6.03112	6.2132	6.32737	6.75221	6.50357	6.28523	6.07618	6.37271	6.1114	5.95461
SEL1L	Q9UBV2	6.69798	6.64656	5.76607	6.79351	6.48224	6.26396	5.90364	6.30318	6.14056	6.06471	5.90343	5.74437	6.29574	6.07516	6.77697	6.14192
VPS51	Q9UID3	6.43406	6.62296	6.7627	6.00344	6.48392	6.49433	6.25248	6.57709	6.37205	6.36724	6.63496	6.44655	5.79039	5.87885	5.53876	5.34023
CCDC124	Q96CT7	6.50857	6.36332	6.92185	6.20137	6.41749	6.17436	6.63239	6.43706	6.36752	6.34675	6.6328	6.34685	5.61315	5.84883	5.80773	5.37995
MRTO4	Q9UKD2	6.39871	6.42993	6.65436	6.47722	6.09946	6.15613	6.09249	6.14624	6.02701	6.2309	6.30144	6.17353	5.99391	6.41613	6.2476	6.15496
STT3B	Q8TCJ2	5.98934	6.28204	5.79866	6.57863	5.83335	5.8898	6.42871	6.01695	6.18017	6.07627	5.62912	5.92742	6.57136	7.01404	6.92844	6.85571
STT3A	P46977	6.47624	6.34856	6.01042	6.5109	6.20453	6.04491	6.41586	5.75204	5.93747	6.11293	6.00553	5.71476	6.59419	6.46042	6.91361	6.49764
YES1	P07947	6.36535	6.0908	6.2043	6.51151	6.27169	6.41711	6.46268	6.02463	6.162	6.25093	6.11263	6.01126	6.35138	6.16057	6.16092	6.44225
PSMB1	P20618	6.41697	6.6748	6.02377	6.27075	6.73224	6.88552	6.21832	7.04772	5.99664	5.60799	6.35233	6.00034	5.78925	6.07075	5.87155	6.04106
TACC2	O95359-3	6.23712	5.99228	6.59929	5.84526	6.67347	6.49439	6.15064	6.28633	6.54119	6.32165	6.70124	6.14658	6.35704	6.22267	5.85191	5.57894
AP1B1	Q10567-3	6.24799	6.26386	6.68464	6.22903	6.02634	6.73147	6.34248	6.63863	6.21173	6.40741	6.47048	6.40471	5.80176	5.75768	5.84499	5.93681
WRN	Q14191	6.24943	6.43426	6.20065	6.71446	6.07993	6.10488	6.1712	6.16765	6.12083	6.21802	6.19273	6.43135	6.37204	5.99722	6.25616	6.28921
UBAP2	Q5T6F2	6.15052	6.1744	6.93692	6.02973	6.27919	6.69695	6.09019	6.60796	6.59296	6.69532	7.13235	7.09463	5.61399	5.55416	4.95751	5.39322
DIEXF	Q68CQ4	5.94822	5.91469	5.90101	6.28703	6.1584	5.89713	6.28507	5.9498	5.963	5.84609	6.09803	6.13174	7.02947	6.61725	7.05014	6.92293
CENPE	Q02224	7.09975	6.86545	7.68018	7.05871	5.99604	6.38081	6.59915	6.46746	6.97575	7.10202	7.118	7.23981	4.33983	4.41357	4.21773	4.44575
SPDL1	Q96EA4	5.60986	5.58137	6.12447	5.45493	6.09918	6.52178	6.08162	6.23895	6.15155	6.01743	6.21131	6.09663	7.60012	6.67248	6.96484	6.5735
DTX3L	Q8TDB6	6.28633	6.29249	6.68111	6.25343	6.10643	6.26495	6.21264	6.44729	6.57309	6.83389	6.75235	6.74444	5.39051	5.74732	5.64758	5.76613
GOLGA5	Q8TBA6	6.56736	6.56789	5.96688	6.47867	6.10622	6.02328	6.11013	6.00002	6.16949	5.96665	6.18088	6.01133	6.72121	6.36158	6.51652	6.25189
FBLL1	A6NHQ2	6.23272	6.32324	6.0155	6.91508	5.811	5.79689	6.4219	5.89701	6.4014	6.15746	6.13191	6.23853	6.43493	6.08989	6.49152	6.64103
DDX50	Q9BQ39	6.25505	6.40851	5.8208	6.80947	6.11258	5.62911	6.28284	5.93484	6.17223	6.06337	6.27257	6.05413	6.33227	6.55059	6.92893	6.37271
ACADM	P11310-2	6.40289	6.29554	5.81186	6.22101	6.47444	6.13628	6.19891	6.14409	6.28439	6.01533	5.94529	5.99297	7.09529	6.20923	6.48173	6.29076
NELFB	Q8WX92-2	5.73194	5.96624	5.86519	5.79185	6.02623	6.12601	6.16207	6.20387	6.21978	5.94611	5.85043	6.03855	7.14037	6.93665	7.11142	6.88329
ARFGEF2	Q9Y6D5	6.80799	6.60333	6.74248	6.67234	6.13712	6.35624	6.10691	6.57595	5.96452	6.41966	6.46244	6.34073	5.66604	5.92541	5.78359	5.43525
EED	A0A5F9ZI63	6.16731	6.3626	6.25431	6.97039	5.72923	5.98223	6.30878	5.92248	6.36807	6.55261	6.45732	6.54479	5.90442	5.91214	6.2872	6.27611
IPO8	O15397	5.65074	5.76712	6.2029	5.63431	5.76747	6.18668	5.73048	6.2721	6.10246	6.26636	6.20648	6.35809	6.77337	7.02163	7.07841	6.98138
MICAL2	O94851	6.49091	6.38244	5.95676	6.63534	6.2551	5.99094	6.49684	5.92813	6.05739	5.7386	5.78722	5.92466	6.61824	6.32573	6.72494	6.68674
RSBN1L	Q6PCB5	6.08495	6.47918	5.82972	6.80993	6.10352	6.15688	6.37249	5.75793	5.93393	6.15641	5.95318	5.9348	6.02549	6.8742	6.84475	6.68265
SAFB	Q15424-3	6.44434	6.57748	6.06749	6.59211	6.19207	5.9363	6.4466	5.91718	6.51487	6.96195	6.0935	5.9277	6.72752	5.95077	6.42627	6.22936
NELFA	Q9H3P2	5.85	5.98918	6.20981	5.81121	6.07897	6.09554	6.05238	5.92854	6.46995	6.14662	6.38347	6.05034	7.04202	6.50826	7.04521	6.33849
PTK7	Q13308-6	6.51116	6.55591	6.20151	6.66505	5.67589	5.81328	6.22663	5.90008	6.29376	6.2768	6.04427	6.46936	6.24627	6.24674	6.39116	6.4821
CYCS	P99999	6.8199	6.59767	6.11913	6.4273	6.37909	5.70231	6.52118	5.86811	6.16967	5.78868	5.90697	5.67963	6.83436	6.09067	6.53529	6.56003
HECTD4	J3KPF0	6.56041	6.46374	6.93535	6.33356	5.97756	6.51348	6.09967	6.48476	6.63255	6.74591	6.77844	6.71718	5.39569	5.77301	5.26535	5.32336
PIEZO1	Q92508	6.41707	6.41591	6.01985	6.81738	6.0175	5.90999	6.03268	6.09092	6.32966	5.99579	6.03146	6.15719	6.22558	6.35631	6.49191	6.6908
VPS4B	O75351	6.29772	6.31288	6.77552	6.15105	6.39718	6.9762	5.91548	6.86654	6.24128	6.60565	6.46745	6.78743	5.84617	5.83242	5.12627	5.40075
ANKRD17	O75179	6.32366	6.35043	6.79381	5.9821	6.20156	6.48751	6.24699	6.50556	6.58593	6.77515	6.68299	6.65132	5.47474	5.69408	5.47432	5.76986
OTUD4	Q01804	6.10631	6.27805	6.40522	6.19686	6.26417	6.50736	6.43192	6.27503	6.55115	6.86442	6.48152	6.5506	5.61931	5.98424	5.68455	5.7993
NCSTN	Q92542	6.3516	6.50942	6.1105	6.47334	6.18755	6.14192	6.26092	6.04587	6.36559	6.24765	6.27611	6.08471	6.36185	6.26276	6.35134	5.96888
RBM26	A0A087X0H9	5.77634	6.29937	6.14843	6.27881	5.99295	5.91734	6.0944	5.95681	5.92875	6.05616	5.75908	6.06079	6.98934	6.61771	7.1881	6.93564
CC2D1B	Q5T0F9	5.93093	6.07315	6.37383	5.69497	6.02541	6.55111	6.07853	6.41666	6.15424	6.51264	6.43259	6.44009	6.3728	6.69178	6.11157	6.1397
ARHGDIA	P52565	6.00555	5.9016	6.92084	6.05312	6.40591	6.89565	6.35616	6.82238	6.3636	6.33455	6.61843	6.59848	5.73536	5.71068	5.77852	5.49919
TM9SF2	Q99805	6.68462	6.68372	6.1551	6.65455	6.61879	6.12825	6.35134	5.86361	6.20371	5.82726	6.07496	5.87109	6.41966	6.19496	6.30665	5.96174
SPTBN2	O15020	6.44492	6.19331	6.19053	6.24196	6.00839	5.81977	6.14273	5.93343	6.25723	6.02287	6.20498	6.2894	6.75452	6.31526	6.65927	6.52145
NLN	Q9BYT8	6.5203	6.45977	6.3059	6.444	6.36745	6.23676	6.3042	6.39563	6.39866	6.2262	6.19908	6.17133	6.11928	5.82707	6.04809	5.97629
POFUT2	Q9Y2G5	6.39904	6.53415	6.11299	6.62009	6.14398	6.06737	6.25691	6.15564	5.91614	5.93451	6.02138	5.93149	6.40815	6.29242	6.66979	6.53596
MTAP	Q13126	6.24514	5.89231	6.87121	6.01895	6.28813	6.74928	5.93956	6.62606	6.23049	6.29727	6.72683	6.48703	6.09048	5.77925	5.98208	5.77595

DMAP1	Q9NPF5	6.43574	6.65372	6.02184	6.70597	6.14848	5.83155	6.46057	6.03332	6.28584	6.28773	6.18243	6.133	6.14368	6.40724	6.20408	6.06481
ACAP2	Q15057	6.66558	6.21089	6.89971	5.94607	6.41701	6.91956	6.09998	6.49685	6.34822	6.4771	6.32342	6.2719	5.74867	5.67271	5.74145	5.76085
PPP1R13L	Q8WUF5	6.57482	6.08418	6.7354	6.08925	6.4542	6.72968	6.06502	6.70067	6.76935	6.3041	6.81442	6.7008	5.53132	5.78958	5.38936	5.26788
DNAJC8	O75937	5.55719	5.77165	6.11927	5.6109	6.23825	6.37747	6.44461	5.74675	6.04151	6.07042	6.44719	6.25921	6.66963	7.19193	6.96188	6.49214
PCCB	P05166-2	5.98932	6.05583	5.61953	6.41286	6.21846	6.0193	6.48006	5.95142	5.79458	5.99231	5.59849	5.78533	7.41003	6.65026	7.05373	6.96848
PTGES2	Q9H7Z7	6.63845	6.13081	5.91552	6.62326	6.19033	6.03071	6.20516	5.97624	5.96152	6.09615	6.22949	6.13009	6.8541	6.33059	6.44823	6.23936
RPL34	P49207	6.3915	6.30622	5.9512	6.00409	6.30638	6.17771	6.70195	6.44541	7.1003	5.97309	6.32793	6.2515	5.77039	6.25046	5.91943	6.12246
RAB10	P61026	6.21497	6.58961	6.29571	6.59782	6.08943	5.94802	6.34302	5.97525	6.14634	6.09565	5.80548	6.08704	6.40553	6.18603	6.73545	6.48464
RAB14	P61106	6.19722	6.54138	5.98682	6.25826	6.37371	6.34799	6.28124	6.25725	6.23266	6.15665	6.1746	5.88261	6.11078	6.37554	6.47116	6.35214
KDM1B	Q8NB78	6.23807	6.71436	6.3694	6.4488	6.36096	6.27998	6.19771	6.05312	6.30646	6.14613	6.09438	6.17107	5.99712	6.29	6.25319	6.07925
PEPD	P12955	6.52005	5.83114	6.8318	5.37763	6.64406	6.56272	6.06951	6.2932	6.39381	7.11755	6.88874	6.46405	6.26306	5.89536	5.51434	5.33299
YY1	P25490	5.69303	6.01836	6.35022	6.15933	6.19556	6.05127	5.90939	6.19113	6.36082	6.50988	6.65782	6.4103	6.33983	6.47969	6.49466	6.17872
PSME2	Q9UL46	6.19415	5.97816	6.62036	5.51681	6.07132	6.47213	5.71182	6.34691	6.03932	6.49029	5.97944	6.35561	6.75446	6.44793	6.6351	6.38621
ARHGAP12	Q8IWW6	6.00797	6.24968	6.49004	5.99074	6.46322	6.20011	6.03087	6.59617	6.51831	6.21336	6.25579	6.42709	6.24984	6.41581	5.89383	5.99719
RFTN1	Q14699	6.45331	6.44574	6.31711	6.47829	6.41632	6.1717	6.33748	6.0292	6.13957	5.9442	6.3038	6.15685	6.20541	6.14052	6.34831	6.11218
NHLRC2	Q8NBF2	6.12475	6.26021	6.90198	5.98739	6.14582	6.47554	6.23951	6.64603	6.36567	6.27186	6.27242	6.59996	5.97734	6.03959	5.7917	5.90023
RCC2	Q9P258	5.70075	5.94005	6.32709	5.62916	5.77428	6.27194	5.99522	6.10651	6.43618	6.16566	6.11709	6.01156	6.39157	7.36724	7.13107	6.62864
SIRT1	Q96EB6	5.40868	5.90841	6.01583	5.66285	5.61716	6.07879	5.77911	5.95974	5.98348	6.20734	6.63619	6.35431	7.17792	6.95981	7.05909	7.19129
RPS15A	P62244	6.36354	6.27406	6.61386	6.263	6.45531	6.26017	6.73717	6.14101	6.7969	5.90726	6.1689	6.21716	5.46775	6.14969	5.96688	6.21733
H2AC6	Q93077	6.02542	5.95773	5.62159	6.71759	5.73397	5.78394	6.68583	6.20811	6.06405	6.15836	6.10903	6.03761	6.38404	6.54716	6.69729	7.26829
GSTK1	Q9Y2Q3	5.91031	4.72577	5.4476	5.26674	6.13441	5.28491	5.99787	6.36006	5.3388	6.09826	5.39435	6.50711	6.59755	8.30077	8.10064	8.53485
RPL22	P35268	6.30434	6.46415	6.37069	6.30289	5.75086	6.44195	6.42097	6.30854	6.60516	6.54361	6.63182	6.29641	5.77965	5.70376	6.2155	5.8597
AIP	O00170	5.84077	5.6952	6.6877	5.46067	6.28046	6.57661	5.92783	6.58589	6.32352	6.37363	6.52881	6.4643	6.72128	6.29664	6.203	6.03367
MICU1	Q9BPX6-3	6.37581	6.55018	5.79213	6.72017	6.13619	6.25393	6.33086	6.04246	5.94855	6.13003	5.93508	6.10957	6.40311	6.25734	6.4853	6.52929
RCC1L	Q96I51	6.46066	6.56116	6.0771	6.75669	6.23205	6.26496	6.55761	6.19355	6.15185	6.21665	6.20018	5.61142	6.1525	6.04295	6.40019	6.12048
ALDH4A1	P30038	6.4871	6.32473	5.94117	6.559	6.39489	6.11284	6.13731	6.09794	6.08014	6.35506	6.29243	5.96106	6.92225	5.89434	6.3134	6.12633
PMS1	P54277	5.50949	6.05836	6.6925	5.84705	5.5594	6.35031	6.00517	6.00991	6.09985	6.27112	6.81013	6.72949	6.37095	6.4448	6.54791	6.69356
PGAM5	Q96HS1	6.41194	6.47225	5.86389	6.86735	6.38423	6.12745	5.97912	6.24109	5.95812	5.91352	5.88329	6.1025	6.40812	6.31682	6.48247	6.58781
USP47	Q96K76	6.34507	6.27344	7.09007	6.02806	6.55329	6.89282	6.28726	6.71499	6.18657	6.42634	6.60429	6.55149	5.60005	5.59875	5.41511	5.43241
RAVER1	E9PAU2	5.87771	5.98668	5.96352	6.16864	6.14364	6.07075	6.00894	6.28489	6.15845	6.08837	6.32318	6.24636	6.97611	6.61804	6.62594	6.45878
XRCC1	P18887	5.70827	5.94026	5.73339	5.67203	5.49187	5.97677	5.90044	5.878	6.03305	6.43641	6.47279	6.24249	6.91521	7.3179	7.27393	7.00718
RANBP6	O60518	5.93293	6.21966	6.18182	6.182	6.07351	6.14823	6.10171	6.0718	6.21942	6.21225	6.28288	6.07097	6.61115	6.40826	6.78981	6.49362
FDFT1	P37268	6.57005	7.2434	6.58371	7.28241	6.31253	6.30869	6.62131	6.4774	6.32828	6.5412	6.29866	6.17833	5.17134	5.36971	5.35512	5.35785
CDC42BPA	Q5VT25-6	6.29903	6.35979	6.66339	6.19332	6.39971	6.53316	6.32369	6.42501	6.27904	6.49041	6.66425	6.43369	5.84399	5.92058	5.67379	5.49715
BRD8	Q9H0E9-2	6.72489	6.69943	6.50504	7.35362	6.04162	6.14916	6.32954	6.13749	6.24297	6.50822	6.3957	6.74849	5.61503	5.4929	5.23227	5.82363
LRSAM1	Q6UWE0	6.42766	6.21107	6.93591	6.18904	6.11807	6.66664	6.45273	6.87202	6.52196	6.35674	6.72239	6.4353	5.75843	5.68781	5.46532	5.17891
DNMT3A	Q9Y6K1	6.90992	6.59727	5.99818	6.65031	6.28634	6.19356	6.65016	6.05527	6.28603	6.32419	6.09161	6.00735	6.1637	5.79308	6.06132	5.93172
PRIM2	P49643	5.62363	6.1889	6.31095	6.07348	6.31604	6.48645	6.01976	5.87004	6.16124	6.24564	6.41678	6.37979	6.54099	6.33511	6.45724	6.57398
SCFD2	Q8WU76	6.35924	6.51422	5.87789	6.27403	6.25207	6.18603	6.36447	5.7867	6.04336	6.11715	5.94889	6.17396	6.65698	6.51287	6.47561	6.45653
CSNK2A2	P19784	6.04036	6.28864	6.60881	6.45093	6.06588	6.3676	6.17093	6.19291	5.80883	6.04228	6.33743	6.25931	6.304	6.45814	6.38544	6.21854
CTDP1	Q9Y5B0	5.86966	6.27667	6.17713	6.03308	5.70575	6.04983	5.81301	6.27695	6.13777	6.16693	5.9808	6.21943	6.7111	7.14783	6.73865	6.69541
PUM1	Q14671-3	6.58857	6.57731	6.59066	5.95528	6.20458	6.52586	6.42654	6.40182	6.30207	6.12266	6.48098	6.34418	5.78085	5.81743	6.03137	5.84985
ABCD3	P28288	6.26468	6.46493	5.92148	6.83851	5.97843	5.76236	6.24625	5.89734	6.05274	5.87847	6.43872	6.28286	6.3831	6.20492	6.77738	6.60784
SNX6	Q9UNH7	6.10218	6.30411	6.56613	5.97125	6.50491	6.69046	6.10427	6.61662	5.96623	6.5195	6.44981	6.36139	6.22458	6.05541	5.79216	5.768
PDP1	Q9P0J1-2	6.55961	6.1553	5.87932	6.3692	6.44296	6.12321	6.25631	6.19985	6.12231	5.7683	5.873	5.89329	6.88365	6.24774	6.76707	6.45887
SNX1	Q13596-3	6.46152	6.23638	6.81948	6.0203	6.56557	6.58801	6.1311	6.61525	6.4727	6.27471	6.45728	6.36548	5.95253	5.74723	5.78596	5.5065
SNX2	O60749	6.14774	6.68401	6.69013	6.54524	6.21718	6.66814	6.07486	6.61235	6.04662	6.1856	6.49771	6.64351	5.92555	5.83527	5.90563	5.32047
SPATA5L1	Q9BVQ7	6.23413	6.09478	6.85795	6.15628	6.41934	6.53104	6.30416	6.38115	6.60326	6.24344	6.63434	6.82313	5.80279	5.76679	5.68855	5.45887
TCEA1	P23193	6.03586	5.70121	6.44054	5.54035	5.93453	5.96249	6.17637	6.09486	6.36864	6.1862	6.50771	5.95323	6.81344	6.97094	6.85597	6.45767
DUS3L	Q96G46	5.48965	5.51696	6.01751	5.15352	5.62405	6.26082	5.93564	5.96512	6.0193	6.07898	6.33902	6.06329	7.17635	7.68895	7.65904	7.01179
INTS2	Q9H0H0	6.46898	6.06247	5.98883	6.77358	6.33642	5.96464	6.13438	5.50319	5.8424	6.30871	6.13202	6.15292	6.37379	6.41792	6.73689	6.80287
RECQL5	O94762	6.07281	6.29015	6.18648	6.36047	6.10511	6.26111	6.16734	6.00364	6.46298	6.53237	6.37512	6.30866	6.31755	6.15723	6.31692	6.08208
PSMB2	P49721	5.67492	5.21988	5.96926	5.33338	6.13864	5.99106	6.52827	6.4507	5.80483	5.65475	5.70563	6.45257	6.04311	7.76817	7.2111	8.05372
EMD	P50402	6.32231	6.47945	5.48046	6.48955	6.34545	6.03231	6.18372	6.42822	5.91625	6.19988	5.59291	5.9609	6.91053	6.59009	6.41009	6.65789
PDCD4	Q53EL6	6.11535	6.57939	5.93457	5.32251	6.25615	6.75508	5.76627	6.22373	5.89126	7.12713	6.05422	5.92795	6.37422	6.68944	6.88079	6.10194
CGAS	Q8N884	6.43863	6.37958	6.13231	6.82436	6.26934	6.25497	6.41353	6.10653	6.44036	6.21849	6.24368	6.31738	5.89559	5.94401	5.9673	6.15394
DDX20	Q9UHI6	6.29143	6.28104	6.60702	6.10372	6.28287	6.57524	6.20183	6.72005	6.26917	6.27225	6.49413	6.64665	5.83881	5.99689	5.74558	5.67331
SMARCB1	Q12824	6.13707	6.23664	6.25103	6.36555	6.37006	5.8308	6.44565	6.03203	6.31973	6.03161	6.02756	6.03297	6.61226	6.28057	6.58424	6.44222
QSER1	Q2KHR3	6.04798	6.36141	6.48557	6.67336	6.12742	5.99389	6.35859	5.97451	5.98509	6.38485	6.20084	6.45068	6.20536	6.03454	6.16307	6.55282
PLAU	P00749	5.50604	5.3988	5.37347	5.6985	6.01007	6.09446	6.65464	6.20077	5.81497	5.64317	5.90528	5.68253	8.02477	7.29188	8.01174	6.68888
DAG1	Q14118	6.64725	6.43728	6.16988	6.67676	5.96387	5.9958	6.19204	6.09373	5.9992	6.48345	6.39711	6.50549	6.22054	6.08622	6.08923	6.04216
PANK4	Q9NVE7	6.36053	6.07513	6.95185	5.99785	6.4042	6.86022	6.30249	6.8824	6.5882	6.58474	6.4262	6.3883	5.57088	5.72595	5.40642	5.47463
COG5	Q9UP83-2	6.27942	6.00606	6.35547	6.37596	6.23241	6.4976	6.30391	6.2463	6.46955	6.44945	6.38035	6.38492	6.39554	5.94396	5.99178	5.68732
EEF1AKNMT	Q8N6R0	6.42615	6.29962	6.69789	6.21549	6.21518	6.53555	6.43261	6.55681	6.55312	6.42546	6.77222	6.56176	5.61434	5.7311	5.46055	5.50215
TUT4	Q5TAX3	6.08189	6.22214	6.95503	6.03372	5.93983	6.44755	6.21189	6.35478	6.3163	6.85145	6.63137	6.5053	5.59995	5.99344	5.89666	5.95871
ZMYM4	Q5VZL5	6.19083	6.48542	6.20975	6.91272	6.21196	6.14116	6.30334	5.79353	6.10938	6.47594	6.28199	5.91887	6.38991	6.06622	6.35051	6.15849
NFRKB	Q6P4R8-2	6.48205	6.65206	6.26621	6.66218	6.2107	5.92078	6.29952	6.14784	6.18556	6.28688	6.20086	6.15114	6.28145	6.04299	6.10696	6.10281
MIS18BP1	Q6P0N0	6.67933	6.56334	6.22969	6.97959	6.42556	6.00367	6.54404	6.25789	6.61422	6.49736	6.14732	6.38339	5.57706	5.49903	5.6989	5.89961
ATP6V1B2	P21281	6.27147	6.8164	6.43163	6.29988	6.1935	6.63611	6.34937	6.30134	6.6018	6.94164	6.87595	6.78494	5.13035	5.29213	5.2364	5.83708
VPS8	Q8N3P4	6.07042	6.24685	6.42385	6.15168	6.08499	6.27297	6.09923	6.22279	6.23961	6.65797	6.36232	6.26563	6.1956	6.32043	6.16398	6.22168
TEX10	Q9NXF1	5.96006	6.11398	5.59772	6.72058	5.7699	5.91251	6.34354	6.02595	6.10686	6.05781	6.13098	6.1782	6.55193	6.90377	6.58293	7.04327
SLU7	O95391	6.24024	6.34657	6.23107	6.5995	6.03828	5.76999	6.1583	6.08613	6.19254	6.1899	6.21607	6.16512	6.10589	6.51501	6.59071	6.55467
PRIM1	P49642	5.91555	6.10777	6.36452	5.76712	6.14139	6.46464	6.079	6.32324	6.23564	6.33499	6.43337	6.37271	6.31628	6.34494	6.55414	6.2447
TGM2	P21980	6.31611	5.72443	7.21956	5.6985	6.80043	6.56292	6.19355	6.49109	6.47395	6.43255	6.70365	6.32855	6.08398	5.64151	5.80428	5.52494
PPAT	Q06203	6.01859	6.24101	6.92571	5.91024	6.44125	6.67969	6.20577	6.58258	6.24003	6.3789	6.63432	6.20916	5.88968	5.91766	5.86976	5.85565
TM9SF4	Q92544	6.14099	6.52953	5.97183	6.65381	6.11777	5.81519	6.30619	5.99183	6.15824	6.16628	6.2303	6.05637	6.2871	6.3679	6.68936	6.5173
NDUFA10	O95299	5.89699	6.12251	5.73826	6.56608	6.47354	6.00349	7.00678	6.12371	6.28772	5.81189	5.73829	5.8034	6.43919	6.45101	6.631	6.90613
PLCB3	Q01970	6.16217	6.75768	6.3987	6.01482	6.1659	6.3115	6.27735	6.34617	6.12462	6.18412	6.51101	6.71127	6.03133	6.3692	5.87045	5.76371
TRIM24	O15164	6.42176	6.6389	5.87675	6.82972	6.22208	5.97581	6.2417	6.13438	6.13159	6.3413	6.2943	6.39384	6.1172	5.9959	6.16671	6.21805
POLDIP3	Q9BY77	6.27793	6.10478	6.18353	6.6955	6.20662	5.85766	6.8168	5.7826	6.3888	5.76766	5.95432	5.93745	6.27628	6.50611	6.59127	6.65267
PPAN	Q9NQ55-3	6.30591	6.23559	5.82853	6.7328	6.00452	5.78422	6.53412	6.2813	6.12619	6.01984	5.92058	6.09265	6.21227	6.64853	6.70207	6.57088
WDR19	Q8NEZ3	6.1361	6.22634	6.4831	5.90098	6.29228	6.52439	6.32186	6.20948	6.20282	6.49026	6.6159	6.17197	6.35331	6.30213	6.05228	5.71681
PARN	O95453	6.20514	6.35094	5.96658	6.63339	6.25198	6.10987	6.24612	6.10966	6.15102	6.04247	6.01624	5.96649	6.54287	6.34613	6.49556	6.56553
FHL2	Q14192	6.25346	6.03108	6.65113	6.15171	6.50468	6.6921	6.12659	6.20407	6.38587	6.06513	6.87892	6.39255	6.18747	5.7546	5.86094	5.8597
TM9SF3	Q9HD45	6.40309	6.91836	6.32383	6.70827	6.35327	6.28255	6.477	5.64566	5.79293	5.97271	5.91762	5.70388	6.67083	6.19311	6.37091	6.26601
NT5C2	P49902	6.35255	6.24348	6.82949	6.09186	6.32503	6.66878	5.92989	6.96888	6.01032	6.46719	6.40195	6.57771	5.54041	5.97561	5.70552	5.91133
RRP9	O43818	6.17565	5.58045	6.01806	6.61855	6.10124	5.99576	6.33706	5.5915	5.87891	6.10875	6.20092	6.15372	6.90961	6.79222	6.55979	6.97783
GNE	Q9Y223-2	6.12179	6.19751	6.70742	6.05324	6.26842	6.79603	6.16673	6.75723	6.00238	6.33636	6.65745	6.28726	5.94633	6.14584	5.82237	5.73364
RBBP4	Q09028	6.16135	5.86676	6.03537	6.33591	6.11697	6.02902	5.90753	6.34385	6.36961	6.02948	6.41217	6.50416	6.99599	6.16006	6.1477	6.58406
CWC22	Q9HCG8	6.37461	6.21222	6.2855	6.56666	6.18937	5.8609	6.14858	5.79426	6.24634	6.16641	6.15461	6.01098	6.63166	6.20207	6.76081	6.39502
PAK1IP1	Q9NWT1	6.05443	6.14284	5.75322	6.67799	6.11609	5.90151	6.2345	5.7656	6.03935	5.99473	5.84647	5.93343	6.64388	6.49135	7.40303	7.00159
LACTB	P83111	6.21506	6.59831	5.78148	6.7798	6.12314	6.02357	6.1682	5.99757	6.04947	6.1037	5.9289	6.32212	6.51858	6.26585	6.47636	6.6479
ESS2	Q96DF8	5.96867	5.9651	6.37913	5.98273	5.76461	6.05126	6.23229	6.14268	6.22554	6.25183	6.33454	6.50656	6.706	6.61262	6.39714	6.47931
MRPL44	Q9H9J2	6.39053	6.45866	6.01429	6.78373	6.1937	5.98488	6.48908	6.05763	5.98613	5.96096	6.00618	6.11323	6.52218	6.25557	6.33125	6.45201
ILK	Q13418	6.44498	6.25461	6.81309	5.96005	6.54657	6.49231	6.27878	6.53762	6.53416	6.63758	6.78978	6.5953	5.82113	5.47722	5.40891	5.4079
RPL23	P62829	6.09035	6.08678	6.27739	6.22681	6.12659	6.13491	6.30054	6.32647	6.23378	6.31476	6.65673	6.46964	6.04817	6.54469	5.98262	6.17976
DCAF13	Q9NV06	5.99835	6.00081	6.15485	6.81201	6.16554	6.21359	6.5268	6.05248	6.18559	6.0453	6.24359	5.90636	6.37277	5.91485	6.77506	6.63203
RHOC	P08134	6.11781	6.23969	6.20372	6.29629	6.18058	6.21974	6.34442	6.25943	6.1824	6.09269	6.26432	6.238	6.36859	6.45214	6.26283	6.27735
DRG1	Q9Y295	6.40318	6.20678	6.60582	5.98667	6.21837	6.36648	6.22179	6.29137	6.61331	6.34023	6.4843	6.39642	5.90096	5.9616	6.08459	5.91813
CAPZA1	P52907	6.3902	6.58326	6.54752	6.44073	6.44009	6.52921	5.92385	6.32924	6.1033	6.12329	6.34327	6.36423	6.13109	5.89336	6.08457	5.77278
FASTKD2	Q9NYY8	6.55621	6.42929	6.30953	6.94792	6.47565	5.97535	6.60299	5.9405	6.48896	5.93635	5.9924	6.01074	6.23937	5.58393	6.27436	6.23644
TFB2M	Q9H5Q4	6.47461	6.39458	5.99445	6.97438	6.47148	6.20223	6.57663	6.22901	6.07981	5.73687	5.92213	6.13373	6.27596	5.9128	6.29294	6.3284
CENPC	Q03188	6.07089	6.02964	5.80698	6.54506	6.13765	5.79046	6.29424	5.74182	6.16525	6.14948	5.83367	5.85084	6.55295	6.99242	7.11012	6.92851
DNPEP	Q9ULA0	6.30118	6.2597	6.47217	5.9539	6.24094	6.7739	6.01129	6.41041	6.31996	6.32403	6.42643	6.52085	6.01657	6.1603	5.95535	5.85303
TMEM43	Q9BTV4	6.30716	6.55313	5.75019	6.50967	6.09609	5.94956	6.22081	6.04002	6.22857	6.02373	5.89242	5.87239	6.6425	6.45851	6.91242	6.54282

MRPL37	Q9BZE1	6.50028	6.24937	5.86742	6.73459	6.58202	6.13345	6.30282	6.24427	6.21247	6.2092	6.00929	5.87513	6.56901	6.04165	6.25955	6.20948
ZC3H15	Q8WU90	5.95215	6.14056	6.57904	5.96718	6.19841	6.69692	6.13107	6.20624	6.40953	6.71032	6.57453	6.57562	6.02748	6.24119	5.71903	5.87071
DPM1	O60762	6.34756	6.41359	5.93345	6.5965	6.34703	6.2339	6.33525	6.07541	6.00483	5.84268	5.84638	5.85925	6.63324	6.47842	6.55817	6.49434
SSBP1	Q04837	6.30497	6.24116	5.95426	6.54869	6.20272	5.98188	6.29481	6.0666	5.99301	6.01169	6.16288	5.99364	7.04788	6.34924	6.31104	6.53553
ZNF326	Q5BKZ1	6.18997	6.74135	5.94693	6.76517	6.05554	6.1988	6.20498	6.02545	5.89365	6.12974	6.07093	6.18559	6.61499	6.25778	6.37343	6.34569
TRMT1L	Q7Z2T5	6.06976	6.36209	6.2201	6.30581	6.04496	6.02581	5.91897	5.70798	6.10381	6.36826	6.30744	6.04713	6.9036	6.40014	6.5985	6.61566
BSG	P35613	6.26145	6.66322	5.68866	6.78334	6.07113	5.91783	6.36199	6.30486	6.23098	6.00896	5.49654	5.94103	6.08354	6.77611	6.51109	6.89927
SAR1A	Q9NR31	6.38128	6.21759	6.64271	6.28773	6.43541	6.31775	6.20317	6.51945	6.34372	6.42618	6.39055	6.58941	6.05031	6.03242	5.54742	5.61493
NPAT	Q14207	6.58624	6.65019	5.96689	7.02628	6.32138	6.04164	6.39606	6.29157	6.23251	5.87845	6.13109	6.11632	6.17961	6.01989	6.02913	6.13275
DNAJA2	O60884	6.32186	6.0782	6.44755	6.17956	6.07581	6.43874	6.30623	6.15144	6.4912	6.48378	6.47757	6.37913	6.14009	5.85189	6.07109	6.10585
EXOC5	O00471	6.2915	6.28322	6.47738	5.86126	6.49073	6.46955	6.05302	6.55525	6.55041	6.5756	6.31026	6.3231	6.053	6.16932	5.81755	5.71885
TRIP13	Q15645	6.40313	6.44733	6.82324	6.23976	6.49797	6.77894	6.28086	6.73861	6.64797	6.19713	6.40668	6.80181	5.46957	5.36472	5.33127	5.571
RBM34	P42696	6.22161	6.40483	6.03084	6.80154	6.07704	5.65488	6.35638	6.01308	6.04325	5.83139	5.91543	5.94488	6.2568	6.80326	6.84852	6.79626
PDIA5	Q14554	6.46396	6.76795	5.60844	6.2928	6.36761	6.14653	6.23193	6.26765	5.86595	5.75873	5.95422	5.76126	6.95594	6.40504	6.87225	6.27975
ALYREF	Q86V81	6.11874	5.80874	5.52831	6.61036	5.95693	5.65401	7.01763	6.02039	6.33341	5.55727	5.8393	5.93806	6.52475	6.85387	6.61659	7.62163
GET3	O43681	6.19626	6.18591	6.48396	5.94021	6.23033	6.69814	5.9061	6.36483	6.12909	6.46253	6.27314	6.37098	6.57646	6.04469	6.19006	5.9473
SRP14	P37108	5.76582	6.14638	5.6598	5.76616	5.99764	6.06865	5.95628	6.19873	5.89681	6.51859	5.68043	6.06233	6.80194	7.20438	7.21456	7.0615
STRN	O43815	6.0152	6.40146	6.49916	6.02072	6.38535	6.54612	6.19075	6.10001	6.31924	6.26019	6.11276	6.21787	6.23775	6.34599	6.19337	6.15406
ABR	Q12979	6.30799	6.29198	6.7511	5.69268	6.38649	6.63513	6.31559	6.54462	6.44455	6.31991	6.35583	6.18242	6.10294	6.2828	5.76339	5.62258
IDH3B	O43837	6.4839	6.71596	6.00593	6.97506	6.19884	5.94569	6.32441	6.05073	6.06444	6.1865	6.31196	6.18815	6.07306	6.2478	6.04283	6.18474
GOLGA1	Q92805	6.73063	6.51599	6.05293	6.51014	6.71408	6.20372	6.23727	6.33542	6.57406	6.16217	6.13738	6.51113	6.42408	5.53559	5.56697	5.78844
TONSL	Q96HA7	6.21896	6.13646	6.03976	6.34954	5.93171	6.02132	6.16552	6.05625	6.17904	6.09745	6.23124	6.31049	6.60048	6.36371	6.79361	6.50446
INTS10	Q9NVR2	5.98475	6.28799	6.30794	6.74186	6.26582	5.83989	6.03963	5.58492	6.2781	6.12768	6.26624	6.05237	6.67941	6.32495	6.75955	6.4589
GTF3C3	Q9Y5Q9	6.26555	6.68888	6.23468	6.5187	6.20947	6.21155	6.45238	6.65403	6.23007	6.25301	6.24109	6.16309	5.97883	6.00862	6.22382	6.26624
TIAL1	Q01085-2	6.4503	6.28487	5.88705	6.49768	6.45411	6.30921	6.09433	6.31221	6.08639	6.0768	6.22425	6.2098	6.51429	6.11265	6.23008	6.25595
YWHAH	Q04917	6.20066	6.22888	6.70982	5.90093	6.14206	6.39945	6.05382	6.45597	6.46932	6.27369	6.66212	6.42471	5.97399	6.09968	6.0125	5.99241
SCAF4	O95104	6.1088	6.59792	5.9573	6.66626	6.15391	5.86167	6.31021	6.22054	6.08742	6.03818	5.59856	5.99966	6.42354	6.35886	6.53282	6.69335
ACAD10	Q6JQN1-5	6.54926	6.34574	5.95127	6.48709	6.12379	5.92504	6.40678	6.11874	5.81304	5.90952	5.82868	6.02414	6.92861	6.63041	6.45571	6.50219
DDX58	O95786	6.28376	6.19763	6.75563	6.11463	6.14803	6.66271	6.3036	6.29856	6.12799	6.53257	6.53235	6.58865	5.91344	5.98361	6.03924	5.51758
ADSL	P30566	6.10253	6.12538	6.85839	5.95966	6.40788	6.99007	5.86961	6.68256	6.58068	6.6147	6.60787	6.53979	5.72598	5.92698	5.5428	5.46511
CDC23	Q9UJX2	6.32727	6.29837	6.58162	6.18585	6.2072	6.39688	6.27709	5.96467	6.1469	6.46384	6.04503	6.0591	6.34231	5.84389	6.4616	6.3984
BCCIP	Q9P287-2	5.92704	6.08838	6.42143	5.73895	6.19709	6.39883	5.98066	6.05936	6.18823	6.03233	6.17653	6.1147	6.65477	6.69632	6.90137	6.42403
PRKAR2A	P13861	6.29669	6.33896	6.05419	6.11652	6.57062	6.31305	6.10305	6.35419	6.30741	6.20476	6.3069	6.15101	6.34959	6.06714	6.50318	5.96275
FOCAD	Q5VW36	6.27914	6.70711	6.7056	6.25246	5.86106	6.23168	5.98426	6.67785	6.33977	6.39565	6.58132	6.73051	5.5602	5.73987	5.9296	6.02393
MECP2	P51608-2	6.22995	6.25166	5.73794	6.54971	5.98064	5.97067	6.27248	5.90837	6.04564	6.00499	5.61829	5.66423	6.68837	7.015	7.19763	6.86445
BCL2L13	Q9BXK5	6.43278	5.8886	5.726	6.31853	6.01914	5.71544	6.12357	5.79533	5.91081	5.93782	6.02078	6.07082	7.05151	6.94375	6.92967	7.11547
TGFBI	Q15582	7.86193	6.41186	3.73245	7.80152	4.84786	5.65602	8.83628	5.79504	7.33048	5.98733	5.09951	5.8852	7.03701	4.45234	6.33783	6.92733
AIMP2	Q13155	6.21377	5.52368	6.59675	5.30451	6.49622	6.07911	5.82545	6.64292	6.07636	6.59078	6.60747	6.98158	6.47318	6.3341	6.18216	6.07196
GOLIM4	O00461	6.25674	6.45459	5.73121	6.27837	6.36586	6.37662	6.441	6.18114	6.30862	5.98289	5.77964	5.8291	6.73685	6.32651	6.54424	6.40661
ADD1	P35611-3	6.10336	6.19053	6.3473	5.7732	5.9346	6.38436	6.05074	6.29275	6.07375	6.41042	6.16233	6.08268	6.76763	6.67602	6.42511	6.32522
PC	P11498	6.1712	6.37676	6.00208	6.4409	6.14367	6.00685	6.14564	5.70161	6.22575	6.10731	6.28737	6.08578	6.8938	6.30859	6.59872	6.50398
BICD2	Q8TD16-2	6.27082	6.11864	6.66446	6.05093	6.47921	6.64581	6.1963	6.45123	6.82159	6.49207	6.81754	6.64588	5.95429	5.4896	5.47856	5.42307
ITGA2	P17301	6.13852	6.49514	5.84309	6.23747	6.49701	6.23794	6.30142	6.06993	5.96246	6.05986	5.90815	5.72735	6.85876	6.52165	6.76934	6.37193
GIGYF2	Q6Y7W6	6.58172	6.65684	6.82347	6.34412	6.50947	6.66143	6.08065	6.27215	6.5622	6.4507	6.60445	6.72928	5.66994	5.37545	5.32886	5.34927
TBC1D9B	Q66K14	6.37749	6.2276	7.01166	6.10549	6.13826	6.5554	6.18541	6.33926	6.53831	6.29166	6.65478	6.78754	5.74193	5.79841	5.64568	5.60112
NUFIP2	Q7Z417	6.33064	6.30669	6.3206	6.2094	6.47635	6.34127	6.2787	5.93049	6.64429	6.26416	6.28484	6.45758	6.0967	5.96268	5.80765	6.28794
MAP2	P11137	5.96565	6.22925	6.68476	5.58497	5.95547	6.67487	6.02948	6.55282	6.07782	6.6835	6.41308	6.23928	6.37146	6.84063	6.05087	5.64608
FNBP1	Q96RU3	6.75676	6.54924	7.28022	6.42034	6.65911	6.6325	6.38133	6.67401	6.37102	6.59817	6.56784	6.42473	5.3706	5.23374	4.98001	5.10039
ABCC4	O15439	6.42094	6.36849	6.11652	6.84249	6.0456	6.02628	6.35933	6.06962	6.33722	6.1564	5.74231	6.0201	6.15534	6.2421	6.18954	6.40772
PAPSS2	O95340-2	5.51709	5.75803	6.19783	5.34716	5.6484	6.41408	5.97597	6.17149	5.91231	6.32737	6.31204	6.35706	6.55436	7.30018	7.22299	6.98363
TBC1D15	Q8TC07-3	5.71457	5.92249	5.99307	5.68218	5.93213	6.32811	5.93078	6.24562	6.27996	6.26514	6.21622	6.43644	6.93364	6.56186	6.7831	6.77469
RALB	P11234-2	6.55818	6.434	5.9499	6.49875	6.28365	6.19287	6.48113	6.23277	6.22893	6.01137	5.93915	5.91955	6.22592	6.48982	6.39044	6.16356
SEC24A	O95486	6.17551	6.35753	6.67488	6.11445	6.28976	6.44895	6.18299	6.38298	6.2655	6.42198	6.54001	6.54155	5.91001	6.14021	5.79056	5.76313
ATXN10	Q9UBB4	6.39609	5.88785	6.94994	5.68269	6.3764	6.53385	6.32425	6.8389	6.27023	6.63009	6.56849	6.74448	5.75812	5.84431	5.47622	5.71809
TOP3B	O95985	6.37009	6.23417	6.60817	6.43088	6.17951	6.12999	6.30042	6.00915	6.7514	6.48755	6.52703	6.52506	5.74215	5.75787	6.07057	5.87601
RRS1	Q15050	6.10737	6.28691	5.86697	6.69482	5.87868	5.8203	6.32475	6.0202	6.00953	5.68522	5.9479	5.77508	6.58311	6.97752	7.14993	6.87172
MRPL38	Q96DV4	6.18258	6.59598	5.96266	6.83613	6.2986	6.13244	6.57422	6.3589	6.43618	6.23651	5.82376	6.00889	6.40084	5.82571	6.23881	6.08778
GPS1	Q13098-7	6.22116	6.1825	6.61885	5.70594	6.30719	6.43359	6.22361	5.84138	6.22046	6.35783	6.51822	6.27151	6.58061	6.10279	6.30217	6.1122
TTLL12	Q14166	5.76285	6.07479	6.66177	5.84609	6.23267	7.0697	5.80985	6.63265	6.05302	6.28397	6.49247	6.6549	6.1898	6.36967	6.06489	5.80092
OTUB1	Q96FW1	6.28575	5.50708	6.61438	5.18352	6.49866	6.63118	6.12487	6.76014	6.41676	6.71405	6.37094	6.45669	6.0959	6.3507	6.07372	5.91566
ITCH	Q96J02	6.32684	6.44355	6.80913	6.5297	6.48157	6.72226	6.23925	6.12901	6.4585	6.4044	6.56587	6.3212	5.61404	5.68201	5.78039	5.49229
HSD17B11	Q8NBQ5	6.30467	6.57865	6.36749	6.59217	6.20508	6.19074	6.39106	5.72387	6.09569	6.50387	6.0965	5.88976	6.23949	6.16496	6.61679	6.03922
BRAT1	Q6PJG6	5.88242	5.5389	6.52289	5.51309	5.93979	6.14486	5.93546	6.08029	6.22554	6.39125	6.75057	6.36988	6.3804	6.62182	6.86065	6.84219
CCDC22	O60826	6.04306	6.17769	6.58872	5.85015	6.43338	6.35035	5.90642	6.46593	6.2767	6.55668	6.40157	6.51704	6.07472	6.19174	6.07095	6.09491
C1QBP	Q07021	6.36734	6.70064	5.54759	6.72103	6.25771	5.89926	6.06377	6.52109	5.93724	6.05613	5.71547	6.22561	6.72956	6.50506	5.81898	6.93352
PGM3	O95394-4	6.16145	6.2039	6.88097	5.86074	6.35596	6.85509	6.29514	6.76078	6.33761	6.54393	6.39716	6.43068	5.81795	5.67123	5.81715	5.61026
ITGA5	P08648	6.00197	6.38655	6.08511	6.53248	6.05881	5.9697	6.11757	6.11734	6.37395	6.19546	6.08312	6.05777	6.64864	6.23122	6.62274	6.51755
GLB1	P16278	6.60853	6.48512	6.03999	6.55853	6.37797	6.11874	6.17028	6.10557	6.05197	6.07273	6.04757	5.8779	6.64751	6.12642	6.59788	6.11329
JAK1	P23458	6.92506	7.25657	6.8737	7.23338	6.56364	6.40881	6.76519	6.2628	6.71794	6.89845	6.84856	6.74353	4.67784	4.57853	4.48481	4.76118
DNAJC11	Q9NVH1	6.70051	6.44649	5.97562	6.53763	6.45192	6.15585	6.31369	6.04715	6.17888	5.78876	5.90144	5.76322	6.49286	6.1996	6.7333	6.31309
TAOK1	Q7L7X3	6.1057	6.26265	6.26288	5.92909	5.94552	5.94522	6.15668	5.85754	6.45245	6.60039	6.64884	6.62975	6.24532	6.36006	6.29117	6.30675
RBBP5	Q15291	6.42436	6.31851	6.07048	6.80115	6.14859	6.2211	6.33939	5.78433	6.10368	5.85346	5.78226	5.73381	6.39609	6.48878	6.7626	6.77142
RETSAT	Q6NUM9	6.44837	6.6629	6.038	6.7058	6.24532	6.15237	6.52155	6.09003	6.24897	6.30826	6.25034	6.31644	6.07046	6.0041	5.97999	5.95709
RRAS	P10301	6.56403	6.40803	6.20628	6.51813	6.16182	6.09883	6.6025	6.0405	6.48201	5.9352	5.93148	6.04835	6.13585	6.1711	6.38278	6.31311
ALDH9A1	P49189-3	6.24021	6.11513	5.83875	6.11114	6.36125	6.35317	6.53677	6.40106	6.45477	6.04962	5.92041	6.31214	5.86627	6.51345	6.33779	6.58807
CXXC1	Q9P0U4-2	6.28765	6.39042	6.14988	6.61875	6.28259	6.09669	6.29677	6.03282	6.13331	6.15088	5.98192	6.147	6.21525	6.30593	6.56717	6.34297
H2AZ1	P0C0S5	6.18897	6.31121	5.83644	6.95884	6.21869	5.83313	6.74025	5.79547	6.21499	5.65216	5.62234	5.79957	6.41693	6.71942	6.67971	7.01188
PRPSAP2	O60256	6.18654	6.20389	7.08297	5.87697	6.39795	6.76742	6.4233	6.44609	6.21894	6.32979	6.52547	6.33944	5.88923	5.8665	5.79719	5.64829
ATP5MF-PTCD1	G3V325	6.16598	6.43583	5.6911	6.78643	6.14126	6.20367	6.4714	5.89548	6.41464	6.07426	6.1232	6.30624	6.5879	6.18205	6.16038	6.36018
AAK1	Q2M2I8	6.16423	6.04187	6.5815	5.86682	6.45757	6.50728	5.88994	6.25047	6.40072	6.56231	6.77424	6.63357	6.0197	6.20432	5.74722	5.89823
ARHGAP35	Q9NRY4	5.92452	6.0346	6.4012	5.92169	5.95505	6.42594	6.08119	6.51968	6.35817	6.33883	6.25941	6.49702	6.34697	6.50639	6.27037	6.15896
HSPA14	Q0VDF9	6.38933	6.23164	6.71131	5.90479	6.48688	6.40715	6.1326	6.79878	6.56606	6.33089	6.69571	6.47053	5.85355	5.76948	5.65481	5.5965
FMR1	Q06787	6.45784	6.78745	6.30265	6.25486	6.28896	6.32768	6.43038	6.24469	6.15423	6.399	6.2056	6.2865	6.10294	5.92813	5.90485	5.92424
MPP5	Q8N3R9	6.32839	6.19091	6.43797	6.25888	6.3839	6.36519	6.48076	6.2057	6.16493	6.14864	6.48063	6.10676	6.30828	6.18532	6.02209	5.93166
ZPR1	O75312	6.67916	6.07236	6.7953	5.77241	6.52613	6.77971	6.0093	6.98551	6.35323	6.3736	6.66174	6.6701	6.0434	5.63246	5.21064	5.43498
ANXA11	P50995	6.06279	6.24616	6.67655	5.77197	6.43343	6.71379	6.00994	6.7757	6.32567	6.35831	6.32687	6.36619	6.04189	6.29976	5.8732	5.71777
FAF2	Q96CS3	6.31643	5.96633	5.94788	6.32632	5.89371	5.93955	6.22938	6.05397	6.30485	6.11292	6.12485	6.20167	6.63447	6.56501	6.74629	6.63636
PHACTR4	Q8IZ21-2	6.18412	6.30026	6.28919	6.06025	6.18994	6.02822	6.49513	6.19764	6.72044	6.30619	6.16478	6.27714	6.16258	6.33194	6.10277	6.1903
ZNF574	Q6ZN55-2	5.39461	5.58405	5.72597	6.41402	5.87791	5.85659	6.87566	5.47976	6.45586	5.04382	5.64412	5.89441	6.22647	7.76464	7.58468	8.17743
STAG1	Q8WVM7	6.24843	6.55703	6.04051	6.81857	6.04509	5.80934	6.18846	5.66615	5.99084	6.02395	6.11258	6.15071	6.59158	6.35367	6.56921	6.83388
ST13	P50502	6.68966	6.30126	6.67157	5.96571	6.88467	6.82385	6.4188	6.91872	6.8437	6.13681	6.34235	6.19723	5.59789	5.49314	5.2009	5.51372
KIF14	Q15058	6.42564	6.49725	6.21544	6.75946	6.20273	6.4826	6.77929	6.18491	6.45768	6.4286	6.23455	6.46406	5.65874	5.79695	5.74675	5.66536
SGTA	O43765	6.34958	5.25011	6.41204	4.86222	6.21544	5.96381	5.63371	6.82776	5.78241	6.72679	6.36149	7.00826	6.57763	6.85129	6.66446	6.513
PPM1G	O15355	5.4302	5.64354	5.95221	5.04141	5.52362	6.297	5.59676	6.23358	5.8325	6.06264	6.40264	6.26827	6.80439	7.87039	7.68726	7.35356
GTF3C2	Q8WUA4	6.19863	6.5584	5.98958	6.5761	6.05075	6.10889	6.11154	6.13751	5.92961	6.32205	6.05359	5.82569	6.35289	6.64152	6.70755	6.4357
GNPAT	O15228	6.23867	6.42367	6.23225	6.71193	6.44612	6.21189	6.26761	5.82239	6.26406	6.29347	6.2384	5.80268	6.3179	6.12271	6.3375	6.26874
EML3	Q32P44	6.45072	6.35674	6.85299	5.92631	6.46653	6.52482	6.13053	6.45684	5.94249	6.31894	6.38022	6.5487	6.11541	6.06924	5.70246	5.75707
RFC4	P35249	5.97399	6.06743	6.04552	5.95368	6.24878	6.17579	6.18696	6.11925	6.06868	6.29732	6.24601	6.19347	6.64637	6.54643	6.67629	6.55403
SRPRB	Q9Y5M8	6.54882	6.27985	5.79284	6.41812	6.50277	5.98089	6.53884	5.99435	6.43385	6.04621	5.74372	5.71424	6.62571	6.27694	6.61326	6.48961
NR2C2	P49116	6.32728	6.47248	6.05176	6.68445	6.13252	6.10078	6.22546	5.90591	6.11938	6.18452	6.11922	6.03675	6.46966	6.23504	6.54987	6.38491
KLC4	Q9NSK0-3	6.58017	6.08993	7.03822	6.28387	6.58926	6.56304	6.04882	6.3607	6.67666	6.47432	6.224	6.44527	6.08769	5.67241	5.43852	5.42711
ARHGEF40	Q8TER5	6.34786	6.39104	6.29438	6.10865	5.99819	6.58659	6.3003	6.40227	6.46016	6.6401	6.50521	6.23431	5.97311	6.24697	5.99339	5.51747
TDRD7	Q8NHU6	6.0575	6.26498	6.69106	6.00142	5.87247	6.42595	6.10678	6.28188	6.23147	6.42182	6.6157	6.27736	6.07031	6.13895	6.64857	5.8938
VPS50	Q96JG6	6.30202	6.1688	6.74018	6.0325	6.26557	6.36992	6.22886	6.40731	6.44326	6.45573	6.6239	6.57443	5.92017	5.81691	5.88502	5.76543
MAP2K3	P46734-3	6.19344	5.89591	7.22328	6.09341	6.21111	6.68794	6.21397	6.3992	6.54549	6.03128	6.36474	6.31411	5.95114	6.02314	6.05901	5.79282

TMOD3	Q9NYL9	6.57501	5.93917	6.39875	5.67368	6.70574	6.21283	5.78594	6.80586	6.25108	6.45245	6.55992	6.96256	6.41841	5.963	5.55433	5.74127
DHX35	Q9H5Z1	6.44515	6.34794	5.63788	6.23081	6.42003	6.30167	6.28366	5.72193	6.05171	5.94905	5.97118	6.06579	6.67282	6.3308	6.99311	6.57646
PPP1R18	Q6NYC8	6.22922	6.43318	5.99939	6.2516	6.30526	6.28432	5.78189	5.90545	6.22406	6.2681	6.03508	6.29253	6.97893	6.47378	6.27368	6.26352
NOM1	Q5C9Z4	6.24673	6.10991	5.41974	6.56336	6.04536	5.79165	6.61861	6.14161	6.6311	5.99077	6.01691	6.08539	6.29799	6.46087	6.50114	7.07885
IPO11	Q9UI26-2	5.79379	5.55082	6.29213	5.39215	5.81039	6.22046	5.95123	6.11476	6.1051	6.1694	6.43614	5.97436	6.88374	7.09295	7.15477	7.05781
CCNB1	P14635	6.50305	5.8036	6.7975	6.26284	6.12448	6.25288	6.45658	6.52411	6.79921	6.21729	6.67952	6.47234	5.96622	5.67897	5.64517	5.81624
FAM193A	A0A1B0GVL4	6.24122	6.57192	6.55063	6.32042	6.15083	6.5181	6.1526	6.59681	6.10571	6.77913	6.25344	6.60205	5.39152	6.17223	5.61475	5.97864
TSC2	P49815	6.57181	6.78808	7.2157	6.67077	6.21292	6.75034	6.13494	6.32487	6.67803	6.82616	6.62626	6.91716	5.01463	5.29078	4.83129	5.14628
MRPS27	Q92552-2	6.55113	6.44253	5.98611	6.71548	6.3208	5.99506	6.44649	6.19017	6.09249	5.71532	6.15785	6.1102	6.50435	6.03955	6.49549	6.23699
NOSIP	Q9Y314	6.29037	6.00349	7.00512	6.38114	6.0917	6.47518	5.97017	6.39046	6.53716	6.79458	6.92636	7.02803	5.3468	5.82252	5.52931	5.40759
HERC5	Q9UII4	6.30171	6.62178	6.84865	6.25542	6.35178	6.34415	6.32765	6.19077	6.55936	6.75422	6.55228	6.50991	5.40374	5.77304	5.61673	5.58879
PLK1	P53350	6.9299	7.12738	6.75244	7.30596	6.19482	6.54369	6.68198	6.34451	6.68401	6.74597	6.54784	6.82301	4.53335	5.01945	4.65502	5.11066
WDR75	Q8IWA0	6.2237	6.1438	6.08983	6.89611	6.10341	5.85212	6.37738	5.94673	6.28864	6.01087	6.01751	6.02466	6.36136	6.09779	6.81177	6.75434
ANKRD27	Q96NW4	6.16529	6.17498	6.60757	6.47505	5.98438	6.39437	6.10742	6.52659	6.41859	6.54549	6.4858	6.59095	5.87572	5.90463	6.24247	6.1007
COPS3	Q9UNS2	5.97188	5.8758	6.4523	5.68651	6.41651	6.53111	6.27035	6.13006	6.16304	6.30019	6.50192	6.231	6.33223	6.48565	6.51909	6.13234
OS9	Q13438	6.32081	6.17863	6.01281	6.47775	6.65646	6.10128	6.27604	6.38766	6.23147	6.25024	6.2245	5.93931	6.595	5.95401	6.15003	6.34442
EFL1	Q7Z2Z2	6.18315	5.85726	6.50993	5.64027	6.2856	6.82075	5.86197	6.69903	6.4682	6.34135	6.26918	6.28949	6.32298	6.41622	5.96054	6.07407
PNP	P00491	6.07919	6.293	6.64281	5.92736	6.54678	6.71729	6.15619	6.7312	6.25068	6.28597	6.6327	6.73182	5.87813	5.92973	5.6872	5.50993
CLPB	Q9H078-2	6.3829	6.44203	5.76905	6.7282	6.35715	5.96933	6.64971	6.04508	6.18797	5.9052	6.09298	5.94205	6.67132	5.97888	6.54604	6.33209
ZNF609	O15014	5.9828	6.3088	5.95316	6.20523	5.97468	5.97499	6.2018	5.86811	6.12785	6.5248	6.09191	6.29192	6.20311	6.83037	6.59076	6.86969
PTBP3	O95758-4	6.44497	6.2555	6.18278	6.4491	6.45435	6.21285	6.27131	6.32732	6.14534	6.17924	6.00059	6.10289	6.46795	5.90921	6.37341	6.22321
UBQLN2	Q9UHD9	6.03958	4.70227	5.27132	4.4159	5.51605	4.55836	5.45812	7.03945	4.84991	5.8677	5.274	7.13777	6.24713	9.74171	8.52773	9.35299
MLEC	Q14165	6.45779	6.45566	5.78088	6.43217	6.34808	6.06418	6.26532	6.04364	6.12797	6.1169	5.80588	5.754	6.68702	6.22748	6.86167	6.57134
USP28	Q96RU2-2	5.96133	6.40973	5.88887	6.41581	6.09651	6.20114	6.03487	6.24441	6.06835	6.26004	5.90556	6.15458	6.64846	6.55335	6.65858	6.4984
GLRX3	O76003	6.10881	6.03453	6.84399	5.67722	6.53735	7.05172	6.13216	6.77203	6.28833	6.55281	6.71268	6.66427	5.73937	5.78309	5.39155	5.71011
PSMD5	Q16401	6.33455	6.08624	6.72534	5.822	6.27844	6.80997	6.00977	6.56426	6.38735	6.52825	6.32278	6.24893	5.8877	6.21269	5.87592	5.90582
DBR1	Q9UK59	5.52177	5.88342	6.31176	5.41876	5.77216	6.01913	5.62579	5.94999	6.039	6.42851	6.19471	6.15667	7.0663	7.41147	7.25936	6.94119
CAVIN1	Q6NZI2	6.31181	6.32401	5.76093	6.64492	6.48046	6.08632	6.76237	6.15285	6.41713	5.72463	5.6971	5.84403	6.32129	6.4104	6.427	6.63475
MRPL4	Q9BYD3	6.3381	6.46001	5.8663	6.91835	6.46667	6.24503	6.55916	6.18377	6.28761	6.20422	5.92933	6.0825	6.21505	6.04488	6.22642	5.97261
FCHO2	Q0JRZ9	6.34725	6.13036	6.70684	5.99053	6.36747	6.51008	6.1762	6.26194	6.65614	6.52531	6.50969	6.67004	6.00534	5.87973	5.56227	5.7008
USP13	Q92995	5.86072	6.04059	6.51225	5.89972	5.83627	6.56806	6.23621	6.50867	6.35791	6.58893	6.6609	6.69084	6.10471	5.95485	6.05463	6.12473
CLINT1	Q14677-3	6.17804	6.13403	6.6551	6.4789	6.33387	6.54707	6.45367	6.18843	6.30167	6.08495	6.2432	6.13737	6.09147	6.17571	5.97787	6.01863
SHTN1	A0MZ66-3	6.48242	6.05494	6.81101	5.95459	6.27949	6.41183	6.25134	6.55802	6.5651	6.29893	6.70114	6.5306	6.11848	5.62106	5.71365	5.64742
MYL12B	O14950	5.87758	6.38171	6.5682	5.8285	6.50184	7.00817	6.27222	7.03617	6.51389	6.40037	6.50582	6.75065	5.68127	6.04625	5.10093	5.52644
TWF2	Q6IBS0	6.04514	6.13491	6.43912	5.9421	6.09588	6.64988	6.7126	6.18449	6.32248	5.84152	6.14417	6.37814	5.83186	6.64463	6.01108	6.62198
ZC3HC1	Q86WB0	6.03591	6.20064	5.90051	6.41779	6.08496	6.1583	6.35546	5.99175	5.934	6.04193	6.11364	5.96753	6.85451	6.38627	6.82859	6.72822
IKBIP	Q70UQ0-4	6.43619	6.54802	5.57062	6.70316	6.21114	6.00138	6.4983	6.20252	5.99091	6.17695	5.89161	5.92988	6.34039	6.31588	6.67933	6.50374
GOPC	Q9HD26	6.35326	6.49777	6.40299	6.83515	6.69435	6.7075	6.22524	6.42368	6.45528	6.62116	6.38046	6.39775	5.45052	5.77206	5.32293	5.4599
SLC25A11	Q02978	6.25728	6.28263	5.7985	6.58754	6.19395	5.82441	6.62156	5.86226	6.5289	5.96369	5.99598	5.74445	6.64814	6.29839	6.83843	6.5539
SHCBP1	Q8NEM2	6.1649	6.73311	5.8951	6.41914	6.4207	6.42749	6.06438	6.45245	6.09123	6.2759	6.14399	6.41974	6.14243	5.96243	6.05644	6.33059
DNAJC9	Q8WXX5	5.94905	5.78027	6.30101	5.84621	5.7732	6.27404	5.99963	6.4859	6.39822	6.32502	6.40504	6.40321	6.11787	6.86223	6.71255	6.36653
SQSTM1	Q13501	5.62182	5.26946	5.79804	6.016	6.44696	6.35605	6.47089	6.28426	6.34251	5.76096	6.01523	6.08771	7.52996	6.52102	6.66168	6.81743
EIF3F	O00303	6.28436	5.46279	6.11002	5.09415	6.42896	6.05107	6.01909	6.75651	5.80497	6.58017	6.17806	6.6618	5.69083	7.19304	6.8209	6.86329
ITGB4	P16144	6.23971	6.57834	6.04674	6.13203	6.02755	5.90182	6.09671	5.95104	6.36344	6.34294	6.05369	6.18543	6.58324	6.6453	6.51215	6.33987
HNRNPA0	Q13151	6.4446	6.53887	5.97403	7.01824	6.25289	6.2108	6.23474	6.06473	6.16336	6.16503	6.04793	6.18746	6.3887	5.92907	6.20699	6.17255
INTS14	Q96SY0	6.21165	6.26968	5.91671	6.41113	6.24994	6.10687	6.07196	6.0428	6.12326	6.13875	6.10938	5.93044	6.7563	6.56143	6.56351	6.53618
KEAP1	Q14145	6.75331	6.48257	6.73028	6.57068	6.50296	6.28671	6.47991	6.43977	6.36307	6.56249	6.62227	6.48677	5.36605	5.42837	5.33485	5.58996
PPP1R7	Q15435	5.87171	6.01648	6.3299	5.66366	6.45321	6.67632	6.1018	7.04498	6.8726	6.83218	6.53499	6.27913	5.86272	5.96927	5.87138	5.61967
PXN	P49023-3	6.427	6.03709	6.92578	5.94504	6.69018	6.74408	6.24118	6.74865	6.51945	6.17291	6.89377	6.58945	6.11556	5.58909	5.11602	5.24477
LRCH4	O75427	6.21795	6.39463	6.08624	6.37375	6.31885	6.35542	6.38882	5.92591	6.13146	6.34565	6.33167	6.30152	6.27557	5.92236	6.35695	6.27327
SDAD1	Q9NVU7	6.15213	6.35291	6.05554	6.89699	6.28004	5.96251	6.51366	6.00405	6.35026	6.07374	5.93005	6.0516	6.19437	6.29303	6.48081	6.40832
ANP32B	Q92688	5.57043	5.80537	6.15929	5.15594	5.80184	6.33378	5.73937	6.27005	6.07881	6.27998	6.47338	6.0971	6.67639	7.30744	7.43925	6.81158
HAUS6	Q7Z4H7	6.4509	6.35	6.86668	6.34681	6.14682	6.81369	6.17267	6.48736	6.42865	6.3383	6.73842	6.54076	5.94801	5.54895	5.30621	5.51579
NKRF	O15226-2	6.4298	6.52708	6.03296	6.85097	6.04703	5.78705	6.48249	5.80013	6.16004	6.03348	6.19376	6.09667	6.4734	5.99418	6.59969	6.49127
METTL16	Q86W50	5.41075	5.74469	5.77047	5.34989	5.49734	6.02208	5.93189	6.07134	5.91486	6.08723	6.36974	6.00904	7.19568	7.33437	7.75878	7.53186
RAPGEF6	Q8TEU7-4	6.20739	5.80264	6.86967	6.24969	6.18489	6.56548	6.2955	6.48559	6.43136	6.6309	6.6198	6.47794	5.8395	5.83714	5.77529	5.72721
PSMD8	P48556	5.93663	5.65058	6.23513	5.5204	6.45695	6.18593	6.06113	6.80286	5.75899	6.56869	6.25401	6.65785	6.36329	6.48456	6.64497	6.41802
ATP13A3	Q9H7F0	6.63813	6.99802	6.58599	7.00314	6.28354	6.16368	6.23396	6.09508	6.30871	6.6111	6.33618	6.42074	5.38941	5.40343	5.53215	5.99672
MRPL19	P49406	6.45812	6.51667	6.00416	6.57002	6.61365	5.8152	6.57529	6.06803	6.09129	5.99146	5.76803	5.77132	6.64298	6.23218	6.54414	6.33746
SENP6	Q9GZR1	5.78945	6.19206	6.3728	6.26139	5.9142	6.22592	6.07988	6.00577	6.30318	6.38569	6.23337	6.32981	6.2429	6.37046	6.60357	6.68955
ZNHIT6	Q9NWK9	6.4727	6.5025	6.65436	6.76741	6.43739	6.47341	6.55137	6.53801	6.5022	6.08713	6.52812	6.36112	5.72326	5.2491	5.66879	5.48312
NKTR	P30414	6.05711	6.18383	6.11693	6.68729	5.73495	5.8056	6.23954	5.77798	6.02198	5.88911	6.04447	5.77893	6.63347	7.07456	6.77343	7.18084
EIF2B1	Q14232	6.16943	6.00583	6.75595	6.018	6.46939	6.86159	6.18669	6.47787	6.44715	6.72605	6.32615	6.49883	5.77925	5.98306	5.56539	5.72935
TPT1	P13693	6.30346	5.08266	6.8311	5.32065	6.36837	6.23951	5.99086	6.79887	6.34473	6.85896	6.96466	7.40599	5.98908	5.98232	5.70287	5.81592
UNC13B	O14795-2	6.31024	5.81625	6.58123	5.85947	6.35086	6.5078	6.3302	6.21434	6.46295	6.68709	6.7675	6.5335	5.91909	6.27129	5.80089	5.5873
CAMSAP2	Q08AD1	6.26488	6.2244	6.80656	6.06858	6.11808	6.64679	6.25151	6.36538	6.31455	6.32731	6.52216	6.60278	5.89487	5.87678	5.81806	5.89732
MIA2	Q96PC5	6.41087	6.28143	6.15784	6.48411	6.26754	5.92759	6.31504	5.84199	6.11234	6.07479	6.15192	6.04823	6.7645	6.14742	6.58727	6.42712
MRPS31	Q92665	6.52626	6.6063	6.08469	6.78607	6.11879	5.86084	6.51886	5.98362	6.05879	6.17107	6.04948	5.95458	6.49543	6.04805	6.46642	6.27076
RPS15	P62841	6.42497	6.26423	6.3231	6.36304	6.01768	5.87884	6.71241	6.09402	6.79522	6.18244	5.56147	6.2589	5.63246	6.36837	5.99947	7.12337
WDR74	Q6RFH5	6.29902	6.39971	6.41332	6.81497	6.26998	6.01519	6.10079	6.35007	6.41459	5.95394	6.30108	6.1639	6.3221	5.82932	6.11185	6.24016
MRPS5	P82675	6.37241	6.07237	5.52618	6.62928	6.28785	6.03224	6.46906	6.13988	6.09386	6.15219	5.83657	5.8846	6.47981	6.7731	6.60204	6.64856
ATP1B3	P54709	6.49144	6.30852	6.2344	6.40191	6.21787	5.95543	6.14573	6.28051	6.64831	6.59533	6.4444	6.70948	6.01626	5.65065	5.85125	6.04851
NFKB1	P19838-2	6.26043	5.8547	6.7639	5.94369	6.27989	6.78342	6.2784	6.62721	6.41253	6.33762	6.60239	6.58319	6.20161	5.69027	5.72872	5.65203
FKBP9	O95302-3	6.47494	6.20257	6.09384	6.62467	6.62114	6.07599	6.41935	6.01996	6.51428	5.74121	6.30084	6.04416	6.54354	5.89675	6.04384	6.38293
DTYMK	P23919	6.2556	6.02465	6.84398	5.75856	6.23357	6.74746	6.04528	6.51387	6.33976	6.80335	6.76277	6.18755	6.08918	5.67468	5.88921	5.83054
RPL13	P26373	6.18444	5.92071	5.97031	5.94101	5.92927	6.0888	6.39877	6.41061	6.91255	6.39521	6.48042	6.37113	6.3943	6.44809	5.65279	6.50159
KPNA1	P52294	6.04447	5.76719	6.39266	6.23764	6.39637	6.4185	6.19959	6.21263	6.09592	6.14041	6.17637	6.02776	6.63323	6.41095	6.46431	6.38198
CTNNBL1	Q8WYA6	6.08912	6.27342	5.71843	6.11829	6.18337	6.01679	6.33338	5.94318	6.12145	6.09879	5.71418	6.04521	7.11785	6.53003	6.81654	6.87999
LRPAP1	P30533	6.67523	6.48111	6.26001	6.37963	6.49438	6.2682	6.31117	6.2741	6.46593	6.08897	6.12236	5.95529	6.53369	5.6934	6.12951	5.86703
MAP2K2	P36507	6.2163	5.95887	6.79731	5.9702	6.54474	6.96282	6.25849	7.00855	6.26455	6.46433	6.63844	6.55422	5.96292	5.98074	5.21578	5.20174
DHODH	Q02127	6.52309	6.33888	5.70651	6.63181	6.48435	6.35996	6.37026	6.36286	5.96769	5.85519	6.01653	5.97875	6.69809	6.01224	6.37244	6.32133
FABP5	Q01469	6.0864	5.77856	6.72247	5.84839	6.99404	6.77474	6.71591	6.27004	7.06399	5.75721	6.37355	6.60132	5.4494	6.11428	5.38676	6.06295
CCDC47	Q96A33	6.50119	6.59257	6.24719	6.60422	6.05302	6.06388	6.4476	5.93427	5.8767	6.17169	5.95771	5.7194	6.53192	6.13746	6.56601	6.59518
WDR61	Q9GZS3	6.00769	6.23779	6.10281	5.98342	6.48491	6.42913	5.70813	6.45789	6.33281	6.37152	6.46588	6.44364	6.38248	6.06094	6.04455	6.48639
PDXDC1	Q6P996	6.22425	6.23879	6.08639	6.55766	6.08333	6.28704	6.14226	6.46269	6.40888	6.68486	6.57558	6.66137	6.17481	6.04598	5.78413	5.58198
SUCLG1	P53597	6.30953	6.32544	5.9464	6.66477	6.39928	6.14399	6.27259	6.17339	6.15066	6.25967	6.27637	6.11203	6.51715	6.053	6.21995	6.17579
COPS5	Q92905	6.12442	5.92761	6.59425	5.78229	6.29311	6.48036	6.28889	6.32014	5.96176	6.5132	6.39897	6.12557	6.37147	6.32026	6.34563	6.15206
CAPNS1	P04632	6.08965	6.41315	6.01919	4.81125	6.00075	5.77232	6.66275	6.64623	6.03346	6.16395	5.78618	7.10599	5.33781	7.94547	6.82882	8.18304
STAG3	Q9UJ98	6.38944	6.61414	6.23333	6.30603	6.36837	6.27437	6.30766	6.33229	6.2546	6.43234	6.44098	6.42806	5.7847	5.85062	5.72706	6.25601
CDC27	P30260-2	6.54817	6.58167	6.54768	6.67401	6.38198	6.36025	6.54577	6.11033	6.56352	6.37273	6.31491	6.29363	5.70806	5.64569	5.68038	5.67123
STRIP1	Q5VSL9	5.95496	6.18634	6.4562	5.94806	5.98201	6.43558	6.14766	6.06906	6.32502	6.46163	6.46179	6.42251	6.40781	6.35075	6.25937	6.13125
CHCHD3	Q9NX63	6.68047	6.16699	5.91805	6.52682	6.55505	6.08834	6.25668	6.20003	6.25252	6.04773	6.15144	5.94925	7.10014	5.85808	6.15925	6.08916
TAF6	P49848-3	6.11597	6.54501	6.22055	6.60253	6.32387	6.36344	6.1686	6.36647	6.34723	6.59825	6.43031	6.3385	6.25236	5.64495	5.71458	5.96738
TEP1	Q99973	6.14821	6.4924	6.74197	6.07572	6.22224	6.67327	6.09816	6.57442	6.33476	6.53482	6.65405	6.36787	5.64034	6.23262	5.73529	5.47386
NUF2	Q9BZD4	6.47144	6.63901	6.97441	6.64508	6.25539	6.79467	6.44142	6.66787	6.80648	6.54644	6.52297	6.38799	5.08465	5.20516	5.11349	5.44352
PCID2	Q5JVF3	6.60673	6.48337	6.35459	6.54617	6.4249	6.21411	6.35683	6.15954	6.40731	6.17813	6.28891	5.95826	6.12669	5.70828	6.20193	5.98425
ALDH1B1	P30837	6.45903	6.34195	5.69883	6.42495	6.70282	6.14075	6.42586	6.43724	6.01741	5.82226	5.86997	6.09397	6.96692	5.98286	6.30174	6.31341
NMD3	Q96D46	6.15169	6.19056	6.33649	6.00676	5.92725	5.97386	5.98627	6.17396	6.28835	5.81656	6.17368	5.90493	6.96709	6.60543	6.8624	6.63473
CDK2	P24941	6.16935	5.56552	6.48794	5.65251	6.09377	6.3093	6.25385	6.4505	6.3864	6.55085	6.83363	6.99072	5.99984	5.83822	6.17751	6.24009
CDK12	Q9NYV4	5.96707	6.02234	5.93895	6.72037	5.87547	5.83877	6.13589	5.68942	5.89388	5.79382	5.89298	6.15107	6.33412	7.24097	7.22311	7.28178
MYO9A	B2RTY4-4	6.26645	6.16839	6.58807	6.38309	6.0121	6.41548	6.18317	6.33193	6.36169	6.46699	6.28812	6.53489	5.94937	6.20959	5.87587	5.96482
CCDC91	Q7Z6B0	6.46703	5.69176	6.84487	5.1248	6.49279	6.84084	5.54728	7.15467	6.09035	6.97878	6.97079	7.31859	6.3347	5.95833	5.63218	5.55224
PARD3	Q8TEW0	6.59036	6.13626	6.3384	6.12005	6.18798	6.13328	6.26118	6.2058	6.20289	6.08224	6.18767	6.22556	6.37997	6.33663	6.40761	6.20413
POLG	P54098	6.5676	6.54355	6.10326	6.79683	6.15118	5.70475	6.26411	5.94799	6.33683	6.51435	6.30986	6.37534	6.32088	5.8628	6.18247	6.01819
TUBB4B	P68371	6.25093	6.08631	6.78045	5.86504	6.69884	7.07622	6.26959	6.92184	6.08927	6.12878	6.6415	6.83441	5.69737	5.8149	5.16844	5.67611

TRMT2A	Q8IZ69	5.51567	6.12558	5.9024	5.39776	6.06601	6.19195	5.92544	6.08562	6.12364	6.26702	6.05396	5.91931	7.16124	7.17294	7.17585	6.91562
ANK2	Q01484	6.04962	6.30292	6.68806	5.74272	6.35204	6.63171	5.88967	6.23698	6.1661	6.16942	6.39493	6.49955	6.19963	6.25654	6.2739	6.1462
HEATR5A	Q86XA9	6.22798	6.21234	6.64007	6.25237	5.99661	6.72155	6.15772	6.17318	6.31886	6.62486	6.62123	6.72525	6.07487	5.96604	5.58382	5.70324
DNAJC2	Q99543	5.88819	5.92592	6.2782	5.86899	5.85106	6.35492	5.83538	6.3656	6.1056	6.50353	6.4988	6.43144	6.4483	6.52844	6.49695	6.61867
OSBPL6	Q9BZF3-5	5.59242	5.95233	6.0351	6.23741	6.08706	6.0761	6.0067	6.03803	6.40045	6.38022	6.78107	6.66906	6.22929	6.24166	6.55732	6.71577
TJP2	Q9UDY2-7	6.33937	6.16058	5.84139	6.16103	6.17603	5.99642	6.18637	6.14676	6.29682	6.17607	6.05815	6.02375	7.02051	6.61094	6.56516	6.24064
MAP1S	Q66K74	6.21208	6.28363	6.48291	5.98809	6.19422	6.54601	6.32832	6.63999	6.4691	6.62803	6.69028	6.43079	5.61694	6.06491	5.74171	5.68297
MMUT	P22033	6.28409	6.43208	5.66803	6.48212	6.1486	5.99025	6.46917	6.07436	6.00347	6.0189	5.99736	5.77998	6.69809	6.53188	6.67944	6.74218
PTPRK	Q15262-2	6.56603	6.39583	6.27743	6.42663	6.33435	6.09599	6.25461	5.96679	6.32562	5.95961	6.15199	6.03418	6.1443	6.32835	6.21967	6.5186
FTO	Q9C0B1	5.81397	5.68969	6.58608	5.39476	6.17054	6.33283	5.81852	6.285	5.913	6.1803	6.48788	6.48872	6.55681	6.81484	7.01786	6.44921
GNB1	P62873	6.53142	6.48478	5.8258	6.18505	6.63665	6.35645	6.60046	6.25726	6.41675	6.26953	6.07123	5.83904	6.21667	5.85137	6.38366	6.07387
HEATR6	Q6AI08	6.41013	6.59471	6.62893	6.00562	6.31198	6.58153	6.03903	6.6787	6.37431	6.33897	6.18762	6.4982	5.95862	6.00511	5.56421	5.82233
PPIL4	Q8WUA2	5.9557	6.22372	6.15117	6.06315	6.27213	6.36661	6.17674	5.8581	6.02233	5.9528	5.77572	5.82003	6.35853	6.86478	7.148	6.99049
MMS22L	Q6ZRQ5	5.95121	6.12053	6.10948	6.08918	6.0292	6.14138	6.23844	6.00438	6.20562	6.1369	5.99726	6.00252	6.86091	6.65969	6.73246	6.72084
ELOB	Q15370	5.81846	4.90944	6.37656	4.23989	5.5	5.6566	5.53398	6.22936	5.69354	6.05765	5.61731	6.62811	5.99355	8.98166	7.85181	8.91209
OCIAD1	Q9NX40	6.48604	6.24817	6.09159	6.59581	6.61894	6.29006	6.38711	6.0565	6.04481	6.36022	6.4464	6.12026	6.65573	5.88723	5.85262	5.85852
SMS	P52788	6.33816	5.91294	7.17626	5.93727	6.64317	6.63455	6.10011	6.65884	6.87621	6.46044	6.63817	6.86953	5.51965	5.39752	5.33522	5.50198
INTS13	Q9NVM9	5.99077	6.44184	5.95462	6.75624	6.24111	6.30421	6.14665	6.35091	6.07432	6.45622	6.00447	6.07823	6.3194	6.35608	6.11089	6.41403
PACSIN2	Q9UNF0	6.18883	6.27557	6.64863	6.52485	6.22749	6.31146	5.78585	6.52022	6.41146	6.69531	6.70211	6.78754	6.16684	5.97419	6.01875	5.6609
SEPTIN6	Q14141	5.98526	5.86537	6.48462	5.73549	6.35845	6.33336	6.3125	6.28127	6.29549	6.15291	6.85804	6.06911	6.71589	6.1998	6.36749	5.98496
CLSPN	Q9HAW4	6.2393	6.11909	6.46001	6.20264	6.0443	5.97817	6.21736	5.77884	6.71836	6.30982	6.5737	6.18557	6.64234	6.13608	6.05343	6.34098
ASH1L	Q9NR48-2	6.65387	6.91938	6.71143	7.4205	5.941	5.82898	6.2813	6.18867	7.30379	6.97968	6.77205	6.91062	4.87134	5.1017	5.08754	5.02814
GTF2F2	P13984	5.51153	5.66885	6.32643	5.57888	5.73715	6.15683	5.87882	5.96821	6.19894	6.05728	6.59679	6.17251	6.81808	7.093	7.32666	6.91004
APC	P25054	6.19963	6.42936	6.54083	6.69468	6.07294	6.18887	6.1122	6.28741	6.28212	6.44154	6.27328	6.28048	5.82876	6.08424	6.05859	6.22506
RCN2	Q14257-2	6.59433	6.04603	6.56888	5.86654	6.84842	6.37748	5.63932	6.0216	6.32389	6.5748	6.3512	5.63727	6.83008	6.25677	5.88735	6.17604
ZNF622	Q969S3	5.79265	5.81359	5.78762	5.67942	5.96279	5.94864	5.68499	6.00826	6.09439	6.32357	6.07053	6.12767	7.18584	7.15391	7.27089	7.09525
COG4	Q9H9E3	6.53061	6.23885	6.59807	6.4219	6.25081	6.45881	6.24182	6.49622	6.66972	6.65163	6.75432	6.48082	5.50057	5.90215	5.49444	5.30926
PHF6	Q8IWS0	5.7922	6.01199	5.91195	5.9536	6.12077	6.49928	5.9557	5.86482	6.18656	6.26084	6.40056	5.91829	6.71704	6.61572	7.22014	6.57052
ATAD3A	Q9NVI7-2	6.20311	6.31516	6.11676	6.72319	5.90752	5.87254	6.21357	5.84664	6.26294	6.17104	6.29447	6.23497	6.56644	6.16967	6.51576	6.58621
IFT122	Q9HBG6-6	6.45296	6.62914	6.96792	6.33031	6.36706	6.7708	6.27751	6.36214	6.47408	6.83816	6.59089	6.63019	5.21606	5.23034	5.37033	5.49213
STK10	O94804	6.00345	6.11259	6.92196	6.07281	6.55467	6.7757	6.36118	6.17836	6.03133	6.28256	6.71236	6.39411	6.21172	5.8859	5.9019	5.59941
ASL	P04424	6.28836	6.21646	6.6381	5.73667	6.55195	6.9144	5.98917	6.71616	6.34652	6.65293	6.30422	6.20454	5.89756	5.96714	5.69012	5.8857
RIPK1	Q13546	6.52861	6.13481	7.06487	6.1816	6.2975	6.51571	6.28733	6.51681	6.93436	6.58771	6.7404	6.44462	5.65743	5.32066	5.52045	5.26712
RMDN1	Q96DB5	6.47463	5.90573	5.81562	6.51059	6.38983	5.85802	6.45882	6.00577	6.08386	5.89005	6.00333	5.94746	6.70519	6.46303	6.7044	6.78367
AASDHPPT	Q9NRN7	6.26653	6.21945	6.70543	5.82033	6.23407	6.49937	6.00071	6.81157	6.4087	6.54593	6.68105	6.63158	5.79386	5.9782	5.71371	5.6895
ORC1	Q13415	7.38141	7.2988	6.87223	7.81971	5.96398	5.94071	6.39425	6.10055	6.81225	6.67563	6.81113	6.60804	4.71371	4.77601	4.83173	4.99986
CARMIL1	Q5VZK9	6.23499	6.17303	6.49624	5.83274	6.16243	6.29208	6.38921	6.21725	6.41759	6.63302	6.57526	6.31872	6.05578	6.44213	5.97766	5.78187
ARHGAP5	Q13017	5.94079	6.33743	6.80648	6.11429	5.77943	6.225	6.26525	6.08925	6.37662	6.72087	6.64124	6.96676	5.58278	6.08633	6.0395	6.02798
SRSF7	Q16629	6.49808	6.05193	6.08883	6.37257	6.35324	5.85186	6.5788	5.58725	6.47598	6.09534	6.2249	5.74964	7.0768	6.06867	6.28431	6.6418
DIS3L2	Q8IYB7	6.29436	6.45049	6.95172	6.12528	6.33588	6.50594	6.16241	6.68578	6.23108	6.49198	6.522	6.66199	5.73282	5.76906	5.51176	5.56746
NUMB	P49757	5.89826	6.37527	5.9831	6.00348	6.53713	6.30929	6.268	6.41504	5.93379	6.27395	6.23042	6.07446	6.41829	6.35879	6.47134	6.44939
OCRL	Q01968	6.16713	6.18118	6.78769	5.85142	6.25306	6.36974	6.07101	6.25219	6.47098	6.45191	6.68273	6.42391	5.99762	5.954	6.04986	6.03556
POLR3E	Q9NVU0	6.29996	6.32626	6.14487	6.11111	6.17893	6.36378	6.18581	6.0827	6.25466	6.29282	6.27421	6.0311	6.56887	6.18428	6.48094	6.2197
GBE1	Q04446	6.43542	6.37922	6.71345	6.05975	6.54628	6.97128	5.97082	7.06795	6.12966	6.3406	6.32975	6.3256	5.32377	6.32694	5.56213	5.5174
SMG9	Q9H0W8	6.72035	6.60902	7.05897	6.16699	6.28035	6.85561	6.29615	7.0159	6.82223	6.68001	6.76167	7.13994	4.89665	5.18561	4.57146	4.93909
THBS1	P07996	4.92678	4.59662	4.74479	5.16973	4.85647	4.9527	5.50638	4.92043	4.82763	4.26645	4.52326	4.54167	11.2203	9.54003	11.5155	9.89116
PSMD14	O00487	5.75341	5.13684	6.03091	5.22669	6.50718	6.13204	6.31059	6.52298	6.17095	6.27621	6.22633	6.7294	6.28215	6.63568	7.20265	6.85599
AGO3	Q9H9G7	6.20622	6.2593	6.5996	6.18518	6.51701	6.65653	6.0801	6.07998	6.35036	6.32124	6.39707	6.20143	6.12858	6.08127	5.92472	6.01141
TNIK	Q9UKE5	6.09034	6.15422	5.89191	6.17843	6.44461	6.6907	6.08339	6.154	6.12333	6.74921	6.09268	5.97279	6.47933	6.49902	6.09497	6.30106
SNX8	Q9Y5X2	6.4941	6.74946	6.59236	6.29382	6.46372	6.7812	6.20998	6.44798	6.18837	6.59385	6.70277	6.63185	5.52652	5.58879	5.62199	5.11323
FGD6	Q6ZV73	6.19301	6.39237	6.53338	6.47655	6.20075	6.48896	6.22167	6.55593	6.1816	6.17448	6.28127	6.53838	6.04131	6.07951	5.78283	5.858
KAT7	O95251	5.83867	6.4135	6.04469	6.77755	6.29342	6.48293	6.50202	5.88674	6.16676	6.32015	6.69498	6.15798	6.39795	5.84899	6.34142	5.83224
DPF2	Q92785	6.07195	6.13396	5.80132	6.33614	6.19564	5.94849	6.43673	5.81431	6.20539	6.38343	6.37772	6.11383	6.54175	6.61693	6.40154	6.62085
HOOK3	Q86VS8	6.3534	6.1379	6.71304	6.16084	6.64811	6.64869	5.92194	6.60783	6.40626	6.30061	6.4375	6.68764	6.16375	5.90718	5.3531	5.5522
SCRN1	Q12765-2	6.66408	6.12141	7.24104	6.01149	6.65547	6.71345	6.10811	6.8396	6.47952	6.52445	6.82891	6.97063	5.70308	5.33502	4.74578	5.05795
CCNB2	O95067	6.84584	6.68192	6.90677	6.55926	6.52061	6.41928	6.37799	6.1176	6.57322	6.31565	6.49856	6.159	5.50995	5.43447	5.41177	5.66812
DOP1B	Q9Y3R5	6.24295	5.91017	6.83633	6.1515	6.22552	6.71229	6.06018	6.51099	6.51207	6.6007	6.63847	6.77351	5.72124	5.92174	5.33233	5.85002
YTHDF3	Q7Z739	6.27792	6.14809	6.45535	6.17581	6.0974	6.37583	5.95296	6.51547	6.24029	6.34151	6.45309	6.60021	6.18089	5.99981	6.13925	6.04611
KIF18A	Q8NI77	6.27149	6.52544	6.01508	6.58398	6.07474	5.99205	6.24232	6.00968	6.20969	6.26648	6.13273	6.25765	6.21434	6.3082	6.43245	6.46369
COPS4	Q9BT78	5.771	4.93874	5.74463	4.83784	6.1023	5.85907	6.28159	6.35927	5.54117	6.23095	5.77913	6.59797	6.48	7.90797	7.69751	7.87086
CPSF7	Q8N684-3	6.28556	6.61703	5.63502	6.62658	6.28965	6.119	5.86392	6.28965	6.19432	6.3463	5.79361	5.97407	6.45829	6.50547	6.62458	6.37695
FHL1	Q13642-4	6.13315	6.14485	6.68612	5.50795	6.26833	6.55248	6.13818	6.26152	6.29191	6.71613	6.40014	6.13977	5.948	6.35706	6.21069	6.24371
TGFB1	P01137	6.46797	7.2633	5.87828	6.94431	6.35419	6.39685	5.9848	6.62251	5.86059	6.40428	6.18152	6.33889	5.86025	5.78269	5.83834	5.82124
USP48	Q86UV5-8	5.82915	5.73671	6.40729	5.89156	5.88978	6.02285	6.01446	5.60065	6.19595	6.41803	6.60303	6.03259	6.78807	6.6203	7.45814	6.49143
KIF13B	Q9NQT8	6.44853	6.30399	6.65493	6.1459	6.22432	6.34887	6.35912	6.64771	6.22143	6.6682	6.23424	6.57902	5.71516	5.99404	5.65428	5.80026
STXBP4	Q6ZWJ1	6.36154	6.16757	6.85956	6.10425	6.42894	6.52675	6.31843	6.87681	6.57877	6.53764	6.78865	6.72539	5.60997	5.43977	5.34097	5.33498
TTC13	Q8NBP0	6.22317	6.33819	5.94922	6.4426	5.91195	6.17695	6.24696	5.88262	6.20066	6.19685	6.03827	6.12074	6.75333	6.46669	6.40858	6.64322
GAPVD1	Q14C86-6	6.48781	6.43273	6.85835	6.11533	6.28469	6.73928	6.39579	6.59364	6.60399	6.39556	6.5948	6.71951	5.5782	5.50317	5.44588	5.25126
ANAPC5	Q9UJX4	6.37032	6.36844	6.54434	6.63276	6.32896	6.45898	6.11743	6.39998	6.27767	6.42805	6.50465	6.63527	5.80542	5.67726	5.79979	5.65068
ME3	Q16798	6.49401	6.33729	5.80287	6.64447	6.08388	5.8359	6.20649	6.17376	6.15555	6.01303	6.13504	6.06773	6.84794	6.23664	6.51696	6.44845
GNAI2	P04899-4	6.64906	6.78469	6.18611	6.60607	6.47801	6.34563	6.25416	6.17736	6.36157	6.14749	5.97753	5.76791	5.99578	5.98996	6.42027	5.85837
ARID4B	Q4LE39	6.10891	6.42421	5.94085	6.81532	6.19533	5.98666	6.28569	6.20209	5.94061	6.03838	6.15758	5.89541	6.59895	6.37268	6.68347	6.35388
CISD1	Q9NZ45	6.20061	6.36013	5.81505	6.52617	6.37637	5.81918	6.81092	6.04819	6.25987	5.62785	5.74655	5.94613	6.43025	6.68209	6.63595	6.71468
MCCC1	Q96RQ3	6.53014	6.26853	5.8203	6.45155	6.41021	5.88419	6.30486	5.86626	6.12196	6.0334	6.05977	5.80251	6.85704	6.29102	7.03461	6.26364
BTF3	P20290	6.34643	6.96557	7.03722	6.2263	6.55474	6.94892	6.45829	6.30318	6.41014	6.68847	7.07526	6.96936	4.77422	5.30822	4.88829	5.04539
MED16	Q9Y2X0-3	6.3936	6.44125	6.11403	6.72575	6.41478	6.05444	6.30163	5.86629	6.30921	6.341	6.01377	6.06546	6.32064	6.08675	6.29729	6.25412
SPIN1	Q9Y657	5.78729	6.45711	5.62496	6.14617	6.09134	6.01472	6.20079	6.20156	6.14396	6.34146	5.95659	5.9885	6.64711	6.48033	7.1182	6.7879
EML2	O95834-3	6.01955	5.99942	6.53748	5.54914	6.51247	6.80199	5.97879	6.28912	6.65637	6.61606	6.45896	6.11263	6.1256	6.46089	5.8089	6.07262
PATL1	Q86TB9	6.28794	6.28511	6.88035	5.93702	6.18155	6.72326	6.23638	6.58774	6.58293	6.50981	6.50184	6.45984	5.86551	5.56304	5.70291	5.69479
SMARCAD1	Q9H4L7	5.85881	6.48901	6.03814	6.247	6.11344	6.13354	6.17718	5.98621	5.93294	6.57554	6.1652	6.32147	6.60819	6.32086	6.58536	6.44712
COPS6	Q7L5N1	5.49593	4.84224	5.77245	5.01081	5.99905	6.0964	6.30473	6.35709	6.11763	6.44284	6.09999	6.64105	6.67688	7.15061	7.35255	7.63976
RFX1	P22670	6.10625	6.33259	6.15379	6.55994	6.13941	5.98833	5.89183	5.88968	6.20876	6.31025	6.05185	5.98538	6.42855	6.75742	6.64837	6.54759
ZNF280C	Q8ND82	6.32906	6.2683	5.92895	6.72372	6.15255	6.07921	6.51705	5.9706	6.36497	5.83801	6.13962	5.92652	6.59274	6.36146	6.4285	6.37876
SRBD1	Q8N5C6	6.38347	6.11722	5.68912	6.73904	6.04177	5.90108	6.31908	5.98793	6.23348	6.21453	5.95902	6.09957	6.53917	6.47719	6.55264	6.74568
MAN2B1	O00754	6.64845	6.566	6.33768	6.6507	6.23108	6.15147	5.97615	6.09674	6.09364	6.44602	6.22741	6.62291	5.9872	6.18638	5.91415	5.86403
NCOA6	Q14686	6.58718	6.71543	5.98636	6.62147	6.33744	6.08686	6.31667	6.17942	6.4105	6.33948	6.08536	6.11953	6.27533	6.15127	5.80297	5.98472
VBP1	P61758	6.21207	5.92515	6.84909	5.4974	6.73573	6.55957	6.29115	6.59925	6.51959	6.46522	6.86206	6.49738	6.18243	5.58587	5.66814	5.54989
ELMO2	Q96JJ3	6.23532	6.31629	6.88814	5.80367	6.32965	6.67977	6.02229	6.79406	6.12347	6.495	6.59735	6.27825	6.03818	6.05346	5.76554	5.58557
MAVS	Q7Z434	6.39093	6.08351	5.83033	6.86235	6.44315	5.7162	6.56572	6.44461	6.03993	5.88447	5.85052	5.99114	6.94897	6.56263	6.7721	6.61344
VASP	P50552	6.24558	5.99587	6.48315	5.80135	6.80481	7.04829	6.2954	6.61332	6.55885	6.13489	6.89521	6.34317	6.28829	6.07508	5.246	5.17074
NEDD4	P46934-4	6.44758	6.311	6.73999	6.05501	6.31892	6.68378	6.25981	6.50424	6.45098	6.44894	6.58809	6.43531	5.63719	5.81707	5.60741	5.69467
BCKDHA	P12694	6.28214	6.13998	5.84392	6.44579	6.45601	6.31799	6.54367	6.21283	6.24383	6.18284	6.35812	6.40702	6.33806	5.9466	6.22334	6.05786
BRPF3	Q9ULD4	6.74944	6.94749	6.20508	7.13378	5.8545	5.75499	6.29549	6.01673	6.61077	6.59754	6.26024	6.40261	5.80714	5.73973	5.76938	5.8551
RAB5C	P51148-2	6.56897	6.75846	6.24665	6.41225	6.55734	6.33414	6.33742	6.3524	6.17907	6.23825	5.90355	5.74592	5.95098	6.03303	6.27859	6.10299
EXOC7	Q9UPT5	6.48083	6.53229	6.80005	5.91465	6.7478	6.85722	6.29074	6.42968	6.56624	6.85814	6.43692	6.41449	5.54222	5.665	5.16973	5.29399
GTPBP1	O00178	6.36866	6.1997	7.00348	6.07175	6.04163	6.52151	6.25948	6.32603	6.12772	6.56111	6.79078	6.66256	5.7836	5.91976	5.64659	5.71564
AK3	Q9UIJ7	6.59886	6.14104	5.81794	6.09311	6.55447	5.85998	6.164	6.36361	6.07921	6.09739	5.86706	5.9107	7.11128	6.36044	6.50077	6.48015
FAM120B	Q96EK7	6.20437	6.21033	6.71549	6.3787	6.026	6.29636	6.36906	6.21422	6.54003	6.45262	6.43678	6.39766	6.06897	5.73977	5.91179	6.03784
SOS1	Q07889	6.23766	6.11274	6.83365	6.01743	6.1461	6.86821	6.4453	6.27356	6.40473	6.447	6.41417	6.5237	5.75675	6.18575	5.67009	5.66316
TRIP6	Q15654	6.38201	6.05481	6.78107	6.2592	6.51515	6.43626	6.07733	6.67138	6.5502	6.44232	6.41838	6.67349	5.82714	5.47208	5.60982	5.82937
ACOT9	Q9Y305-4	6.35027	6.45664	5.67744	6.72305	6.17391	6.06235	6.49812	6.03876	6.16382	5.96047	5.80899	5.92458	6.56735	6.41753	6.43326	6.74348
VPS45	Q9NRW7	6.43444	6.76353	6.13309	6.56318	6.25219	6.47887	6.4042	6.16876	6.17986	6.34146	6.59175	6.5021	5.62054	5.89736	5.56532	6.10336
ROCK1	Q13464	6.1781	6.18998	6.52761	5.63198	6.49621	6.34197	6.07497	6.33604	6.55102	6.56407	6.54835	6.55599	5.91304	6.19476	5.89351	6.0024
PEX1	O43933	6.4129	6.27621	6.49035	6.62289	6.16338	6.42982	6.47279	5.99684	6.52981	6.42339	6.64531	6.43894	5.87279	5.61804	5.77241	5.83414
FAM91A1	Q658Y4	6.31085	6.38843	6.42658	6.1802	6.19783	6.27408	6.22041	6.43853	6.37158	6.42645	6.50065	6.55862	5.90689	5.98901	5.96921	5.84065

TRIM33	Q9UPN9	6.56517	6.48006	6.52811	6.46602	6.21121	6.09431	6.19214	6.07162	6.46928	6.6198	6.64418	6.43207	5.95842	5.83137	5.64159	5.79462
RALGAPB	Q86X10-3	6.32448	6.08136	6.77992	6.24317	6.34276	6.69408	6.29538	6.59251	6.42705	6.31045	6.33367	6.50758	6.04008	5.74207	5.75986	5.52558
MCTS1	Q9ULC4-3	6.09161	6.0267	7.13126	5.91411	6.32076	6.63573	6.20214	6.52892	6.13657	6.61593	6.80894	7.03695	6.11089	5.52725	5.46749	5.44475
PI4K2A	Q9BTU6	6.3825	6.38838	6.47531	6.62189	5.90191	5.95245	6.41973	6.0244	6.55188	6.48098	6.48093	6.67196	6.03831	5.79211	5.85127	5.96599
NR3C1	P04150-3	6.00005	5.87128	6.30377	5.79405	6.10738	6.31994	6.09044	5.91648	5.9208	6.29023	6.24253	5.88434	6.88379	6.94882	6.76381	6.6623
MTCH1	Q9NZJ7	6.1654	6.58949	5.92426	6.90333	6.07839	6.05918	6.33956	6.19536	6.19453	6.15709	6.05514	6.05932	6.43825	6.07999	6.3611	6.39962
NT5C3A	Q9H0P0	6.64262	6.50477	5.87239	6.72049	6.52251	6.01282	6.474	6.11817	6.38974	5.91464	5.76776	6.11035	6.35023	5.85748	6.40887	6.33317
IFT140	Q96RY7	6.20311	6.12726	6.47256	5.97365	6.21645	6.35361	6.21403	6.19773	6.33753	6.25335	6.32513	6.05162	6.39344	6.57951	6.4113	5.88971
SNRNP40	Q96DI7	5.76263	6.43617	5.72076	6.66238	6.45575	6.14953	6.86136	6.16341	6.67635	5.57664	6.09772	5.8423	6.01602	5.98954	6.84795	6.74149
GYG1	P46976	6.39444	6.51197	7.23192	6.31253	6.65204	7.1471	6.27671	6.89574	6.57435	6.54846	6.95443	6.51196	5.35305	4.94699	4.93066	4.75765
PGRMC2	O15173-2	6.45081	5.87427	5.70969	6.00536	6.07229	5.60957	5.9961	6.15477	5.40188	6.2365	5.71721	6.34703	6.76262	7.26887	7.03325	7.35979
TAF4	O00268	6.57209	6.4287	6.16338	6.56984	6.24807	6.13353	6.23579	6.11989	6.18506	6.56948	6.15567	6.15949	6.66216	5.9877	6.39723	6.01194
SUB1	P53999	5.84534	5.73054	6.29504	5.3614	5.81044	6.30554	6.17985	5.78651	6.16246	5.98012	6.75231	6.0754	5.91556	7.24377	7.27916	7.27656
TMEM209	Q96SK2	6.15445	6.12571	5.6875	6.72705	5.97077	5.82184	6.541	6.00657	6.05162	5.43458	5.80834	6.12951	6.36968	7.01905	6.94848	7.20386
XPC	Q01831	6.13022	6.03827	5.66581	6.63816	5.94383	5.77611	6.10262	6.16045	6.15463	6.21029	5.75398	5.97926	6.24656	7.20426	6.92467	7.07089
CWF19L1	Q69YN2	5.45972	5.92773	6.10017	5.56824	5.80047	6.09973	5.61307	6.08777	5.93514	6.34832	6.33183	6.11697	6.84078	7.26208	7.46877	7.03921
PDLIM5	Q96HC4	5.90505	5.98831	6.66419	5.85219	6.38549	6.33876	6.23278	6.19624	6.3998	6.23859	6.5323	6.48116	6.35223	6.27125	6.20878	5.95286
RBPJ	Q06330	5.53842	6.19764	5.9049	5.85136	5.88444	6.14323	5.86136	5.88915	6.02949	6.5231	6.25497	6.18238	6.56525	7.10235	7.1691	6.90284
VAV2	P52735-2	6.15711	6.21942	7.25232	6.02533	6.08454	6.44287	6.23977	6.37387	6.44375	6.5796	6.72666	6.43031	5.75	5.83246	5.65175	5.79026
SCML2	Q9UQR0	6.22098	6.6538	6.13176	7.05623	6.3229	5.98705	6.35219	5.94203	6.11349	6.26188	6.0225	6.07196	6.19327	5.89607	6.33826	6.43562
SDF4	Q9BRK5	7.44335	7.52781	6.87507	7.38452	7.00794	6.54111	6.42329	6.4065	7.21035	6.79674	7.04056	6.84601	4.25598	4.24395	3.78405	4.21277
MOSPD2	Q8NHP6	6.15511	6.2726	5.94795	6.47167	5.92925	6.34748	6.21035	5.97378	5.92721	6.40331	5.97608	5.88528	6.47198	6.40776	6.61828	7.00192
HM13	Q8TCT9-2	6.58	6.14548	6.07091	6.46572	6.17635	5.77028	6.59049	5.89581	6.251	5.97073	6.09704	5.7983	6.45277	6.39423	6.67758	6.6633
RPS12	P25398	6.40527	5.05497	6.40147	5.26126	6.68128	5.73993	5.69546	6.63848	5.73607	6.80263	6.38738	7.00294	5.98223	6.84063	6.69202	6.67797
PTGES3	Q15185	6.14615	6.2902	6.24135	6.07708	6.67076	6.753	6.1939	6.46613	6.14355	6.19415	6.52551	6.17079	6.33181	5.89988	5.67263	6.22311
BAG2	O95816	6.39512	6.06213	6.73079	5.89978	6.41409	6.14005	6.19741	6.17427	6.12136	6.34646	6.75903	6.6253	6.55345	5.63523	6.11365	5.83188
UCHL5	Q9Y5K5-3	6.19076	6.14922	6.37973	5.9517	6.69266	6.30985	5.99988	6.17036	6.09519	6.18559	6.28355	6.31211	6.59428	6.23987	6.24952	6.1927
SET	Q01105	5.61291	5.85907	6.00941	5.50755	6.20138	6.29001	5.92354	5.84774	5.98662	6.10383	6.18696	5.84579	7.6473	7.05793	7.15828	6.7617
RDH11	Q8TC12	6.56091	7.0045	6.31874	6.70523	6.41171	6.00499	6.19075	6.03158	6.15714	5.89705	5.98439	6.06326	6.14203	6.22538	6.39976	5.90259
CABIN1	Q9Y6J0	6.29199	6.24865	5.93877	6.7747	6.3881	6.08842	6.41889	5.9564	6.30003	6.09729	6.0521	6.20074	6.50311	6.02494	6.39164	6.32426
PON2	A0A0J9YXF2	6.82484	6.53128	5.8949	6.31529	6.40859	6.03883	6.19125	6.24293	6.0944	6.10304	5.65127	5.77465	6.69461	6.27763	6.59886	6.35763
TXNL4A	P83876	6.01909	5.99549	5.9921	6.01304	6.50519	6.23162	6.11952	6.26055	6.48013	6.29827	6.16281	6.07779	6.75765	6.05108	6.72737	6.30829
RPF2	Q9H7B2	6.11672	6.50188	5.57415	6.93328	6.05509	5.83586	6.26294	6.30804	5.77234	5.87164	5.6837	6.05064	6.48045	6.80156	6.82794	6.92377
RPS17	P08708	6.273	5.59622	6.40816	5.43882	6.08267	6.56827	6.35823	6.33001	6.70087	6.46977	6.38807	6.37707	5.74008	6.0928	5.53095	6.54501
LUC7L3	O95232	5.56697	5.98005	5.77631	6.45264	5.73406	5.76704	6.83718	5.46965	6.41493	5.50726	5.62472	5.97647	6.30928	7.42903	7.18813	7.96627
PPP1R8	Q12972	6.07583	5.96823	5.95598	6.07024	6.07062	6.09176	6.15336	6.24785	6.22926	6.09618	5.82507	6.11102	6.82195	6.59276	7.0496	6.64028
CTNNA2	P26232	6.46094	6.32364	6.34277	6.60098	6.54137	6.12524	6.06392	6.04895	6.71181	6.4219	6.18765	6.43852	6.0711	5.69255	5.7244	6.24428
EFHD2	Q96C19	6.876	6.43915	6.58054	6.17226	6.65613	6.51191	6.01477	6.50479	6.58191	6.14961	5.98574	6.33544	6.18832	5.81388	5.52879	5.66076
IRGQ	Q8WZA9	6.27621	5.07169	6.96816	4.78895	6.69559	6.09298	5.84427	7.29677	5.61923	6.99257	5.99197	7.38408	6.29262	6.44472	6.1131	6.12709
NAP1L4	Q99733-2	6.41358	6.26145	6.73916	5.90267	6.40678	6.86782	6.07217	6.89089	6.55774	6.42015	6.94484	6.91695	5.45275	5.49039	5.23621	5.42645
LRRC8A	Q8IWT6	6.3851	6.45839	5.98229	6.58354	6.31794	5.75814	6.44942	6.02971	6.40713	6.13893	6.37764	6.04811	6.38159	5.96836	6.48955	6.22416
NDUFV2	P19404	6.37184	5.18924	5.54607	4.96428	5.68349	4.41824	5.48073	6.55587	4.87746	6.13679	5.56333	6.90059	6.66984	8.60903	8.29365	8.73954
ZC3H7B	Q9UGR2	6.10976	6.36486	6.61648	6.2006	6.28696	6.59664	6.33226	6.29472	6.42441	6.6578	6.57946	6.61779	5.67912	5.91651	5.6994	5.62322
TPM4	P67936	6.13975	6.22129	6.2891	6.33977	6.80457	6.90531	5.92836	6.85449	6.1116	6.3196	6.5904	6.63506	6.21628	6.27485	4.99108	5.37848
SRPK1	Q96SB4-3	6.43267	6.13992	6.85756	6.51634	6.02077	6.21452	6.27644	5.80922	6.59136	6.72163	6.6126	6.80352	5.50324	5.65527	5.99777	5.84717
VPS18	Q9P253	6.23007	6.27207	6.65616	6.11519	6.4298	6.16714	6.40076	6.4257	6.21411	6.4813	6.49946	6.32907	5.95855	5.95774	6.01178	5.85112
KIF5A	Q12840	6.32112	6.27107	6.58435	6.10647	6.34246	6.75097	6.4047	6.6774	6.41653	6.65125	6.34209	6.40258	5.61439	5.83683	5.66642	5.61136
AHDC1	Q5TGY3	5.96743	6.47361	5.77862	6.69293	5.68126	5.8631	6.36536	5.98539	6.58303	6.38794	5.97589	6.05086	6.37769	6.58882	6.38558	6.84249
YWHAQ	P27348	6.28595	6.05208	6.82383	5.77569	6.07511	6.65156	6.03848	6.45943	6.3341	6.5604	6.64402	6.41963	6.17434	6.09219	5.91086	5.66635
STXBP1	P61764-2	6.25476	6.35092	6.45806	6.13426	6.25465	6.53908	6.10539	6.38797	6.33327	6.33786	6.35997	6.34532	5.99222	5.96232	6.22832	5.95564
TDRKH	Q9Y2W6	6.35104	6.23472	5.98473	6.6582	6.36008	6.27607	6.35924	6.2027	6.1904	6.24375	6.22099	5.74281	6.42855	6.17793	6.3665	6.20229
CELF1	Q92879-4	6.27686	6.60534	6.16588	6.36333	6.24541	6.07667	6.35035	6.05915	6.08842	6.25031	6.40651	6.19994	6.472	5.92832	6.31045	6.20104
EXOSC7	Q15024	6.13807	6.01657	6.13923	6.20291	6.15385	6.18761	6.22714	5.84758	6.14636	6.0425	6.27004	6.1231	6.83046	6.37759	6.7625	6.53448
FDXR	P22570-3	6.44102	6.28248	5.99439	6.3789	6.44156	6.07343	6.13343	6.23666	6.21657	6.15603	5.86386	5.88695	6.8898	6.30729	6.26055	6.43707
SREBF1	P36956-4	6.82757	6.75814	6.15502	6.55509	6.31354	6.2174	6.08392	6.00661	6.95833	7.35268	6.95512	6.76837	5.19017	5.26227	5.23347	5.3623
RBM33	Q96EV2	5.98951	6.08175	6.05304	6.02453	5.89314	6.10193	6.14861	6.20923	6.1608	6.37964	6.1931	6.01126	6.99527	6.39047	6.66826	6.69946
DIMT1	Q9UNQ2	6.27257	6.38701	5.9916	6.6012	6.38444	6.34194	6.50892	6.05124	6.14419	5.95946	5.98466	6.09007	6.23191	5.91118	6.66335	6.47627
ROBO1	Q9Y6N7-2	6.36275	6.29186	6.23054	6.452	6.23079	6.01417	6.29554	6.15376	6.32558	6.30564	6.19886	5.99617	6.21751	6.50165	6.13279	6.30839
CYC1	P08574	6.26028	6.17242	6.11827	6.48296	6.61929	5.76537	6.60881	5.99252	6.27816	6.54604	6.28203	6.39151	6.42746	6.2545	6.44086	6.25951
SCAF1	Q9H7N4	6.003	6.3582	5.64054	6.69634	5.71237	5.73858	6.04851	6.00309	5.84641	6.03305	5.81225	6.18217	6.16613	7.19117	7.00023	7.56795
PRKAG2	Q9UGJ0	6.05415	5.99827	6.06188	5.47075	6.21719	6.18147	5.91567	6.37986	6.16517	6.22316	6.21667	6.26829	6.91615	6.48702	6.74707	6.69722
MEPCE	Q7L2J0	6.16403	6.17621	6.05904	6.40065	5.96015	5.81664	6.04135	5.86313	6.05344	6.07839	6.10832	6.10329	6.71006	6.49935	7.15973	6.80622
POLR3B	Q9NW08	5.95334	6.52133	6.18668	6.29209	6.23781	6.31819	6.12605	6.08572	6.26111	6.40476	6.30768	6.31334	6.24061	6.08098	6.356	6.31429
OSBPL3	Q9H4L5	5.66477	5.77858	6.04069	5.46679	5.82757	5.80163	5.81258	5.67263	6.36545	6.43578	6.34466	6.19686	7.23389	6.70272	7.45525	7.20015
PPP1R21	Q6ZMI0	6.22306	6.24529	6.30331	5.73173	6.17971	6.66003	6.07708	6.08345	6.35506	6.58692	6.77982	6.36568	6.54798	6.45223	5.84038	5.56827
TRMT6	Q9UJA5	5.348	6.20037	5.82644	5.42861	5.99574	6.3442	5.97024	6.15403	6.00667	6.19634	6.07339	6.13139	7.14402	6.92276	7.38263	6.87517
WDR35	Q9P2L0	6.22313	6.34628	6.63986	6.06475	6.5036	6.54302	6.31229	6.42662	6.63925	6.43954	6.30194	6.42595	5.95606	5.72146	5.71276	5.74348
NAV1	Q8NEY1	5.8399	6.08739	6.82653	6.14405	6.06277	6.43348	6.15426	6.25272	6.39413	6.5262	6.77782	6.52724	5.86804	5.99328	6.00904	6.10314
LASP1	Q14847	6.47727	5.9727	6.69346	5.82869	6.68476	6.62705	6.00172	6.94844	6.59966	6.35828	6.80541	6.44955	6.2912	5.85437	5.28739	5.12008
SCCPDH	Q8NBX0	6.45417	6.3534	5.80362	6.68694	6.64826	6.20039	6.48258	5.97086	6.14248	5.91406	6.06646	5.62296	6.65569	6.52012	6.36443	6.11359
NELFCD	Q8IXH7	5.91254	5.9839	6.13809	5.7497	6.25626	6.19666	6.17891	5.92966	5.90088	5.93819	6.21177	5.99857	7.20414	6.71624	6.94178	6.74271
CHID1	Q9BWS9-2	6.18716	6.09033	5.98948	6.71159	6.33272	6.05347	6.41211	5.82197	6.26761	6.15302	6.05659	5.73771	6.66834	6.27748	6.68134	6.55907
HMGB1	P09429	5.93437	5.93783	6.30932	5.69413	6.17725	7.03066	5.97253	6.50098	5.64774	5.9791	6.34538	6.06257	6.17036	7.75545	6.47923	6.00311
LACTB2	Q53H82	6.29262	6.22382	6.36681	6.08697	6.39452	6.21459	6.47981	6.04436	6.29128	6.20059	6.5511	6.23139	6.40254	6.07048	5.98702	6.1621
ATP6V0A1	Q93050	6.1496	6.61781	6.44893	6.58577	6.00951	6.16366	6.28781	6.50702	6.13718	6.33047	6.49618	6.18132	5.88311	6.08471	6.13303	5.98391
GATD3B	A0A0B4J2D5	6.30335	5.91321	5.65575	6.07713	6.26934	5.97199	6.02163	6.16324	5.79316	6.30143	6.31242	6.51135	6.91197	6.50784	6.6398	6.64639
SEC24D	O94855-2	5.9737	6.11195	6.51372	5.77599	6.17025	6.53803	6.25691	6.25223	5.98747	6.22745	6.30952	6.05995	6.62786	6.59766	6.33456	6.26689
ANKRD52	Q8NB46	6.6878	6.49567	7.2703	6.39152	6.41346	6.78101	6.25562	6.52593	6.85371	6.61341	7.03542	6.85105	4.90435	4.86753	5.03832	5.01488
TXLNG	Q9NUQ3	6.38865	6.45822	6.70873	6.27793	6.32033	6.70465	6.07247	6.46196	6.43922	6.43761	6.66211	6.74284	5.35066	5.80528	5.37683	5.79249
EXOC2	Q96KP1	6.42222	6.3399	6.54105	5.80856	6.43588	6.3797	6.24187	6.38293	6.96744	6.75745	6.43925	6.31103	5.94975	5.84061	5.69522	5.48714
ACOT2	P49753	6.24495	6.32491	5.98429	6.42235	6.44626	6.12477	6.07377	6.20895	6.2559	6.12951	5.85514	5.93043	6.7134	6.3732	6.54544	6.36673
PKN1	Q16512-2	6.80887	6.4115	6.93749	6.28859	5.96594	6.23692	6.19156	6.54641	6.70337	6.52635	7.11466	6.92071	5.58083	5.1846	5.27037	5.31183
PPP1R10	Q96QC0	6.37713	6.30618	6.1386	6.82716	5.76915	5.82498	6.19423	5.78291	6.35115	6.50913	6.16325	6.5473	6.1265	6.12917	6.44069	6.51247
DBNL	Q9UJU6-3	6.39849	6.21444	6.6493	5.76039	6.55048	6.74603	6.05785	6.81732	6.22112	6.51193	6.477	6.70549	6.26812	5.6397	5.46594	5.5164
ARPC4	P59998-3	5.95404	5.68915	6.47232	5.8363	6.07899	6.63883	6.07521	6.45112	6.1116	6.33241	6.51525	6.3667	5.93312	6.76454	6.3616	6.41883
TBK1	Q9UHD2	6.47244	6.29774	6.70762	5.98627	6.31083	6.87031	6.30077	6.6643	6.32825	6.37506	6.16232	6.48364	5.63102	5.94675	5.49088	5.97181
DOCK9	Q9BZ29-4	6.31029	6.3183	7.055	6.28182	6.23196	6.20995	6.09352	6.12296	6.62217	6.446	6.73782	6.56848	5.6793	5.73792	5.982	5.60251
TOR4A	Q9NXH8	6.87353	6.8946	6.27272	6.9598	6.15726	5.79962	6.54254	6.14853	7.20982	6.6979	6.67797	6.53288	5.56039	5.24647	5.2019	5.22409
MYL6	P60660	6.37866	5.26245	5.68164	4.44093	5.91256	5.40737	5.4292	7.27964	5.18185	6.39721	5.6446	7.03687	5.9419	8.78871	7.05764	8.15879
CDK5RAP1	Q96SZ6-3	6.58062	6.53633	6.43038	6.73668	6.37478	6.13026	6.63367	6.25339	6.13488	6.03341	6.37591	6.25608	6.01482	5.45958	5.9504	6.09879
TMTC3	Q6ZXV5	6.22554	6.3945	5.77203	6.5396	6.13069	5.86746	6.20765	5.87895	6.0567	5.89437	5.98878	5.99781	6.66966	6.68627	6.95323	6.73675
APEH	P13798	6.6728	6.15823	6.58556	5.89114	6.68113	6.91103	6.05509	6.55732	6.38169	6.42907	6.5182	6.56609	5.75205	5.99123	5.4022	5.44717
UBA3	Q8TBC4	5.81054	5.77763	6.08428	5.21105	5.91031	6.41871	5.74074	6.52372	5.7511	6.13591	6.19968	6.16086	6.822	7.71308	7.25272	6.48767
CACTIN	Q8WUQ7-2	5.92335	6.22345	6.1497	6.50272	5.68167	6.13769	6.1153	5.82403	6.2041	6.15817	6.41268	6.36746	6.49211	6.37717	6.79296	6.63745
PRDM2	Q13029	6.55438	6.44648	6.08911	7.2125	5.80562	6.0033	6.48238	6.12358	5.81802	6.17868	6.26104	6.29366	6.07336	6.14394	6.25871	6.25526
ERMP1	Q7Z2K6	6.15409	6.42892	5.65762	6.18367	6.11249	5.97622	6.40211	6.08361	6.03318	6.09764	5.86769	5.90365	6.64105	6.63043	6.76838	7.05925
TSPYL4	Q9UJ04	6.46616	6.27737	6.98164	5.96304	6.08281	6.86077	6.36207	6.45166	6.51852	6.47942	6.56684	6.42189	5.67998	5.70783	5.50022	5.67976
INTS5	Q6P9B9	6.43133	6.33292	5.87883	6.41806	6.27373	5.90709	6.22546	6.13735	6.21762	5.85087	6.18998	6.06521	6.65074	6.32262	6.51914	6.57904
RAB3B	P20337	6.41794	6.62188	6.03572	6.60158	6.21165	6.46229	6.18111	6.15938	6.21105	6.22497	5.71102	5.97788	6.24378	6.2009	6.35959	6.37924
COIL	P38432	6.1931	6.40544	6.01779	6.90781	5.9752	5.82763	6.35771	5.9339	6.15225	5.96761	6.04762	6.06626	6.55085	6.37147	6.60111	6.62423
COQ8B	Q96D53	6.57664	6.70981	5.94732	6.4661	6.37274	6.02669	6.24769	6.01943	6.08973	6.06638	6.01986	5.85025	6.67911	6.24955	6.50051	6.17821
ELOA	Q14241	6.06791	6.23057	5.9456	6.46106	6.23207	5.90422	6.21648	6.05477	6.00602	5.84612	6.04998	5.86087	6.46076	7.02613	6.85529	6.78214
ACOX1	Q15067-2	6.25647	5.75018	5.93804	5.998	6.05683	6.29421	6.3738	5.83909	6.17469	6.23759	6.1852	6.10417	6.84058	6.62262	6.49196	6.83656
TUBA1C	Q9BQE3	6.41916	6.00015	7.54484	6.49693	6.4599	6.92437	6.42661	6.12474	6.36299	6.31912	6.76969	6.67186	5.82229	5.26131	5.21415	5.18189
IFT172	Q9UG01	6.28763	6.68117	7.10516	6.2182	6.15181	6.63527	6.02242	6.48086	6.35747	6.98822	6.85426	6.77579	5.06321	5.78403	5.33544	5.25904

ZNF217	O75362	6.14599	6.22059	6.379	6.77967	6.15059	6.15091	6.70331	6.1622	6.47121	6.46955	6.26588	6.56634	5.87945	5.67229	5.82991	6.15312
CRYZ	Q08257	6.28283	5.87729	6.24679	6.33499	6.26705	6.36625	6.0927	6.34807	6.14437	6.48544	6.32685	6.18305	6.25117	6.2269	6.29028	6.27597
OGFR	Q9NZT2	5.4323	5.72083	6.16567	5.46668	6.03818	6.69644	5.92805	6.44312	5.95608	6.19671	6.29477	6.05652	7.03409	7.09448	6.84294	6.63313
KDM3A	Q9Y4C1	5.75497	5.9943	6.07913	5.5773	5.74694	6.35968	5.79571	5.81859	5.99795	6.44159	6.3708	6.16038	6.97145	7.35948	6.80357	6.76819
SLC9A3R1	O14745	6.56009	6.09113	6.59793	5.51857	6.68053	6.45595	5.91391	6.78707	6.11781	6.50741	6.67182	6.77194	6.67994	5.78295	5.31504	5.54791
VPS33B	Q9H267	6.17573	6.5226	6.68542	5.79425	6.28147	6.81286	6.1415	6.63065	6.38827	6.72318	6.42048	6.3165	5.85272	6.00751	5.57247	5.67439
ATP5PD	O75947	6.03307	5.07291	4.95181	4.98628	5.84716	4.98642	5.39428	6.98517	4.94192	6.28052	5.29433	6.36359	6.43463	9.05762	8.15632	9.21398
CARM1	Q86X55	5.76511	5.87145	6.66659	5.72013	5.89654	6.37901	6.05353	6.09633	6.54094	6.49071	6.52578	6.24476	6.33716	6.50863	6.55103	6.35228
PLEKHG2	Q9H7P9	6.1965	6.13151	6.67827	5.85832	6.38013	6.33855	6.34207	6.56399	6.15019	6.46575	6.65178	6.49217	6.07338	6.00056	5.93453	5.74227
NIT2	Q9NQR4	6.43755	6.10956	6.64885	5.76907	6.32081	6.55059	6.05506	6.68236	6.59333	6.25935	6.33086	6.58444	6.03405	6.1834	5.81018	5.63055
NAGK	Q9UJ70-2	6.27297	6.12826	6.81364	5.98701	6.34184	6.82975	6.00603	6.68981	6.2929	6.22016	6.4014	6.39055	5.82162	6.16371	5.81868	5.82167
FAH	P16930	6.50957	5.92172	6.90569	5.46458	6.50195	6.64101	5.96222	7.06992	6.20616	6.57408	6.41232	6.60103	6.08061	5.86598	5.61893	6.56423
HELZ2	Q9BYK8	6.49014	6.39706	6.48233	6.2811	6.13953	6.34701	6.54068	6.94558	6.28911	6.72242	6.45368	6.40723	5.96385	5.80912	6.13843	5.59275
GON4L	Q3T8J9-3	6.44607	6.86927	6.50926	6.95089	6.14848	6.35467	6.38812	6.35644	6.22954	6.25356	6.3318	6.15794	5.58604	5.79729	5.46038	6.16024
RO60	P10155	5.6354	6.25114	6.06291	5.55509	6.17554	6.42888	6.00869	6.31477	5.7407	6.41898	6.269	6.12176	6.86776	6.97782	6.75205	6.41951
INTS8	Q75QN2	6.51419	6.42461	6.03985	6.4577	6.23342	5.89057	6.45473	5.79944	6.33894	6.14261	6.19073	5.98786	6.66584	6.01333	6.65197	6.19522
RGPD8	O14715	6.53974	6.38912	6.04579	6.81291	6.18031	5.92862	6.47842	6.45726	6.29151	6.08515	5.90253	6.04151	6.25344	6.15653	6.1114	6.32576
NBEAL1	Q6ZS30	6.05748	6.02561	6.57755	6.36018	6.42079	6.25508	6.21177	6.06696	6.41306	6.24055	6.3149	6.49269	6.08162	6.31919	6.14457	6.01799
WDR81	Q562E7	6.52619	6.45578	6.69069	5.88426	6.56996	6.45741	6.28728	6.76691	6.73138	6.72441	6.79912	6.78544	5.34893	5.77268	5.02109	5.17848
SFSWAP	Q12872-2	6.28146	6.35215	6.01609	6.69178	6.13622	5.81436	6.36312	5.86302	5.93867	6.0387	6.03514	5.96554	6.38846	6.61543	6.69698	6.80287
BRCA2	P51587	7.11102	7.08504	6.63513	6.91753	6.16108	6.36574	6.34179	6.31852	6.72848	6.59237	6.70627	6.70331	5.11707	4.82354	5.07058	5.32253
SUZ12	Q15022	6.47323	6.51344	6.29855	6.92405	6.20584	5.99016	6.43975	5.83282	6.41042	6.33317	6.47142	6.35621	5.87951	5.75765	6.00983	6.10396
VPS16	Q9H269	6.46335	6.51142	6.52304	6.05497	6.35017	6.6568	6.37195	6.39742	6.49609	6.62488	6.38438	6.22011	5.87436	5.75236	5.78182	5.53689
SNX4	O95219	6.25485	6.36304	6.40137	6.01293	6.05188	6.34522	6.1357	6.56364	5.94834	6.70729	6.19419	6.62549	6.06468	6.36152	5.93129	6.03856
KRR1	Q13601	6.06036	6.25229	5.81218	6.62479	6.00721	5.88285	6.45805	5.95859	5.9906	5.85819	5.85571	5.69667	6.73918	6.6588	7.04131	7.10322
CTSA	P10619	6.47393	6.23331	6.21101	5.99315	6.40521	6.06975	6.02059	6.19023	6.01808	6.43909	6.35335	6.17413	6.67551	6.08887	6.84264	5.81114
ERLEC1	Q96DZ1	6.40234	6.52079	5.86319	6.49206	6.49315	6.20835	6.19321	6.1469	6.00852	6.19753	6.2684	5.8495	6.73038	6.21751	6.33683	6.07133
CWF19L2	Q2TBE0	5.45916	6.08349	5.85851	5.90646	5.67543	5.91022	5.87996	5.94483	5.86726	6.10193	6.06934	5.86715	6.62621	7.58075	7.43468	7.73464
DENND4C	Q5VZ89-7	6.35046	6.30436	6.74564	5.95968	6.17604	6.3818	6.34521	6.36974	6.79248	6.78031	6.42289	6.65004	5.68164	5.73678	5.56641	5.73652
DDX51	Q8N8A6	6.58174	6.1361	6.0449	6.95173	6.09391	5.74066	6.42865	5.57134	6.2968	6.03989	6.11959	6.20332	6.57032	6.21045	6.51761	6.49298
SNX9	Q9Y5X1	6.23328	5.84593	6.83012	6.24197	6.11822	6.5384	5.81006	6.22732	6.26016	6.3721	6.39076	6.56181	6.44748	6.1433	6.10889	5.87021
CRAT	P43155	6.3242	6.30147	5.9353	6.36361	6.34851	5.75704	6.63701	5.98823	6.24358	5.90758	5.77329	5.62526	6.74058	6.53657	6.86054	6.65724
H2BC20P	Q6DN03	6.0885	5.93998	5.67874	6.27108	5.94528	5.70139	6.35294	5.86627	5.84057	5.66313	5.97593	5.96869	7.00253	7.14882	7.39659	7.15956
BRD7	Q9NPI1-2	6.05686	6.67408	5.98358	6.54257	5.97534	6.04783	6.13037	5.96584	5.79272	5.99359	6.19122	6.1483	6.33425	6.75241	6.5616	6.84945
HSPA13	P48723	6.54827	6.59586	6.05966	6.46342	6.25991	6.05237	6.05877	6.23023	6.1273	5.96809	5.90707	6.11895	6.65096	6.22053	6.51324	6.22535
RPL26	P61254	5.73996	6.28015	6.23394	6.24049	5.85995	6.43886	6.89644	6.48628	6.75005	5.85361	5.92289	6.43107	5.36366	6.69431	5.9529	6.85545
RREB1	Q92766-2	6.52701	6.30416	5.93076	6.85763	6.31509	6.00396	6.41596	6.24173	6.06647	6.1587	5.95255	5.92506	6.33247	6.23492	6.37387	6.35965
ADAM17	P78536	6.65666	6.7986	6.20111	6.89771	6.51871	6.2424	6.56921	6.23015	6.29908	6.36951	6.02527	6.19972	5.90785	6.54209	5.68493	5.75699
RPL18	Q07020	6.15487	6.41318	5.65039	5.8155	6.13904	6.1549	6.60858	6.51003	6.73169	6.30924	6.52694	6.12414	6.11761	6.60979	6.07439	6.05971
P4HA2	O15460	6.33819	6.7925	6.27454	6.72923	6.37986	6.312	6.23056	6.02225	6.07094	6.70987	6.21107	6.35616	6.05285	5.77083	5.88351	5.86564
LLGL1	Q15334	6.13397	6.37458	6.11089	6.10978	6.27228	6.25815	5.89698	6.69034	6.06647	6.22764	6.04962	6.32542	6.97115	6.11819	6.58947	6.42608
HLA-A	P04439-2	6.49395	6.46062	6.17319	6.56416	6.45807	6.09886	6.16649	6.22857	6.16832	6.1037	6.11572	6.06241	6.4969	6.11947	6.23268	6.05689
RPS5	P46782	6.38382	6.39163	6.2688	5.92224	6.47262	6.36341	6.27823	6.50341	6.58032	6.45153	6.50981	6.41306	6.0781	5.99829	5.69649	5.68823
NHSL1	Q5SYE7-2	6.1864	6.14229	6.87169	5.97122	6.10721	6.38743	6.42064	6.3096	6.23574	6.59434	6.42707	6.59628	6.02995	6.03885	5.84851	5.83278
PLOD1	Q02809-2	6.62185	6.49904	5.69689	6.28559	6.25191	6.09069	6.28171	6.12095	6.14277	6.17331	5.92583	6.00966	6.40594	6.57563	6.55027	6.36797
ATAD1	Q8NBU5	6.32442	6.30238	6.09902	6.50767	6.26497	5.99451	6.25638	6.152	5.97293	5.93161	6.14454	5.88276	6.6174	6.35973	6.73117	6.45852
HSPB1	P04792	5.63139	5.05297	5.93423	4.40473	6.36883	6.10732	5.74837	7.57879	6.13017	7.44956	6.82678	7.27163	4.94679	7.13399	6.40355	7.01091
CRIM1	Q9NZV1	7.07842	6.38704	6.68773	7.56386	7.02382	6.42158	6.60596	6.2926	6.34502	6.16701	6.65305	6.38084	6.56856	4.58553	5.05175	5.08723
CLYBL	Q8N0X4	6.29668	6.21369	5.73837	6.44226	6.19519	5.91372	6.14227	6.31666	6.14223	5.95663	6.0263	5.87967	6.86554	6.50001	6.76904	6.60174
CPM	P14384	6.03444	5.99038	5.8047	6.3216	6.33998	5.86634	6.75097	5.85776	6.56662	5.98277	6.08524	6.03745	6.61581	6.365	6.90201	6.47893
NAPG	Q99747	6.3792	5.41179	6.18241	5.01644	6.76147	6.02407	5.9659	6.83284	6.07302	6.5945	6.30307	6.62668	6.86918	6.34697	6.25992	6.35253
MVD	P53602	6.34594	6.10847	6.99577	5.69339	6.12066	6.65836	6.16487	6.48401	6.45102	6.6559	6.63312	6.63843	5.79147	5.82784	5.82182	5.60353
HDAC1	Q13547	6.37376	6.12311	6.25696	6.22896	6.12509	5.72093	6.39348	5.98642	6.03398	5.98212	6.32878	5.98682	6.68389	6.27551	7.11643	6.38376
C9orf78	Q9NZ63	5.8376	6.0018	6.0611	5.4786	6.17198	6.25608	5.72168	6.24495	6.37405	6.40224	6.54822	6.479	6.65599	6.68788	6.59859	6.48022
SNX14	Q9Y5W7	6.65603	7.17731	6.61084	7.06179	6.80843	6.47898	6.47428	6.3317	6.49469	6.75735	6.47039	6.36778	5.13902	5.15351	4.93414	5.08374
PRKACB	P22694	6.21789	6.35825	6.43318	6.27074	6.36739	6.49329	6.12585	6.55292	6.00269	6.21805	6.38882	5.90947	6.18709	6.08595	6.37695	6.01148
RRM1	P23921	6.39156	6.14332	6.93737	6.18552	6.46194	6.97019	6.4127	6.71061	6.79925	6.72751	6.75318	6.59307	5.34553	5.22985	5.33732	5.00108
VTA1	Q9NP79	6.15687	5.96071	6.75152	5.35067	6.26623	6.21439	6.28522	6.42974	6.11698	6.43286	6.52827	6.49421	6.08015	6.12519	6.3453	6.4617
AACS	Q86V21	6.27074	6.25124	7.25718	6.35275	6.20722	6.91055	5.96333	6.64115	6.51703	6.88076	6.8032	6.79675	5.25196	5.71607	4.96291	5.21716
UTP15	Q8TED0	6.15939	6.37607	5.80658	6.71054	6.44363	6.11408	6.451	6.15969	6.12358	5.95585	5.94935	5.78809	6.4922	6.1877	6.58506	6.68718
PIK3C3	Q8NEB9	6.45605	6.19477	6.68271	5.8943	6.34757	6.55797	6.29934	6.51448	6.31806	6.44504	6.5767	6.44548	5.96993	5.88074	5.68908	5.72781
MAT2B	Q9NZL9	5.2817	5.83519	6.24305	5.27778	6.08653	6.28153	6.23522	6.1494	6.52809	5.9465	6.28542	6.37311	6.5807	6.98874	6.9468	6.96024
TUBG1	P23258	6.37635	6.35742	6.8168	6.27185	6.3108	6.66824	6.19345	6.85238	6.45598	6.58327	6.82202	6.81951	5.23826	5.4206	5.28203	5.55083
NUP88	Q99567	6.40617	6.24497	6.18417	6.46392	6.32296	6.36985	6.31185	6.13309	6.37665	5.7951	6.02016	6.11556	6.53417	6.08795	6.13745	6.49598
MTMR6	Q9Y217	6.08027	6.05129	6.661	6.36141	6.16163	6.36317	6.24011	6.33626	6.19337	6.47437	6.59828	6.72905	5.75521	6.16458	5.76698	6.06304
SYNRG	Q9UMZ2	6.57261	6.44126	6.96588	6.45886	6.32935	6.83862	6.26945	6.23437	6.14499	6.4332	6.86345	6.74513	5.78498	5.53784	5.22055	5.15947
TXN	P10599	6.27909	5.59887	6.53425	5.11798	6.43985	6.81188	5.76303	7.13361	6.39531	6.85121	6.50621	6.97486	6.00801	6.08134	5.61201	5.89251
AAAS	Q9NRG9	6.54728	6.34922	6.25096	6.8924	6.44895	5.8571	6.33049	6.10718	6.11087	5.97974	6.06093	6.11292	6.25646	6.08624	6.27554	6.33373
NMI	Q13287	6.35889	6.1905	6.62098	5.80512	6.29758	6.92086	6.18575	6.57163	6.43502	6.84999	6.53623	6.20398	5.66141	5.95011	5.75225	5.65967
DROSHA	Q9NRR4	6.25768	6.18663	5.63658	6.69382	6.15031	5.82921	6.32405	5.84411	6.66287	6.67919	6.04961	6.19819	6.35785	6.17831	6.4578	6.49379
RMND1	Q9NWS8	6.47568	6.54005	5.87205	7.15422	6.28022	5.85918	6.65614	5.72312	6.0657	5.92367	5.49583	5.45196	6.52594	6.42741	6.66129	6.88754
PEX5	P50542-2	6.77164	6.04407	6.95211	6.03936	6.24954	6.39581	5.94433	7.04577	6.42888	6.51204	6.92848	7.30377	5.52743	5.21505	5.08186	5.55985
NCOA7	Q8NI08-2	5.40918	6.24745	6.2331	6.0538	5.68418	6.30645	5.79601	6.31594	6.21631	6.57409	6.27726	6.7374	6.36915	6.91339	6.25603	6.61025
SCD	O00767	7.02868	7.01062	6.5331	6.81544	5.93815	6.07194	6.74583	5.85506	6.98002	7.19448	6.99999	6.72246	5.03314	5.19559	4.85396	5.02155
FAM50A	Q14320	5.9231	5.93858	6.51265	5.60769	5.96762	6.45858	5.76766	6.40546	6.12431	6.50231	6.5393	6.56523	5.94476	6.73926	6.97798	6.0255
TERF2	Q15554	6.24899	6.13927	6.1893	6.88241	6.32391	5.85403	6.33207	5.84404	6.50916	6.17005	6.33734	6.17097	6.28754	6.19532	6.3008	6.2148
CAPZB	P47756-2	6.33083	6.29352	6.36296	6.34581	6.52242	6.29711	6.1047	6.30464	6.26525	6.26052	6.19943	6.19631	6.03604	6.30958	6.35047	5.82043
RPL38	P63173	6.30636	6.47363	6.56626	6.29952	6.16603	6.50158	6.28059	6.08704	6.46967	6.50215	6.53665	7.27548	5.51308	5.63991	5.77576	5.60631
YTHDC1	Q96MU7	6.24323	6.46807	5.86044	6.80422	6.10611	5.99524	6.42135	6.019	6.08183	5.85303	6.00896	5.97863	6.42844	6.66081	6.70352	6.36714
DNAJB11	Q9UBS4	6.07847	6.15092	5.91381	6.25929	6.31931	6.11703	6.48462	6.00892	6.12366	6.02709	6.08797	6.01635	6.61306	6.68285	6.59539	6.52124
LPCAT1	Q8NF37	6.40711	6.11945	6.0388	6.45294	6.02386	5.79184	6.17769	5.66823	6.35144	6.15924	6.06149	5.79527	6.78748	6.36908	6.96786	6.82823
VPS13D	Q5THJ4	6.05441	6.10457	6.39982	6.2258	5.77795	6.44039	5.94435	5.92342	6.14697	6.27955	6.56496	6.63282	6.34883	6.54159	6.31303	6.30155
MAP3K4	Q9Y6R4	6.28096	5.95665	6.82126	6.1549	6.24762	6.75441	6.17426	6.29127	6.44893	6.54605	6.57466	6.67777	5.7977	6.00024	5.50544	5.76787
CTTNBP2NL	Q9P2B4	5.96968	5.98845	6.23532	5.71898	6.0559	6.02661	5.7135	6.41923	6.26441	6.21819	6.61548	6.51009	7.10118	6.6949	6.35511	6.11298
DAZAP1	Q96EP5	6.34404	6.50124	6.30989	6.61385	6.85378	6.51357	6.57483	6.66764	6.66963	6.13891	6.56435	6.48879	5.7696	5.41538	5.26233	5.31217
UBE2K	P61086	6.28538	5.6663	6.96236	5.3282	6.57639	6.84938	6.17265	6.72814	6.15837	6.54684	6.64897	6.60094	6.11267	6.05291	5.7591	5.5514
QPCTL	Q9NXS2	6.04605	6.34631	5.88488	6.36917	5.9883	6.10084	6.13096	6.30779	5.67104	6.1084	6.18393	5.99956	6.95195	6.80917	6.42879	6.67286
CAB39	Q9Y376	6.54846	6.46911	6.90795	5.92313	6.51634	6.54875	6.14303	6.41816	6.57349	6.50522	6.75912	6.50699	5.63824	5.73465	5.383	5.42433
RPL27	P61353	6.27864	6.54258	6.21669	6.10221	6.22405	6.37575	6.44661	6.1899	6.64082	6.25381	6.60195	6.50815	5.9066	6.21709	5.8005	5.69465
MRPL15	Q9P015	5.98065	5.9172	5.46966	6.60669	6.1953	5.82309	6.67974	6.22721	6.19435	5.79276	5.99122	5.92136	7.08443	6.61352	6.4631	7.03975
APEX1	P27695	5.60802	5.5418	6.22615	5.67067	5.90091	6.39331	5.89555	5.51619	6.0001	6.08787	6.48621	6.12713	6.867	7.71642	7.17018	6.79248
CDV3	Q9UKY7	6.18119	6.28796	6.71392	5.85742	6.59191	6.83615	5.97218	6.72145	6.47097	6.39886	6.64142	6.80292	5.89831	5.82933	5.33671	5.45931
TRMT5	Q32P41	5.91508	5.93659	5.85272	5.89912	5.8055	6.20175	5.98984	6.16039	6.0266	6.45762	6.17325	6.20011	6.502	7.10295	6.89566	6.88081
DES	P17661	6.20812	6.04546	6.00034	6.52427	6.39182	6.19652	6.06455	6.65761	6.40241	6.03264	6.52158	6.45738	6.76762	5.73033	5.95973	6.03962
IST1	P53990-5	6.52356	5.94122	7.0496	5.49268	6.48243	6.80201	6.19241	6.79253	6.30117	6.59577	6.54454	6.77356	6.05691	5.80641	5.36441	5.2808
CDYL	Q9Y232	6.32649	6.22188	6.05294	6.74683	6.16973	5.85275	6.34471	5.90259	6.09378	6.28873	5.89699	6.06012	6.61919	6.16395	6.51982	6.73949
VRK1	Q99986	5.84312	6.0168	5.89311	6.09508	5.7027	5.66123	6.16794	6.07593	6.32507	6.1196	6.43616	6.20616	6.89921	6.76541	6.9531	6.83937
RPL31	P62899	6.48533	6.34758	6.31311	6.27846	6.1652	6.19052	6.40808	6.46342	6.79357	6.2245	6.51438	6.34152	5.76059	6.01553	5.82247	5.87576
REXO1	Q8N1G1	6.2561	6.27273	6.1577	6.93286	5.93675	5.86769	6.20091	5.90981	6.27387	6.3957	6.20899	6.52669	5.91681	6.27208	6.3842	6.48711
RHEB	Q15382	6.5285	6.34764	6.6147	6.04932	6.22313	6.57379	6.4528	6.63843	6.5048	6.20649	6.23336	6.34657	5.74533	6.04178	5.56868	5.92468
RPL21	P46778	6.51392	6.17988	6.28966	6.28161	6.66016	6.19088	6.50429	6.14262	6.65507	6.29648	6.6635	6.04081	5.89685	5.98693	5.95547	5.74186
HECTD3	Q5T447	6.02397	6.21725	7.04942	6.2041	6.0526	6.54264	6.35595	6.40233	6.37658	6.59104	6.90691	6.80882	5.59501	5.75457	5.24884	5.86996
NECTIN2	Q92692	6.36893	6.5544	6.31866	6.66861	6.11802	6.18196	6.27107	6.19833	6.30431	6.16832	6.30571	6.07567	6.26011	5.97492	6.01192	6.21905
MYDGF	Q969H8	6.13068	6.41153	5.92637	6.32712	6.40998	6.34171	6.42865	5.97437	5.99813	5.88122	5.88443	5.95515	6.46414	6.42276	6.88882	6.55493
SNRPD3	P62318	6.44911	6.3601	5.77835	6.68834	6.26771	5.99456	6.55489	6.01767	6.32858	5.66664	5.88065	5.86779	6.5357	6.22414	6.64784	6.73794

CTSC	P53634	5.99264	5.91147	5.72698	5.93083	6.44617	6.20621	6.43834	6.86432	6.2469	6.52367	6.73424	6.34241	6.09085	6.23795	6.21788	6.08914
INTS7	Q9NVH2	6.14366	6.3722	6.11914	6.74635	5.97704	6.02866	6.23316	6.00362	6.0714	6.23378	6.26932	6.15202	6.5695	6.16553	6.55304	6.36157
TFG	Q92734	6.28068	5.53442	6.75068	5.41416	6.06543	6.53967	6.27612	6.41104	5.8903	6.55359	6.91271	6.90941	6.3818	6.28956	5.85989	5.93052
TRIM56	Q9BRZ2	6.40074	6.09223	6.53618	5.97045	6.0139	6.53696	6.27526	6.46207	6.214	6.29872	6.40262	6.40694	6.14769	5.98842	5.98053	6.27328
STRN3	Q13033	6.08349	5.9227	6.25583	5.82921	6.25747	6.25507	5.95074	6.41773	6.27873	6.04779	6.55512	6.4617	6.65131	6.57037	6.37329	6.08945
RPF1	Q9H9Y2	6.65433	6.11347	5.96929	7.15317	6.03418	5.91608	6.37133	6.21861	6.43772	5.85601	6.23464	6.15551	6.24615	5.89785	6.47484	6.26682
SH3BP4	Q9P0V3	6.5353	6.28633	6.3074	6.69235	6.41303	6.2293	6.48261	5.74392	6.56327	6.31066	6.17428	6.24858	6.15317	5.77979	6.29362	5.78639
UBXN4	Q92575	6.59887	6.44639	5.93376	6.68618	6.21749	5.96913	6.5873	5.72371	6.09369	6.1926	6.04262	6.20527	6.25446	6.26153	6.6277	6.15929
RAD18	Q9NS91	6.25057	6.34572	6.11528	6.57398	6.39366	6.12416	6.46869	6.26798	6.13893	5.9466	6.33644	6.41524	6.31167	5.84554	6.19501	6.27053
LIPE	Q05469	6.65424	6.4826	6.95533	6.26852	6.3306	6.45533	6.40554	6.62121	6.71778	6.61607	6.59095	6.48808	5.47976	5.26167	5.19142	5.4809
ANO6	Q4KMQ2-2	6.2552	6.41794	6.12931	6.42234	6.22207	6.10723	6.3045	5.97111	6.0371	6.2608	6.04198	5.98943	6.46209	6.35938	6.59421	6.4253
KIAA2013	Q8IYS2-2	6.51134	6.72424	5.99356	6.63197	6.08796	6.00083	6.35809	5.74748	6.18704	6.39927	6.22287	6.2675	6.32125	6.18816	6.19617	6.16226
LYN	P07948	6.47573	6.39307	6.19085	6.37525	6.30172	6.20142	6.14291	5.98771	6.371	6.25878	6.10284	5.82781	6.43097	6.29494	6.40299	6.24202
MET	P08581-2	6.43972	6.42791	6.2753	6.46783	6.27596	6.0523	6.47727	6.17273	6.24907	6.13972	6.20304	6.12496	6.26364	6.02292	6.27654	6.13111
CDK13	Q14004	5.99049	5.81464	5.78354	6.41169	5.92086	6.0121	6.2194	5.70169	6.06919	6.00826	5.80964	6.21465	6.51048	7.1173	6.93386	7.48221
CDK5	Q00535	6.42002	6.27348	6.65859	6.06006	6.45621	6.93689	6.59264	6.41485	6.32502	6.49812	6.39452	6.63572	5.59232	5.97318	5.62463	5.80965
RPLP2	P05387	6.84232	5.78124	5.43808	5.22279	5.55427	4.95997	4.97257	6.57202	4.78527	6.27186	5.35232	6.5175	5.61024	9.88542	7.18871	9.04541
MYO1E	Q12965	6.49787	6.14746	7.01538	5.95803	6.23843	6.5978	6.54111	6.23398	6.45439	6.94411	6.43375	6.3685	5.78234	5.88812	5.46714	5.43159
EDRF1	Q3B7T1	6.86777	7.12609	7.82286	7.41827	6.80575	6.92786	6.63417	6.74757	7.15406	7.31541	7.62635	7.16504	3.43921	3.82301	3.34894	3.77763
ANAPC2	Q9UJX6	6.33523	6.23914	6.53405	6.11888	6.34376	6.23952	6.10187	6.02108	6.47452	6.34679	6.28315	6.20493	6.16502	6.12406	6.20531	6.26268
PIK3C2A	O00443	6.30255	6.22136	6.73674	6.68873	5.92886	6.56555	6.50238	6.55703	6.14929	6.50077	6.53849	6.66625	5.57545	5.72906	5.69521	5.64228
PAK4	O96013	6.45205	6.12837	6.40645	6.55922	6.07274	6.52886	6.32609	6.61682	6.26168	6.35749	6.1151	6.11886	5.75732	6.45714	6.0478	5.79402
CHUK	O15111	6.36844	6.29112	7.01339	6.10359	6.21299	6.41127	6.25484	6.16238	6.43204	6.63794	6.52693	6.39606	6.05932	5.81271	5.57411	5.74286
UROD	P06132	6.36417	5.40364	6.778	5.00693	6.40161	6.58755	5.8109	6.87796	5.89018	6.65917	6.62113	6.93133	5.89373	6.63353	6.17533	5.96484
CIAPIN1	Q6FI81	6.34634	5.95404	6.91429	5.81359	6.42341	6.67842	6.05053	6.92604	6.28598	6.55753	6.72541	6.91653	5.73606	5.83014	5.36305	5.47865
NUDT19	A8MXV4	6.38677	6.57585	6.23195	7.00338	6.36397	6.24419	6.13231	6.31481	5.92888	5.93998	6.19029	6.02898	6.41255	5.88448	6.43842	5.92318
YEATS2	Q9ULM3	6.21699	6.09285	6.08027	6.93738	6.64966	6.38227	6.62834	5.9778	6.37752	6.53138	6.45306	6.35634	6.07575	5.97643	5.73758	5.82638
MACF1	A0A0A6YYJ5	6.27405	6.51764	6.07462	6.69873	6.15917	6.39249	6.56401	6.00733	6.1271	6.02518	6.10187	6.20396	6.01016	6.12126	6.42344	6.29898
GRWD1	Q9BQ67	5.88982	6.06543	6.36185	6.68629	6.07214	5.94889	5.86231	6.50318	7.01007	6.55987	6.92794	6.69517	5.79384	5.6328	5.89991	6.09048
FBXO38	Q6PIJ6	6.16803	6.53284	6.13819	6.96941	6.46497	6.02631	6.42639	5.8905	6.47836	6.30885	6.17804	6.15616	5.94179	5.93158	6.39581	5.99277
ERLIN2	O94905	6.33476	6.36946	5.84562	6.34453	6.35801	5.99555	6.65507	6.16496	6.05861	5.90003	5.61453	5.83236	6.68843	6.43218	6.72155	6.68435
OBSL1	O75147	6.3975	6.15492	6.53632	6.28701	6.11349	6.16989	6.34751	5.98859	6.32485	6.39349	6.45895	6.31446	6.16076	6.08369	6.33888	5.9297
DHRS7	Q9Y394	6.39067	6.24375	6.04824	6.61501	6.24993	6.01593	6.69126	5.72802	6.18033	5.70729	6.00616	5.76243	6.72094	6.32371	6.74486	6.57147
TANC1	Q9C0D5	6.05919	6.27064	6.61129	6.27473	6.13558	6.4865	6.24189	6.38301	6.28069	6.45081	6.80836	6.67614	5.91436	5.83126	5.77861	5.79695
RPS21	P63220	6.6956	6.75047	6.03871	5.60005	7.13751	6.85832	5.43866	7.81907	5.85936	6.516	6.89755	6.68591	4.95347	5.6877	5.83016	5.23145
NUCB2	P80303	7.40074	5.41171	5.66299	5.52993	6.26117	4.6613	4.69824	7.03931	4.66582	7.19327	5.85997	7.55319	6.46489	7.5862	6.6454	7.36587
LENG8	Q96PV6	6.34391	6.66407	6.22263	7.17034	6.28416	6.01727	6.48162	5.90479	6.08589	6.40106	6.28849	6.20776	6.21802	5.66692	6.06373	5.97935
SPECC1	Q5M775	6.40931	6.21284	6.3622	6.85334	6.79007	5.95067	6.28341	6.42334	6.39068	6.25286	6.31481	6.09306	6.10559	5.73914	5.66382	5.69407
MORF4L1	Q9UBU8-2	6.53187	6.95777	6.92829	6.83378	5.96859	6.60016	6.71323	6.47967	7.05762	6.78537	6.67143	6.58451	4.73628	5.09862	4.98279	5.07003
MED13	Q9UHV7	6.37129	6.5595	6.30234	6.87541	6.08813	6.27161	6.20728	5.92838	6.17241	6.43751	6.20575	6.197	6.45912	5.80924	6.02979	6.08524
NCOA3	Q9Y6Q9	5.54605	6.21284	6.20164	5.69637	5.9055	6.07097	5.69711	6.16697	6.21735	6.32046	6.50105	6.46242	6.95446	6.34154	6.86587	6.83941
ME1	P48163	6.03601	6.23586	6.73406	6.09682	6.37502	7.11168	6.05904	6.52528	6.44643	6.76008	6.6643	6.62834	5.5602	5.6527	5.62813	5.48603
TBCA	O75347	6.41675	5.86916	7.03499	5.33756	6.79777	6.86966	6.12782	6.92924	6.51785	6.60569	6.62053	6.77459	5.89865	5.57242	5.26952	5.35779
SPART	Q8N0X7	6.16767	6.37883	6.57955	6.18375	5.89145	6.75514	6.22063	6.71041	6.37417	6.66344	6.48688	6.93804	5.4877	5.92081	5.25768	5.98387
PDE4DIP	Q5VU43-13	5.93761	5.80898	6.35924	5.87272	6.29815	6.46463	5.87779	6.64852	6.52193	6.50789	6.51489	6.66497	6.3254	6.17609	6.23573	5.78546
PRKCA	P17252	6.80323	6.94662	7.73894	6.79217	5.9065	6.82434	6.53293	6.58137	6.0256	6.59477	6.57341	6.19384	4.71864	5.35151	5.39985	5.0163
RABGGTA	Q92696	6.32377	6.23451	7.08943	6.03822	6.60402	6.79466	6.36031	6.75474	6.50599	6.45718	6.70819	6.46518	5.29646	5.65366	5.41943	5.29423
SMG6	Q86US8	6.13134	6.48817	6.81945	6.46472	6.11285	6.56375	6.01439	6.37857	6.63626	6.859	6.56595	6.77011	5.47005	5.69804	5.55532	5.47204
MAGEA10	P43363	5.46171	5.70835	5.83188	5.42632	5.70892	6.39407	5.70893	6.25373	5.83082	6.12454	6.32253	6.14238	7.20071	7.56627	7.14097	7.17787
CCZ1B	P86790	6.36445	6.43195	6.88661	6.3625	6.48574	6.61708	6.1874	6.57582	6.76493	6.7168	6.82374	6.72388	5.2028	5.36362	5.29542	5.19725
WDR26	Q9H7D7	6.02065	6.46931	6.36816	6.66959	6.05828	6.5762	5.86877	6.38803	5.98369	6.21594	5.99914	6.09704	6.47961	6.15931	6.09841	6.54788
SEH1L	Q96EE3-1	6.39518	6.30956	6.31043	6.59246	6.31678	6.24782	6.38011	5.80183	6.26924	6.17514	6.21801	6.11775	6.29951	5.96502	6.3078	6.29338
GHITM	Q9H3K2	5.88702	5.70164	5.51386	6.23171	6.44245	6.63089	7.02679	6.54644	5.91182	5.68653	5.64578	5.81817	6.5299	6.82087	6.62654	6.97957
CEP97	Q8IW35-2	6.39419	6.11021	7.05123	6.31407	6.34233	6.58364	6.11115	6.71912	6.47409	6.63477	6.93398	7.07414	5.3677	5.48579	5.06575	5.33785
TFAM	Q00059	6.04198	6.19816	5.55673	6.97356	5.96577	6.10312	6.89796	5.97603	6.15868	5.53752	5.73184	5.68694	6.64552	6.89133	6.6642	6.97068
TTC28	Q96AY4	6.50427	6.3989	6.77022	6.03577	6.18678	6.48829	6.18605	6.42096	6.70755	6.14426	6.49928	6.4408	5.98598	5.87267	5.67073	5.68749
ANTXR2	P58335	6.09501	6.05356	5.94503	6.59536	6.30313	6.2575	6.519	5.94056	6.42667	6.43365	6.60657	6.1697	6.17854	6.1507	6.03188	6.29314
L1RE1	Q9UN81	6.60698	6.54312	6.77892	6.28824	6.17922	6.41573	6.16918	6.11215	6.40847	6.55762	6.23048	6.39025	5.94521	5.79779	5.75649	5.82015
RAB1B	Q9H0U4	5.89829	6.42076	6.11359	6.3816	5.73347	6.09443	6.43838	5.75326	6.23491	6.18177	6.17661	6.32737	6.40424	6.43605	6.55101	6.85426
STEAP3	Q658P3-2	6.428	6.75126	6.29621	6.7722	6.03216	6.08088	6.22205	6.1589	6.93155	6.48543	6.35323	6.68335	5.54861	5.72604	5.86492	5.6652
ESD	P10768	5.84448	6.13371	6.5175	5.66544	6.67223	7.09562	6.18639	7.1523	6.54449	6.64979	6.51221	6.66026	5.66542	5.81405	5.47978	5.40632
ERCC4	Q92889	5.25758	5.72493	5.99656	5.79309	5.49348	6.22954	5.8621	5.81345	6.02392	6.41667	6.28239	6.21856	6.61692	7.33553	7.59512	7.34016
TGFBRAP1	Q8WUH2	6.30522	6.30043	6.59034	6.20219	6.26054	6.49569	5.99571	6.29665	6.20211	6.50063	6.65999	6.74478	5.77267	5.82731	6.13706	5.70868
ZNF384	Q8TF68	6.26453	6.32429	5.70307	7.02791	6.29785	6.02449	6.38468	6.14701	5.87984	6.36829	6.11487	6.0536	6.291	6.194	6.59597	6.32863
CRTAP	O75718	6.69481	6.90856	6.37474	6.68376	6.62815	6.39909	6.37233	6.05545	6.04138	6.08856	6.05263	6.10258	5.98547	6.03348	5.66092	5.91808
OSBPL1A	Q9BXW6	6.6852	6.20771	6.58631	6.13245	6.36156	6.42602	6.23991	6.45207	6.67179	6.65501	6.87186	6.41147	5.76974	5.73905	5.28421	5.50565
LAMA5	O15230	6.80794	7.04268	6.52602	6.79313	5.89351	6.2436	6.34302	5.85663	6.82013	7.21257	6.85008	6.42589	5.16906	5.78622	5.10862	5.12088
CAMK2G	Q13555-6	6.73487	6.54825	7.02225	6.36299	6.08279	6.40343	6.51029	6.54794	5.86675	6.57214	6.36401	6.36089	5.38146	6.01991	5.67322	5.54882
CAMK2D	Q13557-11	6.36404	6.09631	6.89552	5.81805	5.86882	6.48827	6.2747	6.33095	6.03143	6.67412	6.55628	6.39229	6.47221	5.92291	6.13918	5.67491
TAOK2	Q9UL54-2	6.29901	6.35498	6.22048	6.37635	6.23747	6.58577	5.91312	6.07991	6.2394	6.32784	6.39807	6.44862	5.90195	6.41992	6.08994	6.10718
UBN1	Q9NPG3	6.317	6.51721	5.97812	6.7264	6.04637	5.95381	6.40339	5.84999	6.14424	6.3357	6.0415	6.25061	6.25463	6.23217	6.59947	6.34938
YARS2	Q9Y2Z4	6.37187	6.60553	5.93352	6.71333	5.97615	6.08119	6.38838	6.28024	6.28296	6.04633	6.01454	6.14077	6.42157	6.10095	6.45757	6.18509
HIVEP2	P31629	6.2257	6.053	6.45696	6.08495	6.37932	6.43654	6.20139	6.31376	6.35438	6.27238	6.22059	6.25125	6.44569	6.1328	5.96346	6.20784
EEFSEC	P57772	5.8956	5.59787	6.48649	5.79167	6.08755	6.40928	6.06895	6.36489	6.22066	6.44587	6.40373	6.13337	6.30635	6.63933	6.69248	6.4559
MPHOSPH8	Q99549	5.90507	6.33507	5.9205	6.74903	6.08091	6.09555	6.17392	5.79371	5.93213	5.97926	6.10307	5.96101	6.45307	7.16926	6.54032	6.80811
GATB	O75879	6.34878	6.28206	5.89368	6.49699	6.04496	6.23315	6.51508	6.04694	6.26127	6.27654	6.09781	5.96541	6.76621	6.18563	6.34424	6.24126
APPL2	Q8NEU8	6.21252	6.44554	6.87195	6.12709	6.28944	6.59175	6.37997	6.45307	6.2933	6.78163	6.35645	6.6202	5.78428	5.6761	5.58028	5.53643
CAPN7	Q9Y6W3	6.35619	6.34623	6.9961	6.16831	6.272	6.56294	6.52584	6.35288	6.42081	6.76741	6.85139	6.72941	5.34174	5.64226	5.22984	5.43664
RPL35A	P18077	6.27666	6.26564	6.47963	6.29402	6.1299	6.22816	6.47404	6.17024	6.75804	6.47674	6.50565	6.47106	5.86687	5.79593	5.88619	5.92125
VLDLR	P98155	6.18554	6.45294	5.97227	6.12152	6.31215	6.47977	6.22674	6.23547	5.89846	6.15513	6.29206	6.42994	6.3352	6.22929	6.20183	6.47167
ADSS2	P30520	6.28865	6.24081	6.96381	5.8816	6.42427	7.04352	6.14476	6.783	6.46859	6.59687	6.78727	6.646	5.53077	5.56966	5.36253	5.26788
RPS23	P62266	6.44198	6.29265	6.35999	6.10448	6.44524	6.3133	6.35078	6.48189	6.73732	6.51938	6.3287	6.19197	5.82673	6.01811	5.8723	5.71517
EIF2B4	Q9UI10	6.34032	6.45361	6.77111	6.34413	6.32718	6.58765	6.14495	6.79233	6.2508	6.46807	6.67122	6.31037	5.4742	5.82499	5.80953	5.42955
GLMN	Q92990	6.15723	6.05125	6.77949	6.14461	6.19572	6.58351	6.18377	6.48808	6.46599	6.42837	6.86973	6.68206	5.63882	5.85793	5.61221	5.86124
LTV1	Q96GA3	6.22891	6.47549	6.94287	6.28285	6.38048	6.70652	6.42069	6.81171	6.56227	6.62103	6.79816	6.64304	4.82897	5.29886	5.36023	5.63792
C2CD5	Q86YS7-3	6.44555	6.25367	6.38108	6.01283	6.2343	6.60415	6.59281	6.20058	6.28591	6.55648	6.21326	6.08682	6.08614	6.15887	5.95775	5.9298
HOOK1	Q9UJC3	6.5599	6.29678	6.87961	6.08844	6.31601	6.85037	5.96023	6.34094	6.26486	6.61281	6.46274	7.01964	6.13659	5.63113	5.14601	5.43393
ASAP1	Q9ULH1-2	6.04562	5.97665	6.78954	6.01731	6.39333	6.61388	6.36231	6.3109	6.46964	6.66056	6.32885	6.36787	6.00014	6.02461	5.79288	5.84592
ASAP2	O43150	6.36836	6.3736	6.99257	5.74967	6.11703	6.7219	6.07603	6.32132	6.73155	6.65052	6.63256	6.47804	5.73153	5.93361	5.6863	5.43541
AP3S1	Q92572	6.19298	5.93684	6.77023	5.77234	6.36242	6.54853	6.03458	6.52595	6.19549	6.47079	6.43005	6.44914	5.76321	6.51772	5.93934	6.0904
LARP4	Q71RC2-4	6.17416	6.28948	6.62337	6.37361	6.24058	6.60856	6.31625	6.47413	6.46871	6.03086	6.43312	6.50896	6.0894	5.82365	5.71052	5.83463
PPIF	P30405	6.1998	5.98426	6.1061	7.20571	6.56039	5.86447	5.90875	6.20323	6.31477	5.99199	5.96009	6.05863	6.88709	6.01051	6.20469	6.63951
KIF18B	Q86Y91	6.90068	6.77895	6.16759	7.31524	6.24072	5.97497	6.60626	6.00482	6.20784	5.68915	5.91987	5.7477	5.96518	5.88386	6.15696	6.44021
TTC1	Q99614	6.79275	6.26134	6.55122	6.15003	6.74126	6.56868	5.95007	6.80822	6.57125	6.4742	6.70141	6.62389	6.02952	5.26801	5.15742	5.35074
TRAF2	Q12933-2	6.14912	6.25952	6.97047	6.1128	6.04697	6.62516	6.05474	6.64006	6.18894	6.40806	6.55943	6.97908	5.56743	5.72978	6.08969	5.61876
URGCP	Q8TCY9	6.44433	6.32557	6.81015	6.3921	6.28385	6.44162	6.32923	6.54616	6.69705	6.67317	6.80288	6.70671	5.33309	5.57388	5.11429	5.52593
ABCA3	Q99758	6.62385	6.89289	6.16751	6.75757	6.0054	6.03025	6.46053	6.2544	6.3144	6.48907	6.19624	6.04041	5.85728	6.16028	6.02277	5.72715
CTBP2	P56545	5.78044	5.34213	5.9503	6.17486	6.27517	5.47912	7.02925	5.90648	6.13826	5.57792	5.89237	5.51435	6.63349	7.07123	7.29414	7.94051
SETD1A	O15047	6.2136	6.43692	6.16083	6.65478	6.30248	6.58953	6.37237	5.70183	5.96319	6.08455	6.22271	5.92261	6.52304	6.56735	6.77106	6.51316
NGLY1	Q96IV0	6.0585	6.37047	6.91727	6.36682	6.19324	6.50608	6.12079	5.99676	6.09734	6.53764	6.52391	6.62077	5.56939	6.10381	6.06136	5.95585
APBB2	Q92870-4	5.98082	5.95947	6.02067	6.01733	6.06336	6.15892	6.24556	5.85855	6.06367	6.42853	6.29641	6.16101	6.57302	6.6373	6.76497	6.7704
REXO4	Q9GZR2	6.08709	6.35487	5.81105	6.93064	6.28735	5.96715	6.32003	5.88298	6.14403	5.76914	5.60843	5.89252	6.37658	6.83202	6.69841	7.0377
REXO2	Q9Y3B8	6.51028	6.06964	6.75265	6.01022	6.75474	6.60832	6.12633	6.64078	6.87004	6.19819	6.44801	6.10437	6.00566	5.64549	5.67654	5.57872
H6PD	O95479-2	6.34766	6.46301	5.77475	6.33878	6.2533	6.14154	6.17096	5.86899	6.02843	5.98669	5.95715	5.63402	6.89598	6.25765	6.97543	6.90567
PAXBP1	Q9Y5B6	6.38182	6.51503	6.04104	6.72519	6.24355	5.92879	6.13883	5.82701	5.92513	5.97364	6.10398	5.93369	6.75026	6.08416	6.83818	6.5897

TMPO	P42167	6.31215	6.60198	5.7986	6.75518	6.0747	6.15524	6.14554	6.85224	5.78892	5.79651	5.89138	5.77937	6.11864	6.55529	6.79372	6.58054
PLS1	Q14651	6.16183	6.00009	6.63694	5.75064	6.1127	6.49776	6.11761	6.49504	6.55456	6.35336	6.50078	6.41049	6.47816	5.93006	6.06614	5.93383
DDX55	Q8NHQ9	6.1102	6.14138	5.86041	6.36839	6.02311	5.94206	6.1435	5.99036	6.12332	5.95321	6.29916	6.0706	6.13407	7.18082	6.96261	6.6968
PPM1B	O75688	6.47666	6.26831	6.75518	6.07688	6.46196	6.75832	6.18582	6.72306	6.40472	6.59045	6.43014	6.4812	5.62778	5.69208	5.47057	5.59685
PIGT	Q969N2	6.56306	6.76622	6.11662	6.43289	6.51643	6.14026	6.76543	5.9249	5.83967	6.07179	6.00104	5.80005	6.53376	6.08797	6.55054	5.88939
RRP36	Q96EU6	6.57853	6.25923	6.01016	6.89671	5.95878	5.9001	6.40954	6.10899	6.26075	5.8547	6.3703	6.14179	6.25436	6.0228	6.36349	6.60978
POLR2E	P19388	5.84436	6.13851	6.05268	6.19265	6.68755	6.18355	6.79329	6.47183	7.17019	6.14175	6.18267	6.31473	5.8907	5.71464	5.98896	6.23193
TMEM33	P57088	6.24526	6.3579	6.00553	6.58526	6.18139	5.91122	6.35299	5.93689	6.21095	6.10279	5.98375	5.65721	6.76544	6.09615	6.90894	6.69833
DDAH1	O94760	6.22739	5.77242	6.28277	5.35416	6.45142	6.57052	5.8648	6.99723	6.33756	6.49766	6.31045	6.36219	6.30857	6.43883	6.0984	6.12564
SMG8	Q8ND04-2	6.14871	6.16059	7.18829	6.34078	6.16858	6.43671	6.30247	6.16876	6.41604	6.41093	6.66257	6.92245	5.77329	5.7365	5.39047	5.77286
EXOC1	Q9NV70	6.75385	6.51238	6.48164	5.79936	6.73103	6.61954	6.22085	6.46776	6.73708	6.62936	6.58272	6.34793	5.76741	5.48035	5.62871	5.24004
GBA	P04062	6.16234	6.60202	6.0353	6.4166	6.31874	6.32457	6.51563	6.31477	6.21854	6.29816	6.04519	6.1033	6.17757	6.22447	6.12088	6.12193
PHF8	Q9UPP1	5.997	6.37668	6.22726	6.83125	6.09778	6.2703	6.2837	6.15217	6.27066	6.26502	6.37091	6.14128	5.93343	6.00397	6.4337	6.34491
PHLDB1	Q86UU1-2	6.52438	5.59754	6.4881	5.94906	6.22433	6.72417	6.11963	6.41322	6.60922	6.40798	6.52616	6.83043	5.73114	6.01405	5.73856	6.10204
EIF3K	Q9UBQ5	6.2664	5.04402	6.37012	4.99813	6.28938	6.12211	6.19033	6.93186	6.08695	6.40658	6.30307	6.85774	5.23745	7.08628	6.77687	7.0327
RPL14	P50914	6.09668	6.1322	6.34374	6.71793	6.45866	6.30769	6.37075	6.13764	7.23991	5.9669	6.1197	6.35804	5.06246	6.2161	6.26162	6.20997
MYG1	Q9HB07	6.03802	6.17828	6.59641	5.56124	6.59237	6.88787	5.88502	7.01842	6.2817	6.38061	6.4679	6.6301	6.01304	6.06215	5.71542	5.69145
GNAQ	P50148	6.51572	6.6389	6.27211	6.60872	6.10784	6.20853	6.36153	5.87717	6.46901	6.14164	6.25094	6.04106	6.21127	5.84566	6.35284	6.09706
ARID4A	P29374	6.34406	6.43458	5.8338	6.78768	5.93735	5.85133	6.62533	6.0082	6.12007	6.27133	5.8848	6.02688	6.48209	6.15906	6.47914	6.75431
RALGAPA1	Q6GYQ0-6	6.37889	6.27842	6.88518	6.0565	6.17697	6.45035	6.28366	6.60784	6.41675	6.50926	6.63142	6.48542	5.87131	5.7874	5.65081	5.52982
PAK1	Q13153	6.38858	6.10942	6.89535	5.76662	6.53509	6.89372	6.22456	6.6107	6.49987	6.63743	6.4732	6.39964	5.89344	5.71301	5.53373	5.42565
HJURP	Q8NCD3	6.11232	6.33476	6.52809	6.46401	6.40369	6.29443	6.59207	6.23103	6.15509	6.45901	6.131	6.47157	5.88888	5.87505	5.84867	6.21033
NUP35	Q8NFH5	5.99229	5.75863	5.97876	6.35677	6.65694	5.81995	5.97989	6.36239	6.35634	5.95975	6.13233	6.28536	6.96792	6.17256	6.55945	6.66067
ETFDH	Q16134	6.51526	6.44182	5.90875	6.61934	6.48815	6.21106	6.4563	5.94507	6.10439	6.13997	5.78764	5.78114	6.85293	5.88046	6.61671	6.251
EXOSC3	Q9NQT5	6.24251	6.3577	5.96095	6.63027	6.24105	6.25966	6.35479	6.06592	6.14124	6.04527	6.2218	6.11665	6.60991	6.10208	6.46183	6.18836
BUB1	O43683	7.04342	6.87111	7.61794	7.00442	6.60948	6.62612	6.77779	6.57952	7.34179	7.0309	7.2376	7.02164	3.99459	4.23645	3.80907	4.19816
PPP1R9A	Q9ULJ8-3	6.4306	6.09772	6.14619	6.66865	6.12122	5.96569	6.50925	6.0204	6.1189	5.88226	6.10397	6.02104	6.72774	6.16815	6.62596	6.39225
MAPK1	P28482	6.28899	6.31724	6.9372	5.64256	6.15105	6.57282	6.16817	6.41483	6.24224	6.59438	6.90661	6.89664	6.03234	5.74868	5.49133	5.5949
EPB41	P11171	6.27457	6.53601	6.07258	6.19111	6.14274	6.11208	6.31116	5.88708	6.03924	6.26248	6.26705	6.31736	6.54327	6.49254	6.22224	6.32846
WDR12	Q9GZL7	6.11193	6.07738	5.99487	6.45408	6.13763	6.07213	6.40255	6.04251	6.09427	5.94036	6.15073	6.04922	6.49647	6.29563	6.9141	6.76614
RAE1	P78406	6.08837	6.50736	5.82576	6.56542	6.47505	6.24199	6.20168	6.39447	5.92361	6.21485	6.26509	6.40772	6.31659	6.14954	6.08407	6.33843
DECR2	Q9NUI1	6.21332	6.21442	5.81934	6.32671	6.39393	5.83809	6.33709	6.11498	5.89654	6.17437	6.39234	6.02222	6.61125	6.1533	6.75364	6.73847
MRM3	Q9HC36	5.96961	6.11114	5.72323	6.98214	6.21012	6.25947	6.48797	6.07161	6.17523	6.14632	6.09872	6.20561	6.59699	6.18235	6.33893	6.44056
PEBP1	P30086	6.00668	5.92162	6.73285	5.47038	6.71123	6.64242	6.07389	7.01757	5.5618	6.71823	6.93517	6.59941	6.01995	5.66857	5.4907	5.42955
LLGL2	Q6P1M3	6.36487	6.31189	6.39499	6.46322	6.04541	6.11606	6.29131	5.99883	6.37132	6.21247	6.14529	6.11992	6.34396	6.18407	6.51364	6.12276
ATP5PB	P24539	6.29895	5.72584	5.97478	5.94986	6.30543	5.72013	6.44388	6.02289	5.73844	6.21942	6.00695	6.15608	6.93064	6.55225	7.22975	6.72469
KRI1	Q8N9T8	6.03127	5.77878	6.16535	6.86673	6.2971	5.59945	6.26561	5.80516	6.48223	5.91442	5.92211	5.90042	6.64477	6.67535	6.57565	7.07559
TRIOBP	Q9H2D6	6.43371	6.31734	6.15199	5.97404	6.25281	6.2679	5.91532	6.41287	6.4126	6.51099	6.57426	6.14389	6.29304	6.31689	6.26178	5.76058
PPP4R1	Q8TF05	6.33875	6.32102	7.27023	5.72737	6.58763	6.57657	6.4706	6.87932	6.54967	6.27866	6.66119	6.5354	5.66466	5.69176	5.23971	5.20747
LOXL2	Q9Y4K0	6.3613	6.54799	6.71732	7.06878	6.70216	6.42345	6.98501	6.32481	6.55535	5.8999	6.12468	6.16508	5.75437	5.17608	5.61156	5.58216
PHLDB2	Q86SQ0	6.59284	6.56265	6.34382	6.51407	6.39779	6.32429	6.32624	6.38944	6.21051	6.24898	6.09921	6.20249	5.98436	5.91419	6.0298	5.86564
FAM111A	Q96PZ2	6.55831	6.90943	6.74189	6.72241	5.94864	6.27877	6.35467	6.14641	6.81489	6.54298	6.50346	6.52541	5.21286	5.4249	5.67284	6.64214
GALNT1	Q10472	6.29064	6.64607	6.2811	7.06457	6.05335	6.19591	6.4258	5.98486	6.24573	6.32917	6.17173	6.26572	6.04608	6.01634	5.9897	5.99323
PSMB7	Q99436	6.23921	6.23368	6.39476	5.9053	6.61569	6.4562	6.24468	6.78948	6.27913	6.44386	6.29804	6.19612	6.23301	5.89305	5.79763	5.98014
MPHOSPH10	O00566	6.12463	6.3973	5.74804	6.76936	5.99047	5.88716	6.33908	5.99665	6.01907	5.91014	5.63587	6.14235	6.65889	6.77966	6.94906	6.65226
P4HA1	P13674-2	6.47764	6.55988	5.81969	6.5978	6.9014	6.10269	6.31345	6.20121	5.83117	6.08146	5.81455	5.71034	6.69228	6.39157	6.2793	6.22558
WASH2P	Q6VEQ5	6.23246	6.08927	6.43038	6.14941	6.20557	6.43791	6.15942	6.16502	6.42002	6.95608	6.8602	6.80269	5.75937	5.9662	5.64481	5.72118
FYTTD1	Q96QD9	6.60234	6.23335	6.23827	7.10658	6.3877	5.63814	6.71884	6.02352	6.70952	5.99721	6.18143	5.96997	6.2627	5.79545	6.19173	5.94326
TUBGCP3	Q96CW5	6.25238	6.46833	6.79378	6.15777	6.39561	6.78188	6.12393	6.62248	6.15684	6.49654	6.43501	6.449	5.87135	5.81752	5.75316	5.42442
CPOX	P36551	6.14822	6.24805	5.96644	6.50174	6.63199	6.13627	6.47241	5.92218	6.10192	5.85146	6.01301	5.90242	6.97674	6.1581	6.50136	6.46768
MRPS30	Q9NP92	6.24981	6.60063	5.90779	6.98651	6.12256	5.99661	6.30466	6.1321	6.09566	6.15309	5.78866	6.08548	6.45385	6.13545	6.70303	6.2841
CSTF2T	Q9H0L4	6.55226	5.41693	5.70155	5.49218	6.38822	5.25422	5.63711	6.90733	5.47467	6.39358	6.16969	7.00241	7.03851	6.86574	6.94651	6.7621
NRBP1	Q9UHY1	6.53154	6.14375	6.78185	6.07278	6.41291	6.72031	6.23947	6.68087	6.33139	6.7185	6.67406	6.64732	5.72461	5.49171	5.46976	5.35915
ZRANB2	O95218	5.49609	6.23755	5.71599	5.58672	6.16173	6.32628	5.55229	5.99454	5.81576	6.38257	6.07252	6.23454	7.10239	7.37622	6.90442	7.04039
MEAK7	Q6P9B6	6.1962	6.18387	5.89157	6.13706	6.18477	6.5029	6.08865	6.13487	6.02851	5.75925	6.07314	6.0816	6.33927	6.77597	6.68247	6.93991
FAHD1	Q6P587	6.1725	5.77253	5.9322	6.18179	6.44652	5.95043	5.93182	6.09608	6.15333	6.1519	6.24532	6.40991	6.85525	6.33382	6.70456	6.66204
ORC3	Q9UBD5-2	5.97529	6.29229	6.15866	6.17692	5.9509	6.05724	6.22496	5.96061	6.11706	6.14863	6.24666	6.14901	6.33968	6.64615	6.91259	6.64335
LIN54	Q6MZP7	5.98421	6.15715	6.16762	6.58517	6.32761	6.11688	6.46666	5.98751	5.99284	6.33188	6.1748	6.17532	6.76119	6.13915	6.20148	6.41153
FOXK1	P85037	5.75124	5.95508	5.94807	5.53726	6.25046	6.28831	6.01069	6.39302	5.99214	6.08594	6.16309	5.9034	7.1784	6.82358	6.95813	6.76119
ZNFX1	Q9P2E3	6.43293	6.18441	6.98394	6.25341	6.2483	6.87058	6.27155	6.25969	6.47835	6.64804	6.62053	6.43032	5.55373	5.66824	5.40079	5.44558
TPP1	O14773	6.15835	6.36784	6.01848	6.20176	6.66981	6.42156	6.24866	6.31908	5.97188	6.43044	6.06176	6.18369	6.58503	6.05444	5.87576	6.43145
MFF	Q9GZY8	6.65439	5.55656	6.88073	5.93642	6.93549	5.49389	6.06393	6.02805	5.87534	6.73367	6.22565	6.82361	5.86853	6.34694	6.12101	6.45579
RAB31	Q13636	6.07007	6.06482	6.11358	6.09085	6.14813	6.11347	6.37427	5.90577	6.22592	6.26752	6.02317	5.9263	6.59933	6.47243	6.92434	6.68002
GALC	P54803	6.41458	6.59476	5.65203	6.38325	6.28765	6.16596	6.25851	5.88329	6.08836	6.07929	5.90405	6.13497	6.43771	6.60714	6.5285	6.57994
SLC30A1	Q9Y6M5	6.21756	6.47654	5.91421	6.77827	6.03868	5.62409	6.03957	5.65799	5.98637	6.21271	5.87823	6.04029	6.84468	6.6151	6.96144	6.71426
BLMH	Q13867	6.16528	6.30651	6.87403	6.05902	6.34297	6.98412	6.07375	6.34628	6.35142	6.65402	6.71496	6.77924	5.91254	5.40532	5.41136	5.61919
ARHGAP18	Q8N392	6.40728	6.40169	7.03708	5.98661	6.06769	6.48749	6.12475	6.6895	6.17671	6.43747	6.33385	6.47882	5.69697	5.87092	5.74001	6.06316
SH3D19	Q5HYK7	5.58349	6.15889	6.15691	5.64183	6.1598	6.42342	6.3222	6.22475	6.67846	6.68882	6.56108	6.60665	6.57454	6.35985	5.8141	6.04523
CSNK2B	P67870	5.37001	5.60456	5.69782	5.43637	6.76497	6.07627	6.03422	7.36738	6.07381	6.72838	6.36035	6.40849	6.15376	6.45793	6.62455	6.84112
PLAUR	Q03405	6.05629	6.17985	6.02155	6.42258	6.46838	6.21701	6.36632	6.1582	5.54592	5.58918	6.07341	5.45125	6.96034	6.47888	7.14226	6.86856
UBE2Z	Q9H832	6.50155	5.87557	6.83302	5.99918	6.31423	6.65904	6.01206	6.45979	6.06873	6.21712	6.68382	6.52355	6.08625	6.22046	5.76236	5.78325
TIGAR	Q9NQ88	6.13843	6.13989	6.81815	6.2362	6.17265	6.82501	6.05066	6.38991	6.57428	6.68793	6.77586	6.53406	5.72853	5.64693	5.67559	5.60593
BUB3	O43684	5.76873	6.19993	5.89452	5.87236	6.23455	6.17688	6.15522	6.33819	6.46118	6.59896	6.35933	5.77399	6.6704	6.17802	6.58188	6.73585
COG7	P83436	6.3692	5.94716	6.31774	5.96183	6.36717	6.34365	6.41962	6.46968	6.2994	6.48247	6.53589	6.28426	6.34911	6.10633	6.13289	5.6136
MEIOC	A2RUB1	6.71499	6.51108	7.08885	6.47041	6.25218	6.72906	6.16743	6.61274	6.78575	6.72271	6.88122	7.10323	5.24012	4.91573	4.72397	5.08053
SRSF10	O75494	6.19904	6.51691	5.82031	6.61102	6.57315	6.228	6.28141	6.08845	5.80298	6.2368	6.46699	6.39926	6.10563	6.15754	6.14512	6.36739
PPP6R1	Q9UPN7	6.5161	6.16338	6.8751	6.00673	6.45341	6.72409	5.83829	6.86787	6.43257	6.14252	6.34909	6.60569	6.41244	5.64739	5.41871	5.54662
GTPBP10	A4D1E9	6.51394	6.42142	6.07165	6.74865	6.25565	6.02331	6.58567	6.15493	6.20112	6.19451	6.03535	6.04919	6.43982	5.92516	6.38569	5.99395
RELA	Q04206	6.58513	6.43235	6.77666	5.89602	6.347	6.91545	6.07242	6.31879	6.32734	6.37852	6.60215	6.82662	5.83324	5.72504	5.69419	5.26908
EIF2AK2	P19525	6.41917	6.48601	6.44188	6.155	6.3897	6.48795	6.28599	6.60646	6.5306	6.78228	6.3676	6.36633	5.72241	5.86228	5.53059	5.56575
USP11	P51784	5.87463	6.32968	6.4786	5.96772	6.11133	6.565	5.90137	6.49886	6.07553	6.67149	6.71253	6.88231	5.98289	6.13022	5.69606	6.12179
ELP2	Q6IA86-6	6.35146	6.62107	7.01772	6.03114	6.30297	6.98859	6.22953	6.62858	6.51966	6.51421	6.5957	6.68155	5.39458	5.43328	5.35827	5.3317
CYB5R1	Q9UHQ9	6.3761	6.57341	5.80304	6.47491	6.04607	5.96834	6.27924	6.11491	6.09119	6.13709	5.74006	5.90987	6.77067	6.54635	6.61777	6.55096
CTSB	P07858	6.53738	6.49807	6.01759	6.24094	6.77966	6.16085	6.42525	6.62399	6.19196	6.3542	6.38169	6.02738	5.85247	5.97714	5.92468	6.00674
RINT1	Q6NUQ1	6.5501	6.44929	6.25137	6.28619	6.44105	6.15294	6.53587	6.03517	5.97132	6.25643	5.91184	5.84197	6.33792	6.06639	6.57342	6.33873
PIGG	Q5H8A4	6.32052	6.40793	5.98669	6.53691	6.37064	6.09731	6.40491	5.97362	6.06762	6.11508	5.94891	5.67939	6.66872	6.40818	6.54519	6.46837
RESF1	Q9HCM1	6.90967	6.7709	6.5515	7.50118	6.16715	6.03879	6.48209	6.35061	6.46182	6.60402	6.89726	6.55609	5.05386	5.03329	5.09383	5.52795
BEND3	Q5T5X7	6.50414	6.53702	5.88721	7.0206	6.06351	6.01739	6.37229	5.75171	6.208	5.61293	5.97139	5.94053	6.78408	6.26733	6.45452	6.60736
ODF2	Q5BJF6	6.77391	7.00284	7.11644	7.48476	6.20095	6.38687	6.58828	6.80604	6.95232	6.88894	6.60889	7.09037	4.7003	4.30978	4.5244	4.56492
RCOR1	Q9UKL0	6.28285	6.59116	6.25386	6.61796	6.10801	6.33489	6.13883	6.11551	6.11041	6.35752	6.12348	6.03961	6.14816	6.19511	6.3459	6.23674
S100A10	P60903	5.62013	4.93414	5.60865	4.76233	6.11551	5.59898	5.85632	6.36629	5.86876	6.53326	6.2205	6.58026	5.88418	7.92214	8.28143	7.84713
RAB21	Q9UL25	6.23839	6.36336	6.12099	6.51085	6.32062	6.24009	6.34302	6.09247	6.07648	6.38606	5.94719	5.72975	6.40067	6.13547	6.67017	6.42441
SEC13	P55735-3	6.17429	6.26517	6.30902	6.29294	6.17522	6.61578	6.05448	6.51413	6.13839	6.082	6.04968	6.14517	6.33622	6.53616	6.1098	6.20156
PNPO	Q9NVS9	5.88172	5.12392	6.34083	5.19488	6.93875	6.10102	6.10312	6.9111	6.20944	7.01398	7.31727	6.81245	6.0394	5.78064	6.19815	6.03331
PPP4R2	Q9NY27	5.79884	5.94407	5.88869	5.76949	6.40498	6.20658	6.16759	5.81768	5.97172	5.75626	6.06412	6.20257	7.34399	6.70261	6.86513	7.09568
CSK	P41240	6.07531	6.24551	6.84334	5.75671	6.48609	6.7325	6.19518	6.63633	6.46063	6.4064	6.48549	6.34919	5.95473	5.96065	5.8086	5.60333
RIOK2	Q9BVS4	6.39143	6.28187	7.22879	6.25101	6.40697	6.37305	6.28093	6.57312	6.46593	6.65831	6.6999	6.90652	5.39513	5.59606	5.22833	5.26265
ASXL1	Q8IXJ9	6.70897	6.3661	6.62977	6.6303	6.17674	6.1663	6.47928	6.61182	6.57016	6.28744	6.35732	6.67085	5.65439	5.68449	5.39079	5.61526
AMPD2	Q01433	6.18002	6.34316	6.68571	5.99031	6.48294	6.81463	6.39196	6.73168	6.59032	6.5164	6.14862	6.24271	5.72957	5.97306	5.46358	5.71535
NUDT5	Q9UKK9	6.14738	5.6672	6.61247	5.51765	6.59573	7.02791	6.0995	6.70898	6.3283	6.52118	6.62803	6.6173	6.15305	6.10106	5.55516	5.7191
HDAC7	Q8WUI4-5	6.05962	6.21937	6.81669	5.76185	6.12004	6.74047	6.11486	6.7103	6.47491	6.63925	6.64559	6.70883	5.62235	5.9321	5.69301	5.74077
COG3	Q96JB2	6.2431	6.22126	6.23878	6.70175	6.33141	6.23959	6.21492	6.08975	6.16302	6.46841	6.05705	6.42911	6.22596	6.03602	6.25781	6.08208
A0A494C100	A0A494C100	5.96147	6.42146	6.33093	6.2911	6.27348	6.53872	6.4587	6.34564	6.27355	6.30696	6.72335	6.56451	5.82377	5.88306	5.87616	5.92714
MAGT1	Q9H0U3	6.39924	6.15184	5.72244	6.67358	6.39078	5.68304	6.68922	5.88549	6.22931	5.60874	5.58382	5.74774	6.16894	7.04832	6.90835	7.10914
MTG1	Q9BT17	6.77523	6.76356	6.19361	7.05361	6.37523	6.3905	6.46528	5.93237	6.54081	6.36917	6.1873	6.41873	5.76904	5.524	5.55011	5.69146

TPD52L2	O43399-7	6.41904	5.55258	6.72043	5.62045	6.31319	6.24706	5.76164	6.48253	6.14282	6.46016	7.02879	7.04197	6.68451	6.1876	5.65089	5.68634
NDUFA13	Q9P0J0-2	6.00728	5.28134	5.34757	5.92836	6.25274	5.78035	7.10317	5.98878	6.18447	5.15606	5.55152	5.99825	6.22335	7.5341	7.38516	8.2775
NUDCD3	Q8IVD9	5.9593	5.89319	6.73634	5.71648	6.19281	6.57353	6.09948	6.40503	6.37812	6.1304	6.81175	6.43165	6.18385	5.39435	6.08109	6.01263
NDUFAF7	Q7L592	6.37391	6.2781	5.88744	6.33001	6.37994	6.05291	6.44568	6.14182	6.17703	6.0277	6.1664	5.88586	6.76281	6.26886	6.49394	6.32761
IFI16	Q16666	5.63315	6.0188	5.76517	5.86433	5.93195	5.99517	5.93512	5.94766	6.11025	6.51941	6.178	6.09996	6.93275	6.97078	7.29011	6.80739
ATP6V1H	Q9UI12	6.44451	6.86443	6.45547	6.3202	6.40215	6.53525	6.40826	6.69656	6.57662	6.63372	6.55453	6.45793	5.32862	5.54704	5.38643	5.38827
MRPL1	Q9BYD6	6.39027	6.53157	6.01751	6.8784	6.40238	5.80608	6.32072	5.86423	6.16839	6.22783	5.74697	5.88149	6.65162	5.96752	6.67315	6.47187
GFM2	Q969S9	6.39855	5.93537	5.99667	6.69251	6.28566	6.16438	6.41113	5.77082	6.18828	5.87929	6.16828	6.14869	6.62868	6.49756	6.27906	6.55507
SCYL2	Q6P3W7	6.24647	6.49306	6.56979	6.35675	6.08869	6.48741	6.04915	6.51192	6.36128	6.46595	6.16995	6.5221	6.00063	5.79205	5.85431	6.03048
SENP3	Q9H4L4	6.25992	6.18732	5.8456	6.72124	6.24398	5.92894	6.47639	5.9509	6.13075	6.00943	5.93333	5.92163	6.55688	6.36125	6.76835	6.70408
BLVRB	P30043	5.85383	5.44109	6.38008	5.40257	6.50666	6.72039	6.28726	6.97753	6.31807	6.87209	6.50693	6.95799	5.83897	6.2151	5.76565	5.95577
NLE1	Q9NVX2	6.36669	6.35651	6.27728	6.39243	5.79453	6.02723	6.11588	6.20855	6.26315	6.42533	6.39088	6.39134	6.12575	6.0498	6.55967	6.25497
TRAPPC11	Q7Z392	6.06996	6.20599	6.60203	5.85059	6.33795	6.58683	6.04251	6.6466	6.60648	6.6146	6.26241	6.33639	6.00614	6.10379	5.93848	5.78924
RPAP1	Q9BWH6	5.91254	6.18618	7.02001	5.85553	6.01961	6.37158	6.29658	6.58792	6.43837	6.47352	6.6653	6.68909	5.84191	5.89845	5.64768	6.09573
MED17	Q9NVC6	6.16057	6.15543	6.17016	6.36604	6.42587	6.11607	6.04109	5.86824	6.32034	6.20722	6.33171	6.06042	6.5147	6.09975	6.7126	6.44977
EXOC8	Q8IYI6	6.32286	6.55183	6.72928	6.14332	6.18607	6.52466	6.2443	6.29415	6.40639	6.39373	6.71386	6.40189	6.95413	5.85931	5.72065	5.55358
DIP2A	Q14689	6.7864	6.58117	6.36643	6.41199	6.38637	6.38187	6.41535	6.10761	6.23136	6.18671	6.08307	6.09043	6.0203	6.10138	5.9181	5.93146
SIN3B	O75182	6.01839	6.76424	6.20311	6.98671	6.24074	6.47239	5.97577	6.33454	6.04407	6.1951	6.74845	6.22414	5.73276	5.91684	6.36927	5.7735
MTCL1	Q9Y4B5	6.38406	6.15166	6.49034	6.30363	5.96489	6.54363	6.14433	6.70465	6.58804	6.68489	6.55857	6.924	5.63088	5.69553	5.60032	5.63059
AP3M1	Q9Y2T2	6.47119	6.24089	6.92518	6.19215	6.24332	6.42297	6.32631	6.54144	6.47571	6.36379	6.29896	6.00245	5.86648	5.95262	6.01314	5.6634
ALDH16A1	Q8IZ83	6.4331	6.54561	6.67042	5.95499	6.50994	7.12119	5.84387	6.54999	6.52988	6.34894	6.87576	6.90347	5.57147	5.62186	5.12368	5.39582
MARCKS	P29966	6.02299	5.63901	5.8633	5.26109	5.7968	5.07858	5.62802	6.27108	5.46254	6.19049	5.92892	6.54843	8.23039	7.31628	7.51673	7.24537
TOM1L2	Q6ZVM7	6.31294	6.0795	6.74801	5.9135	6.51311	6.49091	6.08834	6.6446	6.52644	6.62807	6.67809	6.72807	6.05479	5.72522	5.37011	5.4983
NECAP2	Q9NVZ3	6.2788	5.94995	6.85593	5.93332	6.06544	6.50415	6.28326	6.7042	6.44944	6.44582	6.85574	6.74614	5.92844	5.77869	5.63993	5.58076
YWHAB	P31946	6.02814	5.89519	6.67447	5.71443	6.29246	6.66518	6.19343	6.39973	6.38529	6.26805	6.34657	6.29369	6.67318	6.07005	6.16584	5.93429
GOLPH3	Q9H4A6	6.32902	6.26369	6.05099	6.27473	6.32514	6.2701	6.29268	6.14765	6.60452	6.26497	6.47408	6.16637	6.27268	6.27141	6.05805	5.93393
TOMM34	Q15785	6.33447	5.95334	6.74978	5.88746	6.45401	6.57527	6.13889	6.41126	6.41536	6.33083	6.65193	6.50572	6.04109	5.96342	5.84164	5.75304
CHMP4B	Q9H444	6.12339	6.07818	6.75748	5.70998	6.39831	6.20807	6.05572	6.97783	6.70263	6.4705	6.48906	6.32913	5.95198	5.95869	5.81492	5.97411
CEP131	Q9UPN4	6.16246	6.43941	6.77841	6.54731	6.19946	6.32267	6.55423	6.43297	6.49431	6.31087	6.78646	6.93165	5.40864	5.77112	5.39246	5.46759
SUN2	Q9UH99	6.41775	6.49694	6.0433	6.78242	6.19448	6.2759	6.35321	5.94508	6.30372	5.91865	5.91604	6.11862	6.3527	6.14667	6.50936	6.22516
PDLIM1	O00151	6.50503	6.02985	6.43227	5.2926	6.76638	6.35205	5.94764	6.05959	6.38055	6.20246	6.70975	6.61376	6.8627	6.02328	5.9147	5.9074
ARHGAP17	Q68EM7	6.31881	6.33917	7.01497	6.21159	6.3887	6.87245	6.12299	6.40758	6.21874	6.68943	6.62665	6.6455	5.84478	5.57707	5.39605	5.32552
PKP2	Q99959	6.40819	6.55664	6.44521	6.56746	6.06522	6.04774	6.12549	6.211	6.38888	6.222	6.28117	6.36655	6.04495	5.92838	6.10986	6.23126
MAN2B2	Q9Y2E5	6.43549	6.35291	6.04117	6.44711	6.27058	6.199	6.30346	6.37549	6.05517	6.50513	6.25704	6.32795	5.78227	6.13569	6.40553	6.10599
GATAD2B	Q8WXI9	6.46311	6.52463	6.00889	6.40802	6.64483	6.28624	6.19266	6.34402	6.20003	6.30011	6.15543	6.27602	6.48251	5.9216	5.88367	5.90822
PRKAG1	P54619	5.66979	5.56326	6.10995	5.32848	5.99869	6.1381	5.81755	5.90905	6.09291	6.16648	6.41832	6.33865	7.10993	6.71091	7.47876	7.14917
CBS	P35520-2	6.09154	6.09916	7.07543	5.27398	6.53822	6.54007	6.40128	6.45771	6.58054	6.38942	6.72563	6.41226	6.06963	5.95981	5.59023	5.79508
SPAG1	Q07617	6.26995	6.35305	6.65893	6.35682	6.33573	6.6022	6.07146	6.35242	6.18523	6.32842	6.17668	6.62971	6.22537	5.90411	5.80877	5.74115
SMARCE1	Q969G3	6.51118	6.79577	5.94741	6.38272	6.60657	6.04606	5.90466	6.00183	6.06381	6.04731	6.30672	5.75592	6.96435	5.93687	6.10148	6.62734
DDRGK1	Q96HY6	6.31625	6.8248	5.77998	6.75249	6.00492	6.27592	6.1741	6.11385	5.93331	6.05638	5.88869	6.22089	6.20056	6.46744	6.22188	6.76853
TNRC18	O15417	6.49679	7.16926	6.50548	6.79239	6.1606	6.08665	6.35581	6.06009	6.33056	7.01351	6.58216	6.4022	5.16261	5.84098	5.39013	5.64778
TRUB2	O95900	6.28196	6.70241	5.99181	6.78643	6.68151	6.30677	6.61421	6.62194	6.1362	6.27442	5.95695	6.05286	6.01523	5.59845	6.05472	5.92412
NAA35	Q5VZE5	6.02089	6.39224	6.41089	5.93842	6.1651	6.35914	6.14907	6.26687	6.12805	6.64264	6.67283	7.01466	5.86414	6.00286	5.83674	6.13545
TOR1AIP2	Q8NFQ8	6.12294	6.17511	5.93395	6.24376	6.23881	6.23388	6.43944	6.36378	5.98165	5.79569	6.38466	6.06823	6.5777	6.33841	6.67073	6.33125
VAT1	Q99536	6.38907	5.97788	6.12667	5.69531	6.36377	6.36085	6.04299	6.16096	6.40437	6.51629	6.34928	6.21164	6.54578	6.53584	6.19285	6.12647
MICAL1	Q8TDZ2-4	6.17675	6.30389	6.97834	6.1903	6.21795	6.82166	6.45534	6.47905	6.3543	6.44967	6.67085	6.3753	5.66479	5.6953	5.60559	5.56092
ATP6V0D1	P61421	6.06869	5.69119	5.75668	5.63772	6.05091	5.98222	6.58093	6.61671	5.51553	6.0044	5.9312	6.31471	5.71601	7.90018	6.44475	7.78817
GSR	P00390	5.85049	6.00711	8.0887	5.51853	6.27787	6.074	9.2076	6.13638	6.02806	6.0104	6.70707	5.46008	4.99547	5.23797	7.31684	5.08345
GUSB	P08236-2	6.65995	6.62155	5.639	6.41629	6.33166	6.03789	6.39941	6.03389	6.21853	6.3394	6.0586	6.2135	6.15276	6.15527	6.36774	6.35457
MRPL24	Q96A35	5.94819	6.12166	5.5797	6.6191	6.68293	5.92073	6.69549	6.46826	6.16909	5.95367	6.16029	5.96912	6.12933	6.24852	6.72724	6.6067
SP140L	Q9H930	6.29776	6.63304	6.33481	6.63835	5.95132	6.15023	6.52916	6.33082	6.22848	6.21327	6.47553	6.04904	5.6171	6.21777	6.04356	6.28977
SP100	P23497-4	6.4146	6.51716	5.69022	6.48746	6.1351	6.18167	5.97774	5.75645	6.19171	6.48059	6.07304	6.08584	6.61082	6.53054	6.3054	6.56166
LRRC47	Q8N1G4	6.15632	6.57402	6.90765	6.00667	6.23469	6.49238	6.02841	6.2834	6.21342	6.26898	7.0848	6.4343	5.93337	5.89532	5.71281	5.77347
MRPL46	Q9H2W6	6.34816	6.41874	5.57893	6.25127	6.54985	5.78735	6.06483	6.65567	6.0306	6.42787	5.93258	6.28113	6.796	6.15891	6.23881	6.47929
YJU2	Q9BW85	5.71318	5.73993	6.07938	5.45017	5.88569	5.74261	5.74858	5.54437	6.15804	6.38447	6.54549	6.32073	7.01416	7.18974	7.37262	7.11084
NHP2	Q9NX24	6.54529	6.53814	6.27428	6.87953	6.3355	5.68743	6.60236	5.62085	5.781	6.01454	6.1108	5.74133	6.55478	6.42024	6.39331	6.5006
VPS4A	Q9UN37	6.60322	6.62999	6.96002	6.4758	6.44273	6.97126	6.00753	6.83725	6.54729	6.58586	6.75421	6.3524	5.40943	5.36099	5.08963	4.97239
RPS14	P62263	6.44565	6.59403	6.53467	6.41853	6.00523	6.31465	6.38142	6.17667	6.66061	6.41973	6.59748	6.44901	5.79098	5.72937	5.74896	5.73303
BCORL1	Q5H9F3-3	6.5323	6.62957	6.2912	7.01233	6.25655	6.25033	6.60496	5.84513	6.44385	6.47603	6.40735	6.19395	5.59108	5.72992	5.73429	6.00117
MAN1B1	Q9UKM7	6.52502	6.50953	6.16568	6.7353	6.45367	6.08952	6.29228	6.10958	5.92914	6.25278	6.17997	5.92736	6.37141	6.28499	6.33657	5.83981
TGOLN2	O43493-7	6.28969	6.2312	6.029	6.31384	6.32089	5.96872	6.11765	5.89483	6.41073	6.35687	6.49923	6.46474	6.55915	6.13125	6.17634	6.23588
SPATS2	Q86XZ4	5.77283	6.06397	6.25962	5.83021	5.97018	6.60443	6.27782	6.43717	6.59848	6.859	6.81614	6.63518	5.77062	6.16559	5.91453	6.02422
ZFHX3	Q15911	6.35077	6.63706	6.11131	6.94206	6.36081	6.11066	6.41408	5.94883	6.00759	6.38731	5.88195	5.86969	6.33712	6.32463	6.35234	5.96379
PNO1	Q9NRX1	6.00093	6.22918	5.91266	6.32017	6.44349	6.34004	6.18639	6.09355	6.1451	6.16132	6.02142	5.93403	6.78029	6.26017	6.5486	6.62265
BASP1	P80723	6.45095	4.82135	5.90312	5.42334	6.63339	5.0825	5.50636	6.29237	5.70369	6.67924	6.62361	6.93631	7.07918	6.87023	7.02933	6.96503
PPP2R2A	P63151-2	5.72033	5.89028	6.66592	5.74831	5.90691	6.65772	6.24668	6.53162	6.20943	6.36403	6.58943	6.49049	6.19395	6.21935	6.45284	6.31272
TOP3A	Q13472	6.24493	6.41193	6.22352	6.8639	6.08027	5.82587	6.51775	5.72934	6.7696	6.22064	6.27362	6.15411	6.1888	5.7621	6.47444	6.25918
VPS52	Q8N1B4	6.17132	6.38406	6.53039	6.13164	6.12477	6.27492	6.40415	6.52376	6.3979	6.53078	6.37708	6.67097	5.74978	6.27252	5.7435	5.71244
CCDC93	Q567U6	5.88959	6.24786	6.54771	5.71495	6.20605	6.26146	6.09228	6.30626	6.51126	6.16297	6.39245	6.05752	6.59672	6.54688	6.45594	6.0101
TRIM27	P14373	6.35869	6.20719	5.72906	6.30806	6.74794	6.36133	6.7116	6.41493	6.36195	5.97831	6.03513	6.15495	6.38693	5.80981	6.33046	6.10367
RAB11FIP1	Q6WKZ4	6.43755	7.12702	6.68375	6.65061	5.87955	6.81177	6.65702	6.15434	5.39088	6.03422	6.15331	6.13551	5.50389	5.79124	6.37791	6.21143
CEP290	O15078	6.24613	6.37496	6.89595	6.14294	6.17167	6.48823	6.11524	6.62132	6.55145	6.21703	6.53831	6.95719	5.5692	5.68106	5.66812	5.7612
ZC3H4	Q9UPT8	6.24028	6.23684	5.787	6.62348	6.09787	5.86049	6.16755	5.84918	6.0505	6.00644	6.17172	5.8362	6.72936	6.68724	6.80697	6.84886
MARK2	Q7KZI7-16	5.98448	6.1501	6.28547	6.31029	6.0057	6.06212	6.22861	6.1474	6.36073	6.18943	6.32828	6.40731	6.43812	5.96995	6.57121	6.5608
HEXIM1	O94992	6.2461	6.60353	6.07367	6.11922	6.15215	6.80064	5.80017	6.23678	6.03885	6.37495	6.12712	6.05951	6.4168	6.11763	6.4236	6.40929
STOML2	Q9UJZ1	6.0804	5.43001	5.86871	5.36413	6.16602	5.50769	6.11909	6.46523	5.68286	6.37668	5.7041	6.64945	6.95041	6.94722	7.49384	7.19417
ZEB1	P37275-2	6.18722	6.15965	5.99682	6.53581	6.48639	6.30694	6.4384	6.10141	6.10778	5.82604	5.8722	6.16025	6.90732	6.11177	6.26987	6.53215
CANT1	Q8WVQ1	6.52092	6.42163	6.11518	6.99414	6.29784	6.0671	6.33741	6.26579	6.25915	6.65287	6.32357	6.682	5.91619	5.51086	5.80175	5.83361
MRPS23	Q9Y3D9	6.16979	6.22544	5.80912	6.97554	6.46491	5.89883	6.63313	6.20496	6.28145	6.10181	6.0459	6.16664	6.59601	5.80412	6.33445	6.28791
SNAPC4	Q5SXM2	6.03179	5.91719	6.32267	6.17598	5.96218	6.03389	6.17446	6.00098	6.43641	5.87384	6.52181	6.20436	6.58742	6.19092	6.84437	6.72172
USP16	Q9Y5T5	6.04246	5.82469	6.5034	5.88627	6.11054	6.47649	5.95994	6.11811	6.40021	6.89501	6.76241	6.59042	5.90034	6.60018	5.77026	6.15926
MBD3	O95983	6.15041	6.4638	6.47053	6.55207	5.9866	5.81528	6.23109	6.07474	6.36874	6.27659	6.28483	6.75294	6.52844	5.67477	6.1305	6.23866
MAIP1	Q8WWC4	6.37804	6.69098	5.87321	6.76598	6.44394	6.24816	6.14935	6.24092	6.06048	5.9324	6.59118	5.7625	6.47756	6.14868	6.52573	6.34088
CAP2	P40123	6.13625	6.10338	6.88769	5.96251	6.27542	6.48717	6.25426	6.68864	6.41598	6.26928	6.61584	6.46597	5.79313	5.94226	5.8784	5.82382
TSPYL1	Q9H0U9	6.24982	6.02839	6.29889	6.52706	6.33492	6.06167	6.40861	6.06255	6.41393	6.35049	6.50668	6.00573	6.38639	6.01381	6.25788	6.09316
PRDX3	P30048	6.17603	4.96299	5.30141	4.97728	5.93424	5.15472	5.77566	6.23683	5.14187	6.26907	5.65072	6.6175	7.04147	8.16412	8.26162	8.33448
CLMN	Q96JQ2	6.17225	6.46692	6.17808	6.56571	6.00459	6.16994	6.12171	5.91084	5.94917	6.22726	6.104	6.36263	6.48949	6.0106	6.65833	6.60847
NUMBL	Q9Y6R0	6.44723	6.41309	6.55894	6.15436	6.23503	6.65157	6.15876	6.57181	6.18394	6.4768	6.5081	6.21695	5.88411	6.03553	5.82041	5.87936
NPC1	O15118	6.41914	6.66423	6.35123	7.00623	6.14808	5.96005	6.3083	6.05216	6.20102	6.28684	6.36168	6.34285	5.85088	5.85221	6.00688	6.18822
ABCB7	O75027-2	6.43387	6.69269	6.17511	6.83922	6.3717	5.88752	6.2151	5.99558	6.15476	6.08219	5.838	5.78867	6.60273	6.07338	6.60825	6.24125
KDM4A	O75164	6.04119	6.45631	6.5685	6.33592	5.49762	5.98407	6.20976	6.04645	6.47761	6.36764	6.62179	7.0627	5.9533	6.28506	6.21855	5.87351
AHCYL2	Q96HN2	5.97652	5.97681	6.58591	5.8979	6.42656	6.84762	6.45473	6.85714	5.96991	6.06871	6.30664	6.01089	6.33738	6.17719	6.1406	5.96549
COQ5	Q5HYK3	6.55054	6.30509	5.77541	6.60184	6.66242	6.07929	6.3187	5.90236	6.35493	6.37529	5.95996	5.60936	6.61974	6.1304	6.5716	6.18308
PIK3CB	P42338	6.32413	6.46157	6.94719	6.00157	6.21393	6.79257	5.90607	6.58305	6.0997	6.57637	6.71981	6.68669	6.6501	5.77617	5.5939	5.66716
EPM2AIP1	Q7L775	6.08637	6.23007	5.98227	6.58736	5.95315	6.18109	5.8836	6.24517	6.24032	6.24314	6.04476	5.94398	6.89445	6.93687	6.85479	6.5926
TCIRG1	Q13488	6.29458	6.65329	6.16439	6.52499	6.39295	6.36267	6.33279	6.32082	6.1145	6.20871	5.81248	6.05777	6.15308	6.11216	6.2915	6.20334
TAX1BP1	Q86VP1	6.4515	6.37146	6.39725	6.35195	6.73011	6.7791	6.26471	6.97827	6.48044	6.53421	6.41961	6.51615	5.57562	5.32558	5.33906	5.48498
MRPL45	Q9BRJ2	6.1663	6.28427	5.78099	6.48722	6.1978	5.9271	6.56924	6.10333	6.17339	5.99225	5.79815	6.07943	6.58815	6.46858	6.67977	6.70403
ZNF687	Q8N1G0	6.30101	6.65057	5.8723	6.64146	5.9767	6.14957	6.43011	6.04731	6.23954	6.44362	5.7357	5.99532	6.38334	6.13442	6.56858	6.43047
SLC27A4	Q6P1M0	6.06522	6.58053	6.5895	6.51902	6.09461	6.42197	6.25494	6.14347	6.16074	6.51725	5.76331	6.0689	6.30195	6.42644	6.32019	6.67197
STK3	Q13188-2	6.61903	6.10233	6.76762	5.97183	6.5829	6.37933	6.20587	6.45537	6.5993	6.17496	6.67195	6.32383	6.00638	5.63144	5.79247	5.71538
TBC1D24	Q9ULP9	5.6987	6.04178	6.34467	5.72041	6.12513	6.17034	6.28313	6.04872	6.40878	6.34758	6.47179	6.33806	6.34147	6.38874	6.81145	6.45925
NCBP3	Q53F19	6.52602	6.53821	6.04336	6.72364	6.20831	6.29479	6.28492	6.1008	5.99903	6.23171	6.12037	6.16065	6.4487	6.1295	6.03064	6.15936
MED15	Q96RN5	6.08784	5.73398	6.19811	5.2229	6.77341	6.3318	6.02162	6.84161	6.19988	6.47735	6.51061	6.54019	6.74433	6.10033	6.00771	6.20833
TXNRD2	Q9NNW7	6.44157	6.22036	5.8076	6.74772	6.12847	5.77048	6.74006	5.76982	6.16325	5.9924	5.95413	5.90884	6.67044	6.45664	6.46801	6.7602

TRPV2	Q9Y5S1	6.4059	6.81853	6.08059	6.72221	6.14198	6.14648	6.3406	5.92514	6.17007	6.1805	5.72055	5.76758	6.52735	6.24316	6.34514	6.46421
CEP128	Q6ZU80	6.30175	6.45442	5.96975	6.48661	6.05058	6.17396	6.125	6.29607	6.17807	6.25424	6.27435	6.21683	6.89002	6.10925	6.11383	6.10525
TRAF1	Q13077	6.25654	5.95761	6.38757	5.6967	6.5919	6.44505	6.18123	6.48025	6.44933	6.11548	6.20126	5.89814	6.5226	6.0844	6.43482	6.29713
BPNT1	O95861	6.13983	5.91935	6.75344	5.58956	6.07016	6.81082	6.31544	6.39469	6.52888	6.65755	6.67626	6.44393	5.89192	6.12385	5.93116	5.75317
FAM111B	Q6SJ93	5.60157	6.29835	6.50903	5.87553	6.10243	6.4737	5.98877	6.41833	6.26281	6.56285	6.28594	6.26309	6.04953	6.56463	6.4352	6.30824
RABEP1	Q15276	6.34727	5.96773	7.06783	5.77986	6.53038	6.3712	6.04949	6.82627	6.60542	6.29411	6.71141	6.88726	6.26235	5.438	5.41405	5.4474
DOT1L	Q8TEK3-1	6.61282	6.56348	6.33515	7.14609	6.34491	6.04999	6.50753	6.38514	6.44653	5.80917	6.40352	6.20122	5.69828	5.77398	5.80239	5.9198
NMT2	O60551	6.3312	6.39782	6.70519	6.13601	5.86552	6.17046	6.39568	6.50391	6.41644	6.4912	6.6456	6.94686	5.65382	5.90209	5.53247	5.90571
STAM2	O75886	6.44858	6.26934	6.74546	6.33904	6.52627	6.59048	6.09219	6.38815	6.36889	6.45967	6.6202	6.58437	6.15831	5.5247	5.399	5.48536
DNAJB12	Q9NXW2-2	6.49536	6.75897	6.25691	6.98906	6.54943	6.37106	6.33634	6.21455	6.35564	5.9703	6.10274	6.13553	6.02233	5.57266	5.90246	5.96667
PSMB4	P28070	6.15197	6.08923	6.18059	5.98865	6.47902	6.33973	6.32843	6.427	6.33934	6.36896	6.22622	5.99393	6.51236	6.11156	6.46582	5.99719
ACTR8	Q9H981	6.66006	6.44124	6.71682	6.56703	6.06378	6.39421	5.85297	6.15772	6.53284	6.21421	6.71449	6.37467	5.6893	5.80251	5.95466	5.86349
POLA2	Q14181	5.69242	6.07219	6.08622	5.83167	5.84617	6.27216	5.83018	6.18337	6.27643	6.01692	6.27427	6.15393	6.79586	6.75211	7.02986	6.88623
TMEM70	Q9BUB7	6.24742	6.72167	5.92063	7.16531	6.21695	5.86721	6.13071	6.33327	6.15152	5.75189	5.91382	6.01526	6.20804	6.21695	6.7577	6.38166
LAMB2	P55268	6.90233	7.4405	6.41309	6.80843	6.42689	6.19409	6.26245	5.85594	6.57918	6.74023	6.303	6.46296	5.57666	5.40345	5.30698	5.32383
ACSF3	Q4G176	6.51076	6.61062	6.08217	6.66537	6.32201	6.20183	6.48943	5.73865	5.86775	6.04167	6.20503	6.10478	6.45344	6.32053	6.26141	6.12455
ABHD10	Q9NUJ1	6.08265	6.40953	5.66451	6.14647	6.09094	6.20141	6.4057	6.27306	6.33404	6.26772	5.9497	6.08909	6.56467	6.06967	6.90704	6.54379
UHRF2	Q96PU4	5.53424	6.32824	6.37149	6.24564	5.67321	6.1927	5.79033	5.86625	6.12474	6.09623	6.25404	6.18661	6.719	6.64187	7.23199	6.74341
NANS	Q9NR45	5.73254	5.74651	6.30279	5.53682	6.50572	6.6047	5.90633	6.58631	6.30784	6.72441	6.66305	6.29402	6.25173	6.45143	6.48058	5.9052
XPO6	Q96QU8	5.55615	5.73521	6.13353	5.48264	5.5066	6.2359	5.68288	6.38332	6.39664	6.31101	6.44349	6.70423	6.24247	7.00274	6.97099	7.2122
PLEKHG4	Q58EX7	6.50315	6.54084	6.60935	6.32585	6.21261	6.52877	6.29968	6.53344	6.59196	6.47648	6.5716	6.46404	5.65771	5.60359	5.41516	5.66576
VARS2	A0A140TA36	6.35237	6.19077	5.94943	6.78576	6.48556	5.85382	6.51675	5.91315	6.36589	6.28873	6.05279	6.12088	6.59289	5.92136	6.30931	6.30054
PPM1A	P35813-3	6.42601	6.0505	7.04389	5.58012	6.31055	6.42443	6.12452	6.56587	6.74317	6.56087	6.72599	6.59188	6.05799	5.95979	5.37293	5.36151
GTF2E2	P29084	5.41608	5.9777	6.21824	5.47903	5.45456	6.16247	5.75709	6.16674	5.81588	6.4335	6.16709	6.20157	6.48056	7.50439	7.55381	7.21128
ARAP3	Q8WWN8	6.47257	6.00763	6.67931	6.06522	6.11631	6.76845	6.18548	6.56762	6.15588	6.57329	6.58579	6.41809	5.94641	5.95359	5.76844	5.73593
NSMCE4A	Q9NXX6	6.06302	6.50809	5.78747	6.50087	6.11559	6.16449	6.14897	6.3085	6.04843	6.04553	6.08142	6.38491	6.73755	6.19989	6.34804	6.55724
SCAP	Q12770	6.33667	6.55471	6.21913	6.49413	6.03367	6.11468	6.30679	6.09235	6.06506	5.84891	5.98765	6.16795	6.43153	6.29944	6.38779	6.65955
PARG	Q86W56	5.61695	6.12567	6.80662	5.34323	6.1823	6.67595	5.97084	6.25599	6.11802	5.96472	6.50959	6.4664	6.57863	6.69039	6.11687	6.57785
SYNJ1	O43426	6.15428	6.44601	6.59424	6.25447	5.95583	6.3477	6.09363	6.22914	6.50797	6.64525	6.48709	6.23811	6.06606	6.12252	5.94116	5.91654
TMEM245	Q9H330	6.33961	6.40573	6.01872	6.51841	6.01202	5.89152	6.19577	6.0266	5.8539	5.93215	5.95072	5.90181	6.69434	6.7358	6.82288	6.7
TTC19	Q6DKK2	6.57733	5.81395	5.75951	5.82606	5.94613	5.58763	5.68163	6.11497	5.56971	6.23065	5.75168	6.42535	6.97209	7.37967	6.92176	7.44186
THG1L	Q9NWX6	6.53836	6.15889	6.16069	6.44643	6.21091	6.31998	6.12491	6.37523	6.24856	6.13282	6.40525	6.15906	6.13979	6.56561	6.03074	5.98278
SLC4A2	P04920	6.59265	6.7445	6.22523	7.02037	5.9166	6.00938	6.3799	6.01498	6.27904	6.25694	6.32805	6.23041	5.8762	6.0994	6.13922	5.88713
PFN2	P35080-2	5.97667	6.10131	6.75946	5.64084	6.68037	6.5187	5.95414	6.80022	6.63937	6.72149	6.56809	6.59297	5.71226	5.82815	5.8014	5.70456
RSU1	Q15404	5.35855	5.8502	6.41916	5.71312	6.30032	6.6183	6.24725	6.83576	6.63072	7.01612	6.97248	6.92778	5.76179	5.83711	5.88132	5.63001
STOM	P27105	6.40219	6.46502	5.78861	6.33965	6.14947	5.97899	6.58487	6.29993	6.36182	6.30802	5.90596	6.12048	6.51814	6.31891	6.35927	6.09866
RFX5	P48382	5.90012	6.16296	5.85774	6.16211	6.06515	5.98299	5.99655	5.97553	6.28089	6.5746	6.04815	5.96776	6.78596	6.57771	6.84289	6.81888
GPT2	Q8TD30	6.35856	6.47601	6.18229	6.68289	6.47811	6.36692	6.25433	5.98735	6.51307	6.38413	6.73366	6.42753	6.03374	5.63469	5.93204	5.9547
IL6ST	P40189	7.19927	7.24556	6.96493	7.14391	6.45669	6.26429	6.45917	6.54194	6.76162	6.91009	6.76155	6.67616	4.6767	4.78942	4.44921	4.69949
MTERF3	Q96E29	6.11256	6.25148	6.11179	6.67208	6.54097	6.28368	6.68805	6.3927	6.08405	6.14627	6.46941	6.2068	6.06241	5.56612	6.16317	6.24847
PUS1	Q9Y606	5.94408	6.20795	5.82818	6.45112	5.74628	6.00759	6.10973	5.91815	6.07311	6.22026	6.18628	6.13577	7.04502	6.51358	6.82477	6.78812
BTF3L4	Q96K17	6.24205	6.1395	6.87387	5.79695	6.24557	6.80013	6.3315	6.59782	6.58509	6.83281	6.71426	6.44069	5.55007	5.81205	5.6011	5.43652
AFF4	Q9UHB7	6.94989	7.30454	7.10496	7.70723	6.20096	6.31813	6.76899	5.7946	6.829	7.21866	7.07565	6.59191	4.38977	4.88089	4.18498	4.67985
FAM98A	Q8NCA5	6.43353	6.62682	6.32381	6.42451	6.48949	6.29982	6.13011	6.62255	6.42693	6.11997	6.25023	6.26842	6.16088	6.0779	5.84965	5.49539
DNAJA3	Q96EY1	6.43611	6.58089	6.0058	6.96024	6.40484	5.90012	6.31972	6.31744	5.98629	5.90602	5.91281	5.85386	6.34797	6.39014	6.27069	6.40709
BCAP29	Q9UHQ4-2	6.28532	6.45875	5.64464	6.77599	6.11275	5.77939	6.54372	5.75811	6.14581	5.76078	5.94797	5.87274	6.49374	6.577	6.65232	7.19098
ATP2B4	P23634	6.47193	6.18944	6.18262	6.32038	6.36489	5.97411	6.26661	6.00588	6.47106	5.98618	5.73952	5.65806	6.80062	6.20645	6.91974	6.44252
IMP3	Q9NV31	6.35513	6.78002	6.19505	6.81315	6.17859	6.02349	6.44694	5.8045	5.80725	6.31041	6.05143	5.99843	6.67316	6.18909	6.14625	6.22712
DENND5B	Q6ZUT9-2	6.31406	6.41541	6.97711	6.07935	6.15005	6.72897	6.26829	6.69414	6.42764	6.68701	6.56147	6.53855	5.71463	5.96114	5.23441	5.24778
CTDSPL2	Q05D32	6.54651	6.44215	5.76589	6.79265	6.17854	5.68898	6.52758	6.11509	6.3819	5.9687	5.76105	5.87101	6.63887	6.18354	6.3036	6.83394
HMGCS1	Q01581	6.72795	6.52168	7.6112	6.06833	6.37314	6.35647	6.30441	6.9411	6.60225	6.5334	6.87049	7.01088	4.99385	5.15319	4.99805	4.93361
ACADSB	P45954	6.24965	6.53166	5.9444	6.20061	6.43106	5.87443	6.39341	5.86244	6.16701	5.86137	5.97759	6.25739	7.01313	6.44765	6.82922	6.65897
RANBP9	Q96S59	6.36077	6.43982	6.53869	6.47228	6.60181	6.40533	6.30586	6.20426	6.4439	6.28174	6.61299	6.53473	5.92972	5.50953	5.6358	5.72276
ARFGEF3	Q5TH69	6.65278	6.33583	6.8181	6.41148	6.20162	6.59979	5.95844	6.17596	6.44909	6.60225	6.70443	6.97455	5.5697	5.58583	5.51767	5.44247
ACAT2	Q9BWD1	6.04819	6.12403	7.04359	5.98684	6.30241	7.09879	5.94084	6.61432	6.17905	6.2882	6.60649	6.58661	5.84668	5.87267	5.48142	5.97989
CDC20	Q12834	6.14007	5.90637	6.70084	6.46481	6.49352	6.38384	6.41625	6.41357	6.77498	6.487	6.60984	6.74541	5.30978	5.35056	5.88713	5.91605
BRPF1	P55201-2	6.13931	6.50506	5.77767	6.96053	6.14437	6.129	6.31215	6.1498	6.16552	5.97143	6.02043	6.07696	6.43361	6.05468	6.49355	6.66591
BRD1	O95696-2	6.28394	6.43795	5.89416	6.65502	6.10102	5.92282	6.32975	5.97387	6.24325	6.10255	6.11126	6.24614	6.39689	6.36755	6.43549	6.49834
KAT2A	Q92830	5.64668	5.97874	6.09725	6.23226	6.20496	6.24887	5.9917	5.95259	6.18497	6.31433	6.59732	6.20723	6.14693	6.30782	6.83911	7.04923
PAPOLG	Q9BWT3	6.0179	6.23323	6.45983	6.16872	6.04782	6.00335	6.17332	5.9411	6.18224	6.5081	6.57086	6.25019	6.27729	6.36356	6.4307	6.3718
STX5	Q13190	6.53369	6.66985	6.29881	6.61017	6.02415	6.11059	6.52669	5.91954	6.16207	6.23304	6.19075	6.02948	6.08033	5.93063	6.4018	6.27839
DSTN	P60981	5.79333	5.9395	6.5157	5.65963	6.48776	6.89022	6.1192	6.51126	6.41143	6.64958	6.44209	6.51463	6.0231	5.94985	5.90559	6.18714
ACAD8	Q9UKU7	6.70773	6.53876	6.04182	6.72307	6.42931	5.85421	6.2007	5.71489	6.0957	6.2474	6.10508	6.05276	6.55464	6.21016	6.39637	6.1274
MRPS10	P82664	6.15017	6.09365	5.92636	6.16434	6.3544	6.13401	6.44072	6.0436	6.28942	6.05512	6.17297	5.97327	6.59623	6.47951	6.5384	6.58783
FBXO11	Q86XK2-5	6.40772	6.9048	6.86817	7.16846	6.40174	6.25401	6.73285	6.07997	6.78627	6.45959	6.92373	6.76854	5.14109	4.73352	5.02434	5.34521
ARPC3	O15145	6.1	5.57094	6.75567	5.57688	6.60373	6.13335	6.15205	7.10627	5.78646	6.48444	6.38946	6.61565	5.84069	6.5011	6.18653	6.20228
ERGIC3	Q9Y282-3	6.69176	6.84642	5.88118	6.43571	6.44501	6.02042	6.25811	5.69054	5.97366	6.06563	6.3385	5.80288	6.8996	6.34373	6.32337	5.98349
GLO1	Q04760	6.41729	4.89316	6.58844	4.60231	6.21137	5.85367	5.58557	7.27138	5.21391	6.95419	6.24537	6.9636	5.96313	7.5408	6.76299	6.93281
RTN3	O95197	6.2314	6.43177	5.96153	6.20262	5.99974	6.10424	6.35	6.14677	5.8609	6.13861	6.04633	5.97906	6.48593	6.60875	6.66369	6.78866
RNGTT	O60942	5.19792	5.62134	6.08066	5.63972	5.61324	5.99966	5.87774	5.88984	6.02943	6.03868	6.28331	5.95594	7.45404	7.49145	7.71943	7.10758
ACTR10	Q9NZ32	6.2769	6.01273	7.05708	6.23944	6.12583	6.52093	6.34642	6.20032	6.4341	6.17096	6.5188	6.40851	5.81406	6.06679	5.88567	5.92334
GSK3A	P49840	6.70376	6.27241	6.94089	6.13972	6.24201	6.42204	6.20068	6.29855	6.65197	6.75088	6.5196	6.38673	5.64964	5.63654	5.59964	5.58494
PYCR2	Q96C36	6.53817	6.23739	5.78404	6.49395	6.30111	6.0915	6.39601	6.3468	6.14569	5.71219	6.16833	5.8384	6.64321	6.24257	6.59421	6.46644
ZNF800	Q2TB10	5.75758	5.62321	5.66798	6.30384	6.02669	5.6361	6.14451	5.52383	5.93283	5.45088	5.64892	5.87444	7.57638	6.94703	7.99229	7.89349
POLR3C	Q9BUI4	6.19965	5.88539	6.22537	5.74991	6.30164	6.23099	5.85255	5.94137	6.24578	6.18809	6.12537	5.92492	6.66909	6.68923	7.02389	6.74674
RPP38	P78345	6.12458	6.32382	5.98004	6.61793	6.273	6.20278	6.32435	5.75003	6.1143	6.19478	5.68184	5.63399	6.67408	6.51766	7.01483	6.57199
LIN9	Q5TKA1-2	6.26884	6.32984	5.98197	6.34907	6.08099	6.12472	6.42429	6.10121	6.1536	6.06805	6.28183	6.02907	6.41627	6.38785	6.56446	6.43792
GAR1	Q9NY12	6.37361	6.55354	5.69299	6.67841	6.24711	6.06693	6.35973	6.26861	6.18136	5.64037	5.70148	5.89161	6.62709	6.34676	6.69969	6.67071
MIOS	Q9NXC5	6.55774	6.74832	6.39461	5.90625	6.31919	6.65265	5.91447	6.55984	5.92597	6.43258	6.63222	6.33378	5.69397	6.11614	6.04407	5.76821
NAE1	Q13564-4	5.81807	5.81265	6.60987	5.70251	5.88976	6.42622	5.87807	6.36835	5.94014	6.33885	6.40931	6.39694	6.01366	6.95343	6.79786	6.64431
IDH2	P48735	6.59225	6.49191	5.83181	6.71887	6.2121	6.26989	6.08712	6.1756	5.95438	5.97976	5.73123	5.95822	6.67462	6.34565	6.28094	6.69565
GFUS	Q13630	6.53416	6.23077	6.95436	5.85885	6.53675	6.89753	5.97159	6.64395	6.26196	6.27137	6.70643	6.2019	5.8085	5.94579	5.62309	5.553
BRWD1	Q9NSI6-2	6.37303	6.74669	6.24	7.25352	6.19301	5.77237	6.38034	5.73868	6.24597	6.26898	5.88145	6.20991	5.85353	6.29236	6.12815	6.42203
SRSF9	Q13242	6.41403	6.73815	5.76362	6.14824	7.44357	5.97717	5.69295	6.67027	7.86183	5.98767	5.93974	6.09064	6.43127	5.53889	5.74021	5.56174
TRIM13	O60858-3	6.25687	6.83085	6.55465	7.06849	6.0391	5.92502	6.59559	5.86777	6.00504	6.20369	6.63321	6.37875	5.69597	5.65236	6.17595	6.11671
DDX49	Q9Y6V7	6.00213	6.09696	5.93364	6.43919	6.39229	6.39468	6.11934	6.04333	6.26162	6.08498	6.12555	6.15871	6.621	6.46103	6.33644	6.52911
LRRC40	Q9H9A6	6.19518	6.35074	6.65617	6.08761	6.20781	6.52944	6.31019	7.0166	5.91067	6.50953	6.34071	6.27695	5.91634	6.30373	5.65342	5.73492
TBC1D23	Q9NUY8	6.30681	6.12023	6.34547	6.13088	6.2251	6.25937	6.54631	6.15393	6.84581	6.35939	6.42507	5.5687	6.07463	5.92608	5.89745	5.81478
EIF3J	O75822	6.36455	6.37809	6.83024	5.80661	6.50521	6.67947	6.27898	6.64601	6.43614	6.38211	6.21686	6.17431	6.14055	5.71321	5.78979	5.65788
CEP152	O94986	6.79132	6.57407	6.40427	6.53762	6.35232	6.43942	6.07844	6.1911	6.46941	6.16362	6.74274	6.61344	6.08807	5.23996	5.77544	5.53874
YAP1	P46937-9	6.80564	4.93284	6.36268	4.62162	6.49398	4.66252	5.25113	7.45241	4.95471	6.5278	6.07049	7.44256	6.60583	7.6054	6.93152	7.27885
CEP135	Q66GS9	6.58851	6.52446	6.71586	6.7042	6.32362	6.72135	6.01266	6.63294	6.53024	6.08076	6.49902	6.45981	5.82426	5.41191	5.35353	5.6169
TAF5	Q15542	6.02212	6.46285	6.28967	6.92431	6.11171	6.18246	6.29075	6.00653	6.24254	5.95834	6.32753	6.14755	6.37593	5.90891	6.34242	6.40638
LMAN2L	Q9H0V9-2	6.30623	6.17224	6.15475	6.39242	6.25151	6.02635	6.21103	5.71729	6.41667	5.90282	5.98073	5.92431	6.44783	6.34523	7.08007	6.67052
MRI1	Q9BV20	6.2418	6.2432	6.80229	5.7282	6.37621	6.67398	6.27495	6.77629	6.00943	6.49498	6.44328	6.53408	5.78539	6.13009	5.8079	5.67795
GTF3C5	Q9Y5Q8-3	6.15139	6.36489	6.01856	6.41831	6.2523	6.31991	6.01757	6.20033	6.06731	6.51724	6.08817	6.17521	6.21132	6.40115	6.45323	6.34312
GRB2	P62993	6.31638	5.7296	6.31526	5.52321	6.65525	6.12341	6.05858	6.76887	6.36368	6.57963	6.69325	6.56944	6.12613	6.20043	6.10623	5.87063
FIG4	Q92562	6.38745	6.3801	6.71165	6.36419	6.03026	6.50508	6.27232	6.42839	6.21159	6.36389	6.44865	5.99103	6.0195	6.00353	6.21315	5.66923
TSNAX	Q99598	5.78038	5.80757	6.11137	5.61205	6.21233	6.58452	6.21686	6.46616	6.23206	6.10912	6.20653	6.32266	6.34052	6.59911	6.87589	6.52286
BCS1L	Q9Y276	6.24927	6.25359	5.93951	6.44903	6.17569	5.92139	6.68714	6.00222	6.35581	5.97116	6.13337	5.9143	6.54208	6.1779	6.84916	6.37839
EMC3	Q9P0I2	6.55464	6.38223	5.97992	6.73944	6.02438	6.1338	6.07455	6.04089	5.92683	6.44363	6.02537	6.11025	6.25811	6.32553	6.53042	6.45
MELK	Q14680	6.78892	6.61101	7.26942	6.62531	6.21904	6.68787	6.34411	6.53174	7.01602	6.99744	6.87794	6.74993	4.90689	4.97483	4.70857	4.69097
PPIL2	Q13356	5.9083	6.17202	6.54296	6.1811	6.1403	6.21963	6.16781	5.72395	6.51758	6.33084	6.20168	5.98921	6.63112	6.23772	6.68201	6.35376
PGM2L1	Q6PCE3	6.61746	6.13685	6.79519	5.79325	6.40428	6.82734	6.19421	6.31408	6.44579	6.31661	6.57342	6.07973	5.97615	5.85679	5.71839	5.95047
BTN2A1	Q7KYR7	6.27665	6.48472	5.96732	6.37362	6.35364	6.06718	6.20479	6.48559	6.23653	6.07047	6.04414	5.91812	6.6815	6.29243	6.34942	6.19387

CD2BP2	O95400	6.37658	6.21978	6.39536	5.99602	6.51928	6.31715	6.00467	6.44144	6.10984	5.96206	6.61475	6.11446	6.96036	5.9094	5.99041	6.06843
PWP1	Q13610	6.81781	5.97078	6.91159	6.97946	6.33847	6.94307	6.5214	6.32884	6.23935	6.30035	6.53807	6.69418	5.37645	5.41473	5.35128	5.27418
PPHLN1	Q8NEY8-3	6.225	6.17188	5.82161	6.821	6.09966	5.64074	6.92767	5.64071	6.23124	5.70391	5.70737	6.06869	6.45678	6.41131	6.92969	7.14274
CNOT9	Q92600	5.94164	5.33654	6.37169	5.27144	6.52359	5.85908	5.80431	6.6301	5.85563	6.84986	6.3885	6.85177	6.0799	6.75087	6.83138	6.65369
WASF2	Q9Y6W5	5.97903	6.47958	6.7929	6.29693	6.70195	6.83728	6.15171	6.45478	6.36832	6.38614	6.81424	6.3454	5.76113	5.85352	5.79141	4.98569
SAMM50	Q9Y512	6.30129	6.37882	5.92564	6.48155	6.58865	5.87955	6.10865	6.26457	6.06517	6.05562	5.83864	5.83538	6.66421	6.24964	6.74074	6.62187
WDR45B	Q5MNZ6	6.71388	6.93124	6.42214	6.49494	6.09082	6.34927	6.37707	6.23358	6.49978	6.62638	6.54562	6.42273	5.59073	5.40922	5.72243	5.57017
MANBA	O00462	6.18089	6.73679	6.17809	6.57769	6.43174	6.2014	6.33198	6.13926	6.10097	6.4399	6.1244	6.31954	6.13907	5.95427	6.15944	5.98457
RELB	D6R992	6.18005	6.36574	7.07109	6.00265	6.53119	6.38756	6.26613	6.46564	6.84813	6.36322	6.96547	6.70723	5.53554	5.38105	5.57942	5.34988
DHFR	P00374	6.15094	6.35416	6.78441	5.88225	6.36837	6.51443	6.43436	7.34657	7.40723	6.89706	7.58213	7.57801	4.48753	4.72279	4.66681	4.82294
TSN	Q15631	6.35289	6.31674	6.21569	6.09105	6.47326	7.06472	5.91045	7.33142	6.13907	6.26755	6.31341	6.2791	5.31053	6.21011	6.19841	5.52559
NSMAF	Q92636	6.35423	6.13125	7.28941	6.33151	6.57584	6.64445	6.02875	6.59387	6.4955	6.65276	6.55256	6.50432	5.39236	5.97768	5.4713	5.00422
NDUFV1	P49821	6.34879	6.02328	6.00542	6.63971	6.09207	5.81222	6.41476	5.4962	6.33242	6.01141	6.25297	6.00092	7.02919	6.30898	6.57501	6.65666
MTX1	Q13505	5.95012	5.74397	5.78794	5.8568	6.03975	6.01446	6.28387	6.46504	5.76934	6.38241	6.24174	6.77717	6.34338	6.57916	6.8891	6.87575
CPSF4	O95639	6.73341	6.71306	5.78424	6.9852	6.42624	6.2949	6.20751	5.95499	5.90712	6.26593	5.83766	6.18912	6.49241	5.9547	6.04507	6.20842
STX4	Q12846	6.20078	6.71996	5.93951	6.76078	5.88961	6.11284	6.38983	5.96291	6.2304	6.4412	6.33974	6.29415	6.05106	6.13822	6.34791	6.18109
MTCH2	Q9Y6C9	6.44871	6.29836	5.97552	6.95377	6.255	6.23296	6.17154	6.24843	6.23441	6.01091	6.42614	5.96769	6.18205	5.89779	6.37858	6.31814
RPL36	Q9Y3U8	5.88772	6.21468	5.89984	5.89805	6.22166	6.29283	6.78458	6.3888	7.09256	5.55842	6.10623	6.27153	6.2288	6.86123	5.75463	6.53841
OPHN1	O60890	6.0568	6.10789	6.45244	5.85489	6.10036	6.1952	6.29051	6.44933	6.16408	6.20065	6.39909	6.42511	6.19385	6.27072	6.52081	6.31275
TENM3	Q9P273	5.92459	6.12658	5.9024	6.59231	6.0149	6.39298	6.38621	6.06753	6.33285	5.97701	6.17673	5.90612	6.72793	6.51973	6.39586	6.55629
GPX8	Q8TED1	6.57636	6.48571	6.06924	6.64657	6.35464	6.14882	6.11799	6.38531	6.0563	5.96536	5.85968	6.06237	6.26871	6.42751	6.33683	6.23861
FAM114A2	Q9NRY5	6.10093	5.92345	6.4821	5.60861	6.32393	6.52904	6.04068	6.15699	6.53015	6.58846	6.82538	6.48781	6.33958	6.07576	5.93989	6.04724
TAP1	Q03518	6.17827	6.35407	6.22103	6.67161	6.07243	5.87036	6.29714	5.96311	6.13947	6.18663	5.86337	5.74449	6.64715	6.13941	6.74711	6.90435
ERI3	O43414	6.03352	5.18525	6.44249	5.10301	6.22824	6.17553	5.96006	6.49837	5.71577	6.48637	6.26963	6.95339	6.26249	6.89907	6.9549	6.83192
NLRP2	Q9NX02	6.49842	5.96788	7.31036	6.23096	6.39796	6.57802	6.46138	6.3315	6.56907	6.41987	6.93687	6.53893	5.50633	5.30665	5.64455	5.30126
AUP1	Q9Y679	6.31942	6.3058	6.15883	6.60189	6.12032	6.16998	6.32283	5.81855	6.2984	6.09682	6.4333	6.2582	6.3975	6.10818	6.38106	6.20892
NT5DC1	Q5TFE4	6.05896	6.17242	6.54166	5.92856	6.17719	6.25121	5.63716	6.81501	6.12087	6.66289	6.6991	6.65571	5.54537	6.35312	6.16492	6.21584
SLC25A22	Q9H936	6.57366	6.10441	5.97343	6.37219	6.28515	6.09116	6.21231	5.74653	6.10651	5.91991	6.05466	5.80233	6.77215	6.00879	7.09528	6.88153
JAK2	O60674	6.40921	6.78748	6.55385	6.91644	6.38336	6.22512	6.43387	6.16484	6.8136	6.74294	6.62219	6.52795	5.24582	5.52018	5.27083	5.38233
GGCT	O75223	6.41179	6.39714	6.94124	6.04727	6.42914	6.90504	6.00628	6.44091	6.6233	6.51085	6.61737	6.53724	5.6236	5.55786	5.5998	5.35118
PSME3	P61289-2	5.24229	5.53301	5.84694	5.081	5.95209	6.08139	5.7453	5.95468	5.76382	5.98252	6.20656	6.10798	7.47965	7.66385	7.81085	7.54808
KIN	O60870	6.00474	6.29022	6.12708	6.31543	6.14522	6.38508	6.3471	5.71284	6.47057	6.20811	6.41478	6.13391	6.43811	6.09566	6.61231	6.29886
MGME1	Q9BQP7	6.42006	6.391	6.09018	7.1588	6.34392	6.24921	6.64417	6.03869	6.29704	6.01065	6.16181	6.20248	6.00625	5.78895	5.98857	6.20823
EIF2B2	P49770	6.4986	6.72856	6.84403	5.69174	6.36797	6.7879	6.06278	7.05166	6.46053	5.75761	6.54113	6.6622	5.43267	5.50965	5.29635	5.30662
ALG5	Q9Y673	6.31704	6.1843	5.98093	6.3986	6.11596	5.65652	6.3132	5.95905	6.21056	5.9013	6.07463	5.75855	6.74964	6.45981	6.92207	6.99785
CYP20A1	Q6UW02	6.26827	6.34737	5.81681	6.42816	6.11784	5.99098	6.32259	5.8551	6.16698	6.18919	5.86158	5.81639	6.77052	6.45249	7.02134	6.57439
LSM4	Q9Y4Z0	6.11095	6.07357	6.04765	6.06817	6.45449	6.01886	6.29021	6.24194	6.28356	6.08155	6.29971	6.2705	6.54579	6.32543	6.53122	6.35641
TAF1	P21675-4	6.23175	6.50626	6.10154	6.8412	5.74835	5.81887	6.22018	6.27275	6.18923	6.03115	6.12782	6.36672	6.23123	6.17693	6.61678	6.51923
MRPL47	Q9HD33	6.3463	6.01786	5.85101	6.46945	6.53497	5.90141	6.75132	5.9341	6.27595	5.90044	6.00385	5.94815	6.57631	6.34241	6.65999	6.48648
SRP9	P49458	5.62557	5.20723	5.71913	5.145	6.34708	5.81628	6.02617	6.11919	6.37371	6.35443	6.23903	6.40662	6.70242	7.08239	7.29523	7.54052
GTF2H1	P32780	6.11963	6.57277	6.12724	6.91536	6.35797	6.16103	6.43271	6.06978	6.14601	6.00356	5.98242	6.04144	6.50798	5.8133	6.64712	6.10168
SNRPE	P62304	6.16471	6.37693	5.73524	6.50682	6.08551	5.92941	6.28919	6.07418	6.12247	5.84795	5.90329	6.08318	6.92843	6.15426	7.01041	6.78802
ATP5MG	O75964	6.39173	5.67204	5.86229	5.73386	6.45533	6.06027	6.2463	6.35487	5.67463	6.01466	5.92001	5.68929	6.79849	6.77542	7.40621	6.94461
RPL19	P84098	5.87668	6.61384	5.72628	6.05293	6.12267	6.36828	6.61273	6.48376	6.76482	6.06511	6.15254	6.43645	5.78206	6.68257	5.77659	6.4827
CISD2	Q8N5K1	6.53138	6.30742	5.99012	6.25651	6.15991	5.93385	6.10698	6.04904	6.26354	6.46077	6.33088	6.28089	6.64186	6.22193	6.20666	6.25825
RPRD1B	Q9NQG5	5.95083	6.11704	5.8985	5.91768	6.20941	6.19279	5.87754	6.09426	6.37543	6.07142	6.27751	6.11699	6.79705	6.68321	7.05828	6.36206
MT-CO2	P00403	5.90398	5.76311	5.1972	5.63737	5.72396	5.9069	6.16442	6.53275	5.86928	5.36295	5.78091	5.77739	6.99606	7.94233	7.17418	8.26721
SH3PXD2B	A1X283	5.72706	5.62208	6.54449	6.02039	5.90213	6.55982	6.24967	6.2827	6.34711	6.52769	6.48397	6.55968	6.12647	6.57283	6.01695	6.45695
HSPA2	P54652	6.0174	6.0092	6.34448	5.59116	5.9982	6.05602	6.07281	5.8956	6.19332	6.29159	6.37535	6.55372	6.84171	6.68595	6.57182	6.50168
GADD45GIP1	Q8TAE8	6.26572	6.24798	5.73053	6.38532	6.3481	6.44452	6.50589	6.43974	5.97945	6.18177	5.92725	6.03995	6.412	6.29852	6.27571	6.51755
SYDE1	Q6ZW31	6.65505	6.16	7.36488	6.6837	6.07148	6.26588	6.30947	6.35425	6.63515	6.70711	6.84586	7.09527	5.33823	5.25512	5.10385	5.15468
TTC4	O95801	6.25806	6.23925	6.88039	6.08685	6.03937	6.64422	6.18504	6.14988	6.47157	6.83006	6.2579	6.52632	6.06541	5.92135	5.58955	5.85477
IRF2BP2	Q7Z5L9	5.91876	6.39135	6.01342	5.78451	6.11742	6.38129	5.94268	6.18112	5.9248	6.06908	6.32346	6.09028	7.34234	6.4508	6.65961	6.40906
ARAP1	Q96P48	6.26485	6.19696	7.00424	6.06675	6.09205	6.4457	6.20299	6.23045	6.3242	6.65401	6.54468	6.37058	5.89766	6.16614	5.95117	5.58756
LRRC8C	Q8TDW0	6.29611	6.83631	6.27492	6.92766	6.21563	6.04997	6.30665	5.82221	6.23642	6.04878	6.24581	6.32021	6.08811	5.92328	6.17495	6.23298
FMNL1	O95466-3	6.46467	6.49103	6.14714	6.28344	6.57699	6.46663	6.26742	6.29589	6.49007	5.98372	6.06714	5.8566	6.1398	6.11433	6.07069	6.28444
PDHX	O00330	6.71824	6.36077	5.76957	6.43766	6.76587	6.14002	6.06981	6.348	6.31849	6.0849	6.15974	6.15353	6.82201	5.67659	6.15783	6.01697
PGLS	O95336	5.8292	5.26559	5.81322	4.55184	5.80067	5.94664	5.51844	7.33908	5.076	6.19039	5.20441	6.19387	5.32105	10.1874	6.77327	8.98895
GRHPR	Q9UBQ7	6.46657	6.31563	6.42968	5.83871	6.51861	6.30041	6.27216	6.48584	6.15226	6.29838	6.25486	6.18421	6.33161	6.11583	6.1	5.93522
CAND2	O75155	6.19379	5.80959	6.27241	5.44387	5.85055	6.2137	5.98486	6.21639	6.0491	6.47952	6.45405	6.21664	6.39954	6.94945	6.83848	6.36127
YBX1	P67809	6.35168	6.20778	7.11107	6.40971	6.21669	6.18786	6.41253	6.01059	6.81995	6.49853	6.91941	6.75123	5.38767	5.60338	5.17079	5.94114
RAD54B	Q9Y620	5.58002	5.77505	6.2967	5.82419	6.27324	6.47312	6.20601	6.06192	6.40619	6.41228	6.59944	6.15018	6.2243	6.50328	6.68451	6.52958
SRM	P19623	6.01409	6.40338	6.84205	5.90127	6.70631	7.05294	6.3552	7.05414	6.60993	6.21009	6.76378	6.45747	5.40713	5.48461	5.35303	5.3846
UBXN6	Q9BZV1	6.24078	6.13652	6.59016	5.98012	6.24568	6.49738	5.83972	6.63841	6.17771	6.63593	6.50695	6.40217	6.32505	5.86687	6.11803	5.79851
CDK6	Q00534	6.32467	6.11527	6.58172	5.74634	6.13175	6.54335	6.4277	6.6768	6.53471	6.27581	6.52153	6.30035	5.89684	6.00885	5.95402	5.96029
FUS	P35637	6.41742	6.47759	6.67202	6.89585	6.22164	6.40431	5.80875	5.91059	6.22076	6.41392	6.4365	6.11652	6.38713	5.64994	5.96437	6.00269
HARS2	P49590	6.40858	5.99577	5.96757	6.53507	6.11374	5.84145	6.82663	6.00221	6.17417	5.8822	5.90274	5.94589	6.44054	6.71166	6.57915	6.67262
DNAJC3	Q13217	6.23736	6.54804	5.81663	6.49778	6.28104	6.11082	6.22731	6.11675	6.14451	6.05748	6.24791	6.0756	6.55082	6.12953	6.82971	6.12871
RPS25	P62851	6.41469	6.41143	6.5142	6.19943	6.47577	6.51106	6.21921	6.66079	6.60031	6.42508	6.03351	6.04422	5.61003	6.18367	5.7588	5.93779
PPP2R5C	Q13362-4	6.227	6.33108	6.62622	6.23216	6.31248	6.4994	6.29992	6.08158	6.24975	6.36957	6.49913	6.40141	6.03075	6.0151	5.93168	5.89278
L3MBTL2	Q969R5	6.2355	6.34088	5.88633	6.87218	5.8215	6.36733	6.4358	5.90844	6.13807	5.94167	6.01224	5.9584	6.26987	6.52106	6.58559	6.70514
CLIP2	Q9UDT6	6.34849	6.44979	6.90712	5.80995	6.6399	6.80641	5.8387	6.77235	6.2332	6.65441	6.67741	6.55701	6.08686	5.66811	5.28591	5.26438
GALK2	Q01415	6.65595	5.93081	6.76765	6.01243	6.14526	6.46516	6.1982	6.38284	6.36122	6.07028	6.36185	6.49421	6.06491	6.13928	6.20785	5.74211
FBXO17	Q96EF6	6.2587	6.2323	6.28251	6.42134	6.1106	6.01514	6.32955	6.59274	6.49551	6.43754	6.09367	6.3615	6.08214	6.35784	6.37294	6.21798
ADD2	P35612	5.92449	5.99101	6.15518	5.5976	6.03853	6.02535	5.86792	6.34868	6.24092	6.2298	6.01021	6.01542	6.85681	6.90794	7.01464	6.7755
YKT6	O15498	6.15914	6.24932	6.68239	5.86272	6.11115	6.74024	6.36824	6.87793	6.346	6.52717	6.21062	6.35475	5.90892	6.12613	5.62271	5.85258
COA7	Q96BR5	6.71058	6.31502	6.1289	6.30514	7.03793	6.31616	5.77124	6.06228	5.80196	5.71227	5.87144	5.91625	7.37278	6.03191	6.34532	6.30081
ARFGAP2	Q8N6H7	6.69802	6.58327	6.63715	6.09977	6.33145	6.92263	6.65084	6.63256	6.28887	6.64687	6.51995	6.3329	5.54657	5.45955	5.31107	5.33853
FAM160B1	Q5W0V3	6.54678	6.37013	6.7459	6.01976	6.28343	6.61743	6.17814	6.68358	6.3248	6.59838	6.29528	6.1852	5.88661	5.94326	5.71578	5.60552
PNPLA8	Q9NP80	6.03928	6.43219	5.71415	6.68165	6.06341	5.982	6.24309	6.04153	5.85413	5.9536	6.04191	6.31809	6.72612	6.56189	6.74362	6.60332
COL18A1	P39060	7.23055	7.3415	6.32316	7.08993	6.72262	6.65567	6.69066	6.1798	6.23673	6.49544	6.40829	6.06961	5.06139	5.26945	5.10389	5.1213
NISCH	Q9Y2I1	6.49451	6.37277	6.21811	6.12204	6.51481	6.38059	6.24199	6.43182	6.34272	6.1846	6.73009	6.41858	6.0107	5.69957	5.94345	5.89364
SFMBT1	Q9UHJ3	6.00718	6.17397	6.17612	6.805	6.20124	5.94976	6.44727	6.16215	6.31552	6.15856	6.36471	6.14157	6.23326	6.18502	6.28428	6.39438
TBC1D10B	Q4KMP7	5.91583	6.31373	5.71573	6.01444	6.30324	5.89916	6.23546	6.01063	6.18367	6.51068	5.95733	6.3342	6.59915	6.7322	6.44395	6.83058
PRMT5	O14744	5.91554	6.00659	6.66193	5.97356	6.13907	6.64849	6.27978	6.00046	6.24832	6.2139	6.45023	6.43471	6.30577	6.20274	6.18753	6.33138
MLYCD	O95822	6.33764	6.67798	5.87232	6.70256	5.96223	5.82836	6.22707	6.27663	5.83496	6.1337	6.23536	6.1664	6.47256	6.39136	6.53587	6.34501
ECSIT	Q9BQ95	6.47018	6.38949	6.29883	6.3657	6.62002	6.21353	6.34578	6.36348	6.18978	6.0354	6.4655	6.1204	6.64266	5.83748	5.90576	5.73601
EHD3	Q9NZN3	6.24209	6.3502	6.42667	5.83717	6.42125	6.51228	6.19758	6.48559	6.50377	6.41912	6.24669	6.17112	6.41039	5.79575	6.15935	5.82097
UGGT2	Q9NYU1	6.37623	6.28898	5.96803	6.86031	6.32121	6.38135	6.28548	6.09031	6.34685	5.94702	5.8853	6.09332	6.46602	6.10062	6.18475	6.40421
ITGA4	P13612	6.41817	6.18622	6.45199	6.44201	6.58939	6.24874	6.37979	5.76032	6.61703	6.0949	6.64443	6.60718	6.05238	5.92186	5.79596	5.78963
GRPEL1	Q9HAV7	6.24055	5.1399	5.57426	5.32528	6.41221	5.14672	5.64226	6.25124	4.92409	6.22064	5.51806	6.79829	6.96535	7.71877	7.86291	8.25944
RPS6KA3	P51812	6.14483	6.07942	6.57552	5.74588	6.23856	6.89718	6.17589	6.91471	6.22854	6.39662	6.66198	6.33272	6.18889	6.18612	5.70229	5.53087
SRGAP1	Q7Z6B7	6.16208	5.98274	6.56635	6.21923	6.09753	6.81499	6.16177	6.49865	6.47059	6.52752	6.67115	6.66366	5.73627	5.81224	5.85653	5.7587
TMEM201	Q5SNT2	6.41707	6.74374	5.95381	7.44044	6.30823	6.04724	6.18608	5.78118	6.14057	6.28353	6.40278	6.23088	5.94594	5.93256	5.91387	6.27211
MBD1	Q9UIS9-9	6.09697	6.29351	5.90453	6.9044	5.89492	5.61851	6.5569	5.55588	6.00433	6.14924	6.11411	6.25236	6.70991	6.57311	6.66873	6.70258
SEPTIN11	Q9NVA2	5.89924	6.43737	6.32104	6.04955	6.3341	6.64271	6.00235	6.52984	6.30551	6.51825	6.33323	6.27331	6.24307	6.34722	6.02853	5.73467
GPHN	Q9NQX3-2	6.53461	6.21008	6.63127	6.07542	6.65266	6.66883	5.98295	6.54247	6.5716	6.18739	6.72777	6.85184	6.04571	5.35258	5.46488	5.49993
MED13L	Q71F56	6.34548	6.47754	6.43184	6.62456	6.21506	6.08533	6.15397	6.14769	6.30753	6.1531	6.15838	6.25943	6.30034	6.06311	6.02557	6.25108
TSC1	Q92574	6.54585	6.2983	6.57721	6.10155	6.26086	6.65798	6.23706	6.42202	6.60072	6.45002	6.82331	6.89791	5.65592	5.9104	5.30205	5.25886
PKN3	Q6P5Z2	6.5625	6.36446	6.67724	6.92864	6.52506	6.42638	6.72112	6.28161	6.58492	6.85775	6.11232	6.4325	5.29127	5.36593	5.25902	5.60928
ATAD2B	Q9ULI0	6.2905	6.2446	6.09076	7.08514	5.96692	5.74046	6.22609	5.66263	6.36527	6.08816	6.28527	6.16257	6.5809	6.08083	6.38164	6.74828
ARHGAP45	Q92619	6.62058	6.27852	6.62204	5.80373	5.84258	6.59714	5.75056	6.11995	6.63111	6.48711	6.33535	6.68389	6.21975	6.0671	5.81511	6.11648
ZC2HC1A	Q96GY0	6.27607	6.22763	6.90588	5.97952	6.12719	6.55335	6.16074	6.43858	6.27904	6.72088	6.56525	6.56261	5.75619	6.0729	5.74135	5.63283
LATS1	O95835	6.26814	6.4062	7.04919	6.3958	6.30068	6.82831	6.16532	6.59871	6.30777	5.98969	6.43659	6.98463	5.80868	5.525	5.43379	5.50151
CCDC6	Q16204	6.29473	6.22122	6.71422	5.79656	6.41216	6.83654	5.8166	6.34246	6.23156	6.50662	6.54025	6.55925	6.45551	5.93691	5.51256	5.82286

TLE3	Q04726-4	5.79974	6.11051	5.85157	5.48683	6.10014	6.5802	5.80936	6.32689	5.88783	5.95808	6.37899	5.98324	7.23232	6.82367	6.95575	6.71487
IGF1R	P08069	6.22525	6.31939	6.00634	6.20198	6.01986	6.12575	6.17728	6.27441	6.07253	6.1247	6.12508	6.01439	6.78843	6.04236	6.83272	6.64952
CCDC186	Q7Z3E2	6.38555	6.27675	6.60478	6.00078	6.17329	6.6176	6.30882	6.67606	6.42049	6.8377	6.89309	7.04291	5.72072	5.74284	5.44123	4.85739
EAPP	Q56P03	6.03127	6.65303	5.97094	6.13067	6.1524	6.60979	6.00104	6.18786	6.06855	6.14869	6.25239	6.16	6.68909	6.39652	6.08371	6.46404
SLC12A9	Q9BXP2	6.56122	6.6038	6.20931	6.63222	6.09744	5.88598	6.23675	6.39148	6.14605	6.46395	6.50555	6.42181	5.84684	6.01499	5.95921	6.02339
PCBP1	Q15365	6.08389	5.68864	6.18552	5.96253	6.43115	6.91533	5.54657	5.97298	6.17969	6.49857	6.49299	6.41359	6.50617	7.03625	5.86825	6.21787
NFIC	P08651	6.29549	5.99669	6.18744	6.24464	6.13016	5.88646	6.50668	5.90924	6.32525	6.13915	6.19298	6.08626	6.61004	6.27581	6.61347	6.60024
HIBCH	Q6NVY1	6.39009	6.87147	5.59323	6.85337	6.11241	6.0447	6.01313	6.46506	5.89046	6.2445	5.95348	5.83532	6.33671	6.48474	6.20096	6.71039
GIPC1	O14908	6.4614	6.40672	7.12957	6.57508	6.49259	6.38129	6.43637	6.24548	6.71741	6.55975	6.75328	6.68783	5.52861	5.30304	5.09406	5.22752
ARHGEF11	O15085-2	6.62634	5.77044	6.70594	6.08514	5.91436	6.44642	5.85499	6.23371	6.03949	6.6324	6.46266	6.47744	6.21467	6.24718	6.07601	6.2128
MED27	Q6P2C8	5.96343	6.4528	6.07059	6.26695	6.00427	6.30861	6.16339	6.14454	6.00218	6.15744	6.46878	6.28489	6.27271	6.25465	6.71535	6.46944
DNM3	Q9UQ16-4	5.97774	6.23598	6.64306	6.0533	6.21492	6.54543	6.0092	6.46196	6.32376	6.26074	6.44983	6.51606	6.05983	6.27249	6.1097	5.866
RAB8A	P61006	6.22325	6.41618	6.09371	6.55414	5.93821	6.19908	6.30165	6.18832	5.98694	6.18747	6.29416	6.27621	6.01366	6.31243	6.5758	6.43879
RAB35	Q15286	6.20589	6.60308	6.18525	6.54282	6.13635	6.31398	6.3213	5.99406	5.99569	6.15197	5.62539	5.79184	6.41915	6.55146	6.52448	6.63731
SEC61A1	P61619	6.19838	5.95036	5.84229	6.59581	6.17421	6.09227	6.23432	5.47969	5.88512	6.16597	5.59617	5.84069	6.89284	6.76847	6.91315	7.37024
CIZ1	Q9ULV3	6.91127	7.04134	6.73744	7.50657	6.05518	6.25842	6.65643	6.04735	6.54842	6.49832	6.69486	6.58785	5.13844	4.99705	5.1326	5.18847
TATDN1	Q6P1N9	5.65862	5.77814	6.52601	5.76906	6.28364	6.54343	6.1122	6.86063	6.61543	6.87943	7.28224	7.2185	5.85835	5.48486	5.44758	5.68188
DCBLD2	Q96PD2-2	6.27981	6.69816	6.28883	6.88777	6.31929	5.7126	6.58728	5.85113	6.28808	6.38055	6.42824	6.22699	6.18422	5.8073	6.22272	5.83702
MAPRE2	Q15555	6.13026	6.25413	6.80519	5.9235	5.99427	6.56709	5.7647	6.75346	6.36419	6.52844	6.54878	6.66852	5.7799	6.01752	6.06735	5.8327
ARPC5L	Q9BPX5	6.10952	5.8385	6.56472	5.77546	6.26126	6.30503	5.88338	6.65209	6.05611	6.47643	6.45245	6.5222	6.42564	6.2378	5.95479	6.48464
RTRAF	Q9Y224	5.83699	4.357	5.72526	4.43188	5.77826	4.19884	4.56884	6.62868	4.28776	6.35048	5.47775	6.67355	6.61865	10.4958	9.07162	9.49865
SFXN3	Q9BWM7	6.71273	6.33065	5.92017	6.43936	6.29371	5.72503	6.27433	5.849	6.3064	6.03481	6.0477	6.20043	6.6895	6.40171	6.49645	6.27801
GRAMD1A	Q96CP6	6.81866	6.86956	6.31344	7.50858	6.61458	6.12851	6.82223	6.58393	6.52953	6.33704	5.89818	6.34085	5.05996	5.24538	5.38703	5.54255
OSBPL9	Q96SU4-2	6.4075	6.47922	6.34238	6.15568	6.09521	6.55302	6.07777	6.09096	6.39689	6.61723	6.40351	6.31722	6.08173	6.12181	5.76879	6.09106
GTF2H4	Q92759	6.16494	6.40561	5.97849	6.47687	6.443	6.14849	6.58892	6.32927	6.12383	6.30412	6.10412	5.94377	6.31332	6.28797	6.16919	6.21809
ARSB	P15848	6.51215	6.17458	6.01184	6.48821	6.28111	6.10305	6.30304	5.85365	6.21568	6.35667	6.29569	6.13711	6.18475	6.28078	6.31439	6.48729
KAT6B	Q8WYB5	6.37398	6.15013	6.0861	6.3045	6.2903	5.92067	6.24446	5.50238	6.49311	6.41391	5.93145	6.16173	6.80752	6.35895	6.32174	6.63907
STAM	Q92783	6.55381	6.08653	6.50456	5.90162	6.75274	6.31999	6.00165	6.46129	6.26004	6.32497	6.51181	6.67087	6.68315	5.68445	5.68885	5.59368
IFIH1	Q9BYX4	6.72588	6.48957	6.95523	6.08313	6.22534	7.04574	6.25041	6.47566	6.58975	6.49161	6.73625	6.61747	5.42021	5.43964	5.1383	5.3158
KYAT3	Q6YP21	6.887	6.93093	6.79021	6.8689	6.90305	7.07167	6.52375	6.64107	6.35382	6.58844	5.69267	6.64387	5.46123	5.45592	5.69636	5.19413
FUCA2	Q9BTY2	6.97408	7.0155	6.36222	6.97305	6.73547	6.42088	6.38967	6.42466	6.55925	6.23458	6.55179	6.36197	5.31191	5.09878	5.27632	5.30987
RABGEF1	Q9UJ41-3	6.41332	6.34851	6.74365	6.07473	6.4883	6.53821	6.31651	6.47756	6.38032	6.61764	6.2432	6.25462	5.86838	5.62539	5.66074	5.94892
DDX52	Q9Y2R4	6.04494	6.15121	5.89566	6.68351	6.19689	5.85679	6.63631	5.85368	5.80225	5.9212	6.03654	5.81769	6.8603	6.59731	6.72157	6.92416
CHAF1B	Q13112	6.43714	6.35282	6.15711	6.67047	6.05884	5.89908	6.13332	5.80038	6.28191	6.08538	6.32579	6.31317	6.22741	6.06525	6.48896	6.70298
SOD2	P04179	6.16681	4.98801	5.68686	5.17261	6.2185	5.20422	5.80261	6.50849	5.42655	6.33392	5.87539	6.71119	7.05762	7.38619	7.85786	7.60318
ATL2	Q8NHH9	6.37234	6.43829	5.99123	6.54417	6.06816	6.33447	6.49925	5.92868	6.03405	6.12325	6.02188	6.06683	6.24117	6.16793	6.42214	6.74616
KIF16B	Q96L93	5.98231	6.42666	6.46129	6.10007	6.24449	6.27696	6.19418	6.14249	6.3095	6.38784	6.01196	5.8778	6.52031	6.36909	6.41015	6.2849
FNBP4	Q8N3X1-2	6.20006	6.55267	5.99394	6.49676	6.12152	6.13646	5.89917	6.80894	6.09689	6.30697	6.03744	6.23644	6.67407	6.17312	6.47659	6.50896
PITPNM1	O00562	6.13854	6.43841	6.19226	5.93965	6.25716	6.27741	5.8878	6.01654	6.16755	6.16196	5.63005	6.0049	6.96419	6.4668	7.00142	6.45536
NQO1	P15559	6.21177	6.08615	6.64743	5.59394	6.51546	6.85597	6.64718	6.95672	6.45799	6.50828	6.19489	5.9143	5.9312	6.20237	5.80009	5.47626
NAXE	Q8NCW5	6.21428	4.81612	5.73189	4.94785	5.9084	5.28948	5.84728	6.76345	5.11101	6.41521	5.62891	6.79002	6.44063	8.02806	7.88094	8.18647
RNF216	Q9NWF9-1	5.83722	6.06466	6.85361	5.94142	5.90962	6.44459	6.30626	6.48325	6.31406	6.41846	6.92965	6.67271	5.81051	5.98266	5.72596	6.30537
DHRS7B	Q6IAN0	6.27157	6.22889	5.78895	6.82742	6.33183	5.93102	6.5485	5.83912	6.12764	5.94622	5.92949	5.84782	6.65954	6.31418	6.80956	6.59825
HPRT1	P00492	6.25972	6.09292	6.95225	5.74741	6.56407	6.64081	6.26326	6.866	6.48177	6.42731	6.64635	6.61273	5.82419	5.65916	5.40814	5.55589
NADK2	Q4G0N4	6.49438	6.51044	5.76966	6.70473	6.25064	6.25368	6.1508	6.07183	6.24491	6.10956	5.76075	5.77691	6.42035	6.50136	6.53259	6.44741
VPS13B	Q7Z7G8	6.70077	6.73939	6.76902	6.90419	6.17374	6.17824	6.60464	6.36858	6.78627	6.47464	6.44921	6.6596	5.24414	5.27226	5.1431	5.53223
DNAJC1	Q96KC8	6.31522	6.69434	6.15236	6.98546	6.24541	6.18566	6.45817	5.98717	6.03364	6.23725	6.12663	6.06315	5.99309	6.08536	6.289	6.19308
PRR11	Q96HE9	7.00199	7.08239	6.48214	7.33597	6.45116	6.14275	6.78739	6.40868	6.71591	6.58853	6.28282	6.535	4.76291	5.01203	4.85467	5.55567
OAT	P04181	5.64441	6.54534	5.66325	6.30341	6.60039	6.31315	6.6866	6.21454	5.96945	6.02157	5.91272	6.00671	6.71342	6.43867	6.84681	7.01954
ZYG11B	Q9C0D3	6.002	6.25376	6.41314	6.11939	6.01568	6.04413	6.15383	6.2811	6.09108	6.15036	6.16289	6.44925	6.19532	6.57313	6.53945	6.55551
PDK2	Q15119	6.67782	6.71222	5.95372	6.49942	6.44184	6.23958	6.3804	6.07288	6.26504	6.4831	6.48453	6.07121	6.16713	5.68614	5.92196	5.94303
COG6	Q9Y2V7	6.18994	6.02532	6.30434	6.51656	6.11631	6.42718	6.07386	6.33932	6.31676	6.61677	6.31722	6.17686	6.3033	6.4502	6.03432	5.79171
CHMP1A	F5H875	5.98727	6.13143	6.87968	6.23544	6.09214	6.57018	6.2286	6.59428	6.61347	6.52241	6.57269	6.73328	5.42645	6.2037	5.39967	5.80931
GNA13	Q14344	6.63879	6.81047	6.01606	6.71033	6.35816	6.21682	6.5214	6.33336	6.33542	6.28508	5.91486	5.78755	5.889	6.02628	5.96781	6.18862
GLE1	Q53GS7	6.40967	6.22902	6.20097	6.51368	6.20975	6.25303	6.1563	6.28088	6.30646	6.55652	6.33706	6.16947	6.51279	6.03606	6.27917	6.14913
JPT2	Q9H910-3	6.45849	5.4717	7.20175	5.29821	6.79781	6.07308	5.44491	7.04351	6.54104	6.57982	7.00824	7.27282	6.11712	5.76658	5.25473	5.67018
PRCP	P42785	6.40712	6.32706	6.1097	6.50625	6.81711	6.42646	6.02102	6.1711	6.39666	6.48206	6.20971	5.95519	6.26017	5.91444	5.89077	6.10519
GTF2B	Q00403	5.83309	5.79955	5.94901	5.55169	5.96997	6.14912	5.80869	6.0079	6.14504	6.54865	6.31995	6.17788	6.64654	7.20575	7.03271	6.85446
CMSS1	Q9BQ75	6.08532	6.16313	5.95107	6.40377	6.53654	6.31241	6.14947	6.26212	6.1519	5.89737	5.70093	5.73303	6.72706	6.54148	6.78606	6.59836
DCTN4	Q9UJW0	6.02994	6.02532	6.9283	6.35332	6.01221	6.61279	6.30048	6.49736	6.42293	6.54127	6.71279	6.57718	5.58357	6.08483	5.79245	5.50604
TBC1D1	Q86TI0	6.58893	6.20748	6.98309	6.1984	6.30093	6.66631	6.45098	6.39456	6.64943	6.63729	6.60253	6.40304	5.60083	5.16666	5.53678	5.61276
SAP18	O00422-2	6.19762	6.27359	5.93674	6.39767	6.25743	6.13977	6.19634	6.20924	6.34946	6.00129	6.38551	6.51075	6.72003	5.91741	6.27088	6.23627
GTF2F1	P35269	5.4719	5.78669	6.25739	5.39826	5.84359	6.35029	5.89722	6.01496	5.94957	6.2774	6.70467	6.28492	6.84395	7.17013	7.02934	6.71971
SPAST	Q9UBP0	6.26702	6.22843	6.88451	6.13548	5.8476	6.50346	6.24958	6.40523	6.3987	6.52952	6.49592	6.4157	5.68189	6.22149	5.84842	5.88703
MYO19	Q96H55	6.43749	6.58321	6.54831	6.90472	6.19861	6.18923	6.2855	6.2385	6.22747	6.05653	5.98401	5.79562	6.35107	5.99908	6.10026	6.10039
CPNE1	Q99829	6.31646	6.05778	6.71691	5.74418	6.20851	6.75368	6.06349	6.72351	6.3268	6.39923	6.63013	6.44655	5.95751	6.07027	5.96167	5.62333
EARS2	Q5JPH6-2	6.60959	6.48525	5.935	6.77146	6.62759	6.03607	6.30681	6.36791	6.20318	5.94936	5.86435	6.06135	6.69704	5.69342	6.14308	6.24854
ORC2	Q13416	5.71175	5.96191	6.2349	6.04712	6.52883	6.48708	6.49861	6.22411	6.22965	5.97759	6.55009	5.95608	6.53231	6.40908	6.48284	6.16805
SPAG5	Q96R06	6.81843	6.4376	6.81508	6.21806	6.42172	6.44494	6.31264	6.68099	6.72395	6.7508	7.16207	7.02453	4.95765	4.9946	4.96108	5.27586
KPNA3	O00505	5.84963	6.01928	6.139	5.87322	6.12785	6.25169	5.97998	6.05198	6.1475	6.25821	6.16964	6.4934	6.36369	6.49929	6.78907	6.98658
RPUSD3	Q6P087	6.44495	6.39513	5.90369	6.75277	6.03168	6.30795	6.35589	6.2381	6.15431	6.39262	5.88747	6.48046	6.11118	6.13049	6.07501	6.33832
PLD1	Q13393	6.42028	6.65669	6.32047	6.58988	5.97003	6.24856	6.22132	6.03485	6.32787	6.60543	6.43471	6.30913	5.98795	6.02254	6.04301	5.8073
CDKN2AIP	Q9NXV6	6.11771	6.0627	5.79682	6.35569	6.29139	5.95077	6.2737	5.898	6.29015	6.06239	6.05184	5.80093	7.1401	6.48052	6.72242	6.70487
PCDHGA3	Q9Y5H0	6.34509	6.41289	5.93327	6.47121	6.3921	6.30674	6.38082	6.09962	6.14114	6.37218	6.18756	6.2974	6.23822	6.20503	6.06829	6.14845
USP33	Q8TEY7	6.96327	6.99362	6.65145	7.21726	6.23834	6.17552	6.68907	6.23663	6.76427	7.13334	6.68405	6.76981	4.57743	4.55583	4.96017	5.38994
EIF6	P56537	6.0531	6.15613	6.23943	5.83928	6.28183	6.48135	6.12124	6.58028	6.54837	6.34051	6.45475	6.54082	6.31531	6.08575	5.95221	6.00964
WDR48	Q8TAF3	5.97366	6.04757	5.99462	6.2896	6.25952	6.26972	6.00496	6.13726	6.30515	6.22469	6.17409	6.29785	6.41106	6.48838	6.52248	6.6054
FAM207A	Q9NSI2	6.27904	6.28944	6.18623	6.74583	6.32447	6.14546	6.86633	6.24992	6.40068	6.19063	6.21712	6.1253	6.07239	5.75581	6.09932	6.05205
CHP1	Q99653	6.40764	5.48219	5.7366	5.53894	6.40947	5.83556	6.22978	6.47502	5.76566	6.68111	6.06366	6.51511	6.44453	6.93882	6.86279	6.61311
ICAM1	P05362	6.01272	5.94322	5.68128	6.34386	6.26597	6.1778	6.19536	5.558	5.64844	5.94345	5.88106	5.87022	7.37442	6.75267	6.94785	7.40367
QTRT1	Q9BXR0	6.40679	6.26792	6.41557	6.0085	6.23115	6.48727	6.20745	6.50572	6.53527	6.41982	6.3499	6.53161	5.94029	5.96336	5.92854	5.80085
CEMIP2	Q9UHN6	6.26169	6.5214	6.34766	6.44933	6.25451	5.875	6.47432	6.15609	6.26558	6.48057	5.91564	6.05555	6.31107	6.12163	6.42544	6.08452
FASTKD5	Q7L8L6	6.61147	6.53671	5.71846	6.88654	6.51577	6.06009	6.44991	5.90991	6.16241	6.1318	5.8426	5.94021	6.41787	6.1392	6.42409	6.25297
ELMOD2	Q8IZ81	6.2944	6.57919	5.79507	6.6182	5.93987	6.07288	6.47247	5.94003	5.86027	6.03404	6.05257	5.99076	6.56265	6.33754	6.82797	6.6221
RAB34	Q9BZG1	6.28656	6.55426	5.58677	6.3403	6.12314	6.36775	6.1172	6.51068	6.1436	6.28781	5.73809	6.08161	6.36323	6.53566	6.41005	6.55328
AFAP1	Q8N556-2	6.11938	6.33814	5.91008	6.54601	5.95666	6.13492	6.22388	5.89344	6.19738	6.13701	5.96399	6.10761	6.65237	6.17238	6.7039	6.94287
CERT1	Q9Y5P4-3	6.26748	6.40713	6.47349	6.11661	6.14545	6.83642	6.19782	6.98236	6.21627	6.49506	6.3227	6.54406	5.92961	6.16793	5.40896	5.48866
KDSR	Q06136	6.24683	6.58954	6.05211	6.37648	6.15858	6.12473	6.7149	5.34407	6.13453	5.81397	5.67642	5.6474	6.80305	6.20427	7.33695	6.75018
GK	P32189	6.40017	6.21457	6.19161	6.42294	6.21036	5.82974	6.42625	5.94911	6.25524	5.94391	6.39066	6.10992	6.54969	6.25624	6.65798	6.19161
MTO1	Q9Y2Z2-6	6.87193	7.08608	6.56803	7.48313	6.15921	6.17612	6.08009	5.7549	6.98639	6.56836	6.62302	6.55958	5.18092	5.12637	5.29828	5.47759
AKR1B1	P15121	5.40934	5.87888	6.34453	5.68835	6.17994	6.81057	6.40357	6.91538	6.41354	6.67802	6.6152	6.68902	5.91045	5.96904	6.22412	5.87006
USP3	Q9Y6I4	5.84548	6.38212	6.54003	6.13873	6.20459	6.05126	6.25365	5.67934	6.21468	5.90286	6.49404	6.4116	6.2938	6.30198	6.53708	6.74876
SUMF2	Q8NBJ7	6.10274	5.52962	5.95788	6.0544	6.77515	5.95722	6.07232	6.65929	6.10578	6.53357	6.10869	6.52412	6.59633	6.06861	6.4527	6.50157
LIMS1	P48059-3	6.0608	6.2221	6.68384	5.94973	5.98781	6.72686	6.07722	6.17415	6.26976	6.69766	6.13876	6.44438	5.98404	6.28893	6.15363	6.14032
RTN1	Q16799	6.34679	6.6505	6.02671	6.46934	6.41269	6.48133	6.58477	6.10336	6.45038	6.38099	6.03972	5.6983	6.08048	5.96018	6.42805	5.88641
TRIM32	Q13049	6.27246	6.11226	6.62519	6.24915	6.10338	6.25141	6.36108	6.22403	6.34865	6.37765	6.50819	6.41497	5.83609	6.23081	6.28336	5.80132
TP53BP2	Q13625-3	6.04707	6.579	6.47495	6.45394	6.4131	6.37743	6.15423	6.27177	6.30596	6.26351	6.49004	6.53594	6.06248	5.96229	5.81931	5.78898
RLF	Q13129	6.28281	6.72938	6.0453	7.18672	6.06952	6.14843	6.55924	6.25294	6.15675	6.4303	6.03421	5.87415	6.12674	5.95447	6.21164	5.93739
TMED9	Q9BVK6	6.25043	5.79864	5.96641	6.16234	5.87221	6.10283	6.39237	6.40145	5.72182	6.01998	6.09749	6.25125	6.77663	6.60226	6.63734	6.94657
CCNH	P51946	5.92588	6.17352	6.35962	6.19421	6.17983	6.28393	6.07207	6.06575	6.06952	6.55332	6.54812	6.39044	6.32926	6.2732	6.38466	6.19667
NEDD1	Q8NHV4-3	6.08819	6.24262	6.55784	5.92036	6.34558	6.57197	6.0901	6.40912	6.4313	6.44322	6.42236	6.31503	5.98499	6.24044	6.18384	5.75305
PYCR1	P32322-3	6.49744	6.34126	5.78216	6.51106	6.63789	6.33647	6.53985	5.93345	5.83009	6.05351	6.19964	5.92975	6.57749	6.06734	6.4608	6.3018
HAT1	O14929	5.54964	5.74606	6.24906	5.50924	5.81398	6.19488	6.18658	5.73007	5.68854	6.20341	6.27788	5.92183	7.17904	7.16916	7.6687	6.91194
SCYL1	Q96KG9	5.90746	6.41208	6.62911	5.91053	5.93312	6.49469	6.17502	6.46881	6.22815	6.40523	6.71384	6.88141	5.85985	6.05834	6.0137	5.90867
SEMA4B	Q9NPR2	6.14298	6.22934	5.83185	6.27961	6.07409	6.3498	6.25503	6.47587	5.99847	6.63312	6.28445	6.32546	6.0951	6.36834	6.13174	6.52475

CBX3	Q13185	6.41702	5.87388	6.27606	6.04935	6.36988	5.91129	6.28051	5.80082	6.55482	6.02094	6.56374	5.83548	7.00686	5.7345	6.78876	6.51609
TGS1	Q96RS0	6.24875	6.20412	6.25126	6.0569	6.11297	6.35487	6.11786	6.16423	6.35287	6.09229	6.10668	6.24013	6.2045	6.28371	6.54606	6.66279
WLS	Q5T9L3-2	6.80583	7.07524	6.68283	6.99208	6.19236	5.93662	6.69926	6.08878	6.42311	6.35259	6.08983	6.19914	5.39259	5.60962	5.76536	5.69476
VAPB	O95292	6.41764	5.2223	6.03793	5.63417	6.23787	5.18325	5.77607	5.97809	5.74965	6.39508	5.90209	6.71552	6.98054	6.8751	7.22714	7.66758
TIPRL	O75663	6.08731	6.03423	6.89976	6.16592	6.13901	7.01411	6.18414	6.85406	6.23246	6.63649	6.56753	6.9189	5.32245	5.7444	5.44255	5.75666
NT5DC3	Q86UY8	6.64012	6.63014	5.89981	6.44374	6.25053	6.09366	6.38436	5.93207	6.1843	6.04137	5.81353	5.7571	6.69033	5.9007	6.61434	6.7239
GLA	P06280	6.65004	6.30853	6.16495	6.34449	6.72026	6.34496	6.43695	6.16838	6.36084	6.13916	6.17387	5.93755	6.61587	5.74572	6.05746	5.83096
NIPSNAP1	Q9BPW8	6.22405	6.80848	5.1434	6.84008	6.80132	6.9344	6.00165	6.96875	5.85207	5.80082	5.90623	5.66886	6.16197	6.43916	6.59392	5.85482
BARD1	Q99728	6.44107	6.63278	5.8988	6.82818	6.42793	6.0263	6.39836	5.96796	6.39233	6.1378	5.90489	5.8705	6.33153	6.2085	6.3654	6.16769
ARF4	P18085	6.11408	6.16385	6.55668	6.21709	5.90868	6.32714	6.48794	6.16374	6.4355	6.58556	6.29902	6.61561	5.87255	6.07645	6.15023	6.02589
NUDT9	Q9BW91	6.35695	6.57437	6.01335	6.40201	6.25994	6.45058	5.90252	6.65615	6.11963	6.2781	6.19845	6.36434	6.50748	6.05215	5.776	6.08796
ACBD3	Q9H3P7	6.29977	6.31065	5.81294	6.32478	6.33741	6.29536	5.79603	6.02611	5.84452	6.459	6.59186	6.23609	6.91579	6.64692	6.22809	5.87467
PREPL	Q4J6C6	6.39999	6.40466	6.77021	6.42148	6.52846	6.71942	6.53817	6.61572	6.71849	6.70944	6.68585	6.36737	5.45718	5.09682	5.40267	5.16407
BRIP1	Q9BX63	6.72978	7.03676	6.77028	6.72382	6.27473	5.923	6.04776	6.28636	6.66632	6.74511	6.38078	6.81806	5.69535	5.18936	5.3526	5.35992
LSM14A	Q8ND56-2	6.32933	5.97166	6.55175	6.06561	6.30432	6.34438	6.22334	6.33775	6.87591	6.6671	6.74444	6.51528	5.69341	5.9664	5.57075	5.83856
TMX1	Q9H3N1	6.47099	6.44936	5.89553	6.3379	6.44451	5.84563	6.58014	5.93367	6.14304	5.91498	6.05576	5.54157	6.72203	6.43035	6.52304	6.7115
PPIH	O43447	5.77083	5.45577	6.11453	5.56559	6.52235	6.24031	5.86487	6.66401	6.07812	6.44612	6.49474	6.90924	6.30779	6.54545	6.46416	6.55613
MFAP1	P55081	6.50904	6.52998	6.1108	6.81962	6.23842	5.99801	6.31137	5.81923	6.09362	5.9541	5.80487	6.13787	6.55623	6.25516	6.49609	6.36557
SHC1	P29353-6	6.59908	6.28253	6.73038	6.32931	6.19771	6.74195	6.08899	6.33147	6.3561	6.22199	6.54782	6.73506	5.76957	5.66822	5.81871	5.5811
NSMCE3	Q96MG7	5.99371	6.02537	5.9869	6.49958	6.29791	5.96964	6.15033	5.97191	6.45182	6.14802	6.29933	6.36501	6.69407	6.16924	6.61807	6.35911
KIRREL1	Q96J84-2	6.48289	6.27053	6.07038	6.52431	6.21309	6.09726	6.5864	6.01998	6.32253	5.99043	6.03863	6.08343	6.61494	6.26562	6.17622	6.24337
CCNT1	O60563	6.03958	6.37311	5.93728	6.33723	5.83936	6.17385	6.58957	5.7599	6.0759	6.12572	5.87514	5.99087	6.29315	6.80665	6.92096	6.86173
STK38	Q15208	6.16535	6.43142	6.95834	6.24273	6.13089	6.42903	6.22319	6.74028	6.4217	6.46486	6.79385	6.65541	5.3496	5.74938	5.71772	5.52625
CHEK1	O14757	6.59852	6.18228	7.31837	6.36383	6.18794	6.59333	6.60942	6.66059	6.59238	6.60468	6.67501	6.8607	5.10778	5.35987	5.05212	5.23318
MINPP1	Q9UNW1	6.1129	6.23768	6.11111	6.59077	6.04573	6.05473	6.20243	5.50559	6.12185	6.2436	6.21994	6.09345	6.71958	6.34482	6.5464	6.84943
VPS26B	Q4G0F5	5.89722	5.97572	6.34407	6.20645	6.2394	6.51864	5.86323	5.85628	6.43483	6.42973	6.79113	6.35347	6.50285	6.36218	6.15549	6.0693
RTCA	O00442-2	5.99629	6.07979	6.55793	5.8499	5.56786	6.48215	6.20547	6.4906	6.17054	6.29393	6.24952	6.64089	5.93956	6.75821	6.29063	6.0267
SSU72	Q9NP77	5.82972	5.84548	6.41617	5.92555	6.06115	6.26657	5.84194	6.19804	6.16683	6.44456	6.51127	6.43597	6.49006	6.39135	6.32091	6.85444
PRPF39	Q86UA1	5.39642	5.93438	6.25249	5.45148	6.0614	6.06081	5.71758	5.90279	5.92294	6.09552	6.02194	5.58825	7.76285	6.85508	7.85585	7.1202
GOLM1	Q8NBJ4	6.74399	6.33873	6.2762	6.42885	6.83049	6.30976	6.62836	6.31246	6.33256	6.37931	6.60001	6.35084	5.85607	5.59001	5.42561	5.59675
STXBP2	Q15833-3	6.38549	6.6695	6.34881	6.10926	6.0811	6.62572	5.89005	6.23947	6.17516	6.52675	6.29539	6.04036	6.18853	6.0324	6.44481	5.94718
PPME1	Q9Y570-4	6.11888	5.91296	7.06197	6.0406	6.34872	6.45954	6.15413	6.57295	6.15845	6.19527	6.69508	6.41987	5.91767	6.0081	6.16504	5.77077
CDK7	P50613	5.77395	6.1604	6.36841	6.01874	5.9609	6.40643	6.15933	6.00705	6.11202	6.31352	6.20861	6.22605	6.52279	6.36187	6.63027	6.76965
STXBP5	Q5T5C0	6.06061	6.23245	6.44468	5.84659	6.08127	6.87171	6.38581	6.42847	6.36326	6.39574	6.65112	6.29406	6.15825	6.22537	5.97473	5.58587
UQCRFS1	P47985	6.94815	6.28032	6.05233	6.21887	6.25329	5.78168	6.1881	5.77432	5.65968	5.90203	5.87482	5.66523	6.12902	7.19285	6.82836	7.25096
BLVRA	P53004	6.32474	6.11614	6.70145	5.78392	6.40708	6.85675	6.42362	6.75514	6.23804	6.38079	6.44322	6.42377	5.76284	6.06027	5.70517	5.61705
MANF	P55145	5.97849	5.45994	6.14911	6.29548	6.57618	6.55164	6.2407	6.27392	6.60344	5.9006	6.39299	6.19398	7.18782	6.38057	5.7508	6.06436
NUP43	Q8NFH3	5.83918	5.98074	5.8784	5.98652	6.73537	6.25161	6.37983	7.15735	6.63529	6.08083	6.46047	6.85422	5.82182	5.8598	6.04398	6.03459
HPF1	Q9NWY4	6.08042	6.22545	6.86326	5.67394	6.03503	6.32477	5.95899	6.20267	6.02133	6.30414	6.45242	6.32071	6.10052	6.79639	6.50601	6.13395
LMTK2	Q8IWU2	6.17373	6.04572	6.13204	6.18224	6.04165	6.01134	5.93263	5.74222	6.37024	6.4501	6.37355	6.44217	6.65968	6.29908	6.67888	6.46474
NOA1	Q8NC60	6.45891	6.5237	6.42238	7.08469	6.26438	5.60605	6.78763	6.00636	6.61494	5.78915	6.43882	6.36134	6.07934	5.63975	5.93631	5.98625
KBTBD6	Q86V97	5.55165	6.01457	6.03338	5.73318	5.94349	6.14159	5.97615	6.12295	6.08291	6.51555	6.4225	5.96859	6.82501	6.97227	7.11939	6.57684
RTTN	Q86VV8	6.25589	6.03336	6.41972	6.36517	6.17935	6.79632	6.51345	6.5128	6.68058	6.25297	6.18957	6.44067	6.24103	6.02051	5.49148	5.60715
FIS1	Q9Y3D6	6.13837	5.72888	5.89276	5.7487	6.33337	5.53119	5.83103	6.3078	5.57909	6.21561	5.94305	6.51688	6.74554	6.9975	7.12926	7.24036
SRSF11	Q05519	6.15799	6.46162	6.02993	6.51091	6.17508	5.95459	6.19922	5.7878	5.98075	6.2757	6.30954	5.91474	6.31984	6.35198	6.77082	6.79949
TRIM4	Q9C037	6.55617	6.70566	6.43571	6.77972	6.20537	6.09619	6.44095	6.07436	6.24712	5.89237	6.00168	5.87333	6.34438	5.96966	6.3801	5.99723
NDUFAF2	Q8N183	6.617	6.32004	6.08587	6.69695	6.26363	5.97626	6.19259	6.30259	6.4002	6.15339	6.05207	6.11023	6.78627	5.93048	5.99707	6.11535
QRICH1	Q2TAL8	5.51926	5.7699	5.87883	5.14927	5.71032	6.08781	5.34531	6.18378	5.9696	6.38933	6.14675	6.21507	7.40079	7.18116	7.68097	7.37186
MRPL28	Q13084	6.45316	6.45118	5.9983	6.76321	6.54626	6.33535	6.31087	5.9643	6.12859	6.44377	6.0652	6.35156	6.14904	5.98833	5.99501	6.05586
SEC11A	P67812-3	6.41566	6.37615	5.69876	6.29899	6.25774	6.10629	6.47445	5.8911	6.03839	5.99258	6.00442	6.10005	6.82845	6.42121	6.50572	6.59004
ATP5ME	P56385	6.13947	5.62633	5.76538	5.7405	6.66079	5.79451	6.53671	6.40756	5.63637	5.97209	5.91504	6.11084	6.55379	6.88612	6.82676	7.42775
UBLCP1	Q8WVY7	6.16333	6.05374	6.33386	5.52641	6.112	6.69978	5.99626	6.32146	6.05455	6.45272	5.98391	6.24266	6.30005	6.94861	6.61853	6.19214
LIAS	O43766	6.55476	6.22494	6.10291	7.1699	6.17955	6.08486	6.47035	6.60351	6.19347	5.81709	6.16483	6.37129	6.17489	5.7548	6.14946	5.98338
BABAM2	Q9NXR7	6.14639	5.95996	6.7466	6.22245	6.49634	6.6676	6.02747	6.32299	6.23958	6.31908	6.61445	6.27487	6.09728	5.91024	5.81697	6.13771
MRPS2	Q9Y399	6.54479	6.33498	6.06445	6.49578	6.18322	6.14899	6.45026	6.16295	6.31222	5.75397	6.08024	6.06496	6.48525	6.12967	6.55869	6.2296
SPCS2	Q15005	6.44039	6.79015	5.6432	6.66186	6.51843	6.40476	6.36267	6.38746	6.06339	5.95934	5.70653	5.92292	6.46212	6.21516	6.27811	6.18349
BCAS3	Q9H6U6-8	6.12223	6.01614	6.69654	6.00837	6.36009	6.71167	6.34755	6.55676	6.35053	6.54667	6.55294	6.18364	6.00662	5.99739	6.10853	5.43431
IRAK4	Q9NWZ3	6.00559	6.15063	6.60908	5.90672	6.20897	6.89266	5.99029	6.5247	6.38125	6.78389	6.63419	6.55751	5.90863	5.86575	5.89181	5.68832
ATPAF2	Q8N5M1	6.81301	6.56626	5.64092	6.82029	6.2449	6.15273	6.34082	6.07472	5.93831	6.18553	5.67398	5.73169	6.56706	6.37528	6.57906	6.29545
GOT1	P17174	6.18722	5.88638	6.80652	5.75634	6.34325	6.93287	6.06318	6.68022	6.57343	6.27186	6.48465	6.36095	5.92395	6.13633	5.75638	5.83648
ACOT13	Q9NPJ3	6.05878	6.46024	5.85089	6.63824	6.22563	6.16717	6.29029	5.97701	5.88552	6.4528	6.246	5.93213	6.68208	6.00804	6.6771	6.44809
DIS3L	Q8TF46	6.44218	6.31052	7.05973	6.05576	6.19355	6.52795	5.96482	6.43798	6.65379	6.61071	6.53596	6.66096	5.64757	5.62904	5.5748	5.69468
SNRNP27	Q8WVK2	6.33984	6.3699	5.87864	6.1917	5.92025	6.0529	6.39165	6.24509	5.86921	5.74128	5.72726	5.66954	6.83948	6.73217	7.01763	7.01347
RPP30	P78346-2	5.96639	6.16627	6.02661	6.30911	6.16034	6.02423	6.13344	5.89943	6.33111	6.45684	6.09622	5.95368	6.86298	6.18244	6.72789	6.70302
USP32	Q8NFA0	5.88352	5.93187	6.58706	5.60871	5.8409	6.47274	6.13449	6.30348	6.41836	6.59721	6.69547	6.47019	6.36107	6.37579	6.11597	6.20317
NDUFA12	Q9UI09	6.1072	6.57467	5.77417	6.52631	6.22131	5.90053	6.52828	6.17163	6.12832	5.94518	5.90935	6.05343	6.96892	6.48753	6.36715	6.33602
PRRC1	Q96M27-2	6.07263	5.83928	5.91479	5.44934	6.13149	6.43641	5.90819	6.09022	6.17541	6.29882	6.45493	6.38422	7.02763	6.50528	6.81092	6.50045
RPL7L1	Q6DKI1	6.41334	6.33744	5.58468	6.74876	5.83879	5.83975	6.49326	6.23075	6.25967	6.10009	5.93724	6.2131	6.54321	6.26248	6.37392	6.82352
MARCHF5	Q9NX47	6.39949	6.51322	6.11704	7.10882	6.28125	6.1402	6.41578	6.09092	6.38983	6.26808	6.0838	5.99346	6.14684	5.84031	6.12681	6.08414
NIP7	Q9Y221	6.3883	6.53529	6.14314	6.94287	6.35776	6.04987	6.26935	6.01564	5.59183	6.00323	6.39103	6.22895	6.45447	6.05092	6.4257	6.15166
RPL27A	P46776	6.10981	6.1409	6.57444	5.7278	5.7355	5.99188	6.55301	6.0308	6.6456	6.29531	6.79594	6.35661	6.27939	6.10698	6.0623	6.59374
RBM7	Q9Y580	6.06772	6.47666	5.80574	6.3538	6.63374	6.01153	6.10269	6.31331	6.37718	6.22014	6.01981	5.9466	6.6998	6.27392	6.5216	6.17575
MRPS15	P82914	6.27757	6.40901	5.67606	6.88144	6.10095	5.95708	6.24954	6.07566	6.04596	6.12985	5.90337	5.99046	6.57399	6.41825	6.64419	6.66662
ANKRD11	Q6UB99	6.2339	6.08067	5.9771	6.81018	6.09804	5.82185	6.52728	6.1007	6.11595	6.35712	6.09016	6.09785	5.87618	6.65316	6.42563	6.73424
NSD3	Q9BZ95	6.26205	6.61218	6.10188	6.86319	5.98191	6.01064	6.38153	6.10796	6.17388	6.2413	5.7876	6.28851	5.99466	6.72975	6.1355	6.32747
WBP2	Q969T9	6.39619	5.84544	7.14191	6.17731	6.42729	6.48038	6.02918	6.75401	6.94243	6.39899	7.06194	6.91166	5.61362	5.56556	5.24368	5.01039
EHBP1	Q8NDI1	5.7267	6.09401	6.42865	5.84838	6.04163	6.27428	6.23383	6.34329	6.39823	6.55193	6.48644	6.22683	6.45861	6.27552	6.28036	6.33133
DFFA	O00273	6.17183	5.86483	6.88621	5.67733	6.16722	6.3622	5.92792	6.80986	6.39583	6.32129	6.55479	6.71408	6.3975	6.03354	5.97979	5.73579
PCGF2	P35227	6.48517	6.77651	6.06321	6.39137	6.20734	6.13081	6.0991	5.8943	6.48158	6.34073	6.26866	6.04689	6.43274	6.0129	6.13611	6.23256
TSPYL5	Q86VY4	6.08054	6.36862	6.34402	5.81084	6.06407	6.14179	6.09916	6.25714	6.33466	6.51876	6.58746	6.58792	5.96629	6.3109	6.25422	6.27359
FGFR1	P11362-21	6.40638	6.58774	6.55378	6.67908	6.5469	6.13767	6.15542	6.34639	6.43671	6.38977	6.52721	6.29093	5.83294	5.78725	5.65455	5.66727
DUT	P33316	6.73764	6.1202	7.95637	6.31849	6.57989	6.42123	5.77076	6.27776	6.32261	6.3057	6.66327	6.76831	5.76417	5.45117	5.11419	5.42821
BUD13	Q9BRD0	6.34709	6.42237	5.86338	6.82432	6.09654	5.84462	6.15925	6.13966	6.23397	6.03886	6.17684	5.9756	6.55969	6.24818	6.40531	6.66432
CNOT3	O75175	5.95552	6.12898	6.33049	5.94539	5.86973	6.2326	6.22908	6.50833	5.73785	6.10275	6.36944	6.2253	6.4228	6.69403	6.77108	6.47664
VWA5A	O00534	5.68513	6.32057	6.14721	5.66935	6.30322	6.70725	6.02664	6.82548	6.01836	6.68696	6.42566	6.30262	6.02185	6.8075	5.65095	6.40125
CARS2	Q9HA77	6.06671	6.35328	5.95701	6.57539	6.15585	6.30133	6.04694	6.46738	6.35813	6.11408	6.06614	5.7865	6.45984	6.15866	6.47676	6.65599
PHKA2	P46019	6.15095	6.46424	6.81509	6.21916	6.25366	6.72597	6.34487	6.84856	6.40401	6.58208	6.81258	7.05702	5.31292	5.5202	5.21429	5.27439
HPS3	Q969F9	6.42075	6.35775	6.98183	6.2511	6.13284	6.66014	6.05404	6.35006	6.3904	6.86987	6.63018	6.59505	5.68783	5.64677	5.39747	5.57392
ADGRL2	O95490-6	5.99422	6.25197	5.88035	6.40361	5.89608	6.09219	6.17947	6.02666	6.13401	6.21281	6.02881	6.10909	6.64398	6.47309	6.8853	6.78836
FANCA	O15360	6.63379	6.58684	6.66187	6.66672	6.45015	6.30581	6.54046	6.91118	6.46662	6.89236	6.66328	6.77393	5.11876	5.30788	4.94468	5.07569
MYLK	Q15746	6.43502	6.41584	6.43227	6.09409	5.65	5.90042	5.6264	5.63559	6.26317	6.39265	5.79685	6.16116	6.48133	6.11887	7.43062	7.16571
STK24	Q9Y6E0	6.23903	6.27941	6.75513	5.79865	6.3705	6.64579	6.09335	6.34594	6.36973	6.43338	6.43919	6.41692	6.31015	5.93679	5.90866	5.65739
METTL1	Q9UBP6	5.11675	5.58329	5.76459	5.08866	5.6124	6.38885	5.4476	5.70323	5.63756	5.98612	6.32471	6.04642	7.12775	8.24815	8.28668	7.63723
MAP7	Q14244-7	5.98533	6.13376	6.1109	5.6474	6.49515	6.4013	6.03587	6.48579	7.11697	6.55567	6.54357	5.94561	6.34401	6.29992	6.02924	5.8695
HSPBP1	Q9NZL4-3	6.39467	6.18089	6.90637	5.76967	6.52682	6.68673	6.08986	6.80471	6.11807	6.46093	7.16462	6.1869	5.53719	6.06215	5.5614	5.54902
HMGCL	P35914	6.35251	6.3284	5.73727	6.16635	6.67369	5.97576	6.29261	5.93164	6.08316	5.95828	6.11234	6.19197	7.06812	6.19784	6.63924	6.29085
PRKAR2B	P31323	6.27451	6.55877	6.18287	6.4785	6.42789	6.62682	6.07203	6.68668	6.28641	6.02591	6.32551	6.11577	6.07717	5.86157	6.20095	5.79861
PPP6R2	O75170	6.45576	6.37683	7.14369	6.04888	6.61388	6.76692	6.05604	6.43902	6.7105	5.88713	6.41356	6.40313	5.84243	5.53566	5.65742	5.64914
ZNF532	Q9HCE3	6.46208	6.49379	6.05488	6.67901	6.2938	6.12488	6.45192	5.80399	6.40012	6.46389	6.08149	5.85401	6.11175	5.8822	6.51139	6.33079
DPP7	Q9UHL4	6.74841	6.45709	6.12463	6.37271	6.49801	6.18677	6.26849	6.38833	6.24214	6.21137	6.23014	6.16558	6.3599	5.74055	6.11878	5.8871
PPP5C	P53041	6.35359	6.3411	6.88549	6.05599	6.49864	6.88674	6.33552	6.52107	6.33899	6.55117	6.10037	6.37446	5.71227	5.92628	5.50562	5.61271
PHF21A	Q96BD5-3	6.57565	6.52794	5.91696	6.49611	6.29701	6.17156	6.29072	6.12056	6.09006	6.46132	6.03382	6.16947	6.44381	6.22868	6.11042	6.06589
CHD9	Q3L8U1	6.25826	6.27498	6.48566	6.49673	6.21334	6.29717	5.92666	6.2162	6.14276	6.25367	6.21696	6.20348	6.08423	6.24636	6.25051	6.43303
CDAN1	Q8IWY9	6.12331	6.53115	7.30098	6.17851	6.32504	6.40256	6.14548	6.29927	6.35224	6.45643	6.59893	6.55649	5.67186	5.25282	5.57496	6.22998
L2HGDH	Q9H9P8	6.66556	6.68089	5.9483	6.70715	6.49005	5.94787	6.26983	6.34282	6.15655	5.95364	5.79995	5.75829	6.3205	6.19627	6.65392	6.10839

ZFYVE1	Q9HBF4	6.61979	6.22333	6.63667	6.04687	6.34053	6.69211	6.13903	6.30436	6.4928	6.37188	6.16329	5.86239	6.17312	6.17595	6.05552	5.70233
SNU13	P55769	5.99878	5.92585	5.70245	5.95926	6.48462	5.89518	6.20944	6.60483	6.08522	6.27898	6.43366	6.60269	6.53042	6.44961	6.44086	6.39816
EXT2	Q93063-3	6.64235	6.64145	5.88626	7.03046	6.47641	6.17098	6.93474	6.50513	6.23904	6.21927	6.01643	6.13249	5.93125	5.68047	5.54908	5.9442
MIPEP	Q99797	6.21399	6.23699	5.74181	6.69622	6.83851	6.52416	6.55169	6.3775	6.25091	5.94269	5.68236	5.96598	6.56226	6.06131	6.21262	6.14098
GORASP2	Q9H8Y8	5.79114	5.6793	5.37838	5.59753	6.12422	6.15856	6.16192	6.85118	6.23707	6.73938	6.25178	6.46045	6.01701	6.62972	6.89651	7.02585
TBC1D2	Q9BYX2	6.51732	5.9106	7.27133	6.25452	6.6188	6.52765	6.41858	6.47236	6.68433	6.31553	6.8552	6.82396	6.52475	5.05949	5.5009	5.24469
DCPS	Q96C86	5.63903	5.48249	5.97092	5.02018	5.88282	6.25644	5.35706	6.34826	5.65113	6.2541	6.30643	6.34267	6.94818	7.943	7.61769	6.97961
BICD1	Q96G01	6.26319	6.2384	6.46764	5.94154	6.17128	6.37373	6.17475	6.15973	6.36683	6.34376	6.49504	6.26461	6.48474	6.09166	6.03648	6.12661
LRCH3	Q96II8-2	6.65155	5.94348	6.79615	6.4085	6.20867	6.29668	6.06654	6.58909	6.41581	6.16577	6.73805	6.80697	6.12448	5.65032	5.62058	5.51738
SP110	Q9HB58-6	6.51659	6.62158	5.92142	6.88784	6.06614	6.03609	6.31651	6.14888	6.28133	6.2082	6.03068	6.05216	5.75953	6.18771	6.72785	6.23749
IL13RA2	Q14627	6.64168	6.68101	6.0141	6.8676	6.57339	6.13245	6.83735	6.05932	6.12618	6.40603	6.41063	5.77896	5.84626	5.36666	6.11278	6.1456
UBXN7	O94888	5.78519	6.09053	5.90418	6.52843	6.33352	6.49437	5.50432	6.68948	5.95758	6.29032	6.55777	6.47841	6.71075	6.88887	6.17016	6.51612
CNTNAP1	P78357	6.34229	6.70236	6.39229	6.48173	6.05939	5.71064	6.20372	6.00092	5.65552	6.28679	6.15186	5.80926	6.54404	6.13086	6.84902	6.67931
MCM10	Q7L590	6.37181	6.60152	6.07998	6.54505	6.09518	6.05119	6.21716	5.93535	6.0806	6.19809	6.23075	6.31121	6.38263	6.08324	6.44375	6.37248
BPNT2	Q9NX62	6.26601	6.34869	5.81802	6.58276	6.22075	5.99181	6.12036	6.11524	6.25139	6.07027	5.94531	6.08105	6.93399	6.20371	6.68255	6.36809
TARS3	A2RTX5	6.23763	6.3956	6.95916	6.33071	6.07131	6.36094	6.5013	6.5352	6.06125	6.72589	6.62678	6.66025	5.60482	5.82252	5.41316	5.69348
MASTL	Q96GX5	5.3281	5.87755	6.52482	5.14974	5.37578	6.34811	5.51089	5.94825	6.06815	6.25342	6.52125	6.20234	6.89702	7.01929	7.60748	7.36781
SEPTIN8	Q92599	5.98189	6.2124	6.33524	5.98033	6.2667	6.62198	6.12303	6.7523	6.23601	6.43576	6.33925	6.46703	6.17888	6.21127	6.09801	5.75992
ARMC1	Q9NVT9	6.88976	6.48658	6.47511	6.30683	6.9087	6.96183	6.96668	6.71118	7.23818	6.96688	6.7261	6.65899	4.70156	4.55159	4.71519	4.73484
GABPA	Q06546	5.84909	5.76985	5.99	5.65368	5.96318	6.14934	5.59554	5.81652	6.21195	6.13343	6.38923	6.22764	7.12205	6.94906	6.94775	7.2317
ANO10	Q9NW15	6.73629	6.45345	6.0593	6.5429	6.11531	5.87257	6.05969	5.8493	6.36024	6.02672	6.23524	6.02489	6.51526	6.02448	6.67487	6.44951
ATP6V1E1	P36543	6.81518	6.60669	6.61766	6.214	6.5333	6.72258	6.43852	6.48734	6.49112	6.63632	6.74408	6.5111	5.59295	5.38331	5.11179	5.09405
MRPS26	Q9BYN8	6.66191	6.05576	5.82158	6.65542	6.35069	5.72494	6.60429	5.8833	6.32338	5.89983	6.24426	6.15109	6.50676	6.16872	6.43938	6.50871
COX6B1	P14854	6.74527	5.04637	5.55656	4.91613	6.00312	4.83931	5.04488	6.25242	4.73583	6.04938	4.8915	5.52817	5.2482	10.3345	8.57571	10.2327
HAUS5	O94927	6.88549	6.9171	7.50823	6.77329	6.37342	6.81582	6.23584	6.29527	6.67488	6.61666	7.13725	6.8176	4.64438	4.8961	4.63913	4.76953
GTPBP6	O43824	6.45915	6.55223	6.12534	7.02926	6.52032	6.56714	6.72103	6.29116	6.03089	6.15774	6.0675	5.98434	5.88823	5.70168	5.84297	6.06101
TRAM1	Q15629	6.0994	6.38062	5.85695	6.54851	5.8643	5.74262	6.33211	5.57086	6.00739	6.1154	5.72802	5.7577	6.87538	6.60492	7.41073	7.10508
LRRFIP2	Q9Y608	6.21373	6.1888	6.5268	5.58973	6.62953	6.59909	5.94992	6.15526	6.6504	6.34168	6.74017	6.30187	6.56809	6.0515	5.76599	5.72744
CNOT10	Q9H9A5-6	6.40159	6.57949	6.54661	6.20362	6.23531	6.52203	6.11727	6.30457	6.3996	6.37738	6.59917	6.42853	5.89554	5.75245	5.93263	5.70423
COG8	Q96MW5	6.23208	5.95867	6.31932	6.21198	6.60829	6.33238	6.62692	6.48138	6.51492	6.54401	6.23547	6.5383	5.99814	5.9917	5.90261	6.02381
ESPL1	Q14674	6.80231	6.6292	7.49424	7.02232	6.21018	6.88164	6.59714	6.43526	7.22393	7.19171	7.06555	7.37568	4.21487	4.21369	4.0276	4.61466
SRSF3	P84103-2	6.14277	6.063	5.26362	5.58339	6.22264	5.5589	5.78428	5.27692	5.77033	6.17362	7.18799	6.75586	8.04146	6.81677	6.76825	6.5902
AGTPBP1	Q9UPW5-3	6.57644	6.34118	6.75206	6.31011	6.40339	6.60386	6.57388	6.58346	6.40911	6.57436	6.55645	6.6552	5.43423	5.40655	5.22153	5.5982
UBE2V2	Q15819	6.21407	5.49969	6.16424	5.00655	6.59978	6.63424	5.78422	7.53783	5.61866	6.83452	6.09178	6.66604	6.18783	7.05595	5.79556	6.30903
ARMC6	Q6NXE6	6.34803	6.25135	6.86105	6.0261	6.70535	6.59885	5.93266	7.18409	6.37514	6.20838	6.77711	6.97675	5.48565	5.52292	5.17999	5.46596
ARHGEF17	Q96PE2	6.45916	6.61758	6.45548	6.47224	6.31207	6.46979	6.17342	6.20929	6.10097	6.2591	6.57551	6.41027	5.89212	5.40151	6.04521	6.1463
SLC9A1	P19634	6.35009	6.54654	6.0257	6.65782	6.09503	6.25049	6.37319	6.28819	6.13057	6.20004	6.20895	5.91487	6.28296	6.25287	6.28984	6.13285
PHAX	Q9H814	5.70393	5.65159	6.0139	5.50296	5.83991	6.30813	5.82101	6.04092	6.27049	6.2798	6.29641	6.17859	7.09367	6.71995	7.13831	7.14043
ARHGAP11A	Q6P4F7	6.76192	6.87628	6.29897	7.42589	6.45327	5.9748	6.44447	6.23743	6.06591	6.07022	5.99464	6.2335	5.48665	5.56056	6.06724	6.04824
LAMP1	P11279	6.29604	6.74898	5.9497	6.51406	6.08615	5.93068	6.16644	6.00719	6.32673	6.38329	6.24759	6.13107	6.25894	5.99408	6.73672	6.22233
FZD6	O60353	6.39308	6.42377	6.09439	6.55584	5.99791	6.56471	6.59956	6.04179	5.98902	6.42335	6.04492	5.9843	6.05345	6.31991	6.30384	6.21016
ODR4	Q5SWX8	6.3601	6.18292	6.1966	6.7643	5.92989	5.83758	6.53095	5.87502	6.00568	5.86095	6.03544	5.98761	6.79507	6.44851	6.70725	6.48214
PRR12	Q9ULL5	6.62616	6.30583	6.10447	6.76242	6.05286	5.86562	6.35411	5.74899	6.33323	6.44848	5.94453	6.16235	6.34285	6.13054	6.47818	6.33938
TMX2	Q9Y320	6.29234	6.57701	6.21717	6.77473	6.4194	5.97739	6.5958	6.10536	6.33239	5.94537	6.05428	5.94842	6.16303	5.99575	6.37231	6.22924
E9PSI1	E9PSI1	6.35542	6.64769	6.02355	6.5455	6.3144	5.99751	6.23587	6.4657	5.96825	6.17039	6.11521	6.01991	6.22763	6.16736	6.3225	6.42311
INO80	Q9ULG1	6.48348	6.54725	5.68412	6.55752	6.1756	5.77783	6.46121	6.06541	6.22053	6.20964	5.79571	5.91656	6.30655	6.50273	6.84528	6.45059
DTL	Q9NZJ0	5.59287	6.02953	6.37004	6.01971	5.52365	5.91522	6.02131	5.63417	6.27616	6.28675	6.77287	6.6673	6.74818	6.67638	6.80198	6.66388
MPP2	Q14168-4	6.22709	6.61321	6.13789	6.69942	6.01784	6.1017	6.31117	6.16394	6.13202	6.16194	6.02736	6.08656	6.46151	6.16443	6.4079	6.28603
ACY1	Q03154	6.36424	4.99785	6.6245	4.6385	6.3726	5.9862	6.5274	7.14052	5.6482	7.24862	6.47658	6.89031	6.01687	7.19924	6.58394	6.18444
BIRC2	Q13490	6.21386	6.84031	6.50462	5.70452	6.47371	6.58055	6.05663	6.62656	6.25759	6.32631	6.53947	6.76037	6.47125	6.10685	5.80661	5.7308
SLC38A10	Q9HBR0	6.37518	6.42205	5.79187	6.62131	6.18515	5.95535	6.36509	6.13346	5.76202	6.22873	6.20514	5.92092	6.58772	6.44406	6.13206	6.86988
SATB2	Q9UPW6	6.10425	6.28884	5.91742	6.81873	6.05962	6.2611	6.24187	6.16493	6.22589	6.39582	6.21157	6.21852	6.96128	5.74255	5.99711	6.3905
STK4	Q13043	6.49353	5.42035	6.90372	6.12985	6.54075	6.3868	6.5368	6.84522	6.51633	6.11531	6.99396	6.67049	5.76233	5.59494	5.52202	5.5676
BMP2K	Q9NSY1	5.99519	6.32909	6.11094	6.53656	6.32276	6.36912	6.23218	6.0774	6.71612	6.43492	6.61347	6.37041	5.91055	6.22764	5.93871	5.81495
RXRB	P28702-3	5.84288	6.6129	6.21953	6.14752	5.80145	6.15113	6.09956	6.26235	6.53744	6.47625	6.30548	6.38084	5.97158	6.58404	6.44727	6.15979
GAA	P10253	6.63738	6.88177	6.1481	6.18967	6.58787	6.35676	6.47639	6.44014	5.98033	6.02806	5.82895	5.76676	6.12492	5.98427	6.2584	6.31023
PREB	Q9HCU5	6.14535	6.27788	6.00121	6.57184	6.16252	5.92203	6.45839	5.80343	5.92028	6.17334	6.10677	5.92653	6.42548	6.49057	6.91681	6.69758
FBXO28	Q9NVF7	6.71795	6.81321	6.66595	7.38812	6.20616	5.92742	6.71109	5.95921	6.44957	6.39759	6.51394	5.6677	5.3265	5.25212	5.48423	5.60925
OSBPL10	Q9BXB5	6.42477	6.58316	6.47948	6.48135	6.37929	6.75921	6.2272	6.4477	6.2038	6.47959	6.40604	6.43586	5.53962	5.73168	5.75846	5.66279
SNAP23	O00161	6.12451	6.35408	5.77772	6.20659	5.82672	5.52414	6.05416	6.2214	6.20456	6.15425	5.88722	6.0893	6.8044	6.74893	6.99724	7.02478
PPP4C	P60510	5.6731	6.0806	6.33294	5.65988	6.0216	6.11474	6.27889	6.02986	5.81137	6.4346	5.96025	6.22917	6.60534	6.80103	6.91414	7.05251
PPP1CB	P62140	6.20054	6.05239	6.31073	6.13483	6.15987	6.40902	6.06223	6.27803	6.28005	6.378	6.30603	6.23751	6.37783	6.37532	6.18751	6.2501
UIMC1	Q96RL1	6.18429	6.11063	6.16018	6.84187	5.60207	5.81239	6.12163	5.87521	6.08068	5.97508	6.10121	6.08225	6.18101	6.90955	7.00081	6.96114
HELZ	P42694	6.36148	6.18966	6.71557	6.26471	6.25648	6.62134	6.20038	5.9131	6.43707	6.39047	6.49928	6.38873	5.7612	6.12989	6.02523	5.84541
ATP7A	Q04656-3	6.29789	6.34434	5.95095	6.13125	6.43734	6.13816	6.47985	5.81783	6.15251	6.31843	6.02933	5.91668	6.75666	6.28726	6.65661	6.28492
SMARCA4	P51532	6.17996	6.73592	5.9198	6.64972	6.13299	6.20502	6.38716	6.02331	6.04629	6.21465	5.87698	5.81257	6.41558	6.33881	6.55526	6.50599
CENPP	Q6IPU0	6.26012	6.13071	6.36859	6.86708	5.7209	6.00232	6.3389	5.68582	6.60212	6.17332	6.19604	6.46594	6.2172	6.06924	6.23248	6.66922
HTATIP2	Q9BUP3-3	6.41375	6.28708	6.18404	6.39375	6.11136	6.15281	6.31425	6.33131	6.39254	6.52364	6.65844	6.49874	6.15914	5.78892	5.95352	5.83671
ZER1	Q7Z7L7	6.3623	6.57308	6.72745	6.13363	6.39071	6.36357	5.91993	6.38604	6.44102	6.78653	6.59495	6.40389	5.61191	5.72005	5.5462	6.03874
EDF1	O60869	6.60965	6.61619	6.502	6.04075	6.6803	6.80699	6.45038	6.55513	6.50255	6.67581	6.54218	6.40148	5.69538	5.46257	5.16888	5.28975
PPID	Q08752	5.85762	6.12018	6.74042	5.65204	6.3593	6.58036	5.99771	6.61122	6.34981	6.38363	6.78821	6.47169	6.24629	6.02501	5.89816	5.91835
NDUFAF1	Q9Y375	6.56617	6.28344	6.11781	6.54841	6.43658	5.79344	6.50748	5.80503	6.06331	5.91051	6.16829	6.12408	6.92704	6.24453	6.27896	6.22491
PWWP2A	Q96N64	6.49519	6.58193	6.31096	7.18299	5.9052	5.86695	6.58415	5.46745	6.01727	6.21703	6.25303	6.20302	6.34053	6.16616	6.32854	6.0796
PDAP1	Q13442	6.2268	6.35717	6.34619	5.35787	6.47796	6.57878	6.22278	6.60664	6.49348	6.13355	5.85934	6.01473	6.33511	6.88009	6.08939	6.0201
RRP7A	Q9Y3A4	6.27198	6.34636	6.07613	6.67794	6.46536	5.90086	6.68515	6.02898	6.25097	5.94903	5.82433	5.64158	6.49747	6.10802	6.62011	6.65574
SREK1	Q8WXA9-2	5.42545	6.16461	5.43651	6.5134	5.97635	5.96663	6.31513	6.27705	5.99509	6.42098	6.24502	6.01057	5.86951	6.82313	7.14106	7.4195
UQCRB	P14927	6.08125	5.35537	5.93297	5.48926	6.72934	5.19757	5.62808	6.78083	5.37101	6.33246	5.74907	5.65908	6.75384	7.44494	7.32691	7.228
PCMT1	P22061-2	5.64926	4.54081	5.92496	4.86949	6.06535	5.44235	5.72575	7.15261	5.49642	7.15485	6.59812	7.49607	5.99253	7.3907	6.88456	7.61617
SORD	Q00796	6.37819	6.08581	6.85101	6.30115	6.00273	6.64273	5.99705	6.24332	6.2327	6.27555	6.50618	6.60295	5.99216	5.9902	5.6852	6.21307
STMN1	P16949-2	6.6257	5.38867	6.83343	4.87938	7.19385	5.93168	5.30786	7.50246	5.84706	6.62806	6.46116	7.11022	6.39711	6.39959	5.63812	5.85565
IFT80	Q9P2H3-3	6.40155	6.47446	6.35713	6.61879	6.21176	6.64791	6.38307	6.17688	6.12042	6.27548	6.1243	6.41473	5.90469	6.03107	5.80743	6.05034
CRK	P46108	5.54464	5.66887	6.35628	5.56609	5.91896	6.54078	5.86769	6.83436	5.99423	5.9683	6.93822	7.15247	6.1456	6.31858	5.78971	6.39523
ZNF207	O43670-4	5.58006	5.77006	5.95011	6.15675	6.16769	6.18888	6.28557	5.99893	6.17587	5.92998	6.00224	6.03567	6.95686	6.93363	6.96097	6.90672
RAB11FIP5	A0A1B0GTL5	6.26339	7.08293	6.11831	6.38671	6.36197	6.45676	6.48629	6.5607	6.28046	6.48777	6.29539	6.33931	5.2369	5.60364	5.95465	6.08483
RIC8A	Q9NPQ8-3	6.18225	6.05362	6.76819	6.27269	6.24813	6.69749	6.0946	6.536	6.47462	6.32906	6.62822	6.87451	5.55987	5.87705	5.39944	6.00428
HEATR3	Q7Z4Q2	5.33457	5.45789	5.98351	5.45753	5.43149	5.9018	5.78294	5.80439	5.79961	5.89759	6.2498	6.02458	7.36029	7.50142	8.04183	7.97075
HEXA	P06865	6.49091	6.2313	5.63788	5.76401	6.56743	6.0635	6.34113	6.44415	6.0094	6.27345	5.73447	5.84923	6.84699	6.60866	6.62337	6.51411
DHX32	Q7L7V1	6.40521	6.28387	6.70034	6.12932	6.63411	6.41635	6.38539	6.47748	6.57605	6.62576	6.60854	6.33414	5.58798	5.67773	5.45667	5.70106
NUB1	Q9Y5A7	6.1337	6.35523	6.60754	6.02171	5.95817	7.00925	6.05597	6.38449	6.09682	6.50773	6.31645	6.42162	6.52414	6.26845	6.14153	6.0972
DGKA	P23743	5.7037	6.1758	6.52535	5.78862	6.08721	6.62733	6.21595	6.56077	6.17726	6.70423	6.81723	6.5438	5.84692	6.01756	6.22911	5.97917
QTRT2	Q9H974-4	6.342	6.31683	6.31182	6.30613	6.1272	6.13787	6.27065	6.13548	6.56433	6.2956	6.22932	6.41622	6.23616	5.92099	6.31276	6.07665
PHF2	O75151	6.38972	5.91179	6.03056	6.93541	6.29536	5.80321	6.16976	5.76397	5.94054	6.12939	5.74061	6.33413	6.26765	6.97821	6.63343	6.67628
EPHB2	P29323	6.19165	6.62022	5.88312	6.61001	6.03393	5.94926	6.11784	6.0757	6.27507	6.22685	5.81078	6.29049	6.5902	6.32978	6.63797	6.35712
EPHB4	P54760	6.40386	6.39821	6.19477	6.62779	6.38613	6.38888	6.38085	5.91604	6.58915	6.48172	6.24837	6.3168	6.63846	6.07341	5.95392	6.00163
VPS28	Q9UK41	6.04583	5.32417	6.29746	4.92036	5.96491	6.03978	5.99059	6.84236	5.59352	6.94244	6.14996	6.8131	5.65481	7.6985	6.66197	7.06012
ANXA7	P20073	6.52625	5.74924	6.48957	5.05085	6.67077	6.74766	5.87043	7.01216	6.00883	6.76004	6.46817	6.79652	5.93998	6.29389	5.82863	5.78701
MTX2	O75431	5.90356	5.14324	5.44366	5.47155	6.09906	5.49323	6.14851	6.4618	5.38202	6.34328	5.71392	6.67407	6.64192	7.51931	7.6531	7.90777
NCAPH2	Q6IBW4-4	5.98095	6.22359	6.61305	6.27372	6.20525	6.29395	6.13405	6.1934	6.45863	6.0974	6.65239	6.62036	6.29524	5.97263	5.94772	6.03768
SEC22B	O75396	6.1534	6.26269	5.53128	6.49298	6.0483	5.96869	6.31773	6.13802	5.9854	5.90754	5.72745	6.00074	6.52486	6.8949	7.0502	6.99582
CDCA8	Q53HL2	6.72464	6.80475	6.4644	7.34021	6.19189	5.95433	6.69651	6.16976	6.63667	6.47353	6.29531	6.47364	5.27667	5.37032	5.33107	5.7963
SDCBP	O00560	6.26443	6.60069	7.03296	6.03153	6.41292	6.57107	6.24298	6.58018	6.50411	6.44157	6.46341	6.45774	5.72703	5.8478	5.48089	5.34069
HOXB4	P17483	6.30527	5.93772	6.34084	6.6001	6.18786	6.02784	6.5584	5.933	6.105	6.35538	6.16478	6.1456	6.15414	6.22662	6.2944	6.66304
DNAJB6	O75190	5.91762	6.08374	6.61556	6.16851	5.98228	6.22349	6.18997	6.19195	6.36797	5.9666	6.58601	6.30678	5.78716	6.5808	6.59301	6.43855
USP54	Q70EL1	6.19597	6.37076	6.43043	6.70976	6.08993	6.15234	6.24175	6.07002	6.12495	6.41586	6.36546	6.53027	5.75703	6.16908	6.06559	6.31081
DPP8	Q6V1X1	6.22609	6.09652	6.97502	6.10226	6.16846	6.43214	6.1768	6.45056	6.34946	6.48982	6.55029	6.56888	5.75556	6.01437	6.04476	5.59903

EXOSC9	Q06265-2	6.1675	6.35049	6.08478	6.52808	6.19202	5.97404	6.3098	5.36649	6.26239	5.94541	5.72812	5.45581	6.40064	6.58616	7.53267	7.11559
KTI12	Q96EK9	5.96628	5.54863	7.1007	5.76006	6.31094	6.70245	6.37646	6.42008	6.27736	6.55143	6.88975	6.87476	6.23954	5.87529	5.44974	5.65652
THNSL1	Q8IYQ7	6.67436	6.3305	5.99682	6.76244	6.21236	5.94941	6.01627	5.91271	6.2003	6.16244	6.05267	5.97102	6.41914	6.32251	6.53493	6.48211
ANKRD26	Q9UPS8	6.36823	6.16927	6.57302	6.25242	6.24466	6.3988	6.14465	6.41528	6.32361	6.53368	6.22578	6.42536	6.29929	6.02348	5.82051	5.78198
HINT1	P49773	5.61419	4.65698	5.76461	4.2265	5.49106	5.0269	5.31185	7.46819	5.09914	6.29134	5.24555	6.70967	5.12772	10.1095	8.35669	9.50007
ATP5MJ	P56378	6.02208	5.70255	5.40132	5.99147	6.44199	6.03099	6.88642	6.18002	5.79953	5.96529	6.18357	6.08202	6.77352	6.70569	6.86375	6.9698
RDH13	Q8NBN7	6.72305	5.87671	5.96883	6.46065	6.66003	5.86683	6.15172	5.85014	6.14985	5.87163	6.25903	6.14531	7.00276	6.18301	6.47626	6.3542
DPH2	Q9BQC3	6.01955	6.1415	7.13161	5.79871	6.15672	6.83954	6.27666	6.5178	6.20463	6.46467	6.81062	6.72744	5.94091	5.68318	5.58354	5.70292
ATG13	O75143-5	6.0915	6.13873	6.79889	6.28044	6.061	6.16337	5.87945	6.51273	6.15469	6.35969	6.20101	6.58947	6.20563	6.0899	6.33524	6.13827
CRIP2	P52943	6.6425	6.25197	6.69829	5.65251	6.43887	6.36268	5.93365	6.69634	6.30602	6.48313	6.29711	6.21128	6.31091	6.20698	6.06023	5.44754
RCL1	Q9Y2P8	6.53635	6.15303	6.03471	6.70922	6.74893	6.26876	6.29057	6.09947	6.21912	5.99712	6.00654	5.97513	6.85202	5.82847	6.30171	5.97886
BBX	Q8WY36	5.76494	5.9732	5.98478	5.93753	5.89392	5.75278	6.46147	5.56475	6.01826	6.09409	6.19276	5.79479	6.50526	7.30915	7.33276	7.41956
ASCC2	Q9H1I8	6.61312	6.62786	6.41106	6.20648	6.13184	6.28388	5.79548	6.22391	6.44744	6.96489	6.23119	6.45005	5.81001	5.94957	5.65141	6.20181
EDEM3	Q9BZQ6	6.40094	6.55049	5.9336	6.72927	6.39862	6.0831	6.19529	5.89329	6.41208	6.32148	6.11156	6.20408	6.39182	5.98693	6.14797	6.23948
BCAT1	P54687-5	6.16555	5.49734	6.81495	5.87938	6.51582	6.57493	6.60353	6.65368	6.32631	6.16284	7.14261	6.5696	5.81352	5.64946	5.77547	5.855
ALG2	Q9H553	6.38688	6.66538	6.11759	6.48851	6.19612	5.97906	6.13436	6.01708	6.22321	6.20077	6.02237	5.6081	6.495	6.17701	6.78797	6.50059
PRMT9	Q6P2P2	5.72321	6.14973	6.33487	5.92876	6.05895	6.31267	5.88313	6.27765	6.1516	6.47439	6.5951	6.52892	6.45966	6.51133	6.3052	6.30483
TTF1	Q15361	6.74617	6.58878	6.05879	6.66429	6.13202	6.03694	6.36147	5.96796	6.17835	6.0012	5.75336	6.21998	6.06702	6.32277	6.16084	6.74007
VPS36	Q86VN1	5.85202	6.16541	6.69605	5.88775	6.24487	6.40522	6.08386	6.26913	6.60159	6.43053	6.58277	6.78986	5.70035	6.1526	6.0574	6.0806
RPL37A	P61513	6.60077	6.85911	6.44897	6.47975	6.72774	6.69641	6.16447	6.58919	6.60635	6.50336	6.22036	6.1376	6.50863	5.40372	5.86698	5.1866
GTSE1	Q9NYZ3	5.85987	5.72711	6.37266	6.26745	5.95017	6.31052	6.03519	6.1324	6.46449	6.16174	6.50853	7.00119	6.15259	6.53079	6.02559	6.49972
AK4	P27144	6.29449	5.25516	5.79332	5.34294	6.54922	5.31812	5.90176	6.59573	5.58888	6.20627	5.91418	6.65226	7.22531	7.1265	7.24782	6.98806
NACC1	Q96RE7	5.48375	6.26411	5.8171	5.73943	6.12684	6.19881	5.63809	5.87625	5.90423	6.41873	6.36761	5.99197	6.95555	7.14878	6.90363	7.16512
PPP3CA	Q08209-2	6.19732	6.18964	6.59377	5.72993	6.51942	6.44996	6.02384	6.5325	6.2216	6.07908	6.43398	6.3408	6.1944	6.23756	6.08619	6.17001
IBTK	Q9P2D0	5.85635	6.54185	6.04371	6.32377	6.51056	6.34385	6.24234	6.60935	6.13507	6.41406	6.3242	5.97143	6.22274	5.95789	6.20077	6.30207
AAMP	Q13685	6.33842	6.41344	6.80814	5.85576	6.58283	7.03714	6.09734	6.99148	6.53942	6.30505	6.65735	6.54099	5.53571	5.60942	5.28941	5.3981
NPC2	P61916	6.68237	6.17228	6.16295	5.85868	6.95895	5.92628	6.26034	6.28233	6.15058	6.14391	6.15401	6.61636	6.25949	6.11714	6.07269	5.82166
PTPN2	P17706	5.62294	6.25288	6.07394	6.54537	5.70323	6.41406	5.93229	6.0279	5.97406	6.18292	6.07721	6.30879	6.44466	6.98821	6.83935	6.61219
EMC7	Q9NPA0	6.48489	6.54949	6.17405	6.64003	6.0345	5.94762	6.4438	5.96837	6.05339	6.11945	6.02108	6.06053	6.34405	6.10944	6.57206	6.47726
CCDC43	Q96MW1	6.19849	6.45263	7.00795	6.26939	6.29886	6.7746	6.47374	6.551	6.04221	6.74725	6.72984	6.46732	5.43409	5.8819	5.3291	5.34162
PBDC1	Q9BVG4	6.79801	6.39754	6.74809	5.85645	6.62386	6.69696	5.95683	6.60399	6.5957	6.51833	6.53935	6.76723	5.9864	5.55345	5.14809	5.20974
RTN4IP1	Q8WWV3	6.75197	6.39987	5.84013	6.6612	6.89502	6.2839	6.06856	5.77928	5.94783	5.8817	5.81246	5.95552	6.92997	6.28789	6.40858	6.09611
CDCA3	Q99618	6.24318	5.3187	5.95203	5.38859	6.77008	5.38096	5.44163	6.72085	5.89758	6.25695	6.00375	6.71578	7.25825	6.96716	6.58506	7.09941
FKBP3	Q00688	6.33826	6.22536	6.32409	6.56459	6.60312	6.65903	5.88662	6.60054	6.32343	6.43836	6.70803	6.3543	6.34138	6.06792	6.06273	5.50225
RANBP1	P43487	6.78849	6.31622	6.94439	6.29351	6.69557	6.93291	6.09978	6.24741	6.69795	6.16676	6.68974	6.57216	5.4156	5.66934	5.05585	5.41432
HCCS	P53701	5.97211	5.90496	5.87963	6.39669	6.62805	6.04522	6.26603	6.53074	6.22735	6.0014	6.18435	6.24257	6.93482	6.21371	6.24936	6.323
GSPT2	Q8IYD1	6.16805	6.30802	6.86968	5.87576	5.78131	6.79767	5.94573	6.78118	6.08633	6.58322	6.73426	6.82516	5.50953	6.01212	5.71297	6.00901
PNKP	Q96T60	5.36032	5.76315	5.8608	5.78065	5.83947	5.87495	5.75818	6.13723	6.12406	6.08743	6.56385	6.24354	7.22729	7.17618	7.27634	6.92656
POLR1C	O15160	6.30944	6.37677	6.49818	6.21395	6.11391	6.59543	5.8448	6.73598	6.34538	5.97407	6.49808	6.50418	6.43443	5.59799	5.95028	6.00715
CNBP	P62633-6	6.44111	6.11805	6.29718	5.80007	6.4897	6.58292	5.92792	6.11755	6.66502	6.89977	6.64655	6.36349	6.26011	5.83816	5.74479	5.80761
ITGA1	P56199	6.03593	6.23267	5.79365	6.29514	6.05698	6.11163	6.19972	6.12688	5.90051	6.38938	6.02193	6.1986	6.57144	6.66369	6.93437	6.46749
RFWD3	Q6PCD5	6.59538	6.57989	7.45519	7.17984	6.61986	6.59942	6.42226	6.248	7.07244	6.52954	6.51392	6.65834	4.83683	4.8833	4.8609	4.94489
PRR14L	Q5THK1	6.31417	6.30117	6.1305	6.65224	6.29921	6.1531	6.5372	6.25719	6.39686	6.41544	6.58393	6.58486	5.87701	5.57475	5.89	6.03238
MICALL1	Q8N3F8	6.37669	6.63105	6.74456	6.08531	6.09243	6.38289	6.23392	6.29382	6.20594	6.37478	6.75148	6.69144	5.8319	5.71218	5.79728	5.79434
TYW1	Q9NV66	6.45468	6.7512	6.39111	6.54687	6.1365	5.96765	6.21835	6.5048	5.99836	6.41851	6.38339	6.18547	6.07447	6.10384	5.78853	6.07626
FBXO30	Q8TB52	6.339	6.17692	6.84633	5.95081	6.53017	6.63409	6.12898	6.64477	6.4809	6.40949	6.55735	6.61514	6.01965	5.65639	5.55578	5.45423
FRG1	Q14331	5.70547	6.01455	6.08614	5.8696	5.75647	5.96885	5.99645	5.81433	6.0442	6.3629	6.05655	6.07118	6.62587	7.28787	7.28248	7.05708
FASTKD1	Q53R41	6.53461	6.08134	6.04695	7.02819	6.15637	6.04136	6.5616	6.03373	6.47533	6.54543	6.13418	6.47267	6.19694	5.77912	6.14917	6.463
CSRP1	P21291	6.43564	6.08146	6.69063	6.01891	6.57667	6.55012	6.18627	6.27408	6.65308	6.32324	6.39435	6.5084	5.96498	6.0643	5.79813	5.59732
WDR6	Q9NNW5	6.5702	6.60743	7.08564	6.47523	6.31978	6.59527	6.70893	6.21051	6.75475	6.73583	6.88932	6.68288	5.13851	5.05019	5.08978	5.08575
CACUL1	Q86Y37	5.73204	5.05921	5.65311	4.61646	5.57152	5.72913	5.49253	6.24145	5.74478	6.52947	6.33435	6.78444	7.18629	8.02126	7.91287	7.39109
CHD1L	Q86WJ1	5.763	5.96983	6.39589	5.336	5.57593	6.38429	5.88884	5.93108	6.12454	6.09168	6.69359	6.32979	6.51382	7.04069	7.1	6.86103
PLEKHO2	Q8TD55	6.48258	5.90211	6.52847	6.03455	6.66583	6.05036	5.83504	6.26419	6.34839	6.69971	6.58971	6.24007	6.65184	6.2515	5.9356	5.52004
PRPF18	Q99633	6.23634	6.34019	6.12021	5.86968	6.1109	6.35155	6.17295	6.57456	6.27491	6.34172	6.14226	6.11426	6.06054	6.6389	6.42219	6.22884
TPMT	P51580	6.17674	6.29418	6.74932	5.93866	6.28001	6.88007	6.17701	6.55078	6.25711	6.57655	6.46196	6.58515	5.8883	5.87565	5.38811	5.92038
APTX	Q7Z2E3	5.72517	6.27979	5.92329	5.7111	5.98801	6.13057	5.92686	6.00611	6.06852	6.32068	6.27783	6.06062	7.07022	6.76253	7.08746	6.66124
NIPSNAP2	O75323	6.15946	6.00797	6.10633	6.39327	6.43477	5.89311	6.73643	5.92621	6.42956	5.8168	6.39381	5.61868	6.56602	6.00252	6.93243	6.58264
MCU	Q8NE86	6.45706	6.11692	6.00248	6.38438	6.50337	5.78526	6.48687	5.87152	6.29097	6.00112	6.03746	5.88822	6.95119	6.15003	6.84785	6.2223
TUFT1	Q9NNX1	6.70889	5.90232	6.84451	6.52856	6.41865	6.72666	6.34229	7.16652	6.27822	6.05397	6.76496	6.70262	5.61877	5.37941	5.09319	5.47047
SLAIN2	Q9P270	6.2398	6.22931	6.41826	5.64844	6.66526	6.60068	5.76654	6.88194	6.53375	6.35949	6.50327	6.38226	6.49877	5.91612	5.62685	5.72925
UFSP2	Q9NUQ7	6.19989	6.39563	6.15307	6.61092	6.11827	5.75934	6.30045	6.10475	6.31827	5.87126	6.23958	5.84237	6.53191	6.25316	6.66571	6.63541
AGRN	O00468	7.28021	7.13625	6.72003	7.25347	6.29625	5.67068	6.11659	5.89703	6.79892	7.0435	7.06055	6.61841	4.97821	4.91355	5.08046	5.1359
RRP15	Q9Y3B9	6.28684	6.40797	6.19982	7.14311	6.23455	5.84471	6.61332	6.12809	5.90586	6.20475	6.16358	5.88352	6.63903	6.18896	6.2616	5.89429
DHX33	Q9H6R0	6.18552	6.44017	6.14066	7.11599	6.18509	5.8647	6.31439	6.05764	6.18421	5.86294	6.1144	5.96746	6.3742	6.12198	6.40548	6.66517
SERPINE1	P05121	6.30032	5.22906	4.96587	6.59592	6.12132	6.24836	7.9408	6.55545	5.49641	4.36721	4.81467	5.00909	8.61529	5.71527	8.04102	7.98393
SYAP1	Q96A49	6.15197	5.9805	6.6337	5.8575	6.40389	6.68409	5.97737	7.00804	6.30412	6.64634	6.72624	6.92373	6.04185	5.77251	5.3415	5.54665
COPS8	Q99627	5.48182	4.8572	5.42877	4.40864	5.73753	5.31427	5.52207	6.87501	5.35885	6.3071	5.57877	6.68248	6.30288	9.07283	8.33945	8.73234
HDHD3	Q9BSH5	5.86371	5.20195	5.56037	5.43786	6.00676	5.66745	5.84263	6.40295	5.64203	6.42231	5.64121	6.584	6.46355	7.48908	7.80543	7.96871
TRAPPC9	Q96Q05-2	6.21747	6.49727	6.59805	6.37011	6.29827	6.60116	6.33684	6.14764	5.98985	5.9408	6.36009	5.87864	5.89017	6.31406	6.45364	6.10596
PAM	P19021-5	6.76264	6.71188	6.37458	6.79925	6.54066	6.55419	6.7587	6.4826	6.0638	6.34909	6.29653	6.55913	5.39249	5.55257	5.22043	5.58146
TFB1M	Q8WVM0	6.44174	6.41126	6.09845	7.06101	6.162	6.05157	6.70635	6.01458	6.04063	6.21146	5.95706	6.17733	6.31377	5.72215	6.30937	6.32128
PSMB6	P28072	6.07012	6.23748	6.10554	5.71227	6.61894	6.72998	6.49534	6.46796	5.99251	6.00788	6.59465	6.29934	5.903	6.39617	5.81137	6.55744
DYNLL1	P63167	6.049	5.5811	6.05967	5.32938	6.49371	6.25592	5.85687	6.55854	5.45656	6.02959	5.75316	6.0509	5.93145	8.10932	7.14389	7.34094
THAP12	O43422	5.92765	6.52943	6.18432	6.37892	6.02	6.01193	6.06144	5.88042	6.19535	6.32362	6.23334	6.14811	6.613	6.53166	6.41562	6.5452
ELP3	Q9H9T3	6.00755	6.1962	6.62893	6.11931	6.40488	7.30889	6.48519	6.43401	6.06294	6.11574	6.74064	6.53724	5.98111	6.03144	5.53713	5.4088
MIGA1	Q8NAN2	6.55424	6.27724	5.76288	6.55155	6.54624	5.45743	6.39586	5.6202	6.39013	6.34622	6.18273	6.07248	6.49689	6.39718	6.15367	6.79507
HAUS3	Q68CZ6	6.675	6.3895	7.08069	6.34076	6.3027	6.58677	6.20164	6.63309	6.42055	7.0274	6.81513	6.94881	5.137	5.37218	4.94768	5.12111
WTAP	Q15007	6.59587	6.27026	6.28618	6.64919	6.55363	6.36668	5.95367	6.35587	6.01504	6.2106	6.34106	6.60915	6.70093	5.53965	5.77356	5.77866
CCNK	O75909-4	6.50887	6.15016	5.98452	6.30865	6.36608	6.14088	6.25654	6.11319	6.14779	6.03022	5.86836	6.06294	6.62825	6.17466	6.53488	6.72403
FIBP	O43427	6.28906	6.39041	6.65289	6.2677	6.24585	6.44866	6.24259	6.44653	6.39558	6.57862	6.50249	6.38453	5.72201	5.78481	5.70483	5.94344
TAF1C	Q15572	6.28165	6.26228	6.43252	7.05704	6.18941	5.97696	6.45411	5.81809	6.47778	6.2579	6.26761	6.21278	6.19311	5.70397	6.17387	6.24091
SMNDC1	O75940	5.91423	6.35586	5.83038	6.1138	5.99201	6.13741	6.00987	5.83982	5.91786	5.91627	6.1905	6.03133	6.83378	6.88952	7.18362	6.84375
CWC15	Q9P013	6.23991	6.48204	5.75583	6.4999	6.14141	6.1379	6.15812	6.26551	6.23231	6.11519	6.11687	6.34467	6.84851	6.26269	6.01788	6.38124
SAP30BP	Q9UHR5	6.13965	5.8817	5.97438	6.09153	5.98389	6.05878	6.01767	6.31882	5.87987	6.06378	6.33314	6.10301	6.91196	6.61863	6.74163	6.88157
N4BP2	Q86UW6	6.14371	6.13999	7.07775	5.84872	5.74337	6.79765	6.30604	6.43533	6.51146	6.26246	7.21953	6.72332	5.3049	5.87455	5.74552	5.8657
LRIF1	Q5T3J3	6.84569	6.9698	6.3647	7.49367	6.29023	5.95399	6.10785	6.09618	6.21598	6.31885	6.28637	6.31099	5.59251	5.7283	5.46329	5.96159
TDP1	Q9NUW8	5.71602	5.89674	5.85802	5.31961	5.76264	6.40182	5.59626	6.32957	6.00532	6.1789	6.25498	6.25085	6.51018	7.41949	7.37981	7.11977
FADS1	A0A0A0MR51	6.96979	6.95711	6.44469	6.79972	6.34076	6.30458	6.5776	6.07673	6.40821	6.58146	6.4052	6.33351	5.51159	5.56157	5.24311	5.48436
STAMBP	O95630	6.28575	6.2701	6.73641	5.66009	6.62679	6.67327	6.08935	6.50253	6.63466	6.29726	6.362	6.31783	6.01229	5.88024	5.99653	6.5549
SLC12A4	Q9UP95	6.05822	6.71893	6.49514	6.38441	6.05591	6.44719	6.27537	6.33874	6.4875	6.29993	6.51211	6.4766	5.79737	6.09826	5.78499	5.76935
EXOC6	Q8TAG9	6.35088	6.28561	6.48534	6.00883	6.65291	6.58694	6.269	6.55753	6.71567	6.56717	6.63573	6.4135	5.76414	5.61024	5.58401	5.5125
LMF2	Q9BU23	6.27901	6.55109	5.84008	6.89195	6.17072	6.07415	6.33082	6.01969	6.196	6.1397	5.83114	6.01299	6.92657	5.94434	6.64817	6.14359
POLD3	Q15054	5.1225	6.05249	6.10469	5.68129	5.67759	6.14606	5.84245	5.67194	5.97741	6.15437	6.37851	6.14558	6.78766	7.30076	7.62941	7.32729
FLCN	Q8NFG4	6.82732	6.50031	7.31008	6.35697	6.08031	6.67033	6.26453	6.55963	6.77002	6.99924	6.91013	6.99351	4.75326	5.00141	4.75463	5.24831
TBC1D13	Q9NVG8	5.88045	5.27372	6.72264	5.20892	5.91939	5.89874	5.83483	6.45337	6.02802	6.51821	6.44264	6.81791	6.22906	7.19704	7.00564	6.56943
NOP53	Q9NZM5	6.52599	6.79248	6.04651	6.89084	6.25534	5.92376	6.54842	6.2187	6.90622	6.00305	6.19273	6.04116	6.22579	5.79805	5.70328	5.92766
NAA50	Q9GZZ1	6.43019	6.10065	7.0285	5.83726	6.32839	6.6964	6.24959	6.8202	6.53307	6.33804	6.96239	6.58711	5.77097	5.58175	5.51993	5.21556
ZNF281	Q9Y2X9	6.11842	6.42588	6.35347	6.80415	6.04376	6.25236	6.68856	5.88481	6.25279	6.13976	6.44786	6.36113	6.10275	5.75435	6.17363	6.19631
NMNAT1	Q9HAN9	5.76375	5.7566	5.97779	5.56689	5.91149	5.6522	6.06614	5.89358	6.17165	6.15226	6.20204	6.0175	7.08028	7.18181	7.58674	7.01929
KIAA0319L	Q8IZA0	6.54173	6.68861	5.98503	6.66248	6.41014	6.12872	6.42255	6.18656	6.33906	6.27708	6.09776	6.07942	6.14052	5.9656	6.04515	6.02958
MEN1	O00255	6.51424	6.44775	6.10999	6.97122	5.83979	6.00827	6.43187	5.84596	6.34108	6.12994	6.06711	6.06056	6.22163	6.20229	6.54339	6.26493
KPNA6	O60684	6.14283	6.0743	6.2076	5.81434	6.17625	6.36448	6.22435	6.10354	6.2703	6.06649	6.1178	6.12519	6.48096	6.3278	6.91456	6.5892
DYNC2I2	Q96EX3	6.72777	6.29813	6.97809	6.275	6.42312	7.23679	6.20522	6.86798	6.62999	6.47924	6.58767	6.89784	5.19441	5.41925	4.79501	4.98449
SCO1	O75880	6.63802	6.39862	5.90073	6.80547	6.30223	5.767	6.09658	6.10013	5.9988	6.23848	6.10437	5.76451	6.66823	6.2667	6.61451	6.33563
RNPC3	Q96LT9	6.32665	6.22475	6.01318	6.88448	6.11964	6.22456	6.17867	6.01697	5.85373	6.10294	6.05455	6.05168	6.86252	6.19764	6.38079	6.50726

RASSF8	Q8NHQ8	6.43654	6.22382	6.52471	6.59535	6.20134	6.08605	6.24059	6.11254	6.48788	6.23886	6.39722	6.13706	6.078	5.89682	6.206	6.13721
PCYT1A	P49585	5.54147	5.35704	6.32621	5.41114	5.70112	6.53938	5.47722	6.31591	6.21527	6.37923	6.46187	6.18293	6.59757	7.28389	7.14026	7.0695
LNPK	Q9C0E8	6.54113	6.26908	5.82149	6.31797	6.37627	6.03207	6.29053	6.14142	5.99964	6.07128	6.04812	6.0807	6.31697	6.5121	6.66695	6.51426
CTIF	O43310-2	6.59003	6.29469	6.99806	6.73158	6.12403	6.60491	6.35996	6.95676	6.61142	6.43903	6.82371	6.64185	5.36822	5.3789	4.9306	5.14626
HMGN5	P82970	5.85842	6.34028	5.70116	6.08845	5.9212	5.99933	6.33623	5.76383	6.06278	6.16974	6.05265	5.76869	7.00664	6.94111	6.70786	7.28164
GLT8D1	Q68CQ7	6.40889	6.45355	5.66754	6.54235	6.50461	6.25175	6.4346	6.42315	6.256	6.38888	5.83381	6.0236	6.24189	6.02902	6.34005	6.2003
PIGK	Q92643	6.29886	6.33081	6.04442	6.6182	6.14649	5.7649	6.25576	5.83635	6.25652	6.38724	5.8662	6.096	6.34212	6.23886	6.98601	6.53125
SSR1	P43307	6.5329	5.76047	6.01797	6.6857	6.61588	5.73139	6.60567	5.69887	6.53474	5.86543	5.86717	6.02459	6.90946	6.053	6.74134	6.35542
SNX27	Q96L92	6.35437	6.32906	6.20129	6.1436	6.25946	6.42007	6.44584	6.21257	6.19502	6.3275	6.50134	6.36758	6.20696	5.6597	6.31282	6.06282
ABRAXAS2	Q15018	6.31516	6.09546	6.8656	5.88234	6.51412	6.3594	6.26508	6.36487	6.42411	6.42555	6.61009	6.36938	5.80034	6.11857	5.77134	5.8186
TAB2	Q9NYJ8	6.09978	6.36523	6.62164	5.92358	6.65479	6.87031	6.40879	6.86939	6.2142	6.24031	6.26027	6.4603	6.48103	5.53923	5.36444	5.6267
SKA1	Q96BD8	6.64462	6.60149	7.19027	6.261	6.3173	6.81049	6.45822	6.48005	6.96491	6.58047	7.06119	6.90687	5.06737	4.92215	4.76045	4.97313
WDR91	A4D1P6	6.49627	6.52779	6.51015	5.95998	6.26333	6.07227	6.00379	5.99668	6.43204	6.60187	6.31436	6.29288	6.46189	6.20401	6.02993	5.83276
CEP89	Q96ST8	6.4507	6.44658	7.0247	6.6672	6.48035	6.67472	6.6812	6.62995	7.00685	6.72844	7.00969	7.20071	4.56547	4.88471	4.60239	4.94636
CWC25	Q9NXE8	6.14709	6.19812	6.01013	7.14647	5.54296	5.82631	6.57469	6.34014	5.92754	5.86759	6.18922	5.88631	6.34596	6.80873	6.53879	6.64996
AURKA	O14965	6.26479	5.9271	6.65572	6.92865	6.13104	6.33105	6.42877	6.05044	6.42623	6.56353	7.09708	7.04788	5.30396	5.39051	5.20109	6.25214
ZNF830	Q96NB3	6.67765	6.4895	7.04227	6.98956	6.44398	6.085	5.87245	5.97477	5.93019	5.93861	5.6053	6.11249	6.33495	6.04659	6.29543	6.16126
HTRA2	O43464	6.36492	6.18051	5.746	6.53186	6.36074	6.12381	6.15307	6.46411	6.07602	6.15989	5.98818	6.09844	6.77847	5.95691	6.43748	6.57959
SNRPD1	P62314	6.28934	6.80335	5.85908	6.97503	6.26416	6.51081	6.38956	5.9721	6.13453	5.98116	5.79392	5.97323	6.11325	5.88798	6.87007	6.18244
VPS29	Q9UBQ0-2	5.97278	5.93547	6.22244	5.56787	6.18793	6.43642	5.91432	6.57034	6.34612	6.43903	6.33191	6.3232	6.18835	6.62683	6.64867	6.28831
MAP4K5	Q9Y4K4	6.17598	6.53171	6.81224	6.06003	6.32195	6.40889	6.26708	6.57212	6.45917	6.61979	6.20627	6.54857	5.6451	5.67276	5.91729	5.78105
SLC39A10	Q9ULF5	5.86609	6.29319	6.04919	6.16695	6.15579	5.98852	6.60282	6.2421	6.10788	6.10942	5.55237	5.96715	6.41519	6.61523	6.86379	7.00434
BCKDHB	P21953	5.83899	6.00635	5.62634	6.30152	6.44923	6.11552	6.54397	5.72009	6.32891	6.26158	6.15619	5.81714	6.80915	6.05505	7.04467	6.92529
LBR	Q14739	6.45584	6.21634	6.35887	6.90112	5.90999	5.77818	6.35894	5.89224	6.02584	5.58266	6.30457	5.89199	6.32066	6.70946	6.37517	6.91811
GPR89A	B7ZAQ6	6.12554	6.15664	6.02376	6.64112	5.93481	5.9227	5.99393	5.87396	6.04647	6.18944	5.96957	6.1709	6.70014	6.45972	6.72601	7.06528
WDR59	Q6PJI9	6.05495	6.16568	6.75425	6.46605	6.08002	6.65825	6.44583	6.20279	6.3925	6.80913	6.76252	6.52565	5.65814	5.76291	5.81475	5.44658
UBFD1	O14562	6.48793	6.24746	6.81581	6.02842	6.41024	6.58799	6.13641	6.92133	6.09494	6.47751	6.72103	6.59497	5.84591	5.61809	5.53041	5.48152
SAMD9	Q5K651	6.17777	6.35516	6.86411	5.90699	6.0622	6.22273	6.32555	6.42794	6.36616	6.52731	6.44832	6.77523	5.74503	6.00652	5.85813	5.93085
MRPS7	Q9Y2R9	6.53441	6.58341	6.21231	6.43624	6.59016	6.36002	6.54067	6.18349	6.1452	6.05428	6.17889	5.73732	6.59876	6.01931	5.98431	5.84123
ARMH3	Q5T2E6	6.22366	5.93183	6.34268	6.17128	6.06207	6.32306	6.50808	6.09041	6.61657	6.439	6.55373	6.36399	5.99165	6.17844	6.0902	6.11284
HSD17B12	Q53GQ0	6.57137	6.13957	6.01914	6.46416	6.32682	5.96586	6.244	5.79252	6.28161	6.10075	5.9693	5.80436	6.77653	6.02315	7.20312	6.31772
RAB18	Q9NP72	5.92173	6.08016	6.20664	6.38869	6.41866	6.07075	6.45882	5.796	6.18186	5.72313	6.01444	5.914	6.65592	6.51627	6.86848	6.78444
MRPL17	Q9NRX2	6.70822	6.34945	6.0175	6.13251	6.75466	5.68767	6.31208	6.33718	6.34625	6.14121	6.41177	6.17596	6.83051	5.72479	6.15233	5.9179
SF3B6	Q9Y3B4	6.56588	6.37855	5.93986	6.37557	6.52115	6.48774	5.97322	6.64862	6.22259	5.94604	6.33599	6.01155	6.36927	5.84663	6.42058	5.95677
PQBP1	O60828	6.22441	6.01439	6.16098	6.06237	6.29072	6.36139	6.11744	6.18371	6.37075	6.21116	6.01532	5.96498	6.59856	6.52443	6.38344	6.51592
RIN1	Q13671	6.55073	6.25517	6.66935	5.85516	6.51904	6.71971	6.38842	6.66355	6.26809	6.53657	6.53671	6.50106	5.94742	5.7092	5.41822	5.4616
ZFPL1	O95159	6.30248	6.86815	5.75512	6.78157	6.4785	6.5529	6.56148	6.12983	6.0612	6.23673	6.15048	5.99278	6.40154	5.82327	5.9237	5.98028
PM20D2	Q8IYS1	6.09943	5.65727	6.50317	5.32885	6.55782	6.6363	5.95311	7.14203	6.29175	6.54686	6.89925	7.03809	5.85433	5.86882	5.74062	5.88229
NSRP1	Q9H0G5	6.09929	6.47097	5.85527	6.5777	6.2275	6.2705	6.20004	6.0182	6.10079	6.19401	6.00202	5.84332	6.38268	6.55405	6.67164	6.53201
NDST1	P52848	6.46467	6.32026	6.06227	6.65715	6.17831	6.06067	6.44024	6.35067	6.21047	6.01963	6.03254	6.19925	6.03451	6.28455	6.04758	6.63722
BRD3	Q15059	6.13785	6.53078	6.17813	6.60441	6.03491	6.12634	6.3918	6.15367	6.24456	6.23737	6.12206	6.39629	6.18698	6.23716	6.22144	6.19625
IRF2BP1	Q8IU81	5.98353	6.18059	5.89138	6.00985	6.2591	6.36079	5.96652	6.25542	6.30539	6.5505	6.36135	6.07256	5.95715	6.30645	6.35542	6.18399
LRRC8E	Q6NSJ5	6.76445	6.59809	6.3095	6.98188	5.82693	6.15445	6.19422	6.02661	5.93968	6.68068	6.02362	6.58286	6.04964	5.52325	6.05108	6.29305
PURA	Q00577	6.23321	6.20401	6.68813	6.0182	6.47533	6.57838	6.43765	6.23694	6.62602	6.42915	6.53737	6.26967	6.13228	5.90451	5.76759	5.46157
TPM2	A7XZE4	6.28209	6.29633	6.82162	6.48117	6.5436	6.58451	5.52117	7.13313	6.16417	6.33689	6.61745	6.85298	6.26235	5.60622	4.94529	5.55104
EIF2B5	Q13144	6.06422	6.05821	6.87404	5.7869	6.26327	6.71051	6.08101	6.34012	6.40076	6.55579	6.89744	6.51824	5.89336	5.92652	5.93256	5.69704
SRPK2	P78362-2	6.37693	6.5163	6.46082	6.1484	6.23902	6.06877	6.10878	6.09318	6.67788	6.37193	6.54636	6.47843	5.86134	6.05948	6.10138	5.89101
KNOP1	Q1ED39	5.94739	6.08041	5.76443	6.67276	5.62886	5.47509	6.07542	5.60541	6.30194	6.04806	6.10463	6.2807	6.26063	7.13167	7.26338	7.35922
SCLY	Q96I15	5.66561	5.73967	6.689	5.66524	5.94607	6.60359	5.68184	5.82618	6.05299	6.66265	6.26308	6.44968	6.66767	6.76538	6.98914	6.3322
IGBP1	P78318	6.38585	5.94872	6.90762	5.95327	6.38781	6.37208	5.80399	6.9576	6.38181	6.53197	7.06896	7.18976	5.79611	5.66257	5.40119	5.25068
SLC16A1	P53985	6.37136	6.44755	5.72367	6.26954	6.07102	5.92833	6.17346	6.29094	6.1166	5.95452	5.83609	6.13175	6.75036	6.53709	6.78426	6.61345
NDUFAF4	Q9P032	6.4363	6.04104	6.05648	6.00719	6.71041	5.73277	5.95695	6.17772	6.1688	5.75211	6.2068	6.26016	7.16736	6.33852	6.63984	6.34755
RC3H1	Q5TC82	6.23362	6.15418	6.18277	6.25338	6.19602	5.97504	6.21211	6.29465	6.22439	6.4518	6.41267	6.84851	6.14263	5.94445	6.06113	6.41263
PALLD	Q8WX93	6.30928	5.99302	6.64496	6.15743	6.4179	6.20472	6.01691	6.24008	6.54195	6.7267	6.57076	6.74016	6.43244	5.86129	5.48941	5.65298
OXSR1	O95747	6.38224	6.28575	6.62314	5.72439	6.47211	6.65132	6.10468	6.50266	6.47496	6.43638	6.19663	6.30738	6.06479	6.16376	5.83404	5.77578
CDK17	Q00537-2	5.59146	6.32593	6.4526	6.25421	5.98104	6.80156	6.65504	6.71496	5.93246	6.26934	6.36679	6.44136	6.0498	6.09336	5.93251	6.13758
PRKD2	Q9BZL6-3	6.34236	6.45492	7.37075	6.32502	6.35422	6.82413	6.1117	6.66298	6.21465	6.45533	6.59855	6.69117	5.4562	5.63222	5.20241	5.30339
MRPL2	Q5T653	6.27585	6.35481	5.77195	6.3376	6.51773	5.91149	6.80679	6.30572	6.20013	6.04761	5.76481	5.75867	6.5569	6.41577	6.43652	6.53765
PTPRS	Q13332	7.12423	6.9229	6.68351	6.52762	6.14869	5.98769	6.36858	5.88931	6.88717	6.72507	6.36852	6.67879	5.54476	6.07641	5.18078	4.88597
GPR107	Q5VW38-2	6.13058	6.19275	5.93177	6.29734	6.41027	6.13431	6.12597	5.79131	6.30084	6.44728	6.22212	6.08939	6.65454	6.52927	6.41371	6.32856
SHMT1	P34896	6.22666	6.26483	6.90347	5.60342	6.52274	6.86256	6.20471	6.75368	6.6765	6.56313	6.50458	6.24626	5.77145	5.67556	5.8168	5.40365
MTR	Q99707	6.7586	6.37442	7.43783	6.24595	6.6554	6.88749	6.37181	6.70335	6.84677	6.83145	6.82352	7.12556	4.64816	4.77541	4.60298	4.91131
NAA10	P41227	6.29995	6.44229	6.55032	5.84571	6.41355	6.89401	5.90295	7.2517	6.55048	6.74464	6.62028	6.96055	5.3472	5.70373	5.01624	5.4564
THUMPD3	Q9BV44	6.66452	6.59963	6.70973	6.1629	6.42185	6.7495	6.36043	6.84279	6.42614	6.30311	5.99402	6.04824	5.8474	5.79223	5.48434	5.59318
GGCX	P38435	6.1943	6.31556	6.26681	6.56846	5.88113	6.14618	6.38673	5.85757	5.86019	6.34322	6.24356	6.12736	6.13346	6.57188	6.58243	6.52117
TRA2A	Q13595	6.27726	6.55802	6.11113	6.7148	6.35671	5.76096	6.20835	5.72136	6.16423	6.02588	6.39079	6.30842	6.46636	5.78379	6.49613	6.65582
NFYC	Q13952	5.80787	5.26481	5.95306	5.17885	5.70735	5.55078	5.92328	5.4973	5.71601	6.58069	6.52727	6.97995	7.63068	6.79815	7.79934	7.08461
MBNL1	Q9NR56	6.46559	6.59917	6.34025	5.59995	6.4648	6.72519	5.7531	5.94756	6.11026	6.15512	6.13512	6.21007	6.62506	6.28498	6.34138	6.24241
PHKB	Q93100-3	6.3093	6.1973	6.7412	5.65039	6.31559	6.63126	6.13802	6.76677	6.56679	6.3609	6.36215	6.29321	6.00135	6.1623	5.68477	5.81869
STBD1	O95210	6.624	6.64062	6.09711	6.61654	6.07358	6.3639	6.33743	6.1009	5.95589	6.29447	5.86745	6.01689	6.27096	6.19274	6.24254	6.30499
EPB41L5	Q9HCM4	6.17441	6.32441	6.27374	6.48782	5.68652	5.99551	6.41277	5.72004	6.46826	6.27724	6.1604	6.1901	6.33558	6.27762	6.60379	6.6118
JADE2	Q9NQC1	5.97079	6.54791	6.30205	6.76977	5.82028	6.14284	6.11116	5.99116	6.4566	6.46157	6.47169	6.63775	5.79847	6.25375	5.93412	6.3301
TBCC	Q15814	6.0872	6.18819	7.17256	5.84819	5.83435	6.44668	6.04112	6.72488	6.23245	6.39378	6.52873	6.87812	5.93625	5.91213	5.82259	5.9528
ISG20L2	Q9H9L3	6.0183	6.41259	5.82133	7.12231	5.5652	6.6356	6.43871	5.90713	6.05011	6.05278	6.13592	6.39871	6.44158	6.34028	6.60842	6.68968
FGD4	F8VWL3	6.36691	6.3801	6.40129	6.28018	5.93079	6.37043	6.00016	5.94917	6.14954	6.04704	6.40375	6.33657	6.19973	6.41885	6.25089	6.51461
PRORP	O15091	6.16237	6.68109	5.80871	6.65412	6.35466	6.19649	6.67303	6.18757	5.84219	6.01739	5.93506	6.08412	6.4923	5.9883	6.32473	6.59787
DNAJB14	Q8TBM8	6.58918	6.78386	6.28453	6.93191	6.15287	6.19689	6.40307	6.27728	6.07152	6.50079	6.23454	6.26016	5.87091	5.59866	5.80503	6.0388
NUTF2	P61970	4.9443	4.92999	5.07702	4.04621	6.10281	6.09631	6.15272	8.94892	6.315	7.12695	5.90386	7.08291	4.49361	8.46151	6.48073	7.83715
UBE2L3	P68036-3	6.12314	5.60388	6.82997	5.41611	6.50637	6.3833	6.07585	6.69518	6.73349	6.98607	7.55237	6.80141	5.96924	5.45639	5.62476	5.24247
SERPINB1	P30740	6.0911	6.02201	6.9281	5.88213	5.9548	6.80345	6.04262	6.84868	6.18408	6.538	6.58422	6.45737	5.73757	6.24241	5.82312	5.86034
ERCC6	Q03468	5.99568	6.10527	6.43069	6.51636	5.83087	6.26452	6.24621	5.94277	6.53391	6.28561	6.05443	6.61215	6.35486	6.16004	6.35216	6.31445
CHTF18	Q8WVB6	5.82587	6.08191	5.97856	6.21274	6.16129	6.5633	6.05688	6.21432	6.03605	5.85517	6.15271	5.79172	6.77174	6.65311	6.7598	6.9434
FIGNL1	Q6PIW4	5.84889	6.46843	6.39696	5.91767	6.03106	6.18669	6.32792	6.41997	6.18356	6.2274	6.55861	6.2883	6.39734	6.20414	6.31669	6.22637
MYSM1	Q5VVJ2	6.00664	5.95643	6.49975	6.37935	6.06062	5.96132	6.23589	5.6222	6.44765	6.29099	6.18093	6.14071	6.48785	6.15501	6.46121	7.11346
AP4E1	Q9UPM8	6.2604	6.47469	6.74955	6.42921	6.14407	6.43028	6.52247	6.65469	6.28001	6.39591	6.43392	6.12791	5.99073	5.8101	5.65532	5.74073
CHML	P26374	6.11603	6.00159	6.73503	5.90832	6.2109	6.45466	5.97226	6.64648	6.15415	6.53663	6.50864	6.84088	5.68925	5.74011	6.2211	6.26398
CHM	P24386	6.02716	6.16906	6.91288	5.95199	6.53537	6.81961	6.41071	6.20447	6.30012	6.53352	6.37321	6.60751	5.82581	5.60729	5.97571	5.74557
LAMC2	Q13753	6.73427	6.36057	5.8889	6.49629	6.94451	6.31309	6.6654	6.48457	5.92967	6.14865	6.02153	6.09763	6.17661	5.50775	6.17914	6.05142
TYMS	P04818	6.31	6.1452	7.50009	6.31921	6.53067	6.88884	6.48669	7.28314	7.01293	6.20028	7.3771	7.2272	4.5466	4.62953	4.34831	5.1942
SURF6	O75683	6.13792	6.30975	6.02084	6.64073	5.89616	5.43456	6.54595	5.74532	6.20502	5.44834	6.05898	5.90792	6.64054	6.71193	7.26524	7.03079
SYCE1	Q8N0S2	6.72711	5.19812	7.32638	5.03428	6.97934	6.0511	5.85113	6.90577	6.39557	6.86937	7.54619	7.09362	6.17351	5.31392	5.24367	5.2909
SMG5	Q9UPR3	6.70137	6.10969	6.72643	6.23295	6.00349	6.65612	6.34168	6.36535	6.04345	6.66942	6.60385	6.80544	5.92464	5.68458	5.51222	5.61933
PRKCE	Q02156	6.81267	6.6725	7.19262	7.25303	6.10864	6.08989	7.62016	6.08374	6.56235	5.67778	5.7308	6.37093	4.83444	5.92658	5.4425	5.62137
TKFC	Q3LXA3	6.12611	5.90278	6.54592	5.80802	6.03204	6.64124	5.9469	6.97923	6.29566	6.21166	6.21645	6.53922	6.01325	6.58927	6.26353	5.88871
RALBP1	Q15311	6.23547	6.0885	6.3434	5.91821	6.0315	6.25424	6.30748	5.95867	6.88874	6.28986	6.33455	6.33376	6.18751	6.0847	6.19572	6.5477
RNF214	Q8ND24	6.18814	6.12732	6.32116	5.65662	6.55806	6.67803	5.86538	6.7522	6.51732	6.63435	6.68056	6.82363	6.59638	5.61408	5.47969	5.50707
ZNF507	Q8TCN5	5.85396	6.21267	6.41669	6.28555	5.5587	6.37286	5.81714	6.71291	6.4252	6.27068	6.87972	6.5137	6.04621	6.032	6.23946	6.36257
SALL3	Q9BXA9	7.32139	6.84328	6.80304	7.84493	6.72182	6.50559	7.15615	5.9605	6.2857	5.84826	6.63772	6.55091	5.26125	4.31149	4.81196	5.13601
EPN1	Q9Y6I3	6.05941	6.28004	6.62134	5.76097	6.27372	6.2981	6.00304	6.97016	5.93055	6.71441	6.35648	7.05552	5.90013	5.96799	5.86023	5.9479
SLC9A6	Q92581-2	6.02897	6.70004	6.32624	6.22004	6.31625	6.27807	6.25539	6.13951	6.21057	6.36822	6.27122	6.22724	5.946	6.37051	6.33845	6.00327
WDR55	Q9H6Y2	5.65408	6.00981	6.03421	5.62055	6.22941	6.296	5.6497	5.99288	6.50561	6.36816	6.43325	6.46749	6.87703	6.55361	6.56858	6.73962
ARHGEF28	Q8N1W1-6	6.62026	6.39164	7.13438	6.20487	6.15098	6.77053	6.46199	6.30912	6.73741	6.571	6.78676	6.65178	5.38788	5.11872	5.4732	5.22948
GET4	Q7L5D6	6.02041	6.24204	6.56309	5.84746	5.80234	6.57721	6.59528	6.17556	6.04829	6.53966	6.36106	6.56605	6.20261	6.16057	6.08301	6.21536
BZW2	Q9Y6E2	6.53399	6.06551	6.80181	6.03859	6.32291	6.31518	6.37979	6.21152	6.38036	6.35166	6.69985	6.54643	5.96316	6.03957	5.72972	5.61993
FNTA	P49354	5.99494	5.89401	6.73545	5.47269	6.52932	6.87738	5.99658	6.43805	6.28558	6.17118	6.48537	6.50765	6.34746	6.06427	6.07961	6.12046

RDH14	Q9HBH5	6.4054	6.227	5.81	6.44576	6.69357	6.15836	6.38159	6.25713	6.17817	6.17683	5.64567	5.91047	6.88507	6.3989	6.22619	6.19991
NDUFB10	O96000	6.19747	6.36086	5.87186	6.72943	6.68227	6.18822	6.57573	5.78217	5.8107	6.12281	6.50472	5.93762	6.49613	6.19948	6.49297	6.04756
ANTXR1	Q9H6X2	6.50264	6.35976	6.21711	6.6056	6.25637	6.17383	6.21667	5.91999	6.03799	6.13307	6.57731	6.39455	6.54601	6.31482	5.69044	6.05382
CBR3	O75828	6.24754	5.85953	6.85064	5.56639	6.88886	6.83902	5.90647	6.53262	6.4564	6.79129	6.70562	6.46495	6.04668	5.87635	5.5488	5.41884
hCG_1984214	I3L0E3	6.54404	6.52925	6.0124	6.80757	5.86711	6.32725	6.58922	6.23389	6.06146	6.29883	5.52489	5.99992	6.16828	6.35455	6.03263	6.6487
WWP1	Q9H0M0	6.28019	6.57204	6.69941	6.47104	6.19756	6.1943	6.48513	6.1043	6.47472	6.29625	6.49474	6.65837	5.97992	5.74225	5.80178	5.54801
RAB12	A0A3B3ITT1	6.32405	6.53249	6.13543	6.45162	6.19364	6.02541	6.03656	6.19796	6.31332	6.32082	6.21097	6.4592	6.01314	6.29367	6.00648	6.48525
RAB6B	Q9NRW1	6.14364	6.27636	6.11037	6.53751	6.39234	5.99952	6.52404	5.81965	6.20682	6.38866	6.31255	6.12027	6.15956	6.05768	6.50373	6.44731
RAB4B	P61018-2	6.28237	6.76173	6.20818	6.63047	6.409	6.67894	6.40593	6.19017	5.96825	6.52282	5.87432	5.84434	5.9677	6.01553	6.14386	6.09639
SPR	P35270	6.23148	5.31706	6.48968	5.2556	6.19565	5.85026	6.0439	6.78282	5.7602	6.72762	6.44684	6.8746	6.27494	6.60757	6.50082	6.64095
UBXN1	Q04323	6.47351	6.24904	6.80527	5.8523	6.38946	6.44132	6.00438	6.60991	6.48762	6.56271	6.98314	6.84457	6.12787	5.50499	5.2927	5.37121
TOLLIP	Q9H0E2	5.71644	6.01208	6.53805	5.31748	6.35732	5.81067	5.91444	6.67421	5.5896	7.15272	6.2765	6.82488	6.21292	6.76546	6.35816	6.47906
NAA20	P61599	6.15749	5.23433	6.37695	4.7821	5.94447	6.59707	6.11801	6.63797	6.20058	6.31154	6.61011	7.23982	5.74904	6.80613	6.39819	6.8362
PBK	Q96KB5-2	6.22366	6.32183	7.55529	6.07317	6.48487	6.29348	6.36133	6.8107	6.85771	6.75556	6.9018	6.7697	5.53536	4.98292	5.01382	5.05882
CROT	Q9UKG9-3	6.35898	5.93911	6.35316	5.84393	6.26758	5.85637	6.3235	6.1793	6.25935	6.24416	6.14543	6.00252	6.27769	6.44781	6.85864	6.64249
MGAT1	P26572	6.40999	6.75236	5.87184	7.16216	5.97445	6.21397	6.64408	5.19404	6.09928	6.31481	6.0511	6.07967	6.16122	5.92184	6.24472	6.18439
CUSTOS	Q96C57	5.90145	6.01177	5.8033	6.28358	5.88613	5.84194	6.20966	5.90176	6.24994	6.02935	6.35254	6.11154	6.75217	6.54595	7.08676	7.03217
TPBG	Q13641	6.22526	6.36524	6.53823	6.07303	6.30094	6.27239	5.99649	6.21198	6.08568	6.49698	6.61999	6.41564	6.71654	5.93535	6.05081	5.69547
GARRE1	O15063	6.33245	6.1953	6.63378	5.95168	6.63088	6.71576	6.09324	6.40807	6.20007	6.38928	6.36563	6.72013	6.34567	5.94735	5.63711	5.43361
RGS12	O14924	6.08424	6.31143	6.8752	6.14814	5.96742	5.92282	6.62058	5.84469	6.67542	6.48932	6.51493	6.78174	6.06402	5.73401	5.86839	6.09766
TAOK3	Q9H2K8	6.23328	6.16228	6.36152	5.76822	6.38505	6.34269	5.92166	6.12952	6.73622	6.64721	6.66676	6.10573	6.25523	6.19638	6.52949	5.55874
PARP9	Q8IXQ6-2	6.32281	6.56673	6.23774	6.22535	6.01698	6.45631	6.15201	6.30851	6.66691	6.82916	6.26971	6.28487	5.84446	5.85068	6.0211	5.94665
PRPF38B	Q5VTL8	6.2177	6.3993	5.59743	6.61048	6.31167	5.99708	6.16871	5.79277	6.10559	5.65118	5.99738	5.76531	6.62142	6.69737	7.19011	6.8765
EMG1	Q92979	6.03009	6.38107	5.70657	6.59112	6.54739	6.30258	6.15708	6.98344	6.03096	6.47264	5.97421	5.81107	5.96225	5.77886	7.1555	6.11516
SLC27A1	Q6PCB7	6.41624	6.40627	5.95633	6.55131	5.87758	6.06988	6.55842	6.24019	6.15477	6.16438	5.87848	5.83987	6.51897	6.45746	6.62323	6.28663
MTA3	Q9BTC8	6.37639	6.71365	5.97588	6.78862	6.14567	5.96146	6.12679	6.09117	6.48746	6.27161	5.87279	6.05634	6.40963	5.90465	6.35739	6.46049
UTP3	Q9NQZ2	6.15532	6.39386	5.81737	6.80988	5.85862	5.73245	6.51476	5.85976	6.09405	5.95578	5.87568	5.88973	6.66467	6.81959	6.59952	6.93107
CENPB	P07199	6.78063	6.49712	6.35609	7.18216	6.23768	5.82025	6.27678	6.04569	6.40397	6.30651	5.73626	6.14158	6.05529	5.93304	5.91956	6.30738
RAD23A	P54725	6.44994	6.27396	6.63682	5.35189	6.16068	6.3066	5.343	6.82671	5.84166	6.54266	6.84115	7.03638	5.84071	6.38576	6.00461	6.15747
FAR1	Q8WVX9	6.6658	6.84744	5.5624	7.06652	6.19108	5.89924	6.58525	6.17273	6.59166	6.56687	6.25777	5.95149	5.48803	5.56666	5.62365	5.96339
WARS2	Q9UGM6	6.62048	6.41106	6.32754	6.63569	6.67585	5.9627	6.6001	5.96171	6.33243	5.79328	5.97483	5.68849	6.44475	5.72193	6.68735	6.16181
CSTF1	Q05048	6.2834	6.59537	6.08474	6.35478	6.42995	6.13781	6.50017	6.22498	6.16288	5.99991	5.77744	5.50426	6.82357	5.93981	6.90198	6.27895
OSBPL11	Q9BXB4	6.45016	6.31838	6.58988	6.60552	6.17899	6.57336	6.08331	6.31832	6.48157	6.75147	6.75465	6.30094	5.77456	5.66009	5.43933	5.71947
ABCB10	Q9NRK6	6.3525	6.22884	6.12472	6.6215	6.05275	5.83994	6.43492	5.74245	6.13488	6.23536	6.14561	5.97272	6.65025	6.15833	6.88104	6.42418
POLD2	P49005	5.12695	5.24556	5.98504	5.07105	6.32404	6.2537	5.88601	6.63477	6.41815	6.89103	6.55969	7.01957	6.38705	6.21561	7.04502	6.93676
MINK1	Q8N4C8	6.02926	5.96533	6.66668	6.20707	5.90166	5.86989	6.14496	5.85141	6.79756	6.88438	6.53354	6.67784	6.15746	6.19402	6.23366	5.88528
TPST1	O60507	6.46856	6.62786	6.09687	6.77114	6.25139	6.0111	6.41217	6.21952	6.18436	6.12491	6.20191	5.9563	6.26833	6.0371	6.08536	6.28314
WDR76	Q9H967	6.5066	6.8613	5.70451	6.91544	6.73606	6.06573	6.38265	6.0123	5.97903	5.97017	5.93806	5.90285	6.33704	6.13176	6.47586	6.08064
DNAJB1	P25685	5.69924	5.8244	6.68457	5.68302	5.52874	6.3587	5.77261	6.33071	6.46016	6.57781	6.80085	6.70818	6.42645	6.3057	6.41378	6.42508
USP40	A0A7I2YQ75	6.51579	6.11159	6.79377	6.0745	6.10114	6.73183	6.24456	6.70782	6.09022	6.51936	6.92208	6.39548	5.62489	5.8592	5.65154	5.65624
AKT1	P31749	6.36737	6.18337	7.03211	5.9699	6.03819	6.68568	6.079	6.28217	6.38587	6.4561	6.5328	6.60234	5.69908	6.03652	5.90255	5.74695
POMGNT1	Q8WZA1-2	6.27421	6.63191	5.9523	6.89982	6.34354	6.0395	6.34601	6.31606	6.56624	6.47659	5.78025	6.21441	5.98747	5.59926	6.25859	6.31384
GCDH	Q92947	5.97295	6.40868	6.04926	6.67065	6.03438	6.15665	6.25188	6.02457	5.57197	6.11009	6.23514	6.17024	6.90087	6.33016	6.57148	6.54102
EFR3A	Q14156	6.21687	6.5136	6.14329	6.58885	6.28746	6.2055	6.24874	6.4989	6.14242	6.09063	6.09129	6.13635	6.13743	6.03901	6.45259	6.20717
PAXIP1	Q6ZW49	6.34523	6.56835	6.29162	6.95912	6.34407	5.98305	6.25764	5.90521	6.24102	5.83585	5.7258	5.99776	6.57465	6.26903	6.34419	6.35741
CSTB	P04080	5.52202	5.19501	6.35759	4.39332	6.43549	6.41938	5.44548	7.18861	5.53734	7.00778	6.20843	6.68386	5.69999	8.00296	6.56454	7.3382
CCDC18	E9PFB9	6.26856	6.35365	6.82259	6.6354	6.56091	6.47717	6.19936	6.8553	6.77857	6.70148	7.03792	7.0812	4.9368	5.16782	4.89939	5.22388
EGLN1	Q9GZT9	6.15953	6.22536	6.72078	6.24409	6.28152	6.33552	5.87198	6.35709	6.34446	6.68625	6.69475	6.80767	6.06447	5.84652	5.78665	5.57335
PPIP5K2	O43314	5.94423	5.73929	6.73867	5.89501	6.20939	6.60583	6.05118	6.4145	6.26779	6.67294	6.39932	6.6681	6.02711	6.32817	6.04093	5.99756
CEP63	Q96MT8	6.43522	6.45435	6.46853	6.7274	6.46133	6.3048	6.369	6.31693	6.2542	6.17185	6.39577	6.44211	6.06083	5.696	5.74502	5.69665
GCC1	Q96CN9	7.09834	6.18618	6.00563	6.43455	7.03703	6.11075	6.31446	6.38975	6.53707	6.12898	6.27414	6.08551	6.64685	5.85614	5.51298	5.38163
C9orf64	Q5T6V5	6.18367	6.00274	6.72761	5.62886	6.42189	7.29152	6.00993	6.60789	6.56338	6.6604	6.38346	6.44622	5.48523	6.08986	5.76014	5.73719
IMPACT	Q9P2X3	6.54747	6.65578	7.00969	5.87641	6.3525	6.83258	6.32332	6.45169	6.20214	6.45922	6.23523	6.50492	5.42691	5.92	5.71012	5.49203
MAK16	Q9BXY0	6.43054	6.31543	5.86018	6.61692	6.32124	5.84417	6.31863	5.82244	6.22113	6.08405	5.93777	5.88417	7.07692	5.9472	6.57757	6.74164
FLAD1	Q8NFF5	6.40359	6.07686	6.42083	6.39729	6.06289	5.84573	6.0049	6.31686	6.225	6.13345	6.24409	6.19855	6.56653	6.21531	6.58518	6.30294
GPD1L	Q8N335	5.97188	6.1535	6.81877	5.84183	6.23057	6.4148	6.5136	6.53074	6.15806	6.41508	6.23675	6.7738	5.98788	6.30979	5.68504	5.9579
KIF13A	Q9H1H9-4	6.6456	6.41965	6.84174	6.2989	6.29276	6.84707	6.06111	6.71853	6.54021	6.53943	6.53688	6.95305	5.34094	5.14438	5.35324	5.46649
KIF1C	O43896	6.65831	6.46226	6.78925	6.15314	6.36178	6.45034	6.18874	6.62153	6.31534	6.56404	6.40277	6.37462	5.69815	5.7364	5.56485	5.65848
IPO13	O94829	5.75391	5.98218	6.16253	5.17427	5.92828	6.41602	5.82036	6.07197	6.58044	6.44832	6.24828	6.24416	6.5595	6.88156	7.01849	6.70973
LYPLA1	O75608	5.66194	4.70677	6.03266	4.71312	6.39554	5.75775	5.95281	6.9827	5.75894	6.74068	5.82337	6.50086	5.76815	7.7006	7.58771	7.9164
SNX5	Q9Y5X3	6.51764	6.56312	6.95802	5.97507	6.4094	7.03104	6.02165	6.68911	6.49383	6.34457	6.47731	6.78666	5.80923	5.30209	5.43206	5.18919
MYBL1	P10243	5.97408	5.88535	5.94076	6.30196	6.47741	6.22168	6.22191	5.96634	6.05456	6.01978	5.65942	5.68911	6.98165	6.56839	6.8885	7.14912
ARMCX3	Q9UH62	6.41304	7.15942	5.92806	6.51263	6.3702	6.48805	6.1394	6.23783	5.96824	6.30317	5.95433	5.75654	6.26529	6.1846	6.50207	5.81712
INTS12	Q96CB8	6.10081	6.10288	6.11586	6.50727	6.61968	6.10223	6.47884	5.99323	5.91319	6.32597	6.15056	5.97632	6.53652	6.13694	6.36749	6.57223
ITPA	Q9BY32	6.70147	5.81171	6.67875	4.87796	6.92576	6.42647	6.09877	7.30548	6.21899	6.78325	6.05293	6.47998	6.22588	6.24598	5.67197	5.49463
DMXL1	Q9Y485	6.34945	6.25875	6.83969	6.55136	6.2903	6.40453	6.3453	6.70877	6.60188	6.3023	6.94513	6.95222	5.41046	5.48446	5.27492	5.28048
UBR3	Q6ZT12-4	5.84579	6.35341	6.62696	5.95151	5.96095	6.52749	6.30753	6.02239	6.33038	6.50544	6.53279	6.51503	6.02238	6.29506	6.29133	5.91157
ABHD16A	O95870	6.57648	6.44041	5.72196	6.7585	6.09071	5.89829	6.09299	6.3339	5.76898	6.15138	6.14946	5.97914	6.26196	6.55358	6.64653	6.57572
NIF3L1	Q9GZT8	5.93223	6.07848	6.61981	5.76286	6.05668	6.40838	6.25333	6.33404	6.29744	6.13714	6.64867	6.25143	6.42724	6.18017	6.31055	6.30156
GCFC2	P16383	5.73831	6.11722	6.03568	6.09549	5.92814	6.26801	6.19662	6.34181	6.06512	6.25321	6.38481	6.16114	6.77097	6.31809	6.73187	6.59351
CSNK1E	P49674	6.88286	6.94908	6.84777	7.14241	6.51589	6.34939	6.35075	6.48709	6.65163	6.72124	6.60188	6.66774	4.89917	4.85193	4.96772	5.11345
SPATA20	Q8TB22-2	6.64878	6.56927	5.88351	6.39943	6.15365	5.99135	6.43743	6.09569	5.888	5.94537	6.23653	5.90746	6.68445	6.38421	6.39937	6.37551
GIGYF1	O75420	6.74859	6.60746	6.93071	6.30605	6.47276	6.37891	5.90696	6.86954	6.65937	6.72935	6.8517	6.72536	5.52415	5.35246	4.87323	5.0634
BIN3	Q9NQY0	6.34738	5.90431	6.56432	5.70367	6.15606	6.72267	5.83382	7.10909	6.40812	6.85959	6.66088	6.82103	6.15632	5.85632	5.23027	5.66615
ABCC3	O15438-4	6.39046	6.26779	6.41558	6.27561	6.21185	5.86365	5.87166	5.63262	6.22328	6.10713	6.03845	6.08869	6.78626	6.43551	6.88538	6.50608
ADPRS	Q9NX46	6.06056	5.74757	6.30375	5.92745	6.23061	6.0859	6.55845	6.07739	6.70839	5.6713	5.92206	6.59174	6.18031	6.68706	6.35594	6.89153
PACSIN3	Q9UKS6	6.36334	6.68379	6.29947	6.2755	6.58257	6.59068	5.85755	6.73227	6.02341	6.07425	6.10161	6.29495	6.20113	5.89654	5.91211	6.11085
MRPL9	Q9BYD2	6.38027	6.47354	6.00636	6.53932	6.61842	6.11127	6.95036	5.93171	6.25158	5.85439	5.5751	5.76749	6.40664	6.03248	6.61179	6.48926
SDF2L1	Q9HCN8	6.6236	6.66391	5.51371	6.24438	6.82691	6.23932	5.81402	6.54344	6.14535	6.11313	5.84046	5.96424	6.81334	6.17674	6.12931	6.34814
TOR1A	O14656	6.71252	6.58508	6.18556	6.47118	6.14371	6.16521	6.41266	5.97196	5.99726	6.41632	6.12867	6.58274	6.55127	5.9333	6.47436	6.1682
KBTBD2	Q8IY47	5.83198	6.05467	6.55553	5.70482	6.03699	6.61681	6.23836	6.16695	6.44379	6.63494	6.4077	6.19005	5.96669	6.25471	6.73706	6.15896
MRPL40	Q9NQ50	6.05902	6.40743	6.00193	6.7209	6.48695	6.15376	6.77548	6.10587	6.21184	6.01881	6.13453	5.90903	6.3749	5.97008	6.06684	6.60263
NSMCE2	Q96MF7	6.27387	6.45809	6.59545	6.63701	6.31427	6.2351	6.43752	5.9918	6.07463	6.15903	6.15688	6.20528	6.26761	5.83161	6.11286	6.249
RAD51	Q06609	6.77994	6.09757	6.48875	6.76887	6.33148	6.1038	6.59959	6.14681	6.7139	6.22723	6.6569	6.75999	5.30363	5.51617	5.49178	6.01359
PTER	Q96BW5	5.89871	5.65878	6.57522	5.13745	6.80005	6.64007	6.13294	6.68181	6.649	6.53844	6.73091	6.35795	6.14046	5.94009	6.14187	5.97625
PTPN14	Q15678	6.09431	6.40155	6.6067	6.2469	6.0179	6.59744	6.242	6.49823	6.25644	6.39649	6.68837	6.7424	5.78768	5.83425	5.95487	5.63448
TCAF1	Q9Y4C2	6.76538	6.71681	7.43198	6.40977	6.23359	6.43747	6.42784	6.51388	6.86947	7.11347	6.96589	7.23731	4.63638	4.99963	4.43454	4.80659
TRPC4AP	Q8TEL6	6.29257	6.40692	7.0893	6.08226	6.4223	7.11996	6.83919	6.83839	6.83641	6.29474	6.63964	6.34072	4.94708	5.20476	4.89827	5.74748
C11orf68	Q9H3H3-3	6.22465	6.21274	6.69639	5.67623	6.59075	7.03775	5.96059	6.72137	6.45142	6.39703	6.58472	6.52191	5.85604	5.71568	5.82851	5.52422
BRAP	Q7Z569	6.37371	6.26025	7.02636	6.27543	6.35375	6.81732	6.1687	6.68582	6.60442	6.32601	6.51285	6.75427	5.54052	5.57018	5.2781	5.45232
ATP6AP2	O75787	6.0341	5.59949	5.72622	5.91846	6.30064	5.65106	6.58739	6.3325	6.02323	6.35848	5.98794	6.56314	6.36007	6.59616	7.01827	6.94285
CEP85	Q6P2H3	7.25429	6.27132	7.24874	6.33488	6.57179	6.75152	6.16272	6.71468	6.44654	6.71895	6.96969	7.14464	5.13779	4.83495	4.39262	5.04487
STAT2	P52630	6.55659	6.59932	6.92657	6.07021	6.41117	7.20852	6.4137	6.65144	6.51328	6.84633	6.23743	6.49463	5.23299	5.51698	5.1683	5.15255
MALT1	Q9UDY8	6.28736	5.98985	6.92344	6.62986	6.31294	6.70935	6.23436	6.51669	6.63092	6.42815	6.9371	7.09707	5.41123	5.13543	5.25896	5.4973
PPM1F	P49593	6.36717	5.77658	7.08372	5.86564	6.38514	6.72569	6.08936	6.5626	6.58501	6.54481	6.49018	6.2742	5.82944	5.82929	5.59931	5.99187
SMTN	P53814-5	5.78985	6.22052	6.27453	5.89617	5.84512	6.18234	6.05401	5.94434	6.32637	6.16791	6.6936	6.69167	6.36198	6.23877	6.51425	6.79859
RASA2	Q15283	6.41482	6.68355	6.75032	6.45242	6.67833	6.64392	6.25777	6.1965	6.31846	6.25801	6.76713	6.2965	5.66157	5.74804	5.56002	5.31265
MRPS18B	Q9Y676	6.26561	6.73895	5.9854	6.60677	6.26586	6.05669	6.57372	6.39032	6.08848	5.91195	6.28547	6.14178	6.52331	6.0972	6.11243	5.95607
ZNF106	Q9H2Y7	6.31868	6.5022	6.24032	6.96653	5.84306	5.90144	6.60058	6.17401	6.3989	6.195	6.25276	6.20876	5.77242	5.97774	6.25205	6.39656
SUOX	P51687	6.45907	6.21798	5.99136	6.55152	6.16176	5.90405	6.24794	5.91874	6.13914	6.34472	6.33333	6.18403	6.42911	6.2913	6.40297	6.42297
PPOX	P50336	6.13227	6.5756	6.24281	6.63074	6.37528	5.94027	6.05368	6.02612	5.99131	5.90548	5.66461	6.22644	6.47136	6.86536	6.50454	6.39412
PHC2	Q8IXK0-5	6.00518	6.03969	6.26989	6.99535	5.84118	6.30136	6.44419	5.92153	6.08781	6.33354	6.16228	6.50448	6.02666	5.94501	6.595	6.52684
KIFAP3	Q92845	6.12916	6.03986	7.10567	6.01991	6.5723	6.80297	6.27377	6.58357	6.65229	6.63431	6.58499	6.08036	5.77404	5.53095	5.54998	5.66588
EML1	O00423-3	6.53379	6.24171	6.70869	5.72679	6.38221	6.73222	6.01824	6.53617	6.46687	6.49026	6.38812	6.34336	6.00021	6.22556	5.67871	5.52709
ZNF654	A0A1B0GWA0	6.32971	6.52024	6.47831	6.83203	6.31799	6.21984	6.48942	5.92905	6.15589	6.24663	6.205	6.38875	6.12974	5.50411	6.18214	6.07116

ZC3H3	Q8IXZ2	6.5481	6.53974	6.28293	6.9069	6.16526	6.26364	6.56395	6.60948	6.66076	6.61056	6.55821	6.42283	5.23277	5.63633	5.28549	5.71305
SCAMP1	O15126	6.17019	6.28701	6.14106	6.17702	6.00767	5.96666	6.30503	6.3001	6.44898	6.48665	6.10236	5.90878	6.06729	6.17702	6.76235	6.69182
AMOT	Q4VCS5	6.89576	6.57633	6.15724	6.18827	6.55165	6.44747	6.25328	5.99977	6.45385	6.64354	6.48204	6.48839	6.01372	5.73605	5.41784	5.69479
FNBP1L	Q5T0N5	6.08237	6.31517	6.89409	6.23393	6.3351	6.72241	6.2514	6.57105	6.54429	6.61811	6.53899	6.51548	5.89458	5.80739	5.44552	5.23011
METTL3	Q86U44	5.53267	5.53317	5.99012	5.84791	6.35146	6.62643	6.03927	5.74791	6.28333	5.93499	6.44979	6.05689	6.88181	6.81587	7.26883	6.63953
SRRM1	Q8IYB3	5.70263	6.39131	5.59819	6.53444	5.61888	5.78324	5.85058	5.88084	5.8023	6.00609	5.91625	6.31831	6.59454	7.49464	6.96564	7.54212
C7orf50	Q9BRJ6	5.87521	6.32302	5.76461	5.92114	5.94266	5.75474	6.01651	6.02585	5.9784	6.49906	6.07575	5.87475	6.59321	7.1126	7.38455	6.85796
RBM45	Q8IUH3	6.67809	6.55161	5.68056	6.54611	6.45744	6.23432	6.25059	5.88447	6.33746	6.08286	6.31773	6.02881	6.68424	6.11634	6.09878	6.0506
TRPS1	Q9UHF7-2	6.3329	6.47005	5.99501	6.46958	6.08667	6.17214	6.28358	6.23381	6.26342	6.33599	5.99492	6.1567	6.37566	6.28342	6.20154	6.3446
PRAME	P78395	6.0343	6.81626	7.23493	6.66378	6.36637	6.65725	6.56329	6.04722	6.48102	7.32794	7.34021	6.87247	4.45556	5.40381	4.71438	5.02121
CHTOP	Q9Y3Y2-3	5.88476	6.56402	4.9889	6.13235	5.4171	5.71223	6.34379	6.34116	7.25499	5.30306	6.07315	5.73482	7.59304	6.73442	6.02526	7.89694
RBM5	P52756	6.30691	6.14506	6.14293	6.48383	6.31127	6.16085	6.74857	6.22872	6.57743	6.35426	6.28429	6.05943	6.26478	6.16416	6.00804	5.75947
FAN1	Q9Y2M0	6.20707	6.28923	6.4056	6.29364	6.47577	5.97246	6.30297	6.01788	6.38482	6.06704	6.29097	6.35356	6.09123	5.94605	6.32118	6.58053
PTGR1	Q14914	6.00106	6.07356	6.65331	5.93619	6.30297	6.86731	6.43962	6.5083	6.242	6.3759	6.50545	6.38761	5.63622	6.38213	5.95369	5.73469
TWNK	Q96RR1	6.01203	6.32286	6.36976	6.97289	6.02306	5.80705	6.45073	6.01541	6.33931	6.13288	5.98457	6.36762	6.26128	5.89003	6.58639	6.46413
ILKAP	Q9H0C8	5.21429	5.54758	6.34816	5.37342	5.60285	6.1783	5.72817	5.86534	5.88262	6.53688	6.15211	6.44181	7.02603	7.52104	7.12671	6.98564
TEX2	Q8IWB9-2	6.57324	6.51437	5.99842	6.67129	6.06572	6.20323	6.28796	6.1419	5.52325	5.92515	5.76089	5.92732	6.49032	6.41143	6.75906	6.74643
HMBS	P08397	6.11185	5.81184	6.96844	5.76754	6.11877	6.43415	5.81553	6.21465	6.56642	6.41051	6.83587	6.44637	6.01261	6.37716	6.16282	5.94548
L3MBTL3	Q96JM7-2	6.26031	6.10178	5.7285	6.6032	6.25494	5.93443	6.51569	6.05042	6.15361	6.1855	5.97161	6.04278	6.72433	6.34136	6.539	6.59255
AK1	P00568	6.32607	5.93811	6.67297	5.41227	6.74943	6.88063	6.40098	6.93776	6.44028	6.43907	6.56404	6.39518	5.95381	6.06231	5.46353	5.36356
WDR4	P57081	5.34865	5.40384	5.86077	5.2989	5.51964	6.25534	5.53339	5.74351	5.90313	5.82353	5.8501	5.66639	7.43888	8.02092	8.82514	7.50787
ANKS1A	Q92625	6.33824	6.20903	6.66607	6.11616	5.99898	6.65444	6.28006	6.56311	6.5815	6.5567	6.35389	6.60173	5.87879	6.52396	5.718	5.85934
FDPS	P14324	6.15539	6.1899	6.3032	5.61099	6.43761	6.99182	5.72841	7.04404	6.03041	6.25483	6.17926	6.2768	5.77846	6.74039	6.3549	5.9236
NEDD4L	Q96PU5	6.25423	6.11935	6.65985	6.14416	5.89415	6.8449	6.24438	6.56572	6.58297	6.52869	6.79018	6.61369	5.62726	5.66684	5.54455	5.91908
RAB5B	P61020	6.73239	6.36775	6.14914	6.19419	6.07128	6.02799	6.75968	6.26027	6.33391	6.39872	6.00492	6.03482	6.17998	6.19026	6.10425	6.19045
SCARB2	Q14108	6.72511	6.93387	6.04842	6.40611	6.21237	6.03515	6.45579	6.15529	6.01518	6.45356	5.75358	5.98057	6.46077	5.85841	6.4129	6.09292
CPNE2	Q96FN4	5.23437	5.67283	5.86708	5.01906	5.88497	6.19354	5.57629	5.69132	6.68391	6.97473	6.69187	6.48092	6.1612	6.80865	7.48294	7.57632
TMLHE	Q9NVH6-8	6.47694	6.14389	5.96223	6.5479	6.42217	6.02246	6.18509	5.98761	6.15341	6.22894	6.10988	5.83277	6.6109	6.14287	6.76922	6.40372
RAC1	P63000-2	6.60636	6.06532	6.56615	6.12237	6.56308	6.55421	6.35309	5.87149	6.24528	6.05982	6.51765	6.121	6.32611	5.9643	5.98568	6.07809
ATG16L1	Q676U5	5.97731	6.00545	6.65687	6.28438	6.18756	6.3358	6.36565	6.4027	6.29231	6.24465	6.92978	6.82154	5.74736	5.9008	6.01515	5.83269
PTPA	Q15257	6.22326	6.14456	6.65396	5.59168	6.25209	6.63886	6.03938	6.89382	6.36089	6.48333	6.48669	6.64809	5.72447	6.23847	5.64499	5.97545
DPYD	Q12882	6.29066	6.39547	7.31456	6.05191	6.65666	6.13918	6.37548	6.42456	6.55293	6.54712	6.17857	6.13369	5.59779	6.22716	5.55728	5.55701
ECI1	P42126	5.75891	4.89791	5.04349	5.0446	5.66725	4.96137	6.07792	6.57464	4.78443	5.44997	5.02763	6.22539	6.27377	9.22428	9.59385	9.39458
CA2	P00918	5.98982	6.24681	7.08806	5.57361	6.61503	7.17467	6.37612	6.82254	6.64806	6.2157	6.24931	6.5477	5.85797	5.78629	5.52534	5.28299
IKBIP	Q70UQ0	6.14044	6.56563	6.06599	6.64423	6.00238	6.01148	6.28883	6.10562	6.16382	6.24354	6.19066	6.12185	6.41524	6.07823	6.4778	6.48426
DLG5	Q8TDM6	7.02956	6.53491	6.51356	6.88254	6.31048	6.36904	6.53949	6.3806	6.26472	6.1134	6.02156	6.38699	5.89921	5.66136	5.47042	5.62217
SLC16A3	O15427	5.8119	6.05388	6.04253	6.19388	6.01365	6.03296	6.44605	5.88608	6.2119	6.27323	6.07589	6.10519	6.53918	6.7365	6.63004	6.94714
CENPI	Q92674	6.34252	6.66967	6.09956	7.16407	6.45645	5.99589	5.9978	6.02056	6.11452	6.18212	6.38381	6.3405	6.13208	5.81873	6.16841	6.1133
M6PR	P20645	6.21826	6.45007	6.45056	6.49088	6.18423	6.01027	6.0073	5.73467	6.52371	6.66232	6.66148	6.58671	6.16705	5.6702	6.1583	6.024
GXYLT1	Q4G148	6.61308	6.7205	5.9189	6.7998	6.29929	6.03648	6.41552	6.16049	6.00426	6.18088	6.1229	6.03501	6.53008	5.95927	6.23677	5.96675
ADA	P00813	6.19815	5.25204	7.1177	4.9774	6.76751	6.69992	6.069	6.43137	6.82848	6.43127	6.89848	6.77692	6.33904	6.14951	5.50373	5.55946
USE1	Q9NZ43	6.1009	6.60954	5.88884	6.51185	6.01811	5.92224	6.4587	5.87664	5.97137	6.30654	6.1397	6.209	6.44029	6.32016	6.75253	6.47358
SDE2	Q6IQ49	6.11166	6.2647	6.45453	6.3538	6.28613	6.11124	6.27958	6.13823	6.57588	6.25113	6.65581	6.3349	6.23928	5.85146	6.02925	6.06242
CFAP36	Q96G28	6.79146	6.47231	7.34685	5.79837	6.39503	6.54521	5.9393	7.13384	6.61693	6.78692	6.97187	6.70217	5.4792	4.93036	5.00723	5.08288
ZBTB40	Q9NUA8	6.25736	5.99461	6.2611	6.80049	5.83531	5.90511	6.63047	5.74426	6.20042	6.15305	6.00278	5.96068	6.76206	6.28052	6.58712	6.62464
PPIG	Q13427	6.20014	6.28319	5.96276	6.55282	6.06055	5.87736	6.30609	5.98528	6.23445	5.86204	5.76525	5.84192	6.54501	6.66694	6.9733	6.88288
LTBP3	Q9NS15	5.99357	6.45844	5.92163	6.21623	6.19338	6.22394	6.51277	6.03717	6.2519	6.57056	6.34694	6.3268	6.5927	6.27646	5.98165	6.09587
ATG4B	Q9Y4P1-2	6.00822	5.96146	7.17323	5.82241	6.12693	6.93795	6.45101	6.40992	6.47894	6.65982	6.99949	6.93964	5.51992	5.63847	5.38301	5.48958
WDR92	Q96MX6	5.95453	6.11188	6.6203	5.76172	6.40914	6.54838	6.16107	6.60206	6.52449	6.83382	6.4277	6.45164	6.17926	5.9297	5.81389	5.67042
TIMM13	Q9Y5L4	6.08821	5.20568	5.54765	5.21352	5.9793	4.37865	5.12711	6.66694	4.53286	5.67266	4.94555	5.68085	5.31273	10.5839	8.61635	10.448
CIAO1	O76071	5.88789	6.37574	6.3403	6.149	6.28155	6.92352	6.08877	6.5198	6.53098	6.74988	6.35651	6.44133	5.83124	5.87497	5.82872	5.8198
FAM114A1	Q8IWE2	6.63412	5.8923	6.93108	5.66578	5.95648	6.73713	6.02061	6.52829	6.61579	6.32268	6.94352	6.57038	6.3058	5.59302	5.42064	5.86236
TTI1	O43156	6.66487	5.90355	6.59233	6.06473	6.20064	6.74796	6.00886	6.19749	6.53199	6.6904	6.8726	6.40639	5.8328	6.16509	5.3372	5.7831
SNX12	Q9UMY4-1	5.94531	5.59263	6.46377	5.37392	6.42645	6.49481	5.92106	6.61905	6.43706	6.92691	6.70773	6.90154	6.25323	6.08163	5.85395	6.00097
ALAS1	P13196	6.50969	6.44384	6.19069	7.07218	5.80684	5.8244	6.1116	5.4471	7.54302	6.69187	6.63599	6.89553	5.99452	5.28204	5.65457	5.8961
AKAP17A	Q02040	6.2829	6.22412	6.25539	6.25785	6.20729	5.92771	6.41663	5.32148	6.0114	5.83164	6.0552	5.59144	6.84987	6.97332	7.40509	6.38866
ANP32A	P39687	5.78394	5.73265	6.03373	5.30774	6.12176	6.24234	5.46345	6.18026	6.02975	6.20784	6.44156	6.205	7.05438	7.46789	6.97172	6.756
HEG1	Q9ULI3	5.93443	6.76808	6.4437	6.99011	6.06765	6.40535	6.10544	6.2901	6.07585	6.50497	6.42788	6.37122	5.82181	5.84833	5.63567	6.3094
ERAL1	O75616	6.32315	6.385	6.0603	6.3746	6.49612	6.33189	6.60018	6.00326	6.06437	6.11966	6.42654	6.10397	6.30081	5.97558	6.1294	6.30517
EXD2	Q9NVH0	6.27825	6.30499	6.11985	6.6577	6.08767	5.89701	6.60058	5.92228	5.95321	6.25173	5.95541	5.81075	6.58309	6.45154	6.70853	6.41742
TTC21B	Q7Z4L5	6.71154	6.64035	6.68478	6.12919	6.42276	6.40636	6.2302	6.52259	6.55558	6.5366	6.53287	6.37471	5.61562	5.62178	5.54659	5.46849
PIN1	Q13526	6.29298	6.09223	6.40217	6.09926	5.91462	6.87262	5.73457	6.26661	6.38356	6.37939	6.50022	6.86729	6.11538	6.1663	5.78649	6.12631
CNST	Q6PJW8	5.91028	6.19635	6.29358	6.87428	6.04936	6.05871	6.3036	6.09076	6.25689	6.37406	6.41658	6.11553	6.224	5.94186	6.3073	6.58687
HSDL1	Q3SXM5	6.49851	6.20161	5.93821	6.74537	5.9875	6.21251	6.24006	5.80593	6.0097	5.98681	6.20234	5.96028	6.757	6.4845	6.36016	6.60951
TRIM14	Q14142	6.19182	6.60601	6.31866	6.45036	6.26384	6.30646	6.57925	5.91737	6.50567	6.11846	6.7928	6.6172	5.68775	5.25136	6.13475	6.25825
SLC1A5	Q15758	6.35096	6.15624	5.90395	6.42275	6.41728	6.13357	6.51115	6.14325	6.59239	5.5927	6.23593	6.13512	6.59303	6.05634	6.20533	6.55002
TBL1XR1	Q9BZK7	6.0781	6.05325	6.08066	6.34332	6.36762	6.4081	5.87251	5.99369	6.24137	6.56347	6.27505	6.44065	6.37917	6.05222	6.475	6.37581
CFAP298	P57076	6.54553	6.38594	7.05722	6.27715	6.76782	6.87809	6.72274	6.90203	6.77756	6.58909	6.8507	6.75174	5.05471	4.77379	4.76496	4.90094
AEBP2	Q6ZN18	6.54771	6.69153	6.69231	6.57887	6.16215	6.20676	6.33618	6.20902	6.66272	6.24867	6.36434	6.61082	5.68103	5.80755	5.56336	5.63698
HMCES	Q96FZ2	6.27321	6.02906	6.53279	5.81275	5.91569	6.15414	5.80228	6.21141	6.4415	6.51212	6.86149	6.54257	5.97128	6.83542	6.10957	5.99471
KDM6A	O15550	6.346	6.30819	6.01828	6.56019	6.1127	6.02557	6.09336	5.61444	6.35354	6.21503	6.11489	6.06788	6.77487	6.44493	6.58703	6.3631
LIN28B	Q6ZN17	6.01479	6.3973	6.1704	6.39942	6.18327	6.2076	6.11689	6.49119	6.29017	6.14135	6.3302	6.06614	6.05423	6.52018	6.41122	6.20566
SPAG7	O75391	5.95775	5.82821	6.5812	5.75634	5.86006	6.54034	5.624	6.1682	6.31816	6.48771	6.56327	6.53567	6.5231	6.71372	6.14668	6.39559
DCK	P27707	5.95615	5.34612	6.99249	5.59313	6.28145	6.05412	6.28308	6.36236	6.4697	6.51846	6.46074	6.18078	6.28461	6.49932	6.73055	5.98695
AIDA	Q96BJ3	5.96434	6.24891	6.28303	6.13355	6.24835	6.11325	6.45379	6.31273	6.22806	6.35169	6.77304	6.51218	6.4814	5.89749	6.04713	5.95105
PLIN2	Q99541	6.21284	5.76994	6.26611	6.58184	6.17404	5.84648	6.20135	6.28148	6.05274	5.87675	5.83066	5.98598	6.75616	6.75604	7.05156	6.35605
ASAH1	Q13510-2	6.43572	6.54082	5.83376	6.24583	6.16977	6.02492	5.98621	6.22596	5.79434	6.43566	6.12158	5.95545	6.87251	6.46931	6.22542	6.66271
PIK3CA	P42336	6.39931	6.19796	7.13639	6.0004	6.44942	6.56003	6.19754	6.2693	6.69577	6.6192	6.65585	6.68206	5.6841	5.51444	5.58428	5.35396
EXOC3	O60645	6.45816	6.29974	6.73781	5.91905	6.65831	6.42409	6.49464	6.36672	6.31819	6.49953	6.34535	6.59752	5.86742	5.97528	5.62739	5.41081
PIGS	Q96S52	6.34032	6.36068	6.26221	6.33529	6.24632	5.9337	6.46578	5.9169	6.11943	5.93539	6.06427	5.88419	6.62558	6.02491	6.89421	6.59083
TXNDC12	O95881	6.22683	5.04455	5.39069	5.2308	6.00593	5.18965	5.68222	6.43725	5.44787	6.54972	5.62833	6.48019	6.87497	7.98057	7.91789	7.91253
KLHDC4	Q8TBB5	6.37956	6.33149	6.84994	6.29711	6.26244	6.28222	6.28141	6.5729	6.31943	6.47349	6.38735	6.54953	5.3326	6.02968	5.90301	5.74785
NUP58	Q9BVL2	6.56612	5.90897	6.18137	5.95261	6.52237	5.95129	6.18555	6.52301	5.99906	6.15726	6.18877	6.2291	7.14995	6.20866	6.21169	6.06422
MIER1	Q8N108-12	6.26337	6.27347	6.39109	6.03634	6.27091	6.09541	6.01783	6.17387	6.13622	5.89314	6.25528	6.21166	6.53469	6.48469	6.51085	6.45115
RABL6	Q3YEC7	6.10411	6.45418	6.85959	6.16595	6.10184	6.296	5.92621	6.41931	6.02788	6.41727	6.58873	6.45512	5.38844	6.56676	5.81919	6.40943
DCAF6	Q58WW2-3	5.69227	6.32887	6.08428	5.60838	6.44571	6.17658	5.93902	6.02764	6.12832	6.00342	5.72883	6.14762	6.82487	6.81021	7.14081	6.91319
ARIH1	Q9Y4X5	6.06197	6.17502	6.54619	5.71035	6.17726	6.43666	6.11255	6.58891	6.21236	6.27804	6.42737	6.15319	5.95861	6.4834	6.3083	6.36981
HDAC3	O15379	6.60854	6.50966	6.48101	6.59609	6.41373	6.25643	6.27801	6.12225	6.14628	6.14466	6.27767	6.25061	6.09671	5.75854	6.12228	5.93756
MRPL22	Q9NWU5	6.54769	6.53562	5.78078	7.08897	6.26576	5.90889	6.70126	5.86006	5.97573	6.07078	5.8104	5.63853	6.50355	6.15349	6.55511	6.60336
USP25	Q9UHP3	6.42528	6.17247	6.87894	6.2493	6.29046	6.39839	6.40066	6.53031	6.14503	6.3508	6.5837	6.634	5.64655	5.8579	5.67386	5.76236
ENOPH1	Q9UHY7	6.15741	6.05115	6.90604	5.60619	6.25843	6.95299	6.10531	6.68773	6.37201	6.58758	7.03389	6.79442	5.68289	5.77325	5.56417	5.46654
CEP41	Q9BYV8	6.34225	6.32356	6.3477	6.01566	5.9729	6.98047	6.1755	6.67808	6.35751	6.81145	6.57635	6.70921	6.06051	5.64702	5.37691	5.62492
POGLUT2	Q6UW63	6.46288	6.1622	6.00705	6.61056	6.38144	6.15676	6.29117	5.69112	6.2797	6.03248	6.17533	5.82474	6.49686	6.4125	6.6262	6.389
SIRT6	Q8N6T7	5.90358	6.44342	6.13814	6.0262	6.22219	6.14021	6.04063	6.03792	6.34694	6.14645	6.37467	6.06065	6.65232	6.42702	6.63601	6.40366
TOR1B	O14657	6.468	6.63908	5.98677	6.62603	6.47465	6.20567	6.12216	6.17645	6.11408	6.2301	6.07414	6.15445	6.53665	6.00872	6.22326	5.95981
STX7	O15400	6.29019	6.11018	6.16551	5.89782	6.24338	6.02384	6.17458	6.26124	6.25255	6.5871	6.52905	6.13199	6.18958	6.31365	6.37394	6.45538
ATG2B	Q96BY7	6.18829	6.18053	6.86648	5.85046	6.47965	6.56701	6.11354	6.55967	6.81277	6.97243	6.62423	6.80719	6.27518	5.70786	5.44437	5.55032
ISY1	Q9ULR0-1	6.64159	6.68088	6.09247	6.68871	6.17513	6.28055	5.96368	6.46043	6.00864	6.1228	6.51607	6.68329	6.27546	5.75068	5.82792	5.8317
NCKIPSD	Q9NZQ3	6.24456	6.54153	6.98524	6.41392	6.26773	6.34713	6.30316	6.5001	6.17439	6.192	6.64295	6.57414	5.86373	5.95803	5.40693	5.58445
LRRK2	Q5S007	5.87642	6.38256	6.8092	6.07031	6.40225	6.1016	6.40599	6.17969	6.74257	6.94199	6.42888	5.92976	6.50472	6.38973	5.984	5.75034
RCOR3	Q9P2K3-3	6.36682	6.58841	5.85037	6.36263	6.30666	6.11965	6.21195	6.09919	6.18074	6.40475	5.881	6.05228	6.68885	6.46633	6.16088	6.25949
INPP4B	O15327	5.90734	6.07745	6.76186	5.86495	6.00254	6.73813	6.16578	6.55707	6.30562	6.66836	6.53199	6.70264	5.74652	6.4013	5.75655	5.81188
BAZ2B	Q9UIF8	6.41945	6.32794	5.80886	6.74811	6.01153	5.84435	6.42607	5.86228	6.0362	6.23361	6.03314	6.46332	6.32254	6.50984	6.51369	6.43907
ZNF451	Q9Y4E5	5.9915	6.27262	6.13951	6.34029	6.42829	6.28869	6.54667	5.92555	6.04498	6.30702	6.38688	5.98126	6.35549	6.14287	6.66883	6.17955
TRMU	O75648	6.48238	6.42554	6.03824	6.75445	6.04702	5.83089	6.42215	6.05661	6.32889	5.88524	6.0676	6.04623	6.64596	6.16613	6.36498	6.43769

EXOC6B	Q9Y2D4	6.28773	6.00627	6.56398	5.96511	6.60459	6.33211	6.24686	6.17141	6.35106	6.86637	6.61614	6.46169	6.10407	6.05379	5.88638	5.48245
SF3B4	Q15427	5.85138	4.75703	5.56142	5.03238	5.7577	5.01014	5.55669	6.52271	5.37955	5.61608	5.68648	7.52261	7.07757	7.77083	7.8214	8.07602
COPE	O14579	5.9456	5.06751	5.59021	4.86135	5.97494	6.23932	5.89897	7.38001	5.22538	6.35436	5.93714	7.12493	5.16209	8.45072	7.07427	7.71318
ACADS	P16219	6.38886	6.08888	5.68691	5.98074	6.55046	5.7541	6.57408	6.2356	6.17665	6.19481	5.79956	5.7037	6.95235	6.41478	6.87883	6.61968
EOGT	Q5NDL2	6.01994	6.64747	6.30942	6.5419	6.20806	5.76244	6.18279	5.73235	6.07068	6.29746	5.93097	5.97208	6.71769	6.39342	6.57167	6.64166
DOCK6	Q96HP0	6.33465	6.54847	5.93348	6.06331	6.05646	6.37774	6.17012	6.09075	5.93312	6.07024	5.9782	5.9482	6.60492	6.58529	6.59509	6.70995
BUD31	P41223-2	6.13646	6.53015	6.21058	6.02	6.78122	6.4272	6.28001	5.86122	5.90598	6.32856	5.80285	5.96772	6.62628	6.13212	6.6908	6.29884
POLDIP2	Q9Y2S7	6.15768	6.11257	6.26389	6.67927	6.2421	5.89811	6.49154	6.05974	6.30164	5.93785	6.30872	6.10483	6.61404	6.0032	6.39459	6.43023
TTC17	Q96AE7	6.80716	6.72877	6.1528	7.2319	6.37221	5.98813	6.06252	6.45136	6.13669	6.51073	6.20428	6.30858	5.83771	5.76537	5.71078	5.73102
PMS2	P54278	5.43962	6.12614	5.84087	5.64252	5.7981	6.17453	5.57569	5.93362	5.81056	6.20561	6.052	6.07217	7.4188	7.49357	7.37763	7.0386
NXN	Q6DKJ4	6.36882	6.20535	7.08332	5.73425	6.24513	7.09798	5.94246	6.47656	6.24215	6.17048	6.69192	6.55981	5.75341	5.68025	5.71662	6.03148
BCAS2	O75934	5.84615	4.78968	4.73769	5.01569	5.00666	3.66331	4.96424	6.01667	3.84559	5.49176	4.65648	5.68389	6.00734	12.0526	10.1809	12.0414
PNISR	Q8TF01	6.42037	6.46806	5.98236	6.56325	6.26096	5.92273	6.04331	6.03758	6.15448	5.84101	6.30303	5.88073	6.5426	6.6543	6.36452	6.56071
NCDN	Q9UBB6-3	6.21596	6.37999	7.30833	5.87712	6.52061	6.90573	6.52204	6.54622	6.58334	6.59076	6.63761	6.58398	5.30035	5.36126	5.25733	5.40936
MED4	Q9NPJ6	6.57704	6.19353	6.08302	6.23856	6.3387	6.08512	5.84083	6.20866	6.10502	6.48845	6.44386	6.54443	6.35839	6.08977	6.14477	6.25986
OGFOD1	Q8N543	5.24331	5.57722	6.20533	5.43538	5.61908	6.2296	5.70081	6.19416	6.04399	6.03072	6.24375	6.37956	6.80063	7.40973	7.5701	7.31662
KHDC4	Q7Z7F0	5.70608	5.58024	5.88116	5.91939	5.95287	6.24161	5.53149	6.62801	6.35038	6.21307	6.6547	6.63105	7.41148	6.31455	6.40676	6.57718
HIRA	P54198	6.42628	6.40418	5.89872	6.96943	6.11031	5.80538	6.33234	5.97704	6.17093	5.94259	6.12898	6.2885	6.25186	6.46732	6.31636	6.5098
SIX5	Q8N196	6.26281	6.34906	6.66397	6.32308	6.04443	6.17485	6.04971	6.46443	6.22436	6.7123	6.50563	6.61758	6.02034	6.21937	5.62321	5.74486
GAN	Q9H2C0	6.17987	6.26892	7.10424	6.10152	6.26735	6.20629	6.1788	6.3336	6.54429	6.50522	6.74331	6.39084	5.54944	5.73959	6.01313	5.77359
GSTM3	P21266	6.13861	5.92435	6.91884	5.39722	6.40769	6.58723	6.5092	6.44902	6.45114	6.65457	6.46527	6.48859	5.82842	5.80933	6.04238	5.92815
JPT1	Q9UK76	6.6497	4.93431	6.92048	4.9905	6.64285	5.3576	5.31951	6.8361	4.66083	7.03677	6.3995	7.80858	5.75676	7.50928	6.05569	7.12155
AK6	Q9Y3D8	5.42951	5.89097	6.64814	5.58498	6.42936	6.31425	6.03952	6.06985	6.16435	6.6142	6.98438	6.61429	6.06139	6.70484	6.18927	6.26072
SAR1B	Q9Y6B6	6.23056	5.7809	6.44927	6.21373	5.69926	5.93487	5.9209	6.18752	6.04989	6.48235	6.83748	6.75216	6.26659	6.55898	6.25765	6.3779
RING1	Q06587	6.12154	5.9135	5.93666	6.34204	6.27448	6.11669	6.17215	6.33416	6.25304	6.07904	6.30209	6.02418	6.83832	6.34858	6.50116	6.44238
TRAPPC12	Q8WVT3	6.57412	6.21386	6.72424	5.84455	6.41529	6.73401	5.89548	6.40941	6.77658	6.31873	6.57497	6.45933	6.02233	5.9563	5.53194	5.54885
MON1B	Q7L1V2	6.22809	6.432	6.78813	6.3303	6.1417	6.48817	6.26677	6.19	6.02205	6.33581	6.29636	6.54283	6.07795	5.91825	6.04143	5.90016
CXADR	P78310	6.33458	6.32766	5.94736	6.67999	6.06735	6.0474	6.38046	6.08919	6.02069	6.33798	6.24019	6.14994	6.27396	6.30988	6.42754	6.36581
FOXK2	Q01167	6.74296	6.39981	5.95799	6.66279	6.34345	6.39818	6.5164	6.49985	6.4081	6.24862	5.88446	6.0287	6.33708	6.07602	6.02395	5.47164
IFI35	P80217-2	5.70989	4.92885	6.04991	4.62986	6.15652	6.31208	6.03578	7.00997	5.9719	7.11283	6.37984	6.91381	6.39741	7.09157	6.70709	6.59269
COX5A	P20674	5.85478	5.67173	4.60632	5.7305	5.58875	4.02396	5.22353	5.38292	4.47522	5.24693	4.36476	4.62392	4.95218	12.4977	8.36175	13.395
MAPKAP1	Q9BPZ7	6.47275	6.59593	6.71088	6.43251	6.0809	6.62959	6.44066	6.4859	6.63338	6.70895	6.49059	6.42944	5.65553	5.52576	5.6511	5.05612
COMMD4	Q9H0A8	6.15667	5.81227	6.47843	5.39291	6.48021	6.64619	6.2295	6.60061	6.42396	6.7544	6.4501	6.24857	6.13122	6.12926	6.21337	5.85232
KDM2B	Q8NHM5	7.0527	6.92118	6.71742	7.10614	6.49686	6.14787	6.86231	5.81978	6.29111	5.59879	5.75075	5.56052	5.44755	6.05498	5.99734	6.17469
TAMM41	Q96BW9-3	6.41327	6.35079	6.26275	6.57495	6.18013	6.08769	6.46041	5.90738	6.12457	6.30479	5.81105	5.98819	6.37748	6.14329	6.64144	6.37183
CMPK1	P30085	6.07263	6.11782	6.82404	5.57415	6.77871	6.9448	6.12396	7.04305	6.56929	6.76399	7.04873	6.68782	5.88785	5.39659	5.03676	5.12983
SYNJ2BP	P57105	6.75783	5.54964	6.05603	5.77269	6.20477	5.90582	5.90296	6.39627	5.71581	6.03028	5.62546	6.10632	6.58799	7.3346	7.07144	6.98209
SASS6	Q6UVJ0	6.42372	5.84377	6.49255	5.64817	6.48764	6.64675	6.12172	6.84479	6.76999	6.41502	6.78235	6.43928	6.24722	5.5642	5.51399	5.75882
SLFN5	Q08AF3	5.40266	5.65575	6.00283	5.48988	5.67444	6.37302	5.60451	5.79735	6.38976	5.97768	6.11569	5.79194	7.33697	7.67796	7.43319	7.27636
CTNNAL1	Q9UBT7	6.92968	6.60092	6.92138	6.8379	6.16742	6.02525	6.82989	6.34849	6.82054	6.6313	7.27044	6.99469	4.69231	4.83788	4.97664	5.11526
OXR1	Q8N573	6.45603	6.30383	7.04097	6.04649	6.01581	6.70764	6.45574	6.59042	6.48081	6.7441	6.52721	6.33648	5.86568	5.56332	5.42354	5.44194
THYN1	Q9P016	5.63651	5.93647	6.2581	5.34134	5.64938	5.83722	5.7997	5.6248	6.33017	6.52288	6.43657	6.18823	6.89022	7.36482	7.49307	6.59053
ZBTB33	Q86T24	6.68686	6.78474	6.38687	6.9572	5.98472	5.91905	6.64812	5.86296	6.55341	6.10582	6.45643	6.27884	5.65645	5.72519	5.88308	6.11023
PIR	O00625	5.82889	6.10398	6.22063	5.55515	6.94848	6.89547	6.25797	7.28126	6.82212	5.95271	6.29493	6.5586	5.38548	6.27057	5.64505	5.9787
LAMP2	P13473-2	6.74396	6.61009	6.20949	6.88527	6.58011	5.75346	5.83498	6.00958	6.09815	6.39788	6.3332	5.93199	6.56424	5.65736	6.63395	5.75627
EXOSC1	Q9Y3B2	6.09534	5.53994	6.20647	6.25502	6.40517	5.9682	6.27993	5.6562	6.52032	6.13417	6.15762	6.31127	6.77859	6.07516	6.91244	6.70415
TIMM23	O14925	6.36914	6.16076	6.13699	6.85474	6.19925	5.90481	6.40159	6.04178	6.56316	6.30332	6.26539	5.93185	6.59131	5.8873	6.46409	5.92454
TEX264	Q9Y6I9	6.47514	6.51565	5.6611	6.45324	6.48192	5.95963	6.14723	5.75085	6.1419	6.2772	5.83306	5.86313	6.86887	6.10995	7.07608	6.38506
MRPS16	Q9Y3D3	6.20102	6.51948	5.89828	6.51669	6.41847	6.07583	6.55775	6.02428	6.21121	6.18985	5.85901	5.89331	6.72869	6.20257	6.41812	6.28544
MRPL43	Q8N983-4	6.5944	6.07623	6.04985	6.14025	6.20602	6.05011	6.19815	6.82755	5.89207	6.11809	6.25301	6.32342	6.94435	6.09995	6.04897	6.1776
CLP1	Q92989	6.02402	6.04866	6.16257	6.498	6.19138	6.15191	6.23387	5.91254	6.32431	6.11595	6.30792	6.17351	6.84362	6.02006	6.68952	6.30216
NDUFB4	O95168	5.98416	5.76132	5.71129	6.49968	6.50921	6.00346	6.56672	6.51818	5.91146	6.42895	5.88335	6.45509	6.6482	6.04706	6.59744	6.47446
FAM172A	Q8WUF8	6.27413	6.28022	6.26983	6.15982	6.27742	6.1359	6.65721	6.02312	6.09368	6.1076	6.20067	6.21976	6.48247	6.05243	6.47913	6.28658
DNPH1	O43598	6.28892	5.58365	6.77823	5.56898	6.40633	6.72032	6.09695	6.6667	6.11192	6.81139	6.78076	6.5827	6.5068	6.15285	5.58128	5.36221
TDRD3	Q9H7E2-3	6.11686	6.64736	6.55418	5.95188	6.22313	6.07114	6.56384	6.42614	6.07477	6.56238	6.4231	6.71823	6.00761	6.13476	5.92166	5.60295
AP2S1	P53680	6.42384	6.54235	6.40506	6.36786	6.04912	6.32336	6.54288	6.09139	6.37102	6.30261	6.2689	6.1906	6.00862	6.0273	6.03971	6.04537
TOMM22	Q9NS69	5.90539	6.14898	5.737	6.47282	5.91599	6.0278	6.21356	6.36961	5.55996	5.59837	6.01216	6.41793	6.47038	7.34007	6.7563	7.05367
GOLT1B	Q9Y3E0	6.15547	6.14739	6.08446	6.24618	6.15677	6.21172	6.32695	5.99605	6.03821	6.23033	5.81916	5.91484	6.54966	6.50771	6.6249	6.99021
FAM169A	Q9Y6X4	6.12153	5.93393	5.62214	6.43882	6.13111	6.03987	6.00877	5.94628	6.22244	6.10484	6.12677	6.03778	7.41038	6.4939	6.7811	6.58035
FBXO22	Q8NEZ5	5.74126	5.98163	6.34075	5.65769	5.82916	6.65575	5.78475	6.0175	5.86656	6.53586	6.62858	6.38577	6.18554	7.04808	6.75957	6.68156
SENP2	Q9HC62	6.051	6.38958	6.50499	6.62441	6.3517	6.09168	6.46505	5.643	6.13942	6.35705	6.16878	6.1046	6.75193	6.15483	5.66375	6.53823
SURF1	Q15526	6.25882	6.13228	5.84215	6.60583	6.18361	5.9847	6.40503	6.11311	6.20157	6.0747	5.95167	6.04462	6.84859	6.21479	6.76836	6.37016
DENND3	E9PF32	6.33154	6.16388	7.01405	6.25569	5.99579	6.47795	6.29444	6.1239	6.3746	6.95185	6.54943	6.36135	6.00551	5.81189	5.68861	5.59952
PISD	Q9UG56	6.25045	6.46857	5.95901	6.67334	6.34884	6.23339	6.49732	6.18253	6.10119	5.93247	5.74121	5.88762	6.5703	5.84641	6.6173	6.69005
UQCC1	Q9NVA1	6.50706	6.62984	6.39124	6.57694	6.60569	5.90987	6.30478	5.89651	5.99465	6.09663	5.74061	5.80621	6.93065	5.95059	6.42572	6.233
NDUFA5	Q16718	5.76679	4.72081	5.14117	4.72476	5.866	5.29079	5.33141	6.50664	5.10351	5.99282	5.32907	6.17638	5.78514	9.4888	9.04084	9.73505
SYF2	O95926	6.82188	7.00902	5.84448	6.47661	6.1427	6.67439	6.82045	6.62012	6.83357	6.31135	6.35051	6.20854	5.86581	5.36096	5.41607	5.24355
HMGB3	O15347	5.94258	5.49841	6.37978	5.7133	6.09569	6.15248	5.96271	6.31128	5.87002	6.47511	6.39553	6.17765	6.20673	7.57174	6.86737	6.37963
GPC1	P35052	6.40661	6.62697	6.21502	6.89659	6.03079	5.81841	6.1594	5.86991	6.47907	6.24507	6.38617	6.46237	6.48524	6.20919	5.91555	5.79364
PTRH2	Q9Y3E5	6.38442	6.45673	6.08834	6.60094	6.38993	6.28031	6.77646	5.89334	6.18788	6.49076	6.15815	5.81718	6.37943	5.52822	6.56832	5.99958
DENR	O43583	5.72531	6.71738	6.70549	6.2199	6.60646	7.65445	5.94027	6.67397	6.25707	6.44151	6.81683	6.67331	5.81465	5.78542	4.99573	4.97225
RUSF1	Q96GQ5	6.2992	6.19646	5.80607	6.30014	6.24092	5.87379	6.60412	5.94477	6.19452	5.85416	5.89277	5.93028	6.89247	6.35586	6.92649	6.68798
MECOM	Q03112-3	6.4777	6.52723	6.44572	7.12041	6.52206	6.20453	6.77853	6.38674	6.28361	6.3536	6.38438	6.19501	5.80178	5.43687	5.36511	5.71671
MIB1	Q86YT6	6.48104	6.61863	6.9521	6.55893	6.11711	6.19299	6.36692	6.37547	6.45351	6.64837	6.99404	6.57345	5.40113	5.49265	5.51528	5.25839
LRRC8D	Q7L1W4	6.22395	7.00418	6.00344	6.69939	6.483	5.86839	6.45346	5.80663	6.5053	5.83399	5.80664	6.19463	6.17754	6.09949	6.49938	6.3406
ATP11A	P98196	6.02545	6.42229	6.27995	6.84109	6.08638	6.24737	6.16838	5.87321	6.00036	6.27153	6.40031	6.03761	6.15543	6.31147	6.5218	6.35736
PRKRA	O75569	6.53049	6.46194	6.568	6.08175	7.00572	6.55989	5.83359	6.7476	6.5173	6.6166	6.67448	6.668	5.57404	5.56406	5.2798	5.31674
SPG7	Q9UQ90	6.396	6.18185	5.80609	6.80316	6.21314	5.67355	6.49763	6.10036	6.06802	5.99766	6.08484	6.00333	6.85109	6.16638	6.80732	6.34956
MAN2A2	P49641	6.30101	6.54631	5.94166	6.82568	6.19288	5.85212	6.28217	6.53008	6.30149	5.96093	6.48932	6.05318	6.08578	6.09843	6.25372	6.28523
DNAJC17	Q9NVM6	5.87672	5.48458	6.12095	5.68265	5.79355	6.1443	5.67548	5.97983	6.07747	6.47273	6.54234	6.54389	6.84525	7.08219	7.28405	6.39401
CBX8	Q9HC52	6.63743	6.45934	5.79173	6.48044	6.10098	5.8387	6.48956	6.32382	6.29619	5.91036	6.22737	6.52662	6.02413	6.1539	6.82607	5.91337
H1-2	P16403	6.17649	6.10128	5.45869	6.21874	6.1383	5.84687	6.09179	5.74945	5.37972	5.43645	5.40454	5.61251	7.06324	7.90954	7.68618	7.72621
ATP6V1D	Q9Y5K8	6.74281	7.02025	7.27885	6.56408	6.23902	6.55204	6.31093	6.36458	6.84775	6.72036	6.97388	6.56339	4.91057	5.20974	4.7187	4.98306
GNPDA2	Q8TDQ7	6.06491	5.98006	6.71289	5.78635	6.26294	6.81715	6.46219	5.86609	6.15786	6.50206	6.8836	6.84531	6.07775	6.00802	6.08371	5.48911
CYP2U1	Q7Z449	6.36669	6.75232	6.05231	6.27323	6.23044	5.85189	6.3242	6.03644	6.1032	6.19921	5.97649	5.94959	6.23552	6.74979	6.71554	6.18312
RPLP1	P05386	5.9889	5.11507	5.04156	4.6047	5.10298	4.78937	5.73582	6.99895	5.33729	6.23073	5.60058	7.54638	5.6825	9.39155	7.43442	9.39921
IQSEC2	Q5JU85	6.01437	6.48578	6.70487	6.07519	5.99258	6.6974	6.07595	6.24056	6.16026	6.26233	7.00315	6.70287	5.83005	6.35201	5.66747	5.73517
CENPU	Q71F23	6.76712	6.70346	5.86703	6.93924	6.19843	5.94529	6.22145	5.89275	6.2875	6.39647	6.39049	6.033	5.94171	6.17752	6.3107	5.92783
GCLC	P48506	6.62066	6.10711	7.09175	5.97238	6.56713	6.61971	6.3046	6.60221	6.43721	6.51391	6.3613	6.3077	5.98943	5.54994	5.46276	5.4922
CCNA2	P20248	6.21578	5.91924	6.19628	5.40796	5.91586	6.02406	6.0362	6.00092	6.26286	6.20957	6.10293	5.99276	6.94765	6.52333	7.22549	7.01911
MLKL	Q8NB16	6.1975	6.19258	6.9409	6.23547	6.08599	6.32896	6.38462	6.28336	6.43878	6.6928	6.90501	6.38647	5.50286	6.0207	5.86823	5.53577
HNRNPLL	Q8WVV9	5.91129	5.87471	6.08853	6.28316	6.09545	5.98905	6.33073	5.89424	6.09803	6.00663	6.22329	5.77131	7.1369	6.50927	7.3269	6.4605
DHX34	Q14147	5.8535	6.1967	6.28349	6.06393	5.96338	6.48992	5.83498	5.77342	6.40832	6.35308	6.46763	6.22511	6.38491	6.53182	6.61245	6.55736
NUDT2	P50583	6.48226	6.07847	6.69038	5.59918	6.16056	6.85766	6.06266	6.84706	6.40313	6.65617	6.37474	6.66303	5.97297	5.79996	5.45251	5.89926
PPP2R5E	Q16537	6.3361	5.96759	6.78326	6.1694	6.53338	6.68976	6.16409	6.04414	6.56509	6.40865	6.5015	6.36419	5.79084	5.89232	5.76305	6.02664
SQLE	Q14534	6.55349	6.59132	6.19028	6.76229	6.06934	6.08565	6.59351	5.99614	6.13629	6.1114	5.9778	6.12581	6.39434	5.9966	6.12923	6.28654
MAPK14	Q16539-2	5.99815	5.8942	7.20795	6.03678	6.28059	6.84548	6.32539	6.48079	6.35347	6.5445	7.21703	6.59297	5.45147	5.89728	5.35699	5.51696
ACIN1	E7EQT4	6.14181	6.06282	5.80866	6.92427	6.09339	5.61362	6.42976	5.5466	6.16308	6.21443	5.95825	5.83431	7.16143	6.40934	7.04667	6.59157
KATNA1	O75449	6.0583	6.0969	6.86795	6.38774	6.45987	6.34858	6.39269	6.89569	6.75117	6.63185	6.9761	6.52502	5.44156	5.52892	5.35198	5.28569
NCOA1	Q15788	5.8355	6.0048	6.16334	5.21136	6.43239	6.41712	5.71034	6.03575	6.01673	6.14225	6.40814	6.53544	7.04306	6.70513	6.49073	6.84791
TTI2	Q6NXR4	6.33177	6.36647	6.63266	6.08165	6.44948	6.67337	6.02106	6.77187	6.60019	6.63938	6.73505	6.71077	5.76631	5.64892	5.34245	5.2286
SBF2	Q86WG5	6.46519	5.80926	6.69385	6.36542	6.1302	6.16898	6.37278	6.27397	6.38182	6.84208	6.59548	6.37827	5.67007	6.09041	5.82375	5.93849
COL6A2	P12110	7.56434	7.58472	6.96289	7.32643	6.77949	6.37449	6.87197	6.44632	6.84679	6.77476	6.70166	6.68534	4.14804	4.4316	4.18651	4.31466
ENAH	Q8N8S7	6.27485	6.31735	6.40118	6.22534	6.21582	6.35566	5.5093	6.56051	6.66866	6.72094	7.12477	6.58739	6.02983	6.08936	5.56118	5.35787
LRP6	O75581	6.3796	6.46908	5.7108	6.54631	6.0442	6.10688	6.61424	6.3893	6.31999	5.98814	6.16526	6.40637	6.28618	6.19674	6.35818	6.01872

SLC25A4	P12235	6.22943	6.21651	5.90665	6.77906	6.19066	5.84679	6.26566	6.1322	6.43634	5.91811	6.1226	5.83489	6.75199	6.01451	6.72749	6.62711
SLC25A6	P12236	6.42042	6.50075	5.90916	6.80306	6.25625	6.03271	6.43262	6.10514	6.04716	5.82312	6.02698	6.00408	6.40104	6.19743	6.60359	6.43649
SLC25A31	Q9H0C2	5.9256	6.22304	6.15253	6.62337	6.17166	5.97707	6.31562	5.98665	5.85574	6.16249	6.19771	6.27498	6.67316	6.43925	6.34427	6.67686
ATP9B	O43861	6.15864	6.65776	6.77258	6.82508	5.69465	5.93288	6.36785	5.97931	6.35851	6.46944	6.55853	6.43389	5.93396	5.93731	5.93865	5.98096
ARMC9	Q7Z3E5	6.33554	6.2673	6.93407	6.05277	6.20888	6.67679	6.34991	6.94322	6.4245	6.51623	6.60923	6.63877	5.55439	5.6508	5.25548	5.58213
HCFC2	Q9Y5Z7	6.48876	6.44571	6.16887	6.57816	6.04907	6.10888	6.07515	5.76303	6.10518	6.41251	6.22443	5.81205	6.2077	6.30978	6.79691	6.45381
SEPHS1	P49903	4.98436	4.37518	5.70795	4.03801	5.55243	5.36218	5.30267	6.43548	5.09079	6.35883	6.06727	6.72637	7.99195	8.64849	9.0139	8.34415
GPAT3	Q53EU6	6.18014	6.12135	5.73313	6.47466	6.12781	5.9844	6.46886	5.94993	5.91591	5.97314	6.1802	5.96195	6.68863	6.74186	6.94089	6.55716
RPA2	P15927-3	5.67805	5.51427	5.7677	5.33974	5.94576	6.15032	5.71561	6.33284	6.1077	6.20678	6.32316	6.33464	6.81447	7.39639	7.47526	6.89731
PEAK1	Q9H792	6.29313	6.38493	6.03907	6.39208	6.05394	6.11737	6.10205	6.43783	6.46397	6.08115	6.2296	6.22162	6.20088	6.0877	6.58779	6.3069
DGUOK	Q16854	6.75958	7.20902	6.30643	7.22087	6.95153	6.65007	6.96514	6.60333	6.40584	5.965	5.45389	5.10636	5.78982	5.5467	5.67181	5.39462
ACTR1A	P61163	6.4889	6.55108	6.58641	6.07497	6.10885	6.55376	6.23607	6.42831	6.76452	6.35417	6.59133	6.55368	5.59428	6.01658	5.54755	5.54958
XPNPEP3	Q9NQH7	6.16137	6.30667	5.60181	6.84246	6.21045	6.23874	6.3076	6.47479	6.05122	6.20209	6.08701	5.94023	6.7927	6.28154	6.10289	6.39844
CWC27	Q6UX04	5.58522	6.01521	5.85229	6.0987	5.90647	6.3248	6.2174	6.29234	5.4825	6.41294	6.13825	6.03501	6.6369	7.09257	6.90115	7.00828
MCM9	Q9NXL9	6.2924	6.31282	6.30079	6.95655	6.17686	6.01399	6.49363	6.20599	6.37574	6.27938	6.32169	6.01119	6.04475	5.99832	6.09315	6.12273
IKBKG	Q9Y6K9-2	6.74197	5.39915	6.97895	4.91689	6.78437	5.80521	5.55478	7.41837	5.96047	6.89575	7.36451	7.111	6.34725	5.6982	5.11174	5.9114
LRCH1	Q9Y2L9-2	6.61449	5.7684	5.92612	6.32398	6.31349	6.67207	6.01185	6.13169	6.46829	5.79836	6.53645	6.46884	6.52344	5.92058	6.12705	6.3949
HMGB2	P26583	5.35346	6.14916	5.81452	5.39267	5.62356	6.73573	5.12294	7.10596	5.25135	6.41892	6.59563	6.13458	5.97497	8.44682	7.37008	6.50965
TERF2IP	Q9NYB0	6.38455	6.07537	6.59903	6.18898	6.00323	6.33538	6.04383	6.31999	6.11291	6.162	6.25551	6.27388	7.01637	6.21885	5.87186	6.13825
ACOT8	O14734	5.69125	5.95938	5.99135	6.36098	6.40345	6.62334	6.58693	6.11657	6.11421	6.17191	6.45655	6.15874	6.75105	5.79819	6.35843	6.45766
ATP6V0A2	Q9Y487	6.26236	6.31944	6.38097	6.85467	5.68434	6.10523	6.62996	6.28502	6.05926	6.3768	6.23659	6.09944	6.16056	6.27595	6.24997	6.01943
HAUS4	Q9H6D7	6.42614	5.5443	6.75983	5.55198	5.87495	6.28651	6.19776	6.54548	5.43571	6.7656	6.35975	7.28023	5.3875	6.80798	6.26638	6.5099
EIF2D	P41214	6.29326	6.56071	6.80155	6.11745	6.29018	6.55744	6.62598	6.48792	6.61515	6.62285	6.42596	6.70795	5.5647	5.37141	5.42724	5.53023
ZCCHC3	Q9NUD5	5.69619	5.78207	6.07986	6.32325	6.14592	5.69524	7.87483	5.82165	7.18109	4.71859	5.42104	6.02807	5.06556	7.58295	6.53186	8.05183
COMMD10	Q9Y6G5	5.68876	5.04369	6.15454	4.67695	5.57462	5.72499	5.46633	6.49537	5.70974	6.93543	5.84406	6.88699	7.09069	7.81344	7.49593	7.29846
MCAT	Q8IVS2	6.52618	6.8131	5.46532	7.49054	6.74486	6.41087	6.02427	6.55547	5.95967	5.64988	6.03015	5.79242	6.26472	6.16914	6.21913	5.88428
SGPP1	Q9BX95	6.09276	6.43153	5.84778	6.37997	6.24032	6.22311	5.97588	5.95666	6.14224	5.95659	5.65044	5.85483	6.95264	6.29132	6.8367	7.12824
ACAA1	P09110	6.7305	6.23753	6.18887	6.59327	6.20108	5.77993	6.3458	5.87989	6.19656	6.16526	5.99493	6.27563	6.88358	6.00095	6.39332	6.1329
TMEM87B	Q96K49	6.31305	5.63594	6.0997	6.44196	5.8995	6.00464	6.71971	5.34322	6.11309	5.90759	5.87049	5.85904	7.37025	6.35438	6.82524	7.24221
EXOSC8	Q96B26	6.13753	6.32446	6.12821	6.77239	6.4048	6.032	5.99549	5.88877	6.40961	6.17686	5.92905	6.33209	6.86188	5.83868	6.41734	6.35083
IFT81	Q8WYA0	6.07291	6.70347	6.55559	6.25131	6.23977	6.67701	6.16201	6.60429	6.43575	6.72164	6.75875	6.59018	5.5085	5.77664	5.53623	5.40592
CLASRP	Q8N2M8	6.09729	6.31742	6.35254	6.86304	5.69107	5.91108	6.27736	5.79996	6.03566	5.91762	6.38147	6.45883	6.6451	6.43774	6.17225	6.64155
KATNB1	Q9BVA0	6.10618	6.23547	6.53488	6.05522	6.23099	6.64133	6.27581	6.30288	6.27908	6.36261	6.31344	6.48783	6.00859	6.33622	5.9814	5.84808
EXOSC5	Q9NQT4	5.95684	6.10719	6.21903	6.13029	6.11569	6.14589	6.22605	6.2264	6.18065	6.07346	5.98942	6.32315	6.56517	6.35848	6.83677	6.54553
UBE2N	P61088	6.01212	5.02368	6.39858	4.66556	5.75006	4.8795	4.96516	7.02505	4.67353	5.84624	5.32103	6.73319	5.63054	9.71845	7.98309	9.37422
RPUSD4	Q96CM3	6.46145	6.4063	6.24123	7.00392	6.13176	6.07076	6.57372	5.97083	6.59109	6.12223	6.21304	6.25279	6.16747	5.70347	6.12947	5.96047
PRCC	Q92733	6.08233	6.23804	5.85945	6.06776	6.55884	6.09758	6.03704	6.27894	5.91641	6.10745	6.09806	6.04044	7.20536	6.58461	6.40504	6.42266
MERTK	Q12866	6.48212	6.49888	6.46384	6.98254	6.57969	6.48032	6.27499	6.20866	6.24152	5.91142	6.42968	6.32892	6.07796	5.80744	5.60822	5.6286
DIABLO	Q9NR28	6.20387	5.33872	5.18904	5.29333	5.94715	5.43134	5.69765	6.30439	4.97967	5.97144	5.53487	6.43667	6.28395	8.30842	8.82737	8.25211
RMI1	Q9H9A7	6.60276	6.42734	6.21322	6.4141	5.81859	5.8641	6.51896	6.23559	6.4278	6.33866	6.30367	6.27638	6.32618	5.81563	6.18181	6.23522
INTS9	Q9NV88	5.62315	6.3363	5.9269	6.26699	6.15308	6.25964	6.33528	6.22222	6.0979	6.21222	5.99562	6.24704	6.39997	6.3517	6.8779	6.69411
SNAPC1	Q16533	6.46244	6.26727	6.71568	6.62736	6.42029	5.94885	6.65166	5.99586	6.22946	6.50514	6.09105	5.80336	6.24845	5.73399	6.88619	6.31295
GMIP	Q9P107	6.5657	6.32201	7.40687	5.93521	6.47122	6.4324	6.17193	6.27167	6.5147	6.60661	6.90169	6.49218	5.46055	5.82334	5.39136	5.23255
MKRN2	Q9H000	6.69397	6.75298	6.41402	6.42961	6.46161	6.5116	6.47211	6.64302	6.49014	6.30725	6.20145	6.38416	5.65116	5.25539	5.80111	5.54563
SETD1B	Q9UPS6	6.2121	6.69236	6.05696	6.54845	6.04493	6.28859	6.39367	5.98979	6.08487	6.43112	5.71281	5.9666	6.5067	6.51659	6.33326	6.22118
ORC4	O43929	6.30923	6.38709	6.20404	6.4938	6.13982	6.50141	6.2723	6.17872	6.27944	6.55742	6.04689	6.02154	6.21981	5.85938	6.53584	5.99327
ZNF184	Q99676	6.27672	6.89202	6.08322	7.15283	6.44861	6.11957	6.43032	6.15766	5.84092	6.39015	6.40578	6.08723	5.60763	6.09044	5.91648	6.10043
ZNF629	Q9UEG4	6.23272	6.22376	6.06308	6.44209	5.88279	6.07236	6.28235	6.03824	6.34639	6.29381	6.21163	5.93227	6.39895	6.44673	6.72385	6.40897
MBD2	Q9UBB5	6.56658	6.0551	5.785	6.80137	6.23079	6.02699	6.47855	6.1233	6.03995	5.93859	6.17406	6.08637	6.71257	6.05548	6.27324	6.65206
ERF	P50548	6.27714	6.47376	7.08705	5.8806	6.07037	6.60441	6.05219	6.47017	6.18153	6.75537	6.61361	6.93016	5.77843	6.19302	5.32412	5.30805
GALK1	P51570	5.75347	5.97045	6.36694	5.59818	6.42552	7.24015	6.38458	6.85501	6.85277	6.60625	6.739	6.38599	6.05806	5.82678	5.42859	5.50826
URI1	O94763-4	6.53209	6.39044	6.60831	6.02582	6.81064	6.42078	6.08808	6.65258	6.60022	6.36158	6.60004	6.60746	5.83899	5.47651	5.51003	5.4764
LANCL2	Q9NS86	6.02492	6.19999	6.96234	5.8978	6.28879	6.32173	5.594	6.4116	6.3018	6.51392	6.60459	6.5738	6.15235	5.91308	6.29243	5.94687
CHST14	Q8NCH0	7.07813	6.45163	6.20455	7.50027	6.36719	6.27291	6.39112	6.11352	6.67888	6.27896	6.33433	6.74809	5.41732	5.37124	5.25643	5.53542
KDM4B	O94953	6.24882	6.69126	6.21361	6.55172	6.2446	6.16313	6.3298	6.21409	6.46607	6.64759	6.304	6.13409	5.69805	6.2563	6.06952	5.76736
CASC3	O15234	6.36774	6.40653	6.97167	6.31036	6.01429	6.54487	6.10921	6.53105	6.16661	6.45816	6.76193	6.60478	5.99201	5.50327	5.5023	5.75522
RAD54L	Q92698	5.44376	5.86726	6.00987	5.34296	5.80856	6.20097	6.21961	5.80836	6.52524	5.95436	6.45844	6.56194	6.88815	7.02228	7.09297	6.79527
H1-10	Q92522	5.84457	6.04373	4.91949	5.79177	5.78216	5.75192	5.81154	5.83702	5.34432	5.72018	5.26185	5.54838	7.26244	8.68869	8.25754	8.13442
AHCYL1	O43865	6.06086	6.35865	6.71971	6.08025	6.23261	6.67958	6.15219	6.37957	5.7869	6.3222	5.72076	5.95091	6.32823	6.69563	6.29636	6.2356
DCP1A	Q9NPI6	6.01724	5.62086	6.69444	5.50842	6.46725	6.4106	5.77529	6.84593	6.08519	7.001	6.61235	6.82699	6.20299	6.24539	5.85201	5.83405
SDC4	P31431	6.99719	6.79308	6.01571	6.42369	6.20367	5.97929	6.18792	6.31724	6.60746	5.9841	5.8802	6.14166	6.44257	5.96729	5.76993	6.28898
ARPC5	O15511	6.11106	6.03271	6.98059	5.60671	6.63198	6.24642	5.91069	6.48874	6.02226	6.36031	6.56243	6.3096	6.55349	6.21028	6.28544	5.68727
ICE2	Q659A1	6.19824	6.47272	6.65508	6.50793	6.04626	6.2416	6.40407	5.77418	6.1425	6.32888	6.52105	6.16111	6.48587	6.26196	5.84164	5.95692
WDSUB1	Q8N9V3	6.33848	6.30513	6.9159	5.86854	6.39296	6.94088	6.33802	6.16596	6.22588	6.49932	6.74091	6.84167	5.90268	5.45786	6.10478	4.96105
ELL	P55199	6.09427	6.46544	6.68158	6.6397	5.69372	6.22288	6.12289	5.96341	6.19465	6.19929	6.24374	6.29532	6.34252	6.15879	6.51097	6.17083
UNK	Q9C0B0	6.12853	6.24248	6.33368	5.7903	6.48515	6.60263	5.90513	6.59342	6.2288	6.52989	6.44375	6.03503	6.44526	6.39606	5.82117	6.01873
SNRPGP15	A8MWD9	5.78173	5.85565	5.51678	5.90295	5.88488	5.69869	6.35833	6.0711	6.6075	6.06565	6.25753	6.48026	6.92677	6.52686	6.87724	7.18808
MAP4K4	O95819-3	6.11783	6.30587	6.27024	6.25316	6.07593	6.42294	5.88353	5.97659	6.27272	6.44937	6.56648	6.50185	6.38135	6.09864	6.10223	6.32128
FLYWCH2	Q96CP2	5.97593	5.70965	6.72943	5.20878	6.26273	6.32024	5.68725	6.4046	5.98006	6.87041	6.95192	6.38832	6.62489	6.63956	6.50097	5.74526
KAT6A	Q92794	6.39149	6.84097	5.9112	6.80386	5.62602	5.62658	6.11646	6.25164	5.95253	6.19133	6.51617	6.27029	6.1996	6.11972	6.15903	7.02312
PASK	Q96RG2-2	6.34288	6.53121	7.36872	6.15617	6.57419	6.49953	6.10044	6.80065	6.65197	6.72744	6.59552	6.93121	5.32341	4.92102	5.34053	5.13512
PDCD10	Q9BUL8	6.40481	6.31706	6.52188	5.72349	6.05398	6.20266	6.47306	6.55914	6.51131	6.33417	6.65504	6.27401	6.45982	5.74552	6.02328	5.74076
DENND2B	P78524	6.47427	6.12414	6.23894	6.65581	6.02827	6.13207	6.61085	5.82176	6.20648	6.41187	6.21505	6.3894	6.22808	6.08075	6.08793	6.29432
EZH2	Q15910-2	6.40285	6.99868	5.9645	7.38511	6.36893	6.64183	6.4628	6.15775	6.27069	6.21131	6.32499	5.41227	5.60914	5.94301	5.66288
MAD2L1	Q13257	5.98675	6.14589	6.37442	6.0895	6.32131	6.50942	6.15679	6.25736	6.08327	6.24531	5.86555	6.38952	6.231	6.21144	6.58192	6.55058
C17orf75	Q9HAS0	6.28557	6.36802	6.47781	5.94559	6.68287	6.24043	5.98396	6.0739	6.3716	6.41524	6.33736	6.46668	6.3995	5.78307	6.02344	6.14496
EPC2	Q52LR7	6.6641	7.13188	5.86062	6.52907	6.05771	5.96237	6.58566	5.71586	6.89656	6.03735	6.20276	6.29432	6.29031	6.03832	5.4578	6.2753
NFKBIL1	Q9UBC1	6.39033	6.42379	5.88319	6.70468	6.5867	6.4475	6.37125	6.05294	6.75444	6.11065	6.60401	6.6105	5.92104	5.66885	5.85598	5.61416
PPIP5K1	Q6PFW1	6.40409	6.68922	6.78029	5.832	6.45824	6.77887	6.18014	6.61479	6.1903	6.40196	6.42129	6.36637	5.79102	5.78865	5.52107	5.78169
TRIM47	Q96LD4	6.07877	6.18413	6.80019	5.507	6.45662	6.60078	5.85173	6.3594	6.42691	6.24306	6.56453	6.58771	6.22436	6.04948	6.22875	5.8366
DMXL2	Q8TDJ6-3	6.35686	6.65366	6.75076	6.24131	6.24405	6.43551	6.1276	6.39533	6.5967	6.81522	6.54306	6.65093	5.4841	5.71284	5.59533	5.39675
UBE2S	Q16763	6.74825	7.15324	7.28717	7.05374	6.29187	6.7722	6.51166	6.57256	6.88195	6.94867	6.8061	6.89248	4.00508	5.0841	4.41148	4.57943
KIF3A	Q9Y496	6.62091	6.42233	6.74802	5.94539	5.94576	6.56552	6.27274	6.72168	6.10446	6.7292	6.64245	6.57151	5.20325	5.96201	5.43311	6.11164
STIM1	Q13586	6.56762	6.55629	5.80992	6.52218	6.32405	6.00765	6.14832	6.18147	5.77991	6.26243	6.05	5.76212	6.42275	6.36011	6.61704	6.62814
MARS2	Q96GW9	5.95017	5.99122	5.95731	6.74691	6.3149	6.21037	6.46988	6.23506	5.90724	6.02386	6.12391	6.21204	6.79467	6.09965	6.42876	6.53406
CEP78	Q5JTW2-2	6.53001	6.59327	6.79816	6.38412	6.20579	6.40828	6.13794	6.32436	6.48351	6.54426	6.62374	6.89156	5.57961	5.43738	5.52366	5.53436
PYM1	Q9BRP8	5.80531	5.92598	6.36719	5.94309	6.24376	6.40329	6.03968	6.55644	6.30577	6.67788	6.59901	6.91862	5.80002	6.29795	6.13794	5.97807
ATG9A	Q7Z3C6	6.20924	6.17535	6.4858	6.34744	6.15716	6.25908	6.14292	6.0131	6.38595	6.50434	6.65524	6.52548	5.99332	6.13058	5.99074	6.02425
TRAPPC10	P48553	6.47117	6.34446	6.53804	6.35853	6.42597	6.46652	5.97738	6.1584	6.43815	5.97652	6.4819	6.0829	6.00048	6.47459	6.12338	6.58163
PUM2	Q8TB72	6.25181	6.33124	6.58683	5.94544	6.44845	6.41402	5.39503	6.09477	5.97015	6.60912	6.87541	6.41181	6.15234	6.20638	6.26323	6.04398
ZHX3	Q9H4I2	5.72948	6.40857	6.25096	6.08576	6.32302	6.48121	5.94846	6.14602	6.0797	6.1194	6.66189	6.42399	6.75452	6.48339	6.24149	5.86212
ALCAM	Q13740	6.43448	6.44931	5.95735	6.3181	6.14911	6.21058	6.32926	6.10734	6.07671	6.21026	5.95214	6.18091	6.37282	6.43801	6.44636	6.36725
PIK3R1	P27986	6.49606	6.21599	6.95737	6.07907	6.41838	6.57204	5.87866	6.52914	6.60012	6.63765	6.49347	6.59711	5.88516	5.63094	5.45905	5.54979
STX18	Q9P2W9	6.10955	6.21154	6.11168	6.60774	6.15608	5.80368	5.87425	6.37531	6.24838	6.22051	5.79112	6.16582	6.32632	6.53859	6.78625	6.67319
BNIP1	Q12981-1	6.58123	6.55515	5.84613	6.4632	6.16061	6.0753	6.22798	5.95668	5.72481	6.40186	5.94398	6.44269	6.10389	6.33568	6.51216	6.66866
SLC25A19	Q9HC21	6.00053	6.04085	5.98802	6.35545	6.07593	5.46536	6.6897	5.57012	6.32724	5.99549	6.2523	5.98676	6.68502	6.46085	7.06732	7.03907
PPP1R37	O75864	5.9963	6.13406	6.57338	5.93131	6.20354	6.57117	5.94808	6.16067	6.3826	6.3327	6.54003	6.43821	6.1501	6.32292	6.17386	6.14108
VPS72	Q15906	6.3877	6.88413	6.0314	7.05258	5.97075	6.19159	6.1576	6.03419	5.95966	6.34698	6.25726	6.32236	6.5775	6.24051	5.6097	5.97609
MNAT1	P51948	5.87496	5.7792	6.30274	6.22116	6.4715	6.47793	6.2506	6.15267	6.38916	6.30566	6.75052	6.63278	6.14539	5.8542	5.96838	6.42315
DDX60	Q8IY21	6.57549	6.20151	6.73775	5.81875	6.63578	6.90005	6.2958	6.51458	6.75092	6.26041	6.6137	6.32266	5.45085	5.85739	5.82627	5.23809
RALGAPA2	Q2PPJ7	5.82941	6.39801	6.86658	6.34938	6.21028	6.59649	6.39352	6.52875	6.35251	6.30406	6.75669	6.7856	5.70728	5.8298	5.65517	5.43648
NOP16	Q9Y3C1	6.65467	6.49408	6.08202	7.12717	6.41727	6.10596	6.74047	5.94685	5.68325	5.42569	5.50984	5.86294	6.36366	6.25373	6.72908	6.60332
PCYT2	Q99447-3	6.22254	6.19344	6.67838	5.81433	6.51383	6.65041	6.33124	6.65169	6.31549	6.60049	6.40702	6.27082	5.98737	5.64365	5.99363	5.72566
IMPDH1	P20839-6	6.02805	6.10053	6.87282	5.76966	6.3595	6.44538	6.25213	6.72534	6.80467	6.64088	6.62418	6.52335	5.72967	5.69005	5.83908	5.59472
NDUFB9	Q9Y6M9	6.24738	6.16814	5.82018	6.44712	6.73008	5.91535	6.47754	6.12961	5.88471	6.11318	6.07933	5.78411	6.92973	6.45963	6.57881	6.23511

PELO	Q9BRX2	5.92408	6.13874	6.70759	6.24185	6.63578	6.55223	6.51938	6.39323	6.24933	6.176	6.17959	6.46844	5.82578	5.84035	5.99493	6.1527
AKR7A2	O43488	6.19771	6.23599	6.17887	6.10285	6.18439	6.01167	6.06845	6.86884	6.33241	6.70972	6.75559	6.59495	6.07904	6.1769	5.67906	5.82357
DPY19L4	Q7Z388	6.29412	6.32531	6.04145	6.62264	6.30882	5.7044	6.37448	5.89559	5.88081	6.1706	6.18853	5.99282	6.42523	6.44525	6.79034	6.53961
EPS8L2	Q9H6S3-3	6.43697	6.16544	7.01206	5.82003	6.1403	6.54562	6.05152	6.2806	6.5571	6.44475	6.13887	6.53808	6.37952	5.84949	5.74377	5.89588
RP2	O75695	6.18631	6.53116	5.95744	6.43564	6.40689	6.1008	6.56442	6.05296	6.19588	5.96109	5.92675	5.85326	6.55176	6.48621	6.50498	6.28445
PPP1R9B	Q96SB3	6.19005	6.72458	6.10406	6.89848	6.4399	6.07598	6.06028	5.93293	5.96778	6.18963	6.58169	6.13242	6.5874	5.57048	6.25988	6.28446
MAPK3	P27361	6.48795	5.45275	6.96201	5.82385	6.14525	6.41469	5.97419	6.41387	6.40782	6.48527	7.0075	6.725	6.18883	5.87664	5.8337	5.80067
PLCD3	Q8N3E9	6.25712	6.28438	6.18177	6.6173	6.6011	6.13257	6.1208	6.0592	6.34136	5.96215	6.12293	6.37986	6.45595	6.21043	6.02402	6.24908
TXNDC9	O14530	6.07876	5.90829	6.88078	6.2217	6.68801	6.72129	6.47728	6.85189	6.63037	6.61561	6.98363	7.04713	5.34434	5.26461	5.24156	5.04475
FARS2	O95363	6.44028	6.49612	6.14243	6.52416	6.52551	6.12546	6.19038	6.01891	6.12278	5.97403	5.88782	5.87633	6.24444	6.08317	6.67698	6.6712
PLPBP	O94903	6.52362	6.43759	6.58962	5.82463	7.27196	6.84445	6.05001	6.96457	6.11882	6.66388	6.44308	6.59399	5.76404	5.48628	5.18262	5.24083
LGMN	Q99538	6.70653	7.28335	6.48327	7.02475	6.72703	6.15569	6.16201	6.52234	6.38914	6.66171	6.45201	6.18883	5.43972	5.30617	5.06438	5.43309
CBLL1	Q75N03	6.52536	6.54886	5.99367	6.67448	6.14448	6.24127	5.93822	6.00279	5.91741	6.36767	6.3335	6.21994	6.41301	6.51041	5.81476	6.35416
C11orf98	E9PRG8	5.97993	6.44038	5.56814	6.08367	6.16439	5.78475	6.71382	6.33012	6.54425	5.88588	6.22104	5.74925	6.5353	6.70259	6.40312	6.89337
CSNK1G3	Q9Y6M4-2	6.42338	6.66186	6.49844	6.59588	6.21625	6.42867	6.50766	5.97456	6.00047	6.30052	6.33207	6.45847	5.88325	6.12262	5.6968	5.8991
AP5Z1	O43299	6.58597	6.58469	6.86132	6.25395	6.1567	6.4166	6.3707	6.68624	6.30223	6.52434	6.5548	6.83907	5.46079	5.4896	5.33818	5.57483
SPRYD4	Q8WW59	5.83589	6.20783	5.67839	6.28743	6.40584	6.20013	6.45629	6.3019	6.36774	6.35558	5.76816	6.09848	6.4191	6.05434	6.91545	6.64746
AGFG1	P52594-4	6.02741	6.04162	6.34973	6.05772	5.99546	6.78102	6.15775	6.33635	6.60401	5.99143	6.57844	6.52001	6.64136	5.89822	6.25222	5.76726
GPAM	Q9HCL2	6.99209	6.75937	6.31444	7.27988	5.88493	5.70014	6.20181	5.79361	6.08176	6.22311	6.41994	6.48785	6.21444	5.51717	6.20957	5.91989
DDHD2	O94830	6.65963	6.41376	6.68079	6.32948	6.37753	6.62118	6.34047	6.984	6.68834	6.38825	6.52999	6.63187	5.70832	5.28619	4.98956	5.37064
DENND1A	Q8TEH3-6	6.52785	6.18245	6.39721	6.17246	6.19544	6.53457	6.11586	6.39694	6.50188	6.95396	6.20451	6.53701	5.93581	5.86703	5.65323	5.82377
CCDC50	Q8IVM0	6.61411	6.43347	6.78418	6.06797	6.62438	6.86368	5.9964	7.15838	6.38147	6.44688	6.36687	6.20164	5.71787	5.54648	5.22266	5.57355
CCDC59	Q9P031	6.13775	5.84902	5.812	6.63429	6.29759	6.12287	6.18116	5.9058	5.66591	5.41947	5.97826	5.96187	6.64226	6.97364	7.38018	7.03792
CZIB	Q9NWV4	6.04124	5.6434	7.01993	5.47325	6.32887	6.53807	6.05583	6.48475	6.63832	6.32661	7.06599	6.89439	6.24913	5.69177	5.72269	5.82575
C17orf80	Q9BSJ5	6.2037	6.74079	6.0809	6.83902	6.13403	6.16955	6.23606	6.25016	6.23272	6.26943	6.18593	6.03239	6.04066	6.02016	6.3701	6.19441
SBF1	O95248-4	6.10358	6.13037	6.52125	5.75343	6.51121	6.79688	6.42344	5.97322	6.29368	6.50682	6.75846	6.43621	5.91311	6.28037	5.80002	5.79794
ARHGAP21	Q5T5U3	6.42082	6.41511	6.15158	6.63476	6.04139	5.91002	6.2301	6.46072	6.12979	6.25823	5.92031	5.74407	6.49517	6.44832	6.3143	6.42532
SCAMP3	O14828	6.15904	6.48016	6.55311	6.64658	6.25474	6.29649	6.02024	6.05377	6.24787	6.41323	6.44604	6.27074	5.89305	6.11831	6.05036	6.09628
MKLN1	Q9UL63	6.27322	6.46388	6.96328	6.78662	6.32963	6.25204	6.44329	6.34795	6.34789	6.41937	7.05069	6.32626	5.37835	5.64128	5.35072	5.62552
ARMC10	Q8N2F6	6.31573	6.23358	6.03273	6.54969	5.97631	6.20957	6.14738	5.84012	5.94444	5.99394	5.97039	6.52875	6.47608	6.55871	6.84	6.38258
MAPKAPK2	P49137	6.7268	6.34545	6.74959	6.14856	6.26377	6.86508	6.14028	6.70498	6.5433	7.01372	6.6189	6.87427	4.95605	5.10046	5.35668	5.59211
CDC7	O00311	6.08625	6.55327	6.29834	6.37084	5.90823	6.08846	5.99716	6.10871	6.75621	6.88287	7.06031	6.80295	6.17785	5.26523	5.9295	5.71384
ANKMY2	Q8IV38	6.13498	6.6543	6.80118	6.17559	6.1794	7.22666	5.99536	6.62359	6.1761	6.6823	6.1545	6.56014	5.54588	5.86805	5.7173	5.50465
ARFGAP1	Q8N6T3	6.05415	6.49699	6.50011	6.0853	6.18944	6.52752	6.168	6.45475	6.40905	6.16895	6.43583	6.17999	6.28526	6.26746	6.5587	6.11848
EIF4ENIF1	Q9NRA8-3	6.01386	6.3171	6.6945	6.33174	6.30921	6.48523	5.89673	6.36969	6.49993	6.68789	7.33672	6.81963	5.75112	5.57988	5.57107	5.33569
RBMS2	Q15434	6.61651	6.37147	7.00812	6.1545	6.56209	6.47487	6.08419	6.38706	6.38884	6.52241	6.79846	6.56308	5.8429	5.40604	5.38983	5.42963
UBAC2	Q8NBM4	6.28472	6.12009	5.8461	6.81398	6.0426	5.91269	6.48664	6.29309	6.19303	5.97556	6.20552	6.29316	6.02621	6.27414	6.30424	6.92823
MTMR2	Q13614	6.42699	6.03791	6.7005	5.87262	6.46428	6.56489	6.17343	6.39407	6.2937	6.04443	6.70635	6.37812	6.47353	5.74044	5.91416	5.81459
STARD13	Q9Y3M8	6.40215	6.22045	7.10099	6.37692	6.32084	6.65792	6.43139	6.83799	6.63012	6.12319	6.66462	7.03436	5.22449	5.19391	5.32867	5.45201
DTD1	Q8TEA8	6.25522	5.88594	6.08755	5.59609	6.01655	6.23199	6.13392	6.36117	6.75401	6.36483	6.50376	6.36111	6.71896	6.37578	6.11388	6.23921
TRNT1	Q96Q11	5.84331	5.02441	6.0178	6.29465	6.24214	6.72224	6.23356	5.50468	6.2597	6.49846	6.55625	6.68911	6.90133	6.56711	6.24106	6.30422
TANGO6	Q9C0B7	6.01559	6.16247	6.19899	5.67142	6.21732	6.51734	6.02232	6.64776	6.1654	6.44038	6.40134	6.11984	6.26166	6.42572	6.29701	6.43544
RRAGB	Q5VZM2	6.71273	6.83617	6.69894	6.76219	5.95738	6.6741	6.3621	6.30834	6.44983	6.80406	6.34395	6.51356	5.17937	5.54514	5.50669	5.34546
RIOX2	Q8IUF8	5.57534	6.39201	5.49323	6.31509	5.80421	5.9112	6.27409	6.91677	7.78351	6.21752	6.45851	6.42741	6.37238	5.6983	5.85073	6.50971
MIDEAS	A0A1C7CYX1	6.27869	6.38261	5.9553	6.78778	6.27179	6.10595	6.22876	6.03218	6.18059	6.43784	6.01961	5.94161	6.33704	6.28837	6.3228	6.42909
KIAA0100	Q14667	6.62235	6.44645	6.02908	6.89255	6.20611	5.70972	6.40531	5.83509	6.01126	6.14627	6.23751	5.98614	6.22553	6.19377	6.50717	6.54567
RABEPK	Q7Z6M1	6.31563	5.79086	7.36119	6.18693	6.45039	6.50897	5.6811	6.52955	6.46568	6.63303	6.98537	7.31042	5.56276	5.45549	5.43109	5.33154
RAB5A	P20339	6.24813	6.42057	6.24036	6.52358	5.99766	6.34261	6.35084	6.15338	6.38604	5.9847	6.4882	5.98508	5.97539	6.02971	6.3939	6.47986
PDCD5	O14737	6.43918	4.96394	6.34995	5.05436	6.22824	5.39907	5.22561	7.24577	5.26095	6.79163	6.27401	7.51621	5.82839	7.62323	6.53057	7.17482
TXNDC17	Q9BRA2	6.02812	5.48576	6.21983	4.89986	6.76135	6.7635	6.07885	7.26868	6.18042	6.93018	6.73215	6.72369	5.90159	6.48484	5.59987	5.94132
MAEA	Q7L5Y9	6.63959	6.57756	6.91573	6.69033	6.47941	6.49535	6.4615	6.27894	6.43264	6.26317	6.49757	6.37115	5.53819	5.19113	5.60261	5.56512
CPNE7	Q9UBL6	6.14579	5.87594	6.32904	5.58427	6.12589	6.19868	5.89105	5.94352	6.55411	6.70361	6.48111	6.61346	6.00606	6.59866	6.65587	6.29294
PCIF1	Q9H4Z3	6.08982	6.10461	5.87149	5.34729	6.06227	6.57718	5.50571	6.39774	6.07563	6.35758	5.96988	6.20047	6.51528	6.81009	7.27853	6.83643
MTMR14	Q8NCE2	6.43207	6.29445	6.50326	6.29113	6.39289	6.55584	6.47895	6.28076	6.13526	6.43578	6.22821	6.26764	6.18752	5.66847	5.97258	5.87517
PTDSS1	P48651	6.13756	6.23911	5.98889	6.88525	5.44345	5.53285	6.76462	5.79167	5.3766	6.05703	6.2087	6.02491	6.64768	6.59338	7.49543	6.81287
SMG7	Q92540	6.39182	6.41921	6.97608	6.12632	6.42282	6.50386	6.30967	6.54905	6.39151	6.4483	6.57355	6.63231	5.6612	5.67507	5.43945	5.4798
DHPS	P49366	5.85248	5.80473	6.36946	5.36366	6.11913	6.56773	6.12089	7.07323	6.35721	6.67596	6.44526	6.86926	6.10385	6.1348	5.976	6.16634
NOVA1	P51513	6.50218	5.97114	5.88031	6.5378	6.37433	6.15295	6.05629	5.98541	6.59469	6.23037	6.03939	6.26504	6.84179	5.93753	6.60073	6.03003
ALG1	Q9BT22	6.30085	6.45259	5.85457	6.718	6.16548	5.9123	6.15072	5.84074	6.09345	5.81239	6.1471	6.02842	6.50036	6.39042	6.96059	6.67203
EIF4E2	O60573	5.97774	5.93646	6.69367	6.30729	6.59946	6.80906	6.08053	6.87389	6.75196	6.39986	6.87908	6.74762	5.74165	5.35418	5.36506	5.4825
ARL6IP5	O75915	6.33921	6.38514	5.65715	6.41062	6.10197	5.99909	7.02237	5.54012	5.7837	5.85748	5.78106	6.12595	7.00969	6.93733	6.27208	6.77704
DNAJC16	Q9Y2G8	6.35949	6.3459	5.97465	6.75658	6.44738	5.86643	6.40296	5.67869	6.24978	6.2987	6.19004	5.78383	6.49682	5.99968	6.65091	6.49817
ATP1B1	P05026	6.47892	6.53242	6.4267	6.60942	6.32433	6.46228	6.35462	5.78604	6.38422	6.00881	5.80122	5.92133	6.08302	6.19237	6.30514	6.32916
WBP4	O75554	5.73873	6.19906	5.9763	5.71239	6.21508	6.46071	5.99368	5.81573	6.26508	6.22589	6.225	5.85432	7.1029	6.72813	6.97334	6.51367
BANF1	O75531	5.92014	5.75894	4.60091	5.91917	5.30287	4.34677	4.94938	6.06993	4.5518	5.10149	4.75218	5.39175	4.37879	12.2653	7.93953	12.7511
MESD	Q14696	6.33333	6.43262	5.97125	6.50196	5.75733	6.06716	6.24553	6.02224	5.57487	6.49793	5.87895	6.28321	6.39999	6.51071	6.60935	6.91358
CCN2	P29279	5.35384	5.01031	4.85731	6.1708	5.35374	5.14016	6.73329	5.44711	4.82564	4.15835	4.77994	4.77907	10.1914	7.6456	10.249	9.30449
DENND10	Q8TCE6	5.77909	6.01724	6.24002	6.02915	5.89893	6.59131	5.6412	6.75985	5.8501	6.76217	6.58851	6.69957	6.09239	6.48404	6.03115	6.53525
DCP1B	Q8IZD4	6.22315	5.73952	6.25903	5.43379	6.05712	6.00102	5.72172	6.55417	6.53472	7.03696	7.20245	7.07704	6.15183	6.13994	5.99852	5.86902
SPRY4	Q9C004	6.02002	6.15353	6.22285	6.15125	6.2249	6.16116	6.29829	5.98218	5.90699	6.26903	6.05153	5.96206	6.51256	6.70859	6.56642	6.80865
R3HCC1	Q9Y3T6	6.76982	6.09756	6.86959	6.3101	6.10342	6.19229	6.50097	6.40112	6.7186	6.28328	6.39642	6.26716	5.817	5.76215	5.63324	5.87726
WDFY1	Q8IWB7	6.22033	6.49517	6.50932	6.43626	5.74885	6.15571	6.27838	5.81671	6.31531	6.53056	6.06695	6.21377	6.03479	5.92622	6.81776	6.43391
CLCN7	P51798	6.47702	6.88228	6.3866	7.04292	5.84001	5.95675	6.26146	5.77947	6.32837	6.13832	6.31452	5.96469	5.87903	5.99024	6.42345	6.33486
FBH1	Q8NFZ0-2	5.58844	6.00061	6.49179	5.70918	6.0527	6.28311	5.75956	5.90006	6.27126	6.52	6.67127	6.13387	6.19783	6.75188	6.784	6.88444
CASP6	P55212	6.09101	6.37766	7.04149	5.63118	6.11598	6.91872	6.17376	6.56946	6.1517	6.87609	6.44793	6.44348	5.6195	6.20125	5.77232	5.56848
S100A11	P31949	5.43077	4.73126	5.98964	4.33894	5.49783	5.50246	5.32779	6.55134	4.97374	6.49316	5.4666	6.43891	5.34285	10.121	8.22723	9.56646
CDC123	O75794	6.29072	6.18528	7.32783	6.32985	6.59004	6.90868	6.31394	6.64245	6.76961	6.53501	7.08632	7.18747	5.09959	4.80742	4.75833	5.16746
SLC1A4	P43007	6.13469	6.80612	5.54624	6.54251	6.02148	6.36487	6.00852	5.82425	6.03921	6.58171	6.02841	6.53662	6.41797	6.19245	6.11756	6.8374
GSK3B	P49841-2	6.40824	5.77386	7.01415	6.03991	6.37318	6.77161	6.29969	5.97509	6.42072	6.55247	6.68956	6.3399	6.08936	6.08929	5.4634	5.69957
OXA1L	Q15070	6.39166	6.17841	5.83291	6.38713	6.51139	6.10272	6.53658	5.99582	6.18539	5.87682	5.81437	5.90688	6.69143	6.10959	6.786	6.69288
SLC25A20	O43772	6.38957	6.31969	5.63029	6.52972	6.21213	5.96478	6.17814	6.07371	5.98317	6.1606	5.85283	5.49889	6.83121	6.57765	7.15569	6.64191
TTC12	Q9H892	6.40112	6.25709	6.48727	6.44858	6.42434	6.704	6.5687	6.93356	7.20421	6.6105	6.61982	6.65069	5.35476	5.34544	4.78511	5.20483
NFX1	Q12986	5.80585	6.28313	6.47789	6.09838	6.24215	6.42967	6.31325	6.33889	6.19242	6.73706	6.56232	6.64451	5.72048	6.15023	5.83085	6.17292
NAA30	Q147X3	6.30862	6.25032	6.60139	5.56902	6.43774	6.32709	6.25009	6.31815	6.74485	6.48942	6.34611	6.37955	6.3651	6.04697	5.87647	5.6891
ICE1	Q9Y2F5	6.78152	6.62813	6.36238	7.18939	6.22472	6.2302	6.29776	5.86451	6.3825	5.89643	6.29612	6.2977	6.12585	5.35308	5.87276	6.19695
MTMR1	Q13613	5.9183	6.30942	6.96994	5.70713	6.17935	6.75724	5.80927	6.49144	6.49519	6.85829	6.62956	6.58507	5.76321	5.96835	5.66633	5.89192
SRSF6	Q13247	6.46235	6.51915	6.04379	6.33353	6.37283	5.8925	6.57133	5.9349	6.09334	6.01072	6.25629	5.90023	6.96101	6.39182	6.23768	6.01853
SGO1	Q5FBB7	6.18337	6.24128	5.9424	6.81745	5.77319	5.49297	6.59053	5.52207	6.19527	6.06412	5.99268	5.91698	6.54769	6.45183	7.23127	7.03691
SPTY2D1	Q68D10	6.37785	6.27964	5.91933	6.92154	6.2887	5.58232	6.35635	6.26484	6.22238	6.11402	6.11506	6.0443	5.79085	6.61902	6.51924	6.58457
TBL1X	O60907	6.52429	6.10571	6.08476	6.36727	6.48407	5.77692	6.37068	5.96299	6.22657	6.4911	6.38166	6.16199	6.36756	5.91751	6.47631	6.30062
NPR2	P20594	6.56837	6.80661	5.84061	6.48918	6.36101	6.25939	6.25562	5.8745	6.2662	6.42941	6.63606	6.22377	6.02623	6.01366	5.97398	5.97541
EMC4	Q5J8M3	6.05433	5.61189	5.86545	5.87023	6.37922	5.74561	5.98537	5.88135	5.80383	6.14447	5.83697	6.10806	6.74322	6.9743	7.60268	7.39302
HIBADH	P31937	6.49863	6.10723	6.09753	5.97312	6.61191	6.19426	6.27416	6.28905	6.31361	5.83905	6.40348	5.72943	7.19718	6.13914	6.26818	6.06408
SRSF2	Q01130	5.86562	6.36204	6.07327	5.8546	6.0922	6.14006	5.83274	5.68507	6.27989	6.35194	6.34324	6.15024	7.16703	6.32384	6.68974	6.78848
PEX19	P40855	6.34667	5.07042	4.36193	4.23196	5.08398	4.56852	5.40797	6.94696	4.61522	5.57699	4.61915	7.01169	5.79669	10.9267	8.68406	10.7511
TJAP1	Q5JTD0	6.45435	6.50335	6.81592	6.18361	6.47629	6.62524	6.26001	6.41888	6.58808	6.76597	7.08515	6.68111	5.77707	5.23462	5.14354	4.98681
AGPAT3	Q9NRZ7-3	6.0762	6.42261	5.72762	6.56277	5.8735	5.67598	6.33726	6.03071	5.89315	6.16077	6.03015	5.85239	6.60511	6.85796	6.90213	6.99167
CIRBP	Q14011	6.5346	6.16985	6.00981	6.38009	6.44108	5.9581	6.14169	6.08952	6.64493	6.29791	6.39014	6.40134	6.37314	5.80927	6.32807	6.03046
WDR24	Q96S15	6.44771	6.27663	6.47847	5.97007	6.1596	6.35648	5.95033	6.10503	6.3934	6.29838	6.31104	6.57072	6.07031	6.39439	6.49694	5.7205
ARF5	P84085	6.49339	6.83339	6.61212	6.69947	6.34944	6.53176	5.98461	6.24208	6.53406	6.88567	6.52899	6.40037	6.58954	5.65803	5.4782	5.07888
PSMB3	P49720	6.06771	6.0288	5.95867	5.99863	6.027	6.07863	6.38876	6.45027	6.42774	6.46894	6.23854	6.12454	6.35877	6.40754	6.43117	6.54429
RNF114	Q9Y508	6.12885	6.07952	6.78258	6.29647	6.64408	7.26539	6.06364	7.0791	6.29535	6.45216	6.86244	6.74336	5.57516	5.51023	5.01948	5.20218
SKA3	Q8IX90	6.78798	6.06933	7.13462	5.7892	6.49669	6.3662	6.56953	6.9977	6.35886	6.55514	7.04276	7.26209	5.78031	5.1516	5.0797	5.45829
GGT7	Q9UJ14	6.69439	6.32384	5.59211	6.23476	6.40032	5.94208	6.18899	6.24824	6.07923	6.35987	6.19653	5.66022	6.66865	6.49807	6.44211	6.47059
TECR	Q9NZ01	5.99044	6.71021	5.48377	6.31526	6.07272	6.28754	6.18022	6.49235	6.02714	6.53783	5.84677	6.08685	6.323	6.8746	6.27585	6.49546

PHF20L1	A0A0A0MQS0	6.35226	6.60892	5.9359	6.83174	5.82671	5.93802	6.05072	6.10663	5.8754	6.1387	5.94436	6.3027	6.08243	6.71308	6.28975	7.00267
AMFR	Q9UKV5	7.08162	7.31549	6.56869	7.23937	6.18807	5.97481	6.72388	6.03461	6.43582	6.64345	6.3586	6.22823	5.15203	5.30812	5.13261	5.61459
PPP1R13B	Q96KQ4	6.22134	6.49714	6.48937	6.42918	6.31635	6.08449	6.18572	6.54448	6.1884	6.02542	6.17587	5.77236	6.63306	6.19439	6.12629	6.11613
DDX11	Q96FC9	6.83654	6.68338	6.90439	6.1782	6.51367	6.76151	5.94186	6.50183	6.51619	6.49292	6.78201	6.67919	5.19289	5.38451	5.34342	5.28749
DGCR8	Q8WYQ5	7.09893	7.01975	5.91897	6.8158	6.22376	6.18739	5.96921	6.07344	6.41794	6.53392	6.39771	6.3183	5.83406	5.71943	5.8207	5.65069
SHKBP1	Q8TBC3	5.80763	6.18462	6.03293	6.09374	5.94083	6.36678	6.68711	6.14257	6.0298	6.17063	6.30959	6.06859	6.49887	6.40435	6.69134	6.57063
LSM7	Q9UK45	5.31435	4.57555	5.26802	4.92838	6.00558	4.9689	5.53443	7.11838	5.05424	5.52146	5.30286	6.47792	6.01239	9.171	8.68816	10.0584
BCAR3	O75815	5.94124	6.09615	7.04829	6.18889	6.23177	6.78241	6.4675	6.55269	6.38257	6.1827	6.76283	6.70097	5.79434	5.38502	5.60515	5.87749
EIF4E	P06730-2	5.95087	5.90959	6.7664	5.69241	6.08614	6.37741	6.01359	7.33759	5.8717	7.12589	7.65599	7.30137	5.49897	5.81424	5.37126	5.22658
RNPS1	Q15287	6.35614	6.2024	5.92127	6.78772	5.94401	5.71735	6.36156	5.77677	6.30654	5.84486	6.06551	5.96064	6.88961	6.19981	6.7815	6.88431
KIF3B	O15066	6.37847	6.17732	6.60201	6.41778	5.92405	6.67783	6.23775	6.55602	6.5248	6.73592	6.22517	6.7866	5.47473	6.11886	5.45298	5.70971
POLB	P06746	5.19479	5.82418	5.72679	5.00643	5.96225	6.36661	5.93125	6.21576	6.40497	6.20942	6.20516	6.29653	7.34798	6.89507	7.27201	7.1408
NUP62	P37198	6.27658	4.97205	4.66102	4.55073	5.01168	5.14274	5.68133	6.31436	4.70849	5.55164	4.81193	6.78404	6.89634	9.62033	9.21733	9.7994
TMEM131	Q92545	6.7536	6.83417	6.37307	6.80991	6.48085	5.75848	6.67988	5.79119	6.01457	6.57377	6.04328	6.22326	6.04827	5.65275	6.01893	5.94404
YIPF3	Q9GZM5	6.38835	6.05009	6.0294	6.90074	6.21614	5.71627	6.42496	5.8247	6.43174	6.11854	6.23862	6.41657	5.87779	6.22947	6.57811	6.55852
PDCD2	Q16342	6.42107	6.46772	7.06907	6.38123	6.17829	6.7714	5.93065	6.52887	6.79226	6.381	7.10531	7.41642	5.4117	5.00404	4.96843	5.17257
DAAM1	Q9Y4D1	6.35382	7.18548	6.3315	6.52654	5.84234	5.66855	6.2011	6.26604	6.44124	6.01184	6.49862	6.18401	5.98221	5.84565	6.40365	6.25743
ABL1	P00519-2	5.95294	6.12397	6.16633	5.73888	5.92768	6.75444	6.63092	6.53591	5.9354	6.29113	6.72109	6.31208	6.15751	6.63321	5.79156	6.32695
ABI1	Q8IZP0	6.42223	5.9023	6.4388	5.88315	6.30969	6.97512	6.09145	6.25699	6.23877	6.58892	6.74372	6.44932	6.52711	6.08723	5.54147	5.54375
RHBDF2	Q6PJF5	6.1838	6.3669	6.14777	6.63307	6.37542	5.8551	6.20054	6.26865	6.47656	6.15708	6.17783	6.15693	6.35701	6.06793	6.29727	6.27813
UGT8	Q16880	6.67135	6.55334	6.04065	6.99498	6.38634	5.94743	6.08352	5.78095	6.1838	5.92195	6.32406	6.05428	6.39339	5.89917	6.70015	6.06461
PIGO	Q8TEQ8	6.16222	6.60869	6.11189	6.54018	6.23243	6.06067	6.32502	5.93738	5.99327	6.25456	6.15084	6.00917	6.4387	6.34478	6.44356	6.38663
RPS6KA5	O75582	5.56726	5.50435	5.81705	5.36155	5.78844	5.97898	5.88716	6.1745	5.93119	6.13382	6.38757	5.98142	6.74972	7.75726	7.68917	7.29056
CAMK4	Q16566	6.32273	6.29445	6.9066	5.68527	6.59448	6.68884	6.03822	6.81361	6.29092	6.57625	6.62898	6.31617	5.74304	6.22272	5.85466	5.66306
ECHDC1	Q9NTX5	6.09618	5.91875	6.45659	6.04614	6.42754	6.51674	6.3481	6.45439	6.1082	6.454	6.31383	6.26261	6.06638	6.33228	6.19835	5.9999
POLR3D	P05423	6.05267	5.74965	6.47392	6.0178	6.16305	5.88391	6.41495	5.85485	6.36634	6.17255	6.62445	6.20804	6.49414	6.22689	6.50099	6.79579
ADAM15	Q13444	7.47664	6.7532	6.44542	6.90369	6.37046	6.40969	6.03408	5.97064	6.45718	6.72321	6.33853	6.24651	5.47138	5.46397	5.62592	5.31347
PIP4K2C	Q8TBX8	6.29917	6.19983	6.49393	5.59658	6.36365	6.76244	6.01426	6.1727	6.21883	6.11902	6.28841	6.33184	6.68274	6.35685	6.15081	5.94894
PEX3	P56589	6.65568	6.28174	6.35302	6.70393	6.34245	5.73778	7.06719	6.09845	6.73483	6.23368	6.18207	6.05782	6.05932	5.44975	6.15409	5.88819
NUDT1	P36639	5.94951	5.72244	6.75363	5.71748	6.39488	6.37761	6.08011	7.19491	6.55525	6.90263	6.80955	7.20085	5.38674	5.74348	5.51873	5.69219
CLK1	P49759-3	6.49969	6.53734	6.07601	6.48841	5.85734	5.42768	5.7807	5.75469	5.82762	6.10143	5.71017	5.92073	6.74721	6.68715	7.19069	7.39312
FAM135A	Q9P2D6	6.09043	5.88396	6.95848	6.57407	6.06043	6.08661	6.70753	6.30589	6.42659	6.4702	6.83919	6.67404	5.57163	5.81524	5.54288	5.99281
TGFBR3	Q03167	6.55323	6.28297	6.1032	6.55971	6.65652	6.43009	6.25341	5.90056	6.25157	6.17249	6.55951	6.35179	6.05195	5.6852	5.91731	6.27048
TOM1L1	O75674	6.32763	6.40717	6.73868	5.97393	6.14321	6.67538	6.10087	6.87615	6.31731	6.43573	6.7446	6.53981	5.70107	5.86931	5.53026	5.61891
SLC12A6	Q9UHW9	6.11617	6.31113	6.181	6.32819	5.99003	6.28534	6.23252	6.33506	6.05595	6.62755	6.29266	6.12789	6.21894	6.27324	6.01255	6.6118
EVI5	O60447-2	6.50745	6.59849	6.79748	6.44775	6.27381	6.67752	6.2735	6.65874	6.68582	6.57841	6.70127	6.73604	5.3661	5.35133	5.0542	5.29207
TTC27	Q6P3X3	6.53423	6.31364	6.8752	5.88486	6.3054	6.56517	6.06153	6.61297	6.74008	6.25266	6.5714	6.18646	5.66945	5.94133	5.81813	5.6675
LPCAT3	Q6P1A2	6.24208	6.18906	5.8648	6.38014	6.32986	5.83519	6.33027	5.93546	5.97068	6.15112	5.68463	5.54659	6.95866	6.61377	7.10013	6.86756
HRAS	P01112	6.40655	6.48846	6.15827	6.70434	6.1501	5.85125	6.06286	6.0312	6.10912	5.92072	6.3889	6.37012	6.19676	6.18003	6.45962	6.5217
NDUFB7	P17568	6.54673	6.14134	5.9872	6.44424	6.4224	6.16291	6.68484	6.07711	6.29544	5.88179	6.1321	5.78828	6.57448	6.05987	6.48066	6.32061
ARNT	P27540	5.80438	6.23	6.55388	5.52246	5.25654	6.01347	5.64147	6.25865	6.18583	6.08112	6.92124	6.2595	6.15311	7.09468	7.14514	6.75853
SEC14L1	Q92503-2	7.55934	8.09783	8.56859	7.97595	5.76262	6.9856	6.68868	6.98444	7.25105	7.43982	8.09113	7.92286	2.43916	2.99978	2.35665	2.87651
RPL36A	P83881	5.83275	5.8756	6.23069	5.83002	6.23685	6.87559	6.51899	6.65618	6.80104	6.07674	6.85524	6.0648	5.82357	6.7981	5.93925	5.58458
ENPP1	P22413	6.34753	6.51734	5.91108	6.5583	6.04853	6.226	5.75411	6.16902	6.28357	6.25877	6.3767	6.24741	6.66935	6.33537	6.01817	6.28106
ATP6AP1	Q15904	6.28302	6.35354	6.16871	6.85365	6.19048	5.98935	6.45667	6.48903	6.31515	5.89823	6.48666	5.97868	6.29552	5.82759	6.26983	6.1439
TOX4	O94842	6.96641	6.67508	6.08791	6.84576	6.23399	5.80265	6.37783	6.21107	6.1792	6.20291	6.49032	6.06414	6.32243	5.70292	5.91928	5.91813
DHTKD1	Q96HY7	5.99192	5.82726	5.66389	6.44369	6.07222	6.38933	5.98295	6.05705	6.17752	5.99602	6.25476	6.35171	6.92226	6.65988	6.42123	6.78829
SOAT1	P35610	6.03388	6.19991	5.95006	6.59055	6.02283	5.75297	6.45186	5.91462	6.18963	6.23728	6.54454	6.08986	6.58571	6.52538	6.56053	6.35041
TRAPPC3	O43617	6.12941	5.34424	6.97772	4.92306	5.44952	6.36036	6.04286	6.1016	6.31075	6.13751	6.47687	6.81452	6.68398	5.72069	6.60818	6.64551	6.72274
DNAJC19	Q96DA6	6.32892	6.2237	5.68327	6.52201	6.07663	5.91768	6.2321	6.02405	6.12125	6.54835	5.91303	6.25103	6.78685	6.44353	6.37295	6.55463
SP1	P08047	5.77896	5.98152	6.02036	6.20986	6.03031	6.1791	5.91879	6.03915	5.96645	6.31779	6.51485	6.29528	6.85003	6.74149	6.62918	6.52687
TRIM16	O95361	6.37043	6.39959	6.84389	5.70321	6.53648	6.81706	6.11885	6.47477	6.29829	6.4481	6.74717	6.23141	5.68369	5.98841	5.87251	5.46612
BCL9L	Q86UU0	6.60733	6.24091	6.80511	5.8118	6.90154	6.43891	5.55752	6.59136	5.94823	6.88253	6.1089	6.25535	6.14715	6.11684	5.7839	5.8026
SLC33A1	O00400	6.01283	6.54135	6.20109	6.7176	6.1542	6.13516	6.19036	5.83268	5.8554	6.14499	5.93518	5.75833	6.48255	6.49403	6.85275	6.6915
NDUFB11	Q9NX14-2	6.3515	6.23971	5.5309	6.21514	6.37929	5.70223	6.4537	6.35787	5.72439	5.86868	5.83969	6.12814	6.42901	7.11461	6.56145	7.10368
RBM8A	Q9Y5S9	6.25003	5.65289	5.99136	6.43748	6.02813	5.52426	6.54355	5.65341	6.24125	6.21637	6.18182	6.02009	6.72202	6.86268	6.83581	6.83885
TNFRSF1A	P19438	6.04074	6.3389	5.99371	6.44233	5.85685	6.10481	6.49295	5.90104	6.26134	6.64896	6.41292	6.63181	6.50239	6.18598	5.94201	6.24326
MRPS25	P82663	6.36702	6.15536	6.07278	6.79576	6.34373	6.0871	6.32396	6.04934	6.30573	6.10878	6.09176	6.24788	6.88908	5.89648	6.19807	6.06716
GPATCH4	Q5T3I0-3	6.10179	6.36769	6.21367	7.00574	5.95047	5.83805	6.0465	5.97536	5.91852	5.96184	6.09645	6.20179	6.27151	6.6941	6.37437	6.98214
ADD3	Q9UEY8	5.88677	5.70656	6.125	5.8532	5.49652	6.35507	5.73995	5.79021	6.19335	6.33026	6.02763	5.99992	7.08737	7.13113	6.99608	7.32797
MVK	Q03426	6.06218	5.8298	6.73769	5.81277	5.79551	6.46416	6.01921	6.30161	6.38173	6.60741	6.73259	6.87042	5.97429	6.32957	6.08842	5.99266
SH2D4A	Q9H788	6.09646	6.13078	6.95065	6.18806	6.16734	6.50502	6.60771	6.32237	6.43256	6.15547	6.4549	6.53519	5.77812	5.95241	6.00931	5.71366
SNRNP48	Q6IEG0	6.22372	6.25939	6.20344	6.70985	5.8126	5.76761	6.41914	5.94811	6.37182	6.51535	6.3007	6.00972	5.94235	6.10091	6.4367	6.67858
SP3	Q02447	6.43005	6.19641	6.46442	6.69968	6.22159	6.51178	6.33293	5.94268	5.88677	6.54511	6.18667	5.96814	6.49992	6.13247	6.04692	5.93447
PTPRA	P18433	6.88707	7.14158	6.22644	6.56016	6.42384	6.54174	6.74606	6.49032	5.86291	6.32708	5.75506	5.52382	5.97428	6.14011	5.79503	5.6045
DHCR24	Q15392	6.22392	6.20162	5.95591	6.74728	6.23356	5.97685	6.442	5.74697	6.16113	6.20597	5.95519	5.85397	6.4042	6.16253	6.89112	6.83778
RNF14	Q9UBS8	6.35679	6.05604	6.78338	5.96408	6.57937	6.69344	5.80353	6.66608	6.40357	6.37879	6.69934	6.82908	5.75059	6.11031	5.47516	5.45044
SENP1	Q9P0U3	6.23828	6.36704	5.69528	6.80656	6.29543	6.13032	6.57097	5.95823	5.6811	5.99193	6.13431	5.85039	6.83459	6.2094	6.75293	6.48325
CDK19	Q9BWU1	6.07774	6.26346	6.21022	6.12293	6.14926	5.99623	6.40358	5.8132	6.22679	6.0066	5.97046	5.99426	6.71778	6.31961	6.77556	6.95232
PEX14	O75381	6.8703	6.28096	5.83042	6.3796	6.91155	6.02686	6.11976	6.36653	6.3049	5.80098	6.03115	5.84396	6.94123	6.02249	6.20592	6.06336
TNRC6A	Q8NDV7	6.34803	6.32547	7.1005	6.49077	6.21104	6.53904	6.66129	6.23537	6.46376	6.87197	6.48613	6.9109	5.25018	5.23679	5.28986	5.5789
NIPSNAP3A	Q9UFN0	6.08126	6.35991	5.80726	6.15362	5.93017	5.63259	9.31044	6.04379	5.4298	6.08982	5.52061	5.32785	6.27837	6.06258	6.93169	7.04024
ERCC5	P28715	5.34953	6.457	5.83186	6.24343	6.0815	6.03438	6.38713	5.92071	6.23281	6.14836	6.44192	6.31227	6.53805	6.64087	6.52733	6.85285
SLX4	Q8IY92	6.38427	6.43284	6.27817	7.05828	6.22102	6.51816	6.37892	5.56072	6.63144	6.63271	5.53644	6.06229	5.95697	6.03047	6.03102	6.28629
GRK2	P25098	6.22899	6.45541	7.37836	6.22265	6.36699	6.36555	6.29219	6.34076	6.4643	6.37946	6.67461	6.7915	5.56471	5.68241	5.22804	5.56408
CCDC85C	A6NKD9	6.38742	5.83827	6.46481	5.99976	6.433	6.28573	5.7332	6.69787	6.76089	6.27996	6.82829	7.27668	6.19967	5.97013	5.33412	5.51019
ATXN1L	P0C7T5	6.28894	5.95254	6.35326	6.44947	6.48136	6.18851	6.10779	6.18221	6.2736	6.32716	6.37533	6.71801	6.35914	5.83241	5.80143	6.30883
ERGIC2	Q96RQ1	6.45745	6.20625	5.93492	6.31506	6.52454	5.95358	6.46802	5.9976	6.27139	5.8127	6.10256	5.60492	6.64078	6.35406	7.1034	6.25277
GOLM2	Q6P4E1	6.44447	6.22992	5.73574	6.28787	6.42691	6.0029	6.43765	6.17013	6.75853	6.24957	6.06138	6.2684	6.38058	6.06844	6.07576	6.40176
POLG2	Q9UHN1	6.43284	6.17978	5.86421	6.6805	6.28404	6.06232	5.97392	5.7357	6.15146	6.12073	6.10606	6.27816	6.76531	6.05968	6.76178	6.5435
GDAP1	Q8TB36	6.25186	6.65151	5.41566	6.36068	6.09149	5.97793	6.19832	6.00899	6.20058	6.08184	5.9593	6.05111	6.75489	6.74874	6.5554	6.6917
PIBF1	Q8WXW3	6.33955	6.46665	6.91742	6.33343	6.59393	6.42464	6.60506	6.43934	6.48222	6.49851	6.63548	6.60876	5.71116	5.03838	5.64178	5.26366
CASP8	Q14790-4	6.38316	6.12983	6.96263	6.07475	6.44211	6.78373	6.2471	6.7321	6.41577	6.58065	6.69156	6.43692	5.55883	5.86571	5.30867	5.38647
GMDS	O60547	6.29686	6.40832	6.9747	5.83988	6.06528	6.39782	6.04878	6.48135	6.34735	6.71596	6.54523	6.5052	5.84333	5.55267	6.12595	5.85132
SUPT4H1	P63272	5.14399	5.91513	5.53618	5.27189	6.35991	5.87737	6.07722	6.842	6.30101	5.93972	6.79328	5.99186	7.31642	7.01738	6.78797	6.82867
TK1	P04183	6.26456	6.16043	6.77582	6.1734	5.96135	6.98548	5.93474	6.6426	6.55921	6.23389	6.61029	6.897	5.84413	5.57052	5.68316	5.70344
GALNT10	Q86SR1	6.35889	6.40795	6.29076	6.79068	6.26526	6.05467	6.50919	6.01297	6.45863	6.25981	6.53772	6.09679	6.23473	6.16316	6.14276	5.41604
HPCAL1	P37235	6.73594	5.2865	5.91973	4.866	5.54003	5.51358	5.21715	6.30063	5.40279	7.02522	5.78539	6.79867	5.79337	8.09365	7.45825	8.26309
NDUFS8	O00217	6.76666	4.91886	5.40934	4.83118	5.74222	4.61562	5.86375	6.68572	4.90836	6.15539	5.47986	6.37922	6.85888	8.39772	8.32677	8.66043
MGAT5	Q09328	6.25924	6.23988	5.7501	6.71517	6.41232	6.17249	6.23837	6.1702	6.02779	6.26605	5.86603	6.13858	6.58492	6.4313	6.4043	6.32324
COQ6	Q9Y2Z9	6.32314	6.40082	6.00662	6.52846	6.17298	6.09357	6.2592	5.89976	6.23181	6.23393	6.23387	6.20111	6.49738	6.42856	6.33541	6.15337
GM2A	P17900	6.68823	5.69406	5.30033	5.06416	5.92933	5.43653	5.44665	6.55801	5.15405	6.39084	5.09157	6.25147	6.34579	8.82829	7.52989	8.29081
DPH1	Q9BZG8	6.40323	6.08681	6.71641	5.78109	6.22996	6.4482	6.33155	6.42534	6.65283	6.21351	6.60268	6.98434	5.77641	5.82129	5.82099	5.70535
SNX7	Q9UNH6-3	6.35694	6.03306	6.65761	6.35179	6.30689	6.73807	6.29054	6.34315	6.09973	6.29734	6.70432	6.34612	5.65707	6.26565	5.75304	5.79869
SEMA4C	Q9C0C4	6.34123	6.72638	6.75636	6.80432	5.8986	5.99298	6.26272	6.05858	6.00865	6.3168	6.31298	6.40282	5.87305	5.95634	6.31626	5.97193
PEX11B	O96011	6.4931	6.0984	5.44604	6.25041	6.16933	5.83472	6.40679	6.28238	5.974	6.47038	6.08891	5.89182	6.84765	6.49518	6.63531	6.61559
NUP37	Q8NFH4	5.93829	6.05435	5.89752	6.24563	6.44558	6.24281	6.36489	6.46597	6.71114	6.57847	6.45124	6.3609	6.21787	5.60945	6.25349	6.16242
ERGIC1	Q969X5	6.51025	6.38559	5.81934	6.21229	6.14729	5.92703	6.29906	6.03722	6.2847	6.14016	5.91521	5.67785	6.68723	6.48813	6.7992	6.66944
PIN4	Q9Y237-2	6.14275	5.97724	6.43883	6.17093	5.94133	6.67205	6.51307	6.52846	6.57495	6.39244	6.23491	6.84326	5.63283	6.13721	5.82726	5.97248
PTAR1	Q7Z6K3	5.96191	5.99869	6.43419	5.76351	6.26961	6.53537	5.94934	6.60492	6.24234	6.65331	6.38562	6.3989	6.06116	6.37263	6.32628	6.04223
ZWILCH	Q9H900	6.47075	6.37455	6.89297	5.97892	6.47109	6.50173	6.24633	6.69499	6.43743	6.56675	6.75858	6.83768	5.41746	5.51538	5.45442	5.38099
STAMBPL1	Q96FJ0-2	5.68243	6.21077	6.71189	6.49969	5.86313	6.77739	6.15331	6.28132	6.0894	6.20756	6.57609	6.59776	5.83966	6.13117	5.98819	6.39024
BECN1	Q14457	6.57416	6.53803	6.61587	6.31474	6.35327	6.40597	6.27578	6.55488	6.38537	6.14881	6.45621	6.61966	6.86936	5.73813	5.60824	5.54153
HACD3	Q9P035	6.08701	6.34401	5.97219	6.52336	6.11391	5.97694	6.28217	6.15027	5.91399	6.18071	5.61009	5.75871	6.76961	6.45468	7.09291	6.76945
MMP14	P50281	5.69784	5.8746	5.3362	5.7719	5.72057	5.46602	5.88904	6.19017	5.37101	5.96001	5.71711	5.85454	6.5039	8.38132	7.72197	8.5438

NDUFA4	O00483	6.1616	5.34373	5.16154	5.63532	5.39043	4.72286	5.4824	6.51244	4.76485	5.64418	5.01601	5.82854	5.75401	10.0897	8.44278	10.0496
SOGA3	Q5TF21	6.05049	6.58813	6.31717	6.1611	6.24434	6.27097	5.86735	6.36262	6.11753	6.20993	6.35106	6.18841	6.05027	6.22898	6.41671	6.57494
TUBGCP4	Q9UGJ1	6.33249	6.18385	6.87324	6.19644	6.2177	6.3914	6.49069	6.6915	6.60673	6.51431	6.62028	6.78654	5.54324	5.40521	5.67257	5.47381
TMEM38B	Q9NVV0	6.28155	6.05143	6.07643	6.64696	6.06861	5.57859	6.36361	5.77462	6.29	5.83503	6.2836	6.12419	6.44101	6.28564	6.88151	7.01722
ADGRG6	Q86SQ4	6.13027	6.18426	6.17464	6.89221	6.0668	6.31934	6.47822	6.29256	5.73606	5.98486	6.11293	6.48482	6.53243	5.84882	6.17525	6.58653
ADI1	Q9BV57	5.12706	5.63483	6.32811	4.62695	6.56189	7.06412	6.00968	7.90117	6.35666	7.33464	6.83341	6.93064	5.00954	6.10373	6.06644	6.11112
USP1	O94782	6.35301	6.79921	6.84101	6.44736	5.99844	6.27908	5.90061	6.46888	6.59567	7.14369	6.88216	6.92347	5.10754	5.50503	5.34676	5.40807
POLR2G	P62487	6.42503	6.33548	6.38266	6.60732	6.63718	6.38021	6.35229	6.5809	6.59734	6.33486	6.5453	6.47992	5.87672	5.61959	5.18895	5.65626
ADNP2	Q6IQ32	6.60751	7.23652	6.1566	6.96144	6.01779	6.1198	6.14725	5.85542	6.2722	6.21382	6.25804	6.25823	6.13545	5.62032	5.90375	6.23588
MRPL23	Q16540	6.09207	6.2383	5.46651	6.58324	6.48714	5.96704	6.59362	6.48458	6.4161	6.45051	5.91608	6.10384	6.49203	5.96038	6.44926	6.2993
DERL1	Q9BUN8	6.12414	6.59446	6.08313	6.41885	5.88176	6.14044	6.01898	6.2019	6.04394	6.22768	6.08371	6.37317	6.5436	6.40051	6.41008	6.45365
CNOT6	Q9ULM6	6.2958	6.67104	6.62956	6.20092	6.34004	6.49492	6.40471	6.51481	6.46148	6.52336	6.73248	6.71913	5.46708	5.60439	5.60977	5.33048
DCTN6	O00399	6.21195	6.2067	6.72301	5.94133	6.70372	6.57937	6.02769	6.78676	6.18847	6.69642	6.5831	6.57004	5.84374	6.00413	5.0768	5.85678
GSE1	Q14687	6.60013	6.48335	6.33762	6.80688	6.44867	6.2799	6.37295	6.14437	6.28015	6.35216	6.51709	6.34186	5.99821	5.7286	5.62837	5.6797
PMM2	O15305	6.44676	6.07827	7.04694	5.64549	6.7689	6.91585	6.32462	6.53248	6.46882	6.34831	6.70072	6.43512	5.7507	5.76684	5.62408	5.14611
TMEM63B	Q5T3F8	6.30882	6.72878	6.79545	6.51793	6.46535	5.99257	6.56427	6.05739	6.05394	6.22703	6.0776	5.90993	6.198	5.95528	6.01291	6.13474
TBCK	Q8TEA7	6.1969	6.25429	6.73944	6.26857	5.97806	6.615	6.08913	5.99175	6.29124	6.46228	6.64703	6.94651	5.74247	6.181	5.90934	5.68701
LGALS3	P17931	5.44257	4.53638	5.44975	4.82484	5.13739	4.10987	5.01294	6.83295	4.45049	6.20608	4.99741	6.26417	5.43435	11.3028	9.21149	10.7865
ARL3	P36405	6.35415	6.05697	6.9131	5.78501	6.426	6.73182	5.94438	6.7568	6.30858	6.83435	6.58764	6.71018	6.12171	5.69313	5.30748	5.46872
RPS20	P60866-2	6.48302	6.68907	6.74053	6.05473	6.33729	6.64934	6.48612	6.15907	6.74133	6.64927	6.04477	6.30657	5.49262	5.99237	5.35804	5.81587
NSUN4	Q96CB9	6.48399	6.22002	6.28387	6.69078	6.37067	5.9532	6.50158	5.9366	6.51513	6.11521	6.07965	5.77763	6.12757	5.91593	6.79084	6.23733
MSL1	Q68DK7	6.19928	6.60633	6.23271	6.29885	6.90507	6.37763	5.50971	6.57365	6.18998	5.63788	6.98249	5.98022	7.62894	5.69742	5.4703	5.70954
C11orf54	Q9H0W9	5.83192	5.55655	6.67307	4.93556	7.11138	6.49915	5.92311	6.69896	6.54037	6.72731	7.17096	7.29213	6.06408	5.89056	5.88197	5.20293
MAP1A	P78559-2	6.01975	5.98788	6.70276	5.49399	5.99015	6.42539	6.13826	6.34424	6.77267	6.04973	6.77303	6.61391	6.01793	6.38272	6.09197	6.19563
PTMS	P20962	6.49887	6.86317	6.54627	5.68409	6.63221	6.35429	5.88074	6.80071	6.29987	6.09588	6.70511	6.01156	6.48907	6.50599	5.78212	5.85006
USP8	P40818	6.16982	6.13012	6.95882	5.85368	6.02271	6.70832	6.22275	6.49685	6.48342	6.38505	6.80705	6.46362	5.71201	6.16397	5.5711	5.85068
LIG4	P49917	5.7479	5.91473	5.93288	5.51331	6.1566	6.038	6.06813	5.89748	6.00732	6.01473	5.92337	5.88271	7.26311	7.06734	7.52516	7.04723
MTPAP	Q9NVV4	6.22273	6.23881	5.60971	6.74289	6.45738	6.25804	6.37525	6.0314	6.11286	6.12732	5.92938	5.67554	6.64021	6.37413	6.55454	6.64982
TIA1	P31483	6.45968	6.41629	5.75702	6.44592	6.25127	6.1982	6.08212	6.09203	5.86822	6.43846	6.27685	6.32462	6.71734	6.16812	6.26763	6.23624
POLE2	P56282	5.78444	6.41603	6.32871	5.88818	5.92544	6.68655	6.20647	5.87755	6.43243	6.41282	6.38936	6.32234	6.51012	6.10116	6.48646	6.23194
LGALS3BP	Q08380	6.72483	6.42019	5.87084	5.81685	6.28976	6.24649	6.63553	6.59513	6.25706	6.28134	6.25777	5.81337	6.20059	6.62068	6.10087	5.8687
CDK9	P50750-2	5.81316	6.2489	6.01123	6.48635	6.63172	5.52512	5.97498	6.23982	6.29942	5.64581	6.34033	6.24235	6.99262	6.37655	6.69144	6.48022
EXTL3	O43909	6.42776	6.62241	6.00294	6.88175	5.9727	6.10265	6.66013	6.30063	6.40492	6.21624	6.28661	6.4812	5.61687	6.05972	5.75798	6.2055
RAB11A	P62491	6.32928	6.50236	6.26613	6.27885	5.72954	5.98948	5.93712	6.0682	6.02075	6.51447	6.13832	6.33607	5.91026	6.42933	6.34813	7.20168
USP6NL	Q92738-2	6.57928	6.74219	6.16655	6.73177	5.68964	5.79379	6.04814	6.0423	6.03335	6.35275	5.75895	6.09638	6.34261	6.01896	6.59788	6.92545
MYO5C	Q9NQX4	6.08785	6.14413	6.01631	6.10189	5.92146	5.95112	5.78603	6.36224	5.95096	6.37576	5.87411	6.11625	6.95523	6.50911	7.5313	6.81625
FEM1B	Q9UK73	6.47316	6.62476	7.34477	6.5909	6.36101	6.24308	6.24856	5.94458	6.44099	6.2654	6.59625	6.76677	5.2951	5.46763	5.56335	5.77369
PHKA1	P46020	6.7721	6.78951	7.2995	6.6173	6.20859	6.69191	6.50586	6.74997	6.60442	6.68924	6.72837	7.04333	4.7824	5.01426	4.78502	4.71823
HMOX1	P09601	6.60141	6.62154	6.3783	6.76785	6.41326	6.16461	6.46044	6.43703	6.35854	6.31498	6.45809	6.45231	5.79581	5.45158	5.53297	5.79126
WASL	O00401	6.08236	6.18789	6.92061	5.54982	6.34037	6.62427	6.47597	6.09746	6.39317	6.63199	6.58867	6.56435	5.86161	6.01025	5.98319	5.68803
GALNS	P34059	5.79377	5.3655	5.96341	5.60709	6.29329	5.46634	6.04604	6.49388	5.86511	6.66073	6.10379	6.58856	6.60419	7.06348	7.19524	6.88956
CNPY2	Q9Y2B0	6.57682	7.04039	5.78398	6.35337	6.69949	6.47325	5.91899	6.9405	5.54173	5.73539	6.18674	6.18512	6.28958	6.06325	6.19585	6.01555
PPP2R5A	Q15172	6.3668	6.31603	6.62928	6.13565	6.15497	6.70438	6.37116	6.68494	6.14247	6.69063	6.14295	6.23368	5.97167	5.87333	5.87069	5.71139
RNF169	Q8NCN4	5.90779	6.13769	5.45337	6.57444	5.63572	5.71363	6.10961	6.08303	6.30368	6.36802	6.52547	6.80822	6.48192	6.29978	6.50765	7.08999
PHRF1	Q9P1Y6	6.32109	6.23991	6.18559	6.76512	6.13272	6.00849	6.3736	5.97647	6.20219	5.90306	6.0523	6.02835	5.97939	6.59222	6.8129	6.42658
ARL6IP4	Q66PJ3	5.67622	5.79336	6.35015	5.4329	5.23384	5.9845	5.54091	5.81012	6.30545	6.61025	6.74304	6.68892	6.27727	7.39666	7.24241	6.91399
SCLT1	Q96NL6	6.63573	6.42365	7.02339	6.45992	6.21667	6.65929	6.50048	6.77034	6.49849	6.55438	6.86679	6.93021	5.18109	5.05635	4.93005	5.29318
MBOAT7	Q96N66	6.08983	6.45225	5.70407	6.48355	6.34632	5.88545	6.50726	6.05612	6.03099	6.50222	5.87859	5.73755	6.44971	6.72465	7.27183	6.77962
ABT1	Q9ULW3	6.31898	6.22373	5.56654	6.64197	6.55512	6.08304	6.14248	6.09166	6.42441	5.65678	6.2504	5.67193	6.34084	6.1329	7.07184	6.82737
ARPC1A	Q92747	5.98696	5.91915	6.75983	6.29264	6.02487	6.60685	6.25031	5.86898	6.15875	6.37737	6.5481	6.66448	6.11005	6.20441	6.15242	6.07483
ARFIP1	P53367	6.19524	5.46153	6.35507	5.72001	6.63908	6.08632	6.4996	6.19256	5.67381	6.52479	6.16335	6.28858	6.85837	6.54348	6.37969	6.41852
TSC22D1	Q15714	6.28105	5.77339	7.19963	5.74746	6.50426	6.76234	5.85824	6.79693	6.29408	6.48971	6.82848	6.67594	6.16888	5.93445	5.20597	5.2992
DCUN1D1	Q96GG9	5.84283	6.24404	6.74857	5.63328	6.32965	6.63508	5.95391	6.11123	6.05733	6.15159	6.53607	5.94997	6.73677	6.4327	6.46933	6.16767
EDC3	Q96F86	6.05905	5.99507	6.49907	5.87984	6.09434	6.65953	5.92782	6.52948	6.75748	6.57391	6.69289	7.04068	6.2353	5.76459	5.69426	5.59669
APIP	Q96GX9	5.90695	5.85663	7.20689	5.58955	5.9678	6.08514	6.52165	6.06495	5.84436	6.08326	6.55936	6.15696	5.95448	6.57986	7.52529	6.09687
ATP4A	P20648	6.60128	6.74059	6.16211	6.70136	5.99034	6.26096	6.44929	5.96331	5.97842	6.39046	6.28854	6.2632	5.8031	5.94317	6.14053	6.32333
GNB4	Q9HAV0	6.48871	6.47409	5.87166	6.49609	6.58097	6.46848	6.22197	6.435	6.45855	6.12045	6.23569	5.96963	6.06774	5.96255	6.13903	6.00938
CRYZL1	O95825	5.89209	6.01905	6.78961	5.61746	6.61292	6.56845	6.4202	6.65666	6.31707	6.35006	6.75682	6.60022	5.98561	5.78985	5.85343	5.77048
TRPM4	Q8TD43	6.39437	6.2525	5.92771	6.93058	6.08699	5.75866	6.58931	5.87461	6.37768	6.11411	5.94914	6.22025	6.42403	6.23348	6.59143	6.27515
PLBD2	Q8NHP8	6.44622	6.5054	5.96466	6.14713	6.55757	6.47513	6.22861	6.1045	5.99741	6.18891	6.33656	6.16929	6.29226	6.27043	6.09361	6.2223
GDPD1	Q8N9F7	5.9906	6.38819	6.06538	6.53665	6.25111	5.99239	6.20597	6.08374	5.98691	6.15992	5.8401	5.62175	6.63313	6.68868	6.85411	6.70139
ETNK1	Q9HBU6	6.46904	6.46042	7.06628	6.82486	6.35876	6.69361	6.17347	6.45609	6.52917	6.76547	6.56754	6.88989	4.88895	5.25339	5.14081	5.46225
CBL	P22681	5.84121	5.89506	6.96554	6.22639	6.26862	6.9005	6.14203	6.49639	6.51228	6.31413	7.19759	6.7304	5.96388	5.25201	5.34918	5.94478
NCBP2	P52298	5.63882	6.06569	5.83388	6.23706	6.04752	6.256	5.81203	6.09727	6.10542	6.44332	6.25212	6.39273	6.69783	6.74022	6.71741	6.66267
PITPNA	Q00169	6.31425	6.72969	6.63836	6.30972	6.17421	6.77552	6.21779	6.94616	6.32795	6.70705	5.98912	6.21225	5.63964	5.83354	5.49949	5.68527
UBAP1	Q9NZ09-4	6.43958	6.57315	6.95557	5.58914	6.16486	6.5383	5.43283	6.88222	5.86561	7.10511	6.86827	7.76088	6.02904	5.75885	5.36059	5.57598
ITPKA	P23677	6.29607	6.39268	6.29218	6.38813	5.89662	6.13431	5.99904	5.85457	6.19419	6.23028	6.15669	6.08476	6.66815	6.16869	6.5771	6.66653
COX7A2L	O14548	6.16602	5.67995	5.69528	5.69844	6.53437	5.87084	6.37064	6.73951	5.32271	6.23298	5.41809	6.12036	5.9063	7.51336	7.27536	7.45579
ZZZ3	Q8IYH5	6.40027	6.43773	6.5054	6.87231	6.10217	5.80989	6.3425	5.91223	6.40188	6.32276	6.20389	5.78255	6.45058	5.95959	6.18505	6.31119
DCAF4	Q8WV16	6.43978	6.86217	6.26466	6.82655	6.7146	6.42069	6.68327	6.49108	6.4496	6.37656	6.20651	6.03031	5.3981	5.61437	5.57088	5.65087
APAF1	O14727	6.43992	6.34679	7.05292	6.11575	6.32753	6.83689	6.1999	6.71124	6.27933	6.46175	6.48881	6.17911	5.50494	6.05232	5.62628	5.37651
AMIGO2	Q86SJ2	6.50208	6.52397	6.6017	6.54168	6.02207	6.04422	6.40541	5.99661	6.13356	6.17194	5.92273	5.88461	6.65404	6.43165	6.24446	5.91927
MORF4L2	Q15014	6.9753	6.97233	6.80617	7.24775	6.29377	6.04654	6.34397	6.9272	7.34673	6.80366	7.23979	6.63055	4.07753	5.06094	4.34224	4.88553
MRM2	Q9UI43	6.29236	6.44745	6.05185	6.93572	6.04526	5.93286	5.87271	5.98657	6.2745	6.13517	6.36355	6.10679	6.69291	6.21134	6.28099	6.36998
LUC7L	Q9NQ29	5.29158	6.23879	6.68953	5.82183	5.58712	6.30382	6.19694	5.86442	5.93542	6.67218	5.69248	6.2809	6.75237	6.91308	6.92356	6.836
LIMD1	Q9UGP4	6.24243	6.14896	6.57754	5.93293	6.37621	6.41538	6.14935	6.31502	6.70843	6.28003	7.00398	6.86278	6.51146	5.66087	5.41509	5.39951
CHMP2A	O43633	6.23733	6.38204	6.50747	6.07128	5.94702	6.4618	6.26835	6.68321	6.05835	6.68244	6.15503	6.06821	5.64138	6.662	6.07425	6.09982
SHPK	Q9UHJ6	6.52701	6.17402	6.72123	5.67381	6.11283	6.84695	6.00975	6.59505	6.62574	6.21566	6.42214	6.65824	5.66627	5.80305	5.98871	5.95954
ZGPAT	Q8N5A5-2	6.57959	6.1485	6.14687	6.23252	6.19817	6.18439	6.17064	6.04395	6.39117	6.21155	6.63871	6.17495	6.30724	6.37021	5.97419	6.22734
HMGA2	P52926	5.81549	5.88008	5.57784	6.12332	5.83638	5.62591	6.33946	5.98736	6.08758	6.13483	5.68505	5.89076	7.05913	7.655	7.2778	7.02398
UHRF1BP1L	A0JNW5	6.45111	6.2196	6.37175	5.96622	6.33265	6.49103	6.28719	6.51979	6.53634	6.4242	6.35088	6.37545	5.73969	6.22654	5.86065	5.84691
CASP4	P49662	6.24139	5.97673	7.16846	5.74783	6.34663	6.75756	6.01716	6.68215	6.71551	6.26759	6.75292	6.73601	5.87328	5.41805	5.60968	5.68903
TMEM87A	Q8NBN3	6.33013	6.36763	5.94299	6.488	6.29573	5.90262	6.01399	6.08563	6.31219	6.39382	5.95941	6.01041	6.37323	6.27066	6.77066	6.48291
RNF10	Q8N5U6-2	6.6071	6.86731	7.20416	6.69603	6.3264	6.56517	6.32533	6.29684	6.91688	7.24196	7.38927	6.99703	4.13799	5.07415	4.49969	4.8547
PRKCD	Q05655-2	7.07273	7.43584	7.10129	7.20576	6.28209	6.89516	6.27734	6.97704	6.31389	5.97178	5.82437	6.11212	5.16717	5.16632	5.08449	5.1126
TUT1	Q9H6E5	5.50966	5.85778	6.39456	6.31593	6.23105	6.26843	6.09476	5.82119	5.92802	6.01537	6.24868	6.3822	6.74116	6.46065	6.68618	7.0444
PARP2	Q9UGN5	6.0434	6.22152	6.18096	6.13745	6.08561	6.45825	6.32326	5.92083	5.84337	6.34794	6.49895	6.01996	6.38833	6.62689	6.48937	6.41392
DVL2	O14641	6.39381	6.55722	6.63706	5.94053	6.48495	6.64791	6.59346	6.90529	6.20382	6.2305	7.03507	6.28389	5.44711	5.84938	5.46642	5.32356
MAN2C1	Q9NTJ4-4	6.04046	5.96286	6.78415	5.89239	6.76557	6.86505	6.10843	6.47188	6.6539	6.08515	6.3881	6.46869	5.65389	6.13525	6.12562	5.59863
TNS3	Q68CZ2	6.0736	5.91255	7.00621	6.1016	5.9672	6.15294	6.42031	6.18095	6.32999	6.59288	6.754	6.17225	6.4934	6.09211	6.22558	5.52443
SPTBN4	Q9H254	6.01552	6.19049	5.95591	6.35932	6.1237	6.13346	6.41989	5.96725	6.3213	5.68837	6.23737	5.87117	6.59001	6.42959	6.7904	6.90625
HEBP2	Q9Y5Z4	6.85495	5.35622	6.55513	4.85338	6.56922	6.55192	5.79264	6.78301	5.99303	6.62971	6.51473	6.67889	6.28707	6.61829	5.81327	6.14855
INSR	P06213	6.01598	6.24499	6.03565	6.20379	6.01854	6.00019	5.9637	5.89701	6.12276	6.35006	6.0275	6.27314	6.42204	6.60745	6.93137	6.88583
LPCAT4	Q643R3	6.22274	6.51577	6.31715	6.76437	6.28274	6.25892	6.35907	5.77834	5.59418	6.05269	6.33211	6.05652	6.64717	6.45502	6.39662	5.96658
SP2	Q02086	6.13226	6.33443	6.31386	6.47748	5.92471	6.20596	6.23964	5.9197	6.11265	6.0446	6.28508	5.97231	6.39274	6.43368	6.67369	6.53722
SLC9A7	Q96T83	6.20689	6.55613	6.30896	6.64493	5.89698	6.3962	6.42638	6.05698	6.08166	6.04145	6.86152	6.63895	5.8393	6.07271	5.89356	6.07739
HELB	Q8NG08	6.33951	5.848	7.20555	6.27131	6.0945	6.67924	6.1761	6.02098	6.00639	6.62511	6.59931	6.38925	5.78055	6.01988	6.1437	5.80063
UPF3B	Q9BZI7	6.11714	6.24223	6.53795	6.16478	6.22145	6.31708	6.27104	6.41782	6.5159	6.35999	6.27011	6.39147	5.80093	6.49396	6.01304	5.86512
SLC30A9	Q6PML9	6.24322	6.29442	5.97816	6.68004	5.96675	5.87544	6.31734	5.67706	6.26347	5.92294	6.15064	5.84414	6.77582	6.65216	6.71487	6.64354
CISD3	P0C7P0	6.44086	6.75274	5.92089	7.21188	6.40152	6.11168	6.1449	5.75083	6.08441	6.21561	6.25646	5.7671	6.68082	6.19396	6.30888	5.75744
MAGI3	Q5TCQ9-1	5.91813	6.19418	6.47751	6.01025	6.43384	6.50992	6.25018	6.1122	6.21326	6.2566	6.18297	6.28988	6.39785	6.34083	6.27931	6.13308
PEX13	Q92968	6.40521	6.4922	6.21719	6.7223	6.26957	6.04426	6.32941	6.00657	6.53125	6.31275	6.22241	5.94808	6.3473	5.80095	6.30765	6.0429
CAMK1D	Q8IU85	6.11882	5.8876	7.11811	6.23324	5.99924	6.47995	6.28043	6.55852	6.41053	6.97681	7.04392	6.50701	5.65287	5.729	5.56009	5.44384
SNX16	P57768	5.76146	6.11359	6.51456	6.54212	6.38648	6.46334	5.82986	6.4067	6.49482	6.57654	6.73922	6.86495	6.01542	5.95008	5.52232	5.81854
ZFYVE21	Q9BQ24-2	6.04891	6.37112	6.84116	6.12892	6.02555	6.48908	6.16764	6.289	6.21038	6.64378	6.97184	6.8322	5.35586	6.10593	5.85114	5.66748

WWP2	O00308	6.67697	6.88446	6.13259	6.52376	6.53691	6.32376	6.61512	6.57685	6.51349	6.18105	6.33157	6.32338	5.65473	5.66131	5.66577	5.39828
RAB15	P59190	6.46669	6.41996	5.93463	6.21031	6.47531	6.25633	6.42181	6.29453	6.46949	6.33859	6.58816	5.7688	6.13102	6.13867	6.71525	6.27046
RAB13	P51153	5.89866	6.66213	6.23854	6.83322	6.70346	6.2633	6.20535	5.91788	6.65453	5.94752	5.97664	6.46495	5.80153	5.95673	6.47969	5.99587
RAB4A	P20338	6.47598	6.8705	6.72132	6.54837	6.11219	6.63489	6.37875	6.48701	6.33121	6.42109	5.91966	6.26167	5.74056	5.69722	5.62576	5.77383
VAMP7	P51809	6.24883	6.68751	6.41654	6.48749	5.98692	6.39938	6.27076	6.02625	6.27073	6.5655	6.69578	6.7404	5.69477	5.85744	5.81204	5.83965
LANCL1	O43813	6.20384	5.62069	6.98031	5.9643	6.50068	6.95541	5.92693	6.35341	6.42812	6.39268	6.44638	6.08927	5.83484	6.18418	6.10159	6.01737
SPC25	Q9HBM1	6.44518	6.36618	6.80937	6.13289	6.71167	6.80909	6.30019	6.88602	6.69436	6.69887	6.4226	6.51692	5.70046	5.16509	5.06536	5.27576
RBIS	Q8N0T1	7.2448	7.47711	7.44236	7.5416	6.6658	6.62242	6.92449	6.9168	7.08875	7.26118	7.27925	7.21803	3.33766	3.68903	3.49014	3.80057
ZNF516	Q92618	6.50777	6.33054	5.72902	6.52997	6.20502	6.41971	6.13854	6.38263	6.29843	6.27505	6.37173	6.15277	6.24384	6.1924	5.99369	6.22891
XXYLT1	Q8NBI6	6.79318	6.56524	5.92076	7.06102	5.8261	5.74974	6.10976	6.20691	6.20802	6.39622	6.2149	5.8295	6.98084	5.8282	6.02245	6.28717
PSMB9	P28065	6.06591	6.61122	6.05997	5.44823	6.6521	6.82917	5.7392	6.96502	6.13991	6.41023	6.05134	6.31786	6.23371	6.072	6.23084	6.17329
DHRS1	Q96LJ7	6.5727	6.29616	5.79467	6.09986	5.82056	5.64831	6.18234	6.19173	6.02973	6.48344	6.40014	6.49718	6.42866	6.36671	6.78987	6.39795
LRRC1	Q9BTT6	6.32336	6.6398	6.29958	6.61582	6.58044	6.14167	6.17488	6.0201	6.25869	6.10335	6.3224	6.31698	6.58085	5.95545	5.68138	5.98523
XIAP	P98170	6.65964	6.11803	6.7564	5.9089	6.48174	6.92352	6.10578	6.79852	6.46912	6.41922	6.57203	6.46876	5.81911	5.61222	5.52917	5.35783
RUSC2	Q8N2Y8	5.87834	6.01815	6.48028	5.87465	6.39091	6.5762	6.28925	6.73267	5.7399	5.81656	6.147	5.89818	6.93963	6.46822	6.7192	6.03087
CENPH	Q9H3R5	6.36835	5.33704	6.87939	5.43888	6.46837	5.64272	5.72729	6.77192	6.07384	6.60094	6.66829	7.28598	6.10845	6.43122	6.11623	6.08108
MAN1A2	O60476	6.59996	6.38917	6.11901	6.64951	5.97813	6.12633	6.33914	6.15316	6.16937	6.11905	5.87021	6.10097	6.3338	6.41947	6.31163	6.32111
COX6C	P09669	6.04036	6.02528	5.42273	6.1972	6.83593	6.56456	6.68735	6.82652	5.53528	5.92588	5.27194	5.90948	7.12189	6.2843	6.74327	6.60803
MBTPS1	Q14703	6.24989	6.94061	6.2507	7.28243	6.09724	5.60188	6.34352	6.04962	6.17061	6.15755	5.79606	5.44819	6.50181	6.45818	6.6061	6.04561
EME1	Q96AY2-2	6.33861	6.70383	6.6472	6.38046	6.20679	6.51182	6.05109	6.33819	6.58068	6.29525	6.50829	6.61984	6.40261	5.89727	5.7738	5.74428
GTPBP2	Q9BX10	6.24849	6.69456	6.67717	6.64108	6.0138	6.26435	6.28498	6.17218	6.9537	7.20709	6.61034	6.55708	5.261	5.2752	5.57993	5.55905
GIT2	Q14161	6.42674	6.31179	7.10987	6.59683	6.13162	7.09151	6.13084	6.31307	6.22264	6.71916	6.85334	6.44512	5.37971	5.43403	5.53661	5.29711
GIT1	Q9Y2X7	6.2772	6.51303	6.51419	5.92005	6.44975	6.67816	6.06964	6.70665	6.28196	6.49778	6.4329	6.47659	6.09786	5.92117	5.74718	5.41589
P3H3	Q8IVL6	6.62503	6.33385	6.12996	6.75325	6.11717	6.34353	6.29324	5.95072	6.19214	6.0995	6.34206	6.23884	6.41366	6.13294	6.1033	5.9308
PHACTR2	O75167-4	6.04276	6.25977	7.17101	5.88736	6.06433	6.14511	6.26703	6.30514	6.48841	6.48924	6.59721	6.30721	5.88615	6.16086	5.80877	6.11966
TSR2	Q969E8	6.81914	6.29588	6.99696	5.73664	6.6741	6.64807	5.85741	6.49814	7.0107	6.50584	7.09648	7.08286	5.80817	5.02389	4.9328	5.01291
KMT2C	Q8NEZ4-3	6.39658	6.40353	6.23562	6.90109	6.06308	6.19598	6.38029	6.10166	6.5537	6.43357	6.56461	6.05418	5.98065	6.36943	6.23536	6.03066
PDCD6	O75340	5.58723	4.87565	5.88386	4.60887	5.9231	5.85848	5.73215	6.66016	5.46446	6.14017	6.06146	6.326	6.34821	8.31451	8.39747	7.81823
MFSD10	Q14728	6.00845	6.32145	5.80722	6.46658	6.30002	6.07975	6.25886	6.25945	6.22996	6.2867	5.77374	5.6209	6.57154	6.45416	7.01046	6.55078
ZNF512	Q96ME7	6.25639	6.46615	5.64551	6.61744	6.21121	5.70959	6.10905	6.6153	5.70036	6.02866	6.55944	5.90024	6.47286	6.95414	6.93484	6.71883
DPF1	Q92782-2	5.96133	6.41303	6.1414	6.2867	6.44383	5.90524	6.61651	6.10726	5.7051	6.19893	6.33892	6.19866	6.21844	6.63324	6.44989	6.38153
CEP295	Q9C0D2-3	6.0974	6.87426	5.90643	6.09951	5.42698	6.35037	6.80896	6.32442	6.48772	6.59642	6.93288	7.18679	5.0109	5.71035	6.24806	5.93856
PTPN9	P43378	5.92801	6.17035	6.19821	6.07047	6.27488	6.52113	6.13635	6.53097	6.2997	6.51424	6.72067	6.39289	5.92447	5.99746	6.49413	5.82607
AP1S1	P61966	6.31334	6.47335	6.57546	6.42794	6.36049	6.46895	7.1057	6.15491	5.78043	6.2773	6.82376	6.23541	5.34208	5.92859	5.81873	5.91357
SETMAR	Q53H47	5.66859	5.33963	5.96937	5.8138	5.72323	5.91754	5.31252	5.97425	5.95007	5.91431	6.41748	6.46233	6.88346	7.34922	7.60725	7.69696
NEK1	Q96PY6-3	6.24422	6.28733	6.5562	6.34286	6.03645	6.44542	6.15757	6.47492	6.18992	6.5287	6.43673	6.11841	6.25246	6.02643	5.92126	6.08114
ABHD11	Q8NFV4	5.76705	4.80472	5.54076	5.07114	5.85572	5.28052	5.33749	6.29723	5.24896	6.7059	5.71245	6.85641	6.99273	8.09917	8.12976	8.29998
YTHDF2	Q9Y5A9	6.90844	6.59403	6.66969	6.17593	6.30259	6.21266	6.08046	6.56294	6.53543	6.20925	6.22931	6.43473	5.87563	5.70258	5.88714	5.82278
ATXN3	P54252-1	6.58766	5.61203	6.58331	5.00918	6.58703	6.63093	5.81596	6.60852	6.1397	6.66875	6.14536	6.8765	5.85503	6.67576	6.10713	6.09715
RPS6KB1	P23443	6.32013	6.30607	6.5147	6.29229	5.8106	6.68814	6.04367	6.53702	6.29393	6.54347	6.37846	6.43843	5.83406	6.10653	5.69746	6.19504
PUS7L	Q9H0K6	5.44775	5.96699	5.84176	5.47023	5.84018	6.65175	5.51451	5.8759	6.18195	6.43772	6.34831	6.66779	6.50492	7.35134	7.15189	6.74702
EEF1E1	O43324	6.18398	5.23517	6.49261	4.59712	6.45539	5.92962	6.07401	6.40155	5.80535	6.52457	6.45213	6.7018	6.95157	6.90591	6.68093	6.60831
SAAL1	Q96ER3	5.22153	5.38462	6.1056	5.2274	5.8038	6.25459	5.68771	5.57743	5.60038	6.0691	6.38639	5.67934	7.78582	7.65855	8.03504	7.52269
RAP2A	P10114	6.26292	6.54858	5.95338	6.58623	6.22158	5.74177	6.42418	6.31995	5.88787	6.33247	6.45861	5.87716	6.23145	6.67059	6.14436	6.33889
RHOT1	Q8IXI2-3	6.37051	6.49372	5.95255	6.74263	6.4095	5.99186	6.38409	5.83969	6.49989	6.09512	5.86955	6.00726	6.31489	6.11013	6.49178	6.42682
ZFP91	Q96JP5	6.06448	5.88173	6.02123	6.17635	5.70739	5.96331	6.0528	5.87551	6.46528	6.20588	6.09221	6.00611	6.74873	6.90001	7.13687	6.70211
MAP4K3	Q8IVH8	6.6391	6.15952	6.57012	6.32995	6.29901	6.7675	6.28969	6.54586	6.5128	6.8915	6.74342	6.57977	5.85665	5.8639	5.2657	5.68552
PIK3R2	O00459	6.54831	6.22719	6.3665	5.70123	6.60707	6.90141	6.49964	6.58963	6.34417	6.24554	6.25681	6.42527	6.24944	6.11467	5.36212	5.56099
FOSL1	P15407	5.5727	5.06427	5.61389	5.57452	7.09122	6.07664	6.29303	7.03354	5.66238	5.73575	5.8774	5.69579	7.81408	6.52996	6.9337	7.43112
SNAP91	O60641-4	5.7134	5.95001	6.28599	5.98738	5.98908	6.51488	5.80355	6.02979	6.37349	6.45319	6.10559	6.49272	6.53387	6.45504	6.95133	6.36069
INPP5A	Q14642	6.25711	6.48131	6.16419	6.47873	6.10165	6.23428	6.37486	5.95126	6.09192	6.13915	6.38322	6.05652	6.2418	6.27313	6.46094	6.30993
UVRAG	Q9P2Y5	6.76252	6.77745	6.88821	6.48467	6.37994	6.68129	5.98049	6.46465	6.14612	6.21032	6.3841	6.52597	5.60843	5.71847	5.70822	5.27913
TRIM65	Q6PJ69	6.16689	6.19948	6.50114	5.76291	6.66109	6.17461	5.77138	6.90555	6.29986	6.12154	6.78484	6.94686	5.90681	6.05782	5.75313	5.9861
ENTPD5	O75356	6.31272	6.1358	6.15856	6.85821	5.91293	6.00733	6.1769	5.98882	5.80397	6.05845	6.01229	6.02406	6.74511	6.58117	6.55483	6.66886
GTF2E1	P29083	5.39466	5.87387	5.55194	5.17924	5.72662	6.3669	5.54355	5.86718	6.08605	6.23864	5.93275	6.49942	7.14253	7.78032	7.31818	7.49816
TPRG1L	Q5T0D9	6.44753	6.4659	6.53242	6.024	6.46014	6.48922	6.17933	6.40434	6.27987	6.87002	6.51351	6.91269	5.31165	5.7938	5.873	5.44259
KRIT1	O00522	6.63907	6.39836	7.09795	6.82	6.62719	6.32072	6.37482	6.38409	7.05377	6.86237	6.63777	6.16817	4.88623	5.38017	5.1205	5.22881
ZEB2	O60315	6.3689	6.26422	6.03937	7.35854	6.28941	6.06016	6.38037	6.22082	6.4082	6.58196	6.43038	6.2098	5.75614	5.81771	5.7994	6.01463
DDAH2	O95865	5.72244	4.44628	5.24711	4.26626	5.73433	5.15582	5.22907	7.05503	4.9867	6.9158	5.45207	7.08481	6.62469	9.24213	8.30508	8.53238
LZTFL1	Q9NQ48	6.4949	5.90842	6.63753	5.81712	6.67129	6.64895	6.08979	6.78063	6.23129	6.71844	6.54048	6.65879	5.95892	5.99255	5.44339	5.4075
AVEN	Q9NQS1	6.49884	6.54967	7.02223	6.36163	6.02569	6.40861	5.97785	6.8242	6.45872	6.80464	6.35541	6.92408	5.88298	5.3677	5.09635	5.44139
DISP2	A7MBM2	6.34457	6.41736	5.96468	6.58811	5.96308	5.60447	6.17317	5.92482	6.25398	6.13351	5.84854	6.41911	6.17726	6.59367	6.50399	7.0897
DENND4A	Q7Z401-2	6.52818	6.43401	6.67358	6.33879	6.26766	7.03048	6.38188	6.61087	6.86404	6.07651	6.66982	6.27533	5.35925	5.69548	5.40348	5.39064
MTF2	Q9Y483	6.40719	6.84143	6.35304	7.12678	6.11656	6.04779	6.77646	6.17007	6.22906	6.20493	5.638	6.16536	5.74293	5.93695	5.9987	6.24475
CTC1	Q2NKJ3	6.05733	6.0661	6.2022	5.95897	5.98946	6.42682	6.30891	6.04554	6.00205	6.2401	6.64055	5.93102	6.46402	6.44209	6.56595	6.6589
STIM2	Q9P246-2	6.82666	6.77161	6.15823	7.15747	5.6158	5.88029	6.24657	6.06614	6.50706	6.62256	6.36642	6.34113	5.75279	5.5606	6.30438	5.8223
PEX6	Q13608	6.58124	6.31301	6.00522	6.65763	6.53077	5.99537	6.39069	5.52999	5.94164	5.88589	6.15629	6.1728	6.381	6.52362	6.49605	6.43879
EIPR1	Q53HC9	6.13592	6.08432	6.14698	5.68417	6.67638	6.71859	6.18285	6.56036	6.55394	6.65559	6.64545	6.62018	6.00243	6.02099	5.74708	5.56478
IRF3	Q14653	6.55834	6.27756	7.27248	6.10743	6.29625	6.54111	6.20212	6.78101	6.57524	6.54604	6.61825	6.42196	5.60485	5.37676	5.53024	5.29037
GNAI3	P08754	6.18335	6.27952	6.29597	6.85181	5.85408	6.03948	6.28477	6.16248	6.38026	6.68106	6.47829	6.57946	5.99559	5.56308	6.35193	6.01887
GNA12	Q03113	6.50723	6.39659	6.32134	6.83272	5.87704	5.982	6.39938	6.22824	6.46998	6.32401	5.98917	6.4199	6.04229	6.58402	6.09898	6.42711
COQ8A	Q8NI60	6.35697	6.41348	6.02111	6.57658	6.01983	5.97196	6.11641	6.00478	6.3167	5.91144	6.12501	5.99919	6.74845	6.44979	6.37137	6.59691
MRPS34	P82930	6.00889	6.28515	5.49528	6.2769	5.99986	5.81848	6.4993	6.19511	6.46978	6.31434	5.94471	6.05813	6.54701	6.45721	6.44958	7.18028
RSPRY1	Q96DX4	6.03688	5.75464	6.45365	7.02895	6.056	5.99653	6.87309	5.40958	6.42929	5.79199	6.44672	6.57285	6.0141	6.00291	6.29295	6.83986
ELOC	Q15369	5.99173	5.24508	5.64376	4.72807	6.08976	5.98396	5.66793	6.94884	5.68318	6.61025	6.38766	6.45929	5.83328	8.05465	7.42158	7.25099
LSR	Q86X29	6.42877	6.54456	5.86205	6.61363	6.16325	6.22362	6.03689	6.01361	5.84756	6.30541	6.22034	6.14866	6.50425	6.29565	6.66835	6.1234
ANKRD12	Q6UB98	6.18487	6.66194	5.72616	6.70722	6.23335	5.94014	6.19575	6.19064	6.01148	6.25259	5.8844	5.96149	5.77457	7.04039	6.52897	6.70603
PSMD9	O00233	6.18807	5.56147	6.29839	5.68957	6.61142	6.27049	6.762	6.88103	6.78722	6.03189	6.62206	6.87921	5.40612	6.13557	5.40202	6.47347
UNG	P13051	6.2013	5.80192	6.3967	5.84281	5.97211	6.31695	6.3247	5.69924	6.12342	6.29246	6.75874	6.22812	6.3177	6.80443	6.57561	6.34379
ULK1	O75385	6.35159	5.60219	6.67676	6.06996	6.26012	6.5185	5.9508	6.1049	6.36746	6.40151	6.28292	6.7934	6.17429	6.22335	5.79841	6.42386
NCK1	P16333	6.44736	6.34142	6.42335	6.10396	6.52971	6.64027	5.99279	7.11205	6.28366	6.09394	6.61603	6.57954	6.07862	5.89654	5.46653	5.39422
LEO1	Q8WVC0	6.3457	6.65275	5.69293	7.0558	6.32609	6.33079	6.31957	5.78835	6.42162	6.26976	5.41936	6.06789	6.23272	6.23786	6.1099	6.3789
CERS2	Q96G23	8.65083	8.39133	7.85232	8.6802	7.8099	7.23306	7.36298	5.79094	5.9558	4.91394	3.93421	4.14264	4.789	4.77093	4.61076	5.11116
REV3L	O60673	6.53152	7.1618	6.74296	7.17725	5.91396	5.8306	6.17269	6.23723	6.17924	6.57798	5.75854	6.7946	5.14093	5.50066	6.07138	6.20865
RFX7	Q2KHR2	6.66746	6.76696	6.54533	6.5441	6.22888	6.18842	6.0739	6.17807	6.52042	6.81236	6.71229	7.02928	5.60867	5.27725	5.22311	5.62349
RASA3	Q14644	6.72069	6.57119	7.02059	6.5911	6.49732	6.36959	6.36	5.97901	6.22931	6.15073	6.2909	6.30315	5.74838	5.61657	5.74535	5.80612
ASCC1	Q8N9N2-2	6.16475	6.29331	6.35961	5.94236	6.52232	6.76332	6.03021	6.64001	6.30856	6.55593	6.3141	6.29459	6.12119	6.07959	5.63971	5.97044
HLA-A	A0A140T8Y4	6.4408	6.59036	6.19707	6.50828	6.60204	6.47645	6.49594	5.92201	6.21034	6.29686	6.31094	6.3628	6.35367	5.80093	5.55442	5.87709
RPL29	P47914	6.07595	6.74445	6.173	6.1163	6.55452	6.51401	6.48734	6.80004	6.5922	6.34331	6.09045	5.80454	5.54762	6.48585	5.91686	5.75357
TSG101	Q99816	6.37094	5.96575	6.65218	5.53719	6.53003	6.62458	6.24753	6.82818	6.25338	6.52481	6.809	6.78981	6.15181	5.74096	5.49637	5.4775
FAM98B	Q52LJ0	5.40985	5.57302	6.03261	5.56332	5.91683	6.28746	5.79043	5.911	5.99348	6.23384	6.3492	6.2515	7.4252	7.12103	7.20003	6.9412
DUS2	Q9NX74	6.13239	5.94328	6.5116	6.33848	6.06449	6.26929	5.77056	5.87948	6.00373	6.57309	6.0779	6.60732	6.22222	6.43338	6.42583	6.74698
ARHGEF18	Q6ZSZ5	6.40364	6.74783	6.77771	6.63338	6.40234	6.6794	6.31048	6.95141	6.3302	6.34855	6.23203	6.22849	5.39297	5.8441	5.26176	5.4557
FAM193B	Q96PV7	6.40211	6.41091	6.87311	6.41306	5.87912	6.05368	6.13733	6.22747	6.71124	6.53109	6.48265	6.58031	5.42437	6.02682	5.93598	5.91074
RRP8	O43159	6.11137	6.07881	5.98724	7.03492	5.92715	5.81888	6.30181	5.86627	6.10514	5.92704	5.93699	6.02459	6.75717	6.41351	6.81724	6.89187
UBQLN4	Q9NRR5	6.21357	5.24386	5.40016	5.11678	5.68007	4.44206	5.21452	6.40883	4.65319	6.37745	5.46027	7.10337	6.41123	9.21569	8.23344	8.82549
MRTFB	Q9ULH7-5	6.32199	6.45348	6.55628	5.43418	6.51448	6.77059	5.96214	6.11288	6.47909	6.59408	6.33277	6.61158	6.34054	6.00274	5.65043	5.86275
CNTLN	Q9NXG0-2	6.43763	6.159	6.75579	6.66752	6.0957	6.56944	6.48135	6.53401	6.40564	6.72224	6.72034	6.86905	5.18648	5.49104	5.31577	5.58899
THOC6	Q86W42	5.71464	5.85501	5.65535	6.62856	6.1668	5.92623	6.18901	6.21244	6.49992	6.65669	6.43511	6.19449	6.58418	5.94642	6.71181	6.62334
PARVA	Q9NVD7	5.72006	6.23872	6.1714	5.64105	6.20993	6.63931	5.88725	6.37439	6.3095	6.78793	7.03904	6.66219	6.17675	6.04211	5.9518	6.14857
TPD52	P55327-3	6.59912	5.37801	6.64576	5.20776	6.22341	6.06676	5.76408	6.65106	6.27926	6.76808	6.4097	6.76012	6.77643	6.39769	6.09089	5.98187
RAB27A	P51159	6.4449	6.58502	6.22647	6.49925	6.13572	6.09631	6.39662	6.18395	5.92009	6.5002	6.04469	6.01992	6.1721	6.09114	6.48966	6.19397
PRDM10	Q9NQV6-6	6.42665	6.23655	5.3813	6.72981	6.18253	6.03797	6.19732	6.30502	5.86019	6.22548	6.38918	5.95374	6.52047	6.29628	6.44149	6.81602
TAB1	Q15750	6.68916	6.51536	6.73224	5.43085	6.71881	6.62591	5.95233	6.73011	6.67766	6.63302	6.49502	6.59144	5.81722	5.54954	5.40149	5.43981
HDAC8	Q9BY41	5.58621	5.50141	6.49366	5.45959	6.02435	6.90908	6.15556	5.843	5.9387	6.26745	6.401	6.16063	6.7034	6.90769	6.95328	6.69499

CNPY3	Q9BT09	6.29432	6.63333	5.85698	6.42453	6.45109	6.16564	6.42761	5.91134	5.9255	5.98318	5.46066	5.92378	6.67704	6.7867	6.51933	6.55896
ARHGAP4	P98171-2	5.91991	6.50005	6.65162	5.99806	6.56901	6.68741	5.67993	6.29182	6.46482	6.87627	6.67315	6.10341	6.20949	6.0873	5.69891	5.58886
C19orf53	Q9UNZ5	5.73295	6.01819	5.76018	5.81881	5.84213	5.85559	5.77841	5.96435	6.1353	6.35455	6.02864	6.2668	6.50959	7.44074	7.22634	7.26744
OMA1	Q96E52	6.33169	6.56702	6.10361	6.70055	6.33346	6.3812	6.6413	5.60847	6.21089	6.22971	5.81321	5.59307	6.35016	6.23283	6.38787	6.51495
PSMD10	O75832	5.9769	5.39887	6.76003	5.16782	6.23976	6.18259	6.26375	6.46268	6.09557	6.62195	6.60407	7.08367	5.94305	6.51101	6.49706	6.19123
HDHD5	Q9BXW7	6.64481	6.1442	6.25418	6.68231	6.22546	5.71987	5.96955	6.08873	5.84156	6.39235	6.59712	6.62913	6.64145	5.86648	6.17925	6.12355
GTF2H2C	Q6P1K8	6.4031	6.10408	6.23335	6.56249	6.46517	6.29889	6.42613	6.2023	6.34873	6.59962	6.3028	6.18801	5.92535	5.90018	6.02048	6.01933
RAB32	Q13637	6.38153	6.51952	6.1464	6.35848	6.00774	6.0689	6.41863	6.19645	6.17635	6.43365	6.16797	6.04618	6.40728	6.19753	6.45926	6.01412
RPA3	P35244	4.44276	4.59777	5.05203	4.04431	5.71946	5.78332	4.89904	7.04515	5.27086	6.31039	5.86568	6.74483	6.12691	10.0773	8.60705	9.41314
NDEL1	Q9GZM8-2	6.51131	6.23514	6.69151	6.22282	6.4341	6.42711	5.56392	6.70588	6.42389	6.64927	6.90106	7.0164	6.21474	5.27663	5.24954	5.47667
BORCS6	Q96GS4	6.51733	5.9069	6.18835	5.47946	6.31962	6.11646	5.63601	6.44507	5.89589	6.42307	6.31177	6.80932	6.88543	6.55694	6.35654	6.15185
ASXL2	Q76L83	6.28406	6.88185	6.09495	6.84544	6.06944	6.46135	6.06523	6.13023	6.38475	6.15051	5.97615	6.42239	6.18324	5.88018	6.11527	6.05496
CALCOCO1	Q9P1Z2	6.35116	6.12091	6.6156	5.58567	6.6671	6.92748	6.12855	6.9537	6.25053	6.65277	7.17431	6.59083	6.26658	5.48067	5.0561	5.17804
FSD1	Q9BTV5	5.99201	6.19214	7.13611	6.13174	6.0098	6.90108	5.78075	6.51746	5.94705	6.92014	6.758	6.69016	5.81792	6.05976	5.31106	5.83483
SCAPER	Q9BY12	5.97104	6.35631	6.75319	6.03567	6.09942	6.66269	6.26759	6.18191	6.36665	6.76599	6.46368	6.56552	5.65801	6.04743	6.02229	5.78263
RNF113A	O15541	5.90323	5.89597	6.08296	5.80225	6.20524	6.39731	5.52961	6.48749	6.12336	6.0659	6.90983	6.36373	6.86209	6.54723	6.50707	6.31671
ABHD17B	Q5VST6-2	6.58354	6.47994	6.44214	7.18368	6.08447	5.72243	6.34952	5.75508	6.13197	6.53772	6.13599	6.62701	5.40045	5.96799	6.01126	6.58682
DUSP23	Q9BVJ7	6.06841	4.99789	6.41692	5.2107	6.45462	5.88114	5.58757	6.58312	5.80394	6.85598	6.48452	6.91408	6.02838	7.06484	6.51349	7.13443
ZNF518A	Q6AHZ1	7.11966	6.98405	6.06578	6.72136	5.93236	5.74674	6.05321	6.15021	6.23088	6.94626	6.39716	6.37219	5.61625	6.18842	5.68296	5.79249
STX10	O60499	6.66202	6.15506	5.97408	6.45985	6.16954	5.83199	6.2099	6.14262	6.31859	6.38229	6.02319	6.54864	6.54723	6.62989	6.51156	6.33355
RMDN3	Q96TC7	6.36778	6.36175	6.03726	6.89448	6.44583	6.23151	6.46686	5.98025	5.99041	5.86406	5.75065	5.92308	6.71866	5.78143	7.01264	6.17335
SNRPB2	P08579	6.1997	6.47078	5.83085	6.57491	6.15101	6.05639	6.16638	6.17106	5.84678	6.19479	5.98487	5.90042	6.76616	6.50228	6.64276	6.54087
LPCAT2	Q7L5N7	6.41344	6.17659	6.29371	6.35293	6.01623	5.96191	6.15737	5.59571	6.05777	6.24569	6.09011	5.83803	6.67621	6.53675	7.02818	6.55936
TAF7	Q15545	6.57635	6.55053	6.98142	6.5972	6.31479	6.50578	5.92461	6.86228	6.74459	6.41827	6.74408	6.83748	5.4695	5.31636	4.97906	5.17772
CYHR1	E9PJD7	5.7536	6.28952	6.01758	6.3097	5.8468	6.08448	6.07393	5.63199	5.98795	6.2764	6.33094	6.16859	7.06192	6.4283	7.0664	6.67189
NDUFA7	O95182	6.78512	6.49191	5.73911	6.83294	6.45048	5.87289	5.86269	5.94789	6.19502	6.1736	6.07185	6.12796	7.05546	6.21093	6.13776	6.04441
EIF1AX	P47813	5.96569	6.09928	6.13678	6.10168	6.25346	6.28207	6.60314	6.1424	6.58135	6.27499	6.32421	6.55396	5.69726	6.45093	6.28526	6.24754
NR2C1	P13056	6.4302	6.74077	5.64521	6.83985	6.44925	6.41619	6.33343	6.4013	6.13083	6.09544	6.19697	6.3743	6.21504	5.75541	6.00561	5.97019
PLEKHM2	Q8IWE5	5.92298	6.5752	6.44155	5.91356	6.16276	5.98971	5.64853	6.36627	6.45137	6.79416	6.55973	6.65955	6.22036	5.87162	6.32405	6.09859
MRPL16	Q9NX20	6.51425	6.86279	5.67148	6.67221	6.20471	6.08954	6.21996	6.14289	5.98498	5.91784	5.73446	5.89356	6.45219	6.43293	6.3968	6.80941
PROCR	Q9UNN8	6.32283	6.2895	5.91987	6.38545	6.4135	6.33739	6.45727	6.13752	5.80289	6.27171	6.41019	6.16349	6.22166	6.20718	6.27309	6.38645
PLEKHF2	Q9H8W4	6.33439	6.70776	6.96485	6.32281	6.22074	6.52871	5.79802	6.60985	6.53686	6.24262	6.18859	6.43946	5.86932	5.97554	5.59379	5.66669
UGCG	Q16739	6.69507	6.50335	6.62399	6.81673	6.90618	6.49045	6.8904	6.91325	6.18754	5.58438	5.94375	6.11214	5.5534	5.27542	5.55353	5.95042
ITGB3	P05106	5.95961	6.64669	6.61347	6.76742	6.08894	6.18757	6.3656	5.38705	5.92964	6.26353	5.79288	6.06171	6.47482	6.0273	6.56049	6.23328
PHF19	Q5T6S3	6.35137	6.6823	6.63027	7.00233	6.29878	6.41597	6.53256	6.59778	6.46657	6.46442	6.68398	6.75777	5.12453	5.35251	5.20432	5.43455
FBLIM1	Q8WUP2	6.59874	6.09169	6.62642	5.66845	6.70983	6.77463	5.8949	6.82547	6.08116	6.20227	7.02891	7.1572	6.10602	5.41881	5.49555	5.31996
SLIRP	Q9GZT3	5.93232	6.48908	5.58324	6.58283	6.71912	6.25742	6.09183	6.58706	6.18394	6.24374	6.02486	6.27629	6.22088	5.9413	6.51457	6.35153
COPS7A	Q9UBW8	6.16789	6.15491	6.12118	5.83525	6.2778	6.59939	6.197	6.58523	6.03148	6.24979	5.90253	5.94935	6.58708	6.41778	6.48043	6.4429
SNX3	O60493	6.10605	6.53356	6.67635	5.69463	6.38679	6.33364	5.94496	6.57554	6.34594	6.81278	7.15223	6.93462	6.02795	5.89646	5.67349	5.80501
MTHFS	P49914	5.82942	6.36811	6.63415	6.45284	6.00654	6.00631	6.16242	6.00191	6.4752	6.23625	6.40964	6.40442	6.26132	6.02857	6.33559	6.38731
ESCO2	Q56NI9	6.63746	6.15304	6.13208	6.16586	6.11784	5.6544	6.13907	5.43948	6.55941	6.12432	5.87361	5.59581	7.29001	6.27952	7.10174	6.73634
WDFY3	Q8IZQ1	6.3351	6.16672	6.74004	5.95506	6.24583	6.3996	6.43598	6.00928	6.29787	6.20138	6.41037	6.35584	6.04251	6.26772	6.04667	6.09004
ECD	O95905-3	6.62705	6.50342	7.53363	6.85047	6.58175	6.7396	6.57332	6.69158	7.12518	6.8299	7.03199	7.33765	4.51864	4.16758	4.00864	4.8796
RAD51AP1	Q96B01	5.70472	5.84141	6.65676	5.80937	5.75926	6.0606	5.9371	6.06417	6.67653	6.56409	6.64165	6.744	5.98216	6.62069	6.28914	6.64836
SLBP	Q14493	5.187	5.60643	5.91739	4.7783	5.62151	5.7392	5.16169	5.66325	6.19445	6.28023	6.1492	5.99829	7.97833	7.6947	8.21348	7.81654
NDRG3	Q9UGV2	6.77311	6.16989	6.93757	5.66086	6.54367	6.87366	6.21207	7.06998	6.27734	6.59409	6.32618	6.91941	6.163	5.56829	5.03365	4.87722
BAP1	Q92560	6.33563	6.25525	6.20931	6.40249	6.4657	6.62096	5.98315	5.91323	5.97966	6.30289	6.17671	5.91475	6.20139	6.44034	6.48621	6.21231
MPDU1	O75352	6.3533	6.18863	5.96234	6.65162	6.02135	5.78346	6.4751	6.13967	6.10894	6.12423	5.98346	5.82503	6.6129	6.29583	6.6308	6.84335
ABCD1	P33897	6.57594	6.43359	6.25705	6.34621	6.18476	5.99414	6.39349	6.58923	5.76614	6.19742	6.15013	6.05785	6.54924	6.30818	6.72048	6.21638
TIMMDC1	Q9NPL8	6.43495	6.69178	5.70943	6.69806	6.27442	6.36743	6.63573	6.04294	6.01778	5.92168	5.85754	5.80178	6.61758	6.41669	6.21029	6.30192
DNAJC21	Q5F1R6-3	6.67458	6.47571	7.11205	6.37096	6.16917	6.41284	6.32385	6.32769	6.61878	6.77658	6.7124	6.45216	4.965	5.77226	5.15772	5.67823
BPHL	Q86WA6	5.68057	6.29313	5.68158	6.33269	6.31761	6.19882	6.17018	6.34296	6.24526	6.05256	5.73911	6.07311	7.2655	6.59311	6.23244	6.78137
APOOL	Q6UXV4	6.60615	6.2718	5.84008	6.41782	6.43734	6.23636	6.7205	5.70433	6.29655	6.68513	6.12138	5.7472	6.62765	5.88502	6.2071	6.19559
SERINC3	Q13530	6.23059	6.46081	6.60552	6.73125	5.76283	5.83939	6.04626	6.19022	6.37935	6.37297	6.71012	6.66345	5.4434	6.1797	5.93459	6.44954
ARFRP1	Q13795	5.94411	5.97016	5.88626	6.6552	6.09527	6.41329	6.40317	5.98020	5.91694	6.26919	6.73258	6.10597	6.71256	6.2612	6.40511	6.42868
PFKFB3	Q16875-4	5.95077	5.608	7.06301	5.44056	5.56814	6.12427	5.83338	6.25975	6.19115	6.35978	6.73757	6.37333	6.52062	6.64376	6.91097	6.41492
THEM4	Q5T1C6	6.2871	6.21782	6.13129	6.75135	5.80795	5.67178	6.35286	5.94888	6.19223	5.91293	6.15075	5.91289	7.08803	6.20317	6.72217	6.6488
TNFRSF10A	O00220	6.36638	6.82918	6.00143	6.37777	5.88588	6.11387	6.62148	6.53265	6.02452	5.93792	5.84911	6.20259	6.02476	6.27435	6.42287	6.53523
UHRF1BP1	Q6BDS2	6.67117	6.08932	7.18333	5.77956	6.35388	6.39321	6.29751	6.49771	6.66522	6.23036	6.54671	6.79726	5.85927	5.41859	5.50816	5.70872
LCMT1	Q9UIC8-2	6.37315	5.70441	6.84268	5.94191	6.53276	6.89679	6.62838	6.37382	6.31852	6.28931	7.00276	6.72318	5.76863	6.06965	5.34576	5.18828
HAX1	O00165	6.75731	6.33575	5.89255	6.35674	6.67043	5.88532	6.07537	6.19663	6.12399	6.26788	6.52139	5.98388	6.32838	6.01571	6.28277	6.3059
SORBS3	O60504	6.33997	6.09616	6.54304	5.84081	6.60256	6.80428	6.09379	6.76834	6.56189	6.60888	6.04457	6.25207	6.0138	5.96277	5.674	5.79305
CASKIN2	Q8WXE0	6.28778	5.71939	6.71195	5.71032	6.01726	6.50183	6.10172	6.35739	6.01194	6.75091	6.25289	6.9752	5.78308	6.85202	5.55655	6.40978
KCTD10	Q9H3F6	6.74596	6.72691	6.98696	6.72039	6.53216	6.6481	6.90258	6.47516	6.80744	6.35437	6.76046	6.37309	5.01431	5.08171	4.86056	5.00983
CCDC97	Q96F63	6.16655	6.38475	6.25051	6.18115	6.52755	6.49564	6.00993	6.44767	6.45031	6.34274	6.54244	6.65478	6.40389	5.5805	5.72611	5.8355
CALU	O43852-3	6.14735	6.2788	5.40349	6.01432	6.55584	6.64251	6.015	6.40631	5.81265	6.05222	5.97313	6.17997	7.22962	6.84219	5.87555	6.57105
HS2ST1	Q7LGA3	5.88576	6.43631	5.69923	6.99635	6.12591	5.58606	5.52631	6.05994	6.05614	6.02078	6.65282	6.99058	7.46119	6.30537	6.30078	5.89647
ANGEL1	Q9UNK9	6.56697	6.71326	6.37847	6.84548	6.8139	6.29722	6.31534	6.2837	6.35353	6.43432	6.31944	5.91102	5.71255	5.65003	5.74035	5.66441
ZRANB3	Q5FWF4	6.01199	5.88828	6.25688	6.40196	6.47739	6.30848	6.69639	5.59347	6.34292	6.30826	6.45018	6.28499	5.89906	6.01289	6.62796	6.43891
F11R	Q9Y624	6.23932	6.4882	6.62162	6.71037	6.20534	6.27816	6.11292	6.37478	5.9086	6.13899	6.30464	6.01086	5.88747	6.51077	6.43325	5.7747
CDC45	O75419-3	6.07309	5.80605	6.31569	5.86475	6.01043	6.23556	5.96644	6.19137	6.48279	6.3169	6.41821	6.49696	6.07535	6.57612	6.53643	6.63386
PIGU	Q9H490	6.54211	5.96958	5.64636	6.63555	6.36891	6.03566	6.65881	5.97082	6.25354	6.15781	6.00751	6.12298	6.47645	6.21786	6.70835	6.22772
UQCRH	P07919	6.81433	6.20069	5.44644	5.65284	5.21028	4.69114	5.47694	5.84689	5.13991	6.07666	5.42733	6.17295	6.6282	8.77817	7.46472	8.97249
ZNF24	P17028	6.03746	6.21687	6.39994	6.61738	5.70613	6.11981	6.17049	6.34863	5.85338	6.25608	6.63491	6.11569	6.50641	6.56302	6.24676	6.20704
MECR	Q9BV79	6.40986	6.04047	5.70841	6.31494	6.51979	5.81349	5.75522	6.54953	6.11702	6.06917	6.35236	6.03781	7.31628	6.79072	6.20789	5.99703
GUK1	Q16774-2	6.11498	5.89813	5.9935	6.36424	6.21343	6.33755	5.8975	6.55254	6.29717	6.73464	6.45712	6.48303	6.36559	6.2834	5.73126	6.27593
CADPS2	Q86UW7	6.4803	6.11261	7.12322	6.2924	6.30855	6.5048	6.34373	6.52831	6.53554	6.39414	6.66321	6.86501	5.53012	5.37251	5.43829	5.50726
GPX4	P36969	6.5648	6.1143	6.43159	5.76938	6.74246	6.38944	6.24809	6.70322	6.47112	6.35654	6.23298	5.99219	6.36119	5.91592	6.09365	5.61314
IRAK1	P51617	6.69084	6.04992	7.04123	6.03886	6.0538	6.46258	5.80055	6.66588	6.12527	6.83168	6.96894	7.46143	5.41106	5.63843	5.31764	5.44191
PRPF38A	Q8NAV1	5.89532	5.95625	6.24061	7.04247	5.79066	6.02266	6.07142	5.54033	6.27443	5.99568	6.23257	6.14111	6.92067	6.09708	7.02833	6.75044
DDX59	Q5T1V6	5.64262	5.60509	6.22693	5.40989	5.66326	6.12296	5.68085	6.18645	6.16385	5.94117	6.25723	6.49575	6.63022	7.51965	7.43361	7.02046
ALDH3B1	P43353	6.21553	6.68476	6.12641	6.56119	6.29954	5.95107	6.15201	6.16636	6.64613	6.06737	6.37357	6.43245	5.94165	6.26866	5.85873	6.25457
RPS26	P62854	6.52786	6.57261	6.59715	6.04216	6.47344	6.58404	6.27149	6.44995	6.44804	6.56634	6.17528	6.43048	5.2769	6.25637	5.63002	5.69788
STX16	O14662	6.6377	6.48987	5.94161	6.58575	6.44648	6.28309	6.45409	6.17506	5.96002	6.43442	6.31719	6.22981	6.00842	6.08929	5.9885	5.9587
RPS6KA4	O75676	6.02746	6.1038	5.95052	5.65708	5.99305	6.34831	5.95055	5.99076	6.13178	6.28786	6.73768	6.04791	6.41825	6.92153	6.72972	6.70372
ARL8A	Q96BM9	6.18563	6.57293	6.27846	6.37495	5.92694	5.89571	6.11586	6.01059	6.18449	6.43295	6.01096	6.34165	6.20745	6.53433	6.36952	6.55757
BABAM1	Q9NWV8	6.42534	6.23583	6.47587	5.83198	6.57837	6.54123	6.06145	6.7831	6.56151	6.5649	6.50603	6.41141	6.4731	5.44164	5.60916	5.49908
NAGA	P17050	6.59549	6.46857	6.46281	5.71549	6.30942	6.31328	6.285	6.71386	6.05272	6.1714	6.40131	5.98997	6.22292	5.82103	6.24996	6.22677
POMT2	Q9UKY4	6.25938	6.57181	5.644	6.45262	6.48499	6.00517	6.1319	5.78429	6.46542	6.09762	6.01256	5.80215	6.56484	6.47713	6.94852	6.2976
ATRIP	Q8WXE1	6.13983	6.10849	6.7525	6.23	6.23978	5.94371	5.9259	5.73787	6.25505	6.4044	6.50058	6.13232	6.3456	6.44814	6.3402	6.49562
MRPS18A	Q9NVS2-2	6.14807	6.12719	5.77883	6.58663	6.01697	5.84693	6.38969	6.13369	6.43311	6.1963	6.11738	6.05205	6.59025	6.4434	6.74533	6.39421
SASH1	O94885	6.15466	6.85406	6.80016	6.28666	6.51143	6.64246	6.37042	6.48009	6.43024	6.4329	6.60574	6.25494	5.54883	5.59988	5.41441	5.61314
SMPDL3A	Q92484	6.44095	6.18507	5.77177	5.92656	6.95318	6.30684	6.17646	6.24212	6.15961	6.22332	6.1921	5.81077	6.89916	6.27208	6.48059	5.95943
VEZT	Q9HBM0	5.85449	6.46776	5.8799	6.60859	5.96411	6.20783	6.2802	5.91053	5.7904	6.45147	6.48993	6.30852	6.48334	6.18136	6.70461	6.41693
POMT1	Q9Y6A1	6.64875	6.50548	6.77667	7.04013	6.35169	5.92587	6.3923	6.61775	6.61188	6.23059	6.28644	6.0979	5.59293	5.13303	6.17288	5.61571
PCNP	Q8WW12	5.69055	6.28532	6.03994	5.763	5.74645	6.72318	5.42253	6.80848	6.05869	6.51974	6.40457	6.41226	6.11822	7.19685	6.239	6.57121
WDR47	O94967-4	6.32524	6.459	6.60979	5.82412	6.65347	6.74963	6.67272	6.33918	6.23615	6.06978	6.54931	6.14448	5.88595	5.76643	6.00003	5.71471
ADAM22	Q9P0K1	6.08884	7.05403	5.94506	6.31821	5.96761	6.17946	6.2418	5.73216	5.65545	6.95089	6.35852	6.02763	6.30777	6.49888	6.46156	6.21215
LZTR1	Q8N653	5.82404	6.51273	6.64412	6.54405	5.81332	6.81069	6.48505	6.33108	6.43485	6.78031	6.97884	6.67679	5.21373	5.95591	5.73888	5.25561
SENP5	Q96HI0	6.50813	6.26829	6.27154	6.89083	6.43481	5.92028	6.448	5.87463	5.80543	6.39632	6.13688	6.0022	6.29757	6.40835	6.14065	6.19608
MRPL50	Q8N5N7	5.29701	5.19443	4.48845	5.78175	5.93035	4.27473	5.2667	6.11507	4.26157	5.50655	4.65705	4.78333	4.60875	12.4156	9.24572	12.173
USP22	Q9UPT9	6.3965	6.38268	6.97107	6.47016	6.19437	6.32176	6.09978	6.49579	6.48018	7.21488	7.27321	7.06005	4.73484	5.45949	5.07606	5.3692
TCP11L1	Q9NUJ3	6.33841	5.32059	6.345	5.26123	6.01192	5.9586	6.10782	7.03175	5.685	6.28549	6.30883	7.35425	6.07029	6.81087	6.46736	6.64259
SAMD4A	Q9UPU9	6.67481	6.58384	7.27001	6.20578	6.05274	6.65701	6.30042	5.99325	6.18121	6.29593	6.56895	6.69396	5.61948	5.7425	5.68947	5.47065
PTGR2	Q8N8N7	6.22214	5.9381	6.81807	5.49647	6.61043	6.88338	6.25317	6.84006	6.29866	6.52505	6.60332	6.40472	5.76818	5.93577	5.50357	5.89891

DNAL1	Q4LDG9	6.33661	5.72619	7.71569	5.4198	6.41663	6.12178	6.00904	6.91029	5.88773	6.65245	6.76293	7.14707	5.68774	5.89839	5.56918	5.73847
MPZL1	O95297	6.20739	6.43177	5.88714	6.67371	6.03541	6.04049	5.83534	5.88467	6.48882	5.95988	5.95727	5.94216	6.71935	6.3472	6.723	6.86639
ALKBH5	Q6P6C2-1	5.92303	6.38344	6.13398	5.94513	5.85335	6.43782	6.16549	5.58311	5.9877	6.40685	6.50119	6.19408	6.4909	6.76523	6.87491	6.35381
KAT14	Q9H8E8	6.26711	6.30218	5.95562	6.65067	6.04785	5.8455	5.94724	5.93207	6.46169	5.79903	6.43365	5.77303	6.89183	6.62014	6.6631	6.40931
HAUS8	Q9BT25	6.47265	6.72475	7.0368	6.76685	6.02416	6.77415	6.20523	6.58163	6.33813	6.65398	6.84083	6.9774	5.00562	5.35595	4.86064	5.38121
TPRN	Q4KMQ1	6.56757	6.75647	6.98201	7.03431	5.90601	5.98661	6.20104	6.36772	6.31905	6.22963	6.84299	6.72952	5.53433	5.42874	5.47002	5.64399
ATG5	Q9H1Y0	6.5769	5.62069	6.74602	5.319	6.4455	6.16059	5.94027	6.91351	6.04184	6.77652	6.74124	7.08368	6.02598	6.12415	5.79349	5.69062
MAPK7	Q13164	6.27692	6.05538	7.08349	6.36544	6.65739	7.03826	6.37641	6.0703	6.09093	6.08385	6.6072	6.54764	5.9865	5.99979	5.43603	5.32448
GGA2	Q9UJY4	6.87276	6.57119	7.17139	6.57282	6.76916	6.67928	6.35984	6.58978	6.23965	6.62223	6.92123	6.32645	5.34504	5.04259	5.14906	4.76754
GGA3	Q9NZ52	6.58743	6.06136	7.01299	5.94114	6.41834	7.18258	6.15974	6.5876	6.31436	6.72771	6.597	6.59693	5.6001	5.85129	4.89139	5.47004
GGA1	Q9UJY5	6.77795	6.67986	6.49639	6.67294	6.83172	7.29311	5.9436	6.51385	5.98348	6.35815	7.08913	6.11128	5.32351	5.62911	5.2479	5.04802
C2orf69	Q8N8R5	6.52596	6.11521	6.78046	6.83086	6.34709	6.59629	6.24874	6.4633	6.38995	6.72562	6.34928	6.51325	5.68032	5.42063	5.40607	5.60697
MTERF4	Q7Z6M4	6.5762	5.99728	6.5077	6.45098	6.55951	6.38665	6.97538	5.95895	6.39815	6.09412	6.05964	5.51115	6.12047	6.14894	6.04343	6.21146
INPP5K	Q9BT40	6.18638	6.33423	5.95967	6.78702	6.25802	6.06499	6.26662	6.06079	6.15617	6.17873	6.05031	5.8098	6.31963	6.05961	6.93227	6.57577
COX5B	P10606	6.25945	4.99256	5.27074	4.78553	5.34834	4.20218	4.77271	6.18738	4.19357	5.27531	4.25802	5.49637	5.64034	11.6503	9.59802	12.0691
HMG20B	Q9P0W2	6.63812	6.59417	6.23986	7.08093	6.21519	6.42709	6.41972	6.20327	5.90775	6.73658	6.52624	6.61417	5.7844	6.08003	5.20331	5.52916
MTFMT	Q96DP5	6.76396	6.64545	5.78632	6.54841	6.19518	5.52965	6.12162	5.89792	6.64662	6.0609	5.93881	5.93684	6.50113	6.75998	6.14481	6.52241
PAWR	Q96IZ0	6.31257	6.04418	6.84813	5.65017	6.18656	6.40459	5.993	6.33536	6.23773	6.68623	6.66991	6.56485	6.19109	5.88128	5.83558	6.15877
ZBTB11	O95625	6.09153	6.33088	6.40246	7.25444	6.02049	5.8656	6.54527	5.75623	6.27807	6.47795	6.46533	6.50331	5.86909	5.65271	6.13621	6.35043
RBSN	Q9H1K0	6.74784	6.58468	6.58708	6.08774	6.41346	6.39788	6.1918	5.95858	6.28317	6.19516	6.19254	6.7973	6.87104	5.81106	5.83528	6.04537
MSRA	Q9UJ68	6.30697	6.09698	6.29055	5.96143	6.65329	6.53115	5.99008	6.7443	6.21968	6.02916	6.59031	6.31164	6.31983	6.23946	5.9031	5.81205
SLC25A32	Q9H2D1	6.0783	6.1631	5.74956	6.81948	6.4393	6.17121	6.86543	6.00736	6.25879	6.05045	6.05429	6.051	6.39278	6.03688	6.25348	6.60857
CAV1	Q03135	6.3733	6.92291	5.91474	6.86155	5.9901	6.27299	6.236	6.25008	6.0088	5.95839	5.70105	6.09879	6.24359	6.62074	6.27435	6.27263
TRAFD1	O14545	6.22767	6.46292	6.48805	6.41828	6.48252	6.57374	6.5297	6.24114	7.24528	5.69772	6.41041	7.1806	5.00713	5.7081	5.22182	6.10492
MYCBP	Q99417	6.11857	4.75403	5.86479	5.13369	4.92364	3.77311	4.6303	5.93437	4.0757	5.67745	4.81849	5.21984	4.75476	12.8275	9.69684	11.797
OFD1	O75665	6.69413	6.83767	6.90555	6.43244	6.57232	6.58226	6.40375	7.03435	6.47864	6.67585	6.56528	6.65868	5.29773	5.17791	4.7289	4.95453
BAG5	Q9UL15-2	6.15204	6.08877	6.54515	6.03682	6.37936	6.1673	6.18666	6.03954	6.50512	6.27054	6.66448	6.05719	6.41202	5.96064	6.51433	6.02006
TRMT11	Q7Z4G4	6.26665	6.34921	6.46917	6.32704	6.14467	6.40736	6.20008	6.49594	6.47704	6.32226	6.29493	6.58832	6.12689	5.69919	5.89907	5.93221
ZXDC	Q2QGD7	5.71714	6.13033	6.57531	6.27377	5.90955	5.76328	6.38175	6.28521	6.10413	6.35936	6.34878	6.14249	6.42307	6.5078	6.48854	6.58948
TIMM21	Q9BVV7	6.36526	6.51274	5.86557	6.70314	5.92874	5.80952	6.20135	6.19342	6.14209	6.18681	5.94346	6.20072	6.69591	6.18813	6.63345	6.4097
SKP1	P63208	6.45551	5.25947	5.57652	4.86749	5.31566	5.10301	5.20028	6.33209	5.54328	6.30259	5.46492	6.57105	6.76813	8.81878	7.73135	8.68986
NCALD	P61601	6.0815	5.08149	5.94212	4.91816	5.9343	5.24837	5.64393	6.36507	5.55285	6.61748	5.9659	6.98161	6.17371	7.98656	7.71077	7.79618
HMGCR	P04035-3	5.08973	5.11989	5.11965	5.48366	7.10401	6.93701	7.68378	7.1615	4.38486	4.77727	4.81038	4.60464	8.2064	7.6229	7.71135	8.18296
HMGA1	P17096-2	5.66025	5.77583	5.25867	6.27383	6.90427	6.64315	6.24464	6.29885	6.07331	5.54198	6.56832	5.54743	6.88898	7.64845	6.72794	5.94409
MYBL2	P10244	6.11944	6.12485	6.33679	6.39158	6.34793	5.89919	6.02546	5.88427	6.1548	6.52622	6.55684	6.12201	6.8267	6.15333	6.21137	6.31921
FUT8	Q9BYC5	6.54503	6.40178	5.66646	6.91958	6.57787	6.17063	6.53914	6.13719	5.97297	6.10448	5.77042	5.81026	6.0888	6.27535	6.43805	6.582
RBFOX2	O43251-8	6.49287	6.35251	6.13336	6.33163	6.20396	5.91096	5.9953	6.28328	6.27881	5.90103	6.0012	6.68952	6.49653	6.07058	6.20223	6.65622
NUBP1	P53384	6.01176	6.29446	7.14912	5.37787	6.09082	6.40389	6.38027	6.54357	6.36039	6.40021	6.62871	6.79898	6.30446	5.80965	5.9573	5.48854
ELP4	Q96EB1-2	6.35613	6.05387	6.56577	6.45788	6.54049	6.99169	6.12989	6.89975	6.52995	6.58772	6.75056	6.5074	6.50151	5.97397	5.102	5.05143
ABHD12	Q8N2K0-2	6.57684	6.3795	6.03004	6.77939	6.30315	6.12653	6.0784	5.9386	5.90612	6.38576	6.07377	5.83267	6.52228	6.09931	6.57211	6.39553
DZIP3	Q86Y13	5.95858	5.7158	6.81053	5.7278	6.35513	6.93868	6.93441	6.27637	6.11649	6.32807	6.92763	6.79641	5.79787	5.80691	5.60714	5.80219
MTX3	Q5HYI7	6.19843	5.59943	5.89089	5.58398	6.21565	5.22561	6.18351	6.38592	5.72043	6.40114	6.15327	6.49816	6.77567	6.91298	7.16185	6.99307
MSI2	A0A6Q8PF05	6.24377	6.07544	5.88888	5.89782	6.42673	6.59258	6.22037	6.69804	6.32459	6.54374	6.45506	6.6836	6.23788	6.15819	5.79147	5.76184
SNX17	Q15036	6.72494	6.80523	6.88157	6.04204	6.47252	6.9864	6.2731	6.15005	7.07824	6.92367	7.1011	6.71198	4.82646	5.19654	5.01973	4.80642
SH2B1	Q9NRF2	6.41488	6.47193	6.77452	6.19514	6.27771	6.2184	6.0682	6.43127	6.56191	6.67825	6.27126	6.60452	6.11286	6.05568	5.25683	5.60666
EXO1	Q9UQ84	6.3887	6.30038	6.54526	6.07657	5.85014	5.90173	5.89061	5.60374	7.25183	6.07717	6.66117	6.45672	6.47792	6.11862	6.1604	6.23905
WDR20	Q8TBZ3-7	6.45419	6.70488	6.33279	6.37353	6.30601	6.49683	6.36706	6.82363	6.36614	6.43371	6.21734	6.93518	5.77133	5.79992	5.94743	5.67003
TICAM2	Q86XR7-2	6.53887	6.61645	6.07961	6.82506	6.59438	5.84888	6.12648	6.1762	6.35974	5.59402	6.06279	5.82108	6.32844	6.03668	6.77645	6.11489
POU2F1	P14859-6	5.55238	5.5944	6.12294	5.63047	5.56252	6.36471	5.73561	5.65905	6.0946	6.11429	7.05673	6.89162	7.33043	6.83292	6.89875	6.55859
PIH1D1	Q9NWS0	5.63816	4.95883	5.77838	4.72743	5.70215	4.92693	5.64337	7.56493	5.41416	6.83724	6.19998	7.59342	5.87831	8.23097	7.22399	7.68176
RMC1	Q96DM3	6.20194	6.69724	6.52182	6.62094	6.1941	6.45998	6.18924	6.38775	6.46233	6.59162	6.05892	6.35451	5.77453	5.64594	6.20071	5.63844
CCDC137	Q6PK04	6.28303	6.492	5.86707	6.93672	6.01953	5.81602	6.22336	6.11441	6.22682	6.05496	6.43427	6.14653	6.29856	6.31786	6.54072	6.22815
XRCC3	O43542	5.82963	5.8099	7.06102	5.92166	6.19572	6.22162	6.12497	5.9188	6.10322	6.31257	7.00525	6.92813	6.03453	6.52546	5.8887	6.11882
HIF1AN	Q9NWT6	6.27917	6.32078	6.52588	6.11435	6.0661	6.42003	6.38538	6.72624	6.26327	6.57331	6.6988	6.91134	5.61431	5.97008	5.5653	5.56566
MRPL58	Q14197	6.32543	6.2186	5.44487	6.83627	6.60698	6.37846	6.14432	5.87096	6.31173	6.37629	5.94717	6.07893	6.60022	6.15977	6.34042	6.35958
SRP19	P09132	5.89632	6.07748	6.42298	6.12651	5.97838	5.95228	6.15807	5.96933	6.10626	5.8821	6.57905	6.35474	6.48537	6.76207	6.55609	6.69296
METTL15	A6NJ78	6.21426	6.45393	6.02913	6.80506	6.20913	6.0164	6.38779	6.24695	6.14384	6.30474	5.86717	6.31078	6.67242	5.98717	5.99046	6.36077
SLC2A1	P11166	6.05742	6.94313	6.09578	6.57186	6.40098	6.01497	6.65461	5.94952	5.90745	5.9336	6.01102	5.97803	6.6785	6.26733	5.92005	6.61576
HPDL	Q96IR7	6.42128	5.20552	5.67427	5.7829	6.29707	5.30581	6.20639	6.23868	5.84822	6.17012	5.92315	6.95277	6.86733	7.1665	6.72392	7.21607
FAM160A2	Q8N612-2	6.59769	6.50576	6.92709	6.04958	6.28756	6.4118	5.59676	6.23399	6.52854	6.64029	7.03888	6.69471	5.49862	5.9936	5.52529	5.46983
FAF1	Q9UNN5	5.84966	6.11906	6.31001	5.75681	6.32937	6.5579	6.16451	6.36471	6.48285	6.14241	6.41702	6.41164	6.25591	6.17274	6.33697	6.32842
LONP2	Q86WA8	6.11234	6.39367	5.69089	6.37721	6.47493	6.23027	6.57642	6.22603	6.13671	6.00185	5.91245	5.98777	6.71334	6.10972	6.52689	6.52951
INPP5F	Q9Y2H2	6.12108	6.23953	6.78716	6.07526	6.38524	6.52954	6.03204	6.16443	6.22964	6.55639	6.30673	6.32354	6.03846	5.8437	6.03944	6.32479
FBRS	J3KNZ9	6.10729	6.89673	6.26011	6.73816	6.32585	6.75376	6.67498	6.27651	6.26951	6.46655	5.93171	6.33874	5.59429	6.22599	5.66877	5.47104
WAC	Q9BTA9	6.35818	6.37309	6.32392	6.73949	6.15001	6.38073	6.02445	5.96012	6.21699	6.11198	6.53262	5.96013	6.62429	5.99207	6.01565	6.23627
SLC25A10	Q9UBX3-2	6.03199	6.40155	6.1938	6.60249	5.8873	6.09341	6.38971	5.63188	5.88121	6.09021	6.01321	6.20021	6.84672	6.41038	6.56719	6.75875
NDUFS7	O75251	6.4323	6.58966	5.45187	6.18266	6.4028	6.24589	6.3932	6.09148	6.19959	5.63576	6.16224	5.48177	7.11993	6.1735	7.04142	6.39594
CLPTM1L	Q96KA5	6.60277	6.67818	6.43178	6.75765	6.13057	6.23144	6.08204	5.67827	5.96653	6.46009	6.03169	5.96701	6.3351	6.33058	6.256	6.0603
LRRC57	Q8N9N7	5.80007	5.82376	6.26226	5.68454	5.93624	6.056	6.196	6.50001	6.5332	6.31115	6.44951	6.86273	6.51785	6.39004	6.35562	6.32104
ZCCHC9	Q8N567	5.90779	6.33594	5.9769	6.4946	6.22575	6.10902	6.16675	5.78683	6.13448	6.45857	6.37232	6.31817	6.15649	6.82031	6.62234	6.11374
TRIM41	Q8WV44	6.23881	6.35766	6.57537	6.80023	6.41319	6.39375	6.0665	5.95656	6.56795	6.63996	6.7154	6.15416	5.79244	5.88284	5.71207	5.7331
CKMT1A	P12532	6.25153	6.70188	5.82839	6.36378	6.53261	6.07264	6.11292	5.92651	6.19926	6.27141	6.23153	5.95904	6.38165	6.12591	6.52787	6.51309
PPP6C	O00743-3	6.01319	5.74002	6.63741	5.74549	6.34972	6.761	6.34653	6.49259	6.48216	6.41267	6.37996	6.72746	5.99382	6.08976	5.98854	5.83968
MRPL21	Q7Z2W9	6.34395	6.35601	6.12443	6.58826	6.55757	6.00464	6.78266	6.218	6.26905	5.80835	5.8953	5.5594	6.38333	6.09229	6.61051	6.40626
PARP12	Q9H0J9	6.91714	6.45303	6.63732	6.84683	6.39193	6.1313	6.48561	6.18956	6.59487	6.45201	6.40993	6.34047	5.65231	5.44579	5.52994	5.52195
COX11	Q9Y6N1	6.61694	6.05864	6.19336	6.55095	6.47335	6.22197	6.39231	5.42052	6.01515	6.19508	5.75982	6.00984	6.63613	6.49066	6.56328	6.40199
TERF1	P54274	6.21091	6.2186	6.12194	6.69429	5.96654	5.91031	6.35807	5.94203	6.25281	6.3647	6.08512	6.10124	6.53451	6.34113	6.46224	6.43557
SAP130	Q9H0E3-3	6.33225	6.49624	6.35157	6.53558	6.21697	6.25856	6.09769	6.04522	6.21787	6.30276	6.25562	5.9634	6.36388	6.04225	6.2628	6.25733
SERINC1	Q9NRX5	6.33369	6.6342	6.2475	6.28329	5.83032	5.97343	6.08172	6.11764	6.33345	6.72693	6.15074	6.22726	6.12604	6.46405	6.40732	6.06239
SLC44A1	Q8WWI5	6.49623	6.6188	6.08757	6.46598	6.04354	6.26049	6.14393	6.00552	6.41484	6.58266	6.32044	5.87309	6.96548	6.30441	6.16875	6.24827
AP4B1	Q9Y6B7	6.61787	6.35267	7.30854	6.45903	6.12018	6.51841	6.1638	6.32002	6.64475	6.69647	6.68079	6.7135	5.44705	5.26014	5.35548	5.34131
C1orf52	Q8N6N3	6.11344	5.73029	6.55793	6.24337	6.35342	5.66873	5.64223	6.21103	6.05775	6.36312	7.3138	6.39694	6.63761	5.93994	6.44399	6.32639
CEP55	Q53EZ4	6.71596	6.83572	7.08386	7.01181	6.39704	7.14571	6.3495	6.9511	6.65875	7.18321	7.22179	7.15904	4.54621	4.51492	3.88179	4.3436
SEMA3C	Q99985	6.96988	6.47196	4.33687	6.80294	5.78395	5.66948	7.4331	5.95955	6.56977	5.95727	5.27767	6.67069	7.30033	5.75558	6.44132	6.59964
GCSH	P23434	7.2083	6.56597	5.53712	6.8352	6.18909	6.52983	5.74429	6.21937	5.98461	6.33982	5.81619	6.09942	6.66345	6.61976	6.06493	6.48265
RRM2B	Q7LG56-6	6.52916	6.17486	6.48316	5.89813	6.48434	6.63797	5.93913	6.64252	6.2798	6.24909	6.33455	6.767	6.02318	6.06774	5.79389	5.69549
C2CD2	Q9Y426	6.07823	6.29257	6.1283	6.19529	6.1784	6.34548	6.80735	5.86617	5.86192	6.02652	6.21489	5.83891	6.59586	6.30554	6.77452	6.49006
BCR	P11274	6.37241	6.17496	6.6668	6.00234	6.43241	6.52178	6.18192	6.17459	6.49731	6.42582	6.59993	6.48439	6.08001	6.02566	5.65037	5.70929
C8orf33	Q9H7E9-2	6.1451	6.26351	6.67947	6.40691	6.30422	6.68759	5.93244	6.49874	6.54904	6.14938	7.0494	6.63612	5.6939	5.6427	5.60803	5.75347
PAXX	Q9BUH6	6.23742	5.96482	6.59311	5.44776	6.53073	6.5242	5.83024	6.71959	6.16382	6.85937	6.29011	6.56192	6.0593	6.11943	6.09032	6.00785
NEK6	Q9HC98-2	6.37147	5.96045	7.1287	6.50131	6.19913	7.09394	6.34594	6.26862	6.41362	6.83036	6.83487	6.64971	5.36108	5.61359	5.00093	5.42627
GTF2H3	Q13889	6.22808	6.35235	6.11003	6.20087	6.13883	6.19491	6.21564	6.15206	6.21311	6.09742	6.24736	6.16964	6.40203	6.10262	6.65545	6.51959
PHF10	Q8WUB8	6.39957	6.50316	6.02224	6.51316	5.85697	5.92511	6.33841	6.03379	6.30647	6.06428	5.9499	5.86373	6.66603	6.38374	6.50022	6.67323
C9orf40	Q8IXQ3	6.01654	5.46646	6.32017	4.94296	5.48254	5.14077	5.49442	6.84069	4.95912	6.87846	6.09582	7.35427	6.35186	7.89159	7.62026	7.14405
CNOT2	Q9NZN8	6.59925	6.14892	6.71956	6.04854	6.47555	6.16952	5.84385	6.72152	6.57922	6.58368	6.45366	6.94623	6.10758	5.67164	5.37398	5.55728
RC3H2	Q9HBD1	6.53313	6.88266	7.0526	6.26422	5.92949	6.29544	6.65213	6.15433	6.81401	6.28056	7.38203	6.32223	4.93374	5.47884	5.60711	5.41746
TARBP2	Q15633	6.58951	6.62664	6.89722	5.74884	6.60391	6.63599	6.36206	6.3653	6.75391	6.11673	6.60408	6.39228	6.06572	5.76328	5.17498	5.29956
GALNT4	Q8N4A0	6.65669	6.51754	6.5266	6.29848	6.53307	5.22679	6.45216	5.75054	6.73786	6.07752	6.04709	5.66975	6.3413	6.02456	6.72964	6.41042
NBEA	Q8NFP9	6.27624	6.46694	6.71251	6.04357	5.98731	6.72649	5.95622	5.93091	6.28688	6.26237	6.58528	6.50221	6.13988	6.2306	6.05748	5.83512
ALDH2	P05091	6.50142	6.1142	5.64998	6.28032	6.63742	5.83691	6.36285	6.17254	6.06113	5.91314	5.93874	5.93664	6.94001	6.41532	6.70539	6.53399
STK39	Q9UEW8	6.26476	6.43482	7.02119	6.13407	6.42215	6.50278	5.89106	6.49263	6.1953	6.62744	6.47717	6.37057	5.64178	5.56342	5.92567	6.03519
NAPRT	Q6XQN6	6.81646	6.01768	6.89487	5.13339	6.33836	6.68708	6.21791	6.81101	5.90704	6.1467	6.48014	6.23448	5.84003	6.82246	5.93922	5.71316
CDK4	P11802	6.45733	5.91598	7.02252	5.91424	6.60175	6.8321	6.37395	6.74366	6.45664	6.56806	6.78455	6.80353	5.49903	5.56606	5.19815	5.26244
ZNF593	O00488	7.24048	7.44668	7.17147	7.58367	6.6109	6.10578	6.34046	6.7046	6.96834	6.51019	6.23247	7.00751	4.05136	4.89828	4.6009	4.5269
SAP30	O75446	6.65432	6.86484	6.37946	7.09192	6.36823	6.29482	6.25766	6.09222	6.3361	6.20336	6.26004	6.15258	5.70844	5.62585	5.89106	5.8191

CYB5R4	Q7L1T6	6.60876	5.82589	7.14215	5.56979	6.01806	6.67615	6.40674	6.17598	6.27541	6.83691	6.56911	6.88542	5.63195	6.08501	5.5245	5.76818
RAB24	Q969Q5	6.59333	6.59959	6.22728	6.69012	6.04259	6.49361	6.88312	5.66841	6.35987	6.43544	6.28986	6.08908	5.75109	5.43779	6.31293	6.1259
PRXL2A	Q9BRX8	6.36174	6.24698	5.89876	6.47645	6.25761	6.29963	6.33412	5.93929	6.31062	5.91527	6.01504	5.52045	7.01171	6.44689	6.62729	6.33816
PAK5	Q9P286	6.28702	6.52366	6.79669	6.33391	6.28494	6.55569	6.10059	6.09231	6.39421	6.63174	6.34381	6.57231	5.85759	5.98776	5.54923	5.68854
RANBP17	Q9H2T7	5.47472	5.06289	6.24473	5.54719	5.83151	6.36811	6.08629	5.9675	6.17123	6.22043	6.17556	6.25124	6.77762	7.26782	7.28924	7.26392
ATF7	P17544-1	6.41632	6.48674	6.05877	6.11241	6.34294	5.9652	6.30196	5.97998	6.67254	6.36482	6.33902	5.95548	6.73071	5.94908	6.09529	6.22874
VANGL1	Q8TAA9	6.90677	7.16911	6.49329	7.2785	5.96915	6.00198	6.38311	5.75009	6.41131	6.61301	6.56663	6.66537	5.4951	5.41494	5.52922	5.35242
STK26	Q9P289	6.36411	6.04903	6.76806	5.89651	6.47263	6.13695	6.2208	6.32108	6.22623	6.31657	6.53122	6.43622	6.44946	5.93385	5.83773	6.03956
ANK3	Q12955	5.92647	6.27696	6.39522	5.89433	6.11653	6.37837	6.09047	6.18686	6.06303	6.1836	6.394	6.40034	6.63999	6.28112	6.76905	6.00366
ZNF592	Q92610	6.45617	6.32851	6.122	6.91005	5.9383	5.93926	6.42815	5.84441	6.11886	6.24507	6.28586	6.04763	6.51699	6.17849	6.47068	6.16957
NUS1	Q96E22	6.66492	6.62705	6.21959	6.68172	6.45043	6.30219	5.89559	5.90811	6.19207	6.51973	6.44488	6.1018	5.55074	5.96423	6.24956	6.2274
CAPN5	O15484	6.45498	6.4124	6.55264	6.02327	6.21722	5.78189	6.5186	6.21739	5.79589	6.59106	6.24434	6.17783	6.28721	6.36381	6.06095	6.30052
JADE3	Q92613	6.07099	6.6149	6.39132	6.85295	6.46778	6.36887	6.33398	5.72874	6.20982	5.88768	6.08968	6.10877	6.37842	5.93534	6.40294	6.15782
HNRNPC	B4DY08	6.08493	5.76176	6.00748	6.55282	6.36088	5.46808	7.22456	6.03602	6.48835	4.93284	5.88425	5.73825	6.42121	7.16198	6.81777	7.05882
ZNF48	Q96MX3	6.4767	6.44679	6.3275	6.73227	5.91966	6.09143	6.24242	5.70289	5.99076	6.15277	6.47495	6.29354	6.24052	6.28867	6.06572	6.55343
ETS1	P14921	5.66994	5.08662	6.68507	6.22962	6.01864	5.9659	5.70696	6.12045	6.17641	5.74637	6.05538	6.04527	6.90416	7.61394	7.47846	6.49683
BTRC	Q9Y297	6.77385	6.49347	7.2768	6.95991	6.39314	6.29437	7.01905	6.08688	6.58538	6.59684	6.09515	5.96174	5.17682	5.24942	5.68465	5.35251
NOL7	Q9UMY1	6.52382	6.69565	5.95479	7.304	5.85154	5.67756	6.30906	5.98543	6.87352	5.98658	5.97585	6.33453	6.26966	5.87889	5.72922	6.64989
DNAJC14	Q6Y2X3	5.94984	7.06483	5.58927	7.02342	6.17086	5.68151	6.01478	5.79023	6.17682	6.39431	6.35271	6.14907	6.5142	6.16371	6.56943	6.395
ARGLU1	Q9NWB6	6.13586	6.01684	6.03301	6.29645	6.06138	5.54281	6.21637	5.71579	6.08554	6.04964	6.20473	6.05446	6.62697	6.75706	7.31469	6.8884
MACF1	H3BQK9	5.78537	6.46847	5.93375	6.52228	5.9788	6.72123	5.95968	6.43697	5.64397	6.24822	6.21099	6.45006	6.20345	6.80812	6.41002	6.21862
SETD5	Q9C0A6	6.86594	6.69151	6.09741	7.59007	5.9433	5.77056	6.49706	5.81206	6.43005	6.65235	6.52056	6.32131	5.39561	5.46902	5.89858	6.04461
ANKIB1	Q9P2G1	6.78553	6.32775	7.52282	6.27436	5.71936	6.34251	5.63188	5.63819	6.69905	6.90986	6.73011	6.92175	5.35434	5.96379	5.84468	5.33403
MOB4	Q9Y3A3	5.93703	6.46563	6.30928	5.97765	6.17721	6.49778	5.90672	6.2617	6.14606	6.42802	6.23764	6.1799	6.33262	6.44887	6.34171	6.35218
PALD1	Q9ULE6	6.3759	6.51538	6.35571	6.38401	6.62999	6.13842	6.30747	6.36914	6.5924	6.30799	6.08848	6.40422	6.35194	5.90487	5.59936	5.67475
WNK3	Q9BYP7	6.06469	6.1982	6.75097	5.94217	6.19201	6.84291	6.02211	6.5562	6.4618	6.55885	6.73055	6.60325	5.64326	6.13733	5.77239	5.52332
BOLA2	Q9H3K6	6.09945	5.70614	6.69117	5.26663	6.42682	6.86375	5.97208	6.71684	6.12526	6.69292	6.28195	6.70607	5.95842	6.50685	5.94138	6.04427
SERPINB9	P50453	6.21872	6.07472	6.73119	5.94129	5.95538	6.71398	6.34828	6.30138	6.17824	6.69506	6.80798	6.3912	5.87435	6.1263	5.72227	5.91965
ITPRIP	Q8IWB1	6.60761	6.73798	6.09021	7.10192	6.46137	6.05769	6.2095	6.09497	6.12406	6.18799	6.04796	6.0572	6.12144	5.95428	6.09038	6.05543
CD46	P15529-2	6.30231	6.63058	5.85128	6.47843	6.018	5.96403	6.43705	5.85246	6.26906	5.99405	5.84412	6.15219	6.41473	6.58962	6.55035	6.65173
FAM98C	Q17RN3	5.95925	5.72839	6.91385	6.38755	6.08899	6.57613	6.21246	6.60992	6.82434	6.80387	7.08221	6.3802	5.46648	5.70987	5.29443	5.96208
TLK1	Q9UKI8	5.55913	6.55962	6.28085	6.30485	5.68786	6.46927	5.74994	6.43877	5.80182	6.3918	5.8574	6.11484	6.48434	6.69814	6.72087	6.88051
ERH	P84090	5.70669	4.80231	4.31955	4.81364	4.45134	3.77871	4.55823	4.96359	3.5941	4.74932	3.99464	4.63671	4.73752	16.087	9.39277	15.4139
UNC119B	A6NIH7	5.87303	5.77343	6.53709	5.76562	6.47175	6.09791	5.94095	6.6739	6.68027	6.48747	6.74822	6.87578	6.00682	6.16291	5.88549	6.01936
ASMTL	O95671	6.21604	5.76877	6.71001	5.71317	6.30334	6.5751	6.18714	6.4244	6.565	6.31474	6.66347	6.75387	6.17746	5.99578	5.81286	5.81885
RAD51C	O43502	5.55526	5.92099	6.39824	5.66204	5.89766	6.03626	5.83948	6.41921	6.44357	6.37029	6.84925	7.02634	6.00782	6.3076	6.51264	6.75336
TICRR	Q7Z2Z1	6.68057	6.99469	6.11895	7.25946	6.26956	5.92662	6.96345	6.45544	6.70774	6.25417	6.54749	6.31657	5.05243	5.34679	5.57497	5.53109
CHPF2	Q9P2E5	6.5063	6.89585	5.87834	6.63229	6.29747	5.84764	6.04253	6.0195	6.5532	6.72755	6.08618	5.87238	6.44295	6.39121	6.10151	5.7051
NKAP	Q8N5F7	6.55073	5.84955	6.05595	6.33959	6.69055	5.97347	6.26272	6.03982	5.94433	6.06727	5.75909	5.68807	6.28335	6.87176	6.75578	6.86798
HPS6	Q86YV9	6.75439	6.53359	6.99714	6.42176	6.16448	6.63978	6.29701	6.705	6.41325	6.63364	6.6457	6.72961	5.33049	5.46237	4.92036	5.35143
PKLR	P30613	6.57394	6.62694	6.62838	5.42124	6.46238	7.04591	6.25299	7.21384	5.86754	6.83644	5.72015	6.54451	5.60694	6.39727	5.32751	5.47403
FAM120C	Q9NX05	6.38632	6.57805	6.68438	6.18379	6.19456	6.88316	5.83112	6.11308	6.51164	6.49632	6.79329	6.13737	5.84458	5.85041	5.85532	5.65658
TBP	P20226	5.80901	5.95458	6.03726	6.2909	6.07355	6.18065	5.83493	5.95626	6.26665	6.31175	6.25608	6.16819	6.81458	6.17318	6.99622	6.8762
IDI1	Q13907-2	6.07328	5.75891	6.60985	5.57879	6.30842	6.56908	5.86448	6.5173	6.00266	6.86765	6.18138	6.95418	6.05521	6.16266	6.10042	6.39574
PGAP1	Q75T13	5.96878	6.47654	6.0697	6.24457	5.84764	6.37112	6.60438	6.21683	5.97838	6.18795	5.89901	5.92352	6.51365	6.40794	6.89632	6.39365
AFF1	P51825-2	7.87665	7.91383	7.3625	7.59533	6.07954	6.5296	6.71419	6.06994	7.1622	7.15807	7.50603	6.76501	3.17113	4.45993	3.58404	4.05203
H2AB2	P0C5Z0	5.90149	6.0811	5.60975	6.3913	6.1636	5.32795	6.72401	5.93572	6.13726	6.09983	6.22724	5.51259	6.63408	7.01772	7.0506	7.18574
TLE1	Q04724	5.85369	5.89929	6.40356	6.32713	5.84015	6.06438	6.00149	6.25043	5.87854	6.28666	6.06578	5.79322	7.17371	6.91339	6.88594	6.36265
PKD2	Q13563	6.53043	6.25056	6.37336	6.51479	6.25529	6.03457	6.39611	5.8781	6.0034	6.04078	5.94981	5.99342	6.33273	6.35377	6.88471	6.20816
GATM	P50440-2	6.31027	6.69197	6.04923	6.58016	6.47091	6.30418	6.09795	6.02902	6.47082	5.6634	6.09846	6.29122	6.67883	5.83209	6.2955	6.13597
PXK	Q7Z7A4	6.16877	6.15176	6.46182	5.73426	6.23793	6.72386	6.36512	6.45634	6.40068	6.5275	5.95082	6.08546	6.46795	6.52309	5.89543	5.84921
NBR1	Q14596	6.19637	5.85577	6.2152	6.37391	6.39747	6.06658	5.9448	6.64178	5.63491	6.48238	6.57876	6.49516	5.97501	6.32516	6.409	6.40776
MYL6B	P14649	6.55637	5.84696	6.72857	5.36365	6.55676	6.18602	6.34781	7.01088	6.26788	6.66556	6.28214	6.75797	5.56028	5.92794	5.94679	5.8944
DPY19L1	A0A1B0GW05	6.20523	6.57684	6.19607	6.56839	5.90267	6.03337	6.05495	5.83157	6.07169	6.45889	5.95313	6.24854	6.48889	6.29293	6.54626	6.57057
PCNX3	Q9H6A9	6.55011	6.46884	6.37617	7.00603	5.99069	5.95206	6.39395	6.23273	6.59298	6.32096	6.076	6.41973	6.03781	5.69188	6.00273	5.88732
TMX4	Q9H1E5	6.1729	6.36063	6.03749	6.38116	6.03046	6.01354	6.63342	6.08979	6.17393	6.25458	6.10834	6.04014	6.76062	5.82031	6.49944	6.62325
CCDC174	Q6PII3	5.63148	6.17736	5.61663	5.69905	6.17971	6.02768	5.82591	5.67536	6.03224	6.15778	6.02339	6.14525	6.9982	7.41942	7.14674	7.24379
CDS2	O95674	6.03422	6.59375	5.85239	6.33816	6.06412	5.92297	6.07218	6.07293	5.90824	6.27758	5.80746	5.98891	6.40406	7.03956	6.87646	6.74701
TFCP2	Q12800	6.31301	6.55095	6.10509	5.79198	5.8826	6.65252	6.16246	6.2574	6.30207	6.29993	6.30298	5.77343	6.44591	5.5804	6.29619	6.2831
ANKRD13A	Q8IZ07	6.43969	6.53052	6.41263	6.30254	6.72421	6.561	6.52805	6.28607	6.31976	6.27096	6.21024	5.87182	5.81903	6.03565	5.76666	5.92117
NFIB	O00712-5	5.74786	6.16328	5.89655	6.3437	6.16068	6.15759	5.89413	5.91855	5.96893	6.25466	6.28363	5.89333	7.06297	6.82334	6.70884	6.72193
ZKSCAN8	Q15776	5.94108	6.53071	6.07162	6.61074	6.01974	6.6059	6.78214	5.93661	5.80403	6.53494	6.48182	6.22616	6.18259	5.81514	6.32324	6.13352
ZNF268	Q14587	5.90998	5.51674	6.21443	6.73681	6.17566	6.21062	6.27732	5.77166	6.30915	6.693	6.43496	6.37998	6.49261	6.19963	6.17121	6.50622
PICK1	Q9NRD5	6.90223	6.3658	6.74686	6.33709	6.16817	7.08928	6.19391	6.71124	6.54827	6.65129	6.70249	6.28136	5.38373	5.6977	4.87801	5.34255
MPST	P25325-2	6.23613	5.55154	6.26233	5.44059	6.21547	5.87918	6.1956	6.7222	5.96566	7.14252	6.13343	6.80182	6.28688	6.4544	6.42099	6.29125
NRF1	Q16656-4	5.76988	5.89185	5.59233	6.08883	6.27363	5.90966	6.71226	5.86761	6.38524	5.98041	5.6229	6.1919	7.00716	6.61559	6.72422	7.36653
TIAM1	Q13009	6.06917	6.36575	6.42729	6.13639	6.08637	6.4907	6.43102	5.9339	6.14619	6.28229	6.29942	5.90735	6.5812	6.0878	6.27456	6.48062
MTHFSD	Q2M296	6.21747	6.21649	6.46877	6.0923	6.31197	6.55014	6.43119	6.33364	6.38599	6.74234	6.00165	6.04145	6.28935	6.26769	5.85275	5.79681
DYRK1A	Q13627	6.22189	6.75937	6.2888	5.96678	6.59075	6.26823	6.24805	6.39328	6.05412	6.2896	5.78687	6.12382	6.38167	6.35703	6.18551	6.08423
RAB2A	P61019	6.29703	6.5456	5.81099	6.32855	6.42574	6.38481	6.34088	5.93682	6.08719	6.05734	5.97766	6.01357	6.68093	6.1918	6.73337	6.18772
TSEN2	Q8NCE0	5.58388	6.13411	6.19714	5.8745	5.56565	6.20082	5.96961	5.76325	6.22423	6.19378	5.62804	6.43481	7.0503	6.79323	7.02006	7.36659
TUBA4A	P68366	6.46004	5.85309	7.11717	5.95882	6.52572	6.84784	6.00265	6.62008	6.62071	6.36572	6.9753	6.82445	5.41199	5.77171	5.06842	5.57631
SUV39H1	O43463-2	6.093	6.21233	5.65868	6.77272	5.64347	5.49099	6.31788	5.73494	6.19483	6.17254	5.68079	6.43925	6.86455	6.4533	7.26527	7.00548
MED18	Q9BUE0	5.70019	5.3292	5.8125	5.35086	5.9496	6.5872	5.89332	5.98549	5.50899	6.29661	6.12879	6.02978	6.81817	7.89552	7.13435	7.57944
RAD54L2	Q9Y4B4	5.93731	6.63563	5.75999	6.46809	6.45648	6.41044	5.88878	5.65319	5.861	6.74439	6.46944	6.23834	6.33092	6.13309	6.32376	6.68916
ALAD	P13716-2	6.12874	5.50545	6.85	5.46297	6.44689	6.2654	6.0441	6.67084	6.19986	6.90962	6.55799	6.86973	5.81833	6.02588	6.32934	5.91487
CHMP4A	Q9BY43-2	6.19547	6.1098	7.11286	6.1966	6.19138	7.11867	5.96065	6.53143	6.29089	6.2603	7.14401	6.96473	5.57235	5.96791	5.39681	4.98616
RFLNA	Q6ZTI6	5.9922	6.02001	6.08557	5.97059	6.17778	6.01225	5.86232	6.61394	6.70451	6.26184	6.22528	6.63427	6.74787	6.24794	6.20396	6.23966
TRAPPC4	Q9Y296	6.55958	5.58943	6.68742	5.5988	6.5818	6.25281	6.38386	6.61535	6.39512	6.52449	6.41108	6.59242	6.11997	6.15359	5.72132	5.81295
VDR	P11473-2	6.81797	6.76031	7.56364	6.74562	5.71891	6.60991	5.95053	6.09235	6.81634	7.04434	7.51878	6.92454	4.78838	5.44101	4.56202	4.64536
RSRC1	Q96IZ7	6.07886	6.45555	5.79524	6.47218	6.15423	6.09257	5.97822	5.75252	6.14599	5.98462	6.14594	5.91957	6.73566	6.50559	6.99007	6.79318
OSGEPL1	Q9H4B0	6.79932	6.87756	6.10968	7.00048	6.19391	5.94757	5.96796	5.91826	6.24581	5.89719	6.04899	6.09157	6.41828	6.06229	6.18348	6.23765
CD59	P13987-2	5.74012	5.32903	5.53032	5.1241	5.23683	5.44938	5.79465	6.54413	5.81182	6.19433	5.69714	6.38102	6.97547	8.22669	8.04759	7.91739
HIVEP1	P15822	6.70654	6.65388	6.41025	6.40977	6.46182	6.2394	6.08588	6.26607	6.23849	6.30577	5.92021	6.123	6.39334	5.88652	6.17361	5.72545
NFAT5	O94916-5	6.36433	5.79682	6.53267	6.20638	6.21722	5.94566	6.19265	6.07936	6.63541	6.46233	6.21434	6.43856	6.7022	5.75501	5.95241	6.50465
HS1BP3	Q53T59	6.25322	5.84387	6.41857	5.35989	6.2112	6.34612	5.67581	6.72098	6.50714	6.8512	6.76383	6.33747	6.34964	6.56884	5.82941	5.9628
MMAB	Q96EY8	6.72696	6.19028	6.14074	6.55197	6.37881	6.33923	6.14672	5.98093	6.12456	5.86502	6.01852	5.86156	6.79424	6.17448	6.40416	6.30182
AAK1	A0A096LP25	6.07409	6.11585	6.54215	5.66935	6.28063	5.63994	6.0259	5.86495	6.23893	6.41081	6.28635	6.06613	6.86039	6.50329	6.20667	6.21468
THBS3	P49746	6.45377	6.63402	6.07612	6.46965	6.30518	6.19226	6.64585	6.23295	6.52942	6.65657	6.78797	6.01473	5.65365	5.89086	5.68325	5.77373
RTEL1	Q9NZ71-2	5.56391	5.90186	6.84069	6.49202	5.84447	6.18683	6.06008	6.12454	6.28246	6.39618	6.69345	6.79508	5.9856	6.37131	6.49515	5.96636
HSD17B8	Q92506	6.62482	6.3295	6.33171	6.51795	6.15149	5.89119	6.23067	5.79633	6.16085	6.50755	6.12456	5.99954	6.46636	6.03265	6.56731	6.26753
GRAMD1B	Q3KR37-4	6.27198	6.1491	6.06111	6.65165	6.47873	6.38528	6.71889	6.22046	5.85223	6.07814	5.87291	5.77222	6.52986	6.39799	6.58225	6.57719
UXT	Q9UBK9-2	5.94399	4.7304	6.10823	5.37315	5.39655	5.93645	5.62335	6.75393	5.35146	6.41451	5.89577	7.16444	5.68789	8.25342	7.29723	8.06924
ADSS1	Q8N142	6.32083	6.44423	6.38621	5.86048	6.46	6.69128	6.36441	6.67797	6.24975	6.53424	6.26265	5.843	5.89558	6.23936	5.94319	5.82683
ABCB8	Q9NUT2	6.23322	6.67754	6.10046	7.13772	6.29662	5.76101	6.78196	5.69067	6.64084	6.00166	5.99574	5.8045	6.27872	6.15556	6.69368	5.75011
PPP1CA	P62136	6.24166	5.9855	6.04747	6.14446	6.34808	5.9989	6.22853	6.02392	6.05852	6.10813	6.16822	6.68961	6.71179	6.58045	6.58365	6.08111
HOMER3	Q9NSC5	6.02747	6.49487	6.78249	6.10708	6.19865	6.29592	5.58141	6.46637	6.47153	6.67562	6.93978	6.93179	6.00584	5.9875	5.49282	5.54087
TTC7B	Q86TV6-2	6.47047	6.30659	6.24287	6.66623	6.31319	6.11925	6.24559	6.01479	6.05526	6.48207	5.98082	6.24544	6.32483	6.05061	6.4228	6.05919
TTC14	Q96N46	6.09694	7.03909	6.50339	6.99356	5.55905	6.60666	5.87093	6.12258	5.83734	6.16247	6.62735	6.09185	6.34439	5.94352	6.27831	5.92259
OTUD6B	Q8N6M0	6.39885	6.49364	6.77654	6.40397	6.48821	6.30665	6.19595	6.90707	6.19321	6.35826	6.49733	6.10713	5.53285	5.93428	5.71809	5.68794
NAA16	Q6N069	6.2221	6.56861	6.35104	6.47166	5.97526	6.70854	5.92288	6.55537	6.17099	7.02028	6.39161	6.61239	5.82745	5.96656	5.5635	5.67179
MOCS1	Q9NZB8	6.3693	6.25633	6.1646	6.24516	6.40936	5.85701	6.35776	5.95262	6.34065	6.5805	6.53055	6.30048	6.12689	6.15611	6.37891	5.97378
REV1	Q9UBZ9	6.83114	6.36674	6.35225	6.52267	6.27991	6.14389	6.18164	5.95927	6.22612	5.90477	5.90253	6.22155	6.36794	5.7874	6.64746	6.30472
TPST2	O60704	6.4887	5.98569	6.10877	6.37463	6.17732	6.26885	6.44669	5.73771	6.55516	6.32282	6.0863	6.1671	6.52979	6.11109	5.95428	6.68511
STON1	Q9Y6Q2-3	5.789	5.86644	5.76136	5.38579	6.10406	6.2	5.86233	6.15622	6.20788	6.33115	6.02418	6.0949	6.74274	7.29264	7.34169	6.83962

ACACB	O00763	6.54944	6.2232	6.88403	6.15051	6.69548	6.60492	6.34701	6.48426	6.67983	6.8302	6.60928	6.25828	5.39631	5.34817	5.47722	5.46186
DNAJB4	Q9UDY4	5.93497	6.31826	6.54987	6.09414	6.2317	6.29822	5.88746	6.31543	5.93729	6.07299	6.12086	6.35756	6.49008	6.2989	6.58581	6.50645
CD9	P21926	6.54529	6.54067	6.28297	5.96793	6.31451	6.38443	6.27254	5.75708	6.36644	6.19118	5.37511	6.07562	6.76171	6.30911	6.51241	6.34301
TBC1D10A	Q9BXI6	6.20845	6.26562	5.92237	6.31231	6.01978	6.12842	6.24419	5.84092	6.44582	6.42806	6.20127	6.15058	6.26325	6.48894	6.36267	6.71733
SLC25A25	Q6KCM7-3	6.47245	6.20072	5.58948	6.35872	6.49409	6.10346	6.34559	6.14429	6.44615	5.99562	6.43993	5.83364	6.66109	6.03039	6.81651	6.06784
DOP1A	Q5JWR5	6.66502	6.04017	7.12185	5.80508	6.1586	6.65476	6.37144	5.72248	6.58098	6.98094	6.47095	6.80377	5.83741	5.69383	5.57672	5.51599
ZMYND11	Q15326	6.15616	6.2971	5.95625	7.14822	6.0027	6.26937	6.65439	5.94548	6.15598	5.91344	6.14788	5.98645	6.19559	6.157	6.27899	6.735
EPC1	Q9H2F5	6.1547	6.60117	6.20564	6.88982	6.16177	5.85956	6.15544	5.98793	6.14207	6.37289	6.57814	6.60543	5.95711	6.30457	6.04003	5.98373
PDCL3	Q9H2J4	6.76287	6.50475	7.08329	5.77356	6.96961	6.779	6.0998	6.90626	6.60752	6.31366	6.1473	6.28128	6.05593	5.16213	5.22089	5.33216
ZADH2	Q8N4Q0	6.24668	6.76073	5.67894	6.5346	6.02764	6.15054	6.36204	6.05088	5.96684	6.25399	5.8005	6.08069	6.5519	6.11723	6.91153	6.50527
EFR3B	Q9Y2G0	6.10634	6.58299	6.37393	6.99098	5.88991	6.11558	6.26907	5.84179	6.55957	6.99	6.50341	6.39839	5.85498	5.95163	5.60168	5.96976
CTBP1	Q13363	6.44334	5.72009	6.1651	5.43125	6.46031	5.95187	5.47272	6.13692	5.78129	6.16504	5.95362	6.83502	6.46527	6.81258	7.01301	7.19258
TGFB2	P61812-2	6.10108	6.35864	6.05388	6.02987	6.22506	6.28512	6.57142	6.33501	5.30597	5.56198	6.04047	5.61027	6.72179	7.14977	6.97153	6.67813
FAM126A	Q9BYI3	6.33516	6.89849	6.56773	6.80716	5.97419	5.89954	6.36728	5.96667	6.25779	6.09218	6.23022	6.5736	6.18252	5.75256	5.90342	6.19148
ABRAXAS1	Q6UWZ7	6.61033	6.17834	5.9942	6.61858	6.1168	5.79194	6.39267	6.2119	6.1388	5.7988	6.41212	6.07407	6.62051	6.21474	6.42498	6.40122
ZBTB21	Q9ULJ3	5.85025	5.96738	5.99625	6.37747	6.06324	6.08311	6.33229	5.82827	5.849	6.00564	5.89629	6.23094	7.11974	6.34703	6.84967	7.20343
TACO1	Q9BSH4	6.43581	6.57069	6.38154	7.09326	6.08053	6.36286	6.66581	6.01775	6.23426	5.67961	5.54568	5.52605	6.04929	6.41157	6.35843	6.58686
RRN3	Q9NYV6	6.69211	6.61557	7.42732	7.55034	5.8864	6.17984	6.21011	6.28666	6.47492	6.40798	6.87465	6.85339	5.11378	4.65258	5.33333	5.44104
EMC2	Q15006	6.1256	6.88752	6.14475	7.12791	6.18011	6.05679	6.24763	5.66095	5.97454	6.55615	6.39299	6.27345	5.86957	6.07776	6.28579	6.1385
MAU2	Q9Y6X3	6.51187	5.97942	5.74462	6.77207	6.20523	6.25874	6.16338	5.91572	5.8752	6.10591	5.84669	6.63943	6.45133	6.553	6.60009	6.37731
TRMT13	Q9NUP7	5.56941	5.76601	6.58413	5.60854	5.78379	6.23113	5.55177	5.93257	6.08715	6.13748	6.25799	6.12487	6.69057	6.82641	7.62485	7.22334
RNF149	Q8NC42	6.82923	6.82193	6.72442	7.70939	6.18425	6.21097	6.14773	6.30346	6.6477	6.93827	6.97862	7.02416	4.77851	4.93808	4.80412	4.95916
PSMF1	Q92530	5.72275	5.73966	5.92225	4.37877	6.41766	6.92158	6.04958	6.71408	5.87768	7.12287	6.57126	7.70096	5.30467	6.48277	6.35986	6.71361
NME3	Q13232	6.51823	6.66631	6.14153	5.96506	6.06885	6.17658	6.16576	5.86319	6.45517	5.96289	5.59255	5.87107	6.61898	6.65147	6.4626	6.45977
FANCB	Q8NB91	5.88521	6.15269	6.31956	6.37416	6.09867	6.17565	6.57075	5.85937	6.25889	6.19473	6.57849	6.28876	6.52929	6.10218	6.61441	6.65721
HPS5	Q9UPZ3	6.56095	6.37673	6.33958	6.4126	6.07564	6.06156	6.89631	6.65999	6.78882	6.31747	6.8101	6.027	4.98069	5.84251	6.03843	5.81163
B4GALNT1	Q00973	6.49799	6.4215	6.06328	6.57184	6.41898	5.74443	6.49412	5.95484	6.75319	6.7622	6.47608	6.37805	5.90891	5.74565	5.94292	5.86601
MRPL14	Q6P1L8	6.46188	6.50783	5.73701	6.54357	6.38543	5.91584	6.74369	5.71028	6.36107	6.17097	6.0977	6.28283	6.21835	5.8777	6.59318	6.39267
SLC39A14	Q15043	6.26566	6.58497	6.58659	6.1878	6.47339	6.6487	6.38669	6.24683	5.98823	6.19181	6.3608	6.3254	6.24509	5.76862	5.88925	5.85018
TOE1	Q96GM8	6.09165	6.0933	5.97727	6.2812	6.16275	5.92146	6.64699	5.76891	6.18001	6.50581	6.13347	6.16312	6.62743	6.34144	6.81522	6.30996
APOE	P02649	6.39343	6.65276	6.45324	5.98108	6.65839	6.19984	6.90306	6.77887	6.21668	6.28726	6.16639	6.53585	5.7938	5.67913	5.51642	5.7838
AJUBA	Q96IF1	6.24819	6.28705	7.29301	6.33647	6.23837	6.56179	6.48285	6.44096	6.26315	6.04497	6.62691	6.37256	5.86301	5.7437	5.63862	5.55837
CSNK1D	P48730-2	6.96186	7.05502	6.97047	7.07365	6.40518	6.70973	5.87713	6.58783	6.47212	6.58371	6.64826	6.49617	5.29295	5.41315	4.82879	4.62398
MCRS1	Q96EZ8-2	6.27008	6.15172	6.10426	6.83672	5.7662	6.12463	6.35325	6.02646	6.38681	6.52026	6.40715	6.37437	6.30751	6.16204	6.22118	5.98736
CHEK2	O96017	5.3206	5.7525	5.59933	5.48473	5.45729	6.48007	5.53692	6.16718	6.04072	6.27512	6.77542	6.44322	6.8101	7.22531	7.32863	7.30285
GSDME	O60443	6.58292	6.00779	7.77494	6.0458	6.10602	6.91721	6.37423	5.77359	6.57951	6.35229	6.02543	6.10767	5.52976	6.30083	5.83169	5.69033
RASSF1	Q9NS23-5	6.71245	6.71543	6.75771	6.61569	6.27691	6.28828	6.14248	6.28008	6.54578	6.17099	6.40681	6.53808	5.60083	5.42153	6.20899	5.31796
CCM2	Q9BSQ5-2	6.42513	6.39929	6.82809	6.28467	6.47941	6.5512	6.04599	6.35885	6.31269	6.7677	6.59352	6.63714	5.80764	5.55129	5.58703	5.34338
AARSD1	Q9BTE6-3	6.21473	6.16525	6.98129	6.31198	6.22302	6.72856	6.18538	6.59847	6.489	6.2831	6.5965	6.68904	5.79525	5.81066	5.43515	5.49262
ZWINT	O95229	6.69838	5.21096	6.20348	4.95784	6.97881	5.63297	5.56922	7.96602	5.88704	6.93261	6.64313	7.45947	5.58322	6.13734	5.71832	6.42118
MCUR1	Q96AQ8	6.64461	6.20868	5.75947	6.47402	6.62292	5.98299	5.99394	6.42745	5.99524	5.99889	6.182	5.67442	6.64459	6.32314	6.73386	6.33377
IL17RA	Q96F46	6.34016	6.73103	6.2928	6.58679	6.03893	5.92171	6.25421	5.98141	6.29776	6.45865	6.08486	6.21104	6.26733	6.17997	6.27771	6.07563
MTRR	Q9UBK8-1	6.52191	6.10707	7.14157	5.99494	6.40933	6.69013	6.31899	6.79831	6.62754	6.32274	6.75752	6.73885	5.5055	5.34162	5.33002	5.39395
RETREG3	Q86VR2	6.36123	6.73971	6.30198	6.50665	5.73688	6.78701	6.07794	5.71466	5.9318	6.03969	6.03004	5.61891	6.37297	6.60579	6.87975	6.29501
OTUD7B	Q6GQQ9	6.1248	6.23351	6.68043	6.14206	6.12562	6.07426	6.00025	6.40897	6.36633	6.41742	6.38868	6.45209	6.09102	6.30494	6.0593	6.13031
PEG10	Q86TG7	6.31289	6.44816	6.47676	6.21322	6.63451	6.49819	6.23433	6.40304	6.24083	6.32498	6.23985	6.50839	5.59199	5.88542	5.8485	6.13893
RBM3	P98179	7.34708	7.2697	6.42509	7.42004	7.00536	6.24381	6.13883	6.55187	6.52915	6.3303	6.57199	6.69368	5.42275	4.87164	4.22937	4.94936
MRPS6	P82932	5.84385	6.22761	6.02618	6.27253	6.43624	5.84287	6.05254	6.31818	6.09216	6.48687	6.53273	6.60248	6.31404	6.05272	6.40344	6.49558
RBBP9	O75884	5.94152	5.87865	6.74553	5.38289	6.08928	6.99805	6.25011	6.5561	6.14825	6.84998	6.26814	6.59821	5.82685	6.53228	5.87657	6.0576
CENPQ	Q7L2Z9	6.53789	6.60698	5.8116	6.83318	6.11315	6.26061	6.45715	5.92792	6.22948	6.05052	6.00611	6.14567	5.85185	6.26597	6.44417	6.45776
IMP4	Q96G21	5.94829	5.95465	6.10724	7.20685	6.21925	5.9176	6.28695	6.6077	6.20322	5.94882	6.23713	5.89414	6.48701	6.07844	6.38365	6.51905
BCLAF3	A2AJT9	6.29559	6.31428	5.8845	6.82656	6.06333	6.20228	6.27902	5.78512	5.89892	5.50818	5.90479	5.91416	6.80649	6.74239	7.07519	6.49921
LRRC42	Q9Y546	5.94706	6.04282	6.65194	5.87588	5.85637	6.19392	6.10838	6.35489	6.38879	6.45462	6.80456	6.37098	5.87692	6.24366	6.60356	6.22563
CHMP5	Q9NZZ3	6.41094	6.25319	7.09215	5.54105	6.50012	6.86545	6.32291	6.78225	6.12804	6.72399	6.34114	6.58348	5.59503	6.13796	5.45213	5.27017
PAAF1	Q9BRP4	6.02209	6.36324	7.1813	5.73023	6.19046	6.65483	6.17891	6.24313	5.9926	6.5591	6.62677	6.45653	6.34444	6.16058	5.86679	5.42898
HLA-C	A2BF26	6.48989	6.31699	6.12938	6.50668	6.4668	6.58743	6.34965	6.45704	6.40627	6.29923	6.21373	6.16231	5.80822	5.74207	5.89525	6.16906
PTBP2	Q9UKA9-2	6.10229	6.30051	6.32563	6.14997	6.34827	5.74168	6.18205	5.89249	6.20195	5.85789	5.89205	6.11002	6.7568	6.23597	6.91424	6.9882
LAMTOR2	Q9Y2Q5	5.87859	5.39191	5.56773	5.106	6.16248	6.04427	5.9678	6.41917	5.72419	6.35658	6.0539	6.16331	6.3095	7.47483	7.67301	7.70673
CENPV	Q7Z7K6	5.98068	6.38151	6.08136	7.01408	5.8987	6.07554	6.23045	5.81643	5.99546	6.15747	6.14941	6.34545	6.25162	6.18726	6.73877	6.6958
IFNAR1	P17181	7.51068	7.16696	6.63351	6.70275	6.50936	6.00596	6.27518	6.07803	7.04999	6.57728	6.72425	6.80343	4.50245	4.92778	5.28503	5.24735
SPNS1	Q9H2V7	6.07742	6.5357	5.92982	6.90538	6.29802	5.88992	6.28818	5.79761	5.96891	6.31413	6.30474	6.04266	6.45498	6.52899	6.21529	6.44825
JUN	P05412	5.60134	5.97032	5.58061	6.19107	6.54885	6.32788	6.6572	6.13349	5.47898	4.75801	5.40615	5.43169	7.85774	6.86485	7.86049	7.33132
RPUSD2	Q8IZ73	5.75726	5.32	6.31302	5.20433	5.76282	6.11884	5.57315	5.81369	6.03673	6.397	6.43437	6.10064	6.94469	7.48777	7.45518	7.28049
TRIM26	Q12899	7.24104	7.03425	6.77773	6.75506	6.44986	7.02314	5.82224	6.64837	7.17478	6.6367	7.25649	6.33256	4.64921	4.72204	4.66458	4.81195
FRMD5	Q7Z6J6	6.09566	6.08381	5.82484	6.57347	6.01799	5.81406	6.22255	5.84014	6.15976	6.28001	6.02309	5.89633	7.03209	6.51478	6.87511	6.74631
BRF1	Q92994	5.36278	5.99797	6.27217	5.93809	5.43324	6.14035	5.8135	5.81146	6.30391	6.48845	6.60618	6.43264	6.48274	7.0355	7.08036	6.80067
CCDC102A	Q96A19	6.43726	6.24267	6.20394	6.46153	5.64324	5.85238	5.92211	5.97953	5.99565	6.50117	6.20302	6.61551	6.89966	6.31734	6.46593	6.25906
ANKZF1	Q9H8Y5	5.95287	6.6424	6.33744	6.41423	6.27094	6.40341	6.18598	6.66105	6.4398	6.62851	6.57679	6.8715	5.69243	5.41933	5.88972	5.61361
NF2	P35240-2	6.06159	6.28479	5.94635	6.38101	6.20219	6.25031	6.31936	6.20197	6.31979	5.90801	6.14568	6.23982	6.35196	6.30404	6.39347	6.68966
PARVB	Q9HBI1-2	6.08149	6.25647	6.33277	6.1369	6.07866	6.69076	6.0519	6.70156	6.10327	6.5007	6.37706	6.58536	5.9304	6.54648	5.62927	5.99693
MRPL48	Q96GC5	6.06607	6.21168	5.51454	6.58124	6.4482	6.186	6.58672	6.30307	6.5101	6.21045	6.02022	6.112	6.1545	5.8605	6.90991	6.32482
SERPINE2	P07093-3	6.66387	6.72408	5.26248	7.21311	5.98816	6.04936	6.6158	6.20749	6.32005	6.69172	5.8928	6.24298	5.79883	5.98396	5.9978	6.34751
GNG12	Q9UBI6	5.84069	5.3573	6.1824	5.49091	5.62317	5.49779	5.96348	6.14939	6.11556	5.80151	5.92462	6.38494	6.3534	8.04117	7.67251	7.60115
ISOC1	Q96CN7	6.37435	6.52816	6.74282	5.98638	6.61084	6.51357	6.0752	6.62536	6.20121	6.19574	6.22013	6.49135	6.1452	6.1081	6.04232	6.16927
SLC35B2	Q8TB61	6.16612	6.12286	5.75956	6.15716	6.39505	6.10804	6.18381	6.1416	6.27394	6.35418	6.17283	6.10337	6.50027	6.46526	6.45677	6.63919
CTAG1A	P78358	5.65332	5.93699	6.31993	5.25245	6.28801	6.10184	5.9092	7.0777	5.83517	6.81462	6.3872	6.6786	5.80608	6.70222	6.84475	6.3919
RBMS1	P29558-2	6.15561	6.77356	6.74307	6.35814	6.33249	6.42705	5.9925	6.09856	6.32223	6.72244	6.62711	6.80242	6.2491	5.46547	5.38186	5.54837
MRPS11	P82912	6.40127	6.29046	6.13143	6.83244	6.07296	5.73251	7.06576	5.64364	6.33217	6.13058	6.15862	5.94398	6.46248	6.55986	6.23781	6.00402
TAPT1	Q6NXT6	6.20107	5.85647	6.17492	6.93108	5.98916	6.17287	6.71025	5.94536	6.16436	6.22534	6.0611	6.03075	6.50767	6.317	6.54186	6.17073
SURF2	Q15527	5.54597	6.13315	5.9505	5.60419	5.91009	6.15519	5.9018	5.79437	6.14337	5.86473	6.90682	6.07007	7.10953	7.43469	6.82335	6.65219
RGS10	O43665	6.1193	5.69323	7.0215	5.6115	6.5501	6.11436	5.78304	6.57805	6.24786	6.70884	6.8675	7.06654	5.80753	6.35426	5.76854	5.70785
PRKCI	P41743	5.7206	6.02769	6.62894	6.01511	5.99959	6.43824	6.03829	6.45386	6.68217	6.30415	6.38957	6.72969	5.96338	6.13234	6.1933	6.28307
TENT4B	Q8NDF8-5	6.41807	6.45548	6.52853	7.18811	6.2261	5.90746	6.26995	5.78816	6.32663	5.94716	6.22363	6.0668	6.25199	5.8882	6.10515	6.40857
PWWP3A	Q2TAK8-3	6.42402	6.67351	6.21855	6.82884	6.01995	6.67743	6.50475	6.46413	6.45255	6.29986	6.14067	6.39581	5.65274	5.65851	5.7276	5.86109
P3H2	Q8IVL5	7.64044	7.09579	6.91654	7.67664	6.5782	6.20467	6.43841	6.59041	6.91712	5.94539	6.67388	6.45858	4.94461	4.32141	4.94938	4.64854
COPS7B	Q9H9Q2	6.35475	5.31281	6.40142	5.9343	6.35281	6.6832	5.9001	6.11631	6.33814	5.94626	6.38021	6.7231	6.87056	6.22189	6.43226	6.03188
PRKACA	P17612	6.35726	6.24413	6.84513	6.20647	6.42929	6.66903	6.31091	6.37829	6.21813	6.12322	5.77447	6.30767	6.63879	5.88797	5.94932	5.65591
RANBP10	Q6VN20	6.34785	6.73589	6.4645	6.35441	6.28147	6.45601	6.14191	5.98371	6.28218	6.28643	6.54237	5.92773	6.2017	6.22901	5.9931	5.77172
ENTPD6	O75354	6.86013	6.44982	6.29325	6.6344	6.1792	5.95583	6.55029	5.96532	6.62024	6.56207	6.89838	6.59662	5.68774	5.50227	5.73779	5.50665
NDUFA3	O95167	6.50535	6.5457	5.82784	6.2125	5.97837	5.51069	6.28712	6.12989	5.78323	5.58932	6.26921	5.2359	7.08658	7.07464	6.82605	7.13762
MSL2	Q9HCI7	6.56855	6.57968	6.11046	7.145	6.09873	6.07564	6.3126	5.91196	6.05253	6.22899	6.43619	6.05334	6.17327	6.06989	6.33715	5.84603
ZDHHC13	Q8IUH4	6.23281	6.5488	6.04811	6.89074	6.62352	6.20323	5.98212	5.73785	6.39222	6.27109	6.10641	5.69607	6.58642	5.85297	6.30998	6.51767
MCPH1	Q8NEM0	6.07561	5.93809	5.82137	6.03488	6.37542	5.94371	6.33936	5.94091	6.1745	6.1325	6.13717	5.88139	6.48985	6.59984	7.03498	7.08039
SH3GLB2	Q9NR46	6.5765	5.91733	6.97401	5.34875	6.53853	6.63991	6.05648	6.65707	6.49265	7.05308	6.80905	7.11264	5.98369	5.21562	5.40005	5.22465
TAF9	Q16594	6.48641	5.92599	6.54258	5.98273	6.47967	6.27768	6.28314	6.60701	6.29977	6.5455	6.81039	6.60767	6.10655	5.60797	5.70298	5.73396
CLSTN1	O94985	7.20378	7.47023	6.87567	7.27805	6.50905	6.43912	6.48617	5.95302	6.99973	7.18091	6.72126	6.5572	4.4876	4.60587	4.67416	4.55819
COMMD7	Q86VX2	6.09995	5.80565	6.21511	5.51704	6.09144	6.15872	5.81627	6.50027	5.96351	7.02416	6.30472	6.66867	6.5386	6.34102	6.53379	6.42108
PLA2G4C	Q9UP65-3	6.65734	6.55621	6.38048	6.94696	6.25246	6.38793	6.05393	5.78107	6.13792	6.52406	6.417	6.15073	5.58061	6.42702	5.5634	6.18289
CSTF2	P33240	6.33487	4.93882	4.88065	5.37693	6.06794	4.8739	5.23124	7.04155	4.58647	6.82818	5.51315	7.55103	6.66625	8.38888	7.25796	8.46217
SEC62	Q99442	6.37911	6.54413	6.1787	6.04206	6.32595	6.18669	6.11109	5.81226	6.43156	6.09135	5.78041	5.88801	6.48857	7.01672	6.35659	6.36679
ACP6	Q9NPH0	6.23981	6.00618	6.08902	6.7599	6.61963	5.73197	6.32202	5.53512	6.35272	6.69341	6.23636	6.57099	6.65819	5.67189	6.22397	6.28881
CD47	Q08722	6.37387	6.54104	5.95871	6.34095	6.31279	6.21673	6.33288	5.86295	6.38009	6.17569	5.72081	5.92302	6.7232	6.47317	6.28783	6.37627
SMURF2	Q9HAU4	6.27078	6.04044	6.51136	6.36552	6.05709	6.00027	6.55642	6.14269	6.49783	6.48228	6.44825	6.3737	6.27292	6.01256	6.06023	5.90767
MAP2K4	P45985-2	6.12268	6.03984	7.04522	5.60428	6.32256	6.4885	5.92808	6.68886	6.1762	6.33173	6.52673	6.10454	6.10493	6.28522	5.9739	6.25673
GMEB1	Q9Y692	6.36221	6.33189	5.98722	6.39201	6.29332	5.92124	5.9188	6.08589	6.24496	5.94016	6.19076	6.03633	6.9706	6.36182	6.17844	6.78433

ABCC5	O15440	6.29281	6.49112	6.39255	6.54147	6.39274	6.20206	6.25711	5.92072	6.12343	6.08096	5.84864	6.26725	6.08494	6.19183	6.30135	6.61102
IDH3G	P51553	6.38842	6.33718	6.28628	6.57359	6.08265	6.01582	6.1358	6.03113	6.26322	6.17582	6.26275	5.88038	6.46399	5.99353	6.87934	6.23011
RHOG	P84095	6.21218	6.00984	5.8821	6.07377	6.40338	6.20123	6.35593	5.89416	6.24629	5.81673	6.396	6.51324	6.2855	6.64702	6.42066	6.64197
EXOG	Q9Y2C4	6.25876	6.58621	6.02542	7.02486	6.04075	6.05723	6.1695	5.92841	6.06501	5.89419	6.30871	5.71444	6.59254	6.66419	6.31807	6.35171
PSMG3	Q9BT73	6.00545	5.87465	6.45957	5.48018	6.3864	6.87256	5.90668	6.9458	6.25732	6.70688	5.9875	6.31274	5.59042	6.50107	6.17287	6.53992
RNF123	Q5XPI4-2	6.00411	6.70117	6.64404	5.77028	6.07713	6.34579	6.39018	6.82714	6.28401	6.33422	6.23313	6.42589	6.01131	5.92875	6.04203	5.98082
TEFM	Q96QE5	6.52026	6.3574	6.18587	6.46933	6.38191	5.82367	6.72321	5.91515	6.26057	5.72695	5.98571	5.46732	6.77777	6.40441	6.53457	6.46592
ZMYM1	Q5SVZ6	6.0759	6.5564	6.47819	6.82034	6.20462	5.98244	6.41167	5.48236	6.20035	6.86369	6.4124	6.66214	5.5961	5.79706	6.31676	6.1396
LARP1B	A0A3B3ISF0	5.7438	6.36849	6.53062	6.84415	6.13912	5.90664	6.20922	6.39294	6.70071	6.40695	6.90028	6.5374	5.86122	6.24211	5.69479	5.52156
COASY	Q13057-2	5.90671	6.64692	6.54649	6.42787	6.56002	6.46329	5.91354	7.41217	5.9976	6.20853	6.53019	6.0316	5.85425	5.87096	6.04844	5.58142
UQCRQ	O14949	6.25938	6.36819	5.79962	6.53369	6.94431	6.21482	6.28456	6.6725	5.47798	5.75576	5.70755	5.70605	6.52732	6.78002	6.49256	6.4757
PHF20	Q9BVI0	6.24147	6.40129	6.00726	6.95877	5.95591	6.17456	6.29266	5.87709	6.47847	6.29037	6.1962	6.3534	5.6438	6.349	6.34462	6.43513
ANKRD28	O15084-1	6.50234	6.39945	6.93341	6.23555	6.46539	6.11397	6.06129	6.0276	6.48231	6.45156	7.00693	6.78755	5.91177	5.67273	5.46378	5.48438
MT1X	P80297	6.51711	5.76416	7.15433	5.4148	6.62106	6.78335	5.95996	6.26752	6.56429	6.87571	7.12937	6.72164	6.20966	5.513	5.23579	5.26822
CSRP2	Q16527	6.28509	6.0142	6.38506	6.34655	6.4038	6.38014	6.16976	6.38182	6.20035	6.49339	6.69146	6.46301	5.96049	6.0858	5.78029	5.9588
RAB22A	Q9UL26	6.33136	6.55252	6.28258	6.53856	6.08105	6.20804	6.17553	6.3279	6.14419	6.52334	6.02558	6.12624	6.15595	6.11225	6.33782	6.07708
SMAD4	Q13485	6.51809	6.21257	6.623	6.03017	6.0711	6.43715	6.16323	6.61687	6.14022	6.33664	6.83299	6.51586	5.8796	5.98827	5.83539	5.79886
CD55	P08174-7	6.71027	6.3365	6.13614	6.54857	5.88629	6.05165	6.12723	5.91431	6.31319	6.2664	5.96917	5.50022	6.93604	6.34915	6.29194	6.66293
ZNF142	P52746	6.64934	6.48769	6.14032	6.7295	6.08347	5.67131	6.56587	6.29573	6.0498	6.13571	6.11742	6.26081	6.27681	5.82309	6.425	6.28812
TMED4	Q7Z7H5	6.41743	6.17094	5.7189	6.40667	6.3977	6.512	6.15474	6.28444	5.84784	6.12973	6.07401	5.96835	6.67236	6.40469	6.57586	6.26434
FAM136A	Q96C01	6.09822	4.68369	5.0829	4.77504	5.68028	4.08426	4.84138	6.17253	3.95281	5.73488	4.76077	5.65849	6.11424	11.0973	10.026	11.2372
PSMG2	Q969U7	6.1426	6.05708	6.67916	5.77919	6.3064	6.51144	6.03542	6.80047	6.20483	6.56417	6.4724	6.21243	6.17473	5.99656	6.4073	6.5558
GLIPR1	P48060	6.45155	7.15522	6.17667	7.30119	6.01667	6.49557	6.33132	6.20721	6.08193	5.42478	6.36964	6.30845	5.93127	5.71332	6.14728	5.88791
CDKN2A	Q8N726	6.03113	6.48691	5.73706	6.19968	6.21067	6.47396	6.45585	6.09636	6.23315	6.01251	5.80183	5.58747	6.62837	6.75213	6.51025	6.78268
SNX30	Q5VWJ9	5.89752	6.38367	6.37126	6.09773	6.10087	6.34017	6.11722	6.54176	6.00679	6.45398	6.52459	6.3957	6.49063	5.98337	6.40174	5.89302
MRPL27	Q9P0M9	6.17756	6.76906	5.51633	6.71383	6.36001	6.11845	6.47581	6.4198	5.97556	6.18564	5.99321	5.99323	6.09494	6.35719	6.38124	6.46813
ARIH2	O95376	6.15685	5.78794	6.7482	6.04096	6.27281	6.36783	6.20954	6.38313	6.53418	6.20735	6.60889	6.70021	5.91885	5.96783	6.20973	5.8857
SNRPF	P62306	5.89039	5.3086	5.86639	6.18054	6.20441	6.02103	6.14198	5.32649	6.42787	6.62917	6.67027	6.65066	7.07462	5.99716	6.57865	7.03178
SUV39H2	Q9H5I1	6.43132	6.52916	5.71853	6.88879	5.76239	5.75152	6.32815	5.70321	6.03632	6.57279	6.31995	6.32266	6.67916	6.1978	6.6411	6.11714
PPT1	P50897	6.77856	6.02626	6.23518	6.16464	6.45704	5.86689	6.71382	7.01837	6.13486	6.01189	6.06428	5.89056	5.85688	5.5719	6.247	5.96187
IRS1	P35568	5.65847	5.9625	6.43622	6.09728	6.28066	6.32876	6.16377	6.57173	6.11368	6.6727	6.4573	6.50289	6.46211	6.32436	6.06281	5.90476
TAF1B	Q53T94	6.54525	6.50484	6.3461	6.94078	5.85888	6.14132	6.17393	6.58805	6.62713	6.13989	6.54609	6.89437	5.73753	5.73046	5.48473	5.74064
RELL1	Q8IUW5	5.75531	6.60815	5.91687	6.5044	6.28316	6.70899	6.7366	6.55503	5.93696	5.99243	6.75406	6.28091	6.58518	6.11879	5.80825	5.45492
YIPF4	Q9BSR8	6.45891	6.13305	5.96855	6.81809	6.12468	6.06999	6.50417	6.12028	6.05158	6.4376	6.209	6.01207	6.22099	6.10402	6.47383	6.29318
MUL1	Q969V5	6.17716	6.50424	6.59743	6.49637	6.6513	5.40643	7.3535	5.88343	6.28761	6.47189	6.65966	5.59117	5.96396	5.51159	6.68785	5.75643
BANP	Q8N9N5	6.37353	6.76783	5.83494	6.78712	6.40602	6.34075	6.40974	6.39345	5.98593	5.99413	5.90645	5.64614	6.51813	6.05928	6.34984	6.22672
RMND5A	Q9H871	6.32245	5.52903	6.44786	5.50067	6.30013	5.93734	5.98	6.27705	5.63907	6.45854	6.30138	7.21986	6.65218	6.33215	6.50957	6.59271
UFC1	Q9Y3C8	6.1396	5.70581	6.47657	5.83818	6.35017	5.78541	5.67346	6.2745	5.78676	6.87404	5.98807	6.57195	6.58732	6.25494	7.03067	6.66255
DGAT1	O75907	6.2448	6.5253	5.86294	6.44315	6.01645	6.2384	6.18162	6.12596	6.18408	6.2521	5.356	5.73297	6.53091	6.71716	6.72101	6.86715
GFER	P55789	6.66029	5.85182	6.39487	6.23038	6.33477	5.68287	5.91916	6.14917	6.04298	6.84438	6.68109	6.30051	6.99635	5.6467	6.05722	6.20746
BACH1	O14867	6.60717	6.34444	6.5947	6.28068	6.26391	6.21515	5.99469	5.59243	6.08133	6.5156	6.26956	6.80086	6.22657	5.85538	5.97325	6.05274
GAS2L3	Q86XJ1	6.37562	6.26287	6.54452	6.53095	6.05541	6.30133	6.19487	5.80942	6.58331	6.51228	6.69307	7.01236	5.0332	6.14893	5.91346	6.0284
ARF6	P62330	6.08818	6.2279	6.3989	6.19415	6.21225	6.38481	6.34617	5.99474	6.39017	6.1414	6.69137	5.80993	6.32538	6.54584	6.06578	6.18303
PGRMC1	O00264	6.56915	5.74703	5.73082	5.64569	5.71282	4.88647	5.54443	5.99448	5.2188	6.38841	5.97806	6.44606	6.95683	7.67007	7.47457	8.03632
EMC8	O43402	6.44882	6.295	5.50722	6.22951	6.23637	6.17726	6.11776	6.66343	5.96348	6.22791	6.07017	6.49835	6.32763	6.50321	5.96364	6.77026
RAB9A	P51151	6.29425	6.0252	5.71126	6.4128	6.29094	6.2976	6.23467	6.51843	6.18868	5.95713	6.36462	6.65741	6.35892	6.26027	6.17408	6.25373
PLEK2	Q9NYT0	6.51602	6.33205	6.8569	6.20557	6.39882	6.32259	6.11317	6.62698	6.42333	6.82708	6.73066	6.79121	5.81279	5.60651	5.25152	5.18478
POLQ	O75417	6.51103	6.87405	6.60012	7.78415	6.51249	6.12458	6.39403	5.64298	6.36403	6.87688	7.08849	6.99875	4.78608	4.90709	5.08353	5.4517
CEP162	Q5TB80	6.33089	5.99675	6.65785	6.15634	6.252	6.41918	6.46145	6.29576	6.58246	6.86037	6.60636	6.11462	6.16572	5.71584	5.86698	5.51744
ZMIZ1	Q9ULJ6	5.25608	5.4705	6.03971	5.49839	5.4776	5.81066	5.84384	6.19803	5.80488	6.09937	6.66582	6.41957	7.2202	7.28994	7.83127	7.07412
EYA3	Q99504	5.86159	5.94184	5.84048	5.51156	6.51367	5.9321	6.19245	6.32256	5.93907	5.92478	6.58592	6.52956	7.11265	6.47508	6.82838	6.48831
MRPS33	Q9Y291	6.09261	6.14909	5.59947	6.83156	5.76736	5.81737	6.36974	5.87823	6.16403	5.98992	6.02094	6.02684	6.84636	6.76881	6.94443	6.73324
SCRN2	Q96FV2	6.25217	5.86102	6.50099	4.98227	6.75484	6.14718	5.78212	7.09788	6.36013	6.89101	6.59873	6.8462	6.39284	5.76494	5.71461	6.05307
MMADHC	Q9H3L0	5.59936	5.71506	5.93913	5.8275	6.75811	7.17817	7.44362	7.50449	6.46875	6.22322	6.6303	6.67614	5.286	5.14043	5.56817	6.04156
TBC1D20	Q96BZ9-3	6.38096	6.47501	6.0212	6.61558	6.3149	5.8373	6.37233	6.01601	6.02853	6.80859	6.22876	6.15618	6.22312	6.3613	6.09904	6.06119
PPIC	P45877	6.63805	6.04155	6.455	6.82343	6.68655	5.9613	6.38951	5.8069	6.56925	6.41976	6.39161	6.25711	6.54961	5.47307	5.80987	5.72742
PATZ1	Q9HBE1	6.06108	5.92397	5.78554	6.58967	6.15157	6.24241	6.57285	5.90741	6.28599	6.43663	6.47671	6.55171	6.09778	6.08105	6.54492	6.29071
KCTD15	Q96SI1	5.86998	5.58406	6.43461	5.06947	6.0337	6.10154	5.49226	6.44227	6.61794	6.96567	6.96824	6.91516	6.54314	6.12025	6.39395	6.44775
ZNF646	O15015	6.28526	6.10124	5.74733	7.06546	5.9808	5.90292	6.35044	5.92203	5.77185	6.26942	6.34512	6.37115	6.64374	6.41782	6.55482	6.2706
FBXW8	Q8N3Y1	6.25572	6.18684	6.70882	6.29553	6.04951	6.85498	6.92942	6.32086	6.69025	6.95179	6.29701	6.58235	5.63248	5.42398	5.61238	5.20807
SNX19	Q92543	6.49648	6.84167	6.32209	6.50188	6.12726	6.43933	6.5532	5.9464	6.40273	6.28	6.2861	6.13229	6.09568	5.70205	5.77348	6.09939
TRAPPC6B	Q86SZ2	6.05588	5.36065	6.41458	5.08911	6.81677	6.51823	5.76962	6.35432	5.88358	6.37527	6.45304	6.71297	6.5773	6.49422	6.67978	6.44469
SNIP1	Q8TAD8	5.61086	6.176	5.53609	6.65464	5.88698	5.72839	6.06899	6.02334	6.37523	6.08523	6.09314	6.03298	6.3119	6.72196	7.11746	7.57682
SLIT3	O75094-4	5.89031	6.21876	6.30542	6.20354	6.22237	6.63568	6.01144	6.07936	6.56612	6.52819	6.24172	6.14753	6.19243	6.09576	6.65021	6.01116
RHOXF2	Q9BQY4	5.43061	4.67997	5.48024	4.73375	4.73865	4.64891	4.96008	6.26194	4.59188	5.91663	5.6762	7.19664	5.23277	10.8817	8.33889	11.2311
PRDM15	P57071	6.15876	6.35312	6.103	6.89714	5.84112	6.13372	6.29861	5.77545	5.78973	6.11462	6.22158	6.3392	6.63907	6.20172	6.30437	6.82879
C1orf174	Q8IYL3	6.17721	6.80222	5.97734	6.8007	5.71838	5.92336	6.12602	6.13356	6.11435	6.7791	6.41149	6.31803	6.00353	6.49787	5.92187	6.29496
HDAC5	Q9UQL6-3	3.58863	4.24916	12.4537	10.3939	14.9459	8.69288	3.63284	3.54675	3.78782	9.50167	4.27645	4.16883	4.54812	4.37363	4.10423	3.73557
MRPL41	Q8IXM3	6.2412	6.23959	5.35944	6.82138	6.59042	5.9549	6.88575	6.27796	6.28301	5.97278	5.91098	5.92443	6.80021	6.0395	6.42933	6.26914
MPHOSPH6	Q99547	6.2058	6.403	6.137	6.47679	6.62872	6.05775	6.38693	6.37032	6.04811	6.28403	6.09309	6.10522	6.69076	5.87118	6.10716	6.13415
STX2	P32856	6.45798	6.3635	6.50758	6.42836	5.88096	6.10932	6.24691	6.0273	6.11644	6.49292	6.37569	6.17309	6.11628	6.44394	5.92261	6.33711
CCNT2	O60583	6.06845	6.84479	5.79697	6.33112	6.18017	6.63536	6.242	6.07356	5.86009	5.96953	5.59572	5.76894	6.72555	7.11903	6.43412	6.3546
SH3BGRL	O75368	5.83688	5.35064	6.51565	4.38436	6.75406	6.40473	5.8746	6.84891	5.84091	6.70447	6.11717	6.989	6.07207	7.24543	6.42084	6.64027
DYNLRB1	Q9NP97	5.58156	4.46821	6.16451	4.33333	5.76938	5.44077	5.4529	7.47085	5.13932	6.28172	5.3894	6.5383	4.97265	9.69828	8.01469	9.28413
GOSR1	O95249	6.17537	6.39944	5.63891	6.49667	6.52394	6.00039	6.04586	6.13777	6.243	6.29263	5.99443	6.29043	6.43036	6.49941	6.34661	6.48477
TMCC3	Q9ULS5	6.52525	6.45561	5.67644	6.22392	5.97796	5.73836	6.37674	5.7941	6.16068	6.18379	6.34684	6.19824	6.74308	6.0173	6.91353	6.66818
PPIL3	Q9H2H8	5.84375	5.56973	5.79663	4.88867	6.61272	6.05312	5.98886	6.89365	6.33071	6.63213	6.61768	6.63235	6.28733	6.79222	6.63846	6.82199
MAOA	P21397	6.41746	6.23377	5.63821	7.17277	6.44852	5.99512	6.68564	6.11319	5.58677	6.46483	5.68015	5.93654	6.22333	6.31017	6.46463	6.62888
UCK2	Q9BZX2	6.33309	5.9697	6.98587	6.38375	6.35317	6.21563	6.45973	6.59345	6.88604	6.42813	6.74442	6.53463	5.59298	5.70985	5.26713	5.54243
CEP43	O95684	6.48794	5.88489	6.67872	5.95548	6.48139	6.58539	6.05236	6.66958	6.38193	6.33786	6.92248	6.67533	6.24326	5.56047	5.28288	5.80004
NPHP3	Q7Z494	6.12222	6.3781	6.53501	6.11939	6.3153	6.71965	6.7542	6.83594	6.50407	6.29719	6.60324	6.59729	5.49125	5.46111	5.3803	5.88575
PPP1R2	P41236	6.38091	6.05411	6.17111	5.97991	6.67526	6.46978	6.08651	7.0641	6.8342	5.91841	5.7532	6.59611	5.78931	6.40816	5.38517	6.43374
EEPD1	Q7L9B9	7.05623	6.29132	6.37599	6.55086	6.20294	5.92821	6.08307	5.92563	6.4212	6.53519	5.6664	6.87749	5.84444	5.75863	5.66055	5.92185
BLOC1S3	Q6QNY0	6.52044	5.8889	6.78956	5.79658	6.4402	6.88453	5.91602	6.46228	6.47078	6.32607	6.39623	6.33855	6.25954	6.10387	5.74193	5.66452
PPM1L	Q5SGD2	6.15599	6.31012	6.04167	6.85302	5.91293	5.81851	6.50465	5.8689	6.31658	6.3895	6.18197	6.45035	6.30057	6.13668	6.20007	6.55847
MMGT1	Q8N4V1-2	6.24662	6.26843	5.84847	6.21672	6.24083	6.14813	5.828	6.2044	5.9112	6.51405	5.7048	6.29334	6.37853	6.61565	6.72701	6.85382
DCAF7	P61962	5.31049	5.9785	6.03271	5.72129	6.54503	6.49863	6.51895	7.39321	6.71905	7.25349	6.53458	6.7435	5.467	5.48464	5.75808	6.04086
NDUFB8	O95169	6.54636	5.85787	5.39563	5.59835	6.33416	5.52254	5.9189	6.01819	5.33244	5.26951	5.6164	5.37838	6.74897	8.38196	7.39768	8.68267
MVB12A	Q96EY5	5.90918	6.27191	6.53423	6.01839	6.12275	6.53299	6.7112	6.32912	6.40652	6.811	7.05811	6.81665	6.06879	5.51612	5.52337	5.36966
ABHD6	Q9BV23	6.28663	6.4706	5.91492	6.56616	5.99303	5.78563	6.51737	6.13635	6.27258	6.13461	6.07457	6.05136	6.56503	6.41385	6.32697	6.49035
NTMT1	Q9BV86	6.28783	5.84281	6.56022	5.60325	6.80965	6.73675	6.22491	6.99454	6.12574	6.11464	7.25397	6.82034	5.77762	5.4657	5.73834	5.64368
SUCO	Q9UBS9	6.07414	6.20334	5.75296	6.3707	5.94705	6.27376	6.22035	6.11638	5.80768	6.31634	5.85443	5.96236	6.86468	6.8858	6.86424	6.48579
CYLD	Q9NQC7	6.58755	5.87021	6.71272	6.31591	6.48628	6.52741	6.53993	6.63509	6.14594	6.76644	6.43695	6.35486	5.29235	5.97321	5.74059	5.61455
DCTD	P32321-2	6.01287	5.84975	7.10166	5.70488	6.66633	6.87994	6.0248	6.65626	6.40741	6.7749	6.75599	7.03023	5.72237	5.50168	5.56324	5.34769
PAN3	Q58A45	6.25972	6.15952	7.11778	6.02804	6.36484	5.99589	6.43356	6.16985	6.51389	6.33177	6.35053	6.35766	6.25391	6.10728	5.84055	5.71521
ACP2	P11117	6.55948	6.56685	6.03923	6.35498	6.56555	6.19413	6.22512	6.44602	6.27313	6.05056	5.77101	5.75984	6.44731	6.13118	6.39497	6.22066
LRCH2	Q5VUJ6	6.61749	6.18886	6.2011	5.57808	6.33142	5.98273	6.31057	6.35018	6.33138	6.19691	6.32302	6.2139	6.42435	6.42808	6.45001	6.07193
ENDOD1	O94919	6.07032	6.66954	5.8557	6.41301	6.02143	6.1729	6.5559	5.91003	5.97896	6.13533	5.66196	5.90159	6.5863	6.10035	6.99503	6.97164
PRPS1	P60891	6.48534	6.0771	6.50929	6.08254	6.28221	7.18265	6.25776	6.47734	5.82975	6.06293	6.62838	6.32947	6.2825	5.72573	5.74509	6.04192
ULBP3	Q9BZM4	6.58687	6.90962	6.50744	7.33336	6.04815	5.97284	6.35794	6.02184	6.1608	6.43141	6.29137	6.46655	6.01075	5.37969	5.77345	5.74792
ZMAT2	Q96NC0	6.70665	6.24336	5.81569	6.26861	5.98832	6.15165	6.23291	6.10078	6.38276	6.17811	5.71996	5.42207	6.87981	6.72942	6.7909	6.389
MTBP	Q96DY7	6.02548	6.33646	6.66762	6.95801	5.7804	6.31009	6.67133	6.04878	6.13994	6.33098	6.2988	6.71752	5.7152	5.9219	6.07885	5.99865
SLC26A2	P50443	6.62789	6.77937	6.14483	6.5719	6.45616	6.14116	6.38216	6.18796	6.21884	6.31438	6.12713	5.90038	6.15668	5.94661	5.9891	6.05545
NFYA	P23511	5.80317	6.18594	6.17206	5.98934	6.13541	6.35631	6.04235	6.31656	5.77858	6.19534	6.31503	6.15828	6.66733	6.71924	6.6283	6.53676
MPI	P34949	5.51256	4.79802	6.1827	4.80089	6.11507	6.45278	6.06823	7.15688	5.76335	7.06284	6.77807	7.41864	5.80612	7.10134	6.29717	6.68535

GBP1	P32455	6.18849	5.96	6.63719	5.60614	6.15628	6.34408	6.40977	6.26964	6.08692	6.57107	6.40466	6.70582	5.99834	6.59549	6.07275	5.99336
PSME3IP1	Q9GZU8	5.19728	5.93666	6.32489	5.06986	5.49579	6.69439	5.7027	6.82326	5.88038	6.2684	5.86139	6.2666	7.20895	7.33133	6.85739	7.0807
LLPH	Q9BRT6	6.03364	6.15425	5.89913	6.52854	5.94716	5.65276	6.22635	6.02656	6.0515	5.91051	6.0672	6.01273	6.10064	7.16609	7.16192	7.06101
WIPI2	Q9Y4P8	6.55858	6.4982	6.75692	5.9619	6.11882	6.64838	6.68469	6.32876	6.51989	6.24543	6.19114	6.34651	5.79657	6.01677	5.77124	5.5562
DPCD	Q9BVM2	5.9568	5.98427	6.49554	5.5704	6.06485	6.75148	6.36349	6.5063	6.503	6.54556	6.40553	6.25063	6.01792	6.52164	6.03067	6.03192
CRIP1	P50238	6.00038	6.05348	6.53546	5.63602	6.83119	7.65076	5.91916	7.08681	6.01125	6.92213	7.0769	6.63215	5.98849	5.89531	4.94239	4.81812
CARNMT1	Q8N4J0	5.55478	5.62443	6.23055	5.7944	5.85658	6.50846	5.67652	5.99263	5.66984	6.3537	6.56829	6.31108	6.39666	7.60036	6.85807	7.00365
CHSY3	Q70JA7	6.47592	6.62373	6.19606	7.2539	6.55499	6.51537	6.18609	6.13113	6.07782	6.46456	5.89133	6.44873	6.02002	5.89277	5.50024	5.76732
GALE	Q14376	5.79368	5.70474	6.54526	5.5088	5.86442	6.32969	5.83005	6.38199	6.12979	6.96153	6.89199	6.82248	6.73602	6.69067	6.14698	6.56191
DUSP11	O75319	6.49435	5.90246	6.22886	6.89236	6.382	6.35317	6.68215	5.72153	6.24711	5.87615	6.12008	6.16655	6.30786	5.79984	6.32037	6.50516
B3GLCT	Q6Y288	6.28909	6.64732	5.95676	6.5808	6.68828	6.41932	5.94895	6.72579	5.72444	5.85424	6.14965	5.60027	6.37809	6.37351	6.46201	6.20149
UPP1	Q16831	5.77052	6.10346	6.30747	5.55705	6.11865	6.37797	6.04764	6.17941	6.06268	5.92914	6.16878	6.16143	6.34204	7.42073	6.95365	6.49936
HAGH	Q16775	6.63599	6.05307	6.3903	6.08397	6.54224	6.22527	6.23704	6.38254	6.40118	6.25889	6.63798	6.24767	6.45268	6.05451	5.91291	5.48375
ERRFI1	Q9UJM3	5.67941	5.47329	6.32992	5.17511	6.26121	6.71116	5.81767	6.55751	5.77196	5.79404	6.35428	6.20028	7.8247	6.33038	7.08127	6.63782
TCEAL4	Q96EI5-2	5.97903	6.08857	6.67747	5.79627	5.99285	6.79155	5.60734	6.5758	6.29419	6.96149	7.07816	7.30643	5.75102	5.95577	5.70451	5.43955
ITM2C	Q9NQX7	7.29152	8.09227	6.64491	7.34986	6.47407	6.43198	6.74749	5.97999	6.86068	7.09833	6.61721	6.58033	4.3541	4.72029	4.24207	4.5149
KLHL36	Q8N4N3	5.51505	5.97353	6.36749	5.54426	6.01875	6.4809	6.07081	5.85445	6.25617	6.24141	6.31832	6.33307	6.47329	6.88824	7.02197	6.64228
PDCD2L	Q9BRP1	6.78947	5.7636	7.07466	5.8652	6.05564	6.25756	6.14497	6.64927	6.30139	6.31808	6.70116	7.03069	5.89714	5.77251	5.60422	5.77443
MANSC1	Q9H8J5	6.75832	6.34595	6.40567	6.35528	6.49146	6.07903	5.53496	6.59916	6.2078	6.44544	6.58661	6.46827	5.99604	5.85058	5.87264	6.00278
POLR2I	P36954	6.98647	5.4207	6.02796	5.23091	6.23836	5.76185	5.7021	7.09863	5.95332	6.73948	5.87265	6.80025	5.19055	7.0414	6.67345	7.26191
TRMT61A	Q96FX7	5.48013	5.92144	5.59857	5.60305	6.33694	6.86724	5.59427	5.96099	5.97603	6.0174	6.30871	6.18112	6.88373	6.79741	7.47793	6.99504
CFDP1	Q9UEE9	5.52964	5.64498	6.14123	5.39726	5.38549	6.14731	5.50778	6.36477	6.02302	6.05926	6.74145	6.26495	6.72772	7.44492	7.40074	7.21949
MGAT4B	Q9UQ53-3	6.49061	6.18482	5.89644	6.8264	6.16208	6.11762	6.55943	5.86296	6.40407	6.35664	6.1521	6.07485	6.48898	5.97968	6.37401	6.06932
TNIP1	Q15025	6.97512	6.5689	6.92187	5.99099	6.53786	6.55192	6.17426	6.65641	6.80809	6.71383	6.67778	6.77352	5.5935	5.23025	4.86596	4.95973
GSTM2	P28161	6.17615	5.46696	7.02968	5.11236	6.51418	6.60516	5.9604	6.70218	6.1799	6.83553	6.53922	6.30971	6.04413	6.49987	5.96269	6.06187
HYPK	Q9NX55	6.29451	5.2081	5.50454	4.82475	5.07262	4.66773	5.15991	6.21512	4.34703	6.08505	5.40962	6.61861	5.31072	10.6512	8.00616	10.6243
RAB23	Q9ULC3	6.1162	5.98919	6.26714	6.66922	6.04206	6.08051	6.60133	5.9997	6.59866	6.5903	6.26999	6.1275	6.17262	5.85411	6.3255	6.29598
PALM3	A6NDB9	6.08046	6.56199	6.41358	5.89296	6.22777	6.21991	6.41017	5.80685	5.87441	6.45298	6.71616	6.60033	6.58751	6.16109	5.97721	6.01662
NDUFC2	O95298	6.65833	5.58937	6.12409	6.14323	6.36073	5.72548	6.67536	6.1029	6.22105	6.07753	6.32314	5.76544	6.40445	6.75034	6.69357	6.38499
SLC25A17	O43808	6.42806	6.36659	6.01919	6.79782	5.9718	6.00403	6.1664	6.18123	6.09126	5.65226	5.77009	5.74191	6.89109	6.48432	6.5977	6.83625
PMF1	Q6P1K2	5.76606	4.88609	5.6297	4.90663	5.92772	4.68247	5.16285	6.98426	4.62623	6.19363	5.37808	7.41807	5.92729	9.01367	8.2352	9.26206
SIGMAR1	Q99720	6.3347	6.39269	5.56149	6.07526	6.05273	6.01376	6.29284	6.03326	6.4887	6.12284	6.00059	6.13376	6.63131	6.41335	6.66472	6.788
CNNM4	Q6P4Q7	6.4848	6.38278	6.0843	6.97715	6.412	6.10472	6.49346	5.85822	6.20215	6.15655	5.88123	5.88818	6.20283	6.29936	6.32815	6.24412
MADD	Q8WXG6-4	6.25009	6.50925	6.43723	6.50476	6.11547	6.15669	6.45173	6.79617	6.31416	6.67152	6.36853	6.38904	5.78413	6.89422	5.63948	5.61753
PANX1	Q96RD7	6.2531	6.56927	5.93046	6.70676	6.34922	6.04514	5.67478	6.19288	5.77182	6.14897	6.13792	6.46121	6.28781	6.39322	6.23601	6.84144
MOXD1	Q6UVY6	6.67466	6.9171	6.54243	6.63226	6.00368	5.99346	6.70641	6.5646	5.7821	6.24977	6.18694	6.0991	5.88327	5.77429	6.23784	5.75209
HEBP1	Q9NRV9	5.48078	5.5563	6.09285	5.45585	6.56513	6.06684	5.96401	6.37424	7.05456	7.002	6.51184	7.25627	6.19909	6.02419	6.00523	6.39081
STING1	Q86WV6	6.68202	6.46037	5.70867	5.87363	6.07592	5.98643	6.18753	5.90025	6.16766	5.90064	5.90386	5.57283	6.88466	6.81802	7.13065	6.74685
MTMR9	Q96QG7	6.14622	6.70752	6.8142	6.09513	6.39726	6.13141	6.16383	6.89329	6.35278	6.46138	6.7743	6.35158	5.78638	5.79043	5.643	5.4913
NME7	Q9Y5B8	6.17828	5.34926	6.94581	6.28666	6.33173	6.98585	6.35138	6.39996	6.16871	6.44948	7.03651	6.74806	5.7617	5.56985	5.46172	5.97504
WDR37	Q9Y2I8-2	5.97662	6.43271	7.14521	6.42643	6.55925	6.51016	6.34138	6.56457	6.83144	6.25793	6.15711	5.99316	5.90656	5.90184	5.48458	5.51105
C12orf29	Q8N999	6.19474	5.95542	6.92597	6.09978	6.29117	6.54616	6.1014	6.6312	6.40848	6.59187	6.67107	6.99741	5.78153	5.55939	5.49786	5.74656
TTC3	P53804	6.6544	6.62749	6.68236	6.52056	6.20005	6.72017	6.14963	6.37944	6.54755	6.80998	6.89955	6.81058	5.00777	5.59893	5.0961	5.29543
GCLM	P48507	5.98168	6.23034	6.98651	6.42494	6.40874	6.76557	6.24629	6.59125	5.77447	6.73155	6.59454	6.90559	5.64489	5.66012	5.35101	5.79809
FARP2	O94887	6.68467	6.71788	6.54693	6.86488	6.07136	6.10977	6.73649	6.21152	6.51255	6.31544	6.14139	6.21347	6.58795	5.53715	5.64972	5.99884
SEZ6L2	Q6UXD5-5	6.6715	6.59691	6.49305	6.30429	6.28837	6.41453	6.39936	6.56918	6.41823	6.59283	6.61715	6.49751	5.4976	6.07131	5.26912	5.29906
DHDDS	Q86SQ9-2	6.28639	7.31244	6.54517	6.69675	6.4539	6.57917	6.24689	6.10481	6.23619	6.52455	5.96522	6.22667	5.86952	5.54261	5.75808	5.65165
TST	Q16762	6.37303	6.40553	6.03967	6.73207	5.85873	5.94978	6.22256	6.10581	6.09662	6.16673	5.6773	5.96887	6.84199	6.43402	6.42987	6.69741
WIPF2	Q8TF74	6.30659	6.33989	6.65943	6.02119	6.01683	6.87301	6.20776	6.68691	6.69529	6.34913	6.71155	7.03328	5.78681	5.84285	4.92151	5.54797
GMPPB	Q9Y5P6-2	6.30198	5.83153	6.68915	5.79273	6.34883	6.63897	6.06275	6.52301	6.34556	6.50861	5.9657	5.84637	6.31981	6.67535	6.10463	6.04503
VMP1	Q96GC9	6.18122	6.59816	6.5731	6.86254	6.26058	5.73404	6.06377	5.44754	5.90872	6.38768	5.32185	5.73192	6.74279	6.3749	7.04402	6.76717
OCLN	Q16625	6.56554	6.57106	5.96047	6.93154	5.96532	6.36818	5.85061	6.29965	5.93125	5.50709	6.17969	6.523	5.82415	6.43975	6.05181	7.03089
NME6	O75414	6.16798	5.09565	5.71694	5.66644	6.1343	5.36701	5.99781	6.30161	5.27417	6.28766	6.04672	6.57188	6.48689	7.43655	7.52743	7.92098
ZHX1	Q9UKY1	5.86379	5.82817	5.65123	6.15553	6.24364	6.10623	6.48188	5.75209	5.95859	6.07217	5.88243	5.85508	7.05087	6.99903	6.85153	7.24774
REEP4	Q9H6H4	6.19032	6.58327	5.57435	6.62965	6.06392	6.20873	6.31916	6.03518	6.17363	6.45693	6.60106	6.3644	6.04411	5.99436	6.39786	6.36308
THOC7	Q6I9Y2	6.57977	6.36508	5.69684	6.52401	6.1727	6.29672	6.00511	6.21929	6.38832	5.97639	6.08024	6.27882	6.75217	5.85377	6.37432	6.43643
OBI1	Q5W0B1	5.90221	5.83682	5.9434	6.7248	6.11783	6.25813	6.26181	6.08658	5.75404	5.67217	6.10885	6.34404	6.73295	6.35657	6.84937	7.05042
CASP8AP2	Q9UKL3	7.30153	6.92554	6.4806	7.34883	6.70688	6.09023	6.68429	6.31543	6.8219	6.18712	6.65873	6.0173	5.20365	5.09476	5.11342	5.0498
SPC24	Q8NBT2	6.19875	5.07519	5.6881	4.70306	5.5576	5.63231	5.29814	6.96232	5.30739	6.34752	5.34086	7.22918	4.9907	9.61801	7.32206	8.72882
FCHSD2	O94868	6.42231	6.21882	6.68536	6.29461	6.58273	6.73665	6.1849	6.59129	6.39081	6.78546	6.78317	6.55613	5.78432	5.67878	5.25019	5.71946
ANKRD50	Q9ULJ7	5.96383	6.15706	6.59646	6.13874	6.05848	6.12126	6.14269	5.52471	6.44844	6.33662	6.27595	6.05033	6.62671	6.51836	6.73966	6.30069
GMNN	O75496	5.38752	6.00909	6.22065	5.14841	6.07251	6.27683	4.98573	6.81061	6.53334	6.47312	6.74935	6.83879	6.60708	6.73598	6.53346	6.61753
DAXX	Q9UER7	5.68451	5.90091	6.25084	6.47952	6.25405	6.03236	6.37938	6.38614	5.6808	6.37007	6.51828	6.30964	6.55423	6.4808	6.18301	6.53544
ATP5PF	P18859-2	6.46762	5.15637	4.23162	5.77867	5.31721	3.57846	4.47505	5.39703	3.71721	4.55445	4.65038	4.39567	4.6889	14.331	9.65135	13.609
NCKAP5L	Q9HCH0	6.37194	6.80677	6.63132	5.96263	6.00615	6.30243	6.20777	5.96056	6.26583	6.88093	6.55922	6.68305	6.29169	5.86447	5.38599	5.81924
ARHGEF7	Q14155-5	6.37174	5.92112	7.02084	5.90757	5.94968	6.82652	6.3817	5.89513	6.63534	6.3711	6.90848	6.12232	6.41114	5.66134	5.92578	5.69022
ARHGEF6	Q15052	5.98586	6.19349	6.66407	5.81639	6.49724	6.69503	5.57427	6.45173	6.38236	6.75951	6.28249	6.4076	6.17321	6.46311	5.98657	5.66707
PLEKHM1	Q9Y4G2	6.67273	7.00484	6.26766	6.23141	6.09697	6.44082	6.15149	6.27408	6.7448	6.67005	6.22067	6.28647	5.56121	5.85637	5.64118	5.87924
PDE6D	O43924	5.08898	5.90801	5.95839	5.37758	6.45994	6.51158	6.23152	7.6336	6.86999	7.37908	7.07191	6.45706	5.33211	5.74632	6.10708	5.86687
WWC2	Q6AWC2-6	6.83342	6.09565	6.97333	6.21141	6.27046	6.72837	5.63641	6.52048	6.638	6.09675	6.2708	6.36587	5.82244	5.4263	5.47044	5.73986
RNASEH2B	Q5TBB1	5.42079	5.8563	6.59117	5.91568	5.50002	6.07845	5.86677	5.70791	5.82468	6.22515	6.493	6.43986	6.45823	7.26543	7.35835	6.9982
TRUB1	Q8WWH5	6.01453	5.90997	6.25984	5.95002	6.14558	6.22976	5.90192	6.20868	6.19494	6.3201	6.31292	6.30787	6.92694	6.62383	6.63913	6.05398
KANSL3	Q9P2N6-3	6.72868	6.53909	6.40722	6.71115	6.28553	5.79446	6.45184	6.11956	6.61188	6.40225	6.35417	6.44477	5.97271	5.84235	5.63246	5.70187
FASTKD3	Q14CZ7	6.25538	6.16536	6.218	7.08085	6.17392	5.94833	6.64688	5.53504	6.27312	5.89557	6.00131	6.52361	6.55094	5.79623	6.68172	6.25373
RERE	Q9P2R6	6.13979	6.50482	6.33897	6.31696	6.52237	6.23726	6.38223	6.0317	5.84203	6.22889	6.31983	5.92736	6.21644	6.22631	6.22226	6.54279
COX15	Q7KZN9	6.05589	6.59785	5.67714	6.38567	6.20144	6.19839	6.28434	6.31603	6.51146	5.8497	5.95524	6.08017	6.35677	6.22989	6.77271	6.52732
TMEM126A	Q9H061	6.43971	5.99887	6.17667	6.4328	6.39179	5.7652	6.24899	6.27794	5.79162	6.39741	5.8422	6.36673	6.38859	6.51634	6.67742	6.28774
CLK3	P49761	6.13876	6.18883	6.06883	6.45262	6.31983	6.02565	6.45342	5.887	6.60106	6.04004	6.39795	6.19597	6.14953	6.07928	6.53578	6.46547
ASF1A	Q9Y294	5.99091	6.46532	6.1404	5.97176	5.95598	6.39969	5.7409	6.34024	6.39253	6.66692	6.70567	6.53666	6.19576	6.21355	5.86705	6.41666
TRAPPC5	Q8IUR0	6.07017	6.00691	6.59886	5.96207	6.20567	6.875	6.47577	6.65472	6.46578	6.67771	6.7623	6.71667	5.68828	5.82032	5.63302	5.38674
ALG10	Q5BKT4	6.27349	6.92753	5.97054	6.43009	5.47227	6.22994	6.4288	6.0922	6.07418	6.07294	5.36109	6.1084	6.37247	6.50462	6.859	6.82242
LIN7C	Q9NUP9	5.99736	6.43823	6.49358	6.53168	6.32166	6.27385	5.97852	6.36333	6.33829	6.00372	6.92654	6.37665	6.43683	5.62282	5.89743	5.99953
IFT74	Q96LB3	6.36589	6.20612	6.61868	5.7557	6.46797	6.9782	6.08423	6.87431	6.77445	6.36068	6.56434	6.70541	6.03823	5.57697	5.48761	5.14118
COMMD1	Q8N668	6.17867	5.97425	6.78701	5.77567	6.24202	6.31641	6.69446	6.20868	6.1382	6.08283	6.90604	5.63729	6.52316	6.81361	5.87336	5.84834
FRMD6	Q96NE9	6.04728	5.87115	6.2778	6.30776	6.48163	6.34047	6.44375	5.97572	5.79518	6.01176	6.15192	5.6938	6.86854	6.44085	6.7757	6.51669
YEATS4	O95619	6.48157	6.4916	6.34479	6.71854	6.43231	6.23151	6.41861	6.1243	6.27727	6.75595	5.95566	6.03196	6.0153	5.80444	5.90074	6.01545
COMMD9	Q9P000	5.65006	4.8624	5.9343	4.33657	5.64854	4.9777	5.46124	6.10332	4.64801	6.25793	5.42273	6.32701	5.62069	10.8668	7.93773	9.94498
UTP11	Q9Y3A2	6.48538	6.23962	5.15525	6.29179	6.38974	6.13677	6.26124	6.37458	5.80494	6.0412	5.51981	5.63641	6.96548	6.75455	7.15507	6.78814
RASAL2	Q9UJF2-2	6.23334	6.04231	6.51622	6.33175	6.20444	6.33829	6.08153	6.58998	6.00063	6.10138	6.33994	6.242	6.10359	6.31417	6.34394	6.21649
PPRC1	Q5VV67	7.28172	6.6871	7.61828	7.76511	6.28766	5.84797	7.11264	6.31804	6.63874	6.20614	7.06612	6.89048	4.79018	4.04009	4.66745	4.78227
NDUFS5	O43920	6.4719	6.40875	6.19219	6.50399	6.27544	6.18371	6.39759	5.80069	6.224	6.28575	5.78519	5.60806	6.68759	6.43755	6.40403	6.33358
MTMR10	Q9NXD2	6.10672	6.24963	6.25489	6.20156	6.22988	6.13983	6.22804	6.22968	6.14709	6.22698	6.12748	6.16823	6.54916	6.36135	6.34753	6.43193
TBC1D22B	Q9NU19	6.62726	7.29101	6.76928	6.46921	6.18633	6.25222	6.06097	7.00456	6.15242	6.69453	6.32077	6.1099	5.31498	5.80533	5.4027	5.53855
FEZ2	Q9UHY8-2	6.43231	6.57863	7.36997	6.36758	6.28298	6.96684	6.50313	6.52998	6.90571	7.09787	7.47601	6.98587	4.81946	4.71688	4.2641	4.70269
EIF1	P41567	6.18157	5.59062	7.34432	5.80571	6.43014	6.0307	6.05762	6.29764	6.37123	7.35868	7.4848	7.22207	5.1102	5.54526	5.6153	5.55415
SPG21	Q9NZD8	6.24545	5.94913	6.24102	5.87815	6.29661	6.09847	6.13584	6.14032	6.17587	6.70229	6.20565	6.67669	5.80696	6.40234	6.43369	6.61151
AGAP3	Q96P47-4	5.86907	6.17583	6.36458	5.73175	6.15693	6.37117	6.39694	6.25585	6.4314	6.33859	6.68193	6.41937	6.34955	6.21511	6.28867	5.95327
PIAS1	O75925-2	5.69109	5.80455	6.23273	6.30552	6.16671	6.54486	6.16378	6.0915	5.87416	6.50402	6.55907	6.42583	6.40884	6.48987	6.42239	6.31509
PARS2	Q7L3T8	6.10464	6.56097	5.55089	6.5229	6.18388	6.06044	6.35158	6.10614	6.11945	6.109	5.96484	6.11596	6.5625	6.7491	6.54523	6.39248
PCYOX1L	Q8NBM8	6.38968	6.20997	5.3055	6.79991	6.32059	5.7722	6.59869	6.01989	6.40459	6.31938	5.66633	5.69348	7.00324	5.93635	6.84272	6.71749
YIF1B	Q5BJH7	6.37585	6.6554	5.72072	6.48962	6.24103	6.22664	6.4376	6.56915	6.12085	6.38584	5.98173	6.00013	6.19711	6.37798	6.20965	6.01069
NOB1	Q9ULX3	6.12987	6.65883	6.93259	6.16658	6.32093	7.23529	6.33903	6.70524	6.48999	6.61657	6.9522	6.98067	4.89928	5.39996	4.9328	5.24017
KNSTRN	Q9Y448	6.35654	6.20073	7.13334	6.34372	6.26089	6.89166	6.65762	7.04455	6.59339	6.58292	7.50421	7.28027	5.27071	4.82903	4.35817	4.69224
DHCR7	Q9UBM7	6.22766	6.35721	6.25364	6.35244	6.04672	5.95476	6.466	5.75512	6.24334	6.68907	5.89044	5.85053	6.44398	6.26329	6.72025	6.48554
TMEM248	Q9NWD8	7.40967	7.30786	7.28789	7.51043	6.45166	6.4473	6.60931	6.44675	6.81955	7.02233	7.14327	7.20323	3.91163	4.13793	3.96047	4.33073

AAR2	Q9Y312	6.44104	6.75603	6.55311	6.2546	6.9428	6.77733	6.30971	6.55472	6.25584	6.06353	5.97883	5.69933	6.09742	5.90498	5.82288	5.58786
COQ7	Q99807	6.46516	5.6907	5.5923	5.69716	5.75059	5.5622	5.99712	5.86925	6.13723	6.12202	5.63857	6.01953	7.03405	7.08847	7.45281	7.88283
RHOF	Q9HBH0	6.89447	6.83141	6.28402	6.62168	6.21417	6.04221	6.49242	6.24354	6.55819	6.47011	6.21082	5.97611	5.80565	5.67383	5.76535	5.91601
PHF12	Q96QT6	6.61132	6.6938	6.1681	6.5193	6.09587	5.98566	6.56853	5.93273	6.35292	6.17853	6.02439	6.35371	6.10176	6.11917	6.24539	6.04883
SNX33	Q8WV41	6.52332	6.36963	6.87644	6.55706	6.42998	6.86256	6.5134	6.79306	6.40126	6.42981	6.32654	6.43638	5.5826	5.45252	5.42652	5.01892
COMMD5	Q9GZQ3	5.74052	4.97014	6.0344	4.36098	5.89341	5.37665	5.36622	6.84162	5.06134	6.50586	5.82784	6.57345	6.40031	8.8335	8.07081	8.14295
NMRAL1	Q9HBL8	6.44938	6.47503	7.05393	6.10536	5.99983	6.88317	6.36631	7.12123	6.27731	6.63675	6.76561	6.52948	5.45421	5.41445	5.03162	5.43635
PSPH	P78330	6.59969	6.48933	6.31413	5.8592	6.83125	7.40852	5.9105	7.49531	5.98948	6.39308	6.09795	6.04619	5.78777	6.15035	5.42335	5.2039
DAPK3	O43293	5.87242	6.31803	5.95683	6.25422	6.1092	6.47027	6.28305	6.119	6.17032	6.62788	6.26442	6.25451	6.13749	6.58063	6.108	6.47374
ADRM1	Q16186	6.38609	6.19404	6.33916	6.14029	6.22143	6.49101	6.07206	6.35163	6.28573	6.24291	6.44388	6.49868	6.3837	5.72414	6.19185	6.0334
SLC25A16	P16260	6.48744	6.43775	5.90101	7.03673	6.33383	6.24236	6.59602	6.02862	6.30486	5.90176	5.89052	6.18141	6.33273	5.96182	6.59365	5.76951
PHKG2	P15735	7.07575	6.77349	7.34956	6.95655	6.6383	6.86183	6.15722	6.48036	7.24762	7.41804	6.82581	7.00134	3.98383	4.60447	4.06372	4.5621
UBE2Q1	Q7Z7E8	6.06249	6.14452	7.04273	6.25748	5.90617	6.30722	6.21997	6.60516	6.16931	6.52456	6.92499	6.89058	5.78736	5.99191	5.7661	5.39945
EFNB1	P98172	6.54138	6.01639	5.86446	6.55271	6.12744	5.98506	6.37751	5.83832	5.80844	5.8918	6.24888	6.56876	6.28682	6.36271	6.8209	6.70842
NTPCR	Q9BSD7	6.07522	6.05231	6.13578	5.98678	6.44784	6.71812	6.16596	6.89015	6.59585	6.5266	6.27563	6.24367	6.40304	5.92059	5.80919	5.75328
CLNS1A	P54105	6.58684	5.91101	6.69452	5.38311	6.84961	6.53697	6.42571	7.27564	6.99907	6.57769	6.71371	6.51021	5.74954	5.36743	5.33657	5.08238
POLR1D	P0DPB6	6.43329	5.4734	5.91955	5.34057	6.17485	5.55427	6.5622	6.59134	5.5374	6.34924	6.07897	6.95852	6.20192	7.14572	7.22212	7.36663
SLC25A15	Q9Y619	6.55774	6.56457	5.82887	6.42557	6.47656	5.8041	6.34431	5.81612	6.06427	5.83786	6.00283	6.27214	6.4143	6.40968	6.60654	6.57454
MT-ND1	P03886	5.70983	6.70445	6.02912	6.30827	6.04237	5.84435	6.34524	6.26543	6.02387	5.9109	5.75812	6.09574	6.60327	6.67374	6.94948	6.73582
TROAP	Q12815	6.80752	6.36355	6.43867	6.26574	6.44541	6.00781	6.30426	6.89661	6.6806	6.0291	6.56771	6.80776	5.71483	5.67699	5.39718	5.59626
ATOX1	O00244	6.60438	6.4452	7.3469	5.77647	6.70375	7.33249	5.20163	7.19274	5.41201	5.8592	5.61905	6.07834	4.62596	7.60825	6.31185	5.88178
PPP4R4	Q6NUP7	6.17305	6.03977	7.07004	6.13264	6.69496	6.34113	6.39854	6.23614	6.42047	6.49954	6.22463	6.65164	6.06759	5.84788	5.61488	5.5871
GULP1	Q9UBP9	6.30642	6.26621	6.17978	6.91744	6.01052	6.38146	6.11232	5.53915	6.16395	6.4555	6.3669	6.32497	5.94406	6.35732	6.1941	6.47989
MEMO1	Q9Y316-3	5.92218	6.00807	6.95495	5.93656	6.13284	6.88901	6.14565	6.53203	6.42939	6.57453	7.17991	7.01727	5.76256	5.47444	5.11277	5.92785
NR2F2	P24468	6.40252	6.44578	6.16792	6.73695	6.28417	6.21919	6.38779	6.49239	5.81372	6.10276	5.70891	6.08568	6.39036	6.58201	6.4452	6.33465
SELENOT	P62341	6.40179	6.50563	5.98183	6.21096	6.4116	6.49605	6.03792	6.12673	5.89342	6.25726	5.78296	5.72821	6.48038	6.28016	7.01792	6.38719
ACP1	P24666-2	6.12536	5.40258	6.90263	5.44134	6.57733	6.16367	6.2336	6.90921	6.19863	6.76514	6.54811	6.70575	6.09718	6.22413	6.04859	5.65674
TULP3	O75386-2	6.44026	6.69011	6.51995	7.00024	5.98224	6.08016	6.33912	6.31505	7.4414	7.55813	6.29852	6.8546	5.14757	4.6376	5.61468	5.08036
OSGEP	Q9NPF4	6.10584	5.56345	6.1301	5.50055	5.85074	5.94736	5.69237	5.77854	6.08491	5.95667	6.26808	6.06787	7.02717	7.34987	7.72774	6.94875
ATG101	Q9BSB4	6.20159	6.50418	6.77163	6.22486	6.06593	6.90721	6.18622	6.48003	6.38013	6.30196	6.65715	6.66559	5.81876	5.77676	5.62832	5.42967
LRIG2	O94898	5.99609	6.32321	6.35985	6.87021	6.46715	6.20209	6.15303	5.99609	6.25373	5.89572	6.44804	6.30436	6.40218	6.15352	6.21116	5.96356
CHMP1B	Q7LBR1	6.26814	6.13614	6.4864	5.58208	6.26299	6.59905	6.12873	6.4917	6.34838	6.5053	6.56924	6.21119	6.34853	6.18656	6.11037	5.7652
RFT1	Q96AA3	6.44994	6.4076	5.84336	6.46466	6.03119	5.94037	6.30448	6.19287	5.85696	5.48048	5.93521	6.0028	6.62929	6.72588	6.9762	6.75871
RPL35	P42766	6.04281	6.39606	5.7183	5.85266	6.09055	6.20976	6.40836	6.75665	6.70886	6.33602	6.40205	6.58908	6.0929	6.54334	5.71497	6.13762
GPAA1	O43292	6.52252	6.3754	5.89079	6.37554	6.12661	6.06328	6.33143	6.18522	6.08447	6.16172	5.97584	5.86852	6.54932	6.54771	6.66678	6.27486
UBA5	Q9GZZ9	6.42292	6.28658	7.02045	6.21031	6.64073	7.12471	6.29898	6.86186	6.65605	6.54833	6.36732	6.41182	5.16628	5.74711	5.33482	4.90173
MLF2	Q15773	5.5505	6.05747	6.16545	5.62352	5.91945	6.41151	6.27297	6.64407	6.06394	6.54571	6.6081	6.24678	6.5244	6.28571	6.41478	6.66564
SURF4	O15260	5.98732	5.95498	5.5757	6.12606	6.03671	6.17204	6.03621	6.17245	5.98245	6.37148	5.93693	6.10243	6.62292	6.83299	6.94953	7.13978
PSMG1	O95456	6.08448	6.39793	7.0306	5.96383	6.12653	6.6958	6.2923	6.55479	6.40939	6.67323	6.20283	6.56588	5.45916	5.54118	6.16697	5.83508
CD151	P48509	6.40677	6.54956	6.0682	6.26323	6.28299	6.08825	6.45902	6.22776	6.2543	6.17902	5.78264	6.11102	6.43088	6.39392	6.15731	6.34515
CMTR2	Q8IYT2	6.25482	6.07917	6.73422	6.05644	6.44159	6.54871	5.76602	6.47922	6.11312	6.7927	6.46807	5.96528	6.05584	6.2412	6.18172	5.82186
MLST8	A0A0A0MR05	6.46202	6.08186	6.18517	6.12453	6.4992	7.15774	5.53352	7.12611	6.94412	7.06986	6.98108	7.09262	5.74564	5.32449	4.87995	4.79208
OSTC	Q9NRP0	6.3662	6.15915	5.67007	6.63088	6.22057	5.96376	6.56969	5.95742	6.08295	6.33737	5.88578	5.78291	6.50336	6.48878	6.81073	6.57037
MIB2	Q96AX9-14	6.26125	6.50698	6.32498	6.28088	5.70708	6.49208	5.74316	6.04613	6.69165	6.78009	6.71691	6.59842	5.90206	6.13842	5.97845	5.733
MPC2	O95563	6.31707	6.38578	5.80688	6.57354	6.32585	6.02184	6.81723	6.23665	6.30154	6.0715	5.63834	5.7138	6.62226	5.79036	6.66523	6.71212
NDUFS4	O43181	6.2928	6.13409	5.54578	7.12827	6.10526	5.63581	6.47392	5.79658	5.72175	6.04503	6.03649	6.15874	6.49798	6.54768	6.49069	7.38912
CD81	P60033	5.9441	6.20381	6.29617	6.28526	5.80953	5.74665	6.4803	5.9779	6.0725	6.30694	6.27974	6.23463	6.43578	6.67881	6.2129	7.03497
AKAP10	O43572	6.32306	6.38436	7.10481	6.56303	6.22516	6.24969	5.97329	6.80031	6.0435	6.48827	5.79136	6.02994	5.97234	5.80369	6.21805	6.02916
GAGE5	Q13069	5.91263	5.32467	5.31666	5.197	4.04871	3.3324	4.02414	5.17178	3.28928	5.05684	4.38084	4.45133	3.76142	16.7337	9.70004	14.2986
PGS1	Q32NB8	6.28981	6.41809	5.60064	6.3994	6.44162	6.10433	6.59622	6.31143	6.11939	6.40338	5.79501	5.92584	6.5936	6.00568	6.62358	6.37195
PHPT1	Q9NRX4	6.36478	5.64255	6.88454	4.8778	6.6281	6.36958	5.64637	6.92112	6.20863	6.84342	6.46829	6.76446	6.24805	6.29147	5.6102	6.23065
SUMO3	P55854-2	5.91469	5.14167	6.31324	5.03161	6.19264	5.40302	5.4302	6.05321	5.93534	6.86963	6.58982	6.97406	6.71299	7.62783	6.95865	6.85141
SLC15A4	Q8N697	6.30141	6.56103	6.14136	6.92527	6.11746	6.04517	5.72415	5.95271	6.11327	6.57858	6.05595	6.76285	6.36874	6.23189	6.04714	6.073
SELENOS	Q9BQE4	6.69621	6.85853	5.77391	7.04091	6.35652	7.20071	6.00929	6.86493	6.10471	6.3255	5.88671	5.61145	5.55678	5.5529	5.92422	6.23673
MAGED1	Q9Y5V3-2	6.83396	6.80794	6.59162	6.40182	6.31643	6.53213	6.43492	7.16732	6.60545	6.87315	6.83838	7.46258	4.8991	5.27693	4.45675	4.50151
SINHCAF	Q9NP50	5.81548	5.71397	5.52727	6.54416	6.4791	5.8238	6.54142	6.24614	5.53543	5.79811	5.83279	5.69553	7.57444	6.62989	7.08457	7.1579
B3GAT3	O94766	6.35509	6.386	5.89263	6.23017	6.04665	5.97221	6.42513	6.07078	6.25004	5.98212	6.00495	6.33167	6.37358	6.35057	6.62699	6.70142
RUFY2	Q8WXA3-1	6.5262	6.26739	6.31036	6.59644	5.96441	6.72478	6.23019	6.37734	6.29409	6.63243	6.41113	7.16079	6.52291	6.09183	5.20807	5.58163
TADA3	O75528	5.92251	6.55663	6.21449	6.48452	6.56515	5.78073	5.97592	5.69139	6.43435	7.51302	6.19423	6.91911	5.67971	5.79027	6.35295	6.82502
TPM2	Q5TCU3	6.78822	5.87486	6.60325	6.11682	6.93098	6.24454	6.19553	7.42069	6.37881	6.021	6.78572	6.5941	6.37963	5.56987	4.83443	5.26156
WIZ	A0A669KBH9	6.28932	6.32586	6.1492	6.83711	6.21331	6.01248	6.22675	6.07268	6.2056	5.74462	6.28055	6.4154	6.64134	6.32188	5.92449	6.33942
YBX2	Q9Y2T7	5.96431	6.34807	6.53007	6.33861	5.68291	6.01547	6.174	6.49459	6.41284	6.7437	6.12156	6.19111	6.03817	6.32087	6.1429	6.48081
CLTA	P09496-2	7.28636	5.35152	5.48557	4.7612	5.65464	4.61278	5.42653	6.85838	4.92827	5.66132	5.13207	5.97894	6.77481	9.60855	7.95085	8.5282
TBC1D8B	Q0IIM8	6.70684	5.33939	5.64141	5.37632	5.19433	4.93868	5.15297	6.67447	5.23828	6.1608	5.78928	6.54275	6.43581	8.92212	7.77123	8.11533
PPP1R12C	Q9BZL4	6.19737	5.94615	6.27963	6.2307	6.2053	7.13068	6.52882	6.3986	6.2457	6.73877	6.48807	6.61367	5.85539	6.04997	5.5773	5.51387
PTRH1	Q86Y79	6.65818	6.43703	5.41067	6.55233	6.01884	5.58737	6.59756	6.34497	6.08348	5.95036	6.8738	6.47636	7.37888	5.73494	5.8088	6.08644
KIF5C	O60282	6.35283	6.28256	6.74573	6.3824	6.51977	6.20972	5.78674	6.42605	6.22663	7.15375	6.48915	6.67826	5.39822	6.06161	5.60206	5.68452
PCDHB11	Q9Y5F2	5.95702	5.82789	5.37706	5.06879	6.91644	6.66155	6.65268	5.84067	5.57826	5.89465	5.55973	5.73542	7.47383	7.60654	6.6704	7.17907
SFN	P31947	6.35696	6.70686	6.49413	6.14698	6.12641	6.7453	6.14126	6.62723	6.4875	6.00933	5.98941	6.10845	6.40768	5.96117	5.8291	5.86223
CUL9	Q8IWT3	6.48022	6.60578	6.55517	6.40287	5.93074	6.56461	6.61917	6.71138	6.68658	7.1113	6.72763	6.37737	5.39555	5.65899	5.19501	4.97764
IQGAP2	Q13576	5.95044	6.13409	5.6575	5.93371	5.85342	6.21068	6.37472	6.29757	6.65055	6.24871	5.32178	5.79657	6.98848	6.87755	6.87938	6.82484
H1-4	P10412	6.3791	6.58229	5.43037	6.03031	6.30023	6.21311	6.09255	6.28549	5.82364	6.26864	5.6317	5.1836	6.42138	7.24519	7.04083	7.07155
DERA	Q9Y315	6.60716	6.71628	7.06077	6.22587	6.48542	6.72608	6.32347	6.4869	6.52172	6.61953	6.87916	6.3186	5.49117	5.26596	5.37433	4.89759
CDK16	Q00536-2	6.20401	6.51519	6.66891	6.3828	6.439	6.69394	6.43254	6.48894	6.33744	6.29618	7.07569	6.333	5.55553	5.33379	5.58723	5.65581
PCBD1	P61457	6.57442	5.04274	6.87985	4.68341	6.33258	5.75805	5.5191	7.10272	5.41921	6.87264	6.21912	7.50818	5.01978	7.40495	6.86882	6.79446
IQSEC1	Q6DN90-3	6.20446	6.17562	6.92922	6.62715	6.45895	6.3027	6.55513	6.03379	6.24864	6.24674	6.33764	6.50553	5.96421	6.0095	5.49295	5.90776
DCBLD1	Q8N8Z6	6.39549	6.2732	5.99922	6.49942	6.20975	6.213	6.78449	6.20064	6.72574	6.19933	6.40214	6.46058	5.87965	6.05392	6.0744	5.82913
ISCU	Q9H1K1	6.4069	6.07003	6.2287	5.94006	6.4336	5.94515	6.05119	6.02745	6.53446	6.28347	6.36656	6.78115	6.65768	6.04446	6.20803	6.02109
BCCIP	Q9P287	5.68222	5.84895	6.70972	6.06785	5.70091	5.67678	5.99917	5.64519	6.08959	6.20157	6.50218	6.6313	6.54109	6.54208	7.44767	6.71372
SPICE1	Q8N0Z3	6.14317	6.07461	6.36457	6.63471	5.88324	6.29979	6.57133	6.68603	6.16352	6.4215	6.52959	6.54196	5.97103	6.04275	5.73664	5.93557
HNRNPL	A0A3B3ITJ4	6.07294	6.47073	6.10282	6.68076	6.35579	5.81483	6.16244	6.07456	6.19921	6.52036	6.49216	6.11925	6.66091	5.84174	6.2736	6.15791
PIGA	P37287	6.20478	6.47042	6.3156	6.88478	5.92625	6.03328	6.61706	6.2766	5.88676	6.21251	5.82073	6.14985	6.38095	6.05338	6.42965	6.3374
ATF2	P15336	6.1953	6.10911	6.08344	6.16258	6.59203	6.08103	6.23395	6.04287	6.21716	6.6896	6.6105	6.43901	6.72878	6.06537	5.97328	6.42599
IMMT	Q16891-2	6.66909	5.6743	5.47776	7.00112	6.16155	5.82971	5.66638	5.82002	6.31539	6.21723	5.7334	6.36198	6.96914	6.49087	6.34974	7.26231
MAPK8	P45983-4	6.44594	6.28466	7.01576	6.00071	6.23592	6.77829	5.98598	6.63491	6.35252	6.35028	6.0925	6.36192	5.96391	5.84187	5.98623	5.66861
VRK2	Q86Y07	6.90475	7.06958	6.46336	6.96504	6.51404	6.09795	6.23374	5.84279	6.72408	6.3384	6.23286	6.53786	5.44573	5.41352	5.44862	5.76767
VGF	O15240	7.02626	6.80292	6.24628	6.3498	6.98985	6.1089	6.32913	6.77122	5.81036	5.95163	6.10651	6.67221	6.32492	5.59304	5.41359	5.50338
NPTN	Q9Y639	6.30639	6.87712	6.23685	6.99888	6.23925	6.37598	5.88712	6.05269	5.95157	6.89081	6.04362	6.3876	5.82384	5.82273	5.93751	6.16802
ITPRIPL2	Q3MIP1	6.41951	6.46904	6.41423	7.2123	6.08252	6.18836	6.46663	6.00931	6.39479	6.22121	6.13103	5.92621	6.03869	5.80773	6.22159	5.99684
PHF23	Q9BUL5	5.91077	6.25167	5.73408	6.25704	5.92379	6.21663	6.30437	5.9652	5.9813	6.75728	6.4542	5.68352	6.56044	6.92355	6.38982	6.68633
KATNAL1	Q9BW62	6.32421	6.04846	7.1227	5.95461	6.57559	6.81265	6.55583	7.07119	6.45849	6.4171	6.56231	6.46748	5.5384	5.36907	5.25884	5.46307
ADARB1	P78563-5	6.64723	6.61267	5.43928	6.29879	6.16637	6.14784	6.50867	6.19215	6.38731	6.05431	6.13789	5.82736	6.69071	6.10323	6.58896	6.19724
CLIP1	A0A3B3ISJ8	6.52331	6.61002	6.55188	6.30616	6.50481	6.52422	6.082	6.30335	5.76205	6.82867	6.27394	6.3727	6.0514	5.64485	5.78983	5.87081
DMAC2L	Q99766	6.33854	5.88046	5.55194	5.61851	6.32388	5.7734	6.05213	6.36366	5.66898	6.12144	5.17174	6.15027	7.09619	7.29493	7.46618	7.12775
AMOTL2	Q9Y2J4-4	6.78117	6.1652	6.61297	6.35116	6.25469	6.14748	5.89606	6.57525	6.50617	6.0892	6.95896	6.57164	6.53651	5.30678	5.68608	5.56068
SCAMP2	O15127	6.34916	6.70242	6.70183	6.97598	6.25699	6.30271	6.34967	6.45027	6.076	6.82563	6.90989	6.60227	5.2568	5.54817	5.21233	5.47987
TTC26	A0AVF1	6.74932	6.25049	7.11951	6.70806	6.37673	6.8819	5.31076	6.51298	6.4554	6.96891	6.01354	6.92691	5.27974	5.61392	5.09539	5.73643
TMEM131L	A2VDJ0-5	7.19894	7.38721	6.64537	7.44786	6.25855	6.12012	6.62025	6.15685	6.37027	6.65742	6.54725	6.4326	4.89018	5.14779	5.07772	5.04163
ATRN	O75882	6.30572	6.87228	6.34842	7.02638	6.48787	6.36144	6.5327	6.08493	6.21109	6.39969	6.40746	6.30646	5.6463	5.76485	5.49598	5.74842
DAGLB	Q8NCG7	6.58532	6.52476	6.21519	6.02002	6.09837	6.18109	5.79671	6.18237	6.19048	6.41859	6.67405	6.47181	6.47058	6.40491	5.83976	5.92598
GNB2	P62879	6.25883	6.25667	6.27561	6.08204	6.49046	6.0942	6.44104	6.01868	6.66594	6.24805	6.04653	5.94254	6.27885	5.96693	6.47096	6.46264
USF2	Q15853	6.73532	6.55275	6.32718	6.35861	6.35294	5.77944	6.37553	5.74301	6.23608	5.65496	6.64558	5.6586	6.25603	5.61029	6.88417	5.92951
NUDT3	O95989	6.16492	6.14489	6.91803	5.76221	6.52828	6.6058	6.35003	6.50277	6.55734	6.69349	6.78345	6.44744	5.92879	5.70843	5.65043	5.25371
ERBB2	P04626	6.30631	6.20169	6.69944	6.29649	5.68434	6.64981	6.19037	6.5914	5.91703	6.46009	6.72675	6.6661	6.07359	6.0991	5.74842	5.68906
GPAT4	Q86UL3	6.25087	6.35358	5.75265	6.7414	5.83219	5.96805	6.51293	5.97677	6.06883	6.05449	6.2598	5.89365	6.64052	6.5035	6.8023	6.38847

PCLAF	Q15004	7.4072	7.21781	7.90876	7.37349	6.67105	6.52302	6.4004	6.30551	7.92055	7.22796	8.24017	7.85722	3.23952	3.4572	3.03057	3.21956
PI4KB	Q9UBF8	6.51987	6.5763	6.85153	6.12235	6.57338	6.5154	6.68371	6.56995	6.11578	6.4768	6.37199	6.24597	5.71104	5.89168	5.4104	5.36384
RECQL4	O94761	5.77542	6.00482	6.34354	6.28911	6.11659	6.10819	6.33086	5.95428	6.308	6.92814	6.72494	6.27174	6.10089	6.06816	6.29207	6.38324
MFSD5	Q6N075-2	6.10846	6.39024	5.78159	5.73551	5.80218	5.91917	6.03887	5.75461	6.22397	6.26181	6.61766	6.18289	6.52773	6.73966	6.63628	7.27936
CBLB	Q13191	6.7473	6.18874	6.96206	6.37873	6.3393	6.90222	6.29957	6.22549	6.22664	6.83075	6.86549	6.32102	5.42157	6.05974	5.24367	4.98773
B3GALT6	Q96L58	6.45477	6.21885	5.83708	6.86155	6.02357	6.03702	6.70203	5.79347	6.42855	6.31927	6.15811	6.23375	6.19963	5.97409	6.61411	6.14415
FAM76B	Q5HYJ3	6.42568	6.37907	5.79629	6.54681	6.35944	5.52335	5.88519	6.00365	6.59277	5.82864	5.8795	5.90551	7.12888	6.79647	6.47066	6.47808
JUND	P17535	6.33307	6.27236	6.18895	6.75398	6.08262	6.28488	6.29947	6.28832	5.66013	5.87503	6.39172	6.31771	6.28258	6.16184	6.11708	6.69025
SECISBP2	Q96T21	6.17577	6.48202	6.71532	6.96887	5.93359	6.10132	5.88647	6.38629	6.75469	6.49719	6.61815	6.79967	5.73224	5.87832	5.54314	5.52695
SECISBP2L	Q93073	6.68278	6.56188	6.57608	6.11644	6.11496	6.14346	6.59859	6.52622	6.77994	6.88316	7.15519	6.5439	5.39127	5.22098	5.60699	5.09817
POP4	O95707	6.40681	7.05054	5.68366	6.99356	6.47978	6.44235	6.62402	7.05925	6.05946	5.7876	6.1931	6.14797	5.34392	6.21133	5.94679	5.56985
KCTD9	Q7L273	6.2525	6.01905	6.74394	5.92231	6.53908	6.97192	5.75434	6.68952	6.36567	6.54956	6.55501	6.70223	5.8551	5.65782	5.72944	5.6925
CDC42EP4	Q9H3Q1	6.36053	6.28426	6.49223	6.41935	6.53474	6.3651	5.94907	6.49477	6.09056	6.09091	6.32664	6.09695	6.93063	6.34923	5.85622	5.3588
COPRS	Q9NQ92	7.42024	6.15585	5.59981	4.99904	4.82636	4.85222	5.21885	5.78023	5.50951	6.25419	6.34149	6.84781	5.75283	8.67418	7.31504	8.45235
FERMT1	Q9BQL6	6.05708	6.43092	6.54741	6.0164	5.76509	6.74797	5.95277	6.64799	6.90643	6.85289	6.28264	7.06095	5.54709	5.86915	5.75177	5.56344
LSM1	O15116	5.49916	4.41023	5.55958	4.25352	5.66751	5.35562	5.02023	7.56847	5.80092	6.21971	5.85937	6.30695	5.05438	9.41261	8.71655	9.29519
DDX19A	Q9NUU7	5.96327	6.48649	7.35091	6.51842	6.29165	6.51239	5.9582	6.7964	6.38309	5.98052	6.90455	6.84182	5.40864	5.71611	5.4832	5.40433
ZFAND5	O76080	6.23303	5.62331	6.80229	5.84327	6.6974	6.95032	7.06798	6.56748	6.15908	6.69048	6.94132	6.22813	5.99485	5.40036	5.32364	5.47706
BAP18	Q8IXM2-3	5.99395	4.83445	5.29999	4.90122	5.85452	4.38473	5.18348	6.49256	4.46164	6.23878	5.15004	6.17517	6.70641	9.78698	9.24829	9.28779
PARL	Q9H300	6.54473	6.55045	5.75324	6.18336	6.39414	6.05938	6.11461	5.58172	5.48435	6.19515	5.96371	6.14361	6.58043	6.21118	7.00152	7.23842
KDM5B	Q9UGL1-2	6.26034	6.63011	6.69886	6.84689	6.37747	6.47786	6.71963	6.33209	6.35871	6.28755	5.9946	6.40957	5.35932	5.75796	5.74038	5.74866
TIMM29	Q9BSF4	6.97758	6.501	6.38354	6.51244	5.96726	5.70089	6.32159	5.78288	6.19843	5.77902	6.09835	6.28414	6.53374	5.89509	6.52689	6.53715
ADAT2	Q7Z6V5	5.25572	5.49601	6.56138	5.65337	6.6462	6.51694	5.31967	7.01913	6.24822	6.70628	6.77752	6.58475	6.74659	6.15287	6.08607	6.22929
RABEP2	Q9H5N1	6.91605	5.01862	5.69787	3.91425	5.03954	4.83545	5.71943	7.10333	5.46423	6.13561	5.73155	7.41404	6.86717	8.50267	7.23522	8.40496
RALA	P11233	6.31588	6.59675	6.03764	6.30718	6.06639	5.93093	6.31706	6.11528	6.36341	6.38484	6.27851	5.83342	6.27192	6.49822	6.31238	6.3702
CGGBP1	Q9UFW8	5.40135	5.59754	6.12696	4.67314	5.97575	6.7182	5.43267	5.34854	7.49788	6.72574	6.25834	6.0934	7.22933	7.23307	6.98713	6.70093
HMGN3	Q15651	5.84714	5.79555	5.71464	6.59785	6.18115	6.14456	5.84323	5.99886	5.71406	5.7277	5.52878	5.83919	7.02258	7.59104	6.84888	7.60878
SMYD2	Q9NRG4	7.08266	6.42481	7.33572	6.29948	6.56648	7.01278	5.89771	7.05012	6.62429	6.43487	6.59755	6.52378	5.2288	5.21981	4.93259	4.76856
RNF31	Q96EP0	6.49124	5.73199	6.62146	5.84119	6.15595	6.47113	6.39702	6.61389	6.63016	6.39457	6.80493	6.58158	6.05928	5.59363	5.75627	5.85572
BAHCC1	Q9P281	6.54071	6.5426	6.30162	5.56644	6.17835	6.40992	6.0779	5.68221	6.48479	6.68041	5.64394	6.31405	6.10307	6.09142	6.20859	7.17397
UBE2D3	P61077-3	6.00287	5.50285	6.99042	5.93368	5.86019	6.54921	5.59797	6.89774	5.94592	7.19755	6.65134	6.55891	4.95496	6.92848	6.50626	5.92164
DOCK11	Q5JSL3	6.0992	6.02626	6.38329	5.88721	6.00751	6.38032	5.97778	6.26091	6.37044	6.59238	6.36208	6.50372	6.38762	6.67944	6.03955	6.0423
MSL3	Q8N5Y2	6.49512	6.41942	6.43987	6.83052	6.39753	6.16819	6.33535	6.08084	5.91072	6.49128	6.2576	6.01833	5.9764	5.99215	6.1519	6.03479
DSE	Q9UL01	5.8842	6.44074	6.59806	5.90327	6.16853	6.17688	6.28925	5.98633	6.44917	5.89267	6.267	6.32015	6.56076	6.66613	6.10896	6.28789
ASB7	Q9H672	5.89298	5.89483	6.28282	6.4006	6.38939	5.92137	6.3753	6.32903	6.72747	6.53707	6.14462	6.06552	6.56433	5.98902	6.50219	5.98346
HACE1	Q8IYU2	5.92052	5.90584	6.68613	5.88047	5.73515	6.46329	6.18387	5.84357	6.63602	6.88947	6.71536	6.83619	6.2679	5.9865	5.66291	6.38681
PCBP2	F8VRH0	5.82759	5.33587	6.27041	6.11259	6.20899	6.6468	5.77962	6.12837	6.17807	6.30061	6.81482	7.01545	6.48878	6.2724	6.0619	6.55773
PUS3	Q9BZE2	5.73093	5.64376	5.96971	5.67311	5.64492	6.72911	5.67394	6.18342	5.78985	5.77254	6.13395	6.17993	7.37073	6.82294	7.71423	6.96703
FAM3A	P98173	6.78142	6.55919	6.11952	6.95937	6.65605	6.30831	6.44076	6.29228	6.17945	6.32024	6.31184	6.36686	6.18297	5.47882	5.54146	5.50147
SLC2A3	P11169	6.79978	6.22998	6.07812	6.34776	6.54421	6.3551	6.00532	5.91967	5.38157	6.05397	5.78227	5.7517	6.7567	6.81515	6.57093	6.60778
FKTN	O75072	6.06954	5.95663	6.34828	6.98455	6.40946	6.20863	6.44236	5.9883	6.36873	6.30073	5.79593	6.04046	6.66259	5.8637	6.11806	6.44205
ZNF740	Q8NDX6	5.916	6.4841	6.05996	6.60799	6.01285	6.0988	6.59285	5.83842	5.97512	6.65934	5.81938	6.36031	6.5514	6.19282	6.43437	6.3963
ZNF724	A8MTY0	6.54193	7.37183	6.80851	7.50114	5.87738	6.25921	5.8118	5.92005	6.4789	6.23404	6.65356	6.92291	4.89289	5.55888	5.75953	5.40744
ZKSCAN1	P17029	5.99165	6.31325	6.39858	6.04861	6.10752	6.58415	6.00875	5.95589	6.38651	6.08834	6.47564	5.95307	6.23104	6.11307	6.38681	6.05916
ZNF829	Q3KNS6-3	6.36	6.3668	6.19634	6.75225	6.01558	6.2267	6.31332	6.21304	6.28551	6.00524	6.58324	6.6425	6.012	5.87564	6.12723	6.02462
RBAK	Q9NYW8	6.53976	6.22843	5.84665	7.00471	6.06321	5.86121	5.95633	6.00172	6.43728	6.31552	6.07053	6.03249	6.44513	6.10734	6.70417	6.38551
C1GALT1C1	Q96EU7	6.53251	6.50217	6.3126	6.68564	6.52513	6.21181	6.36925	6.28482	6.42083	6.10021	6.28627	5.86386	6.14527	5.65921	6.03559	6.06483
PPCS	Q9HAB8	6.57641	5.61548	7.04466	5.583	6.23413	6.51818	5.80685	6.70125	6.25245	6.31053	6.65084	6.73055	6.02636	5.8437	6.17127	5.93434
LDAH	Q9H6V9-2	6.82821	6.0101	6.31853	6.16824	5.71059	5.69325	6.37814	6.23955	5.88023	6.12466	5.84245	6.2937	7.009	6.36389	6.87767	6.26178
ZCCHC4	Q9H5U6	5.36395	6.34062	6.17633	6.10316	5.63978	6.13274	6.05975	5.96868	6.42006	6.15771	6.29982	6.0762	6.54561	7.42941	6.81371	6.47247
RAP1A	P62834	6.19195	6.26825	6.19865	6.55297	5.95426	5.56226	6.71761	5.85503	6.61618	5.75047	6.10953	6.14999	6.31442	6.36605	6.71935	6.67303
RAB39A	Q14964	6.50397	6.19489	6.1898	6.73721	6.21262	6.18181	6.24351	5.84694	6.0409	6.67242	5.66337	6.28449	6.4818	5.36271	6.16566	7.2179
RAB6A	P20340-2	6.21417	6.30988	6.20466	6.15887	6.23011	6.01204	6.18216	5.9944	6.27673	6.37577	6.27994	5.85956	6.60812	6.15291	6.84206	6.29861
RAB33B	Q9H082	6.1333	6.56311	6.12346	6.72746	6.45335	6.03214	6.6635	6.20945	6.11763	6.21568	5.76304	5.84492	6.24706	6.26344	6.5249	6.11757
DNASE2	O00115	6.34582	5.69492	5.66887	5.98347	5.74126	5.85123	6.16642	5.5803	5.91302	6.10503	6.66883	6.23881	6.11499	7.51904	7.65426	6.65372
ZSCAN21	Q9Y5A6	5.65893	6.22124	6.31243	6.33702	6.28337	5.99065	6.29203	6.39957	6.1903	6.06478	6.0516	5.35568	6.72304	6.77524	6.58241	6.76171
MXRA7	P84157	6.48032	5.4948	4.52516	5.22712	4.22145	2.86547	4.79476	5.26058	4.21208	4.78158	5.15754	4.85856	5.52944	13.0756	10.7164	12.7991
SNX15	Q9NRS6	5.95464	6.00009	6.41997	5.7816	6.49808	6.62516	5.92594	7.29251	6.42141	6.29698	6.75389	7.08323	5.86005	5.40347	5.93753	5.74544
BUD23	O43709-3	6.32965	6.03153	6.01335	5.72896	5.80551	5.83605	6.10206	6.4556	6.58097	6.39629	6.15369	6.43372	6.39278	6.54279	6.6405	6.55655
MED26	O95402	6.51117	6.44084	6.41935	6.90037	5.90776	5.58254	6.15941	5.87156	6.38916	6.43449	6.32408	6.4541	6.18059	5.74423	6.50369	6.17666
SYNGR2	O43760-2	5.70413	6.50802	5.70206	5.7904	6.03439	6.39368	6.19812	6.24695	6.07735	6.41983	6.43814	6.39736	6.31601	6.51593	6.54751	6.71012
C8orf82	Q6P1X6	6.49324	6.02093	6.21852	6.01573	6.72552	5.85163	6.2486	5.84816	5.97379	6.08067	6.19715	6.10276	6.72395	6.17075	6.70697	6.62163
DAP	P51397	5.02201	4.40979	5.29487	3.95668	5.4456	4.65882	4.06757	5.80549	4.27818	5.45857	5.20072	6.24313	10.154	10.3139	10.0121	10.0786
MAN1A1	P33908	6.45506	6.6707	6.38183	7.17096	6.21449	6.17412	6.25829	5.8096	6.57118	6.2429	6.39935	6.01876	5.73565	5.8382	5.82597	6.23293
RNASEH1	O60930	6.11197	6.20555	5.98991	7.08628	6.56553	6.20469	5.9322	6.41771	5.89232	6.22492	6.60057	6.24394	6.16648	5.86173	6.06574	6.43046
LCORL	Q8N3X6	6.50471	6.15503	6.19016	6.71028	6.10973	6.59379	6.39176	6.29334	6.10741	6.90843	6.74845	6.7098	5.37785	5.51751	5.85401	5.82773
TRHDE	Q9UKU6	6.49574	6.7155	6.47358	6.71706	6.03807	5.55624	6.15408	6.17136	6.12212	6.13481	6.55007	6.41549	5.93627	5.94968	6.25384	6.3161
ALKBH6	Q3KRA9-2	6.47812	6.2272	7.37419	6.39318	6.5264	6.4061	6.42334	6.56045	6.44419	6.43805	6.65265	6.80246	5.49416	5.19493	5.40042	5.18417
SPOUT1	Q5T280	6.0939	6.29929	5.69817	6.47118	6.01739	5.72882	6.49244	6.0391	6.28507	5.96035	6.22196	6.06498	6.46328	6.74244	6.67282	6.74882
FKBPL	Q9UIM3	5.65686	5.33878	6.47764	5.09248	6.37617	6.04636	5.72705	7.32143	5.82765	7.08614	6.91391	8.06909	5.8137	6.55791	5.05136	5.74348
DUSP12	Q9UNI6	5.82489	5.81504	6.23655	5.52935	5.86195	6.09781	5.80897	5.7902	5.7749	6.23441	6.38801	6.28672	7.03938	6.96654	7.60663	6.73866
MAPRE3	Q9UPY8	6.39967	5.84066	6.46837	5.55316	6.41999	6.66127	5.6556	6.54137	6.38799	6.43754	6.39992	6.59367	6.10862	6.19546	5.76656	5.66016
ABCC10	Q5T3U5	6.56909	7.09866	6.29409	6.90104	6.23546	6.04704	6.03097	6.15278	6.53102	6.41441	6.50944	6.3541	5.64478	5.61534	5.76515	5.83663
ALG9	Q9H6U8-3	6.17557	6.35881	5.73501	6.43811	5.99195	6.19366	6.59281	6.29586	5.66607	5.72689	6.15461	5.56141	6.88135	6.43833	6.87112	6.91844
CDC6	Q99741	7.70104	6.85201	7.37645	7.41411	6.1591	6.6922	6.87485	6.39683	6.89952	6.6292	7.5965	7.27852	3.93084	4.08558	3.82022	4.29304
IBA57	Q5T440	6.60243	6.42875	6.02841	6.76675	6.3019	6.27713	6.11694	6.17135	6.01448	6.10102	6.17166	5.97468	6.19833	5.92957	6.50888	6.40773
CLCN3	P51790-2	6.34589	6.48721	6.41297	6.48786	6.4029	5.97099	6.15585	6.11097	6.06795	6.72355	6.37291	6.22014	6.21978	6.06949	6.08612	5.86544
MAX	P61244	6.6152	6.33715	5.72615	6.18091	6.64718	5.89098	6.16374	6.78594	6.282	6.09369	6.47613	6.20188	6.5835	5.89093	5.89498	6.22965
PPM1D	O15297	6.37952	6.61045	5.90644	6.76367	6.00946	6.36584	6.54877	6.1011	5.98714	5.43593	6.03754	6.30265	6.23064	6.37476	6.14634	6.79975
UBE2G1	P62253	5.84866	5.56433	6.62947	4.80582	6.48551	5.71446	5.83301	7.23806	6.03375	7.00318	6.44098	7.31601	5.77959	6.74678	6.339	6.22141
KANSL1	Q7Z3B3	6.59574	6.58967	6.11543	6.9778	6.53036	6.14855	6.95747	5.58272	6.29223	6.28973	6.3582	6.12694	5.80377	6.06415	5.7307	5.83654
ZFAND1	Q8TCF1	6.37278	5.52778	7.31174	5.95632	5.84808	6.55236	5.85114	6.38787	6.40239	6.62626	7.00725	6.83571	5.99713	5.9653	5.48101	5.87689
KAT5	Q92993-3	6.16605	6.35021	6.37174	6.97648	5.82822	6.16389	6.19667	6.23403	6.36151	6.15918	6.07608	6.33443	6.37429	5.75685	6.41656	6.23378
ACOT11	Q8WXI4	6.30321	6.50789	6.08477	6.32316	6.32132	6.73351	5.81257	7.07438	6.46843	6.54069	6.33953	6.93725	5.29109	6.11868	5.79752	5.346
FBXL4	Q9UKA2	6.04986	6.79284	6.37309	6.92788	6.46536	5.99965	6.32043	6.02415	6.63429	6.18642	7.08338	6.87442	5.29513	5.41815	5.7321	5.82285
GYS1	P13807	6.21663	6.3294	6.48131	5.86161	6.51442	6.68626	6.30486	6.65355	6.2372	6.93819	6.41309	6.41056	5.75458	6.06869	5.5624	5.56723
GABARAPL2	P60520	6.38202	5.71024	6.2749	5.78457	6.42618	6.84295	6.27821	7.13048	6.53358	6.36582	6.72944	6.66416	5.75703	6.02021	5.36531	5.73489
LPIN1	Q14693-7	5.97338	6.22595	6.76977	6.36594	5.58297	6.17847	5.77869	6.24811	6.33029	6.85912	6.75234	6.65755	6.06242	6.13126	6.16348	5.92026
NSMCE1	Q8WV22	6.0066	6.16039	6.21519	6.38811	6.0588	6.25957	5.97425	5.8253	6.53615	6.22884	6.66125	6.54729	6.29055	6.00486	6.61747	6.22537
COPZ1	P61923-4	6.65988	5.69174	5.92619	5.39614	6.44082	6.62408	6.46912	6.6356	5.43348	6.12677	6.65104	7.07726	5.4771	7.44391	5.82289	6.12399
ARL1	P40616	6.82669	6.42747	5.92247	7.01239	6.55054	6.44782	6.55664	6.14856	6.26777	6.42666	6.22082	6.06984	5.6824	5.99749	5.96056	5.48189
SDR39U1	Q9NRG7	6.56019	6.74783	5.92449	6.39103	6.60857	5.70923	6.11763	5.82021	5.93263	6.3508	6.32646	6.12122	7.06976	5.93941	6.11033	6.27022
ICA1	Q05084	6.57327	6.53316	6.96817	6.09444	6.15355	7.42473	5.977	6.70822	6.22088	6.41962	6.47116	6.58864	5.5891	5.50638	5.23765	5.53404
TSPAN6	O43657	6.12079	6.5059	5.84084	6.04228	6.10902	6.21447	6.01573	6.71244	6.61923	6.25775	6.20487	6.19896	5.96646	6.37447	6.37644	6.44035
SMAD2	Q15796	6.62018	6.56531	6.65727	5.79629	6.60363	7.12376	6.00336	6.76407	6.38113	5.98544	6.39759	5.90981	5.81772	6.22589	5.65172	5.49684
XPR1	Q9UBH6	6.78805	6.30177	6.0275	6.68816	6.11864	6.20044	6.60741	6.51656	6.69664	5.66847	5.8234	5.94947	5.72941	6.21167	6.60409	6.26832
SLC25A33	Q9BSK2	5.6484	6.33543	5.48306	6.77418	6.41243	6.83541	6.89618	6.17889	5.98322	6.54756	6.48648	5.79018	6.65425	5.79116	6.54668	6.53649
FAHD2B	Q6P2I3	5.63115	5.76411	5.62921	5.57992	6.43747	5.94542	6.34061	6.79302	6.30149	6.67914	6.55373	6.79759	6.45239	6.10013	6.50211	6.4925
SH3BGRL3	Q9H299	6.05364	4.94692	6.00997	4.69924	6.20091	6.30658	5.43132	7.32112	6.20556	6.74826	6.01348	6.83086	5.9278	7.34751	6.47916	7.47767
ESRRA	P11474	6.35756	6.24231	6.46541	6.87922	6.37861	6.35651	6.36635	6.03636	6.27933	5.84369	6.50555	6.23402	6.03236	5.72056	6.21772	6.08444
SPINDOC	Q9BUA3	6.63142	6.8331	6.44487	6.68713	6.70087	6.80569	5.63405	6.05282	6.06555	6.19682	6.26107	6.30723	6.24663	5.71858	5.55456	5.85962
SPIRE1	Q08AE8-2	6.24382	6.24019	6.43366	6.74264	5.79765	6.73127	5.1292	5.98234	6.32534	6.20995	6.81646	6.28058	6.7785	6.13522	6.0297	6.12348
RBMXL3	Q8N7X1	6.35815	6.2905	6.09985	6.7849	5.86302	6.13405	6.36295	5.95452	6.10805	5.9869	6.26312	6.23961	6.26835	6.22183	6.49782	6.56639
ATG3	Q9NT62	6.5555	6.48679	7.03607	5.81476	6.69888	6.60646	6.11225	7.31666	6.76106	6.16088	6.87618	6.47029	6.1578	5.55246	4.58054	4.81342
AREL1	O15033	6.42657	6.49543	6.25891	6.72582	6.31558	6.09228	6.113	5.08583	6.19698	6.35529	6.49222	5.99948	6.26349	6.03351	6.71677	6.42886

ACAD11	Q709F0	6.28981	6.3148	6.07092	6.70352	6.22998	6.20239	6.14923	5.87408	6.05937	6.10493	5.96175	5.75062	6.84712	6.25666	6.54481	6.64
CETN3	O15182	5.93845	7.19161	5.88643	6.74053	6.28299	6.80709	5.53556	7.37745	5.88205	6.9484	5.47638	7.09024	5.42012	6.14584	4.87754	6.39932
ATPAF1	Q5TC12	6.52692	6.17932	6.11094	6.59326	6.73658	6.76327	6.71469	6.41988	6.59742	6.03257	6.03887	6.63651	5.52565	5.2762	6.04723	5.80068
KIF1B	O60333-3	6.57991	6.50206	6.71549	6.16355	6.00596	6.33922	6.76459	6.79688	6.68142	6.44694	6.51097	6.33366	5.1712	5.51951	5.7828	5.68584
TAPBP	O15533-3	6.35935	6.89784	7.22973	6.66469	6.904	6.3002	5.6777	5.65486	6.17772	6.40274	5.5716	5.45358	6.7625	5.64071	6.24576	6.057
ACTR5	Q9H9F9	6.21137	6.3998	6.41312	6.66491	6.3993	6.20813	6.09794	6.32401	5.91373	6.26567	6.34356	6.40102	6.31631	6.20065	5.97792	5.86255
MTFR1	Q15390	6.9434	7.16309	7.00494	7.56509	6.89326	5.62008	6.52186	6.34475	6.80368	6.24976	6.9218	6.73283	4.37873	4.86239	4.86849	5.12581
KLHL15	Q96M94	7.24201	6.91492	7.18123	7.67278	6.11302	6.06075	6.71645	5.76095	7.00595	6.10396	6.39874	6.27921	4.76686	5.08313	5.20874	5.4913
DCTN3	O75935	6.25879	5.48474	6.34561	4.81254	6.38966	6.22581	6.11841	6.7335	5.43131	6.80173	6.06147	7.49328	5.6138	6.91978	6.50271	6.80689
ACBD6	Q9BR61	6.63717	6.34275	6.9618	5.96002	6.67333	6.56769	6.14018	6.88934	6.49645	6.51212	6.6345	7.09436	5.89272	5.14772	4.86004	5.18982
FAM20B	O75063	6.51852	6.40323	5.65808	7.26289	6.27801	6.15145	6.10816	6.16361	6.06066	6.54303	5.85345	5.66839	6.1479	5.98858	6.68462	6.50943
CDC34	P49427	6.745	6.16878	7.22988	6.09314	6.60128	6.48206	6.54336	6.85036	7.07306	6.50922	7.21981	6.76277	5.13024	5.00298	4.68275	4.90532
CDIPT	O14735	6.20799	6.67265	5.99148	6.49276	6.13084	6.00875	6.01279	6.09088	5.67196	5.97185	6.00279	6.10502	6.38722	6.48045	7.14627	6.6263
UVSSA	Q2YD98	6.15166	6.02631	6.77693	6.66653	5.63953	6.23897	6.10572	5.57785	5.72458	6.51275	6.80557	6.35377	6.47356	6.42978	6.56533	5.95116
NT5C	Q8TCD5	5.0907	5.37899	5.82573	5.56088	6.2522	6.35322	6.64635	7.23947	6.86904	7.03221	6.60386	7.00885	6.03592	6.02838	5.83561	6.2386
P3H4	Q92791	6.73952	6.48982	5.98089	6.64916	6.83055	6.3174	5.73565	6.66954	5.77807	6.54505	6.24251	6.1627	6.04482	5.70717	5.94364	6.16352
ATXN1	P54253	6.16528	6.25794	5.96383	6.10778	5.97256	5.8794	6.26767	5.75968	6.21811	6.40085	6.07886	6.14043	7.00994	6.34266	6.59765	6.83735
TIGD1	Q96MW7	6.56397	6.64889	5.96048	6.68968	6.85139	6.04443	6.70285	6.46771	6.33159	6.00818	6.27218	6.44143	5.82109	5.46177	5.61603	6.11832
IFFO2	Q5TF58	5.8108	6.40375	6.79689	6.6903	5.98948	5.93788	6.36814	6.11011	7.93482	6.54325	7.36601	7.16125	6.41727	4.60015	4.95441	4.91548
ENDOG	Q14249	5.98011	6.16226	6.09183	6.61578	6.6349	6.07841	6.44717	5.9446	6.2107	6.35659	6.07342	6.70721	6.42384	5.81376	6.38987	6.06757
WRAP53	Q9BUR4	5.92007	6.28893	6.55355	5.71683	6.37086	6.3257	6.47703	6.45531	6.27476	6.64957	6.88826	6.39341	5.91869	5.63154	5.77062	6.36486
MICB	Q29980	6.0658	6.52587	6.26539	6.61319	6.12164	6.55202	6.07937	6.41622	5.73166	6.12935	6.30755	6.19514	6.36486	5.97163	6.22271	6.70761
ORC5	O43913	6.23971	6.55041	6.17718	6.36567	6.34218	6.19569	6.03847	5.93668	5.93586	5.98327	6.31356	5.9416	6.63696	6.43362	6.53351	6.37563
ARVCF	O00192	6.51072	6.61556	6.40537	7.05692	6.21983	6.2706	6.26586	6.19708	6.1641	6.16875	6.46531	6.31117	5.85258	5.81437	5.79957	5.88219
DUSP3	P51452	6.24223	5.90196	6.9391	5.9526	7.02419	6.97444	6.14125	6.7431	6.39659	6.30984	6.75298	6.22305	5.33248	5.91054	5.70451	5.45115
PHF1	O43189	6.07494	6.73069	6.5734	6.87772	6.13512	6.39491	6.57601	5.6057	5.8428	7.03918	6.61836	5.76036	6.01166	6.00067	6.37372	5.38475
MCAM	P43121	6.26441	6.85634	6.32079	6.76892	6.08402	6.13324	5.95465	5.76825	6.03692	5.88356	6.18678	6.34903	6.47954	6.34168	6.31091	6.26096
BRMS1	Q9HCU9	6.38296	6.32384	6.16989	6.59241	6.20018	6.06686	6.2427	6.04495	6.28485	6.11719	6.04718	6.06714	6.48548	6.27348	6.25706	6.44381
NCK2	O43639	6.60416	6.36213	6.63679	6.62571	6.13408	6.60117	6.53189	6.16435	6.41942	6.39622	6.54101	6.40865	5.97543	5.64135	5.50219	5.45546
PAIP1	Q9H074	6.26761	5.67445	6.74927	6.07649	6.44276	6.68295	6.24364	6.40077	6.41973	6.41315	6.71467	6.79693	6.10492	5.96891	5.41052	5.63322
ADPGK	Q9BRR6	6.22821	6.26413	5.93162	6.53987	6.15428	6.11962	6.48457	5.99405	6.27108	5.65204	5.92366	5.87068	6.68944	6.34637	6.61632	6.91406
SLC9A3R2	Q15599	6.49943	6.53761	6.06867	6.32238	6.60865	5.94308	5.87149	6.27345	6.28768	5.90475	6.09509	6.475	7.07131	5.80777	6.12684	6.1068
MEGF8	Q7Z7M0	6.78411	6.4155	6.42593	6.57665	6.11466	6.14732	6.4542	5.90548	6.03422	6.93337	6.64005	6.95601	5.44602	5.71828	5.73669	5.71152
ZSWIM8	A7E2V4-4	6.94251	6.61017	6.92233	6.42092	6.46646	6.9082	5.31478	6.65935	6.81477	6.82921	6.37093	6.42503	5.1448	5.46288	5.52	5.18767
MTG2	Q9H4K7	7.24655	7.19937	6.41819	6.96262	6.37562	6.41567	6.47068	5.96555	6.27747	6.49862	6.29024	6.47299	5.73921	4.85523	5.43673	5.37525
CTBS	Q01459	6.50445	6.6403	5.72668	6.4736	6.45828	6.44807	6.07277	6.29777	6.08859	6.18887	6.3632	6.23082	6.24593	6.07646	6.01601	6.16818
PDXK	O00764	6.398	6.43796	7.18528	5.86171	6.2829	7.89009	6.01638	7.11274	6.47126	6.27519	6.65207	6.31414	5.2094	5.52896	5.80231	4.56161
RIPK2	O43353	6.42345	5.93783	6.77769	5.90367	6.47388	6.06795	6.56569	6.30088	6.32511	6.26918	6.68326	6.61466	5.19743	6.34632	5.84829	6.26471
NSA2	O95478	6.83551	5.98092	6.05828	7.32948	6.45229	6.84026	6.43447	6.15339	6.84595	5.88168	6.38565	6.20347	5.23562	6.02465	5.64864	5.68977
MAP3K6	O95382	6.19878	6.63375	6.93346	6.37756	6.06565	6.58652	6.0945	6.65669	6.21617	6.92086	6.77966	6.5333	5.56764	5.74941	5.40247	5.28358
MAP3K5	Q99683	6.28873	5.56452	6.9227	6.53181	6.03463	6.84724	6.56065	6.24319	7.09283	6.1684	6.78352	7.08147	5.70934	5.64102	4.91584	5.6141
PDF	Q9HBH1	6.48961	6.26855	6.23728	6.69015	6.03306	5.76297	6.08024	6.14553	6.13691	6.21188	6.21509	6.28224	6.71695	5.93793	6.53071	6.2609
IFIT5	Q13325	6.44278	6.43022	7.17306	6.31124	6.34357	6.73832	6.22252	6.90586	6.15798	6.09155	6.56895	6.38019	5.56416	5.70811	5.33781	5.62369
SIK2	Q9H0K1	6.30153	6.58011	7.28554	5.92553	5.85954	7.32983	6.33643	6.78413	6.63935	6.78145	6.9329	6.52227	5.27882	5.54755	5.02583	5.26586
PDLIM3	Q53GG5-2	6.68052	6.15072	6.35833	6.23231	6.58815	6.31131	5.90716	6.28056	6.27551	6.25	6.60922	6.99719	5.91481	5.96168	5.46634	6.01617
HINT2	Q9BX68	5.13811	4.93828	4.34893	4.52743	5.58967	4.69285	5.54387	7.39315	5.14589	5.67443	4.80369	5.26352	4.85889	10.6859	9.27122	12.1242
UBQLN1	Q9UMX0	6.09893	4.46305	4.86201	4.26933	4.87342	3.7979	5.39863	6.63573	4.35711	5.24904	4.77465	6.9702	5.50572	11.1592	9.87682	11.7083
STARD7	Q9NQZ5	6.39005	5.8788	6.05695	6.48406	6.5756	6.5946	6.27543	6.1443	5.96476	6.27075	6.25603	6.75728	6.43282	6.47228	5.47837	5.96791
TM7SF2	O76062	5.85235	5.91032	6.13598	6.6277	5.83878	5.67642	6.15677	5.55032	6.09761	6.06306	5.79218	6.06693	7.01598	6.86796	7.10746	7.24018
PRMT7	Q9NVM4	6.61929	5.93723	7.41864	5.86799	6.28487	7.11643	6.15715	6.57328	6.60676	5.88634	6.92236	7.65172	5.43898	4.83973	5.41961	5.25964
MRPS28	Q9Y2Q9	6.34909	6.26762	6.25152	6.82671	6.27057	6.00579	6.29757	6.00607	6.04916	6.47878	6.10422	6.30025	6.55615	6.03382	6.13636	6.06633
PINX1	Q96BK5	6.52535	6.57709	6.0199	6.95929	6.31304	6.44508	6.44581	5.93516	5.9478	5.40826	6.187	6.56324	5.69591	6.41489	6.09774	6.46443
GLIPR2	Q9H4G4	6.15381	6.51016	6.2195	6.4898	6.30158	6.06707	6.31991	5.9483	5.91832	6.25873	6.07914	6.17911	6.7357	6.33568	6.58873	5.89445
CASP7	P55210-3	6.15631	6.43084	6.74318	5.87061	6.3213	6.22601	5.82069	6.7991	5.90567	6.48703	6.93953	6.7651	6.166	5.85921	5.61642	5.89301
NEPRO	Q6NW34	6.50589	6.75058	6.38929	7.03161	6.01276	6.11798	6.51519	5.95666	6.25296	5.91937	6.19509	5.9548	5.59008	5.73797	6.43432	6.63544
RUNX1	Q01196-8	5.87718	6.52679	6.11707	6.36763	6.1531	5.95066	6.04648	6.05553	5.86581	6.07221	6.20002	6.23412	6.91661	6.39919	6.57328	6.64432
PTDSS2	Q9BVG9	6.33151	6.0562	6.5223	7.11	5.76535	6.56345	6.02478	6.09092	6.07869	6.27309	5.60688	6.16095	6.49803	6.38887	6.36702	6.16197
ZNF280D	Q6N043	6.3682	6.86981	6.14305	7.22087	6.15623	6.1543	6.47551	5.65303	6.28902	6.30468	5.9461	5.964	6.38161	5.93937	5.96965	6.16459
MEF2D	Q14814-4	5.79966	6.04825	6.08642	6.0325	5.963	6.29421	5.91011	5.73804	5.97875	6.34564	6.56836	6.32503	6.76086	6.80485	6.70031	6.64402
R3HDM1	Q15032-2	6.67794	6.34282	6.77105	6.42358	6.14573	6.82563	6.0445	5.93191	6.57066	6.62105	6.36127	7.14129	5.94913	5.30472	5.20865	5.57516
MTM1	Q13496	6.10727	6.54002	6.44116	6.04615	6.18555	6.55091	6.5234	6.19291	6.17234	6.43208	6.31228	6.23645	5.9528	6.21842	6.21412	5.87415
TAF3	Q5VWG9	6.44878	6.50038	6.12769	6.69494	5.64755	5.65659	6.00234	5.76559	5.99164	5.87808	6.2332	5.96943	6.1701	6.8865	6.93434	7.09283
CEP44	Q9C0F1	5.81521	6.03353	7.01503	5.97862	6.01906	6.58508	5.77292	6.15665	6.23481	6.79774	7.08828	7.10217	5.64067	6.08602	5.98703	5.68717
ZFYVE19	Q96K21	5.81731	6.11468	6.87745	5.88794	5.8482	7.05172	6.01351	6.21327	5.96628	7.10999	6.65295	6.82579	5.52496	6.13338	5.72575	6.23681
TPD52L1	Q16890	6.50156	6.09116	7.04427	5.73322	6.37562	6.82481	5.89913	6.70684	6.04571	6.31659	6.50972	6.65955	6.20562	5.71172	5.80962	5.56486
SYTL4	Q96C24	6.8371	7.02709	6.44643	6.69966	5.4552	6.40084	6.0563	5.56551	6.18758	6.33957	6.08181	6.62953	6.40954	6.4239	6.23181	5.20814
NDE1	Q9NXR1-1	6.73105	6.46411	6.41059	5.99584	6.70363	6.31636	6.0509	6.77694	6.1561	6.71851	6.54613	6.55985	5.96268	5.65293	5.33264	5.62174
ARFGAP3	Q9NP61	6.28525	6.03692	6.50386	6.1462	6.59055	6.20694	6.35615	6.5343	6.50986	6.60752	6.16531	6.59057	5.94797	5.96731	5.64452	5.90675
DCAF16	Q9NXF7	5.48021	5.93496	6.06779	5.67441	6.06752	6.09351	5.79147	6.59321	5.79886	6.10077	6.41556	6.39645	6.88863	7.35648	6.51435	6.82581
GLCCI1	Q86VQ1	6.63057	5.81432	7.33275	6.17093	6.10994	7.34127	6.06479	6.0275	6.57118	6.94449	7.00226	6.83616	5.8759	5.16285	4.75525	5.3598
SIX4	Q9UIU6	5.91462	6.35729	5.97716	5.81577	6.23476	6.14958	6.00154	6.4435	6.30785	6.1761	6.83527	6.55155	6.4837	6.41358	5.806	6.53174
KIAA1549L	Q6ZVL6	6.14424	6.50713	6.3065	6.66699	5.97592	6.3783	6.25378	5.79894	6.07645	5.65699	6.48614	6.47628	6.46662	5.89314	6.19143	6.72115
RETREG2	Q8NC44	5.94163	5.88896	5.4908	5.76693	5.95225	5.5576	5.6544	5.94219	5.97543	6.07909	5.56259	6.03723	7.77735	7.21836	7.83844	7.31675
POLH	Q9Y253	6.3467	6.75443	6.46461	6.70098	6.07485	6.44987	5.91323	5.97439	6.37006	6.57843	7.02391	6.86016	5.43659	5.5302	5.78963	5.75225
REEP5	Q00765	6.1456	6.19337	6.07762	5.89193	6.36578	6.26854	6.36212	6.14613	6.18453	6.34115	6.03457	6.27667	6.80926	6.29927	6.50992	6.09354
TBPL1	P62380	5.26	6.04917	6.23041	5.56943	5.26626	6.16578	5.64362	5.83093	5.78635	6.36703	6.11374	6.11727	6.63534	7.81989	7.77301	7.37176
SETD7	Q8WTS6	6.08253	5.69863	6.52927	5.80467	6.39225	6.36053	6.18398	6.76024	5.94862	6.62325	6.61793	6.74679	6.26966	6.08086	5.72286	6.23194
DNTTIP1	Q9H147	6.36048	6.26326	6.36966	6.77606	6.14497	6.05351	6.34355	6.12011	6.29089	6.30662	6.3657	6.44395	6.09978	5.91728	6.19713	5.94705
SMURF1	Q9HCE7	6.80879	5.90706	6.25561	6.22964	5.87051	6.66687	6.10621	6.65407	6.21134	6.63427	6.9707	6.73798	5.49197	5.80339	5.56336	6.08823
ST7	Q9NRC1	6.68354	6.52158	6.07452	6.71675	6.16139	5.88601	6.28	5.72726	6.29149	5.99117	6.48679	6.08598	6.29742	6.03912	6.50272	6.25426
GALM	Q96C23	5.58695	5.58817	6.34096	5.77118	6.63465	6.86416	5.97491	6.57245	6.20595	6.72947	6.80119	6.14277	6.14097	6.47655	6.00646	6.06421
DCP2	Q8IU60	5.27008	5.87588	7.74035	6.23335	5.8522	6.11331	5.52171	6.23483	6.83697	6.47771	6.71515	6.56685	5.85797	5.92582	6.32127	6.45654
SLC30A5	Q8TAD4	6.34499	6.29867	5.92333	6.43791	6.2946	6.01122	6.37231	5.84189	6.44071	5.70188	5.84095	5.80417	6.74156	6.557	6.66133	6.72749
GMPPA	Q96IJ6-2	6.224	6.00854	6.75851	6.01626	6.07821	6.59402	6.1075	6.55894	6.42901	6.5859	6.4696	6.51229	6.13581	5.98135	5.78756	5.75251
FANCC	Q00597	6.40998	6.01821	6.7723	6.19644	6.51372	6.56949	6.32923	6.96922	6.65898	6.75894	6.88505	7.06466	5.16214	5.12008	5.04765	5.52391
JRK	O75564-2	6.01238	6.07853	5.79896	6.30483	5.942	6.20522	6.36437	6.44046	6.17002	6.38327	5.98577	6.16914	6.46235	6.38045	6.52578	6.77647
ERO1B	Q86YB8	6.36178	6.22536	6.03485	6.63776	6.16847	6.00577	6.49366	5.90847	5.83204	6.10687	6.24921	6.06157	6.62711	6.25296	6.63049	6.40362
STX6	O43752	6.37495	6.71177	5.96031	6.52464	6.02845	6.5907	6.36967	6.18403	6.23738	6.24173	6.51171	6.18788	5.72866	5.99295	6.24422	6.11106
PPP3CB	P16298-4	6.20705	6.21054	6.79796	5.92779	6.23201	6.33225	5.90914	6.13174	6.16801	6.56423	6.52046	6.4189	6.27298	6.0567	5.93764	6.3126
NOVA2	Q9UNW9	6.14293	6.3407	6.24692	6.03762	6.4628	5.93851	6.065	6.20599	5.96446	6.06194	6.29554	6.05395	6.7818	6.32904	6.68012	6.39266
ZBTB47	Q9UFB7	6.16025	6.58339	6.36579	6.94401	5.972	6.22967	5.90646	5.66027	6.19333	6.63989	6.4288	6.26541	6.18489	6.07469	6.31402	6.07713
WDR73	Q6P4I2	5.91784	5.82642	6.39414	5.66687	6.54887	6.81049	5.77456	7.00554	6.98057	6.28859	6.47431	6.80741	5.40887	5.7822	6.36189	5.95141
SRFBP1	Q8NEF9	5.99751	5.95777	5.69551	6.59007	5.82042	5.62404	6.21795	5.7876	6.14772	5.80556	6.16988	5.82534	7.05888	6.62149	7.20467	7.47557
AXIN1	O15169	6.8216	6.99677	6.85247	6.66526	6.19091	6.31734	6.55639	6.42707	6.50034	6.35609	6.42454	6.60147	5.21178	5.22577	5.38445	5.46773
SPPL2A	Q8TCT8	6.76269	6.83346	6.43473	6.83919	6.37811	5.90616	6.51512	6.16531	5.94058	6.23703	6.66095	6.36757	5.83976	5.79593	5.60288	5.72054
GDAP2	Q9NXN4	6.02405	6.17151	6.40011	6.26266	6.12449	6.02024	6.13249	6.16751	6.28052	6.39216	6.38414	6.22094	6.77163	6.10577	6.2807	6.26108
DGKE	P52429	6.24716	6.2647	6.66225	6.87829	5.90671	5.86928	6.04246	5.76808	7.13444	5.97086	5.84876	6.22622	6.30222	6.00736	6.49986	6.37137
CTU2	Q2VPK5	6.64246	6.3428	7.02143	6.66972	6.79241	6.38036	6.15048	7.12203	6.23147	6.17567	6.41013	6.81195	5.43423	5.45914	4.9166	5.43913
RPP40	O75818	6.05927	6.39107	6.69836	6.46866	6.17619	6.36134	5.98348	6.01899	6.43378	6.19713	5.95984	6.19363	6.39155	6.1319	6.50178	6.03303
BBS1	Q8NFJ9-2	6.77881	6.09376	6.35223	5.85952	6.2626	6.75496	6.44693	6.84287	6.36742	6.43149	6.3072	6.38905	5.68061	6.25752	5.96871	5.20632
PI4K2B	Q8TCG2	6.68299	6.90783	6.64278	6.55729	6.52369	5.9978	6.48345	6.23868	6.44647	6.68856	6.74875	6.17613	5.33387	5.62038	5.2658	5.68553
NAXD	Q8IW45-2	5.83736	6.07209	6.54673	6.05594	6.65588	6.09493	6.19168	6.59321	6.34491	6.33269	6.30436	6.15052	6.259	6.06908	6.27257	6.21904
USP37	Q86T82	5.20544	6.02189	6.13713	5.71805	5.41122	5.85049	5.83261	5.76433	5.95311	6.35199	6.51673	6.51738	6.64246	7.44413	7.26749	7.36554
NDUFA8	P51970	6.70808	5.13557	6.10273	5.51903	7.26245	5.64958	5.96837	6.25617	5.92783	6.2305	6.29674	6.27398	7.31854	6.22738	6.34805	6.77499
IRF2	P14316	5.9423	6.1068	6.43295	6.85479	6.17898	6.2658	6.04528	5.62086	6.19598	6.59176	6.5036	6.46435	6.6813	6.12675	6.08719	5.90129

TMEM67	Q5HYA8	6.35705	6.69629	5.88032	6.58206	5.89109	6.26085	6.26676	6.37583	5.90545	5.92946	6.00675	6.15286	6.36413	6.54997	6.29702	6.48411
SIRT5	Q9NXA8	6.63055	6.59251	5.81618	6.3435	6.17639	6.41205	6.18534	6.15504	6.2822	6.25524	5.86636	6.20268	6.23467	6.30533	6.40787	6.13409
ZNF335	Q9H4Z2	6.26361	6.38093	6.39728	6.70438	6.08409	5.82102	6.258	6.04114	5.94016	6.42692	6.18732	6.06546	6.06436	6.00171	6.87139	6.49222
FAS	P25445	6.0968	6.54941	5.97967	6.60391	6.34007	6.3588	6.2683	5.86763	6.08519	6.10863	6.15119	6.06301	6.67351	6.09369	6.39912	6.36108
SDSL	Q96GA7	6.33189	6.06883	6.94485	5.92015	6.28892	6.72528	6.14123	6.23622	6.24999	6.56593	6.42523	6.36078	5.91874	6.00838	5.93629	5.8773
EP300	Q09472	5.55797	6.19107	6.31121	6.93692	6.05238	6.31339	6.36355	6.25338	5.90449	6.26826	6.69526	5.81438	6.49369	6.75209	6.13681	5.95514
SLC25A40	Q8TBP6	6.14928	6.25184	6.14361	6.77512	6.05211	6.01157	6.25966	5.84533	6.30009	6.07834	5.60743	5.86512	6.75976	6.67735	6.54602	6.67738
ZBTB1	Q9Y2K1	6.47736	6.92824	6.0828	6.54728	5.93978	6.12143	6.2286	6.0197	6.6914	6.6997	6.31918	6.47097	6.02266	5.22212	6.08094	6.14785
LDB1	Q86U70	6.35742	6.55491	6.30199	6.05682	5.8337	7.20722	6.10148	4.997	6.2508	6.71023	6.14347	6.29472	6.64523	6.59802	6.56623	5.38073
WDR45	Q9Y484-2	6.32925	6.59167	6.48373	6.48367	5.93104	6.10144	5.92221	6.33953	6.38359	6.35795	6.90543	6.94232	5.70888	5.75778	6.07703	5.68448
TRIM5	Q9C035	6.14798	6.03004	7.26923	6.35203	6.44203	6.81165	6.22843	6.24232	6.57861	6.25415	6.705	6.45933	5.83926	5.56007	5.31683	5.76305
CRELD2	Q6UXH1-5	5.73633	6.86698	6.04221	6.51687	6.06392	6.5517	6.11888	6.13847	5.84015	6.5684	5.72361	6.09728	6.62498	6.60187	6.11989	6.38848
MYPN	Q86TC9	5.97092	5.69459	7.03582	5.82348	6.17626	6.50379	6.40704	6.07938	6.45984	6.52348	6.77253	6.41301	6.27121	5.85043	5.88541	6.13282
THOC3	Q96J01	6.13219	6.18066	6.11391	6.20159	6.18035	6.57813	6.24941	5.68501	6.13261	6.80849	6.39381	6.65109	6.1136	5.9259	6.33133	6.32193
CRELD1	Q96HD1-2	6.21933	6.38997	5.79229	6.11365	6.19861	6.28313	6.40564	5.74695	5.95274	6.54132	5.92588	5.27105	7.01475	6.65003	6.94986	6.54481
TMEM9B	Q9NQ34	6.04377	7.1194	6.44712	6.35527	6.20669	6.41423	6.52021	6.2931	6.56388	6.16622	6.03717	6.22479	5.87737	5.91395	5.84136	5.97546
FNIP1	Q8TF40	7.61737	7.02369	7.50254	7.30288	6.55178	6.55622	6.42051	6.82159	7.34579	6.93132	7.19177	7.40703	4.02257	4.05316	3.26913	3.98267
PDZD8	Q8NEN9	6.45025	6.44125	5.79819	6.84253	5.98067	5.85244	6.45465	5.96966	5.777	6.41847	5.55727	6.45459	6.26282	6.55023	6.61119	6.57881
FSTL1	Q12841	6.81316	6.32413	5.67856	6.36614	7.1372	6.47858	6.76007	6.79075	5.81564	5.26621	6.13122	5.47897	7.05805	5.49814	5.83298	6.57019
USP12	O75317	6.21421	6.31981	6.08582	6.27048	5.99131	5.53703	6.30121	6.20576	6.23599	6.92191	6.29061	6.54753	6.36068	5.94835	6.51643	6.25286
FHL3	Q13643	6.56437	6.07468	6.54199	5.75791	5.97274	6.48874	5.87025	6.08803	6.31986	6.27911	6.43315	6.73763	6.16573	6.17624	6.28107	6.24849
ESCO1	Q5FWF5	6.05256	5.90211	6.03687	6.14103	5.82746	5.68626	6.28834	5.86479	5.63387	5.89639	5.95931	5.48121	6.71621	7.46665	7.64346	7.40347
RECK	O95980	5.60451	6.40351	5.72633	6.88676	6.46246	6.1509	6.46697	5.93319	6.09902	6.60675	6.19569	6.48522	6.34861	5.92047	6.21119	6.49843
NDRG2	Q9UN36	6.4376	5.80978	6.6388	5.15925	6.67185	6.17442	5.39788	6.87996	5.5894	7.14551	6.10689	7.04087	6.88619	6.55701	5.51991	5.98467
NUDT16L1	Q9BRJ7	6.34585	5.99648	5.95781	6.00897	6.50319	6.10161	6.37308	6.21707	6.17019	6.52638	6.36654	6.16977	6.43302	6.05722	6.43035	6.34246
MRPL20	Q9BYC9	6.09122	6.174	5.91112	6.82449	6.14506	6.11059	6.75015	5.85812	6.00743	6.03545	6.34275	6.05769	6.99427	5.91355	6.02996	6.75415
SMN1	Q16637	5.84779	6.27687	6.7149	6.83207	6.31477	6.37333	5.96833	6.00386	6.07967	5.83547	6.71207	6.69993	6.65087	5.96054	5.84929	5.88023
ARHGAP29	Q52LW3	6.22255	6.41593	7.05874	6.37495	6.1063	6.92131	6.0338	6.46934	6.4423	6.18543	6.96191	7.26604	5.77818	5.32519	5.19847	5.23954
SYNPO	Q8N3V7-2	6.31981	5.99981	5.73851	6.46589	6.45905	6.06321	6.20505	5.65598	6.20965	5.97311	5.66916	5.84943	7.59121	6.37248	6.69298	6.73468
SRSF5	Q13243	6.60876	6.47472	5.73684	5.84411	6.53893	6.16014	6.47851	6.28965	6.48786	6.34516	6.20323	5.91432	6.53317	6.10983	6.37558	5.89917
TADA2B	Q86TJ2	6.0489	6.57136	6.01601	6.91913	6.02312	5.96712	6.30141	6.1466	6.15478	6.35398	6.30622	6.57449	5.99617	5.95915	6.17683	6.48472
STIL	Q15468-2	5.92329	6.55312	6.57155	6.2424	6.19896	6.29928	6.56014	6.51795	6.92283	6.85832	6.9147	7.20335	5.1329	5.85746	4.8978	5.34595
GINS1	Q14691	6.12019	5.84976	6.43496	5.35927	5.89327	6.41397	5.93242	5.81859	6.21495	6.69036	6.42068	6.79072	6.00224	6.84014	6.58802	6.63046
PHF5A	Q7RTV0	6.57732	6.42231	6.27562	6.55707	6.098	6.08664	6.04212	5.89756	5.98469	6.32288	6.13984	5.96527	6.81908	6.08754	6.29802	6.42604
ZCCHC7	Q8N3Z6	5.92894	6.26837	6.19445	6.96051	5.81331	5.50449	6.52664	6.05836	5.58303	5.96534	6.27175	6.01081	6.53879	6.56824	7.10512	6.70184
ZC3H8	Q8N5P1	6.53699	6.1871	6.02324	6.27951	5.72525	5.73854	5.94872	5.46236	6.26001	6.21	6.26942	6.28689	6.50447	6.93023	6.84876	6.78848
NARF	Q9UHQ1-2	6.75836	6.93072	7.07908	6.16691	6.06992	6.41387	5.71278	5.95397	6.78479	7.29018	6.9631	7.02909	4.74863	5.89345	5.25517	4.94998
DPH5	Q9H2P9-5	6.16486	6.13086	7.39038	5.58682	6.16694	6.422	6.38832	7.06793	6.1865	6.46316	6.67847	7.16664	5.36641	5.60186	5.6077	5.61116
CCDC134	Q9H6E4	6.40119	6.17423	6.44122	6.32843	6.04321	6.41681	6.53077	5.92035	6.38579	6.32941	5.80776	5.83441	6.67165	5.94877	6.54081	6.2252
PTOV1	Q86YD1	6.40891	6.14831	6.4108	6.84612	6.12112	6.2854	6.09163	6.27334	6.00793	6.39873	6.76594	6.67148	6.25577	5.54748	5.88907	5.87797
TRIM23	P36406	6.23076	6.4181	6.3661	6.37096	6.12229	6.29791	6.45984	6.28199	6.60391	6.29802	6.46952	6.40953	6.52708	5.86431	6.07044	5.90926
TMEM161A	Q9NX61	6.43942	6.1752	5.68916	6.78645	6.13607	5.9725	6.26917	5.83902	6.22329	6.04512	5.91441	5.82473	6.9955	6.21898	6.53281	6.93818
USP42	Q9H9J4	6.29367	6.41545	5.97388	6.81154	6.08621	6.12614	6.14187	5.95227	6.7346	6.46451	6.23156	6.44416	5.83627	6.16118	5.93477	6.39191
NADK	O95544-2	6.41597	5.81112	7.38028	6.23224	5.83034	6.4443	6.47816	6.81296	6.62091	6.65171	6.7503	6.90882	5.49097	5.47824	5.25774	5.43594
RPAP2	Q8IXW5	6.4837	6.28779	6.78477	6.20808	6.07264	6.36147	6.80842	6.61464	6.6625	6.48119	6.64864	6.94152	5.444	5.27741	5.35599	5.56723
DOK1	Q99704	6.93597	5.61552	7.10915	5.79408	5.9205	5.86868	6.19019	6.4682	5.74804	6.25163	6.99323	6.72294	5.67073	6.86209	6.03756	5.81149
RIOK1	Q9BRS2	6.08643	5.92131	6.71019	6.56436	5.92383	6.5666	5.99248	6.40063	5.92042	6.24361	5.93263	6.06088	5.73943	6.51935	6.9124	6.50546
NDOR1	Q9UHB4-2	6.3324	5.81416	7.13627	6.39117	5.91765	6.63919	6.13951	6.37445	6.252	6.5295	7.2552	6.66125	5.66266	5.87441	5.66806	5.35214
STX3	Q13277	6.66093	6.70742	6.11	7.03131	6.17267	6.18414	6.26082	6.63795	6.01586	6.33513	6.51228	6.0824	5.59576	5.74473	6.20977	5.73882
ZMIZ2	Q8NF64	5.74093	5.53726	6.145	4.92551	5.62638	5.67718	5.71842	5.77161	6.38109	6.6351	5.99753	5.92636	7.16067	7.5392	7.6236	7.59416
UBE2G2	P60604	5.24974	5.28632	4.65551	6.54695	6.09291	5.01263	5.59442	6.75663	4.36992	5.30533	5.00421	5.24009	5.04413	11.1419	7.42542	11.2739
TMEM30A	Q9NV96	6.71301	6.74252	6.21822	6.88004	6.14562	6.25254	6.21505	6.13205	6.59308	6.70193	6.42621	6.44524	5.74293	5.55556	5.48448	5.75153
HSF1	Q00613	6.08938	6.36004	6.08581	5.4608	6.15241	6.49042	5.5764	6.57648	6.01217	6.48375	6.36095	6.6098	6.20764	6.73308	6.44489	6.35599
SIRT7	Q9NRC8	6.13001	6.77976	5.71369	6.58755	5.46261	6.41	6.28789	5.82523	5.94813	6.23821	6.35956	6.46576	6.29787	6.21227	6.73448	6.54699
TAF1A	Q15573	5.8699	6.25283	6.13314	6.47449	6.10855	6.21667	6.04779	5.657	6.01581	6.03371	6.08788	6.20887	6.68511	6.15591	7.03198	7.02035
RABGGTB	P53611	6.42454	5.40009	6.86247	5.51123	6.16915	6.83019	5.90198	6.79817	5.93658	6.42567	6.56637	7.06836	5.11063	6.61732	5.96449	6.41275
UBE2I	P63279	5.52515	4.66109	6.09172	5.00578	6.27851	5.40396	5.41256	6.84832	5.65013	6.75758	6.22447	6.98272	6.83165	7.72364	7.34359	7.25912
FAM50B	Q9Y247	5.73806	6.70972	6.51602	6.07656	6.32042	6.27687	6.12696	5.98189	6.55267	6.76906	6.8019	6.39273	5.83317	6.19054	6.00185	5.81146
FAM83G	A6ND36	5.72677	5.68628	6.42925	5.63519	6.04924	5.83441	6.19811	5.81928	6.51961	5.93925	6.35215	6.2662	7.26578	5.95387	7.36297	6.96163
PPIL1	Q9Y3C6	6.17816	5.66556	5.88073	5.37096	6.16991	6.20664	5.65745	6.80565	5.57417	6.47542	5.90246	6.58054	6.82634	7.23414	6.88512	6.58976
ATG4C	Q96DT6	6.27837	6.02818	6.66133	5.59239	5.27208	6.82925	6.12706	5.68705	7.01017	7.18907	6.36386	6.59388	6.18568	6.26251	6.03204	5.88708
APOB	P04114	6.27791	7.22909	6.55147	7.26506	5.78957	5.56498	6.28761	6.27759	6.97883	6.55235	6.08865	6.96015	5.25439	5.32552	5.98034	5.6165
STX12	Q86Y82	6.37014	6.23145	6.56771	6.23163	6.21504	6.57854	6.35127	6.02349	6.27491	6.22284	6.7235	6.50814	6.03854	5.97367	6.08919	5.59993
ZBTB3	Q9H5J0	6.70768	5.81718	5.26092	5.96996	6.7829	6.11297	5.54105	6.59384	6.51103	6.44287	6.51021	6.25739	7.00233	6.00302	6.49768	5.98898
MYORG	Q6NSJ0	6.41663	6.60928	6.06604	6.88309	6.36322	5.80903	6.51732	5.8571	6.19818	6.99855	6.35657	5.94484	6.2063	6.0541	6.13617	5.5836
HDHD2	Q9H0R4	6.2605	5.83038	7.38584	5.76973	6.77776	7.75532	6.51028	6.29712	6.09588	6.3898	6.51569	6.41556	5.43302	6.02264	5.52745	5.01302
CASP2	P42575	6.51469	6.00714	6.90711	5.76802	5.41664	6.20403	6.04437	6.54345	6.33313	6.31684	6.8504	6.92379	5.30836	6.42859	6.74017	5.69328
CCDC9	A0A2R8Y4Z8	6.49159	6.26927	6.66817	6.14567	6.32942	6.19137	6.49168	6.69538	6.52946	5.92214	6.38265	6.47563	6.00669	5.93488	5.85834	5.60765
COA1	Q9GZY4	6.30454	6.15193	5.69957	6.45688	6.29286	5.78738	6.43763	5.96788	6.29713	5.98919	5.96593	6.09947	6.90309	6.31744	6.70511	6.61398
MICU2	Q8IYU8	6.14765	6.24417	6.21929	7.34158	6.45523	5.95274	6.5381	5.81028	6.12977	6.07147	5.7426	5.92673	6.44282	5.77178	6.70351	6.50229
PRKAB2	O43741	5.33801	5.51898	5.73434	5.32259	6.09627	6.56014	5.76429	6.15283	6.26448	6.27727	6.82013	6.75574	7.08677	6.92407	6.9682	6.41588
RHOA	P61586	5.81564	6.38184	5.78064	6.32207	6.95334	7.67496	6.78054	6.57574	6.46581	5.71537	6.30304	5.87111	6.04151	6.34913	5.63404	5.33521
RHOB	P62745	6.3676	6.37775	6.98672	6.77175	6.40995	6.34721	6.33126	5.95802	6.147	6.18057	6.03972	6.41828	5.98403	5.59323	5.87699	6.20991
DLGAP4	Q9Y2H0	6.1551	6.11494	6.57008	5.97275	6.87551	6.84962	6.37149	6.86292	6.77949	6.46407	6.61274	6.16425	5.63113	5.55126	5.31166	5.713
FBXO2	Q9UK22	6.30328	5.97619	6.69142	5.8243	6.49429	6.93081	6.08159	7.45816	6.41528	6.10506	7.00254	5.99117	5.99249	5.8874	5.48673	5.3593
SNAP29	O95721	6.2132	5.70058	6.79942	5.68413	6.43841	6.5095	5.91901	6.57609	6.57994	6.89993	6.93096	7.25486	6.18722	5.45613	5.36403	5.48658
SCAI	Q8N9R8-2	6.33729	6.3584	5.94069	6.8384	6.58256	5.97229	6.61515	5.94178	6.20166	6.16118	6.48513	6.02477	6.21161	6.17936	6.109	6.04072
SMYD4	Q8IYR2	5.71262	6.50278	6.97893	6.41704	6.35962	7.04738	6.04523	6.3201	6.54303	6.59288	6.48745	6.3639	5.88359	5.77386	5.24884	5.72274
BMPR2	Q13873	6.70802	6.76965	6.59232	7.11025	5.79811	6.27735	6.47793	6.43696	6.24726	6.88661	6.75356	7.08302	5.38694	5.43805	5.02426	5.00969
UBE2M	P61081	6.07631	5.60837	6.74648	5.44985	6.14887	6.35434	6.02101	6.23001	6.7407	6.4494	6.97817	6.43241	6.30348	5.92258	6.38542	6.1526
ANXA3	P12429	6.16644	6.12338	6.6631	5.95302	6.23517	6.22538	6.07775	6.67324	6.94841	7.07901	6.28701	6.23001	6.01509	5.69773	6.09017	5.53509
DIXDC1	Q155Q3	6.72622	6.39231	6.12181	6.91003	6.2964	6.23084	6.19802	6.52933	6.44465	6.06602	6.24708	6.78197	6.31721	5.80016	5.45175	6.3862
ABI2	Q9NYB9	6.65706	6.08528	6.74371	5.74224	6.89899	6.47539	6.13291	6.59172	6.31836	6.43861	6.23226	6.23386	6.04671	5.75923	5.77628	5.86738
PAFAH1B3	Q15102	5.60063	3.86664	6.71439	4.81175	5.07562	4.89796	5.31768	6.14933	5.2293	6.19426	5.76913	6.64197	5.82033	9.7077	8.75863	9.44467
LYPLA2	O95372	5.43236	5.31265	4.89681	4.57687	5.45973	5.48043	5.4243	7.04825	4.84787	5.9159	5.09476	5.49184	4.72329	11.8259	7.59436	10.8747
GRK6	P43250-2	7.17265	7.53129	7.16296	7.61383	6.45874	5.68826	6.03272	6.46168	6.57834	6.94089	7.10442	6.56944	4.3095	5.06604	4.76408	4.54516
AIFM2	Q9BRQ8	5.85871	5.75935	5.80764	6.37146	6.03712	5.87329	6.45713	6.26078	6.2671	6.5325	6.13527	6.22432	6.24749	6.70782	6.70946	6.75055
POLR2C	P19387	6.72671	6.19017	6.4514	6.23008	6.9358	6.25998	6.73859	6.41892	6.66053	6.37745	6.73371	6.58208	5.68054	5.23897	5.51286	5.19001
CLUAP1	Q96AJ1	6.25967	5.94864	7.0405	6.16252	6.12457	6.55817	6.20002	6.94071	6.78681	6.21347	6.71357	6.62506	5.53833	5.47082	5.56396	5.85319
MACO1	Q8N5G2	6.16708	6.56775	6.38907	6.90723	5.94574	5.91965	6.36804	5.79419	6.70399	6.2496	6.43518	6.33994	5.87566	5.78181	6.22291	6.33217
KIAA1191	Q96A73	6.01307	5.82489	5.80383	6.0851	6.03829	6.01761	5.50985	6.6229	5.63484	5.95762	5.98505	6.2575	7.74075	6.85868	6.51694	7.13309
CHMP7	Q8WUX9	6.23613	6.53244	7.01567	6.34287	6.37878	7.03947	6.03048	6.39965	6.49007	6.67501	6.42716	6.81114	5.9798	4.90903	4.95811	5.77418
CD276	Q5ZPR3	6.36748	6.25749	6.06702	6.29356	6.15889	6.18812	6.06648	6.16164	6.02272	5.99904	6.18783	6.28376	6.93918	6.45233	5.86719	6.68727
GALT	P07902	6.47635	5.94295	7.29481	4.9577	6.35523	6.42785	5.95278	6.84387	6.45585	6.65066	6.4992	6.90733	5.90154	6.02813	5.86981	5.43593
MMTAG2	Q9BU76	6.06525	6.04164	5.81995	6.3332	5.7842	5.88525	6.03355	5.583	5.96092	6.05037	6.01314	6.13903	6.57159	7.58221	6.883	7.25372
DRAP1	Q14919-2	5.77615	6.1256	6.22019	5.51417	5.78323	6.41821	5.77098	6.05228	6.14146	6.52761	6.24595	6.49342	6.4388	6.95501	6.92776	6.60919
L3HYPDH	Q96EM0	6.13994	6.14434	7.02296	5.60038	6.84161	6.96818	6.24742	6.37121	6.92396	6.33593	6.21334	6.44987	6.3319	5.54804	5.61157	5.24933
GTF3C6	Q969F1	6.13212	6.70891	6.01576	6.66496	6.68345	6.75448	5.605	6.71806	5.91797	5.82375	5.65562	6.72894	6.36099	6.43567	5.9043	5.89001
BBS2	Q9BXC9	6.031	6.56334	7.43273	6.54204	6.27163	5.73409	6.21643	6.34114	6.54246	7.14552	6.82094	5.95181	5.54577	5.77812	5.41992	5.66306
ANKRD13C	Q8N6S4	6.81596	6.7308	6.83376	6.48923	6.24187	6.07336	6.41148	6.58771	6.60863	6.59344	7.00971	6.90666	5.20833	5.27541	5.10397	5.10966
CLK4	Q9HAZ1	6.4824	6.00758	5.86905	6.86845	5.55187	5.16274	5.92988	4.71178	6.01642	6.233	5.99513	5.76262	7.22169	6.91301	7.5427	7.73168
RNF139	Q8WU17	6.89836	6.31114	5.80142	6.38592	6.28317	5.70299	6.55999	6.09111	6.06578	6.12907	5.89371	5.59883	6.63888	6.66551	6.81567	6.15844
UBE2C	O00762	6.12922	4.64159	6.49119	5.21586	5.72254	4.80777	5.998	7.95065	4.62093	6.28187	5.61577	7.23231	3.97623	8.93617	7.33845	9.04143
TNS2	Q63HR2-4	6.36786	6.55912	6.47371	6.19822	6.08911	6.19099	6.18642	6.15374	5.87817	6.56879	6.52446	6.10623	6.31995	6.48605	5.97374	5.92345
RAPGEF1	Q13905-3	6.71156	6.34751	6.79283	6.31831	6.35869	6.61203	6.11913	6.37887	6.4967	6.3277	6.45322	6.28632	6.19487	5.58129	5.51733	5.50364

DDA1	Q9BW61	6.18346	5.52494	6.40743	5.98997	6.20643	5.98495	5.4942	6.6509	5.83699	6.58097	7.00189	6.60854	6.33459	6.06284	6.38059	6.75133
RAF1	P04049-2	6.77362	6.83957	6.40255	6.15259	6.30837	7.04326	6.20339	6.68948	6.19808	6.37069	6.79645	6.52972	5.33432	5.52596	5.37943	5.45252
ARAF	P10398	6.10587	6.71149	6.9856	6.22443	6.60593	6.56684	6.32869	6.46039	6.34505	6.84923	6.52739	6.50284	5.50413	5.13347	5.64226	5.50642
HAUS1	Q96CS2	6.5698	4.78357	6.27293	4.85387	5.90697	4.44786	5.3233	7.98661	4.64066	6.71441	6.03127	7.486	4.90938	8.54836	7.38668	8.13832
PLEKHG3	A1L390	6.53497	6.62793	6.5221	6.66571	6.29795	6.31736	6.59458	5.86497	6.10823	6.17946	6.20888	6.49776	5.99524	5.37496	6.05553	6.15435
KRAS	P01116-2	5.6928	6.39403	5.95212	6.33968	6.0486	6.36661	6.06971	6.17163	6.0484	6.00074	6.0377	6.01874	6.35757	6.96954	6.59067	6.94146
RAD1	O60671	5.97626	6.40338	6.7874	5.60093	6.04145	6.36732	5.88199	6.08335	6.64804	6.31576	6.45456	6.24933	6.40805	5.95734	7.01633	5.80851
SLF2	Q8IX21-2	6.26082	6.56446	5.94244	6.96386	5.88277	5.88344	6.1976	6.12757	5.93005	6.02164	6.15384	6.08401	6.34585	6.32374	6.63684	6.68107
WDR89	Q96FK6	5.90746	7.08035	5.69715	6.94821	6.38117	6.79488	6.70564	6.2864	5.66907	6.08869	5.84274	5.96223	6.41315	5.66959	6.29134	6.26193
PURB	Q96QR8	6.4861	6.11695	6.48702	6.24916	6.5938	6.3959	6.06439	6.18234	6.23964	6.11318	6.65652	6.52759	6.07684	5.73989	6.06413	6.00657
S100A13	Q99584	6.02625	5.10567	6.31843	4.3861	7.13701	6.34865	5.7712	6.61243	5.76096	6.51927	6.19282	6.26275	6.12166	7.30016	7.25443	6.88222
APOBEC3D	Q96AK3	6.53369	6.29595	6.48275	6.65238	6.24835	6.28281	6.31433	6.16573	6.36878	6.41364	6.10412	6.20928	6.07974	5.88563	5.90775	6.05508
C5orf22	Q49AR2	5.36347	5.69033	6.12432	5.43071	6.08547	6.41908	5.35517	6.60634	5.90112	6.00067	6.80775	6.44221	6.43825	7.07407	7.2353	7.02574
FAM83D	Q9H4H8	6.52192	7.0005	6.97409	7.12726	6.36043	6.47794	6.28125	6.84336	7.28879	6.5462	7.12251	6.83259	4.43354	4.97646	4.428	4.78515
CHMP3	Q9Y3E7	6.88864	6.41505	6.97845	6.22444	6.56654	6.77414	6.20889	6.35868	6.44568	6.51606	6.59257	6.2911	5.49576	5.44775	5.28056	5.5157
C1orf131	Q8NDD1-5	6.36242	6.57897	6.27057	6.796	6.40454	5.96325	6.37085	6.11497	6.10928	6.1506	6.30442	6.18655	5.73877	5.99068	6.53751	6.12063
FCGRT	P55899	6.5521	6.36029	6.15748	6.56658	6.12401	6.31968	6.16238	6.56681	6.29214	6.19607	6.08592	5.99691	6.38533	6.90881	5.86606	6.35942
DCTPP1	Q9H773	6.03657	5.19097	5.8543	4.94664	5.00416	4.60068	4.87032	6.35763	4.67953	5.7823	5.08565	6.1754	5.23826	11.1783	8.48899	10.5103
CCPG1	Q9ULG6-5	7.15426	6.7097	5.8085	6.67	6.29835	6.11038	6.18017	6.39901	6.98971	5.92498	6.27311	5.8899	6.20426	5.68892	5.81028	5.88849
UBTD2	Q8WUN7	6.39466	6.03623	6.39964	5.79454	6.10593	6.27717	5.88469	5.83751	6.2195	6.52722	7.1298	6.64244	6.19213	6.17607	6.76364	5.61884
NDUFAF3	Q9BU61	5.06248	4.85626	4.77473	4.75692	6.03502	5.49478	6.41349	7.01182	5.46849	6.78279	5.88164	6.667	5.27599	8.03059	8.3304	9.15762
DYNLT1	P63172	6.26924	6.19745	6.61332	5.69985	6.17734	7.4234	6.26954	6.41042	6.50518	6.92184	6.52748	6.26222	5.88919	5.84645	5.6193	5.36778
TMEM68	Q96MH6	6.76151	6.34559	5.78651	6.57288	6.16369	5.87972	6.3791	5.91458	6.33379	6.13132	5.81737	5.58597	6.62385	6.64016	6.62487	6.4391
DENND6A	Q8IWF6	6.42739	6.91615	6.81038	6.38652	6.14726	6.62316	6.36983	6.48579	6.46732	6.42599	6.65185	6.9072	5.51255	5.23069	5.44775	5.19018
ZNF668	Q96K58-2	6.24941	5.85429	6.03951	6.21629	6.18412	6.03177	6.55032	5.8766	6.44411	6.17901	5.6664	6.19172	6.46818	6.71949	6.86027	6.46851
NUBPL	Q8TB37	6.49885	6.41034	6.03848	6.40651	6.30234	5.90077	6.39448	6.06056	5.98515	6.13236	5.924	6.08075	6.94545	6.3133	6.53974	6.06692
RBL1	P28749	5.32147	5.55773	6.00693	4.92139	5.84883	6.1602	5.46449	5.57399	5.89268	6.16394	6.30744	5.98373	7.60199	7.7852	7.77154	7.63845
PLCD1	P51178-2	5.94875	6.39211	5.9044	5.89029	6.05964	5.93371	6.20638	5.64543	6.1693	6.29931	6.51461	6.31743	6.88536	6.85077	6.55688	6.42563
TMED5	Q9Y3A6	6.19108	6.33865	5.45373	6.17929	5.65518	6.07793	6.23802	5.82015	5.95909	5.96041	5.84055	6.39456	6.88356	6.91141	6.96419	7.13221
KCNMA1	Q12791-3	6.56567	6.95754	6.08889	6.91274	6.36588	6.00418	6.23095	5.98119	6.43214	5.98119	5.93458	5.92781	6.15799	5.94279	6.1122	6.40427
GID8	Q9NWU2	5.52307	4.98174	4.57707	5.11923	4.45515	3.43726	4.20799	5.31436	3.58043	4.90394	4.10065	4.38976	4.04793	15.6586	10.107	15.5958
SLC8A1	P32418-2	6.13711	6.44435	6.22453	6.51584	6.26251	5.92011	6.50787	5.95757	6.16299	6.02683	6.34442	6.05488	6.64102	6.29954	6.32505	6.17539
FAM162A	Q96A26	6.06033	6.19693	5.41827	6.0856	5.74775	6.60841	5.55207	6.55083	5.12546	7.17206	5.86171	6.50418	6.28489	7.41272	6.33904	7.07976
KIAA1143	Q96AT1	6.51363	6.16142	6.79307	5.81398	5.96116	6.15045	6.12922	6.78978	5.91145	6.43209	6.91038	5.96465	5.99512	6.01956	6.20162	6.25245
TP53I3	Q53FA7	7.05069	5.83419	6.78978	5.5649	6.72276	6.27065	5.83705	6.93044	6.38216	6.12612	6.56656	6.47819	5.89891	5.85596	6.30529	5.38634
NUDT15	Q9NV35	6.25991	6.04809	6.84317	5.64608	6.42577	6.83571	6.14534	7.17297	6.49152	7.04896	6.89999	7.20167	4.82365	5.54844	5.16058	5.44816
HMGN1	P05114	6.02225	6.06363	5.88206	6.44056	5.68361	5.6192	6.22081	5.67723	5.99236	6.13855	5.94442	6.00276	6.65509	7.4383	7.04824	7.17092
SH3BP1	Q9Y3L3	6.25186	6.25895	7.00968	5.4785	6.42816	6.31234	6.05141	6.60404	6.69062	6.60813	7.0952	6.03827	6.30034	5.79003	5.84063	5.24185
MRGBP	Q9NV56	6.30186	6.40904	6.4036	6.32526	5.93386	5.96338	6.43241	5.93959	6.6479	6.62142	6.45431	6.42708	6.22523	5.8274	5.91353	6.17414
FER	P16591	6.19736	6.41779	6.63646	6.34885	5.93431	6.82831	6.14637	6.46935	6.07239	6.68478	6.72209	6.93573	5.90983	5.93051	5.01111	5.75477
BNC2	Q6ZN30	6.563	6.65906	6.03294	7.19178	5.92393	6.09845	6.42217	5.75934	6.19497	6.2107	6.33863	6.06631	6.23212	5.69907	6.22326	6.38427
CHST3	Q7LGC8	6.32706	6.42758	5.47555	6.92831	6.4091	6.51581	6.71338	5.44805	6.34643	6.24992	6.07185	5.9879	6.51104	5.60025	6.58787	6.39988
GPATCH11	Q8N954	5.80192	5.39147	6.43716	5.73119	5.82479	5.83153	5.88551	5.847	6.4475	6.6896	6.63882	6.38376	6.93417	6.50408	6.88249	6.769
GTF3A	Q92664	5.36046	5.80076	6.22467	5.87428	5.74294	6.35352	5.67886	5.93299	6.00376	6.16987	6.62809	6.50953	6.7995	7.08215	7.15929	6.97903
CNNM3	Q8NE01	6.69938	6.55659	6.61665	6.63444	6.41764	6.11453	5.97541	5.53351	6.61146	6.53059	6.81776	6.29455	5.7107	5.71203	5.68676	6.08801
LAMTOR1	Q6IAA8	6.39738	5.96285	5.59585	5.89944	6.1569	6.20702	6.1875	6.19516	5.64249	6.23918	6.60517	6.2735	6.06299	7.33899	6.58118	6.6544
PSMG4	Q5JS54-2	5.70933	4.66939	6.20447	4.20182	6.30974	6.55416	6.03541	7.71986	5.5995	6.15889	5.84646	6.76577	5.72999	8.8798	6.27146	7.34394
SEPHS2	Q99611	5.15489	5.30011	5.97301	5.09039	6.14279	6.30866	5.64138	6.1004	6.21837	6.27944	6.20586	6.25499	7.34407	7.30072	7.43634	7.24859
BTBD9	Q96Q07	6.38051	6.64616	6.58607	6.42569	6.49202	6.64223	6.05442	6.76788	5.94672	6.36331	6.79817	6.86101	5.3527	5.52225	5.4	5.8326
RNF2	Q99496	6.34239	6.36116	6.25751	6.78849	6.185	6.02048	6.25702	5.68016	6.57982	6.18062	6.35507	6.51321	6.26582	5.74643	6.40649	6.06034
PAN2	Q504Q3	6.76278	5.9692	7.17771	5.79515	6.33147	6.23884	6.82468	6.04436	6.79661	6.41425	6.80448	5.96027	6.22422	5.27069	6.14456	5.24072
NDUFB6	O95139	6.11453	5.92046	6.00849	6.40928	6.26148	5.87996	6.68072	5.79412	6.09265	6.03087	5.99886	5.90975	7.13872	6.436	6.65975	6.66435
FTSJ1	Q9UET6	6.27969	6.14345	7.29918	6.41894	6.74669	6.82386	5.80246	6.46241	6.05487	6.65437	6.92249	6.73819	5.54632	5.70867	5.21562	5.1828
BCL9	O00512	6.00666	5.49548	6.38394	4.88181	6.72078	5.96673	5.63254	6.40257	5.6938	6.37793	6.55964	6.69771	7.76335	6.54311	6.4101	6.46385
MRPL35	Q9NZE8	6.51363	6.38528	6.09163	5.86813	6.50591	5.88424	6.79833	6.12239	6.22936	5.68381	5.3	5.59319	7.18615	6.58665	6.38352	6.86777
ZSCAN18	Q8TBC5-3	6.88404	6.39581	6.21424	5.75219	6.63885	6.35874	5.6431	6.85542	6.34917	6.16841	6.56129	6.34867	6.46017	5.88847	5.93192	5.5495
BTN3A3	O00478	6.56226	6.39167	6.2891	6.50141	5.98184	5.81736	5.89664	6.15366	6.33109	5.95948	5.80819	5.66095	6.36968	6.49681	6.86042	6.91945
TANC2	Q9HCD6-2	6.09501	5.56249	6.82295	5.74884	6.06751	6.25755	5.90998	6.34281	6.38027	6.63686	6.76463	6.56219	5.79581	7.00379	5.93724	6.10765
ATG2A	Q2TAZ0-3	6.25814	6.31143	6.49094	5.95726	6.32501	6.73792	6.61483	6.28226	6.20314	6.75069	6.50619	7.36652	5.551	5.62241	5.49637	5.52588
BDP1	A6H8Y1	6.54184	6.19511	6.5441	6.98202	6.0863	5.93115	6.27602	5.90244	6.80045	6.2952	6.55096	6.47947	5.72327	5.59931	5.88807	6.20428
NCR3LG1	Q68D85	6.9118	6.11394	6.09944	6.33019	6.4702	5.99007	6.14554	6.22841	6.41793	5.81985	5.91454	6.14268	6.74983	6.06114	6.10648	6.49798
EPHX4	Q8IUS5	6.38094	6.77862	6.28108	6.49164	5.70271	5.76441	6.18458	6.13857	5.92015	6.13822	5.97814	6.22869	6.48507	6.22629	6.85371	6.4472
TRAF6	Q9Y4K3	6.54424	5.57569	6.6874	6.03723	6.39288	6.55711	5.79351	6.49435	6.19492	6.47789	7.02378	6.88152	6.02683	5.54648	5.47802	6.28814
INPP4A	Q96PE3	5.88447	5.98637	7.08311	6.2112	5.90118	6.27984	6.06012	6.48441	6.19987	6.67495	6.60835	6.79036	5.97172	6.22925	5.99986	5.63496
CLU	P10909-2	7.11578	7.10641	6.20935	6.92782	6.55681	6.24807	6.39238	6.26027	6.14026	7.06551	6.30566	6.058	5.39327	5.60309	5.3077	5.30962
SHOC2	Q9UQ13	6.04554	6.11278	6.48914	6.3443	5.92675	6.13745	6.18199	5.95237	6.55558	6.4573	6.30035	6.23255	6.33769	6.24162	6.35151	6.33309
FBXL6	Q8N531	6.198	6.86707	6.39335	6.63111	5.82147	6.80423	6.18449	6.66669	6.40337	5.97877	6.88401	6.50498	5.59171	5.48564	5.59895	5.98618
MAP2K1	Q02750	6.10818	6.03954	6.85634	5.99795	6.25793	7.00468	6.38791	6.6864	6.48883	6.41098	7.07531	6.84228	5.66093	5.62944	5.11809	5.43521
UXS1	Q8NBZ7-2	6.41464	6.40991	6.17864	6.86434	6.16689	5.7822	6.49354	6.00781	6.10148	6.12489	6.23298	6.28752	6.32369	5.95464	6.33741	6.31943
TSEN54	Q7Z6J9	5.76288	5.76104	6.7852	5.89722	6.35444	6.25181	5.63879	6.06554	6.21298	6.15533	6.79484	6.43727	6.63162	6.37089	6.69351	6.18664
CNOT8	Q9UFF9	6.1338	6.22474	6.26996	5.8849	6.24336	6.51274	6.47854	5.98475	6.35192	6.1653	6.06605	6.77975	6.3484	6.4028	5.65447	6.49851
SOWAHC	Q53LP3	6.63268	6.87998	6.94171	6.25624	6.61406	6.26466	6.56754	6.72282	6.49798	6.14764	6.56349	6.10668	6.29444	5.26967	5.09918	5.14121
NGEF	Q8N5V2	6.61841	6.33839	6.65031	5.93826	6.2681	6.97914	6.47618	6.38046	6.66492	6.57183	7.09182	6.64253	5.39199	5.5057	5.45769	5.02427
NDUFB5	O43674	6.37793	6.47874	5.82809	6.58585	6.36127	5.97591	6.46675	6.03362	5.92178	6.15821	5.83405	5.94417	6.9582	6.26904	6.73792	6.06848
MAML1	Q92585	5.65888	5.93368	6.56671	5.55242	5.56406	6.47586	5.83748	6.26827	6.25939	6.55515	7.37533	6.94308	6.50534	6.13462	6.19729	6.17243
C5orf51	A6NDU8	6.46997	5.35092	6.50508	5.28834	5.84561	5.89861	5.88997	6.58999	5.57441	6.55442	6.30092	7.15917	6.16137	7.28422	6.52266	6.60432
ZNF639	Q9UID6	6.54308	6.52603	5.98474	6.79343	6.41249	6.39204	6.05496	6.11376	6.32562	6.2075	6.07607	6.22175	6.21956	6.0465	6.06568	6.01681
LIMK2	P53671-3	6.34366	5.03599	6.84807	5.30912	6.32344	5.82316	5.44933	6.19161	6.05369	6.8205	6.50784	7.32183	6.17867	6.47755	6.27005	7.04549
FPGT	O14772	6.16843	6.26229	6.93935	5.83805	6.15081	6.6284	6.44994	6.72609	6.25366	6.33993	6.51038	6.50645	5.69415	6.11246	5.70063	5.719
TCF12	Q99081-3	5.59447	5.90815	6.52873	5.23177	5.64973	6.24185	5.86222	6.29312	6.26376	7.14654	6.4963	6.08604	6.51117	6.72275	6.97171	6.4991
JPH1	Q9HDC5	6.24635	6.40375	5.92643	6.57121	5.87123	6.17991	6.38822	6.14089	6.39059	6.13869	6.107	5.69022	6.6862	6.62881	6.39549	6.23502
CC2D2A	Q9P2K1	7.2228	6.95616	7.20698	6.69087	6.10841	5.78415	6.44372	6.06114	6.95486	7.01781	6.93752	6.5218	4.90993	4.97725	4.99565	5.21096
AURKB	Q96GD4-5	6.82298	6.56927	6.28461	6.29564	6.53083	6.50655	6.52261	6.65269	6.26544	5.87827	6.24028	6.12948	5.71448	6.10045	5.74152	5.7449
BROX	Q5VW32	6.47085	6.06664	6.69886	5.73269	5.70151	6.83787	6.29646	6.27796	6.38292	6.35299	6.56947	6.34073	5.9177	6.16684	6.20248	5.98405
CHN1	P15882	6.24933	6.16011	7.33413	5.47767	6.74725	6.80348	6.01896	5.96033	6.88856	6.69565	6.46379	6.6515	5.36588	5.77099	6.17883	5.23355
ASF1B	Q9NVP2	6.06977	6.22529	6.34301	5.8588	6.53378	6.00065	6.5602	7.02327	6.71002	6.79409	6.22297	7.15145	5.5719	5.38406	5.68923	5.86151
PJA2	O43164	6.79209	6.17701	7.93064	6.76094	6.28534	7.15832	6.9256	7.38158	6.06052	6.83039	7.04203	7.70496	4.12904	4.79296	3.67229	4.3563
CLPP	Q16740	5.97228	4.991	4.81085	5.15137	5.74662	4.65819	6.08632	6.69435	4.82163	5.36638	4.93163	6.21183	6.07373	9.7716	8.85902	9.86851
R3HCC1L	A0A384DVK4	6.80019	6.25792	6.8525	6.29505	6.23419	6.22528	6.2419	6.68985	6.39636	6.60414	6.53947	6.85378	5.56982	5.70405	5.22191	5.51359
TRMT61B	Q9BVS5	6.32872	6.37623	5.86581	6.81102	6.28621	6.19666	6.49187	6.54772	5.74606	5.90075	5.87409	5.757	6.54319	6.18166	6.69134	6.40166
MRRF	Q96E11	6.17157	6.49251	5.66513	6.83214	5.9812	5.70147	6.54871	5.86888	6.04125	6.29753	6.38316	6.408	6.4488	6.01271	6.38263	6.76429
SOD1	P00441	6.88485	5.59252	6.6528	4.71031	5.94785	5.73241	5.4452	7.30699	5.73776	6.5426	6.83196	6.61925	6.0536	6.96517	6.00368	6.97304
OXNAD1	Q96HP4	6.42279	6.33648	5.29595	6.78682	6.72983	5.77791	6.35592	6.10847	5.97706	6.0158	5.92809	5.96627	6.66939	6.31765	6.62863	6.68295
TDRD9	Q8NDG6	8.17124	7.65851	8.24565	7.46901	6.8973	6.69687	6.28443	6.72556	7.71234	7.97219	7.37685	7.57567	2.5065	3.29171	2.51223	2.90396
UBAC1	Q9BSL1	6.95293	6.14877	6.94212	6.28456	6.05947	6.22024	5.94792	6.77048	6.76361	6.91315	6.88073	7.07332	5.63323	4.64326	5.40443	5.36177
ARL2	P36404	6.09166	6.42537	6.59597	6.11555	6.01879	6.51767	6.58987	7.09274	6.50855	6.70019	6.43889	6.691	5.8295	5.45785	5.4101	5.51629
RBMX2	Q9Y388	5.78061	6.68235	6.16917	6.71172	5.89669	6.00573	6.53791	6.08748	6.43305	5.71668	5.95978	6.16348	5.78903	6.73211	6.74866	6.58553
NKIRAS2	Q9NYR9	6.51737	6.3432	7.09179	6.97722	6.42814	6.60918	6.26727	6.59058	6.92392	7.2016	6.92062	7.13541	4.62967	5.04374	4.36351	4.95678
FRA10AC1	Q70Z53	5.99022	5.96418	6.87994	6.10578	6.26437	6.32114	5.80964	5.79483	6.42681	6.54939	6.44446	6.58338	5.98899	6.47072	6.53621	5.86994
TBC1D22A	Q8WUA7	6.1503	5.90974	6.62765	6.20386	6.53942	6.76018	6.15597	6.81287	6.24403	6.34601	6.37919	6.06555	5.80135	6.43525	5.80396	5.76468
TRAM2	Q15035	6.61118	6.73873	6.36425	7.14559	6.15617	6.0322	6.21782	5.99732	6.02013	6.07404	6.17121	5.83139	6.18836	5.94359	6.45067	6.05734
SNX29	Q8TEQ0	6.51845	6.63588	6.08228	6.23021	6.26016	6.11205	5.92339	5.62634	5.95982	6.5538	6.50805	6.60879	6.09803	6.35541	6.35718	6.17016
NSUN6	Q8TEA1	5.58962	5.89377	6.4923	6.06955	6.55868	6.58479	6.17461	6.69336	6.1992	6.65254	6.65644	6.97841	6.15414	5.94347	5.61196	5.74718
OARD1	Q9Y530	6.48854	6.2135	6.14592	6.14698	6.21501	5.95233	6.46309	6.37005	6.09423	5.94786	5.73101	5.92114	6.74763	6.52404	6.70602	6.33265
ASTE1	Q2TB18	6.13601	6.18323	6.40024	6.22097	6.14737	6.20021	6.26976	5.88287	6.71917	6.48404	6.73399	6.87594	5.82309	5.86035	6.07074	5.99201

UBN2	Q6ZU65	6.23394	6.50055	5.96485	6.95434	6.21009	6.0992	6.23981	6.0573	6.14351	6.49055	5.93832	6.36357	5.9262	6.11428	6.32376	6.43974
TUBGCP6	Q96RT7	5.71467	6.22739	7.04534	6.38871	5.66079	6.45644	6.14052	6.41907	6.21373	6.89996	6.96398	6.44509	5.57662	6.07651	5.91444	5.85673
HBA1	P69905	6.841	5.93769	5.57511	5.92881	4.46862	5.64473	8.49192	4.26809	5.62311	6.17991	5.55702	6.38675	6.6213	5.70695	8.43547	8.33353
COA3	Q9Y2R0	6.40886	5.59446	5.88239	5.26385	6.5235	5.36718	5.85172	6.34996	5.73395	6.07687	5.85866	6.02883	6.74973	7.63887	7.47509	7.19609
UCHL3	P15374	6.07803	5.30026	6.34102	4.45899	6.05758	6.42142	6.75049	7.58293	5.16896	6.56911	5.77346	6.82509	6.19964	7.1402	6.38707	6.94577
CTSZ	Q9UBR2	6.55693	6.51328	6.11876	6.01107	6.49223	6.30375	6.11557	6.25202	6.13622	5.93093	5.8547	5.50197	6.47365	6.85108	6.46119	6.42666
PBX3	P40426	6.49157	6.084	6.41951	6.43111	6.31569	6.25736	6.05833	5.69015	6.24326	6.0546	6.57296	6.3645	6.31886	6.17329	5.78796	6.73685
FAM219A	Q8IW50	5.75676	5.77747	5.94512	6.22887	5.14119	5.71607	6.46658	4.90027	5.83753	5.8835	6.20245	6.50447	6.23072	7.85643	7.44675	8.10582
BAK1	Q16611	5.83123	4.77568	5.3543	5.26718	5.90287	5.77027	5.8486	6.80409	5.29021	5.98041	5.46894	5.8447	6.42477	8.65841	8.06853	8.70983
RER1	O15258	6.20998	6.58036	5.78702	6.2843	6.09295	6.10544	6.38386	6.25633	5.94583	6.1575	6.08771	6.12556	6.32815	6.5335	6.61528	6.50624
NFATC2IP	Q8NCF5	5.99203	5.93666	6.1132	5.66708	5.70922	6.0442	5.69965	6.47643	5.86718	6.25971	6.35201	6.33062	6.46285	7.1589	6.85993	7.07033
MRPL53	Q96EL3	6.21966	5.38435	5.64322	5.72565	6.44242	5.89843	5.89587	6.70885	5.3875	6.15351	5.92563	6.86539	6.39121	7.19077	7.13532	7.03222
STX8	Q9UNK0	6.28302	5.76816	5.83768	6.68993	6.20343	5.88164	6.07786	6.25118	5.64501	6.49722	6.10361	6.81682	6.06007	6.74053	6.29933	6.84451
SETD9	Q8NE22	6.11166	6.47578	5.76615	6.65143	6.1766	6.37907	6.36139	5.98256	6.0734	6.47897	6.29053	5.94877	6.21448	6.16977	6.3468	6.57264
FKBP2	P26885	6.16465	6.62324	5.52194	6.27084	6.99796	6.72234	6.19241	6.906	5.58411	6.15192	6.10909	5.89498	6.58667	6.21082	6.09486	5.96817
MRPL12	P52815	6.076	4.82979	4.85707	5.20204	5.22888	3.65035	5.27583	5.47598	4.24322	4.72324	4.32032	4.82667	5.22902	12.7936	9.57485	13.6932
MAGI1	Q96QZ7	6.50772	6.71083	6.43002	5.77519	6.30093	6.56426	6.15155	5.81649	6.08096	6.22759	6.14204	6.06337	6.51711	6.43536	6.2116	6.06499
TEC	P42680	6.53595	6.48582	7.40966	6.7894	5.79766	6.30267	6.01018	6.64919	6.11865	6.59488	6.37216	6.83882	5.42469	5.72351	5.31226	5.63451
ATN1	P54259	6.2275	5.73082	6.85769	6.26264	6.24493	6.40236	6.22477	6.25194	7.02326	6.12224	7.07836	6.92258	6.34539	4.8274	5.61869	5.85943
SESTD1	Q86VW0	6.17131	6.47643	6.83821	6.23692	6.13505	6.60382	6.23929	6.3254	6.88947	6.86333	6.41836	6.381	5.49841	5.75529	5.58494	5.58276
ALG6	Q9Y672	6.29795	6.35683	5.53474	6.67131	6.01567	5.89532	6.24148	6.01865	6.00605	6.28793	5.65951	5.74554	6.76584	6.54605	7.06375	6.89339
GALNT11	Q8NCW6	6.47113	6.7103	5.81079	6.52831	6.28146	6.27754	6.47428	5.81495	6.38046	5.96091	6.07982	5.74391	6.71651	6.33197	6.58243	5.83523
NUBP2	Q9Y5Y2	6.13095	5.66658	6.51278	5.61447	6.36734	6.29705	6.15671	6.63133	6.42379	6.08555	7.2113	7.10453	6.03696	6.00261	5.87917	5.87886
BAX	Q07812-2	5.89136	5.65708	6.25492	5.84861	5.97452	6.7021	6.40638	6.19338	6.19716	6.47939	6.11946	6.23819	5.48675	7.10581	6.54518	6.89969
CNOT11	Q9UKZ1	6.60273	6.0748	6.40882	5.51987	6.44563	6.31252	6.20168	6.70707	6.27224	6.45927	6.54638	6.84101	5.75231	5.70898	5.90343	6.24326
POT1	Q9NUX5	5.89085	6.2079	6.09033	6.10935	5.92456	6.39455	6.42076	6.16489	5.97811	6.1649	6.18063	6.32651	6.64128	6.63934	6.52313	6.34291
MTRF1	O75570	5.69628	6.15545	5.97854	6.73224	6.46143	6.48568	6.0737	6.09058	6.48156	6.1028	6.5788	5.96163	6.2192	6.33538	6.14698	6.49976
CXorf38	Q8TB03	6.8368	6.08907	7.15616	5.93761	6.54203	6.74753	6.1892	6.63684	6.42007	6.52518	6.76953	6.59888	5.64513	5.29459	5.09825	5.51315
RAVER2	Q9HCJ3	5.47913	6.01631	6.35626	5.51438	5.99481	6.05897	6.01545	6.33257	6.11291	6.03361	6.576	6.24518	6.29629	6.87577	7.44625	6.64612
FBXL12	Q9NXK8	5.66673	5.67996	6.05022	5.18598	5.87044	6.15655	5.4845	6.32336	6.03711	6.38175	6.03329	6.08803	7.30212	7.2383	7.77749	6.72418
MTIF3	Q9H2K0	5.93874	7.07599	5.48331	6.58917	5.6764	6.46692	6.08653	5.81818	6.24406	6.36002	5.65093	5.99959	6.43506	6.84433	6.80552	6.52526
LSM6	P62312	5.29847	5.47287	5.56049	5.16648	6.71987	6.19048	6.32569	6.87724	6.26712	6.62077	6.37909	6.17883	6.49727	6.58287	7.12234	6.74012
NAGPA	Q9UK23	6.4544	6.83395	6.21446	6.59507	6.57971	5.94638	6.22463	5.97095	6.42279	6.51021	6.5937	6.53022	6.33745	5.56198	5.55592	5.66817
UCKL1	Q9NWZ5	6.34979	6.22907	6.73324	5.81197	6.38216	6.7834	6.25811	6.47608	6.46074	6.58797	6.79791	6.352	5.63473	5.73705	5.7206	6.58519
SLC5A3	P53794	6.10428	6.32489	6.37124	6.45721	5.86359	6.2861	6.20696	6.54544	6.32977	6.44043	6.39696	6.09805	6.11988	6.4185	6.29357	6.64312
PMM1	Q92871	7.70455	7.26257	8.10169	7.48896	6.92686	6.56949	5.91966	6.24201	6.99254	8.04352	7.59255	7.49205	3.46418	4.08854	3.43015	3.68066
IDUA	P35475	6.21402	6.41218	6.13353	6.3905	5.98537	5.99522	6.02464	6.16383	6.54159	6.19283	6.05857	6.16295	6.61357	6.35531	6.67128	6.08462
APOO	Q9BUR5	6.44425	6.52082	5.91305	6.12024	6.51064	5.95735	5.79302	6.42721	6.03494	6.40672	6.12709	6.05783	6.76604	6.41601	6.29877	6.206
PRIMPOL	Q96LW4	6.39963	6.92753	7.12256	6.55196	6.19953	6.57238	6.17964	5.95317	6.99052	6.86176	6.4254	6.56038	5.28989	5.21884	5.58936	5.15746
RASA4	O43374	6.63041	6.59389	6.65981	6.08407	6.20634	6.279	6.40022	6.61291	6.39827	6.761	6.44889	6.31646	5.53528	5.95529	5.45727	5.66088
LCLAT1	Q6UWP7	6.34248	6.20158	5.87918	6.42196	5.82788	5.97639	6.29558	6.038	6.28575	5.974	6.40759	6.03658	6.86072	6.39605	6.70022	6.35595
DDI2	Q5TDH0-3	6.08349	6.13316	6.60774	6.13294	6.09203	6.68074	6.36162	6.4061	6.27915	6.09688	6.59268	7.15813	5.8998	5.88546	5.80851	5.78156
DCAKD	Q8WVC6	6.61091	6.51837	5.80104	6.30792	6.55163	5.7838	6.77014	6.38191	6.32927	5.89351	5.91505	5.8122	6.58469	6.03248	6.72792	5.97917
GNB1L	Q9BYB4	6.16882	6.29742	6.72311	5.81115	6.19298	6.76449	6.23745	6.85398	6.32431	6.32929	6.62672	6.88711	5.6288	5.95129	5.50556	5.69753
SUMO1	P63165	6.41234	5.94724	5.97164	5.88904	6.19608	5.75391	5.58647	6.31051	5.51195	6.59965	6.26679	6.56717	6.73401	6.74808	6.77632	6.7288
CNKSR2	Q8WXI2	5.91216	6.01695	6.58305	6.85245	6.56584	6.03983	6.21934	6.50718	6.66085	6.39172	6.77064	6.40453	5.84434	5.63248	5.5524	6.04623
MGST3	O14880	6.23252	6.47475	5.56145	6.63424	6.4317	6.86365	6.30761	6.16943	5.25591	6.22237	6.20196	5.74156	6.69792	6.68788	6.36977	6.14727
RPRD1A	Q96P16	5.91142	6.40393	5.94543	6.69397	6.03074	5.79614	5.53282	6.66093	6.03554	6.34956	6.26083	6.30645	6.93663	6.17202	6.39982	6.56377
TMA16	Q96EY4	6.35379	6.26367	6.34993	6.32187	6.25499	6.02422	5.97911	5.89489	5.96826	5.90902	6.35659	6.04947	6.31579	6.75153	6.68649	6.52542
MIX23	Q4VC31	6.27385	5.50533	5.95144	5.65617	6.9755	5.73869	5.49512	6.27815	5.61461	6.60689	6.37281	6.70676	6.98686	6.35213	6.7491	6.73657
NDUFB3	O43676	6.00876	6.23247	5.61234	6.417	6.57779	5.72882	7.25934	6.32919	5.89555	5.12695	5.79	5.84882	6.60217	6.82589	6.92603	6.81887
PDPK1	O15530	6.79261	6.19456	6.82615	6.40473	6.5206	6.78209	6.28174	6.49682	6.88233	6.49676	6.38242	6.39941	5.76724	5.24579	5.42446	5.10229
SZRD1	Q7Z422	6.93781	5.92532	7.27053	5.60248	6.48454	6.27135	6.23397	6.32694	6.65944	6.21831	7.29877	6.3897	5.93564	5.83017	5.28245	5.33259
SLC35B1	P78383-2	5.84523	6.30987	6.15562	6.75316	6.70058	6.63201	6.23023	5.88608	6.01797	6.36556	6.15631	5.96101	6.5676	6.2801	6.05311	6.08557
CREB1	P16220-1	6.46976	5.94265	6.31635	6.25614	6.45432	5.84964	6.23441	6.21441	6.50455	5.85524	6.28818	6.3872	6.32113	6.02278	6.53397	6.34927
DSN1	Q9H410	6.41482	6.2227	6.37155	6.08865	5.74979	6.02716	5.98893	6.09133	6.67284	5.97027	6.42056	6.07045	6.94594	6.40622	6.21641	6.34239
CLN5	O75503	6.77395	6.5637	6.08711	6.51998	6.19162	6.02436	6.42876	5.97902	6.21467	6.3466	6.17434	6.14013	6.24997	5.90813	6.28094	6.1167
CDCA5	Q96FF9	6.4275	6.16065	5.98882	6.70764	5.88067	5.97889	6.23995	5.64945	6.33227	6.03865	6.04554	6.05294	6.52066	6.28818	6.9295	6.75869
FBXO21	O94952	6.15082	6.18917	7.19014	6.13641	6.36636	6.59828	6.20288	6.66132	6.22595	6.00995	6.38429	6.51269	5.81659	5.80311	5.75242	5.99961
ZNF346	Q9UL40-2	6.54906	6.71115	6.44367	6.56306	6.20559	6.57057	6.5816	6.25188	6.02737	6.31393	5.96903	5.79033	5.89364	6.14774	6.38421	5.97177
TMEM11	P17152	6.39649	5.91489	6.09431	6.06463	6.5454	7.08141	6.54067	5.75252	5.93068	6.02883	6.24074	5.45805	6.69926	6.13425	6.72479	6.39309
NUP42	O15504	6.13938	6.66279	5.85802	6.75377	6.03504	6.35673	5.42488	6.57369	6.07039	6.69639	5.4438	6.57911	6.20631	6.20137	6.12943	6.86889
IQCB1	Q15051	7.01546	6.97399	8.00806	6.93597	7.07	6.53315	6.45642	6.05734	6.00012	5.45297	5.77024	5.82547	5.38597	5.49472	5.21476	5.80536
MGAT2	Q10469	6.28499	6.07157	6.10264	6.95197	5.9974	6.52058	6.26651	5.8368	6.33999	6.37866	5.85521	6.48677	6.19869	6.25863	6.51032	5.93926
CINP	Q9BW66-3	6.0914	4.96607	5.7003	4.71433	6.5151	6.32104	6.03563	8.05752	6.32946	7.27381	7.21052	7.62961	4.87659	5.9539	6.28925	6.03546
TARBP1	Q13395	5.66712	6.57925	5.89402	6.3667	5.98702	6.86448	5.50422	6.56713	6.06262	5.94849	6.15903	6.22957	7.7563	6.23601	5.97175	6.20629
SRA1	Q9HD15	6.6653	6.00844	7.02732	5.42682	6.73584	6.86487	6.82595	7.20786	6.2719	6.47221	6.63425	7.0059	5.69347	5.70331	5.47466	4.9819
GTSF1	Q8WW33	6.51856	5.53448	7.13627	5.2568	6.57083	5.96532	5.5173	7.23847	5.7992	6.92292	6.66521	8.51652	5.3233	5.99787	5.2963	5.74065
OSTM1	Q86WC4	6.72147	6.48987	5.97347	6.92077	6.51876	6.01508	6.67109	5.63524	6.6472	5.91227	6.45652	6.32148	5.93226	5.98507	5.46992	6.32951
CFAP20	Q9Y6A4	6.20826	6.24417	6.47313	6.4779	6.13449	6.19371	6.41852	5.59933	6.33246	6.15293	6.22225	6.2048	6.85881	5.54138	6.69844	6.23942
FOSL2	P15408	5.82699	5.88306	6.16625	6.3659	5.9206	5.96944	6.21519	5.92865	5.54435	5.28695	5.3178	5.92964	8.20979	6.85728	7.27297	7.40485
HMG20A	Q9NP66	6.20563	6.38116	6.00169	6.97673	5.84672	5.92313	6.43307	5.62513	6.16793	5.92671	6.30805	6.5569	6.67155	6.18555	6.51995	6.2701
MIF	P14174	4.90263	4.93444	5.92188	4.77896	5.53475	5.12195	5.85182	7.0121	5.8324	6.1358	6.56257	6.02668	4.3977	9.89445	7.86251	9.22937
PASD1	Q8IV76	6.07065	6.40319	6.15387	6.44462	6.15424	6.10734	6.79101	6.04035	5.9095	6.33751	6.07491	6.10591	6.28788	6.06792	6.38838	6.66271
MED10	Q9BTT4	5.87207	5.32917	4.56892	5.3738	4.95297	3.45141	4.74916	5.21174	3.96249	5.0354	4.38969	4.4923	4.4782	14.0158	9.82129	14.2955
MRPL30	Q8TCC3-2	6.31752	6.51334	5.80575	6.58908	6.19997	6.2735	6.54751	6.00425	6.02108	6.20495	5.6827	5.7305	6.45968	6.35711	6.76231	6.53075
XYLT2	Q9H1B5	6.32678	6.46203	6.30755	6.8879	6.22173	6.05506	6.31191	5.74904	6.62249	6.58562	6.00336	6.45688	5.94515	5.99888	6.02156	6.24406
H1-0	P07305	6.31446	6.11284	5.3881	6.36308	6.02183	5.82366	6.21082	6.06063	5.81486	5.61871	5.49983	5.53816	7.03993	7.61699	7.40497	7.17114
SPCS3	P61009	6.46593	6.27894	6.01192	6.44169	6.4178	6.00143	6.4791	6.12764	6.16561	6.31108	6.02964	5.92541	6.42316	6.08909	6.60313	6.22845
MRPL42	Q9Y6G3	6.11035	5.19488	5.3074	6.71335	6.35663	5.12194	7.48055	5.86975	6.88893	4.78042	5.35725	5.95411	6.58422	6.85052	7.12912	8.30058
SNAPC2	Q13487	5.94281	6.30286	6.42456	6.13348	5.7763	6.24223	5.95991	6.07472	6.09634	6.2833	6.47522	6.64173	6.50264	6.39816	6.54126	6.20448
DEF8	Q6ZN54	6.29977	6.6565	6.65597	6.99658	6.11008	5.92578	6.40458	6.42557	7.44066	6.38122	6.01764	6.5515	6.06436	5.30802	5.69983	5.06194
TMEM115	Q12893	5.89685	6.37047	5.95146	6.98224	6.00633	5.87981	6.11372	6.26364	5.93656	6.28631	6.27043	6.3183	6.47504	6.49023	6.1951	6.56352
CRABP2	P29373	7.46952	5.97305	6.33995	5.4401	6.62198	7.20923	6.40023	5.99132	6.5152	7.0335	6.40342	6.56866	5.85179	5.47618	4.92019	5.78567
STRN4	Q9NRL3-3	5.92854	6.38358	6.31287	5.79584	6.27651	6.78771	5.73889	6.5608	6.43514	6.13312	6.05107	6.53994	6.69196	6.03218	6.01209	6.31976
STRIP2	Q9ULQ0	6.19016	6.56126	6.57203	6.17443	5.93265	6.44246	5.91367	6.06768	6.77352	6.67702	6.34876	6.10058	5.98416	6.11304	6.2041	5.94448
BLOC1S4	Q9NUP1	5.72899	5.28921	6.65481	5.15365	6.1572	6.21801	5.26029	6.58326	5.50377	6.675	6.57769	7.11107	6.47322	7.31656	6.57934	6.71793
RBM23	Q86U06	5.7038	6.36813	6.28213	6.68565	5.27549	6.39305	6.00506	5.9516	6.93892	7.11898	6.98356	6.70786	5.86334	5.86129	6.0469	5.85024
NEK7	Q8TDX7	5.95418	6.89568	6.83596	5.448	6.98109	7.08657	5.93892	6.20988	6.34506	6.14525	6.79725	6.10241	6.45809	6.28884	5.25479	5.25804
BOD1	Q96IK1	5.99423	5.27396	5.75944	5.46439	5.56586	4.16999	4.94877	6.65227	4.67677	5.63154	4.78756	5.91282	5.02754	11.4472	8.42862	10.259
C5orf24	Q7Z6I8	6.48685	6.17584	6.58679	6.48389	6.20526	5.93961	6.16551	5.62109	5.86088	6.44966	6.51028	6.01162	6.18388	6.47344	6.62847	6.21694
GSTCD	Q8NEC7	6.14569	6.29127	6.88653	5.88839	6.48943	6.82868	6.46098	6.85024	6.4099	6.6156	6.22675	6.50053	6.58049	5.70308	5.36841	5.65403
SYVN1	Q86TM6	6.45489	6.3885	5.95427	6.43582	6.58641	6.07824	6.08909	6.27213	6.04784	6.56618	5.3161	5.83889	6.83527	6.41482	6.46985	6.25168
COMMD3	Q9UBI1	5.80286	4.87088	5.73645	5.03143	5.3755	5.49608	5.4941	6.74913	5.31454	6.04091	5.42422	5.50885	5.7467	9.40906	7.84909	10.1502
FYN	P06241	5.99541	6.40308	5.97744	6.91455	6.30762	6.67621	5.99411	6.07947	6.25217	6.01411	5.95397	6.60781	6.83109	6.179	5.76218	6.05178
PRKD1	Q15139	6.15331	6.20448	6.76027	6.16711	6.15893	6.13449	6.21313	6.25574	6.49694	6.67458	6.71422	6.65805	5.75317	6.02392	5.644	5.98766
STK36	Q9NRP7	6.79451	6.44475	6.9755	6.77367	6.56291	6.69055	6.36196	6.73937	6.57272	6.59964	7.1293	6.36335	5.32692	5.09376	4.71631	4.85478
MACROD1	Q9BQ69	6.20668	6.46512	6.35032	6.32062	6.90012	6.23254	5.76465	5.68745	6.08212	5.52408	6.25414	5.96618	6.80667	6.71699	6.79297	5.92935
SEPSECS	Q9HD40	6.43999	6.98103	6.47374	5.67067	6.75507	6.52136	6.6274	6.95594	6.39775	6.20963	6.80727	6.08013	5.68032	5.53915	5.57501	5.28553
PCBD2	Q9H0N5	6.01064	5.33377	6.28537	5.58123	6.58566	5.59388	5.80132	6.6937	5.4554	6.77004	6.44381	6.71786	6.30436	6.84396	6.72209	6.85689
NEURL4	Q96JN8	5.93499	6.20051	6.93052	6.11684	5.84049	5.93929	6.6049	6.27596	6.32083	6.47089	6.12507	6.87268	5.71622	6.33705	6.28297	6.03078
MIEF1	Q9NQG6	6.53866	5.78089	5.557	6.86481	6.85818	5.65959	6.3387	5.72703	5.53279	5.94741	5.622	6.32505	6.84392	6.52865	6.6234	7.25194
KLHL24	Q6TFL4	5.55383	6.09899	5.2838	5.01957	5.69835	5.60797	5.36528	5.92194	6.25159	7.59918	6.58189	6.07751	6.77922	7.14028	7.4699	7.5507
GHDC	Q8N2G8	6.23268	6.3408	5.75085	6.1857	6.23337	5.90208	6.18695	6.19506	6.00764	5.92882	5.88583	5.89991	6.97801	6.57543	6.96983	6.72704
VMA21	Q3ZAQ7	6.18861	5.36527	5.92551	5.11606	6.22035	5.44094	6.55031	6.34368	5.63608	6.40349	5.6518	5.47641	6.12267	8.05711	7.49797	8.00373

ADGRL1	O94910	7.24255	6.72128	6.00456	6.46093	6.0702	6.23646	6.65668	6.25628	6.38137	6.00102	6.23897	5.94445	6.0638	5.60029	6.14559	5.97558
PRDM11	A0A087WWZ6	5.95747	6.27812	6.17359	6.10329	6.10206	6.24946	6.24262	5.65284	6.20286	6.37226	6.42026	6.24621	6.64598	6.78864	6.39292	6.17142
NAA40	Q86UY6	6.44781	7.04488	6.27921	6.7843	5.55829	6.70234	5.69478	6.48333	6.54892	7.36035	5.95418	6.78285	5.20517	5.97412	5.29973	5.87973
PLD2	O14939	6.72534	6.83526	6.37873	6.64903	6.13131	5.95315	6.1454	6.2795	6.24715	6.35236	6.45203	6.29877	5.75325	5.96412	5.96697	5.86762
IKBKB	O14920	6.16429	6.68076	6.82007	5.43058	6.15374	7.08353	6.42907	6.59012	6.70732	6.62082	6.78663	6.13006	5.52498	5.78562	5.55595	5.53646
MAPK9	P45984	6.08801	6.53566	6.41608	5.91915	5.92773	6.33436	6.00391	6.61448	6.78991	6.62835	6.48724	6.39933	5.87326	6.36068	5.55232	6.06954
CUX1	Q13948	6.21736	6.55369	6.16484	6.07594	6.22765	6.39218	6.32345	5.99791	5.74405	5.77923	6.10907	5.78139	7.19934	6.57165	6.56824	6.29401
STK25	O00506	6.4991	5.82965	7.11242	5.66108	6.2475	6.44693	6.35403	6.95107	6.62469	6.62442	6.63852	6.18007	5.85093	5.93764	5.63877	5.4032
ARID3A	Q99856	6.25714	5.83873	6.26473	6.57216	6.22097	6.09888	6.13588	6.6259	6.20793	6.09655	6.10266	6.2587	6.81438	5.97834	6.19747	6.3296
NR0B1	P51843	6.54673	5.5005	6.12835	7.30301	6.91309	6.08345	6.3735	6.00078	6.05711	5.79254	6.22988	5.42893	6.93929	5.85884	6.15542	6.68857
ANK1	P16157-14	6.21629	6.23422	7.01237	6.09128	6.40871	6.32386	5.62302	6.63016	6.63738	6.52615	6.49042	6.43958	6.17087	6.45379	5.62862	5.11329
ALDOC	P09972	5.45819	5.97404	6.64769	5.50309	6.25928	6.25988	6.98567	6.63443	6.22647	6.12396	6.39653	6.95224	5.7567	6.84538	6.04082	5.93561
PALM2	Q8IXS6-2	6.26357	5.80268	6.20232	5.86202	5.9993	6.1187	5.90064	5.85364	6.30339	5.81462	6.20029	5.7263	7.56738	6.67261	7.29012	6.42243
WASHC2C	Q9Y4E1-5	6.06815	5.77347	6.9202	5.43886	5.76841	6.40716	6.02876	5.7573	6.01079	6.46984	6.72974	6.72915	6.37223	6.52289	6.72776	6.27531
MB21D2	Q8IYB1	6.02058	6.01655	6.57899	6.54229	6.09617	6.55349	6.13579	6.5007	6.39272	6.82525	6.73513	6.60755	5.78818	5.76968	5.85275	5.58418
ARFIP2	P53365	6.24794	5.20993	6.32133	5.32867	6.24802	5.91395	6.26195	6.21492	5.65679	6.61829	6.23931	6.44585	6.92672	6.83581	7.16476	6.36578
PYCR3	Q53H96	6.30869	5.62186	6.40712	6.23732	6.07969	6.58339	6.41063	5.93015	6.22447	6.06914	6.68551	6.10552	6.55402	6.03731	6.13031	6.61488
SARAF	Q96BY9	7.1938	7.92334	7.25391	7.50286	6.65318	6.26341	6.33617	6.54702	6.06915	7.20359	6.83147	7.21689	3.99089	4.52821	4.03589	4.45023
TSC22D4	Q9Y3Q8	6.22386	6.06194	7.20558	5.76825	6.62807	6.40147	5.49253	6.57048	6.2056	6.85793	7.16908	6.87449	6.26822	5.41202	5.31814	5.54233
PDIA3	H7BZJ3	6.31839	6.22999	6.07282	5.96425	5.99201	5.6704	6.51066	6.04586	6.05288	6.36818	5.98115	6.05765	6.55192	6.19824	6.93482	7.05079
JADE1	Q6IE81	6.26759	5.91996	6.68833	6.55995	6.42062	5.95855	6.43627	5.76638	6.7724	5.86668	6.5095	6.49321	6.2218	5.92842	6.13989	6.05045
ASRGL1	Q7L266	5.91745	6.0086	7.1245	4.99862	6.433	6.59339	6.60138	7.70736	6.57405	6.58191	6.41168	6.85776	5.6721	5.66025	5.71398	5.14397
TTC38	Q5R3I4	5.83607	5.87884	6.85547	4.99639	6.4531	6.72678	6.60655	6.82618	6.54551	7.3418	6.30791	6.54175	5.36246	6.42795	5.96254	5.33071
M0R2C6	M0R2C6	6.26489	6.33766	5.99589	6.9478	6.18225	5.87567	6.40092	6.35135	6.27375	6.12969	6.24079	6.03753	6.33002	6.00043	6.16226	6.4691
LZTS2	Q9BRK4	6.39855	6.33132	5.97965	6.18652	6.73034	6.18736	6.19732	6.08554	5.89962	6.00476	6.33103	6.00504	6.77417	6.1256	6.37956	6.38362
RPS19BP1	Q86WX3	6.26051	6.72396	5.95896	6.92634	5.83196	5.45086	6.55553	5.65029	6.51674	6.07526	5.99293	5.89164	6.24445	6.13532	6.89029	6.89497
ASB3	Q9Y575-3	6.55329	6.58598	6.84162	6.2885	6.13445	6.78629	6.32573	6.23862	6.32852	6.18769	6.50878	6.41069	5.44592	6.00503	5.65075	5.70813
KMT2E	Q8IZD2	6.7912	6.49328	5.93456	6.87399	5.91965	6.08082	6.25964	5.92106	6.01088	6.48976	5.74745	6.26749	6.07926	6.45655	6.73544	5.93895
FAM160B2	Q86V87	6.51983	6.43786	6.38315	5.47501	6.11346	6.44941	6.06262	6.30271	6.17527	5.89019	5.89117	6.3335	6.58083	6.77805	6.22997	6.37697
AFG1L	Q8WV93	6.6689	6.25563	5.93884	6.68513	6.50158	6.06531	6.0469	5.78695	6.24252	6.14978	5.87427	5.59941	6.62872	6.46835	6.39844	6.68926
ARRB2	P32121-4	6.0853	6.01268	6.9844	6.27061	6.21007	6.55822	6.55467	6.81546	6.7655	6.6189	6.41542	6.62753	5.52897	5.39575	5.4947	5.66182
MAP3K11	Q16584	5.9642	6.38026	5.93351	6.8461	6.14603	5.15778	5.92682	6.33192	6.19337	6.70805	5.96254	5.80606	7.12067	6.56062	6.83451	6.12757
RSBN1	Q5VWQ0	6.42235	6.40574	6.09912	6.63479	5.89298	6.02616	6.26691	6.04028	6.0101	5.91523	6.16726	5.97254	6.02118	6.81915	6.97448	6.33172
INO80D	Q53TQ3	6.30988	6.72496	5.96305	6.90695	6.34466	6.31144	6.48573	6.09374	6.19649	7.17738	5.99468	6.31137	5.59619	5.88956	5.77669	5.91723
UBE2T	Q9NPD8	6.30171	5.87518	6.87311	5.79915	6.28819	6.97305	6.29969	6.90489	6.4484	7.19755	7.15388	6.98551	5.19348	5.31759	5.11944	5.26919
DAD1	P61803	6.87482	6.43526	6.07861	6.16647	6.11647	6.07641	6.10932	5.97975	5.52915	5.64847	5.70731	5.94165	6.66538	7.66207	6.13089	6.87799
SERPINB8	P50452	7.92794	6.76821	7.14513	5.77427	4.77536	7.45715	6.77539	6.57534	7.47321	6.51924	6.54012	6.33864	5.57161	4.85457	4.27911	5.22472
ITPKB	P27987	6.31617	6.54316	6.23135	5.63754	6.02207	6.47057	5.98181	5.81003	7.31347	6.88582	6.14715	6.77549	6.24053	5.77928	5.76967	6.0759
SNTB1	Q13884	6.59342	6.2868	5.64767	6.60849	6.58074	5.67374	5.6624	5.97286	5.98696	6.02608	5.94073	5.8764	6.85566	6.42705	6.28408	6.67692
TLK2	Q86UE8-2	5.2617	6.06436	6.2154	5.84523	5.68783	6.10242	5.97761	5.84443	6.10984	6.42138	6.217	6.17575	7.03808	7.10854	7.10783	6.82259
SLC20A1	Q8WUM9	5.40797	5.75683	5.42828	6.13435	5.47596	5.41254	6.06626	5.75865	6.07154	5.578	5.86237	5.47838	7.91867	7.62108	8.27081	7.75211
OSBPL2	Q9H1P3	6.07316	5.72833	6.5661	5.88016	6.34025	5.84746	6.38963	5.75607	6.68596	6.6131	7.2207	6.5901	6.38574	6.27327	5.88176	5.76819
METTL25	Q8N6Q8	6.60514	6.37973	7.31999	6.64575	6.20746	6.57488	6.20342	6.6052	6.53401	6.59212	6.66883	6.35071	5.43485	5.17193	5.24995	5.45602
ERI1	Q8IV48	5.73835	6.59475	6.69474	5.30471	6.4161	6.16559	6.52022	6.56431	6.73252	6.52744	6.44633	6.25205	6.10165	6.04338	6.13792	6.16494
SSH1	Q8WYL5	6.30729	6.56426	6.59965	5.98462	6.55832	5.77971	6.11812	5.90826	6.26858	5.91797	5.28192	6.27157	7.03721	6.49178	6.24318	6.66757
FMC1	Q96HJ9	6.14894	6.41387	5.89938	6.25562	6.39237	5.92151	6.48867	5.74806	6.10807	6.17527	6.26179	5.70981	6.61319	6.20232	6.83348	6.82767
ITPK1	Q13572	6.53952	6.17152	7.25125	6.15968	5.85328	6.74329	6.4388	6.33237	6.68383	6.57871	7.29757	6.30702	5.25453	5.3325	5.70602	5.35013
RGS19	P49795	5.88854	6.18734	6.31876	6.74243	6.1657	6.11931	6.29497	6.09885	5.84802	6.12436	6.3243	6.23114	6.16745	6.4539	6.59591	6.43901
SH2B3	Q9UQQ2	6.47951	5.86744	6.42366	5.71518	5.99806	6.79422	6.2856	6.71984	6.02128	6.01214	6.07102	6.44066	6.23075	6.37451	6.49805	6.06809
RBL2	Q08999	5.83671	5.81933	6.18759	5.52258	5.81946	6.31665	6.24814	5.91131	5.85769	6.26833	6.7094	6.38228	6.29767	7.314	7.02793	6.48092
POLK	Q9UBT6	5.31364	6.30352	6.28795	5.38431	5.58997	6.41442	5.35444	6.28652	6.8706	5.95396	6.69537	6.50026	6.55173	6.30907	7.09499	7.08924
POC5	Q8NA72	6.58302	5.87272	7.30648	6.40802	6.92139	7.15586	6.7926	6.43606	7.14071	5.48164	6.46455	5.83241	5.44202	4.99795	5.23866	5.92591
PRKCH	P24723	6.77774	7.12462	7.33017	7.79366	6.00222	6.22434	6.5329	5.99797	6.60605	6.8284	6.93238	6.77604	4.53951	5.00311	4.98042	4.65046
ELP6	Q0PNE2	6.22218	6.17295	6.8214	6.00843	6.44421	6.71004	6.26444	6.51099	6.88992	6.72442	6.37199	6.37133	5.52111	5.7551	5.55241	5.65907
LATS2	Q9NRM7	6.35518	5.94988	7.03156	6.94863	6.07922	6.58039	6.03477	6.36327	6.5872	6.50552	6.54078	6.89444	5.39497	5.85172	5.40108	5.4814
VPS25	Q9BRG1	6.79524	5.09812	6.42809	4.96978	5.79249	6.36069	6.51142	6.34535	6.37061	6.85527	6.39752	7.04587	5.93569	6.54443	5.97403	6.57538
RCN3	Q96D15	8.25357	7.24979	6.66508	7.07225	6.46946	6.17157	6.31575	7.2871	6.94203	7.29524	6.71528	7.39286	4.05877	4.15587	3.5781	4.37728
FKBP7	Q9Y680	6.72036	6.12633	5.94839	6.30614	6.46156	5.49927	6.13875	5.73538	6.44968	5.90089	6.49898	6.29684	6.93359	6.44081	6.47929	6.06374
HIRIP3	Q9BW71	5.10034	5.20525	6.18464	4.82207	5.65507	6.38424	5.63148	5.95247	6.53312	6.06644	6.32165	6.5742	7.58005	7.36913	7.38035	7.23949
APRT	P07741	6.02547	5.47094	6.53448	4.77144	6.2562	6.37908	5.55512	7.05749	5.87474	6.76733	6.14544	7.40165	5.97769	6.85766	6.3235	6.60176
SLC41A3	Q96GZ6-9	6.26991	6.40361	6.03032	6.72842	6.051	5.55556	6.38538	6.59431	5.90805	6.17842	5.87541	5.81885	6.63901	6.38075	6.7347	7.11632
ACSS2	Q9NR19-2	6.14395	5.46087	6.97949	5.67519	6.42317	6.54811	5.62423	6.31165	6.2846	6.59348	6.43184	6.45288	5.64825	7.02039	6.64804	5.75386
PRPF40B	Q6NWY9	6.31826	6.57585	5.99602	6.08636	6.45583	6.28108	6.24164	5.89863	6.37812	5.56699	6.06138	5.58295	7.05382	6.36679	6.81137	6.32492
MAFG	O15525	6.58605	6.77994	5.56468	6.5722	6.36434	6.39312	6.57368	6.4244	5.95532	6.10336	5.46108	6.06539	6.232	6.30718	6.27203	6.34522
JUNB	P17275	6.46029	6.2313	6.07133	6.72246	6.4407	5.93901	5.89841	6.49131	6.44203	5.25872	6.17323	5.29386	6.87863	6.49721	7.01264	6.18888
ACTR3B	Q9P1U1	6.15619	6.21795	6.88238	6.01443	6.2459	6.15255	6.13592	6.67015	5.99318	6.24596	6.72542	6.78866	5.73005	6.22521	5.77757	6.03848
LPGAT1	Q92604	6.54586	6.56836	5.94819	6.78161	6.38842	5.73122	6.28701	6.03608	5.88874	6.08172	5.90157	5.98468	6.60643	6.20093	6.28612	6.76305
EPN2	O95208	6.46431	6.16753	6.24022	6.29676	6.46025	6.08295	6.48557	6.4003	6.06605	6.13268	6.45369	6.37598	6.73956	5.9872	6.0046	5.64235
UBP1	Q9NZI7	6.52237	5.7147	5.74537	5.82694	6.77069	6.81342	6.17179	6.28245	6.03605	5.72156	6.71299	6.20826	6.45045	6.08274	6.97372	5.96649
MYEF2	A0A0A0MQW0	6.16366	6.4131	5.88827	6.73438	6.04882	6.45635	6.25869	6.26746	6.09188	5.92638	5.93439	5.917	6.5741	6.17516	6.7111	6.43926
FAAP100	Q0VG06	5.80481	5.93915	6.09791	6.2618	5.91593	6.16097	5.96417	6.4116	6.02063	6.18948	6.45502	6.21884	6.63889	6.8832	6.26731	6.77029
ZNF768	Q9H5H4	6.21942	6.32414	5.66371	6.15866	6.08738	6.30418	6.59387	5.75299	6.21291	6.24489	6.25575	5.76251	6.79795	6.52035	6.64013	6.46115
ZNF595	Q8IYB9	6.40778	6.45661	6.63225	6.76919	6.31594	6.28687	6.7941	5.7508	6.20453	6.17508	6.46715	6.43549	5.75172	5.5593	5.91356	6.07963
ZNF667	Q5HYK9	5.87662	6.23445	5.2116	6.44465	5.92725	6.42746	6.06907	6.0354	5.61375	6.31334	5.41537	5.75737	7.18904	7.53926	7.23865	6.70675
ZNF567	Q8N184	6.43197	6.56487	5.69691	6.68306	6.04699	6.04739	6.18276	6.48467	5.57457	5.98444	5.74727	5.86742	6.47461	6.79799	7.06303	6.35205
ZNF146	Q15072	5.53398	5.45998	5.78329	5.87173	5.9008	6.12764	6.1711	5.50733	6.10535	6.00733	5.89412	5.83191	7.75719	7.18678	7.48217	7.37929
ZNF569	Q5MCW4	5.94031	6.44317	5.86662	7.24589	5.86824	6.35776	6.21883	6.62867	6.07797	6.89839	5.85235	6.40011	5.56968	6.25862	5.46555	6.37723
ZNF316	A6NFI3	5.47067	6.51382	6.60981	6.64692	6.81765	6.15617	5.89422	5.90956	6.81997	5.47924	6.56104	6.48328	6.03438	6.56797	6.69428	6.26101
XYLB	O75191	6.43238	5.7705	6.8431	5.47024	6.78152	6.49127	5.99678	6.84189	6.21275	6.32049	6.99498	7.12975	5.74016	5.3513	6.236	5.38687
RRAGD	Q9NQL2	6.15281	7.48041	6.11261	6.01075	6.30519	6.39852	5.85189	6.10337	6.33718	7.09278	6.91121	6.20579	6.48201	6.19726	5.69124	5.66698
MPG	P29372	5.89851	6.49835	6.73033	6.38476	5.96494	5.89568	6.03603	6.06371	6.47006	6.5563	6.84683	6.70911	5.67792	6.27516	5.94251	6.04981
CFAP97	Q9P2B7	5.42337	6.29596	8.39558	6.66689	5.46246	6.47992	6.19142	7.16351	6.47298	6.85642	6.77022	7.09067	4.93141	4.98959	5.57199	5.23759
DENND4B	O75064	6.12392	5.45632	6.20388	6.29096	5.67597	6.12026	6.4487	5.83138	5.96574	6.88481	6.22429	6.29297	6.45336	6.4032	6.93795	6.68629
NDUFAF6	Q330K2	6.64507	6.55252	6.31335	6.97446	6.13821	5.43856	6.28194	5.97963	6.48038	5.93975	6.02932	5.73524	6.56295	5.87862	6.79958	6.25041
GOLGB1	Q14789	5.79171	6.30046	6.42361	6.47548	5.94979	5.74506	6.3908	6.15858	6.22139	6.26603	5.43953	6.34768	6.70528	6.32821	6.78083	6.67558
DOHH	Q9BU89	6.25793	4.25098	5.68506	4.98607	5.66919	4.83549	5.95584	6.80029	5.05694	5.54954	5.17638	7.34219	5.79088	9.37998	7.18823	10.075
CDCA7	Q9BWT1	6.41607	7.24964	7.16468	8.41757	6.3059	6.4391	7.27511	6.39602	7.07239	7.15178	6.93479	6.4894	4.39299	4.26486	3.72748	4.30221
ZNF655	Q8N720-3	6.17887	6.42433	5.95467	7.01076	6.36695	6.00975	6.41375	6.23219	6.29832	6.2	6.15673	6.29168	6.43859	5.84436	6.2092	5.96985
RBBP8	Q99708-2	5.82429	6.21639	6.11337	6.58464	6.23861	5.67667	6.30262	6.13976	6.13541	5.6763	6.76202	6.51072	6.45027	6.40381	6.46238	6.50275
C2CD2L	O14523-2	6.30397	5.99728	5.84565	6.40526	5.92312	6.07883	6.36294	6.1095	5.92389	6.1542	6.36628	6.1319	7.14744	6.35104	6.54452	6.35419
TCF3	P15923-3	5.98342	5.95026	6.9348	5.72068	5.78098	6.22822	5.87113	6.47912	6.41476	6.66094	7.76278	7.35666	5.72583	5.8066	5.81295	5.51087
OPLAH	O14841	5.92352	6.02652	6.29812	5.35558	5.91616	6.90707	6.53328	6.36201	5.95655	6.12669	6.60191	6.62499	6.08817	6.66105	6.46627	6.15211
TCTN3	Q6NUS6	7.1299	7.21413	6.42851	7.0806	7.02185	6.0613	6.61211	6.25781	6.14393	6.85737	6.50362	6.84388	4.8834	4.68829	4.92622	5.34708
POLR2D	O15514	6.55698	5.60406	6.5262	5.95653	6.05848	5.83506	6.32972	6.22976	5.67827	6.76813	6.63346	7.00913	5.26025	6.47276	6.48702	6.59417
TAF10	Q12962	6.14815	5.29366	6.06715	5.11753	6.22173	4.78061	5.0495	6.7105	5.08829	6.86248	6.2679	7.06998	6.14707	7.93908	7.43743	7.79894
NPM3	O75607	6.43348	6.49552	6.78374	6.59023	6.3047	6.27163	5.94211	6.37586	6.20273	5.76129	7.06988	6.54468	6.2752	5.57184	5.46577	5.91135
RRAS2	P62070-4	6.3963	6.40716	6.51059	6.451	5.88912	5.86704	5.83937	6.16237	6.14432	6.41877	6.14689	6.33973	6.86053	5.77588	6.59181	6.19912
METTL17	Q9H7H0-3	6.60362	5.95522	6.49449	6.62904	6.54006	6.20007	6.3549	5.7453	6.28724	6.27095	6.24816	5.98302	6.05582	5.71508	6.68598	6.23104
SATB1	Q01826-2	6.41714	6.78105	6.18842	6.95092	6.12474	5.76502	6.57162	5.90003	6.48178	5.64729	5.45648	6.42233	7.06698	5.6268	5.8342	5.76519
ARL6IP1	Q15041	6.39321	6.29773	5.64532	6.25492	6.49959	6.59068	6.3839	5.66526	5.7921	6.45975	5.86384	5.42929	6.54955	6.98207	6.42557	6.7672
BET1	O15155	6.03733	5.84952	6.27962	6.58818	5.96001	5.86889	6.09262	5.66613	6.19973	5.85892	6.08712	6.27154	6.72605	6.7564	7.13279	6.62515
TIMM9	Q9Y5J7	6.54952	5.46421	5.77606	5.58361	4.62252	4.49238	5.72669	5.41243	4.82915	5.5817	4.8684	5.27464	4.67461	10.703	8.70906	11.7321
EXOSC6	Q5RKV6	6.54936	6.2034	6.25546	6.09328	6.57604	6.0701	6.39993	6.26276	6.03754	5.87019	6.22685	6.33768	6.97343	5.58179	6.20778	6.35439
PDZD11	Q5EBL8-2	5.68465	5.02301	6.45495	5.1453	6.20869	5.64423	5.996	7.38027	5.6049	6.90848	6.47708	7.04426	6.04961	7.03089	6.45984	6.88782
FRMD8	Q9BZ67	5.92335	6.81228	5.4158	6.51686	6.08578	6.34124	6.18831	5.65521	6.42592	6.54027	5.70367	6.30878	6.13602	6.5895	6.69742	6.65959

NIPA2	Q8N8Q9	6.91913	5.91734	6.70485	6.26136	6.21398	5.71487	5.89937	6.80409	6.339	5.83126	5.44267	5.98713	6.07268	5.64911	6.66111	7.58206
BCOR	Q6W2J9	7.29764	7.00336	6.8688	7.14818	6.02778	6.37641	6.60058	5.87888	6.54827	6.08898	6.64963	6.26705	5.63827	4.92331	5.16359	5.51925
RPP25L	Q8N5L8	5.9155	6.13702	6.38213	6.34043	5.98029	5.63499	6.05525	5.91472	6.78621	6.63152	6.88369	6.7939	6.68539	6.17904	5.51813	6.16179
THAP11	Q96EK4	6.71553	6.59372	6.00433	6.88537	6.46451	6.26236	6.26472	6.24159	6.35494	6.63234	5.90041	6.1559	5.99779	5.77451	5.85121	5.90077
CLCN5	P51795	6.28439	6.22586	7.50129	6.38774	5.90287	7.1247	6.68898	6.16614	6.33205	6.37196	6.79445	5.74051	5.90904	5.28062	5.73856	5.55085
UBE2R2	Q712K3	6.23062	6.54241	7.02422	5.90465	7.12796	6.41995	6.18134	7.2436	6.84577	6.5569	6.75044	6.57724	5.57356	5.13469	5.03373	4.85291
CCDC12	Q8WUD4	6.4826	6.22074	5.90652	6.28413	6.1713	6.19256	5.97968	6.19146	6.21044	6.19005	6.36292	5.97092	6.94869	6.24867	6.2595	6.37982
AGO1	Q9UL18	5.77537	6.38565	6.30342	6.0549	6.70992	5.94366	6.18409	5.73126	6.52913	6.28953	6.49701	6.64001	5.72303	6.13885	6.62604	6.46813
SPRED1	Q7Z699	5.90884	6.60526	6.34234	6.48835	5.59328	6.45726	6.75237	6.09948	6.19405	6.52226	5.7945	6.23642	6.50358	6.05949	5.83301	6.60949
KAT8	Q9H7Z6-2	6.46772	6.60515	5.91794	6.80741	6.41152	6.17524	6.23267	5.9911	6.29001	6.00986	6.10579	6.44205	6.24509	5.79815	6.38078	6.11952
OTULIN	Q96BN8	5.96111	5.8741	7.1041	6.12812	6.1535	6.9747	6.37398	6.88734	6.41719	6.28267	6.18462	6.48993	5.95664	5.50045	6.06892	5.64263
CORO1A	P31146	6.46773	6.61623	6.28771	6.14298	6.07971	6.23325	6.36642	6.36984	5.93043	6.72526	6.42091	6.57229	6.02902	6.36033	5.75752	5.64037
APPBP2	Q92624	6.26872	7.10831	6.86083	6.51198	6.24471	6.87828	6.44793	6.22559	6.30785	6.58605	6.87791	7.55971	5.38809	4.80003	4.97272	4.9613
LPIN2	Q92539	6.54175	6.73887	6.78604	6.28916	6.43973	6.88796	6.14579	6.64543	6.73857	6.72098	7.03676	7.20117	4.96406	5.28862	4.86551	4.70959
AUH	Q13825	6.21476	6.33331	6.53651	6.11566	6.29354	6.076	6.56117	5.86329	5.57474	6.09803	6.42716	5.66898	7.13691	6.53644	6.60308	5.96041
DNAJC18	Q9H819	7.39333	7.14629	7.3454	8.47483	6.90899	6.85175	6.85389	6.64534	6.74456	7.22021	7.0433	6.93723	3.55127	3.661	3.27467	3.94795
ATE1	O95260-2	5.77698	6.00623	6.78195	5.72142	5.92906	6.44756	6.44847	6.1363	6.21365	6.3616	6.15661	6.3137	6.40433	6.46715	6.4976	6.33739
AKT3	Q9Y243	6.01966	6.31758	6.81528	6.43618	5.66561	6.72604	5.96607	6.64387	6.28505	6.40768	6.74054	6.54707	5.33493	6.10172	5.91853	6.07419
SMAD3	P84022	6.69203	6.22305	6.41859	5.83733	6.40999	6.44041	6.0515	6.47506	5.71593	6.49476	6.07136	6.3661	6.16507	6.99346	6.0199	5.62545
OIP5	O43482	6.6738	6.55188	6.82677	6.67975	6.34782	6.4103	6.30221	6.39335	6.68262	6.8214	6.65696	6.80846	5.11394	4.95367	5.37658	5.40047
PDK1	Q15118	6.476	6.72229	5.85012	6.39209	6.77749	6.00418	6.48932	6.39023	5.6518	5.50916	5.96662	5.72854	6.37848	6.72602	6.69329	6.24436
DBI	P07108-5	5.60097	5.43027	5.87867	5.08938	5.95004	5.19839	4.86254	6.44054	4.40574	5.30356	4.97358	4.62607	4.36912	13.8858	7.34022	10.6451
APBB1	O00213	6.13871	6.1763	6.23643	5.98043	6.86649	5.89502	6.20775	6.1541	6.21965	6.70106	6.83684	6.05496	6.5567	5.71455	6.04526	6.21575
RAP2B	P61225	5.91164	6.38508	5.98269	6.2816	5.81709	6.18036	6.11079	5.94644	6.15195	6.05162	6.07575	6.25616	6.5754	6.67041	6.77074	6.83227
RAP2C	Q9Y3L5	6.37435	6.73184	6.66972	6.87498	6.56688	6.55524	6.17237	6.25236	5.1833	6.2222	6.04232	5.95169	6.23065	6.64781	5.86473	5.65957
KLC2	Q9H0B6	6.12927	6.32431	5.85265	5.82082	6.00591	6.55724	6.68114	6.59094	7.47915	6.27853	6.42379	6.58134	6.46663	5.57864	5.46006	5.7696
SLC30A7	Q8NEW0	5.7109	6.2219	5.83979	5.82958	6.63465	5.68272	6.00409	5.90508	5.90459	6.29559	6.0403	6.38913	6.90545	6.71546	7.34292	6.82008
EXOSC4	Q9NPD3	6.27503	6.17979	6.18155	6.5138	6.59825	6.01483	6.05408	5.94675	6.47343	6.42443	5.92676	6.09152	6.64725	5.89053	6.60069	6.18131
HUS1	O60921	5.51153	5.69236	6.19919	5.7068	5.59708	5.84278	5.53755	5.57296	5.95983	5.99773	6.53744	6.0985	7.12963	7.57134	7.73281	7.31247
FCHSD1	Q86WN1	6.26605	6.21057	6.98498	5.93061	6.08471	6.65783	6.02178	6.37095	6.63775	6.37865	6.45571	6.59449	5.93162	5.76859	5.80454	5.90117
ING3	Q9NXR8	6.21506	6.03891	6.18613	6.99945	6.4407	5.94646	6.44105	6.62021	6.07019	6.40463	6.66541	5.75915	6.1834	5.97879	6.05352	5.99694
ZFAND2B	Q8WV99	6.39804	6.3961	6.5227	6.39049	6.04019	6.24452	5.93013	6.48961	6.81399	6.37254	6.21237	7.01731	6.22796	5.54499	5.33423	6.06482
PNMA1	Q8ND90	6.79105	5.62184	6.07794	5.35829	5.2668	5.37547	6.0561	6.98777	5.9007	7.3047	6.37207	8.18046	4.62117	6.96246	6.17593	6.94724
ZHX2	Q9Y6X8	6.40005	6.37423	5.9125	6.79223	5.64957	6.10539	6.49048	5.76178	5.95167	6.20348	5.80146	6.27255	6.70081	6.17532	6.44074	6.96775
HSD17B7	P56937	6.98367	6.6219	6.99015	6.48229	6.59483	6.17212	6.0019	5.18671	6.17141	5.84218	6.01253	5.68981	6.64323	5.96566	6.67591	5.96569
MTMR12	Q9C0I1	6.48819	6.21581	6.3866	6.23076	6.49307	6.28315	5.86889	6.404	6.73492	6.5682	6.07349	6.86788	6.15885	5.58175	5.54678	6.09765
TMEM199	Q8N511	6.81582	6.84406	5.36226	6.74988	6.48894	6.68304	5.8759	6.23733	6.46183	5.6889	6.13824	5.72913	6.47599	5.76341	5.7256	6.95967
DTWD1	Q8N5C7	5.83713	6.45415	7.27295	6.06279	5.86179	6.46528	6.14544	6.42958	6.49603	5.94781	6.79861	7.35984	5.3685	5.8679	5.81683	5.81537
TRMT10A	Q8TBZ6	5.57094	5.76903	6.048	5.46258	5.35467	6.1876	5.71334	6.23657	5.74065	6.03232	6.51477	6.65797	6.50874	7.06224	7.67666	7.46392
TBCEL	Q5QJ74	5.74595	5.96142	6.23271	5.76066	6.60722	6.42981	5.95241	7.12041	6.43259	6.68154	7.03038	7.09701	6.19703	5.82123	5.60961	5.32002
TMEM132A	Q24JP5-2	6.56988	6.82981	6.32827	6.73908	6.27483	6.32471	6.60687	6.41274	6.8275	6.45519	6.51024	6.42845	5.46318	5.49543	5.44331	5.29052
RTL8A	Q9BWD3	5.75298	4.56474	5.8609	4.37437	5.74147	6.26329	5.65982	6.88984	5.55987	5.53734	6.21036	7.19236	6.52566	8.53922	7.19307	8.13471
CENPT	Q96BT3	6.46712	6.44846	5.6917	6.92511	6.08142	5.53769	6.44927	5.87295	6.19739	6.35299	6.2134	6.34715	6.81004	6.01921	6.23273	6.35335
AP5M1	Q9H0R1	6.9032	6.71031	7.13699	6.55222	6.2172	6.02211	5.59101	6.29718	6.34894	6.67832	6.82326	6.42165	5.73725	5.61818	5.43144	5.51075
CCDC51	Q96ER9	6.20412	5.8695	6.0642	6.7883	5.69551	6.44311	6.46324	5.71419	6.14545	6.27268	6.30462	6.37704	6.66164	5.86543	6.22027	6.91068
TTC39C	Q8N584	6.31783	6.03072	7.19928	6.69123	6.6222	6.06818	6.35613	6.37077	6.58226	5.9301	6.83323	6.82509	5.70311	5.60609	5.10744	5.75635
AMBRA1	Q9C0C7-5	5.53149	5.69371	6.27223	5.92581	6.01611	6.52863	6.3434	6.19133	6.19757	6.42624	6.44456	6.43502	6.55066	6.18353	6.8094	6.45032
PRTFDC1	Q9NRG1	6.34691	6.23136	6.86692	6.56291	6.47864	6.68625	6.2342	6.95618	6.47325	6.1849	6.4317	6.20966	6.21912	5.68243	5.89351	5.54207
PPT2	Q9UMR5-3	6.55346	6.81949	6.11205	5.85343	6.8164	6.20953	6.16041	6.35487	5.85569	6.07786	6.16991	5.79241	6.79485	6.23864	6.1911	5.99991
FANCG	O15287	6.73498	6.41115	7.04711	6.45156	6.15384	6.04398	6.19873	6.90919	6.27025	7.14242	6.86312	7.24303	5.21072	5.00141	4.85762	5.4609
LIPT1	Q9Y234	6.4648	6.56865	6.03045	7.43639	5.93195	5.60346	5.80079	5.5129	6.57528	6.51459	6.35294	6.14645	6.38233	5.91317	6.41429	6.35157
SCRN3	Q0VDG4	6.01551	6.08824	6.23027	5.96691	6.17782	6.7317	5.99246	6.64326	6.19924	6.95068	6.79282	7.16375	5.2671	6.089	5.83346	5.85776
PROSER3	Q2NL68	6.80184	6.85225	6.75099	5.16387	6.73157	6.67363	6.27458	6.68794	6.32637	6.35087	7.34446	7.33298	5.57459	5.10169	5.13397	4.89839
ADO	Q96SZ5	6.68935	5.77996	6.70069	6.04689	6.07667	6.10087	5.75836	6.48868	6.62729	6.59944	6.17585	6.84579	6.23653	6.22786	5.91249	5.7333
RHBDD1	Q8TEB9	6.33931	5.44469	6.08545	6.28448	5.91879	6.19173	6.08734	5.73747	5.91354	6.64898	5.97497	5.642	6.91859	6.92623	6.37828	7.50815
ASDURF	L0R819	6.42601	5.73423	6.62128	5.23585	6.46	6.15186	5.89581	6.79713	6.32382	6.9425	7.165	7.04195	5.94605	5.82467	5.5682	5.86564
GTPBP3	Q969Y2-2	6.35698	6.37907	6.11296	6.63962	6.2798	6.35915	5.91327	6.00054	6.35065	5.9208	6.09242	6.40152	6.72139	5.91354	6.40354	6.15474
PRMT3	O60678	6.26698	6.51678	6.83899	6.25506	6.23125	6.51967	6.32264	6.79023	6.34131	6.35497	6.69319	6.93172	5.55124	5.45316	5.48312	5.6507
CEPT1	Q9Y6K0	6.25128	6.33823	6.15166	6.5827	5.88732	6.15792	6.32937	5.72739	6.17209	6.26037	5.85722	5.68757	6.62174	6.34507	6.94682	6.68326
HBS1L	Q9Y450-2	6.3182	6.13039	6.9598	6.14735	5.87751	6.31648	5.94574	6.13529	6.88888	5.88553	6.37938	6.67551	6.62083	5.86929	5.87228	5.97753
CDT1	Q9H211	6.84125	6.98126	6.93741	7.12871	6.33373	6.23614	6.74612	7.09492	7.09557	7.43444	7.96845	7.35712	4.17463	4.1673	3.44687	4.05609
IFIT3	O14879	6.97643	6.71752	6.26254	6.01572	7.05401	6.76777	6.15895	6.92714	6.50902	6.38654	6.45879	6.24948	5.58917	5.79395	5.08989	5.04307
GCA	P28676	6.60267	5.55799	6.48862	4.9067	6.46308	6.31702	5.66169	6.81478	5.7827	6.85431	6.86357	6.9974	6.09025	6.38354	6.07871	6.13695
TRNAU1AP	Q9NX07	5.48319	5.71206	5.84827	5.55264	6.24479	6.49686	5.90207	6.60234	6.25428	6.6953	6.67342	6.45317	6.22234	6.78061	6.34223	6.73644
GNA11	P29992	6.51609	6.90626	5.90429	6.44935	6.44318	6.39136	6.31618	6.442	6.38313	6.24334	6.32848	6.07553	5.52743	5.63217	6.3449	6.09631
GATD1	Q8NB37	5.98218	5.77118	6.71065	5.39843	6.38495	6.5394	5.88729	6.54129	6.48606	6.75268	6.89636	6.88876	5.99916	5.8958	5.92354	5.94226
KDM4D	Q6B0I6	6.09562	7.17042	5.83907	6.98573	6.17214	5.99871	6.52307	6.20118	6.44114	6.60884	6.32474	5.83039	6.50337	6.04436	6.3038	5.85742
ACYP1	P07311	6.09053	5.8963	6.10371	5.03183	7.07982	6.58344	6.13439	7.68073	6.23587	6.79762	7.08609	6.28671	5.96071	5.78721	5.80549	5.43956
SPRED2	Q7Z698	6.74643	6.77412	5.41834	6.42524	6.70551	6.49274	5.93526	6.2277	6.11836	5.77057	7.10769	5.90074	6.26113	5.80677	6.6076	5.70179
TSR3	Q9UJK0	7.18593	6.79782	6.81856	6.71338	5.88008	6.38444	6.6396	6.39204	7.54018	7.00947	6.93365	6.73864	4.65011	4.97731	4.55199	4.78678
DPY19L3	Q6ZPD9	6.21984	6.57727	5.59995	6.90937	6.05343	5.91807	6.50539	5.65682	5.99882	6.23023	5.97629	5.78541	6.73093	6.14877	6.95518	6.73424
MAPK6	Q16659	5.33351	5.1986	5.8428	5.09139	6.51621	6.85893	6.50948	7.31063	5.28468	5.28895	5.87847	5.70859	7.65222	7.30833	6.77401	7.44321
PCGF3	Q3KNV8-2	6.38025	6.48016	6.47045	6.43337	5.93863	5.89468	6.24771	5.75027	5.96623	6.16474	6.64329	6.01563	6.54279	5.99489	6.7501	6.3268
ZNF579	Q8NAF0	5.87951	6.58469	5.82309	6.39393	6.34774	5.84891	5.89775	6.481	5.90711	6.7542	6.27616	5.86985	6.5541	6.39209	6.53562	6.45425
PLA2G15	Q8NCC3	6.66208	6.47265	5.90804	6.2502	6.50674	5.93391	6.30854	6.04493	6.40508	6.19254	5.99759	5.79382	6.75789	6.21816	6.32442	6.22341
SAMD8	A0A5F9ZGS6	6.6048	6.78308	6.55948	7.23944	6.62011	5.95623	6.41546	4.9417	6.13336	6.03934	5.86435	5.72537	6.26915	5.80969	6.78165	6.25676
CGNL1	Q0VF96	6.16628	6.33125	6.31981	6.27926	6.22194	6.24481	6.71746	6.40594	6.31982	6.45781	6.11621	6.2406	6.16797	5.74472	5.90323	6.36289
MRPL32	Q9BYC8	6.76681	6.42022	6.06048	6.60809	5.85586	5.93549	6.26128	5.07215	6.13648	6.35891	6.28041	6.43947	6.4988	6.2797	6.56572	6.46014
SPIN2B	Q9BPZ2	6.13051	6.71253	5.66274	7.21885	6.12673	6.02844	6.17082	6.20219	6.78109	6.51371	6.11375	6.35819	5.64037	5.87385	6.3231	6.1431
HLA-B	P01889	6.10198	6.73702	6.20636	5.93118	6.73724	6.24078	6.4721	6.58684	5.9627	5.94424	5.97332	5.95409	6.24288	6.59489	5.6962	6.61817
HLA-C	A0A140T9M0	5.93108	6.7991	6.17022	6.30995	6.29294	6.94824	6.35147	6.39634	5.84942	6.33341	6.52285	6.90002	5.28219	6.29865	5.48548	6.12866
GLCE	O94923	5.98072	6.30169	6.14991	6.05737	6.11058	6.05861	6.68978	5.85232	7.16986	5.94298	6.11892	5.75682	7.211	5.79567	6.36492	6.43887
TAF6L	Q9Y6J9	6.14857	6.38446	6.36624	6.34408	5.82514	6.1631	6.0935	6.38274	6.20705	6.6542	5.87979	6.183	6.77347	6.03986	5.97545	6.57936
MOB3A	Q96BX8	6.57419	6.85868	6.89652	6.22529	6.3026	6.94471	6.23709	6.92168	6.27138	6.62242	6.40337	6.62843	5.25042	5.30626	5.27354	5.28343
NUDCD2	Q8WVJ2	6.06023	5.5808	6.23203	4.95795	6.796	6.74098	5.66567	7.42241	6.18943	6.87468	6.56249	6.92357	5.72618	6.73283	5.35937	6.17537
DONSON	Q9NYP3	6.12427	6.31422	6.67461	6.38739	5.8241	6.20522	5.88394	5.53322	6.74745	6.23478	6.92913	6.44772	6.14148	6.07151	6.09241	6.38857
APOL2	Q9BQE5	6.76959	6.51418	6.22043	7.20415	6.52564	5.91353	7.28354	5.68311	5.84688	5.94228	6.38114	7.36811	5.71684	6.04707	5.20896	5.37454
ZNF787	Q6DD87	5.74342	6.31898	5.96457	5.71423	5.86752	6.46877	6.22216	6.34497	5.90409	6.13188	6.69386	6.21406	6.70879	6.65017	6.49946	6.55306
MRPS18C	Q9Y3D5	6.1605	6.13781	5.73118	6.31712	6.39884	5.80137	6.09485	6.43723	5.8275	6.84371	6.43862	6.63305	6.61758	6.2885	6.10271	6.16944
BIN1	O00499	6.85389	6.08165	7.00238	6.50124	5.82642	6.22556	6.2816	6.69158	5.63087	6.90769	6.56624	6.93004	6.00208	5.65455	5.30309	5.54114
CBFB	Q13951	6.46516	6.00541	6.78449	5.67996	6.47132	6.85446	6.27584	6.59441	5.69961	6.44221	6.27866	6.27787	5.79411	5.72823	5.73338	5.62279
AMOTL1	Q8IY63	6.47007	6.34816	5.4818	6.0343	6.67296	6.10542	5.79328	6.40695	6.47998	6.44496	6.4778	6.21663	6.94971	5.9983	5.81696	6.30273
DTNA	Q9Y4J8	6.32948	6.27228	6.57744	6.179	5.92893	6.15785	6.46525	5.98362	5.83812	6.11597	6.20027	6.26702	6.33482	6.94805	6.50434	5.89757
DTNB	O60941	6.14375	6.42664	6.33769	6.63153	6.41873	5.70272	6.16955	6.26075	7.02447	5.85553	5.94274	5.93366	6.21813	6.07214	6.71979	6.14217
MRTFA	Q969V6	6.26699	5.89487	6.17638	5.74961	5.99467	6.49019	5.62268	5.80204	6.53244	6.60767	6.93111	7.03382	6.17978	6.18926	6.04818	6.48031
UBE2J1	Q9Y385	6.38695	6.40959	5.92315	6.93348	6.57156	6.25409	6.67548	6.48975	6.25299	5.86048	5.90724	6.34047	6.23973	5.55919	5.96887	6.22698
ATP11B	Q9Y2G3	6.04853	6.24897	6.36753	6.6163	5.84985	6.17685	6.45136	6.22142	6.10604	6.67168	6.7384	6.39856	5.97699	5.87372	6.37186	5.88194
BET1L	Q9NYM9	6.1973	5.15526	5.28298	5.41906	5.98197	5.61834	5.56485	6.06579	5.33104	5.57862	5.61601	6.95963	6.31665	8.3809	7.86842	8.66318
TRIP12	Q14669-2	6.95234	6.25697	6.18426	7.24813	6.60655	5.34122	5.46784	7.06872	6.48698	5.49628	6.18718	6.4259	6.27193	5.83719	6.63734	5.53118
SCAMP4	Q969E2	6.05024	5.79006	6.61153	5.93825	5.58874	6.09754	6.40451	5.66581	6.70535	6.37331	6.26201	6.83081	5.87554	6.4602	6.53121	6.81489
MLLT10	P55197	6.10991	6.91755	6.08039	7.26993	5.7374	5.9724	5.85386	6.00694	5.88265	6.00523	5.80724	6.17625	5.49105	6.87136	6.6125	7.20535
PSMB8	P28062	6.08669	6.16413	6.29768	6.04641	6.52788	6.43688	6.14216	6.20034	6.06923	6.42127	6.38351	5.91553	6.70764	5.93863	6.54864	6.11337
TBC1D31	Q96DN5	6.89469	6.40295	6.6258	6.31807	6.01575	6.48839	6.30682	6.18498	7.39941	6.77152	5.91346	7.03001	5.14763	5.54464	5.13815	5.81772
KANSL2	Q9H9L4	5.81512	6.21147	5.86455	6.82	6.00303	6.20104	6.77337	6.27718	6.21877	5.96697	6.17974	6.00151	6.20674	6.62378	6.42024	6.41649
CLTB	P09497-2	6.51367	5.12899	6.23755	5.37801	5.81109	4.48103	4.85932	6.5077	5.10274	6.64401	6.0817	7.23666	6.00525	8.03188	7.81478	8.16563

TUBGCP5	Q96RT8	6.40165	6.79337	7.24893	6.72652	6.30135	6.74327	6.03088	6.80866	6.61636	6.82037	6.71729	6.69825	5.0889	5.18463	4.61478	5.20479
CEP112	Q8N8E3	6.1471	6.31725	6.77597	5.67261	6.51551	6.74059	6.26367	6.42211	6.59291	6.5352	6.06963	6.44709	6.12371	5.66818	6.08773	5.82073
CENPN	Q96H22-3	6.73866	6.83323	6.43776	6.46158	5.36967	6.51526	6.07986	5.85386	6.23315	6.14353	6.53068	6.76834	6.23858	5.84347	5.85347	6.0989
IFT88	Q13099-1	6.59203	6.6618	6.63522	6.05797	6.4524	6.48375	6.13436	6.79763	6.56301	6.60556	6.71547	6.48023	5.48342	5.55621	5.30469	5.47626
SLC45A4	Q5BKX6-2	5.95089	6.55154	6.2427	6.83458	6.01096	5.77906	5.97113	5.80617	5.91167	6.55453	6.34137	6.84171	6.07451	6.22257	6.2796	6.62702
MIER3	Q7Z3K6-2	5.89664	6.11752	6.3187	6.13745	6.17835	6.23131	5.82156	6.01468	6.15539	6.10314	6.7538	6.38066	6.47485	6.70683	6.30296	6.40616
HMGXB4	Q9UGU5	5.25272	5.57504	4.67663	4.98053	5.28915	5.56595	5.12008	5.2585	4.83546	5.41012	4.6183	4.88558	8.83836	10.1971	9.36416	10.1323
PKMYT1	Q99640	6.96285	7.28043	6.44014	7.08194	6.35748	6.59223	6.42059	6.56644	6.35002	5.98977	5.99217	6.07263	5.51172	5.30432	5.61175	5.4655
TRIT1	Q9H3H1	5.97351	6.6287	5.533	5.87533	7.53622	6.74103	5.67904	6.55811	5.96847	5.8304	5.76642	5.87229	6.25541	6.5395	6.16398	7.07858
PODXL	O00592	6.38622	6.39186	6.18387	6.34724	6.39318	6.34594	6.19147	6.03976	6.26817	6.29815	6.22225	6.15882	6.3712	5.99946	6.12079	6.28159
KIAA1109	Q2LD37	7.00879	6.70244	6.65497	6.81459	5.49168	6.11389	6.2131	5.70681	6.01585	6.51171	6.45191	6.29115	6.13702	5.96827	5.65023	6.2676
CYP27C1	Q4G0S4	6.14303	6.3091	5.67286	5.91684	5.97557	5.88384	6.59967	5.85182	6.40317	6.24619	6.22524	6.07041	6.61663	6.77616	6.86414	6.44534
PATJ	Q8NI35	6.38574	6.70879	5.94228	6.00797	6.65118	6.35812	6.07174	6.36796	6.24422	6.30427	5.89356	6.23926	6.44508	6.31936	5.93266	6.1278
CHMP2B	Q9UQN3	6.77062	6.38825	6.33729	5.79807	6.33166	6.30413	6.46681	6.96477	6.55155	6.74807	6.52525	7.03362	5.1352	5.67712	5.30433	5.66325
PANK1	Q8TE04	6.37085	6.2454	6.75072	6.16101	6.01313	6.51533	5.96259	6.87126	6.56491	6.4048	6.69095	6.46182	5.55812	5.84554	6.09961	5.48396
STAT5B	P51692	6.17153	6.2881	6.64864	5.92011	6.29609	6.36383	6.38762	6.83251	6.33003	5.9192	6.18231	6.09932	6.31254	6.11562	6.19502	5.93752
FAM117B	Q6P1L5	6.26294	6.27583	6.86223	5.94884	6.20146	6.54374	6.21786	6.26503	6.44663	6.5973	6.56558	6.65838	5.85415	5.78073	5.58838	5.93092
KIAA0586	Q9BVV6-3	6.08029	6.79422	6.40259	6.37661	6.43586	6.78697	6.43772	7.34031	7.00517	6.69287	6.17255	7.07506	4.99893	5.15805	4.88183	5.36098
CTH	P32929	5.94352	5.65496	5.8366	5.05885	6.09353	5.97374	6.72791	7.09067	6.80466	6.80186	6.61433	6.57603	6.38165	6.18231	6.84209	5.41731
TFAP2A	P05549	6.30112	6.62925	6.32294	6.8137	6.35592	6.37632	6.19503	5.67029	6.64112	6.61151	6.42851	6.29956	5.62303	5.88194	5.60425	6.24551
KAT2B	Q92831	5.75474	6.42441	5.55016	6.44958	6.81209	6.47612	6.04636	6.55095	6.47485	6.20692	6.67059	6.26437	6.34097	6.41658	6.30653	5.25477
NTM	Q9P121-4	6.29812	6.27569	5.98149	6.05141	6.49616	6.29557	6.02758	6.24279	6.18283	6.00786	5.81987	5.88094	6.77237	6.48133	6.72243	6.46356
PUSL1	Q8N0Z8	6.61203	6.28157	6.39398	6.99392	6.06716	5.72553	6.22856	5.75415	6.37522	6.53062	6.30474	6.94176	6.45369	5.69251	6.12994	6.41462
U2AF1	Q01081	6.19773	6.41607	5.76794	6.8497	6.35148	5.79804	5.63482	5.59176	6.20556	5.80896	5.67934	5.73128	7.01561	6.72904	7.07256	7.1501
LRRCC1	Q9C099	6.43068	6.58108	6.50188	6.596	6.01989	6.45046	6.54867	6.52971	6.09391	6.66911	6.70478	7.51231	5.08582	5.53686	5.32797	5.41087
KLHL11	Q9NVR0	6.04944	6.27746	6.67936	6.77353	6.20611	6.44357	5.93582	6.33257	6.23103	6.66137	6.87039	6.74541	6.08713	5.5107	5.63796	5.55814
MRS2	Q9HD23	6.59234	6.30444	5.89771	6.41279	6.37346	6.05265	6.65333	5.33892	6.2121	6.26155	6.52398	5.6174	6.12399	6.11652	6.86134	6.6575
S100A16	Q96FQ6	6.09028	6.98978	5.34168	5.79355	6.04222	4.99648	5.32972	6.38913	5.06107	6.41703	5.73859	6.68993	6.02722	7.7681	7.70801	7.61722
IREB2	P48200	7.15267	7.1113	7.6061	6.95664	6.03095	7.11273	6.14092	7.17421	6.96422	7.41	7.74292	7.34497	3.38022	4.63477	3.97581	3.26157
N4BP1	O75113	6.01682	5.88987	6.6751	5.91272	5.7035	5.73129	6.07393	6.1585	6.49401	6.86142	6.82296	6.51136	5.9567	6.85651	6.37794	5.95737
PPTC7	Q8NI37	6.57001	6.872	6.29839	6.73161	6.29923	5.48563	6.3692	5.92793	6.32797	5.86942	5.61561	6.67267	6.62377	5.83809	6.1599	6.33856
DNA2	P51530	6.81449	6.84065	8.01261	6.80395	6.04952	7.04292	6.00247	6.59917	7.6074	7.80184	7.97877	7.40217	3.31919	3.92059	3.58608	4.21817
POLR3F	Q9H1D9	5.8185	5.93753	6.049	5.9001	5.80165	5.7698	5.53704	6.09445	6.20789	6.48516	6.46904	6.4004	6.30164	6.72096	6.94512	6.5617
DDR1	Q08345-5	6.40979	6.87903	6.69286	6.5543	6.18218	6.40696	6.2805	6.63191	6.25925	6.65403	6.20105	6.46278	5.32872	5.83406	5.50977	5.71282
NSL1	Q96IY1	6.15315	6.30265	6.46346	6.43305	6.33782	5.69236	6.33042	5.88584	6.27456	5.98752	6.40879	6.26301	7.10164	6.0925	6.05895	6.21428
ANKH	Q9HCJ1	6.30222	6.41368	6.13169	6.75486	6.45881	5.92113	6.04317	5.9328	6.43924	6.20781	5.9719	6.31911	6.03985	6.14206	6.76157	6.16009
STARD8	Q92502-2	6.9593	7.18503	6.27839	7.21141	6.91098	6.92814	6.69231	6.76145	7.06078	7.35198	6.99725	7.55113	3.58052	4.13597	3.90419	4.49117
GALNT14	Q96FL9-3	6.75024	6.40764	5.94122	6.98097	5.91225	5.63241	6.86417	6.15291	6.03723	5.91992	6.04844	6.38431	6.35751	5.88176	6.37266	6.35636
TMUB1	Q9BVT8	6.84546	6.45384	5.37573	6.37004	6.50404	5.5732	6.24417	6.21177	5.2933	4.98028	5.79588	5.48828	5.62651	8.32304	7.06409	7.85039
PHLDB3	Q6NSJ2	6.43585	6.0684	6.00557	5.97092	6.41537	6.35629	6.51622	6.33827	6.1018	6.62093	6.82472	6.45724	6.1472	5.61602	5.57225	6.55295
PCMTD1	Q96MG8	5.11561	5.95744	6.45821	5.67691	5.77569	6.23129	5.47	5.97594	6.08193	6.67656	6.65701	6.31372	6.88117	7.1252	6.86729	6.73603
GEMIN6	Q8WXD5	6.34654	6.28846	6.35365	5.48346	6.76645	5.99533	6.05796	6.84025	6.62835	6.47682	7.28761	6.99609	5.94261	6.23859	5.87143	5.42639
NDUFA11	Q86Y39	6.08904	6.42356	5.56098	6.03476	6.44426	6.47262	6.46697	6.42768	5.98442	6.02982	5.61629	5.70904	6.536	6.53642	6.79333	6.87482
LRRC41	Q15345	5.45858	5.90445	6.01751	5.685	5.76909	6.03857	5.92371	5.96264	6.41336	6.23806	6.74764	6.04893	7.02603	6.7773	7.09356	6.89001
CMC4	P56277	6.56481	5.91872	5.5477	5.78127	6.36284	5.40776	5.76894	5.75697	5.04743	5.87513	6.45288	5.97479	7.29287	7.20841	7.43843	7.60105
IRAK2	O43187	6.32463	6.20572	6.73032	6.14622	5.84028	6.46695	6.07008	6.35074	6.37886	5.71113	6.40882	6.60864	6.30633	6.34974	5.96707	6.13448
DMAC2	Q9NW81-4	6.71479	6.07091	5.95496	6.37655	6.79618	5.87454	6.33403	6.27177	6.52073	6.42365	6.02223	6.37745	6.73082	5.56185	6.29218	5.67735
PHLDA1	Q8WV24	5.76456	5.37431	5.96898	5.19238	6.25414	6.18808	6.33902	6.01976	5.95595	5.395	5.66545	5.79495	7.58103	6.82742	7.54378	8.13517
ZFX	P17010-3	6.86579	6.55134	6.53922	7.12987	6.12222	5.86537	6.44274	4.98961	6.29137	6.04422	6.5474	6.55341	6.24224	5.96434	5.26626	6.58459
MAZ	P56270-2	5.79609	5.90473	6.3772	6.75566	5.86545	5.95925	5.9836	5.66039	6.14162	6.45777	6.48249	6.83469	7.04231	6.32633	5.96758	6.44484
VEZF1	Q14119	6.63767	5.76285	6.11475	6.78892	6.36366	6.09058	6.0805	5.73263	6.1639	6.18721	6.10824	6.14583	6.86866	6.10463	6.39523	6.45475
MAP3K20	Q9NYL2	5.95893	6.28294	6.25172	5.90328	6.2718	6.85137	6.06289	6.79641	6.57468	6.97059	6.22258	6.83982	5.62449	5.7703	5.72855	5.88965
ZFAND3	Q9H8U3	6.25694	6.90926	7.77366	6.85064	6.64058	6.68809	6.42667	6.96488	5.91397	6.73788	6.42611	6.2352	4.94532	5.29605	4.93364	5.00112
RABGAP1L	Q5R372-3	6.42517	5.94384	7.12257	6.08759	6.31155	6.62224	5.98483	6.63143	6.55412	6.28958	6.09398	6.18475	6.26289	6.41529	5.40087	5.66928
STEEP1	Q9H5V9	5.59905	9.14431	5.89236	6.13843	5.56894	5.57634	5.85612	7.22774	6.18358	6.06076	6.29668	5.55183	5.87862	5.94565	7.5318	5.54779
LTF	P02788	5.29057	6.68759	5.42045	6.17004	5.39907	7.18298	6.47102	5.55273	6.504	5.57381	6.37341	5.83798	5.01067	8.89804	7.1828	6.44484
PRAF2	O60831	6.37552	6.30582	6.32587	6.15391	6.33898	5.92985	6.51779	6.45898	6.17675	5.67839	6.22753	5.66816	7.08262	6.29428	6.31075	6.15479
IFT27	Q9BW83	5.99357	6.31705	6.30944	5.04371	6.49462	6.932	5.64437	7.05197	6.96358	7.38619	7.12333	6.94761	5.5988	5.66852	5.48728	5.55076
SLC49A4	Q96SL1	6.27789	6.60339	6.02583	6.91465	6.10182	6.22706	6.19524	6.03328	6.26884	6.6104	6.5136	6.15708	5.92528	5.84583	5.95607	6.34374
TRIM58	Q8NG06	6.59359	5.61337	7.96852	6.72754	6.17828	6.1189	5.76898	6.97906	6.84868	6.01865	6.88825	6.22138	5.20615	5.88174	5.69499	5.29193
LYPLAL1	Q5VWZ2	6.63611	6.11742	6.79964	6.17537	6.24697	6.4486	5.85055	6.386	6.07273	6.48821	5.91851	6.37093	5.96461	6.40423	6.2088	5.91133
DCUN1D5	Q9BTE7	5.56309	5.69541	6.31633	5.76869	5.32416	6.26605	5.3017	6.23954	5.84135	6.12663	6.46286	6.23325	6.57148	7.8352	7.05506	7.39919
ERG28	Q9UKR5	5.64136	5.03527	5.74688	5.94734	6.65292	5.9333	6.10188	5.30706	5.67668	6.51899	5.43822	5.9544	6.61582	7.62032	7.52466	8.28489
ARL13B	Q3SXY8	6.34524	6.20458	6.82893	6.26052	6.55204	6.61807	6.41109	5.93908	6.38891	6.12147	5.90266	6.18486	5.6818	6.40802	6.07427	6.07848
CHCHD7	Q9BUK0-4	6.43888	6.97113	6.11744	7.03688	6.65571	5.71512	7.42637	5.51806	5.60987	6.18688	6.3503	6.41885	6.2807	6.11846	6.21142	4.94394
GZF1	Q9H116	6.18341	6.38842	6.51507	7.41371	5.89423	5.84261	6.4685	5.73721	6.06743	6.36874	6.37656	6.4057	5.93012	5.92502	6.00191	6.48136
MTMR8	Q96EF0	6.27498	6.4414	6.50552	6.54591	5.6796	6.33902	5.41147	6.42442	6.69085	6.6144	7.05651	6.70645	5.29335	5.70589	5.56826	5.74196
KCMF1	Q9P0J7	6.98313	5.83717	7.33749	6.25042	5.94183	6.41463	5.67457	6.17357	6.02569	6.44626	7.23371	7.40434	6.03672	5.80213	5.00589	5.43247
MICALL2	Q8IY33	6.39372	6.08358	6.12767	5.44926	5.96301	5.74792	5.9856	6.34679	6.5953	6.03128	6.22801	6.32595	7.21846	6.53409	6.42666	6.54267
BCKDK	O14874	6.32877	6.91661	5.7881	7.08858	6.5377	6.06145	5.73518	6.14812	5.85739	5.56772	6.16156	5.7333	6.40046	6.89823	6.09027	6.68655
OXSM	Q9NWU1	6.41361	8.73857	5.54753	6.69585	5.41017	5.40376	6.2575	6.79481	7.02338	6.09167	5.54821	5.98437	6.13005	5.80807	5.95784	5.29338
UBL5	Q9BZL1	6.61605	5.93626	6.93406	6.4845	6.48823	6.72248	6.87961	6.06244	6.73714	7.04431	7.20568	6.92098	5.65854	4.80798	4.42336	5.07836
GRN	P28799	7.26583	6.48041	6.47598	7.15941	6.27772	5.91235	5.80197	5.70858	6.34971	6.38013	6.8576	6.39215	5.56703	5.68555	5.62773	6.05786
ZNF219	Q9P2Y4	6.08996	6.41939	6.33346	7.02621	6.59071	6.84572	5.73215	5.93557	6.18958	7.02907	5.90581	6.1851	5.4782	5.61417	6.62356	6.00135
ANP32E	Q9BTT0	5.42036	5.2943	5.99262	5.18853	5.92505	6.37165	5.36148	5.82796	5.76993	6.22181	6.48008	6.22988	7.25093	7.5513	7.47366	7.64045
CMC1	Q7Z7K0	7.39661	6.8242	6.9343	6.30003	6.3466	4.96041	5.32801	5.30819	6.2275	6.05131	6.52056	6.2313	7.23123	6.16277	6.07766	6.09933
ARHGEF5	Q12774	6.42002	6.25988	6.77601	6.08456	7.42525	6.16732	5.50407	6.52137	6.44408	6.86538	6.52881	5.99593	6.34328	6.06433	5.3873	5.60239
NDUFAF5	Q5TEU4	6.22228	5.77784	5.9058	6.51403	5.68609	5.88586	6.70445	5.73954	6.43967	6.26958	6.62042	6.43334	6.68795	6.30568	6.34906	6.45842
CARD10	Q9BWT7	6.64874	6.72981	7.20273	6.96059	6.10818	6.54168	6.12568	7.00313	7.07745	6.8177	7.36159	7.23358	4.71876	4.47735	4.41037	4.58265
DBF4	Q9UBU7	6.53077	5.99627	6.23945	6.56144	5.95792	6.03785	5.687	5.61081	6.59116	5.92371	6.5243	6.44708	6.15159	6.32036	6.6338	6.7865
BCL10	O95999	6.60123	5.89552	6.95184	5.57042	6.55281	6.46962	5.6248	7.10896	6.19567	6.7846	6.91438	7.37689	5.39086	5.40861	5.19595	5.95782
IL13RA1	P78552	7.22798	6.78132	6.48375	6.99779	5.834	6.38353	6.68748	5.81561	7.16466	7.48129	7.76904	6.59571	5.07143	4.46482	4.86516	4.37642
RSAD1	Q9HA92	5.89343	6.47575	6.30767	7.08558	6.35689	6.38495	6.2533	6.31971	6.16161	5.93186	6.42778	5.9936	5.99752	5.9071	6.25719	6.24608
ARL14EP	Q8N8R7	6.5483	6.28018	6.61885	6.56763	6.14229	6.29061	6.70851	5.81659	6.31892	6.68884	6.05912	6.21626	6.36744	5.7198	6.0468	5.60987
SORT1	Q99523	6.41817	6.66206	6.99795	6.91864	5.92543	6.25199	6.61882	6.22701	6.3079	7.02633	6.69392	6.74355	5.28851	5.06942	5.38157	5.46872
SRRD	Q9UH36	6.06527	6.05465	7.40393	6.78002	6.10392	6.7256	6.00689	6.18025	6.0966	6.24373	6.71942	7.32429	5.72095	5.06039	5.36718	6.14691
DGKD	Q16760	5.86999	6.00642	6.31345	6.56842	6.66695	5.93801	5.93204	6.10273	6.65624	6.46389	6.42364	6.37073	5.99351	6.05514	6.25407	6.38476
TRABD	Q9H4I3	6.46469	6.82332	6.41601	7.23456	6.10833	5.78699	6.15758	5.94609	6.57547	6.15477	6.67916	6.26232	5.73779	5.56925	6.09322	5.99045
WDR77	Q9BQA1	5.72422	5.91757	6.31639	5.56246	5.96714	6.45772	5.6276	6.61723	6.45943	7.04579	6.55299	6.54764	6.40224	6.26697	5.84376	6.69084
SNRNP35	Q16560-2	6.03283	6.4218	6.05374	6.84808	6.15016	6.84821	6.62863	6.17372	6.20803	6.41002	6.23914	6.03093	5.9768	5.90809	6.05774	6.01207
SRSF8	Q9BRL6	6.14967	6.32368	5.5987	6.2557	5.99575	5.93081	6.12543	6.43258	6.34193	6.34364	6.23504	5.56301	6.94264	6.55384	6.61163	6.59595
STK19	P49842	6.34239	5.90136	6.07799	5.9819	5.66149	5.86942	6.283	5.95965	6.96641	5.91796	6.6278	7.35543	6.40135	6.33033	5.89332	6.43021
BLZF1	Q9H2G9	6.47552	7.04759	6.22874	6.87836	6.7773	7.14133	6.82318	6.59056	6.27446	6.25014	5.78566	7.00633	5.12621	5.02293	5.49388	5.0778
MED25	M0QZQ2	6.03276	6.26905	6.38083	6.21701	6.22174	5.7393	6.52685	6.33054	6.49879	5.95901	6.48999	5.98101	6.34503	6.41971	5.40962	6.17875
NARS2	Q96I59	6.37599	6.01475	5.79091	6.71348	6.3238	6.09691	6.29925	6.05886	6.33079	5.95313	6.00999	5.99289	6.3328	6.52037	6.65475	6.53134
SLC29A1	Q99808-2	6.02034	6.36199	5.91479	6.79312	5.53136	5.73155	6.05418	6.29385	6.66841	6.84927	5.83073	6.95829	5.99121	6.567	6.18607	6.24782
MARF1	Q9Y4F3	6.11125	6.4405	6.96123	6.09297	5.82592	6.9045	5.85588	6.55867	6.03144	6.61469	6.58673	6.6084	5.14936	6.81891	5.86629	5.57326
TRAF7	Q6Q0C0	6.41192	6.91532	6.72617	6.80709	6.54783	6.11175	6.4302	6.30572	6.57204	7.1548	6.85918	6.47054	5.31608	4.84278	5.1505	5.37808
HMGN4	O00479	5.64561	6.04396	5.609	7.09682	6.04704	5.75483	6.19492	5.34183	5.59948	5.57566	6.2114	6.44654	6.1022	7.38206	7.2979	7.65077
DTX3	Q8N9I9-2	6.30653	6.13634	6.5304	5.55446	6.17671	6.87208	6.24876	6.57069	6.46457	6.79158	6.56083	6.53586	5.88112	6.00961	5.71406	5.64642
FZR1	Q9UM11	6.73917	6.89105	7.18387	6.86529	6.01506	6.88958	6.60847	6.62652	7.12966	7.25161	7.64437	6.92913	4.03794	4.70132	4.14078	4.34619
RTF2	Q9BY42	6.27694	6.30582	6.97383	5.83415	6.19306	6.57048	6.43597	6.46795	6.6248	6.26847	6.64465	6.35269	5.59131	6.22108	5.81586	5.42293
GMEB2	Q9UKD1	6.84835	6.73477	6.20505	6.61378	6.78167	6.45943	6.62221	6.34767	6.51284	5.83144	5.96478	5.88801	6.51812	5.56559	5.61414	5.49217
SFXN4	Q6P4A7	6.74758	6.84906	5.61738	6.46744	6.11318	6.20842	5.93717	6.80293	5.70743	6.27393	5.41028	6.42776	6.14807	6.32231	6.46406	6.50299
NCS1	P62166	6.18164	5.01285	5.70159	4.69765	5.91658	5.21648	5.45271	6.64045	5.20314	6.62089	5.51603	6.74138	6.36509	8.30964	8.11112	8.31275

SNUPN	Q95149	6.77152	6.46159	6.70824	5.95423	6.91052	6.81148	6.70409	6.4664	5.83073	6.62369	7.00819	7.06148	5.1819	5.68111	4.75464	5.07017
MGRN1	O60291-2	6.50946	6.08836	6.68002	7.47992	6.09839	5.83147	5.72485	6.34054	6.6585	6.8356	5.31752	6.71804	5.86007	6.62393	5.47716	5.75617
SENP7	Q9BQF6	6.26665	6.42041	5.59385	6.46132	5.97318	6.11425	6.44679	5.94524	5.95975	6.38055	6.15635	6.23173	6.74728	6.58898	6.45039	6.26328
NEU1	Q99519	6.74854	6.81158	6.1253	6.71978	6.84162	6.62861	6.21247	5.75252	6.35222	6.47377	6.1339	5.55139	5.58585	5.87654	6.23297	5.95293
PRR3	P79522	6.33353	6.84658	5.7429	6.79777	7.34427	5.50473	6.64997	6.07007	6.31289	6.28543	5.82973	5.58934	6.02466	6.22859	6.34654	6.09302
MANEA	Q5SRI9	6.33465	6.98963	5.99666	6.2368	6.06493	6.3458	6.27827	5.88332	6.14475	6.14653	6.33065	6.49282	6.41574	6.07302	5.98688	6.27954
JAM3	Q9BX67	6.04824	6.60747	5.90774	6.2986	5.61892	5.9225	6.19816	6.16001	6.17123	6.17126	5.87202	6.14274	6.99802	6.597	6.30967	6.97641
SCO2	O43819	6.43873	6.64728	6.17165	6.81325	6.21318	5.93211	5.74729	5.93995	6.09874	6.31037	6.57108	6.75014	6.45936	5.93683	6.04616	5.92389
GALNT6	Q8NCL4	6.44318	6.02347	5.40748	7.01422	6.43981	5.93981	6.48135	5.87597	6.11588	5.81672	6.10204	5.63571	6.68753	6.34193	6.63658	7.03832
PRSS23	O95084	7.72006	6.40268	5.83759	7.23298	5.52582	5.6286	7.68971	6.14843	7.04309	6.77239	6.09547	6.41335	5.75384	4.41754	5.38204	5.93639
TMEM222	Q9H0R3	6.60707	6.51842	5.91456	6.25635	6.08824	6.33156	6.30068	6.44077	5.92926	5.46405	6.42464	6.18792	5.47835	6.74582	6.74868	6.56362
CLEC11A	Q9Y240	6.63249	6.12527	5.4787	5.64764	6.77948	5.95255	5.82848	7.5786	6.23162	7.64537	6.81846	6.89932	5.95593	6.11955	5.00761	5.2989
FAT4	Q6V0I7	6.34848	6.25034	5.76099	6.00131	6.53692	7.17641	6.87204	6.31505	6.02999	6.55594	6.01753	6.12106	6.5382	5.88148	5.36936	6.22489
KLHDC3	Q9BQ90	6.2254	6.60129	7.01959	6.23494	6.54889	5.93682	6.29937	6.47071	6.2947	6.50686	6.72519	6.81521	5.88373	5.48042	5.577	5.37988
D2HGDH	Q8N465	6.42709	6.56518	6.26796	6.86195	5.90501	5.98761	6.14026	6.1024	6.29281	6.20999	5.90634	6.12781	6.51681	6.28974	5.91699	6.48205
GPR108	Q9NPR9	6.26726	5.92769	5.98518	6.54681	6.05137	5.92468	6.3447	5.39556	6.11492	6.44062	6.58511	6.02317	6.79735	6.84971	6.4762	6.26967
ARRB1	P49407	5.65825	6.05739	7.00028	5.47779	5.58398	6.89772	6.20225	6.46161	6.09251	6.10696	6.22924	6.41805	6.25785	7.40622	6.27513	5.87478
FKBP14	Q9NWM8	6.52794	6.11692	6.04044	6.37078	6.80869	5.80546	6.38124	6.77228	6.17782	5.92913	6.19203	5.89214	6.85437	5.81322	6.02355	6.29399
HDAC4	P56524	6.20545	6.14262	6.40072	6.46583	6.55506	6.28495	6.84978	6.60914	6.81032	6.14257	6.58514	6.20127	5.83822	6.07393	5.62141	5.21359
PSEN1	P49768	6.65365	6.54149	5.77859	6.69912	6.11695	5.52742	5.9657	7.03564	5.70232	6.35169	6.51786	6.05828	5.78348	6.84434	6.24032	6.18316
PTPMT1	Q8WUK0	6.20524	5.56427	5.95476	6.72576	6.22003	5.76741	6.47654	5.62471	6.57126	6.28309	6.26855	6.50078	6.93355	6.15524	6.25459	6.49424
PPP3R1	P63098	5.71072	4.76116	6.15349	4.76513	6.35219	5.23556	5.94103	6.77409	5.90105	6.87359	6.20654	7.05407	6.11803	6.91449	7.84573	7.39312
KIFC3	Q9BVG8-6	6.60857	7.27494	6.79219	6.61316	6.71685	6.63455	6.31348	6.7683	6.65579	5.86495	6.576	7.73335	5.09347	5.23049	4.64519	4.47872
DYNC2LI1	Q8TCX1	6.71148	6.00392	7.63251	6.45906	5.91522	6.66172	5.98725	6.35541	6.88306	6.84787	6.74197	6.95478	5.51824	5.43075	4.947	4.94976
ELOVL5	Q9NYP7-2	6.00975	7.5337	6.03761	7.32849	6.17641	6.3045	6.02222	6.06991	5.78743	6.48988	6.2124	5.86071	5.31852	5.99337	6.37079	6.48428
TSSC4	Q9Y5U2	6.29395	6.2878	6.0416	5.77231	6.42882	6.44361	5.6193	6.74143	6.18093	5.98794	6.92386	7.13051	6.46503	5.41399	6.15686	6.11206
E2F7	Q96AV8	6.42268	6.44363	6.60742	6.28417	5.63174	5.60797	6.15162	6.31032	5.84785	5.74394	6.31932	6.48444	6.45893	6.22229	6.88658	6.5051
PITPNC1	Q9UKF7	6.27689	6.36109	6.7704	6.05647	6.20044	6.49393	6.29042	6.45864	6.61734	6.88093	6.50468	6.5747	5.81321	5.60061	5.56011	5.54015
MTFR2	Q6P444	7.30708	7.39729	6.79928	7.42504	6.57801	6.218	6.70568	6.40373	6.38506	6.63385	6.68073	6.94208	4.86008	4.46632	4.58368	4.61409
TMCC1	O94876	6.33386	6.60046	5.91743	6.10138	6.44875	5.9875	6.15282	6.35251	6.00309	6.03601	6.40437	6.03759	6.79564	6.23249	6.43004	6.16604
ZRSR2	Q15696	6.52451	6.52793	5.70928	6.49192	5.63653	5.70859	6.45817	5.70666	5.83081	5.79062	5.93061	6.05093	6.38198	6.49975	7.52743	7.22429
CPEB2	Q7Z5Q1-6	6.18994	6.292	6.56286	6.30305	6.45636	6.37795	6.28454	6.1634	6.28141	6.51147	7.10392	6.85309	5.79432	5.58135	5.83492	5.40941
DVL3	Q92997	6.25197	6.09974	7.62632	6.70079	6.31372	6.73667	5.70428	6.73243	6.75115	6.68601	6.954	6.67119	4.91576	5.36669	5.19372	5.29554
USP20	Q9Y2K6	6.71274	6.51954	6.07658	6.78477	6.1122	6.16228	6.75576	5.95215	5.93813	6.25918	6.1529	5.93825	6.07666	5.97986	6.1732	6.4058
CNTRL	Q7Z7A1	5.89324	5.9022	7.13987	6.43279	6.76729	6.93293	5.57644	6.6736	6.41266	6.0596	6.36439	6.80277	6.25002	5.50507	5.54526	5.74189
PZP	P20742	5.21593	6.72871	5.31973	5.00224	4.81789	6.6257	6.28891	5.19154	7.04723	5.5769	6.55239	6.31568	6.52136	6.41844	9.32248	7.05485
FANCE	Q9HB96	6.19271	6.23608	6.02839	6.10628	6.63231	6.20638	5.51624	7.16684	6.55236	6.39225	6.69083	6.37259	5.61045	6.33502	6.44757	5.51371
TRAPPC13	A5PLN9-5	6.11802	6.22055	6.38104	5.81041	6.29752	6.57122	6.39474	6.72866	6.45112	6.54159	6.39762	6.39089	6.04234	5.87076	5.81027	5.97326
FBXO4	Q9UKT5	6.55199	6.02194	6.94887	5.44288	6.26276	7.05995	5.78546	6.83603	5.85251	6.45	6.59136	6.51699	6.2727	6.14621	5.75849	5.50184
INO80B	Q9C086	6.16568	6.29739	6.66648	7.13505	6.28618	5.65034	6.24881	5.52572	6.57886	6.41109	6.518	6.02065	5.72761	6.24323	5.92303	6.60187
PITHD1	Q9GZP4	6.53806	5.81187	7.13633	6.03267	5.65057	6.1541	6.0786	5.94679	6.39309	7.08629	6.58795	6.94443	5.69909	5.97831	6.31385	5.64803
GRPEL2	Q8TAA5	5.54993	5.51141	5.49947	6.08084	6.26093	5.80175	6.38655	6.17374	5.19511	4.81845	5.55884	5.54748	8.64096	7.21038	8.28229	7.48287
AVL9	Q8NBF6	6.05043	6.37272	6.47292	6.13178	6.25335	6.5491	6.30314	6.44861	6.76741	6.98891	6.25998	6.29898	5.84726	5.7321	6.01532	5.50799
TRAPPC6A	O75865-2	6.28019	6.49284	6.40129	4.67399	5.84526	6.47563	6.06363	6.69702	5.69123	6.73573	6.1189	6.70664	5.52918	8.48082	6.45927	6.72836
MCC	P23508	5.98916	5.26082	6.8709	5.55341	6.25148	5.82835	6.15876	6.82616	6.43396	5.45664	6.50062	6.53531	6.24036	6.17259	6.36479	7.55667
ZSCAN25	Q6NSZ9	6.91142	6.71198	6.18498	6.29691	6.16192	5.81639	6.76549	5.81075	7.16141	6.04109	6.50672	7.11613	5.78379	5.19371	5.93622	5.60111
MKRN1	Q9UHC7	6.74199	6.4617	7.4056	6.21812	6.63029	6.25303	5.83748	6.28683	6.56329	6.61116	6.73446	6.35726	5.39745	5.93594	5.20474	5.36065
PIP4K2B	P78356	5.88116	6.41966	6.59038	6.08637	6.1943	6.0649	5.67097	6.23842	6.4999	6.13404	6.76819	6.23329	6.14211	6.53744	6.44657	6.09232
ANKRD44	Q8N8A2-2	6.12186	6.37696	6.62924	6.04934	6.10633	6.83109	6.11863	6.55644	6.54817	6.98349	6.30339	7.00152	5.78806	5.53477	5.67907	5.37162
VRK3	Q8IV63	5.95784	6.53524	5.78772	6.43122	6.19154	6.40582	6.14987	6.25953	6.4391	6.45693	6.37464	6.06587	6.54017	5.99525	6.5483	6.76099
ANGEL2	Q5VTE6	5.55023	6.34079	5.93841	6.29994	5.84663	6.03318	6.37288	6.01776	6.24094	6.20586	5.94866	5.77684	6.80993	6.63842	7.26368	6.71586
HM13	Q8TCT9	6.25065	6.74789	6.0981	6.62284	6.00882	6.14796	6.24595	6.23517	5.75389	6.18641	5.7251	5.91491	6.68518	6.42468	6.50102	6.45143
CENPL	Q8N0S6	6.57512	6.39502	6.19417	7.05773	6.07323	5.92125	6.50884	5.91372	6.24508	6.23656	5.89959	6.22831	6.43727	5.92303	6.15507	6.236
TMEM97	Q5BJF2	5.88138	6.2881	5.83259	7.02324	6.72307	6.54902	6.3034	6.4086	6.39802	6.02051	6.1356	5.79624	6.51351	6.30219	6.05398	5.77055
LYRM4	Q9HD34	6.67288	6.41556	5.87937	6.6295	6.01368	5.8572	6.45886	5.99441	6.14082	6.01686	6.0407	5.81143	6.49061	6.48351	6.64439	6.45022
GATAD1	Q8WUU5	6.4213	6.46831	7.27299	7.02151	6.12594	6.0883	6.41529	5.88137	6.73532	6.55527	6.9019	6.497	5.01657	5.17133	5.69097	5.73663
FAM9C	Q8IZT9	5.61965	5.67857	5.87728	4.74809	5.75524	5.98693	5.82423	6.19051	6.03136	6.09903	6.12933	5.85063	7.60847	8.04773	7.47508	7.07787
FAM83H	Q6ZRV2	6.11956	6.56712	6.2199	6.13669	6.17722	6.53791	6.53812	6.22603	6.2968	6.94952	5.91142	6.06535	6.24328	6.47532	6.50049	6.03528
CMIP	Q8IY22	6.28024	6.66368	7.27653	6.53893	6.19466	6.67318	6.1411	7.48266	6.71508	6.71989	7.06471	7.70846	4.61899	4.5663	4.60285	4.75278
RBM15B	Q8NDT2	6.37599	6.3083	5.61384	6.49509	6.2095	5.72823	6.3407	5.8962	6.46813	6.13078	5.84654	6.05024	6.60786	6.18126	6.91832	6.82902
MINDY2	Q8NBR6	6.07986	6.26026	6.91981	5.83737	5.97852	5.92597	6.62289	6.68693	5.73369	5.70719	6.70626	6.36319	6.44257	6.09086	6.56216	6.08246
CRLF3	Q8IUI8	6.37354	6.45633	6.85642	6.04242	5.78209	6.41815	6.77268	6.13673	5.96594	6.01296	6.57359	6.34981	6.41845	5.86139	5.88862	6.09088
GLMP	Q8WWB7	6.82049	6.78421	5.07041	6.82517	7.88243	6.26304	5.88378	5.53713	5.39157	6.38172	7.49233	6.54279	6.43645	5.95744	4.97992	5.75112
JMJD6	Q6NYC1-3	5.38787	5.94169	6.18706	6.16003	5.54688	5.27568	5.90205	5.91492	5.59402	5.87567	5.85504	5.86122	6.65491	7.2267	8.7778	7.83845
TMEM177	Q53S58	6.39189	6.05558	5.93381	6.14636	6.27606	6.32626	6.18396	5.59627	6.34531	6.11908	6.29344	5.53076	6.70331	6.55619	6.38946	7.15225
SLC27A6	Q9Y2P4	6.54517	6.33127	5.96252	6.08921	6.35547	5.92146	6.26388	5.76586	6.37481	6.01371	5.71226	5.63738	6.46579	6.87288	6.94545	6.74288
MICOS13	Q5XKP0	6.59707	5.85673	5.67586	6.44213	6.34033	5.8202	6.27901	5.85787	6.20065	6.02355	6.19103	6.14481	7.0644	6.62458	6.2763	6.60546
PEF1	Q9UBV8	5.10072	4.32786	5.6346	4.24344	5.35796	5.63894	4.86986	5.85941	5.1566	6.06883	6.03638	6.69009	6.12237	10.0047	9.54776	9.34047
PTPRJ	Q12913-3	6.54798	6.39188	6.10335	6.79556	6.01491	5.73234	6.37466	6.22699	6.00569	5.80196	6.31193	6.22874	6.84494	6.1213	6.02668	6.4711
APOBEC3C	Q9NRW3	6.34749	6.09801	6.63785	6.39268	6.4954	6.25912	6.6315	5.98963	6.62171	6.76948	6.32151	6.51898	5.82129	5.83795	5.33306	5.92436
DSC2	Q02487	6.81954	6.9935	5.58136	6.56597	5.87251	5.60834	6.6973	6.4598	5.95857	6.46045	6.42119	6.27751	5.89061	6.24167	5.48459	6.6671
TMEM104	Q8NE00	6.13873	6.73878	6.61056	6.24275	5.94306	5.96547	6.30496	5.82664	6.19631	6.13947	6.78522	6.0201	6.37807	6.17916	6.16852	6.36221
KATNBL1	Q9H079	6.17856	6.43096	6.13296	6.07226	6.18689	7.1105	6.68031	6.06255	6.58775	6.12798	6.53057	6.92765	5.58423	5.69198	6.62984	5.06501
KIAA1217	Q5T5P2	6.31166	6.15918	6.18037	6.21691	6.04117	6.50474	5.98332	6.44846	6.28201	5.5623	5.98804	5.78996	5.6659	6.89134	5.9903	6.08435
CCNL2	Q96S94	5.72049	5.99627	5.85925	6.39997	5.84222	5.88187	6.26188	6.197	6.13057	5.9184	5.18487	5.60653	7.21042	6.99788	7.30329	7.48909
GMPR2	Q9P2T1-2	6.07865	6.51565	6.88645	6.05861	6.40963	6.58872	6.26982	6.33294	6.42908	6.55706	6.46494	6.34704	5.7278	5.49928	6.01003	5.82432
AGPAT5	Q9NUQ2	6.6908	6.31351	6.8237	7.38154	6.32752	5.12503	6.53049	6.24193	6.06803	6.21424	5.57446	5.84132	5.84039	6.26377	6.38966	6.37363
MTFR1L	Q9H019	6.73227	6.40471	6.42771	6.16411	6.6891	6.44032	6.51653	6.96975	6.4724	6.70703	7.00746	6.31339	5.28166	5.26688	5.09888	5.5078
TMEM168	Q9H0V1	6.33832	6.40366	6.48279	6.61571	5.86042	5.67919	6.49673	5.92712	6.83647	6.23011	6.45529	5.61329	6.22246	6.51583	6.59812	5.72448
GLRX5	Q86SX6	6.15172	6.89834	4.65099	4.73854	5.5996	4.68766	5.54046	7.01091	4.93902	5.55379	4.80131	5.95165	5.38897	10.7044	8.88392	10.4987
FCSK	Q8N0W3-2	6.05479	5.91443	6.24839	5.90216	6.05821	6.80552	6.25289	6.3514	6.17974	6.68507	6.58989	6.55612	6.00931	6.0612	6.10417	6.2267
TMEM161B	Q8NDZ6	6.07528	6.43116	6.32863	6.78141	6.22245	5.74643	5.99638	5.69589	6.40828	6.05532	6.09773	5.45843	6.8008	6.52728	6.50842	6.8661
TTC5	Q8N0Z6	6.38624	5.72139	7.16168	6.01404	6.16913	7.03922	6.17339	6.34909	6.71953	6.32283	6.77111	6.61115	5.47482	5.53833	5.8876	5.66045
FOXJ3	Q9UPW0	5.71888	5.84369	6.15608	5.73584	6.17155	6.28481	5.7103	6.32214	6.53499	6.5022	6.39731	6.4656	7.01946	6.44236	6.40299	6.2918
RUSC1	Q9BVN2	6.95713	5.4045	6.21661	5.7148	6.36448	7.29101	6.51085	6.61611	5.62252	6.68489	6.65157	5.97583	6.2078	6.04896	6.07086	5.6621
RWDD1	Q9H446	6.39407	6.68652	6.49552	6.06989	7.18266	6.91557	6.92151	7.6223	6.13186	6.48881	6.44924	6.6387	5.82611	5.56634	4.62578	4.98511
BCL2L1	Q07817	7.08982	6.43839	6.25265	6.16729	6.50564	6.03835	6.14237	6.39838	6.48059	6.68899	6.30073	6.51832	5.3873	5.97492	5.75018	5.86609
ATP5IF1	Q9UII2	6.93376	6.47555	5.4807	5.91118	6.76653	5.55454	6.03095	6.79995	6.14136	5.93216	5.84799	6.32423	7.38091	6.17265	6.12434	6.14753
C19orf47	Q8N9M1	6.42575	6.52574	5.70625	6.66637	6.56254	5.77493	6.38577	5.99861	6.1911	5.81139	5.57039	5.75441	6.62124	6.58673	6.68618	6.7326
XRCC4	Q13426	5.47233	6.40966	5.7897	5.42603	5.80369	6.5552	5.83134	6.43311	5.73181	6.54064	6.21773	5.9229	6.94434	7.14753	7.02286	6.75113
GBA2	Q9HCG7	6.52735	6.51376	5.95222	6.26795	5.8832	5.93876	6.42017	6.39128	6.18966	6.38817	5.53626	6.34579	6.27252	6.24544	6.33261	6.79484
C20orf27	Q9GZN8-2	6.21935	6.56458	6.82337	6.14439	6.433	6.95423	6.17849	7.06411	6.13522	6.3863	6.77731	6.30869	5.48513	5.76384	5.41364	5.34834
IGHMBP2	P38935	5.99535	6.2956	6.80209	6.66448	6.19052	6.41169	6.55707	6.0872	6.34493	6.58005	6.3968	6.55646	5.60088	6.06716	5.56004	5.88967
CPLANE1	Q9H799	7.60937	7.05442	8.1101	7.31724	5.90212	6.2601	7.03348	6.97596	7.17635	6.97043	7.63092	7.78128	3.14582	3.88231	3.47859	3.67152
ZFP82	Q8N141	6.32633	6.44594	5.94147	7.0361	6.36558	5.99859	6.68911	5.64905	5.7241	5.67227	6.39091	6.56114	6.75649	5.99873	6.06667	6.37752
NFYB	P25208	6.12801	5.62046	6.16391	5.46346	5.8171	5.3795	5.13031	5.95081	5.33914	5.4125	6.56934	7.48167	7.26111	7.63913	6.75732	7.88623
TTL	Q8NG68	6.52479	6.73847	7.25809	6.13581	5.99969	7.05383	6.58033	6.90212	6.93646	6.80423	6.08775	6.30071	5.14807	5.29347	5.04245	5.19373
TIMM17A	Q99595	6.48184	5.56707	6.53163	6.58811	5.99245	6.10053	6.66932	6.20071	6.502	6.37065	6.28769	6.10071	6.29054	5.72379	6.44003	6.15291
UBASH3B	Q8TF42	5.76611	5.96296	7.37514	6.37879	5.80091	7.16961	5.44186	6.47018	6.35133	6.63315	6.93537	7.0862	5.63417	6.01152	5.35065	5.63206
ACTR6	Q9GZN1	6.39824	6.48086	5.69845	6.54151	6.51409	6.0689	6.57641	5.85427	6.4874	6.41393	5.92155	6.3739	6.18719	5.66511	6.37971	6.43848
CNPY4	Q8N129	6.43091	6.23603	6.3346	6.31043	6.82142	6.43774	6.29407	5.70563	6.22561	6.14727	6.46978	6.11438	6.7701	6.15843	5.85572	5.68789
FBXO3	Q9UK99	6.23128	6.47555	6.33311	5.56787	6.47815	6.69187	5.96825	6.76986	6.08362	6.22984	5.94851	7.19703	5.44042	5.99361	6.3552	6.23581
CCDC77	Q9BR77	6.09558	6.7665	6.62476	6.85105	5.99429	6.66739	6.5327	6.5342	6.27466	6.45223	5.95262	6.40082	5.77733	5.36972	5.73277	5.97338
ARL6	Q9H0F7-2	6.23059	5.32885	6.91759	6.46342	6.49191	6.02852	6.24877	6.12928	6.01879	6.34348	5.90551	6.76886	6.15055	6.5322	5.71826	6.72344
CNNM1	Q9NRU3	6.29792	6.42727	6.7127	6.72511	5.6703	6.29418	6.39327	6.48833	6.64374	5.63211	6.21971	6.58804	6.20715	5.28483	5.65198	6.76336

TENT2	Q6PIY7	6.8219	6.33575	5.83619	6.95045	6.61185	6.57188	6.26858	5.67242	6.61121	6.00574	5.7293	6.29209	6.21168	5.91904	6.24181	5.9201
PTP4A1	Q93096	5.99919	6.11957	6.30014	6.21717	5.92928	6.61525	6.37229	5.9884	6.54324	6.75233	6.47325	6.65888	5.71893	5.93949	6.33567	6.03694
C6orf47	O95873	5.97053	6.19969	5.79438	5.3574	6.492	5.68464	5.66836	6.81411	6.22993	5.97053	6.20227	6.15953	6.90405	6.61541	6.60155	7.3356
PTPN21	Q16825	6.9736	6.25136	7.35513	6.31751	6.05897	6.80723	6.61314	6.3766	6.65696	6.11845	6.41455	6.9501	5.30359	5.61401	5.1413	5.04749
SIL1	Q9H173	6.25086	6.23979	5.94221	6.25076	7.18572	6.14896	6.62378	6.09213	6.65528	5.59501	5.82621	6.13334	7.09153	5.90638	6.06166	5.99639
NEMP1	O14524	6.67622	6.41138	6.33729	6.50982	6.34149	5.66172	6.53027	5.70458	6.36849	5.70061	6.25465	5.47402	6.90773	6.27057	6.6757	6.17546
GPRASP2	Q96D09	6.07084	6.87958	7.01822	5.50676	6.4198	6.19406	6.21309	6.54952	6.77203	6.07609	6.60994	6.62566	6.17087	5.63022	5.88214	5.38118
TRIM11	Q96F44	6.05907	6.47303	5.68226	6.03007	6.2883	6.18001	6.03441	5.828	5.81376	6.71948	5.69982	6.33758	6.50762	6.80943	6.18216	7.35502
SDC1	P18827	6.60151	6.70457	5.71246	6.67022	6.55823	5.71497	6.18952	6.18023	6.38766	5.76001	6.3904	6.15444	6.13468	6.31002	5.85422	6.67685
EIF1AD	Q8N9N8	6.2352	6.28768	6.99102	5.98256	6.34421	7.00877	5.77266	7.52541	6.21419	6.52226	6.54086	6.52016	5.95335	5.84941	4.91358	5.33868
QDPR	P09417	6.21473	5.93645	6.89874	6.00396	6.39406	6.69843	6.01116	5.64759	6.82386	6.03848	6.57117	6.054	6.34647	6.09384	6.62583	5.64123
SAMD1	Q6SPF0	6.24721	6.48072	6.08343	7.21235	6.21026	6.78552	5.78477	5.69857	5.95964	6.27675	6.4656	6.58804	5.84549	6.3112	5.95266	6.09779
ENSA	O43768-4	6.16975	5.50004	7.21322	5.23089	6.75585	6.1651	5.50577	7.12012	6.19201	6.66893	6.6439	7.22727	5.78892	6.50183	5.55891	5.75749
NEDD8-MDP1	E9PL57	6.32297	5.43367	6.72245	5.10838	5.44501	5.53318	6.11427	6.13965	5.97064	6.72919	6.28266	6.57609	4.87495	7.99084	7.14235	7.6137
FOXRED1	Q96CU9	6.61084	6.18798	6.16524	6.16683	6.10714	5.47506	5.93537	5.39199	6.5525	5.85486	6.5954	6.78283	6.67573	6.56095	6.59133	6.34596
EEF1AKMT2	A0A494BZY7	6.06784	6.24634	7.07649	6.3387	6.38057	6.75418	6.55055	7.33108	5.85296	6.3491	6.36887	6.67747	5.67209	5.91662	4.89993	5.5172
LSM12	Q3MHD2-2	6.49476	6.38833	6.55444	5.76829	6.38006	6.6198	6.01589	6.39668	6.35566	6.62566	6.49519	6.80068	6.15645	5.71186	5.64226	5.59398
NIPSNAP3B	Q9BS92	6.28653	6.58459	5.84089	6.42708	5.83764	5.62965	7.84626	6.16131	5.89275	5.88798	5.65582	5.98141	6.18297	5.98836	7.22345	6.57333
ZBTB2	Q8N680	6.30182	6.54499	6.83503	6.93658	6.44149	5.29171	6.29111	6.8896	5.84901	5.60435	5.88924	5.67231	6.87011	5.57355	6.72954	6.27956
MRPL10	Q7Z7H8-2	6.38408	5.79211	5.81777	6.70308	5.85003	5.91462	6.52211	5.29165	6.44495	6.05748	6.76888	5.73129	7.19374	6.45812	6.81039	6.25971
MED30	Q96HR3	6.31132	5.19264	6.06689	5.35101	6.73186	5.21912	5.11099	7.04897	5.14161	6.07722	6.15176	6.62841	6.64233	7.50255	7.4091	7.41424
REEP3	Q6NUK4	6.32671	5.98049	5.91781	6.34449	6.29379	5.81661	6.43278	5.79789	6.1517	6.00849	6.08597	5.75846	6.3197	6.83485	6.85613	7.07413
ATL1	Q8WXF7	6.03245	7.11872	6.24618	6.42176	6.41461	6.31233	5.67861	5.49337	5.96257	6.4387	6.32456	5.97101	6.35843	6.31716	6.50539	6.40414
ANAPC10	Q9UM13	6.03365	6.45839	6.39983	6.30771	5.74421	6.48944	6.26536	6.15649	6.26758	6.16039	6.43565	6.35856	6.2521	6.27345	6.09609	6.30109
BRK1	Q8WUW1-2	5.68949	5.35277	5.68472	4.80922	5.54514	5.64721	5.41278	6.46972	5.30018	6.1834	6.084	6.37408	4.71122	10.2933	6.91517	9.52755
ATXN7L1	Q9ULK2	6.19846	5.36198	6.02934	6.75869	6.32796	5.98541	6.59906	5.79283	6.35578	6.4774	5.7379	6.45549	6.31695	6.95489	5.98548	6.66238
PLEKHA3	Q9HB20	6.84564	6.67016	6.50636	6.6292	5.96426	6.25108	6.59618	6.80137	7.43113	6.92773	7.44102	7.09597	4.81326	4.93943	4.48139	4.60582
RYBP	Q8N488	5.53119	5.9042	5.99024	6.20816	6.39058	5.47715	5.63932	5.25756	6.11123	6.10954	6.25338	6.42715	7.58725	6.85379	6.96536	7.29389
GNPTAB	Q3T906	6.90563	7.126	6.42568	6.85524	6.15615	6.6832	6.444	5.97013	6.69625	6.21594	6.45663	6.56304	5.2396	5.27303	5.69266	5.29683
LAMTOR3	Q9UHA4	5.50266	5.84841	5.67204	5.60058	6.18454	5.98854	6.61939	6.65896	6.55657	6.84632	6.31439	6.51968	5.75537	6.26633	6.90641	6.46792
MOB1B	Q7L9L4-2	6.37258	5.87937	6.95789	6.06518	6.3147	6.628	6.48957	6.46721	6.2196	6.43119	6.80567	6.90902	5.46096	5.75897	5.94364	5.29645
RNF168	Q8IYW5	6.27204	6.63188	6.63894	6.99113	6.01312	6.25929	6.42741	5.99225	6.39271	6.13308	6.68777	6.43591	5.68853	5.73963	5.78879	5.90754
NENF	Q9UMX5	6.29434	5.01035	4.97913	5.00692	5.34825	3.96239	5.18062	6.39837	4.27081	5.73782	4.81125	5.7731	6.09093	10.9541	9.46757	10.714
FTH1	P02794	6.4747	6.34884	7.7414	6.62051	5.11321	5.3764	5.09649	6.96909	7.03105	6.68104	6.88682	6.45477	4.43127	6.79159	5.61062	6.37221
UBE2W	Q96B02-3	6.44957	6.40712	6.90876	6.00106	6.62575	6.38858	6.97534	6.51316	6.46989	7.04645	7.29729	6.54662	5.16306	5.22484	5.10382	4.87868
DEPDC7	Q96QD5	6.33438	7.00932	7.23724	6.05281	6.48149	6.3025	6.6612	6.26483	6.73839	6.40989	7.18163	7.26666	5.01626	4.9145	5.02153	5.10737
PPP1R14B	Q96C90	6.5344	5.52698	6.65977	5.09835	6.49067	6.43181	5.41643	6.67202	5.95712	6.79166	6.62178	6.96453	5.49816	6.64279	5.77277	6.92076
PFDN5	Q99471	6.13032	5.42622	5.56159	4.54325	5.60263	4.60817	4.67059	7.10299	4.46061	5.92186	4.90365	5.16104	4.14333	12.3684	7.91154	11.4838
DXO	O77932	5.67012	5.62859	7.26897	5.48847	5.70267	5.71767	5.72939	6.19431	6.37126	6.19976	6.6607	6.14438	6.65397	7.09078	6.6477	6.83126
MED19	A0JLT2	6.0424	5.90045	5.75106	6.77297	5.82855	5.99926	6.52745	5.9233	6.31284	5.76491	5.95049	6.23238	5.75838	7.4046	6.60276	7.22821
CEP83	Q9Y592	6.45046	6.30555	7.02013	6.20918	5.88881	6.59405	6.31393	6.35601	6.64412	6.44533	6.61928	6.45122	5.86666	5.30329	5.83288	5.69908
RWDD4	Q6NW29	6.54803	5.7957	7.04749	6.18834	6.67789	6.65998	5.825	6.53556	6.38811	6.64616	6.74975	7.09275	6.36875	5.33485	4.7604	5.38124
DEPDC1	Q5TB30	6.80303	6.7325	6.73137	6.61829	6.59444	6.48866	6.66649	6.13383	6.88529	6.79699	6.53807	6.72149	4.82345	4.97147	4.91976	5.57486
CHST12	Q9NRB3	6.67051	6.75927	6.29587	7.01642	6.10371	6.17254	6.48545	6.26244	6.4746	6.6789	6.52918	6.82305	5.33615	5.55393	5.22751	5.61049
VPS9D1	Q9Y2B5-2	6.3055	6.7614	6.39172	6.51649	5.86913	6.20932	6.34553	6.38345	6.28502	6.56989	6.73304	6.66331	6.01757	5.78779	4.97732	6.18352
OGFRL1	Q5TC84	6.44352	6.73344	6.79172	6.97587	5.90271	6.42679	5.90512	6.35291	6.63466	6.48528	6.54047	6.54573	5.6679	5.87654	5.21912	5.49821
DR1	Q01658	6.15488	5.36299	6.22736	5.47288	6.02518	6.9478	5.31973	5.92399	5.86434	6.42809	6.75973	6.27486	6.71654	6.68183	6.76908	7.07173
MRNIP	Q6NTE8	5.4794	5.69919	6.0837	6.0115	5.53424	6.53267	6.22808	5.80549	5.66675	6.59272	6.97068	6.4189	6.61041	7.42489	6.12449	6.81691
DMWD	Q09019	6.91646	6.26512	7.4333	6.30222	6.07173	7.31879	6.54049	6.23711	6.80004	6.83243	7.57973	7.37302	4.55673	4.7317	4.39625	4.6449
TMA7	Q9Y2S6	6.00918	5.97935	6.66465	5.68006	6.01289	6.29466	6.17491	6.38249	6.3855	6.57722	6.61417	6.57453	5.52453	6.97821	5.93749	6.21017
BICC1	Q9H694	6.02117	6.78261	6.49927	6.14004	6.41096	6.48477	5.96733	7.18092	6.69502	6.28789	6.17715	6.12156	6.24478	5.67245	5.85976	5.45431
TUSC3	Q13454-2	6.62151	6.14876	6.20689	7.05394	5.87702	5.89587	6.03773	6.11781	6.11167	5.73959	6.14579	6.31435	6.0241	6.63865	6.32618	6.74013
SYNJ2BP-COX16	A0A087WYV9	6.77207	6.1896	6.2317	6.6039	6.69616	5.96661	6.35997	5.84374	6.01573	5.72264	6.46187	5.98264	6.80015	5.67792	6.57747	6.09782
CTSL	P07711	6.49435	6.40957	6.02221	6.40604	6.05471	5.0823	5.57353	6.28697	6.0779	6.67083	5.87107	5.45793	6.07843	7.56123	6.82623	7.12669
SUDS3	Q9H7L9	6.20087	5.62264	6.30033	6.8177	6.53693	6.11269	6.23511	5.96506	6.02298	6.04089	6.01929	6.51938	6.61559	6.03104	5.97174	6.68784
PFDN2	Q9UHV9	5.92735	5.10709	6.47848	4.9365	6.3724	6.27382	5.76776	6.90092	5.84534	6.89887	6.90676	7.23793	6.28025	6.40964	6.31201	6.34488
TRAPPC2L	Q9UL33	5.41293	5.16022	6.25921	5.22293	6.70809	6.07397	6.62344	6.74387	7.71498	7.10057	6.65742	7.26415	5.22448	5.32	6.16183	6.3519
CHCHD6	Q9BRQ6	6.75499	6.40423	6.4261	6.36117	6.2733	6.00574	6.06688	6.05393	6.70429	6.40996	6.66782	6.52191	6.67809	5.28361	5.77431	5.61367
REST	Q13127	6.2058	6.04486	5.98203	6.97238	6.26145	5.85913	6.20877	6.10594	6.11894	6.12192	5.97226	5.84367	6.16513	7.10246	6.30398	6.73126
CSGALNACT2	Q8N6G5	6.48434	6.63266	5.91603	7.07109	5.96909	6.01391	6.26469	5.65636	5.84741	6.18782	6.12655	6.36621	6.57017	6.0273	6.12003	6.74633
SNAPIN	O95295	6.50619	4.89973	6.13967	5.55484	6.29205	5.55396	5.42971	6.14236	5.23074	6.42501	5.68451	6.7573	5.95929	8.24025	7.47607	7.70834
POLR1F	Q3B726	6.40065	6.50362	6.26242	7.19889	5.44615	6.13774	6.41464	5.32522	6.57577	5.83224	5.7554	6.06764	6.52975	6.66194	6.81664	6.07128
HDAC6	Q9UBN7	6.66944	6.26599	6.9076	5.82015	6.56116	6.51212	6.85886	6.46707	6.74593	5.92773	6.12403	6.10539	6.10569	6.22772	5.583	5.11812
PDSS2	Q86YH6	6.19611	6.2647	5.98266	6.49381	6.44497	5.79898	6.32171	5.923	6.31789	6.60828	6.09071	6.39589	6.67151	6.08474	6.3316	6.07344
SCARB1	Q8WTV0	6.33338	6.55677	5.91687	6.26382	6.75705	6.20096	6.40984	6.33533	6.79662	5.84432	6.4042	6.06336	6.27054	5.74772	5.98845	6.11077
OVCA2	Q8WZ82	6.23809	6.57349	6.55357	5.68155	6.49906	5.95762	6.17384	6.26572	6.40802	7.23524	6.47929	7.07562	5.94137	5.74011	6.02735	6.05005
SH3BGRL2	Q9UJC5	5.86765	5.74486	6.29268	5.5047	5.9167	6.50495	5.78706	6.50264	6.21437	6.39208	6.36447	6.59074	6.52362	7.04028	6.47381	6.27939
GPALPP1	Q8IXQ4	5.52503	5.92235	5.81538	5.80717	5.62554	6.33026	5.51627	6.43424	6.83076	5.90053	6.16625	6.11068	7.10043	7.0723	6.92865	6.91417
ING1	Q9UK53	6.54059	6.47077	5.65711	6.90688	5.66852	5.65156	6.24439	5.89434	6.11463	5.68394	5.77214	5.57486	6.53541	6.72478	7.11211	7.44798
SMCR8	Q8TEV9	6.48653	6.90348	7.09172	6.19667	6.4416	6.91612	5.94338	5.74188	7.16435	6.71991	6.35185	7.5976	5.19556	5.61984	4.96426	4.66528
C1GALT1	Q9NS00	6.34337	6.26627	5.63253	6.59654	6.37921	5.9422	6.35474	6.1218	6.40403	6.01677	6.29559	6.183	6.77422	6.22951	6.31556	6.14466
SKIL	P12757	6.77111	7.49763	5.94429	7.51752	6.12204	6.27658	6.67719	6.37311	6.35877	6.87885	6.63423	7.27492	4.45577	5.12082	5.10106	4.99611
TMCO1	Q9UM00	6.11124	6.26705	5.66059	6.73152	6.07677	6.11456	6.85688	5.92101	5.91087	5.99933	6.00496	5.97312	6.04037	6.99258	6.80219	6.53695
LZIC	Q8WZA0-2	6.18293	5.23378	7.05134	5.07431	6.25536	5.95263	5.77576	6.96509	5.89983	7.01414	6.95297	7.77609	5.92708	6.41625	5.72261	5.79983
GMFB	P60983	5.52861	5.46564	6.15195	5.14824	6.40245	6.20665	5.81565	7.61045	6.49083	6.7879	6.37951	7.4052	5.94592	6.27234	5.96419	6.42449
ZC3H6	P61129	6.20175	6.59231	6.03774	6.32317	5.47295	5.76062	6.29366	5.97251	5.7088	5.84358	6.06988	5.60134	6.5021	7.52311	7.29945	6.79702
GNPNAT1	Q96EK6	6.22537	6.40117	6.88604	5.91114	6.38899	6.71237	6.39401	6.71269	6.10957	6.25913	6.33201	6.86626	5.79876	6.56674	5.53187	5.80388
STK11IP	Q8N1F8	6.86342	6.78809	6.43448	6.44298	6.59455	5.63945	6.16649	5.93846	6.9622	5.71159	6.41116	5.93954	6.01816	5.73455	6.2759	6.07897
REX1BD	Q96EN9	6.47487	5.0918	6.85249	5.1433	6.3399	6.07455	5.91699	6.96773	5.33612	5.75136	6.14458	7.29886	4.44187	7.79709	7.06854	7.29993
CCDC25	Q86WR0	5.89128	6.15732	6.5527	5.91668	6.34021	6.46935	6.30959	6.92534	5.98408	7.13251	6.51457	6.96156	5.4951	5.85865	5.48725	6.00382
MFGE8	Q08431	7.10901	6.52047	6.24836	7.01147	5.37062	5.85994	7.06215	5.88744	6.51422	6.84521	6.48123	6.87797	5.75485	5.39446	5.49696	5.56563
PHYH	O14832	5.56592	5.76681	5.73036	5.78125	6.53926	6.30323	6.48372	6.15073	5.90506	6.06051	6.04452	5.65087	7.53501	6.35032	6.9886	7.14383
CCNE2	O96020	6.34522	6.49049	6.5495	6.70122	6.228	6.02391	6.18181	5.75236	6.36143	6.4932	6.61182	6.06765	6.19228	5.75702	5.98761	6.25649
NRP1	O14786	6.01375	6.57824	5.91437	6.47319	6.15561	6.25294	6.00517	6.14897	5.99186	6.66441	5.99385	6.36041	6.02926	6.08959	6.67616	6.65224
PDE8A	O60658	6.52468	6.77185	6.18494	6.64608	6.00626	5.93793	6.06202	6.27052	5.88864	6.2836	6.53407	6.62283	6.45581	6.05129	5.7732	5.98628
SDHC	Q99643	6.02869	5.6631	5.50644	6.01203	6.14575	5.93273	6.10012	6.00849	5.85331	5.92631	5.7633	5.76811	7.22128	7.33865	7.27146	7.46023
ITM2B	Q9Y287	7.1338	7.23784	6.8916	7.4855	6.04104	6.17352	6.69963	6.20037	6.62678	6.95391	6.9961	6.9756	4.28865	4.55154	4.72319	5.02093
SEC14L2	O76054	6.0857	6.19953	6.85636	6.05338	6.76806	6.76148	6.01182	6.40742	6.07098	6.67419	6.25752	6.36268	6.01184	6.22052	5.91457	5.34394
SGTB	Q96EQ0	6.24255	5.63221	6.14194	5.17968	6.65281	6.16146	6.01018	7.23999	5.93844	6.53373	6.51837	6.72474	6.54919	6.28117	6.17887	6.01469
RNF220	Q5VTB9	5.87914	5.36905	6.12044	5.70076	6.39827	5.87089	5.80511	6.16529	6.51894	6.52877	6.69769	6.60998	6.83115	6.56272	6.25585	6.68595
C3orf38	Q5JPI3	6.11094	5.84778	6.25009	5.51722	6.0803	6.55845	5.72136	5.97722	6.76149	5.93637	6.66254	6.7137	6.40607	5.90093	6.94062	6.61491
TMEM200A	Q86VY9	6.29695	6.32919	6.00257	6.31627	6.19406	6.3013	6.64437	6.42478	5.74266	6.2042	5.85769	5.56734	6.49609	6.55599	6.64016	6.42637
CMC2	Q9NRP2	6.48235	5.26459	5.60893	5.6302	5.87512	5.18648	5.64996	6.32705	5.53655	6.40395	6.23597	6.33041	6.69381	7.54169	7.48513	7.74781
PIAS3	Q9Y6X2	7.06744	6.86362	5.66517	6.54155	5.88518	6.09387	6.23854	6.62877	6.07543	7.28884	6.42406	6.01579	5.89331	5.7191	5.92084	5.67848
TIFA	Q96CG3	6.11231	6.24064	7.14008	6.1352	6.41783	6.8879	6.0539	6.46875	6.74036	6.62041	7.13871	7.0377	5.11237	5.03147	5.71332	5.14906
TXNDC11	Q6PKC3	6.91073	5.58594	6.36847	6.49171	6.15546	6.40503	6.06143	6.18834	5.62634	6.3331	6.58782	6.00078	7.02266	6.34807	5.69594	6.21818
GSTZ1	O43708	6.52211	6.30617	6.25205	6.3284	6.81336	5.93491	6.15227	6.10753	6.36135	5.73733	5.9919	5.67401	6.39887	6.39134	6.45435	6.57405
MSANTD2	Q6P1R3	5.77824	5.26295	6.54255	6.31889	5.7803	6.10058	6.28063	5.70766	6.06342	6.18079	5.95216	5.99447	7.28	6.98063	6.97236	6.80436
UBL4A	P11441	6.15622	4.67268	6.40899	4.8159	6.6066	5.22591	5.34174	7.16246	5.32873	6.89018	5.94006	6.74491	6.65508	7.70732	7.17407	7.16915
RGS17	Q9UGC6	6.13152	6.52494	6.12704	6.50632	6.20658	6.13723	6.25682	6.45951	6.30635	6.3459	5.94702	5.87159	6.05029	6.48653	6.39973	6.2426
ALG3	Q92685	6.0916	6.48147	5.80134	6.63779	6.10918	5.91699	6.08099	6.32452	6.28682	5.991	5.801	6.17015	6.25962	6.35325	6.85935	6.83492
TBC1D17	Q9HA65	6.01423	6.73268	6.75434	5.72509	6.20432	7.40899	5.93538	6.99966	6.33185	6.03364	6.76011	6.34773	5.78284	6.28186	5.64124	5.04604
MAP1LC3B	Q9GZQ8	5.71379	6.42782	6.64546	5.95947	5.65581	6.51275	6.6218	6.18775	6.77398	5.9378	6.14929	6.28738	6.61472	6.7484	5.56379	6.19999
ZBTB38	Q8NAP3	7.0901	6.6121	5.36609	5.87689	6.07853	6.3677	5.86062	5.87309	5.80426	6.56885	6.2668	6.21659	6.40244	6.8339	6.61177	6.17027
SCYL3	Q8IZE3	6.53913	6.05566	6.41365	6.29056	6.29322	5.73323	5.95422	5.40792	7.10903	6.57062	6.55656	6.79605	6.25309	6.24799	5.76924	6.00983

SELENOF	O60613	6.01012	5.35911	5.51902	5.30824	6.17035	5.7871	5.84821	6.31478	5.49521	6.30727	5.91224	6.5456	7.27666	7.22586	7.37776	7.54247
MT-ND5	P03915	6.08809	6.23392	5.25573	6.61416	5.73773	5.79915	6.38904	6.49494	6.65614	5.37504	6.54758	5.61154	6.82557	6.80165	6.8088	7.6609
PBX2	P40425	6.87227	6.96693	5.77125	7.24081	6.74937	6.87296	5.9453	7.08163	5.96969	6.68459	6.63454	5.64743	4.96802	5.63409	5.59457	5.36655
NES	P48681	6.54338	6.2789	5.91184	6.64002	6.15631	5.88457	6.40806	6.01913	5.95981	6.28538	6.53868	6.3707	6.6556	6.03418	6.17155	6.14189
CDC25B	P30305-3	6.9373	7.28598	7.8666	6.2943	5.92627	7.12351	6.49263	6.87355	7.73222	7.60199	8.4235	7.87026	3.1027	3.70747	3.08931	3.67239
MBD4	O95243	6.61044	6.86388	6.84763	7.14262	6.18797	6.17073	6.46641	6.32094	7.01852	6.85149	6.50133	6.71198	4.90883	4.99599	5.17812	5.22314
CTSF	Q9UBX1	7.26112	7.03093	6.14887	6.13918	6.82835	6.48828	6.2941	6.36214	6.67051	6.1551	5.89363	5.5835	6.61554	5.46343	5.54114	5.52417
GPATCH2L	Q9NWQ4	6.04003	6.92825	6.62662	6.91164	6.04817	6.39789	6.84766	6.59049	5.70395	5.66407	5.99649	6.10746	5.54226	6.26611	5.89959	6.42932
SLC38A2	Q96QD8	5.96232	7.07209	5.82756	6.32593	5.5075	6.5518	5.96599	6.47586	6.27415	6.52871	5.7098	6.34247	6.45689	7.00885	5.77917	6.21093
VPS26C	O14972	5.71615	5.76606	6.09115	6.12971	6.07683	6.18756	5.69724	6.0868	6.20122	6.38172	6.67157	6.42909	6.39103	6.89562	6.89465	6.38361
CERS5	Q8N5B7	6.84974	6.53238	5.55696	6.71159	6.30904	6.24884	6.70954	5.90486	5.82957	5.44905	5.3528	5.31397	7.11015	6.60608	6.64306	6.87236
NKAPD1	Q6ZUT1-2	6.01299	6.43986	6.28229	6.13635	6.11545	5.44407	6.57947	5.96763	6.3845	6.88964	6.65929	5.39004	5.50891	6.45107	7.42406	7.31439
ATPSCKMT	Q6P4H8	6.24523	6.62238	6.26264	6.42455	6.23083	6.38274	6.70246	5.77085	6.17501	6.07342	5.54625	5.9487	6.36417	6.34852	6.51344	6.38881
SEC61B	P60468	6.16768	7.43124	5.5473	7.10698	6.09763	6.75494	6.12492	6.96533	5.27598	6.01375	4.81974	5.63208	6.43999	6.63035	6.32894	6.66315
LMCD1	Q9NZU5	6.15394	6.11726	7.1593	5.89581	5.86432	6.71272	5.70486	6.6441	5.89373	6.62474	7.07669	7.13575	5.88899	6.19734	5.34194	5.58852
TOMM7	Q9P0U1	6.53548	5.57677	6.21968	6.43458	6.28523	5.35377	5.83239	5.98059	4.83805	5.80293	5.67251	6.8802	6.46654	6.71861	7.52685	7.87581
SRXN1	Q9BYN0	6.43388	5.26067	6.68917	5.12175	5.97936	5.83052	5.70479	7.93069	5.58416	6.42247	6.24813	7.91918	4.14242	7.41076	6.12956	7.1925
POP7	O75817	6.70967	6.64156	6.29599	6.48115	6.22673	6.24759	6.26276	6.31763	6.73909	5.9746	6.49875	5.94375	6.25826	5.79863	5.92755	5.67628
LIMK1	P53667	6.89687	6.43317	6.88471	5.94436	6.29553	6.86661	5.90202	6.72856	6.54494	6.24234	6.4715	6.36882	5.84439	5.71303	5.76975	5.0934
NR2C2AP	Q86WQ0-2	5.60187	5.53178	6.58379	5.21422	6.30964	6.60716	6.05034	7.54833	6.52238	6.79035	6.78588	7.09067	5.33175	6.27891	5.74822	6.00472
TECPR1	Q7Z6L1-4	6.68544	6.64743	7.62449	5.76697	6.01619	6.37006	6.49965	6.6394	6.08009	6.30123	6.36454	6.90798	5.56399	5.40975	5.81148	5.31132
CARD8	Q9Y2G2-5	6.13285	5.52424	6.65944	5.0356	6.08505	6.20322	6.18171	6.74952	5.728	6.51862	6.40602	7.33479	6.68184	6.71013	5.93509	6.11388
MBLAC2	Q68D91	6.12605	6.57316	5.76615	6.17813	6.61866	6.03099	6.36802	6.50581	6.00061	6.01607	6.57611	6.50907	6.27314	6.18858	6.49749	5.77194
SLC25A29	Q8N8R3	6.23165	6.23423	6.04926	6.32637	6.01586	6.53335	6.39388	6.00099	6.27654	6.86092	6.27224	5.96521	6.54338	6.16518	5.82119	6.30976
STARD3	Q14849-3	6.65288	6.98473	6.14468	7.33569	6.4751	6.13692	6.54729	5.52106	6.65794	6.71515	6.80635	6.18037	5.63864	5.57346	5.4829	5.44684
OGFOD3	Q6PK18-2	6.44188	6.65795	5.51256	7.82373	6.1669	6.08743	7.12172	6.05525	6.41156	5.9614	6.42198	5.63612	5.88015	5.80263	6.08131	5.93742
CALB2	P22676	6.38908	6.45158	5.99615	4.60217	6.09751	5.88875	5.56262	7.06659	5.78537	7.07805	6.26415	7.11858	6.61224	7.31286	6.51945	7.12125
ZNF483	Q8TF39	7.66285	7.42571	5.95484	6.48815	6.89711	6.52615	7.38025	6.32366	6.20586	6.16518	6.4205	6.48924	4.4138	5.59975	4.88093	5.16602
NDST2	P52849	6.23686	6.42162	5.95876	6.71246	6.48923	5.8843	6.13367	6.03239	6.11141	6.14364	5.70624	6.25685	6.56406	6.10481	6.46063	6.78308
RNF5	Q99942	5.84951	5.727	5.84282	5.66059	5.91335	5.84083	6.24737	6.41033	5.5556	6.03953	5.82309	6.7155	6.59005	6.94811	7.42446	7.41186
CUTC	Q9NTM9	5.69889	5.68569	5.80047	5.5848	6.12577	6.84277	5.30432	5.46551	6.04596	6.30215	5.52645	6.61076	7.13478	7.84128	7.13042	6.89998
NRP2	O60462	5.97939	6.44072	5.8267	6.35538	5.85973	6.23225	6.19517	5.98075	6.18367	5.84802	6.51875	6.52126	6.60489	6.40144	6.55129	6.50059
AGTRAP	Q6RW13	6.95037	6.90675	5.79014	5.79106	5.82808	6.80813	5.92943	6.73346	6.49432	6.76425	5.88657	5.87881	6.02537	6.41571	6.0082	5.78933
AGPAT1	Q99943	6.30753	6.60494	5.70408	6.38624	5.8108	5.97666	6.4738	6.29376	6.23588	6.52192	5.88767	6.38333	6.35406	6.47569	6.29445	6.2892
HSPBAP1	Q96EW2	5.50374	6.11973	6.45854	5.46873	5.9619	6.18433	5.77396	6.39695	6.06429	6.50782	6.53872	6.59597	6.54446	6.88282	6.5247	6.47335
AP5B1	Q2VPB7	6.41327	5.70271	6.89429	5.94985	6.29514	5.83636	6.21096	6.41457	6.32705	6.96545	6.88799	7.51226	5.23849	5.72912	5.62168	6.00082
TMEM185B	Q9H7F4	6.60864	7.33789	6.34639	6.384	5.82988	4.97322	5.86876	6.47739	6.27377	6.17868	7.0729	5.98415	6.06664	5.82631	6.50836	6.26302
GGPS1	O95749	5.81672	5.32104	6.44927	4.75342	6.42818	6.24673	6.08939	7.09527	6.08984	6.41777	6.34629	6.90416	6.38794	6.57717	6.73598	6.34082
SLC25A35	Q3KQZ1	6.48861	6.28412	6.33413	6.49151	6.06986	5.99146	6.34363	5.44525	5.84216	6.42903	5.93129	5.75375	6.97196	6.20117	6.85135	6.57071
LDLRAP1	Q5SW96	6.21078	6.84204	6.88897	6.41135	5.79708	6.44125	6.02012	5.96539	6.28464	6.76747	6.33343	6.51037	6.02433	5.84324	5.97484	5.68471
GINM1	Q9NU53	7.36016	7.81586	7.23979	7.86418	6.61578	6.13667	6.60287	6.2303	6.71527	6.84923	6.92938	6.8913	4.0253	4.42274	4.09036	4.21081
CHST10	O43529	6.35608	6.64471	6.68401	6.69933	6.02921	6.45483	6.83336	5.86818	6.6231	6.54412	7.06129	6.87271	5.54396	5.50403	5.20424	5.07684
GLB1L2	Q8IW92	6.23216	6.69709	6.02749	6.44558	6.16298	5.98601	6.71452	5.8344	6.1401	6.24178	6.35063	6.14228	6.09262	5.83397	6.41758	6.68081
TIMM17B	O60830-2	6.26119	5.90784	6.19583	6.28405	6.09805	6.2765	6.67258	5.73924	6.06825	6.12574	5.77862	6.35658	6.67097	6.35069	6.7305	6.48768
BEND7	Q8N7W2	6.34948	6.62969	6.50623	6.88827	6.02497	6.206	6.03619	6.01728	6.50096	6.46275	5.8298	6.09502	5.98673	6.18547	6.11827	6.16289
TOM1	O60784	6.79421	5.53298	6.15668	5.16663	5.84682	6.60451	6.22578	6.70323	5.72978	6.34576	7.25027	7.09648	5.9909	7.1352	6.24037	5.1804
TMEM205	Q6UW68	5.91176	6.87766	5.84509	6.29748	5.69774	6.00713	5.74894	6.27333	5.82108	6.43796	5.87534	6.17586	6.39147	6.59616	6.67851	7.3645
BAD	Q92934	8.65729	8.91428	7.37965	8.76754	7.2788	6.74926	6.27303	7.47371	7.44389	8.33792	7.32585	7.85941	1.68224	2.42477	1.43207	2.0003
WDR7	Q9Y4E6	6.51084	6.36771	6.43724	5.96544	6.36668	6.27629	6.19068	6.39782	6.77198	6.86522	6.57467	6.40915	6.05286	5.61305	5.74471	5.45568
LETMD1	Q6P1Q0-7	6.63061	6.58333	5.74557	7.10621	6.20263	6.21115	6.40804	6.69497	5.46632	5.96607	6.11204	6.03876	5.98078	5.98829	6.23947	6.62576
NGDN	Q8NEJ9	6.43503	6.39678	5.70037	6.51882	6.15019	5.76258	6.31323	5.81573	6.22018	6.07896	5.67421	5.83762	6.74159	6.37163	7.07257	6.91051
IAH1	Q2TAA2	6.52645	5.64621	6.37366	5.39529	6.25365	6.48064	6.68341	6.97026	6.38048	6.53575	6.53233	6.87004	6.08104	6.1324	5.6834	5.45501
CNOT6L	Q96LI5	6.05089	6.0577	6.40002	6.25967	6.12761	6.86518	6.39974	6.34723	6.11558	6.64513	6.74004	6.83514	5.71667	5.6919	5.97811	5.76936
TRMT112	Q9UI30	4.31088	4.58365	4.62601	4.90122	5.48323	5.1994	5.62529	8.72779	5.52209	6.34461	5.45682	6.92227	4.29216	9.64248	8.43036	9.93174
ARHGAP19	Q14CB8-2	6.2653	6.028	6.23533	5.81809	5.9774	5.72653	6.49994	5.75284	6.69032	6.32831	6.46015	6.10945	6.45965	6.63216	6.64621	6.37032
KLHL13	Q9P2N7-5	6.42911	6.54639	6.9953	6.32922	6.29705	6.31899	6.42025	6.2188	6.53479	6.93395	7.11282	6.97352	5.12946	5.33167	5.38581	5.04288
DCAF15	Q66K64	5.15381	5.75173	6.2095	5.86813	5.69562	6.13379	6.02704	6.04784	5.96215	6.81904	7.29872	6.63579	6.24699	6.25898	6.80685	7.08402
COX19	Q49B96	6.72889	5.67881	5.98878	5.79177	6.89958	5.49949	5.56991	6.76677	5.27169	6.13303	5.52726	6.13205	6.88422	7.20277	6.90281	7.02216
FN3KRP	Q9HA64	5.67546	5.35559	6.07357	5.32964	6.54781	6.44049	5.91495	7.24152	6.97003	6.43746	6.05266	6.77473	6.69049	5.85858	6.00485	6.63215
BSDC1	Q9NW68	6.65613	6.14495	6.0845	5.6248	6.13508	6.03183	5.81312	7.22411	6.04209	5.63132	6.54516	6.30052	7.33724	6.68693	6.00838	5.73384
METTL5	Q9NRN9	6.44876	6.23449	7.26076	6.09785	6.62275	6.58037	5.60882	6.89616	7.38765	6.52275	6.49193	7.17258	4.97	5.07916	5.2231	5.40288
ORMDL3	Q8N138	6.07394	5.6833	5.4024	6.1849	5.87446	5.70394	6.15963	5.92161	6.17262	6.07946	5.93501	6.01981	6.34854	7.53094	7.39359	7.51585
FAM32A	Q9Y421	6.14072	6.45247	6.06369	6.14025	6.26462	6.60794	6.17375	6.61151	6.29695	6.58596	6.42731	6.21505	5.56249	6.59052	6.07306	5.7937
CCDC138	Q96M89	6.96211	6.64559	6.80953	6.86967	6.41579	6.43059	6.0799	6.11494	6.60615	6.48818	7.25815	7.54022	4.64409	5.37968	4.44833	5.30709
ZBTB7A	O95365	6.23129	6.63436	6.25964	6.42721	6.56612	5.92974	6.57952	6.17832	6.65614	6.30595	6.41816	5.80091	6.40911	5.6091	6.18234	5.81209
DCXR	Q7Z4W1	6.35529	6.04926	6.14067	6.62944	6.35798	6.01416	6.46652	5.88425	6.1266	6.07313	6.2578	6.33168	6.27763	6.07615	6.63576	6.3237
RBP7	Q96R05	6.53406	6.5387	6.52822	6.86983	7.00887	7.12611	6.57063	7.48116	6.18999	6.89191	6.74214	6.47381	4.98171	5.09497	4.76441	5.20347
PCGF6	Q9BYE7	6.22667	5.68077	6.39189	6.46478	6.73785	6.48749	6.1227	6.18042	6.34199	6.32393	6.52998	6.55899	5.95809	5.89487	5.99299	6.1066
ZNHIT2	Q9UHR6	6.68088	6.19046	6.71305	5.67934	6.16787	7.75807	5.82405	6.20907	6.53293	6.82557	6.8035	6.67479	6.12523	5.11235	5.29635	5.40648
TRIM3	O75382	6.19992	6.40576	6.67935	5.94938	5.98412	6.34621	6.22835	5.7754	6.48756	6.99147	6.88228	6.56191	6.23571	5.96547	5.53705	5.77006
FAM92A	A1XBS5	6.58141	6.46686	7.39269	6.32083	6.44737	6.4706	6.30685	6.65756	6.55971	6.39762	6.7717	6.98405	5.31335	5.07331	5.15558	5.1005
SNX24	Q9Y343	6.29215	6.27417	7.10968	6.00198	6.42639	6.85827	6.53131	6.42333	6.25381	6.92065	6.65402	6.47645	5.26019	5.31426	5.47337	5.72996
RFK	Q969G6	5.30334	6.20276	6.48079	6.04664	6.205	7.14915	6.49079	7.11826	6.96512	7.00041	7.4271	7.30207	5.02419	5.23766	5.12837	4.91834
R3HDM2	Q9Y2K5	6.15682	7.13847	6.1715	6.25724	5.84919	6.82454	5.91309	6.30845	6.36125	6.37536	6.89007	6.42137	5.81783	5.99623	5.55239	5.9662
CCDC117	Q8IWD4	4.96834	5.27829	6.52117	5.5917	6.03851	6.13376	6.50591	6.20656	6.24089	6.23087	7.08277	6.59674	7.02357	6.80815	6.0477	6.72506
C18orf21	Q32NC0	6.51263	6.19285	6.01824	6.77819	6.15237	5.97739	6.28448	6.14462	5.94153	5.94994	6.36098	5.84689	6.54747	6.25173	6.61707	6.42362
KIAA1841	Q6NSI8	6.19672	6.73847	6.49464	6.72596	5.96144	6.40426	6.48766	6.40087	6.3108	6.40738	6.61688	6.75734	5.22764	5.39665	5.74742	6.12588
SYPL1	Q16563	6.70172	6.4069	5.85896	6.16488	6.34631	5.7563	6.27911	5.98898	6.49396	7.11056	6.70727	6.6279	5.74312	5.82155	6.17527	5.81721
BOLA3	Q53S33	6.56103	6.31906	6.50442	6.60699	6.61089	5.99231	5.24852	5.94665	6.42565	6.43578	6.36333	6.24301	6.50772	5.96535	6.16935	6.09992
ROR1	Q01973	6.32346	6.42948	6.33453	6.99945	6.42487	6.06151	6.57616	6.31624	6.69254	6.56965	5.45742	5.76498	6.12669	6.15394	6.57111	6.09794
RPE	Q96AT9	5.53787	4.55811	6.19025	4.09953	4.59238	4.1893	5.26553	7.46337	4.80949	5.70495	5.06304	6.55506	4.77563	10.8234	8.80284	11.5692
NBEAL2	Q6ZNJ1	6.55741	6.60291	6.46498	6.80327	6.35704	6.37847	6.901	6.63782	7.33012	6.33346	6.30337	6.74173	5.45863	5.24794	4.86016	5.02169
POLL	Q9UGP5	5.34301	5.83385	6.53247	5.6198	5.75049	6.90958	5.66994	6.07041	6.25986	6.49879	6.83358	6.46638	6.51998	6.65853	6.8697	6.16363
GOSR2	O14653-2	6.7281	7.09531	6.69967	6.1608	6.57951	6.57201	6.48502	5.96834	5.65683	6.25309	5.46537	5.79738	6.44919	5.63285	6.26946	6.18709
MAP2K7	O14733-2	5.72822	6.73722	6.13968	6.13182	5.64707	6.9212	6.79554	6.90554	6.59133	6.38811	7.11841	6.96873	5.3218	5.75694	5.23291	5.61546
COX20	Q5RI15-2	6.68279	6.56557	6.21124	6.56254	6.2382	6.25254	5.97303	6.12983	5.95572	6.00884	5.83461	5.80142	6.69827	6.0877	6.40989	6.58781
R3HDM4	Q96D70	5.12344	5.85351	5.66024	5.33806	6.9009	7.38468	6.68215	6.12529	6.14761	5.89815	6.15567	6.13387	6.99585	6.51829	6.54172	6.54056
GEMIN8	Q9NWZ8	6.20674	5.87672	6.80459	6.17495	6.56944	6.133	5.86779	6.86757	6.30431	6.3639	7.36965	7.22326	5.92888	5.28311	5.8368	5.18929
SLC38A1	Q9H2H9	6.24184	6.9193	6.06615	6.21744	6.16939	6.41175	6.44458	6.74573	6.26817	5.78122	5.77657	6.26423	6.04446	6.03369	6.50714	6.10833
BBS7	Q8IWZ6	6.74847	6.48321	6.45917	6.49749	6.76824	6.48201	6.03568	7.08511	6.86739	6.20843	6.41941	6.50849	5.21119	5.78153	5.23954	5.20464
FDX1	P10109	6.34709	6.32199	5.73633	5.55747	6.77919	6.10735	6.73057	5.1913	6.75529	5.84325	6.17414	7.404	6.44107	5.36283	6.8385	6.40962
EXT1	Q16394	6.29613	6.68882	6.11642	6.40173	6.28281	6.17848	6.76403	6.31669	6.13995	5.98889	5.54363	6.05612	6.38574	6.33912	6.3409	6.16053
MGST1	P10620	5.78145	6.3163	5.71609	6.32526	6.97761	6.11431	6.36879	6.33184	6.17139	5.53609	6.53852	6.11166	6.44031	6.36404	7.06647	5.83988
TNK2	Q07912	6.2683	6.99181	7.37713	6.99633	5.86325	6.62663	6.69021	6.2628	6.3661	6.72255	6.92012	6.8904	4.9075	4.97669	4.84797	5.29221
CIR1	Q86X95	6.05399	6.5955	5.99232	6.40807	6.20987	6.03997	6.15792	5.92564	5.91748	6.258	6.25219	5.99546	6.32345	6.95524	6.22869	6.68622
KLHL22	Q53GT1	5.86549	6.57233	6.53646	6.49811	5.71488	6.29499	6.40461	6.50122	6.19689	6.24189	6.37836	6.31428	6.17438	6.2027	6.12188	5.98154
DPAGT1	Q9H3H5	6.11218	5.75621	5.93372	6.5584	6.02411	5.87653	6.10983	6.04025	6.31165	5.92846	5.79807	5.68449	7.10649	6.87717	6.83397	7.04848
SSX2IP	Q9Y2D8	6.56477	6.59254	6.91886	6.524	6.20856	6.39573	6.08958	6.68706	6.37789	7.07541	6.80682	6.46992	5.54966	5.7105	4.93775	5.09095
GPAT2	Q6NUI2-4	7.19251	7.17679	7.1449	7.97992	6.71623	6.956	7.15651	6.2274	7.58853	6.48305	7.59463	7.51398	3.73294	3.67975	3.35142	3.50544
AFTPH	Q6ULP2-9	6.35643	5.94973	6.95912	6.34771	6.34232	6.8481	6.24938	6.24126	6.44835	6.58252	7.21813	6.79524	5.72802	5.11672	5.3304	5.48659
GLRX2	Q9NS18-2	6.58524	7.09771	6.54183	7.26423	6.38406	5.91326	6.10107	6.72097	6.59719	6.39279	7.06977	6.45516	5.51586	5.08331	5.2736	5.00394
SLC26A6	Q9BXS9	5.97103	6.65048	6.1329	6.73537	5.82755	6.23867	5.90695	5.62429	6.58275	6.22158	5.74405	6.34077	6.63846	6.44336	6.71916	6.22264
YIF1A	O95070	6.22607	6.50816	6.17733	6.68692	6.40342	5.80756	5.90011	5.71775	6.14899	5.97329	6.10171	5.89014	6.63954	6.35494	6.99711	6.46695
COMMD6	Q7Z4G1-2	5.95687	5.0446	6.30204	4.48889	5.89336	5.80078	5.31935	6.82063	5.81679	6.43206	6.09072	6.46296	5.79091	8.66627	7.44166	7.67211
TRAPPC1	Q9Y5R8	6.2696	5.84825	6.75291	5.68259	6.43513	6.44546	6.3539	6.63841	6.38848	7.10038	6.76793	6.72199	5.75863	5.41011	6.13995	5.28628

NMES1	Q9C002	6.24938	5.7126	6.63092	6.40051	5.52626	5.54235	6.06117	6.03861	6.04121	5.28974	5.33957	5.27439	6.32812	8.00399	7.32611	8.23507
TFAP4	Q01664	6.17356	6.65506	6.52834	6.54205	7.45513	6.20411	6.58905	6.55506	6.35944	6.06758	6.15512	6.28988	6.70296	4.81064	5.11367	5.75836
TECPR2	O15040	5.67478	6.18805	6.88452	6.15402	6.27375	6.23549	6.15964	5.78289	6.48014	6.33721	6.2215	6.64146	6.09993	6.56547	6.29074	6.01042
ZNF280B	Q86YH2	6.28209	6.8003	6.21854	6.95342	6.67478	6.15495	5.75914	5.92892	6.71107	6.15704	6.57562	6.14987	6.24788	5.66648	6.03748	5.68242
IGSF8	Q969P0	6.75542	6.64085	6.06725	7.01194	6.15747	6.33035	6.52208	6.12473	6.20219	6.50066	6.20748	6.28191	5.95347	5.2418	6.05805	5.94435
FAM118B	Q9BPY3	5.95862	5.76245	6.1212	4.99005	5.67608	5.76486	5.64229	5.91804	6.37563	6.72419	6.48567	5.90178	7.08404	7.31261	7.5767	6.7058
UBE2B	P63146	6.02508	5.59962	6.64982	5.21094	6.19385	5.74428	5.83849	6.79622	5.43721	7.21763	6.21418	7.55665	5.15477	7.36785	6.22676	6.76664
GPCPD1	Q9NPB8	6.26932	6.47837	6.32926	5.85324	5.99595	7.06944	6.36497	6.58972	6.09493	5.98303	6.62516	6.50046	6.13206	5.98035	5.81856	5.91518
MRPL51	Q4U2R6	6.21409	6.45052	5.58501	6.56609	6.63851	6.16488	6.2718	6.21855	5.92612	6.22304	5.74585	5.44062	6.63863	6.58336	6.55193	6.781
RAPH1	Q70E73	5.91883	5.53613	6.94587	5.76062	6.60291	6.59259	6.93639	6.06992	6.77322	6.02907	6.97	6.59242	6.38911	5.91862	5.38669	5.57763
MRPS36	P82909	6.09897	5.80566	6.32639	6.77424	6.10525	5.55155	5.81688	6.25498	6.21073	6.103	6.63247	6.84571	7.00922	6.27616	5.97543	6.21337
SMAP2	Q8WU79	6.20432	6.07283	7.48379	6.12036	6.16923	7.00382	6.11127	6.52937	6.23259	6.56116	6.76623	6.43585	6.52611	5.90508	5.18988	5.58811
RIC1	Q4ADV7	6.06379	7.16476	6.57197	6.22557	6.32343	6.47899	6.1661	5.94388	6.49894	6.58863	6.83649	6.56123	5.29217	5.8961	5.59473	5.79323
LYRM2	Q9NU23	5.91171	5.82711	6.48395	6.31816	6.08858	5.87337	6.34197	6.49514	5.51431	6.64063	5.91341	6.02458	6.44538	6.6018	6.75656	6.76333
ALG11	Q2TAA5	5.98356	6.44619	5.63359	7.07578	5.84037	5.68642	6.05801	5.86603	5.84799	5.99055	6.50309	6.09679	6.88734	6.47188	7.14996	6.46248
VPS54	Q9P1Q0	5.8453	6.13624	6.78	6.14563	5.91641	6.19903	5.78869	6.57457	7.05973	6.62644	6.13292	6.35965	5.70303	5.89802	6.29326	6.54108
SSR4	P51571	6.60704	6.71368	5.76216	6.57987	7.17926	6.52258	6.01647	6.57817	6.04972	5.5946	5.93581	5.72905	6.37087	6.10471	6.57467	5.68134
PNPLA2	Q96AD5	6.0689	5.89115	6.751	5.76276	6.96538	7.26089	6.99432	6.70126	6.09801	6.11698	6.6798	6.57781	5.80253	5.40241	5.44619	5.48061
UNC93B1	Q9H1C4	6.33988	6.53686	6.26204	6.23918	5.99339	5.90059	5.90655	6.41447	6.32672	6.47285	6.19121	6.07406	6.21557	6.44853	6.20376	6.47436
IFI30	P13284	5.6651	6.2306	5.90878	5.76403	5.85441	6.09177	5.18444	6.31023	5.62722	5.7344	5.89639	6.37843	6.75483	7.63173	6.82971	7.23792
PVR	P15151	5.60959	7.063	5.48684	6.37518	6.08726	6.00986	6.26603	6.55374	6.50335	5.52377	5.23006	6.39316	6.71069	6.02654	6.9282	7.23273
UBE3D	Q7Z6J8	6.59247	6.61747	7.31498	6.46166	6.26522	7.06604	6.4288	6.48352	6.51444	6.15249	6.59881	6.58492	5.13874	5.42259	5.18785	5.16929
FBXO10	Q9UK96	6.27367	6.87448	7.06372	6.69054	5.9881	6.14068	5.74613	6.07052	7.23833	7.53473	6.63592	6.96553	4.84518	5.13185	5.58293	5.21768
SYT1	P21579	6.28522	6.34431	5.79288	6.30942	6.39498	5.85479	6.21204	5.89255	6.37214	6.23943	5.73714	5.78264	6.53564	6.68977	7.00323	6.55381
SNRPC	P09234	6.04476	4.97374	5.52863	5.4466	6.66144	5.25123	5.68412	6.36614	5.1527	6.43298	6.13885	7.00903	6.5397	7.30349	7.56215	7.90444
PRXL2B	Q8TBF2-3	6.21295	6.17145	7.06145	5.92832	6.25788	6.8031	6.41458	6.89809	6.67933	5.86373	6.38525	6.54131	5.78233	5.77598	5.18312	6.04115
TMEM14C	Q9P0S9	6.50151	6.2712	5.97244	6.41825	5.97592	6.85092	4.9151	6.11304	5.53929	6.58657	5.54063	6.17083	6.98113	6.86037	7.22951	6.97429
CNTN1	Q12860	5.86189	6.26761	6.23417	6.54067	6.44431	6.07718	6.05705	6.73175	5.82174	6.43663	5.81452	5.87634	6.43506	6.29692	6.63582	6.46832
PIP5K1A	Q99755	6.58844	6.16818	6.69868	6.9642	6.5764	5.6031	6.72004	6.12031	6.49712	6.20679	6.3495	6.08271	6.49386	5.16394	6.04477	5.72198
PIP5K1C	O60331-3	6.21267	5.91611	6.96627	5.25221	6.39117	7.45152	6.39135	5.97294	6.4534	7.0616	6.39796	7.01231	5.98124	5.74169	5.58174	5.21551
PDCD7	Q8N8D1	6.23036	5.85133	7.13253	7.701	7.33657	6.26679	6.2622	5.19903	5.71981	6.37202	6.12057	6.17842	6.07471	5.53064	6.01353	6.01047
PFDN4	Q9NQP4	6.82202	5.5178	6.53544	4.37506	5.84214	5.289	5.72027	6.83482	5.53159	6.73391	6.04093	6.4128	5.77005	7.94145	7.28099	7.35173
RUFY3	Q7L099-3	5.43126	6.03281	6.6758	5.46053	6.74433	6.73869	6.1157	6.73778	6.07651	6.899	6.92081	6.68327	6.10897	5.86854	5.47394	6.03204
HSPA1L	P34931	6.41855	6.3828	6.12478	6.70888	5.78101	5.97814	6.17832	6.18475	6.59724	6.11424	6.65639	6.87851	6.30276	5.77468	6.30956	5.60939
SLC39A11	Q8N1S5	5.95655	6.55301	5.65352	5.80966	6.28189	5.23366	6.86815	6.01592	5.82099	6.20997	6.25814	6.45148	7.01953	6.47758	7.1231	6.26685
TPM1	H0YK48	6.58332	6.44925	6.91461	5.67381	5.85623	5.62609	5.81672	6.27377	6.33266	6.15887	6.62966	6.48199	7.2507	6.36363	5.78374	5.80494
ASAP3	Q8TDY4	6.04235	6.02007	6.94132	5.88186	6.24566	6.54188	6.18581	6.54476	6.58993	6.9831	6.70815	6.35808	5.27894	6.14759	6.06216	5.46835
YBX3	P16989-2	6.72231	6.02338	6.76845	6.38551	6.14233	5.98566	6.76256	6.07469	6.67122	6.29254	6.70895	6.45211	6.11396	5.56523	5.7111	5.64793
PTMA	P06454-2	6.36031	6.08491	6.88917	5.91781	5.88917	6.38566	5.5194	7.23808	6.2942	6.23455	6.56165	6.49347	5.9251	6.62218	5.67112	5.91325
NXT2	Q9NPJ8-3	5.84037	5.6418	5.68806	6.65041	6.08851	5.78455	6.42422	6.32717	5.96948	6.05201	6.22253	6.58628	6.50392	6.49086	6.68838	7.04144
MARCHF6	O60337	7.36499	7.77719	6.81956	7.86275	6.41517	6.07445	6.28364	6.31594	6.51381	6.66931	6.40382	6.56521	4.58685	4.79334	4.52648	5.0275
ZNF131	P52739	6.62764	6.32169	6.20166	6.56739	6.07472	5.56486	6.30621	6.24493	6.2807	6.73657	5.89199	7.10177	5.42631	6.33063	5.87961	6.44333
MSTO1	Q9BUK6	5.88147	5.43468	6.51876	5.43058	6.75835	5.96873	6.32966	6.63281	6.95298	6.72049	7.07795	6.59655	5.87533	5.77568	5.88042	6.16556
PCDHB2	Q9Y5E7	6.07852	6.4236	5.69702	5.8873	7.17042	6.55856	6.71479	6.69598	5.65428	5.93005	5.76362	6.38748	6.46822	6.2615	6.05679	6.25186
PCDHB14	Q9Y5E9	6.11376	6.07632	6.21594	6.81697	6.33703	6.42908	6.79344	6.5275	6.5793	6.77773	6.25541	6.50229	6.57162	5.67685	5.63461	5.59215
PCDHB6	Q9Y5E3	6.27395	6.13014	5.88025	6.49465	6.5828	6.1337	6.45917	6.7212	5.74446	6.14727	5.68855	6.08259	6.42215	6.01056	6.37575	6.85279
TYSND1	Q2T9J0	6.83795	5.88971	6.43495	6.85299	5.77008	6.77488	6.45305	5.81139	5.91456	5.84016	6.12843	6.53765	6.05083	5.95839	6.31618	6.42881
SPATS2L	C9JGM8	6.21374	6.55666	6.5332	6.03421	5.24828	5.93572	5.97082	6.2587	6.73631	6.06875	6.11608	6.70226	6.01283	5.60844	7.19326	6.81073
SMARCD3	Q6STE5	5.56478	6.1344	6.02897	5.78007	5.96504	6.48371	6.31318	6.10658	5.58226	5.90399	6.37936	5.87202	6.91699	6.91149	6.70845	7.34872
RIDA	P52758	6.12689	4.75321	5.23395	5.003	5.60578	5.33222	5.42833	6.47583	5.33439	6.63812	5.97885	6.9932	6.20446	8.75252	8.09046	8.0488
SRC	P12931-2	6.37272	6.18556	6.16505	6.12079	5.71027	6.10044	6.43455	6.12005	6.26057	6.3879	6.22855	6.28284	6.14766	6.49093	6.52024	6.47188
PARD3B	Q8TEW8	5.81373	6.6787	6.08049	7.16574	5.84414	5.87323	6.11416	6.17878	6.34823	6.41649	6.54236	6.35703	6.54868	6.04771	6.228	6.84252
SIDT2	Q8NBJ9-2	6.56095	6.66266	6.45837	6.86666	5.64739	6.18023	6.32606	6.0499	5.96531	6.85855	6.15357	6.58551	5.80124	6.37974	5.98916	5.51472
TCOF1	Q13428-7	5.18104	5.48816	5.67097	6.89977	5.76968	5.90601	5.31551	5.54868	5.99214	5.79081	5.18605	5.24832	7.46517	7.7041	8.3894	8.44417
CELF2	O95319-3	6.56279	6.50158	5.94297	6.07398	6.22553	6.27199	6.1845	6.1232	6.24239	6.17063	6.54687	6.25476	6.18089	5.76594	6.46168	6.49029
ZNF706	Q9Y5V0	6.53458	6.74059	6.92299	6.41232	6.16747	6.34337	5.91465	6.55309	6.57661	6.7557	6.26333	6.35773	4.95783	5.892	5.57765	6.03008
FOXO3	O43524	6.4328	6.29843	7.09864	6.30756	6.22543	6.55903	6.3734	7.04856	6.28926	6.15306	6.86551	6.80469	5.81151	5.70918	5.02599	4.99696
PPP2R5B	Q15173	5.9587	6.05355	6.51109	6.28013	5.58354	6.68596	5.69951	6.04467	6.17377	6.3296	7.37679	6.83387	6.00182	6.29612	6.43935	5.73152
TUBB4A	P04350	6.39339	6.1041	7.30238	6.58161	6.12366	6.87658	5.53973	6.7665	6.9331	6.90446	6.4984	7.07645	5.62733	5.30184	5.03969	4.93079
TRIM44	Q96DX7	6.54458	6.72286	7.06825	6.37089	6.82519	6.44954	6.23278	6.74974	6.53196	7.19982	7.04494	7.07177	4.73635	5.11172	4.73925	5.03618
MCL1	Q07820	8.1306	6.51753	6.68421	8.00613	6.43114	7.16452	7.9033	6.37429	5.89721	6.10078	6.05894	6.39482	4.30527	4.49241	4.60624	4.93261
CHCHD2	Q9Y6H1	3.95829	4.0799	3.99225	4.14772	8.49044	8.02642	7.9231	8.29987	4.60756	4.23197	4.07059	4.51126	8.96932	8.01646	7.47037	9.20449
MBTD1	Q05BQ5	6.98387	6.35384	5.94576	7.4473	6.91812	6.39094	6.60535	5.6087	6.13953	6.62975	5.9636	5.98646	6.10725	5.57931	5.7729	5.56733
ZNRD2	O60232	6.46866	4.80527	5.88105	4.08432	5.99135	5.38214	6.0242	6.4481	4.70408	5.7334	5.10142	6.07339	5.69024	9.82349	7.77135	10.0175
ZNF827	Q17R98	5.959	6.50509	5.94541	6.5612	6.12742	5.68172	5.86411	6.28505	6.57983	5.94263	6.19074	6.33632	6.48521	5.99422	6.7678	6.77427
P2RX4	Q99571-2	6.70221	7.11496	6.54509	6.80912	5.66002	6.5528	6.25666	5.53619	5.54365	6.56347	6.35131	6.03877	6.34969	6.39529	5.99075	5.59003
DYSF	O75923-13	6.23995	6.22161	6.50926	6.37956	6.04114	5.71979	6.51368	5.95204	6.13723	6.46925	6.24235	5.93617	6.20525	6.54278	6.4234	6.46653
KCNQ5	Q9NR82-6	6.65062	6.20587	6.47021	7.1999	6.05282	5.34256	6.49859	5.91509	6.4599	6.24313	6.47951	6.29051	6.03742	5.92226	6.0982	6.13342
CCDC71L	Q8N9Z2	6.36196	6.20309	5.36341	6.69644	6.16237	6.262	6.48871	5.2884	6.75139	5.30384	5.84583	5.46735	6.59659	7.86388	6.33351	7.01223
TSHZ3	Q63HK5	6.50106	6.15435	5.72469	6.42908	6.1503	6.16973	6.43667	5.4076	6.21416	6.60509	6.57414	6.19198	6.3897	6.30468	6.18351	6.56326
ZNF518B	Q9C0D4	6.75779	6.90387	5.90101	6.74409	6.22372	6.44958	6.86154	5.96021	6.25877	6.56613	6.44636	6.6544	5.57985	5.43855	5.1132	6.14094
AKAP11	Q9UKA4	6.48806	6.05966	6.12053	6.38355	6.59527	5.85712	6.55951	6.46611	5.87142	6.02916	6.74655	6.41377	5.93633	6.58537	5.49024	6.39735
TBC1D25	Q3MII6	5.81807	5.47263	6.41226	5.64694	6.42126	6.58299	6.39568	6.83461	6.26863	6.10281	5.83936	6.63941	6.33742	6.49287	6.18177	6.55328
LCMT2	O60294	6.16355	7.07382	6.63629	6.41045	6.33269	5.63526	6.80526	6.88638	6.60053	7.20841	6.50353	6.68683	6.48217	5.0181	5.17111	4.38563
DST	Q03001-11	6.20685	7.00831	5.59408	5.94027	6.27799	6.20064	6.80462	6.15115	6.72345	5.94522	5.89794	5.68297	6.70348	6.61774	6.48509	5.76019
CYTH1	Q15438	6.1187	5.91871	6.81253	5.87191	6.15607	6.58992	6.40183	5.98507	6.04137	6.31725	7.25046	6.32982	5.91885	6.29425	6.13503	5.85824
CYTH3	O43739	5.92509	6.07031	7.06492	6.7594	6.14176	5.89716	6.82	6.82975	6.59375	6.36183	6.36421	6.36283	5.89824	5.70176	5.38229	5.82671
SDHAF3	Q9NRP4	7.10955	7.08275	6.86304	6.84657	6.01997	5.62238	6.23383	5.91658	7.00338	7.01956	5.92174	7.03081	5.84873	5.57847	4.59126	5.31137
DDHD1	Q8NEL9	5.55367	7.02893	7.49379	5.37886	5.36409	6.46498	6.00294	5.93607	6.75902	6.61658	6.39204	6.94355	6.22583	5.53623	6.22251	6.08091
GLUL	P15104	6.28717	7.81743	7.08993	7.61527	6.44899	6.3934	6.07314	6.74295	7.17242	6.67842	7.18537	6.43008	4.85207	4.45235	4.40082	4.3602
RPS3	E9PJN9	6.16443	6.24297	7.39461	6.0764	5.36314	6.10033	6.44901	6.67659	6.50596	6.62028	6.92935	6.66622	6.12185	5.34732	5.98742	5.35411
ZFYVE9	O95405	6.40207	6.37473	6.65296	6.60397	6.64284	5.92971	6.24038	6.3474	6.54119	6.54186	6.41372	6.65398	5.5404	5.87599	5.87937	5.35945
VAMP2	P63027	6.46221	6.65	5.80431	6.38078	6.31306	5.51721	6.29884	6.39528	5.65506	5.88404	5.87394	6.00149	6.17634	7.14923	6.8566	6.58161
VAMP3	Q15836	6.23143	7.39246	5.23551	6.37633	6.59753	5.8781	6.41447	5.82352	6.43717	7.08435	6.22433	6.52083	5.77215	5.68023	6.25945	6.07215
RNASEH2A	O75792	6.01327	5.73056	6.74361	5.20019	6.24281	6.43726	6.16757	6.9245	5.9735	6.68173	6.70877	6.15788	5.93319	6.28006	6.66987	6.13523
KLHDC2	Q9Y2U9	7.07102	6.59242	6.60862	6.59721	5.76635	6.4686	5.97071	5.79377	6.80453	7.37005	7.02356	6.88132	5.15776	5.11476	5.65231	5.12701
CCDC127	Q96BQ5	6.53956	6.51445	5.96976	5.8817	6.53698	6.17251	6.5593	5.7939	6.17093	5.86332	6.25917	6.02814	6.47983	6.06293	6.85599	6.31155
CTPS2	Q9NRF8	6.38613	6.34946	7.39901	6.66649	5.83653	6.36097	6.5459	6.07125	7.06345	6.04247	6.40941	6.60977	5.8615	5.66215	5.56793	5.16756
TMED1	Q13445	6.16699	5.88753	5.5772	6.07042	6.39573	6.029	6.12385	5.83521	6.38055	5.93885	5.62926	6.0457	6.88416	7.09529	7.11432	6.82595
SYNCRIP	O60506-3	6.45251	6.16293	6.3781	6.4843	6.55786	6.06011	6.07012	6.70177	6.84535	6.20798	6.07297	6.01205	6.15817	6.10878	5.67011	6.05689
HNRNPR	A0A6Q8PHG0	6.64969	6.42501	5.71521	6.90499	6.28013	6.21001	6.03164	6.15330	6.13989	5.6264	6.04657	5.92507	6.68088	6.05937	6.71174	6.45801
LHPP	Q9H008	6.58283	5.64917	6.96188	5.62889	6.32282	7.01295	5.77125	5.67397	7.22086	6.09596	6.09114	6.57485	6.46939	5.84064	6.12309	5.98031
NRIP1	P48552	6.33501	6.15274	6.48694	6.44814	6.42845	6.13353	7.04118	6.14785	6.26577	5.78673	6.65503	6.29341	6.11962	5.8235	5.85281	6.02928
SGSH	P51688	4.71424	5.48767	9.58196	5.46793	4.9046	4.73435	17.118	4.72661	5.04218	5.10256	6.22366	4.74207	4.39032	4.03729	9.90494	3.82167
SNTA1	Q13424	6.42802	6.59352	6.04697	6.62918	6.40018	5.91285	6.00321	6.25958	6.72467	6.66983	6.21093	6.04603	6.35583	5.65569	6.12017	5.94334
MAST2	Q6P0Q8	6.39004	6.67846	6.74756	5.80649	6.23353	6.55639	5.85314	6.76168	6.57748	5.99049	6.54517	6.85186	5.62571	5.61407	5.84558	5.92235
ZFAND6	Q6FIF0	6.66402	6.2707	7.18788	6.1052	6.10162	6.1185	6.29274	6.78672	6.80769	6.28362	7.20174	6.66269	5.29882	5.69099	5.59869	4.92838
CHPF	Q8IZ52	7.00112	6.4187	6.4084	7.55974	6.00967	6.18853	6.86089	6.18776	6.43937	6.18255	6.66754	6.35343	5.37611	5.65032	5.68074	5.01512
SIRT3	Q9NTG7	6.87937	5.55451	6.01221	4.99174	5.79723	4.8332	6.22993	5.77296	5.33795	6.49613	5.59052	6.35216	7.20854	7.76628	7.41597	7.76131
ALKBH1	Q13686	7.15873	6.88781	7.06044	7.50464	6.79374	5.898	6.78263	6.00394	6.2078	6.54874	7.2067	6.24337	5.75197	4.93197	4.94714	4.97237
CMTM6	Q9NX76	6.56787	6.77065	5.77113	6.08781	5.76792	6.08433	6.39423	6.05456	5.76352	6.16032	5.71525	6.01293	6.72185	6.93687	6.72578	6.46499
ICAM5	Q9UMF0	6.01552	6.58122	6.13961	5.49053	6.3239	6.22106	5.83927	6.7449	6.971	5.89687	5.92653	6.20622	6.75621	6.93647	6.50639	5.4443
CHURC1-FNTB	B4DL54	6.28974	6.22366	6.84309	5.6422	6.76483	6.07076	6.05573	6.90803	6.69616	5.5705	6.89949	5.88525	6.07359	6.08882	6.06623	5.92191
EEF2K	O00418	6.64237	6.65338	6.60122	6.27926	7.30034	7.92582	6.59462	7.29269	6.67129	6.62177	6.74879	6.661	4.61071	4.82473	4.12289	4.44911
ZNF277	Q9NRM2	6.0815	6.18357	5.99386	6.14847	7.03612	6.56621	6.67652	6.21858	6.58563	6.11474	5.44972	5.28333	6.70066	5.83924	6.57134	6.55048

PAG1	Q9NWQ8	6.88553	6.26958	6.14887	6.60484	5.77893	5.64765	6.22279	5.54037	5.9754	6.31929	6.33874	6.04497	7.26406	6.39973	6.61134	5.94792
ARL6IP6	Q8N6S5	6.29124	6.25007	5.52902	6.75411	5.77001	5.75221	6.28556	5.75485	6.18104	5.84928	6.03109	5.65451	6.84746	6.81645	7.31629	6.91682
HMGN2	P05204	6.45776	6.02944	5.11682	6.63558	5.74021	5.66159	6.3304	5.43797	5.80654	6.26307	6.31547	5.94839	6.1389	6.79462	7.81259	7.51066
MALSU1	Q96EH3	6.03067	6.07754	5.86353	6.2629	6.41003	6.24952	6.42241	6.23764	5.7132	6.40765	5.29299	6.29161	5.95727	6.97388	6.61496	7.19419
ING5	A0A1B0GW41	5.99726	5.92464	6.29614	6.76482	5.9655	5.53216	6.40135	5.67215	6.37765	6.43213	6.32326	6.105	6.20181	5.92479	7.17056	6.91079
MTHFD1L	B7ZM99	6.19612	6.37246	5.8593	7.33227	6.76396	6.40187	6.21997	5.86617	6.09286	6.3883	6.28181	6.2418	6.7093	5.63016	5.83614	5.80751
SVBP	Q8N300	6.27863	6.41529	6.91475	6.00029	6.03321	7.24349	6.48357	6.81704	6.63078	7.62814	6.78287	7.30892	4.87027	5.52807	4.41591	4.64877
DLX6	P56179-3	6.67018	6.71735	6.53004	6.80308	6.13696	6.21632	6.33222	5.9376	6.8359	6.29837	6.0689	6.45521	5.57959	5.34394	5.87301	6.20133
PIP4P1	Q86T03-2	6.25564	6.28876	6.28967	6.41206	6.31555	6.17804	6.45339	6.083	6.65044	6.41653	6.34446	5.64858	6.18151	5.99961	6.41643	6.06633
LSM8	O95777	6.67756	6.49769	5.48954	5.29471	6.82434	6.69084	5.94484	6.33627	5.55814	5.63902	6.34749	6.13908	6.37145	7.08651	6.58352	6.51901
TMED3	Q9Y3Q3	6.12458	5.9159	6.412	7.28016	6.26922	6.42191	6.67133	5.71919	6.78788	6.09173	6.07946	6.49014	5.59011	5.57203	6.30194	6.27243
TMTC4	Q5T4D3-3	5.86533	6.71613	6.80539	6.91318	5.8175	6.14112	5.92373	5.39395	5.85878	6.50096	6.14011	5.43394	5.75676	5.95409	6.84558	6.93346
CDS1	Q92903	5.82806	6.36587	6.10405	6.9747	5.46314	5.63335	6.14162	5.45598	6.57726	6.7125	6.34142	5.36177	6.66476	6.13522	6.84034	7.39997
SMARCA1	A0A0A0MRP6	5.88057	5.89976	5.59476	6.43141	5.94243	5.43416	6.14061	6.33903	6.97354	6.34555	6.19745	6.1701	6.53058	6.26842	6.83199	7.01964
PML	P29590-13	6.48335	6.28827	6.10461	6.83278	5.77036	5.27097	6.44289	6.21999	6.10146	6.25964	6.66267	6.34776	6.48394	6.04443	6.39778	6.28909
ASB6	Q9NWX5	6.81031	6.56874	7.2023	5.90176	6.26329	7.18629	6.27434	6.85017	7.00232	6.66657	6.6736	6.89523	5.01648	4.95755	4.6089	5.12216
HABP4	Q5JVS0	5.54457	5.90887	6.61965	6.2135	7.32401	7.17538	6.30919	6.5739	6.696	6.65105	7.55036	6.93049	5.81673	5.71056	4.56566	4.41009
PCBP4	P57723	6.24565	6.39562	6.46613	6.18357	6.08939	6.08145	5.69594	6.64637	6.87731	6.39904	5.93183	6.56765	6.09425	6.46223	6.05551	5.80805
NFIA	Q12857-4	5.63637	5.83426	6.34404	7.16776	6.07305	6.08449	5.30433	5.95195	5.81618	6.47939	6.20489	6.22511	6.57276	7.11328	7.14215	6.04998
CST3	P01034	6.93832	6.83096	6.20721	5.3068	7.22426	6.19437	6.53989	7.0971	6.75857	6.35059	6.43526	6.57797	5.46883	5.58281	5.1842	5.30286
STEAP2	Q8NFT2	6.24776	6.20934	6.34024	6.91039	5.55612	5.93419	5.8703	5.90755	6.43692	6.31572	5.66697	6.11849	6.39432	6.28572	6.98545	6.82052
TBC1D7	Q9P0N9	6.42904	6.47678	7.25954	6.44648	6.18694	7.12232	6.33369	6.72881	6.70836	6.9461	6.7139	6.88564	4.78799	4.62264	5.30333	5.04844
ZNF93	P35789	6.46037	6.03215	6.7618	6.7276	5.78589	5.91656	6.34613	6.44471	6.19556	5.89347	6.46538	5.93492	6.45739	6.2452	5.71335	6.61953
DAB2	P98082	6.35651	5.67383	6.86252	5.97568	6.15811	5.70635	5.81629	6.3624	6.84885	5.92154	6.96538	6.85853	6.26232	6.22562	5.78858	6.2175
N4BP2L2	Q92802-2	7.33971	6.51016	6.74628	7.48169	7.33776	6.44773	6.85944	6.4276	6.94937	6.96956	6.47903	7.10826	4.38794	4.61211	3.76571	4.57764
RRAGC	Q9HB90	7.21275	7.26208	6.47578	6.40952	5.9995	6.93579	6.2568	5.71777	6.73105	7.29572	5.90838	5.69329	5.30432	5.62927	5.79982	5.36817
ETV3	P41162	6.11132	7.31807	7.48312	6.64743	6.02562	6.40391	5.53364	5.24982	5.79692	6.26968	6.41508	6.60369	5.94375	5.4021	6.57322	6.22263
SGK3	Q96BR1	6.5712	6.47529	6.7102	6.30876	6.27691	6.7608	6.2949	6.70824	6.76881	6.66427	6.51958	6.72022	5.53446	5.63509	5.11073	4.94054
RAB3D	O95716	6.2362	6.38589	5.97472	6.6276	5.83741	5.99092	6.30776	6.26806	6.31759	6.04669	6.2198	6.317	6.40856	6.31055	6.37118	6.38007
RAB8B	Q92930	6.10571	6.10919	6.2779	6.55548	5.75055	5.9977	5.83965	6.25882	6.36031	6.22176	6.28284	6.30541	6.50353	6.46723	6.43989	6.52403
MKS1	Q9NXB0	6.38313	6.46371	6.38017	6.60631	7.38954	6.54108	6.10638	6.04713	6.21111	6.08275	6.04834	6.32249	5.82757	6.00728	6.02919	5.55382
ZNF100	Q8IYN0	6.26191	6.52644	7.77147	6.91416	6.69512	6.96377	7.3155	6.25988	5.55669	6.13044	6.02557	6.07063	5.17348	5.70192	5.46308	5.16995
MOCS3	O95396	6.18659	5.67271	6.33274	6.19681	6.44108	6.42619	6.25738	6.6847	6.31752	6.53093	6.73424	6.27584	5.88686	5.83454	6.15304	6.06882
CDCA7L	Q96GN5	7.28554	6.58165	6.90487	7.66446	6.63374	6.84757	7.01208	6.23301	7.0786	6.42732	6.14586	6.17171	4.90298	4.78225	4.7975	4.53085
ZNF780A	O75290-3	7.15473	6.15022	6.35978	7.34127	5.91627	5.57339	6.79363	6.83136	6.18418	6.58126	6.735	6.60348	5.49808	5.22143	5.82371	5.2322
PFDN1	O60925	5.88841	5.84795	5.71195	5.411	5.07723	4.00537	4.98592	6.12249	4.44751	5.07366	4.43055	5.10171	3.99815	12.3714	8.72443	12.8023
ZNF362	Q5T0B9	6.72093	6.34619	5.37558	6.3101	5.76177	5.32007	6.20041	6.29237	6.02996	6.82132	6.35875	6.99047	6.08332	6.17957	6.85947	6.34973
NFKBIE	O00221	6.53736	4.75353	6.12479	4.889	5.51556	5.33596	6.54509	7.12025	5.64346	6.47715	6.11663	7.61713	6.08974	7.45876	7.31424	7.46134
GATA6	Q92908	5.8688	6.2688	6.26227	6.351	6.36886	6.74287	5.96712	6.47318	6.16217	6.23046	6.19505	5.86241	6.65329	6.18433	6.98562	5.42378
FBRSL1	A0A3B3IRR3	6.37456	5.6569	6.98748	5.66943	6.57544	6.2488	5.92361	6.99271	6.65703	6.19375	6.42196	6.71735	6.14053	5.80425	5.48496	6.15126
HDGFL3	Q9Y3E1	5.35452	6.12046	5.26933	5.74602	6.28606	6.27342	5.66889	5.93361	5.97333	6.46792	5.50292	5.99838	6.57348	7.75705	7.39169	7.68293
SMPDL3B	Q92485	6.01459	6.42411	6.41042	6.2535	5.95207	5.81869	5.90721	5.8893	6.0024	6.11535	6.06256	6.36756	6.91918	6.83639	6.48876	6.53792
LYSMD2	Q8IV50	6.26333	6.19322	7.24679	5.70338	6.27889	6.14176	6.00352	6.61168	6.26315	6.24076	7.45889	6.84508	6.16348	5.57893	5.78385	5.22329
ANO8	Q9HCE9	6.57174	5.98519	6.30497	6.69133	5.71201	5.32309	6.47823	6.96507	6.34476	6.33249	6.23846	5.40236	5.78832	5.95918	6.98155	6.92525
SEC11C	Q9BY50	6.7483	6.18503	6.02228	6.15662	6.79312	5.8009	6.74573	5.86592	6.30637	5.77907	6.72373	6.37204	6.776	5.70932	5.77721	6.23837
DERPC	P0CG12	4.79127	3.99871	5.24149	3.92783	6.1742	5.5777	5.12913	7.34847	4.76756	5.95078	5.67528	6.77726	8.30823	9.23749	8.31028	8.78431
TMEM223	A0PJW6	6.25645	6.4006	6.04784	6.73264	6.08854	6.27723	6.19527	6.25262	6.20553	6.47767	5.63338	5.34005	7.04017	6.50772	6.46695	6.07733
DYNC2I1	Q8WVS4	5.90943	6.56872	6.52608	5.31145	6.0564	6.38065	6.49159	6.65744	6.01823	5.90763	6.69406	6.5368	5.51919	6.68741	7.01057	5.72435
MLLT1	Q03111	6.78958	6.8796	6.45997	7.03865	6.51646	5.96591	6.43654	6.1503	5.6421	6.52446	6.96542	6.23814	5.91972	5.89001	5.23489	5.54925
ARHGAP31	Q2M1Z3	5.87385	6.27852	6.85468	6.2472	6.23495	6.48399	6.35525	6.92695	6.04312	6.30784	6.52423	6.88361	6.16712	6.25161	5.42138	5.1457
C1orf122	Q6ZSJ8	5.95204	5.65856	6.68986	6.11926	6.20907	6.25843	5.89582	6.07937	6.25091	6.44586	6.79856	6.90834	5.60006	6.54899	5.91554	6.66935
H2AC11	P0C0S8	4.70122	8.32503	7.95775	8.97304	5.15871	6.95432	4.40059	6.54327	5.54683	5.35302	3.72366	5.00475	6.18909	6.53213	8.27005	6.36654
IL1RAP	Q9NPH3-5	6.57786	6.50709	5.9188	6.3122	5.53764	5.50167	6.42992	6.3955	6.71653	5.9178	5.53143	6.31561	6.45832	6.59792	7.16427	6.11744
HOMER1	Q86YM7	6.39185	6.25756	6.68018	5.94967	6.44147	6.25413	5.81942	6.68822	6.0327	6.34403	6.85966	6.64317	5.7352	6.14866	5.71106	6.04302
POLR2M	P0CAP2	6.42144	6.37607	6.76959	5.76639	6.87718	6.7747	5.60166	6.25263	6.5364	6.47572	7.10538	6.35864	6.06682	5.64707	5.56045	5.40987
GATC	O43716	5.31673	4.85895	4.7141	4.96051	5.30524	4.27617	5.83789	6.76306	4.275	5.08303	4.43265	5.71218	5.59588	11.6134	8.74818	12.5071
NOTCH1	P46531	7.10656	7.65429	5.60818	6.38867	5.40629	6.63846	6.99688	5.04539	6.63698	5.81543	6.59304	6.65919	5.51071	6.31113	5.10705	6.52174
CAPN15	O75808	5.93895	5.90427	6.9489	6.38599	6.17438	5.768	6.8098	6.43833	6.53167	6.9576	6.58788	6.79976	5.98137	5.31959	5.64211	5.81141
DERL2	Q9GZP9	6.61888	6.40406	6.01159	6.54849	6.18969	6.00293	6.00462	5.83849	5.43365	6.53414	6.2852	6.30477	6.11205	6.5967	6.63666	6.47808
DTX2	Q86UW9	5.6832	6.04179	6.47209	6.13448	5.237	6.65984	6.32054	5.81014	6.91211	6.41081	6.65496	5.9591	6.72023	6.08754	6.27811	6.61804
SLC25A23	Q9BV35	6.29707	6.36975	6.38226	6.76624	5.69373	5.58562	6.95822	5.43026	6.32416	5.94573	5.9201	6.3181	7.22289	5.973	6.0833	6.72958
YTHDF1	Q9BYJ9	6.64775	6.723	6.90415	6.11184	6.20253	6.23668	6.28025	6.394	6.11343	6.53079	6.65842	6.17689	5.63048	5.8011	6.00472	5.58397
ADCK5	Q3MIX3	6.61653	6.61737	5.48745	6.49882	6.16853	5.77854	6.25211	5.97243	6.30269	6.34395	6.12721	5.28292	7.01811	6.63967	6.10154	6.79211
CNOT4	O95628-9	6.50709	6.48673	6.62692	5.94144	6.133	6.21941	5.82412	6.52246	6.26683	6.39106	6.50315	6.80235	5.38999	5.76677	6.15607	6.4626
ICA1	E7ENI6	6.37331	5.94091	5.75817	6.43829	6.13488	5.72538	6.71057	5.33909	6.2667	6.03624	5.7988	6.12695	6.98313	6.50592	6.74046	7.12121
WDTC1	Q8N5D0	5.73961	6.12848	6.53031	5.8322	6.22057	6.66989	5.86641	6.41989	6.32031	6.77808	6.45054	6.02793	6.70928	6.18255	6.22635	5.8976
ALS2	Q96Q42	6.13077	6.65674	6.57174	6.05148	6.37938	6.57658	5.94869	6.72191	6.49354	6.22312	6.56388	6.15529	5.58376	6.34761	5.92216	5.67334
SMAD5	Q99717	5.97331	6.11058	6.74021	6.06204	6.12993	6.74774	6.43308	6.32631	6.35782	6.1271	7.0423	6.3607	6.44473	5.66419	6.00713	5.47282
FAR2	Q96K12	6.91018	6.89998	6.91617	7.68881	6.18599	5.78025	6.25324	5.94887	7.21894	7.12107	7.50073	7.62001	4.10892	4.48912	4.68723	4.67049
DNMT3B	Q9UBC3	6.73657	6.69591	6.36581	7.93826	6.4736	6.35359	6.40569	5.74349	6.50305	6.69042	6.18069	6.61709	5.96892	5.56238	4.99377	4.77077
SLC25A36	Q96CQ1	6.58221	6.45808	5.56716	6.56845	6.13293	6.48528	6.61474	5.84799	5.3499	5.95626	5.97034	5.83069	6.8869	6.18941	7.05496	6.5047
TTPAL	Q9BTX7	6.40605	6.31135	7.04002	6.0339	6.39902	6.73016	6.15309	6.48672	6.60431	6.61436	6.47696	6.82084	5.85498	5.3905	5.26762	5.41014
RUNDC1	Q96C34	6.54991	8.33132	7.56469	5.29227	6.76831	5.97395	6.87662	7.94943	6.77023	6.88071	6.68904	6.81742	4.98094	3.73161	3.89407	4.92948
SERBP1	Q8NC51-3	5.44778	5.82462	5.67079	5.45905	6.43614	6.23606	5.39613	6.57063	6.88775	6.64241	6.2817	7.40128	6.1007	7.306	6.23569	6.04298
MPV17	P39210	6.57265	6.31599	5.73109	5.25297	6.04147	6.12131	5.86205	6.04644	5.44079	6.2942	5.6379	6.37784	6.49025	7.03067	7.3197	7.46468
SLC7A5	Q01650	6.35113	6.46311	5.34946	5.92485	6.77234	6.13448	6.78412	6.25963	5.9773	5.784	5.3663	5.90965	6.67644	7.05336	6.76096	6.43287
FAHD2A	Q96GK7	5.91042	5.46344	5.76976	5.94678	6.35148	6.37514	6.08094	6.47126	6.39966	6.67083	6.70849	6.98336	6.52348	5.88304	6.29285	6.16905
PNKD	Q8N490	5.73552	5.84704	6.38002	6.49755	6.21553	6.08019	5.95934	5.90395	5.99203	6.90992	6.30042	5.93781	7.15201	6.27023	6.36636	6.45208
SIAE	Q9HAT2	7.37115	6.03926	5.32842	6.76603	5.84451	6.10014	6.31352	5.60828	6.71165	6.59903	5.55115	5.577	6.89886	5.9044	6.49249	6.89411
TWSG1	Q9GZX9	7.45272	6.89956	5.85067	6.49074	7.03599	5.99548	6.20283	6.78594	5.86865	6.10649	6.56927	6.22121	6.30172	5.4456	5.34512	5.42802
VKORC1L1	Q8N0U8	6.15237	6.05812	5.62319	6.36548	6.06351	6.13142	6.8435	5.68513	6.15064	5.69762	5.61823	5.73175	6.8119	6.85031	6.80927	7.40758
MDM1	Q8TC05	6.20237	6.32955	6.14002	6.74448	6.16255	5.88642	6.2202	6.21692	6.21288	6.09018	6.47062	6.56885	5.57244	6.61383	6.61707	5.95162
TRIM37	O94972	6.48732	6.62949	6.79721	6.35734	6.25624	6.31249	6.80154	6.35283	6.30579	6.7269	6.60073	6.57933	5.53907	5.44413	5.42072	5.38886
STK17A	Q9UEE5	6.39793	6.17561	6.23419	5.99199	6.41987	7.32926	5.2055	6.42877	6.35189	6.89514	7.56874	7.49305	5.18936	5.91123	4.93942	5.46806
NDUFA2	O43678	6.00576	5.18032	5.74011	5.22457	6.71663	5.20257	5.92495	6.33739	5.92576	6.67262	6.60251	6.40689	6.26578	6.96739	7.86927	6.95748
IFIT2	P09913	7.37902	5.81906	6.70985	5.99234	7.62133	6.69554	6.08339	6.13604	6.01612	5.81951	7.37485	5.93297	5.72887	6.01174	5.46579	5.21359
GTPBP8	Q8N3Z3	6.26103	6.43007	6.07534	6.53138	7.01063	6.09407	6.72428	6.01348	6.20659	6.24413	6.39244	6.66378	5.93779	5.02865	6.50983	5.8765
RPGRIP1L	Q68CZ1	6.97618	6.39964	7.2206	6.77616	5.96136	6.64062	7.17168	6.66136	6.58485	6.81346	7.21542	6.85301	4.8411	4.52309	4.92918	4.43801
SLC38A9	Q8NBW4	6.70676	6.25851	6.50671	6.5515	6.1751	6.29601	6.0502	5.93925	5.89155	6.45219	6.32532	6.53925	6.34202	5.98007	6.13232	5.85324
PLCL2	Q9UPR0	6.08687	6.43939	6.40873	5.93585	6.2124	6.33573	6.3297	6.4813	6.52213	6.06012	6.36517	6.53851	5.74495	6.03625	6.49571	6.00719
MAGEA6	P43360	6.61296	6.74437	7.39146	6.23209	6.18103	6.5173	6.95239	7.1332	6.99221	6.85838	7.32836	6.90737	4.20129	4.53674	4.30786	5.10298
NACA4P	Q9BZK3	6.81216	6.68107	6.9798	5.82649	6.74593	6.24039	6.7103	6.27191	7.22975	6.653	7.04038	6.42303	5.41426	5.00627	5.01564	4.94963
SLC39A1	Q9NY26	6.52774	7.3411	6.29885	8.02934	6.52298	6.5806	6.32194	6.08344	6.80484	6.53224	5.87739	6.3076	5.4612	5.3015	5.30741	4.70181
ETV6	P41212	6.8156	6.44802	5.90167	6.86873	6.86199	5.73233	6.30408	7.06582	6.2165	6.08006	6.37985	6.39633	5.80818	5.52082	6.19387	5.40615
MUTYH	Q9UIF7	5.56602	5.9912	6.60564	6.24768	6.23978	6.19441	5.59512	5.90956	6.63426	6.43277	6.596	6.77666	6.0876	6.49116	6.33961	6.29253
APEX2	Q9UBZ4	6.09874	6.3004	7.20214	6.45312	5.57197	6.19984	6.2872	6.57921	7.09964	7.10056	7.56658	7.47448	4.66813	5.0338	5.10858	5.25561
TTC31	Q49AM3	6.30748	6.42626	6.70695	6.64687	6.97657	6.58278	6.65107	6.05545	6.93844	7.55958	6.88355	6.67923	5.3161	4.74653	4.97426	4.54886
ZDHHC20	Q5W0Z9	7.04138	6.44817	5.87902	7.09944	6.99283	5.58858	5.83884	5.69732	6.75077	6.65455	6.50861	6.66098	5.13536	5.6885	6.53124	5.4844
CCDC130	P13994	5.48587	6.59962	6.06721	5.85377	5.80153	6.64095	5.81837	6.21877	6.62057	6.4754	6.20653	6.84739	6.41207	6.09497	5.93094	6.92605
ENOSF1	Q7L5Y1-5	6.52191	6.38104	7.03293	5.52245	6.21555	7.04575	6.0402	6.70055	6.48026	6.54413	6.68769	6.72765	5.45421	5.48689	5.54451	5.61427
TMCO3	Q6UWJ1	6.84166	6.42441	6.20316	7.49625	6.07492	5.59353	6.52153	6.28214	6.32198	6.08805	5.94632	6.35978	6.17371	5.80458	5.98064	5.88733
ASNSD1	Q9NWL6	6.3091	6.46196	7.50966	6.29994	6.40045	6.15135	6.36566	6.35699	7.05418	6.97497	6.82725	7.26954	4.76357	4.76985	5.15675	5.32878
ELOVL1	Q9BW60	5.81296	6.78492	5.75004	6.50915	5.46156	6.12257	6.68235	6.59904	5.93445	5.73397	6.42948	6.11527	6.21364	6.31302	7.03299	6.50458
TRADD	Q15628	6.60986	6.46901	7.04925	6.22856	6.41998	6.42819	5.75597	6.4482	6.02975	6.93479	6.05198	6.33173	6.05816	6.08061	5.11964	5.98434
SLC4A8	Q2Y0W8	6.02272	7.0767	6.16777	6.98897	5.9787	6.04322	6.35392	6.16438	6.35971	6.72631	6.73422	6.38317	5.59223	6.08809	5.79627	5.52362

SMPD1	P17405	6.59275	6.58443	6.35757	6.26937	6.23819	5.97923	6.12864	6.01106	6.16369	6.42566	6.18433	6.76548	5.78756	5.95649	6.27572	6.27983
TBKBP1	A7MCY6	6.70096	6.58825	6.65699	6.85133	6.75703	7.51064	5.80289	8.13024	6.37139	5.51008	6.67083	6.12505	5.43848	4.60599	5.6013	4.67857
TCEANC2	Q96MN5	6.1075	6.86319	6.22478	5.19264	6.17205	6.40683	6.16554	5.73431	5.88307	7.32433	6.85559	6.23387	6.0886	6.15841	6.12886	6.46043
PFN2	P35080	6.33558	6.12999	6.96676	5.64572	6.48584	6.96729	6.03687	6.7257	6.70938	6.49995	6.46698	6.66332	5.70198	5.55776	5.53853	5.56833
ENY2	Q9NPA8	6.31278	5.18974	5.74793	5.48464	6.59968	5.14357	4.98763	6.50661	4.93804	6.62516	5.65994	6.53429	6.03971	8.56147	7.63224	8.03656
GNAI1	P63096	6.28619	6.82267	5.64294	6.61294	6.73522	6.51291	6.05842	6.16208	5.38189	6.60206	5.72054	5.93119	5.91363	6.62399	6.34781	6.64553
OTUD7A	Q8TE49-2	5.30049	4.15294	4.46822	5.04991	4.81249	5.27453	4.7562	5.57585	5.45955	7.51869	6.87959	8.17387	7.88183	8.93895	8.20322	7.55368
SDF2	Q99470	5.91064	6.04729	6.06392	6.20216	5.80411	6.68272	6.06721	6.01105	5.93695	6.82567	7.02379	6.29199	6.68134	6.00197	6.3687	6.08049
TOR2A	Q5JU69	6.13568	6.63858	6.12364	6.35303	6.26844	5.55679	6.16462	6.1627	6.19728	6.08458	5.85647	6.14615	6.40065	6.51215	6.83951	6.55973
INPP1	P49441	5.91362	5.93506	6.46179	6.15007	6.64968	6.98913	5.81003	7.00445	6.56064	6.54575	6.45582	6.09784	5.59893	5.61373	6.20667	6.00682
VTI1B	Q9UEU0	6.74921	6.96995	6.61842	6.52684	6.06957	6.22428	6.55023	6.04481	6.12345	6.65641	6.5757	6.3575	5.49816	5.49259	5.70335	5.83953
TEAD3	Q99594	6.41999	6.36324	6.65257	7.50047	6.97435	5.19909	5.69446	6.04351	6.54821	6.60573	6.44043	6.43583	4.89854	5.64472	6.26815	6.31071
TEAD1	P28347	6.36677	6.34693	5.92296	6.64584	6.82839	6.02763	6.27478	5.84157	5.87929	5.8301	6.19897	5.94314	6.54611	5.91642	6.96168	6.4694
WWTR1	Q9GZV5	6.81952	6.43326	6.5798	5.23388	7.01276	4.83019	5.47589	7.36935	4.77478	5.78095	5.97812	7.10001	6.20938	7.66902	7.37836	7.15472
APBA2	Q99767	6.73538	7.40102	7.05936	7.34011	6.29519	6.65222	6.14833	6.77656	6.30535	6.61402	6.49978	6.38168	4.76638	5.51059	4.5587	4.95531
PITPNM2	Q9BZ72	6.89625	6.38812	7.1194	6.0399	5.28411	6.61152	6.30698	4.78227	5.7935	6.32665	5.99386	6.40592	5.81976	6.80286	6.15077	7.27813
CETN2	P41208	6.37001	6.30188	6.44866	5.64507	6.58854	6.26416	5.99748	6.77904	6.13401	6.58581	6.31693	6.3712	6.24146	6.26706	5.97454	5.71415
C2orf76	Q3KRA6	6.11627	5.8917	6.55879	5.23805	6.32572	6.86225	6.45134	7.03556	6.74513	6.95765	7.06996	6.63877	5.69238	5.54778	5.38871	5.47993
MEX3D	Q86XN8-3	6.70755	6.64115	7.68119	6.48235	6.69218	6.43565	5.87031	6.92676	6.3376	6.62787	6.78335	7.22967	5.27241	4.94029	4.6624	4.70929
FBXO6	Q9NRD1	5.96641	6.07145	6.82053	6.51963	6.2512	6.57853	5.93952	6.66578	6.16051	6.12841	6.44155	6.63481	6.58177	6.1222	5.79583	6.22187
ARMCX1	Q9P291	7.02896	7.30835	7.13631	7.10959	6.66533	6.54118	6.83184	6.6403	6.86992	7.7902	7.05883	7.65735	3.50057	4.07816	3.86696	3.91615
APBA3	O96018	6.33638	6.01974	6.1675	6.07777	6.44929	6.84088	6.46858	5.98302	6.42982	6.45742	7.50033	6.34607	5.61818	5.8478	5.72776	5.72945
DNAJC5	Q9H3Z4	6.36942	6.40312	6.02538	6.43036	5.80857	5.84841	6.23	6.1298	6.41206	6.2731	6.2107	6.69874	6.09203	6.20864	6.42692	6.43277
POLR2M	Q6EEV4	6.36568	4.71338	5.39578	4.79779	5.23119	4.53274	4.78752	7.0741	3.9272	6.3935	5.58148	5.88577	4.65375	12.6309	7.38792	10.6413
PXMP4	Q9Y6I8	6.08019	6.20597	5.4735	6.39694	6.08499	5.43715	6.2488	6.581	6.08996	6.55305	6.2039	6.5164	6.72601	6.54685	6.53609	6.3192
EPB41L2	O43491-4	6.42915	7.56329	5.6491	7.11333	6.57694	6.85756	5.9759	7.24597	5.66069	5.36861	6.32112	6.07134	5.24552	6.50652	6.16937	5.2456
ZMAT5	Q9UDW3	5.61595	5.96397	6.51383	6.78292	6.16068	5.79639	6.48306	5.59823	6.60182	6.24475	6.9859	6.75649	5.91821	5.6753	6.22177	6.34234
CEBPB	P17676	5.50883	6.43642	6.10198	7.06588	6.31785	6.08024	6.49917	6.44994	5.45169	6.43549	5.944	6.09937	6.09877	6.18684	6.83861	6.48491
HNRNPU	Q00839-2	5.13308	5.68084	5.52857	6.76496	5.62028	5.89392	6.07917	6.38493	5.64087	5.96062	5.96681	7.09486	6.56042	6.68499	7.02535	7.98035
PHC3	Q8NDX5-7	6.11805	6.25715	5.95332	6.53623	6.18393	5.98476	6.41975	5.8712	6.36532	6.47643	6.43792	6.36558	6.4297	6.09992	6.29888	6.20188
MAPKBP1	O60336	5.83122	6.17342	6.93143	5.92125	6.13499	6.82227	6.51694	5.73364	6.74974	6.71975	7.07988	6.92988	5.92392	5.75011	5.06633	5.71524
MPP1	Q00013	6.50998	6.29053	6.51053	6.35052	6.20964	6.10408	6.56273	6.3654	6.15847	6.51781	6.48426	6.81494	5.62999	5.74569	5.87634	5.86907
SH3BP5L	Q7L8J4	6.54399	6.49517	6.52477	5.80456	6.93494	6.67712	6.07765	6.92733	6.31598	6.22451	6.57983	6.29955	6.41469	5.87859	5.56199	4.73933
TIMM8B	Q9Y5J9	6.20742	5.36778	4.44549	5.40993	5.85659	4.86415	5.64465	6.7611	4.9757	5.27375	4.74861	5.40552	5.40645	10.8221	8.08496	10.7258
CDK11A	Q9UQ88	6.06216	6.5464	5.74839	6.4502	6.01625	5.82229	5.98199	5.91837	6.29234	6.19075	6.10113	5.8924	6.64841	6.84061	6.85142	6.6369
SAP30L	Q9HAJ7	6.79992	6.87646	5.81654	6.70553	6.08652	6.14342	6.22165	6.05341	6.00143	6.16335	6.17385	6.19545	6.46805	6.19952	6.20767	5.88723
TMEM260	Q9NX78	6.05366	6.50526	6.41588	6.75744	5.82661	5.77565	6.08912	6.32264	6.30907	6.01753	5.68779	5.68544	6.43429	6.17636	7.25908	6.68417
CCNY	Q8ND76	5.79712	5.82624	5.94926	6.56764	6.05784	6.36089	6.58215	6.23607	5.65494	6.40687	6.63912	6.41982	6.25102	6.48973	6.1106	6.65068
ARHGAP23	Q9P227	6.42873	6.29561	5.85485	6.45713	6.49818	6.38417	6.33857	6.40948	6.45573	5.8594	6.12012	6.33366	5.93649	6.42281	5.96592	6.23914
FPGS	Q05932	6.3008	5.83042	5.85748	6.52852	7.07188	6.63608	6.95735	6.75833	6.35205	5.78677	6.12558	6.40634	5.95529	5.41423	5.73801	6.28087
RNASEL	Q05823	6.21841	6.05125	6.28961	6.45034	6.371	6.37029	6.28951	6.33826	6.23766	6.16957	6.61174	6.30144	6.14043	6.28956	6.03819	5.83273
MAPKAPK3	Q16644	6.251	6.30319	6.71311	6.15043	6.55482	6.48448	6.21858	6.63059	6.90567	6.5922	6.47435	6.27919	5.69879	5.76022	5.51109	5.4723
IFT43	Q96FT9	6.61222	5.56722	6.42985	5.63946	6.57736	6.55343	6.53934	6.56071	6.64383	6.67591	6.72768	6.79767	6.53095	5.52357	5.19188	5.42895
RFX3	P48380	6.36921	6.45562	6.05779	5.92022	5.81799	5.80853	6.92984	6.6052	6.434	6.17999	5.8261	6.15804	6.43276	6.33412	6.08312	6.58745
ABHD15	Q6UXT9	6.41509	6.2656	6.22916	6.17401	6.34067	6.2058	5.98949	6.46165	6.37125	6.35949	6.1084	6.40324	5.90672	6.26165	6.31888	6.1889
GPSM2	P81274	6.07058	6.37084	6.89814	6.47628	5.95636	6.71381	6.53549	6.66856	6.62903	7.25101	7.41526	7.55336	4.33507	5.22962	4.68571	5.21085
TMEM109	Q9BVC6	6.04927	6.43839	5.77481	6.63099	6.06657	5.95026	6.21161	6.16038	5.87568	6.07612	5.74419	5.98355	6.57411	6.72826	6.77371	6.96211
RAB27B	O00194	6.44288	6.61852	6.05012	6.44536	6.57454	6.37823	6.29818	5.99961	6.05368	6.14695	6.02883	5.70069	6.43215	6.55629	6.35241	5.92155
SBNO2	Q9Y2G9	5.79451	5.90242	6.70503	5.6961	5.58983	6.43425	6.12914	5.94109	6.71154	6.62886	6.87317	6.61357	5.98358	6.10371	6.68979	6.2034
RAD17	O75943	5.62777	5.99073	5.79868	5.40835	5.94417	6.02421	6.01165	6.40188	6.27657	6.32666	6.38441	6.15841	6.94442	6.82667	7.40986	6.46557
CARHSP1	Q9Y2V2	6.77575	6.04577	6.71207	5.3892	6.99784	6.74559	5.72551	6.69491	5.99695	6.75925	6.99598	6.7396	6.0692	5.88049	5.47732	4.99455
MAP3K1	Q13233	6.98341	6.2605	6.17348	6.48396	6.26102	6.33377	6.14519	6.42178	6.63475	6.88274	6.71731	6.47681	5.99528	5.63836	4.9284	5.29724
TAF8	Q7Z7C8-2	6.07371	5.94515	6.41161	6.48222	6.25283	6.00731	6.37956	5.85707	6.42479	6.29868	6.47506	6.65716	6.81775	6.07909	5.9121	5.9259
SLC22A18	Q96BI1	5.90607	5.93526	5.83145	6.12717	6.39875	6.09708	6.10521	5.88565	6.37076	6.10339	5.74806	5.65419	7.01705	6.55547	7.38401	6.88043
LRP11	Q86VZ4	7.66838	7.5303	7.04349	7.46342	6.70646	6.00004	6.68786	6.04141	6.89802	7.3006	7.54972	7.16214	3.92558	4.3256	3.64969	4.04729
MYC	P01106-2	4.37851	5.06141	4.92982	6.16136	5.2986	5.51219	6.04151	5.8606	4.46191	5.4076	5.02671	5.52099	9.33064	7.50289	10.1854	9.31987
APLF	Q8IW19	6.0517	6.01297	5.93063	5.79051	6.63276	6.12549	5.44062	6.48641	5.517	5.48615	6.50957	5.76321	7.06921	7.69417	6.16607	7.32353
CHRAC1	Q9NRG0	6.35139	6.41895	6.56236	6.1357	6.17323	6.5264	5.71654	6.2572	5.88291	6.68482	6.92144	6.80121	6.29421	6.31917	5.61828	5.33619
EDEM2	Q9BV94	6.67362	6.83071	6.18048	6.76232	6.63862	6.52469	6.37051	6.42292	6.1036	6.37783	6.1669	5.83861	5.9946	5.60529	5.72876	5.78053
BAG1	Q99933	6.30953	6.50706	6.15805	5.91703	6.3219	6.76689	5.86398	6.76652	6.09045	6.59659	5.89963	5.9918	5.99593	6.62067	5.59407	6.59991
CORO2A	Q92828	6.23927	6.56027	6.45651	5.93611	6.47771	6.37664	6.4075	6.42033	6.53465	6.45041	5.90007	5.79794	6.28257	6.02604	6.02011	6.11387
DAB2IP	Q5VWQ8	6.73126	6.36443	6.78828	6.61361	6.11256	6.04645	6.21295	5.83449	6.06135	6.44473	6.21779	6.11276	6.34172	6.23354	6.22526	5.65881
ZDHHC17	Q8IUH5	6.60195	6.25178	5.79759	6.82414	6.29415	6.26133	6.65817	5.86257	6.25148	5.80915	6.1276	5.8917	6.90432	6.0088	6.72216	5.73312
DIPK2A	Q8NDZ4	6.79272	6.77927	6.11779	6.93151	5.90066	5.85746	6.54823	5.69075	6.32023	6.18783	6.13494	6.41038	6.03735	5.47538	6.48099	6.33451
BTBD8	A0A1B0GUL6	6.19285	5.73832	6.46829	6.19432	6.59024	6.96119	6.10056	6.00108	6.91593	6.80461	6.03649	6.64646	5.37528	6.46035	6.20897	5.30508
NUCKS1	Q9H1E3	5.55239	5.58385	5.59702	5.68883	5.72979	5.94635	5.38639	6.45312	5.47622	6.28578	5.33324	5.55038	7.01125	8.77847	7.98322	7.64369
CUEDC2	Q9H467	6.07929	5.83315	6.91869	5.41747	6.68616	6.30882	6.24711	6.60979	6.29333	6.47523	6.84312	7.01329	5.95069	5.91068	5.62161	5.79158
CHRNB1	P11230	8.68878	8.83069	7.96913	8.48416	6.30665	6.34694	6.2172	6.71969	7.20076	7.20399	7.21396	8.19195	2.42824	2.42122	2.53689	3.23975
ABHD17A	Q96GS6-2	6.32334	6.82184	6.07311	6.46381	5.80613	6.21074	5.93398	6.29629	5.94904	6.33985	6.38555	6.70573	5.86379	6.32823	6.06595	6.43263
BLOC1S6	Q9UL45	6.14583	5.94326	6.67971	5.18878	6.40946	6.44924	5.83025	6.67142	5.98864	6.62266	6.72882	6.76602	6.58987	6.10768	5.83668	6.04168
ZFP36L1	Q07352	4.33688	4.28409	4.8717	4.34261	5.81616	6.0078	5.46032	5.64493	4.59376	4.7636	5.5977	4.72774	11.7657	9.01805	9.63273	9.13621
ZFP36L2	P47974	6.70582	6.29072	6.90331	5.37146	5.78721	6.14075	6.23457	7.88786	7.25647	6.19831	5.24185	6.27009	7.11906	5.90876	5.86546	4.81829
DPEP3	Q9H4B8	6.3433	6.32109	6.18716	6.57853	6.21895	5.9782	6.37745	5.8781	6.17331	7.00687	6.55599	6.37686	6.26958	6.06395	6.06198	5.60867
NACC2	Q96BF6	6.65049	5.85304	6.19427	5.84649	5.94316	6.2482	5.93171	5.71481	6.16979	7.0927	7.10114	6.41982	6.24929	6.30631	6.03718	6.24161
NLRX1	Q86UT6	6.69037	6.53045	6.08492	5.91412	6.53267	6.229	6.13914	5.60062	5.66079	6.70535	6.16578	5.74635	6.21496	6.649	6.44131	6.69518
YBEY	P58557	5.97241	5.65956	5.14304	5.86806	6.26317	5.53842	6.45408	6.21011	6.58964	5.65232	5.80516	6.23195	7.26212	6.97764	7.00635	7.36599
ATF6B	Q99941	6.54175	7.72616	6.92741	6.90558	6.26374	7.15949	7.58948	6.78528	6.17682	7.00422	6.77629	6.98125	4.05266	4.71174	4.1357	4.26242
GINS4	Q9BRT9	6.00536	5.62109	6.20063	5.65083	5.72773	5.8318	5.73679	7.58389	6.05666	5.9866	6.64068	6.41347	6.49992	6.60842	6.46588	6.97027
PLEKHA1	Q9HB21	5.99613	6.08246	6.89392	6.06001	5.88639	6.36148	6.36889	6.60613	6.26966	6.27599	6.70228	6.90003	5.84275	6.24146	5.9255	5.58693
PDSS1	Q5T2R2	6.56621	6.04035	6.12451	6.56189	7.14206	5.96351	5.90536	7.07948	6.13391	5.90535	6.80539	6.13381	5.90903	5.69689	6.27977	5.7525
SH3RF1	Q7Z6J0	6.62626	6.4613	6.4423	6.57465	6.20006	6.62589	5.88909	6.62442	5.8549	6.51896	6.92111	6.51306	5.7777	5.79244	5.35934	5.81852
ZNF451	Q9Y4E5-4	6.34415	6.48598	7.28857	6.38163	5.68849	6.5258	6.63605	6.35818	6.72537	6.53017	6.68945	6.84155	5.19262	5.68499	5.62689	5.00009
GLTP	Q9NZD2	6.84763	5.66959	7.58256	5.64975	6.1031	5.82007	6.34126	6.79728	6.03514	6.5828	6.58865	6.44315	5.28469	6.11589	5.85344	6.28499
FGD1	P98174	6.23001	6.75074	6.67489	5.91415	5.88727	6.87497	6.36326	7.15982	6.32995	6.67387	6.2987	6.40489	5.62878	5.78445	5.51314	5.51112
CRACD	Q6ZU35	6.26553	6.83961	6.35392	6.1778	5.82045	6.1997	6.54618	5.7483	6.30771	6.27062	5.88973	6.24415	6.54394	6.43902	5.90525	6.44808
PSTPIP2	Q9H939	6.35397	5.73675	6.43996	5.98269	6.25816	6.3123	5.9835	6.67517	5.92908	5.24909	6.50844	6.96638	5.95401	6.96368	6.0954	6.59142
DVL1	O14640	6.4334	6.27374	6.13888	6.55468	6.21837	5.88042	5.69925	6.05501	6.47642	6.69299	6.92291	7.36551	5.59416	6.36296	5.6306	5.70071
PPP1R11	O60927	6.23212	6.41437	6.88433	5.87389	5.82305	6.44689	5.42779	7.16921	7.01035	6.81942	7.51994	7.04548	5.91626	5.09082	4.79697	5.52911
MEGF9	Q9H1U4	7.07082	7.08464	5.99864	6.48088	6.7547	6.71	5.7729	5.33269	6.29671	6.82629	6.2972	6.12942	6.19099	6.16472	5.52862	5.36079
METTL2B	Q6P1Q9	6.39009	6.24929	6.86479	5.84486	6.49368	6.8251	6.57349	7.00649	6.29824	6.49314	6.34902	6.68755	5.40038	5.56607	5.26666	5.69117
FBXL3	Q9UKT7	5.71155	6.43106	6.75392	5.97603	5.90647	6.12216	5.3492	5.97588	6.40548	6.59245	6.50517	6.17177	5.64875	6.80578	6.91355	6.73078
RABGAP1L	Q5R372-5	6.40827	6.01632	6.87539	6.117	6.04829	6.37808	6.4178	6.1958	6.72893	6.27629	6.59713	6.64288	6.25577	5.73405	5.76112	5.54687
RIN3	Q8TB24	5.72823	5.61804	6.06851	5.64851	5.63423	5.98419	5.91351	6.28581	6.80451	6.77702	6.56917	7.56503	6.61088	6.70785	6.54499	5.53953
CAV2	P51636	6.34588	6.11077	6.69046	6.67201	6.53739	6.06505	6.38312	5.44678	6.49951	6.1451	6.16895	6.06134	6.18052	5.55079	6.45808	6.68424
ANTKMT	Q9BQD7	6.41399	5.99328	7.17766	5.93092	7.32417	6.40371	6.40107	5.72874	7.11936	6.35168	6.60062	6.69912	5.87664	5.18619	5.50624	5.2866
ATXN7L3B	Q96GX2	5.4476	6.01585	5.96895	4.80512	7.40863	6.75379	6.16462	6.74588	5.55182	6.90912	6.68797	6.15515	5.96982	7.00652	6.04649	6.36267
CASP3	P42574	6.29678	7.8369	7.16222	6.25374	6.95984	6.95926	5.49206	6.35644	6.08996	6.62891	6.38325	6.21576	5.30891	5.10174	5.58726	5.36697
NADSYN1	Q6IA69	7.34886	6.57508	6.81322	6.78156	6.81981	6.62436	5.5795	6.09775	6.54745	6.59097	6.02892	5.39668	6.69648	6.39988	5.09561	4.60389
TMEM147	Q9BVK8	6.38313	5.7954	6.9686	6.68615	6.73547	6.01508	6.2286	5.78736	6.26566	6.00363	5.85616	5.72594	6.31644	5.72884	6.95109	6.55245
TMEM120A	Q9BXJ8	6.00297	6.03128	6.14656	6.71207	6.17809	5.76384	6.02921	5.72235	6.60588	6.38054	5.98094	5.92216	6.68939	6.26076	7.00856	6.56538
COA6	Q5JTJ3-2	6.42408	5.51503	5.92284	5.83613	6.41683	4.80412	6.12084	6.435	5.44857	5.86708	5.33189	6.00276	7.39612	7.49696	7.28778	7.69398
LTO1	Q8WV07	6.79562	6.0391	7.2991	6.06452	6.54463	6.52068	5.66968	6.72298	6.88	6.92561	7.10445	7.35006	4.92128	5.21762	4.70252	5.24214
ID1	P41134	5.00431	4.95152	5.81929	4.10038	5.59974	5.27774	5.24824	6.49936	5.21914	5.61097	6.3385	6.45901	6.8702	8.9632	8.04159	9.09681
NR1D1	P20393	5.84569	6.23763	5.60679	5.17815	6.15634	6.63236	5.8825	5.90034	6.51816	6.05464	6.3936	5.89804	6.80979	6.65509	7.40213	6.82875
NR1D2	Q14995	5.67294	5.77685	10.8745	5.91068	7.51835	6.6918	10.099	5.33044	6.06059	4.94405	6.09797	5.80442	4.95982	4.45803	5.00995	4.79063

WDR41	Q9HAD4	5.66103	6.47403	6.51388	6.18905	5.52434	6.29488	6.36062	5.86605	6.38656	6.74851	6.79248	7.16988	5.47881	5.45904	6.66728	6.41358
INTS6L	Q5JSJ4	5.14298	6.8953	5.38558	6.95352	5.82967	6.5703	6.26191	5.91077	6.67241	5.75473	5.81362	7.61493	6.52901	5.87807	6.61516	6.17204
PLAUR	Q03405-2	7.24839	7.72203	6.73499	7.69989	6.75172	6.69973	6.7959	6.69188	6.10063	6.349	6.36823	6.43609	4.67588	4.28556	4.52745	4.91263
NAB2	Q15742	5.72145	6.1106	6.20043	5.59484	6.07034	5.81728	5.93111	5.81607	6.17703	6.62006	5.92313	5.9294	6.86091	7.20579	7.22848	6.79306
TMEM192	Q8IY95	6.72286	6.50872	6.46906	5.80647	6.48497	5.57743	6.14265	5.62696	6.49686	5.53025	6.20739	6.4661	6.714	5.81765	6.72871	6.69991
LTBP2	Q14767	6.39795	5.09114	4.76724	7.27965	5.29982	5.38543	6.66727	5.59505	7.05962	5.84517	6.74636	6.06764	8.50873	6.0717	6.8934	6.32384
NT5C3B	Q969T7	6.2001	6.10483	6.9712	5.95836	7.26906	7.2003	6.23453	6.48034	6.26317	6.82797	6.64286	6.36789	5.78518	5.58009	4.85043	5.26369
JAG1	P78504	6.19608	6.81729	6.72488	6.40227	5.96942	5.83502	5.66744	5.24998	6.14514	5.94729	6.61451	6.40578	7.06339	6.31332	6.4129	6.23529
FRMD4A	Q9P2Q2	6.11623	6.25277	6.78152	6.26843	5.9607	6.68265	6.1061	6.02285	6.11555	6.10862	6.62798	6.78942	6.12843	6.07608	5.64632	6.31636
E2F6	O75461	6.52609	6.48155	6.21336	7.03404	6.19502	5.88008	6.47153	5.61223	6.33265	6.16492	6.56007	6.65789	6.36716	5.31785	5.84546	6.34011
NIT1	Q86X76-3	6.02265	5.07978	6.60735	5.89446	6.85991	6.48205	5.6111	6.24932	5.87006	6.9341	7.12206	6.45427	6.1743	6.20043	6.49649	5.94166
FBXL20	Q96IG2	5.61078	6.38545	5.91557	6.63527	6.75616	5.71236	6.18471	5.90532	6.03428	5.84761	6.12511	5.75806	7.08134	6.85766	6.89862	6.2917
TRAF3	Q13114	6.41851	6.11262	6.45978	5.96446	5.94443	6.80246	6.11951	6.63839	5.85421	6.45741	5.86479	6.2629	5.86839	6.87153	6.46179	5.89881
MOB2	Q70IA6-3	6.19559	6.28147	6.97612	5.31225	5.98664	6.82825	6.27174	5.94367	6.16186	6.1181	7.08137	6.27782	6.22399	6.28024	6.08931	5.97159
DGKH	Q86XP1	6.80762	6.14987	6.69376	6.56196	6.42501	6.59258	6.15244	6.79686	6.2234	6.5941	6.3617	6.05941	5.59748	5.60749	5.60936	5.76696
METTL14	Q9HCE5	5.44684	5.20666	6.37483	6.39965	5.93724	5.918	5.8855	5.21224	6.00978	6.10354	6.26043	6.29323	7.24699	6.86593	7.40613	7.43302
RMND5B	Q96G75	6.61379	5.8828	7.42059	6.50902	6.28651	6.11249	6.58133	6.40163	7.09751	6.84903	8.23686	6.33543	4.5374	4.66793	5.42402	5.04367
RSL24D1	Q9UHA3	5.93106	6.43999	6.11176	7.03828	6.50684	5.97555	6.54544	5.71535	6.23151	5.90244	5.74071	6.19753	5.92317	6.25173	6.75394	6.73469
RMDN2	Q96LZ7-2	6.8992	6.72586	6.63982	6.49108	6.45222	6.12272	6.35324	6.54505	6.45356	6.5984	5.77555	5.63714	6.3202	5.77323	6.53903	6.47372
BAHD1	Q8TBE0	6.56821	6.82406	6.3288	7.62336	6.16871	5.62663	7.20641	5.8478	6.66475	6.41775	6.88328	6.95882	5.16012	4.95466	4.94563	5.82103
CACHD1	Q5VU97	6.23782	6.00991	6.26016	6.36204	5.69681	6.54038	6.12852	5.68803	6.44968	7.0414	6.76697	6.06972	6.22037	5.8186	6.42708	6.28251
CIAO3	Q9H6Q4	6.57214	6.24134	6.94876	5.7953	6.43049	7.15994	5.76628	6.59272	5.96964	6.98815	6.6977	6.56813	5.65528	5.65251	5.28052	5.58111
RMI2	Q96E14	6.36591	5.31135	6.14974	5.57231	5.3249	4.68054	5.71471	7.4823	5.7591	6.84998	5.70982	6.95451	5.91702	7.19419	6.95251	8.06111
STYX	Q8WUJ0	6.27125	5.64147	6.59638	5.6612	6.54765	7.02427	6.53221	6.46437	5.95853	6.51216	6.20837	5.69239	5.71182	6.49679	6.44168	6.23945
NCBP2AS2	Q69YL0	5.84482	5.9469	5.24028	6.34083	6.6056	6.22873	6.55471	6.83376	6.22785	6.16613	6.16187	5.83871	7.08222	6.2586	5.85998	6.80903
TFDP1	Q14186	6.00512	6.33642	6.25716	5.66318	6.21811	6.40839	5.9403	6.36467	6.40354	6.46047	6.13331	5.77011	6.91141	6.43571	6.38063	6.31148
TAF4B	Q92750	6.7677	6.06059	6.17299	6.16583	5.9052	6.34086	6.13979	5.52696	5.59526	5.89288	6.80816	5.78293	6.65028	6.06955	6.90416	7.21686
STN1	Q9H668	5.61687	6.39015	5.88108	6.17497	6.30626	6.18841	5.96169	6.27511	5.78978	5.71078	6.25758	5.87612	7.13839	6.83473	6.98103	6.61706
PRKCZ	Q05513	6.58675	6.48326	7.28065	6.32084	6.08243	5.83963	6.44442	6.02	6.44957	6.95088	6.65091	6.56914	5.49765	5.7025	5.24645	5.87493
HSF2	Q03933	6.40365	6.19084	6.14912	6.15343	6.73097	6.96967	6.35654	6.25526	6.4886	7.70038	7.67645	6.99973	5.3845	4.9445	4.47945	5.11693
SUPT20H	Q8NEM7-3	6.11018	5.63989	6.24387	7.23124	6.536	6.50104	6.04128	5.29227	5.47378	5.97872	6.90984	6.84818	6.17873	6.47558	6.16303	6.37637
SNX25	Q9H3E2	6.50771	6.44455	5.76648	6.7376	5.54945	5.87768	6.14423	6.35272	6.76926	6.16303	5.95157	6.3848	5.44735	6.65841	6.17022	7.07494
TMEM175	Q9BSA9	6.76569	6.53734	5.79335	6.83587	6.0273	5.60448	6.45191	6.094	6.38703	6.12797	6.08435	6.6693	5.98876	6.16418	6.2175	6.25096
NEU3	Q9UQ49-2	6.01214	6.67453	6.87707	6.21037	6.16356	6.16349	6.76255	6.30011	6.51971	6.96838	6.20871	6.3169	5.69642	6.00588	6.20268	5.9175
TXN2	Q99757	5.38782	5.16178	4.3971	5.95307	5.33537	4.8584	5.5434	6.71513	5.10151	6.33901	5.15738	6.22835	6.16875	9.77099	7.35239	10.5295
DSCC1	Q9BVC3	5.60077	6.42636	5.91204	6.07767	6.57398	5.64811	6.87463	6.40214	6.34534	5.60948	6.58227	6.39381	5.94888	6.85175	6.4729	7.17986
SLC25A46	Q96AG3	5.97137	6.38659	6.23504	6.03638	6.03195	6.09868	6.20678	5.54745	6.09562	6.2314	6.19248	6.677	6.65945	6.47317	6.45927	6.69738
LSM14B	Q9BX40	6.75292	6.90731	7.37498	6.16039	6.41644	7.3476	6.32099	5.89424	6.52548	6.48498	6.5988	7.42306	4.83342	5.05035	4.87016	5.09017
PARP16	Q8N5Y8-3	6.16179	6.50434	6.44312	6.83092	6.44789	6.1345	6.63682	5.78131	6.2607	6.2446	6.28885	5.68202	6.09365	6.02286	6.58939	5.87724
SHB	Q15464	6.06877	6.73694	5.79378	6.21035	6.31057	6.04313	6.06119	6.65565	6.74348	5.77962	6.41142	5.92487	5.63653	6.62244	7.09172	5.90955
ENGASE	Q8NFI3	5.70913	5.80607	6.0304	5.92604	5.62832	5.6975	5.78664	6.14034	6.12571	6.4784	6.36948	6.34369	6.81968	6.99709	7.14718	6.99431
ARG2	P78540	5.27784	4.67726	5.72083	5.25483	7.12966	6.5106	6.75905	6.89966	6.07259	6.04946	6.19902	6.17691	7.19209	6.15819	6.47721	7.44479
QSOX1	O00391	6.03705	7.09729	5.35421	6.56569	6.14692	7.2302	6.78523	6.15983	6.82483	6.06426	5.87034	6.31603	6.11698	4.89533	5.29642	7.23939
MAD2L2	Q9UI95	6.52063	6.46249	6.12346	6.01395	6.54405	5.95362	6.43665	6.10716	6.27972	6.54796	6.4688	6.61239	6.5537	5.90033	6.07827	6.29682
OSMR	Q99650	7.13171	7.25161	6.69108	7.76858	6.32455	6.10866	5.87559	6.18653	6.78054	6.40889	7.03515	6.75646	4.82005	4.63262	5.04591	5.18206
FBXW5	Q969U6	6.25911	5.74397	6.6516	6.11986	6.28312	6.75288	6.46673	6.3721	6.14995	6.57257	7.48405	6.43297	5.8396	5.79527	5.84318	5.23303
EMB	Q6PCB8	6.47566	6.7356	6.30878	6.223	6.23217	6.61425	6.33213	6.23472	6.64316	6.57308	6.03611	6.30112	5.98992	6.14863	5.13086	6.02082
SEC22A	Q96IW7	6.6633	7.01599	5.85705	5.65327	4.64565	7.3248	6.06163	5.96064	6.65054	5.89272	6.27865	6.17336	6.27241	6.11592	7.86855	5.5655
PALB2	Q86YC2	6.69853	6.54899	6.32141	7.08006	5.93214	6.23671	6.29776	5.51031	6.39811	6.55279	6.26137	6.21604	6.07263	5.52606	5.85184	6.49525
CEP120	Q8N960	7.33452	6.20368	6.4139	7.22759	6.00803	6.72789	6.94642	5.4288	7.70701	5.83407	5.59186	6.44084	5.33899	5.45237	5.51303	5.83101
COQ9	O75208	6.24615	5.2129	5.06052	5.32446	6.30487	5.21057	6.49	6.34455	5.31449	6.70753	5.12674	6.12052	6.73851	8.13216	7.95472	7.71131
ZSWIM9	Q86XI8-2	6.89145	6.3151	6.41674	7.01906	6.45202	5.64139	7.25624	5.60150	6.41173	6.23814	6.25764	6.59935	5.86135	5.60873	5.08215	6.34732
C18orf25	Q96B23	5.59119	6.03217	5.83107	5.49214	6.04957	5.9262	5.93339	6.28764	5.62893	6.03181	6.25892	6.07451	7.38927	6.84851	7.59778	7.02692
FGFR1OP2	Q9NVK5	7.25145	5.31175	4.9777	5.34898	6.34389	4.87598	5.64767	7.31054	5.90548	6.5892	6.02131	7.6056	6.45534	6.48946	6.4657	7.39992
RNF6	Q9Y252	7.18789	6.87804	6.6971	7.40605	6.51563	6.87265	7.23634	5.75965	6.53259	6.73432	6.40105	6.71711	4.71458	4.59186	5.26499	4.49016
KIF3C	O14782	6.60578	6.40384	6.61987	5.88575	6.34549	6.31295	6.3207	6.80671	6.42441	6.30856	6.44347	6.5358	5.41024	5.83552	5.8806	5.86031
STARD9	Q9P2P6	6.33938	6.33026	7.05251	6.69017	6.00496	6.33115	6.40925	6.1614	6.3406	6.68248	6.22132	6.4169	5.4545	5.87989	5.65268	6.03253
TMEM263	Q8WUH6	5.40608	5.22238	4.78974	4.77964	5.82967	5.04636	7.15764	7.66767	8.58476	6.26385	6.32113	5.63688	7.06011	7.61481	6.83321	5.78606
NDUFB1	O75438-2	5.86157	5.59642	5.6841	6.02171	5.75221	5.08731	5.10166	6.64446	5.21575	5.55501	4.77151	5.05567	5.99559	9.37511	7.5086	10.7733
PPP2R3A	Q06190	6.20877	6.21831	6.65229	6.40895	5.91164	5.53537	6.30612	6.82968	6.80033	6.52847	6.37648	6.51553	6.50242	6.23779	5.35353	5.60116
POLR2H	P52434	5.86287	6.05062	5.66086	6.06571	6.3396	6.84809	6.15217	6.85175	6.49779	6.82944	6.62497	7.05634	5.63013	5.62402	5.71945	6.18619
LYSMD3	Q7Z3D4	5.99981	5.6686	5.32811	6.09223	6.52104	5.76732	5.86852	6.20788	5.959	5.75274	6.18603	5.93867	7.37823	6.78545	7.40611	7.14025
SLC6A6	P31641	6.14489	5.83988	6.38212	6.82833	6.3793	6.9124	6.56778	5.76441	6.83181	5.69648	6.88574	6.45997	5.42146	6.40629	6.46242	5.01672
MAML2	Q8IZL2	5.83514	5.86683	6.63909	4.99948	6.11617	6.00881	5.54734	6.29251	6.78199	6.11871	6.41428	6.13261	7.4236	6.93346	6.61976	6.27023
PLCG2	P16885	5.92726	6.49998	7.21999	6.07068	6.29097	7.05027	6.10441	6.95153	6.93785	6.12228	6.78213	6.617	5.8697	5.03491	5.78621	4.73484
ZNF302	Q9NR11-2	5.75748	7.82219	6.71947	7.89718	6.35332	5.42374	6.61446	5.75715	6.57943	7.2065	6.79336	7.13501	4.85874	5.23878	4.66469	5.17851
PAFAH1B2	P68402	6.57544	5.09734	6.50791	4.25945	6.1242	5.79661	4.6483	6.92481	4.79462	6.21622	5.34922	7.21761	5.75687	8.72859	8.35779	7.64503
CPEB4	Q17RY0	6.37305	5.79638	6.47299	5.91494	6.77833	6.97963	5.78124	6.67659	6.89103	6.44203	6.32852	6.56591	6.27219	5.40163	5.62018	5.70537
CRAMP1	Q96RY5	6.15205	6.91937	6.4977	6.85557	6.23601	5.87696	6.89689	5.95373	7.03862	6.06108	7.26354	5.2481	5.62728	6.39537	5.47342	5.52229
CCDC85B	Q15834	6.63687	5.87348	6.5799	5.3887	6.31561	6.53345	5.81441	6.46711	6.47471	6.99023	7.54865	7.00385	6.12651	5.22436	5.47225	5.5499
B4GALT1	P15291	6.61625	6.45293	6.03856	6.67061	6.12181	6.21635	6.41513	5.91946	6.96367	6.61577	6.51621	6.27703	5.96894	5.66387	5.64084	5.90256
CENPS	Q8N2Z9	6.172	7.15871	7.4033	6.42757	6.09268	6.46671	5.28816	6.16688	6.74294	6.3841	6.07061	6.35606	5.73511	5.63957	5.42433	6.47127
A2M	P01023	4.84701	7.0326	5.06876	4.43066	4.29987	6.85082	5.83431	5.19752	7.64031	5.75659	6.20769	6.47827	6.71778	6.28238	9.77102	7.58443
FBXO7	Q9Y3I1	5.82118	5.49778	6.66453	6.3356	5.94861	6.83756	5.81466	5.98244	6.3628	6.84957	6.12986	6.60813	6.67338	6.15637	5.61891	6.69861
F8A1	P23610	5.63564	5.59736	6.20297	5.19495	6.33521	6.53741	5.47451	6.92149	5.78406	7.01368	6.88502	7.27551	5.60902	6.46878	6.63921	6.42516
MAP3K7	O43318	6.63191	6.51298	6.3499	6.15743	6.3901	6.66909	6.01924	6.67685	6.27181	6.66506	6.47973	6.50041	5.7978	5.85524	5.53131	5.49113
RPS27	P42677	5.98009	6.06902	7.00779	6.47944	6.57588	6.33819	7.05467	6.15683	6.55405	5.43235	6.64115	6.63981	4.92643	6.42511	5.81993	5.89925
WEE1	P30291	5.33296	5.74564	6.21797	5.3078	6.3006	6.04981	6.05748	5.35971	6.22558	6.55545	6.90767	5.78013	7.17872	6.96841	7.22038	6.79168
PSRC1	Q6PGN9	6.29962	6.9742	7.71623	6.47107	6.15665	6.93716	6.20789	6.87916	6.53696	6.82857	7.57282	8.22781	4.60168	4.36095	3.49399	4.73526
MCOLN2	Q8IZK6	6.82717	6.35147	6.59613	6.71767	6.46668	5.8761	6.58959	6.28862	6.26141	6.32328	6.48999	6.03687	5.65007	5.56261	5.95509	6.00725
CASTOR2	A6NHX0	6.42384	6.0527	6.58685	5.60472	6.05724	7.02197	6.39643	6.57887	6.37751	6.86792	6.48704	6.63754	5.54871	6.23703	5.55606	5.56557
ELL2	O00472	7.42138	7.307	7.55652	8.26716	6.48174	6.79104	7.40568	6.95891	7.32951	7.2527	7.0273	6.4535	3.22586	3.43733	3.38353	3.70085
SMIM8	Q96KF7	6.26361	6.28123	5.70428	6.79023	6.16792	6.74977	6.25426	7.52327	5.2047	5.93495	6.0254	6.24134	5.79457	6.50858	6.26315	6.29274
PIP4K2A	P48426	5.99815	6.16462	6.3103	6.07202	6.22137	6.75842	6.84937	5.54823	5.94547	6.14889	6.0973	5.79397	6.25813	6.4524	6.95481	6.42656
PEA15	Q15121-2	6.28675	4.88705	6.40581	4.24504	5.72159	5.35364	5.44527	7.39643	4.76937	6.4784	5.66562	7.50109	5.05988	8.95834	7.27979	8.54593
PPP1R15B	Q5SWA1	6.29041	6.6938	6.66005	7.20025	5.76802	5.70916	6.66033	6.03897	6.49019	5.96637	6.59099	6.33568	5.74231	5.74614	5.92823	6.17909
UBE2E3	Q969T4	5.89848	5.37861	5.85367	5.73565	5.34016	6.09945	6.37105	7.74713	7.40775	7.05142	5.66724	6.7358	6.46415	6.45414	7.15176	4.64352
GTDC1	Q4AE62	6.39593	6.73832	7.03879	6.20244	5.53493	7.48562	5.61462	5.78499	5.75737	6.61602	6.55024	6.49371	5.76234	6.58504	6.00964	5.43
RNASET2	O00584	6.6643	5.35383	5.99075	5.20814	6.43724	4.98496	5.42883	6.60464	5.25738	6.22132	5.62319	6.17454	6.93722	7.84538	7.53137	7.7369
MRPL49	Q13405	5.14643	5.0229	5.00485	4.911	5.8017	4.82142	6.01575	7.18372	4.47923	6.69498	5.51216	7.16151	5.5612	8.51478	8.53286	9.63553
BICRAL	Q6AI39	6.32679	6.52505	6.13354	6.19069	5.81938	6.10924	6.0388	6.07189	6.11498	6.4751	6.32264	6.84878	6.75437	6.14356	6.14717	5.97803
SLC12A7	Q9Y666	6.05078	6.20897	6.39437	6.06311	6.32867	6.26986	5.74578	5.38929	6.73086	6.98903	6.69612	6.50872	5.9756	6.09176	5.87591	6.68117
SLC12A6	Q9UHW9-2	6.76504	6.67374	6.60199	6.64807	6.75873	6.16345	6.33561	5.70085	6.02322	6.09397	6.60221	6.3212	5.70821	5.51703	6.14392	5.94276
BRAF	A0A2R8Y8E0	6.51387	6.19232	7.07647	5.84153	6.40678	6.63234	6.40914	6.89679	6.51098	6.18439	6.17294	6.75292	5.76264	5.78285	5.48371	5.38033
PER1	O15534	6.16188	6.69036	6.56317	5.6678	6.02995	6.46588	6.26668	6.4757	6.68819	6.65812	6.48664	6.3507	5.88978	5.8585	5.66916	6.07749
NRAS	P01111	5.90972	6.06744	6.1549	6.73918	5.88133	5.68679	6.54046	6.41935	6.06447	5.85142	6.01893	6.54662	7.10387	6.37646	6.39053	6.24852
USPL1	Q5W0Q7	6.35002	6.52117	6.52848	7.16132	5.81694	5.77336	6.46963	6.15724	7.0344	6.36838	6.57468	6.61753	5.63324	5.34511	6.05063	5.59786
C2orf49	Q9BVC5	5.83993	5.81379	5.43428	5.72233	6.41957	5.91026	6.25747	5.82647	5.58396	6.09204	5.83047	5.99665	7.75049	7.37085	7.62651	6.52494
MCEE	Q96PE7	5.34329	4.86251	5.0161	4.84866	5.80121	5.20901	5.45319	7.01805	5.09205	6.88507	5.78302	6.96822	6.36065	8.51095	8.35799	8.49001
C12orf45	Q8N5I9	6.25271	6.33456	7.10824	6.46653	6.20562	6.87102	5.97291	5.99564	6.31058	6.24941	7.37839	7.12936	5.77787	5.35719	5.09864	5.49132
SPIN4	Q56A73	5.80353	6.42785	5.73915	6.38458	6.03045	6.54435	6.99174	6.14715	5.89127	5.82849	6.47234	5.55115	6.51982	5.90014	7.13147	6.63653
USP38	Q8NB14	5.28935	5.80043	6.188	5.74201	5.72883	6.15523	5.95673	6.65702	5.94138	6.03041	6.30278	6.16462	6.66741	7.35988	6.93131	7.08459
STEAP1	Q9UHE8	5.96113	7.24986	6.31845	7.29283	6.11738	6.19344	5.83346	6.71071	6.32248	6.15757	5.94128	6.54669	5.33286	5.93254	5.92669	6.16264
PPHLN1	Q8NEY8-2	5.9962	6.40914	5.45468	6.61927	6.21617	6.69536	6.41872	6.54953	5.91966	6.27365	5.95966	6.14155	6.5996	6.08781	6.05609	6.60292
MKNK1	Q9BUB5	6.14863	6.88777	7.81744	6.43134	6.2617	6.9127	6.59729	6.53268	6.73666	6.86989	7.48555	6.83074	4.90031	4.77197	4.34576	4.46957

EMC10	Q5UCC4	7.00638	6.73356	5.51983	5.89185	7.14365	5.88257	6.16001	6.35884	5.87357	6.62273	5.45588	7.02154	6.19496	5.70523	6.38282	6.04656
ZNF8	P17098	5.82081	5.72838	5.76138	6.37851	5.78873	5.57521	6.90578	5.98753	6.10712	5.80849	5.71834	6.5729	7.14009	6.64956	6.76623	7.29092
CCNL1	Q9UK58	5.89069	6.97479	6.24503	6.52149	6.17324	5.81278	6.30561	5.38906	6.10538	4.98654	6.01684	5.18448	7.27901	7.16414	7.69465	7.25626
FAM177A1	Q8N128-2	6.38861	6.01795	6.31232	7.03484	6.09148	6.17253	6.70956	6.1586	6.20324	6.84928	6.14385	5.94501	5.77007	5.55737	6.22617	6.41912
RYK	P34925-2	6.06231	6.59771	7.21242	7.12253	6.17558	5.91712	6.30203	5.6141	6.40848	6.45735	6.6518	7.0772	5.62837	5.17524	5.77817	5.81959
RILPL1	Q5EBL4	6.59412	6.30663	7.39428	5.91308	6.37827	6.66894	6.4354	6.01745	7.20481	6.77395	8.17803	5.3923	5.16004	4.93604	4.80203	5.84463
TMED8	Q6PL24	5.87228	5.99306	6.92218	6.15037	5.95299	6.83677	6.31489	5.22606	6.17371	6.24964	6.30295	7.09345	6.49297	5.8557	6.24869	6.31428
AMD1	P17707	6.90776	6.24136	8.3935	6.25045	6.28345	5.84357	5.99329	7.13252	7.01962	7.51832	7.16319	8.65522	3.49268	4.66113	4.00584	4.43811
LSM2	Q9Y333	5.75652	5.30093	5.63371	5.53499	6.78855	5.92573	6.60227	6.48474	6.60359	6.28667	6.37781	5.82103	6.58914	6.33136	7.49926	6.4637
VTN	P04004	7.15015	4.85787	3.23793	7.58909	4.15028	5.58133	9.07061	5.10655	6.64181	5.93235	3.95489	4.59312	9.04676	5.09792	8.03871	9.95063
NAB1	Q13506	5.51422	5.49061	6.15936	5.16082	5.88498	6.23958	5.34232	6.50908	6.29416	6.38492	6.73317	6.45727	6.8288	6.7887	7.51697	6.69503
MBOAT2	Q6ZWT7	6.15475	6.71045	6.04923	7.06139	6.26699	6.06805	5.8722	6.0863	6.09178	6.48834	5.66993	6.12576	5.58722	6.69893	6.70257	6.36612
NECTIN3	Q9NQS3	6.26732	7.20006	5.72421	6.537	6.02821	5.94157	6.4765	5.72429	6.14543	6.36557	6.10791	6.17869	6.91569	6.16863	5.96223	6.25667
RFFL	Q8WZ73	6.86951	6.92878	6.35605	6.95144	6.59144	6.08435	6.93988	5.81594	5.80419	6.02557	7.02548	6.40739	5.59338	5.18394	6.35921	5.06345
WDCP	Q9H6R7	5.96168	6.12323	6.66632	6.45861	6.2923	6.68263	5.79913	6.38052	6.28428	6.73259	6.20657	6.9195	6.50102	5.95224	5.5085	5.53088
C12orf65	Q9H3J6	6.29325	6.43967	5.83791	6.7792	5.96656	5.52162	6.60456	5.61097	6.21349	5.78512	5.63429	6.42017	6.66547	6.56691	7.06904	6.59177
FOXJ2	Q9P0K8	5.68933	5.41152	5.8965	5.04575	6.27528	6.64913	5.58269	5.19352	6.37194	7.17979	6.26357	6.172	7.53862	7.20755	6.74914	6.77367
ELAPOR2	A8MWY0	6.5052	7.25744	6.08763	6.24929	5.97866	6.35493	6.06675	5.89975	6.2471	6.1346	6.822	5.77817	5.80437	6.51667	6.1092	6.18824
AKT1S1	Q96B36-3	6.91998	6.27003	6.29423	6.03422	6.03744	5.79027	5.59033	7.76049	6.53709	6.75503	6.64398	6.87368	6.49415	5.2562	5.2325	5.51038
MED6	O75586-2	6.45473	5.90092	6.7156	6.19893	6.29684	5.82657	6.31316	6.38108	6.15273	5.64805	6.59588	6.23445	6.87549	6.39174	6.1075	5.90635
CADM4	Q8NFZ8	6.12197	6.99896	6.61252	6.34894	6.40392	6.15101	6.68982	5.8954	6.08298	5.42353	6.31976	5.48885	6.90649	6.40163	5.98474	6.16949
DNAJC15	Q9Y5T4	6.12943	6.18043	5.94104	6.69792	6.63546	6.7035	7.01448	6.48484	5.67993	6.767	6.3365	6.25464	5.65714	5.69164	5.89361	5.93245
C16orf72	Q14CZ0	7.42735	6.769	7.05453	6.52424	6.7917	6.86874	6.38852	6.14968	6.53643	6.34836	7.08719	6.59294	4.87991	4.28212	5.00067	5.29862
ACD	Q96AP0	6.50322	5.95135	6.94795	6.58575	6.66132	6.23455	6.1386	6.72112	6.32091	5.63019	6.2345	7.1424	6.81651	5.04377	5.24719	5.82067
POLE3	Q9NRF9	6.44137	6.46603	6.85375	6.65338	6.2599	6.50238	6.09797	6.62506	6.21897	7.48576	6.46793	5.91111	6.07974	5.60776	5.59019	5.7387
KLHL42	Q9P2K6	6.04963	5.75208	7.03018	5.66209	6.59796	7.47855	6.02262	6.08177	5.90259	6.22583	6.82969	6.06608	6.38179	5.92453	5.81406	6.18054
MRPL55	Q7Z7F7-2	6.21359	6.32414	5.61423	6.80344	5.80133	5.93762	6.47652	6.34604	6.26037	6.1836	6.3261	6.23158	6.28763	6.40622	6.44349	6.3261
RP9	Q8TA86	5.84898	5.91364	6.55256	5.84119	6.24785	6.19933	6.39941	4.9765	6.23191	6.41364	5.49908	5.76825	6.52795	6.976	7.26749	7.33624
LIN37	Q96GY3	6.47143	5.97922	6.23649	5.92588	5.90462	6.36966	6.8788	5.78833	6.22124	6.96058	6.57065	5.91071	5.98795	6.34336	6.62751	5.82357
DEGS1	O15121	6.45765	7.37231	6.52846	7.31253	7.18478	6.3019	6.52125	5.67303	5.78176	6.11053	6.46033	6.25233	5.92861	5.75792	5.48076	4.87587
RASSF3	Q86WH2	6.12409	6.657	7.1102	6.85179	6.33189	5.01797	6.35014	5.72746	6.95101	6.55738	7.41227	6.6384	5.39779	5.3121	6.10956	5.45095
PDXP	Q96GD0	5.18978	5.63333	6.14105	6.17745	5.95469	6.96388	6.48189	6.9411	6.24898	6.64496	7.0143	6.83024	5.41379	6.83842	5.81491	5.71123
SNAP47	Q5SQN1	5.98759	5.76775	6.60794	6.07098	6.44846	6.50109	6.02711	5.75075	6.88951	6.83101	6.62458	6.10007	6.30876	6.09128	6.16902	5.82411
ZKSCAN4	Q969J2	5.88961	6.42538	6.14575	6.01769	5.61445	5.96885	6.43785	5.85296	5.87103	6.58867	6.33143	6.14965	6.91131	6.83881	6.41598	6.54059
C1orf198	Q9H425	7.28589	6.04016	6.89235	5.92692	6.09657	6.75719	6.49837	6.50126	6.39041	5.94046	7.34997	6.6942	5.71762	5.78372	4.74206	5.38284
CBWD2	Q8IUF1	6.4415	6.47821	6.74436	6.01166	6.28824	6.63953	6.64542	7.19896	6.54023	6.5917	6.89272	6.56902	5.20108	4.99778	5.19441	5.56518
KHNYN	O15037	6.34313	6.41928	6.32629	5.16048	6.17587	6.96815	6.30307	6.5481	5.98916	6.74666	6.56608	5.94864	6.38918	6.60732	6.02799	5.48059
DNAJC25	Q9H1X3	6.51247	6.34833	6.02895	6.59893	5.77986	5.79716	6.84768	5.0244	6.02053	6.35828	5.65402	6.0679	6.75652	6.73786	6.82548	6.64163
ZBTB9	Q96C00	7.08152	6.88147	6.29102	6.74178	5.95347	5.86191	7.055	6.23391	7.0599	6.49332	6.25128	6.73521	5.23334	4.82537	5.58015	5.72133
TOR3A	Q9H497	6.18736	6.65992	6.32324	7.42288	6.51714	6.49958	6.34419	6.08377	5.52218	6.03086	5.84604	6.13922	5.86453	5.84796	6.43801	6.27312
STX1A	Q16623	6.26968	6.29971	7.16139	6.98133	6.13562	6.50723	6.69888	7.05169	6.45743	6.63696	6.93029	6.71817	5.27463	4.87078	4.67703	5.32918
CNNM2	Q9H8M5	6.43662	6.86741	6.06478	6.81785	5.74041	6.31225	5.90797	6.10296	6.13599	6.22855	5.76327	6.78781	5.86927	5.95835	6.24886	5.75765
RAB5IF	Q9BUV8-5	6.45329	6.0684	6.5079	6.61702	6.18255	5.42602	6.38274	5.50815	6.23115	6.09812	6.25834	6.02472	6.4153	6.14126	7.11889	6.56614
FRS2	Q8WU20	6.46743	6.79061	6.33169	6.83462	6.12368	6.34051	6.8844	6.35033	6.49017	5.7147	5.8118	5.78305	5.99675	6.01068	5.92738	6.14219
TYW5	A2RUC4	5.99635	5.9818	6.69276	5.78618	6.09522	6.60558	6.13399	6.53853	6.81339	6.78726	7.1288	6.87624	5.58139	5.75889	5.79475	5.42886
TOPORS	Q9NS56	6.40471	6.90405	5.90033	7.35148	5.88983	5.75196	6.69658	6.24462	6.03614	6.13225	6.19337	6.21205	5.91252	6.77447	5.44061	6.15501
GINS2	Q9Y248	4.93201	4.40932	5.3682	4.41537	4.94899	5.15272	5.84273	7.0107	5.27572	6.24303	5.39492	6.86322	4.59721	9.87332	9.05471	10.6178
GRAMD4	Q6IC98	6.18084	6.91821	5.79893	6.36592	5.88016	6.01965	5.93234	6.00417	6.20831	6.60957	5.84518	5.53398	6.45426	6.8088	6.48094	6.95874
YIPF6	Q96EC8	5.8791	6.18619	5.8625	6.22451	6.41506	5.85437	5.76868	6.56472	6.59499	6.2069	6.28941	6.3245	6.03512	6.59723	6.61291	6.5838
MTPN	P58546	5.83975	5.10235	5.32633	4.28325	5.82846	5.38132	4.8969	7.37693	5.05663	5.74344	4.98095	5.61836	4.2349	11.8883	7.46541	10.9767
RPS28	P62857	6.68653	6.11973	6.63386	5.67282	6.95078	6.24342	5.74388	6.29535	7.21255	6.13643	7.02602	6.96438	5.84102	5.39953	5.65097	5.42274
MARCKSL1	P49006	6.04054	5.12438	5.75212	5.36841	6.62506	4.89751	5.47773	5.92354	5.43797	6.2491	6.17903	6.48069	7.95617	7.31779	7.74804	7.42194
PSD3	Q9NYI0	4.87958	7.02581	6.08265	5.3841	6.17977	5.49243	5.77601	5.69501	6.61751	6.21479	7.35753	6.93029	7.46175	5.67286	6.2168	7.0131
PNMA2	Q9UL42	6.19324	6.37713	6.93786	5.72421	6.22862	6.88446	6.06442	6.85384	6.23513	6.79991	6.46133	6.99708	5.86535	5.72643	4.91175	5.73924
RIC8B	Q9NVN3-1	5.49791	5.90083	7.42545	7.27533	6.39019	6.81613	6.49939	6.67675	6.30946	5.95148	6.123	7.04295	5.56436	5.98301	4.6066	5.93716
EMC6	Q9BV81	6.36471	6.55667	6.0851	6.66215	6.16067	6.04479	6.19609	5.66483	6.50965	5.48203	6.55947	6.3981	6.23954	6.04371	6.27791	6.7546
MRPL54	Q6P161	6.16994	6.53636	6.35625	6.7359	6.02085	5.86314	6.79153	6.00641	6.01006	5.53616	6.6316	5.72823	6.3256	6.54117	6.43021	6.3166
TNRC6C	Q9HCJ0-2	6.54163	6.59929	6.61273	5.86824	6.41377	6.70749	6.12517	5.98348	6.51793	6.5486	6.71001	6.87142	6.28005	5.70071	5.30584	5.92036
BTBD10	Q9BSF8-2	6.19522	6.78079	7.21951	6.79795	6.20908	6.80283	6.34106	6.65652	6.40805	6.57566	7.26036	7.21716	4.70734	5.10155	4.79394	4.93298
CA13	Q8N1Q1	5.58807	6.08321	6.45631	5.88579	6.54265	6.71603	6.80916	5.98998	6.24187	5.98901	7.37688	6.77227	6.17681	6.13944	5.16551	6.06701
REEP2	Q9BRK0-2	6.18034	6.22794	6.01309	5.82374	5.98976	6.07364	7.02774	6.51666	5.62876	6.14821	5.67966	5.76217	6.93151	7.2992	6.19266	6.50491
SSH3	Q8TE77	5.83068	6.26184	5.49874	6.3692	5.86072	6.91593	6.24559	6.52395	6.27049	5.65243	6.46616	5.94697	6.69013	6.65834	5.9892	6.81962
TBC1D2B	Q9UPU7	6.6476	5.56123	6.48462	5.86567	5.96998	6.72752	6.31217	5.92046	6.13328	6.89697	7.49556	7.02429	6.25588	5.24511	5.93089	5.52876
HLCS	P50747	6.37999	6.11854	7.1239	5.95491	6.10779	6.67793	6.34361	6.36573	6.5067	6.63072	7.02369	6.25191	5.85245	6.02441	5.23304	5.40468
CHCHD1	Q96BP2	5.25907	5.46856	6.22743	6.11672	6.96153	7.75254	6.79843	5.93943	5.52145	5.58452	7.87389	6.39526	6.69814	6.23023	5.47709	5.69572
ZNF174	Q15697	6.39091	6.93316	6.01616	6.92724	6.10008	6.28459	6.16734	6.16355	6.16133	6.33923	6.2491	6.5872	6.05451	6.04876	5.70199	5.87485
DPH6	Q7L8W6	6.10438	5.81422	6.77774	5.59391	6.94109	6.80427	6.11787	7.73655	7.31889	6.06194	7.09839	6.72051	5.15703	5.79011	4.86968	5.0934
AP1AR	Q63HQ0	7.0423	6.51612	6.47349	6.12074	5.88144	6.09106	6.89388	6.00726	6.53032	6.75928	6.54538	5.96768	5.75506	5.94478	6.01101	5.46021
RHOBTB3	O94955	6.69057	6.68827	7.01934	7.34925	6.3541	6.47416	6.83085	6.0238	6.75314	6.95678	6.64795	6.43978	4.68498	5.07701	4.77419	5.23584
NEIL3	Q8TAT5	6.94381	7.07151	6.89195	6.43141	6.57371	6.11351	6.49335	5.9336	7.06535	7.1013	7.58929	6.86547	4.58857	5.18217	4.6544	4.5006
ALKBH7	Q9BT30	7.60633	6.43604	6.05069	6.86216	6.00409	5.59379	5.68685	6.1515	6.54968	6.60119	6.57144	7.50128	5.60592	5.78094	5.36654	5.63156
IFT46	Q9NQC8-2	6.5113	6.54234	6.38721	5.60217	7.08547	6.17818	5.87869	6.74923	6.14418	6.41714	6.54556	6.65012	6.60468	5.75007	5.42655	5.52712
TEDC1	Q86SX3	6.13797	6.83127	7.08746	6.71863	6.51308	6.36742	6.68068	6.12393	6.73386	7.04667	6.88288	7.81368	4.83283	4.69092	4.39932	5.13941
NSUN3	Q9H649	6.29126	6.56874	6.05795	7.26845	5.95716	5.8975	6.15102	6.46881	5.96284	6.34529	6.13004	6.22603	6.76898	5.74608	6.11977	5.94009
TSPO	P30536	6.49029	5.63172	5.84511	5.86589	6.38624	5.7164	6.24831	6.00359	6.05775	6.07127	5.97616	5.77531	6.56313	6.84643	7.42756	7.09485
LRRC20	Q8TCA0	6.02519	4.66002	6.42263	4.87824	6.17629	6.11789	6.0169	7.17604	5.45041	7.25492	6.50878	6.6493	5.20656	7.45052	6.82983	7.17648
GPN3	Q9UHW5-2	6.54401	6.22419	6.66383	6.47205	5.83358	5.98515	6.56279	7.02075	6.90724	6.06057	6.44022	6.96106	5.42841	5.33377	5.3662	6.19618
DEPDC1B	Q8WUY9	6.6984	6.54091	6.26369	6.13278	6.26227	6.06399	6.71062	6.44625	6.05598	6.60663	7.03436	6.89582	5.6722	5.40548	5.68268	5.52796
DPM3	Q9P2X0-2	6.37771	5.68159	5.19311	5.64301	5.70855	6.28564	6.19604	6.23528	5.7354	5.48659	5.93015	6.08858	6.35676	7.87409	6.78454	8.42296
RWDD2B	P57060	6.64647	6.43844	7.73352	6.2295	5.88448	6.95099	5.82732	5.99541	5.6552	5.95861	7.30222	6.38785	5.48615	5.53488	5.55011	5.50886
MED31	Q9Y3C7	6.3135	4.8528	6.23194	5.89329	6.05826	5.13084	5.00533	5.91349	5.08425	6.3747	5.97252	6.67013	6.00565	7.97007	8.32962	8.19361
CYB5B	O43169	6.02962	5.48245	4.93355	4.96364	5.06881	5.11602	5.95883	6.51139	4.84249	6.19115	4.95635	6.16328	6.75957	9.33168	8.38425	9.30692
PET100	P0DJ07	6.2924	5.26971	5.86993	5.6052	6.78692	6.1125	7.11146	6.26909	6.44189	5.66787	6.06676	5.66787	6.83897	6.16528	7.23658	6.59757
SIRT2	Q8IXJ6	6.55688	6.25124	6.2623	5.60609	6.62606	6.75265	6.12774	6.74407	6.55866	6.61042	6.36194	6.38279	6.16897	6.02312	5.52562	5.44146
CCNC	P24863	6.70388	6.42856	5.93221	6.16768	6.65154	5.70307	6.25349	6.11878	6.54033	6.48656	5.5959	5.99247	6.61628	6.29149	6.50558	6.11219
SPOP	O43791	6.42491	5.8297	5.31013	6.21574	5.21169	5.55998	6.2405	5.03815	6.43505	5.09431	5.83384	6.98321	8.3307	6.14142	7.77386	7.57682
UQCC3	Q6UW78	5.37481	6.26993	5.67942	6.29361	5.61913	5.83638	6.10899	6.19466	6.11268	5.373	6.04158	6.11799	6.886	7.49768	6.72573	7.8684
PRKAR1B	P31321	5.4382	6.35838	7.28412	6.25245	6.16179	6.23722	6.16354	6.4923	7.12135	6.88133	6.56665	6.58938	5.74819	6.02152	5.21911	5.46447
CTSV	O60911	6.74862	8.16861	6.42947	7.03328	6.4068	5.91612	6.30277	6.46394	6.42387	6.5445	6.32586	5.6427	5.74361	5.13636	5.37347	5.34
GEN1	Q17RS7	6.25498	6.06194	7.24289	7.20368	6.59888	6.93333	6.17224	6.52358	6.90309	6.46375	7.1445	7.03449	4.94355	4.95167	4.25723	5.3102
CCDC14	Q49A88	7.03931	7.97433	7.04364	7.08316	6.63366	6.3576	5.52633	5.67582	7.01124	7.00106	7.04809	7.60111	4.40497	4.40764	4.60859	4.58345
SMYD5	Q6GMV2	6.83274	6.64796	7.30593	6.59648	6.01143	5.91396	6.24691	6.47558	5.77656	6.97022	5.67464	6.33619	5.91598	5.77001	6.20788	5.31751
TIMP1	P01033	8.59737	9.80922	5.8103	8.23361	7.05525	8.40153	5.74761	8.23392	5.95706	5.85215	7.4401	5.74599	3.08477	3.73933	3.18243	3.10936
TMEM165	Q9HC07	6.0112	5.74766	5.77473	6.80612	6.73314	5.6016	7.0776	5.34666	7.50609	4.12974	5.36299	5.9353	5.19379	7.57364	6.65167	8.54807
TEPSIN	Q96N21-2	6.40366	6.12993	6.72271	6.2985	6.03751	6.38178	6.10103	6.5837	6.2441	6.37238	6.98666	6.33767	5.94616	6.04122	5.85656	5.55642
ADGRA3	Q8IWK6	6.98354	6.78761	6.73322	6.33557	6.51622	6.16159	5.92879	5.49104	6.67582	6.4994	6.67784	6.75744	5.53173	5.53182	5.52581	5.86257
PRICKLE3	O43900	6.13055	6.53914	6.38273	6.49651	6.0479	6.19226	6.48125	6.33123	5.91127	6.47466	6.38299	5.92781	6.21438	6.04469	6.17403	6.26859
ASS1	P00966	6.03805	5.6647	6.0187	5.65494	6.09901	5.47923	6.10936	6.25164	7.25424	7.14897	6.3595	6.40484	6.79061	6.51256	6.60137	5.61228
MMAA	Q8IVH4	6.65043	6.10107	6.02953	6.56435	6.2628	5.5484	6.78757	5.75773	6.21814	5.92238	6.33073	5.442	7.10392	6.05376	6.75763	6.46956
TMEM94	Q12767-2	7.00193	6.60025	5.99159	5.75564	5.9191	5.95162	6.66629	5.12028	6.97394	6.19206	6.50827	5.65976	6.80675	6.2202	6.57139	6.06094
MCUB	Q9NWR8	6.90454	6.24185	5.83879	6.39569	6.72515	6.47938	6.4803	6.35896	5.99358	5.95727	5.7602	6.10343	6.53476	5.79696	6.30102	6.12812
LIN7A	O14910	6.42344	6.62244	6.21992	6.3991	6.66119	7.48022	5.90117	6.59763	5.73316	5.83657	6.56739	5.84091	6.1295	5.2413	5.99059	6.13568
KCNN4	O15554	6.63053	6.59282	6.26177	6.34041	6.49628	6.21732	6.23139	5.70788	6.88004	6.62393	5.97795	5.27135	6.55867	5.91502	6.477	5.81766
ITFG1	Q8TB96	6.8066	6.71275	6.86304	6.53074	6.57536	6.66115	6.81197	6.29053	6.3926	6.29121	6.03801	5.91664	5.5211	5.56706	5.6823	5.33896
YPEL5	P62699	5.78183	6.35207	6.06953	6.18735	5.65992	6.51058	6.77619	6.83337	6.39889	6.64673	6.38959	6.44056	5.20511	6.10865	6.24844	6.39119

PGAP6	Q9HCN3	6.63523	6.46441	6.84624	7.0841	6.47857	6.20511	6.37668	5.65574	6.28107	7.41814	6.53319	6.42843	5.60791	5.58962	5.01197	5.38359
GPATCH2	Q9NW75	6.06767	5.66027	6.61858	6.54417	6.78397	6.38701	6.20388	6.19318	6.18556	6.20472	6.30851	6.12152	5.61049	6.68677	6.40203	6.02168
PLEKHG5	O94827-7	6.40065	7.00434	6.67175	6.68574	6.25631	6.74864	5.5142	6.85429	6.09123	5.09436	7.04144	5.44386	6.2742	5.55341	6.18567	6.1799
PRNP	P04156	7.08693	6.83781	6.02906	6.41009	6.43437	6.05842	6.52666	5.66524	6.23211	6.3297	5.87104	5.75708	6.34714	6.02433	6.11865	6.27137
ARHGAP10	A1A4S6	5.49344	6.4805	6.61869	5.90779	7.68075	6.22185	6.15294	7.20029	5.19998	5.98784	7.41917	6.84956	5.74149	5.75296	5.7277	5.56506
NHEJ1	Q9H9Q4	5.89468	5.56091	5.04966	5.62084	6.26819	5.69215	5.66623	6.7269	5.69935	6.03536	6.46858	6.57626	6.67894	8.67483	6.80528	6.58184
FOXM1	A0A0D9SFF0	6.07377	6.14585	7.32418	5.27537	5.61946	5.46278	6.06502	5.96201	7.01244	5.97367	6.89436	6.76002	6.0989	6.31237	6.72848	6.2913
PTPRG	P23470	6.19976	6.0879	5.84949	6.38447	6.08733	6.17194	6.05211	5.03966	6.53359	6.08505	6.09732	6.37324	7.22608	6.29245	6.78975	6.72986
MIS12	Q9H081	6.22266	5.52347	5.60485	5.5412	5.63758	5.01616	5.39919	5.97292	4.89477	5.87849	5.03595	6.12044	6.00829	9.2704	8.71682	9.15682
ZFYVE27	Q5T4F4-3	6.77726	6.44992	6.04259	6.56752	6.0534	6.40696	6.33145	6.01207	6.35555	6.13858	6.26484	6.31249	6.00591	5.96899	6.32354	5.98893
SUSD5	O60279	6.48452	6.45961	6.20913	5.88719	6.18411	5.56164	6.28527	6.07261	6.52961	6.62774	6.04798	6.59373	6.5076	6.3434	6.22631	5.97954
SGCE	O43556-4	7.04314	6.55386	6.09696	6.20564	7.16269	6.06966	6.96231	5.95468	6.18445	6.55327	6.12672	5.81391	5.94252	5.72704	5.96674	5.63642
HDX	Q7Z353	5.71542	5.87604	6.54057	6.04875	6.09888	6.44019	6.35434	6.19054	6.23019	7.17316	6.45175	6.27616	6.4942	5.46523	6.08958	6.55501
MTERF1	Q99551	6.79519	6.81779	6.48809	7.58082	6.43723	6.13794	6.76189	5.95562	6.3901	6.28645	6.20726	5.57293	5.96982	5.20222	5.81858	5.57807
AMN1	Q8IY45	5.545	6.06258	7.42136	5.41122	6.021	6.53097	6.7233	6.42751	6.69035	6.64638	6.33314	6.17823	5.77082	5.55341	6.51174	6.17299
MBIP	Q9NS73	6.42942	5.81111	5.5897	6.77563	7.1406	6.21618	5.96431	6.46748	5.88677	6.15821	6.55616	6.00622	6.29412	6.24557	6.13697	6.32156
E2F3	O00716	5.95334	6.10036	6.70727	6.1654	6.14505	5.94145	5.81966	5.54585	5.63421	6.62336	6.64481	6.68107	6.95033	6.14942	6.51028	6.42812
PIGN	O95427	5.96529	6.79445	5.9647	6.29498	6.18915	6.04979	6.32249	5.98742	5.76386	6.1692	5.71891	6.00996	6.6851	6.50404	6.76352	6.81713
ATF6	P18850	7.35519	5.9782	6.33519	7.44814	7.05603	6.36465	6.84032	6.11372	6.33995	6.52314	6.69974	6.72527	5.47338	5.084	4.30346	5.35963
RAMAC	Q9BTL3	6.05108	5.82089	5.62848	5.75762	6.83999	5.76649	4.90664	6.63289	5.1535	6.46678	6.02724	7.33036	6.75047	7.14466	6.98154	6.74138
VPS37B	Q9H9H4	5.22879	4.80649	6.92416	4.81643	5.76234	5.30461	5.14698	7.22611	5.18443	6.84582	5.96949	6.96899	5.22477	9.13056	7.51401	7.94602
PUS10	Q3MIT2	5.32094	5.94115	6.14076	5.27299	5.75198	6.32772	5.71267	5.67847	5.71394	6.38605	6.55108	5.8226	7.15975	7.67184	7.47858	7.06948
DCUN1D3	Q8IWE4	6.53601	5.25662	6.68216	7.10479	5.76683	5.42114	6.20139	5.45192	6.16663	6.43361	6.32466	6.22078	6.47119	6.43826	6.56702	6.95697
PLA2G6	O60733	6.09622	6.1887	7.03351	6.16545	5.90255	6.34136	6.4695	5.86671	6.144	6.21643	6.93251	6.32229	6.52999	6.0646	5.40981	6.31637
MTFP1	Q9UDX5	7.10947	5.92471	6.79935	6.68387	6.34622	5.84217	6.6236	6.22285	5.70574	5.7834	5.24351	5.96365	5.76623	6.58905	6.70334	6.69282
MLLT3	P42568	6.29601	6.72722	5.83559	6.79721	6.30957	6.13136	6.93653	6.32937	6.08857	6.31173	6.42695	6.19879	5.36542	6.19361	6.02417	6.0279
GANC	Q8TET4	5.68066	6.79254	7.04655	6.19837	6.36287	6.87392	6.07234	6.07111	7.12195	6.0905	6.16175	6.29653	5.30721	6.50773	5.70709	6.70888
FBF1	Q8TES7-6	5.84498	6.69955	7.30724	6.12971	6.06891	6.41296	5.386	6.13847	6.5473	6.43721	6.13567	6.78212	6.62857	6.02776	5.74398	5.70957
ARHGEF26	Q96DR7	6.34465	6.47729	6.41729	5.99768	6.50428	6.16002	5.76778	5.55443	6.88471	6.67415	6.52507	6.41196	6.02159	6.53457	5.65225	6.07226
PIAS2	O75928	5.8667	5.82459	6.08821	6.8472	5.90776	6.76193	6.01389	5.30834	6.32241	5.71601	5.80168	6.31412	6.09077	7.29176	6.9571	6.88755
FZD2	Q14332	6.67849	6.9948	6.21661	6.98693	6.20896	6.62913	5.98539	5.98573	6.86231	6.19156	6.3299	5.96582	5.4998	5.81899	5.92975	5.71582
SERAC1	Q96JX3	6.17709	6.15978	5.78326	7.15278	5.93175	5.96726	6.3244	6.02839	5.93208	6.03261	6.47071	6.47567	6.09879	6.42551	6.34904	6.69087
TRPM7	Q96QT4	6.61703	6.95063	6.18452	6.35956	6.42872	5.89124	6.00293	5.87178	5.12738	7.17757	6.75728	6.28945	5.80383	5.56265	6.57084	6.40459
RABL3	Q5HYI8	5.99437	6.17295	5.47487	6.16075	6.48361	6.0391	5.88771	6.42349	6.3958	6.29584	5.98212	6.15659	6.54708	6.34467	6.95267	6.68837
SZT2	Q5T011	6.66247	6.3667	6.76067	6.11516	6.18339	6.56995	6.14023	6.33163	6.27599	6.39643	7.01422	6.35846	5.76852	6.38441	5.5642	5.10758
TMEM62	Q0P6H9	6.74212	6.19598	5.87743	7.14674	5.56849	5.82468	6.78534	6.18669	5.74047	6.47418	6.41262	6.17809	6.10699	5.98077	6.14709	6.63232
KSR1	Q8IVT5	6.23508	5.32166	7.25355	5.83765	6.30084	7.11012	6.57164	6.01679	6.73453	6.63034	6.45095	5.85009	6.19533	6.37031	5.75795	5.36318
FKRP	Q9H9S5	5.53722	6.86102	6.27313	6.80221	6.09161	5.89719	6.37637	6.36479	6.28687	6.29644	6.35021	6.49231	6.03194	6.58128	6.53906	7.01835
TXNDC15	Q96J42	7.07153	6.69397	6.64711	7.20571	6.99326	6.86596	6.21833	5.96983	6.42008	6.49346	7.14947	6.27533	5.17455	4.70933	4.79307	5.319
TXNRD3	Q86VQ6	6.01805	6.21493	6.5068	5.8898	5.81	6.64709	6.24455	6.37679	6.78983	6.32205	6.09098	6.47958	5.68315	6.43311	5.92428	6.569
NFU1	Q9UMS0	5.58335	5.11161	3.94069	5.29062	4.99178	4.84047	5.64313	7.17424	5.02862	6.54439	5.63805	7.1944	5.87442	8.8504	8.22165	10.0722
GID4	Q8IVV7	5.82719	6.09044	7.29162	5.98035	6.78214	6.21823	7.01798	6.54794	5.61797	6.36928	6.80644	6.59748	5.73364	5.95497	5.38031	5.784
DOLPP1	Q86YN1	6.43845	5.26446	5.58623	6.62608	5.97554	6.20763	5.77192	5.92497	5.97723	6.23709	6.30355	6.27582	7.10746	6.69261	6.63015	6.98081
ALG8	Q9BVK2	6.17418	5.97954	5.87439	6.41993	6.06478	5.89012	6.61058	6.25657	5.69241	6.46308	5.80056	5.84731	6.50569	6.12339	7.26024	7.03721
ABCD4	O14678	6.72658	6.83579	6.48885	6.87978	6.37575	5.92681	5.96368	6.0668	6.6283	6.57166	6.1556	6.29692	5.48804	5.74625	5.92744	5.92177
MNT	Q99583	7.51485	6.11282	5.63269	5.86056	6.47846	5.29963	6.26428	6.58183	6.5125	6.45089	5.34766	6.65809	6.85653	6.35977	6.07672	5.99273
UBL3	O95164	6.57641	5.97594	6.03234	6.23506	5.63001	6.14443	6.25942	6.68041	5.58306	6.10168	5.69607	6.38096	6.04716	6.78802	6.02407	7.84496
BBS9	Q3SYG4-3	7.46813	6.25528	6.61104	5.57341	5.4787	6.20443	7.23987	6.29245	6.43183	6.50972	6.81201	6.68106	5.75475	5.91121	5.1368	5.6393
HDDC2	Q7Z4H3	6.54195	5.38796	7.00967	5.40723	6.04294	6.49198	5.81588	7.21388	6.25534	6.17939	6.45489	6.87488	5.97708	6.33518	5.68478	6.32698
GOLGA7	Q7Z5G4	6.75063	6.72782	7.04582	6.62234	6.33825	6.37451	6.24197	6.0423	6.73768	6.30255	5.60599	6.25171	5.39291	5.57815	6.05816	5.9292
ARPIN	Q7Z6K5-2	6.51692	6.12371	6.48606	5.99949	6.48421	6.4995	6.27163	6.59811	6.26418	6.28455	6.85536	6.77156	6.17076	6.00724	5.37577	5.29095
SELENON	Q9NZV5	7.3454	7.18144	6.778	7.82077	6.72917	5.82369	6.57193	5.86905	6.34834	5.98193	6.54696	5.98447	5.23765	5.36568	5.46425	5.85127
PAFAH2	Q99487	6.1866	6.31689	6.45487	6.2596	6.51673	6.26311	6.51285	6.28081	6.377	6.27713	5.96279	6.17828	5.93848	5.91265	6.17573	6.38646
DTWD2	Q8NBA8	5.71872	5.57121	7.87365	6.01826	6.56399	6.50069	6.37816	6.12955	6.23851	6.01022	7.22185	7.83668	5.48709	5.24845	5.2295	5.97348
MROH1	Q8NDA8	7.01434	5.2799	5.07879	6.08221	6.99404	6.27097	5.58173	6.53035	7.07838	7.21029	6.93427	6.65617	5.85415	6.35812	6.19613	4.88016
ST3GAL2	Q16842	6.49405	6.49051	5.86277	6.57435	6.87582	6.23537	6.46714	5.71835	6.56205	6.14493	6.03304	5.96342	6.31343	6.14651	5.88721	6.23104
SLC37A4	O43826-2	6.08479	6.23887	6.15239	7.0026	6.32162	5.65983	5.36797	6.39154	6.2918	5.94837	5.80838	5.975	7.15263	6.20312	6.54377	6.85733
TM2D3	Q9BRN9	6.32651	5.32086	5.95365	6.13005	6.12673	6.49633	6.96495	5.54551	6.21676	5.76882	5.94701	6.6884	6.7704	6.10913	6.58948	7.04541
CERCAM	Q5T4B2	7.10638	8.65705	6.1819	6.82572	6.53435	6.30331	6.71858	7.30415	7.04579	6.7501	6.84459	6.86411	3.83195	4.54758	3.9889	4.49554
NDUFA6	P56556	6.1733	6.33254	5.3675	6.01209	7.2729	6.14827	6.46403	6.43657	5.66719	5.78537	5.52937	5.34967	7.11453	6.78447	6.81621	6.74597
ARHGAP39	Q9C0H5-2	6.51603	5.91996	6.88821	7.18572	6.96751	5.58593	6.48456	5.60781	5.94708	6.18352	7.08506	6.14026	5.67775	6.06301	6.17718	5.57038
FBXO5	Q9UKT4	6.71586	6.24159	7.07446	6.17979	6.11669	6.29807	6.26862	6.49818	6.80122	6.65292	7.21124	6.68405	5.11633	5.30717	5.00141	5.83241
DAPK1	P53355-3	6.76188	6.99169	6.44541	6.17486	6.279	6.56666	6.31463	6.14837	6.43041	6.18268	6.02974	6.10792	5.69444	5.85637	5.74958	6.26637
TANK	Q92844	6.21996	4.75274	6.92048	5.07605	7.12472	5.62477	6.10019	6.88542	6.25655	6.52966	6.98653	7.19599	7.24108	5.94382	5.64185	5.50019
ISCA2	Q86U28	5.83335	5.13728	6.43103	5.16974	5.07323	5.61474	5.75308	6.43432	5.6517	6.62707	6.14437	7.36681	5.40441	7.95329	7.71851	7.68707
C15orf40	Q8WUR7	6.12659	5.92592	6.51293	6.03842	6.67575	6.34732	6.24004	6.58292	6.66911	6.36045	6.77996	6.45247	5.82304	5.59094	6.03302	5.8411
NECAP1	Q8NC96	6.49664	5.4429	7.15163	5.79053	6.35361	6.58369	6.58419	6.62806	7.06258	6.37544	6.88943	6.44399	5.88193	5.6584	5.88692	5.67007
UPRT	Q96BW1	6.12588	5.87201	6.61269	6.09658	6.58456	6.51905	5.96878	6.31147	6.18163	5.8981	6.92292	6.37688	6.33538	6.19651	5.58939	6.40818
ZBTB24	O43167	6.4349	6.30711	5.73066	7.07379	6.15882	5.75548	5.73361	5.27623	6.1993	6.76701	5.86318	5.80759	6.65178	6.74667	7.15583	6.33804
PLGRKT	Q9HBL7	5.8675	5.79004	6.5527	5.94782	6.00601	6.69291	6.298	6.39741	5.39412	5.98466	6.11619	6.43688	6.37959	7.09946	7.00465	6.93206
ZC3HAV1L	Q96H79	5.45762	5.94271	6.32017	6.34035	5.79836	6.45325	5.84846	6.43648	5.97578	6.79264	6.62102	6.79367	6.19431	6.69017	6.0577	6.27731
UBXN2B	Q14CS0	6.17372	6.51918	7.42558	6.18968	6.81048	6.36741	5.89861	6.39804	6.28878	6.7813	7.62392	7.06683	5.47037	5.30486	4.44109	5.24014
TSEN34	Q9BSV6	5.9035	6.26273	6.35414	5.63712	5.79871	5.88173	6.25961	6.49121	6.65196	6.37755	6.22022	6.12336	6.70487	6.3336	6.97273	6.02696
TAF1D	Q9H5J8	6.37067	6.17003	6.22964	6.85759	5.6991	5.97692	5.84832	5.20528	5.85268	6.49874	6.62232	6.47385	6.14317	6.35217	6.79939	6.90013
PDRG1	Q9NUG6	6.13446	5.20876	6.51291	5.1807	6.83081	5.48544	5.58747	7.2497	5.46847	7.19581	6.23308	7.69447	5.58386	7.04031	6.01462	6.57912
CNOT7	Q9UIV1	5.99593	5.94832	7.263	6.08933	6.20815	6.21161	6.04489	5.85248	6.55021	6.51971	7.0962	6.81341	6.02812	5.83114	5.68279	5.8647
PDLIM2	Q96JY6-5	6.76838	5.49118	7.29016	5.69321	6.84792	7.00098	5.8967	6.88192	6.24184	6.53509	7.25705	7.24814	5.84002	5.38419	4.56766	5.05556
COX7A2	P14406	5.70303	5.37753	5.93226	4.71549	5.96194	5.82159	6.95466	5.40334	5.94233	5.49307	5.41663	6.12725	6.72194	7.58162	7.8267	9.02059
ZFP62	Q8NB50	5.96144	6.3578	6.06453	7.05994	5.49211	5.72155	6.354	6.18559	5.22601	6.35663	6.09744	6.53066	6.77138	6.40379	6.70356	6.71359
SLC25A51	Q9H1U9	6.13493	6.14368	5.6651	6.64663	6.19332	6.07583	6.70023	5.76644	6.58709	6.06148	5.93038	6.00457	6.60776	6.31339	6.69505	6.47409
NAV2	Q8IVL1	6.47344	5.70621	6.88307	6.43892	6.40074	6.37333	6.77253	5.87606	6.1228	6.48648	6.77058	6.61867	5.36617	6.08566	6.2677	5.35764
NAV3	Q8IVL0	6.2604	6.36981	6.60146	6.41658	6.53852	7.3905	6.29072	6.56928	5.83475	6.54479	6.78459	5.83043	6.256	5.63126	5.96825	5.61267
SLC19A1	P41440	7.30423	6.37594	7.00822	7.33236	5.7688	6.61602	6.79744	6.75561	5.73724	5.79648	6.26434	6.73582	5.68634	4.76685	5.43944	5.60486
PBLD	P30039	6.93331	6.29691	6.19672	6.17821	6.21989	6.71462	6.42315	6.9874	6.39015	6.62392	6.01763	6.89729	5.65499	5.84538	5.29104	5.32939
CD58	P19256	5.7319	6.80432	6.07452	6.0219	6.38162	6.71341	5.59254	6.3435	6.79821	6.01683	6.16101	6.38893	7.02219	6.02427	6.85554	5.06931
FXN	Q16595	5.81829	5.07262	4.79752	5.34653	5.57962	3.98427	5.7421	6.53157	4.71069	5.73599	5.1302	5.6527	5.11371	10.9247	8.68753	11.172
CEP164	Q9UPV0	6.69031	7.51977	8.42815	7.80969	6.24184	5.94492	6.11241	6.7344	7.40661	7.60502	6.76336	6.95894	3.85992	4.0208	3.69056	4.21331
DNAJB5	O75953-5	6.00504	6.36954	6.92269	5.97672	6.04047	6.01798	6.33499	6.67084	6.55038	7.2801	6.93208	6.73861	5.14807	5.74107	5.46433	5.8071
RSRC2	Q7L4I2	5.37948	5.72859	5.82563	5.45319	5.80134	6.16459	5.53094	5.51794	5.50909	6.3177	6.4263	5.84978	7.25574	6.92321	8.00021	8.31629
LAMTOR4	Q0VGL1	6.10549	6.45797	6.26409	6.59075	6.55328	6.31524	6.42691	6.23773	6.14804	6.70316	6.4515	6.41957	5.79992	5.71951	5.81767	5.98918
LY75	O60449-2	5.55151	7.04059	6.11439	6.5858	6.73851	6.75047	5.80574	6.5696	6.31119	6.84418	6.69537	6.43596	5.25239	6.41619	5.21606	5.67206
MORC4	Q8TE76	6.75929	7.06139	5.77683	6.53438	6.21765	6.61096	6.66402	6.86929	6.76019	6.40152	6.7021	5.77203	4.93275	5.64203	5.18767	6.1079
FAM8A1	Q9UBU6	6.75114	6.81315	6.3524	6.55683	6.10018	6.04284	6.1823	6.21299	5.73666	6.36596	6.23008	6.4934	5.82869	5.92195	6.24779	6.16364
GINS3	Q9BRX5-3	5.86068	4.29027	5.75444	4.66117	4.88704	5.27687	4.83022	6.11409	5.38836	6.77245	6.03488	7.86464	6.13559	8.5175	8.93443	8.67738
CAMKK2	Q96RR4	6.45751	6.18941	6.71354	5.48989	6.10883	6.21211	6.01429	6.97551	6.3419	6.68578	7.24411	7.05479	5.60456	6.05634	5.80333	5.0481
INO80E	Q8NBZ0	6.27019	6.53585	6.71981	6.88447	5.64665	5.30701	6.23637	6.60564	6.76404	6.54182	6.66294	6.42704	6.29732	5.74671	6.13952	5.43462
NAT14	Q8WUY8	6.25551	5.87351	6.0384	6.54771	6.34669	6.14211	6.17515	5.60306	5.99187	6.28112	5.9648	5.89788	7.04726	6.77124	6.40944	6.65427
NEK3	P51956	6.92369	6.57206	6.73197	5.92975	6.64493	6.97385	6.71255	6.95897	6.60717	6.96557	6.46504	6.93566	4.83147	5.1076	4.83315	4.80659
FAAP24	Q9BTP7	6.631	6.88557	7.18585	7.1898	5.63126	6.36366	6.40725	6.57845	6.83686	7.2372	7.6477	7.23461	4.74269	4.81538	4.22103	4.39169
GRIP1	Q9Y3R0	6.60092	6.29845	6.72822	6.64346	5.78763	6.09417	6.36161	6.45835	5.88346	6.31134	6.25603	6.5235	6.01125	5.7839	6.20369	6.05403
ZBTB48	P10074	6.28304	6.4191	6.42538	6.96677	6.1816	6.61258	6.49896	5.76	6.12818	6.19684	6.76048	6.36795	6.06665	5.57989	5.64803	6.10453
TCEAL5	Q5H9L2	6.16385	5.73307	6.86349	5.65091	6.1031	6.2011	5.45458	6.69188	5.99596	6.54987	6.83791	6.39274	6.69245	6.77642	6.02023	5.87245
MFSD11	O43934	6.58015	6.86957	6.3593	6.59432	5.78691	6.10654	6.74674	6.25556	6.23286	6.30255	6.6691	6.00216	5.97465	5.85412	5.60364	6.06183
RUNX2	Q13950	6.4691	6.07046	6.19385	6.62241	6.94609	6.08401	5.73849	5.66353	6.45695	6.42201	6.26335	6.2016	6.9862	6.17313	5.952	5.75683
MRPS12	O15235	6.03038	6.76588	6.04246	6.5784	5.95015	6.3367	6.80123	6.04682	5.80306	6.07571	5.62841	6.10274	6.47265	6.44482	6.28655	6.63404

MRPS24	Q96EL2	6.24389	6.81559	5.70889	6.84817	6.03828	6.24543	6.33115	6.46921	6.00527	6.40925	5.54756	5.76237	6.35378	6.24049	6.6377	6.34296
ASPHD1	Q5U4P2	6.1212	6.63746	6.16202	7.19275	6.08538	6.56376	6.87067	5.673	6.57693	6.23233	6.32489	6.35605	5.67315	6.16403	5.95315	5.61754
ITGB1BP1	O14713	6.7022	6.77322	6.63745	6.76096	5.94882	6.37045	6.1276	6.58453	8.04335	6.91546	7.65413	7.26581	4.48114	4.85404	4.36336	4.51749
HAUS2	Q9NVX0	6.14452	5.53419	6.99089	5.47776	6.43976	6.32212	6.40903	6.42178	5.86687	6.43694	6.22781	6.93988	5.67682	6.59691	6.02015	6.49458
UBE2H	P62256	5.90279	6.29711	5.37336	5.82681	6.67509	6.72375	6.42324	6.83238	6.09673	6.44814	7.27561	6.86409	6.10647	5.91052	5.17461	6.0693
TINF2	Q9BSI4	6.02478	6.271	5.23167	5.70954	6.39849	5.69369	6.48209	6.36088	6.03082	6.11039	5.51674	6.06136	6.56924	6.54499	7.67534	7.31895
MCOLN1	Q9GZU1	6.51233	6.9751	7.03215	6.35449	5.9153	6.16618	5.56804	6.54332	6.72158	6.92123	6.15946	6.12198	5.38062	5.2944	5.88551	6.44831
PTPN3	P26045	6.40602	6.58648	6.37023	6.32883	6.51114	5.92589	6.37213	6.51484	6.49035	6.71969	6.56044	6.3156	5.50001	5.62495	5.9245	5.84889
GPBP1	Q86WP2-2	7.06232	6.77728	7.60361	6.44536	6.32724	6.71913	6.62821	5.98899	6.58626	6.65333	6.71447	6.71182	5.17828	4.8653	4.68884	5.04954
PLP2	Q04941	6.76906	6.53936	6.38133	6.32311	5.90944	6.31087	6.81345	6.09199	6.19572	6.93161	7.13778	6.58638	5.34278	5.58377	5.51383	5.56951
SLC7A1	P30825	6.20027	6.34788	6.23004	6.84756	6.22576	6.30434	6.29216	5.66545	5.86818	6.07645	6.67299	6.36718	5.88087	5.91696	6.43755	6.66634
FAM102B	Q5T8I3	5.5623	6.29297	6.48028	6.3037	6.10574	6.19283	6.15716	5.75531	6.82125	6.39993	6.26408	6.53513	6.2828	6.63339	6.09426	6.11885
PRR14	Q9BWN1	6.62746	6.20055	5.75278	6.97829	5.97606	6.03265	6.13601	6.00626	6.42124	5.78096	6.07205	6.1235	6.27039	6.09177	6.78926	6.74077
EVI5L	Q96CN4-2	7.33093	5.77522	6.94232	6.64636	6.37253	6.85381	6.66753	6.83437	5.72339	6.23571	6.59154	6.49791	5.44385	5.69458	4.93979	5.45016
KBTBD4	Q9NVX7-2	5.69447	5.68472	6.69654	5.46085	5.51854	6.16684	5.93071	5.64595	5.89303	6.33768	6.13253	6.11853	6.78427	7.58061	7.47801	6.87674
PDE3B	Q13370	6.91576	6.59661	6.33898	6.97583	6.44736	6.18519	6.34277	5.90813	6.09477	5.90519	6.06202	6.08175	6.31001	5.74138	6.24702	5.84724
KMT5A	Q9NQR1	6.78687	6.26395	6.6179	6.79373	6.24753	6.35914	6.67792	6.77033	6.36057	6.84272	6.76424	6.44747	4.64414	5.49225	5.2204	5.71086
GORAB	Q5T7V8	6.72056	6.31739	6.43151	6.34219	5.77413	5.71811	6.1864	6.72772	5.80927	6.26568	6.24552	6.64952	6.7692	6.15104	5.80698	6.08478
TOMM5	Q8N4H5-2	5.43399	5.37497	5.16403	6.25463	6.33434	4.758	5.52326	4.68859	4.69274	4.28141	4.17654	4.95581	5.55936	11.1652	9.12229	12.5148
ACP1	P24666	6.14505	5.51667	6.71549	5.06509	6.6064	6.28532	6.24697	7.28255	5.56899	6.63282	6.63361	6.97321	5.91217	6.04427	5.88257	6.48881
RRAD	P55042	6.81354	6.50665	6.85584	5.50848	6.22571	6.1706	6.74838	6.6032	7.01132	6.60443	7.02268	6.12808	5.56651	5.50626	5.24078	5.48752
GPR39	O43194	6.31773	6.10279	5.82589	6.75852	6.26303	5.97531	5.9162	5.62671	6.44952	6.19276	6.19389	5.47023	7.14807	6.66816	6.90156	6.18962
DENND11	A4D1U4	6.78218	7.29563	6.67881	6.74547	6.05394	6.10279	6.00955	5.92733	6.42561	6.16039	6.71891	6.74195	5.36527	5.72144	5.70316	5.56756
SMIM19	Q96E16	6.42436	5.77797	6.37058	6.73672	6.0067	6.36533	6.81989	6.01711	6.38013	6.15877	6.45555	6.17889	5.81178	6.42637	6.23488	5.83499
FICD	Q9BVA6	6.39234	6.68591	6.51331	6.43987	6.10582	6.03443	6.54041	5.58112	6.72268	6.95675	5.89857	6.08113	5.93233	5.49715	6.10747	6.51071
PSMB10	P40306	6.23042	6.31986	6.17873	5.78571	6.26352	6.39721	6.01848	6.74762	6.39272	6.20415	6.00284	6.24169	6.57775	6.20535	6.24221	6.19172
CPE	P16870	6.38034	6.12129	6.2071	6.35236	6.6763	6.25036	6.06119	6.30246	6.31835	6.28493	7.05857	6.61568	5.83778	6.09474	5.46156	5.97699
XPA	P23025	5.63502	5.47565	6.22513	5.76397	6.32212	6.55709	5.78665	5.89432	6.23515	6.26674	6.1249	6.23941	6.50179	7.29373	6.90853	6.7698
PCMTD2	Q9NV79	5.80784	6.18251	5.59662	5.04048	5.84511	5.86173	6.0872	6.23334	6.82569	6.32108	6.61001	6.30926	6.77339	7.16502	6.15948	7.18125
C9orf72	Q96LT7	6.24601	5.39268	6.25276	6.1238	6.0521	6.55164	6.81663	6.89281	6.47041	5.36744	6.34381	6.62441	5.98615	6.112	6.40369	6.36367
CARD19	Q96LW7-2	6.51048	5.4664	5.70501	6.09343	6.39258	5.61129	6.00847	6.09161	5.53855	6.4843	6.12506	6.50667	7.20972	6.79539	6.82822	6.6328
AMZ2	Q86W34	6.03066	6.10808	6.41298	5.99297	6.32123	6.47551	6.22709	6.0965	6.85031	6.25045	6.33537	6.53488	6.28648	6.2445	5.78676	6.04624
ELF2	Q15723	6.30235	6.42913	5.71702	6.64071	6.68109	5.89853	6.64433	5.54344	6.50336	6.0839	6.67596	5.99668	6.4812	5.90684	6.08569	6.40977
ODF2L	Q9ULJ1-4	6.55483	7.29412	7.00193	7.33342	6.4934	6.70314	6.68774	6.70807	6.66919	6.55011	7.14087	7.56266	4.19884	4.41056	4.30791	4.38319
POC1B	Q8TC44	6.36754	6.03602	6.3053	6.54018	5.9235	6.30125	5.9549	6.31133	6.63954	6.61858	6.77473	6.73957	5.76209	6.01379	6.02251	5.68915
BRD9	Q9H8M2	6.10941	6.44472	6.00585	6.93879	6.13288	6.32783	6.6748	5.9667	5.97775	6.2969	5.93305	6.48923	5.59922	6.27975	6.19563	6.62751
WRAP73	Q9P2S5	6.3081	6.86635	7.12308	6.72707	6.01987	6.56096	6.65287	6.9486	6.8073	6.52427	6.83906	7.68068	4.66852	4.42589	5.02954	4.81784
MRPS14	O60783	6.13592	6.11511	5.48891	6.07169	5.98005	5.87297	6.60218	6.59694	6.30885	5.87691	6.07593	5.88098	7.50555	6.7628	6.77328	6.85194
DGKZ	Q13574-1	6.25785	6.02329	6.8406	6.56924	5.89916	6.28373	6.33281	6.63347	6.49332	6.36793	6.58587	6.72162	5.83048	5.67427	5.72765	5.75872
REEP6	Q96HR9	6.11761	5.1546	5.71503	5.21298	5.74895	5.83889	5.66111	5.80472	4.90833	6.05545	5.95007	6.6319	6.32469	8.2538	8.07052	8.55134
SMAP	O00193	5.79702	5.77992	6.00112	5.45333	6.12425	6.33673	5.60424	6.68655	5.88211	6.31785	6.55394	6.13274	7.03275	6.77679	6.79606	6.72461
SESN1	Q9Y6P5-2	6.25614	6.40149	7.31722	5.97296	6.45029	6.91454	6.19083	6.49794	5.95067	5.98117	7.15644	7.01231	5.37419	5.93038	5.26895	5.32448
ITIH2	P19823	5.69126	7.08149	4.85036	5.26066	4.493	6.16833	6.79733	5.0273	6.9651	5.26416	6.02137	5.83506	6.58744	5.96826	10.1082	7.88067
MDFIC	Q9P1T7-1	6.7552	6.6619	6.53678	6.52094	5.85443	6.31509	6.48716	6.00239	6.35002	6.24848	6.19258	6.20412	6.25055	5.8761	5.9188	5.82544
ZBTB20	Q9HC78	6.6913	6.55393	5.89878	6.56663	5.65301	5.83241	6.47114	5.84169	6.04042	6.58166	5.49948	5.86645	6.7989	6.30844	7.062	7.05375
SLC35B3	Q9H1N7	6.11811	6.84833	5.5972	6.55377	6.11777	5.94904	6.54807	5.9631	6.1421	6.35544	6.20529	6.16355	6.1204	6.23436	6.82921	6.25427
TSPYL2	Q9H2G4	7.07765	6.36355	5.85639	7.59458	6.62911	6.80597	6.9004	6.17306	6.43677	6.62495	6.41624	5.58508	6.20089	5.10884	4.88011	5.34642
LYST	Q99698	6.44143	7.01155	6.46684	6.1992	5.94787	6.38152	6.00875	6.04134	6.70875	7.07643	7.04033	7.04177	5.52414	5.44022	5.2669	5.40295
KDM4C	Q9H3R0	5.47775	6.21757	6.45015	6.05158	6.21883	6.71385	6.49295	5.98154	5.78163	6.13867	6.22149	6.65907	6.44974	6.18568	6.13565	6.82383
JMY	Q8N9B5	7.44354	6.17401	7.15317	6.03819	5.82465	6.10931	5.80867	6.18097	6.13738	6.1062	5.50932	5.46054	6.76222	5.7576	7.04031	6.49393
COQ10B	Q9H8M1	6.92995	6.29708	5.10293	6.82656	6.95307	5.94596	6.28547	6.41714	6.72275	6.57267	6.45016	5.53352	6.10368	5.57902	5.74274	6.53729
C4A	P0C0L4	6.41198	6.54846	5.38039	6.60875	5.77459	5.96271	6.22475	5.7489	6.12218	5.98862	6.64777	6.64317	6.36398	5.67024	7.18267	6.72084
MAF1	Q9H063	4.57792	4.63498	4.96139	4.47134	5.41684	5.15481	5.86921	5.64719	4.85301	4.96492	4.8217	4.61258	10.2812	10.4491	10.1065	9.17723
TNKS	O95271	7.05332	6.61447	7.34765	6.71616	6.72214	6.71503	6.81836	6.24782	6.87188	6.35177	7.17715	7.19699	4.78048	4.56455	4.27667	4.54555
DCAF11	Q8TEB1	5.42503	5.81331	6.5288	6.02624	6.95902	6.43374	6.10962	6.26533	6.48432	5.83928	6.16254	5.48025	6.96725	6.27216	6.93087	6.30223
KIAA1586	Q9HCI6	6.6866	6.9559	6.96644	6.7609	6.15708	6.39926	6.76044	6.62273	6.75699	7.4749	7.3088	6.71212	4.75282	4.70626	4.50431	4.47444
RAB28	P51157	6.32252	5.88386	6.74945	5.95885	6.50211	6.62418	6.35889	6.46382	6.66192	6.33151	6.62917	6.78917	5.48054	5.57397	5.74181	5.92821
ZNF414	Q96IQ9-2	5.62749	5.80006	5.60875	5.80504	6.47744	6.42831	5.99745	6.44496	6.05281	7.13161	6.43962	6.36544	6.69985	6.43742	6.41435	6.2694
RPS29	P62273-2	5.86323	6.68098	6.52772	5.41179	6.32665	7.55396	6.01911	6.22721	7.03766	7.09062	6.87456	6.50515	5.88789	5.65174	5.23426	5.10745
ADCY3	O60266	6.85853	6.33056	6.85105	6.44419	5.61088	7.01342	6.58473	6.3822	6.29576	5.87093	6.8562	6.073	5.55207	6.00125	6.24614	5.02908
RTKN	Q9BST9-2	6.39231	6.38274	6.90815	6.19938	6.77428	6.90376	6.1887	6.15401	6.80384	6.69278	6.6246	6.555	5.46855	5.43645	5.34961	5.16584
UMAD1	C9J7I0	5.39265	4.53714	4.86284	4.724	5.37298	4.48543	5.47895	6.54039	4.23382	4.78002	5.28611	5.69827	5.92917	12.5685	7.68898	12.4207
CUTA	O60888-2	6.81518	4.86134	5.55263	4.9753	6.40033	5.83617	5.25811	8.30251	5.12271	6.30548	5.71586	5.44829	4.5136	9.30104	7.18511	8.40633
ARHGAP33	O14559	7.65606	7.03057	6.67332	7.23135	6.5858	6.15642	6.64513	6.69361	6.69403	5.87531	6.90083	6.29436	4.89362	4.93579	4.85593	4.87787
C3orf33	Q6P1S2	5.694	6.23554	6.48967	6.75281	5.51437	6.19619	6.7771	6.35556	5.75839	7.42045	6.10613	5.66097	6.30926	5.31958	6.97477	6.43519
HIKESHI	Q53FT3	6.68322	5.92088	6.68694	5.19376	6.31266	6.18919	6.61736	6.97404	6.00346	6.23479	6.52557	6.44224	5.52183	6.5492	6.25017	5.8947
ZNF428	Q96B54	6.56803	6.26385	6.92249	5.92299	6.52488	6.45941	5.97704	6.00923	6.01174	6.68437	7.35899	6.37808	6.39707	5.86386	5.37373	5.28422
CDC26	Q8NHZ8	5.93704	5.43537	5.79077	5.36121	5.75402	5.21209	4.54265	6.56893	4.0372	6.81288	5.38575	7.05677	5.73754	9.64775	7.24019	9.47985
PIAS4	Q8N2W9	5.37792	6.11716	6.97767	5.94549	5.98381	6.88531	6.79503	5.71223	6.45726	6.82492	6.88971	6.87982	5.62726	5.67863	5.83041	6.01736
C1D	Q13901	6.25036	6.23413	6.08563	6.68288	5.88089	6.01399	6.74298	5.70467	6.5063	5.82331	6.77353	6.09215	6.73483	5.83604	6.47094	6.16739
CCHCR1	Q8TD31-2	6.37297	6.6113	6.54326	6.40154	6.9085	6.74667	6.35192	6.28493	6.51644	6.71771	7.25961	6.89074	5.11616	5.28068	4.84784	5.14974
RWDD2A	Q9UIY3	6.26613	6.36096	7.11295	5.73507	6.6592	6.94975	6.37118	6.92899	7.16137	7.19046	6.55322	6.90672	4.68261	5.55346	4.70114	4.86677
SELENOO	Q9BVL4	6.12599	6.75225	6.99073	5.92844	6.05577	7.05305	6.57623	5.48802	5.53021	6.33571	7.05196	6.26295	5.94359	6.57656	6.01075	5.31779
IMMP2L	Q96T52	5.60494	6.76178	5.62099	6.77312	6.97213	6.75528	6.8628	6.11546	5.83881	5.48859	6.60102	6.49932	6.21161	4.76977	6.60647	6.51794
CEP95	Q96GE4	6.09926	7.02355	6.65149	6.90498	6.51243	6.71312	6.1729	6.57577	6.42862	6.75699	6.49139	6.52428	4.9127	5.41743	5.4057	5.4094
TRIM36	Q9NQ86	6.91617	6.44085	6.4316	6.12554	6.31372	6.37881	6.04757	6.49785	6.10701	6.41667	6.68098	6.82696	5.41057	5.72223	5.64256	6.0409
CRTC3	Q6UUV7	6.13698	5.14467	6.97901	5.03642	6.17682	5.92791	5.44871	6.40792	6.91579	5.76416	6.41446	6.61829	7.76559	6.68639	5.92129	6.6556
TMEM126B	Q8IUX1	6.48769	6.63848	6.46024	6.8117	6.24598	5.80648	6.36224	5.8931	5.93095	6.19204	6.00172	5.94033	6.34542	6.19255	6.41903	6.27205
ASB1	Q9Y576	6.10526	6.00067	6.66758	6.4028	6.07644	6.61536	6.28927	6.88042	6.18596	5.92788	6.19358	6.47914	5.89018	5.70559	6.12104	6.45884
NRM	Q8IXM6	6.56518	6.71798	5.35242	5.89945	6.05066	6.1631	5.93604	6.54043	6.05074	5.5449	5.72243	5.79358	6.29512	7.05172	7.00542	7.31084
ARSK	Q6UWY0	6.00379	6.1451	5.52987	5.99161	6.47227	6.19271	6.20178	6.59065	6.23091	6.35456	6.53993	6.52738	6.41345	6.24341	6.26515	6.29743
HBD	P02042	7.45788	5.7221	4.01406	5.48446	4.64208	5.5092	8.11355	5.93899	5.82515	5.47437	5.36408	5.88046	6.22036	5.93419	9.14493	9.27414
TBC1D32	Q96NH3-4	6.32223	6.96339	7.46063	6.71908	6.78959	7.29673	6.28115	6.48893	6.73809	6.89226	6.55614	6.81812	4.48315	5.4227	3.85343	4.91439
NPRL2	Q8WTW4	6.43919	6.30298	6.63077	6.36959	5.90881	6.03655	5.76559	6.46325	6.46554	6.6026	6.9774	7.13526	5.85069	5.84807	5.57822	5.6255
TMTC2	Q8N394	6.24198	7.02049	5.42301	6.40995	6.63767	6.34617	6.06405	6.04672	6.01817	6.31945	5.97157	6.01755	6.89811	6.20712	6.4931	5.8849
TEX30	Q5JUR7	6.3107	6.01109	6.73019	6.2141	6.50832	6.81944	6.28733	6.13144	6.62962	6.95582	6.8092	6.70647	5.42726	5.67757	5.08814	5.69334
TRDMT1	O14717	6.6117	6.43276	6.68526	5.9155	6.23503	6.69165	6.04115	7.28957	6.43467	6.59624	6.51575	6.89163	5.51646	5.35925	5.34725	5.43614
NAGLU	P54802	5.97817	6.45247	5.87121	6.71092	6.76727	6.21465	6.79465	6.20804	5.91421	5.75333	6.31622	6.31482	5.88496	6.06036	6.4145	6.34423
CDK10	Q15131	5.76241	5.97793	5.58526	5.7493	6.11271	6.27664	6.33062	5.8435	6.01584	6.20801	5.77173	6.11306	7.27349	6.83736	7.15673	6.9854
TLCD1	Q96CP7	6.3507	5.98422	5.79182	6.76745	5.71996	6.02178	6.36558	6.04554	6.05309	6.09983	6.32384	5.92846	6.32415	6.55563	6.6932	6.97477
B4GALT3	O60512	6.56185	6.67583	6.4177	7.63141	6.16608	5.84144	6.12898	6.55938	6.0112	6.29584	6.56044	6.30742	6.2177	5.62128	5.32171	5.68174
SLC5A6	Q9Y289	6.16373	6.24237	6.57842	6.62427	5.94027	5.9028	6.09241	6.40862	6.3775	6.06701	6.53005	6.26261	5.79531	6.09666	6.14616	6.77181
SMAP1	Q8IYB5-2	6.12783	6.47591	6.94651	5.89313	5.71124	6.34144	6.60681	6.42194	6.57773	6.34534	6.29279	6.43534	6.18376	6.11535	5.52741	5.99746
KCTD2	Q14681	6.48936	5.51478	6.72613	6.46048	6.48121	6.7539	6.19905	6.14988	6.3573	6.56592	7.10083	6.74499	5.96009	5.3648	5.14823	5.98306
TTC9C	Q8N5M4	4.97706	5.42194	6.29697	4.99089	6.60212	5.83788	5.87716	7.48241	6.11559	7.59117	6.49337	6.85718	6.10498	6.07253	6.59224	6.6865
TAF5L	O75529	6.53085	6.62297	6.38571	5.72251	6.25761	6.08698	6.01977	6.44732	5.93585	5.57	6.48794	6.48153	6.52712	6.45792	6.41862	6.04731
CREB3L1	Q96BA8	7.38337	6.78969	6.2744	7.09709	6.57102	6.18547	6.47232	6.38944	6.25528	7.36278	7.01824	7.20618	4.10276	5.34086	4.65632	4.8948
RXYLT1	Q9Y2B1	6.50475	6.80344	6.55089	7.27293	5.78459	5.94255	6.50333	5.76514	5.87654	6.33554	6.05411	6.59093	5.86936	5.90651	5.93317	6.30624
MSANTD4	Q8NCY6	6.81664	7.20079	6.80505	7.01026	6.51727	6.34549	6.69814	6.49218	6.72272	6.46084	5.9691	5.71644	5.61373	4.97072	5.28349	5.37716
AMACR	Q9UHK6-5	6.50855	5.27589	6.14891	6.48726	6.24385	5.98687	5.70041	5.8368	7.02639	6.77706	7.08805	6.91642	5.75339	5.91313	5.84346	6.49359
ZDHHC5	Q9C0B5	6.29984	6.33515	5.44437	6.41526	6.46784	6.10531	6.44134	6.04902	6.25577	6.06314	5.84855	5.61939	6.38878	6.62547	6.71257	6.92819
TAF12	Q16514	6.04436	6.48286	6.1772	6.31157	5.95234	6.31497	6.41986	6.46779	6.19876	6.26751	6.6097	7.06043	6.04267	5.75424	5.82247	6.07329
CNEP1R1	Q8N9A8-2	6.72532	5.80837	6.3714	7.22946	5.48145	6.67851	5.9177	5.32265	6.88122	6.60849	5.78075	5.9814	6.10125	5.45839	6.69231	6.96133
PRXL2C	Q7RTV5	6.67971	6.65756	6.86702	7.07668	5.87415	6.62284	6.33693	6.78957	6.75387	6.4637	7.42407	7.34806	4.46637	5.0075	4.55871	5.07328
MAD2L1BP	Q15013-3	6.01434	6.30494	6.52503	6.25138	6.0003	6.17371	5.94764	6.5735	6.29059	6.97277	6.48324	6.80442	6.25232	5.86166	5.70847	5.83567

GET1	O00258	6.22148	6.14868	6.58613	6.28922	5.53586	6.01164	5.72877	5.95699	6.43475	6.7344	5.99864	6.58643	6.56157	6.43686	5.93963	6.82893
NOCT	Q9UK39	5.60443	6.41043	6.54231	5.09694	6.18874	6.22971	6.81749	6.0604	5.83516	6.01124	6.27168	7.35217	6.53659	6.02525	6.71027	6.30719
CDR2	Q01850	6.74657	6.27433	5.91472	6.35905	6.51354	6.23675	6.0746	6.2902	6.24484	5.97097	6.11194	7.15254	6.28742	5.71811	6.05646	6.04797
MRFAP1	Q9Y605	7.26074	6.14252	7.62474	5.63649	6.67429	5.60437	5.0544	7.61549	6.00046	8.05349	7.20012	7.45622	3.5876	6.25459	4.76582	5.06867
ATP5MK	Q96IX5	6.17702	6.05107	6.48632	7.47333	6.01574	6.39101	6.31267	6.3895	6.58828	6.56718	6.07345	6.45021	5.31835	5.99034	5.95125	5.76426
CRTC2	Q53ET0	6.69026	6.00759	6.89571	5.84235	7.45346	6.63862	5.68021	6.02149	5.6238	6.40127	6.90164	6.88549	6.00883	5.97906	5.53612	5.43409
FAM241A	Q8N8J7	6.63254	6.13068	5.76746	5.61368	6.19508	6.01558	6.5908	6.65581	5.44513	6.45165	5.05709	5.73198	7.56618	6.61323	7.29472	6.23837
SF3B5	Q9BWJ5	5.79756	4.43685	5.10446	5.22353	5.96077	4.94738	5.1963	5.61236	5.24522	5.73633	4.94364	5.06134	5.34759	10.427	9.16076	11.7989
IL10RB	Q08334	6.59913	7.4795	7.42056	7.16807	6.24843	6.2438	6.76515	6.4748	6.6294	6.88627	7.23117	7.10401	4.19049	4.62441	4.27258	4.66224
XAGE1A	Q9HD64	6.72905	6.7053	7.18435	6.89587	6.40855	6.13423	6.75117	7.16128	7.29176	6.62101	7.09109	6.50355	4.98831	4.78564	4.2283	4.52053
PIGBOS1	A0A0B4J2F0	6.36905	6.28389	6.03151	6.25411	5.73424	5.62337	6.05119	6.36646	4.88861	5.82623	5.63485	6.02121	5.65635	8.78443	6.84451	7.62998
TMEM59	Q9BXS4	7.12148	7.49678	6.69692	7.74319	6.14194	5.7514	6.35701	6.13756	6.79562	6.81213	7.19177	6.92846	4.42324	4.93235	4.46408	5.00605
HECA	Q9UBI9	6.1945	6.43916	6.42217	5.81401	5.99114	6.25709	6.2553	6.20607	6.44	6.58831	6.75846	6.40207	5.90611	6.14483	5.90826	6.27251
ZDHHC15	Q96MV8	6.11169	6.29494	6.03627	6.66969	5.9097	6.34895	6.23915	5.90631	5.98212	6.60313	6.36698	6.50259	6.03564	6.30609	6.25784	6.42891
TMEM167A	Q8TBQ9	5.70021	6.74085	5.81033	6.5557	5.80616	6.41927	5.86099	6.4645	6.52446	5.99268	6.16948	6.26087	6.40998	6.33136	6.64506	6.30808
FUT10	Q6P4F1-2	6.11574	7.08297	5.91036	6.98524	5.93248	5.94553	5.9982	5.86929	6.16857	6.3397	5.97479	5.82823	6.39689	5.89026	7.10199	6.45977
SCMH1	Q96GD3	6.21516	6.82318	6.29426	6.8201	6.35444	6.42473	6.74775	5.9017	5.94213	6.56045	6.04999	6.05956	6.15834	5.85982	6.33641	6.35197
C12orf4	Q9NQ89	5.69601	5.99113	6.83649	6.13672	6.7517	6.66315	6.64024	6.16266	6.37269	6.72526	6.5789	6.86802	5.51131	5.9034	5.94226	5.22004
CD63	P08962	6.41463	6.46873	6.72192	6.46393	5.40219	5.87494	6.53358	5.62435	6.48559	6.98711	6.92437	6.60378	5.76972	5.90132	6.30524	5.5186
COL1A2	P08123	5.99559	6.36555	5.28227	6.12822	4.16246	4.46436	4.74235	4.89516	6.01368	6.32849	6.78917	6.23049	8.38435	8.07834	8.90945	7.23008
DCUN1D4	Q92564-3	5.46995	5.71217	6.2799	5.33907	5.45909	6.47063	5.57315	6.03214	5.94395	6.34176	6.33598	6.14521	7.0615	7.73889	7.28268	6.81393
TFDP2	Q14188	5.98075	6.4514	6.42264	6.38631	6.05756	6.09096	5.91298	6.27206	6.31746	6.22498	6.84072	5.52121	7.01486	6.50251	6.16352	5.8401
ZNF410	Q86VK4-5	5.91191	5.79508	6.49167	6.0544	5.83132	6.14821	5.96738	5.96338	6.33307	6.51841	6.45744	6.56145	5.7074	6.49032	6.90378	6.86479
MCRIP1	C9JLW8	6.53909	4.75745	6.46305	5.03978	6.48346	5.30351	4.97292	6.9537	5.50939	6.83518	5.67889	7.26498	5.9659	7.79064	6.60139	7.84068
ISG15	P05161	5.5249	4.81256	5.87162	5.14938	6.16493	6.12295	6.12886	6.33858	7.13819	7.15506	6.90096	6.93908	5.80069	6.08717	6.5912	7.27388
FUCA1	P04066	6.12765	5.73902	5.77895	6.35002	6.1415	6.0893	5.92033	5.8696	6.37109	6.21763	5.57437	5.9382	6.77863	6.71988	7.04351	7.34032
ZCCHC17	Q9NP64	6.18042	5.97842	5.74741	6.36629	5.7106	5.81096	6.16186	5.6636	6.01711	5.994	5.81731	5.99345	6.46114	7.32141	7.14389	7.63213
CAAP1	Q9H8G2	6.39778	6.27638	5.75831	6.6998	6.22259	5.70105	5.71159	5.75603	6.42691	6.24458	6.08872	6.10709	6.58286	6.47895	6.52584	7.02152
ATP5F1EP2	Q5VTU8	6.60512	6.73084	5.48178	7.03429	5.9617	5.51368	6.40908	6.37813	6.79835	6.16023	6.4459	6.2159	6.41986	5.5345	6.18642	6.12421
NID1	P14543	6.37785	8.01696	6.71774	6.62876	6.42437	5.91189	6.43155	6.62802	6.25202	7.50284	7.19645	6.13042	4.41836	4.72614	5.06176	5.57484
GAGE12F	P0CL80	5.2612	4.38359	4.09454	4.93913	3.81163	2.35217	4.92276	4.47657	3.64761	2.70507	3.37566	3.88248	2.98243	19.6807	10.4321	19.0524
HINT3	Q9NQE9	5.87807	5.45239	6.19716	5.16569	5.85595	5.66335	5.8548	7.24896	6.27538	6.44938	5.80191	6.55963	6.52915	7.11506	7.14389	6.80921
FAM110A	Q9BQ89	5.94121	6.95534	7.74999	6.5142	6.3065	6.32336	6.42761	6.46297	6.34962	6.66335	7.16779	7.01714	5.05512	5.30219	4.9553	4.80832
TAX1BP3	O14907	6.04277	4.52032	6.2019	5.08213	6.63468	5.76825	5.81666	6.71044	5.62319	6.94628	6.23449	7.09175	5.41842	7.47425	6.60581	7.82866
CKS1B	P61024	5.24206	5.12301	6.43414	5.49313	6.66797	5.99673	5.92782	7.96582	5.78022	7.61855	7.20022	7.44282	4.37848	6.25375	5.73942	6.73585
POP5	Q969H6	5.90277	6.44047	6.37895	6.40789	6.25137	6.42817	6.18718	5.76216	5.47682	6.60541	6.13495	5.95201	6.32579	6.30874	6.9696	6.46772
TUBE1	Q9UJT0	6.41487	6.01308	7.19487	6.15974	6.95493	6.52258	6.24391	6.29211	6.48634	6.8225	6.72444	6.5028	4.95506	5.56153	5.33485	5.81636
ICMT	O60725	6.1706	6.03859	5.80009	6.97936	5.72511	6.05104	6.25994	6.1404	6.10782	6.17602	6.2884	6.25727	6.58285	6.32183	6.47133	6.62933
ADCY9	O60503	5.73187	6.8829	6.95006	7.23449	6.4394	6.55587	5.63637	6.14935	6.1612	5.8935	5.83882	6.64227	6.0449	5.54909	6.08055	6.20937
IPP	Q9Y573	6.4465	6.23159	6.68971	5.8223	6.37688	6.79425	6.07694	6.66127	6.81381	7.03001	6.5513	6.38252	5.53426	5.85561	5.47136	5.2617
BDH2	Q9BUT1	6.12777	6.46561	6.99879	5.79755	6.17513	7.03698	6.4761	5.72913	6.00153	6.079	6.47457	6.03878	6.19003	6.26429	6.1899	5.95483
ATP6V0C	P27449	5.88913	6.03351	5.49119	6.49604	6.28022	5.34879	7.97545	5.53506	7.33784	4.42078	5.2895	6.40235	5.67872	6.78043	6.45307	8.58792
B2M	P61769	6.3291	5.24599	5.46028	5.62424	6.14301	5.16449	5.19513	7.84943	5.1378	5.78019	5.67948	6.46951	5.64719	8.45746	7.53232	8.28439
LRP12	Q9Y561	6.49103	6.84711	6.32856	6.4368	6.51461	6.65637	6.38312	6.43451	6.24238	6.41132	6.06012	6.5187	5.91545	5.24959	5.38124	6.22908
CALHM5	Q8N5C1	6.14545	6.36292	6.65305	7.00883	6.91454	6.4838	5.77336	5.8213	5.68428	6.0018	7.01808	6.5208	5.34305	6.25144	5.85374	6.16356
NDUFS6	O75380	6.84174	6.08813	5.91381	5.78438	6.106	5.96892	5.30906	6.29864	5.33151	6.41376	5.88103	6.74412	6.84261	6.6897	6.49328	7.2933
TMEM230	Q96A57-2	6.39787	6.24011	6.08123	6.36807	6.50934	6.17341	6.63152	6.11822	6.08304	6.09569	5.88072	5.32111	6.58806	6.36718	6.59851	6.54592
FUBP1	Q96AE4	6.89993	6.22279	5.06606	6.39607	6.83603	6.19727	5.8165	6.39287	5.8404	5.95184	6.85446	5.78696	7.32703	5.66826	6.47252	6.27102
UNKL	Q9H9P5-6	6.22568	6.32062	6.70813	6.13415	5.23095	5.48639	5.71883	5.44752	7.19211	6.32455	5.37061	6.86474	6.53288	5.91174	6.82526	7.70585
SH3PXD2A	Q5TCZ1	6.06138	6.37016	6.62541	5.99845	6.05956	6.46908	6.52001	6.1396	6.68576	6.54213	6.7938	6.8306	5.53649	5.90717	5.91741	5.54299
TRO	Q12816	7.06992	6.23835	5.96095	5.31157	6.77835	6.86148	6.18833	6.35918	6.64054	7.25874	5.63822	6.05852	4.73321	7.57316	5.62088	5.7086
TFR2	Q9UP52	5.96689	5.7851	6.37393	6.63856	5.83498	6.37008	6.16311	6.49697	6.57393	6.30306	6.23127	5.79684	5.96077	5.98865	6.71587	6.79999
PPP1R35	Q8TAP8	6.5873	5.92205	7.3622	6.6306	5.71316	6.17854	5.84346	7.36328	7.38799	6.38087	7.87618	8.12136	4.89898	4.46669	3.86383	5.40351
WDR87	Q6ZQQ6-2	6.13412	6.33614	4.64305	6.64755	5.83625	6.60852	5.14176	7.10212	6.08031	6.31913	6.57921	6.62311	6.31517	6.24896	6.57289	6.81171
SYDE2	Q5VT97	6.50675	6.26947	7.22303	6.75741	5.81977	6.59166	7.01065	5.99678	6.59333	5.69464	6.56278	6.59094	5.81833	5.54856	5.39114	5.43155
SMARCC2	Q8TAQ2-3	6.529	6.35063	6.30778	6.39721	6.35036	6.03154	5.83018	6.16247	5.85643	6.24069	5.593	5.21947	6.85234	6.87895	6.43256	6.9674
LRRC8B	Q6P9F7	5.26827	5.49046	5.86683	6.94236	7.17121	6.91184	6.61422	5.77685	7.64199	6.20825	6.13754	6.06352	6.57157	5.40695	6.07936	5.84879
NRBF2	Q96F24	7.03226	5.0769	7.8378	5.38037	6.28806	6.52446	5.29689	7.12161	6.58541	5.46341	7.10136	7.61055	6.64838	5.37482	5.01932	5.63841
RARA	P10276	6.06361	6.19876	6.00757	5.79312	6.05504	6.23225	6.54542	6.02075	6.3138	6.28256	6.44681	6.63459	6.24618	5.83253	6.9196	6.40742
MTMR4	Q9NYA4	6.49443	5.81364	6.4004	6.89912	6.1624	6.31843	7.20947	7.07298	6.11554	6.26092	5.45823	7.29835	5.50859	5.23063	5.36058	5.3963
TMEM231	Q9H6L2-2	6.17568	6.90138	5.51933	5.92091	6.20738	6.05872	6.82597	6.07943	6.24845	6.00784	5.868	5.77358	6.63344	6.31568	6.25966	7.20454
PLXNA3	P51805	5.78463	6.64328	6.15937	7.03324	5.57762	6.25073	6.43682	6.04866	6.19798	6.48	6.91557	5.97807	5.94181	6.63567	5.69727	6.21927
AP3M2	P53677	6.2103	5.96545	6.76431	6.7248	6.40541	6.64343	5.26892	6.82731	6.75137	7.73787	6.56344	7.29686	5.20299	6.28458	5.1089	4.24406
CCAR2	G3V119	6.36032	6.09692	6.37113	6.15554	5.18701	5.26428	6.45631	5.71415	6.16114	4.67242	5.72171	5.70601	6.62904	6.59918	7.49774	9.40708
NXT1	Q9UKK6	5.79029	6.04955	6.02376	6.51083	5.7195	6.07193	5.77814	6.29613	6.11181	6.41051	6.92527	6.29335	6.06881	6.56523	6.88734	6.49756
AFDN	P55196-1	5.499	6.87611	5.87142	7.11772	5.75305	6.6753	6.12917	6.49938	6.21308	6.3833	5.70177	6.22075	5.52396	6.46072	6.67696	6.39829
PCDHB10	Q9UN67	6.03599	6.42302	5.81765	6.22041	7.62251	6.48792	6.27078	6.3892	6.11055	5.7287	5.47671	5.66342	6.31517	5.89105	6.35276	7.19416
CLTCL1	P53675	6.23658	7.00907	7.21005	6.28449	6.1995	6.95603	6.4182	6.44773	6.43933	6.46155	6.56664	5.35755	5.63831	5.70285	5.23513	5.83701
BOK	Q9UMX3	5.9017	6.13315	5.54421	5.36971	6.2369	5.95831	6.54326	6.12106	6.28696	7.55993	6.18945	4.87933	7.83901	6.04659	6.30084	7.0896
ZNF696	Q9H7X3	5.3718	7.26776	5.24343	6.65165	6.63867	6.20811	6.53341	7.16161	6.06049	6.12945	6.56783	6.09417	6.39066	5.08223	6.21176	6.38697
ALDH1A2	O94788	6.66092	6.09721	5.9914	6.69176	6.75267	5.39166	6.52274	6.27566	6.11408	5.73762	6.09283	6.04394	6.71672	5.92247	6.69128	6.29705
COMMD3-BMI1	R4GMX3	5.86752	6.483	5.80712	6.84956	6.10116	6.27012	6.22067	6.18803	6.49699	6.5587	5.3453	5.98995	6.41445	6.13068	6.37431	6.90243
CEP104	O60308	6.2234	5.91635	6.87253	6.13378	7.18233	6.27377	6.51611	5.60076	6.65201	5.87048	6.19077	6.22672	6.10665	5.68696	5.89915	6.64823
GOLPH3L	Q9H4A5	6.10073	5.79174	6.16776	6.8333	6.30294	6.18302	6.61875	6.39029	5.7962	6.40859	6.56595	6.43246	6.81748	5.70251	5.58626	6.10202
LYN	P07948-2	6.87019	6.57269	5.23768	6.15416	6.42336	5.86645	5.93383	6.16923	6.06999	5.99491	5.84893	6.47206	6.77471	6.26557	6.67409	6.67216
FLNB	O75369	5.79715	6.07962	6.73498	6.61843	5.79887	5.75462	6.10804	6.81918	6.26692	6.26424	6.94433	6.69148	5.9934	5.71544	5.9256	6.48771
SMU1	Q2TAY7-2	5.99491	6.15745	4.89557	5.7878	6.33116	7.59504	4.91597	6.66791	7.50459	7.37839	7.02595	7.42245	8.20029	5.58477	4.79909	3.73868
RBM4B	Q9BQ04	6.57093	6.24221	6.39807	7.00362	5.74197	6.47865	5.34195	5.55509	6.69837	6.86373	6.80648	6.15421	5.91974	5.83203	5.41531	6.97763
NOL12	Q9UGY1	6.27531	5.82089	5.92536	6.55111	7.68408	6.03071	6.33396	6.01652	6.95044	4.84619	7.48603	5.27497	8.20393	6.12301	5.65033	4.82715
CBFA2T2	O43439-4	5.81379	5.77594	6.63454	5.5228	5.48412	6.0379	6.31556	6.49386	7.24618	6.51426	6.43433	7.25913	6.48357	6.38604	5.21349	5.38449
NPRL3	Q12980	6.393	6.42904	6.96666	7.15611	5.79856	7.4216	6.5453	6.4643	6.8683	6.75292	6.66375	6.37367	5.07227	5.39939	4.95478	4.74036
ATXN2	F8W0B5	6.15477	6.25482	6.7538	6.54996	6.43791	6.26294	6.26639	6.37081	6.65326	5.89916	6.42122	6.69118	5.52588	6.15829	5.19303	6.10659
ATXN2L	Q8WWM7	5.99269	5.50835	6.84782	5.36256	6.43511	6.24902	5.95988	6.75212	6.33198	5.9846	7.22647	7.74096	5.67708	6.18174	6.07731	5.67232
STARD3NL	O95772	7.18836	6.37323	6.35481	7.07808	7.27389	6.008	6.44343	6.70432	6.4698	5.99833	7.91519	6.13923	4.8394	5.31878	5.07113	4.82402
ISCA1	Q9BUE6-2	6.68725	6.36359	5.89002	7.40999	5.58175	5.73275	5.12291	5.84163	5.91276	5.97164	6.05239	6.64306	6.79339	5.62366	7.034	7.33921
CEP170B	Q9Y4F5	5.74858	6.4837	6.40514	6.11874	6.22709	6.45307	6.02836	6.64965	6.7814	6.53658	6.30347	6.79789	5.92944	6.16079	5.71619	5.65989
MEAF6	Q9HAF1-3	6.27086	6.48271	6.20192	6.79908	6.12934	6.10263	6.8819	5.84703	5.87217	6.17877	6.52294	6.12701	6.36948	6.19889	5.92755	6.08772
GPANK1	O95872	5.94041	6.87891	6.17766	6.90215	6.09001	6.64716	6.91872	7.15169	7.24963	6.44223	6.15956	6.07511	5.65149	4.5063	5.42637	5.7726
MBNL1	Q9NR56-5	6.17226	6.88983	6.36788	6.22367	6.7477	6.2736	5.84833	6.71796	6.62959	5.86047	6.93747	5.95822	6.78607	5.65829	6.01419	4.91447
B3GNTL1	Q67FW5	6.46449	6.12221	6.884	5.33746	6.06574	6.84749	6.43189	4.83411	8.10259	6.01101	7.08878	5.87295	6.63973	6.74161	5.06084	5.4951
ARMC5	Q96C12	5.67604	5.6205	5.74689	6.46893	5.92205	5.77736	6.15088	6.23312	6.73014	6.64519	6.55884	6.81441	6.83859	6.05229	6.37986	6.38491
SDHD	O14521	5.81694	5.2427	6.41964	5.75974	5.26636	5.60655	6.83941	7.12952	5.63506	5.35935	5.80975	6.13083	6.82766	7.10544	6.21718	8.83389
AAGAB	Q6PD74	6.1715	6.51722	6.75912	6.37855	6.05121	7.11959	5.78445	6.61691	7.21372	5.57134	7.07306	7.03844	4.8709	5.66696	4.89	6.27704
EWSR1	H7BY36	6.42994	6.11535	5.1983	6.69625	6.91727	6.38436	5.72623	5.72749	6.45126	5.99472	6.41853	6.29739	6.61103	5.36218	6.62218	7.04754
SHROOM1	Q2M3G4	6.98712	6.80089	7.03574	6.45202	6.29596	5.06295	5.63774	7.24588	7.6778	6.07295	6.4859	6.75536	5.39039	5.48347	5.25037	5.36547
FBXW11	Q9UKB1	6.48212	6.15838	7.02874	6.50986	6.70862	6.15251	6.52807	6.10535	7.31169	6.71522	6.62074	6.53531	4.99022	5.05775	5.79639	5.29901
PGK2	P07205	6.37804	6.19097	6.71202	5.80267	6.62002	6.82167	6.31842	6.06374	6.19773	7.15026	6.39861	5.96392	5.77687	6.47549	5.34783	5.78175
PROSER2	Q86WR7	6.52246	6.39092	7.15634	6.32427	6.15167	5.98466	5.59803	7.0605	6.32706	6.48125	6.4202	6.24574	6.07184	5.70863	5.59307	5.96336
EBP	Q15125	6.28999	6.81769	5.81503	7.33165	6.03462	5.43588	6.95883	5.90333	5.66047	5.79046	5.58301	5.99435	6.78163	5.73184	7.2834	6.58782
LRP5	O75197	6.13242	6.17766	5.97076	6.83347	5.73034	6.29511	6.86874	5.99246	6.18677	6.24068	5.86801	6.17968	6.48538	6.70506	6.40815	5.92528
EFCAB7	A8K855	6.16573	6.10828	6.31302	5.70725	6.3842	6.40384	6.43057	5.98983	7.0055	7.19087	6.34591	6.46214	6.2976	6.17305	5.40985	5.61237
DST	F6QMI7	6.00804	6.50723	5.89079	6.15244	6.09675	6.86385	5.78386	6.36096	5.71555	6.04406	5.9753	6.2483	6.30866	6.58638	6.54162	6.91621
PLEC	Q15149-4	6.62786	6.55051	6.92656	6.76073	5.76174	5.07945	6.69981	6.02022	6.12131	6.12642	7.16358	5.94777	5.58455	6.37188	6.90931	5.34831
PLEC	H0YDN1	6.40676	5.93032	6.16482	5.96052	5.50662	6.11856	5.48165	5.90277	7.20314	6.60428	5.36619	7.27824	6.059	6.66131	6.57658	6.77922
PLEC	E9PKG0	5.98057	5.50037	5.90431	6.20563	6.53746	5.99482	5.89521	6.63291	6.82778	5.78867	6.40461	6.03028	6.35089	6.71772	6.61726	6.61151

C3	P01024	5.19533	7.45981	4.80204	5.45667	3.77159	5.99524	6.45334	5.50307	7.43078	5.6484	6.15845	5.73312	6.52785	5.95786	10.8108	7.09569
CYTH2	Q99418	5.25276	6.34195	6.38236	5.96375	5.71698	6.05317	6.29166	5.51278	6.51347	6.6311	6.60821	6.7289	6.80106	6.30093	6.49804	6.40288
CXCL2	P19875	7.59363	7.28536	7.35412	7.49515	6.84154	5.73835	7.09511	6.86003	7.12273	7.24483	8.04328	7.02691	4.07638	2.90113	3.24784	4.0736
SGF29	Q96ES7	6.27002	5.42143	5.32305	6.35125	6.26199	4.82198	6.27605	6.02662	7.14788	5.91401	6.714	6.3659	7.46243	5.51191	6.55486	7.57661
HSP90AB3P	Q58FF7	5.5146	6.02085	6.49907	6.44939	5.76185	7.01266	6.39778	6.71355	5.84609	6.4292	6.43856	6.70788	5.82146	6.24635	5.88864	6.25208
APH1A	Q96BI3	5.98249	5.27152	5.18475	5.73851	6.36408	6.32963	6.91513	6.46271	5.89736	5.68794	6.14397	5.6813	6.99608	6.74813	6.82446	7.77194
ITPKC	Q96DU7	6.06147	6.62335	6.67549	6.06722	5.52224	6.84415	6.71251	6.63597	6.6417	6.68574	6.1824	6.34986	5.47752	5.69735	6.16104	5.66199
CELSR1	Q9NYQ6	7.39077	6.44345	5.89487	7.15684	5.84757	6.27486	6.78668	6.33974	6.31514	5.78048	6.41984	5.52557	6.35338	5.3195	5.55145	6.59985
RGS20	O76081	6.41393	6.25051	6.15469	6.42651	6.49015	6.0983	6.18769	5.63434	6.03305	4.89352	6.94534	6.17059	6.5154	5.95075	6.81701	7.01823
PIEZO2	Q9H5I5-4	6.31104	6.45693	6.10837	5.74362	6.13082	6.4966	6.36525	6.03196	6.55951	6.19724	5.9963	6.13078	6.84099	6.04106	6.43941	6.1501
ERCC6	P0DP91	5.73644	6.19275	6.45501	6.66666	6.0446	5.59232	6.35935	6.19257	6.50672	5.72682	6.38522	6.20086	6.32761	6.3443	6.38237	6.88641
MATN2	O00339	6.26655	7.13712	6.24391	7.11811	6.47569	5.4143	6.66035	6.2445	6.09891	6.18225	5.61233	6.56337	5.97819	4.66508	6.20279	7.13655
ALG13	Q9NP73	6.37448	6.3428	6.87898	5.93056	6.30133	6.29447	6.85127	6.04514	6.67203	6.22572	6.33721	6.19659	5.63471	5.68406	6.13984	6.09079
CCDC112	Q8NEF3-2	4.43919	3.73919	16.1869	5.95908	11.5285	9.08698	13.9234	4.46574	4.62182	3.16798	6.20182	3.83651	2.52498	2.88708	3.68424	3.54671
CEND1	Q8N111	5.60804	4.83503	5.92373	5.7246	6.36391	5.8194	6.05933	5.77954	5.40521	7.38867	6.49552	7.32163	7.40289	6.94632	6.20369	6.7225
SNAP25	P60880-2	5.89902	4.98367	6.46917	5.71235	5.6546	5.59624	6.35507	5.02697	5.67848	7.06298	6.10155	6.48834	7.27636	7.65119	6.12984	6.68415
SEPTIN3	Q9UH03	5.82162	6.38193	7.43475	6.53154	6.15716	6.11083	5.47265	6.09667	5.34877	5.78577	6.30454	6.92341	6.28537	6.59396	5.87671	6.87433
SMPD2	O60906	6.03356	6.53513	5.75946	6.54788	5.73904	6.43372	6.43052	6.30554	5.84644	5.74584	7.4484	6.04145	6.85328	5.72028	5.95366	6.60579
SELENOH	Q8IZQ5	5.79182	5.99586	5.26589	5.27937	5.63388	5.59531	6.04566	5.66334	6.25323	6.12434	5.95988	5.85208	7.21998	7.92353	8.27766	7.11817
TGIF2	Q9GZN2	6.22564	6.39557	7.17385	5.63359	7.91685	6.64051	6.30305	6.75027	7.5971	8.524	7.74561	6.96257	4.89169	3.82665	3.03478	4.37827
NOMO1	Q15155	5.32291	6.53105	6.36535	5.58144	6.92095	5.15273	6.84828	5.55036	6.48461	6.38308	5.11772	7.37981	7.3986	5.79608	6.60651	6.5605
CHURC1	Q8WUH1-2	6.00006	4.88766	6.08555	6.33899	6.13695	6.94447	7.64662	6.8512	7.19463	6.09677	7.19956	7.74682	4.09216	6.05405	5.24924	5.47527
KIF23	Q02241-3	7.16709	7.08551	7.05588	6.758	6.33044	6.45711	6.76632	5.75579	5.63592	6.3005	6.30042	6.04734	5.50787	5.28506	5.31425	6.23251
BTBD2	Q9BX70	6.30192	6.39443	7.74006	7.32826	5.91881	6.23896	6.16633	6.14987	6.57428	7.06891	7.01227	6.98886	5.02773	5.32646	5.30211	4.46074
RNF130	Q86XS8	8.09081	8.25074	7.37783	7.80382	5.44084	6.64064	6.13414	6.59985	7.94988	8.38546	7.45815	7.76984	2.95321	3.21067	3.3385	3.49564
CDC37L1	Q7L3B6	6.57679	5.72636	6.38269	5.30225	5.94503	5.37908	5.40508	6.95499	5.66063	6.51814	6.51693	7.31507	5.9765	6.90761	6.78991	6.64292
CDSN	Q15517	6.49241	5.9627	5.60398	7.72332	7.39575	7.81673	6.08057	5.26651	6.05551	5.42659	5.09541	5.87706	8.50732	5.84623	5.29453	5.55538
CCND1	P24385	6.86223	6.10594	8.13413	7.45061	7.34945	7.48192	7.5709	7.7706	6.49112	6.60135	7.7499	7.08258	3.01642	3.44997	3.22849	3.65442
TOP1MT	Q969P6	6.30721	6.23078	5.96287	6.89542	6.20611	6.46785	7.51241	6.66695	5.72912	6.07143	6.76472	6.72169	5.57109	5.08218	5.60834	6.20184
FUNDC2	Q9BWH2	6.79321	6.86125	5.73009	6.8112	6.53905	6.06143	6.59719	5.91734	6.60766	6.28431	6.13108	6.28192	5.86307	5.25838	6.09349	6.16933
FAM120A	A0A494C0Y9	6.29972	6.66094	6.80477	6.82205	6.01001	6.43793	6.13644	6.36984	6.59815	6.67423	6.56014	6.70318	5.27447	5.79251	5.35381	5.5018
PPP1R26	Q5T8A7	5.6037	7.63733	5.31106	4.91214	5.80438	5.68092	6.90557	6.33452	5.83192	5.47065	6.63868	6.37806	6.42197	6.49284	5.70425	8.87202
ALDH18A1	P54886-2	6.22772	6.25141	5.92717	6.5676	6.58596	5.17026	6.40731	6.11854	5.79313	6.47036	6.1438	6.78055	6.27256	6.45437	6.85532	5.97392
STK17B	O94768	6.31511	6.32648	6.69749	5.818	6.02131	6.0122	5.87989	6.51673	6.12905	6.37407	7.25785	7.12117	5.26289	6.25595	6.10216	5.90967
DLX1	P56177	6.75386	6.68134	6.21955	7.15965	6.40369	6.43359	6.14452	6.06868	6.08761	6.26632	6.21199	6.54332	5.73954	5.67559	5.89961	5.71113
TRAF3IP1	Q8TDR0	5.7307	6.13966	6.90429	6.2024	5.51853	6.20339	6.37969	6.24121	6.55844	6.66996	6.87842	6.47662	5.28504	6.44566	6.25827	6.10773
ACTN4	F5GXS2	6.8805	6.69769	5.71491	6.12214	6.54507	6.44364	5.24468	6.00849	6.53713	5.78356	5.39816	6.60485	6.31716	6.12065	6.65308	6.92829
PRPF40A	O75400-3	6.16526	5.9843	6.23543	6.24765	6.25356	5.56068	6.51525	5.36391	6.519	5.81427	6.78415	5.78854	7.04359	5.95596	7.30636	6.46207
MAFF	Q9ULX9	6.41252	5.79023	5.77111	5.73027	6.6934	6.88316	6.67412	5.26475	6.01589	6.34984	6.15681	6.06835	6.27381	5.16561	7.1022	7.64794
MAGEA1	P43355	7.20543	6.58169	6.95974	6.49878	6.31762	6.61183	6.29932	6.6671	6.63801	7.05961	6.5435	6.52289	5.04063	4.87451	4.84461	5.33473
PHC1	P78364	7.09949	6.68634	6.05938	6.74348	6.25239	6.03597	6.61793	6.10249	6.13348	6.72318	6.74526	6.28494	6.03017	5.09066	5.82949	5.56534
SAC3D1	A6NKF1	7.86404	7.12761	7.08619	7.83325	6.07257	6.34829	6.51143	6.80637	5.90682	6.78723	6.64944	6.34419	4.30368	4.14059	4.99412	5.22418
CHST7	Q9NS84	6.74317	6.75373	5.8819	6.99799	6.29942	6.73331	6.36603	5.54035	6.49451	6.09819	6.484	6.43363	5.65335	5.60529	5.87818	6.03693
ZNF850	A8MQ14	6.2328	5.63983	7.68326	7.80351	6.13431	5.6142	5.96055	5.53806	5.83906	6.99721	5.98424	7.21462	5.44975	5.13668	6.63125	6.14067
ZNF92	Q03936	7.10241	7.06286	7.5624	7.20798	6.34176	5.71786	7.52272	6.57717	5.7778	6.61133	6.84127	6.32769	4.99588	4.86798	5.01553	4.46735
ZNF749	O43361	6.24763	7.26479	6.55393	6.27324	5.96487	6.08291	7.18321	6.00335	6.18275	5.77188	5.81678	5.85882	6.37968	6.51114	5.84727	6.05774
ZNF260	Q3ZCT1	6.17469	6.54381	5.83684	6.5526	6.08376	6.76319	6.15878	6.09315	5.83796	6.43341	6.06859	6.07237	6.02132	6.04235	7.08434	6.23284
ZNF16	P17020	6.47673	6.45476	6.10857	6.60201	6.28592	6.5742	6.25117	4.82741	6.24416	6.13598	6.63766	6.39052	6.89834	5.90755	5.86371	6.34133
ZNF445	P59923	6.5822	7.53977	6.40771	7.3179	6.36832	6.48004	6.31486	5.99667	5.7221	6.94278	6.80099	5.28526	5.21625	5.08007	5.68905	6.25603
ZNF354A	O60765	6.19609	6.41178	6.2546	7.18623	6.03352	5.93442	6.94767	5.93438	7.18186	6.26421	6.73833	7.22485	5.20374	5.39593	5.47517	5.61724
ZNF836	Q6ZNA1	6.43056	6.27833	5.939	6.39126	6.03026	6.00301	6.20958	5.90448	6.2784	6.37586	6.36662	6.49186	6.42923	6.13613	6.58879	6.14662
ZNF180	Q9UJW8	6.43531	5.73391	5.91384	6.71006	6.50531	6.0412	6.24545	5.97099	6.36404	6.86986	6.03723	6.41156	6.53867	6.34429	5.73762	6.14065
ZNF7	P17097-2	5.95789	6.62323	5.69747	6.44707	6.80447	5.89288	6.64644	5.95284	5.77657	6.04428	5.64324	5.35587	5.6405	6.26401	6.79259	7.56065
ZNF33B	Q06732	6.47701	5.95649	6.05765	6.22856	5.5022	6.05551	5.81338	5.56655	6.54409	6.36883	6.18495	6.37457	6.69557	6.08825	6.44364	7.64274
FADS3	Q9Y5Q0	6.40776	6.51875	5.96819	6.73703	6.08923	6.13192	6.1836	5.85226	6.67991	6.10616	6.68034	6.6121	5.96013	6.01662	6.34094	5.71505
SNAPC3	Q92966	5.16747	5.85179	6.01748	6.51388	5.67239	6.00045	6.6044	6.16684	6.5231	5.80885	7.16433	6.29785	6.54812	6.13627	6.62738	6.8994
CCNDBP1	O95273	7.55078	5.96234	7.72356	6.18002	6.09356	6.44927	5.90872	6.57776	7.01159	7.75025	7.57028	7.76341	4.00928	4.68217	4.12641	4.64059
ZNF564	Q8TBZ8	6.26735	7.25956	5.63044	6.05291	6.14288	6.53561	5.337	6.11015	5.37089	6.84578	6.62819	6.35102	5.91725	7.01722	6.39163	6.14212
PEDS1	A5PLL7	7.40041	6.87368	5.55369	6.50793	7.17606	5.40518	6.65271	5.80932	6.54378	6.52821	6.93138	6.47043	5.14141	6.48463	5.5708	5.85038
PEDS1-UBE2V1	I3L0A0	7.0808	5.37012	6.91188	5.50984	6.62097	6.40528	5.94458	6.753	5.65067	7.0348	6.47832	7.168	6.17479	5.80151	5.36595	5.72947
DYRK1B	Q9Y463	6.45787	6.57869	5.93525	6.06323	6.02311	6.33936	5.95372	6.34064	6.02321	7.33435	6.24233	5.85479	6.3137	6.76371	6.26651	5.50953
ZNF408	Q9H9D4	5.74874	6.87911	6.22613	7.05031	6.02383	7.01824	5.95895	7.16987	5.6702	6.36236	7.04014	6.71103	6.24347	5.55025	4.97881	5.36856
RAB30	Q15771	6.64134	6.95919	6.455	6.57587	6.2503	5.59927	6.73659	6.06407	6.34702	6.33384	6.36995	6.26978	5.81448	5.61986	5.89619	6.06725
RAB43	Q86YS6	6.12867	6.32234	6.578	6.79789	6.15082	5.62107	6.59024	6.28649	6.27252	6.22627	6.63632	6.12936	5.61729	6.02488	6.79811	5.81975
ZFP30	Q9Y2G7	6.64944	7.1855	5.93813	7.34285	6.44785	6.37579	6.7533	6.46166	6.46353	5.75952	5.78599	6.17291	6.06276	5.32202	5.64875	5.62999
FITM2	Q8N6M3	6.71032	6.38969	5.60166	6.51211	6.57869	5.52235	6.12687	6.29041	6.20229	6.06104	6.30955	5.90584	6.66928	6.10523	6.56903	6.44565
ZFP14	Q9HCL3	6.76056	6.69417	6.26151	7.04416	6.66725	6.17559	7.0562	6.74806	6.88558	5.98554	5.77483	6.49363	5.67381	5.33812	4.8083	5.63267
DCLRE1B	Q9H816	6.65577	6.77248	6.31837	6.39959	5.86177	6.02502	6.79115	6.32136	6.29107	6.64145	6.48942	6.35596	5.32645	6.23056	5.14653	6.37305
FANCL	Q9NW38-2	6.71802	5.51118	5.9545	6.3792	6.27216	6.53105	6.09864	5.62103	6.35635	7.08103	6.43541	6.39445	6.91289	4.74752	6.72204	6.26452
WTIP	A6NIX2	7.14403	6.64205	7.31026	7.12689	5.95181	6.38683	6.36939	6.25698	6.67423	5.89099	6.65223	6.53472	5.56648	4.91714	5.02225	5.45471
PSIP1	V9GYT7	5.82303	6.18966	6.71075	5.53639	6.23843	5.64231	6.7598	5.49454	6.77324	6.98543	5.83366	6.08966	7.21438	6.36326	5.82666	6.51881
LYSMD1	Q96S90	5.42609	4.88584	5.96959	4.47619	6.07194	5.42142	5.14686	5.97386	6.18522	6.65216	7.44473	6.86345	7.86023	7.32453	7.36738	6.93051
HOXC8	P31273	6.04178	6.14882	6.4487	5.7857	5.80023	6.48831	5.69678	6.06325	6.4899	6.77021	6.88434	7.04487	6.16114	5.95797	6.16118	6.05683
JMJD8	Q96S16	6.98787	7.0666	6.30421	6.999	5.89447	6.31871	6.27606	6.6459	6.93494	7.04145	7.41236	6.83316	5.10745	4.43013	4.56064	5.18705
ACTBL2	Q562R1	6.54899	7.39856	7.20573	6.66394	7.65436	7.65868	5.99229	5.34408	5.28247	5.96556	5.89178	5.55295	6.88661	5.69102	5.4295	4.83348
BMP1	P13497	7.32095	6.76649	5.44775	6.81743	5.7651	5.9968	7.66934	5.6228	6.73728	6.73398	5.89301	5.81466	6.56493	4.73546	6.02295	6.09105
BIRC3	Q13489	5.71669	6.09716	6.40981	5.32869	6.80678	6.75297	5.84732	6.86051	7.62205	6.05451	7.12908	6.84912	6.11672	5.51259	5.38335	5.51265
CYBC1	Q9BQA9	6.54665	6.55922	5.66973	6.46194	6.21574	6.08739	6.30177	5.33174	5.94408	5.9469	5.98649	5.62381	6.98235	6.68933	7.34718	6.30567
ALDH9A1	P49189	6.04939	6.29866	5.61916	6.25064	6.02617	6.01557	7.24422	5.17964	7.4505	5.03399	5.53943	5.93128	6.44866	6.6408	6.27066	8.00123
C19orf54	Q5BKX5	6.40693	6.23572	6.72288	6.52552	6.59709	6.16792	5.48349	7.01572	6.39559	6.50852	6.90456	6.77851	6.6895	5.51609	5.32153	5.63041
NR1H2	P55055	6.12409	6.12182	5.95085	6.44304	5.9717	6.03813	6.32034	5.93774	6.13815	6.41118	6.42765	6.31131	6.20006	6.06986	6.96912	6.56495
MTA1	Q13330-3	6.62994	7.17481	6.35386	6.68826	6.40353	5.16218	6.44294	6.03992	5.64152	6.46513	5.79834	5.88732	6.51415	5.79779	6.65631	6.15736
MTMR11	A4FU01	6.98287	6.12417	6.00699	6.29328	6.70722	6.68217	6.98785	5.91865	6.42166	5.41731	6.01727	5.77153	5.88815	5.86315	6.5429	6.37484
AKAP5	P24588	7.26921	6.2311	6.54763	6.04547	6.04606	6.48015	5.98484	5.77527	5.75807	5.39916	5.83596	7.17299	5.90832	6.27595	6.58117	6.68864
HIC2	Q96JB3	6.01767	5.17317	6.94405	7.28456	6.94277	6.09166	6.51934	6.27103	6.40374	5.89741	6.45189	6.77166	6.27567	4.96418	6.08269	5.90851
YRDC	Q86U90	6.12469	5.91379	6.05352	6.45969	7.1863	5.36794	5.98736	7.18202	6.45208	6.83799	6.16543	7.42494	6.21212	5.71086	5.44187	5.47939
POGK	Q9P215	5.4736	6.1667	6.8599	5.58932	6.18892	7.18263	6.77681	5.9143	5.7092	6.50082	6.11712	5.15436	5.85597	6.7158	6.67462	7.11993
ERICH1	Q86X53	5.25645	5.76243	5.71797	5.38247	5.57531	6.00872	5.93644	5.76265	5.85822	6.34114	6.51654	6.57838	6.6556	7.72122	7.83272	7.29373
MT-ND4	P03905	5.64488	6.47849	5.78622	5.8847	6.0943	6.58093	6.52979	6.80996	6.00831	6.55503	5.52939	5.51081	6.5613	6.78138	6.69817	6.54635
POLR1D	P0DPB5	6.75773	6.54878	5.89772	6.98169	6.5994	5.99594	6.33612	6.76635	6.81014	5.85732	6.56642	6.21673	5.18564	5.42404	5.71013	6.34585
AP1S2	P56377	6.04397	6.94346	7.01357	6.60094	6.17746	6.78697	6.67783	5.92261	6.5391	6.25008	6.3489	5.98016	5.55315	5.7857	5.66208	5.71402
GABARAPL1	Q9H0R8-2	6.51946	6.39211	6.39907	5.8207	7.11333	7.32825	6.53233	6.84261	6.89053	6.3279	5.8464	6.13625	5.69356	5.45677	5.29165	5.40909
CEP57	Q86XR8	6.3452	7.94214	6.18921	6.59952	5.98712	6.83389	7.73076	7.71689	6.92988	7.44704	6.57286	6.23745	4.35759	5.3436	4.05731	3.70953
PYROXD1	Q8WU10	6.52498	5.9276	7.21137	5.86489	6.1609	6.45655	6.0295	6.35162	7.01593	6.16479	6.78111	7.21046	5.38288	5.10954	6.2891	5.51876
XRCC1	F5H8D7	5.78275	5.62202	5.89996	5.81036	5.95599	5.78298	6.08134	6.23565	6.25736	6.28908	6.60671	5.963	6.39012	7.24439	6.4224	7.65589
KYAT1	Q16773	6.03566	5.84171	6.65736	5.4901	6.47074	7.01509	6.26237	6.55138	6.23222	6.38554	6.88586	6.78082	5.87037	5.90198	5.97637	5.64246
CPQ	Q9Y646	5.8957	6.28021	5.8653	6.31503	6.1919	6.89359	6.91524	6.28795	5.11531	6.40306	6.31152	6.16803	5.76138	6.03566	6.72107	6.83903
FGF1	P05230	6.06001	6.06119	7.16735	6.41604	6.69296	6.22183	7.64424	7.8501	6.159	5.3544	7.24826	7.84297	4.99423	4.70977	5.00598	4.57169
FGF2	P09038	5.99454	6.50029	6.07103	6.05165	6.69527	6.27955	6.14064	6.19906	6.36051	5.92097	6.20375	5.71454	6.82203	6.48278	6.62428	5.93911
RGPD5	Q99666	6.72382	5.86391	6.50801	7.26216	6.18904	6.00978	6.06888	6.08672	6.61381	6.04206	5.70128	5.59079	5.73157	6.73487	6.02546	6.84784
ZC3H14	Q6PJT7-9	5.97156	6.20198	5.81596	6.81941	7.3473	6.52336	6.18152	6.07387	5.94649	5.70882	6.63899	6.87295	6.49648	5.5603	5.62695	6.21406
REL	Q04864	6.25948	6.90387	7.0456	5.87532	5.6667	6.38229	6.75495	7.15723	6.86307	6.12295	5.7783	6.86631	5.34432	6.09523	5.55962	5.32477
SMAD1	Q15797	6.31957	5.72205	6.77194	6.17189	5.78483	6.6348	5.69209	6.68416	7.63476	6.08367	5.99803	6.43459	5.65836	6.37698	5.75622	6.27607
SMAD9	O15198	6.63087	6.45878	6.54415	6.42928	6.35926	6.58455	5.98815	5.94992	6.05025	6.66764	6.28257	6.47911	5.93371	6.0168	6.08642	5.53852
ULK3	Q6PHR2	5.91867	5.91658	7.34654	6.27294	6.41633	6.8	6.19976	5.70989	6.13028	6.84775	6.92786	6.77847	5.29239	5.75109	6.37036	5.32108

PDK3	Q15120-2	6.69968	6.20773	5.4764	7.19445	6.21725	6.61444	6.25605	6.60335	6.05037	6.15776	6.78893	5.73208	6.28635	5.65296	6.68618	5.37603
KHDRBS3	O75525	7.01569	6.44668	5.85694	6.64845	6.15793	6.02177	6.63889	6.27027	6.42075	6.00437	5.94507	6.00999	6.45231	5.43692	6.14345	6.53052
RPL10	F8W7C6	5.9353	5.97332	5.78588	5.43095	5.15395	6.54556	7.07994	7.81358	7.16532	6.19313	7.44498	5.88579	6.27483	6.07035	4.95453	6.29258
FAM133B	Q5BKY9	5.63401	5.18558	6.2451	5.03427	5.00482	5.32105	5.36149	5.41533	6.10635	5.65749	7.06226	6.48619	7.71129	8.0586	7.98318	7.733
WDR46	A0A0G2JJL1	5.67435	6.08369	5.93437	6.34078	5.67145	5.42659	6.18259	6.38265	6.53161	5.84084	6.07526	6.1813	6.50022	6.74222	7.02433	7.40774
COA8	Q96IL0	6.46017	5.62309	6.95151	7.36866	7.25942	6.45511	5.17526	6.55465	6.61643	5.95619	5.79619	6.9121	6.43544	5.14454	5.03202	6.2592
DHRS4	Q9BTZ2	6.34268	7.08716	6.02812	6.44945	6.50118	6.57259	5.9896	6.72256	5.75791	6.42172	5.73156	5.56486	6.3386	6.24763	6.43867	5.80571
PXMP2	Q9NR77	6.24925	6.13165	5.72164	6.70495	7.29762	6.54696	5.86132	6.43785	6.06123	6.24348	5.18373	5.94235	6.53403	6.13919	6.3396	6.60514
PNKD	Q8N490-2	6.04889	5.40277	5.59191	6.49831	6.76099	6.0862	6.27696	5.91122	5.96463	6.10294	5.76864	6.03251	7.21916	6.9278	6.88732	6.51974
VKORC1	Q9BQB6	5.72554	6.07307	5.84178	6.35751	6.16656	6.29814	6.41291	5.98013	6.12261	6.06388	6.46317	5.90838	6.96483	6.71057	6.42818	6.48274
SIVA1	O15304	6.16498	6.46195	7.47523	5.73949	5.77762	6.5003	6.00222	6.25069	6.51706	7.03525	7.25333	7.23363	4.77276	5.4076	5.82999	5.57789
FAM126A	Q9BYI3-3	6.00499	5.80624	5.00375	5.87311	6.56874	7.07049	6.21275	5.41861	6.09276	6.9012	7.81436	5.83825	6.8885	6.9151	6.1182	5.47295
ZNF608	Q9ULD9	6.49416	7.92746	6.04741	6.24191	6.36294	6.80493	4.89719	5.03498	6.38085	7.12613	5.87284	5.36914	6.2415	6.75136	6.50703	5.94016
TMOD1	P28289	6.49788	6.5457	6.08501	5.5232	6.48836	6.23839	6.1242	6.48532	5.89455	6.54467	6.65653	6.12672	6.57267	6.37642	5.72351	5.75687
DLG3	Q92796-2	6.49676	6.47545	5.62111	6.6633	6.18802	6.49473	5.94698	6.52698	5.77857	5.88267	6.28566	6.66727	6.008	6.02331	6.89008	6.0511
MAP4K2	Q12851	6.63947	6.27999	6.32008	6.27124	5.65488	6.25538	6.44943	6.60916	5.99509	6.56347	7.19745	6.42329	5.75521	6.20744	5.69691	5.6815
ZNF207	O43670-2	5.87306	4.65201	7.11101	6.54032	6.49696	5.04185	5.95822	6.0763	6.92976	5.4115	6.43048	6.22786	7.05552	7.05979	6.52295	6.61241
FTL	P02792	8.30203	7.22846	6.62593	6.88623	5.47758	4.95109	4.91862	7.15309	6.6897	6.66962	6.31298	6.72325	3.84082	6.40366	6.22391	5.59303
ANAPC16	Q96DE5	6.34097	5.39837	6.20947	4.54243	5.65545	5.27414	5.75067	6.7791	5.19313	6.33811	5.68043	7.14629	5.18806	8.48919	7.85341	8.16076
PIK3R3	Q92569	6.34257	6.15222	6.88221	6.53518	5.83773	7.52639	6.01585	6.61321	6.4887	7.6087	8.17698	7.3792	4.55015	4.96753	4.11693	4.80646
VGLL4	Q14135-4	4.79752	4.87995	6.78178	4.66696	6.08882	5.15715	5.51902	6.98545	5.42833	7.72004	7.37927	6.82337	6.86473	8.24766	6.15356	6.50639
SERPINI1	Q99574	6.24176	6.30811	6.75755	7.02436	5.97776	5.54594	6.44488	6.27177	5.9844	6.75004	5.73121	6.47323	6.46824	5.53151	5.69199	6.79724
NMNAT3	Q96T66	6.30118	5.99758	5.98651	6.91679	5.93016	6.1111	5.79021	5.93537	5.82056	6.59358	6.66784	6.52265	6.70603	6.37579	6.06956	6.27509
TMEM242	Q9NWH2	6.66621	5.81755	5.33334	6.09223	6.44057	6.07481	7.11044	7.23055	6.75719	6.08022	6.30845	5.79182	6.51718	5.41965	6.51828	5.84151
ING2	Q9H160	5.88565	7.95937	5.11401	7.15777	4.87444	6.65131	5.80168	6.56191	5.59224	6.42015	5.10886	6.43055	5.54177	7.37486	6.17693	7.34848
SRF	P11831	4.8758	5.08691	6.53222	5.72844	5.18136	6.53945	5.66959	5.47033	6.01829	5.59646	7.73907	5.84019	7.21001	7.64161	7.25394	7.61632
EEF1AKMT4-ECE2	P0DPD8	6.61876	6.38844	6.94473	6.10514	5.74115	6.74569	6.61541	6.94159	6.22034	6.92911	7.0794	6.90638	5.28556	5.01271	4.63676	5.82883
METTL26	Q96S19	5.6191	5.31317	5.61823	4.91506	5.07126	4.94511	5.50322	6.80758	4.68223	6.43852	5.32381	6.68089	4.69455	11.2569	7.48694	9.64341
EPDR1	Q9UM22	6.48318	6.08091	5.47497	6.38629	5.6574	6.05262	6.10686	6.34209	6.19822	6.11134	6.69179	6.03916	6.94691	6.60925	6.10066	6.71834
FAM171A1	Q5VUB5	6.42441	6.37784	5.98505	6.53101	5.93345	5.99932	6.3493	6.14863	6.48962	6.06426	5.9657	6.40321	6.48486	6.16007	6.33987	6.3434
TPCN1	Q9ULQ1-3	6.26091	6.91392	6.06594	6.19599	6.35878	6.22347	6.53696	6.25462	6.1295	5.4692	5.84614	5.98285	6.4116	5.89885	6.60614	6.84513
PAPLN	O95428	6.83574	5.6894	5.86866	7.43354	5.91947	6.33031	5.88634	6.75169	6.27634	6.61114	7.38712	6.49296	5.72961	5.25842	6.00264	5.52663
WIPF1	O43516-3	6.53411	5.6206	7.79668	5.75772	5.83733	6.37926	6.42638	5.79826	6.43953	6.03445	7.01564	6.70726	5.51388	6.2642	6.1413	5.7334
MAP4	E7EVA0	6.57166	6.0138	6.38092	5.9839	5.83708	6.28768	6.08335	6.44085	6.65774	6.66982	6.45204	6.75198	5.33403	6.61445	5.8913	6.0294
COLGALT2	Q8IYK4	6.36507	6.79143	6.09912	6.45555	6.31555	6.33715	6.81623	5.68652	6.57019	5.55367	6.39153	6.31658	6.26468	5.48338	6.5263	6.02704
B3GNT2	Q9NY97	7.00432	7.23442	6.88229	6.8107	6.68115	6.62168	6.75303	6.03767	5.92242	6.61446	5.97528	6.16294	5.24573	5.6302	5.06683	5.35689
NOSIP	A0A075B6F9	5.73122	6.54106	7.75825	6.00604	6.37069	6.604	6.38961	6.59216	6.79002	6.38864	7.20692	6.52183	5.16158	5.69882	5.28765	4.95149
GTF2IRD1	Q9UHL9-3	6.93955	7.21695	7.37538	7.569	6.96391	6.34318	6.16677	5.70106	6.43937	6.55266	6.19467	6.48409	5.33251	4.48734	5.02163	5.21193
RNPEPL1	Q9HAU8	5.90915	5.87652	6.94797	6.10534	6.13499	7.01125	6.25743	6.44775	6.29118	6.5024	6.01937	7.07248	5.90403	6.6654	4.90716	5.94758
VASN	Q6EMK4	7.60389	6.53059	6.67344	6.49949	5.52391	5.44102	5.72776	5.64254	6.21061	7.13735	7.32438	6.87761	5.44409	5.98043	5.14153	6.24138
ST3GAL4	Q11206-4	6.2402	6.68171	6.21848	6.90681	6.34859	5.98483	6.65446	5.71401	6.23546	6.8906	6.55109	6.06853	6.12862	5.69567	5.52805	6.1529
TRIM62	Q9BVG3	6.23782	7.08144	6.33912	5.9852	6.22675	6.69893	5.70616	7.1825	6.72386	7.06221	6.89379	7.82953	4.62588	5.61709	4.54071	5.24901
ZDHHC3	Q9NYG2-2	5.98561	6.73128	6.64726	6.85474	6.22002	6.21316	5.84125	5.54504	5.95919	6.07265	6.19225	5.90006	6.49784	6.54565	6.69842	6.09558
TEAD2	Q15562-4	6.99637	6.52733	6.90621	6.55224	6.48788	5.47025	5.69984	6.25042	6.34354	6.01496	5.73392	6.2108	5.94359	5.47477	6.77928	6.6086
TEAD4	Q15561	6.42312	5.92991	5.42079	6.53027	6.10119	6.14278	6.43186	5.88678	5.83146	6.48323	6.46683	6.58478	6.43765	6.34185	6.41093	6.57657
TCEA2	Q15560	6.05716	5.77462	6.3738	5.58941	6.46703	6.221	6.23471	6.01992	6.29682	6.51617	6.29023	6.17735	6.74735	6.6351	6.43063	6.1687
G2E3	Q7L622	6.53537	7.86168	6.91267	8.57594	6.20386	6.64025	7.76697	6.88825	6.06296	6.11773	6.80424	7.6484	3.82692	4.5585	3.29292	4.30334
FBXL14	Q8N1E6	6.86686	6.64603	6.59675	6.74784	6.02806	6.32928	6.40943	6.13411	7.0368	7.05344	7.17554	6.70991	4.67143	5.04027	5.13376	5.42047
TRPT1	Q86TN4	6.45428	6.0913	7.10702	6.39877	6.36429	6.7066	5.86805	5.96592	6.53841	6.93911	6.25185	6.77595	5.68554	5.88738	5.61628	5.36362
CDC42EP3	Q9UKI2	6.06669	5.80567	7.1654	6.27098	6.59145	6.51591	6.63771	7.86114	5.91486	5.76749	6.55964	6.98408	5.88569	5.21266	5.87566	4.88497
GJC1	P36383	6.55419	7.04081	7.167	6.13248	6.17091	5.61712	6.26127	6.55356	6.4542	5.99901	5.5868	5.94526	5.68872	5.79655	6.50664	6.52549
CHSY1	Q86X52	6.4325	6.22554	5.78128	6.83243	6.38768	6.34739	6.87568	6.19445	5.9832	6.0999	6.07728	5.9393	6.12794	6.38999	6.2967	6.00876
SDCBP	O00560-2	5.59617	6.12114	6.66422	6.23575	5.59251	7.018	6.29567	6.47418	7.11634	6.99366	7.05019	6.79597	5.00582	6.16089	5.20673	5.67276
MEX3C	Q5U5Q3	7.40682	7.24029	7.39092	5.76453	5.86764	6.68942	6.44256	6.71357	6.24752	7.20194	6.75852	7.27597	3.91464	4.21987	5.80542	5.06037
PMAIP1	Q13794	6.66963	6.66482	5.82625	6.24652	7.08749	6.09407	6.51072	6.24184	6.1203	5.89255	5.89879	5.37317	6.06592	6.28023	6.49935	6.52837
TSPAN4	O14817	6.74139	7.00801	6.68456	7.07382	5.97521	6.4057	6.95134	6.59866	5.47425	6.46006	5.82101	6.06674	5.07491	5.98745	6.00279	5.67409
IL27RA	Q6UWB1	6.45163	6.71564	6.68683	7.06565	7.07928	6.46994	6.58803	6.25371	7.47393	7.00129	6.51294	5.72586	4.45772	5.06998	5.70134	4.74624
SCARF2	Q96GP6	6.79347	6.70928	6.2708	7.30307	5.99768	5.43413	6.43507	6.36265	5.68785	6.3726	6.78071	6.2113	6.08997	6.10175	5.71779	5.73188
EPB41L2	H0Y5B0	7.48852	5.75263	6.40882	6.53449	6.20008	6.04361	6.96843	5.37	5.45401	6.30399	6.49324	6.51389	5.84714	6.34938	6.73005	6.54173
ABCA6	Q8N139	6.28494	6.19316	6.77261	6.18202	6.71275	6.61961	5.98046	6.4291	5.62475	6.95521	5.79172	7.06311	6.12735	5.22132	5.92512	6.11677
KIAA1522	Q9P206-2	6.26045	6.55058	5.96317	6.46636	6.11976	5.88311	6.00845	6.55876	6.17322	6.58878	5.95804	6.50378	6.51406	6.67573	5.51707	6.25868
MGA	H3BU53	5.5459	6.48934	5.71804	7.77761	6.65356	6.08022	5.69396	5.95774	6.28134	5.90367	6.49397	6.49322	5.12689	6.00015	6.91036	6.87403
ZNF576	Q9H609	6.39448	6.17087	7.09738	5.73886	6.29368	6.48761	5.76191	6.9243	6.70794	6.07913	6.47136	6.51427	5.77167	5.73023	5.91959	5.93671
CSNK1G1	Q9HCP0	6.26267	6.01366	5.89035	6.82687	6.13392	6.20623	5.8908	6.62353	6.79575	6.35276	6.43851	6.75871	5.6994	5.99681	6.46007	5.64997
PLEKHA2	Q9HB19	6.0344	5.71623	7.13228	5.80742	5.83151	7.35451	7.41102	5.65609	5.89582	6.69567	5.91206	6.40988	5.50414	6.66401	5.64415	6.33079
IFNGR1	P15260	6.79275	6.89663	7.39654	7.782	5.67109	5.80984	6.23693	5.93979	6.7175	6.74486	7.16897	6.88412	4.48784	5.12494	4.81116	5.53504
TMEM259	Q4ZIN3	6.86521	6.05807	6.17887	7.92511	6.21954	6.57087	6.09172	5.42298	5.81488	6.76937	6.55402	5.59117	5.62523	5.49929	6.36493	6.44874
ZFP64	Q9NTW7-5	6.72948	7.29793	6.28195	6.95099	5.96769	6.41546	6.05319	5.21557	5.32829	6.45096	5.24634	6.66824	7.08384	5.71502	6.22761	6.36745
HLA-C	A0A140T912	6.78069	6.50222	5.54711	6.75996	6.57011	6.3795	6.47801	6.57515	6.10585	6.65012	6.01112	6.34082	6.24581	5.48367	5.73155	5.83831
HLA-E	P13747	7.88233	7.04728	7.95238	6.72715	7.49347	6.20343	6.56314	5.99267	7.38	6.75029	7.67343	7.89547	3.95183	3.47339	2.90352	4.11022
HLA-F	P30511-3	5.84655	5.21871	5.6276	6.25844	5.89573	5.63764	6.25692	6.28835	6.83634	6.40384	6.68622	6.41485	6.68267	6.38918	6.58036	7.58155
MAP3K15	Q6ZN16	6.51297	6.29436	7.02708	5.94311	6.10996	6.76782	6.16817	6.62585	6.67712	6.80037	6.82077	6.77272	5.52192	5.65251	5.16018	5.14508
ZDHHC18	Q9NUE0	6.75439	7.62691	7.24597	6.34725	7.08252	5.25853	6.87436	6.39364	6.65529	5.32043	6.56897	6.13479	5.42567	5.13155	5.68165	5.49806
MPRIP	A0A494BZV2	6.3582	5.28415	7.44868	6.90501	5.46537	5.24861	5.77703	6.46373	6.80684	6.23721	6.89006	6.11207	7.01423	5.81527	5.24875	6.92479
MIS18A	Q9NYP9	6.13194	6.59462	5.92782	6.54434	6.152	6.57998	6.14237	6.79101	5.99293	6.91278	5.94558	6.92684	5.83444	5.38196	5.63419	6.50722
NSD2	O96028-3	6.65616	6.07959	6.06955	7.99085	6.26438	5.12187	6.30617	5.2125	6.72012	7.04176	6.28494	5.60928	6.04892	5.45789	5.75496	7.38107
IFIT1	P09914	7.32086	7.19468	6.08387	6.1456	6.06167	7.1197	6.57002	6.66754	7.39425	5.90041	6.07856	6.44697	5.10425	5.2524	4.95732	5.70192
CCNYL1	Q8N7R7	6.30056	5.94305	5.36211	6.75855	6.29486	6.45271	5.54941	5.87799	5.6155	6.99501	6.23664	6.15045	5.86497	5.96606	7.57132	7.06081
COPA	A0A3B3ITX2	6.19436	6.94354	7.42073	5.61171	5.59174	7.35506	5.65568	6.59913	6.1781	6.51997	5.9815	5.98835	5.82183	6.30887	5.71245	6.11697
ATP8B2	P98198-3	6.37532	6.60489	6.41579	6.44188	6.05486	5.72182	6.50574	5.76713	6.62923	6.7124	5.90004	6.09969	6.37259	6.08689	6.18099	6.13074
ATP8B1	O43520	6.26096	5.89698	6.54204	6.70142	6.82279	5.80002	6.22019	6.81173	5.73967	6.59505	6.43232	6.40205	5.39649	6.03068	6.97385	5.37375
MICA	A0A140T986	6.14661	7.02645	6.7603	7.93775	5.66657	6.26779	6.78096	5.78258	6.19463	6.29971	6.46577	6.706	5.25613	5.40059	5.30108	6.00709
CIB1	Q99828-2	5.95205	5.40799	5.85169	5.18655	5.24981	4.44179	6.08238	5.55597	5.18186	5.92872	5.8535	5.89213	5.65977	10.1954	7.28447	10.2759
ADCK1	Q86TW2	6.13953	6.68265	5.87985	6.52262	6.61076	6.02305	6.37124	5.4296	5.9666	6.60036	5.95317	5.9094	7.13497	5.83936	6.72129	6.21555
PPCDC	Q96CD2	6.61107	5.11381	6.84005	4.71013	5.54591	5.57325	5.05489	7.57332	4.69554	6.63522	5.49607	7.30336	5.3703	9.07607	6.82524	7.57578
IL31RA	Q8NI17-2	6.19251	5.86363	6.83698	6.78443	6.37123	6.44469	6.07558	5.85503	6.26193	5.3908	6.95127	6.13614	6.83763	5.90864	6.04854	6.04097
NOL3	O60936-3	6.98542	6.23825	6.97072	4.59737	5.30059	5.34974	5.76746	7.12654	4.81128	7.02231	6.60058	7.16672	5.71437	7.53693	6.21699	6.59471
NME2P1	O60361	7.19747	5.02213	5.93824	4.82872	6.31469	6.11088	7.03727	5.94725	7.88587	6.43248	7.05758	6.49548	5.77769	5.95214	6.00001	6.00659
SLC2A13	Q96QE2	6.10275	6.47592	6.08822	6.91092	5.99976	6.65087	5.5994	6.54297	6.22083	5.91931	6.52652	6.00908	5.51033	6.2961	6.72753	6.41948
DDB2	Q92466	6.15463	6.22846	5.98973	6.13393	6.22763	6.07057	5.6883	5.63206	6.27272	5.98788	6.83854	5.77355	7.24009	6.6131	6.64279	6.50603
TRIM38	O00635	5.2392	6.23388	6.52978	6.18706	6.6821	6.55857	6.00883	5.3272	6.16564	6.43128	6.35832	6.1771	6.34459	6.67126	6.78185	6.30335
ELFN1	P0C7U0	5.41378	5.85413	5.76228	6.42679	6.30871	6.16296	5.92382	5.98554	6.80652	6.31216	5.85232	5.53172	7.05434	6.77923	7.37621	6.44949
ZDHHC21	Q8IVQ6	6.38076	5.98723	6.19752	6.34582	5.92015	6.21284	6.79372	5.91979	5.06373	5.58045	6.92487	6.01027	6.56787	7.45266	6.37522	6.2671
ZNRF1	Q8ND25	6.84297	6.78179	7.57014	5.92341	6.02322	5.7158	6.02319	7.01984	6.63418	7.93712	7.25961	8.43195	3.99172	4.52426	4.61389	4.7069
NRG1	Q02297-2	5.86398	5.42009	6.34834	6.41243	6.42929	6.18294	6.5625	5.59679	6.83511	6.1807	6.88531	6.51273	6.33216	6.05399	5.75107	6.63258
OTUD5	Q96G74	6.65412	6.07778	7.66136	6.11538	6.03245	6.29728	6.19	6.11445	6.61898	5.63708	7.6043	6.53877	5.79384	5.11914	5.40196	6.1431
TENT4A	Q5XG87	6.53337	6.64404	6.25311	6.97819	6.02964	5.99445	6.35296	6.00363	6.18516	6.29059	6.82795	6.58778	5.96861	5.58345	5.90313	5.86393
ELMO1	Q92556	6.17645	5.76424	6.58945	6.29355	5.83683	6.7692	5.63816	6.70026	6.62244	6.69632	6.37596	6.52257	5.95862	6.2686	5.82806	5.9593
DHRS11	Q6UWP2	5.93655	5.76609	6.82484	6.23497	7.28993	7.31524	6.23696	5.56142	6.36515	7.09178	6.8612	6.45794	5.61097	5.63891	5.1487	5.65936
RRAGA	Q7L523	5.94499	6.12359	7.37896	6.9299	6.52864	6.13731	6.18693	6.56946	7.07884	6.26357	6.64292	6.40622	5.37042	5.86629	5.4	5.17196
RAB29	O14966	6.50221	6.60612	6.3251	6.64563	6.30258	6.10793	6.43706	6.25229	5.95706	6.11805	5.90214	5.99147	5.90521	6.24851	6.58442	6.11421
PANK3	Q9H999	6.64142	7.00297	8.21728	7.15892	6.34377	6.81783	6.68684	6.25931	7.67322	6.25009	6.78997	7.98866	3.47985	4.19475	3.94083	4.55428
SLC35E1	Q96K37	6.41143	6.52704	6.43476	7.24643	5.81925	5.63218	6.36288	5.51911	6.29735	6.12131	6.49029	6.51166	6.58069	5.65661	6.42722	5.96178
TMEM44	Q2T9K0	6.37689	6.11947	6.03193	8.15123	5.88554	6.82168	6.39696	6.04323	6.14845	6.24737	6.54656	6.69502	5.782	5.85723	4.89179	6.00466
MYL9	P24844	6.76281	6.31065	7.91669	6.10916	5.47758	6.53046	4.84063	7.05205	6.27528	6.94177	7.02439	7.61847	5.74658	6.20251	4.9015	4.28946

SH3RF2	Q8TEC5	6.12101	7.36793	6.62793	7.00712	5.34668	5.97522	5.91561	5.42739	6.86021	7.62127	6.25105	6.67786	5.47931	5.98946	5.31781	6.01414
TP53RK	Q96S44	6.12726	6.54523	6.87899	5.59206	5.81604	6.16608	5.75227	5.56506	6.27527	6.4982	6.71446	6.26436	6.58465	6.52788	7.68201	6.11016
CSDE1	O75534-3	6.57775	6.76109	7.27617	5.9809	6.11837	7.17743	6.0653	6.42165	7.87416	6.68493	6.78418	6.68573	4.86057	4.88652	4.83591	5.00935
TBRG1	Q3YBR2	5.93989	6.51394	5.61794	7.59854	6.75381	6.02062	6.18939	6.05453	6.04788	6.79637	5.92716	4.86116	6.59739	6.36639	6.85009	5.86489
TTC39B	Q5VTQ0	6.30994	5.72141	6.13246	5.8243	5.48246	6.15515	6.28783	6.76256	6.29042	6.57015	6.1626	7.02902	5.82639	6.45363	6.96517	6.02651
ADCY6	O43306	6.26483	6.42654	6.5556	7.35366	5.97369	5.85252	6.17134	5.59211	6.99099	6.20372	5.88146	6.93035	6.75841	5.83849	5.81957	5.38672
CBR4	Q8N4T8	6.61128	6.4581	5.95186	6.94441	6.01005	6.13178	5.48701	5.18736	6.42973	6.61194	5.81066	5.80141	7.03051	6.20528	6.93512	6.3935
LMLN	Q96KR4-3	6.14677	6.60869	6.17404	6.69315	6.45358	6.56526	6.26145	5.6761	6.5966	6.6212	5.8083	5.68842	6.81121	6.01994	6.01444	5.86087
ATP23	Q9Y6H3	6.38609	6.4193	6.10064	6.81049	6.65083	6.02493	5.831	6.239	6.10903	6.50112	6.28707	6.36228	5.72002	6.23965	5.44511	6.87344
AKIP1	Q9NQ31	6.6836	6.50237	6.78888	6.038	6.2074	6.58388	7.28137	6.42479	5.67454	7.93121	7.13155	7.98903	4.5332	4.35776	4.34688	5.52555
MBTPS2	O43462	5.54917	6.53423	6.68378	6.95066	6.59791	6.74162	5.91893	6.76355	6.13955	6.30857	6.78812	5.41428	5.54748	6.01378	6.60509	5.44327
CFLAR	O15519	6.59429	5.32672	6.58162	6.40503	6.01381	6.24171	6.43616	7.131	6.35812	5.92002	6.93354	6.899	6.08343	5.48778	5.54033	6.04744
NANP	Q8TBE9	6.28698	6.02457	6.81584	6.06578	5.99394	6.68286	5.50763	7.20641	6.12833	6.85423	6.09423	7.25394	5.24542	5.80579	6.07384	5.9602
ATG12	O94817	6.89466	5.90338	6.45871	5.40876	6.79344	5.83556	5.89266	7.02797	6.10576	6.25945	7.13971	6.7028	6.21961	5.6594	5.98803	5.71011
NFATC3	Q12968	6.41251	6.43224	7.03684	5.58781	6.3517	5.84727	6.09579	6.38846	6.30082	6.76434	6.91826	7.40035	5.81856	6.14087	5.01432	5.48987
ROGDI	Q9GZN7	6.28305	6.1669	6.4414	5.32907	6.625	5.78695	6.16241	6.7933	6.42147	6.40108	6.27313	6.41704	5.98882	6.82062	6.23437	6.8554
JRKL	Q9Y4A0	6.31714	5.59189	6.20963	7.15368	5.79759	6.02464	6.3899	6.80253	5.79014	6.39811	5.45325	5.63255	7.48612	6.82061	5.75974	6.37247
PPP3CC	P48454-3	7.10947	5.64593	6.78832	5.27791	6.25697	6.18406	6.04549	6.22282	6.45827	6.63552	6.6633	6.24709	6.05345	5.82415	6.61552	5.97172
ZMYM5	Q9UJ78	6.35146	6.63557	6.62203	6.24394	5.98855	6.0444	6.09013	6.00633	6.37064	6.55346	6.80261	6.55747	5.88066	6.36603	5.72101	5.76571
PARD6B	Q9BYG5	5.60206	5.84936	6.87916	6.10272	6.27914	6.25376	6.01255	6.46878	6.34405	5.80641	6.62955	6.48983	6.66383	5.88219	6.61992	6.11669
SLC31A1	O15431	6.61273	6.65134	6.31561	6.76856	6.47639	5.85821	6.07845	5.61989	6.37924	5.96142	6.63212	6.41566	5.65943	5.87099	6.34431	6.35566
SLC25A28	Q96A46	5.87026	6.07548	6.53669	6.53438	6.93168	6.68731	6.55272	6.50123	6.27399	6.53211	5.85003	5.06613	6.42759	6.49914	6.01475	5.66052
SMIM15	Q7Z3B0	6.73123	6.59297	6.37947	6.63013	5.82496	5.78784	6.13627	5.18056	6.30708	6.07745	5.84279	6.35635	6.24346	6.11059	6.58445	7.21441
VIPAS39	Q9H9C1	6.79759	6.14112	5.95956	6.54582	5.91018	5.95324	6.71865	6.45927	6.38441	6.56576	6.83666	7.01915	5.92427	5.77873	5.37383	5.63174
CRY2	Q49AN0	6.48506	7.28092	7.24019	7.28369	6.23272	6.0278	6.19556	6.02955	6.58966	6.82374	6.50786	6.38156	4.99197	5.40069	5.40595	5.12308
C8orf37	Q96NL8	5.94168	6.45415	6.56121	5.9923	5.84186	7.11767	5.81456	7.09707	6.21751	7.12287	6.22464	7.37533	5.56354	5.97431	5.16771	5.53359
MMACHC	Q9Y4U1	5.55327	6.17659	6.01315	6.73886	6.36462	6.04882	6.43145	6.65882	6.28922	7.24257	7.65606	7.75815	6.07526	5.08306	4.51216	5.39794
MAP3K2	Q9Y2U5	6.80528	6.4437	7.0073	6.41602	5.62143	7.36091	5.81153	6.64232	6.88439	6.47062	6.65708	7.18531	5.26718	4.98098	5.24161	5.20433
MXRA8	Q9BRK3-2	7.01782	6.55414	6.74119	6.80047	6.14419	6.49643	6.49157	6.60879	6.19082	6.52986	6.51649	5.51029	5.7963	5.43632	5.21767	5.94766
FOXC1	Q12948	6.15637	6.36413	7.27572	7.46847	6.73003	6.56522	6.32531	5.56652	6.26201	6.06036	6.52708	7.08374	6.14632	5.11444	4.6641	5.69017
RPUSD1	Q9UJJ7	7.12369	6.9439	8.08191	7.15521	5.8269	5.9848	6.94913	6.07226	7.30122	6.09623	7.39088	7.24654	4.53554	4.54549	3.78101	4.96528
ALKBH8	Q96BT7-4	5.9271	7.45124	7.43733	6.64281	5.96795	6.58876	6.10311	6.30518	5.9777	6.74947	7.04898	5.99792	6.13447	5.03878	5.09462	5.53458
GSKIP	Q9P0R6	5.85411	5.23374	6.04278	4.57777	5.42456	5.33324	5.90687	8.16622	4.63166	5.87985	5.23874	7.00788	5.3088	9.72459	6.86265	8.80654
TADA2A	O75478	6.98289	5.78478	6.96473	6.71564	6.25539	6.32309	5.79253	5.9203	5.84036	6.4288	5.96668	6.85774	6.31802	5.05921	5.91036	6.87948
RAB3IP	Q96QF0	5.94462	5.9214	6.51958	5.89576	6.10444	6.68495	6.70331	6.61386	6.47429	6.55343	6.83505	6.58871	5.99816	5.78996	5.64914	5.72333
SLAIN1	Q8ND83	6.55209	5.41006	6.9292	5.35241	5.84258	6.77292	6.26285	6.1118	6.13857	6.54872	6.69636	8.51369	6.60453	5.99661	5.08158	5.18602
DNAH5	Q8TE73	6.99753	6.65245	5.00497	6.16206	5.98279	6.9451	5.17917	6.92735	6.81948	6.0575	5.66646	6.5667	5.53586	6.57022	5.84311	7.08924
ZNF22	P17026	6.58259	6.4624	5.31212	6.51316	5.71445	5.62664	6.1552	5.55695	5.85212	6.11626	5.43323	6.03223	6.9416	7.43192	6.89854	7.37058
OTUB2	Q96DC9	5.94904	5.88418	6.5567	6.05486	6.25998	7.08405	5.94535	6.18193	6.20032	6.92432	6.63362	6.53899	5.93775	6.26103	5.46791	6.11999
DNASE1L1	P49184	6.10225	6.55586	6.92809	5.14401	4.76242	6.09026	6.68085	7.08184	5.8119	5.72493	6.20391	5.72079	6.5157	7.36783	7.3768	5.93256
DUS1L	Q6P1R4	5.44683	4.9009	6.98355	6.35735	5.88881	7.15253	5.90763	6.12469	6.4183	6.24135	6.51553	6.84713	5.64489	6.88856	6.34401	6.33794
RTN3	O95197-5	6.96424	7.46276	5.81831	6.67343	6.39151	6.09698	6.19554	7.54427	6.09683	5.863	6.27409	6.30178	4.77882	7.46333	4.72091	5.35423
CRBN	Q96SW2	5.15264	5.64558	6.20785	4.55824	7.05717	5.25486	6.98568	5.70392	6.26134	6.87823	7.3298	6.29567	6.55504	6.98973	6.58501	6.53923
E2F4	Q16254	6.53275	8.0471	6.60305	6.96046	7.00652	6.15735	4.69514	7.01806	5.6328	7.35504	6.65565	6.63793	5.72862	5.52502	4.47177	4.97276
HNRNPA1	H0YH80	6.36016	6.34616	5.98534	7.38038	5.47487	5.65502	6.00284	5.93239	5.74845	7.40187	6.01583	5.63221	6.4919	5.21803	7.0988	7.25575
EEF2KMT	Q96G04	6.53754	5.84479	7.48254	6.37967	6.06391	6.54056	6.46604	6.09135	7.16223	6.16943	7.02231	7.26385	5.10682	5.36865	4.97777	5.52254
GNPTG	Q9UJJ9	6.36684	5.52906	5.2475	6.58094	5.97976	6.56033	6.37213	6.15078	7.32263	5.73704	6.08482	5.89481	6.16907	6.27772	6.8783	6.84828
PLEKHF1	Q96S99	6.24667	5.28962	7.0111	7.10168	5.94311	6.39281	6.31542	6.74718	6.19809	6.17302	6.1929	7.22671	5.12132	6.28591	5.91137	5.84309
LRRC58	Q96CX6	7.63148	6.94774	8.73483	8.39221	7.38147	7.48117	6.69266	7.40958	6.12172	5.16934	7.09254	6.73017	3.37528	2.97584	3.65683	4.20714
DPYSL5	Q9BPU6	6.05403	5.89162	7.04958	5.9732	6.07474	7.10371	6.22395	6.80752	6.66344	6.72969	6.89568	6.72822	6.22816	4.96429	5.01583	5.59632
TMEM9	Q9P0T7	6.12768	6.77262	6.83146	6.50828	6.26016	6.32262	5.55982	6.39581	6.02227	6.63855	7.55105	7.18168	6.07019	5.01546	5.73537	5.00697
LDLRAD3	Q86YD5	7.16601	6.68535	6.86852	6.46254	6.27859	6.06152	6.7023	7.63748	6.32925	5.69699	6.81219	6.46142	5.04808	4.66283	4.73409	5.49283
SLC6A15	Q9H2J7	6.16071	6.76504	6.64826	6.29899	5.47958	7.29675	6.49752	6.33144	6.04663	6.58313	6.29716	6.50595	5.95676	5.50821	5.67968	5.94419
FBLN1	P23142	7.17905	7.05961	6.3585	7.54305	6.84724	6.23043	6.58829	6.52814	7.05806	6.30346	7.31218	6.34295	4.95139	4.52807	4.36913	4.80046
POLDIP3	Q9BY77-2	6.72729	6.29895	6.43019	6.00308	6.16878	5.76534	6.64244	6.68064	6.24655	5.41673	6.38614	5.60542	7.17271	6.28971	6.6349	6.12573
CENPK	Q9BS16	6.54635	5.25684	6.0196	5.05518	6.0244	5.16569	5.07137	5.91875	5.6623	6.07723	5.91817	6.9673	6.62192	7.47983	7.76909	8.44598
CEP57L1	Q8IYX8	6.2551	6.62515	7.31465	7.13482	6.34706	6.7746	6.74865	6.53181	6.63561	6.85658	6.62355	6.77598	4.68135	5.09887	4.63546	4.96077
POLI	Q9UNA4	6.1517	5.35203	7.48659	5.82594	5.92504	5.99414	5.66168	6.45901	5.84038	6.13728	6.63061	6.28137	6.59145	6.31134	6.79469	6.55676
METTL18	O95568	6.47071	6.48269	7.12499	7.35381	5.80602	6.17187	6.2856	5.81557	6.13418	7.26359	7.15136	7.13568	4.90155	5.60969	5.6909	4.60179
OLFML2A	Q68BL7	7.79898	7.35111	6.30322	6.92277	6.53781	6.6223	6.59098	6.1425	6.16139	7.58436	7.03055	6.65242	4.19382	5.19754	4.44429	4.46596
ETV5	P41161-2	4.21389	4.45753	5.80071	4.36691	6.75436	6.35531	6.1601	6.33699	6.36091	5.4579	5.36863	5.77644	8.26112	7.60957	8.23248	8.48415
SMO	Q99835	5.82325	6.95704	7.72113	6.90163	6.25443	6.43752	5.96706	6.18354	5.82355	6.55017	7.71241	6.66071	4.8217	5.38676	5.82321	4.97588
RNF167	Q9H6Y7	7.1484	6.83767	6.10691	7.03611	6.55991	5.78749	6.12627	6.61981	6.16668	6.44406	6.53668	6.31773	5.50488	5.88477	5.41415	5.50848
PEX12	O00623	6.15967	6.42231	6.02551	6.20766	6.46052	6.21021	6.27474	5.87971	6.2341	6.16352	5.81571	6.04802	6.40771	6.30628	6.65483	6.72953
PHLPP1	O60346	6.41687	6.52029	7.31493	7.13676	6.31654	5.95992	6.53259	5.86242	7.37416	6.53075	7.56215	6.64834	5.4813	4.92642	4.62899	4.78756
MSRB3	Q8IXL7	7.31448	6.03154	5.70892	6.11829	6.42623	6.35077	5.35618	6.36913	6.46647	6.15191	6.51279	5.92379	6.99963	6.09385	5.9988	6.17723
PROSER1	Q86XN7	6.30539	5.20428	6.05561	5.55295	6.83681	6.45263	6.18414	6.52807	5.69682	6.98324	6.38476	6.2191	6.74012	6.74522	6.12482	5.98602
MED7	O43513	6.38959	5.80082	5.6835	5.60259	5.75536	5.52959	5.84847	6.11638	5.05946	6.86194	5.38694	6.40725	6.96894	7.38829	6.38199	8.81888
KLF5	Q13887	5.50944	5.8597	6.6257	5.94049	6.46687	5.58002	5.21567	6.28255	6.07422	6.79346	7.19297	6.48728	6.76707	6.45499	6.55155	6.19804
BRWD3	Q6RI45	6.32655	7.38676	6.00126	7.26947	5.6464	6.1969	6.59459	5.91913	6.34688	7.22539	6.57855	6.14891	5.3302	5.45695	5.68399	5.88807
RABAC1	Q9UI14	6.61074	6.57784	6.54353	5.83304	5.84559	6.18517	6.32793	5.31305	7.00631	7.02872	7.71455	6.81202	4.94728	6.47807	4.91144	5.86472
AGPAT4	Q9NRZ5	5.89251	6.12577	6.10719	7.20357	6.34753	5.95826	6.41458	5.50034	6.52102	6.16327	5.35178	5.83797	6.54602	6.38474	7.1793	6.46616
NFKBIB	Q15653	7.15174	6.08486	7.54679	5.75726	7.2744	6.6346	5.73477	5.76195	6.45587	6.08372	7.01286	7.40865	5.99393	4.64798	5.08597	5.36465
ABCD2	Q9UBJ2	7.07136	5.31451	7.40865	6.74886	5.69606	6.41946	5.64633	5.52583	6.96431	5.27638	5.69504	6.74083	6.96113	6.06318	6.09755	6.37053
NTHL1	P78549	5.84072	5.72789	6.08849	6.56909	6.12649	6.19822	5.97551	5.76145	6.56734	6.23151	7.18622	6.67086	6.22238	6.00268	7.04516	5.786
RNASEH2C	Q8TDP1	5.91467	5.3114	5.61499	4.41625	4.54925	5.69941	6.26232	6.43607	5.0233	6.90424	6.27114	7.52461	6.40429	7.65737	7.2227	8.78801
TBX18	O95935	5.63407	4.80812	6.31574	5.97283	6.16004	6.00795	7.41408	6.06327	6.83681	7.39065	6.42339	6.4069	6.44238	5.72897	6.07366	6.32116
NPEPL1	Q8NDH3	4.8928	5.32339	4.73181	6.14836	5.65709	6.41828	7.2234	6.41561	7.49257	4.77886	4.8328	5.20609	6.36297	8.76466	7.45156	8.29975
PFKFB2	O60825	5.9438	5.75288	6.57555	6.10631	6.36253	6.33334	5.89833	6.31773	5.58011	6.73714	6.80923	6.27654	6.65772	6.28334	6.65098	5.71447
TTC7A	Q9ULT0-4	6.54911	6.97853	6.76821	7.11448	6.14928	5.97272	6.10781	6.11927	6.07526	6.09765	6.31668	6.4841	5.75362	5.49533	5.89899	6.11896
NET1	Q7Z628	6.49067	6.48858	6.41557	6.53144	6.75171	5.96182	6.40313	5.96764	6.92445	6.66844	6.0965	5.96061	6.56913	5.6957	5.87455	6.10007
STX17	P56962	6.90388	6.60849	6.03982	7.5055	6.98417	5.65401	6.63928	6.66107	6.46399	5.9203	5.91126	6.55233	6.27017	5.4328	5.26023	5.19272
PPP1R12B	O60237-6	5.50618	6.43018	6.85109	6.18618	5.89422	7.30447	6.52047	5.45783	6.58812	6.38455	6.4391	7.35482	6.1441	5.59489	6.35099	4.9928
PIK3CD	O00329	5.76037	5.83531	6.79346	6.11903	6.31826	5.61614	6.49753	5.65667	6.96689	6.03232	6.79551	6.8384	6.79774	5.50882	6.0211	5.44246
EIF2AK3	Q9NZJ5	5.50045	6.56769	4.79553	5.25794	5.8529	5.76134	5.83045	5.44171	5.92709	6.41608	5.43488	6.60575	6.94068	7.91308	7.90433	7.85013
SLC27A2	O14975	6.14461	6.16565	5.40797	6.32747	5.71715	5.262	5.95313	5.67516	5.92204	6.48856	6.43819	6.42936	6.76372	6.97706	6.87088	7.45703
OPA3	Q9H6K4-2	6.21347	6.17594	5.63543	6.32218	6.48884	5.81624	6.17326	6.61095	6.11196	5.74352	5.6745	5.51373	7.16781	6.95496	6.7339	6.66329
ATP6V1G1	O75348	6.20802	6.19101	7.57165	6.30147	6.51935	6.2447	6.54438	6.47124	6.80854	7.24343	6.70096	6.6105	5.50646	4.6809	5.51456	4.91693
C12orf60	Q5U649	6.48834	6.35547	6.06149	7.19233	6.20102	5.92253	7.27133	7.05424	5.85192	7.09983	6.95008	8.2569	4.15107	5.2888	4.90963	4.94502
NUFIP1	Q9UHK0	7.10852	5.75496	7.25034	7.64561	5.63521	5.13412	6.6349	6.03117	6.59272	4.73615	6.48038	6.18858	6.12942	6.07811	6.72421	5.87562
TMEM65	Q6PI78	6.47297	7.05361	5.89659	5.49735	6.65555	6.95399	6.87944	7.13875	5.29565	6.4304	6.60753	5.7605	5.08964	7.40299	6.66304	6.002
CUEDC1	Q9NWM3	6.93503	6.22934	6.32002	5.99956	6.04776	6.23763	6.07733	6.39236	6.45297	6.25083	6.81313	6.97981	5.54149	5.9834	5.57045	6.16888
PIGL	Q9Y2B2	7.13789	6.17373	6.0104	6.87015	6.1212	7.39003	6.12299	4.89142	6.27296	5.93972	6.24149	6.45244	6.31461	5.06315	6.8741	6.12373
IFRD1	O00458	6.00595	6.16358	7.03659	6.88324	6.18215	6.6046	6.47604	5.80259	6.69211	6.89724	6.69279	6.69668	5.67962	5.0655	5.68961	5.4317
INPP5B	P32019	5.8338	6.6724	6.48616	7.27353	6.69592	6.32268	5.94855	6.15183	6.55358	6.30792	6.91216	5.99372	6.07266	5.5355	5.8406	5.39898
NAF1	Q96HR8	5.8262	6.1399	6.93491	6.01014	5.72687	6.61997	6.30683	6.53456	5.6135	6.2364	6.44915	6.72345	5.79868	5.75671	6.87768	6.44507
AIF1L	Q9BQI0-2	6.36074	5.28811	6.97825	6.53127	6.07116	5.65961	5.96113	6.08691	6.43488	6.0759	6.57839	7.4649	6.29746	5.67235	6.08059	6.45836
SLC4A11	Q8NBS3-4	6.9812	7.49525	5.62179	6.70844	5.93307	6.73359	5.90625	6.33948	5.99659	5.8614	6.08906	5.68062	6.21083	6.80583	5.9597	5.6769
GDE1	Q9NZC3	6.23654	6.77496	5.97012	6.21028	6.19948	6.19129	6.41118	6.2573	5.96543	6.46294	5.94048	5.68535	6.61976	6.0994	6.51543	6.46006
ALKBH2	Q6NS38	5.79652	4.63597	6.05324	6.144	5.33351	5.31184	6.0496	6.25126	6.20715	6.94402	7.56129	6.61843	6.36178	6.31241	7.0131	7.40589
LAYN	Q6UX15	6.84808	5.6491	7.31313	6.29917	5.99283	5.8938	6.47365	5.28751	6.74984	6.16906	6.10144	6.43072	6.59189	6.50928	6.15329	5.53722
TRERF1	Q96PN7	6.44299	6.20203	6.04436	6.62363	6.00952	5.71777	5.82093	6.39841	6.31847	5.91986	6.45311	6.15368	6.53062	6.50235	6.49661	6.36565
FBXW2	Q9UKT8	6.05007	5.67244	6.78123	6.26298	6.03835	6.80457	6.53654	6.24066	6.09475	6.73591	6.74825	6.35906	6.0098	6.29727	5.93217	5.43596
CCDC115	Q96NT0	5.5716	5.33566	5.15017	5.19287	6.32346	5.52572	6.42839	6.95886	5.92329	7.21759	5.92827	7.61444	6.40489	6.83279	6.39897	7.19304
RNF187	Q5TA31	8.25441	8.22095	8.60242	6.82277	4.69931	6.01953	6.73031	7.15453	8.17546	8.58132	7.28649	7.10893	2.59416	3.25529	2.74487	3.74923

NTAN1	Q96AB6	6.64952	7.0235	6.36374	6.26769	6.35557	5.59491	5.97863	7.20473	5.91396	6.67238	7.10924	7.44368	5.26477	5.71375	5.02301	5.42093
ADAM20	O43506	5.92798	5.99	6.6	6.33877	6.04905	6.10375	6.20241	6.15748	6.37022	6.63609	6.6829	6.89401	5.85244	6.45668	5.65801	6.0802
PRKAB1	Q9Y478	5.93782	6.59314	6.15325	6.02565	6.56488	6.96437	5.29912	6.95565	6.54392	5.93379	6.51744	6.13954	5.7992	5.89944	6.80281	5.86997
ZBED5	Q49AG3	5.90393	6.44542	6.304	6.204	5.94409	6.43782	6.23044	6.20371	6.676	6.69209	6.43105	6.93513	5.76549	5.96077	5.99916	5.86693
ATP5MC2	Q06055-2	6.31642	6.3017	5.42731	6.03008	6.20946	6.05757	5.83977	6.25425	6.00068	6.16671	5.16716	5.63697	6.81782	7.33592	7.33376	7.10441
FAM234B	A2RU67	6.47592	6.17989	6.75303	7.44858	5.67917	6.25147	5.49005	6.2143	6.79192	5.67618	6.94015	6.82925	6.20054	6.2574	5.83735	4.97481
STK38L	Q9Y2H1	6.83109	7.89874	6.64496	6.23004	6.50621	6.27693	7.2948	5.3744	5.64258	7.06207	6.80898	6.5221	4.89117	5.37883	5.79704	4.84005
DCLK2	Q8N568-3	5.97721	6.04127	5.82966	5.75576	6.42533	6.65334	5.62309	6.07172	6.15035	6.74728	6.03255	7.05715	6.674	6.5526	6.42745	5.98123
TADA1	Q96BN2	6.66419	6.4604	5.9578	6.44919	5.96392	6.19839	6.21619	6.48797	5.95232	5.78387	5.78274	6.41092	6.67373	6.15179	6.2812	6.56538
STX11	O75558	6.17277	7.19315	6.66401	6.84322	5.60039	5.83258	6.11888	6.37504	6.41369	6.36465	6.11907	6.11968	5.98704	5.81926	6.07873	6.29783
ABL2	P42684	5.71183	6.00444	6.10224	5.83275	6.2174	6.35178	5.9633	6.55872	6.10041	6.05182	6.3589	6.04782	6.61908	6.60994	6.75041	6.71915
NQO2	P16083	6.98107	4.78573	6.6777	5.03237	6.48544	5.48328	6.63692	7.37294	5.64633	7.69203	5.62886	6.52372	5.12536	6.88771	6.47194	6.56659
PIGH	Q14442	6.60595	6.39934	6.67988	7.00351	6.51573	5.65119	5.96846	5.54622	7.27951	6.57327	5.39996	6.39169	6.19307	5.04086	7.01003	5.74132
ERCC6L2	Q5T890	5.80098	6.36728	5.86177	5.59659	5.71195	6.94407	6.43351	5.93268	6.05644	6.43385	6.46261	6.33709	6.09256	7.13014	6.54353	6.29496
NCOA4	Q13772-4	6.29225	6.38924	6.70126	7.98781	6.47074	7.04254	6.30903	5.73754	6.44072	6.07596	7.01343	6.74209	4.32613	6.23003	4.88551	5.35572
BID	P55957-2	6.33406	4.77801	5.67919	4.72233	5.25365	4.15607	4.96387	6.93916	4.03713	5.93629	5.22242	6.53024	4.91619	11.4881	8.66124	10.3821
TATDN3	Q17R31-3	5.81002	5.797	6.96844	5.74687	5.96825	6.55781	6.16082	6.44981	6.13248	7.05997	7.03625	6.99985	5.86542	5.94222	5.78378	5.721
CCDC167	Q9P0B6	7.28265	6.59194	5.96421	6.18135	5.67069	6.12982	6.65775	5.60515	5.79877	5.6658	7.47152	6.12633	6.17123	6.12452	5.76526	6.79301
CD99L2	Q8TCZ2-5	6.95698	5.7309	6.03492	5.38577	6.36977	5.87364	5.82787	6.44883	5.82174	6.45918	5.90376	5.93852	7.38513	7.10938	6.10818	6.64544
GRK5	P34947	5.34232	5.73641	5.48445	4.90499	5.28679	6.75262	5.09667	5.18224	6.77383	5.97249	6.89416	7.3628	7.50535	7.22178	6.76126	7.72183
PAGR1	Q9BTK6	6.57766	5.64542	6.20882	5.8949	5.22531	6.46324	5.60713	6.56621	5.86969	7.1562	6.51043	6.73178	5.68664	5.99235	6.63175	7.23247
BMPR1A	P36894	6.91228	7.23427	6.91367	7.11365	5.7525	5.82622	6.26093	6.89552	6.68423	7.21115	6.20214	6.25978	4.97472	4.61414	5.41882	5.72596
RPS27L	Q71UM5	6.39993	7.19452	5.48378	6.40017	7.30469	7.72734	5.75222	8.10377	5.62445	6.07759	5.66108	5.57883	5.05598	6.74717	5.51585	5.37265
LYRM7	Q5U5X0	8.11646	6.51102	6.39383	5.5803	6.63355	5.68588	5.86417	5.93766	6.24123	6.10787	6.6402	5.67406	6.74801	6.22354	6.70019	4.94205
FBXO42	Q6P3S6	6.18775	6.05701	6.65129	6.32243	5.50066	5.60306	6.47458	6.41871	6.38979	7.02605	7.4152	7.19493	5.52096	5.64985	5.04204	6.54568
KLHL12	Q53G59-2	6.37557	7.18055	6.58726	6.01137	5.5821	6.66921	5.99948	6.22077	6.52311	7.02579	6.47546	6.81777	6.21452	4.96072	5.49752	5.8588
KLHL18	O94889	6.0006	6.41649	7.59801	5.46395	5.73745	6.38426	5.92193	6.70124	6.44871	7.54622	4.60855	6.16762	6.548	5.66156	6.36599	6.42941
HSPB11	Q9Y547	6.28003	6.05355	7.14796	5.63811	6.64834	6.5888	6.38296	6.06557	6.80472	6.70201	6.89216	6.19447	5.56287	5.42756	6.06243	5.54847
TET2	Q6N021	6.84428	6.38565	6.54451	6.84108	5.87735	6.42346	5.91374	5.88069	6.28125	6.16655	6.57303	6.43861	6.18105	5.84559	5.85195	5.95121
MOCS2	O96007	6.71701	5.22431	6.54416	6.41302	5.86724	5.34965	6.40519	6.11809	6.1568	7.38159	6.69253	7.07492	5.91089	5.71471	6.34427	6.08563
GPN1	Q9HCN4-5	6.6435	6.16212	7.07291	6.18952	6.36268	6.5388	5.69798	6.96427	6.45449	6.38518	7.21374	6.42976	5.58124	5.53255	5.14789	5.62336
CHST2	Q9Y4C5	6.241	6.50113	5.45135	6.03594	5.80061	6.11216	6.04954	6.78413	6.26191	6.77689	6.1664	6.82425	5.95019	5.85373	6.59157	6.5992
STRADA	Q7RTN6	6.3388	6.27867	6.67743	6.5266	6.94805	6.50964	6.14504	6.86844	6.03882	6.70266	7.39982	7.11854	4.85725	5.75491	4.46468	5.37065
RBFA	Q8N0V3	5.67215	5.49977	6.18347	7.46697	5.09364	6.24304	6.46736	6.66482	6.62404	6.33341	5.1058	7.14332	6.67005	6.96406	5.73379	6.13431
RLIM	Q9NVW2	5.87321	6.93619	6.08689	7.07219	6.78093	6.94479	5.96571	6.4936	6.77845	6.82969	6.00748	7.05881	5.01382	4.57499	5.4998	6.08345
CLK2	P49760	4.69323	6.32414	6.4421	5.62564	6.20688	6.75599	6.51073	5.57265	6.52458	6.94184	6.08238	5.97423	5.92159	6.5446	7.27163	6.60778
PAQR3	Q6TCH7	6.1942	6.57605	6.26657	6.96615	6.48251	5.35279	6.04992	5.90858	6.57818	5.66912	5.64757	5.21218	6.72013	6.78814	6.20397	7.38393
ZNF511	Q8NB15-1	5.61028	5.92428	6.74647	5.15893	5.90371	6.63735	6.20926	6.85917	6.44008	6.13827	7.30693	6.01709	6.01764	6.17005	6.55326	6.30724
NFE2L1	Q14494	6.21347	6.47329	7.48185	7.46184	6.13919	6.46241	7.00217	6.51218	6.06607	6.35099	5.91901	6.91858	5.10655	5.66432	4.67446	5.55363
AMDHD2	Q9Y303-3	6.16461	6.49563	6.23576	5.74642	6.7317	7.03505	6.21684	7.21164	6.41392	6.3021	6.42903	6.21962	5.9945	5.97567	5.40913	5.41836
SLC25A21	Q9BQT8	6.13872	5.53785	5.63139	6.03895	6.45345	5.78115	6.3836	6.13348	5.85362	6.49482	6.32497	5.58419	7.30174	5.90268	6.85179	7.5876
ELP5	Q8TE02-4	5.97675	5.88116	6.72354	6.05567	6.17188	6.45232	6.3762	7.19209	6.60862	6.65491	6.6426	7.13407	5.80208	5.9674	5.22697	5.13373
NPHP1	O15259-4	6.22516	6.57052	6.88332	6.63323	5.90809	5.69319	6.29055	6.44117	6.34094	6.7068	6.53565	7.34419	5.32818	5.23575	5.47176	6.39152
SMIM12	Q96EX1	6.60038	5.23171	5.61819	5.1938	5.86888	5.15065	5.5778	6.33101	5.71442	5.75311	5.40129	5.90911	7.04194	7.73557	8.08105	8.79107
ITPRIPL1	Q6GPH6-2	6.26723	6.19278	6.38154	6.95537	6.3854	6.26096	6.88343	5.41847	6.4613	6.82033	6.47037	6.54224	5.54802	5.18016	6.17808	6.05433
ELF1	P32519	6.2754	6.74967	6.69231	6.6662	5.76161	5.96	6.56446	6.64415	5.70707	6.78047	6.27086	7.00472	5.59804	5.70556	5.53103	6.08845
CCDC15	Q0P6D6	6.73432	5.47625	7.01852	7.07025	5.27005	6.53283	6.51396	6.63132	7.7319	6.73031	6.44764	7.31931	4.84856	5.77457	4.70822	5.19201
GKAP1	Q5VSY0	5.8691	6.30292	7.11019	5.86796	6.05167	6.22507	5.75679	6.34319	6.74556	6.94777	7.10818	6.73739	5.50907	5.96848	5.84513	5.61153
ANKRD54	Q6NXT1	5.62617	6.1512	6.00119	5.83234	6.0434	6.5491	5.69423	6.73441	6.16293	6.42785	6.17153	6.85711	6.42808	6.01354	6.24937	7.05757
ZCRB1	Q8TBF4	6.55037	6.4332	6.30278	7.06724	5.99547	6.02689	6.63521	5.94784	6.4461	6.2548	6.28145	6.07258	6.25443	6.06208	5.63879	6.03079
KLF13	Q9Y2Y9	6.91342	6.10478	5.8626	7.38742	5.04448	6.18145	5.9162	6.99032	4.77071	6.9179	8.41388	7.2975	6.14084	4.88347	5.05824	6.11679
HOXB7	P09629	6.31794	6.53789	6.07137	7.39207	6.37733	7.05491	5.76745	5.79012	7.14327	7.31988	7.08912	6.47402	4.95609	5.05848	4.77185	5.8782
ANKRD16	Q6P6B7	6.95698	6.03481	7.17524	5.79366	6.47847	6.43649	5.8987	7.11261	6.77593	6.09898	7.09744	6.38069	5.92927	5.81933	4.72821	5.2832
KMT5B	Q4FZB7	6.43855	6.74567	6.18662	7.27258	5.55395	5.92535	6.9221	6.48746	6.3674	6.67158	6.60819	6.28645	5.48235	5.64711	5.39062	6.01402
TMEM106C	Q9BVX2	6.33236	5.56143	6.49641	6.01676	7.28819	6.69555	6.94404	7.03443	6.27923	7.2621	6.81847	6.92182	4.87698	4.55629	5.37503	5.44089
FLVCR1	Q9Y5Y0	6.66826	6.51951	6.70513	6.55669	6.41859	6.59876	6.68013	5.9867	6.47114	5.49004	6.11411	6.17691	4.93977	6.29013	5.5321	6.84905
KLHL7	Q8IXQ5	6.45835	6.1745	6.38723	7.48076	6.27073	6.15531	6.62273	5.55342	6.66268	6.45767	6.49309	6.42872	5.49093	5.63202	6.03238	5.69947
WDR53	Q7Z5U6	6.29228	5.9734	7.76525	6.42262	6.28833	6.03945	6.26549	6.9568	6.3561	6.61269	7.07187	6.95622	5.21194	5.77262	5.00732	5.00761
SPRYD3	Q8NCJ5	6.00739	6.805	7.84232	7.47073	6.23076	7.06912	6.24863	6.39882	6.84082	6.89831	6.37062	6.70959	4.01758	5.27142	4.83319	4.9857
RGP1	Q92546	6.22664	6.41221	7.0494	7.44542	6.04489	6.2725	6.3921	5.70672	6.74174	6.90324	6.55651	7.05907	4.98062	4.81509	6.01488	5.37896
APOBEC3F	Q8IUX4	6.06598	5.89843	6.56331	6.27337	6.81429	6.20371	5.61572	6.00049	5.84586	7.1584	6.22087	6.05812	6.18429	6.57321	6.39749	6.12646
WDR83	Q9BRX9	5.65726	6.57132	5.72737	5.88603	6.43704	6.34798	5.49532	6.40405	5.90159	6.3088	5.84493	6.26989	7.09839	6.97135	6.54089	6.5378
NHLRC3	Q5JS37	6.49637	6.82655	6.22987	6.96687	6.2181	5.98141	6.05312	6.07831	5.58924	5.39086	6.13678	6.20916	6.48495	6.53561	7.04955	5.75325
SRPX	P78539	6.90411	7.76173	5.50754	7.25423	5.61234	6.53112	7.2727	5.69245	6.93705	7.0517	6.30056	6.95891	5.07655	4.20008	4.48706	6.45187
CDC25C	P30307-3	6.65672	6.40515	7.6495	7.25021	6.17525	6.69047	6.78815	6.43455	6.51046	7.74027	7.11971	7.94042	3.94102	4.45797	3.6437	4.59646
STMN3	Q9NZ72	7.50723	6.92508	7.55703	7.30338	5.79294	5.81924	6.87757	6.80468	7.53217	8.92076	7.61136	8.06004	3.39039	3.41904	3.00144	3.47764
RILPL2	Q969X0	6.18188	7.05488	7.1035	6.60472	6.42813	6.63993	5.9175	6.77586	6.88129	6.60928	6.59026	7.65618	4.40896	4.99273	5.12301	5.03189
MT-CYB	P00156	5.831	6.15318	5.33863	6.59191	6.94258	6.11018	6.74089	6.21099	5.51303	6.21822	6.64522	5.58264	6.36621	7.65061	6.24274	5.86196
USP30	Q70CQ3	6.20927	6.88498	6.63989	6.74897	6.00291	6.27718	6.49495	5.92107	5.79364	6.18995	5.66609	5.53106	6.86396	6.4016	6.29118	6.0833
TGIF1	Q15583	3.87283	4.78737	4.7154	4.83855	4.94754	5.65968	4.88901	4.82098	4.86027	4.41351	4.81731	4.71454	11.7028	10.0572	10.8961	10.0069
CLCN6	P51797	6.434	6.56592	6.62855	6.05876	5.47005	5.74328	5.8153	5.62979	6.54356	6.50237	6.70368	5.98634	5.87725	7.18391	6.97807	6.77917
MBOAT1	Q6ZNC8	5.66237	6.34093	6.32343	6.8889	6.07449	5.23963	5.80726	5.88519	6.30439	6.59012	5.8532	5.76724	6.9246	6.44451	6.98851	6.90522
WWC1	Q8IX03-2	6.2521	5.93466	7.10546	6.22431	6.56232	6.56268	6.23236	6.23327	6.3479	6.31088	7.12507	6.7694	5.88836	5.23416	5.62633	5.59073
UBTD1	Q9HAC8	5.95722	6.78964	5.74645	6.57334	5.85998	6.34743	6.07744	6.22661	6.75386	6.58541	6.67285	6.83836	5.61883	6.18573	5.8709	5.89594
TAF11	Q15544	6.66505	6.08451	6.14625	6.1485	6.93945	6.13721	6.23608	6.16793	6.87099	6.29827	5.99299	5.52141	6.30471	5.25846	6.2792	6.949
FOXN3	O00409	6.90621	6.98104	6.22799	6.86208	6.2938	6.29093	5.79204	6.67026	6.02954	6.49791	7.47127	6.44765	4.83348	5.78905	5.24442	5.66233
FOXG1	P55316	6.16036	6.21928	6.00605	6.8472	6.08186	6.11627	5.83934	6.87566	6.41205	6.30298	6.2271	6.62785	6.19666	5.59626	5.52945	6.59153
ADAT1	Q9BUB4	6.77375	7.25026	7.75525	7.31214	7.26207	5.71171	6.64289	4.93065	6.09009	5.59089	5.30072	5.8152	5.31968	6.51396	6.38672	5.34403
TGFBR1	P36897	6.79701	6.69616	6.28609	6.91497	6.64041	5.9993	6.48967	6.22944	6.86704	6.19088	6.41647	5.87144	5.812	5.58902	5.6587	5.5414
C16orf72	A0A087X1J2	6.60533	6.72191	7.2052	6.60882	5.86828	6.17216	6.76145	6.38359	6.61088	6.74821	7.21878	7.35996	5.01992	4.74028	5.11117	4.86407
AURKAIP1	Q9NWT8	6.05398	6.72752	5.06674	6.49833	6.5832	6.2	6.93801	6.3708	6.57873	5.99621	6.17326	5.95994	5.5622	6.34786	6.16959	6.77361
CBX4	O00257	6.66393	7.13719	5.8675	8.00708	6.28246	5.88892	5.77803	5.80925	6.06905	6.31522	6.34677	6.47286	5.94954	5.4816	5.93421	5.99638
LAT2	Q9GZY6	6.66982	6.19234	5.47528	6.43188	5.96316	6.17214	5.72594	6.00653	6.35851	6.51006	6.38855	6.10362	6.58311	7.4278	5.83423	6.15705
SFXN5	Q8TD22	6.18128	6.08048	6.22431	6.79593	5.76938	5.82347	5.92275	5.76117	6.94099	6.50251	5.51354	5.8546	6.44775	6.55329	6.35593	7.27261
PLCH1	Q4KWH8	6.61655	7.16307	5.1937	5.76894	6.1465	6.95271	6.04838	5.934	5.84427	6.14825	6.25296	5.97004	6.45586	6.88345	6.26118	6.36013
CLMP	Q9H6B4	6.20848	6.3299	5.98269	6.78524	6.47597	6.15218	6.05453	6.5821	6.06208	5.97784	5.73764	5.96125	5.93093	6.61789	6.70417	6.43712
CENPM	Q9NSP4	6.36365	6.75067	6.87459	7.94247	6.23303	5.90192	5.67717	5.66434	6.58004	5.5067	6.92244	6.22907	5.98901	5.47007	5.57784	6.31698
MTMR3	Q13615	5.97253	6.25967	6.65353	6.44669	6.16824	7.04838	5.57407	5.56732	6.42799	6.44837	6.15509	5.61014	5.87278	5.76812	6.49069	7.53638
TXNDC16	Q9P2K2	6.89053	6.36787	6.42663	6.47485	6.373	5.91831	7.00325	5.41533	6.21718	6.04106	5.38384	6.37577	5.99602	6.55478	6.74464	5.81696
EFCAB14	O75071	6.48392	6.92704	6.52191	6.66862	5.47864	6.67249	6.36934	6.34118	6.19483	6.44567	6.12089	6.64908	5.85032	5.77756	5.55235	5.94616
RNF115	Q9Y4L5	6.52031	6.03413	6.63448	6.09287	7.16429	7.13892	6.32723	6.63711	6.49759	6.0916	6.89019	7.58405	5.11773	4.31062	5.48568	5.4732
CCDC9B	Q6ZUT6	5.16905	7.21055	6.94802	6.55631	6.40325	6.62255	6.31393	5.10937	5.9073	6.28653	5.66032	6.32586	5.7921	5.79321	6.50137	7.40027
BRCC3	P46736	5.6064	6.31435	5.8528	6.71514	6.74695	6.41272	5.93979	6.17651	6.98606	6.42225	6.47412	6.20237	6.37424	5.95756	6.27534	5.54341
OSCP1	Q8WVF1	6.53402	5.55585	7.12854	6.3573	6.52408	6.10857	5.59871	7.1032	6.4413	6.51048	7.17788	7.60198	6.23761	4.91673	4.79411	5.40964
CHKA	P35790	6.1688	6.2002	7.85762	6.52093	6.0933	6.59464	6.14156	6.59457	6.61253	6.52658	7.52608	6.94874	5.5189	5.18064	5.08872	4.42619
PTTG1IP	P53801	6.59938	6.37552	6.48059	7.08879	5.76574	5.96376	6.24249	5.72934	6.0997	7.11911	6.26176	6.2596	5.62142	6.33917	5.67478	6.37884
FAM210A	Q96ND0	7.10148	6.14576	6.11911	6.59791	6.51591	6.05685	6.44586	5.8781	5.77807	6.56131	5.58559	5.71844	6.73646	5.96438	6.43021	6.36456
BCL7B	Q9BQE9	6.56366	6.65589	6.20874	7.02882	6.30612	5.90301	5.93783	6.4708	6.02026	6.40864	6.74261	6.6038	5.84479	5.66622	5.79759	5.84121
PDCL	Q13371	6.06646	6.14363	6.75079	5.7669	6.61596	6.88859	6.16192	6.31846	6.3825	6.37356	6.58194	6.40451	6.01056	6.30533	5.47907	5.74982
ATXN7	O15265-2	6.90711	6.28421	6.11127	6.83398	5.82324	6.06895	6.43288	5.71721	6.00145	5.80706	7.15173	6.10632	5.8758	6.04392	6.35923	6.47565
BIRC5	O15392	6.41849	5.62579	6.13093	5.63007	6.80749	5.04144	6.15845	6.82346	5.71863	6.98736	7.32007	7.78715	5.42783	6.18158	5.71624	6.22503
RWDD3	Q9Y3V2	4.92802	6.99976	8.81416	5.51895	5.55075	6.73568	6.8792	6.35688	5.78843	6.82917	7.31251	5.90681	6.15961	5.83056	5.32863	5.06088
MSANTD3	Q96H12	7.37289	7.26781	6.44138	7.01221	6.7599	6.08457	6.66818	6.54479	6.40271	6.02463	5.82869	5.93245	5.80238	5.20265	5.32979	5.32498
CDK8	P49336	6.49594	6.21619	6.34872	6.9409	5.94283	6.27151	6.28039	5.88163	6.24107	6.32936	5.95685	6.66149	6.22306	5.94514	6.16553	6.09939
NIPA1	Q7RTP0	6.11496	6.09712	6.2766	6.48267	5.93339	6.00483	6.10734	5.46807	5.79508	5.98625	5.27351	6.00996	6.94509	6.86266	7.57996	7.06252

NAP1L5	Q96NT1	6.89327	6.66245	7.38665	5.65015	7.09607	6.00804	5.27373	6.77518	6.4658	6.34235	7.37361	7.12205	5.85628	5.27361	4.86908	4.95171
NLRP1	Q9C000	6.17247	6.51189	6.43416	5.97178	6.24632	6.02081	5.71676	5.70827	7.15818	5.85954	6.05025	6.53777	6.32339	5.7929	6.87864	6.61687
TMEM128	Q5BJH2	6.48296	6.58598	5.98611	6.83268	5.74705	6.42786	6.44381	5.77272	5.80857	6.32669	6.25348	6.03938	6.23747	6.27753	6.24748	6.53025
ABHD14B	Q96IU4	4.3107	5.4662	5.57069	5.16468	7.08122	5.3548	7.01174	7.5712	5.99826	7.07354	5.48204	7.0006	5.40813	7.33569	6.32479	7.84572
COA5	Q86WW8	6.84266	6.55996	6.99819	7.41536	6.61336	6.78533	6.16453	5.85519	6.34402	6.29932	6.64104	6.11547	5.76532	4.93576	5.38307	5.28143
NKX2-6	A6NCS4	6.8059	7.04432	6.47538	6.63125	6.53796	6.29144	6.76722	6.13481	6.35457	6.38498	6.57628	6.26383	6.30638	5.33464	5.79573	5.29531
DCLK1	O15075	6.46369	5.94794	7.00024	5.74904	5.44553	6.66682	6.38453	5.87638	5.86149	6.34635	6.20875	6.41363	6.39758	6.59463	6.49187	6.1515
NEO1	Q92859	6.22752	6.69371	6.47539	7.16882	5.96769	6.13354	5.77793	5.76966	6.70277	6.31689	6.36683	6.28035	6.73612	5.95124	5.83011	5.60142
LEF1	Q9UJU2	6.41242	5.82808	6.69737	6.19284	5.38392	5.47153	6.29561	5.52759	6.54928	6.67519	6.08693	6.60011	6.81877	5.92548	6.73811	6.79675
H3-3A	P84243	6.64346	6.68043	6.1227	6.49438	6.14837	6.04491	6.25788	5.90952	6.03637	5.97621	6.22626	6.32623	6.16706	6.48416	6.20312	6.27896
DOCK4	Q8N1I0-3	5.85646	6.41407	6.70955	6.71479	6.50427	6.52578	6.17703	5.57186	5.5283	7.02777	6.35186	6.76799	6.18843	6.84852	5.5581	5.25521
ABCG2	Q9UNQ0	5.6626	6.44596	5.60836	6.60879	6.31226	5.75713	5.90097	5.68966	6.19237	6.45071	6.88987	6.69796	6.32631	5.82069	6.36693	7.26945
MID1	O15344	6.25598	6.30954	6.93118	5.98677	5.84935	6.61485	6.17187	6.34708	6.28941	6.59615	6.54039	6.60205	5.66117	5.96627	5.9899	5.88803
SYS1	Q8N2H4	6.04053	6.19825	5.54306	6.32513	5.99362	5.93505	6.59765	6.35065	5.19611	6.3027	5.42297	6.34712	6.30023	7.56161	6.76863	7.11668
GCNT2	Q8N0V5	6.54372	6.37674	6.11495	6.79427	6.98324	6.23472	5.99182	7.11717	6.57478	6.47246	5.50705	5.29029	5.69953	6.28157	5.70556	6.31215
PTRHD1	Q6GMV3	6.45693	5.34853	5.68115	4.68125	5.81217	4.68968	5.37244	6.78021	5.04871	6.20201	4.86154	5.8388	4.90166	10.6348	7.94878	9.74133
HGH1	Q9BTY7	5.28141	5.88666	5.36043	4.92877	4.84137	4.91054	6.39011	6.30601	4.6369	4.31326	5.19227	6.39306	5.60914	9.74373	8.14433	12.062
BRMS1L	Q5PSV4	6.18707	6.33843	5.87404	6.81863	6.24884	6.20666	6.23581	6.02553	6.18278	6.29757	6.09194	5.90353	6.35768	6.22312	6.54794	6.46043
RNF146	Q9NTX7	5.71442	5.64737	6.67598	5.66774	5.8129	7.31792	7.623	7.01744	6.6496	6.52093	7.02968	6.91352	5.4476	5.41538	5.02248	5.52403
C6orf89	Q6UWU4-2	6.72952	6.48249	6.16663	6.48184	6.58326	6.09698	6.15089	6.3752	6.15908	6.11607	5.92178	5.72717	6.66707	5.99139	5.94962	6.401
AKAP7	F5GXD1	6.37523	6.41486	6.39902	4.5908	6.3574	5.38218	7.09748	5.7309	5.71956	6.51577	7.464	5.61229	7.1726	6.23952	6.15918	6.76922
TMEM184B	Q9Y519	6.12281	6.06568	6.27804	5.60614	6.25962	5.3757	5.72244	6.41915	6.99645	6.20076	6.35732	6.19507	6.15607	6.84914	6.93099	6.46462
ROMO1	P60602	6.48032	6.1015	5.88465	6.22996	6.7944	6.34652	6.26929	6.78057	6.28292	5.92395	5.88745	6.01268	6.7724	6.16386	6.10937	5.96015
CDC42EP2	O14613	5.87955	4.95228	5.54558	6.45858	5.18149	5.16053	5.68762	7.30219	5.30837	6.33754	6.75585	6.41674	5.13027	8.14231	7.13481	8.6063
SAYSD1	Q9NPB0	6.93407	5.49615	6.0488	6.03021	5.94398	5.80803	6.14911	6.05129	6.01282	6.48776	6.34589	6.32052	6.1237	8.6635	6.58616	6.79801
PIMREG	Q9BSJ6	5.94118	6.29631	6.34266	6.34253	6.28879	6.16416	6.39898	6.01695	6.37884	5.71344	6.07552	6.26807	6.43459	6.58489	6.55679	6.19629
ALKBH4	Q9NXW9	7.3996	5.57449	7.01381	4.32526	6.57836	6.66467	7.16948	6.36257	7.04802	6.46221	7.34308	5.33339	5.79773	5.76396	4.82971	6.33367
TMEM41A	Q96HV5	6.4248	6.71614	7.2049	6.14508	5.7903	5.62259	6.28625	6.01131	6.52372	5.67041	5.98729	6.48497	5.58876	6.22091	6.39287	6.9297
GMCL1	Q96IK5	5.87391	7.18723	5.6557	7.08742	5.29132	5.63456	6.30988	5.98612	6.75525	6.17906	6.3287	6.88771	6.27556	5.96342	6.09705	6.48711
HASPIN	Q8TF76	6.43417	6.27322	6.28876	7.31021	5.90319	5.76533	7.09727	6.4702	6.5086	6.87748	5.59945	6.14545	5.56716	5.59628	5.67672	6.09049
BTG3	Q14201-2	6.6927	6.14684	6.86057	6.22373	7.24364	6.91856	6.85006	6.97445	5.84705	6.3538	6.69596	6.92898	4.92702	5.2476	4.24934	5.8397
CHFR	Q96EP1	5.25737	5.65855	6.26975	5.9457	5.64964	6.93194	5.81344	6.34937	5.76527	5.9992	6.41338	6.84063	6.30697	6.85602	6.74501	7.19778
MOCOS	Q96EN8	6.49832	7.25536	6.72798	6.08216	6.42161	7.47303	5.8748	6.00926	6.60046	5.60269	6.93241	6.25735	5.60768	5.75138	4.9848	5.92073
IGFBP3	P17936-2	6.80338	5.72889	6.08294	7.61021	5.08722	5.90744	8.03108	5.79132	5.60302	5.02324	5.72952	6.22419	7.55281	4.80787	6.83803	7.17886
NUSAP1	Q9BXS6-7	4.91054	5.59531	6.1365	4.45593	5.52459	5.59767	6.27683	5.61325	6.6492	7.16728	7.08402	7.03757	6.47942	6.82053	7.2759	7.37546
B4GAT1	O43505	5.96485	6.44812	6.0656	6.77764	6.61611	6.73418	6.53703	6.23839	6.19717	5.966	6.20722	5.93583	5.77094	6.08622	6.24999	6.2047
SLF1	Q9BQI6	6.56487	6.79164	6.49683	7.94821	6.04412	5.5236	6.39124	6.05471	6.28319	7.11488	6.0731	6.94118	4.80187	5.44866	5.51621	6.0057
TMC7	Q7Z402	6.89705	6.11821	6.51726	6.63287	6.18967	5.42861	6.43923	5.67775	6.74729	5.91	5.70087	6.2452	6.90388	5.95363	6.447	6.1915
CCDC90B	Q9GZT6	6.04889	4.81907	4.93499	5.67597	5.21155	4.41748	5.36766	6.38222	4.52328	5.6823	4.25345	5.80621	5.51487	10.3718	10.3559	10.6344
POGLUT1	Q8NBL1	6.2982	6.71668	5.92772	6.64414	6.48836	6.28272	6.42242	6.34002	5.76062	5.94429	5.64091	5.95549	5.9901	6.53387	6.6717	6.38275
C5orf34	Q96MH7	7.22378	6.87622	8.22004	7.10436	6.07459	7.35914	7.03055	6.94163	7.20216	7.06272	7.88264	8.41134	3.14303	3.21978	2.78695	3.46108
CCDC61	Q9Y6R9	5.79592	6.23376	6.43014	6.78877	6.73122	6.56688	6.62532	6.25418	6.39937	6.34663	5.91315	6.14099	5.96992	5.62962	5.81763	6.35649
DEAF1	O75398	5.68828	6.80158	5.71276	6.43225	5.92058	6.2131	5.77213	6.22621	6.20923	6.10373	6.46068	5.69161	6.84068	6.13614	6.88202	6.90901
B4GALT2	O60909-3	6.9326	7.21924	5.14771	6.88504	5.5995	5.63422	6.61107	6.41307	6.10045	6.18566	5.95433	6.2245	6.08515	6.34946	6.14435	5.51365
PLD6	Q8N2A8	6.67135	6.59851	5.47449	7.02871	6.31231	5.83739	7.43796	6.99152	6.00316	4.57473	4.87938	5.91431	6.18526	5.52045	6.69884	7.87162
RDH10	Q8IZV5	7.12644	6.16617	6.31639	7.34308	6.25458	6.22041	6.09471	5.7026	6.54765	6.01203	7.02608	6.13696	5.89716	5.69531	5.42927	6.03118
SSH2	Q76I76	5.80243	5.98491	6.05242	5.54716	6.34883	6.16847	6.61249	6.56093	6.34353	5.94522	6.27611	7.00679	6.54375	5.80788	6.4287	5.5704
FOXD1	Q16676	6.10593	4.71639	5.72026	4.88395	6.17462	6.01927	5.87163	6.12468	5.36492	5.69772	6.95491	6.35046	8.2388	6.82444	7.74313	7.20889
RNF25	Q96BH1	6.38997	6.03819	7.19065	6.00217	6.38397	6.44532	6.41319	6.28838	6.5291	6.45239	6.57063	6.34457	6.02464	5.47596	5.66732	5.78354
BTBD1	Q9H0C5	6.88236	6.17878	6.71246	6.49424	6.56069	6.58603	7.10218	6.43872	6.26449	6.54811	7.14108	6.07858	4.85996	5.03224	5.26044	5.85964
BRF2	Q9HAW0	5.79996	5.99554	6.69749	6.03549	6.42208	5.91311	5.90031	6.2198	6.97202	6.28634	8.41985	6.45368	4.82875	6.16691	5.95356	5.93511
NDUFC1	O43677	5.73611	6.25594	5.26892	6.18517	5.93323	5.63189	5.42372	5.89849	4.90172	5.1265	4.96262	5.31633	5.76564	10.3885	7.42241	9.78284
VHL	P40337	6.77778	5.76073	6.26281	5.2053	6.8603	5.96129	6.64668	6.55371	6.46722	6.73897	5.99387	8.14063	5.26715	6.09719	5.51088	5.75548
ZNF585A	Q6P3V2	5.68971	6.47493	6.13918	6.28851	6.73113	5.66101	6.63276	5.47485	6.76402	7.4792	6.774	6.79233	6.09925	5.99956	5.13833	5.86123
AMMECR1	Q9Y4X0	5.79409	6.26416	6.13931	6.2228	5.5878	6.7585	6.14141	7.26417	6.13575	6.43249	6.92743	6.46378	5.38697	6.91243	6.02085	5.54804
PHTF1	Q9UMS5	6.58227	6.59828	5.95588	7.13585	6.05001	6.06741	6.06661	5.81592	6.42764	5.46025	6.30638	7.06882	6.55692	6.19009	5.99955	5.71912
PHTF2	Q8N3S3-2	6.47366	7.27265	6.76371	6.99283	5.80175	6.83366	6.27661	5.44204	6.81192	6.1485	6.89882	7.47509	4.83044	5.27956	4.81361	5.88517
PMVK	Q15126	5.27939	5.80807	6.94077	6.26844	6.21875	6.85557	6.04317	6.65608	6.98276	6.22733	6.77947	6.31877	5.90841	6.46187	5.71811	5.53305
CCS	O14618	6.22362	5.339	6.35799	5.13872	5.81529	5.87556	5.65988	7.2903	4.90702	6.59281	6.70888	6.88681	6.8304	7.05006	6.07204	7.25162
TCF7L2	Q9NQB0	6.51432	6.6847	6.29076	7.01667	5.59419	4.66731	5.86139	7.44369	5.92197	6.10471	6.59947	6.27956	5.86405	6.42108	6.11197	6.62416
CLSTN3	Q9BQT9-2	7.87204	6.55748	5.70717	6.49504	5.82536	7.30607	6.52827	7.53478	7.06332	7.46414	4.44142	7.14337	5.3473	6.59543	5.42763	4.49121
EMP3	P54852	6.07729	6.55896	6.20589	6.81492	6.31877	6.11579	6.48874	5.70459	5.89226	6.34339	5.38047	5.37553	6.67171	6.26273	7.03992	6.74903
ARHGAP26	Q9UNA1	6.33998	6.38397	6.04768	6.47403	6.94382	5.76821	6.9391	6.53957	5.90541	6.2944	5.81543	6.16331	5.93714	5.27205	6.63665	6.53927
ZSWIM6	Q9HCJ5	6.50825	6.20688	6.81583	6.84501	5.64453	6.79657	5.93993	7.13988	6.29667	6.56585	6.48723	6.29627	5.66837	5.6976	5.19951	5.89162
RIOK3	O14730	7.5239	6.9047	6.33026	6.64447	6.80434	5.94254	6.23602	7.46984	6.69253	5.69928	6.83765	5.9649	4.99613	5.45213	5.29385	5.20747
ACVR1	Q04771	6.46679	6.91639	5.95472	6.6684	6.10642	6.55972	6.7599	6.11223	6.456	6.82609	5.60987	6.61419	6.10901	5.2753	5.74528	5.81969
FBXL19	Q6PCT2	4.96134	6.07187	6.42072	6.41204	5.34943	5.69831	6.13666	5.50287	6.40615	6.69467	5.91335	5.89818	7.12833	7.44309	7.0069	6.9561
DNAAF2	Q9NVR5	6.64423	6.11965	6.45033	5.60625	6.35354	7.69484	6.03342	6.60319	6.77134	6.82006	6.94555	6.40776	5.21385	5.72666	5.17095	5.43838
MEIOB	Q8N635-2	6.73402	7.22907	7.1856	6.53128	6.49508	6.50443	5.76096	6.56136	6.58523	7.04813	7.35148	8.39495	4.24959	4.61399	4.04297	4.71185
CCND3	P30281	6.75081	6.96616	7.47901	6.61565	6.22566	7.77859	5.55656	6.39173	6.79309	8.39498	7.9913	8.3196	3.47561	4.2734	2.84748	4.14036
AHI1	Q8N157	6.06903	5.36686	6.40706	6.61761	5.65108	6.37228	6.34191	6.13508	6.44229	5.95562	7.12806	7.24618	5.61548	6.49119	5.96918	6.1911
VCAN	P13611	6.29006	6.55155	5.43723	7.95826	5.60672	6.07543	6.90152	6.23445	6.86127	6.06533	6.27132	6.62313	6.33688	5.23289	5.37056	6.1834
TXNL4B	Q9NX01	5.15314	4.60211	5.80856	4.50959	5.35259	5.13056	5.63979	8.07045	6.06131	7.12716	6.50364	7.62126	4.46994	7.96129	7.50549	8.48311
PACC1	Q9H813-2	5.68272	6.15837	7.05865	7.04709	6.3474	7.16854	6.47265	5.77001	6.23401	6.59633	6.70267	7.57409	5.31032	5.56019	4.91093	5.40602
PCDH9	Q9HC56	6.68561	6.60497	6.44227	6.39962	4.82583	6.42315	6.32945	6.26395	6.28294	7.21637	6.01129	5.27526	6.39337	6.23677	6.74906	5.86009
SARM1	Q6SZW1	5.95982	5.79816	6.872	6.92721	6.07411	5.57303	6.3993	6.78499	5.57908	6.7724	6.33059	5.98485	5.72709	6.26573	6.22785	6.7238
CCDC120	Q96HB5-4	6.50424	6.30713	4.80366	5.79838	6.2502	8.40816	5.3832	5.96785	5.80594	6.92821	7.17408	6.81273	7.09224	6.1497	5.76606	4.84821
DBF4B	Q8NFT6	6.28241	7.33012	7.19179	8.10912	6.53093	7.60029	7.70804	5.38531	6.34761	6.93709	6.7713	4.99711	4.84613	4.47191	5.4465	4.04435
FOXP4	Q8IVH2	6.12467	6.33505	6.98664	5.69267	6.10697	6.57134	5.94793	5.54028	6.38373	6.97611	6.50297	6.78825	6.23097	5.9488	5.91898	5.94463
ULBP2	Q9BZM5	5.62034	6.6614	6.99858	6.52643	5.93657	6.43296	6.9838	5.86259	7.73591	4.76874	7.14906	6.71233	5.59214	5.97825	5.44998	5.59091
NUDT4	Q9NZJ9	6.39597	6.1165	6.59722	6.10276	6.36878	7.45075	6.08042	7.22223	6.57068	6.83455	6.61942	7.01539	5.06654	5.37306	4.78515	5.40058
USP43	Q70EL4	6.37577	6.06585	5.19891	7.46209	6.63806	6.08383	5.40459	5.77234	6.20678	6.46861	5.59123	6.04572	6.38432	5.96391	6.97024	7.36774
UFM1	P61960-2	6.15249	5.90374	5.88682	5.47468	5.43207	5.23644	5.57596	6.42193	5.41312	6.19756	6.03629	6.67197	5.25177	8.46002	7.24114	8.64402
PPP1R3D	O95685	6.33711	6.5831	7.26277	5.86628	6.42334	7.1958	6.38282	5.82114	6.91269	6.2995	6.97007	6.62452	5.20217	5.60083	5.22835	5.28949
GPR155	Q7Z3F1	5.77891	7.00082	6.58732	5.96862	5.48964	5.69195	6.25253	5.87293	6.388	6.40646	5.77641	6.5288	6.05703	6.97377	6.11804	7.10876
TM7SF3	Q9NS93	6.74484	7.4054	6.39169	6.42077	6.86233	6.24964	6.40715	6.81586	6.54643	6.33061	6.34386	5.6163	5.25881	5.45237	5.4328	5.72115
ZNF771	Q7L3S4	5.73696	6.39418	5.98103	6.24341	6.25444	6.2407	6.44972	6.22243	6.05301	6.38791	6.3446	6.2687	6.06472	6.3071	6.69907	6.35202
TMEM129	A0AVI4	6.33204	7.48931	6.52788	6.64827	4.88519	5.84561	6.696	5.45854	6.45336	5.85751	6.89504	6.18885	5.70146	6.09507	6.64384	6.28202
PEX2	P28328	5.86509	6.20915	5.86639	6.20803	6.44618	5.85877	7.19962	6.06567	6.3136	6.00437	5.73566	5.73378	6.88934	5.85934	6.89348	6.85153
ZNF689	Q96CS4	6.19152	6.45249	6.1069	6.27517	5.82475	6.6639	5.98119	6.204	6.07536	6.2954	5.83496	5.97232	6.34508	6.52173	6.81892	6.43632
GUCD1	Q96NT3-3	7.34872	7.27642	7.20862	7.78889	5.63745	6.41672	6.66995	5.29731	5.93976	6.79494	7.25461	6.2731	5.38025	4.85051	5.19271	4.67003
LMBR1	Q8WVP7-3	5.92345	6.262	5.93768	6.46938	5.74336	5.91811	6.70665	5.85132	5.98725	6.29071	5.49386	5.73989	6.58033	6.51033	6.79759	7.7881
ZDHHC6	Q9H6R6	5.92419	6.58405	6.01782	7.12622	5.80916	5.49847	6.08124	5.90749	5.91577	6.30568	6.28641	6.43726	6.09386	6.47065	6.85047	6.69126
RBKS	Q9H477	5.99514	6.10154	7.42589	5.744	6.52452	6.45417	6.82272	6.40076	5.7678	6.08493	6.36607	5.9113	6.12461	5.86176	6.81286	5.60192
FZD1	Q9UP38	6.77842	6.16139	6.63437	6.82147	6.7315	6.23943	6.94873	6.65792	6.0227	5.88459	6.19307	6.23841	5.30512	5.68413	5.71154	5.9872
COCH	O43405	7.3066	7.58152	7.16278	7.15406	5.37308	6.73033	6.88655	5.27223	6.9194	6.98892	7.58704	7.20674	4.77388	4.24905	4.34836	4.45945
SUMF1	Q8NBK3	6.4469	6.53646	6.23192	6.43087	6.2926	6.44231	6.16367	5.99125	5.19879	6.00878	6.20406	6.53531	6.58509	6.03919	6.27335	6.61944
PTCD2	Q8WV60	6.52892	6.78885	6.07548	6.28297	6.22195	5.89961	6.78509	5.26639	6.06265	6.21222	5.59881	5.99027	6.78415	5.91926	6.75922	6.82414
POMK	Q9H5K3	6.08621	6.42071	5.79393	6.67189	6.28737	6.3242	6.3005	6.36752	6.00707	6.52134	5.6339	6.26789	5.5608	6.40509	6.2	6.1516
ARHGAP32	A7KAX9	6.87043	6.90846	6.23985	6.46945	6.27003	6.04631	6.3653	6.64516	6.57452	6.14426	5.52575	6.01684	6.22656	6.44043	5.51198	5.74465
MRPL57	Q9BQC6	5.98155	5.28558	6.4216	7.04747	7.43148	6.10162	7.07488	6.66565	5.94818	6.0883	6.4299	5.90109	5.0846	5.86053	5.10486	7.57273
NUDT7	P0C024	6.79666	6.17592	6.10375	5.53076	6.47255	5.98441	5.94097	6.31921	5.99962	6.09534	6.2188	6.00483	6.47238	5.86667	7.05811	6.96001
RPIA	P49247	4.82064	5.96626	9.0476	5.6115	6.58426	5.13751	7.837	5.99107	7.58021	6.46362	8.64103	5.91449	4.72245	3.96286	7.59166	4.12784
CLOCK	O15516	6.79696	6.45363	6.50724	6.24972	5.94872	6.37664	5.73422	5.85078	6.17048	6.57367	5.61113	6.0245	5.48475	6.5911	6.36926	7.25721
TMUB2	Q71RG4	6.68596	7.62591	6.86987	6.47702	6.28811	6.68602	5.35415	6.18004	6.65448	6.80562	6.82797	6.54013	5.47706	4.7055	4.91861	5.90353

CNTNAP3	Q9BZ76	5.68625	5.09631	6.41663	6.94209	6.00408	5.40938	5.76296	7.27264	7.71819	6.62961	6.47367	6.49279	6.23995	7.2717	5.34842	5.23532
DCTN5	Q9BTE1	5.70289	6.42574	6.56468	5.88184	6.45028	7.1345	6.19733	6.8347	6.80871	6.75862	6.33067	6.23424	5.36159	5.79414	5.78531	5.73474
C12orf66	Q96MD2	6.24432	6.55884	6.30828	6.43723	6.51005	6.69121	6.1238	6.9523	5.75749	5.85946	5.73391	5.74408	6.46464	6.45774	5.84432	6.31231
PTS	Q03393	6.43144	6.38894	6.58539	5.29121	6.70276	7.05969	5.98477	7.75305	6.45795	6.56768	6.4845	6.54741	5.58268	5.85642	4.9356	5.37052
FANCM	Q8IYD8	7.64554	7.16799	7.39159	7.30074	6.68768	5.51454	5.93812	6.28842	6.9848	6.4363	5.18552	6.51514	5.78895	4.68776	5.31068	5.15623
SLC43A2	Q8N370-3	6.33287	6.64088	6.0884	6.61681	5.65132	5.77416	6.09931	6.15885	6.71542	6.83623	6.69116	7.30016	5.48187	5.50089	5.5347	6.57797
LIX1L	Q8IVB5	5.70584	5.97026	6.20996	6.13954	7.0603	7.55212	6.06172	5.75651	5.45531	7.07178	7.59501	6.82322	5.40797	5.39686	5.69316	6.10044
JMJD7	P0C870	5.86041	5.10316	5.97163	5.02998	5.88973	6.09248	5.46732	7.53664	6.00007	6.69616	6.84226	7.36214	6.72111	7.37245	6.21392	5.84054
ETNPPL	Q8TBG4	6.02284	5.99471	5.46367	6.52479	5.76731	6.32327	6.94021	7.02097	5.63242	6.88041	5.81164	5.55463	7.05907	5.98763	7.0336	5.98284
AFAP1L2	Q8N4X5	5.47977	5.53744	6.63784	5.44981	6.51583	6.92207	5.86835	6.01009	6.42838	6.26449	6.38849	7.32017	6.09375	6.56331	6.55632	5.96388
SLITRK5	O94991	6.36818	7.67409	5.51821	5.71512	5.48712	6.23295	5.0231	7.09325	7.2265	6.47279	6.89387	6.69763	6.47391	5.95509	5.37922	5.78898
TTC8	Q8TAM2	6.93162	5.99794	6.61914	6.11811	6.35675	6.58228	5.98692	7.88439	7.4009	7.49508	7.36844	6.58872	4.74657	5.45401	4.20503	4.2641
ZNF580	Q9UK33	6.90385	6.90161	6.64574	7.17218	6.8088	6.39548	6.48785	6.27495	7.19968	8.1336	7.73847	7.61678	3.62568	4.33279	3.71968	4.04285
RIPK4	P57078	6.4553	6.09975	6.56684	6.40365	6.60109	6.75233	5.80352	6.39581	5.78542	6.20815	5.96454	5.61982	6.41176	6.74559	6.60133	5.58511
SAMD9L	Q8IVG5	5.96799	6.31926	5.83728	6.1382	5.72877	7.27229	6.47196	6.94815	6.71029	6.45161	6.36212	6.28867	5.5823	5.97234	5.9269	6.02188
LRRC14	Q15048	6.2323	6.10856	6.20902	5.39621	6.30151	6.79535	5.869	6.3401	5.84551	6.44038	6.95215	6.51351	6.42149	6.51884	6.58733	5.46874
STOML1	Q9UBI4	6.83954	6.94527	6.45416	6.45969	6.5263	5.97251	6.19402	5.67973	6.84234	6.03981	6.31685	6.29641	6.48962	5.88615	5.18019	5.87743
SIK3	Q9Y2K2	6.37442	6.62581	7.8614	6.59324	7.15121	7.50223	7.87051	7.94231	5.40515	5.22065	5.52204	5.60759	5.33905	4.46732	5.90658	4.61049
CLN3	Q13286	6.13839	7.0343	6.51524	6.50327	6.13104	6.76879	5.92047	6.26826	6.42161	6.81264	6.00822	6.67956	5.72527	5.59719	5.95747	5.51827
CABLES1	Q8TDN4	6.61588	6.08611	7.78828	6.2373	5.63896	7.08606	6.79477	7.27355	7.10092	4.73639	5.72207	7.0165	5.78622	4.76351	5.52801	5.82548
C7orf25	Q9BPX7-2	6.38478	6.32778	6.37898	6.04412	6.06439	6.71581	6.0492	6.71912	6.3795	6.53137	7.34225	6.4941	5.24482	5.82713	5.69304	5.80362
TSPAN15	O95858	5.62051	5.99356	6.09622	6.62883	5.74282	6.34826	6.26347	5.98249	6.53258	5.90856	6.18544	6.18772	6.55428	6.51231	6.68675	6.75621
CDC14A	Q9UNH5-2	6.57231	6.08635	6.95032	6.39573	6.41829	6.66852	6.38963	6.69734	6.4536	6.72174	6.65894	6.60998	6.54675	5.3917	4.91033	5.42948
ZNF143	P52747	5.88825	6.40768	6.51929	5.99238	6.04	6.26087	5.90494	6.0899	6.60175	5.95996	6.17551	6.01329	7.15962	6.57368	6.30334	6.10952
TNFRSF12A	Q9NP84	7.09673	7.13605	6.94075	6.59495	5.15217	5.02194	6.23721	6.54697	6.16353	7.58979	6.62804	8.24207	3.27346	6.44541	4.87456	6.05637
FAU	P62861	6.80834	6.45377	6.37771	5.69442	6.77682	6.31149	6.8826	6.47286	6.46549	6.15458	5.70167	5.70602	5.80265	6.63115	5.72583	6.0346
RNF166	Q96A37	6.35797	7.06907	7.58323	6.88946	5.22163	6.09074	6.39043	6.61441	6.74587	6.65944	7.20548	6.40996	4.74572	5.81335	5.1783	5.02495
LCA5	Q86VQ0	6.94386	6.16351	6.08688	6.62406	5.91229	6.15378	6.19056	5.9998	5.88996	6.32365	6.56778	6.55197	5.96888	6.03592	5.95455	6.63254
CASZ1	Q86V15	5.83602	6.03749	6.53199	6.74538	6.12274	5.85953	6.19379	5.91301	5.98044	6.53059	6.35935	6.4697	6.3892	6.09968	6.58222	6.34889
TTLL7	Q6ZT98	6.43613	6.42868	7.68643	7.05859	6.38932	6.22674	7.49225	6.55709	6.60347	6.54778	6.72565	5.95083	3.75642	5.58372	5.51754	5.03935
NAALAD2	Q9Y3Q0	6.80906	6.28759	6.01245	6.81318	5.77806	6.25391	6.43944	6.64391	6.12536	6.24692	5.27097	6.68262	6.02506	6.20304	6.55971	5.84872
TYW3	Q6IPR3	6.00241	6.99104	7.16818	5.99651	6.46018	7.45313	6.30813	6.3283	6.24005	6.02011	6.58822	5.8678	6.03636	5.85399	5.54904	5.13656
HFM1	A2PYH4	5.99454	7.18182	5.29055	6.19277	6.05757	6.18802	5.86777	6.30108	6.04747	5.84066	4.65498	5.31268	6.10597	6.81103	8.36079	7.79231
WASF1	Q92558	7.36275	6.29415	7.05849	6.14487	6.49655	6.59436	6.72221	6.13186	6.63227	5.82395	6.5156	6.88695	6.24074	4.64386	5.26423	5.18717
PECR	Q9BY49	6.28621	5.97609	6.02203	6.7758	6.18941	6.39894	6.64368	5.38657	5.72513	6.03741	6.14188	6.32307	7.22279	6.40402	6.10787	6.35912
EAF1	Q96JC9	6.11857	6.14874	6.49975	6.26689	5.98397	5.92417	5.68326	6.16415	6.26548	6.35411	6.51285	6.53256	6.17146	6.35436	6.27894	6.74075
LSM3	P62310	5.91132	4.74138	6.02083	5.60637	5.18327	4.59796	5.39772	7.19933	5.46866	6.716	6.20856	6.45417	6.62098	8.401	7.20271	8.26974
IGFBP2	P18065	7.30449	6.56042	6.69782	7.33712	5.93913	6.02479	6.10529	7.29789	6.65738	6.37744	7.12057	7.59119	3.76628	4.47942	6.13715	4.60361
IFRD2	Q12894-2	6.09938	5.98414	6.13141	6.51699	6.37347	6.53529	6.34654	6.39148	6.66996	6.62288	6.48097	7.13454	5.6178	5.83015	5.91182	5.3532
KPTN	Q9Y664	6.1821	6.20211	6.55915	6.00932	5.96307	6.81023	6.16527	6.75415	6.29886	6.43937	6.56608	6.6948	5.81265	6.23985	5.82148	5.48151
CLEC16A	Q2KHT3	6.56891	7.02817	5.62106	6.07234	7.12842	5.05261	6.77775	7.37077	6.03567	6.7107	6.19929	5.50856	6.69981	5.77655	5.18093	6.26845
ZNF467	Q7Z7K2	6.0634	6.08041	5.62335	6.39022	6.55851	6.30997	5.75834	5.93059	5.94193	6.98465	6.5373	6.32527	6.35375	6.71946	5.94803	6.47481
TJP3	O95049-3	7.00404	5.40943	5.76615	6.43491	5.76416	5.12007	6.9326	6.01456	5.41615	6.47966	6.2688	6.27143	6.84028	7.31384	6.91324	6.05067
ZNF526	Q8TF50	5.17041	7.00568	6.5231	5.9499	5.42881	6.02474	5.40789	6.71809	5.703	6.69013	6.03103	5.90323	6.54865	6.80775	7.17498	6.91261
ZNF226	Q9NYT6	6.45279	6.63124	6.32503	6.51636	6.11145	6.62033	6.83024	6.36183	6.47725	6.99948	6.1087	6.22811	5.29377	5.67435	5.48634	5.88274
ZNF234	Q14588	5.77136	6.266	5.60333	7.26625	6.51951	6.39023	7.5774	5.88643	6.38594	6.01662	5.31689	5.87623	6.41924	6.6146	6.15548	5.93448
SLC35A4	L0R6Q1	6.52862	5.25697	5.49594	4.93503	5.48163	4.83296	6.24563	6.75653	4.88285	5.54585	5.38147	5.74534	5.73818	9.33973	8.64806	9.18518
PLEKHA8	Q96JA3	6.8904	7.38286	7.08132	6.10188	6.12716	6.92098	6.71014	6.01723	5.56128	6.57728	6.50492	7.07818	5.28637	5.60183	4.70263	5.45556
ZNF597	Q96LX8	5.92473	5.63093	6.69205	6.30313	6.07786	5.25376	6.51294	6.2456	6.6532	7.06352	5.74608	6.84006	6.44196	5.43394	6.11226	7.06798
CEP76	Q8TAP6	6.60792	6.31356	6.72275	5.97143	6.28333	6.60655	6.29734	6.20625	6.3779	6.5141	6.99896	7.19817	5.59033	5.40888	5.61749	5.28502
NOL4	O94818	5.64529	6.23764	5.73639	6.42	6.57275	5.72579	6.49199	5.67734	5.609	5.84123	6.40493	6.72905	6.7939	6.28911	6.8423	6.9833
SLC26A11	Q86WA9	6.43577	6.19635	6.01365	6.72369	5.81215	6.14335	6.15781	5.65453	6.11983	6.61361	6.40087	6.45671	6.31126	6.24637	6.35352	6.36052
PEX16	Q9Y5Y5-2	6.27394	5.89481	5.94436	6.58448	5.64546	5.78033	6.51622	6.02735	6.37262	5.54751	6.51169	6.42802	6.36662	6.63843	6.54005	6.9281
ACADL	P28330	5.89551	6.79369	6.48006	6.27457	6.94562	5.91955	5.95085	5.86194	5.66596	5.69739	6.06198	6.51355	6.5898	6.6851	6.31534	6.3491
ZC3H10	Q96K80	5.98519	6.23085	6.15517	5.98503	5.85958	6.35462	6.25591	5.99274	6.30949	6.22499	6.61252	6.45981	6.57147	6.03138	6.47924	6.49202
GGNBP2	Q9H3C7	6.64644	6.96337	7.22151	6.7331	6.30543	6.43985	6.25238	6.27022	6.91953	6.94931	6.95417	7.46317	4.58362	4.88639	4.46044	4.95109
TK2	O00142	6.45204	6.98562	6.06715	7.13	5.73953	5.7651	5.98858	5.94806	6.63328	6.23428	5.92564	6.27842	6.63678	5.42893	6.55801	6.22858
ZC3H12A	Q5D1E8	5.75408	6.62507	8.14242	6.1342	5.23934	7.17915	6.36981	5.47166	6.76928	6.38538	7.47584	6.56486	5.22682	5.34212	5.68577	5.63419
ZC3H12C	Q9C0D7-2	5.57184	6.71385	7.1903	5.24787	6.565	6.97619	5.22783	5.42278	5.90866	6.47108	8.0121	8.80398	6.44237	5.04259	5.00757	5.39599
CRIPT	Q9P021	6.21719	6.11194	6.47776	5.96433	6.74873	6.8959	6.26239	6.26604	6.61739	6.8657	6.78709	6.08762	5.48755	5.95096	5.57052	5.29289
RPS6KC1	Q96S38	6.57807	6.81846	6.73632	6.39556	5.94461	6.59211	5.623	5.83759	6.03124	6.40309	6.90242	6.456	6.41904	5.83033	5.86779	5.56439
CCP110	O43303	6.33666	5.6453	5.83809	6.01143	6.03453	5.75469	5.60561	5.71229	6.46269	6.07052	6.21795	7.59056	7.26874	6.87335	6.6073	5.9703
GPC2	Q8N158	5.92078	6.75049	6.18836	6.78589	5.69324	6.76603	6.16439	6.07974	5.53259	6.36759	6.33696	6.73739	5.97667	6.19576	5.78992	6.71419
NABP1	Q96AH0	6.86585	6.18775	6.68104	7.06821	6.68307	6.19487	6.37809	7.10749	6.16454	5.40763	5.95207	6.37603	5.77277	5.87326	5.88328	5.40405
BORCS5	Q969J3	5.58308	4.99435	5.16849	5.36374	5.38516	4.66205	5.3083	6.35803	5.03496	5.98421	5.14879	6.59044	6.19856	9.48635	8.94479	9.78871
LRRC28	Q86X40	7.06411	5.63608	6.25761	6.27465	6.35073	7.49721	6.74506	5.86975	6.70233	6.17249	6.64498	6.77193	4.90943	5.95914	5.87196	5.27254
HPSE	Q9Y251	6.33446	6.69447	6.32257	5.76846	6.03907	5.91933	6.3937	6.13394	6.562	6.74503	6.555	6.08826	6.26079	6.02612	5.6961	6.46069
ENG	P17813	6.23983	6.37633	6.3858	6.90964	5.84015	6.20163	6.27922	5.70103	6.36816	5.98848	6.69444	6.54656	6.5334	6.10572	5.7718	6.0578
CASD1	Q96PB1	5.68813	6.39725	6.28661	6.56965	5.75901	6.14322	7.68181	5.86623	6.36843	6.52454	6.07698	5.67585	6.18972	6.13071	6.88682	6.65506
PLPP2	O43688-2	6.12926	7.33662	6.31184	6.46099	4.76709	6.27527	6.13908	5.8625	6.61242	6.99305	6.39438	5.81496	4.93643	6.81365	6.28692	6.86553
NLGN2	Q8NFZ4	7.25664	6.83447	6.81358	5.77044	6.51073	5.97914	6.16301	6.34687	6.33102	6.91106	6.26039	5.99859	5.38366	5.53782	5.87053	6.23205
PPP1R14A	Q96A00	6.57351	5.99475	6.09984	5.77805	6.66968	6.64379	6.50187	6.10372	6.41556	6.97732	7.13583	6.12243	6.48972	5.05229	5.882	5.55962
AGFG2	O95081	6.75746	6.29121	7.37214	5.25573	6.30918	6.31157	5.7145	6.47656	6.30266	6.48024	6.28974	6.8253	6.77035	5.68339	5.9161	5.24387
PLEKHH3	Q7Z736	6.86465	6.4457	6.63589	7.09184	5.7073	6.99834	6.71708	6.10828	6.82723	7.54839	7.23081	6.52983	4.56089	5.48598	4.60346	4.64433
VSIG10	Q8N0Z9	6.83385	6.27521	6.83644	5.72393	5.9659	6.51108	5.12768	5.80153	6.43228	6.9687	7.41502	6.3821	6.52705	5.69981	5.96193	5.5375
NEIL2	Q969S2	6.08132	5.26302	6.44442	5.44534	5.7441	6.41486	6.1972	5.29442	5.13795	7.417	6.06612	5.98456	7.02355	7.21961	7.5036	6.76292
GAS2L1	Q99501	6.10479	6.848	5.55555	4.85909	5.41078	6.05789	8.49828	6.03022	4.62796	6.58208	5.38757	5.18826	6.50059	7.3081	7.90457	7.13628
SLC35F6	Q8N357	6.94001	6.54934	6.6369	6.17595	5.65685	5.62703	6.66139	5.63514	5.49023	7.44873	6.8393	6.71453	4.85413	6.18789	5.68501	6.89758
PHLPP2	Q6ZVD8	7.18052	6.6795	6.73614	8.07886	6.70938	5.786	5.8637	7.28834	7.25679	5.81981	6.60872	6.86246	5.10381	4.95671	4.41773	4.65153
BNIP2	Q12982-2	6.77215	7.53835	7.02487	6.77218	4.76621	7.65922	7.25809	7.03751	5.359	4.55126	5.43291	5.65678	5.6126	5.29264	6.94181	6.32442
RIMS2	Q9UQ26	6.3806	5.62462	7.28832	6.72547	5.98238	7.15019	6.17465	6.30722	5.39404	5.6359	6.59722	7.58011	6.18551	5.72742	5.78206	6.45429
BBS10	Q8TAM1	6.44966	6.23629	7.15505	6.0839	6.43176	6.65437	6.94292	6.58471	6.38536	6.48129	7.36487	6.13358	5.51048	5.16983	5.15578	5.26016
TMEM164	Q5U3C3	5.5858	5.55447	5.48869	5.97722	5.45297	5.65339	6.08466	6.07639	6.52166	5.71598	5.82665	6.61358	7.24814	7.28676	7.60184	7.31179
ORMDL1	Q9P0S3	5.69874	6.78127	5.27392	5.43184	5.79527	6.38641	6.01864	5.56132	5.36173	6.34231	5.52213	6.62486	5.72879	7.53826	7.8196	8.11491
SLC25A14	O95258-3	7.11661	5.47081	5.91832	6.89489	6.37051	4.58378	6.36978	6.81128	6.21914	6.3981	6.58725	6.53686	7.36836	6.12365	6.82238	5.30829
SNF8	Q96H20	6.33455	6.46453	7.01944	5.92672	6.11331	6.6122	5.91112	6.8281	6.19731	6.78153	6.84602	7.24379	5.20317	5.78087	5.26821	5.46911
FCF1	Q9Y324	6.24063	6.61843	5.7342	7.02652	5.34358	6.19144	6.35448	6.69511	5.66235	5.96712	5.5081	5.70973	6.73743	6.79778	6.50615	6.90694
ADAT3	Q96EY9	6.00977	5.40756	8.20099	4.97966	6.8255	6.75245	6.643	5.83952	6.49827	6.52458	6.98152	7.06886	6.33721	6.04762	5.72696	5.05653
DDX43	Q9NXZ2	6.59479	6.31808	6.76341	6.0568	6.16564	6.64924	6.60897	6.42576	6.13723	6.02002	6.45932	6.67843	5.91086	5.45579	6.008	5.74767
UST	Q9Y2C2	7.62993	5.62166	5.18121	6.72648	6.11934	6.4277	5.96592	5.41006	6.30502	6.49444	6.23363	6.28697	6.59207	5.77902	6.41762	6.80893
SLC25A30	Q5SVS4	6.30367	6.52743	5.73315	7.15637	6.35466	6.32361	5.82567	6.64355	7.20652	6.55235	6.26318	6.10572	5.7269	5.98937	5.62444	5.66341
LACC1	Q8IV20	6.13006	6.23082	7.06647	5.9598	6.15622	6.83465	6.45875	7.39501	7.22257	5.56545	6.92419	5.5953	5.90592	4.94541	5.86372	5.74567
TMEM167B	Q9NRX6	5.44722	6.37865	6.4242	6.31247	6.51233	6.184	6.02394	6.27245	6.36549	6.04489	5.7843	6.19601	6.8817	6.14795	6.66666	6.35774
PRRT3	Q5FWE3	6.16685	5.5279	7.15148	6.60252	6.21212	7.05169	6.11167	6.30713	6.60034	6.17077	6.52412	6.23523	5.92657	5.36127	5.74088	6.30947
FKBP1A	P62942	5.36908	5.70332	6.40589	4.80858	6.44616	6.5044	5.72531	7.7935	5.61471	7.25668	7.24185	6.78525	5.53726	6.37708	6.04455	6.38637
WDFY2	Q96P53	6.49546	7.16031	6.50566	5.91391	6.48033	5.98466	6.01023	5.90072	5.60308	6.19508	6.49761	5.92958	5.73636	6.34351	6.96168	6.28182
DCAF12	Q5T6F0	5.40815	5.94371	5.88235	6.09145	5.65415	6.0569	5.75892	5.77723	6.51196	5.75213	6.27021	7.44234	6.1317	6.78139	7.10691	7.4305
NABP2	Q9BQ15	6.77143	6.36339	6.66186	6.42218	6.35024	6.03183	5.71772	5.75371	6.37107	7.04134	5.90132	6.03911	6.69367	5.3448	6.46269	6.07364
RPGR	Q92834	5.92454	6.90628	6.74663	6.90249	6.89021	6.55131	6.31281	6.47989	6.14519	5.74049	7.64496	6.86339	4.87953	5.54219	4.56248	5.9076
CAMLG	P49069	5.90548	5.77728	6.56147	7.78456	6.14439	5.35442	6.6325	6.19675	6.30081	5.85527	6.63034	5.56669	6.25044	6.68634	6.0079	6.34537
CNTROB	Q8N137-2	6.94308	6.46106	6.94688	7.38314	5.90091	5.87761	5.56563	6.17348	6.13704	6.0583	7.3389	6.93401	5.93324	4.60102	5.85201	5.89369
MND1	Q9BWT6	5.65605	6.48184	6.78332	5.80407	5.99007	6.45452	5.58702	5.89604	6.11758	6.53823	7.43454	7.11299	5.96678	6.46128	6.01643	5.69925
CDCA4	Q9BXL8	5.97046	5.21269	6.46786	5.41481	5.95257	5.79507	5.79892	6.92777	6.15543	6.90907	6.94545	7.97717	5.1558	6.64528	5.96158	6.71008
ZNF770	Q6IQ21	6.71015	6.18491	5.71336	7.23668	5.82615	5.741	6.31842	5.71591	6.39128	6.29137	6.89443	6.16524	6.3659	6.01805	6.50701	5.92013
C9orf85	Q96MD7-1	6.05578	6.22736	6.36949	5.95343	5.87393	5.98059	6.21018	5.98631	6.37128	6.07239	6.16702	6.16205	6.52402	6.59903	6.9895	6.45765

GC	P02774-3	5.01351	7.86775	5.1255	3.71094	3.73459	7.02965	6.53983	5.72122	7.00709	5.51696	7.03074	7.08279	5.12796	5.69093	11.2709	6.52962
ATP13A2	Q9NQ11	6.02936	6.06976	6.67489	6.38134	6.03213	6.20229	6.61178	6.13354	6.24297	6.66394	6.40168	6.16608	5.84746	5.98089	6.35822	6.20368
PRMT2	P55345	5.31737	6.47183	6.12526	5.27822	7.38795	5.64885	5.89814	6.58381	6.64217	5.85503	6.16104	6.97397	6.87301	6.14815	6.16154	6.47366
USP45	Q70EL2	5.37988	6.10436	6.88392	6.46257	6.26035	5.88735	5.22732	5.41134	6.27611	6.09794	6.3145	7.38329	6.84831	7.01172	6.11854	6.33249
SLC7A2	P52569-3	6.95501	7.0286	6.43556	6.63998	6.55565	5.75944	6.32888	6.37693	6.58851	6.01317	5.84601	6.77576	5.63935	5.48775	5.96026	5.60914
METAP1D	Q6UB28	5.34668	5.60104	5.65311	5.40222	6.45012	5.86921	5.94625	6.74056	6.14634	6.8217	5.93426	6.32866	6.89005	6.63994	7.24356	6.98629
CRYL1	Q9Y2S2	6.56305	6.08298	7.09561	6.25447	6.36228	6.52232	5.97194	6.61798	5.95027	6.70502	6.34258	6.5303	5.68115	6.16694	5.72826	5.42484
PIGM	Q9H3S5	6.44577	6.35326	5.66427	6.83883	6.17042	5.52211	5.93975	5.97706	5.87423	6.28934	6.06904	5.84735	6.91328	6.54899	6.58053	6.96578
MED28	Q9H204	5.71138	5.11769	5.28162	4.57292	4.8334	4.67711	4.70958	5.907	4.78062	5.90493	4.54703	5.03418	5.21593	12.3272	9.72272	11.6567
VTI1A	Q96AJ9	6.26538	5.6488	6.58845	5.98746	5.56587	5.46875	6.02636	5.46311	5.98935	6.41392	6.48131	6.95984	7.0362	7.0568	6.59286	6.45555
SPRYD7	Q5W111	6.46147	6.54116	6.42599	6.58196	6.07779	5.85265	6.44281	6.0387	6.44966	6.0558	6.45843	6.13832	6.03427	5.67463	6.56351	6.20285
GPM6A	P51674	6.20684	6.52769	5.9591	6.1313	5.95602	6.01776	6.40084	6.18585	6.25017	5.88084	5.63775	5.6636	6.92175	6.68075	6.37672	7.20303
CRTC1	Q6UUV9-2	6.13639	6.15773	7.52923	5.61228	6.62408	6.4524	6.09769	6.65703	6.02875	7.12933	6.41333	6.87733	6.23738	5.52211	4.93825	5.58671
SERGEF	Q9UGK8	6.53375	6.63025	6.83524	6.15524	6.31265	6.39313	5.76105	5.88434	6.27907	6.1756	7.08183	6.86522	5.92767	5.44152	5.91189	5.81157
EBAG9	O00559-2	6.80886	5.88125	5.35324	6.62293	6.20663	6.86829	6.57433	6.4769	6.36241	5.73196	5.64591	5.61295	6.44248	6.86341	6.06156	6.48689
GAREM1	Q9H706	6.87142	6.65716	6.90135	5.75791	6.17209	6.77775	6.6609	6.61328	6.20491	6.5933	6.07482	6.63725	5.38786	5.90649	5.38139	5.70213
LRRC49	Q8IUZ0-2	6.19487	7.00738	7.63639	6.23708	6.21292	6.96623	5.5827	5.81166	7.47837	7.62687	6.27185	6.09645	4.80811	5.01773	5.00255	6.04886
ARHGEF16	Q5VV41	6.10721	6.15088	7.23636	6.25174	6.25933	5.8975	6.0648	6.79736	5.97193	6.17901	6.20096	6.5409	6.30336	6.19675	5.90996	5.93193
HOXD8	P13378	6.27208	6.59079	5.80086	5.57996	5.7544	6.13581	5.98758	5.99593	6.35746	6.32009	6.37578	6.96433	6.32913	6.48235	6.94119	6.11224
SLC44A2	Q8IWA5-2	5.83353	5.68067	6.00326	6.68051	6.14989	6.06507	6.08185	5.57342	5.50318	6.62116	5.73041	6.20079	7.39493	6.56382	6.48436	7.43317
MSMO1	Q15800	5.9185	6.51789	6.50203	6.79768	5.49075	5.91956	7.0606	6.79747	5.7291	6.31431	6.23211	6.45679	5.46487	6.67663	6.10845	6.01329
STAB1	Q9NY15	6.22759	6.93935	6.30175	6.05158	4.86286	6.03351	5.86209	5.5589	5.92436	6.11834	5.55432	6.10997	6.34221	6.75067	7.36359	7.99889
SYTL2	Q9HCH5-8	6.09536	6.43387	5.58107	6.19911	5.52772	6.53974	7.63751	5.69952	6.87769	7.01704	5.05407	5.96471	5.93203	6.49986	6.11385	6.82687
SUPT3H	O75486-4	6.09306	6.08286	6.74515	7.34129	6.8672	6.85037	5.92993	5.55674	7.68907	6.18932	6.66168	5.34871	5.93062	6.57398	6.52794	4.51209
MAPT	P10636-9	6.64796	6.90956	7.13349	5.78143	6.7092	7.01169	6.27725	6.24767	6.10752	6.5076	5.99615	6.54454	5.60856	5.50776	5.5111	5.49853
DDT	P30046	3.52158	3.87786	3.9674	3.5709	5.89583	6.15136	6.5212	9.31516	7.12851	7.95059	5.66879	7.62137	4.81634	7.62265	7.50095	8.86951
PCGF1	Q9BSM1	6.45992	6.32914	5.83936	6.77123	6.88923	6.50072	5.91694	6.63948	5.85359	6.11423	6.09385	5.75842	6.58573	6.35712	5.93557	5.96147
SLC38A7	Q9NVC3	6.39725	6.72043	5.86426	6.3572	6.53283	6.55851	6.34835	6.2827	5.87541	6.16278	5.66333	6.0561	6.3276	6.36409	6.37489	6.11426
TWIST1	Q15672	6.44796	6.87684	5.28452	7.11861	6.76223	7.42677	7.22803	5.18958	5.2942	6.45276	6.94276	8.09307	4.41814	5.32607	5.2571	5.88137
ZMYM6	O95789	6.50871	6.17095	6.90735	7.59204	6.3438	5.91118	6.74256	6.07128	6.67198	6.36371	6.57664	6.86236	4.7343	5.45612	5.75749	5.32952
POC1A	Q8NBT0	6.0077	6.2487	6.54986	6.61719	5.68297	6.41631	6.34431	6.35958	6.61057	6.8948	6.906	7.13111	5.16454	5.86527	5.39942	5.80166
ORC6	Q9Y5N6	6.51741	6.2606	5.70671	5.73987	7.52464	6.02642	5.62314	5.26176	6.96985	5.24399	7.06744	7.63377	5.42429	6.78119	6.08711	6.13182
UBIAD1	Q9Y5Z9	6.20632	7.15316	5.98073	6.53674	5.97679	5.28832	6.34141	7.18349	6.9816	5.82686	5.92668	5.88203	6.1076	5.40007	6.31855	6.88963
SLC66A2	Q8N2U9	6.13261	5.9094	5.74122	5.72899	6.15895	6.08119	5.95374	6.23182	6.15181	6.58389	5.5589	6.06163	6.72901	6.63712	8.03853	7.20119
ENTPD4	Q9Y227	6.60063	6.58431	6.24039	6.94606	5.88559	6.4517	6.2409	6.35754	6.8335	6.62997	4.757	6.15498	6.50398	6.1237	6.20478	5.48496
PKNOX1	P55347	6.3919	6.79726	6.16706	7.1421	6.14839	6.45136	5.85365	6.20168	6.0374	5.55492	6.47292	6.9111	6.31098	5.82456	5.56757	5.96713
POMGNT2	Q8NAT1	5.68097	6.38628	6.40638	7.28704	6.25935	5.61168	6.88376	6.72808	8.14525	5.15132	5.17313	6.01473	6.15659	6.40061	5.34936	6.36549
KXD1	Q9BQD3	6.58272	5.58017	5.84033	5.12369	5.41462	5.17953	5.67264	6.21574	5.36469	6.17433	5.56966	7.31762	5.47228	8.9081	7.50844	8.07543
MRPS21	P82921	6.4525	5.76044	6.12159	6.69304	6.0677	5.52305	6.53407	5.7594	6.47289	6.04192	6.18812	6.24209	6.84884	5.86262	6.69612	6.73561
TMBIM6	P55061-2	5.69385	6.43787	5.52897	5.8754	6.6383	5.61084	7.15158	5.83935	5.72684	5.49208	4.72065	5.6238	7.21166	6.97942	9.35117	6.11822
CHMP6	Q96FZ7	6.13177	7.00552	5.9685	6.20759	6.31494	6.85319	6.3494	6.65595	6.28744	5.66232	5.85145	5.35661	6.70222	6.43134	6.35873	5.86302
RAB11FIP2	Q7L804-2	7.02037	6.50478	8.33799	7.33387	6.65587	6.3337	5.62286	4.72251	6.2899	6.82265	6.73585	6.33874	4.95736	5.77401	4.9428	5.60672
SCD5	Q86SK9	6.58291	6.75387	6.23346	6.76753	5.97422	5.80963	6.48535	5.88919	6.17735	6.47887	6.51396	6.07996	5.90318	6.02667	6.10942	6.21444
CNRIP1	Q96F85	5.74635	5.23539	5.90718	5.35743	5.88481	6.5254	5.73472	6.93506	7.27592	7.43719	7.1216	7.0446	5.63197	5.73837	5.87451	6.54951
ZFYVE28	Q9HCC9	6.15994	5.91817	6.32497	5.96265	5.91445	7.41729	5.96728	5.78003	5.83964	6.55817	6.399	6.25158	6.47741	5.83651	6.38566	6.80724
FAM210B	Q96KR6	5.93289	5.83153	6.03685	6.602	5.7266	5.85367	6.38463	6.09954	5.93746	6.78488	6.74846	6.42552	6.02848	6.48013	6.57991	6.54744
DNAJC30	Q96LL9	6.75317	6.45867	6.09213	7.15591	6.38386	6.17609	6.175	6.1225	6.12278	5.93686	5.9924	5.63341	6.67829	5.9488	6.1172	6.25294
COQ4	Q9Y3A0	7.16988	6.05485	7.13692	6.33641	4.79116	4.64702	5.5378	8.08755	5.54006	7.43144	7.1505	8.44483	4.12459	5.62139	5.95466	5.97093
DIPK1A	Q5T7M9	6.29156	7.26077	6.38787	5.9844	5.3851	6.04615	6.37449	5.01211	6.74343	6.8208	6.00395	5.95601	7.07947	5.59442	6.88959	6.16988
UEVLD	Q8IX04	6.09475	6.01809	7.45305	6.0467	6.14425	6.92044	6.35893	6.60169	6.32748	6.00938	6.33777	6.54687	5.45017	5.99004	5.54424	6.15616
AHR	P35869	7.11076	6.75452	7.74364	6.4765	6.00898	6.38179	6.22317	5.90026	7.03813	7.5979	7.76441	7.7378	4.15748	4.62253	4.20167	4.28044
ZBTB43	O43298	5.26193	6.66629	5.98768	6.3209	5.94662	6.27573	5.64079	5.92551	5.27939	6.27994	6.23239	6.22905	6.76397	6.67237	7.38859	7.12887
PIK3AP1	Q6ZUJ8	6.20898	6.13935	7.07849	6.30732	5.83191	6.34082	6.7687	6.73779	6.32404	6.05538	6.46556	7.03819	5.91867	5.3733	5.69501	5.71649
TOMM20	Q15388	7.12614	6.44985	6.15699	7.03234	5.95618	6.73571	6.80864	5.65248	5.58083	6.26055	5.94995	5.83116	6.15896	6.12871	6.34332	5.82818
TIGD2	Q4W5G0	6.43848	6.82403	6.42949	7.24853	5.44739	5.97645	6.27334	6.56357	6.09909	6.60133	5.61332	4.82502	6.18296	6.11008	6.65177	6.61517
SLC39A6	Q13433	6.15376	7.26852	5.42743	6.63942	6.0877	6.429	6.71662	6.62989	6.02795	5.76785	5.86049	5.89541	5.4201	5.45344	7.02739	7.19502
NME4	O00746	6.33942	6.48128	5.92462	7.57266	6.39923	4.94731	5.56693	6.40452	7.26143	6.69419	6.75817	7.03656	5.68278	6.05008	6.57264	4.30817
SHQ1	Q6PI26	7.78887	6.72574	6.38979	6.28705	6.5734	6.06283	7.0885	7.2072	7.60901	7.05221	7.36267	6.68328	4.05009	4.72244	4.04519	4.35172
UBL7	Q96S82	5.99179	5.26306	5.47538	4.99989	4.72554	4.82222	6.18389	6.63256	4.47388	6.51606	4.96465	6.62353	6.55016	9.43895	6.56257	10.7759
MTRF1L	Q9UGC7	6.58548	6.49344	6.41184	6.61813	6.21038	5.82198	5.81977	5.87872	6.61303	6.33496	5.78073	6.02959	6.47851	6.38143	6.31057	6.23145
RPP25	Q9BUL9	5.72295	5.94106	5.80494	5.8382	5.99569	5.88242	6.04828	6.114	5.76591	5.93328	6.34985	5.68324	7.2828	7.37003	7.11125	7.1561
NUDT8	Q8WV74	6.526	5.09503	4.39041	7.33038	7.97762	6.0083	6.36196	5.75681	6.11926	7.68148	5.96654	6.90111	7.22682	4.85461	6.27375	5.52994
PIGB	Q92521	5.55956	6.41184	4.75477	6.3232	6.23108	6.47338	6.79556	6.46224	5.68946	5.90919	5.53415	6.54642	6.7811	6.17057	7.12927	7.22822
MIF4GD	A9UHW6-3	6.88261	5.17959	6.20565	5.1599	6.41677	6.02541	6.31505	6.5415	5.72489	6.75949	6.12447	6.45253	5.98365	6.43211	6.50725	7.28911
LSM11	P83369	6.80569	7.10427	5.72028	6.01516	5.90315	6.38026	6.28386	6.06276	6.05916	6.61603	6.27001	6.47056	5.93773	6.24382	5.91306	6.21418
ING4	Q9UNL4	6.56676	6.67454	5.86138	6.5264	6.50013	5.70414	6.97481	5.75723	6.0205	6.96681	6.55884	5.94891	6.55822	5.16695	6.10861	6.10577
APH1B	Q8WW43	6.43514	6.97746	6.7569	6.67515	6.34471	6.03695	6.59661	6.33845	6.90914	7.04774	6.63511	6.56389	4.98852	5.33597	5.12557	5.23269
DFFB	O76075	4.97452	5.29008	6.97097	5.61552	4.7483	5.68381	5.62667	5.43362	5.27313	6.45327	5.89521	6.14517	6.98927	8.79203	8.04407	8.06437
NRIP3	Q9NQ35	7.32758	6.26588	7.50664	7.45689	7.63494	5.94061	7.51752	6.54973	7.56453	5.99935	7.06519	7.38524	4.4498	3.45228	3.95233	3.93149
PRUNE1	Q86TP1	6.39028	6.17655	6.58672	5.83898	6.88099	6.41872	6.58192	7.08692	6.67966	5.85193	6.34321	6.63321	6.14449	5.55418	5.47861	5.35362
NPDC1	Q9NQX5	7.88478	6.7679	7.13668	7.04644	6.29586	6.63978	7.59081	6.66703	6.96599	6.12139	7.61345	7.27067	3.91047	4.26307	3.6418	4.18389
DLC1	Q96QB1	6.12086	5.61917	6.64979	6.09271	6.10889	6.47036	6.33723	6.56625	6.14546	5.77892	6.02591	5.99625	6.71627	6.46664	6.70604	6.19926
SLC35F2	Q8IXU6	6.14681	6.66813	6.15931	6.58647	6.26645	5.92788	5.92333	6.29828	6.08843	6.32297	6.31505	6.31784	6.10067	6.22094	6.59681	6.06061
RFXAP	O00287	6.16801	5.97459	6.121	6.52177	5.95271	5.98493	6.38179	6.15132	6.52787	6.37706	6.81987	6.25563	6.86951	5.87731	6.04076	5.97587
ISG20	Q96AZ6	6.46113	6.43948	6.22794	6.33999	6.79987	5.91452	6.22026	6.68168	6.77996	6.60279	6.49873	6.43307	6.01307	5.56668	5.65134	5.36949
JDP2	Q8WYK2-2	6.8562	7.89147	6.96039	7.38747	6.21761	6.25983	6.15047	6.09305	7.21696	7.58487	6.85535	7.09569	4.27645	4.53714	3.99811	4.61894
CHKB	Q9Y259	6.90192	7.25058	6.5437	6.39097	6.61037	6.88983	5.7201	5.40746	5.36104	5.97154	6.19225	6.95012	5.84974	6.85071	5.34651	5.76319
TLCD3A	Q8TBR7	5.25015	6.73453	5.54238	6.66944	6.64211	5.97594	6.17849	5.31634	7.27538	6.05339	6.02168	6.39571	6.31956	6.13787	6.99965	6.48738
LMO4	P61968	6.28975	6.41188	7.02004	6.05946	6.7004	6.21783	6.87522	6.77626	6.32411	6.4451	6.86291	6.35967	6.04483	4.9018	5.5041	5.20666
OSTF1	Q92882	6.38312	5.93806	6.92989	5.34493	6.24823	6.46991	5.99428	7.01437	6.23349	6.54157	7.06326	7.27669	5.34387	6.32363	5.25286	5.64184
PGP	A6NDG6	6.42285	6.07759	7.33086	5.5686	7.61688	7.29673	6.57065	6.60454	6.9348	6.77758	5.91042	5.51825	5.67397	5.63744	5.35249	4.70633
ATG4D	Q86TL0	6.48886	6.16247	7.64099	7.07176	6.38199	5.95657	6.62794	6.20667	6.76302	7.49447	7.22454	7.28403	4.28667	5.20449	4.59745	4.5121
EGLN2	Q96KS0	6.2208	6.70414	6.23862	7.17203	5.23754	5.58741	5.9265	7.86739	7.62558	7.59665	7.30753	7.20523	4.82378	4.66544	5.06577	4.75561
ARMC7	Q9H6L4	5.85047	4.86563	6.41476	5.77123	5.62492	4.96481	5.17357	6.73575	6.0665	6.45817	6.12966	6.08365	4.49974	9.38472	7.39673	8.57971
STK32C	Q86UX6	6.04845	6.59544	7.09394	6.36921	6.2532	6.64586	6.05994	6.11534	5.97185	6.10958	7.07017	5.96859	5.66287	5.79124	5.97508	7.16924
NUDT22	Q9BRQ3	6.54235	9.90707	6.00628	4.33985	5.13422	5.12047	6.64557	13.1086	7.85828	6.79756	6.38548	7.14693	2.71357	4.25061	3.59808	4.44503
TCEAL9	Q9UHQ7	6.20382	5.27353	5.62251	4.86414	6.42963	6.71533	5.9684	6.60588	5.8385	6.62613	6.88904	6.7817	6.49324	6.83037	5.9309	6.92687
MT-ATP6	P00846	5.91683	6.19675	5.57228	6.54874	5.59708	5.86247	6.16002	6.42188	6.05936	6.13929	5.53113	6.17122	6.34889	6.58631	7.55179	7.33596
AP5S1	Q9NUS5	6.65012	6.01361	6.4023	5.84402	4.8397	6.20074	5.51677	6.71485	6.60699	6.38642	5.94041	6.47067	4.88595	8.38317	5.91336	7.23092
BMT2	Q1RMZ1	6.40394	6.09196	7.53189	6.12438	6.74964	6.68873	5.72782	6.13965	6.01248	6.93629	6.43355	7.09797	5.6346	5.73396	5.54568	5.14744
TIMM22	Q9Y584	6.91554	6.45212	6.17278	5.86487	6.60592	6.46875	6.7261	6.45111	5.99914	6.44231	5.66731	5.60188	6.47338	6.2794	5.89205	5.98733
UBE3B	Q7Z3V4	6.41516	5.49559	6.19788	6.33702	6.22508	7.71676	6.46624	6.66182	6.87742	5.88539	6.32322	5.6672	5.79085	6.49337	5.76559	5.68141
ISOC2	Q96AB3-2	5.991	4.84032	5.56059	6.12067	6.34093	6.04444	6.15681	6.36231	5.39797	5.86015	4.98686	5.71751	6.0217	8.17618	7.82065	8.60191
ZNF276	Q8N554	5.28617	6.13551	6.05323	5.32748	6.24321	5.6598	5.95113	6.32919	5.95659	6.21746	7.43469	6.7257	6.35946	7.00639	7.24276	6.07123
PDP2	Q9P2J9	5.57135	5.21067	4.86655	5.64114	6.80251	6.62655	7.07951	7.19737	6.271	6.20001	5.82065	6.17303	6.41961	6.6272	6.04298	7.44985
CCNG1	P51959	7.82491	7.85822	8.21363	8.44413	6.05803	5.91988	7.95982	6.56999	8.10977	8.41665	8.25733	8.87399	1.59653	1.91153	1.89061	2.09497
C15orf61	A6NNL5	6.44785	5.25839	6.08736	5.78442	6.12415	6.09657	6.57259	6.44109	5.78066	5.92142	7.06014	7.01549	6.31589	6.82555	5.7982	6.47023
POLR2L	P62875	5.36911	5.25351	5.53201	5.70502	5.93092	6.61875	6.18669	7.20801	6.26634	6.71982	6.40756	6.29547	4.94617	7.92099	5.96667	7.67295
GPR176	Q14439	6.00132	6.39249	6.46198	6.10835	5.96627	5.78382	6.37568	6.0535	6.24473	6.65229	6.58528	6.62359	6.08867	6.09726	6.02959	6.53518
TBC1D14	Q9P2M4	6.29166	6.43122	7.70717	5.9098	5.87193	6.89002	6.39701	6.77892	7.01234	6.68836	7.11084	6.64096	5.08118	4.93146	5.03736	5.21978
TIPIN	Q9BVW5	5.76732	5.8111	6.30779	6.05368	6.2617	5.93107	5.47258	6.76064	5.15151	7.22312	6.52247	7.50698	6.77042	6.27173	5.58953	6.59838
TNFRSF10B	O14763	7.09961	5.88033	5.40994	5.51282	6.788	5.56836	6.86974	6.03932	6.20133	6.62697	5.61123	4.99872	7.12229	6.68188	6.9207	6.66876
MPPE1	Q53F39	6.56096	6.44054	5.53263	7.28068	5.75733	5.59584	6.4256	5.94313	6.34684	5.918	5.79506	5.95598	6.26618	5.98812	6.92852	7.26459
POLR3H	Q9Y535	5.62006	6.83462	6.29314	5.52637	6.22826	5.84464	6.25335	6.36976	6.37489	6.31356	5.44739	6.01413	6.86821	6.53577	6.39728	7.07856
POLR3G	O15318	5.82272	5.71984	6.05767	5.69202	5.80254	7.16002	5.04003	5.77901	5.39339	6.93	6.84114	6.38667	6.26235	7.63308	6.52861	6.95093

ZNF330	Q9Y3S2	5.7528	6.34514	6.8108	6.39441	6.17356	6.91407	5.76435	6.70785	6.38967	7.17008	6.72798	6.2845	5.52337	5.85951	5.85151	5.33039
JAK3	P52333	6.52464	6.75212	7.19214	6.18167	6.87139	7.19151	5.95721	6.4729	5.75298	6.24803	6.60043	6.92069	4.83316	5.92962	4.78318	5.78831
CCDC28A	Q8IWP9	6.22875	6.03479	7.03938	6.84302	5.68014	6.28593	6.90987	6.85578	5.68181	6.41981	6.62226	7.02397	5.87062	5.44609	5.70269	5.35509
ORAI1	Q96D31	5.70131	6.23732	6.49684	6.59707	6.15927	5.76297	5.59392	5.91238	6.21234	6.46087	6.60389	6.80315	6.10243	5.80433	6.55118	7.00075
ELK3	P41970	5.75421	6.26125	5.51427	5.55731	6.99038	6.82614	5.87228	7.10243	7.38482	5.0788	6.30705	5.51703	6.21898	6.11419	7.58581	5.91505
RNF34	Q969K3-2	5.77062	5.7285	7.67449	6.45453	5.98769	5.47482	6.65892	5.98502	6.29072	6.72875	7.00628	6.9387	6.19663	5.80413	5.43308	5.86711
SERPINC1	P01008	5.43402	7.4436	4.64816	4.93373	4.53693	6.8278	7.12437	6.35921	6.21524	5.40066	5.71288	5.60178	5.86766	5.71987	10.371	7.80307
TRAIP	Q9BWF2	6.89894	6.78181	6.04881	7.33234	6.41271	6.28031	6.67088	6.05928	6.48034	6.99954	7.55527	7.21389	4.35028	4.99788	4.45968	5.45805
SSR3	Q9UNL2-2	6.18808	6.52813	6.08599	6.50056	6.10486	5.89405	6.44246	5.8326	6.22016	5.84985	6.11502	5.84099	6.41639	6.46804	6.72126	6.79155
PAQR7	Q86WK9	6.31465	6.43213	6.49788	7.56936	6.77429	5.88619	6.42051	6.61577	6.25181	5.76405	6.37883	6.71012	5.42	5.44564	5.64102	5.87776
DESI1	Q6ICB0	5.60892	5.39852	4.86084	5.23837	6.10725	5.54577	6.4899	8.28571	5.25923	6.79382	7.23995	7.86855	4.37099	6.93392	6.37391	7.62436
TGFBR2	P37173-2	6.84653	7.2445	7.2467	7.56953	6.37332	6.31215	6.346	6.39324	6.99177	6.89196	6.67599	6.65056	4.40425	4.91722	4.49376	4.64251
EID1	Q9Y6B2	7.2969	6.75522	7.84101	6.44499	6.70481	7.11619	6.21527	7.0444	7.39666	7.29729	7.90536	7.40873	3.43945	3.74663	3.40086	3.98622
TIMM10	P62072	6.26957	4.77631	4.80732	5.66388	4.62698	2.73332	4.60767	4.99274	3.9208	4.96827	4.53325	4.56254	4.42855	14.6934	10.1172	14.2982
CREBRF	Q8IUR6-3	5.64468	6.30517	6.20292	5.38217	6.06862	5.68367	5.80846	5.52995	4.96876	5.65808	5.27203	6.01187	8.70113	7.35657	7.69009	7.71581
FAM161A	Q3B820-3	6.55217	5.46205	6.95789	6.45819	6.23038	6.26449	5.84173	6.49917	6.45316	6.2912	6.27503	6.49027	6.48679	6.06676	5.30232	6.36841
LARP6	Q9BRS8	5.54243	6.1395	6.48534	5.50023	6.34031	7.17755	5.83144	6.70533	6.1165	5.6795	6.85278	5.76727	6.467	6.6636	6.05402	5.77719
CCDC82	Q8N4S0	5.68523	6.61957	5.97162	4.69269	6.06874	6.15243	6.39708	6.04724	6.13532	6.92467	7.47647	6.70549	6.76219	5.90192	7.00491	5.45442
IQCJ-SCHIP1	B3KU38	6.61135	5.89023	5.93869	5.88676	5.87037	6.33038	6.65041	6.27594	6.50402	5.83936	7.22631	6.7935	6.45525	5.20186	6.80617	5.7194
HORMAD1	Q86X24	5.3304	6.51803	6.10124	6.82914	5.71403	5.45148	7.03676	6.50071	6.66094	4.73797	6.08795	6.28048	6.54805	7.14087	6.70543	7.25651
NOP10	Q9NPE3	5.30258	6.51728	6.68542	6.14436	6.52847	6.53453	6.40881	6.59432	6.09221	6.17238	5.75008	6.35095	6.10151	6.29482	6.34635	6.17593
GAS6	Q14393-1	5.96957	6.0985	5.85401	5.68079	6.51951	6.11434	6.20608	6.31961	5.5996	7.05022	6.71879	6.30084	5.91387	6.96056	6.29523	6.39846
SLC16A2	P36021	6.31005	5.9287	5.62374	5.29562	6.65734	6.1875	6.55636	5.5744	7.08764	6.80935	6.81897	6.85963	7.02404	5.09498	6.26947	5.90221
MED29	Q9NX70	5.80504	5.11942	4.89499	4.89865	5.11251	5.2674	5.70775	6.36892	5.04399	5.42517	4.86619	4.8787	5.35409	11.7421	7.69616	11.8189
NTAQ1	Q96HA8	6.01165	5.29992	7.10421	5.46587	6.31346	6.82421	5.55004	7.04577	6.34576	6.67582	7.29295	7.21084	6.0067	5.62943	5.84165	5.38173
ELOF1	P60002	5.27319	5.68352	4.97622	6.41978	5.81204	5.61306	7.64718	5.2652	6.48879	5.0898	5.48099	5.52473	5.70107	9.17393	7.16267	8.68783
C1QL1	O75973	7.88498	6.882	6.4608	6.94094	6.52309	6.15088	5.87973	6.59962	7.03797	7.40513	6.74127	6.35836	4.75073	5.40402	4.38866	4.59182
WASHC3	Q9Y3C0	6.13308	5.70884	6.14173	5.64887	6.15022	6.35067	6.04851	6.64734	5.81315	6.32715	7.26078	6.74769	6.49647	5.97	6.48666	6.06884
FBXO9	Q9UK97	6.8664	6.45804	7.39785	6.3996	6.1882	6.55572	6.40512	6.8205	6.95124	6.79972	6.71868	7.08322	4.89551	5.0986	4.66053	4.70107
MFSD14B	Q5SR56	5.91526	6.49295	5.9597	6.8384	6.7328	5.8865	6.30898	5.94782	6.08134	6.2227	6.1677	6.52139	6.132	6.36117	6.37547	6.05672
SNX11	Q9Y5W9	6.26082	5.90131	6.6032	6.9082	5.92404	5.98043	6.92927	5.97987	6.57241	6.51669	7.02503	6.86672	6.26119	5.22996	5.94869	6.09215
KIF24	Q5T7B8	6.56392	6.32314	6.86992	5.88149	6.29961	6.84199	6.6063	5.90025	6.61533	6.79299	6.63303	6.84052	5.00136	5.7212	5.74913	5.35982
MKKS	Q9HB66	5.84057	5.11092	5.93916	5.53333	5.31359	4.53142	5.61923	5.28816	5.82828	6.00837	5.56216	6.34791	6.36167	8.52476	8.35678	9.8337
AGPAT2	O15120	7.073	7.29918	6.09144	6.46222	6.0156	6.133	5.7992	5.88263	6.12701	5.08108	6.47664	5.19161	6.61725	6.10574	7.10188	6.54253
AP4M1	O00189	5.49328	5.74063	7.23319	6.16452	6.85293	6.12117	6.06171	6.32294	7.13151	5.6335	7.37823	7.86113	5.63067	5.82369	5.06817	5.48272
TMEM106B	Q9NUM4	6.34767	6.05486	6.12184	5.89204	6.20755	6.05362	6.32579	6.2381	6.04291	5.6988	6.72696	7.83316	4.87677	6.56543	6.83963	6.17487
ZDBF2	Q9HCK1	7.74413	6.28259	5.37895	7.36205	5.31195	6.552	5.86045	5.01254	7.053	6.8755	6.8957	6.33874	5.8222	6.43616	5.30102	5.77302
ZNF556	Q9HAH1	6.20682	5.75561	6.52534	6.55867	5.28648	5.83076	6.54001	6.38177	6.14353	5.22131	6.8583	6.20474	6.82343	6.48445	6.26822	6.91058
MIIP	Q5JXC2	6.48187	6.46631	7.38973	7.50012	6.0967	6.93243	6.18654	6.41132	7.33077	6.75168	8.20425	8.60207	3.87645	4.3107	3.70951	3.74954
CDKN1B	P46527	5.89641	5.58451	6.59885	5.26383	5.8451	5.92243	5.48331	6.769	5.61403	7.48689	7.15612	7.54149	6.31251	7.22863	5.73984	5.55705
SUPT7L	O94864	6.02457	5.30382	7.10646	5.70082	6.19385	5.80091	6.69186	5.36384	6.0199	6.8022	5.9931	6.46558	7.21672	6.30552	6.57773	6.43311
TPRKB	Q9Y3C4-3	6.48701	6.17383	6.00449	5.15715	5.96991	6.6849	6.16013	6.03489	6.02701	6.484	7.19076	5.94079	6.85672	6.44617	6.89964	6.4736
COX7C	P15954	6.13634	5.39863	5.57344	6.32253	5.75236	5.29722	5.6503	5.86881	5.97022	5.47345	5.55531	5.83364	5.88615	8.97577	7.14685	9.15899
FN1	P02751	7.48119	7.25857	7.06307	7.33353	6.71767	6.68163	6.22316	5.94646	6.04678	7.04283	6.48265	7.16573	4.42268	5.49898	4.77713	3.85795
TOMM6	Q96B49	4.58781	4.32807	4.4802	6.65201	5.76492	6.2113	6.38658	5.41979	6.14164	5.97953	6.23268	6.76032	5.35882	8.51124	6.83518	10.3499
CDIN1	Q9Y2V0	5.4578	5.80257	6.64296	5.60925	6.36179	6.87313	6.18687	6.83993	7.18299	6.91987	6.87886	6.74792	6.00418	6.21855	5.42084	4.85248
CHAC2	Q8WUX2	6.43963	6.11204	6.70494	6.04452	7.29086	5.83653	6.2352	7.21192	6.20528	6.51926	6.37447	6.52161	5.95177	5.6376	5.44566	5.4687
BLOC1S5	Q8TDH9	5.83764	5.2255	6.47695	4.70345	6.62526	5.50457	4.9095	8.26969	5.38419	7.1647	7.06009	7.36625	6.33729	6.63559	6.63133	5.868
BORCS7	Q96B45	6.65966	4.87677	6.39093	4.56636	6.93353	5.77544	5.52526	6.44281	4.75521	5.82368	5.97138	6.42991	5.56491	8.94314	7.93243	7.40858
UBXN2A	P68543	7.17435	7.10546	7.9543	7.31165	6.3902	5.62892	7.30211	6.10622	7.0119	6.67774	7.41693	7.05116	4.3114	4.25601	4.0451	4.25656
CERS6	Q6ZMG9-2	6.19196	6.20166	5.92214	6.53521	6.06407	5.97788	6.07838	6.10083	5.41069	5.90135	6.18636	6.11493	6.76904	6.71832	6.80979	7.0174
SLC17A5	Q9NRA2	6.42333	6.69634	6.86008	6.01421	6.19389	5.52917	6.30805	6.45034	5.36857	6.80782	6.25076	5.42715	6.35037	6.52064	6.02504	6.77424
ZBTB44	Q8NCP5	6.03233	6.68801	5.70818	6.97669	5.95585	5.34232	6.19491	5.39531	6.02132	6.71399	6.08722	6.62124	6.90511	6.82195	5.95559	6.58099
UNC119	Q13432	5.96142	6.24358	7.29959	5.95156	6.0125	6.41448	5.84882	5.3632	6.09522	8.59943	7.03948	6.39505	4.69418	5.38334	6.03344	6.66472
ARL5B	Q96KC2	5.89633	5.78163	6.34441	6.04567	5.97883	6.67296	6.73542	6.62928	6.9915	6.34209	5.60729	6.31568	6.91888	5.78961	6.07397	5.87644
ILRUN	Q9H6K1	6.79847	5.92255	8.72328	4.87713	5.89396	6.62434	5.90536	6.40005	6.05972	7.21414	6.8109	5.93896	6.0118	5.77116	5.96755	5.08062
TMEM51	Q9NW97	6.79239	7.475	7.24338	6.90124	6.25449	6.28012	5.91584	5.779	7.04263	6.95996	5.98112	6.5442	5.45675	5.83351	4.43591	5.10445
SGMS1	Q86VZ5	6.31303	6.43814	6.63457	7.10699	6.11936	5.21652	6.68259	5.05102	6.09789	6.12538	6.32399	6.21641	5.97744	5.81005	6.71477	7.17183
DMAC1	Q96GE9	6.45104	4.98724	5.89507	6.50698	6.43581	6.56579	6.4251	6.75246	5.89205	5.48463	6.70842	6.24119	6.56898	6.49032	6.30975	6.28518
ERVK3-1	A0A0U1RQV1	6.56188	4.57906	4.84915	5.02773	5.21713	4.144	4.35468	5.19974	4.38133	6.63793	5.75571	6.13401	4.59161	12.5744	8.15658	11.8351
ZIC2	O95409	6.42352	5.49685	6.02857	7.42143	6.53372	6.37855	5.79336	5.23554	6.86263	7.0844	6.98537	7.66766	5.68688	5.31871	5.50434	5.57845
COX6A1	P12074	6.0708	4.3956	4.83327	5.7904	5.77345	6.08148	7.10886	6.65527	4.61178	4.86872	5.5829	4.78345	6.65609	9.64743	7.30874	9.83177
CHTF8	P0CG13	5.54618	5.88307	6.05383	5.51348	6.21648	5.91133	5.90314	6.37979	6.14336	6.1558	6.22549	6.58465	6.66808	7.4341	6.78481	6.59641
GEMIN2	O14893	6.72306	4.8969	6.59686	5.84005	6.69506	6.32668	6.16148	6.24328	5.12047	6.95412	6.98559	6.94263	7.31835	6.20671	5.28441	5.70434
SLC25A38	Q96DW6	5.56921	5.88608	5.9603	7.10193	6.77437	6.57754	7.03767	6.24892	6.41625	6.02069	5.94622	5.96501	6.22003	5.80862	6.20356	6.2636
FBXW9	Q5XUX1-2	5.94504	5.52974	6.24382	6.31597	5.98558	6.90998	5.75814	7.25871	7.13259	5.99299	6.51379	6.43246	6.17377	6.76274	5.43227	5.6124
CIAO2B	Q9Y3D0	6.27094	5.41377	5.94506	5.40603	5.7924	6.50317	5.36518	6.41567	5.47964	5.92242	5.86689	6.44789	5.39636	9.01701	6.88772	7.86986
APLP1	P51693-2	7.75621	8.40556	7.47306	7.00272	6.06402	6.96593	6.02464	6.89107	6.83898	7.12973	7.15654	6.45901	4.29356	4.01597	3.60098	3.922
TPPP3	Q9BW30	6.3852	6.06351	7.81239	5.53052	6.32892	6.15543	5.1417	6.91728	6.40189	6.6558	6.31844	6.55547	6.56495	6.46125	4.9805	5.72673
KCTD20	Q7Z5Y7	6.94999	6.9875	7.12844	6.70714	6.13554	6.75786	6.21144	6.95178	7.06745	6.75146	6.73752	7.66765	4.315	4.83276	4.43965	4.35883
WDR13	Q9H1Z4	5.68555	6.13951	7.02092	7.47761	5.30158	6.93914	5.80374	6.07297	7.02354	6.08473	5.6385	6.99117	6.80342	4.96697	6.10748	5.94317
STK35	Q8TDR2	6.90197	6.51897	6.3011	6.52727	6.46348	6.0197	6.58538	5.8807	6.4436	6.43115	6.33583	6.55116	5.72613	5.60596	5.79974	5.90787
CLN6	Q9NWW5	7.35102	5.81445	6.12049	6.66983	5.84866	5.85926	6.75357	5.74372	5.51837	6.25535	5.87506	6.0292	5.69458	6.90698	7.04037	6.5191
GABPB1	Q06547	6.08772	5.20953	6.20922	5.48772	6.08945	5.91983	5.9206	4.71658	6.06337	6.10077	5.85194	6.53336	7.19355	7.76829	7.56946	7.27859
RABIF	P47224	5.01686	5.77146	8.0932	4.51995	5.94572	7.47882	5.76686	7.63286	7.05018	6.37236	6.39904	7.3554	6.17825	6.84028	5.84926	5.34949
HDAC9	Q9UKV0-7	5.40781	5.79217	6.54385	5.66858	6.20425	6.10583	6.95152	6.10794	5.40064	5.02605	5.71198	6.30385	6.88634	6.57559	7.71557	7.59801
MT-CO3	P00414	6.07452	6.09711	5.08446	7.10868	6.76573	6.08216	5.97043	7.22281	6.04968	5.72444	5.97417	5.83583	6.25712	6.3583	6.75047	6.64412
ARPP19	P56211	5.27344	6.08184	6.90784	5.35616	7.67445	6.39944	5.68625	7.54875	5.83289	6.33583	5.96202	7.46121	4.8839	6.93309	5.11274	6.55015
SNX21	Q969T3	6.60147	6.68907	6.59288	6.22471	6.64634	7.47127	5.83263	6.62665	6.98859	6.32225	5.97317	6.31587	5.63832	5.24033	5.29129	5.54517
ALG12	Q9BV10	6.49559	6.10075	5.95706	6.31185	6.30182	6.11593	6.98002	6.02474	5.51673	6.30673	6.04738	6.07535	6.56782	6.48332	6.26424	6.45069
F2RL1	P55085	5.56669	5.42103	4.24985	5.70386	5.94566	5.86115	6.2042	5.60375	5.60801	5.29918	5.10656	4.99356	9.94159	7.32238	9.01684	8.15568
RPL39P5	Q59GN2	5.93288	6.38528	6.13066	5.72631	5.87649	6.13289	6.07038	6.72125	6.57171	6.22005	6.27593	6.31691	6.61989	6.8301	5.92621	6.26307
SCML1	Q9UN30	6.25274	6.64994	6.3525	7.30567	6.28171	6.58261	6.16048	6.03034	5.7852	5.85002	5.98536	6.45244	5.77058	6.06565	5.71417	6.7606
BLOC1S1	P78537	5.40965	5.25514	5.74068	5.26281	5.37283	4.81657	5.13123	6.82511	4.76803	6.29069	5.79724	6.04186	5.11179	10.765	7.93323	9.47818
CTDSP1	Q9GZU7	6.61765	6.59122	6.37363	5.97546	6.51011	6.72534	5.96648	5.98833	5.48333	6.69215	6.17241	6.1523	6.75857	6.38523	5.95413	5.65817
PCDHGC3	Q9UN70	7.58807	6.24351	5.98282	7.88872	6.3923	6.44626	6.6183	5.80573	6.80759	6.78343	5.88318	6.93754	4.93269	5.50829	4.79695	5.38461
NXPE3	Q969Y0	6.60082	7.61421	6.06856	6.87322	6.32782	6.15949	6.0273	5.96728	5.62893	6.12719	6.87971	6.30031	6.12889	5.9641	5.74359	5.58857
PCTP	Q9UKL6	6.89793	6.07448	6.84669	6.11463	6.13563	7.66126	5.77402	6.00178	6.53257	6.62096	6.24598	6.82179	5.94179	5.79368	5.87679	4.68607
YIPF5	Q969M3-3	6.28966	6.50053	5.64375	6.33477	5.7769	5.89719	5.73444	6.18639	5.94548	6.14771	5.87929	6.60195	6.76868	6.71243	6.69684	6.884
TTYH3	Q9C0H2-4	7.09211	6.99635	6.43688	7.28572	6.12354	5.98571	5.7745	5.37606	6.9544	7.65016	6.9336	6.24468	4.85687	5.86378	5.2575	5.16814
NTN4	Q9HB63	7.05089	6.488	5.42432	7.56262	5.53717	6.8555	6.69811	6.09171	6.09309	6.11161	6.07486	6.19948	6.56395	4.93154	5.07843	7.23872
COX7B	P24311	6.03067	5.67523	5.90155	5.91341	6.57183	6.13709	6.29862	6.45314	6.16774	5.92197	5.90489	6.2918	6.26801	6.92049	6.77316	6.7704
BCL7C	Q8WUZ0-2	6.75394	6.39931	7.00565	6.9873	6.85661	6.16147	5.95268	6.4526	6.07886	7.17547	7.19748	5.76932	5.50907	5.4541	4.50183	5.74431
SLC7A6OS	Q96CW6	6.24715	6.71706	6.9872	6.60135	6.14246	6.93241	6.1149	6.15251	6.58278	6.69029	7.15201	6.67197	5.50441	5.04025	4.9314	5.53186
SSBP4	Q9BWG4	6.09837	5.85648	5.44363	5.28197	5.84244	5.81349	5.43418	6.10143	5.54258	6.38335	6.18302	6.88362	7.00499	7.8127	7.74659	6.57116
PHLDA2	Q53GA4	6.53011	6.67319	7.41995	7.188	5.53607	6.40001	5.09898	6.87967	7.18062	6.39413	8.19788	6.7854	4.79119	5.16438	4.45057	5.30984
CDH13	P55290-4	5.39009	5.96773	5.16203	6.53338	6.95347	6.87679	6.28798	6.88513	6.11755	5.84824	5.71069	5.61261	7.68284	7.38556	5.75417	5.83174
MYNN	Q9NPC7	6.03699	5.92953	6.2772	5.56023	5.56666	4.75743	6.33304	6.19342	6.51395	5.93402	6.63387	6.53924	6.60358	7.21959	7.08341	6.81783
CEBPZOS	A8MTT3	6.18506	7.35357	6.00265	5.69321	7.28624	6.03778	6.60652	6.13374	6.3298	6.49259	5.64488	5.37458	6.85198	5.76235	6.93863	5.30642
TVP23C	Q96ET8	5.90406	6.26762	6.08591	6.64402	6.03562	5.92295	6.43708	5.70393	6.02817	6.50531	6.28688	6.35849	6.50383	6.5189	6.41557	6.38167
FAXC	Q5TGI0	7.33215	6.48037	5.41907	6.10358	6.35796	5.72033	5.79559	6.70319	6.63932	6.67861	5.4131	4.48192	5.76987	6.67536	6.78154	7.64806
RAB36	O95755	7.7709	6.81967	6.91189	5.71188	5.72431	6.88658	6.0487	5.87333	6.08056	6.06203	6.61752	6.35729	6.44526	6.27746	4.90318	5.50945
PTPRZ1	P23471	6.28376	6.40336	5.64199	6.94714	6.39212	6.06983	7.04347	6.05206	6.844	6.31543	5.99396	5.93556	6.58659	5.8979	6.19253	6.3003
MT-ND3	P03897	5.82528	5.87153	4.92055	6.7097	6.00582	5.76539	6.09596	5.51551	6.40034	5.78087	6.55297	5.19661	7.20462	8.09392	6.52058	7.54033
ZDHHC7	Q9NXF8	6.2152	6.22976	5.21929	6.75563	6.4882	6.52848	6.73218	6.49588	6.53759	6.32558	6.20851	6.20482	6.72916	6.0716	5.43151	5.82661

DYNC1I1	O14576	5.97817	5.71861	6.84394	6.04491	6.34761	6.57179	6.45367	7.26694	6.59509	6.34266	6.65227	6.52421	4.94813	6.12097	5.6765	5.91453
PRADC1	Q9BSG0	6.62118	6.00349	6.41818	6.47282	6.49654	6.20237	5.74902	6.70366	6.54132	7.02321	6.62651	6.87795	5.12985	5.70513	5.56951	5.85924
FAM126B	Q8IXS8	6.35471	5.87019	6.50105	7.79286	6.22937	5.255	7.53697	6.02874	6.10397	5.47796	5.72297	6.8438	6.89665	5.65626	6.32486	5.40463
CD40	P25942	6.88074	6.47211	5.90625	6.49284	6.28078	6.30104	5.99092	5.69002	5.7513	6.06641	6.20089	6.04879	6.10616	7.40012	5.93432	6.47732
CLDND1	Q9NY35	6.13717	6.91725	6.03411	6.04962	6.30222	6.19288	5.97177	6.24969	6.33331	6.21644	5.86428	6.11685	6.67413	6.17869	6.17296	6.58862
ARRDC1	Q8N5I2	6.62352	6.54077	6.23272	5.77693	6.48817	6.28908	6.20455	6.52005	6.58386	6.4574	6.4416	6.30466	5.77186	5.87786	6.00731	5.87965
MIPOL1	Q8TD10	6.17251	5.97229	6.69795	6.54294	5.82782	5.86382	6.39399	6.84922	6.97701	6.34179	6.69121	6.60094	5.75971	5.38417	6.07847	5.84618
HIPK1	Q86Z02	5.79487	6.03511	6.27447	6.2691	6.3493	6.2439	6.54341	6.45117	5.97626	6.24929	6.72157	6.82659	5.98096	6.10684	6.12466	6.05253
CDKN3	Q16667	6.71924	6.6938	7.93979	6.64339	6.94409	6.90994	6.96301	6.75527	7.14292	6.90212	7.14497	6.69467	4.04565	3.99435	4.15996	4.34681
HOXC9	P31274	7.27045	6.35863	6.54395	7.62867	7.12229	6.21728	6.04162	5.8252	5.92522	6.86359	7.31016	7.16731	5.37035	4.93024	4.72879	4.69624
RAB34	P0DI83	6.56471	6.8691	6.75047	6.45217	6.80829	6.58311	6.01453	6.55468	6.74911	6.87557	6.31296	6.57018	6.42544	5.35717	4.73231	5.38019
MTRES1	Q9P0P8	6.26367	6.21436	6.10863	5.94747	5.81448	5.33393	7.4086	5.87831	6.27182	6.24953	6.4498	5.83443	6.75225	6.45401	7.03609	5.98266
KCNAB2	Q13303-3	6.27639	6.10138	6.40978	5.86766	6.22249	6.75726	6.29205	6.17131	6.4099	6.66281	5.93096	6.17338	6.51095	6.21704	6.06568	5.93096
PLEKHB2	Q96CS7-2	6.0828	6.17192	7.23881	6.19487	6.58452	6.61974	7.38185	6.65845	5.74218	6.03769	6.07298	6.97087	5.83457	5.21982	5.72497	5.46395
ARSD	P51689	6.4934	6.48441	6.35196	6.92249	6.22748	5.76263	5.9752	5.76058	6.34842	6.22823	6.25571	6.27345	6.35352	5.72832	6.58025	6.25395
SLC35C1	Q96A29	7.94776	6.35403	6.97062	6.5778	6.58373	5.015	5.46334	4.21201	5.5945	5.63175	4.94076	5.69219	7.77813	7.98448	7.40871	6.74519
FADD	Q13158	6.58141	5.67748	7.47097	5.40483	6.31746	6.13437	5.9321	6.64991	7.04766	7.36655	6.85279	6.96484	5.74045	5.62292	4.77901	5.45725
TLCD4	Q96MV1	6.41042	6.60713	5.94262	6.79333	5.76924	5.7761	6.11474	6.00244	5.79995	6.31895	5.86815	6.10449	6.51296	6.4115	6.70191	6.86608
LEPROT	O15243	6.44467	5.8619	6.94236	6.73352	5.77314	6.99486	7.17363	6.10822	7.01304	5.82925	6.36924	5.87455	5.4021	5.91366	5.52036	6.04549
MT-ND2	P03891	6.0256	6.50477	5.29669	6.84999	6.2166	6.24634	6.46025	6.72126	5.65017	6.36578	5.40894	5.72663	6.40832	6.87247	6.53419	6.71201
C16orf70	Q9BSU1	6.65937	6.72557	6.78793	5.98111	6.94915	6.44728	6.72688	7.08774	6.05162	6.38575	6.4281	6.17086	5.27278	5.11195	5.05815	6.15577
CRISPLD1	Q9H336	7.04651	7.50223	6.40677	7.69585	6.15713	6.66683	6.79181	6.10142	6.80251	7.86758	6.21792	6.90881	4.33841	4.42164	4.38527	4.68931
PPP2R3C	Q969Q6	5.77903	7.6645	6.17429	6.33543	5.6855	7.41816	4.71117	6.26719	6.89237	6.86399	6.78392	7.61078	5.49049	5.57365	5.26611	5.48342
ARL2BP	Q9Y2Y0	5.73551	5.93603	5.88389	5.58289	5.98949	6.28748	6.06645	7.19247	6.49795	6.90792	6.83504	6.84092	5.10555	6.6645	6.01106	6.46284
CNIH4	Q9P003	5.54869	6.84034	5.53202	7.78976	6.73067	7.4092	5.66184	6.02876	4.08871	5.11451	7.30605	6.45239	6.46682	6.87761	6.43381	5.71881
SELENOI	Q9C0D9	5.23662	7.19113	6.78615	6.12862	5.51771	5.59417	6.59314	6.3753	5.53355	6.47042	6.3264	6.44391	5.77289	6.20906	7.11379	6.70714
PHOX2B	Q99453	6.53128	6.05472	6.14393	6.73625	5.11997	5.4965	5.88815	6.05163	6.08438	7.33818	5.72816	6.80901	6.52315	6.31881	6.54767	7.62822
MUC5B	Q9HC84	6.01393	6.48157	6.60351	6.3595	6.51051	6.35339	6.1314	6.47084	7.04447	6.38684	6.44423	6.18852	6.36429	5.34564	5.8518	6.44957
PSMD4	Q5VWC4	6.29669	6.93626	5.17548	5.64042	4.52879	6.22436	6.26207	7.10534	6.8017	6.51729	6.32564	7.28888	6.5552	5.87601	5.10893	7.35694
MAGED4	Q96JG8-4	6.30333	6.29414	5.80379	5.8089	5.21673	7.06997	7.1675	5.90478	6.01741	6.82508	7.14249	6.88151	5.95574	6.1461	5.70293	5.7596
PRR36	Q9H6K5	6.92176	6.7573	6.50412	6.44631	5.7988	5.291	6.79807	6.25494	6.50677	5.90836	5.88447	6.36895	6.40015	7.2837	5.53448	5.34082
HSPA8	E9PN89	6.26611	5.84964	7.31773	5.94414	5.91769	5.84438	5.87217	6.49061	6.53543	6.35345	5.70238	5.5586	6.34389	6.11013	6.63314	7.2605
HSPA8	E9PQK7	6.71568	6.1037	6.25504	6.29422	4.90533	5.75726	5.19515	5.69971	6.24979	7.28617	5.53026	6.74309	6.59995	6.59756	6.33691	7.73017
SFXN2	Q96NB2	4.93696	4.28956	7.31097	6.8935	5.21382	7.8677	7.49533	5.35961	5.29854	6.87662	6.02899	6.09847	7.33265	5.38982	7.0563	6.55118
NDFIP2	Q9NV92	5.95737	6.85241	7.39915	7.05472	6.33609	5.96945	5.71163	6.08705	6.5122	7.90875	8.1752	9.40498	4.44887	4.4636	4.13901	3.5795
HDAC10	Q969S8	6.35324	4.99405	6.41632	5.71313	5.62971	4.99314	5.61145	7.6786	7.09259	6.2147	9.09383	6.7541	6.3399	4.93609	5.05736	7.12178
CAMSAP3	Q9P1Y5-2	6.09284	6.46091	5.46424	6.78193	6.90364	7.39705	6.67988	6.73758	7.12943	6.90794	4.67783	6.03632	5.46331	5.79229	5.8834	5.59139
NAP1L1	P55209-3	6.47573	6.52293	7.06005	5.80949	5.74645	6.03431	5.97134	7.77363	6.96793	7.27538	6.57193	6.59176	5.72826	4.65515	5.44983	5.36583
NCL	H7BY16	6.4429	6.73121	5.41114	6.45477	6.45526	6.49695	6.37633	6.0561	6.2974	5.63687	6.35703	5.81111	6.42974	6.03872	6.44253	6.56193
ODC1	P11926	8.68024	9.07862	10.1775	8.98882	5.68524	5.68434	6.26482	6.64413	7.05111	6.43778	8.08726	7.14616	2.76537	2.17109	2.48924	2.6483
ANKRD30B	Q9BXX2	4.87333	7.34143	7.29748	4.65793	4.99987	7.22406	4.45085	7.54246	6.27562	4.75734	5.70219	10.8193	7.81064	5.2452	5.37888	5.62338
SETX	Q7Z333	7.21573	7.16237	5.85177	6.36112	5.15246	6.06637	7.26221	4.86415	6.67732	6.19125	7.34669	5.75183	5.38585	5.77485	7.68001	5.25602
TPM1	A0A087WTJ7	6.44329	6.11374	6.7196	4.41608	6.26029	5.34004	4.97347	7.55105	3.70455	6.53386	4.99155	6.9343	6.316	9.07834	7.69546	6.92836
ZBTB41	Q5SVQ8	5.81554	5.65316	5.74648	7.12653	7.08557	6.35663	6.3056	6.12829	5.91972	6.09414	6.16087	5.69915	6.71905	6.65216	6.05726	6.47983
TRPM6	Q9BX84	7.43078	7.68104	6.49542	6.63432	6.12855	7.0089	6.76981	6.62637	6.01146	5.70314	6.1045	6.2906	5.35895	5.12491	5.53807	5.09318
EIF3B	C9JQN7	7.62523	7.71441	8.42459	5.76423	6.47523	8.28694	4.31686	6.35802	7.59436	4.49129	4.91551	4.78802	6.77187	6.0479	5.37949	5.04603
DPYS	Q14117	6.62824	6.76751	5.94116	6.26462	6.43093	6.425	6.16482	6.54665	5.93946	6.7275	6.40044	5.4518	5.76205	6.83211	5.34908	6.36862
POLE4	Q9NR33	4.39813	3.94173	7.51129	4.27262	4.99229	3.43194	5.40252	7.55928	5.74117	4.36529	5.49648	6.98047	6.26419	12.7464	8.00999	8.88616
PCDHB5	Q9Y5E4	6.69827	5.5503	5.27713	5.64059	7.31994	6.79793	6.75953	5.88963	6.0146	5.82778	5.72695	5.02376	7.63102	6.2132	7.31388	6.3155
HNRNPM	M0R019	5.15592	7.41343	5.54503	4.78957	5.83071	5.87098	3.53805	5.93714	7.98596	8.33437	5.18994	8.42674	7.52574	4.94174	6.71624	6.79842
HNRNPM	M0QZM1	7.23021	5.97531	5.67552	4.5632	6.30415	5.28605	7.08781	4.2926	5.08818	5.63354	6.67108	8.82468	7.72184	6.29707	7.29643	6.05234
YWHAZ	E7ESK7	6.51615	5.23036	6.43924	6.33447	6.85156	5.97998	5.90113	6.97416	5.58045	7.89017	6.11942	6.20855	6.10335	5.18799	5.4448	7.23824
GOLGA4	Q13439	6.76028	6.27289	5.66362	5.73452	6.40567	6.93173	6.66898	5.86839	7.7438	6.69602	6.85295	7.32733	6.30024	4.92232	5.14404	4.70722
TMEM208	Q9BTX3	6.4872	6.10126	6.68398	7.18187	4.60761	5.95449	6.77315	5.49249	6.02422	6.25907	6.36753	6.62766	6.4439	6.30818	6.34142	6.34597
KCTD17	Q8N5Z5	6.1788	5.3074	5.86011	6.57266	6.42469	5.92358	5.02288	7.41319	6.62082	6.36098	6.92335	6.44558	6.76246	5.85875	5.96064	6.36411
PRKD3	O94806	6.03699	6.93675	6.67443	5.50696	5.89215	7.07275	4.68513	5.40705	6.21274	5.07249	7.31831	8.4627	5.91491	5.65511	6.56415	6.58737
PEX7	O00628	7.26864	6.4307	6.65432	5.58849	6.80422	5.83708	6.47397	7.07186	6.22416	5.8612	6.66731	6.94743	5.37276	4.42227	6.43228	5.9433
JAGN1	Q8N5M9	5.70499	6.63502	6.91128	7.06162	5.64545	6.23663	5.24294	5.84047	6.355	6.32292	6.82311	5.95683	4.9528	6.67032	7.25871	6.38192
MYO1B	C9K0I9	6.6567	6.79022	7.64389	5.78531	5.70966	5.97778	5.15048	6.20636	5.93002	5.25856	5.78403	6.20284	6.23492	6.23384	7.05806	7.37732
RBM4	Q9BWF3-3	8.40498	6.53489	6.90887	4.19109	6.82482	6.17382	5.70834	6.70116	6.10622	7.0778	7.8004	7.67186	3.96553	4.87162	5.06104	5.99757
BBS4	Q96RK4-2	6.75206	6.68415	7.45762	6.17501	6.40185	6.05599	6.16123	7.00964	7.3494	6.54508	7.29545	7.43955	4.96985	4.87907	4.43188	4.39218
PRC1	G3V3N7	5.96526	6.66388	6.09892	5.07516	6.25662	5.18473	7.73851	6.54777	6.34029	4.98137	6.63692	6.2019	5.45713	7.02189	6.88548	6.94417
CHD3	H7C0J3	7.54248	7.16087	5.96626	6.05697	5.17698	7.54591	6.91827	6.73653	4.85514	5.08314	6.44414	5.58953	5.71173	8.34639	5.62698	5.23868
MYO5B	Q9ULV0	5.56875	5.06225	7.03581	5.28288	6.28954	5.92299	6.17411	7.10164	5.78163	7.32894	6.60941	7.34462	6.32885	6.34634	5.97555	5.84669
KIAA1671	Q9BY89-2	6.74341	4.60015	6.7404	6.48337	6.4684	5.33137	6.00285	6.47087	5.90757	7.01261	6.02081	6.3408	5.54565	7.83413	4.88604	7.61155
ARRDC3	Q96B67	5.86366	7.01204	5.37974	6.3817	6.19922	5.25143	4.90288	5.98218	5.66848	6.45973	6.10484	6.29714	7.40828	7.32233	7.98844	5.7779
SYNE3	Q6ZMZ3-2	5.68147	6.96202	6.99449	5.85674	4.71443	6.8106	6.10382	6.37725	5.36163	6.00211	7.23717	5.60771	6.70305	5.32762	7.21785	7.04204
SHMT1	J3KRZ5	7.4912	6.8412	7.31734	4.41098	4.38882	5.77091	6.22447	6.12571	6.35731	6.80443	5.38597	6.27495	7.7156	4.76602	5.93144	8.19366
SREBF2	Q12772	7.43885	5.24617	6.56426	8.05146	6.03766	7.83337	6.15289	5.82868	7.50634	6.67847	5.2883	5.94996	6.08701	4.59545	5.46459	5.27654
ULK2	Q8IYT8	7.70145	5.56345	5.74491	6.60589	5.76506	6.33044	5.48482	6.58595	5.74492	6.01674	6.60477	5.37125	7.17503	6.59243	6.16291	6.54998
ADGRL2	A0A6I8PLG0	5.84509	6.35394	5.98417	6.60276	5.66714	6.09504	6.20595	5.89822	5.99137	6.53689	6.32902	6.12934	5.7851	6.86146	6.11324	7.51129
HOMEZ	Q8IX15-3	7.53068	5.9272	6.16046	5.91252	5.43802	6.99292	5.35332	6.09012	6.04165	5.84026	7.15667	5.0274	7.03306	5.72948	8.39011	5.37615
PPP2R5C	Q13362	5.91885	4.03313	5.22397	7.37456	6.1863	5.97093	8.79768	6.33072	7.78877	3.89411	4.53876	5.13073	6.90267	6.68739	5.88717	9.33426
NOLC1	Q14978-3	6.43341	7.12818	5.49335	7.37909	6.27243	5.34716	5.18744	4.94519	6.41965	7.46956	5.45798	7.04852	6.01605	6.27763	6.85373	6.27064
TMEM267	Q0VDI3	5.35979	6.88691	6.69071	6.68101	6.5849	6.6333	5.84867	6.83595	5.8734	4.57287	6.39718	7.2202	5.52738	5.90252	5.72494	7.26028
TUBB2A	Q13885	7.44168	8.54185	6.60621	6.3346	5.88242	7.3364	6.52171	8.23591	5.45398	5.08092	4.80665	6.4078	5.5092	5.86316	4.18113	5.7964
MAPK11	Q15759	5.72916	5.81574	9.96582	6.02355	6.36069	7.64453	5.23795	5.07416	5.95781	6.60011	6.25203	7.46059	5.99631	4.90776	5.49316	5.48064
MAPK12	P53778	6.79753	6.10262	7.1694	6.38221	5.95652	6.03628	6.22129	8.28767	5.80916	6.33744	5.46862	6.85642	5.95991	5.19719	5.3063	6.11141
CEP170P1	Q96L14	7.06073	5.94681	5.62598	5.46919	4.29846	7.43822	5.56901	5.49417	8.82523	6.43543	5.59239	6.27144	7.79909	6.09537	5.53187	6.54662
MAP7D1	Q3KQU3-3	5.52224	4.98063	7.70935	5.76837	6.45417	6.48239	6.6311	5.74006	6.07857	7.11825	6.69041	5.46543	5.66021	5.23065	7.9491	6.51906
FUNDC1	Q8IVP5	8.46374	6.14018	6.71638	6.83198	6.01637	6.85434	7.46855	5.72882	6.29935	5.30675	6.39406	5.86295	6.22282	5.57614	6.26968	3.84788
CLCN2	P51788	6.44712	6.34411	6.88178	6.98642	5.52237	6.30694	6.29447	6.4571	6.00238	5.65215	5.69892	6.60177	6.3495	6.7935	5.59628	6.06519
STAU2	Q9NUL3-7	6.90056	7.16714	6.25444	6.62719	6.07583	6.21302	7.53975	5.99774	6.21311	6.66584	6.06452	6.74394	5.17479	6.06755	5.07745	5.21714
SON	P18583-6	6.93838	5.89194	4.74163	7.22362	5.33295	4.99552	7.54202	6.27613	5.20174	4.60501	7.31506	5.22794	6.34464	6.87702	8.17676	7.30963
PHF23	Q9BUL5-3	8.03909	6.2121	6.28892	5.93325	5.65045	5.70097	9.83068	7.04498	6.49856	4.98991	5.29704	5.68416	5.23755	6.0969	6.12972	5.36574
SMARCE1	Q969G3-2	6.71127	6.07766	6.15629	5.78004	6.66509	6.326	6.33486	5.65193	6.69502	5.53114	6.32623	6.40801	7.14982	5.72368	6.614	5.84897
VARS1	A0A0G2JJT9	6.20074	6.2277	6.71533	6.69194	5.93383	4.83406	6.43848	5.65015	7.62187	5.69556	6.72631	6.73859	6.31241	6.16193	5.82185	6.22926
FOCAD	S4R400	6.7392	6.50901	6.21295	6.1716	6.43382	6.82446	5.83772	6.8392	6.3465	6.61439	6.4451	5.77734	5.91328	6.35304	5.08519	5.8972
TNKS1BP1	A0A2R8Y5C4	7.10542	6.00475	5.58961	5.2686	7.76269	6.62582	6.44968	6.27376	6.85361	7.94656	6.62776	5.03877	5.10126	6.96211	4.26245	6.12714
TSC22D2	O75157	6.49441	4.67648	6.1712	7.47981	6.80262	6.03201	5.15235	6.49727	7.14067	6.94614	7.20747	7.20099	4.75977	7.03123	4.22199	6.18561
PDIA4	A0A499FI48	5.72222	6.2421	5.7189	5.41692	6.15349	6.7582	7.11847	5.33507	6.08964	7.04245	5.92705	6.02903	5.69691	7.21337	6.4385	7.09769
GOLIM4	F8W785	4.77737	6.41572	6.49355	6.08604	7.58215	5.93787	6.34375	6.10784	6.61555	4.77056	6.29855	6.33733	7.39764	6.02244	6.58487	6.22877
ILVBL	E9PL44	7.36814	7.34169	6.19993	6.35627	5.99259	5.75065	7.90849	4.35915	6.864	4.87107	4.51831	5.88941	6.54604	5.96194	6.57196	7.50036
HAUS7	Q99871-2	6.75816	5.3333	6.09651	5.77595	6.19133	6.06921	6.28539	7.95842	5.20538	5.42247	4.82494	7.15035	5.93883	7.63123	6.14292	7.3156
CHD1	A0A087WVF4	5.41143	7.77072	4.05499	6.80064	4.28979	7.61309	5.7824	5.21992	4.26112	7.30978	5.94696	6.27468	6.95697	8.64869	5.12326	8.53557
PDS5B	A9IYQ1	6.13415	6.3226	6.36789	6.65828	4.82335	5.66534	5.84581	5.69401	6.25621	5.42178	5.61493	5.76504	6.74097	6.89913	7.3852	8.40532
TRPC1	P48995	7.26418	6.78036	5.37318	6.70764	5.22815	5.99034	5.77959	5.94138	6.79733	6.78564	6.61718	5.8549	7.22967	5.05074	6.9712	5.62853
SF3B2	E9PJ04	6.54958	6.33994	6.95336	5.5986	6.53918	6.1503	7.60009	5.33362	7.15224	6.77478	5.80268	6.06046	5.95367	6.2545	5.55266	5.38435
ENO3	P13929	5.73176	5.12952	7.30416	5.92468	7.08218	7.38789	6.9817	8.06523	6.19067	6.61012	6.17112	6.14677	5.92136	5.00539	5.42129	4.92615
ENO1	P06733-2	5.34089	5.60532	7.25262	5.5328	6.88145	7.78372	5.1894	7.14192	6.96758	6.77758	9.21724	5.50548	7.25424	5.75184	4.0603	3.73762
ATRX	P46100-4	5.7225	6.12428	5.21376	7.59758	6.02473	6.8729	7.09601	5.95364	6.15118	5.63313	6.54	5.20478	6.19477	7.24926	6.16528	6.25619
ATRX	A0A096LNL9	6.07955	6.06085	5.49721	6.70411	6.9949	6.81612	5.5341	6.24774	6.67348	5.4045	5.98416	6.3302	7.50758	6.58988	6.47807	5.09754
ATRX	A0A096LNL6	6.18943	6.36056	5.46311	6.876	6.725	5.63722	7.94556	6.32565	5.542	5.01529	5.63437	7.23908	6.73044	6.2987	6.96644	5.05115
PABPC1	A0A7I2YQ88	6.9917	6.65795	7.12912	6.05792	6.17068	6.29952	6.28403	6.66454	7.11611	6.32073	6.85291	6.85435	5.19216	4.9443	5.25991	5.20407
ATP10D	Q9P241	6.06437	6.06995	6.74883	6.53854	8.75547	7.55483	7.00288	6.53661	5.39614	5.94502	5.14266	5.82131	5.47708	6.30192	5.49242	5.15196

HCFC1	P51610	6.12618	6.0587	5.34199	5.59381	5.84364	5.51023	6.04472	6.49804	5.81936	5.77445	6.34514	6.57955	7.26936	6.70458	6.69646	7.79378
USF1	P22415	6.19612	6.77991	5.43993	5.80467	6.0277	7.64243	6.05484	7.25272	8.64898	5.01749	7.7231	4.92346	6.6353	4.69023	5.44352	5.71959
DST	A0A0U1RQJ2	6.47387	6.25896	5.68376	6.46274	6.09606	6.68157	5.34276	5.28755	5.77874	5.76895	5.85015	6.04019	6.15859	7.45369	7.73798	6.92445
MACF1	E9PLY5	6.4081	6.37365	7.27359	6.24762	5.19698	6.71257	6.01596	6.1931	7.11562	5.94032	6.42101	5.32242	6.14299	6.17828	6.71072	5.74705
USF2	Q15853-3	7.20521	3.98108	7.02096	7.97522	7.37431	5.44373	4.89897	6.65124	6.79082	5.92363	7.43113	5.27957	6.525	6.21769	5.12071	6.16073
CENPO	Q9BU64	5.82199	5.88941	6.54837	6.07172	5.77197	6.92128	6.35231	7.00779	6.65139	6.00456	6.12744	7.23881	5.26534	6.04705	6.17356	6.10703
TPP2	A0A494C0U1	6.72378	6.16896	6.30128	6.57068	6.59975	5.85292	6.0817	6.07016	6.19659	5.78357	5.7445	5.877	6.72508	5.89699	6.62102	6.78603
NUP85	J3QL91	6.97271	7.56538	5.63508	6.07572	5.21785	5.13026	6.85114	6.16729	5.5491	5.98079	5.2124	5.23783	7.32896	6.52476	7.23061	7.32012
FBXO38	Q6PIJ6-2	6.50215	6.41622	6.26515	6.47799	6.21965	6.19463	6.0821	6.0862	6.28709	6.23484	6.1093	6.23535	6.32969	6.33409	6.18014	6.04541
HSP90AA2P	Q14568	5.42042	7.39842	6.50549	5.66802	5.67952	7.85921	5.6381	6.11568	5.39413	5.63295	6.51079	7.0934	5.82428	5.98248	6.36718	6.90992
BCL3	P20749	7.15905	5.96851	7.65928	5.88828	6.61021	5.79399	6.04121	6.52493	5.1419	7.28953	7.08587	5.57416	6.7989	6.22646	4.81956	5.41816
WNK4	Q96J92	6.42722	7.25998	5.93326	5.2648	5.01188	6.96808	6.78042	6.81296	7.70809	5.13101	7.03282	6.56385	5.97506	5.15625	5.95726	6.01704
SLC48A1	Q6P1K1	6.60768	6.80424	7.14135	7.32141	5.37116	6.4993	5.96672	6.80698	4.39687	5.7088	4.10641	5.93202	6.99806	8.09071	5.37215	6.87612
OCIAD1	D6RDK6	7.15101	5.1188	6.49857	6.5515	5.98504	4.59765	7.43156	5.20013	10.6193	3.91075	6.1861	5.48574	6.14376	4.52765	6.62602	7.96642
CD46	P15529	6.65258	6.69613	5.49394	6.56435	6.70329	5.78988	6.2427	6.307	6.65728	6.01186	6.65186	5.83698	6.31433	5.80329	6.3876	5.88694
ZBTB46	Q86UZ6	7.67555	6.38537	5.67001	6.59825	6.50773	6.3282	5.86585	4.63808	7.0609	7.09985	5.22827	5.78614	6.09633	6.92345	6.97393	5.16208
ZNF664	Q8N3J9	5.45563	6.16184	4.64912	5.32505	6.06278	5.95964	6.25148	6.71474	5.24843	5.50252	6.40523	5.99989	6.84694	6.9379	8.78441	7.6944
RNF213	Q63HN8	6.4827	6.98761	6.52362	6.10013	6.31455	6.44177	6.84152	6.39162	5.97609	6.43296	5.69007	6.15099	5.9524	6.10979	5.77764	5.82655
FLII	Q13045-2	6.06578	5.99529	6.94383	6.00189	6.22814	6.40592	6.4381	6.50142	6.57558	6.89298	7.11635	6.55118	5.65477	5.7558	5.2522	5.62077
ALG13	Q9NP73-2	6.47884	4.35075	5.5105	4.9508	6.25933	4.95612	5.40871	6.14837	6.13407	5.47043	5.6824	6.60938	6.7549	8.4543	8.59382	8.23728
SLC20A2	Q08357	6.49041	6.27635	4.43967	6.44333	6.73598	5.39201	7.0314	6.13197	6.973	5.46083	6.79895	6.82133	5.84458	6.22154	6.40659	6.53204
SRGAP2C	P0DJJ0	7.55885	7.8373	6.81948	6.81234	6.42396	7.94359	5.80865	7.31313	4.43316	4.69323	6.47644	6.83093	7.64725	4.75089	4.21157	4.43923
OTUD4	Q01804-5	6.15822	5.33979	7.54283	5.38038	6.51921	6.77233	5.85117	4.88233	5.99327	6.81179	7.27605	7.26486	5.6647	7.43276	5.36395	5.74637
MKI67	P46013-2	7.20466	6.53551	5.89958	6.92614	6.26416	6.05079	5.97323	5.43244	5.65328	6.34174	5.87894	6.33036	6.06223	5.76705	6.15416	7.52573
MAP2	P11137-4	5.86016	5.7502	5.60006	6.48401	6.17596	6.66128	5.89618	6.90396	5.20841	6.76072	6.69286	6.97866	7.44979	6.47349	5.7954	5.30886
BAP18	Q8IXM2-2	5.5296	4.15581	4.94154	4.9988	4.68233	4.99165	5.09236	6.97957	4.29703	5.41953	5.33517	5.60444	6.81282	10.6152	10.3656	10.1785
PARL	C9JNP8	5.97859	6.01393	5.72446	6.18296	5.82319	5.93113	6.13337	6.32967	5.91268	6.46	6.28106	6.36538	6.73839	6.50689	6.78796	6.83036
PSMC3IP	Q9P2W1	5.66275	5.96459	6.12219	5.40375	6.57959	6.46507	5.4266	5.86703	5.96493	6.37759	6.76074	6.47601	7.00525	6.62425	6.99288	6.30677
CHMP2A	M0R1T5	7.74826	5.64071	7.72582	6.8078	5.94471	6.57865	6.88003	6.78564	5.09985	6.90342	4.85158	8.08485	2.81175	6.46343	5.41939	6.2541
HYOU1	A0A494C039	7.66145	6.61359	7.29655	6.43563	5.87157	6.78537	6.75145	7.15221	6.22543	6.10032	5.56582	5.54395	6.84095	4.83935	6.21068	4.10568
HYOU1	J3QL06	7.67446	6.44312	6.4199	8.14672	7.7154	7.11275	7.03296	3.87704	6.65658	7.10203	3.69435	4.07991	6.63606	4.42213	5.79656	7.19004
MACIR	Q96GV9	6.02447	4.75276	6.84521	5.61709	6.74313	7.61348	6.30184	6.59152	6.37571	7.36942	6.12398	6.17406	6.86986	5.59988	5.51455	5.48304
CCDC8	Q9H0W5	6.26148	6.30228	7.90583	6.74723	6.73242	7.78377	6.49914	6.04981	6.20315	5.85667	7.46755	7.26257	5.1073	4.76548	5.41617	3.63915
SCNM1	Q9BWG6	5.9952	6.41764	6.05104	6.64339	6.01924	7.20134	6.07932	6.72163	5.60913	7.15874	6.59648	7.87591	5.71508	5.96062	4.21687	5.73839
CBX6	O95503	6.80609	6.77889	5.61529	5.76706	7.14839	6.7885	6.4704	7.16875	7.46589	6.91035	5.79704	6.36534	5.7317	6.90381	4.46606	4.81645
THAP4	Q8WY91	6.36093	6.95578	9.12612	9.05827	6.02568	5.25767	7.38883	7.46461	6.14526	6.13028	7.36048	6.13704	4.46474	3.54969	4.36817	4.20646
HMGA2	H0YFY4	5.3726	6.16321	5.08564	5.93265	5.08246	5.23699	4.72217	5.97654	6.15785	6.29068	6.31298	5.37334	8.0798	7.93237	6.75144	9.52928
NDFIP1	Q9BT67	6.96466	6.98358	6.84964	6.02896	6.63414	7.13177	7.15344	6.17015	6.59394	7.5835	8.31862	6.6513	3.96769	4.05086	4.52244	4.39531
UBTF	E9PLT2	6.3549	5.89709	5.68791	7.2439	5.09308	5.10987	6.15	5.42418	6.28243	6.66087	5.18556	6.56289	6.41363	6.57844	7.10638	8.24887
FAM204A	Q9H8W3	5.99568	6.32713	6.94942	6.43057	5.95064	7.03532	6.33922	5.83205	6.6981	6.75441	6.45943	5.91478	5.56426	5.86592	5.78511	6.09798
SFT2D3	Q587I9	6.34006	6.53493	5.55163	6.8481	6.24034	6.55295	5.97337	6.80801	6.91276	6.00548	5.32605	6.0767	6.41544	6.37141	6.03305	6.00974
PCDHAC2	Q9Y5I4	6.94708	5.71892	6.88454	6.33357	5.38234	6.4958	6.04594	7.86212	8.6513	5.64229	6.73449	6.47276	5.00971	5.9274	4.90099	4.99075
BCLAF1	Q9NYF8-3	5.96027	7.20376	5.88218	6.89199	6.26067	5.8455	6.10598	6.10649	5.63334	5.44117	6.1507	6.79808	6.11289	6.23411	6.5089	6.86396
NDUFV3	P56181	6.40448	5.85507	6.6214	4.79422	7.29148	5.91136	6.01536	5.07255	5.00597	5.37227	5.90601	7.33608	8.6552	5.64645	6.63836	7.47374
PPP2R2D	Q66LE6	6.40466	5.24108	6.68457	5.50193	6.50833	6.4382	6.92348	6.39661	5.86231	7.23166	6.7456	6.78304	5.5968	5.9277	6.44769	5.30636
TMPPE	Q6ZT21	6.39073	5.94422	5.9787	7.02527	5.93903	5.58008	6.53146	6.2165	5.93644	5.84521	5.80394	5.63398	7.06117	6.5259	6.92786	6.6595
FOSB	P53539	6.83579	5.33645	5.50898	6.15341	6.85125	5.75931	6.16398	6.1007	6.99089	5.69599	6.09431	5.37854	8.17094	5.66672	6.60258	6.69018
SPTBN1	Q01082-3	4.9555	7.0532	5.27015	7.39193	5.93152	6.13238	5.49739	5.51311	7.38405	5.64594	6.13999	4.94237	7.1115	7.03027	7.48618	6.51454
SPTB	P11277-2	9.37833	5.94972	7.42347	7.37955	5.52939	5.46713	5.28941	5.00889	5.33121	5.50508	4.68682	6.15204	6.50328	7.22781	5.5629	7.60496
PIP4P2	Q8N4L2	5.65599	6.95997	5.80956	5.60417	6.26165	6.39563	6.94734	6.19559	6.11831	7.5226	6.24202	6.02484	5.9227	5.9901	6.57904	5.77048
MAFK	O60675	7.54779	5.65341	6.11963	6.80538	5.52925	6.59212	7.70383	5.82479	5.96538	6.30457	4.5016	5.96962	5.70942	5.7277	7.0811	6.96443
UBE2D1	P51668	6.44388	6.59575	6.86417	5.16216	6.02914	6.31258	6.02348	6.78336	5.64869	6.28138	6.15098	6.44808	5.39185	7.20651	6.67453	6.88347
GPATCH3	Q96I76	5.75093	5.93701	6.29491	6.5462	6.92273	5.87082	6.22603	6.23358	6.26862	6.55791	7.28332	7.50449	5.89887	6.13433	5.34615	5.2241
SLC10A4	Q96EP9	6.33345	6.77369	7.31902	6.37414	6.25477	6.64173	4.53251	6.38595	6.52544	7.52797	5.65545	6.22684	5.99767	7.3438	5.49769	4.60985
ZNF865	P0CJ78	6.36673	6.43486	5.94411	5.90655	7.43198	6.46809	7.06414	5.55152	3.83624	5.67098	6.95367	6.35892	7.08532	6.90092	7.03494	5.99103
MARK4	Q96L34	7.41622	7.07546	7.04939	7.17145	5.71813	5.99839	6.35127	7.52023	7.37735	6.54189	6.92894	6.58608	4.79135	4.81121	4.1599	4.50275
MYL6	G3V1V0	6.44553	4.66355	6.97161	5.68883	6.96287	4.19352	4.70988	5.71737	4.87811	4.91657	3.93671	6.82647	6.99833	8.88978	9.35906	8.84181
RTL5	Q5HYW3	6.87821	6.391	4.77187	6.90147	6.91183	6.77171	6.33897	5.44204	6.74147	6.17642	6.92001	6.17534	6.13461	6.42969	5.78713	5.22824
RACK1	D6RHH4	6.618	6.50617	6.06486	8.04582	5.84393	4.86076	7.33013	7.17374	5.59699	4.04158	7.58481	6.33202	6.97748	4.56639	6.75806	5.69924
EPN1	Q9Y6I3-3	6.08422	5.72533	5.836	5.99123	8.03586	6.01548	5.54114	5.17991	6.07955	6.4468	5.78698	6.98925	7.04764	5.84613	7.21952	6.17497
RTN4	Q9NQC3-5	6.15939	5.90939	6.03444	5.86719	4.8455	5.74995	6.76538	6.62602	6.86783	5.98113	5.0148	6.94233	6.84259	6.71341	6.89774	6.1439
RTN4	Q9NQC3-2	6.61188	7.26479	6.13648	6.59491	6.41557	4.7233	7.03687	6.5775	6.3803	6.92926	5.11935	5.55226	5.86588	6.40655	6.56585	5.81925
CIT	O14578-3	6.24033	8.81516	7.25625	5.76256	7.22578	4.4718	5.24849	6.21483	6.27036	7.65792	7.44367	5.90627	4.82909	6.4171	5.13679	5.10359
KRT18	F8VZY9	6.43478	6.75191	5.63921	5.91397	5.77104	5.5071	6.68305	6.66898	6.14057	5.62361	5.40585	6.34223	6.76175	6.33953	7.31291	6.70352
PML	P29590-5	7.38218	5.11212	5.54429	5.62791	6.79805	6.59486	5.34096	8.30896	5.31747	7.45784	4.69565	5.84973	6.33753	7.13141	5.45672	7.04433
PCBP2	F8VZX2	7.33848	5.59764	7.10888	5.17581	5.48854	7.78032	5.41191	5.59586	5.73586	6.62845	7.20215	6.2605	6.44843	5.45469	6.15763	6.61483
PCBP2	F8W0G4	6.40075	5.22424	7.38863	5.86192	5.79684	5.9019	5.54304	4.75424	7.22148	6.57189	6.03339	6.908	5.90617	6.14999	6.85012	7.48737
NFIC	P08651-5	4.39762	5.57172	7.52179	6.19092	6.35646	6.93624	6.23992	5.74519	7.72398	5.36752	5.79916	5.85263	6.51895	6.5666	5.54382	7.66747
NFIX	Q14938	7.05531	7.1936	7.56785	7.35714	6.37982	7.88058	6.14429	4.6775	5.09219	6.35915	7.06509	5.67099	6.09179	5.63879	5.36849	4.45743
ZNF627	Q7L945	4.26781	8.07135	7.19302	7.17246	6.34637	6.52631	6.19869	6.54606	4.52981	4.57524	5.60666	7.31064	7.34136	7.06016	6.38618	4.8679
ZNF791	Q3KP31	7.19054	8.04142	6.0823	6.45561	6.79126	5.78844	7.00605	6.28305	5.65817	6.31461	6.54086	6.68167	5.07079	5.05626	5.63501	5.40397
LMO7	E9PMS6	6.08928	5.9985	5.8825	6.96514	5.65876	5.9643	6.2149	5.33023	6.042	6.28658	5.88803	6.46881	6.86459	6.42036	6.95203	6.97399
TMEM158	Q8WZ71	6.23278	5.95278	5.66623	7.29276	7.22996	7.03899	7.42863	6.42356	5.81227	6.52737	7.54543	6.98079	5.30106	5.1795	4.53153	4.85638
ZNF568	Q3ZCX4	5.70484	5.70496	5.76296	6.01428	5.46394	5.2895	6.7164	6.29904	4.76138	5.562	5.84364	7.02859	7.42685	8.32442	6.98358	7.11363
ZNF625	Q96I27-2	6.16995	6.54312	6.26403	7.05069	5.96168	6.02808	6.65635	6.04552	5.66567	5.80788	5.94968	6.65943	5.99625	6.57963	6.40965	6.21239
ZFP1	Q6P2D0	5.55913	6.57261	6.62195	6.26325	5.83723	6.56992	6.66614	4.48841	5.86191	6.95452	5.92644	6.02274	7.40513	5.93509	6.04858	7.26694
ZNF624	Q9P2J8	6.63492	6.09624	5.80639	6.15736	6.66314	6.19794	5.8929	5.62924	5.50233	6.28632	7.08142	6.32318	6.33281	7.15074	5.48244	6.76264
ZNF23	P17027	4.77215	7.25401	5.86923	8.79012	7.56574	4.05513	6.98853	5.86596	5.61776	4.17505	4.66136	4.63314	6.94781	7.96279	8.50139	6.33985
ZNF286B	P0CG31	6.9061	6.39464	5.86923	6.82795	3.84439	4.8115	6.28815	5.95183	6.62245	7.02658	7.06543	6.73726	5.35075	6.5172	7.05455	6.732
ZNF90	Q03938	6.76752	7.70984	6.79617	8.40644	5.27017	6.37945	6.86134	5.86414	6.29232	6.86471	6.46387	5.97132	4.35271	5.0496	5.63576	5.31464
ZNF391	Q9UJN7	6.25992	6.3602	6.42934	6.19221	6.5997	5.72415	5.35399	5.01848	4.98845	5.44577	5.86855	7.41249	6.53087	6.39697	6.98019	8.43871
ZNF3	P17036	6.12387	7.69048	6.5359	7.97341	5.73981	6.47753	7.51047	6.23793	4.31157	5.83125	7.09676	3.96372	5.94178	7.01828	5.5754	5.97183
ZFP37	Q9Y6Q3-2	5.42873	6.92658	6.28081	6.10349	7.14233	5.61576	5.15436	5.31202	5.74341	5.22748	5.47753	4.87502	7.44253	7.86712	8.00779	7.39503
ZNF121	P58317	5.79191	6.08593	5.62154	7.11297	6.51462	5.98602	5.91909	6.22524	5.7085	5.62503	6.15064	5.86323	6.46076	6.80424	7.54024	6.59004
ZNF350	Q9GZX5	5.65018	6.8514	8.89077	5.71263	9.44965	4.4558	4.78934	6.98066	8.25936	6.91974	5.50901	6.17191	4.67179	5.01805	4.85495	5.72477
ZSCAN30	Q86W11	7.63463	6.0721	7.66322	4.56119	8.31127	5.78825	4.20979	6.79975	5.79972	6.7806	4.93008	4.46152	5.80467	9.31815	7.01877	4.84629
ZKSCAN7	Q9P0L1	6.3943	7.27828	5.51078	5.56379	5.45448	6.31645	7.05837	7.35072	6.5475	4.74959	7.13741	6.80944	5.72291	6.60196	6.08236	5.42165
ZNF14	P17017	6.22559	5.99904	6.34659	6.81328	6.619	6.62183	6.43748	5.85676	5.43025	6.10218	6.46014	6.63182	6.00712	6.21666	6.40612	5.77422
DNM1	Q05193	6.915	6.11005	6.80725	6.4538	6.26252	6.78992	6.83757	7.92554	5.82059	6.56608	7.05308	5.27646	5.54754	5.94885	5.61846	4.06729
MTHFSD	Q2M296-3	6.14396	6.98825	6.07496	6.05766	5.61086	6.34282	5.44533	6.27374	6.35981	7.00725	6.50151	6.833	5.17877	6.28869	6.3146	6.57878
PLD3	M0R1F7	6.06542	6.06451	6.60561	7.48661	4.94077	5.97994	5.76811	7.15683	8.33413	5.87486	6.81254	5.71757	5.65992	5.77733	6.01348	5.74238
RAB39B	Q96DA2	4.86953	6.8476	6.27237	6.56215	6.6503	4.76407	6.65609	5.30929	4.9619	7.37201	8.01236	5.89095	6.68529	5.46954	7.10046	6.57609
TRIM7	Q9C029	6.96034	6.56032	4.97398	8.35647	6.13494	6.16718	7.06612	6.57765	6.4411	6.4425	6.84798	6.70702	5.38802	4.29486	5.80505	5.27647
ZNF670	Q9BS34	6.29436	6.56151	6.28617	7.24053	5.66269	6.24081	6.29066	5.83234	5.83383	6.33517	5.86663	6.06764	6.39349	5.9398	7.40192	5.75246
KIF7	Q2M1P5	5.52823	6.67798	6.945	6.37998	5.12742	5.88495	7.96523	4.384	5.83511	7.78418	7.11364	7.03812	4.64606	6.43235	6.56655	5.69119
GOLGB1	Q14789-4	7.28644	4.75973	7.27847	6.92996	6.4507	5.82764	5.19728	5.83537	5.25437	6.79596	5.87628	6.00216	6.13086	6.21955	6.95758	7.19764
ZNF799	Q96GE5-2	5.90403	7.23045	7.897	7.34415	6.28241	5.73813	5.78428	6.83617	3.80995	4.92817	6.27205	7.59005	6.06953	5.51598	6.1101	6.68755
ZNF607	Q96SK3	5.57616	6.76103	6.00075	7.79888	6.58084	6.80558	6.17003	6.7889	5.67647	6.26131	5.94464	6.95279	5.04373	5.99506	6.3902	5.25364
ZNF317	Q96PQ6	6.08177	6.5879	6.47967	6.59289	6.06745	6.23651	6.29685	6.19399	6.13952	5.9083	6.19508	6.10191	6.47489	6.35243	6.58006	5.71078
ANKLE1	Q8NAG6	6.15653	5.88536	7.07233	6.93597	6.20097	6.80693	5.37513	5.79107	7.13698	6.32382	5.65493	6.41598	7.02411	6.10933	5.82635	5.28422
SEC61A2	Q9H9S3	5.79875	7.49189	5.5496	5.84433	6.60992	6.31146	6.94088	6.2937	5.76799	6.05747	5.59118	5.492	7.14082	7.23326	6.26338	5.61338
ZNF136	P52737	6.82788	6.58906	5.18176	6.55582	6.01328	5.95087	6.38577	7.74148	6.68457	5.31407	6.48099	6.30004	5.11877	6.59974	7.10883	5.14707
THUMPD2	Q9BTF0	4.95593	5.74676	6.49283	5.34348	5.71886	6.55029	5.57707	5.83508	5.82646	6.26336	6.15197	7.03956	6.44213	6.8536	7.81666	7.38594
ZNF367	Q7RTV3	6.6234	6.36874	6.58419	6.67212	5.95655	6.34293	6.3603	6.20997	6.61293	6.66987	6.094	5.95789	6.36007	5.38506	6.08381	5.71817
ZNF669	Q96BR6	6.23951	7.01163	6.03178	6.45029	7.58517	6.68024	5.59236	5.45289	6.26201	7.64358	6.50014	7.37255	4.68766	4.67853	6.29527	5.5164

ARHGAP12	Q8IWW6-3	6.08999	6.14877	6.17616	5.7854	6.65926	6.4032	6.22208	6.78744	6.32343	6.35115	6.47861	6.09345	6.35976	6.04676	6.31054	5.76402
ARHGEF2	V9GYF0	6.91888	5.36557	7.87872	5.39956	6.25491	5.64716	6.17733	7.74722	6.01829	5.87117	6.4914	7.90396	4.63114	6.55389	5.21632	5.92448
ATP5F1B	F8W079	9.08271	6.27301	7.02826	6.3258	5.14208	4.87894	5.46553	4.15762	4.11613	4.93786	5.24944	5.13625	7.5461	10.5795	6.47458	7.60621
MXRA7	P84157-3	5.5859	4.98351	3.7268	5.88349	3.94792	3.19957	4.95309	5.33259	3.88081	4.54176	4.94511	6.24216	6.05332	12.3814	10.2158	14.1267
NFKB2	Q00653-4	5.94871	6.107	6.58356	5.77828	6.83401	6.842	7.08281	5.44637	6.55688	6.10543	6.04666	5.58374	6.82915	6.28435	5.87453	6.09652
TMEM160	Q9NX00	6.023	4.33495	5.12179	7.56003	6.46385	5.24893	6.60886	6.63604	8.13788	4.74673	5.96303	7.52009	7.59225	6.57938	6.44764	5.01554
CAMK2A	Q9UQM7-2	5.49095	6.99596	6.8977	7.58304	7.40174	4.83622	6.77158	7.07459	5.15071	6.87678	5.58541	4.95651	5.17417	6.6965	5.83069	6.67745
CAMK2D	Q13557-12	5.7141	6.41341	7.13752	6.14987	5.91667	6.65299	6.20091	6.50954	6.05681	6.25192	6.8556	6.3934	5.98185	6.03193	6.19525	5.53824
SDHB	A0A087WWT1	8.03635	6.24217	5.49251	6.60872	5.45208	6.07807	5.27839	6.59245	6.72748	5.98137	5.81705	5.79358	6.38854	4.86799	7.55247	7.09076
C19orf25	Q9UFG5	6.72654	5.2207	4.78059	5.43118	6.30828	2.95755	5.30931	4.9172	3.70587	6.4662	4.37347	5.67768	4.70981	13.2209	8.75826	11.4365
FAM174A	Q8TBP5	6.72468	6.29721	5.95839	5.27648	5.97646	5.6602	6.03001	5.32162	6.16818	6.11305	6.05236	6.51001	6.81422	5.79768	7.73576	7.5637
TOP2B	H7BZ82	6.61254	6.45124	5.52883	6.80997	5.11877	4.35749	6.82054	5.5629	6.86881	6.42269	5.44377	6.13466	6.34343	6.3701	7.89861	7.16565
POTEKP	Q9BYX7	2.7958	1.76318	21.6659	4.64235	13.1508	11.0517	18.4916	3.64628	2.48746	1.66901	6.05085	2.24354	1.90833	2.19441	3.25519	2.98356
ANGPTL4	Q9BY76	6.86041	5.57114	5.39875	6.48477	4.15576	6.10771	9.12497	4.46075	8.17131	6.14142	5.21056	5.98347	6.89878	5.55062	7.42311	6.45649
METTL9	Q9H1A3	6.435	6.35414	5.72352	6.85933	6.10475	6.24605	6.32495	6.15536	5.99707	5.88533	5.57576	6.52123	6.34047	6.43542	6.30284	6.73878
RBBP5	Q15291-2	5.60154	7.60489	6.09042	6.85788	5.61932	6.26061	6.31517	5.69078	6.73045	6.74645	5.12739	5.83555	6.59415	6.56289	5.7655	6.597
CACNA1C	Q13936-2	4.91858	6.0642	6.75418	6.26348	5.56659	7.15349	6.09411	6.47132	6.36427	5.63025	6.53784	5.02516	8.18066	7.04202	6.39949	5.53439
EIF4EBP1	Q13541	4.58847	5.76961	4.27003	4.08849	3.92253	3.37673	4.08843	4.21546	3.45786	4.99481	3.89085	3.87616	4.90732	16.5347	9.94082	18.0777
PYGO2	Q9BRQ0	5.15555	5.97155	7.73387	5.87973	5.88647	5.89935	4.50041	6.72997	7.88969	6.48383	5.8883	5.73495	6.08556	7.01361	7.27545	5.87171
MBD6	Q96DN6	6.33367	6.27106	5.0754	5.65779	5.3243	6.9799	8.1237	6.04769	7.04935	7.5064	6.58745	7.47853	4.93769	6.7581	5.39023	4.47875
SMIM20	Q8N5G0-2	5.98433	5.95203	5.46063	5.3322	5.99488	5.00334	5.46375	6.64715	5.91102	5.87072	5.84524	5.51213	6.85321	8.38222	7.57518	8.21198
PRMT6	Q96LA8	5.02187	5.39312	5.70962	5.3062	5.7983	6.39897	6.27638	4.77406	6.05897	6.181	6.53238	5.8119	7.50801	7.79198	7.92441	7.51283
H2AC21	Q8IUE6	6.14901	5.72402	5.44662	8.73283	6.4125	5.08977	7.39355	4.67568	5.1826	7.03646	5.92807	5.6039	6.4698	7.48676	5.8957	6.77273
H2AZ2	Q71UI9-2	7.67371	5.97344	5.41943	6.65462	6.20626	5.6033	8.95222	6.56641	7.53839	5.31509	4.73557	7.33295	4.95119	5.5395	6.34401	5.19391
DUSP12	V9GYV5	6.75668	6.08397	4.38595	5.39215	5.16628	7.32487	5.4009	6.87226	5.32208	6.15847	7.07505	5.82048	7.48262	7.49752	6.89821	8.36252
F3	P13726-2	5.67373	6.88621	5.80578	6.80637	6.03977	4.86007	6.90793	6.43419	6.12044	6.5196	5.80502	5.54628	6.74462	5.8718	6.56811	7.41008
GAP43	P17677-2	7.38986	6.82238	5.66376	6.06608	6.2734	4.96655	5.52861	6.92371	6.77472	6.33205	6.30092	6.68203	6.14445	6.07209	6.04511	6.01429
BAG6	F6WML8	5.08949	6.6624	6.90514	5.446	6.32065	6.53388	7.05019	6.33419	6.09871	6.35595	6.49023	8.50389	6.31724	4.64695	5.94228	5.30282
NETO2	Q8NC67	7.10535	7.39814	6.48774	6.98052	6.668	6.32843	6.78859	6.12325	7.00143	7.49957	6.13912	6.51932	4.32997	5.08889	4.90398	4.63771
CLCN3	P51790	6.91312	5.73114	5.6462	5.41293	6.30313	7.3065	6.0979	6.05481	6.63133	6.79277	6.14443	6.23802	6.2845	5.74669	5.84585	6.8507
HOMER2	Q9NSB8	6.78264	5.81616	5.22628	6.04756	6.93573	6.21268	6.401	6.8836	6.80985	5.95282	6.51927	6.30702	6.73013	6.43542	5.22332	5.71651
FRYL	O94915-2	4.99103	5.62046	4.52277	6.34428	6.5194	6.66082	6.71379	6.22815	6.77712	7.42663	6.40572	6.61466	6.67233	7.55603	5.42876	5.51804
RBM41	Q96IZ5	5.7698	6.54887	6.1492	5.53147	6.88796	6.00256	6.73385	5.65111	7.42143	5.58346	6.12846	6.75414	5.18658	6.3224	6.56379	6.76493
NOTCH3	Q9UM47	6.41937	5.80969	6.65172	6.41028	6.48845	6.15212	6.16195	6.52135	6.14755	6.6345	7.21707	6.25441	5.88719	5.42506	6.13461	5.68467
NBPF20	P0DPF2	4.96038	6.68412	6.09689	5.98191	6.50358	7.25394	5.76567	5.00209	4.98527	6.08126	7.18201	6.00679	6.74087	7.11241	6.61881	7.024
TNIK	Q9UKE5-4	6.74276	5.367	6.43426	6.45469	5.62579	5.99313	6.58739	6.54055	6.81095	5.72907	6.64052	6.66461	5.07932	6.4647	6.92	6.84526
NAA15	A0A0B4J1W3	6.61429	5.79234	8.01441	5.99839	5.71336	5.66832	5.81854	6.11458	7.06654	6.23274	6.42094	4.99627	6.70467	5.44408	5.7148	7.68573
GABARAP	O95166	6.24632	5.37978	6.83421	6.83099	6.24146	6.04666	7.14261	6.38521	7.58926	6.41676	7.69858	6.20937	5.6192	5.3972	4.85178	5.11061
DHX30	Q7L2E3	5.70192	6.54926	7.92139	5.5341	5.51237	5.56117	6.54672	6.28307	6.01297	7.42844	6.39157	7.04444	6.21935	5.28421	6.6722	5.33681
GTF2A1	P52655	5.91894	5.96014	5.18802	4.10638	6.13994	7.48422	5.75737	5.54584	5.82524	6.16172	6.35471	6.7027	7.53064	6.81513	7.3701	7.1389
DNAJB1	M0R128	5.73086	5.71671	6.69389	5.77542	5.42323	6.38636	6.40723	5.82297	6.14799	6.59218	5.87913	5.61609	6.06263	5.93802	7.26355	7.64373
RPS7	A0A2R8Y623	3.90403	4.0586	4.19705	14.2481	5.48691	10.989	4.33642	4.5539	4.95001	10.6488	6.27952	9.27713	4.80001	4.81114	4.16438	3.295
MCM3	Q7Z6P5	4.0536	5.96054	6.0981	6.92767	4.64253	7.33903	5.40895	5.37784	6.00272	6.72142	7.46879	5.95943	6.60372	7.56871	6.88712	6.97983
GK5	Q6ZS86	6.50693	8.10728	6.84109	6.62946	6.16503	6.51054	7.07643	5.79744	5.71574	5.58004	7.27219	6.1705	5.48077	5.75369	5.07128	5.32161
ANXA6	E5RK69	6.85387	6.57096	6.54944	5.7416	6.46103	6.22003	6.53867	6.55789	6.27174	6.87083	6.56459	6.6317	5.88327	5.9041	5.29108	6.08921
UBAP2L	F8W726	6.79017	6.70443	6.05746	6.21207	6.60244	6.53227	5.56613	6.58264	6.58094	6.32452	6.75145	7.20141	5.36418	5.82933	5.28828	5.61228
NIN	Q8N4C6-7	6.09486	6.78288	7.80494	5.22432	7.15665	6.5306	6.97698	6.44994	5.53638	7.07931	5.16116	7.97842	5.12965	6.64791	4.63615	4.80986
EZH1	Q92800-2	6.40667	7.31399	6.78443	9.3871	3.9579	6.2911	6.85056	5.33481	5.09887	7.42534	5.14893	7.10761	4.73114	4.77008	5.91537	7.47608
CNOT4	O95628-6	7.2673	6.61434	6.71676	5.90979	5.64131	7.41547	5.58514	6.24112	6.88243	7.82988	5.92409	6.7039	5.83621	5.87949	4.97847	4.57432
SHLD3	Q6ZNX1	5.92819	5.49861	5.23253	5.26751	5.45589	4.5955	5.60229	6.64214	7.74699	6.55658	5.96938	6.81671	5.84141	6.92944	8.78972	7.12708
RPS6KB2	Q9UBS0	5.54784	7.23454	7.2967	4.35584	6.09406	7.63567	4.98007	5.3925	7.46609	7.69666	5.69877	6.77113	4.8866	7.14912	6.50176	5.29265
TRAPPC14	Q8WVR3	6.32152	6.72365	6.4967	5.45711	6.13904	7.66528	6.68293	6.20138	6.11553	6.09569	5.39794	7.01355	6.01944	5.38097	6.0761	6.21318
MYH16	Q9H6N6	5.85845	6.74705	5.65451	6.71566	6.74818	8.17235	7.23667	6.61522	8.48777	7.06973	6.57279	4.59873	5.31477	4.78502	4.70052	4.72259
OXLD1	Q5BKU9	5.65113	4.97909	4.71533	5.51339	4.59724	4.48986	4.95672	4.28482	4.28195	5.05003	4.49865	4.41768	5.19394	13.7797	8.54595	15.0445
UROS	P10746	5.84934	5.81007	6.25536	4.32524	7.02574	6.48632	7.05963	7.55667	5.23124	7.66116	6.53144	6.51567	5.17308	6.28338	6.02508	6.21056
APBB2	Q92870-2	7.67641	8.51311	7.67174	6.52682	5.00583	6.91278	7.51484	5.18243	5.34739	4.48653	5.13303	4.66409	6.0684	6.11759	6.47307	6.70595
PPIP5K2	H0Y9S9	5.72461	6.15573	6.59195	5.39082	6.26061	7.03025	7.05279	7.40468	5.72701	6.45552	6.66073	6.12363	4.32148	6.785	6.38486	5.93035
ESRRB	O95718-1	6.39331	6.45314	7.09444	6.96798	6.02834	6.10202	6.60576	6.3754	6.6833	6.50748	7.04366	6.09309	5.33958	5.49608	5.70001	5.11641
DHRS4L1	P0CG22	5.44587	6.47531	5.85989	6.74278	6.89239	5.4123	5.98505	5.6668	6.42129	6.48939	5.91301	5.975	6.92871	6.13116	7.34515	6.31591
CTU1	Q7Z7A3	6.09571	7.12956	6.76219	6.21125	7.54189	8.94292	5.12759	7.00398	4.35074	5.5243	8.07397	6.09531	4.38038	6.24301	5.47681	5.04039
RNF138	Q8WVD3	5.48267	6.05029	5.46131	4.34226	6.54179	6.88208	5.09772	5.61808	6.12886	6.62772	8.41301	6.68526	7.50232	6.66112	5.66365	6.84186
S100A4	P26447	6.21577	4.7631	7.04079	5.19254	5.5324	6.26074	6.15621	6.21468	5.69754	6.76051	5.78918	7.48683	5.62315	8.06302	6.6416	6.56194
MED11	Q9P086	5.15454	4.614	4.44408	4.01755	5.89829	5.30979	5.38771	6.38904	5.14358	5.9574	6.00042	5.98052	4.42358	11.5073	8.16461	11.6076
RILP	Q96NA2	5.21163	4.53251	3.64719	4.49695	5.13793	5.15009	5.94608	5.65466	6.64982	5.82529	7.03221	8.99013	8.78156	7.36218	8.27653	7.30523
PRG4	Q92954	6.53435	6.06249	5.19373	6.64684	6.2972	5.42798	6.59497	5.97256	6.25661	6.20676	5.07187	6.38495	7.44825	6.81639	6.54962	6.53545
ZNF653	Q96CK0	6.0271	7.00349	6.18728	6.45931	6.39128	7.02211	6.02736	6.16187	5.92263	5.69126	6.60716	5.57846	6.22582	6.62505	6.24424	5.82557
PUM2	Q8TB72-3	6.84999	6.3118	6.91766	6.51016	6.29525	6.32087	7.2463	5.96504	5.44433	6.36213	5.80923	5.68151	6.22928	5.76475	6.11925	6.17246
CIAO2A	Q9H5X1	5.22225	4.95041	6.29551	5.74909	7.37196	6.87972	4.27699	6.22164	7.75676	6.35399	6.26293	6.48293	8.22692	5.18006	5.91053	6.85831
TEX9	Q8N6V9	6.83786	6.89292	6.42454	6.95996	6.88439	6.41303	6.66489	6.73914	6.75203	7.28016	7.27997	6.71612	4.24926	4.84036	4.261	4.80436
PPFIBP1	Q86W92-2	6.89759	5.46742	6.35703	6.21078	5.6693	7.18001	5.31795	6.62766	6.49738	5.95052	6.00599	6.26466	6.79255	6.23628	6.80744	5.71743
CRK	P46108-2	5.56817	5.1975	6.13067	5.2582	6.97217	5.31642	4.84674	8.05446	6.36622	6.84417	7.5255	6.82523	5.55855	6.39907	7.02902	6.1079
LENG1	Q96BZ8	6.5899	7.80247	7.06365	5.32264	5.92336	5.15322	5.74024	6.78897	5.45851	7.41422	7.37728	5.89222	5.31031	4.43781	6.97162	6.75358
PICALM	H0YEH1	6.02525	4.74279	6.30196	6.32861	7.49017	7.06816	6.50741	7.03561	5.84792	6.37249	7.73772	6.72951	6.31904	5.30601	4.93502	5.25234
LYPLA1	A0A087X1K9	4.93505	4.28681	4.81339	5.16218	5.36455	5.2333	5.41986	6.09948	7.45952	9.34375	4.98595	6.51584	6.98477	7.33277	7.72104	8.34174
C1orf43	Q9BWL3	5.61692	6.61468	6.92772	7.092	5.44877	6.62431	7.6713	6.4398	7.30136	7.19803	5.97883	5.81825	5.06993	5.19986	5.22687	5.77138
B4GALNT1	Q00973-3	6.40112	7.05374	7.98406	7.32523	6.54524	6.85447	7.37775	6.57026	6.37361	6.96911	6.82119	6.26121	4.10497	4.40095	4.28742	4.66966
MFSD1	Q9H3U5-6	6.11391	5.81928	4.29129	6.94335	6.35167	5.91122	6.3522	7.84206	6.77802	6.56156	6.10316	7.45217	6.67693	6.3681	5.49368	5.84141
RTL8C	A6ZKI3	5.89207	5.29804	6.58897	4.76707	6.06929	6.80211	6.25882	6.38709	5.30257	6.24909	6.59428	6.06503	6.52107	8.29867	5.99319	6.91262
CNK3/IPCEF1	G9CGD6-2	6.25742	6.42313	6.34826	6.40643	7.80865	6.33227	6.90455	6.04235	6.66672	5.95904	6.18218	6.53783	5.55901	5.55765	5.61143	5.40308
CEP85L	Q5SZL2-4	6.84323	6.48553	6.6047	5.8442	5.34326	7.45343	6.43503	6.21652	6.22644	7.79607	7.95066	5.70788	4.55471	5.60067	5.69621	5.24148
MPV17L2	Q567V2	6.0653	6.1183	6.62413	6.99554	6.39597	5.96468	7.24609	5.35278	6.11227	5.99292	5.96657	4.9464	6.9915	6.06169	6.36019	6.80566
ICOSLG	O75144-2	5.87411	6.40169	6.05226	6.40265	5.92212	6.48163	6.03904	5.94797	5.81118	6.49946	6.51263	6.69324	6.04572	6.63813	5.93294	6.74524
PROSER3	A0A2R8Y8D9	6.55976	5.00097	7.3839	5.42109	6.17311	8.37836	5.26954	8.1235	5.70146	5.33927	8.01883	7.67054	5.69599	4.84844	4.93979	5.47545
PROSER3	Q2NL68-2	5.97135	5.77164	4.95723	6.1072	7.86818	7.16996	6.55268	5.86827	7.76483	6.7215	7.01106	7.27899	4.14347	6.21987	4.90109	5.69269
PGGT1B	P53609	6.05585	5.61125	6.04781	6.29015	5.74618	7.29164	6.33491	6.17809	6.62874	6.71866	6.8668	7.75968	5.45564	5.8566	5.90404	5.25396
PPIA	P62937-2	5.37366	4.82512	6.13782	5.45964	5.46881	6.3125	4.95629	7.45234	7.23584	7.45737	6.7543	7.46914	5.62971	6.44163	6.58482	6.44102
IMPA2	O14732	6.0268	6.39852	6.33172	6.59791	5.97114	6.55492	6.56154	6.65461	6.65821	6.60432	6.29016	7.03621	5.70306	5.62034	5.26014	5.73038
TTLL4	Q14679	6.75876	7.04354	7.94807	6.74064	6.59785	6.37114	6.10922	6.53348	6.59278	6.97359	6.77407	7.81856	4.43072	4.37372	4.15609	4.38828
KDELR2	P33947	5.58651	6.07796	5.69293	6.01093	5.39823	5.89674	6.01461	6.24791	6.40011	6.67024	6.08996	6.31308	6.90926	6.40465	7.06121	7.22567
DDX46	A0A0C4DG89	5.43227	6.00594	5.14954	5.17083	6.54255	5.53543	5.46046	6.28177	7.0433	7.67196	7.95062	5.67392	5.97997	6.25318	6.54904	7.29923
BRD7	Q9NPI1	6.61768	6.39428	6.40983	6.31198	5.56518	6.15799	6.26881	5.73959	5.22216	5.85347	5.7284	6.67764	6.25057	7.06532	6.87318	6.86391
GPBP1L1	Q9HC44	4.86344	4.69917	5.79561	5.82479	6.3125	6.30125	6.33591	6.63475	6.15866	7.35975	6.89901	6.16758	7.23396	6.4836	7.20901	5.72103
PEG10	Q86TG7-2	6.23201	6.21711	5.96888	5.58881	6.41294	6.67304	6.81462	6.30736	6.58058	5.69005	6.44217	5.76326	5.7289	6.79728	6.84255	5.94045
JPT2	Q9H910-2	5.77294	6.36626	5.86012	5.07182	8.55054	6.54442	5.50472	6.56777	7.31367	6.61271	6.89546	6.63855	6.02435	5.59049	6.67014	4.01604
RAD50	A0A494C0Y7	6.76044	6.74396	5.58335	6.96487	6.5605	6.48676	6.34756	5.74646	5.29897	6.00406	5.4837	5.29074	6.93015	5.37404	6.93923	7.4852
CRADD	P78560	5.31701	5.13976	7.61017	5.23973	6.10935	6.38391	6.38174	7.74005	5.39649	7.27696	6.81198	7.41175	4.96995	6.05251	5.6995	6.45913
RADX	Q6NSI4	6.71641	5.6815	5.84242	5.44939	6.46306	6.5992	6.37742	7.30532	6.97326	5.61621	6.03061	6.06013	6.32405	6.08357	6.13309	6.34437
RBM38	Q9H0Z9	7.56552	5.78806	5.01311	5.79361	6.39236	5.08996	4.53703	7.12577	5.85864	6.0168	6.45668	7.88932	6.81423	6.76914	6.64032	6.24945
PLCH2	O75038-4	5.97809	3.79406	5.93062	5.23708	5.97926	3.89519	6.68308	5.9478	5.76516	5.27007	4.93295	6.56382	6.84225	7.89736	8.48935	10.7938
UNG	P13051-2	6.62514	5.90895	5.32206	6.72494	6.166	5.98351	6.9226	6.18989	6.62584	5.30673	6.82744	6.5858	6.54877	6.16991	6.11489	5.97753
LRIG1	Q96JA1	6.70287	6.17202	5.82972	6.53403	6.43274	5.63778	6.62108	6.13319	6.16095	5.89614	6.10156	6.00942	6.92756	5.92073	6.69399	6.22621
SKI	P12755	8.14105	6.90494	7.81643	9.92879	6.00591	7.09304	6.79816	6.13431	7.47788	6.42361	8.03065	7.09499	1.67168	3.33373	3.43707	3.70776
KLF16	Q9BXK1	6.34782	5.71151	6.3478	5.97996	6.14217	5.94564	6.08937	7.01866	6.3526	6.82775	6.28369	6.87827	6.99257	5.56568	5.42389	6.09261
RHNO1	Q9BSD3	6.08056	6.18323	6.36466	6.04954	5.75122	6.03864	7.13071	6.43695	5.86967	6.50776	6.87454	8.72782	5.62228	5.73546	4.51057	6.11639
FOXS1	O43638	5.55452	5.19574	5.58851	4.53458	5.37476	6.78583	6.21465	6.87649	6.25255	7.17592	5.37529	4.59956	7.20813	10.5276	7.49938	5.23647

ATP8A1	Q9Y2Q0	6.52454	6.31302	6.08739	6.44247	6.56894	5.92014	5.82936	5.93393	5.99583	6.61888	7.67567	7.07979	6.31089	5.28134	5.61482	5.80298
POM121C	A8CG34	6.75479	5.22904	6.05844	7.04322	6.2793	6.14891	6.56651	5.43627	6.40224	5.56842	6.36649	6.08677	6.8997	6.65232	5.93879	6.56878
CSNK1G3	Q9Y6M4-4	6.21455	6.12464	6.58644	6.9018	5.18773	6.00159	5.30681	7.25003	6.3701	6.41369	6.11808	6.31678	6.1937	7.10099	5.72046	6.19263
HLA-A	A0A140T9X5	6.8937	6.79447	5.59095	6.24684	6.5976	6.68195	6.92196	6.43191	6.60274	6.24721	6.47801	6.22645	6.20166	5.26092	5.53633	5.28731
YLPM1	P49750-3	6.02516	6.10551	6.73865	6.97576	5.68472	5.5376	6.08357	5.30682	5.69992	6.04305	6.25766	6.50653	6.88333	6.74915	6.41861	6.98396
DENND1B	Q6P3S1	8.2126	5.78321	6.84069	5.42949	5.60498	7.09244	6.47655	6.59925	5.80153	5.81529	6.35521	6.80031	4.92567	5.52373	6.69115	6.04891
ATP5F1D	P30049	5.48793	4.65516	4.7172	4.25024	7.23529	4.31529	5.28038	7.35268	4.27555	5.46792	6.51608	5.81756	6.07451	9.66579	8.37478	10.5136
SHFL	Q9NUL5	5.73457	7.41726	7.38206	6.52926	6.01491	7.36832	6.22921	7.59108	5.962	6.5617	5.15125	4.27801	5.47251	6.68725	6.28123	5.3394
DONSON	H7C006	6.08498	6.13084	7.08894	5.85164	6.55672	6.62294	6.47305	3.85863	5.91374	5.51241	6.23893	6.80032	6.06285	9.03647	5.83393	5.93362
GLRX	P35754	5.15905	5.3859	5.71465	5.03901	5.47522	4.69988	5.53956	5.3651	4.328	6.00715	4.91325	5.8473	4.68968	12.5804	7.09576	12.16
KIF2C	Q5JR91	6.20365	5.72483	5.72669	5.94557	6.00269	6.28412	6.28332	6.36988	7.01318	5.34993	6.03669	5.46792	7.40229	6.34627	6.76832	7.07465
WDR5B	Q86VZ2	6.45744	6.74618	5.97576	6.51707	6.486	6.00299	7.27145	6.74106	6.59048	6.5776	6.27522	5.97408	5.58881	5.76054	5.58027	5.45505
TTC33	Q6PID6	6.1253	5.76025	6.15605	6.38731	5.8237	6.0087	6.33734	6.35216	5.70472	6.18839	6.36604	5.9152	6.92232	6.6137	6.70453	6.63429
SAE1	M0R054	5.59021	5.98377	3.68819	5.24812	5.12101	5.68139	6.40132	6.14264	5.37579	4.74073	5.81194	6.67467	7.74225	8.74461	9.86939	7.18398
KIAA0753	Q2KHM9	6.42184	6.22511	6.49543	7.04557	5.53012	5.63518	6.34667	6.76652	6.06201	5.81446	6.41703	6.35537	5.96954	6.27677	5.81831	6.82006
HNRNPK	P61978-3	6.38584	6.37169	5.55852	6.381	7.54088	6.13567	6.42335	5.21441	6.63912	5.98146	6.40473	6.66818	6.8267	5.28192	6.24395	5.94258
PRKCSH	K7ELL7	7.02742	6.11815	5.74316	5.45301	6.92619	5.76676	6.15867	5.49801	6.17549	5.9206	6.91695	6.61651	6.38232	6.07016	6.24254	6.98405
PDGFD	Q9GZP0	6.15569	5.79201	6.59736	6.76777	6.79923	6.34992	6.33209	6.84005	6.94696	5.88272	6.1746	6.38802	6.60272	5.92597	5.29948	5.14541
ARMC10	Q8N2F6-2	9.51563	8.25103	8.03572	9.40909	6.02744	5.13106	5.01048	4.81151	9.66361	9.10757	8.01393	7.35267	2.02484	2.60379	2.77968	2.26194
ZFPM2	Q8WW38	6.87002	6.07101	5.81615	6.2194	6.60652	6.34585	6.37321	6.34649	7.3439	5.42242	5.50709	6.26863	6.16459	6.2251	6.71995	5.69965
MED9	Q9NWA0	6.56389	4.89272	4.32029	4.38215	4.57454	3.08766	4.18754	5.05627	4.48395	3.83175	3.22799	4.20056	4.11544	17.1179	9.48816	16.4692
LNX2	Q8N448	6.21147	6.19074	6.14921	6.63014	6.35793	6.60688	6.07739	6.37509	6.78663	6.17437	6.41467	6.86055	5.7616	5.64933	5.82427	5.92973
TGFB1I1	O43294	7.3891	6.08977	5.0914	4.82921	9.13396	5.90698	5.015	6.26559	8.41647	7.07177	6.41538	7.08373	7.42543	5.28239	3.90553	4.67827
IQCG	Q9H095	6.5758	7.71388	7.28027	6.88004	5.22567	6.67184	7.47456	7.4373	6.84621	7.72648	8.5806	8.90302	3.36601	4.07198	2.46836	2.77796
API5	Q9BZZ5-2	6.6616	6.28254	4.32747	6.39881	5.08058	6.32323	4.43575	5.59418	6.32005	5.62969	7.54386	8.33594	7.31785	7.35415	6.15276	5.84154
AP2M1	C9JTK4	5.62715	7.14186	5.44699	5.51416	5.40999	6.48506	6.59575	6.19102	7.20164	6.78002	5.72983	5.98235	5.6063	6.29079	6.58086	7.41623
GPSM1	Q86YR5	6.71477	6.32771	6.58154	6.19481	5.99482	6.61871	5.8402	7.06895	6.13742	6.44913	7.19342	6.77011	5.81884	5.54545	5.5156	5.22853
ZC3HAV1	Q7Z2W4-3	7.07276	5.97019	5.74481	5.5193	7.43936	5.68562	4.77498	7.49488	6.49507	5.79982	7.8069	7.34645	6.05809	5.79198	5.85275	5.14704
SUPT5H	O00267-2	5.73501	5.87657	5.91072	6.08389	4.18756	5.65101	5.5066	6.84433	10.0207	6.00633	6.52872	6.31599	5.43808	6.81434	6.16276	6.9174
RPL23A	H7BY10	6.17176	4.50884	6.34571	4.50414	5.51423	6.65872	5.58125	5.51889	6.30175	6.42371	7.40519	7.63061	7.40117	6.70167	6.44547	6.88689
MEF2A	Q02078	6.12209	5.86275	5.64316	6.51928	6.17663	6.2725	6.32312	6.12704	5.73732	6.62055	6.43216	5.92297	6.12904	7.30781	6.38147	6.42211
MEF2C	Q06413-5	5.46849	5.89879	6.24619	5.41692	5.31174	5.9563	6.50929	6.67085	6.20919	6.23952	6.54808	6.67267	6.55114	7.66751	5.74241	6.8909
TFPT	P0C1Z6	6.14698	5.76544	7.98616	5.82527	5.74285	8.17566	6.91643	5.70087	8.58857	5.59726	6.04235	5.45568	5.73725	4.9721	4.76999	5.57714
CARHSP1	H3BNU9	9.18088	6.83728	9.02783	6.17483	5.28841	6.08331	4.42864	5.35407	5.08845	4.40811	5.49454	5.5736	8.93939	7.93862	5.28293	4.89912
CCNF	P41002	8.44924	7.25399	9.18267	6.56565	6.55648	5.03364	8.1151	7.30954	6.41434	5.94264	11.9973	7.48038	1.11439	3.44043	2.20389	2.93831
DNAH9	Q9NYC9	5.38757	6.40747	6.33769	5.10674	6.87095	6.39653	6.79665	6.81113	6.24311	6.63911	5.75143	6.41546	6.39957	6.2988	5.5798	6.55798
ZFYVE19	Q96K21-3	5.7497	5.65803	7.66229	6.23039	5.94959	7.06097	4.91109	5.6202	6.39923	7.49285	7.23431	6.99399	6.22492	5.61047	6.09994	5.10202
SMIM26	A0A096LP01	5.89096	6.49824	6.23736	7.47644	6.29477	6.5901	6.99305	6.03369	6.09114	6.5937	6.07599	6.75842	5.05188	5.92687	5.21416	6.32164
RCL1	Q5VZU2	6.49026	6.3503	6.74481	7.36026	5.7023	5.54964	6.86508	5.81637	7.52055	7.27526	4.68298	5.43115	6.32694	4.08967	7.30303	6.49141
PLPP6	Q8IY26	6.67447	6.3898	6.63701	7.28182	5.84598	5.54094	6.31073	5.96763	4.91807	6.50789	6.5716	6.27954	6.18599	6.35199	6.31483	6.22169
C1orf159	Q96HA4	6.55238	6.98508	6.31094	7.08909	6.1575	5.90933	6.58117	6.23981	6.64181	6.07363	6.02948	6.46974	5.6091	5.27356	6.08905	5.99033
MPDZ	H0YGQ3	6.38154	6.049	6.39179	6.7255	6.48179	6.25973	6.24877	6.56999	6.47327	6.69123	5.71434	6.54321	5.53194	6.05625	5.69584	6.1858
RIOX2	Q8IUF8-4	6.01643	6.65454	6.33839	6.67032	6.80378	6.45109	5.55068	6.38133	6.6198	6.39065	4.65562	7.40943	6.49738	5.77555	6.2716	5.51339
WDR25	Q64LD2	7.34007	7.07289	6.44878	5.94979	6.76005	5.59182	6.48357	5.59619	6.0727	6.23559	4.9542	5.62848	6.8048	6.31551	6.18917	6.55638
P4HA1	P13674	5.83324	8.37048	6.03974	5.62901	6.32689	5.97951	6.58308	6.33624	5.62817	5.8329	6.53557	6.61817	6.41085	6.36452	5.37861	6.13303
CBARP	Q8N350	6.33234	6.73264	6.56953	6.65777	6.07377	5.29866	6.77306	5.5715	6.08249	5.34117	5.73373	6.33218	7.08458	6.7882	6.2614	6.36698
PUDP	Q08623-4	5.43053	4.39869	6.07845	4.31574	6.02177	6.11109	5.49246	7.31747	4.98698	7.01632	6.56798	6.6602	5.90033	7.80155	8.01601	7.88441
CLINT1	Q14677	5.95	5.87021	6.58695	6.86193	7.16682	6.187	4.97796	6.15161	6.42054	6.91708	7.03167	5.68995	5.53818	6.36475	6.64987	5.63548
CBX2	Q14781	6.88132	6.66154	6.72776	7.30127	6.60776	6.23126	6.56216	5.45528	6.19129	6.34721	6.08277	5.92668	5.56286	5.89283	5.59279	5.97523
TTLL1	O95922	7.02874	7.28587	6.28284	6.22953	5.78982	6.65903	5.64583	6.21847	5.80389	6.59514	6.36195	7.24216	5.46911	5.11061	5.95419	6.3228
PNMA8A	Q86V59	6.33946	5.95778	6.4313	6.98577	5.76268	5.19268	6.99042	5.66397	7.3734	6.43571	6.17364	6.92949	5.22192	6.08433	6.37268	6.08476
MLLT6	P55198	7.51812	6.20255	6.53668	6.52458	5.54568	6.45911	5.79325	6.8188	7.19365	5.25797	6.0161	5.32213	6.0095	6.34564	6.60009	5.85615
TKT	A0A0B4J1R6	5.50571	5.4305	6.51624	5.22134	5.36421	6.38429	4.71192	5.23345	7.0164	5.66489	6.0092	7.26831	6.84677	6.03424	8.96747	7.82505
UTP23	Q9BRU9	6.55131	7.26414	4.86406	7.36567	6.34819	7.04011	6.18144	7.46161	5.46891	5.46608	6.17816	5.74514	5.47897	5.90553	6.34495	6.33573
FLYWCH1	Q4VC44-3	7.27652	7.19743	6.84815	8.06969	5.83051	6.35755	6.33419	5.89453	6.53259	7.6406	6.60808	5.95695	4.62106	4.8914	4.55444	5.38631
WSB1	Q9Y6I7	9.37438	7.3053	8.41205	7.21533	6.08444	7.74692	6.93037	4.78975	9.03268	5.76501	6.69559	5.22863	3.42703	4.24193	4.60481	3.1458
NFATC1	O95644	5.44674	5.38664	6.78063	5.06583	6.50971	7.02203	7.04779	6.20036	5.49758	6.46324	6.94022	8.05864	6.30965	5.95451	6.09457	5.22186
KLHL8	Q9P2G9	6.53891	6.21623	7.09149	6.93499	6.02499	7.01053	5.97842	6.56853	6.65531	6.60226	6.90133	6.68901	4.64347	5.11841	5.48702	5.53909
POMP	Q9Y244	5.05126	7.14868	6.46815	7.08025	5.96257	6.64703	8.40785	6.06515	4.48504	6.37676	6.23354	7.95559	5.00522	6.58947	5.71927	4.80417
TMBIM1	Q969X1	6.82327	6.46554	6.67125	6.10577	6.16304	5.5686	6.76346	6.06597	5.97299	6.21094	6.37151	6.56906	6.08701	5.85306	6.51784	5.7907
ZNF652	Q9Y2D9	5.65398	7.16154	5.3993	6.25385	6.7056	6.63085	6.56495	6.957	5.77065	5.88838	6.77769	5.01665	5.3818	7.05137	6.80755	5.97886
CA8	P35219	7.13493	4.64812	7.63321	5.63004	5.24951	7.05952	6.33134	6.91024	5.44629	7.33059	6.54369	5.71213	5.06037	7.78892	5.29195	6.22913
LARP1	H0YC73	4.90863	5.76646	6.91836	5.72011	6.12479	7.13756	6.47619	5.33854	7.04756	7.03691	5.51084	5.84849	5.38424	6.93282	7.94275	5.90574
ZSWIM1	Q9BR11	6.54543	6.34955	6.986	5.33841	5.94156	6.83778	6.27329	6.42075	6.54517	7.36781	7.06846	7.06136	5.00632	5.21097	5.71312	5.33403
CPPED1	Q9BRF8	6.12658	6.20814	10.1224	7.81296	5.16216	7.37055	6.54917	6.25899	8.62558	4.88499	4.55929	5.43051	4.5297	3.70231	6.02887	6.62778
CRY1	Q16526	6.62715	6.78712	7.2799	6.87647	6.76882	5.11199	6.20984	7.27461	5.6337	6.07518	7.61397	8.00704	5.25267	5.23005	4.48377	4.76771
FN3K	Q9H479	6.4476	6.35955	7.01805	4.29075	6.48731	6.0209	6.34116	6.68306	6.60125	6.28321	7.90708	6.59904	6.18031	5.58161	5.40662	5.79249
ELOVL2	Q9NXB9	5.81137	5.7432	5.88873	7.11295	5.26805	5.33415	6.53322	6.37248	5.62814	7.1092	5.91375	6.89347	6.39563	6.38322	6.98959	6.62286
MAP3K3	Q99759-2	6.77384	7.35972	5.86091	8.73825	4.34837	8.10937	5.50157	4.67543	7.2364	6.48252	5.30854	5.59235	5.72186	7.47252	5.86278	4.95558
MIGA2	Q7L4E1	4.48465	7.43355	7.13858	6.74019	4.73739	5.66915	4.57033	5.67921	6.73035	6.55657	7.33893	7.01357	6.51427	5.49913	7.20859	6.68554
SF1	H7C561	6.52879	4.97504	6.31979	7.01933	6.71321	5.63187	5.77957	7.02212	5.88735	6.86203	6.6332	6.30651	5.30356	6.59319	6.85371	5.57072
FAM221A	A4D161	6.93968	5.95219	7.56331	6.07623	6.47009	6.93072	5.96862	6.77788	5.98491	6.67607	6.58636	6.91844	5.10442	5.60027	5.64825	4.80257
SLC52A2	Q9HAB3	6.36585	8.26915	6.13623	6.96918	5.77392	5.29327	6.58752	4.28129	7.17083	7.42066	5.35657	5.41043	7.27912	5.9245	5.06201	6.69948
EIF4H	Q15056-2	7.18689	6.78505	8.26378	7.38234	7.95921	9.24472	5.71098	6.72256	6.54509	7.32035	8.23178	6.52411	2.9599	3.51328	2.72416	2.82578
BAG4	O95429	5.69937	6.25718	6.19581	4.10345	7.93496	5.55907	4.54217	6.54243	6.58916	7.15302	7.03903	7.4959	6.88521	6.35162	5.17177	6.47985
MT2A	P02795	6.83789	5.98892	7.25934	6.29672	7.16493	7.042	5.96231	6.92333	7.12664	7.99114	7.61144	7.0828	4.92224	3.94003	3.81712	4.03318
TSPAN9	O75954	5.22995	6.9582	6.294	7.05563	4.72345	6.00291	5.57935	5.31094	5.77326	6.03088	5.9711	8.02791	7.73854	3.74768	8.86273	6.69347
SFT2D1	Q8WV19	5.70145	6.85351	7.67983	7.02913	6.70713	7.36759	6.22136	5.04311	5.74655	6.67382	7.31153	7.45103	5.02378	5.35047	5.17452	4.6652
KLF12	Q9Y4X4	6.13249	5.81968	5.69712	6.54985	4.98379	6.69888	6.01424	4.72708	6.29914	6.67326	7.65376	6.46163	6.92419	7.02872	6.75299	5.58316
RTN3	O95197-7	5.969	7.22544	7.49414	5.70574	7.00453	6.30051	6.83593	6.41918	5.23443	5.83934	5.76725	5.59333	6.08996	5.96716	5.86236	6.69169
HNRNPA1	F8W646	5.74465	5.96548	6.1697	5.87554	8.80158	4.84866	5.15046	6.35335	7.24258	4.95726	5.74562	7.4324	5.73065	4.69833	7.45024	6.83349
HNRNPA3	A0A7I2V2R3	6.18295	7.66467	6.10342	7.50483	5.73742	8.55033	4.9255	4.62689	5.33163	5.90272	6.20008	7.06252	4.73083	6.29025	7.11422	6.07174
TRIM33	H0Y612	7.66738	6.36415	5.9362	5.09674	5.78507	5.96281	5.6975	5.95306	6.72218	7.12352	6.03101	7.79271	5.15068	5.49188	6.12348	7.10162
LTBP1	Q14766-4	7.74983	7.983	4.95432	5.50587	5.13244	6.29588	7.80644	5.73536	5.37098	6.03629	5.4272	7.2995	4.98404	7.2394	4.68128	7.79817
ZNF737	O75373	6.54562	7.10052	6.91788	7.69446	5.9341	6.1587	5.50563	5.71366	7.50721	6.17224	7.8187	5.95688	5.18099	6.77239	4.88944	4.13159
SRD5A3	Q9H8P0	6.04657	5.28019	4.98734	6.44974	6.74892	5.42133	7.1574	6.29578	6.16113	5.71777	5.01509	6.60565	7.55536	6.69036	7.07616	6.7912
TDP2	O95551-2	5.11601	5.96703	9.14978	7.32274	5.79037	6.50132	5.34246	5.57942	7.02407	5.99707	6.31544	5.64209	5.53834	5.98805	6.20987	6.51591
PPP2CB	E5RHC1	9.44335	5.98583	8.47033	6.75035	5.62981	5.13421	5.41441	4.66035	4.93859	3.87229	5.43112	5.25149	8.14017	8.03286	6.46069	6.38417
TSPAN5	P62079	5.64211	6.80008	6.13993	6.49958	6.53583	6.40222	6.63078	6.72894	6.15693	6.80066	6.35033	6.14364	5.62821	5.85622	5.75775	5.9268
ZFP36	P26651	5.80805	5.98592	6.7938	5.48638	6.08965	7.46375	6.32895	6.92079	6.44495	6.04986	6.59527	6.89477	5.752	5.97466	5.96553	5.4457
SSNA1	O43805	6.59294	4.69441	5.93309	4.28842	5.14535	3.86958	4.53171	6.11897	3.85853	5.46475	5.54988	5.30584	4.53972	12.6169	9.5398	11.9501
CALR	K7EJB9	10.3219	6.89518	6.03079	7.27787	7.0038	5.85192	3.9243	3.94085	4.79496	4.43822	4.81838	5.21174	10.6631	6.53666	6.55043	5.73981
DNAAF1	Q8NEP3	6.17693	6.53137	6.30027	5.56015	6.76497	5.24842	6.69022	5.8109	6.34331	6.01655	4.87111	7.84236	7.27841	6.48842	4.92982	7.14678
CORO7	P57737	4.93833	5.1028	5.61451	6.26432	6.5746	8.52354	4.79107	6.76984	6.29298	6.50816	7.50191	4.66602	4.71729	7.30715	5.68187	8.7456
RPP21	A0A096LP39	5.51678	6.34705	6.4155	6.50267	5.64781	6.19239	6.64993	5.62672	6.22558	7.14469	6.80528	5.75723	6.24156	6.74678	6.33092	5.84911
ZNF319	Q9P2F9	6.25962	6.74801	5.92523	7.38759	6.33595	6.1488	6.56165	5.6824	5.86773	6.99203	7.23967	6.56421	5.6432	4.98	5.14579	6.51812
COP1	Q8NHY2	5.13281	6.46875	5.19604	6.96165	7.16958	5.66685	7.23062	6.20376	6.17962	6.26674	5.91861	5.99721	7.13713	5.51663	6.45435	6.49964
PRKAA2	P54646	5.80662	5.07367	5.91473	5.81821	5.71408	6.7879	6.06619	5.64311	6.60899	5.38075	6.75347	6.26576	7.01166	7.21091	6.8391	7.10485
APOD	P05090	1.14005	33.2493	0.734335	10.8843	1.3147	1.96855	2.90334	3.67176	4.35899	3.52948	2.27193	2.47371	8.45605	3.91017	3.55157	15.5818
NUDT16	Q96DE0-4	6.19866	5.46618	5.82472	4.94943	6.34188	7.89783	6.02819	6.84851	6.40387	6.46526	7.25046	6.9533	4.85214	6.76108	6.61778	5.14071
TPRA1	Q86W33	6.14147	7.08835	5.79278	5.20929	6.10371	6.39702	5.62375	5.6774	6.02376	5.73834	6.20484	6.2579	6.39273	7.29604	6.42485	7.62777
FRMD4B	C9JAW0	5.32823	7.72124	6.33323	5.65831	6.23072	6.63005	6.99686	5.73181	4.26835	7.51025	7.9789	5.4255	4.58037	6.46009	5.95371	7.19238
GLI4	P10075	6.13755	7.34165	6.04251	6.85218	6.60459	5.29077	6.41385	4.80398	6.53933	7.42407	7.12387	7.22323	6.08029	4.55673	5.85615	5.70925
KCTD5	Q9NXV2	6.38146	6.29218	6.83773	7.01253	6.04862	6.70047	6.24041	6.42103	6.66913	6.40964	7.00029	6.86253	5.51712	5.3017	4.90972	5.39543
FBLN2	P98095-2	6.94877	6.12792	5.01979	6.53927	6.67434	6.1204	6.04429	6.49044	6.78159	6.9393	6.92355	7.86459	5.25256	5.25271	4.71304	6.30745

TMEM50A	O95807	6.11654	6.92541	6.85044	6.52435	6.60004	6.67095	7.42569	6.56072	6.05513	5.90161	6.4962	6.2527	5.60468	5.2515	4.66389	6.10015
DCAF10	Q5QP82	5.64618	6.26627	6.41329	5.72475	6.08448	6.05297	6.38335	6.1512	6.46968	6.03966	7.4487	7.5706	5.13858	6.03291	5.63097	6.94641
RAPGEF2	Q9Y4G8	7.20715	7	6.47141	6.07983	5.66975	5.82767	6.82105	7.51638	6.73199	7.46714	6.41917	4.72466	5.22541	6.57712	6.01084	4.25042
NAT9	Q9BTE0	6.05848	6.48649	6.29293	5.50748	7.07808	5.36048	6.68822	7.59718	6.29223	6.20487	5.38433	6.795	5.98321	6.06055	5.96188	6.24861
SLC30A4	O14863	5.88898	7.41926	6.41161	5.47898	5.90683	5.36431	7.92445	5.2342	7.78298	6.18387	4.82859	7.80412	7.35936	6.01425	4.99109	5.4071
RNF13	O43567	6.18382	6.59197	6.17547	6.82078	6.28496	6.47691	6.03518	6.13477	6.29437	6.29852	6.13866	6.31857	5.66938	5.88552	6.02881	6.66232
GCAT	O75600-2	7.24509	7.3568	7.70365	5.97177	6.79658	4.382	6.19737	5.68828	4.62957	5.78058	7.35718	5.84535	5.59633	6.52873	6.67813	6.24259
ATG14	Q6ZNE5	5.96606	7.62171	7.63362	5.8155	5.43236	5.03432	6.9936	7.07858	5.48173	6.30321	6.61227	5.62529	4.76247	8.01464	5.01865	6.606
FAM72D	Q6L9T8	6.44769	5.61863	6.85785	5.61856	6.42096	6.70385	7.01695	8.40115	7.66758	7.11003	6.59366	7.13751	5.32537	4.66491	3.6636	4.75168
EIF3G	K7ER90	6.15772	6.92322	5.23887	6.92872	6.771	5.84594	7.40848	6.46244	5.57767	4.49314	7.18411	6.15651	6.73652	5.58642	6.55336	5.97589
HSCB	Q8IWL3	6.98056	5.46364	6.33075	5.32498	6.59533	5.46459	5.3945	6.20838	5.12466	5.90196	5.84169	6.52825	6.46785	7.78815	7.5586	7.02611
HNRNPD	Q14103-3	7.15069	5.28386	7.38218	6.51495	7.35627	7.13699	5.6041	5.63105	5.66771	5.64622	7.35047	6.29809	5.68274	6.74935	4.5161	6.02922
COX17	Q14061	6.4676	6.35244	4.6804	4.86286	4.51098	3.5744	4.21196	5.41429	3.28023	5.27377	4.28975	4.38865	4.63666	13.2245	9.67548	15.156
ZC3H13	Q5T200-2	7.57451	6.87348	5.91935	5.80352	5.66968	5.41703	6.09786	6.02554	6.41456	6.87189	5.8618	7.03966	5.94623	5.37167	6.40213	6.71108
SRSF12	Q8WXF0	7.03102	8.45446	5.6696	5.64751	6.05618	3.5302	4.80196	6.86526	6.49764	8.54137	5.97089	7.2289	7.36556	5.41965	6.06063	4.85917
SFXN4	Q6P4A7-3	5.96662	5.50472	6.21641	6.42576	4.96618	6.45799	5.96301	7.63711	6.70446	6.39593	6.50562	7.08696	5.44481	5.61891	6.67158	6.43393
DTNBP1	Q96EV8	5.72652	5.5249	5.82579	6.53198	6.28525	7.18425	6.77407	7.52267	6.31574	7.04821	6.95923	7.51968	4.67541	5.87763	4.83615	5.39253
RASSF4	Q9H2L5-2	6.27794	5.9257	7.14048	6.16191	6.31678	6.73782	6.26591	6.15321	7.05477	6.64203	6.92266	6.42718	5.89701	5.05107	5.67341	5.35212
RASSF2	P50749	6.80936	6.46388	6.54717	6.13889	6.11118	7.08462	5.89308	6.81568	6.45332	6.11188	5.87601	6.29685	6.36866	5.63088	5.81641	5.58215
MDK	P21741	7.34246	7.19379	7.13416	7.3725	6.01431	6.4033	7.71372	6.789	6.16063	5.85893	5.50004	5.48545	4.78591	5.75682	4.96596	5.52304
ERCC8	Q13216	5.49958	5.87592	5.75746	5.43716	6.31786	5.55898	5.50647	5.58666	7.47656	4.87518	5.35966	6.07032	7.40854	7.7519	8.15541	7.36232
PFKFB4	Q16877	7.11454	7.1123	5.78777	5.29295	7.05646	7.0183	4.95471	5.89368	5.90109	6.97134	5.70543	6.02311	5.44807	8.24225	6.18884	5.28917
SPSB2	Q99619-2	7.05136	6.0726	4.72906	5.01285	7.25274	7.12817	5.53326	5.39801	6.50265	6.69226	6.90052	6.30042	7.18495	6.95824	4.99756	6.28534
BLOC1S2	Q6QNY1	4.46229	5.30496	7.91496	5.94033	7.00543	5.11609	5.36455	6.13552	5.52999	6.46146	1.85794	5.46034	6.48601	9.58005	7.67224	9.70783
EVC2	Q86UK5	6.34771	7.29847	6.19193	7.46258	5.90059	6.48998	6.95303	7.06342	7.60137	7.16254	6.46609	5.72819	4.65611	5.51451	4.25593	4.90754
ST14	Q9Y5Y6	6.32203	5.96812	5.86452	5.66729	5.73326	6.8594	6.12045	7.30178	7.43557	6.42424	6.40278	7.57326	6.0555	6.55499	4.90892	4.80787
ADGRL3	Q9HAR2-4	7.40401	7.15654	7.16532	7.1891	5.82934	6.02577	7.05793	7.9576	8.59655	7.75353	6.683	8.14761	3.13884	3.64791	3.0866	3.16034
MTHFR	P42898-2	6.05265	7.08582	6.7234	5.53461	5.39459	6.59901	6.43639	6.36249	6.48697	6.55919	6.19901	7.62943	5.62076	5.45135	6.14977	5.71457
PHF20L1	A8MXR8	7.2045	7.10323	5.94226	8.67814	5.75745	6.2266	6.23516	5.72219	5.85307	5.91555	6.34847	6.4624	5.2883	6.09527	5.47231	5.69508
SLC9B2	Q86UD5	5.22365	5.54441	7.24291	6.59988	5.26529	6.68704	7.65535	6.89684	5.49538	5.83757	5.62086	7.22501	5.38366	5.88564	5.76099	7.67553
GALNT3	Q14435	6.49137	5.99535	6.21336	6.39901	7.44043	4.6035	6.29708	5.26978	6.5402	6.47271	5.39205	6.45538	5.37556	5.7768	7.713	7.5644
RNF4	P78317	6.50238	7.36538	7.70536	7.95797	5.57123	6.4289	6.12154	8.40234	5.82654	5.51225	8.54517	4.35081	4.75314	5.13836	4.94711	4.87151
RBBP7	Q5JP01	6.39612	5.39918	3.58682	4.96969	6.52662	5.00842	6.75096	7.97925	6.20093	7.37398	6.23122	7.44135	6.02874	9.3391	4.82592	5.94168
RGL2	O15211	6.25096	5.69872	7.01299	5.47134	5.74283	6.67904	5.67594	6.58063	5.86803	6.99878	6.42924	6.79223	5.85038	6.71343	5.57566	6.65981
NPM1	E5RGW4	7.71846	7.30107	4.38738	7.54769	6.69421	5.16966	6.26177	5.25076	7.61008	3.9261	5.91004	6.5332	6.51679	6.64586	6.25154	6.27537
KCTD3	Q9Y597	5.79421	6.13102	6.30557	6.03986	6.22435	6.61949	6.04523	6.02891	5.8155	6.41565	5.87612	6.05238	7.40169	6.03754	6.67036	6.54211
OPA3	Q9H6K4	5.93111	6.10048	5.66422	6.74989	5.75453	6.27662	6.12465	5.94878	6.02967	5.91983	5.61553	6.4737	6.80416	6.55026	7.03371	7.02286
C2orf42	Q9NWW7	5.96565	7.74743	6.20546	6.13372	6.42663	6.4925	5.46524	6.12412	6.06491	5.55053	6.36496	5.9983	6.43256	6.23819	6.30497	6.48482
CCDC80	Q76M96-2	6.78884	6.34248	4.381	7.96745	4.71514	6.31615	8.00368	5.64409	7.38036	5.9679	4.87342	7.03765	6.43718	5.48158	5.94877	6.71431
TCTN2	Q96GX1	6.54833	6.74273	6.25663	6.63774	5.99706	6.15852	6.15205	6.21083	6.30798	6.65502	6.1795	6.70273	6.12175	5.83282	5.42349	6.0728
OTULINL	Q9NUU6	6.72131	5.97133	6.0893	6.72398	6.09996	5.80056	6.26901	5.11058	5.70813	6.06318	6.52196	5.88655	6.39216	7.06461	6.64592	6.93149
NPHP4	O75161	6.29936	7.44642	7.37638	5.48481	5.9109	6.6653	5.97203	5.06206	7.28264	7.37334	6.19112	7.45681	6.14187	5.58895	4.40472	5.34331
FIZ1	Q96SL8	5.46413	5.8279	6.02738	6.41388	5.98742	5.77089	6.06575	6.28988	6.32654	5.97643	6.35636	6.08548	6.72334	6.82721	7.19407	6.66335
OGFOD2	Q6N063	6.86913	5.83994	6.71329	6.45279	7.02926	5.86049	6.38397	6.69733	6.4263	6.0857	6.4393	5.90753	6.56606	5.74765	5.57053	5.41074
KCTD1	Q719H9	6.27961	5.80623	7.40589	2.63674	8.45142	8.01283	5.65542	4.16756	5.88541	5.11344	7.72648	8.56796	5.92788	6.20922	5.24469	6.90922
COL22A1	Q8NFW1	6.26617	6.19507	6.36732	5.97209	6.56728	7.18953	6.46421	6.3606	5.70388	6.41509	6.94664	6.46268	5.60837	6.37681	5.7572	5.34706
SHC1	P29353-3	7.09647	6.80096	7.33907	6.65393	6.32472	5.28735	6.66104	6.71159	6.46903	6.089	6.41781	6.56196	4.52326	5.43244	5.66681	5.96455
CLIP3	Q96DZ5	6.29653	8.69043	6.81879	6.35679	7.4045	7.32674	6.15672	6.00196	7.83545	7.93876	6.8813	7.69037	3.19546	4.49678	2.92162	3.9878
SLC39A7	Q92504	6.49106	5.63139	5.51317	6.30322	6.01387	5.67746	7.78063	5.93121	6.33739	5.98083	6.05888	6.27751	6.70982	6.11314	6.36009	6.82034
TRIM21	P19474	6.05701	6.67226	7.73434	5.04966	5.65815	7.68563	6.63966	5.62607	7.11241	6.32771	4.98555	6.08391	6.29879	7.52109	3.68254	6.8652
TPGS1	Q6ZTW0	6.84651	6.36569	7.62796	6.4418	6.25394	6.66536	5.86315	7.61169	6.62352	7.47382	6.96618	7.84342	4.00481	4.69542	3.93417	4.78255
NOP2	P46087-2	5.44525	5.45558	5.77712	7.3163	5.18766	5.9694	7.21098	5.13226	6.06361	5.87995	6.58111	7.14461	6.64683	6.74977	5.99771	7.44186
DYNLL2	Q96FJ2	6.03803	5.68444	6.07874	4.94328	6.61826	6.42265	5.99825	6.53779	5.52857	6.11032	5.4541	5.36265	6.60033	7.91717	7.4453	7.26011
COX18	Q8N8Q8	6.6791	5.94174	7.60368	7.81617	5.44197	5.72438	6.00879	6.39825	5.4326	3.96467	5.46528	4.87856	8.71264	8.07096	7.10309	4.75813
ZNF816	Q0VGE8	6.11277	7.16439	6.29335	7.77107	5.82746	5.26015	6.0986	6.39188	6.90918	6.66802	6.53599	6.22054	5.59054	5.84603	5.9996	5.31042
GSDMD	P57764	6.83927	6.23183	6.5108	5.7241	6.41467	6.93584	5.70606	6.11053	6.56197	6.07893	6.25822	6.39774	5.79135	6.84987	5.66301	5.9258
HNF4G	Q14541-2	6.37889	5.91038	6.62031	6.37533	6.25332	5.62929	6.61182	6.17986	6.51675	6.34202	6.12975	6.42469	5.87583	5.18661	6.32965	7.23552
TAF2	H0YEX2	5.41527	6.34846	7.03198	6.91436	5.06704	5.20336	4.00933	7.38335	4.15017	5.57349	6.68545	5.85364	7.44838	9.84203	5.12491	7.94878
ZBTB18	Q99592-2	6.57086	6.97447	7.45167	7.24172	6.59516	6.43781	6.40759	6.10111	5.54269	5.97792	6.34575	6.61978	5.3603	5.22377	5.55504	5.59436
ABLIM1	O14639	6.82488	5.09201	6.44992	6.2933	5.32613	6.80536	6.13871	6.06246	7.02232	6.20238	5.37546	5.86391	6.70962	7.45508	5.50477	6.8737
B4GALT5	O43286	7.23858	7.43665	6.43607	7.90985	7.20281	5.58893	5.81436	5.51392	7.17411	8.07908	8.11462	6.84209	4.3447	3.90021	3.87733	4.52668
B4GALT6	Q9UBX8	7.6314	6.25123	5.73804	6.31595	4.98758	5.1994	6.33071	7.15561	6.41297	5.31345	7.49069	7.48784	7.19679	5.86558	6.27404	4.34873
UCK1	Q9HA47-4	6.02581	4.66488	7.88724	5.4766	6.16849	6.05427	6.06924	6.02946	6.69734	6.62288	6.85778	7.09039	6.36519	5.23912	6.29492	6.45638
TCEAL1	Q15170-2	6.49437	5.9483	5.97574	5.29334	6.97921	6.60555	5.84424	6.0896	5.61981	6.76062	7.01242	7.05705	5.71206	6.30695	5.80822	6.49255
CEBPG	P53567	5.95378	6.79681	6.76549	7.04009	7.40348	6.3763	5.79015	8.10653	7.38957	4.85224	5.41848	4.66472	5.60163	4.28013	5.51692	8.0437
POTEH	Q6S545	6.07002	5.29985	6.14269	5.95841	5.93978	6.25789	6.23968	6.15773	6.26884	6.83737	6.63923	6.5384	6.37615	6.55085	6.4496	6.27349
FEM1A	Q9BSK4	6.16161	6.63785	7.31423	6.67227	6.11903	6.00717	5.64958	6.98269	7.44374	5.55065	6.60239	7.40398	4.93185	6.00534	5.26426	5.25335
FEM1C	Q96JP0	6.70194	6.75268	8.04556	6.67212	6.0489	6.47385	6.03935	6.13614	7.02526	5.98794	6.44967	7.11872	4.7189	4.88599	5.87815	5.06484
PACS2	Q86VP3-2	6.31479	5.42354	5.47193	6.11949	6.72434	6.48478	7.33724	5.56087	6.74731	8.9845	6.47482	6.46953	5.78583	4.47635	5.19708	6.42758
ARL8B	Q9NVJ2	4.07994	4.26972	5.61046	4.98793	2.58751	4.08341	4.43947	4.59052	5.23452	4.91879	8.49057	9.38427	9.40702	8.56667	9.83288	9.51632
SYNGR1	O43759-3	6.30239	5.98563	3.84925	5.72091	6.02746	5.62479	5.99188	6.11035	6.09389	5.36504	8.05376	7.06936	6.01146	6.30711	7.49847	7.98826
SNX13	Q9Y5W8	6.49492	6.36189	6.15303	6.59993	5.87972	6.19503	6.66241	6.3383	6.20997	5.85004	6.79188	5.40661	6.43963	6.29694	6.29451	6.02519
KLHL20	Q9Y2M5	5.87366	6.30372	6.54496	6.13538	5.86757	6.17886	6.01058	6.70609	6.55574	6.70088	6.48859	6.98575	5.8253	6.025	5.64792	6.15002
SEMA3E	O15041	6.81234	5.89154	4.83021	7.31798	4.96354	4.95827	8.62052	5.66681	6.07594	4.96926	4.51071	7.14928	8.20838	6.66423	6.49572	6.86526
TNRC6B	Q9UPQ9-2	6.29446	4.23944	6.55435	6.64779	7.31463	7.6011	4.74176	5.17362	6.74523	7.11672	6.2082	7.82454	6.13585	5.80373	5.34838	6.2502
TLCD2	A6NGC4	6.58299	6.57145	5.65851	5.88813	6.51858	6.01705	6.63564	6.96039	5.63327	7.11172	6.56849	5.78749	5.98801	6.9642	4.9684	6.14569
RCC1	M0R1Q8	7.066	5.78688	4.73789	5.40348	5.81589	7.177	4.28728	7.19337	9.73408	9.31422	7.39517	9.54333	6.41998	4.20521	3.45614	2.46408
ANAPC15	P60006-2	6.44214	6.29014	6.0055	6.58647	6.66757	6.53373	5.44103	7.11803	5.88969	6.53297	6.02018	6.26463	6.74378	5.99572	5.51914	5.94929
ADAM23	O75077	7.22234	6.76856	6.90726	6.62873	6.17385	6.58885	6.81696	5.91786	6.55762	7.24162	7.45444	5.39261	5.02208	5.50957	4.90488	4.89278
POLR2J	P52435	5.70385	5.8814	4.8353	5.30736	6.04183	4.94884	5.36967	7.32404	5.16249	6.17136	4.7169	5.64025	4.58602	9.88126	7.37238	11.057
CYP2J2	P51589	5.57778	5.19418	6.34171	5.56477	5.66459	5.59409	6.83317	4.66155	4.74977	6.84108	7.44724	6.55163	7.85404	6.84965	7.37136	6.90338
EVPL	Q92817	5.86495	6.77277	6.50088	6.56877	5.9558	6.61056	5.44726	6.17715	5.9911	5.47395	6.75608	6.20114	8.2935	6.01867	5.83957	5.52785
CCDC74B	Q96LY2	7.0048	6.96164	7.30231	6.86785	6.25932	6.86597	7.04458	6.15826	7.79928	7.23637	7.33991	7.14734	3.67097	4.73429	3.39286	4.21425
POSTN	Q15063	5.32927	6.90341	5.05375	8.27399	4.56265	6.42154	7.38762	6.33518	5.48188	5.33631	6.64636	5.84061	6.2959	5.28289	6.99071	7.85793
TBC1D8	O95759	7.09601	6.25647	6.19203	6.04364	6.01712	6.79252	7.05098	6.41589	6.29932	6.8849	5.48159	6.71424	5.78021	5.41257	6.06319	5.49933
LMBRD2	Q68DH5	5.56811	7.06771	5.37066	4.32376	3.53484	6.60691	7.59291	4.78579	7.11266	5.2304	6.90026	7.4663	4.43905	5.85425	10.6321	7.51428
TMEM186	Q96B77	5.49171	7.06217	5.345	6.83254	6.88154	6.64876	6.2418	6.51886	5.10327	5.78808	6.32597	6.00895	7.00809	6.66162	7.73489	4.34674
MBP	P02686	6.04096	5.29423	6.91013	5.27442	7.01007	4.91382	4.74149	6.02783	5.54114	7.77201	5.80147	5.95061	7.95755	7.06613	6.10273	7.59541
CMBL	Q96DG6	7.14279	5.88458	6.08149	5.42382	6.88042	6.2414	5.51019	6.65707	7.3892	6.20526	6.58216	6.45948	6.15507	5.39174	6.00678	5.98856
SERF2	P84101	5.81279	7.08882	7.11313	5.68325	6.84319	6.73393	6.21041	6.83056	5.76415	7.16348	7.04072	6.76567	5.96524	6.47052	4.35588	4.15827
ARL15	Q9NXU5	6.20178	6.07979	5.91972	6.32731	6.16493	5.67502	6.29508	6.28612	6.66426	5.84192	6.61044	5.70014	5.80994	6.86142	6.42929	7.13283
G6PC3	Q9BUM1	6.17214	6.06665	5.71071	6.78787	6.51121	5.87268	6.19111	6.03185	5.96257	6.32235	6.43547	6.01556	6.2329	6.59058	6.05671	7.03964
HOOK2	Q96ED9-2	5.39686	6.40623	6.63109	5.3111	6.64266	6.16079	5.6429	6.50368	7.15595	6.63955	7.18057	6.39379	6.29574	6.15749	5.74017	5.7414
WDR47	O94967	6.14397	5.99996	6.53713	5.79296	7.21435	6.23773	6.76179	5.76212	5.75456	6.1036	6.2909	6.04903	7.32065	6.60884	5.84078	5.58164
DPH7	Q9BTV6	5.79304	6.78442	6.01059	6.91585	5.87839	6.54543	6.57665	8.07218	6.36602	6.46051	6.62034	6.8996	4.88344	5.80069	5.64594	4.74693
CAB39L	Q9H9S4	6.91048	6.6924	6.1435	5.89889	5.58423	7.08003	4.70125	6.3776	6.91222	7.288	5.02359	7.96068	6.27226	6.25804	5.44388	5.45294
OSBPL8	F8W060	6.05414	5.96496	6.50611	6.19037	5.88904	5.64166	6.48618	5.92515	6.34638	5.61222	5.33638	5.50613	7.30293	6.2812	7.74694	7.2102
ZNF326	Q5BKZ1-3	6.75346	8.1551	5.36039	6.83766	6.43034	5.42404	6.17668	7.33285	5.39943	5.72313	5.42403	6.35812	5.15305	6.02428	7.47899	5.96847
ZNF224	Q9NZL3	6.79111	6.93624	5.5495	7.11783	6.66237	5.40405	3.99957	4.13334	7.68229	6.20856	8.58178	7.32268	4.34917	5.13002	7.04586	7.08564
ZNF222	Q9UK12-2	6.53947	7.45902	4.39045	7.70045	6.29273	8.22565	6.28273	5.56412	7.16014	6.94861	8.03072	5.24156	4.86904	5.88872	4.79123	4.61535
TTC17	Q96AE7-2	6.04662	6.40698	5.69417	8.49685	6.63776	6.29404	6.65809	6.21681	6.50093	6.08355	6.24912	6.3095	6.34519	4.82525	5.87671	5.35844
DMTF1	Q9Y222	6.39092	6.38612	6.8617	6.29244	6.54959	6.62466	6.21489	6.69506	6.58208	6.51668	6.86578	6.7834	5.44329	5.21004	5.32642	5.25694
AZGP1	P25311	2.73644	11.9609	1.57969	19.5019	4.64369	7.30564	4.30315	2.07198	4.45907	4.52779	1.87617	4.33486	9.96102	6.07112	3.85721	10.8093
hCG_2039718	K7EN88	7.26367	6.6173	5.69227	5.72943	5.26644	7.83739	5.8672	6.23983	6.68249	7.09913	6.23424	6.31851	4.71119	6.40055	5.67556	6.3648
EID2	Q8N6I1	5.51778	5.20577	8.41544	5.7351	5.7899	7.35298	6.594	7.34503	7.49362	7.51163	8.46507	8.81388	3.41844	4.3209	3.88122	4.13924

KPNA5	O15131	5.95382	6.30791	6.86511	6.76094	6.2973	6.32626	6.02243	6.36963	6.73638	6.07457	7.12526	6.7123	5.70307	5.39087	5.77155	5.58258
FAM13B	Q9NYF5	7.42844	7.32483	9.44755	6.00467	5.07782	5.26217	6.72192	6.20451	6.85872	7.83798	7.16004	6.76541	4.26436	3.90766	4.17608	5.55783
ACVR1B	P36896-4	6.21949	7.01167	5.1956	7.26743	6.57623	6.28778	5.90648	6.56485	6.34015	6.34398	5.9503	6.23086	5.72965	6.24832	7.00042	6.02678
PLSCR1	O15162	6.36387	5.52938	5.98191	7.38656	7.85848	6.60707	5.62219	5.10074	5.68464	6.60604	5.60267	5.96314	6.42211	5.48725	6.83311	6.95083
TMEM143	Q96AN5	6.48061	6.07303	6.00808	6.79258	5.94118	6.0837	6.08546	6.13818	6.07395	6.65215	5.92519	5.51129	6.69435	6.95607	6.42629	6.1579
ABCB4	P21439	5.54357	6.61961	6.36107	6.87067	5.41885	5.69587	6.03748	5.43587	6.39318	7.17105	6.40479	6.74882	6.30066	5.89114	6.76545	6.34191
STAT1	P42224-2	6.58652	6.75688	5.6577	4.66644	5.98913	6.856	5.65991	6.52883	6.75912	5.37514	6.82662	6.9234	6.08388	7.28553	6.81078	5.2341
NUDT14	O95848	7.67746	6.28469	7.8443	6.49882	6.48312	7.45899	4.73221	6.68256	6.31445	6.65253	5.62583	5.41908	5.09349	5.93944	5.51364	5.77936
GSTM4	Q03013	6.29377	4.46164	6.488	4.08398	6.0423	6.07529	5.72643	5.83993	5.51975	7.13041	6.36191	7.08929	5.50469	7.66971	8.46883	7.24406
ACBD4	Q8NC06-3	5.76219	6.89779	5.37814	5.19655	5.47668	5.24433	7.38787	6.71839	5.7731	7.18569	5.69918	5.91434	6.60856	7.95482	6.87054	5.93181
LAMA2	P24043	6.81868	7.41766	5.96249	7.23836	6.96592	6.07632	6.50279	6.5907	6.27905	7.49382	7.67179	6.98733	3.96968	4.73418	4.1975	5.09374
MTDH	E5RJU9	6.89317	5.75786	6.38792	6.0426	5.91263	4.4361	6.80656	4.45705	7.5674	7.4184	6.0689	7.79441	5.41841	6.16126	7.17752	5.6998
MT-ND6	P03923	5.81337	6.32912	6.39455	4.18071	6.86909	6.73387	6.72051	6.92577	5.15246	6.91038	6.0345	5.61824	7.05847	6.21087	5.98482	7.06326
UBASH3A	P57075	5.46132	6.28938	6.34544	6.37463	6.29039	6.90198	5.37989	7.93887	6.72236	7.0232	5.75425	7.03561	5.31146	6.03869	5.36031	5.77223
SORCS2	Q96PQ0	5.4192	7.50425	5.8442	7.85808	5.79607	3.99822	6.47293	6.53043	5.19113	6.1821	5.8793	6.25428	7.14656	6.98643	7.19327	5.74354
TMEM120B	A0PK00	5.863	6.52341	6.23774	6.77986	6.77841	5.67023	5.94621	5.28838	6.20511	5.61322	6.75125	5.32925	6.58291	6.72455	6.60203	7.10444
YIPF1	Q9Y548	5.73227	6.48472	6.30582	6.34763	5.69762	5.91978	6.34958	5.80087	5.91822	6.64282	5.9266	6.41983	6.59696	6.59191	6.25164	7.01374
SLC39A13	Q96H72	5.80249	5.85565	6.62331	6.90782	6.78731	5.72148	6.07773	4.90045	6.07357	5.0501	6.25518	6.88577	7.27179	5.56262	7.08812	7.1366
SELENOM	Q8WWX9	4.83738	4.91143	4.47853	4.97172	5.75127	4.0874	5.91708	7.20369	4.1771	5.40342	4.90566	5.7545	4.80468	12.4551	10.1356	10.2054
SHARPIN	Q9H0F6	3.65255	3.61466	6.28166	3.2272	6.17887	5.24842	5.09453	9.36453	6.31123	2.45977	5.54081	9.54797	4.40303	9.49234	8.92954	10.6529
DYNLT2B	Q8WW35	5.61991	5.96891	6.48603	4.98185	5.67834	5.82898	6.73321	6.06578	4.83676	8.82013	7.59844	6.25515	5.80121	6.15906	7.00458	6.16166
PALM	O75781	6.19194	6.00469	6.35506	4.46777	6.76721	7.27239	5.98449	5.63415	4.5978	6.4856	7.02354	6.62023	7.72562	7.34355	5.74468	5.78127
PTP4A2	Q12974	6.42258	5.92496	6.81188	5.73189	6.4074	5.90717	6.85981	6.51185	6.72323	6.68454	6.40358	6.75987	6.77811	5.47769	5.31222	5.28322
PLEKHJ1	Q9NW61	8.28697	5.80062	6.65808	4.5905	5.63902	7.73358	6.17869	6.09385	8.07073	7.87141	7.56395	5.32174	5.06667	6.23159	4.49885	4.44473
TTBK2	Q6IQ55	8.15849	5.12896	2.95544	7.6577	9.40544	5.14226	6.06001	6.60471	5.45311	5.65059	3.68344	6.73137	7.33743	5.47457	8.47231	6.08417
PAIP2	Q9BPZ3	7.55064	6.19898	6.19951	4.97216	5.68441	6.26316	5.33265	7.29858	6.21601	7.4794	6.96931	7.3471	5.09516	6.78244	4.89035	5.72017
PRRG1	O14668	6.13913	6.62248	4.47641	6.66064	7.02094	6.33472	6.39808	5.93789	5.91681	6.01955	6.36817	6.10681	5.77602	6.03419	7.31492	6.87325
ABCA1	O95477	6.2124	7.14966	5.67591	6.19482	6.5802	5.71712	6.0806	6.03851	6.00744	6.98021	5.93462	6.01157	6.04498	6.44726	6.32074	6.60396
STOX2	Q9P2F5	6.24	7.22974	7.53906	6.65033	5.24721	4.97917	6.62841	5.46121	6.48389	6.24063	7.9039	6.35396	6.33764	5.48202	6.51314	4.70971
MON1A	Q86VX9	6.4992	6.1568	7.33476	5.78869	5.21096	6.43034	6.20836	6.33872	7.03434	6.76952	6.68559	6.65088	5.76641	6.22382	5.45958	5.44264
PCNX1	Q96RV3	7.43402	7.76256	4.17331	9.56313	5.49207	4.74167	7.0328	6.05758	6.47065	5.30919	5.91683	7.37793	5.03931	3.8142	5.16665	8.6481
CDKN2A	P42771-4	6.92444	5.47091	6.11793	4.46083	5.47565	4.95263	4.77646	6.6682	4.78841	5.92949	5.66342	4.99364	4.35695	11.7194	8.0731	9.62856
RHBDF1	Q96CC6	6.10453	4.6802	5.14561	5.79073	6.3244	5.86172	5.73095	6.60584	8.07722	6.30923	6.276	6.67127	6.65378	6.84267	6.29406	6.6318
TTC30B	Q8N4P2	6.39551	6.6264	7.9636	6.04124	6.17581	5.66039	6.25862	5.83631	6.88509	7.20503	7.03137	7.27921	4.76532	5.43581	5.71685	4.72345
SH3BGR	P55822	6.01309	6.43637	5.76883	5.56941	7.24112	6.64041	5.84968	4.87132	6.62724	7.11929	6.25733	7.67614	6.2531	6.51537	6.42333	5.63796
SORBS1	Q9BX66-12	6.26166	5.4034	5.5725	6.22118	5.64108	5.49208	6.6957	5.90764	6.21227	6.94949	5.69742	6.25128	6.67578	6.63074	7.80155	7.58626
ZNF764	Q96H86	6.1597	6.51016	6.06078	6.44095	7.18899	6.33048	6.15452	5.58895	6.06891	6.08874	6.553	6.37116	6.48884	6.6968	5.81234	5.48569
SNCA	P37840	6.53748	6.37925	6.79566	6.24582	5.04435	6.3375	5.82294	7.0128	6.32753	6.71244	7.37534	6.92587	6.29182	6.02924	5.81226	4.34972
ALKBH3	Q96Q83	6.23316	6.50741	7.28146	5.72099	6.3772	6.59139	6.27176	6.69813	6.60171	6.6873	7.02077	7.38706	5.38767	5.17989	4.92789	5.1262
SNX18	Q96RF0	6.76632	5.90861	7.03418	5.94575	6.64519	6.52534	6.19231	5.67223	7.05548	6.49914	7.17362	6.38288	6.13503	5.5352	5.66203	4.86668
VDAC1	C9JI87	5.66201	5.49855	4.44185	6.87354	6.12525	5.55158	6.1273	5.85058	7.45639	5.36728	5.28166	6.31036	6.83242	6.80592	8.7801	7.03521
SEMA4G	Q9NTN9-2	6.31741	7.36066	5.15585	7.59959	6.73478	6.78295	5.28063	5.64515	7.4705	7.4439	6.43168	5.48062	5.77567	6.52851	5.26443	4.72769
MAPKAP1	H0Y866	5.56731	5.59679	5.58068	6.07534	6.75669	6.05973	6.21313	5.52162	7.8055	6.2149	4.89549	5.85041	6.45956	6.4473	7.89949	7.05608
DNAAF3	Q8N9W5-3	5.88261	5.85015	6.46811	5.10075	7.04681	6.31952	6.30876	6.11434	7.16177	7.40191	6.10191	6.47001	6.42268	5.95311	6.29011	5.10745
C2CD3	Q4AC94	7.5611	7.86571	4.81422	4.93004	5.83108	8.39438	6.34732	4.79366	5.44465	6.06107	5.93041	5.98666	7.42584	7.15254	6.46199	4.99933
NEK2	P51955	5.71514	6.57011	7.22862	7.4413	6.13682	6.76869	5.80367	5.62125	7.55303	8.40541	8.8882	8.12361	3.96476	3.88405	3.93082	3.96454
TSPOAP1	O95153-3	5.97616	6.05361	6.67206	5.50971	6.14024	4.68796	7.56321	6.23369	6.46129	6.59557	6.08671	7.34581	6.43628	6.08143	6.14139	6.0149
TEDC2	Q7L2K0	6.43478	6.13916	6.96142	6.21693	6.14205	6.12212	6.75941	5.82216	6.71366	6.73031	7.11057	7.01084	5.56083	5.45204	5.45882	5.36491
SPIRE2	Q8WWL2	6.97789	7.51015	6.27557	6.26324	5.77949	5.39316	5.38301	7.00768	7.35567	6.63854	5.65707	7.12841	6.40327	6.07541	5.7244	5.42703
LRP8	Q14114	6.79438	5.8316	7.0199	5.73585	7.63981	5.78565	5.47807	5.93166	6.55711	5.067	7.0329	6.6904	5.97329	5.04296	6.55877	6.86065
ETFRF1	Q6IPR1	6.39989	5.79774	5.74377	6.45587	6.66129	6.11715	6.6539	5.76745	5.47214	5.85216	6.22081	6.1004	8.07536	6.52298	5.73425	6.42482
ATP5F1C	P36542-2	6.54279	6.41078	5.50812	7.37278	6.20116	5.71072	6.45498	6.30071	8.1925	6.21843	5.16851	4.57976	5.75061	6.05446	7.17377	6.35994
ECM1	Q16610-4	6.1957	6.91038	5.28892	6.26147	5.90917	6.57404	6.17805	6.4693	6.43294	6.61767	6.45729	6.92706	6.22099	5.7815	5.53085	6.24468
PGAP2	Q9UHJ9-2	6.12075	6.08758	5.90339	6.49306	6.67557	5.89299	6.0195	6.31546	7.03646	6.11944	5.63656	6.89021	6.4315	5.51097	5.98676	6.8798
PLAAT3	P53816	6.35395	5.58764	7.73682	5.66741	6.93954	7.14295	6.73323	6.93548	6.62286	6.83649	7.64397	7.36206	4.00957	5.71388	4.03159	4.58256
ZNF692	Q9BU19-5	6.5793	6.78833	6.27537	7.05036	5.04155	6.37572	6.52486	5.9584	6.51407	6.51866	5.28529	5.7278	5.92916	7.32362	5.87637	6.23114
TSKU	Q8WUA8	5.79234	6.05369	5.12383	5.46345	6.85408	5.15878	6.14368	7.13701	5.49504	6.44231	6.59248	8.49924	6.27298	5.70152	6.70016	6.56943
NGRN	Q9NPE2	6.28716	5.64574	6.55203	6.57176	6.76523	5.31606	7.31624	6.49684	6.09322	5.97423	6.8211	6.62705	5.9756	5.05777	6.05636	6.4436
MED31	I3L2J1	6.0066	4.46276	5.78454	5.65219	5.8544	4.19334	4.83211	6.78553	4.91067	6.60062	5.67512	7.75562	6.25048	7.56865	7.82862	9.83877
ITFG2	Q969R8	4.36763	5.08263	8.1251	6.40825	4.63349	7.6672	6.92012	5.68403	7.95387	7.86857	6.2328	4.81423	6.90647	6.19104	6.98088	4.16368
LRRC9	Q6ZRR7	6.24241	6.44014	5.95591	4.80426	6.84797	6.77718	5.94226	6.17654	7.55252	6.58984	7.24542	6.82735	5.59631	5.74622	5.71004	5.54566
TMEM102	Q8N9M5	7.81132	5.35381	7.80532	6.39117	6.42142	5.07221	5.61965	7.09542	5.22375	5.46975	5.71538	6.82398	4.80759	7.6105	7.55505	5.22367
TK1	K7ERV3	5.23638	7.10873	9.49484	4.97387	5.5467	6.00329	5.96894	5.41311	6.05895	6.01465	6.34629	6.03606	5.89813	7.11785	5.93623	6.84598
COMTD1	Q86VU5	7.0091	5.78122	6.44792	6.81262	5.88951	5.25381	7.70016	5.67079	5.95234	6.361	5.76878	5.05816	5.59412	6.27579	7.25674	7.16796
CDPF1	Q6NVV7	7.31836	5.1969	8.02235	4.7802	5.1318	7.00426	5.56259	4.46157	8.06087	5.07004	6.30904	7.36303	6.46224	5.08112	7.7847	6.39093
MEIS2	O14770	6.55306	6.21392	5.8555	4.93223	6.36387	5.50076	5.126	5.67828	6.77126	7.47844	5.52346	7.73704	5.89174	6.93848	6.05286	7.3831
A0A1W2PSA9	A0A1W2PSA9	6.48025	6.09019	5.84464	6.28863	5.52243	5.72315	6.24737	6.03217	6.15957	6.33166	5.15687	5.98676	6.94787	6.74656	7.16727	7.27461
CYBRD1	Q53TN4	6.05226	6.49721	5.37067	6.74952	6.30522	6.73804	6.70681	5.70589	6.29677	5.44314	6.21691	6.30098	6.2978	6.84371	6.85513	5.61995
CYB5A	P00167	5.47974	5.89706	4.8675	4.77401	6.08569	5.20346	6.20708	7.12026	4.24469	4.49843	4.50517	5.43003	5.80202	11.0908	8.83211	9.9619
AMMECR1L	Q6DCA0	4.66894	5.91245	6.02268	4.6267	5.62017	5.47232	4.46919	7.42412	9.90275	5.81413	4.99093	7.12628	5.53708	6.71866	8.3788	7.3148
ETAA1	Q9NY74	6.06633	6.40224	5.48737	6.17954	6.20393	7.32157	5.99081	5.47866	6.99496	6.40723	6.83384	6.36996	6.82654	5.43394	6.20291	6.83096
SDCCAG8	A0A0G2JQM2	6.32398	5.39506	6.46098	5.56909	5.86288	5.58403	6.80526	7.51775	6.90064	5.62413	6.25818	6.67437	6.63632	5.60941	6.27502	6.50289
SDCCAG8	Q86SQ7	6.35632	5.53137	6.23138	6.28631	5.84325	6.35243	6.18038	4.83553	5.49005	7.12254	6.60987	7.09814	8.06184	6.59176	5.55246	5.85638
NAT1	P18440	6.78789	5.57603	5.79566	6.35977	5.85905	7.54133	7.19207	6.34511	6.67926	7.28619	6.14272	6.83182	5.92887	5.71325	5.60248	4.3585
SLC2A4	P14672	7.10794	7.713	5.82301	7.25265	6.37564	5.63877	5.70412	6.65747	5.61538	8.03318	6.34875	6.13948	4.91361	5.10682	5.55789	6.01228
CTHRC1	Q96CG8	6.94462	6.33539	6.00934	6.894	5.74054	5.88112	6.75612	5.40264	5.90491	6.44393	5.94782	5.95405	6.49723	6.72423	6.14278	6.4213
RNLS	Q5VYX0	5.29429	7.02511	6.23491	7.96577	6.75882	5.19335	6.99385	5.63246	5.99037	5.32348	7.05564	7.38835	5.0629	5.79778	6.22766	6.05524
NAA38	Q9BRA0-2	6.30107	6.26487	5.8648	4.89209	4.63668	5.46289	5.01698	8.23159	6.31047	7.87798	6.70403	6.7804	5.02003	6.98637	5.81064	7.8391
NEMP2	A6NFY4	5.9416	9.06006	6.03773	4.07273	7.41186	6.1112	8.46172	4.43244	7.78581	6.9401	4.3603	6.81953	3.56664	6.04851	6.59598	6.35379
F2R	P25116	6.26982	7.28269	6.29482	7.4663	6.27559	6.56138	6.10678	6.31231	6.30191	6.2076	6.07877	5.84697	5.3947	5.68663	5.71752	6.19621
KLHL26	Q53HC5	6.50525	5.90868	6.85076	5.95592	6.03571	6.48264	6.00419	7.3216	6.32969	5.72822	6.47807	6.89492	5.88123	5.93185	6.00667	6.58459
VPS29	Q9UBQ0	5.78066	6.09028	6.1149	5.92117	6.09637	6.29111	5.6554	6.08205	6.55082	6.42546	6.25606	6.00857	6.38779	6.54401	7.13902	6.65633
HENMT1	Q5T8I9	4.95742	7.84345	5.62365	7.43992	5.06347	5.76979	7.2673	8.40655	6.14599	5.76284	6.87139	6.00652	6.59229	6.70559	5.35251	4.19131
PRELID1	Q9Y255	5.76253	5.78363	4.5158	6.50037	5.75097	7.27746	7.58297	7.67744	8.10615	9.7394	8.80327	8.99319	2.45175	4.42543	2.5585	4.07116
LRRC23	Q53EV4	6.5016	7.35518	6.16403	6.3697	8.14389	6.16366	5.97685	6.99655	5.4114	6.26719	7.07056	7.36926	5.28265	5.16209	3.61902	6.14637
ZNF324	O75467	6.10108	6.01911	6.44937	6.63293	6.95858	6.4273	6.53703	5.68514	6.11988	5.47719	5.9501	5.7363	6.93419	6.56569	7.30898	5.09711
TMSB10	P63313	5.34879	5.79394	4.46245	5.57105	4.79127	4.57486	5.0071	6.83166	3.84459	5.16072	4.30069	5.68271	3.37852	14.6365	7.00097	13.6142
TNFRSF10D	Q9UBN6	8.10546	7.36715	7.19668	7.61629	6.79665	6.17273	7.1444	5.76961	7.17596	6.43797	6.83522	6.5827	4.6213	4.07867	3.98177	4.11744
PFDN6	O15212	4.88733	4.62319	5.21187	5.00597	4.97604	4.3183	3.97875	7.53212	3.98733	6.39957	4.58367	6.42351	4.80098	12.6633	8.85371	11.7543
MED22	Q15528	5.55331	4.62159	5.14789	4.05641	5.17856	4.53136	4.89463	6.22173	4.54432	5.802	4.78377	5.49714	5.41701	11.7674	10.3793	11.6036
CERK	Q8TCT0	7.3806	7.22731	8.34249	7.20171	5.53264	5.83804	6.0188	6.23247	8.73251	6.39603	8.17526	6.17164	3.70407	4.32563	4.43247	4.28831
PGBD4	Q96DM1	5.99955	6.22454	5.58655	5.7383	5.9917	6.23898	6.29343	6.18818	5.213	6.38731	5.99537	6.45614	6.40174	6.73747	7.60805	6.93968
TRAK2	O60296	6.02327	6.98818	6.30338	6.98179	6.49386	5.81974	7.97394	8.97623	7.35496	4.37863	6.95013	7.42252	5.10035	5.28929	4.81003	3.1337
CCDC66	A2RUB6	6.23454	6.20037	6.30062	6.63623	7.05661	7.20044	5.95282	7.29128	6.95762	6.13143	7.04579	6.95965	4.54301	5.55172	4.68481	5.25304
POLR3K	Q9Y2Y1	6.04826	5.18242	6.41347	5.51079	6.41946	5.54652	6.18316	7.24431	5.15375	7.32551	6.67436	7.61019	5.73078	6.33823	6.47411	6.14467
DKK3	Q9UBP4	8.60488	6.73544	7.84689	6.80131	7.61706	6.06807	8.25283	7.06096	7.47581	7.27825	7.29083	6.32668	3.61403	3.56239	2.37861	3.08595
TRMT44	Q8IYL2	6.47415	6.52491	7.27244	6.86019	7.32715	7.80101	6.35336	5.61145	6.59396	5.37177	6.67694	5.66512	5.40179	5.75963	4.07078	6.23536
KBTBD8	Q8NFY9	6.1138	6.21884	6.88179	6.40203	5.82589	6.97297	6.00472	7.05262	6.63514	6.4461	6.97878	6.85301	4.94584	5.35065	5.44213	5.87568
CABYR	O75952	7.10291	7.32085	6.05182	6.78367	5.68782	6.1897	6.33061	6.81035	5.47998	5.57437	8.50836	8.22804	5.58472	3.96798	5.39642	4.98241
CAPG	P40121	6.20122	4.87732	7.04086	6.54763	6.2448	6.27596	6.20797	6.25835	6.90509	6.70766	9.21958	8.54566	5.47645	3.77384	5.6341	4.08351
DBF4B	Q8NFT6-3	5.51412	6.17156	6.30972	6.4854	6.37839	6.29689	4.4598	7.40516	5.86338	6.40754	7.52993	6.80712	5.99635	5.97329	6.31093	6.09042
MOSPD1	Q9UJG1-3	6.05789	6.08777	5.96794	6.12818	6.65163	5.84961	6.37241	6.29277	6.09487	5.79299	6.19205	5.8721	6.74582	6.72604	6.80569	6.36223
GSTA4	O15217	3.69343	7.47527	8.50167	5.4789	5.92591	7.9903	7.03775	7.32551	7.81727	5.7943	6.18886	5.37665	4.97664	5.5784	5.02801	5.81114
TCEA3	O75764	5.88182	6.35246	6.74071	6.40377	6.25851	6.16043	6.17971	5.5524	6.35677	6.91099	6.39043	5.91504	5.63249	7.92048	5.61598	5.72801
NUDT11	Q96G61	6.5987	7.27781	6.41481	5.67365	7.07286	6.57645	5.40951	7.56517	6.93027	7.31565	7.15332	6.13186	5.4323	5.17671	4.47719	4.79373

APOC1	P02654	7.26046	6.15744	4.89344	6.48297	5.98798	4.44296	5.65463	5.48727	4.70119	6.15937	5.67593	4.89152	3.39198	10.9958	6.68641	11.1306
NOS1AP	O75052	6.41815	7.11624	6.7559	6.46278	5.72853	5.96031	7.49433	5.93318	5.90914	7.63201	6.08024	5.14323	5.84592	4.92115	7.06512	5.53378
LAPTM4A	Q15012	6.85841	7.27304	5.5402	6.65562	6.48367	7.1572	7.98676	5.61175	5.87631	6.06824	6.87287	7.49369	4.53575	5.79862	5.01941	4.76845
EIF1B	O60739	6.18236	5.47344	7.39038	6.30629	6.92824	6.97629	5.09185	5.8285	5.64685	6.66641	6.66643	7.01422	5.94897	5.78276	5.72682	6.37021
SULT1A3	P0DMM9-3	5.01124	4.83185	6.72981	4.57661	7.38046	6.20683	5.26592	8.91184	6.98547	7.4491	6.26992	6.48918	7.13173	5.5919	5.67035	5.4978
SDHAF4	Q5VUM1	6.1682	5.34211	5.78839	5.27026	7.76031	6.3188	5.72546	6.91078	5.87287	6.32189	5.37379	6.07808	6.53892	7.02161	6.35065	7.15789
RAP1GAP2	Q684P5	5.23542	7.97744	8.34268	6.73065	6.23771	5.93277	7.65294	7.36791	5.53926	5.50728	5.93856	6.46141	5.52655	4.70193	5.10898	5.73849
AGAP1	Q9UPQ3	6.59007	6.65645	6.9218	5.41616	4.6162	4.62989	7.19751	4.63754	6.81317	6.41276	6.61958	7.84426	6.59801	8.44645	5.55272	5.04742
TFPI	P10646	6.40596	6.15747	5.75163	6.74057	6.0309	6.16324	6.27089	5.9317	6.17097	6.45971	6.56951	6.04473	6.52053	5.31492	7.33205	6.13523
USB1	Q9BQ65	5.89237	6.35511	6.64578	5.75346	6.16473	6.44468	6.00145	6.10775	6.51716	6.35851	6.28224	6.58303	5.74178	5.95862	7.09473	6.09859
FZD7	O75084	7.63996	7.37436	5.76975	6.51234	4.80858	5.38247	7.12799	6.05299	6.43681	5.55131	6.63852	6.04037	5.83637	6.24591	5.94612	6.63613
PIGX	Q8TBF5-2	6.44175	6.92199	6.13225	7.37237	5.77851	5.88135	5.86709	5.29431	5.5627	7.43643	7.42773	5.70569	6.58598	5.89824	6.49256	5.20104
USP31	Q70CQ4	6.82533	6.71454	6.22625	6.65954	6.60476	6.03141	5.78681	5.74091	5.51373	5.99735	6.53722	6.03128	6.37579	6.39168	6.32677	6.23662
SPRED3	Q2MJR0	7.16764	5.12626	5.78031	7.61652	3.54831	6.59157	6.84396	5.20209	5.88915	7.78107	7.98449	6.46863	6.42625	4.6238	6.28479	6.66516
MPLKIP	Q8TAP9	6.24552	4.88496	7.60225	3.69159	6.87738	6.42843	6.1906	5.25815	7.5126	6.95517	7.03964	6.38685	5.58641	4.98179	6.16891	8.18976
TMEM17	Q86X19	5.56721	5.96898	5.03562	6.54613	6.36179	6.12178	5.99496	6.17955	5.46316	5.09332	6.01287	6.03229	6.61186	7.79414	6.76637	8.44997
DNAJC24	Q6P3W2	6.4442	5.68172	7.05998	5.34591	6.0993	6.44867	5.57661	6.8734	5.60451	7.14044	7.15036	7.58427	4.79473	6.07856	5.60181	6.51555
TRAPPC2	P0DI81-3	4.90641	5.84042	6.63659	5.21052	6.4909	6.765	5.5551	5.90733	5.40331	6.52338	6.87582	6.45315	5.29892	7.82979	6.79887	7.5045
TASP1	Q9H6P5	7.22238	7.03706	5.87517	5.7926	6.17083	5.78147	6.07719	8.00524	6.14247	5.43668	5.64271	5.71996	6.9424	5.57973	7.22072	5.35339
ARHGAP24	Q8N264	5.89204	5.71626	6.34417	5.88324	6.10886	7.44935	5.50303	6.9249	6.09865	6.54339	6.74529	5.97681	6.68267	7.65311	5.42965	5.94857
ZCCHC14	Q8WYQ9-2	5.65021	5.76859	7.42156	6.34589	5.57461	8.02712	6.73052	6.74022	5.2326	6.87111	6.93829	8.50516	5.49033	4.89759	4.27848	5.52772
TLNRD1	Q9H1K6	6.04884	6.5422	5.28513	5.40833	6.92699	6.05002	6.24157	6.35841	6.54989	8.27013	6.24968	5.42468	5.85152	6.50787	6.19866	6.08609
MAP1LC3A	Q9H492-2	5.35327	5.02829	6.53221	5.65816	6.94427	6.44174	6.27466	6.46433	7.14231	5.07291	5.8873	6.86478	5.8334	6.62781	6.97176	6.90281
RRNAD1	Q96FB5	5.76819	4.97302	8.32561	6.93071	5.35814	5.85193	5.94138	6.29949	7.19915	6.15161	7.6454	7.12559	5.80171	5.33685	5.73489	5.55633
ZNF581	Q9P0T4	5.90878	6.21791	5.66827	7.12819	5.84056	5.56638	6.37931	5.76133	6.64619	6.64941	7.04307	7.02585	6.67359	5.17453	6.27571	6.04093
RIBC2	Q9H4K1	6.95991	6.2129	6.4328	6.77853	6.69078	6.23353	6.25266	6.4248	6.84986	6.86238	7.5315	6.36164	5.13528	4.60748	5.23154	5.43443
ZBED6	P86452	5.35063	5.90501	6.10696	4.48132	6.27034	7.16097	5.45853	6.90405	6.28428	6.03825	5.69897	6.59172	8.04944	7.73408	6.49641	5.46905
RPL22L1	Q6P5R6	7.31378	7.58687	8.53219	8.6382	5.93027	5.87845	6.54853	4.80728	5.97692	7.09588	7.55815	7.34134	4.17264	4.20865	3.96779	4.44307
ZNF407	Q9C0G0	5.64636	6.21268	5.28898	5.33996	6.1946	6.15555	6.09792	6.57483	6.92835	6.88859	5.51734	5.71506	6.3918	6.4977	7.37536	7.17493
TFPI2	P48307	7.09558	6.36443	5.40664	8.096	5.21477	6.77228	7.15409	6.84536	7.03296	6.85354	5.6711	6.36106	5.65084	4.15167	5.91781	5.41185
AP3S2	P59780	6.94927	8.64615	7.18903	5.13612	5.07945	6.77962	8.29683	6.1223	7.65273	5.4954	6.81546	6.21712	4.33904	4.09829	6.57752	4.60568
SLC35C2	Q9NQQ7-3	6.15949	6.73546	6.20107	6.02758	5.76748	7.04927	7.12632	6.91232	7.12458	4.80269	5.32999	5.56543	6.22	6.53997	6.83009	5.60829
CIPC	Q9C0C6	5.94665	6.52281	5.68142	6.28772	6.30087	6.21127	5.79404	5.63602	6.14574	7.16712	6.19112	6.50341	6.45009	6.55986	5.9547	6.64715
IER2	Q9BTL4	4.32769	5.18629	4.85765	5.42941	6.0782	5.0429	6.15901	6.26354	4.90213	4.89709	4.661	5.79503	9.05131	8.47717	9.57098	9.30059
PLSCR3	Q9NRY6	5.23463	5.9713	4.78443	6.2564	6.46069	4.78119	5.89231	5.54154	6.39586	6.36223	6.59385	6.69073	7.57236	7.74143	5.622	8.09907
MITD1	Q8WV92	5.79361	6.85667	7.18341	6.12191	6.8969	6.31236	6.11529	6.15165	5.64847	6.96667	6.56381	6.04706	5.77478	5.58123	6.02033	6.96585
MICU3	Q86XE3	7.40689	7.10052	5.40394	7.36011	7.06952	6.0285	5.4991	6.19328	5.8399	6.15425	5.05538	6.44993	6.31984	6.2985	6.13667	5.68366
INO80C	Q6PI98-4	4.33336	5.83597	4.87671	7.79769	6.60199	6.34275	6.00609	7.05882	5.3411	6.48757	8.13017	6.86245	4.84169	7.04444	6.11643	6.32278
SLC39A8	Q9C0K1	6.19336	6.77572	6.83673	7.17297	5.72324	5.88598	6.88605	5.93372	5.92409	6.37334	6.45659	5.89743	5.89736	6.30762	6.26751	5.46829
CES2	O00748-4	7.20934	6.59514	6.18303	6.03589	5.34519	5.68687	6.7346	8.46475	5.42243	4.61207	5.38067	5.46775	7.11332	7.34814	5.98381	6.417
AGGF1	Q8N302	6.0529	6.33699	5.6013	6.44213	5.30271	6.19577	6.32555	5.9614	6.00161	6.52285	6.49545	6.6169	6.5795	6.43325	6.81851	6.31017
HMBOX1	Q6NT76-5	5.93565	6.26077	6.16298	6.36113	6.43988	6.63676	6.04532	6.1461	6.23162	6.75958	6.60517	5.78459	6.05788	6.09864	6.65524	5.8187
EFNA1	P20827	7.3151	8.37737	7.48628	8.56173	6.87951	5.60666	6.57944	6.52815	5.72803	7.7378	6.94972	7.15938	4.30135	4.07163	3.84042	2.87743
B9D1	Q9UPM9	6.215	5.68246	6.43629	5.92375	5.40223	5.52423	5.66187	7.08367	6.80337	6.32433	6.28519	6.64415	6.30177	6.75698	7.01889	5.93582
COL3A1	P02461	6.59204	6.29521	5.61264	5.9397	4.66899	4.94227	4.90441	4.65131	6.91356	5.70361	6.40664	5.51675	7.98707	7.43778	9.24871	7.1793
PODXL2	Q9NZ53	6.45897	5.68531	5.8535	7.65119	6.31717	6.15067	6.2738	5.67376	6.43264	6.29119	7.67058	7.73402	5.61634	5.20862	5.30007	5.68215
ERCC1	P07992-3	6.12156	5.73862	6.50413	5.02241	6.49244	6.82331	6.78592	6.42876	6.38451	6.23034	7.64947	6.73633	5.48954	5.95966	5.79547	5.83753
ARNTL2	Q8WYA1	5.89538	6.91746	5.90567	7.21765	6.84735	6.41769	6.53987	7.25857	7.41275	6.03283	6.13914	5.25315	4.99629	5.68728	5.46922	6.00969
TM2D1	Q9BX74	4.94744	4.88796	5.65059	7.23665	6.25682	6.99606	4.93714	6.94542	7.85085	7.37658	5.95614	6.55638	5.24018	6.79032	5.98139	6.39006
SPA17	Q15506	7.1816	6.52604	7.04044	5.44128	7.16694	6.79174	5.89524	7.45828	6.41917	6.47472	6.58967	7.66277	4.79693	5.38862	4.24667	4.91989
UBXN8	O00124	4.84256	5.6807	5.69671	7.65266	5.57429	6.15983	6.3824	6.74202	4.71889	7.12935	6.62316	6.30302	7.18653	5.84734	7.2616	6.19894
C18orf32	Q8TCD1	6.3555	5.78856	5.16713	5.13039	5.59189	4.45795	5.39998	5.62146	5.34204	5.78689	5.70701	5.87763	6.08554	9.02207	8.65016	10.0158
ATAT1	Q5SQI0	5.79725	6.60747	6.1821	5.36164	6.1178	7.21705	6.2739	6.4188	6.36761	6.83935	6.35154	6.59734	6.95706	4.95595	5.73903	6.2161
MOSPD3	O75425	5.85972	5.50048	6.35396	6.09762	5.71649	5.67238	5.09385	5.74688	6.71795	5.42864	6.15838	7.37878	7.45973	6.02799	6.45872	8.32843
ABITRAM	Q9NX38	5.25977	5.75992	6.9459	5.42957	5.75921	6.50654	6.27504	5.15297	6.36549	6.19495	6.56857	6.59959	6.50795	6.47153	7.03187	7.17112
ARNTL	O00327	5.59959	5.86581	5.95907	6.63046	6.10093	6.37619	5.99123	5.96738	5.8954	6.61251	6.03601	5.93579	6.77183	7.05206	6.31262	6.89311
FOXP2	O15409-4	5.90422	6.48237	6.41591	5.79285	6.37975	6.32857	5.91103	6.55655	5.87143	6.11817	6.16294	5.76146	6.7711	6.96745	6.86118	5.71502
HOXC10	Q9NYD6	5.49244	6.19575	4.99294	6.28614	6.52838	6.48157	7.87527	5.66185	7.69769	7.32986	5.35845	6.23939	6.05082	6.05939	6.27968	5.47037
LRR1	Q96L50	5.8552	6.48151	7.23925	6.19609	5.81502	6.4248	5.81611	5.76225	6.65203	7.0058	7.16291	6.41168	5.54956	5.19162	5.93681	6.49935
B3GNT9	Q6UX72	9.26034	5.52191	4.24871	7.08741	5.59471	5.56619	6.46652	5.15591	9.09312	7.46427	4.01494	7.15639	6.59297	6.03117	5.60888	5.08156
ATRAID	Q6UW56-3	5.61897	6.7266	6.3608	5.726	6.70479	5.88471	6.15549	5.59619	6.45378	6.38343	6.66464	6.14361	5.89761	6.25596	7.48688	5.94054
CFAP251	Q8TBY9	9.06185	5.97626	5.82333	5.65436	7.91795	6.56076	7.28675	6.05109	5.57973	6.50545	6.36716	7.60019	5.44795	3.75913	4.38075	6.0273
TNFAIP8L1	Q8WVP5	5.29871	5.76705	5.70496	5.45624	5.93723	6.94526	7.35849	7.15464	6.40474	7.2513	7.93944	7.79393	5.95871	5.4943	3.96293	5.5784
TF	P02787	5.73132	6.51263	6.27168	5.84821	5.26827	6.81432	5.81652	6.40872	5.57308	4.7876	6.14507	7.44999	5.21118	6.63138	9.1793	6.35073
CNTN3	Q9P232	5.82988	5.8412	6.84366	6.01765	5.66763	5.80209	6.52669	7.23297	6.43428	7.13489	6.62679	6.9052	5.61357	5.94123	5.47678	6.10548
ERN1	O75460	6.30467	6.48084	6.27544	7.19903	6.70305	6.15333	6.86702	6.04954	6.09894	6.44177	5.9199	5.88346	6.04614	5.56009	5.7899	6.22689
CD82	P27701	6.6783	6.96245	6.28081	5.81154	5.77827	5.59258	5.8757	6.20285	6.69038	5.80498	6.86518	5.89336	5.55316	6.75586	6.62112	6.63346
SKAP2	O75563	6.90902	7.89557	7.80125	6.83771	6.27555	8.14268	6.10169	5.9405	6.49304	6.75495	7.66361	7.16428	4.56627	3.60109	3.96024	3.89255
IFT57	Q9NWB7	6.10131	6.67407	6.43361	5.25682	4.97439	6.97857	6.06841	7.60546	5.5979	6.74232	5.67949	6.59141	7.39417	4.66603	7.09722	6.13881
SS18	Q15532	6.83802	3.41583	4.78684	3.70522	4.62219	3.63962	3.69785	4.94553	3.14638	6.18333	4.90879	5.60024	6.30186	12.9766	11.0164	14.2153
NUAK1	O60285	5.62323	6.10265	6.68651	4.80406	5.38083	7.28485	7.28778	4.8791	5.14003	7.14669	7.13873	6.34415	6.84742	4.88317	7.35354	7.09728
ANKRD40	Q6AI12	6.19787	5.99438	6.8927	4.16794	6.51262	6.45547	5.42756	6.58854	6.35285	6.50432	6.79579	7.15371	5.3476	6.12054	7.27751	6.21058
KATNIP	O60303	7.01641	6.49142	5.87256	6.2481	5.85658	8.78079	5.06925	6.90131	6.31124	4.65014	8.69565	7.03064	5.70882	4.4082	5.09531	5.86357
ZDHHC23	Q8IYP9	5.66052	6.22443	6.49101	6.35799	6.51981	5.91297	7.92076	5.71338	7.72539	5.03405	4.87054	5.47647	4.60272	5.87182	6.32324	9.2949
ZDHHC23	A0A1W2PRJ8	5.83121	6.08141	6.21241	6.40756	5.49211	5.45042	6.1372	7.0494	6.05698	5.8708	6.11061	6.99703	6.34783	6.43965	6.87732	6.63808
NKX2-4	Q9H2Z4	6.26004	6.88557	6.12458	6.92623	6.74048	7.40728	6.22961	5.99786	6.17039	6.03694	6.39705	5.92814	6.37151	5.40299	6.11763	5.00371
N4BP2L1	Q5TBK1	5.84256	6.40462	6.25837	6.24313	5.00558	7.14226	6.61791	7.12213	5.99469	6.51819	5.83938	7.52526	5.69379	5.16195	5.81214	6.81805
ZCCHC2	Q9C0B9	6.67949	6.55399	8.25142	6.37387	5.58731	6.80756	6.35925	6.4554	7.71097	7.34802	7.52819	7.10748	3.97014	4.70727	4.18373	4.37592
FUT4	P22083	6.74048	5.69598	6.15509	6.71707	6.1192	5.72211	6.43599	6.18308	5.79705	6.07287	5.89906	6.78653	6.83268	6.01093	7.2795	5.55238
CDC42SE2	Q9NRR3	5.93834	6.09426	6.48743	5.80595	5.6128	6.07812	5.71487	6.28137	6.07783	5.55636	6.25632	6.28826	6.26425	6.94556	7.05022	7.54806
ANKRD39	Q53RE8	5.81211	4.61864	6.592	4.51081	6.58446	5.72729	5.35577	7.70116	5.33948	7.15185	6.57882	7.89554	4.29412	7.33301	6.69227	7.81267
ZBED8	Q8IZ13	6.13275	6.5698	6.32333	5.67996	6.56589	6.82222	6.14207	5.9447	6.58301	6.34357	6.34545	7.0395	5.76071	6.47059	6.26774	5.90871
IFRD1	A0A6Q8PF03	7.49382	7.17321	6.49006	5.5714	4.80227	5.04826	5.95877	6.08696	5.17767	5.01174	5.26482	5.39161	7.36657	5.97231	8.13001	9.06054
SUFU	Q9UMX1	5.24386	6.36772	6.77573	6.27311	6.86523	7.4733	7.21502	5.57834	5.93863	7.70883	5.87028	8.66421	6.22084	5.08036	4.29271	4.43182
MFHAS1	Q9Y4C4	5.39803	7.04748	5.82905	6.07576	5.07885	6.85167	6.98441	6.46382	6.04519	5.67732	5.98615	5.81708	7.52079	6.49052	7.40627	5.3276
SVIP	Q8NHG7	3.66829	6.83679	6.17068	5.47697	4.78715	5.65921	5.98892	5.87169	5.08105	6.37598	4.55646	4.6071	7.47205	9.05687	8.20493	10.1859
FAM185A	Q8N0U4	7.08984	6.08518	6.39496	6.99306	6.09345	6.24221	6.44764	5.47571	5.07912	6.3611	4.87728	6.29155	6.3418	6.76106	6.84269	6.62335
DCHS1	Q96JQ0	6.64909	6.26294	4.27881	5.71579	6.58882	6.78168	6.01609	5.93947	7.55269	6.83935	4.88936	7.94679	8.1096	4.46921	4.59194	5.37041
SEMA4D	Q92854	6.59311	6.73363	5.99198	7.52573	6.37659	6.33515	5.93876	5.17578	6.59101	5.88989	5.56874	7.12844	6.62125	5.85782	4.73593	6.93618
CLPTM1L	G5E9Z2	6.32994	5.91096	5.22802	6.15491	5.75595	6.80174	6.4708	5.94311	6.56834	5.64964	6.41532	6.18511	6.19088	5.78595	7.43282	7.17652
LIN52	Q52LA3	4.96466	6.0373	5.49137	4.76839	5.40072	4.43423	4.71723	5.31389	4.46688	6.08721	5.71785	5.10842	5.8643	11.6553	7.48553	12.4868
ITGAE	P38570	7.28994	5.68072	5.85911	6.76306	6.10004	7.65764	4.98248	6.48473	6.14527	5.55852	6.79317	6.68725	8.02457	5.28282	5.41489	5.27579
SLC11A2	P49281-4	5.70649	5.93267	6.38851	6.66713	5.98683	6.03595	6.13256	5.59806	5.91626	6.53296	6.38358	6.55806	6.20892	6.27402	6.62386	7.05412
RNF111	Q6ZNA4-3	6.90866	5.48451	6.53556	6.08783	6.75217	7.26509	7.19965	5.21232	6.19878	5.70468	5.98626	7.04235	5.73807	6.51216	5.11732	6.25461
SLC25A44	Q96H78	7.38348	7.16128	5.79851	7.46529	6.00267	4.66263	6.37373	6.16771	5.60433	6.05353	5.43418	6.6342	5.99825	7.23411	6.23305	5.79306
LRRC45	Q96CN5	7.69702	6.59609	5.70455	5.34485	5.25178	5.96065	6.16097	7.57255	6.34456	6.55132	6.68164	7.72554	6.27106	5.73095	4.51898	5.88746
DYM	Q7RTS9	6.31659	5.73302	6.49567	5.77081	5.58871	6.5069	5.79972	5.8462	6.06039	6.09994	7.09326	7.14738	6.34542	6.9659	5.87287	6.35724
UQCC2	Q9BRT2	6.68577	5.93796	6.0988	6.42125	7.45525	6.71291	6.49195	6.58816	5.64901	6.14529	7.26066	6.30322	6.70822	5.81931	5.09561	4.62661
SLC10A7	Q0GE19-1	6.63825	5.48107	6.01638	7.23026	5.77403	7.09763	5.76915	5.86428	5.40927	6.96839	5.30861	6.03568	7.26075	5.77757	6.3247	7.04398
TAPBPL	Q9BX59-7	7.09191	4.44978	5.00076	4.94933	7.26603	5.68736	9.20868	5.35687	6.82613	7.18162	5.89484	4.89435	8.35777	7.24318	4.48267	6.1087
KLHL9	Q9P2J3	6.03085	6.3737	7.75534	6.83883	6.12081	7.34056	7.51796	5.82451	6.31488	6.90343	7.23614	6.63667	4.70223	5.11076	4.72686	4.56647
TYRO3	Q06418	6.55095	8.76356	8.73481	7.23809	5.98094	5.21367	5.90605	7.72034	8.46886	7.22617	7.33115	6.25583	2.96793	2.14736	4.21275	5.28154
HMCN2	Q8NDA2	6.83158	5.95877	5.32317	7.48925	7.07844	6.04958	7.90386	5.2551	7.72358	5.3789	4.80502	5.75539	5.70333	5.5911	6.56129	6.59163
S100PBP	Q96BU1	5.921	6.41838	6.63273	7.6364	6.34697	5.97077	6.58241	6.13245	5.80959	4.34401	7.02841	5.88732	7.00499	5.95076	5.93313	6.40068
SMAGP	Q0VAQ4	4.9726	5.15324	3.5813	5.60316	5.0979	5.39604	6.0626	6.99105	3.60949	4.99121	3.3749	6.00947	5.30172	11.8026	7.52586	14.5269

SLC30A6	Q6NXT4-2	6.1881	6.29766	5.05445	6.21778	6.60934	5.27805	6.49485	6.21533	6.62622	5.54712	5.64504	5.85662	6.67341	6.7161	7.49182	7.08812
HELQ	Q8TDG4	5.65246	7.33572	6.60234	5.34626	5.74423	6.0366	5.9133	6.98694	6.42709	6.05843	7.69894	6.96326	5.82158	5.49536	5.78535	6.13212
ACYP2	P14621	6.0353	5.53556	5.81942	5.55511	5.90385	7.24933	6.57395	6.52565	6.73671	7.53407	6.22854	6.9048	6.91935	6.53136	5.83423	4.11277
ENKD1	Q9H0I2	6.65808	6.88778	6.04589	6.57728	6.56696	6.82108	6.34827	6.36559	6.7276	6.72298	6.39458	7.12898	5.22031	5.20064	5.19768	5.13632
PAQR4	Q8N4S7	7.12736	6.61832	5.757	6.50917	6.43676	5.70896	8.01449	6.7693	5.38116	6.33953	7.90515	6.62027	5.77878	4.46753	5.52291	5.04331
PLLP	Q9Y342	5.47457	6.30983	5.61108	6.2747	6.03012	5.58041	5.54489	6.43724	8.54309	4.74051	5.05563	6.62293	6.58417	8.33438	6.0916	6.76487
CCDC171	Q6TFL3-4	6.35263	6.89164	6.315	6.59629	6.93372	5.79662	5.65724	6.65549	6.06131	5.87082	5.851	6.31019	6.07209	5.65542	5.81473	7.16582
IP6K2	Q9UHH9	7.21422	7.17577	8.22053	7.76411	6.2909	6.66516	6.5552	6.23265	8.00449	8.40359	9.01877	8.71848	2.1281	3.04669	1.84573	2.71562
RBM18	Q96H35	6.38229	6.7231	6.39897	6.60914	6.54739	6.28243	5.91462	6.1759	5.93517	6.30406	6.12936	6.34781	5.92223	5.48729	6.1367	6.70355
LRP10	Q7Z4F1	6.24281	6.62105	6.71705	7.76386	6.26841	7.03228	6.90106	6.27985	7.95878	6.88701	5.76493	6.26416	4.49639	4.84413	4.81932	5.13893
FBXO45	P0C2W1	6.16083	6.2087	6.37641	7.48725	5.95939	6.50058	5.5358	6.10342	6.50583	7.82025	5.48281	6.22945	6.58975	5.9902	5.65725	5.39206
LDHD	Q86WU2	6.64892	5.79969	5.68109	6.36254	6.60915	6.03922	6.06714	6.26893	5.78091	6.02143	6.28245	6.15162	6.89754	6.44067	6.51965	6.42904
TLR3	O15455	5.83753	5.65926	5.74976	7.53857	6.41087	6.47417	6.75878	6.10379	6.04933	6.40877	5.16053	6.51104	5.86801	6.29601	7.17204	6.00156
EIF4EBP2	Q13542	6.07649	4.86185	5.05232	4.65473	4.92374	3.81536	4.96298	5.53248	3.84327	4.62269	3.64672	5.55315	4.14505	15.3989	9.55125	13.359
DYNLT3	P51808	5.72424	5.83775	6.68207	5.248	6.2326	7.11747	6.89216	6.26196	6.73286	6.85873	6.60516	6.94456	6.10733	5.60224	4.86054	6.29235
DCLRE1A	Q6PJP8	7.48667	6.98332	7.98537	7.22708	7.8415	6.25877	4.73287	6.03138	8.48782	6.54002	5.90307	5.34411	4.70516	5.99338	4.22822	4.25126
NUDT12	Q9BQG2	6.82982	6.55398	6.29434	6.44818	6.43614	6.76164	6.15228	6.50863	6.50907	6.50176	6.95138	5.7674	5.79276	5.92664	5.47463	5.09134
SFR1	Q86XK3-2	5.07539	5.80638	5.93573	5.92719	6.47641	6.69948	6.24085	6.03183	6.01188	6.65039	6.00506	7.18184	5.84851	6.90618	7.07421	6.12866
NUDT18	Q6ZVK8	6.52162	5.30442	7.48324	6.70671	6.83877	5.88427	6.18607	5.90697	7.20921	6.42592	7.57917	3.43457	5.76703	6.67588	5.86102	6.21513
C14orf93	Q9H972	6.78014	6.69727	6.71393	7.51799	6.43859	6.35159	5.87962	6.32848	6.22696	6.56697	6.81543	6.2033	5.34848	5.29151	5.4417	5.39808
EPB41L4A	Q9HCS5	6.71731	6.92022	6.71283	5.797	5.28814	6.39396	5.2279	6.85602	6.56652	7.09297	5.94896	6.2782	7.08581	5.21122	6.0161	5.88684
TMEM183B	Q1AE95	6.83399	6.60702	6.15313	7.2911	6.48603	7.20278	6.1594	6.66669	9.62304	6.78883	7.85489	6.57628	3.26235	4.53498	3.78802	4.17146
UBXN11	Q5T124	6.85731	5.60633	7.38636	6.39372	6.94132	7.00343	6.02748	7.43877	6.19234	6.8153	8.2331	7.58515	5.06723	4.51376	3.91338	4.02501
EEF1AKMT1	Q8WVE0	5.86089	6.19137	6.479	5.55331	5.81404	7.0506	6.81221	6.39784	6.45612	6.29846	6.74979	7.37313	5.66682	5.72138	5.4598	6.11525
COMMD2	Q86X83	5.77681	5.93192	6.34216	5.31714	6.77536	6.27332	5.59676	5.83248	7.51231	5.08923	7.94371	5.02424	6.74427	7.51918	5.53071	6.79039
HMGXB3	Q12766	5.74093	6.28118	6.73436	7.28988	5.9006	6.50464	6.32429	6.21197	6.37522	6.94113	6.75768	7.05111	5.39727	5.41464	5.52156	5.55352
VPS37C	A5D8V6	6.91963	4.4206	5.84106	6.0481	7.23289	3.61705	3.37651	8.57262	5.00022	5.10773	4.55397	6.39736	5.06847	10.299	9.12802	8.41679
RCAN3	Q9UKA8	6.95374	7.00083	7.01407	6.31778	6.57448	7.23296	6.22792	6.59532	6.84786	6.65575	6.9972	6.96843	4.07016	5.11844	5.02623	4.39882
PRDM4	Q9UKN5	5.52286	7.04973	5.21681	6.3497	6.4329	5.62672	7.52981	5.09823	6.11119	6.87152	6.45604	5.55656	6.32855	5.62713	5.96345	8.25881
KLHDC10	Q6PID8	6.42011	6.66918	7.8142	6.19288	6.45506	6.66898	6.45331	5.9004	5.85001	6.8563	5.69454	6.6272	6.0064	6.10451	5.14777	5.13917
B3GNT5	Q9BYG0	7.10272	7.0462	6.50404	7.6773	6.62324	7.21463	6.76069	5.88248	5.62832	6.00087	5.84116	6.5054	5.11883	5.59114	4.95408	5.5489
SH3GLB1	Q9Y371-2	6.46702	6.57255	6.63643	6.01277	6.1725	6.83862	6.59116	6.76483	6.20798	6.53317	6.69439	6.84432	5.66735	5.34854	5.13701	5.51136
ZNF562	Q6V9R5	6.9183	7.01655	6.69099	7.2401	6.33127	5.57118	6.15232	5.97756	6.27482	6.20854	7.20923	6.33216	5.51012	5.37275	5.69495	5.49916
CAMKK1	Q8N5S9-2	6.76901	6.39077	6.38418	6.19306	6.39655	6.2784	6.2996	6.77967	6.26243	6.2837	6.36038	6.18047	5.74518	6.44551	5.86435	5.36675
AZI2	Q9H6S1	6.65828	6.87811	8.54155	5.14797	5.88131	5.37596	3.9971	6.05482	5.79635	6.21715	8.18604	7.6523	6.62855	6.78088	4.56207	5.64156
SLC39A3	Q9BRY0	6.54215	6.30522	7.29837	7.55171	3.81576	5.17829	5.13854	5.83805	6.52197	4.88848	8.13589	7.25832	5.04678	6.37327	6.99224	7.11497
SLC35D2	Q76EJ3	6.968	6.59816	6.228	6.62147	6.04581	5.65507	6.19991	5.57609	6.10538	6.43769	5.89143	6.27607	6.24776	5.82179	6.33372	6.99366
ZBTB7B	O15156-2	7.17288	6.1905	5.94176	6.87559	6.05367	5.81332	5.86504	5.22745	6.99925	6.76862	6.44212	5.99439	5.62444	5.43704	6.50638	7.08755
ZFP90	Q8TF47	6.26505	7.69877	5.84247	5.90482	5.06615	5.51526	5.92795	6.28547	5.52879	6.41497	6.38572	6.86283	6.96534	7.05719	6.92218	5.35702
AP4S1	Q9Y587	6.34185	6.74184	6.92556	6.31508	6.194	6.43157	6.46447	6.6119	6.20391	6.7786	6.42959	6.22872	5.88362	5.82828	5.15421	5.46679
TPGS2	Q68CL5-5	7.34697	6.91827	7.02656	6.70865	5.86912	7.25789	6.49369	6.05228	6.72054	6.73724	7.37405	7.41725	4.59489	5.00736	3.93355	4.54169
IFT22	Q9H7X7	5.89707	4.16413	4.05391	3.95764	4.12129	5.08789	4.97515	7.93344	5.77023	7.32209	4.9702	6.87165	4.28693	10.0913	8.89421	11.6029
IGSF9B	Q9UPX0-2	5.46969	6.10196	5.90283	6.3469	6.52757	5.40869	5.14863	5.73803	5.88331	6.61784	5.22105	5.66947	6.39029	7.44215	8.52378	7.60781
SIMC1	Q8NDZ2-5	6.54478	6.7525	6.61251	7.07585	6.15286	6.28016	6.63322	6.75241	6.81329	6.11012	6.10526	6.27634	5.65887	5.40459	5.24382	5.58342
CREM	Q03060-1	6.29377	6.30355	6.21852	6.51827	7.12591	5.75275	6.10401	6.4165	5.59641	6.49681	7.54506	5.73359	6.24255	5.96378	6.1294	5.55915
ATF1	P18846	5.79071	6.06562	7.7926	6.81317	5.10956	5.29078	4.67507	3.47068	6.95816	6.46811	6.19022	10.0587	7.13788	6.18496	5.6323	6.36145
HOXD9	P28356	5.82993	6.32007	5.83129	5.85863	5.89704	5.91888	6.21288	6.35024	5.7795	6.95137	5.38328	5.61533	6.07303	6.64486	8.04599	7.28767
PKP3	Q9Y446-2	5.5213	8.36485	4.18839	5.45672	5.90709	5.75714	5.72629	7.26435	6.67223	6.60877	6.21904	5.79835	7.06676	6.95442	6.58529	5.909
PGPEP1	M0R3H3	6.14806	5.91004	5.30003	6.2701	5.91977	5.15695	5.23697	6.75518	6.63568	5.89931	6.06346	6.27603	6.85388	7.11741	6.6671	7.79004
HACD2	Q6Y1H2	6.04308	6.26389	5.5269	6.93449	6.25223	5.93614	6.22414	6.04374	5.77493	6.55046	6.24603	5.8747	6.42259	6.53979	6.7342	6.63269
KIAA0930	Q6ICG6-2	6.1345	6.72948	7.16113	6.40856	6.36656	7.75812	6.69207	5.3544	6.81195	5.83452	7.3884	7.84239	4.92843	4.24194	5.50209	4.84546
DNAJB13	P59910	7.12404	7.21592	5.58062	6.51118	5.97388	5.22828	6.08417	6.02269	7.40056	5.74914	5.69052	6.32783	6.05377	4.77437	8.11086	6.15219
RNF170	Q96K19	5.77733	7.48961	8.79778	4.4492	5.96106	6.06948	7.81179	7.35863	6.72405	3.786	6.24507	6.73456	6.28086	4.14561	7.86183	4.50715
NELL2	Q99435-3	6.74792	6.60766	5.9751	6.88429	6.38611	6.65848	5.65246	5.8169	6.93326	6.4206	6.25034	6.33441	5.88595	6.01432	5.8392	5.593
OTUD3	Q5T2D3	7.11775	7.10979	6.03384	6.20489	6.46773	6.20322	6.07243	7.59507	6.43813	7.74993	6.17549	6.35417	5.1406	5.2892	5.08769	4.96007
CYP24A1	Q07973	5.57536	6.78251	4.53111	6.6183	5.82497	5.4471	5.38091	6.07325	6.33726	6.28318	5.38496	6.83317	6.38136	6.3035	8.68169	7.56138
GPR180	Q86V85	6.95651	6.91657	5.88233	6.4643	5.96281	6.25368	6.48826	5.99016	6.06673	6.31504	5.25297	4.92136	6.92426	5.96796	7.27853	6.35855
ALG14	Q96F25	6.56508	5.63765	5.93361	7.34413	5.15325	5.29389	5.52392	5.73677	6.08982	6.87238	6.83515	6.61805	6.33507	6.32851	6.8115	6.92122
C16orf87	Q6PH81	6.02346	6.34598	6.08651	6.81963	5.87837	6.09347	6.62151	5.81222	6.36409	6.68889	6.33091	6.64265	6.17283	5.5692	6.21454	6.33576
DPPA2	Q7Z7J5	6.37653	7.14058	5.03791	7.12744	6.06338	6.64947	6.17314	6.07029	6.4598	7.29714	6.48324	6.34014	5.42299	6.32951	5.71513	5.31332
CHMP6	I3L4G8	7.2359	5.97038	5.39741	6.07356	5.21977	5.7863	6.56255	5.6762	6.53347	5.986	6.36899	6.38463	7.07576	5.34215	8.3623	6.02461
STK11	Q15831	5.42683	6.57679	6.51409	7.43891	6.42133	6.06269	5.68714	7.41016	6.90275	6.58495	5.85156	5.93689	5.17833	5.89776	6.10267	6.00715
NHSL1	H0YDF6	7.72172	5.71739	7.66542	7.23271	4.52923	7.22545	6.02345	4.81549	8.20737	6.79841	5.52507	5.44559	7.45248	4.25584	4.14054	7.24384
NIPAL2	Q9H841	6.76945	6.6153	6.75759	6.94169	4.66528	6.32321	6.67259	6.45862	6.05392	6.54116	7.81296	5.98857	5.86945	5.42628	6.11915	4.98477
LGALSL	Q3ZCW2	5.07344	5.94701	6.31392	6.00597	6.21634	5.53086	6.26197	6.492	4.98905	6.377	5.20418	6.8979	5.22984	8.56923	6.31141	8.57986
SYPL2	Q5VXT5	5.24938	6.15408	5.65538	6.28832	4.70086	6.74776	7.46858	7.01501	5.58036	5.99456	6.30123	6.24945	6.57092	6.69996	6.98687	6.33729
PPM1K	Q8N3J5	6.48966	6.9176	7.535	6.78787	4.71497	5.41431	5.49825	5.67065	7.33339	6.65523	5.78644	7.55612	5.16217	5.30314	6.74478	6.4304
RP1	P56715	6.82686	6.13567	5.50222	5.96435	6.11141	7.04351	7.19062	5.33149	5.16312	4.6563	6.3883	5.57049	7.81068	6.56612	6.45279	7.28607
CRPPA	A4D126	6.02469	7.12547	7.54349	5.31612	5.37868	6.39983	5.81245	6.15861	5.91475	6.25129	5.50302	7.3885	6.73697	5.90331	5.85368	6.68912
KRTCAP2	Q8N6L1-2	5.41107	6.1044	6.23634	7.0984	6.20524	5.91401	6.39313	6.01981	5.9503	5.71647	5.26325	6.13556	6.59004	7.00498	7.49585	6.46112
POLR3GL	Q9BT43	5.77026	6.51981	6.74568	5.43703	5.80732	6.95025	6.3311	5.57147	6.58683	6.79055	6.80932	5.72904	7.15431	7.20624	5.23495	5.35585
RFESD	Q8TAC1-2	5.65509	6.71546	7.46434	5.40493	7.18799	6.56849	6.4909	8.20689	7.55456	4.95428	7.6774	5.89972	5.42793	4.19792	6.1745	4.41961
GTF2A2	P52657	5.67965	5.46929	5.58554	4.67707	6.5417	6.6859	5.86204	5.73981	5.9357	5.6769	5.65461	6.07461	7.18942	8.03879	7.68934	7.49964
TNKS2	Q9H2K2	6.39176	7.16968	6.53306	7.22203	7.20209	6.92633	7.27665	6.12908	5.75781	6.08815	6.54585	6.3182	5.20806	5.79202	4.61513	4.8241
PSMA3	H0YJ03	5.75121	6.04309	6.88782	5.9575	7.41408	8.75806	4.2097	7.70406	7.69244	6.16165	5.82631	5.87558	8.31997	4.17913	4.57	4.64941
SP4	Q02446	6.34532	5.47476	7.32265	7.32524	5.10586	6.09858	6.32613	5.53543	5.77317	5.92609	6.0452	5.79676	6.82845	6.1294	7.13379	6.83317
CYBA	P13498	5.73744	6.9536	5.50371	6.61292	5.70146	6.22603	6.06825	6.75587	6.87595	7.72927	6.30615	7.13837	4.30099	5.74799	4.97129	7.37071
B4GALT4	O60513	6.70791	5.73864	6.6653	5.61071	6.34531	8.06193	6.99929	4.27598	5.8501	7.39214	5.96222	5.08772	6.63796	5.79622	5.99781	6.87074
SERPINF1	P36955	6.44609	6.33671	4.76241	6.27983	4.91177	6.99632	7.26613	4.52151	7.29313	5.40332	5.99871	5.20046	6.58858	5.73996	9.24011	7.01498
LRFN3	Q9BTN0	6.39644	7.38286	6.4884	7.23692	4.77014	6.78441	6.20366	4.85553	8.11485	6.31222	6.65169	5.43501	5.41385	5.14915	6.72119	6.0837
TRMT2B	Q96GJ1	7.71832	5.94511	5.56471	7.39725	6.82295	6.86347	5.77378	5.7371	6.55251	5.85563	6.34307	4.91244	5.33598	6.83493	6.12471	6.21804
C8orf76	Q96K31	7.75968	7.28999	7.36188	4.81553	6.80823	6.87512	6.7141	7.06626	5.42179	6.68653	6.95929	8.16007	4.81249	4.56439	4.32702	4.37763
CACFD1	Q9UGQ2	6.87918	6.49161	6.32324	6.14517	6.76253	6.60788	6.20939	5.70317	5.73262	6.03163	5.11308	6.61297	6.66582	6.68997	6.30724	5.72449
DSEL	Q8IZU8	6.2843	6.90493	7.42819	6.77511	7.30948	5.72328	6.48966	6.6247	4.88433	6.45857	6.87864	6.06875	6.1756	4.20898	5.13375	6.65173
MZT2B	Q6NZ67	5.78041	5.20391	6.55202	5.19181	5.71829	6.85206	7.77997	7.86873	6.21775	8.28197	6.16438	6.84568	6.28237	4.78563	5.51431	4.96073
MINDY1	Q8N5J2-3	5.44614	6.14015	6.92746	5.92598	5.76598	6.2962	5.70148	5.88571	5.85704	6.81079	8.22527	7.18304	5.82044	5.9387	5.11543	6.96019
CTNS	O60931-2	6.06917	6.82073	5.06357	5.52764	5.64495	5.47197	6.20941	6.95882	5.91781	6.87173	5.59579	7.14434	6.48805	6.10602	7.3775	6.73249
TBCCD1	Q9NVR7	5.58745	5.48759	6.88379	7.23029	6.42952	6.65365	6.70093	6.6513	6.72405	6.44916	6.78017	7.22628	5.16804	5.24067	5.25828	5.52883
MFSD3	Q96ES6	5.30083	6.00929	6.06151	6.25112	5.63383	5.73706	7.07897	6.96485	6.99256	6.86579	5.79491	5.15381	6.19083	6.83065	6.71277	6.42121
DESI2	Q9BSY9	6.17364	5.74666	5.03802	4.63232	6.61407	5.71269	5.77776	5.81599	7.21063	5.18716	6.07797	6.67586	5.85486	7.74797	7.16554	8.56887
ENOX2	Q16206	6.54316	5.5689	6.81667	5.64337	6.14978	6.20786	6.98847	5.84692	6.37552	6.25398	6.70238	5.72553	6.30003	5.91172	7.00568	5.96002
TNFAIP8	O95379-4	5.79159	4.93128	7.93149	5.62778	6.1849	7.55157	5.96187	5.53403	6.53709	6.49805	6.73279	5.78401	6.34225	6.57477	5.16934	6.8472
CCDC154	A6NI56	4.64194	5.83056	7.45492	5.81627	7.49796	8.47086	6.0984	5.84706	6.38782	6.58797	7.89682	5.95158	6.06039	4.66798	5.22128	5.5682
ZNF229	Q9UJW7	6.56667	7.31578	4.99416	6.194	5.72163	5.43615	5.02776	7.54552	7.51137	7.51196	5.79451	6.78726	5.63536	5.5892	6.13262	6.23605
TCTN1	Q2MV58-2	7.35007	7.07429	6.38105	7.03348	6.20582	6.16804	6.32411	5.94097	6.18524	7.38438	6.35094	6.64873	5.49064	5.22729	5.05389	5.18106
KCT2	Q8NC54	6.11903	6.83875	5.44318	6.05748	5.74959	6.33673	5.44668	6.96998	5.85516	7.17328	5.79663	7.48168	6.47328	6.1526	5.64542	6.46053
RHBDD2	Q6NTF9-2	6.34774	6.49515	5.93868	6.95027	5.82139	5.48364	6.89777	7.00969	6.69542	6.11233	5.56431	5.17162	6.19091	6.30219	5.97881	7.04008
SOCS2	O14508	6.48903	5.10687	6.97049	5.93264	7.14982	7.05227	5.72994	6.16571	6.98156	7.29619	7.08569	7.27376	5.51884	4.70336	5.55695	4.98689
MED21	Q13503	4.94926	6.03583	5.01398	6.24967	7.27753	4.70462	4.506	5.72429	6.18054	4.80059	5.528	3.65985	4.29842	10.3038	6.35175	14.4158
TBC1D12	O60347	7.06611	6.56354	6.56649	6.17415	5.61662	6.95467	6.44855	5.8443	6.54662	5.94038	6.09665	5.97971	6.76273	5.95341	5.09758	6.3885
MGMT	P16455	6.54118	4.04332	5.05201	6.21106	5.53751	5.10211	6.40918	6.49453	5.33996	6.18774	6.27946	7.10473	5.43941	9.50874	8.8225	5.92656
MED20	Q9H944	6.16988	6.40507	7.93766	4.56172	6.49874	5.80844	5.82079	5.55988	5.66885	5.50285	7.17043	5.48791	7.58666	6.84989	6.01184	6.95939
ZNF394	Q53GI3	6.27331	6.85807	7.98443	5.77607	6.37705	5.68264	6.36527	5.6157	6.27145	5.62346	5.02997	5.47256	5.98789	6.21799	7.30291	7.16125
ANKRD46	Q86W74-1	7.47077	5.39139	6.91333	5.37607	6.83248	5.58392	7.56793	6.3919	6.36582	6.40289	6.56095	6.28346	5.26978	5.42266	5.85905	6.30761
RNF19A	Q9NV58	7.51002	6.41123	7.11069	7.33187	4.95697	5.38004	6.27608	5.63493	6.13086	6.07982	6.60588	7.58524	6.79977	4.48687	6.70459	4.99513

RALGPS2	Q86X27	6.75541	6.97717	6.81079	5.7067	6.25108	8.21425	7.13083	5.47519	6.15697	7.28107	5.97933	5.18885	5.69024	5.73066	5.29488	5.35657
FAM217B	Q9NTX9	6.00494	8.46703	7.63002	6.83119	6.21488	5.11268	5.23011	6.68928	6.97282	7.95754	7.56774	7.86517	3.89065	5.72081	3.36189	4.48326
GXYLT2	A0PJZ3	6.08028	6.28548	6.10538	6.85732	6.22567	6.03562	6.15101	6.02885	6.207	6.68026	6.37455	6.37264	6.3565	6.05753	6.13588	6.04605
C19orf12	Q9NSK7	6.02902	4.96991	6.49482	5.35862	6.12287	6.46581	5.98161	6.9828	6.08425	6.67421	6.12989	6.59259	5.76522	7.25423	6.52692	6.5672
MUC15	Q8N387	6.86475	5.79003	6.60927	5.57906	5.92991	6.38256	5.98832	6.15138	6.17357	6.39675	6.42017	7.0464	6.33023	6.21264	6.5859	5.53906
MLXIP	Q9HAP2	5.99553	6.45501	7.67213	6.03863	6.03192	6.63924	6.27568	6.27895	7.264	6.58355	6.61665	6.7072	4.89523	5.16542	5.51913	5.86173
ZNF736	B4DX44	5.74864	7.21478	6.69052	5.88737	5.53629	7.24249	7.04153	5.27889	5.24627	7.62507	7.09324	7.01631	5.93174	5.27023	4.97206	6.20459
FAM241B	Q96D05-2	5.88078	5.005	4.64234	4.54017	4.58968	3.93135	4.69277	6.35462	2.98672	5.00948	5.56653	4.66143	5.54952	12.9366	11.2112	12.4419
ZG16B	Q96DA0	1.52909	5.383	0	20.1748	3.40063	8.36336	8.79426	2.41076	2.67717	2.36978	2.76281	2.46121	4.56238	26.6321	2.73229	5.74643
TMEM138	Q9NPI0	6.33743	6.96315	5.58938	6.79634	6.76973	6.81622	6.44243	6.3091	5.84631	6.9617	6.26669	5.88469	4.97751	6.36376	5.747	5.92859
MIEF2	Q96C03-3	7.61576	8.5187	7.77543	4.59194	8.75738	5.7319	7.72758	6.86877	6.79023	3.05983	7.88232	4.88644	5.64059	5.24793	4.64657	4.25865
FBXO46	Q6PJ61	4.93482	6.87091	6.1361	6.13445	5.83175	6.20064	6.54333	5.05773	6.86951	7.13031	5.63924	6.44484	6.57086	6.59379	6.95173	6.08998
C1orf167	Q5SNV9-2	6.12835	5.97116	5.44405	6.12695	6.06857	5.37807	6.92633	6.60074	6.40389	5.60363	5.25097	5.51009	6.73172	6.62908	7.62456	7.60185
UBE2F	Q969M7-2	6.15509	4.48489	5.98945	7.76266	6.70044	5.72085	5.75969	6.09523	5.28854	7.02122	7.02263	5.64918	5.87551	6.92228	5.89667	7.65567
FHOD3	Q2V2M9-4	5.80455	6.3723	6.10407	5.7732	6.15822	6.5333	6.12473	6.2383	6.728	7.1326	6.87698	6.83153	6.28985	5.89416	5.44016	5.69803
MREG	Q8N565-2	6.49256	6.41083	6.13528	6.3373	5.663	5.70677	6.50799	6.83293	6.19666	6.63542	6.32581	7.02405	5.75857	5.45636	6.39926	6.11721
NFIL3	Q16649	6.20268	6.17063	6.15133	6.81775	6.30746	5.80452	6.84457	6.20142	6.7598	5.71061	6.43822	6.47796	6.40277	5.63592	6.21757	5.85679
YOD1	Q5VVQ6	5.94299	6.68662	6.62757	7.35664	6.05091	6.96484	5.96694	6.20798	6.88769	6.06881	7.34683	6.87088	5.80679	5.72553	4.63552	4.85346
RIN2	Q8WYP3-2	5.78065	6.30278	6.22459	5.62151	6.12002	7.21293	5.94812	6.93346	6.82974	6.60346	6.22891	6.55886	6.16952	6.03948	5.71633	5.70964
UQCR11	O14957	5.40396	6.11822	5.35886	5.69375	6.20849	6.15127	7.09572	5.9291	5.43802	6.1813	6.15994	6.04898	6.29036	7.3841	7.53605	7.00188
SCOC	Q9UIL1	6.82265	5.20498	3.44405	5.67975	5.31707	5.691	5.42485	6.66648	5.17328	6.29873	5.22835	5.10608	4.14553	10.5703	8.69139	10.5355
CTRB2	Q6GPI1	6.53921	5.28561	5.88409	6.04395	6.66006	5.64044	6.4258	6.07778	6.62141	4.97356	4.38983	5.29513	6.1913	7.23495	8.24219	8.49469
PIGW	Q7Z7B1	5.76614	6.97095	5.67684	6.59221	5.17133	5.63556	6.40324	5.95997	7.22026	5.70721	6.12235	5.97633	6.79724	6.235	5.95343	7.81194
POLD4	Q9HCU8	5.45235	4.97462	5.2743	4.67603	5.96171	5.37285	5.83346	6.58185	4.93307	6.49761	7.33909	5.53322	6.92322	7.97816	8.83828	7.83016
MTHFD2L	Q9H903	6.41898	6.64056	5.58122	6.4722	6.27087	6.44123	6.00842	5.20694	6.75814	6.29785	5.7479	6.57295	6.68652	6.26382	6.04505	6.58734
GPS2	Q13227	7.19707	6.17819	7.22561	5.34935	5.93431	5.86249	5.97207	6.25533	5.96199	6.81615	6.68956	6.29727	7.336	5.36154	5.31109	6.25199
ADRB2	P07550	4.69902	6.1099	5.86907	7.71927	5.14832	6.60561	6.70237	5.93204	6.60612	4.23068	5.58516	6.39264	7.25755	6.36561	8.11105	6.66559
ZNF225	Q9UK10	6.92136	5.91104	6.60636	7.1834	6.29592	6.12149	6.65599	6.60551	7.56624	6.71177	6.36314	6.17944	5.22481	5.50137	5.29365	4.8585
ADGRV1	Q8WXG9	6.19313	5.04843	6.46008	7.45727	5.94325	5.52949	8.102	6.25894	6.23709	6.27702	6.19952	4.10206	7.45018	5.61334	7.12033	6.00786
CKLF	Q9UBR5	4.9263	6.24766	6.40279	7.46965	6.94393	6.77586	6.72455	6.68579	5.77046	6.08522	5.37286	5.30456	5.35493	6.69241	6.82372	6.41932
PET117	Q6UWS5	5.99937	5.65853	5.47808	6.30078	6.1244	5.12917	5.71181	6.41748	5.54582	6.38924	5.39726	6.56184	5.98027	7.60517	7.67997	8.02081
PPM1M	Q96MI6	6.52964	6.4113	6.52573	5.71655	5.09856	6.32462	6.9144	8.07103	8.53059	6.33126	8.71605	8.64225	3.67873	5.29377	3.31576	3.89976
ZMYND19	Q96E35	6.36105	7.51916	7.47075	7.06963	6.41966	5.5103	7.05725	7.93185	6.11323	6.9652	7.03572	8.42786	4.51982	2.90219	4.01066	4.68566
ZBTB45	Q96K62	5.9334	5.91956	6.40999	6.92838	5.34134	5.88407	5.95545	6.27652	6.58021	6.43802	6.50632	5.97534	6.45415	6.52123	6.67606	6.19996
ABTB2	Q8N961	5.07222	6.77204	6.94618	5.77257	4.34702	7.53883	6.2731	4.25541	5.87143	6.89338	6.26381	6.11514	6.25451	6.66165	6.34715	8.61556
ZCCHC10	Q8TBK6	5.9086	6.5689	5.70587	7.13541	6.56055	6.5356	6.50669	6.36328	6.3338	5.62814	6.13649	6.19018	5.96364	6.36663	6.07434	6.02188
NUDT6	P53370	7.76893	6.52327	6.21184	7.84838	5.66985	5.3117	4.77353	5.42941	5.60646	7.99693	6.18526	6.36432	6.84605	5.74624	5.99463	5.7232
PIGQ	Q9BRB3-2	6.93682	6.24302	6.80692	7.05582	6.86916	5.37608	5.47283	6.8182	4.98811	5.9398	5.75529	6.08856	5.9811	6.83704	7.09434	5.7369
EXTL2	Q9UBQ6	6.15048	6.28149	5.2689	6.33922	6.4818	6.0686	7.08748	6.62202	6.26079	5.76717	5.95024	6.22056	6.03931	6.41634	6.08196	6.96365
PARP8	Q8N3A8	5.42883	6.12626	6.77907	6.29219	5.54696	5.93541	5.86301	6.24056	6.55053	6.85496	6.88499	5.71899	7.18203	6.26313	5.3911	6.94198
PRELID3B	Q9Y3B1	5.71527	6.78297	7.11709	6.99682	8.35906	7.05654	7.8337	9.27049	7.22073	6.70723	5.31772	6.1422	4.27703	4.48866	1.12261	5.59189
SLC35D1	Q9NTN3	7.95973	4.8901	5.31063	7.19301	6.99752	5.14477	5.12379	7.07241	4.66627	6.06814	6.07465	5.18559	6.42311	5.62691	9.40143	6.86194
ZNF839	A8K0R7-5	5.80535	6.30497	5.80905	6.29505	6.15814	6.42283	6.24258	6.77627	5.91476	6.07965	7.02038	6.58489	5.9705	5.90507	6.02939	6.68114
B3GALNT2	Q8NCR0	7.55634	6.91329	5.8885	6.06024	6.07469	6.64748	6.42108	5.67298	6.30706	6.34024	6.40658	6.06278	5.62351	6.4123	5.52994	6.08299
COMMD8	Q9NX08	6.24204	5.74639	5.25439	5.34892	5.22966	6.01127	5.54497	6.36012	5.33108	5.81213	5.55385	5.722	5.46769	9.88639	7.12842	9.36068
SHF	Q7M4L6	4.26846	7.84522	6.47152	6.19442	5.02012	3.42164	5.40985	8.74063	5.12898	8.89977	7.04867	7.71383	6.80978	6.32635	5.27361	5.42716
RAB40C	Q96S21	6.73584	6.1053	6.63762	6.64174	6.47036	5.33375	6.21254	6.50204	5.984	5.79475	6.05717	6.04924	5.3927	6.73609	6.53867	6.8082
GDPGP1	Q6ZNW5	6.34717	6.21369	8.0643	6.13324	6.05368	4.65343	7.11535	7.0472	6.0159	4.9969	4.81192	6.3793	8.27458	5.081	5.21364	7.59869
DCAF5	Q96JK2	6.35075	6.6129	7.37726	7.11288	6.13062	6.32167	6.10195	6.27835	7.69858	7.31071	6.83537	6.74103	4.24111	5.21171	4.59662	5.07849
ZNHIT1	O43257	6.56138	6.83343	6.83171	6.59588	6.38383	6.40211	6.58697	6.97378	6.52667	6.86188	7.18261	5.91897	5.20031	4.3456	5.83405	4.96081
SLCO2A1	Q92959	6.11723	6.23095	6.29523	6.97342	6.44514	5.46555	6.49867	5.74481	6.93716	5.80298	5.64253	7.12851	6.38564	5.98257	6.20546	6.14415
RASSF7	Q02833	5.76584	5.55611	6.69087	4.91099	6.65832	6.76535	6.23107	6.1305	5.6579	6.62724	6.96159	7.48074	5.56661	6.41457	6.45951	6.12277
DACH1	Q9UI36	6.4925	6.24581	5.80972	6.37993	6.50684	5.87297	6.48852	5.98589	6.874	6.75463	6.07656	5.7969	6.78691	6.13266	6.40735	5.38882
SPRTN	Q9H040	6.01644	5.50659	6.55519	7.3855	5.33792	5.6823	8.78751	6.52055	5.43815	5.40598	6.98785	5.55965	6.61829	5.99464	5.8544	6.34903
EPOP	A6NHQ4	6.49193	6.61253	5.95754	6.83772	6.36963	6.50582	6.47056	5.59823	6.99111	8.10734	5.49621	6.71811	6.42598	4.55856	5.67905	5.17969
ENPP4	Q9Y6X5	6.08288	7.42863	6.32416	5.79404	5.87123	7.441	5.33116	5.90561	4.97598	5.79231	7.28825	8.02777	5.55075	6.00157	5.42325	6.7614
MRPL52	Q86TS9	6.1048	5.40911	5.04812	6.00518	8.08495	5.56917	5.46377	7.61051	4.79816	8.07669	5.64749	8.11909	6.64647	6.06539	6.09035	5.26073
DHRSX	Q8N5I4	7.12208	5.76446	6.0005	8.50912	7.59958	4.90447	6.57506	7.94275	2.88244	7.20034	7.32549	4.54828	6.80888	5.92752	5.42708	5.46196
PPP1R16A	Q96I34	6.53232	7.77911	6.05798	5.60736	6.23977	6.2084	5.79607	8.47561	6.30961	5.33511	5.48818	5.42362	6.52682	5.95965	7.12348	5.13691
MRPL18	Q9H0U6	5.38271	5.99863	5.65116	5.97448	7.35429	8.15795	6.67714	5.61532	7.49502	5.94771	4.93137	6.22461	6.67251	5.54301	6.58114	5.79294
SLC50A1	Q9BRV3	4.9948	7.21459	5.8388	6.62795	6.63447	4.20525	4.23323	6.36068	7.2003	8.12065	5.1178	6.52697	6.90989	5.97413	8.46269	5.57779
G0S2	P27469	3.80017	5.64642	6.71819	6.2582	5.00773	6.16506	5.17925	6.59957	6.62751	5.07296	6.82773	8.08071	5.79514	7.81423	6.25911	8.14801
SLC23A2	Q9UGH3	6.70681	7.29656	6.00778	6.21391	6.20535	5.92293	6.4432	6.25988	6.42516	6.05588	6.11558	5.42877	6.47439	5.79156	6.29112	6.26112
TMEM35B	Q8NCS4	5.56726	5.47523	5.01029	6.94702	4.74446	5.54563	6.66805	3.96274	4.83717	6.45318	4.4568	8.10543	8.86388	8.21964	7.46294	7.68029
ZUP1	Q96AP4	7.00715	7.24767	8.46658	6.75364	6.85502	7.16994	7.01745	7.27671	6.1733	6.47543	7.67712	7.89702	2.91578	3.57764	4.18575	3.3038
LPAR2	Q9HBW0	7.16914	5.35792	6.20821	5.20207	6.53799	7.58604	6.09388	6.96398	5.93024	6.54987	5.8856	5.89066	6.29206	5.62229	6.08078	6.62928
SYTL1	Q8IYJ3	6.54258	5.84022	7.29256	6.77651	4.87103	7.47627	5.73265	4.22055	4.99098	7.62986	4.88731	6.54054	7.3587	5.67855	6.95463	7.20704
DOCK8-AS1	Q5T8R8	7.04861	6.89849	6.62742	6.4048	6.51215	6.06344	6.26676	7.02132	5.84358	5.50806	6.6684	6.92487	4.93086	5.51653	6.00829	5.7564
C8orf88	P0DMB2	6.78863	7.6764	8.1938	6.61994	7.19155	6.68233	6.53212	6.9757	6.87698	6.69232	6.35838	8.17661	3.06032	3.31636	3.84425	3.61431
BNIP3L	O60238	9.54536	9.24723	5.31404	7.99385	4.80712	3.49096	4.97072	5.30977	8.01303	8.5	7.57826	9.72605	2.73245	5.11162	3.24671	4.41282
FBXL17	Q9UF56	6.1247	6.62314	6.87199	6.97586	4.54644	5.48937	6.19871	6.28074	5.47411	6.30379	5.70971	6.34364	5.89666	7.94374	6.71932	6.49807
CCDC34	Q96HJ3	6.01565	7.07651	6.74918	7.00008	6.62548	6.171	5.61436	6.69617	7.65333	7.09951	6.70058	6.94594	5.29975	5.01912	4.75041	4.58294
UBE2A	P49459	5.77081	6.06927	6.10541	4.91314	6.13838	5.38796	5.30556	7.8682	5.84146	6.98524	5.50313	6.22882	5.51914	7.82339	7.34768	7.19239
AMIGO1	Q86WK6	7.43845	7.52254	7.2374	6.46629	7.88208	6.76452	4.78521	7.61535	6.13114	3.47891	7.33281	5.9939	6.89293	4.90559	4.02139	5.53149
ADGRD1	Q6QNK2-4	7.11516	4.84413	6.09603	6.84436	7.02733	6.68556	5.45437	7.47921	5.76036	4.26658	5.94762	5.88348	6.05322	6.74132	7.55139	6.24989
CLUAP1	I3L2E1	4.20834	5.23446	7.01612	6.16077	7.09911	9.15975	5.96606	5.86314	7.13341	5.94485	6.8596	6.11119	7.42925	5.55029	5.68396	4.57969
PPARD	Q03181	3.19345	5.58436	5.19805	5.71456	4.19254	6.16992	6.68824	7.08887	7.08239	6.9294	7.05729	6.79887	8.1757	4.99561	7.88041	7.25036
LEPROTL1	O95214-2	6.89166	7.0787	6.48065	6.24549	5.73943	6.2018	7.39738	5.72367	7.39233	6.87071	7.39281	6.03055	5.07145	5.3633	4.91655	5.20351
SLC16A7	O60669	7.17835	6.9195	6.97219	5.90989	6.03567	6.23248	6.26617	5.88134	7.5293	5.96754	6.41957	5.33656	6.16163	6.06518	4.4684	6.65622
HDDC3	Q8N4P3	6.06437	4.67151	7.33254	3.9884	7.01019	5.29045	5.17263	5.8217	5.92366	7.2233	7.06292	6.59351	6.75277	7.74548	7.42806	5.91853
BCL2L12	Q9HB09	4.85621	7.09388	6.63255	4.86189	4.48852	3.71567	4.54325	7.22668	4.79562	3.49832	6.32815	8.05209	5.71162	9.29808	10.9054	7.99203
AKTIP	Q9H8T0-2	6.37216	6.61417	7.00555	5.83913	6.82943	7.32414	6.10045	6.02207	6.06236	6.26563	5.8993	6.18361	5.49435	6.41925	5.65397	5.91443
C6orf136	Q5SQH8-4	6.46471	6.65062	6.4663	6.54563	5.92597	5.91544	6.83013	5.60714	6.39272	5.97126	5.83742	6.33822	5.89863	6.07467	6.27113	6.81002
ZNF239	Q16600	4.57236	5.84922	5.69879	6.0363	5.34895	7.40767	5.12957	5.09356	6.70179	5.746	6.4486	6.44076	8.42263	7.86373	7.15518	6.08489
ZYG11A	Q6WRX3	7.00099	7.53502	7.37266	6.36664	6.11888	6.52567	7.47463	7.03904	6.52823	6.67533	6.92717	6.75201	4.10781	4.4339	4.86786	4.27415
XRCC2	O43543	5.29889	5.89549	6.02034	6.11377	5.56688	5.68136	5.84531	6.25934	6.45749	6.16759	6.28367	6.53212	6.74224	7.19485	6.96519	6.97546
POLR2K	P53803	7.14603	6.94986	7.31059	6.57833	6.93371	6.88688	6.57515	6.45151	6.46661	5.93719	5.92329	5.76212	6.26805	5.077	4.61442	5.11928
RPP14	O95059	5.74462	7.05728	6.41827	6.52438	6.17695	5.88311	5.7912	5.80687	5.21923	6.29015	6.01387	6.271	6.50235	6.16801	7.02182	7.1109
NAT8L	Q8N9F0	6.77083	6.92607	6.94346	7.28494	5.85274	7.46108	6.98213	4.18364	7.03733	6.6748	6.9136	5.85419	6.68652	4.35952	4.07171	5.99743
CD70	P32970-2	7.16706	5.4435	7.37907	5.40601	6.19664	6.33389	6.33183	5.42468	5.97622	6.7727	6.65839	6.48419	5.77855	8.17744	5.14288	5.32695
DISP1	Q96F81	5.80962	7.18734	6.82538	7.03506	6.36943	6.2931	6.1706	6.21972	6.41559	6.18675	5.78702	6.09833	5.95107	6.23353	5.97225	5.44521
ANAPC11	Q9NYG5	7.9702	6.01755	6.17336	5.81067	4.12801	6.13488	8.31583	5.86792	6.40598	7.14142	7.13022	5.65303	5.33115	7.50943	5.15148	5.25888
TMEM258	P61165	6.71951	7.81538	5.6389	8.17155	5.62499	6.12686	6.54373	6.80903	6.21603	5.10836	6.90682	5.07462	4.83146	6.63832	5.48808	6.28635
PNKP	A0A1B0GVJ3	6.06597	6.49054	6.73614	5.19458	5.21039	6.1809	6.45489	4.63757	6.48505	7.2418	6.64972	6.85228	7.21965	7.0183	6.52826	5.03397
AKIRIN2	Q53H80	6.48877	6.36568	6.31608	6.70514	7.30005	6.37381	6.16929	7.14884	5.76114	9.03742	7.73097	7.03648	3.40126	5.18874	4.4737	4.50263
TIMP2	P16035	5.02436	7.13114	6.37415	6.87247	6.4876	4.41517	5.74229	7.30184	5.05795	7.0646	6.11227	6.60251	7.86364	6.81609	6.70537	4.42854
IP6K1	Q92551	6.88908	7.13857	6.14598	7.68811	5.74993	6.76698	5.88245	6.60416	5.63055	6.2177	6.07406	6.12437	5.26495	5.91268	6.27546	5.63497
SPCS1	A0A5F9YFS9	6.55626	6.80012	5.56938	6.71523	6.16822	6.45929	8.60536	5.26845	5.92078	5.68887	4.91838	6.21754	5.12291	6.7935	7.653	5.54271
FAM25A	B3EWG3	4.88381	7.47299	7.40875	5.56117	6.11221	5.49382	4.73942	6.69101	5.31297	3.88729	7.54039	6.41379	8.73013	8.60285	5.94402	5.20539
MT-ATP8	P03928	5.24253	4.55492	5.49217	5.72763	5.74207	4.7284	4.97882	5.80461	3.74259	6.24167	4.91269	5.82226	6.2248	11.9169	8.83148	10.0365
RAD51B	O15315	6.16174	5.97543	7.19806	6.54832	5.31082	6.41183	5.77207	6.03752	4.91016	5.11826	6.07436	8.59244	6.01045	6.19376	7.81362	5.87116
GAB3	Q8WWW8-2	7.62004	6.27239	6.91015	6.83944	6.82349	6.62506	5.72191	5.65285	6.17234	6.51339	6.50339	5.78148	6.55053	6.09538	4.79461	5.12355
MLH3	Q9UHC1	5.48255	6.42113	6.33551	5.50888	6.06298	7.09483	6.84101	6.05053	6.78152	6.83924	5.36395	4.84469	6.28335	7.72792	7.08163	5.28026
TMEM181	Q9P2C4	6.14479	6.5185	6.46265	5.89488	6.67632	6.1203	5.55179	5.7949	7.10842	6.61693	6.66375	6.36809	5.61013	5.22322	6.49638	6.74893
CD99	P14209	5.67687	4.3262	4.7442	4.92828	4.7731	3.59745	5.71333	5.83076	3.77619	5.32262	4.36218	6.60362	5.88812	12.6138	9.32336	12.5199

GATA2	P23769	6.27498	6.19766	5.99517	8.14866	6.43809	5.86258	6.64618	5.98937	6.4598	6.82922	6.47281	6.34532	6.80976	3.57831	5.9083	6.04379
CCSER2	Q9H7U1-3	7.25798	6.36473	7.84659	6.84561	3.4649	5.46712	7.4024	7.4744	5.797	6.67244	5.95964	5.74412	6.52233	5.44874	4.81671	6.91529
FBXO33	Q7Z6M2	4.48944	4.01399	7.70941	4.33607	5.79896	8.25466	5.48636	4.38003	6.04007	6.05143	5.18141	6.82825	6.24395	7.97725	7.70783	9.50087
FAM200B	P0CF97	6.87162	6.30111	5.73826	5.89114	6.72223	7.50216	5.2685	5.47472	4.98027	6.30502	6.67544	7.43562	6.2987	5.32262	7.15193	6.06067
SMDT1	Q9H4I9	6.74664	7.24443	5.87936	7.34899	6.46827	6.12068	6.10419	4.85984	5.95835	5.52116	5.7661	6.02061	6.53987	5.78345	6.0702	7.56788
ZDHHC9	Q9Y397	6.78039	7.11833	7.12752	7.28878	6.53179	6.0047	6.86884	6.1753	6.66655	6.54661	6.85251	7.04841	4.79641	4.61049	4.47595	5.1074
ATXN7L3	Q14CW9-2	7.15263	6.55392	6.79757	6.4037	5.86848	5.60352	5.56509	4.88826	7.33375	6.24145	5.65666	5.7233	6.25888	7.15625	5.86815	5.92841
FAM104A	Q969W3-2	6.13227	6.40474	6.90023	7.0784	6.3915	5.64665	6.19354	5.86311	6.76886	7.31636	7.19213	7.34564	5.57665	5.43551	4.66114	5.09326
PKDREJ	Q9NTG1	6.33549	6.617	5.58015	6.25576	7.17616	5.88125	6.41666	5.70175	5.89893	5.96371	6.33002	5.57078	6.57244	6.1204	7.48166	6.09782
IER3IP1	Q9Y5U9	5.86139	5.91147	5.9695	6.25241	6.05241	5.77057	6.8555	5.42281	6.91573	5.00882	6.01954	5.72847	6.51413	6.56307	7.50431	7.64986
DUSP22	Q9NRW4	6.40357	6.6701	6.96446	5.63473	6.74028	6.80806	6.66387	6.2643	7.12646	6.28127	7.30785	7.09657	4.84143	5.30388	4.48706	5.40612
ZNF124	Q15973	6.99102	6.75892	7.74799	7.70578	5.51165	5.33766	6.31752	6.84534	6.3848	7.68671	6.56927	6.87501	4.75534	5.01644	4.14781	5.34872
ZNF57	Q68EA5	6.09154	5.42283	6.0974	6.1353	5.95718	6.56768	6.95053	6.05789	6.21647	5.70377	6.16713	5.86131	6.14472	6.90794	6.7467	6.97161
TUSC2	O75896	6.75234	5.66426	6.46857	5.71339	6.84121	6.43735	5.12672	5.14027	6.21649	8.04509	6.69379	5.83573	8.10094	5.13865	6.63774	5.18745
VPS37A	Q8NEZ2	6.41136	6.25018	6.49501	5.35421	5.6177	5.67578	6.29717	6.76128	6.45863	7.05404	6.84672	7.19309	5.68323	5.90277	5.89981	6.09902
RABL2A	Q9UBK7-3	6.13358	8.13158	7.17842	6.25468	5.13448	7.18183	5.90425	6.57532	8.22672	6.01156	5.88887	7.02976	4.25111	6.1629	4.63047	5.30445
MEST	Q5EB52	5.47158	5.04995	6.55524	7.73295	5.10094	5.87944	8.2506	6.90206	4.71089	5.61092	6.82587	6.75369	6.90397	5.49549	7.16655	5.58987
MCFD2	Q8NI22	5.39192	4.74671	4.07395	4.06731	5.13953	3.5822	5.80479	6.13758	3.80269	4.36155	4.14161	5.96642	6.77544	13.9061	8.25817	13.844
EYA4	O95677	6.2065	6.13586	4.79305	6.42372	7.20661	7.15248	6.21996	5.62906	6.86079	6.29583	6.69405	5.10524	6.45268	7.81099	6.41434	4.59884
NEXMIF	Q5QGS0	6.87167	6.83868	6.45745	7.22511	5.1477	6.41942	7.0563	6.15201	6.25006	6.51454	6.36295	5.71849	5.17415	5.38683	6.30265	6.12199
TUBD1	Q9UJT1	6.36667	6.25837	6.28811	6.68366	6.09385	7.22563	6.07437	6.37636	6.07755	7.30497	6.70439	7.04424	5.49652	5.72159	5.00983	5.27389
GPRC5A	Q8NFJ5	6.40016	6.18179	7.69283	6.76855	5.63822	5.34605	6.36579	5.58183	6.62845	5.90589	6.09536	5.9214	6.25059	5.73413	6.83812	6.65084
SNRNP25	Q9BV90	7.06765	7.68231	7.60308	6.58744	6.95396	6.30459	6.73141	5.9285	5.47924	6.05009	6.06553	4.64975	5.77706	5.01706	5.50309	6.59925
MED8	Q96G25-2	5.87921	4.33972	6.05816	4.81501	5.84623	3.83626	5.49665	6.49679	5.06502	6.95495	5.65611	7.94965	7.23426	8.17684	6.49526	8.69988
DNAJA4	Q8WW22-2	7.14342	6.15457	7.20541	5.47892	5.35141	5.56344	6.79254	5.4036	6.82829	6.78933	6.94767	7.27823	5.49083	5.78083	5.88528	5.90622
FBXW4	P57775	6.88588	5.31164	6.31475	6.28059	5.86713	4.97908	5.95435	6.56638	6.20869	6.41453	6.38127	6.92674	6.68535	6.02278	7.06189	6.13895
CHST11	Q9NPF2	7.56045	5.67419	6.53947	6.70221	5.46795	6.59313	6.7398	4.94454	6.87142	6.01184	6.71854	7.28752	4.85266	6.15138	6.26929	5.61562
CADPS	Q9ULU8-4	6.46358	6.35483	6.56676	6.34464	6.66633	6.07907	5.92336	6.31701	5.89729	6.40404	6.46116	6.31298	6.31139	6.44536	6.0449	5.4073
NTN1	O95631	5.92394	5.55897	6.80106	4.78111	5.7024	6.85042	6.67157	6.70383	5.90112	6.35072	6.14654	7.05619	5.86843	6.23451	7.02418	6.42501
ZNF444	Q8N0Y2	5.95444	5.11755	7.02735	7.20481	6.39244	6.17963	6.66393	5.72144	6.51666	6.76162	7.26919	6.59692	5.70964	6.28228	5.76207	4.84003
ZNF205	O95201	6.16961	6.70113	6.47952	7.39115	6.47052	6.83332	6.04018	6.55548	6.33069	6.64808	6.49033	7.09795	5.25571	5.58417	4.63436	5.31779
CMTM3	Q96MX0	6.21698	4.81703	5.88866	6.73643	5.75215	5.86808	6.60147	5.70102	5.55308	6.67011	5.97824	6.3954	6.52326	7.07145	7.23034	6.99631
IMUP	Q9GZP8	6.32476	5.45574	7.44202	6.23235	6.40701	6.89926	6.21407	6.30443	6.79896	6.87047	6.84685	7.55444	5.26662	5.19841	4.82762	5.357
COX10	Q12887	6.42539	4.77158	5.91044	6.36797	6.49979	6.18339	6.90196	6.36407	5.85555	5.83964	6.85914	5.88458	6.11039	6.36348	6.39703	7.26559
SH2D5	Q6ZV89	6.1637	4.96054	7.92228	5.54793	6.50685	5.17186	8.51477	5.81926	6.45677	5.88332	5.76723	5.7145	7.62538	6.82644	5.63705	5.4821
DUSP16	Q9BY84	6.23001	5.28096	7.28253	6.16414	7.13671	6.93886	6.95155	6.72032	6.11741	6.3262	6.42645	6.78003	5.98826	5.28808	4.75947	5.60903
ROBO2	Q9HCK4-3	6.56403	6.87929	5.3375	5.75946	5.71855	5.20669	7.09551	5.74052	5.2541	5.58209	5.3072	5.91229	7.00072	7.46283	7.40923	7.76999
YAF2	Q8IY57-5	5.45427	5.15276	8.58283	6.09387	5.78472	6.16735	4.52701	6.06777	5.60488	5.86718	6.34923	7.29594	6.72173	7.26529	6.03014	7.03501
JCAD	Q9P266	6.43559	7.39404	6.6307	7.47699	5.84033	6.27467	6.5025	6.27591	5.8576	5.673	6.21511	7.16114	5.51875	5.80387	5.97402	5.96577
CD164	Q04900	7.39214	6.31158	7.31293	8.21022	7.10201	6.04126	6.71923	5.69183	6.8945	7.3185	6.31193	6.98836	4.61964	4.25902	3.9893	4.83754
ACBD7	Q8N6N7	4.89848	4.3163	4.71149	4.4782	4.69593	5.10333	5.70165	5.87777	4.52608	6.19659	4.9083	4.72709	4.80148	13.4597	9.06697	12.5307
KIAA0513	O60268	6.81759	6.00522	7.24735	6.20266	6.36502	6.10616	6.91088	6.12842	6.36283	7.15883	8.03491	7.14833	4.0431	4.81497	5.43677	5.21695
HERPUD2	Q9BSE4	8.35629	5.57862	7.14889	6.78537	6.19029	7.49962	6.7168	5.12766	6.30692	6.27524	6.36068	5.83842	5.05545	6.25399	4.51132	5.99445
GAGE13	Q4V321	4.70409	5.36749	5.57772	5.21037	3.81807	2.66388	3.84672	5.75886	3.15367	5.19043	4.05466	4.63258	4.76601	17.9557	8.98433	14.3155
ENTR1	Q96C92	6.04531	7.07722	6.3705	5.16666	7.12808	6.13297	5.50087	6.01573	7.68369	6.04707	5.82046	6.83227	6.56573	5.83842	6.00996	5.76508
ZBED9	Q6R2W3	6.41173	7.07866	6.11342	7.82887	5.44134	6.59857	6.15234	7.51545	6.14658	7.00593	6.47411	6.28909	4.72784	6.24574	4.78079	5.18954
ACOT9	F8WDI2	5.22922	4.84577	6.28487	6.52664	5.55878	6.47839	6.68031	5.87147	5.81159	6.19123	5.03749	6.28311	8.03217	7.28404	6.84502	7.03989
SLC35E2B	P0CK96	5.50621	6.19721	6.03267	7.20743	6.23385	6.66056	6.31817	6.10062	5.90527	7.2038	6.89338	6.36194	5.73345	6.05418	5.75212	5.83914
MAP2K5	Q13163	5.58821	6.22422	7.03493	6.37634	7.22556	6.0247	5.93354	5.79332	6.48828	6.49463	7.15244	7.17595	5.9075	5.68076	5.55485	5.34477
CKS2	P33552	5.89286	5.3765	6.9413	5.63634	5.10586	5.96466	5.49547	7.4375	5.12823	7.00152	5.92559	6.69477	3.57549	8.69854	5.87355	9.25181
INSIG2	Q9Y5U4	6.41496	6.34617	6.72884	6.64418	5.94537	6.20812	6.32594	5.28283	6.40599	6.17359	6.03459	6.41252	6.37078	6.26361	6.0187	6.42381
UBE2J2	Q8N2K1-3	6.7699	6.70581	6.43868	6.83337	6.54481	5.67288	6.85942	5.90464	6.18515	5.70659	7.72332	6.54434	5.57657	5.31637	5.18657	6.03157
BBX	H7C1U1	4.80677	5.26574	7.18379	6.36131	6.99249	5.05831	5.51311	6.8834	7.26981	5.61579	6.31588	5.47917	6.49412	7.0811	6.4783	7.2009
CGRRF1	Q99675	6.06407	8.04613	7.06277	5.91943	5.39327	4.52342	9.08182	4.12875	6.68812	6.90683	6.46577	7.24216	5.78348	5.36901	5.45586	5.86912
CREB5	Q02930	5.81899	5.71656	6.21311	6.20152	6.28178	5.54516	6.67605	6.1418	5.90796	6.3138	7.57765	7.06019	6.56433	5.73461	5.63453	6.61196
CDK2AP1	O14519	5.44015	4.79918	6.02533	5.55562	6.83671	4.57051	5.81397	5.77765	5.95267	6.82069	6.7893	8.04415	5.58687	7.15054	7.30753	7.52914
RAD52	P43351	6.14288	6.3747	6.31906	6.10707	6.50513	6.24816	6.74499	6.09052	6.72755	5.4188	6.02147	6.26872	6.69725	5.87776	6.34658	6.10937
IGHMBP2	A0A6Q8PGC0	5.80955	5.39192	5.66217	6.19124	5.33861	5.38622	6.03451	6.39365	6.94397	6.05836	5.98329	6.22585	7.22363	6.22818	7.45756	7.67129
C1orf109	Q9NX04	6.09922	4.61439	6.43527	4.78821	6.78501	5.32332	5.84908	6.36758	7.34228	6.87219	5.596	6.59483	5.37606	7.66388	7.19367	7.09903
EMC9	Q9Y3B6	5.52832	5.21973	6.75112	6.38798	6.18804	6.20473	6.69905	6.84604	5.40912	5.18721	6.9041	7.82461	5.0867	6.44713	6.49553	6.82058
C4orf3	Q8WVX3-2	5.86037	5.9539	5.18164	6.02323	6.95512	7.38327	6.64015	6.36813	6.829	5.24089	4.84474	5.61825	5.98607	7.50238	6.00609	7.60675
ARID3B	Q8IVW6	7.13357	8.12247	6.12358	7.71425	5.57036	4.71087	6.32539	5.42952	6.54435	7.16916	8.26077	5.8587	5.07528	6.7893	4.79969	4.37274
SPRR2G	Q9BYE4	0.907881	70.0743	0.893704	9.52228	0.237164	0	1.18567	0.948212	0.652258	2.28694	0.555697	1.07625	2.0006	1.91064	1.30149	6.44695
PIGC	Q92535	6.06164	6.25274	6.48134	6.4912	6.32071	5.725	5.88951	6.09582	6.73352	5.97053	6.36596	6.01023	6.39661	6.22875	6.77497	6.20147
NLRP3	Q96P20	5.4205	6.40177	6.66649	7.35005	6.87542	4.96519	6.07314	6.18812	6.53321	4.88538	5.78508	7.10451	6.45972	6.68869	6.15637	6.44636
TSNARE1	A0A590UJA6	4.64351	6.61307	4.33399	5.74869	7.78413	5.21964	4.97742	7.11944	5.86202	4.62499	6.83008	5.37513	8.47042	8.2299	6.90992	7.25764
SAV1	Q9H4B6	7.18133	6.19542	5.77234	6.45273	6.55837	6.35911	5.93082	6.2202	5.81483	7.83134	6.30282	6.67221	5.79534	5.20292	6.05401	5.65621
GRAMD2B	Q96HH9-3	6.63325	6.46087	6.56225	6.61317	5.93507	6.91751	6.46265	5.39061	6.26687	5.72249	6.34442	6.41892	5.966	6.1234	6.40151	5.781
RNF175	Q8N4F7	6.88492	6.14456	6.02139	7.41581	5.31314	5.80186	7.51237	3.76192	5.19746	8.20609	6.97082	6.53869	5.88331	6.66406	5.2353	6.44831
LHFPL4	Q7Z7J7	7.05546	6.25354	5.71544	5.13348	5.20575	6.20831	6.11847	5.55985	6.19155	6.39358	6.3474	6.70517	6.48617	6.71706	6.60462	7.30415
RBX1	P62877	6.57251	6.21148	6.72207	7.04417	4.94078	5.57409	6.177	6.57796	6.33595	8.23319	5.05102	5.12631	6.99261	5.94273	7.13162	5.36652
C20orf194	Q5TEA3	6.8196	6.37096	7.89288	5.87626	6.48939	6.75863	6.6018	6.35114	6.91832	5.52837	6.28581	6.7499	4.43722	5.59684	5.87268	5.4502
NECAB3	Q96P71	5.09947	6.44064	7.75116	6.57191	6.46127	6.3952	6.33016	6.40034	7.63793	7.68096	8.31679	5.87699	4.3064	5.36026	4.19	5.18049
CCSMST1	Q4G0I0	6.39187	6.26315	5.53518	5.08757	5.45993	4.92564	5.65967	5.75912	3.67924	5.62578	6.15098	6.56809	6.47704	9.70163	10.066	9.10753
LYRM1	O43325	5.44228	7.27236	6.82106	6.82128	5.58348	5.28356	6.27997	6.25537	6.80107	5.59975	4.75866	7.54047	8.0859	5.58928	5.46859	6.39692
ST3GAL3	Q11203-4	6.17553	5.77217	5.48285	6.83028	5.64398	5.68894	5.91739	6.17942	6.47667	6.33922	5.87168	6.40478	6.54573	6.23149	7.19043	7.24944
TOMM40L	Q969M1	5.99146	6.5362	6.03596	6.65732	6.18351	6.73638	5.69551	6.16427	5.66753	5.79949	6.5561	6.09581	6.60114	6.26474	6.80033	6.21426
ARSG	Q96EG1	6.43146	6.72323	6.19284	6.46058	7.71329	5.97846	6.52681	5.045	5.58777	6.67001	7.89854	6.48868	6.04301	6.13419	5.35114	5.75499
SUMO2	P61956	5.53117	6.07326	4.80931	5.52978	5.79184	6.22666	4.79376	7.15953	5.15093	7.36655	5.30798	7.30175	6.53889	8.56871	6.11104	7.73883
HYLS1	Q96M11	5.92656	7.51281	7.29491	6.54024	5.24649	6.49662	6.85245	5.83284	8.07165	6.55486	7.50048	7.07665	3.17834	6.59489	3.98126	5.33895
PEX26	Q7Z412	6.15427	6.66467	5.894	6.14849	5.85378	6.13164	6.19266	6.785	5.85063	5.61648	6.61403	6.51589	6.56694	6.28307	6.76247	5.96598
FOXRED2	Q8IWF2	6.28448	6.8741	7.05362	7.69172	5.33539	6.08125	6.53997	6.85011	7.11327	6.2594	8.84656	7.37384	4.60153	5.54023	4.31483	3.23971
S1PR1	P21453	6.2774	6.65478	6.03482	6.81247	5.02193	6.22879	7.46553	6.82004	6.32912	5.78548	7.36128	7.01198	5.15519	5.34221	6.1941	5.50487
KNG1	P01042	5.54205	8.17934	4.67889	6.01665	3.83777	5.5811	9.30239	4.00438	6.46498	5.62481	3.18655	5.4819	7.97034	6.33403	8.96761	8.82722
FASTK	Q14296	9.25894	7.2865	5.0535	7.1284	4.77995	7.81698	6.10106	5.90423	6.81769	3.79379	3.41865	7.38871	7.19996	7.64059	5.72858	4.68246
GABPB2	Q5SZG2	4.44605	4.1622	6.78569	6.21589	5.10816	4.96959	5.90004	6.18066	7.38162	6.40231	6.84623	7.97252	7.90199	6.61683	6.22076	6.88945
LPIN3	Q9BQK8-2	5.63603	6.55808	7.77124	6.38443	6.56987	8.27617	6.10488	6.4907	5.63621	5.8211	5.48879	6.37143	4.92829	5.90753	6.25051	5.80474
YIPF2	Q9BWQ6	6.453	5.33868	6.71907	6.85715	6.93597	6.44958	5.70508	6.74639	6.38877	5.84702	7.02017	5.44402	6.81069	6.64117	5.13629	5.50696
TTC16	Q8NEE8	5.5455	6.1138	5.94978	6.0648	5.52112	6.14749	5.91403	5.59799	6.00911	5.84713	5.84301	6.2698	6.8878	7.65137	7.25524	7.38203
KLHL5	Q96PQ7-3	6.16266	5.59542	8.45485	5.93662	7.67088	4.93021	5.44689	6.36153	6.26896	6.46733	7.78104	7.11833	5.49577	6.04355	4.80602	5.45994
RGS3	P49796	7.09775	7.97248	6.44234	7.23719	6.8123	5.20331	6.07246	6.88121	6.69587	5.63745	6.57887	7.97705	4.75906	5.2523	5.46593	3.91443
RAD9A	Q99638	3.92546	5.72793	5.86962	5.50523	6.4585	7.19785	6.08415	6.00647	5.28511	5.45084	7.57487	6.5011	6.92501	6.53804	7.29275	7.65708
WSB2	Q9NYS7-2	5.5119	6.4304	7.09218	5.63453	5.94706	5.62151	6.01053	6.45209	6.76928	7.31338	6.82554	6.31445	5.27198	6.01186	5.8282	6.96511
CENPW	Q5EE01	6.14634	6.64362	5.77223	6.98713	6.34935	6.36814	6.85761	5.97269	6.3737	5.97889	6.03616	6.25521	6.3318	6.00549	5.96226	5.95938
PDE4A	P27815	8.40745	5.70235	7.02365	6.41046	5.5158	6.47404	5.31401	6.04168	6.67685	6.28891	5.51645	6.34284	6.51711	6.3824	5.24623	6.13976
MYADM	Q96S97	7.33185	7.10287	6.57859	6.18867	7.66125	5.73665	5.6232	5.41388	5.28828	5.58621	6.75413	5.12248	5.89227	7.08042	7.09183	5.54742
PEMT	Q9UBM1-2	6.62983	6.05714	5.34824	5.86027	6.11135	6.8162	5.976	5.76696	6.45291	6.20463	5.79845	5.51193	7.23431	6.37871	6.73663	7.11645
MANBAL	Q9NQG1	6.27629	6.53936	5.87517	6.64213	6.17757	6.42482	6.3674	6.14792	6.00282	6.48881	5.86861	6.15461	6.35539	6.3042	6.10598	6.26892
CTDNEP1	O95476	7.42697	6.85176	6.13637	6.99426	5.76463	6.56464	5.64427	5.81424	6.13104	6.46118	5.51956	6.09719	5.13622	7.0042	5.81556	6.63792
NBDY	A0A0U1RRE5	5.89558	4.58127	7.85633	5.56969	5.95422	4.61786	4.95196	6.10059	5.36132	6.7893	7.51709	5.79754	4.68204	8.36304	7.16656	8.79562
PLAGL1	Q9UM63	6.09063	7.53099	6.39964	5.95023	6.31439	8.53708	6.44213	5.97204	6.81644	7.25529	6.42472	6.60238	5.15316	5.10369	3.65395	5.75324
CMTM8	Q8IZV2	7.13231	7.71502	7.25539	6.78939	7.08391	6.32389	5.99682	8.44105	5.3122	5.85933	7.00813	6.26762	5.58177	3.98305	5.36291	3.88721
ZBTB8OS	Q8IWT0	6.67497	5.66016	6.38941	4.94373	6.75315	6.02506	6.6938	8.03911	8.19211	6.18365	6.49544	7.25778	4.58452	6.36282	4.63765	5.10663
TMEM268	Q5VZI3-3	7.598	7.21813	6.2152	4.7313	6.17014	5.91236	5.45748	7.08644	6.23473	5.32024	7.1554	7.49142	5.44692	6.88501	4.55898	6.51825
PNRC1	Q12796	7.41903	8.4509	7.37693	7.71722	5.83904	5.87091	6.05101	5.92524	6.33037	7.04586	7.72207	6.78597	4.33607	4.2787	4.04996	4.80072

ZNHIT3	Q15649	6.92733	6.43694	7.06331	6.95788	6.66329	5.97622	6.63703	6.69366	6.6347	5.39246	6.56901	6.77182	5.71587	5.00146	5.05794	5.50106
IPPK	Q9H8X2	6.6966	6.96284	7.3513	7.77709	6.21238	6.20833	6.04354	5.99036	6.7602	7.46566	7.62458	5.7193	5.22678	4.86718	4.56882	4.52504
SPPL3	Q8TCT6	6.71171	6.37783	6.07432	7.06852	6.07561	6.16512	5.9523	5.25117	6.18512	7.2585	7.15119	5.5925	6.10356	5.83036	6.24837	5.95381
BORCS8	Q96FH0	6.2182	5.35473	4.83354	5.85628	5.83177	4.70354	5.59653	6.33355	4.90595	5.98034	4.60456	5.24142	4.83859	10.1409	8.7108	10.8493
CPTP	Q5TA50	5.94774	6.07539	6.34012	6.14856	6.48141	6.98295	6.75559	7.48326	5.97901	7.10525	5.30122	7.10244	4.82412	5.79473	5.48317	6.19503
TLR8	Q9NR97-2	7.78113	5.72043	5.99178	5.27079	5.08607	4.32732	5.57533	6.78956	5.88464	6.37837	5.99113	5.72486	7.27898	6.27732	7.65726	8.26503
CEP126	Q9P2H0	5.9975	6.49254	6.24554	7.21312	7.4392	5.76988	4.86789	7.18668	7.44954	6.65953	6.93915	6.93441	5.24734	4.87287	4.39792	6.2869
ZDHHC4	Q9NPG8	7.05824	5.12826	6.07218	4.93749	6.06146	7.42251	6.53856	5.07751	6.37587	7.43489	5.74224	6.08898	6.77839	6.90333	6.28982	6.09026
SLC6A8	P48029	6.64896	7.04925	6.41447	6.12664	5.23848	6.4025	5.64325	7.21738	6.97995	5.77023	6.28735	5.12024	6.08355	5.83214	6.50822	6.67741
KCNE2	Q9Y6J6	6.69869	6.22919	5.46522	5.25095	6.09992	4.86861	5.13079	7.15291	5.12001	7.21094	6.29989	6.60595	6.25708	7.88408	7.26151	6.46427
SLC18B1	Q6NT16	6.5315	7.4988	7.73247	4.94331	4.87324	6.22508	5.38716	7.15056	7.00577	5.84776	6.96961	6.03625	6.28754	5.05279	6.37332	6.08483
TSPAN3	O60637	6.60552	7.21669	6.75227	7.04784	7.30123	6.66228	5.84737	5.70378	6.09344	7.91394	7.75734	5.92704	4.36986	5.59056	4.76856	4.44229
IGHV4-28	A0A0C4DH34	6.4954	6.25186	5.96212	6.25483	6.76811	6.66157	5.05724	7.03511	6.38207	5.98834	6.96344	5.8661	7.71183	5.96826	4.98012	5.6536
C2orf16	C9JG08	5.42241	6.62246	6.529	5.93038	5.9365	6.02819	5.69259	5.93875	6.82002	6.0621	6.88945	7.7305	6.13892	6.28495	6.00376	5.97002
TMBIM4	Q9HC24	5.60841	6.8858	5.98258	4.9523	6.49593	5.45106	7.15504	8.37978	5.5251	5.97069	4.68173	7.71359	3.78614	6.60575	6.53957	8.26653
ZSCAN29	Q8IWY8	5.591	7.14078	6.65344	7.39676	7.59075	5.38286	4.86609	6.38171	6.35142	6.70677	5.87914	5.09042	6.03599	6.20208	6.10068	6.6301
SHROOM3	Q8TF72	6.6786	7.33522	7.38381	6.53715	9.69723	6.4331	6.99107	4.56549	5.7909	4.52176	6.77231	3.24452	6.29798	6.97236	5.5058	5.27272
ZNF160	Q9HCG1	6.82429	7.37654	4.43282	7.47281	6.39116	6.47076	6.89142	5.42675	5.62651	6.43928	4.86481	6.16278	6.78004	7.154	4.81794	6.86809
SLC66A1	Q6ZP29	5.85549	7.15622	6.20646	5.5533	5.69723	5.10789	7.35502	5.06982	6.65208	5.71864	5.71556	6.14599	6.22356	6.98767	7.15772	7.39735
ZBTB5	O15062	6.17991	6.52445	6.17161	6.35637	6.277	6.28249	6.09638	5.85491	6.32403	5.83647	5.80868	5.67733	6.71273	6.61708	6.88414	6.39644
NCOR1	H0Y459	6.05154	6.0626	6.17279	6.90345	6.29694	5.7545	6.16202	5.9954	6.27881	6.03055	6.82346	6.71549	6.61951	5.73241	6.36598	6.03456
SPATA2	Q9UM82	6.61153	5.80107	6.86173	5.6355	6.1019	6.47383	7.22573	6.71483	7.34353	5.53233	6.10926	7.01069	5.61046	5.58697	5.31463	6.06601
B4GALT7	Q9UBV7	5.25416	6.78861	6.62085	6.72065	6.12689	6.27846	6.57116	4.3495	6.97952	7.3625	5.30296	5.95271	6.29918	5.85324	6.71471	6.82489
DHRS13	Q6UX07	6.63249	6.1351	5.82147	6.33071	6.02174	6.06916	6.56097	5.95182	6.09409	6.55238	6.12681	5.90425	6.50795	6.34079	6.43192	6.51832
RNF128	Q8TEB7	5.57809	7.28972	7.18105	7.26421	6.26843	6.09863	6.78629	6.0425	6.47645	8.26798	6.53355	6.30748	4.13221	5.91372	5.62573	4.23396
ST8SIA5	O15466-2	6.96959	4.94082	5.89374	6.74704	6.44201	7.26875	6.31002	6.40782	6.50345	4.45533	6.87935	8.17899	5.32948	7.24658	4.57796	5.84906
TMCO6	Q96DC7-2	4.76975	5.23827	5.91688	5.48471	5.83735	6.3175	5.48058	5.88714	6.22986	7.20092	7.6189	6.39982	7.03505	6.81266	6.9574	6.81322
PYURF	Q96I23	6.44124	4.91503	5.79448	5.51552	6.47347	4.58684	5.18879	6.23984	4.47508	6.23688	5.50415	7.29657	5.57949	8.3247	8.40558	9.02235
METTL8	Q9H825	6.19778	6.37306	5.67867	6.76329	7.18984	6.61287	6.36527	6.56984	6.61027	5.74472	5.21509	5.38724	6.4209	6.01318	6.34259	6.51539
SEMA3A	Q14563	7.6695	7.07748	5.32535	6.22427	4.24208	5.55416	7.98034	5.39347	6.48683	6.2113	5.9929	4.31135	7.12134	7.04816	5.71729	7.64419
SLC25A26	Q70HW3	6.15544	5.59195	5.94913	6.65201	6.1376	5.63123	7.6606	6.35267	6.63833	5.43728	6.20561	5.54007	7.10528	5.68457	6.84442	6.41382
ZSCAN26	Q16670	5.84823	5.86418	5.88447	5.96852	6.65169	6.62449	5.93368	6.12275	6.02729	6.61136	6.36183	6.72115	6.60995	6.95115	6.09576	5.72349
RNFT1	Q5M7Z0	5.72358	4.56551	6.36394	5.7358	5.73208	5.46094	6.54788	3.88136	6.14986	7.16586	8.11203	7.67117	4.56882	8.35294	7.67822	6.29002
AUNIP	Q9H7T9-2	5.46788	6.76111	3.68323	4.61779	6.26471	4.37872	6.73622	8.05496	6.78342	7.92327	9.05331	6.75283	5.53932	5.96195	4.9979	7.02336
EVA1B	Q9NVM1	6.57826	6.67324	5.90661	5.71686	4.88592	5.69543	7.26083	7.1617	5.08351	4.73761	6.31663	7.10083	3.86875	7.35422	6.79081	8.86878
JAM2	P57087-3	5.98937	6.48909	5.9937	5.91137	6.29854	6.46826	6.61977	6.06617	6.45359	6.39144	5.58511	5.73504	6.76998	6.83822	6.04583	6.24451
TBC1D19	Q8N5T2	6.50247	5.90503	6.22638	5.83317	6.75986	6.64938	6.93489	6.75397	6.01377	5.52548	5.91518	7.11195	5.66098	6.59253	5.95045	5.66452
TMEM123	Q8N131	6.67922	8.44898	6.09572	9.04463	5.42307	7.90125	5.56826	4.90281	5.81866	5.72585	3.97504	5.69673	6.51797	5.07692	5.62883	7.49606
TNC	P24821-4	7.56972	7.44572	3.6281	8.4341	5.32811	4.32394	6.6196	6.07557	8.0771	6.61693	4.65011	4.66574	7.33979	4.56906	6.42342	8.233
TMEM18	Q96B42	6.26494	6.49309	6.63475	7.02417	6.53151	5.30714	5.98793	6.10693	6.60403	5.95629	5.23235	5.85651	6.0038	5.9352	6.94489	7.11647
SLC16A6	O15403	6.28215	6.12585	6.53227	7.49792	6.9771	8.1062	6.13442	7.17172	6.1233	4.83513	5.32082	5.67664	4.72811	4.40363	6.75031	7.33442
KDELR1	P24390	5.62911	6.32268	6.20622	5.66035	5.76134	5.95995	6.33398	6.38801	6.76354	5.83258	5.95022	6.00197	6.41921	6.17751	7.30671	7.28663
MEA1	Q16626	6.11895	4.22822	8.59872	6.27568	4.03277	4.16293	4.99792	8.22316	4.90696	7.05695	5.33586	6.33494	4.23711	9.28092	7.68033	8.52858
CACNB3	P54284	7.13973	5.57432	6.58645	6.98382	6.09221	7.3858	5.22955	7.76719	4.9038	5.41331	6.09111	7.55541	6.27887	6.27585	5.52385	5.19872
PLG	P00747	8.14753	6.31514	4.80657	7.54193	5.0038	5.70762	7.83021	6.84373	5.69054	5.97433	4.72553	6.82716	6.84831	4.99625	6.60963	6.13172
ZNF649	Q9BS31	5.79364	5.58335	5.4455	6.45045	6.0511	3.96983	6.36805	6.05783	6.9836	5.86786	6.05187	5.50877	7.52099	7.16875	8.0833	7.09511
SEL1L3	Q68CR1	7.0928	7.81491	5.84935	8.1349	6.88953	7.36911	5.92758	6.03055	7.37194	6.66317	5.68408	7.49755	4.79399	4.10102	5.02137	3.75813
TAS2R42	Q7RTR8	7.38273	6.54008	6.06592	4.39606	8.0319	5.61601	8.35749	6.83591	6.90804	6.1364	4.31104	6.15933	6.3057	6.39858	4.69792	5.8569
RBM48	Q5RL73-2	6.06209	6.60542	5.23845	5.33787	6.40122	6.32177	5.27533	5.98352	6.15275	6.80793	5.96469	6.04536	7.17506	6.7705	7.34813	6.50989
MINDY4	Q4G0A6	5.54066	6.37454	6.83203	7.17685	6.81412	6.26105	6.30983	6.18896	6.09209	6.1996	7.6768	7.31187	4.74638	5.85666	4.99862	5.61994
SEMA4F	O95754	6.83101	6.85721	7.53192	9.27019	6.20035	6.93087	6.28015	7.64039	4.32691	4.49742	7.1409	5.40096	6.49983	3.7626	5.14175	5.68756
RGMB	Q6NW40	6.45104	5.98808	5.35136	8.14021	6.56749	7.99094	5.91633	4.36527	3.98549	6.61663	6.9327	6.5212	5.61737	8.6423	6.06739	4.84619
CMTM7	Q96FZ5	5.14499	6.56458	8.03751	7.34391	4.83245	7.35275	7.43962	5.95172	6.7693	7.64887	6.94304	6.89012	4.22234	6.56358	3.83201	4.4632
JTB	O76095	6.94788	6.13911	6.09777	6.63185	6.38969	6.04388	6.43448	5.48047	6.65966	5.85428	6.62247	5.96928	6.17966	6.18871	6.58208	5.77872
PWWP2B	Q6NUJ5	6.92997	6.75907	6.01924	6.39465	6.11473	6.58993	6.01638	5.67138	6.01338	6.45513	6.05358	6.30678	6.30238	5.83056	6.34984	6.19908
PLAT	P00750	5.81589	6.45249	5.23075	9.92171	5.7993	4.97009	6.82344	6.67445	6.59851	5.00974	6.39902	6.94521	4.39615	5.60225	6.74276	6.61825
OASL	Q15646	6.35151	6.36412	5.25407	6.01756	6.79439	5.02095	6.38956	6.78405	6.28593	7.09445	6.13052	5.4088	6.54113	6.47183	5.94012	7.15101
EFNB2	P52799	5.82553	8.69756	6.11956	5.5384	7.49293	6.68823	6.45925	5.37886	6.89022	3.00846	6.1654	6.08299	7.049	6.55403	6.55992	5.48966
ARL4A	P40617	6.86743	7.13497	6.4558	7.48221	6.37044	6.43466	7.74483	6.60029	7.0345	5.69001	5.13595	6.29113	4.30147	5.94482	4.95994	5.55154
RNF181	Q9P0P0	7.75245	6.06683	6.29259	3.89125	4.80808	4.39371	5.32783	6.43435	5.38636	6.13836	4.81265	5.70832	4.49736	14.0045	5.6805	8.80485
TMEM243	Q9BU79	6.9309	6.15389	5.98652	5.9324	6.40252	6.14246	7.37797	7.63489	5.90154	5.54023	7.27868	5.33562	5.29861	5.97472	5.73866	6.37039
SIAH1	H3BU09	5.87402	6.88093	5.95586	5.56834	5.57864	5.33419	6.37212	6.28421	7.72138	6.28228	5.99251	7.08586	5.72272	5.44446	7.16417	6.7383
DHRS2	Q13268-2	5.80972	5.02808	6.14624	6.17881	7.10582	6.31969	5.88006	5.25636	6.23188	4.20494	7.74119	5.08947	6.89922	5.96788	7.68502	8.45563
COL6A3	P12111	8.37011	7.12307	8.03252	4.80798	4.40924	5.63128	6.86896	9.29093	6.70002	10.5161	6.15197	6.83773	4.5053	2.83406	3.66228	4.25841
BPGM	P07738	5.90274	6.6177	6.48714	6.17299	6.66006	7.59754	6.60756	6.11814	4.91348	7.03191	5.89619	6.25381	6.23719	5.77321	5.48544	6.24492
PLAG1	Q6DJT9	5.84574	7.24472	6.47579	6.81996	4.45441	7.73244	5.79668	6.08447	6.92089	6.36192	6.45407	6.85589	5.73212	5.66058	6.55446	5.00586
TOB1	P50616	6.97402	6.43768	7.92791	5.91105	6.82526	6.11412	6.15245	7.26418	7.79738	8.10603	7.78462	7.57273	3.90587	4.10029	3.09263	4.03378
DNLZ	Q5SXM8	6.99501	6.07039	5.6937	6.88118	6.32418	6.53615	6.39721	5.40425	6.07178	6.47862	6.65257	6.00368	6.31457	5.67925	6.0923	6.40517
IPMK	Q8NFU5	6.79744	6.55326	6.8258	6.2223	5.74356	6.55901	5.69894	6.67381	6.57347	7.24404	7.24569	7.31603	5.27595	5.61855	4.97411	4.67804
THEM6	Q8WUY1	6.96649	5.84107	5.92493	7.3363	6.31961	4.35193	5.61364	8.32738	5.86231	5.10978	6.41793	6.65671	6.51795	7.17306	5.76891	5.81199
PROS1	P07225	5.50165	5.99929	4.88678	5.80707	5.30833	7.82772	7.97439	6.20483	4.6239	5.2096	5.57087	5.72155	7.04777	8.0043	7.42563	6.88632
NHLRC1	Q6VVB1	6.34323	5.9991	6.84331	5.76903	6.07899	6.39597	6.50326	6.71077	6.68403	6.79118	7.29723	6.79021	5.73212	5.00726	5.5636	5.4907
METTL6	Q8TCB7	7.25237	5.65437	6.52922	7.7003	6.057	5.72148	5.64606	7.73116	4.8012	6.33481	8.3492	7.19411	5.53112	3.79654	6.37475	5.32634
FDX2	Q6P4F2	6.07338	4.34024	4.28022	4.34778	7.56637	4.8405	4.54513	5.61404	4.41272	5.96864	6.14107	4.82377	5.70576	10.8965	7.5844	11.8595
SMIM4	Q8WVI0	6.8363	6.27184	6.07784	6.27392	6.71136	5.83858	7.16032	7.10781	5.42795	5.65552	6.36692	6.5023	5.56111	5.35622	6.91962	5.93239
DLK2	Q6UY11	7.14621	6.05321	7.6454	8.09388	5.46572	5.72257	6.59809	8.14077	6.77351	6.45184	7.5761	6.72712	4.28342	4.6471	4.50267	4.17239
N6AMT1	Q9Y5N5	4.97368	4.18729	5.96901	4.75196	5.19033	5.0028	5.72343	7.21707	5.39815	6.81384	5.49299	6.76963	5.25413	9.21049	8.28657	9.75863
ZNF720	Q7Z2F6-2	5.75223	6.93341	5.10613	6.44548	6.26091	6.4356	7.31079	6.48414	5.72861	6.97891	6.04497	6.50146	6.76468	7.4455	5.8117	3.9955
MARCHF3	Q86UD3	7.06767	5.57364	6.66126	7.38187	6.5337	5.41583	7.95478	6.57889	6.30231	5.81039	7.27527	6.57933	5.14716	5.0936	5.37214	5.25214
NSUN7	Q8NE18	7.66229	6.79724	7.72921	8.66614	7.17456	6.7386	6.72911	4.64021	7.76327	5.26623	4.51708	5.76673	5.09411	4.5902	4.96783	5.89718
ADIPOR1	Q96A54	7.05834	7.89171	7.34754	7.78215	6.31503	5.11421	5.89447	5.92768	8.26953	8.11663	7.6543	9.17315	3.48585	2.89417	2.72512	4.35011
MSH5	O43196-3	7.72731	6.25917	6.32385	5.55621	7.30279	7.26667	5.68728	7.74509	7.16523	8.5366	8.53463	6.54371	3.75411	3.21599	3.00876	5.37261
RHOU	Q7L0Q8	5.5486	6.20352	7.04835	6.81667	5.98141	6.60749	5.02243	6.20218	6.76893	5.66843	6.87957	6.39176	8.09268	5.49781	6.17784	5.09235
TIRAP	P58753-3	5.7311	5.57963	5.77684	7.31791	6.11198	5.57754	6.0569	6.49092	7.5746	5.60307	6.19169	6.43027	6.57365	5.41544	6.59338	6.9751
MT-CO1	P00395	6.40402	7.22732	5.1693	6.80064	7.02277	6.64066	5.62698	7.21359	5.60825	5.44612	5.32931	5.49309	6.54027	6.14648	6.89645	6.43474
MGLL	Q99685	6.74127	7.02484	8.09055	5.73549	6.73449	6.55858	7.98001	7.2743	5.60755	5.481	5.07271	6.79844	5.27459	4.89161	4.97722	5.75737
CSKMT	A8MUP2	6.5982	5.03168	7.48025	5.46716	6.97036	5.99148	6.18223	6.42306	5.62573	5.79999	4.65656	8.42663	4.46375	6.72044	7.26338	6.89908
TSC22D1	H7C4J9	6.011	6.05028	6.85165	6.85232	5.87606	7.45432	5.2549	6.15069	7.69544	5.23668	6.91255	4.58357	6.31757	6.65998	5.79928	5.74092
AAMDC	Q9H7C9	5.81749	4.23006	5.58249	4.35789	5.64535	5.98739	5.38843	7.30053	4.81931	7.21102	5.28377	5.78864	6.05789	10.1962	8.01619	8.31732
SFT2D2	O95562	6.05817	5.78198	6.15157	6.55762	6.3905	6.5471	6.30874	6.10853	6.32326	6.60602	6.06331	6.75267	5.91289	6.08821	6.13086	6.21858
ARMCX5	Q6P1M9	6.61886	7.14768	6.85231	7.15342	6.00573	6.16869	6.13528	5.52265	5.72252	7.43704	6.60364	6.96962	5.36652	5.06796	5.70643	5.51604
CYP1B1	Q16678	5.79765	7.26245	5.41617	6.59853	6.67147	6.29769	4.26672	5.88635	7.84386	5.07901	6.94864	5.70541	6.93688	5.17207	5.65394	8.46315
ZBTB14	O43829	6.78003	7.25975	6.41534	5.41419	6.48508	6.72272	5.81975	6.86442	6.36247	5.93691	5.93222	6.67724	5.74067	5.34483	5.97218	6.27218
ITIH4	Q14624	7.00696	6.85556	5.13781	5.07275	4.26817	5.92446	6.36209	6.54605	6.20864	5.40889	6.12974	6.42253	7.04947	5.56759	9.29969	6.73961
GON7	Q9BXV9	5.09192	4.14476	3.84773	6.24807	5.9448	2.88323	4.88203	5.64415	4.16188	4.42504	4.48748	5.35875	4.84161	16.268	7.74823	14.0223
TSPAN33	Q86UF1	7.32232	6.02446	5.87001	6.32979	5.75949	5.15325	6.75223	6.23945	6.37939	6.50853	6.2485	5.72336	5.80077	6.19545	6.51005	7.18297
IDS	P22304	6.40782	6.17126	5.88476	5.352	6.23658	7.39515	6.83941	5.82116	5.8336	7.06993	6.85321	6.20263	5.85681	6.44573	6.48985	5.14011
TMEM107	Q6UX40-2	6.20574	5.71638	4.83556	7.85752	5.23442	5.0734	3.55878	4.87309	6.41272	6.6792	6.51896	6.7757	7.49354	8.06782	6.74625	7.95091
GNG5	P63218	6.14797	5.52258	5.23196	5.18989	6.72816	5.71163	6.13663	6.44059	5.34797	5.59153	5.1565	5.55467	6.09583	8.81285	7.73138	8.59985
PABIR1	Q96E09	5.59469	4.72762	6.48197	3.42724	5.69384	5.831	6.33294	5.39394	4.74253	5.23842	6.37615	8.14052	9.232	7.51617	6.7412	8.52977
E4F1	Q66K89	6.30866	6.87877	6.86413	7.19549	6.83242	6.67339	5.80219	5.94136	6.46339	6.74892	6.61107	5.7237	5.47957	6.01517	4.86285	5.59891
WDR90	Q96KV7	6.41227	5.71915	6.87274	7.53598	7.37385	6.25605	6.00185	5.99419	7.33659	6.97551	7.20478	6.67534	4.43904	4.40903	6.62816	4.16547
LSAMP	Q13449	6.34893	5.8914	5.81922	7.21411	6.07429	6.77406	5.42912	5.96772	5.17033	6.24084	6.63995	5.83821	6.47295	6.93655	7.13984	6.04247
TMEM53	Q6P2H8	6.31201	6.26982	5.70686	6.06647	5.25275	6.28642	6.20304	6.11901	6.02682	6.06823	5.5904	5.63896	7.07982	7.26333	7.22949	6.88656
TSPAN14	Q8NG11	6.25824	6.24989	5.60909	6.70972	6.3995	8.10416	6.45453	5.38607	5.2889	4.95826	4.95766	7.04169	7.09326	6.17339	7.23753	6.07812

GFRA1	P56159-2	7.14401	7.45983	6.06085	6.67399	7.19414	6.77982	6.31098	5.96358	5.99464	5.41499	6.54782	5.54851	5.0828	6.31716	5.49014	6.01673
MTURN	Q8N3F0	4.78629	5.4086	5.2303	4.68514	4.21051	5.54837	7.88669	6.3698	5.80056	6.98018	4.22392	6.72556	5.83064	9.51519	8.05521	8.74302
BEAN1	J3KRN6	8.37358	8.78316	8.56522	8.8046	7.00773	7.89478	9.04564	3.33904	7.03057	4.14739	4.11796	3.54173	5.13263	4.30396	4.49759	5.41445
ACAP3	Q96P50	6.12241	6.12642	6.18475	6.36676	6.62256	7.15837	6.0684	7.0879	6.27356	6.27782	6.77469	5.81222	4.78776	6.1885	6.53541	5.61249
KLHL29	Q96CT2	4.75633	5.97602	6.01418	5.70681	5.79233	6.32832	7.02191	5.75444	5.54386	4.02646	4.20337	6.73512	7.74053	7.34094	8.77426	8.28512
POU3F2	P20265	8.02498	5.69793	6.6321	7.69944	5.66346	4.08855	5.12964	6.53391	5.74414	5.31667	7.04085	7.12569	6.1444	6.77312	7.10415	5.28096
SUGCT	Q9HAC7-3	6.598	6.08761	5.77701	7.28302	5.79956	6.42187	5.73921	5.80411	6.07502	6.72354	6.65694	5.95296	6.0512	6.79669	6.4456	5.78768
RNF145	Q96MT1-5	7.42208	6.92263	6.2372	7.2354	7.09139	6.78668	6.65839	6.276	5.87141	6.45124	6.05134	5.75012	4.97798	5.99667	5.65394	4.61752
USP53	Q70EK8	6.09254	6.12572	6.52037	6.1805	5.94084	6.61719	6.8482	6.19504	6.20643	5.58168	6.14654	5.76915	6.44113	6.62435	7.10833	5.60197
F2	P00734	7.71392	6.5104	4.50318	5.47692	5.44537	7.59342	8.73857	5.03138	6.96877	6.16685	3.49115	8.25491	5.4451	2.82434	8.34678	7.48894
ST6GAL1	P15907	6.81303	6.19366	5.2328	6.5769	5.58121	5.92567	7.81041	5.41471	5.53199	5.86142	6.03575	6.29074	5.11161	6.73551	8.15321	6.73139
CTDSPL	O15194	6.36392	6.32589	6.05272	6.39114	6.37591	6.2477	7.41248	6.67635	5.81585	5.4146	5.58673	5.92964	6.21719	5.51132	7.00088	6.67768
ZNF32	P17041	6.64149	7.32473	6.62549	6.97194	6.17494	6.04353	6.11437	5.81476	5.72588	5.92985	5.84914	6.96262	6.20826	6.18413	5.45122	5.97764
LHFPL6	Q9Y693	6.91679	8.09165	7.63626	5.75686	5.77079	4.11237	7.46234	5.40051	6.55452	5.91475	7.1683	6.28071	5.97016	5.41897	5.55719	5.98783
RCBTB1	Q8NDN9	7.25701	5.91822	8.2966	4.95693	5.25581	5.46508	7.4845	6.75746	6.27677	6.70493	6.54123	6.91153	6.96617	3.77028	6.35097	5.08651
PDGFC	Q9NRA1	8.57338	6.64639	9.07972	8.41452	5.97743	6.24474	6.44489	6.01658	6.50292	6.2694	7.1308	5.85433	3.19701	4.46968	4.52621	4.65201
ZNF529	Q6P280	6.98279	6.98217	5.91504	7.39843	5.58288	5.83824	5.41503	5.85088	6.07589	5.79372	4.84724	5.2463	6.64387	7.41541	7.10809	6.90402
TMEM250	H0YL14	6.44824	5.91026	6.04387	7.03328	6.07719	7.76136	5.40077	4.78818	5.69301	5.66872	6.05899	7.79195	6.2864	7.91856	6.18906	4.93015
CHCHD4	Q8N4Q1-2	7.87466	5.22975	6.20687	4.81326	6.30708	5.25589	6.61304	5.61699	4.05014	6.28186	6.43723	6.874	7.29759	7.25323	6.37298	7.51545
MIEF1	L0R8F8	7.06256	5.42068	6.30725	7.61376	7.01018	5.45105	8.58645	6.45081	5.71671	5.44233	3.73833	6.10336	6.94589	5.92533	4.89543	7.32988
TTLL5	Q6EMB2	6.74104	6.18471	5.15176	6.0642	8.13131	6.36662	6.04845	5.7295	6.51879	6.29415	5.9618	6.74206	6.41745	5.26573	6.2623	6.12013
BRAWNIN	Q69YU5	6.60449	6.20758	5.53823	5.7707	5.97638	5.57942	4.87082	6.58552	4.8044	5.99787	5.5285	5.28744	5.12848	8.28372	8.79797	9.03849

Phosphopeptide identification					TMT Quant Data															
Phosphopeptide	Gene	Uniprot	Site	Motif	DMSO_1	DMSO_2	DMSO_3	DMSO_4	IACS_1	IACS_2	IACS_3	IACS_4	KD025_1	KD025_2	KD025_3	KD025_4	Combo_1	Combo_2	Combo_3	Combo_4
AAAS_S495	AAAS	Q9NRG9	495	AQFPRFSPVLGRA	6.7966	6.7425	6.0810	6.5925	6.3399	5.9616	6.4219	6.0478	6.3369	6.2046	5.8592	6.0276	6.2327	6.1126	6.0997	6.1432
AAGAB_S310;S311	AAGAB	Q6PD74	310;311	NA	6.4578	6.2594	6.8777	6.2193	6.2984	6.4757	6.0010	6.8358	6.5829	6.7463	6.6736	6.8579	5.6903	5.8223	5.0095	5.1920
AAK1_S1093	AAK1	A0A096LP25	637	GHRRILSDVTHSA	5.7476	5.7773	6.5809	5.7927	5.5097	5.9630	6.0672	5.8631	6.3859	5.6521	5.8307	6.1322	7.0411	7.5238	7.1190	7.0134
AAK1_S21	AAK1	A0A096LP25	901	FAPPEVSPEQAKT	6.1565	6.5889	6.8427	6.8420	6.1847	6.9516	6.4525	6.8420	6.9271	6.8545	7.0018	7.2140	4.6673	5.0751	4.4190	4.9803
AAK1_S618;S624	AAK1	A0A096LP25	606	QPKVQTTPPPAVQ	5.7509	6.4520	6.8475	6.3342	6.1435	6.7424	6.4719	6.7520	6.6289	6.6783	6.8964	7.1215	4.9871	5.8619	4.9614	5.3702
AAK1_S637	AAK1	A0A096LP25	389	PIQPALTPRKRAT	6.2730	6.7749	6.8456	6.8560	6.0387	7.2891	6.8824	6.5049	7.2946	7.4117	6.4819	6.9476	4.5803	5.4603	4.0133	4.3456
AAK1_S678	AAK1	A0A096LP25	731	PEKLGGSAESLIP	6.2099	5.8948	6.3851	6.3323	5.9396	5.9583	6.2876	6.3113	6.2010	6.1028	6.2526	6.6424	6.4504	6.4588	6.2471	6.3259
AAK1_S682	AAK1	A0A096LP25	682	SGSPRTSQQNVYN	5.4006	5.7372	6.7789	5.3123	5.5742	5.9319	5.7207	6.1254	6.3310	5.6034	6.1555	6.1344	7.1506	7.5503	7.3387	7.1549
AAK1_S731	AAK1	A0A096LP25	875	RPLLMESEEEDES	5.3070	5.5607	6.4673	6.1154	5.6648	6.0758	5.5834	6.0686	7.3180	6.2550	6.4823	6.0104	6.7451	7.0634	6.8088	6.4741
AAK1_S875	AAK1	A0A096LP25	1093	KPFRPQSLSKYSR	7.6339	5.8628	6.9214	6.4657	5.7306	6.9235	6.4732	5.5317	8.3476	6.8064	7.2879	7.6232	4.3903	4.8514	3.9884	5.1620
AAK1_S901	AAK1	A0A096LP25	678	TTTPSGSPRTSQQ	6.9039	6.8962	6.4732	6.4979	8.3140	5.9880	5.5533	6.7302	6.5059	6.2550	5.7653	5.9281	6.2219	5.9210	5.5326	4.5134
AAK1_T389	AAK1	A0A096LP25	620	QKVGSLTPPSSPK	5.4995	5.5407	6.7017	6.3507	6.0973	6.0539	5.1831	6.0598	6.1027	5.6534	5.8231	6.5764	7.3187	6.9759	7.1273	6.9356
AAK1_T606	AAK1	A0A096LP25	21	GLGSGSSGGGGST	6.5703	6.2389	7.3850	6.6629	6.5582	6.4776	7.3457	6.5743	6.0929	6.9358	6.7747	6.0053	4.7374	5.9650	5.3265	4.3496
AAK1_T620	AAK1	A0A096LP25	620;624	NA	6.2659	5.8619	6.6102	5.9849	6.4747	6.0913	6.2860	6.1170	6.2762	6.5276	6.4169	6.4115	6.3005	6.5495	5.9655	5.8602
AAK1_T620;S624	AAK1	A0A096LP25	618;624	NA	6.7492	6.5095	7.1754	6.7967	5.9969	6.1485	6.3577	6.3701	6.9229	6.3003	6.5560	7.0988	5.0064	5.5702	5.0052	5.4361
AASDH_S724	AASDH	Q4L235	724	NLKGLNSPVLIGK	7.1858	7.6116	6.9594	7.0593	5.5876	7.1256	7.7233	6.7615	6.9915	7.3434	7.8023	8.3725	3.0108	4.3117	2.8507	3.3030
AATF_S153	AATF	Q9NY61	203	RAGDRNSEDDGVV	5.2711	6.2842	6.1292	6.3922	6.9355	5.5470	5.9521	6.7532	5.5847	5.9697	5.7405	5.8976	6.8915	6.6843	6.6891	7.2783
AATF_S203	AATF	Q9NY61	153	PGFSVQSISDFEK	6.6281	6.7666	5.8337	7.5166	5.8085	5.6490	6.1866	6.0062	6.7580	5.5283	6.0690	6.1004	6.6416	6.5930	5.8110	6.1034
AATF_S273	AATF	Q9NY61	273	GGPEFSSALKNSH	7.1717	7.2258	7.4670	6.7163	5.7844	6.1480	6.8481	6.1151	6.2939	6.7269	6.5222	5.4514	5.2126	6.1198	5.1008	5.0961
AATF_S316;S320;S321	AATF	Q9NY61	316;320;321	NA	6.0509	6.3349	6.1250	6.7210	5.9682	6.3373	6.0924	5.9737	6.0948	6.0846	6.2106	6.6946	6.2933	6.2602	6.2742	6.4845
AATF_S320;S321	AATF	Q9NY61	320;321	NA	5.8681	6.0768	5.9120	6.2245	5.8574	5.4256	6.4193	5.4440	6.5653	7.4298	7.1807	6.9085	6.2189	6.1833	6.3316	5.9543
ABCA3_S866	ABCA3	Q99758	866	QHERRASDWAVDS	5.8136	5.7880	5.7520	6.1146	6.6930	4.9398	5.6725	6.2557	6.4393	7.4286	6.5870	7.1560	6.2793	7.0017	6.4914	5.5873
ABCC1_S929	ABCC1	P33527-9	940	ISRHHNSTAELQK	5.8932	6.0492	5.6175	6.0783	6.1876	5.7951	6.0723	5.8709	5.9762	5.6032	5.7125	5.6049	7.4325	7.5339	7.5160	7.0568
ABCC1_S940	ABCC1	P33527-9	929	QLSSSSSYSGDIS	6.3514	6.9994	6.4900	6.9272	5.7787	6.5542	6.5675	6.6861	6.5011	6.8661	6.7231	6.5760	5.0229	5.6597	5.1379	5.1588
ABCC3_S895	ABCC3	O15438-4	895	LLQRNGSFANFLC	5.3862	5.6280	5.8548	5.1286	4.9448	6.0883	6.0567	5.9535	5.8013	6.9192	5.3425	6.6375	8.2021	7.5897	8.1660	6.3008
ABCC4_S404	ABCC4	O15439	646	QPPVPGTPTLRNR	6.3657	6.5752	6.4159	6.8507	6.8207	5.9783	6.3640	6.3342	6.1299	6.0517	6.0207	6.1956	6.0864	6.1643	5.8873	5.7592
ABCC4_S629	ABCC4	O15439	404	RNRQLPSDGKKMV	6.5934	6.6174	6.1956	6.8266	6.0144	5.8116	6.4420	6.1341	5.9159	5.8128	5.2988	5.8406	6.3920	6.4925	6.6426	6.9698
ABCC4_S655	ABCC4	O15439	655	LRNRTFSESSVWS	5.9524	5.8033	5.8224	6.1476	5.5774	5.7599	5.9109	6.5293	9.0711	6.5501	6.2734	6.7843	6.8433	5.9248	5.2357	5.8141
ABCC4_S664;S668	ABCC4	O15439	629	SGIDFGSLLKKDN	3.4774	3.4859	3.6286	4.0593	4.0198	3.9771	4.0387	3.8126	9.0098	9.0209	8.3232	8.5845	8.3129	8.6632	9.1415	8.4446
ABCC4_T646	ABCC4	O15439	664;668	NA	6.6526	6.9149	7.0787	7.3639	5.8765	6.2537	6.3478	5.9156	6.6093	6.6470	6.1477	6.8609	6.0684	6.1259	4.4878	4.6495
ABCE1_T42	ABCE1	P61221	42	KLCIEVTPQSKIA	6.9045	6.4641	7.0351	6.4749	5.4927	6.5591	7.6168	6.9110	6.7879	6.9649	6.3065	5.5488	5.3254	5.9525	4.7318	4.9239
ABCF1_S105;T108;S109	ABCF1	Q8NE71	140	ALIQDQSEEEEEE	5.9637	6.2644	6.5853	6.4559	6.1159	6.2175	6.2798	6.2668	6.3182	6.4478	6.4271	6.5242	5.7249	6.4800	5.5740	6.3546
ABCF1_S108	ABCF1	Q8NE71	109	KLSVPTSDEEDEV	5.9301	6.3340	6.4192	6.4164	5.9121	5.7303	5.9631	6.3013	7.0036	7.1461	7.0078	7.2156	5.2786	6.1626	5.5140	5.6652
ABCF1_S109	ABCF1	Q8NE71	22	GDGESTSPSDKVV	6.0563	6.3995	6.8784	6.9496	6.1039	6.5914	6.4496	6.5442	6.5168	6.6796	6.7290	6.8566	5.0382	5.7523	5.0110	5.4438
ABCF1_S140	ABCF1	Q8NE71	228	KKAEQGSEEEGEG	6.2171	6.0371	6.3545	6.4492	5.9079	6.5334	5.8943	6.2315	6.4676	6.6600	6.6869	6.8038	5.2480	6.4719	5.9302	6.1067
ABCF1_S22	ABCF1	Q8NE71	621	PDPPPLSPPVLGL	5.7667	6.4052	6.7487	6.3144	6.3345	6.6933	6.2052	6.3651	5.9964	5.9719	6.4670	6.2747	5.6450	6.8243	6.0161	5.9714
ABCF1_S228	ABCF1	Q8NE71	108	KKLSVPTSDEEDE	5.4093	5.2029	6.9019	5.5754	5.8926	5.6157	6.7585	5.8699	6.6452	7.3475	6.2349	8.0069	6.5948	5.7516	7.9113	4.2816
ABCF1_S621	ABCF1	Q8NE71	105;108;109	NA	5.1705	5.3544	5.1467	5.2092	5.9901	6.0059	5.4946	5.9025	5.8540	5.6021	5.5197	5.6023	7.6496	8.9230	8.2732	8.3022
ABCF1_T108;S109	ABCF1	Q8NE71	108;109	NA	7.1539	7.7539	7.4134	7.1348	6.4915	6.6148	6.8970	7.0234	5.9423	6.5425	6.2082	6.6404	3.8432	5.0354	4.2124	5.0930
ABCF2_T218	ABCF2	Q9UG63-2	218	LHGLGFTPAMQRK	6.8216	6.1449	7.2458	7.7065	6.5134	5.8367	5.9796	6.5849	6.6485	6.4753	7.1997	6.9630	4.2989	5.5968	5.2759	4.7087
ABHD15_S434	ABHD15	Q6UXT9	434	LSRHRASFLGGRR	4.7174	4.4481	5.1880	5.3161	5.7050	5.0604	5.7069	5.4796	5.6607	5.4186	4.8945	5.0216	9.6721	9.8030	9.4925	8.4155
ABI1_S183	ABI1	Q8IZP0	183	PTQKPPSPPMSGR	6.2046	5.9359	6.6089	6.0355	6.2213	6.2565	6.1297	6.1021	6.6482	6.7141	6.4836	6.6166	6.2905	6.2491	5.7321	5.7713
ABI1_S216	ABI1	Q8IZP0	225	RLGSQHSPGRTAS	6.7457	7.3820	6.6218	6.5232	6.8370	7.6766	5.9318	7.7927	6.5921	6.0276	7.3698	6.8772	4.2701	4.6132	4.3042	4.4350
ABI1_S225	ABI1	Q8IZP0	216	PNDYMTSPARLGS	6.6893	6.8511	6.9546	6.7902	6.5608	7.3050	6.8935	7.2743	7.5851	7.2432	7.4361	7.3165	3.5418	4.4887	3.2325	3.8373
ABI1_S231	ABI1	Q8IZP0	213	PTVPNDYMTSPAR	5.4251	5.5070	6.7591	5.4381	6.2728	6.5905	6.0490	6.7558	5.9324	7.1853	7.1590	7.0053	6.2358	6.0539	5.6155	6.0156
ABI1_Y213	ABI1	Q8IZP0	231	SPGRTASLNQRPR	7.6577	6.5923	7.5806	5.9906	5.5127	6.5578	5.7428	6.5010	6.9510	7.1704	7.1561	7.3881	5.6989	6.1322	3.6683	3.6994
ABI2_S183	ABI2	Q9NYB9	227	MAPSQQSPVRTAS	6.8225	6.7270	7.0428	6.3282	6.1638	6.8499	6.3317	6.5635	6.8697	7.0539	7.2947	7.3555	4.6407	4.9588	4.2756	4.7217
ABI2_S227	ABI2	Q9NYB9	183	PTQKPPSPPMSGK	6.7724	5.6261	6.8617	5.7122	6.4883	5.9137	5.9912	6.2547	6.4342	6.3190	6.4658	6.7010	6.6172	6.1625	5.6732	6.0069
ABI2_S368	ABI2	Q9NYB9	368	PPTIGGSLPYRRP	6.6930	6.7024	6.6618	6.0484	6.0644	6.3357	5.7394	6.7899	6.4554	6.7099	6.5363	6.4589	5.7010	6.2525	5.7508	5.1002
ABL1_T800	ABL1	P00519-2	833	SSPPNLTPKPLRR	5.3878	5.8831	5.9170	5.7543	5.5556	6.4060	6.6579	5.7927	6.2372	6.1090	6.1118	6.5986	6.7505	7.0035	6.8329	7.0023
ABL1_T833	ABL1	P00519-2	800	VTRGTVTPPPRLV	5.6338	5.5177	5.8278	5.8892	5.7518	6.0712	7.6331	7.4428	9.8455	5.9204	6.5022	5.9167	5.1075	5.6301	5.5865	5.7237
ABL1_T871	ABL1	P00519-2	871	AAAEPVTPTSKAG	6.1695	6.9369	5.9515	7.4987	5.0051	5.4091	6.6356	5.8841	7.5182	6.5806	7.3233	6.7579	6.1252	6.0862	4.2713	5.8469
ABL2_S620	ABL2	P42684	655	KGGFFSSFMKKRN	10.7357	6.4003	8.0673	6.8597	6.4191	5.6016	6.3108	5.2159	6.8055	5.3157	6.3277	5.1879	5.5725	6.6379	4.3011	4.2414
ABL2_S631	ABL2	P42684	936	KVPVLISPTLKHT	6.6122	6.7395	6.7033	6.2415	6.4620	7.2370	7.2134	7.6027	6.7349	6.6830	7.0762	6.7583	4.1987	4.8363	4.1025	4.7986
ABL2_S655	ABL2	P42684	631	RKQRDKSPSSLLE	6.0547	5.8367	6.4554	6.0396	5.7858	6.3644	6.1591	6.3585	6.3225	6.4165	6.2431	6.3735	6.3114	6.6130	6.2603	6.4055
ABL2_S72	ABL2	P42684	620	AQASSGSPALPRK	6.0950	6.0752	6.7046	6.1800	5.8051	6.5830	6.2524	6.2067	6.7278	6.6155	6.7311	6.7793	5.6662	6.1775	5.2341	6.1665
ABL2_S915	ABL2	P42684	915	EQPGWPSPAKAAP	7.1203	6.6404	7.1936	6.0668	6.0262	6.8763	7.0349	7.7834	6.1380	7.4091	6.4980	6.2153	4.2852	4.8567	4.3869	5.4689
ABL2_S936	ABL2	P42684	72	DKTGGSSPEALHR	6.4005	6.7167	6.1403	6.2837	6.4546	6.5675	5.9695	6.8866	6.2189	5.7259	5.8536	6.5704	5.4313	6.6451	6.1158	6.0195
ABLIM1_S706	ABLIM1	O14639	706	RMDRGVSMPNMLE	6.5851	6.5095	6.6881	5.7422	6.8733	6.1023	6.2074	6.7848	6.6855	6.8475	7.0766	6.7706	5.2195	5.4633	5.3101	5.1341
ABR_S33	ABR	Q12979-2	33	ELGTPTSPGSGSP	5.8996	6.2778	6.5055	6.5848	5.9954	5.9171	6.9660	6.7476	6.3581	7.0423	6.8135	7.0453	5.9903	5.2433	5.6245	4.9888
ABRAXAS1_S336	ABRAXAS1	Q6UWZ7	48	KNSITDSQMDDVE	5.9047	6.3349	5.4923	7.0545	5.8303	5.7117	5.7763	6.7350	6.8542	6.4369	6.1450	5.4893	7.4159	6.3804	6.2882	6.1505
ABRAXAS1_S48	ABRAXAS1	Q6UWZ7	336	TDIPEASPASTPQ	6.6605	6.3246	6.3058	6.4875	6.0517	7.1258	6.0785	6.3930	6.1221	6.4909	6.1322	5.8747	6.6478	6.1704	5.5991	5.5354
ABRAXAS2_S280	ABRAXAS2	Q15018	280	SLDPAFSPRMPSS	5.3675	4.8870	5.1104	6.1148	6.8612	6.4319	6.3913	6.0143	5.7088	6.6640	6.0240	5.3317	7.3929	7.2817	6.9482	7.4702
ABTB2_S365	ABTB2	Q8N961	365	PLCPGASPARQAR	5.9437	6.6772	7.5040	6.4992	6.3715	6.7674	6.8863	6.9339	6.6251	6.9327	7.2517	6.4827	4.2900	5.1665	4.5779	5.0902
ABTB2_S67	ABTB2	Q8N961	67	MNSRHNSWDTVNT	5.6435	6.4715	7.6954	6.7843	5.8369	6.0152	5.9608	7.5353	6.5909	5.9472	6.7224	5.9744	5.9004	5.1827	5.2356	6.5033
ACACA_S115	ACACA	Q13085-4	117	HIRSSMSGLHLVK	5.0268	4.9292	4.8007	4.6188	7.4863	6.3242	5.9453	6.5739	4.3704	3.9618	4.7043	4.5081	9.5283	9.8425	9.1398	8.2395
ACACA_S117	ACACA	Q13085-4	66	SVSEDNSEDEISN	5.7776	6.2404	6.9012	6.1282	6.1791	6.6124	6.1074	6.5127	6.0020	6.3150	5.9272	6.1992	6.0975	6.5789	6.1308	6.2905
ACACA_S60	ACACA	Q13085-4	60	SRFIIGSVSEDNS	6.2460	6.6165	6.4560	6.2675	6.5561	6.3664	5.8781	6.9314	6.1617	5.9447	6.1817	6.0140	6.2136	6.3457	6.1255	5.6950
ACACA_S60;S66	ACACA	Q13085-4	62	FIIGSVSEDNSED	6.4854	5.9976	6.8360	6.2178	6.1928	6.0629	6.0533	6.4079	6.1958	6.7328	6.4406	6.1154	5.9236	6.3757	5.9206	6.0416
ACACA_S62	ACACA	Q13085-4	115	ALHIRSSMSGLHL	9.0176	8.2806	7.2076	8.2614	6.5175	5.7757	6.0202	5.4995	8.6546	7.7813	6.8818	7.2889	2.8507	4.0270	3.1186	2.8169
ACACA_S62;S66	ACACA	Q13085-4	62;66	NA	6.6010	6.6582	7.6033	6.9127	5.9894	6.4924	6.1537	6.4225	6.5868	6.7838	6.6471	6.5961	5.4378	5.2634	4.5154	5.3365
ACACA_S66	ACACA	Q13085-4	60;66	NA	7.2590	7.4813	7.6100	7.2091	6.6399	5.8445	6.0645	6.5708	7.3712	6.6545	6.8967	7.1435	4.4163	4.6812	4.0274	4.1300
ACAP2_S334	ACAP2	Q15057	521	KYSISLSPPEQQK	5.8696	6.2439	6.7696	6.0629	6.3374	6.7338	6.4105	6.5972	6.4497	6.6795	6.4835	6.6220	5.8177	5.8191	5.3944	5.7094
ACAP2_S521	ACAP2	Q15057	334	FCFEVVSPTKSCM	5.3066	6.8229	7.2201	6.4595	6.2465	7.7084	7.2873	6.1344	7.0311	6.9815	6.0864	6.9879	4.1729	4.7221	5.6957	5.1368
ACBD3_S43	ACBD3	Q9H3P7	43	PPLPPPSPPGSGR	6.7170	5.7079	5.3767	5.6484	7.0741	5.5475	4.5462	7.5994	6.0288	5.8088	5.9330	6.6523	7.4131	8.1246	6.7972	5.0248
ACBD5_T172	ACBD5	Q5T8D3-3	172	GNVLTSTPNAKTV	6.2942	6.5940	6.3345	6.4339	6.8165	6.6215	6.5400	6.5075	7.0782	6.6972	6.6107	6.7504	5.0032	5.2005	5.0264	5.4913
ACBD5_T181	ACBD5	Q5T8D3	181	GNVLTSTPNAKTV	6.4686	6.8605	7.0907	7.1486	6.6745	7.4175	7.7446	6.4324	7.0501	6.7653	6.3817	7.0335	3.9302	4.4452	3.5926	4.9642
ACD_S25	ACD	Q96AP0	25	GSETPSSPRAGQL	6.4412	6.7139	6.4924	6.5065	6.2860	6.1521	6.1347	6.6586	6.0634	6.7832	6.6464	6.1742	5.6240	5.8870	5.8463	5.5902
ACD_S338	ACD	Q96AP0	349	SCTPSLSPRSHVP	6.1949	6.4372	5.9956	6.2029	5.8382	6.0076	6.1754	6.8879	7.0938	6.8439	6.8235	6.1609	6.6871	6.0825	4.9723	5.5964
ACD_S338;S349	ACD	Q96AP0	338	HASRTPSSPLQSC	5.0746	5.3230	5.8407	5.8301	6.1874	5.4776	6.3719	5.9027	6.3168	6.1833	5.4899	6.1569	8.0363	7.2972	7.4969	7.0149
ACD_S349	ACD	Q96AP0	338;349	NA	6.2869	6.2650	6.3085	6.9039	6.4496	6.3251	6.5204	6.6804	6.3647	6.0682	7.0503	7.0400	5.4285	5.3229	5.5413	5.4444
ACIN1_S1004	ACIN1	Q9UKV3	898	ADDSRISEDETER	6.0638	6.0291	6.5973	6.6870	6.4793	6.3975	6.4528	6.0466	6.1522	6.2784	6.5454	6.2699	6.3244	6.1558	5.7417	5.7788
ACIN1_S1104	ACIN1	Q9UKV3	1004	RTAQVPSPPRGKI	6.5081	6.6643	6.1958	6.6851	6.0081	5.9067	6.2854	6.1039	6.2772	6.3304	6.1254	6.2740	6.2071	6.1555	6.0062	6.2669
ACIN1_S1161	ACIN1	Q9UKV3	414	TASEEETPPPLLT	5.9196	6.0860	5.6910	6.1637	6.0643	5.8699	6.0053	5.8085	6.0062	6.1138	5.9489	5.9101	7.0767	7.1340	7.1737	7.0282
ACIN1_S166	ACIN1	Q9UKV3-5	885	ADDSRISEDETER	6.3718	6.3923	6.0873	6.9665	6.2492	6.0145	6.3037	5.9288	6.2889	6.0723	6.0575	5.9018	6.4108	6.0542	6.5050	6.3956
ACIN1_S207	ACIN1	Q9UKV3	682	SGQEVATPPVPQL	6.1936	6.4776	6.1129	6.3857	6.1741	6.0566	6.3058	6.0160	6.1531	6.0843	6.2220	6.0712	6.4550	6.3871	6.4118	6.4933
ACIN1_S386;S388	ACIN1	Q9UKV3	490	SDTKAESPAEKVP	6.0541	6.2304	5.7743	6.3720	6.2890	5.9125	6.1443	6.0475	6.2017	6.2347	5.8610	5.8802	6.8451	6.5732	6.8742	6.7058
ACIN1_S400	ACIN1	Q9UKV3	825	SFKRKISVVSATK	5.5017	4.5985	4.8805	5.3571	7.9258	4.9234	4.3385	5.0984	9.1500	9.0548	7.8346	8.5901	6.6031	7.2436	3.9379	4.9620
ACIN1_S410	ACIN1	Q9UKV3	561	KEGRRASHTLLPS	4.6012	4.1959	4.4373	5.2186	8.0471	5.1774	4.8013	5.7814	7.4751	7.7978	6.9069	6.9752	7.9393	7.8942	6.4690	6.2823
ACIN1_S410;T414	ACIN1	Q9UKV3	838	GVPAGNSDTEGGQ	7.0516	7.4709	7.0173	7.7953	7.1055	7.0813	7.0263	7.5756	5.3538	5.0957	5.0284	5.2073	5.0570	4.9932	5.4863	5.6546
ACIN1_S414	ACIN1	Q9UKV3	410	LPEQTASEEETPP	6.5085	6.5421	6.4062	6.9351	6.5855	5.9280	6.4346	6.4568	6.0241	6.1390	5.8041	6.3753	5.9196	6.2029	5.8519	5.8861
ACIN1_S490	ACIN1	Q9UKV3	657	NSRKSLSPGVSRD	6.2662	6.5676	5.8363	6.4141	6.0588	5.7278	5.8351	5.8677	6.3813	6.1431	5.9863	5.8841	6.8727	6.7724	6.7828	6.6037
ACIN1_S561	ACIN1	Q9UKV3	729	LQPERGSPKKCEA	7.2753	6.5780	6.5573	6.3161	6.5400	6.3592	6.6927	5.2185	5.8987	6.0181	6.1342	6.2010	6.6537	6.1168	5.6018	5.8386
ACIN1_S655;S657	ACIN1	Q9UKV3	710	VQARRLSQPESAE	4.6173	4.6490	4.4420	5.0452	6.7770	5.6425	5.9656	5.9569	5.0982	4.9752	4.5887	4.7748	10.0753	8.5993	9.6829	9.1102
ACIN1_S655;S661	ACIN1	Q9UKV3	1332	RSRSRSTPVRDRG	7.2751	7.0820	6.6388	7.1700	6.4266	6.1297	5.9797	6.2088	6.4609	6.9722	6.8980	6.7418	4.9337	5.3453	4.8821	4.8552
ACIN1_S657	ACIN1	Q9UKV3	676	ETKDPSSGQEVAT	6.6024	6.5077	6.4985	6.6033	6.4455	5.8892	6.4661	6.7813	6.0957	5.6534	6.0172	5.7989	6.1522	6.1728	6.4113	5.9046
ACIN1_S667	ACIN1	Q9UKV3	408	VALPEQTASEEET	6.8755	6.5422	6.8320	6.6415	6.1379	5.6843	6.2705	6.7291	6.2980	6.5994	5.6488	6.0661	5.6633	6.2647	6.1016	5.6452
ACIN1_S676	ACIN1	Q9UKV3	207	HSPRKSSSISEEK	6.1353	5.7095	6.8393	7.2453	6.0560	6.1505	6.2489	6.1930	6.2305	6.6181	6.7119	6.8978	5.3482	5.4362	5.7626	6.4168
ACIN1_S710	ACIN1	Q9UKV3	400	EDRKKASLVALPE	5.7100	5.2039	5.0544	5.6672	5.8886	5.3105	5.5101	5.2063	5.8572	5.9053	6.2100	5.1887	8.2379	8.0575	8.6132	8.3794
ACIN1_S729	ACIN1	Q9UKV3	895	DLHADDSRISEDE	6.2250	6.3344	10.4689	5.8398	10.8513	6.0053	5.3237	5.9315	6.7542	6.4868	6.7664	6.9543	3.6081	4.1326	4.2607	4.0568
ACIN1_S815	ACIN1	Q9UKV3	1104	LLVDRPSETKTEE	7.3471	7.0329	7.2401	7.3830	6.4026	6.3361	6.8494	6.5826	7.1702	6.0983	6.7073	6.3952	4.6426	4.5845	4.5749	4.6532
ACIN1_S825	ACIN1	Q9UKV3	1161	RRERTRSEREWDR	7.7645	7.5598	7.7377	8.4340	6.2550	6.5212	6.8645	5.0968	5.6389	6.4494	7.6445	6.4740	4.5805	4.7005	4.0743	4.2044
ACIN1_S825	ACIN1	Q9UKV3	987	SISQQKSGVSITI	5.9946	6.8193	6.8457	6.7525	6.5894	5.3762	6.0072	5.9161	6.9043	6.4271	6.5223	6.3425	5.7428	6.1415	5.8347	5.7839
ACIN1_S838	ACIN1	Q9UKV3	166	AELEEASAESEDE	5.9418	6.4922	6.4824	6.1570	6.6243	5.5308	6.0494	6.6659	7.2529	5.7877	6.1204	4.5988	6.7637	7.5416	6.4481	5.5431
ACIN1_S885	ACIN1	Q9UKV3	667	SRDSSTSYTETKD	6.2494	6.6512	6.0355	6.3166	6.3490	6.4033	7.0096	6.3925	6.0907	6.3836	6.5548	6.3095	5.7634	5.6801	5.7558	6.0549
ACIN1_S895	ACIN1	Q9UKV3	815	EEKKESSLPKSFK	5.7400	5.4577	4.9763	5.2174	5.7665	5.9866	5.8640	4.7926	4.9725	5.5349	5.2408	4.7517	10.2423	8.2315	8.6800	8.5452
ACIN1_S895;S898	ACIN1	Q9UKV3-5	825	SFKRKISVVSTKG	5.4429	5.5337	5.2260	5.6513	7.9588	5.3869	5.2789	6.0070	8.4626	7.7368	7.0873	7.4465	6.3675	6.2435	4.4128	5.7575
ACIN1_S898	ACIN1	Q9UKV3	655;657	NA	6.3431	6.2981	6.0324	6.6745	6.0093	5.9186	6.1610	6.1095	6.4309	6.3279	5.9150	6.2528	6.3695	6.2850	6.3925	6.4800
ACIN1_S987	ACIN1	Q9UKV3	410;414	NA	6.3042	6.4954	6.3273	6.3282	6.5471	5.7841	6.2473	6.4013	6.0995	6.2420	5.9930	5.8654	6.4623	6.3134	6.4458	6.1435
ACIN1_T1332	ACIN1	Q9UKV3	655;661	NA	6.4364	6.6544	5.8070	6.5561	6.1322	5.7761	6.2298	5.8808	6.4722	6.1914	6.0332	6.1972	6.3689	6.4219	6.3071	6.5352
ACIN1_T408	ACIN1	Q9UKV3	386;388	NA	5.9754	6.2965	6.2348	6.5925	6.0922	6.0173	5.9041	5.4778	6.3149	6.1360	5.8926	6.4326	6.9321	6.5907	6.6004	6.5101
ACIN1_T682	ACIN1	Q9UKV3	895;898	NA	6.7780	6.2165	6.4391	6.8426	6.6872	6.5901	6.5333	6.5707	6.3655	7.0855	6.5621	6.3843	4.4771	5.2776	5.0925	6.0978
ACLY_S455	ACLY	P53396	481	PQDSVPSPRSLQG	6.0529	6.0603	6.6931	6.1803	5.9522	6.2338	6.3367	5.9072	6.5166	6.6619	6.7349	6.6238	5.8395	6.4184	5.6648	6.1236
ACLY_S481	ACLY	P53396	455	APSRTASFSESRA	4.5625	4.5877	4.9599	4.2974	5.7602	5.9701	5.7606	6.1475	4.7001	4.5040	4.6626	4.7535	10.2940	9.7039	10.0840	9.2520
ACLY_T447	ACLY	P53396	447	ASGSTSTPAPSRT	4.6747	5.1347	5.6409	5.2540	6.0287	6.3741	6.6060	5.9931	5.0770	5.4436	5.3022	5.3763	8.5200	8.7327	7.6934	8.1486
ACOX1_S26	ACOX1	Q15067-2	26	THILDGSPEKTRR	5.6346	6.6594	5.7742	7.2711	6.0839	5.8777	6.8961	6.4878	6.7445	6.8562	6.5827	6.3842	5.2983	5.6681	5.9445	5.8368
ACSL3_S683	ACSL3	O95573	683	PVKIRLSPEPWTP	6.1141	6.2145	5.8788	6.4700	6.0788	5.9361	6.0422	6.2632	6.0851	6.2747	6.1291	6.2144	6.2758	6.7997	6.5620	6.6614
ACSL3_T688	ACSL3	O95573	688	LSPEPWTPETGLV	5.4365	5.9300	5.7919	6.5995	5.6903	6.5509	6.0134	6.6744	6.5165	6.3724	6.3239	6.5627	6.0356	6.7643	6.3825	6.3550
ACSL4_S674	ACSL4	O60488	674	PIKVRLSPEPWTP	5.1961	6.1375	6.6279	6.9834	5.9492	5.5895	6.6518	6.3117	5.9366	5.4504	6.1979	6.3525	7.0199	6.2977	7.2082	6.0897
[truncated: 3,513,867 more chars]
